# *PLOS ONE* 2015 Reviewer Thank You

**DOI:** 10.1371/journal.pone.0150341

**Published:** 2016-02-23

PLOS and the *PLOS ONE* editorial team would like to express our gratitude to all those individuals who participated in the peer review process of submissions to *PLOS ONE* over this past year. During 2015 *PLOS ONE* published over 28,000 research articles. This would not have been possible without the contribution of more than 76,000 reviewers from around the world and a wide range of disciplines.

The names of our 2015 *PLOS ONE* reviewers are listed in S1–S5 Reviewer Lists. Thank you to all our reviewers for generously sharing your time, insight and expertise with *PLOS ONE* authors in the evaluation of their work. Your efforts are a key reason for *PLOS ONE’s* success as an innovative and influential publication.

## Supporting Information

S1 Reviewer List Alphabetical list of 2015 *PLOS ONE* reviewers (A-D). (PDF)

S2 Reviewer List Alphabetical list of 2015 *PLOS ONE* reviewers (E-J). (PDF)

S3 Reviewer List Alphabetical list of 2015 *PLOS ONE* reviewers (K-O). (PDF)

S4 Reviewer List Alphabetical list of 2015 *PLOS ONE* reviewers (P-T). (PDF)

S5 Reviewer List Alphabetical list of 2015 *PLOS ONE* reviewers (U-Z). (PDF)

## References

[1] (2015) PLOS ONE 2014 Reviewer Thank You. PLoS ONE 10(2): e0121093 doi: 10.1371/journal.pone.0121093

