# Supplementary material for: PLOS ONE 2015 Reviewer Thank You
Source: PLoS One. 2016 Feb 23;11(2):e0150341. doi: 10.1371/journal.pone.0150341 (PMC4764340; doi:10.1371/journal.pone.0150341)
Supplement: S1 Reviewer List — (PDF) [file pone.0150341.s001.pdf]

PLOS | ONE

PLOS ONE would like to thank all those who reviewed on behalf of the journal in 2015:

Finn Lillelund Aachmann
Eivind Aadland
Claus Aagaard
Rasmus Aagaard
Philip Aagaard
Eivind Aakhus
Ågot Aakra
Rob Aalberse
Lauri Aaltonen
Mari Aaltonen
Eric Aamodt
Håvard Aanes
Nikolaos Aantonakakis
Ardalan Aarabi
Jiska Aardoom
Julie Aarestrup
Gloster Aaron
Erika Aaron
Shawn Aaron
Philip Aaronson
Scott Aaronson
Henk Aarts
Jos Aarts
Ellen Aasum
Mohd Nadhir Ab Wahab
Jose Abad
Jorge Abad
Francisca Abad
Eduardo Abade
Fernando Abad-Franch
Fitsum Abadi
Javier Abadia
Valerie Abadie
Tatjana Abaffy
Grace Abakpa
Xesus Abalo
Inmaculada Aban
Daniel Abankwa
Jenny Abanto
Federico Abascal
Aniekan Abasiattai
Zaid Abassi
Solomon Abay
Yusuf Abba
Nathlee Abbai
Alexander Abbas
Syed Abbas
Ash Mohammad Abbas
Muhammad Abbas
Ata Abbas
Z. Abbas
Aamir Abbas
James Abbas
Faisal Abbas
Saleem Abbasi
Ali Abbasi
M. Kaleem Abbasi
Siddique Abbasi
S.A Abbasi
Jennifer Abbass-Dick
Maggie Abbassi
Jessica Abbate
Mauro Abbate
Giovanni Abbate-Daga
Souheila Abbeddou
Olivier Abbo
Susan Abbondanzo
Derek Abbott
David H. Abbott
Jessica Abbott
William Abbott
Sabra Abbott
Daniel Abbott
Andrew Abbott
Peter Abbott
Gavin Abbott
Giovanni Abbruzzese
Sophie Abby
Ekram Abd El Wahab
Ahmed Abd El Wahed
Aini Ismafairus Abd Hamid
M Abdalla
Walid Abdallah
F Abdallah
Mohamed Abdel Hakeem
Bahaa-Eldin Abdel Rahim

Mohamed Abdelbary
Mohamed Abdel-Daim
Hesham Abdeldayem
Sonia Abdelhak
Asmaa Abdelhamid
Maha Abdelhaq
Nasser Abdel-Latif
Ahmad Abdel-Mawgoud
Mohamed Abdelmegeed
Kotb Abdelmohsen
Mohamed Abdel-Rafei
Yasser Abdelrahman
Eman Abd-Elsalam
Hossam Abdelsamed
Hamdy Abdel-Shafy
Mona Abdel-Tawab
Sayed Abdelwahab
Reza Abdi
Hamidreza Abdi
Jahangir Abdi
David Abdo
Amir Abdollahi
Hamid Abdollahi
Masoud Abdollahi
Salim Abdool Karim
Ahmed S. Abdoon
Amr Abdou
Azad Abdu
Dayang Anita Abdul Aziz
Azidah Abdul Kadir
Nazia Abdul Majid
Dania Abdul Malak
M Rizal Abdul Manaf
Muhammad Abdul Wahab
Misra Abdulahi
Regina Abdulkader
Afnizanfaizal Abdullah
Maha Abdullah
Adina Abdullah
Kalil Abdullah
Jibril Abdulmalik
Aly Abdulrahim
Muhammad Kamil Abdulrahman
Samia Abdul-Rahman
Midhat Abdulreda
Mamdouh Abdulrhman
Ali Abdul-Sater
Amal Abdul-Sattar
Keiko Abe
Takeru Abe
Nobuhito Abe
Toshiaki Abe
Hiroshi Abe
Kensaku Abe
Keietsu Abe
Kohtaro Abe
Makoto Abe
Sumiyoshi Abe
Solomon Abebe
Alfonso Abecia
Nadia Abed
Haider Abdul Razzaq Abed Al-Darraji
Salma Abedelmalek
Vida Abedi
Behnoush Abedi-Ardekani
Lily Abedipour
Nima Abedpour
Dele Abegunde
Laurent Abel
Christian Abel
Brian Abel
Jonathan Abel
Sharon Abel
Thomas Abeli
Andrew Abell
Monica Abella
Gina Abelló
Shirley Abelman
Bart Aben
Ludovico Abenavoli
Michael Abend
Nicholas Abend
Heather Abercrombie
Daniel Aberdam
Matilda Aberese-Ako
Kristoffer Aberg
Avraham Abergel
Javier Aberturas
Chathurika Abeyrathne
Hima Abeysinghe
Dr Abhilash
Abhimanyu Abhimanyu
Yogindra Abhyankar
Vinay Abhyankar
Charbel Abi Khalil
Javier Abian-Vicen

Sabia Abidi
Syed Hani Abidi
Giuseppina Abignano
Gilbert Abiiro
Niels Abildgaard
Alash'Le Abimiku
Laurent Abi-Rached
John Abisheganaden
Marc Abitbol
Pedro Abizanda
Manouk Abkarian
Hinrich Abken
Dharam Ablashi
Jason Able
Carine Ablitzer
Zsolt Ablonczy
Susan Abmayr
Erin L Abner
Christian Abnet
Kingsley Abode-Iyamah
Tawfik Aboellail
Gabriella Abolafio
Mostafa A. Abolfotouh
Steve Abolins
Andras Abonyi
Raymond Aborigo
David Aborn
Hadi Abou El Hassan
Wassim Abou-Kheir
Fahmy Aboulenein-Djamshidian
Abdul Abou-Samra
Hazem Abou-Youssef
Carla Abouzahr
Iosief Abraha
Carmela Abraham
David Abraham
Nader Abraham
Wolf-Rainer Abraham
Isaac Abraham
Thomas Abraham
Sam Abraham
E.M. Abraham
Naeemah Abrahams
Heidi Abrahamse
Shafika Abrahams-Gessel
Dale Abrahamson
Arman Abrahamyan
Juan G. Abraldes
Giovanni Abramo
Michael Abramoff
Andrey Abramov
Sergey Abramov
Steven Abramowitch
Thomas Abrams
Cameron Abrams
Marshall Abrams
Shaun Abrams
Julian Abrams
Jakub Abramson
Elsa Abranches
Catarina Abrantes
Maria Abreu
José Abreu
Patrícia Antonia Estima Abreu
Ana Lucia Abreu-Silva
Maryline Abrial
Gwenael Abril
Vanessa Abril
Ravi Abrol
Nora Abrous
Waleed Abu Al-Soud
Diala Abu Awad
Asad Abu Bakar Md Ali
Hatem Abu Hashim
Ahmad Abu-Akel
Ali Abu-Alfa
Yousef Abu-Amer
Amina Abubakar
Yassir F Abubakar
A'Edah Abu-Bakar
A. Omar Abubaker
Kuerbanjiang Abuduxikuer
Sara Abuelmaali
Muhammad Abu-Elmagd
James Abugri
Sami Abuhamdeh
Liron Abuhatzira
Peter Abuja
Sumayeh Abujaber
Abde Abukhdeir
Nada Abumrad
Laith Abu-Raddad
Felipe Aburto
Jaime Aburto
Octavio Aburto-Oropeza
Loreto Abusleme

Husam Abu-Soud
Timothy Abuya
Arlet Acanda De La Rocha
Ali Acar
H. Volkan Acar
Bilgehan Acar
Rosita Accardi
Luisa Accardi
Tomaž Accetto
Horacio Acciaresi
Ettore Accolla
Roberto Accolla
Frank Accurso
Pedro Aceituno Aceituno
Marcio Acencio
Alberto Acerbi
Arianna Aceti
Paola Aceto
Reinaldo Acevedo
Miguel Acevedo
Edmund Acevedo
Ashley Acevedo
Flor Acevedo
Karla Acevedo
Melissa Acevedo
Sandra Acevedo-Gallegos
Seema Aceves
Thomas Ach
Joana Acha
Animesh Acharjee
K Ravi Acharya
Chaitanya Acharya
Kshitish Acharya
Bhoj Acharya
B.N. Acharya
Ganesh Acharya
Rajendra Acharya
Vishal Acharya
Kamal Acharya
Chirag Acharya
Maria Achatz
Rujira Achawanantakun
Arnab Achaya
Guillaume Achaz
Nicole Achee
Chad Achenbach
Ashley Acheson
Rebecca Achey
Amit Achhra
Thomas Achia
Sharon Achilles
Jean-Paul Achkar
Tom Achoki
Adnane Achour
Hammadi Achour
Mark Achtman
Jure Acimovic
Bartolomeu Acioli-Santos
Margaret Ackerman
Hans Ackerman
William Ackerman
A. Lenore Ackerman
Sandra Ackermann
Maegen Ackermann
Uwe Ackermann
Camila Ackermann
Marta-Louise Ackers
Brian Ackley
Hervé Acloque
Sandra Acosta
Cristian Acosta
Monica Acosta
Alicia Acosta
Stefan Acosta
Victor Acosta
Maricedes Acosta-Martinez
Elizabeth Acosta-Ramirez
Alvaro Acosta-Serrano
George Acquaah-Mensah
Alberto Acquadro
Rosaria Acquaviva
Claudia Acquisti
Jennifer Acton
Jose Acuin
Sergio Acuna
Dario Acuña-Castroviejo
Stefan Aczel
Akio Adachi
Hisashi Adachi
Akihiko Adachi
Paul Adachi
Makoto Adachi
Ganesan Adaikan
David Adaikkalasamy
Ishag Adam
Barry Adam

Laura Adam
Vincent Adam
Alejandro Adam
Jos Adam
Iris Adam
Éva Ádám
Antoine Adamantidis
Maria Fernanda Adame
Lubomír Adamec
Chris Adami
George Adamidis
Shelley Adamo
Vincenzo Adamo
Vic Adamowicz
Sarah Adamowicz
John Adams
Tim Adams
Paul Adams
Josh Adams
Rick Adams
Mark Adams
Volker Adams
Christopher Adams
Amy Adams
Rachel Adams
Daniel Adams
Jose Adams
Jimi Adams
Matthew Adams
Richard Adams
Henry Adams
Zachary Adams
Susan Adams
James Adams
Keith Adams
Meredith Adams
Tempe Adams
Colin Adams
Maja Adamska
Zofia Adamska
Mateusz Adamski
Zbigniew Adamski
Samuel Adamson
Grazyna Adamus
Jordi Adamuz
Roger Adan
Luis Adan
Michael Adang
Ram Adapa
Sara Adar
Eli Adashi
Stanislaw Adaszewski
Ian Adcock
Francesco Addabbo
Anna Addamo
Jagannadha Rao Addicam
Russell Addis
Maria Filippa Addis
Owen Addison
Anthony Addison
Juliet Addo
Nii Addy
Carl Ade
Sylvia Adebajo
Ezekiel Adebiyi
Adeyemi Adedeji
Dennis Adeegbe
Filomena Adega
Amanda Adegboye
Olusola Adejumo
Tameem Adel
Zach Adelman
Guillaume Adelmant
Davies Adeloye
David Adelson
Stephen Adelstein
Coen Adema
Gosse Adema
Beyene Ademe
Foluso Ademuyiwa
Adetola Adesida
Daniel Adesse
Adewole Adewumi
Adebowale Adeyemo
Sirin Adham
Neill Adhikari
Atin Adhikari
Neeta Adhikari
Kabindra Adhikari
Bishwa Adhikari
Koushik Adhikari
Till Adhikary
Gautam Adhikary
Keyur Adhvaryu
Tapan Adhya
Fadzilah Adibah Abdul Majid

Mehdi Adibi
Hadi Adibi
Satish Adiga
Anbu Adikesavan
Juma Adinan
Irit Adini
Salvatore Adinolfi
Elena Adinolfi
Luigi Elio Adinolfi
Noam Adir
Olufolake Adisa
James Adisa
Vitria Adisetiyo
Visseho Adjiwanou
Deanna L Adkins
Elizabeth Adkins-Regan
Wolfram Adlassnig
Frederick Adler
Heiko Adler
Nancy Adler
Andreas Adler
Peter Adler
Carolyn Adler
Jeremy Adler
Amos Adler
Werner Adler
Tanja Adnadevic
Sama Adnan
Serge Adnot
Stephen Adolph
Rosanna Adone
Annagrazia Adornetto
Véronique Adoue
Thierry Adoukonou
Marlou Adriaanse
Eric Adriaenssens
Troncoso-Ponce Adrian
Molly Adrian
Lopes Adriana
Indra Adrianto
Gregor Adriany
Emily Adrion
Sahil Adriouch
Daniel Adrover-Roig
Samuel Adunyah
Sahir Advani
Ryusuke Ae
Gemma Aellah
Abhinav Aeron
Joachim Aerts
Jean-Marie Aerts
Peter Aerts
Johan Aerts
Johannes Aerts
Farrukh Afaq
Niloofar Afari
El Bachir Affar
Nabeel Affara
Nicholas Affrunti
Enis Afgan
Gijs Afink
Sergiy Afonin
Claudio Afonso
Philippe Afonso
Asad Afridi
Bahmam Afsari
Neda Afsharkhamseh
Vahid Afshar-Kharghan
Rebecca Aft
Aqeela Afzal
Rizwan Afzal
Behdad Afzali
Jacques Agabriel
Abbas Agaimy
Hervé Agaisse
Jola Agaj
Ilir Agalliu
Dritan Agalliu
Brian Agan
Iman Aganj
Anette Agardh
Sunita Agarwal
Anupam Agarwal
Sudha Agarwal
Ritesh Agarwal
Manu Agarwal
Nisheeth Agarwal
Subhash Agarwal
Ameeta Agarwal
Pratul Agarwal
Koki Agarwal
Seema Agarwal
Pinky Agarwal
Supreet Agarwal
Anil Agarwal
Dinesh Agarwal

Vr Agarwal
Rachna Agarwal
Suresh Kumar Agarwal
Beamon Agarwal
Manjree Agarwal
Anila Agarwal
Deepa Agashe
Nirit Agay
Keren Agay-Shay
Martin-Paul Agbaga
Kemal Agbaht
Prince Agbedanu
Onnik Agbulut
Alper Agca
Sofia Agelaki
Walter Ageno
Arun Aggarwal
Rohit Aggarwal
Annu Aggarwal
Dau Aggarwal
Suresh Aggarwal
Monika Aggarwal
Saurabh Aggarwal
George Aggelis
Samuel Aggrey
Golareh Agha
Mohmoud Aghaei
Ada Aghaj
Lusine Aghajanova
Amirhossein Aghamohammadi
Ali Aghdassi
Alessio Aghemo
Manish Aghi
Rokhsareh Aghili
Ahmad Agil
Mehmet Agilli
Ion Agirrezabal
Hermann Agis
Paul Agius
Giorgio Aglieri
Ingi Agnarsson
Michelle Agne
Giulio Agnetti
Dalen Agnew
Navneet Agnihotri
Sergio Agnoli
Federico Agnolin
Antonella Agodi
George Agogo
Simon Agolory
Rosana Agondi
Rafiou Agoro
Jackeline Agorreta
Federica Agosta
Donat Agosti
Marco Agostini
Sylvain Agostini
Tiziano Agostini
Ilaria Agostini
Pietro Agostini
Carlo Agostoni
Massimo Agostoni
Kawango Agot
Irina Agoulnik
Dan Agranoff
Namita Agrawal
A. K. Agrawal
Swastik Agrawal
Suraksha Agrawal
Gopal Agrawal
Samir Agrawal
Gaurav Agrawal
Pavan Agrawal
Sandeep Agrawal
Sumit Agrawal
Manish Agrawal
Anil Agrawal
Vijay Agrawal
Anurag Agrawal
Arpana Agrawal
Vinayak Agrawal
Varkha Agrawal
Sangeeta Agrawal
Ruben Agrelo
Eustachio Agricola
Begoña Aguado
Inmaculada Aguado
José María Aguado
Jazmin Aguado-Sierra
Marta Aguar
Francisco Aguayo
Baltazar Aguda
Celine Aguer
Jaume Aguero
Renato Aguiar
Tatiana Aguiar

Paulo Aguiar
Fernando Aguiar
Anna Caroline Aguiar
Cristhiane Aguiar
Pedro Aguiar Junior
Gianluca Aguiari
Hector Aguila
Raúl Aguilar
Carlos Aguilar
Sebastian Aguilar Pierlé
Adriana Aguilar-Lemarroy
Antoni Aguilar-Mogas
Nancy Edith Aguilar-Olivos
Raul Aguilar-Roblero
Arturo Aguilar-Rojas
Moisés Aguilera
Renato Aguilera
Ximena Aguilera
Jose Aguilera
Leopoldo Aguilera-Aguirre
Ma. Guadalupe Aguilera-Arreola
Escolástico Aguilera-Tejero
Aitor Aguirre
Windsor Aguirre
Urko Aguirre
Sebastian Aguirre
Miquel Aguirre
Luis Aguirre
Jesús Aguirre-Gutiérrez
Luis Agulló
Tolulope Agunbiade
Blessing Agunwamba
Trevor Agus
Mirian Agus
Tetsuro Agusa
Susana Agusti Montolio
Dwi Agustian
Anna Maria Agustsdottir
Montserrat Agut
Ambrose Agweyu
Ghasem Ahangari
Anand Ahankari
Efrat Aharonov-Majar
Sean Ahearn
Christopher Ahern
Sara Ahern
Carol Aherne
Ehud Ahissar
Merav Ahissar
Per Ahlberg
Kathrin Ahlbrecht
Thomas Ahlering
Adam Ahlers
Volker Ahlers
Shawn Ahlfeld
Mark Ahlman
Tarunveer Ahluwalia
Amrita Ahluwalia
Kavita Ahluwalia
Nafees Ahmad
Iqbal Ahmad
Aasim Ahmad
Imran Ahmad
Nihal Ahmad
Shama Ahmad
Ali Ahmad
Saheem Ahmad
Basir Ahmad
Shafqat Ahmad
Faizan Ahmad
Aijaz Ahmad
Aftab Ahmad
Balsam Ahmad
Firdos Ahmad
Iman Ahmad
Parvaiz Ahmad
Farrukh Ahmad
Waqas Ahmad
A. Ahmad
Mohiuddin Ahmad
Zulfiqar Ahmad
Zaiton Ahmad
Wasim Ahmad
Atta Ahmad
Mubashir Ahmad
Riaz Ahmad
Ferhaan Ahmad
Alireza Ahmadi
Gabriela Ahmadia
Akefeh Ahmadiafshar
Ali Ahmadian
S. Ansar Ahmed
Kamruddin Ahmed
Alaa Ahmed
Naseer Ahmed
Selena Ahmed

Muhammad Ahmed
Shubbir Ahmed
Syed Masud Ahmed
Mohamed Ahmed
Luai Ahmed
Zeeshan Ahmed
Sharifah Ahmed
Ashfaq Ahmed
Chulbul Ahmed
Umar Ahmed
Tofael Ahmed
Saifuddin Ahmed
Sayem Ahmed
R. G. Ahmed
Ahmed Ahmed
Hafiz Ahmed
Ali Ahmed
Warish Ahmed
W. Ahmed
Mukhtar Ahmed
S. Faisal Ahmed
Eman Ahmed
Noha Ahmed Nasef
Ismayil Ahmet
Misol Ahn
G-One Ahn
Richard Ahn
Yong-Yeol Ahn
Kwng Seok Ahn
Curie Ahn
Sung Gwe Ahn
Dong Ahn
Sung-Ju Ahn
Myung Douk Ahn
Tae-Hyuk Ahn
Bum Ju Ahn
Woo Ahn
Daniel Ahn
Young-Hoon Ahn
Younghee Ahn
Mihye Ahn
Jooeun Ahn
Youngkeun Ahn
Ingrid Ahnesjö
Josefin Ahnstrom
Aima Ahonkhai
Bo Ahren
Dag Ahren
Dirk Ahrens
Jens Ahrens
Robert Ahrens
Tyler D. Ahrenstorff
Julie Ahringer
Nazmul Ahsan
Nita Ahuja
Manuj Ahuja
Shane Ahyong
Jinglu Ai
Huashui Ai
Tomohiko Ai
Midan Ai
Qinghui Ai
Tao Ai
Yongfeng Ai
Hiroji Aiba
Takeshi Aiba
Omowunmi Aibana
Palok Aich
Sue Aicher
Christopher Aicher
Wilhelm Aicher
Tomomi Aida
Junko Aida
Pamela Aidelsburger
Vassilis Aidinis
Luca Maria Aiello
Brett Aiello
Toshiro Aigaki
Bertrand Aigle
Achim Aigner
Michael Aigner
Ken-Ichi Aihara
Takuya Aikawa
Glen Aiken
Melissa Aikens
James Aikens
Reid Aikin
Laurie Ailles
Vishukumar Aimanianda
Gianluca Aimaretti
Flavio Aimbire
Jason Aimone
Qurrat Ain
Mattias Aine
John Ainembabazi
David Ainley

Jose Ainsa
Philip Ainslie
Steven Aird
Marta Aires-De-Sousa
Gr Aispuru
Dylan Aïssi
Tahar Ait-Ali
Hafid Ait-Oufella
Slimane Ait-Si-Ali
Tero Aittokallio
Ke Aiwu
Hiroyuki Aizawa
Toru Aizawa
Carlos Aizenman
Ayal Aizer
Felipe Aizpuru
Kanchan Ajbani
Vladeta Ajdacic-Gross
Dragana Ajdic
Marco Ajelli
Matthew Ajemian
A. Bolu Ajiboye
Olujimi Ajijola
Hiroharu Ajiro
Seena Ajit
Dharani Ajithdoss
Daniel Ajona
Kolapo Ajuwon
Dilek Ak
Rinji Akada
Mitsugu Akagawa
Keichi Akahane
Wataru Akahata
Yasuhiro Akai
Takaaki Akaike
Hiroyuki Akama
Kazahito Akama
Eliana Akamine
Takashi Akamizu
Masaharu Akao
Fatma Akar
Muhammad Sajid Hamid Akash
Sajid Hamid Akash
Yoshiki Akatsuka
Uri Akavia
Turgay Akay
Chihiro Akazawa
Yuko Akazawa
Rehan Akbani
Arne Akbar
Umer Akbar
Noreen Akbar
Hamid Akbarali
Mohammad Akbari
Schahram Akbarian
Berna Akbulut
H. Resit Akcakaya
Pinar Akcora
Guray Akdogan
Lindstrom Ake
Oluwaseun Akeju
Victor Akelo
Karl Akerman
Katherine Akers
Tobias Akerstrom
Agneta Åkesson
Bünyamin Akgül
Alireza Akhondi-Asl
Riaz Akhtar
Wajiha Akhtar
Noori Akhtar-Danesh
Yusuf Akhter
Eduard Akhunov
Tamar Akhvlediani
Toshihiko Aki
Suminori Akiba
Chaza Akik
Pierre Akilimali
Ainur Akilzhanova
Marie-Andree Akimenko
Kazuya Akimitsu
Takayuki Akimoto
Ata Akin
Ayse Akincigil
Oyediran Akinrinade
Ralitsa Akins
Oluyemisi Akinwande
Rufus Akinyemi
Tomi Akinyemiju
Sheila Akinyi Okoth
Kazuki Akira
Eyal Akiva
Yoshimitsu Akiyama
Masato Akiyama
Taniguchi Akiyoshi
Takashi Akiyoshi

Ramesh Akkina
Gokhan Akkoyunlu
Erkihun Aklilu
Muktak Aklujkar
Alp Akman
Levent Akman
Junya Ako
Jacob Akoh
Angela Akol
Pradip Akolkar
Zsuzsa Ákos
Patrick Akpaka
Ani Akpinar
S. Akpotu
Khondoker Akram
M. Usman Akram
Sahar Akram
Michel Akselrod
Ezra Aksoy
Munir Aktas
Serpil Aktas
Nasreen Akter
Shamima Akter
Klaus Aktories
Anastasios Aktypis
Shaw Akula
Shailaja Akunuru
Sercan Akyalcin
Rana Akyazi
Filiz Akyüz
Noor Al Dahhan
Ayesha Al Dhaheri
Lamia Al Naama
Nashwan Al Naiemi
Faiez Al Nimer
Nada Alaaeddine
Olufunke Alaba
Olufemi Alabi
Armin Alaedini
Kaat Alaerts
Adriana Alagna
Suresh Alahari
Fares Alahdab
Abraham Al-Ahmad
Tommy Alain
Ayodele Alaiya
Ivan Alajbeg
Petri Ala-Laurila
Shereen Al-Ali
Ziyad Al-Aly
Tausif Alam
Rafeul Alam
Feroz Alam
Mohammad Alam
Monzurul Alam
Hunain Alam
Shafiq Alam
Dewan Alam
Asim Alam
Md. Amirul Alam
Luis Alameda
Samah Alamery
Asm Alamgir
Arsham Alamian
Helene Alami-Durante
Ashraf Al-Amoudi
Alexandre Alanio
Sarah Alansari
Lubna Al-Ansary
Merve Alanyali
John Alao
Satish Alapati
Tarek Al-Arabi
Vernadeth Alarcon
Emilio I. Alarcon
Francisco Alarcon-Chaidez
Marta Alarcón-Riquelme
Michel Alary
Waddhaah Al-Asbahy
Jordi Alastruey
Ana Alastruey-Izquierdo
Ali Alatabbi
Marta Alatriste Contreras
Cédric Alaux
Juan Jose Alava
Mikko Alava
Ali Alawieh
Sandra Alba
Samir Al-Badri
Lucy Alba-Ferrara
Aitor Albaina
Rafael Albaladejo
Ricard Albalat
Amaya Albalat
Yousef Albalawi
Emiliano Albanese

Emanuele Albano
Gian Luigi Albano
Umberto Albarella
Dario Albarello
Marion Albares
Cesar Albarino
Shadi Albarqouni
Pedro Albarran
Ana Albarran-Lara
Jawdat Al-Bassam
Yakup Albayrak
Shannon Albeke
Luca Albergante
Angel Alberich-Bayarri
Kathryn Albers
Sonja-Verena Albers
Kees Albers
James Albert
Jan Albert
Jeff Albert
Joerg Albert
M. John Albert
Bensman Albert
Timothy Albert
David Albert
Gianmarco Alberti
Kurt Albertine
David Albertini
María F. Albertoni Borghese
Hanneke Alberts
Hilke Alberts-Hubatsch
Elisabetta Albi
Mattia Albiero
Roger Albin
Sandra Albino
Simone Albisinni
Maria Albo
Julie Albon
Silvia Alboni
Alessandro Alboresi
Urs Albrecht
Randy Albrecht
Philipp Albrecht
Jörg Albrecht
Dirk Albrecht
Letusa Albrecht
Jennifer Albrecht
Jan Albrecht
Benedicte Albrectsen
Werner Albrich
Lisa A Cannon Albright
David Albright
Laura-Oana Albulescu
Patricia Albuquerque
Maicon Albuquerque
Fabio Albuquerque
Philip Alcabes
Fernando Alcaide
Maria Alcaide
Héctor Alcalá
Luis Alcalá
Sergio Alcalá-Corona
Miguel Alcalde
Jose Alcalde
Ignacio Alcalde
Suzana Alcantara
José Carlos Alcantud
Luis David Alcaraz
Stefano Alcaro
Ruben Alcazar
Rosa Alcazar
Antonio Alcina
Pedro Alcolea
John Alcorn
Martin Alda
Nasser Al-Daghri
Naroa Aldanondo
Ana Aldea
Rodrigo Aldecoa
Kieran Alden
Joel Alderete
Brandon Alderman
Harold Alderman
Robyn Alders
Luke Alderwick
Bashir Al-Diri
David Aldous
Anna Aldovini
Thomas Aldrich
Daniel Aldrich
Howard Aldrich
J. Aldridge
Cameron Aldridge
Kristina Aldridge
David Aldridge
Yousif Aldryhim

Hakan Aldskogius
Carlo Aleci
Juan Aledo
Jorge Alegre-Cebollada
Fares Al-Ejeh
Ramon Alemany
Marià Alemany
Barbara Alemar
Francis Alenghat
Natalia Alenina
Maria Alera
Samir Aleryani
Vacca Ales
Laura Maria Alessandretti
Francesca Alessandrini
Edoardo Alesse
John Aletta
Kaio Alevi
Vasileia-Ismini Alexaki
Caroline Alexander
Heather Alexander
Trevor Alexander
Helen Alexander
Yvonne Alexander
Mallika Alexander
Jill Alexander
Thomas Alexander
Angela Alexander
Clark Alexander
Justin Alexander
Jennifer Alexander
William Alexander
Ruby Alexander-Lindo
Gladys Alexandre
Mebazaa Alexandre
Frederic Alexandre
Hervé Alexandre
Tiago Alexandre
Marie-Cecile Alexandre-Gouabau
Thomas Alexandridis
Konstantina Alexandropoulos
Govaris Alexandros
Evan Alexandrou
Mark Alexandrow
Gabriela Alexe
Stacey Alexeeff
Kharitonenkov Alexei
Mikhail Alexeyev
Alessio Alexiadis
Christoph Alexiou
Michael Alexis
Frank Alexis
Johanna Alexopoulos
Emil Alexov
Tamas Alexy
Dominique Alfandari
Simone Alfarano
Carlos Alfaro
Manuel Alfaro
M.C. Alfaro-De La Torre
Abdullah Al-Farraj
Judith Alferink
Carlo Alfieri
Rosa Alfonso-Rosa
Lucy Alford
Serge Alfos
Dave Algar
Russ Algar
Jamaan Alghamdi
Majid Alghatrif
Majed Alghoribi
Holly Algood
Abdalla Al-Haj
Sahal Al-Hajoj
Fatimah Alhamlan
Lena Al-Harthi
Hadi Al-Hasani
Jacob Al-Hashemi
Leena Al-Hassan
Albert Alhatem
Turki Alhazzazi
Juergen Alheit
Mohammed Al-Hubaishi
Layla Alhyas
Mohammad Ali
Nahid Ali
Mir Ali
Carine Ali
Manir Ali
Syed Ali
M. Panna Ali
Asad Ali
Naushad Ali
Nasir Ali
Akhtar Ali
Shafaqat Ali

Ishtiaq Ali
Gul Ali
Abduladhem Ali
Susanna Ali
Mustafa Ali
Golestani Ali
Tazeen Ali
Ajmol Ali
A. Ali
Basharat Ali
Abbas Ali Mahdi
A Alia
Ricardo Alia
Lorenzo Alibardi
Stefano Aliberti
Bradly Alicea
Jesús Alierta
Pietro Alifano
Marco Alifano
Sarah Ali-Khan
Siamak Alikhani
Temitope Alimi
Mauro Alini
Matthew Aliota
Jason Aliotta
Ahmadou Alioum
Antonios Aliprantis
Christian Alis
Malcolm Alison
Marianne Alison
Gordon Alison
Kari Alitalo
Noorjahan Alitheen
Noorjahan Banu Alitheen
Fabio Alivernini
Alessandro Aliverti
Gambo Aliyu
Abubakar Aliyu
Ash Alizadeh
Patrick Alizai
Bilal Al-Jaidi
Omar Aljitawi
Ron Alkalay
Noam Alkan
Leontine Alkema
Rob Alkemade
Huda Al-Khalaf
Souhaila Al-Khodor
Mahmoud Al-Khrasani
Khawla Al-Kuraya
Angelo All
Robin Allaby
Mohan Alladi
Sima Allahverdian
Jean-Pierre Allain
Fabrice Allain
Joel Allainguillaume
Florent Allais
Marco Allaix
Mohamed Allam
Jean Pierre Allam
Valérie Allamand
Alison Allan
Andrew Allan
Bridie Allan
Raymond Allan
Charlotte Allan
Craig Allan
Patrick Allard
Antoine Allard
Eric Allard
Veerajalandhar Allareddy
Lupe Furtado Alle
Karel Allegaert
Eugenia Allegra
Mario Allegra
Andrew Allegretti
Isabelle Allemand
Lee-Ann Allen
Richard Allen
Matthew Allen
Caitilyn Allen
Irving Allen
Sandra Allen
Naomi Allen
Shelley Allen
Benjamin Allen
Heather Allen
Jessica Allen
Leon Allen
Geraldine Allen
Randy D. Allen
Lee Allen
David Allen
Antino Allen
Maximilian Allen

E. Kaitlynn Allen
Ben Allen
Bryan Allen
Isabel Allen
Matti Allen
Kara Allen
Jonathan Allen
Will Allen
Melissa Allen
Daniel C. Allen
Marcus Allen
Rachele Allena
Srinivas Allena
Kristina Allen-Brady
Diane Allen-Gipson
Karin Allenspach
Morten Allentoft
Adam Allentuck
Emma Allen-Verco
Alberto Allepuz
Patricio Aller
Kristina Allers
Karina Alleva
Maria Allhorn
Julie Allickson
Ney Alliey-Rodriguez
Arthur Allignol
Juri Allik
Kelly Allison
W. Ted Allison
Andrew Allison
Rachel Allison
Thomas Allison
Melissa Allman
Jens Allmer
Mariacarmela Allocca
Rodrigue Allodji
Heather Allore
Yves Allory
Iraide Alloza
Amanda Allshouse
Richard Allsopp
Olivier Alluin
Vinoo Alluri
Carl Allwood
Jessica Alm
Alberto Alma
Asma Almaidhan
Abdulrahman Al-Malki
Andres Almansa
Emily Almberg
Carlos Almeciga-Diaz
Seba Almedawar
Abu-Bakr Al-Mehdi
Rashid Almehrizi
Raquel Almeida
Cecilia Almeida
Adelaide Almeida
Afonso Almeida
Catarina Almeida
Rafael Almeida
António Almeida
Patricia Elaine Almeida
Karen Almeida
Ana Maria Almeida
Leonardo Almeida
Danilo Almeida
Roque Almeida
Rodrigo Almeida
Ab Almeida
Marcia Almeida
Luciana Almeida
F. Q. Almeida
Ana Almeida
Hilário Almeida
Graca Almeida-Porada
Conny Almekinders
Nicole Almenrader
Jose Almirall
Eva Almiron-Roig
Sharilyn Almodovar
Ahuva Almogi-Labin
Catarina Almqvist
Kristian Almstrup
Hisham Al-Mubaid
Mohammed Alnaggar
Ali Alnahdi
Hassan Alnuaimat
Ali Alnuaimi
Alessio Alogna
Anna Maria Aloisi
Abhay Alok
Soha Alomar
Abdulrasoul Al-Omran
Jose-Manuel Alonso
Santos Alonso

Juan Alonso
Covadonga Alonso
Alejandra Alonso
Alfonso Alonso
Javier Alonso
Maria Jesus Alonso
Ana Alonso
Omar Alonso
Alvaro Alonso
Carmen Alonso
Cristina Alonso Bouzón
Virginia Alonso Roldán
Luz Maria Alonso Valerdi
Daniel Alonso-Alconada
Carlos Alonso-Alvarez
Sílvia Alonso-Alvarez
David Alonso-Caneiro
Pablo Alonso-Coello
Paloma Alonso-Magdalena
Rebeca Alonso-Monge
Ana Alonso-Varona
Michael Alosco
Kannan Alpadi
Oral Alpan
Scott Alper
Deborah Alperovitch-Najenson
Nina Alphey
Gianfranco Alpini
Deshire Alpizar
Melchor Alpízar Salazar
Vildan Alptuzun
Ahmad Alqudah
Thierry Alquier
Abdulla Al-Rawabdeh
Waddah Alrefai
Karen Alroy
Saad Alsaadi
Manal Alsaadi
Abdullah Al-Sadi
Aylin Alsaffar
Eben Alsberg
Mohamed Al-Shabrawey
Rustam Al-Shahi Salman
Mohammed Alshalalfa
Berta Alsina
Fatmah Alsolami
David Alsop
Inger Alsos
J. Alspaugh
David Alsteens
Mohammed Alsultan
Kurt Alt
Karen Alt
Andrew Alt
Arshad Altaf
Mohamed Altai
Gennaro Altamura
Sandro Altamura
Cestmir Altaner
C. Altar
Chiara Altare
Mark Altaweel
Servet Altay
Mekibib Altaye
William Altemeier
Ammar Altemimi
Elizabeth Alter
Christopher Alteri
Claudia Alteri
Fernando Altermatt
Neus Altet
Nadja Althaus
Franziska Althaus
Katharina Althaus
Kristina Althoff
Sandy Althomsons
Benjamin Althouse
Christophe Altier
Craig Altier
Andrew Altieri
Mahmut Altinbas
Sevilay Altintas
John Altman
Maria Altman
Deena Altman
Daniel Altmann
Christian Altmann
Lori Altmann
Erik Altmann
Tobias Altmann
Janine Altmüller
Deborah Altomare
Claudio Altomare
Nezam Altorok
Jaan Altosaar
Philipp Altrock

Nicole Altvater-Mackensen
Laura Altweck
Res Altwegg
Rotimi Aluko
Alessandro Alunni
Ashok Alva
Severine Alvain
Amir-Houshang Alvandi
Beatriz Alvarado
Allison Alvarado
Alvaro Alvarado
Sebastian Alvarado
Maria Alvarado Barrientos
Michael Alvard
Fernando Alvarez
Gonzalo Alvarez
Tara Alvarez
Carlos Alvarez
Susana Alvarez
Sophie Alvarez
Mar Alvarez
Luis Alvarez
Julio Alvarez
Iñaki Alvarez
Francisco Alvarez
Nadir Alvarez
Ignacio Alvarez
Juan B. Alvarez
Jessica Alvarez
Mariana Alvarez
María Álvarez
Mónica Álvarez
Diego Alvarez De La Rosa
Cesar Alvarez Gonzalez
Maria Alvarez Sanchez
José Miguel Alvarez Suárez
Christopher Alvarez-Breckenridge
Javier Alvarez-Cienfuegos
Carlos Álvarez-Dardet Díaz
Daniel Alvarez-Fischer
Begoña Alvarez-Gonzalez
Juan Raúl Alvarez-Idaboy
Carmen Alvarez-Lorenzo
Jose Manuel Álvarez-Martínez
Ana Isabel Alvarez-Mercado
Luis Alvarez-Vallina
Fernando Alvarez-Vasquez
Domenico Alvaro
Rômulo Alves
Rui Alves
Frauke Alves
Sandro Alves
Joao Alves
Sandra Alves
Alberto Alves
Nuno Alves
Monica Alves
Cecília Alves
Paulo Alves
Jorge Alves
Nb Alves
Davi Alves
Marta Alves Da Silva
Marcio Alves-Ferreira
Estevao Alves-Silva
Berti Alvise
Stephen Alway
Frederike Alwes
Sharif Aly
Miriam Aly
Akram Alyass
Irina Alymova
Andrei Alyokhin
Christian Alzheimer
Raúl Alzogaray
Francisco Amado
Norma Amador-Licona
Daniel Amador-Noguez
Alicia Amadoz
T Amagai
S Amalfitano
Derek Amanatullah
Praveen K. Amancha
Diego Amancio
Arno Amann
Benedikt Amann
Mariane Amano
Masashi Amano
Koichi Amano
Farzaneh Amanpour
Arjun Amar
Khaled Amara
Olavo Amaral
Luís Amaral
Flavio Amaral
André Amaral

Isabel Amaral
David Amarantini
Kaushalya Amarasekare
Gaya Amarasinghe
Sergi Amaro
S Amatachaya
Francesca Amati
Alberto Amato
Valdir Amato
Paula Amato
Katherine Amato
Francesco Amato
Mary Amato
Patricia Amavet
Enrique Amaya
Peter Amaya
Eric Amazeen
Suleiman F. Ambali
Rajesh Ambasudhan
Kiran Ambatipudi
Alemayehu Amberbir
Gregory Amberg
Sandeep Amberkar
Richard Ambinder
Gareth Ambler
Ben Ambridge
Peter Ambros
Stanley Ambrose
Divakar Ambrose
Vincent Ambrosia
Ettore Ambrosini
Geza Ambrus
Stefan Ambs
Suresh Ambudkar
Shazhan Amed
Stephanie Ameis
Aurely Ameller
Giovanni Amendola
Brad Amendt
Peter Amenta
Johnny Amer
Aliaa Amer
Amal Amer
Kurosh Ameri
Pietro Ameri
Nancy Ames
Tohti Amet
Patricia Ame-Thomas
Simon Amiard
Fernanda Amicarelli
Mohamed Amiche
Augusto Amici
Daniel Amick
Rivet Amico
Patrizia Amico
Brett Amidan
Mansoor Amiji
Marcel Amills
Janaki Amin
Mohsen Amin
Muhammad Amin
M. G. Mostofa Amin
Saber Amin Yavari
Sow Amina
Sepideh Amin-Hanjani
Mitra Amini
Minoo Aminian
Kamiar Aminian
Norhaniza Aminudin
Samreen Amir
Israel Amirav
Alireza Amirbaigloo
Steve Amireault
Esmaeil Amiri
E. Amirian
Yuri Amirkhanian
A. Amirkhosravi
Lucy Amissah
Yali Amit
Noh Amit
Gabi Amitai
Eisuke Amiya
Hope Amm
Nischala Ammannagari
El-Desouky Ammar
Sonia Ammar
Martine Ammassari-Teule
Elske Ammenwerth
Hermann Ammer
James Ammeran
Robert Ammerman
Ole Ammerpohl
G. Paul Amminger
Paolo Ammirante
Enrico Ammirati
Mario Ammirati

John Ammori
Luisa Amo
Mary Amoakoh-Coleman
Winfried Amoaku
Gabriela Amodeo
Antonio Amodeo
Diva Amon
Maria Joao Amorim
Felipe Amorim
Adelina Amorim
João Amorim
Lucia Amoruso
William Amos
Christopher Amos
John Amos
Zahir Amoura
Francisco Amparo
Luca Ampollini
Ofer Amram
Abdelaziz Amrani
Rohit Amritanand
Ribka Amsalu
Ben Amsel
Charles Amsler
Ursula Amstutz
Sebastian Amsuess
John Amuasi
Dickson Amugsi
Florin Amzica
Ping An
Ruisheng An
Yong-Qiang (Charles) An
Seong An
Xinmin An
Ruopeng An
Yom An
Gang An
Jingang An
Haizhong An
Yu An
Lili An
Wei An
Chansik An
Hongyu An
Zhenyi An
Le An
Jing An
Qianli An
Qi An
Lingling An
Wenfeng An
Ramon Anadon
Evdokia Anagnostou
Paras Anand
Madhur Anand
Sanjay Anand
Shuchi Anand
Vibha Anand
Paul Anand
Manisha Anand
Bela Anand-Apte
Sophia Ananiadou
Meena Ananthanarayanan
G. Anantharamaiah
Sirinart Ananvoranich
Hany Anany
Abdulaziz Anas
George Anasontzis
Thomas Anastasio
Natasa Anastasov
Konstantinos Anastassiadis
K. Anastos
Vikas Anathy
Takashi Anayama
Benedict Anchang
Leonardo Ancillotto
Dan Anco
Marc Ancrenaz
David Andaluz-Ojeda
Cheryl Andam
Nelly Andarawis-Puri
Martina Andellini
Ladislav Andera
Robert Anderberg
Leander Anderegg
Ulf Anderegg
Gregor Anderluh
Mark Andermann
Robin Anders
Juanita Anders
Christoph Anders
Nicole Anders
Alan Andersen
John Andersen
Robert Andersen
Aase Andersen

Nicholas Andersen
Jeremy Andersen
Judith Andersen
Kathryn Andersen
Erik Andersen
Lars Andersen
Ab Andersen
Roxane Andersen
Peter Andersen
Barbara Andersen
Lasse Andersen
Elizabeth Andersen
Vibeke Andersen
Paul Anderson
Kathryn Anderson
Larry Anderson
David Anderson
Colin Anderson
Jean Anderson
Deborah Anderson
John Anderson
O. Roger Anderson
Lorinda Anderson
Robin Anderson
Patrick Anderson
Michael Anderson
Alisha Anderson
James Anderson
Adam Anderson
Eric Anderson
Brian Anderson
William Anderson
Richard Anderson
Bradley Anderson
Jeffrey Anderson
Carryn Anderson
Robert Anderson
Jason Anderson
Charles Anderson
Jennifer Anderson
Craig Anderson
Pamela Anderson
Peter Anderson
Christopher Anderson
Alyssa Anderson
James R. Anderson
Elizabeth Anderson
Kristin Anderson
Mark Anderson
Kevin Anderson
Kristy Anderson
Kerry Anderson
Joel Anderson
Hugh Anderson
George Anderson
Kristen Anderson
Laura Anderson
Cajsa Anderson
Gregory Anderson
Roger Anderson
Bradford Anderson
Ann Anderson-Berry
Kristina Anderson-Teixeira
Björn Andersson
Göran Andersson
David Andersson
Magnus Andersson
Karl-Erik Andersson
Daniel Andersson
Karin Andersson
Jesper Andersson
Inger Andersson
John Andersson
Lars-Magnus Andersson
Philip Anderton
David Andes
Anand Kumar Andiappan
Thomas Andl
Kiyoshi Ando
Istvan Ando
Soichi Ando
Yuichi Ando
Kiyohiro Ando
Tatsuro Ando
Hiroyasu Ando
Jamila Andoh
T. Andoh
Pulung Nurtantio Andono
Rumen Andonov
Magi Andorra
David Andow
Jackie Andrade
Paula Andrade
Maxuel Andrade
Roberto Andrade
José Luis Andrade

Francisco Andrade
Arturo Andrade
Andrey Andrade
Leonardo Andrade
José Dilermando Andrade Filho
Jose Andrade Jr.
Valter Andrade Neto
Miguel Andrade-Navarro
Aline Andrade-Silva
Johanna Andrae
Peter Andras
Ibolya Andras
Michele Andrasik
Prabha Andraweera
Ingemar Andre
Nicolas Andre
Zandra Andre
Andrea Andrea Rinaldi
Theodore Andreadis
Stelios Andreadis
Elisabeth Andreadou
Ioanna Andreadou
Jessica Andreani
Paul Andreassen
Trygve Andreassen
Maria Grazia Andreassi
Maria Grazia Andreassi
Katrin Andreasson
Sten Andreasson
Gabriele Andreatta
Eran Andrechek
Michael Andreeff
Claudio Andreeti
Victor Andreev
Viktoria Andreeva
Serge Andréfouët
Claire Andréjak
Henrik Andren
Pietro Andreone
Daniela Andres
Fernando Andres
Michael Andresen
Angeline Andrew
Deborah Andrew
Erin Andrew
Audra Andrew
Zeitlin Andrew
Margaret Andrew
Russell Andrews
Nick Andrews
Timothy Andrews
William Andrews
Gavin Andrews
Steven Andrews
Zane Andrews
Elizabeth Andrews
Kimberly Andrews
Sally Andrews
Chris Andrews
Peter Andrews
Anne M. Andrews
Joel Andrews
Helene Andrews-Polymenis
Pierpaolo Andriani
Ramaroson Andriantsitohaina
Nathalie Andrieu-Abadie
Giulia Andrighetto
Regis Bruni Andriolo
Leslie Andritsos
Angelo Andriulli
Lazaros Andronis
E. Andronov
Oleh Andrukhov
Hagen Andruszkow
Jan Andrysek
Isabel Andújar
Jan Andzelm
Ritu Aneja
Praju Vikas Anekal
Filomena Anelli
Gianfranco Anfora
C. Wim Ang
Marcus Ang
Kai Keng Ang
Mei-Kim Ang
Rafael Angarita
Jonathan Angel
Sergio Angel
Nathan Angel
Manuel Angeles-Castellanos
Peter Angeletti
Davide Angeletti
Silvia Angeletti
Fabio Angeli
Veronique Angeli
Francesco Angelico

Apostolos Angelidis
Anne Angelillo-Scherrer
Gianni Angelini
Christine Angelini
Lefteris Angelis
D. Angelis
Alex Angell
Tommaso Angelone
Arild Angelsen
Alessandra Angelucci
Emanuele Angelucci
Francesco Angelucci
Largus Angenent
Paul Angermeier
Manuela Angioi
Chaisiri Angkurawaranon
Eduardo Angles-Cano
Jeromy Anglim
Denis Angoulvant
Evelina Angov
Pierre-Olivier Angrand
Alessandro Angrilli
Joaquin Anguera
Sergio Anguissola
Juan Anguita
Elena Angulo
Javier Angulo
Jaime F. Angulo-Mora
Brian Angus
Daniel Angus
J Angus
Simon Angus
Jacob Anhøj
Robert Anholt
Luis Anibarro
Lalin Anik
Yonca Anik
Laurent Aniksztejn
Stefano Anile
Gopalakrishnapillai Anilkumar
Lesya Anishchenko
Andrey Anisimov
Abu Syed Md Anisuzzaman
Eduardo Anitua
Fernando Anjos-Afonso
Muna Anjum
Ashiq Anjum
Tycho Anker-Nilssen
Adrian Ankiewicz
Augustine Ankomah
Pim Ankum
David Ann
Digilio Anna
Pink Anna
Pieter Annaert
Wim Annaert
Damodaran Annamalai
Laura Annaratone
Vito Annese
Brian Annex
Alessia Annibale
Peter Annighöfer
Jean-Marie Annoni
Giorgio Annoni
Takeshi Annoura
Cm Annunziata
Clorinda Annunziata
Andrey Anokhin
Jennifer Anolik
Ephraim Ansa-Addo
Evelyn Ansah
John Ansah
Israr-Ul Ansari
Hifzur Ansari
Ali Ansari
Sardar Ansari
K. Mark Ansel
Dean Ansell
J. Ansermino
Sten Anslan
Emmanuel Ansong
Richard Ansorge
Hermann Ansorge
Lynn Anspaugh
Matthew Anstey
Nicola Anstice
Caterina Ansuini
Andrea Antal
Taras Antal
Tiago Antao
Alli Antar
Douglas Antczak
Adam Antebi
Gregory Antell
Haike Antelmann
Leandro Antero

Alison Antes
Iris Antes
Marios Anthimopoulos
James Anthony
Richard Anthony
Donald Anthony
Nicola Anthony
Cousien Anthony
Whitney Anthonysamy
Rustom Antia
Jonathan Anticamara
Daniele Antichi
Abigail Antigua
Joseph Antin
Marlies Antlanger
Michael Antle
Marina Antoch
Gerald Antoch
Cinzia Antognelli
Bénédicte Antoine
Daniel Antoine
Thessicar Antoine
Sophie Antoine-Jonville
Michael Antolin
Salome Antolin
D. Antolová
Dalibor Antolovic
Sylvia Anton
Stephen Anton
Martina Anton
Bernat Anton
Halina Anton
Gabriele Anton
Amina Antonacci
Alessandro Antonelli
Maria Antonelli
Raffaele Antonelli Incalzi
Anthony Antonellis
Zeus Antonello
Marianna Antonelou
Costin Antonescu
Michael Antoni
Chrystalina Antoniades
Mark Antoniades
Anastasia Antoniadou
Silvio Antoniak
Cristina Antoniali
Frank Antonicelli
Carla Antonio
Diez-Juan Antonio
Alberto Antonioni
Georgios Antonoglou
Annika Antonsson
Emilia Antonucci
Serena Antonucci
David Antonuccio
Veena Antony
Christopher Antos
Michael Antosh
Jedrzej Antosiewicz
Kevin Antshel
Agostinho Antunes
Mauricio Antunes
Edson Antunes
Flora Antunes
Andre Antunes
Alberto Antunes
Julie Antvorskov
Werner Antweiler
Markus Antwerpen
Rodolphe Anty
Justus M. Anumonwo
Krishnamurthy Anupama
S. Anuradha
Anupkumar Anvikar
Haroon Anwar
Diana Chinyere Anyaogu
Toshihisa Anzai
Yoshimi Anzai
Michele Anzidei
Serenella Anzilotti
Joshua Anzinger
Hushan Ao
Mingfang Ao
Mingxin Ao
Ping Ao
Tomohiko Aoe
Quyen Aoh
Hiroki Aoki
Junken Aoki
Jun-Ya Aoki
Yasunori Aoki
Tatsuo Aoki
Koh Aoki
Kiyoko Aoki-Kinoshita
Fawzi Aoudjit

Alexei Aouriadov
Kunihiko Aoyagi
Mineyoshi Aoyama
Takuma Aoyama
Paschal Apanga
Lucas Aparicio
Ágota Apáti
Ludwig Apers
Sebastian Apesteguia
Cristian Apetrei
Coren Apicella
A Apkarian
Daniel Apolinario
Francesca Apollonio
John Apolzan
Sokratis Apostolidis
Bénédicte Apouey
Emil Appel
Lieuwe Appel
Lior Appelbaum
Marc Appelhans
Michael Appell
Bernard Appiah
Paul Appleby
Brian Appleby
Tanya Applegate
Richard Applegate
Wendy Applequist
Jessica Appler
Jose Appolinario
Matthew Apps
Michael April
Giuseppe Aprile
Francesco Aprile
Suneel Apte
Advait Apte
Udayan Apte
Swapna Apte
Deborah Apthorp
André Aptroot
Onur Apul
Rami Aqeilan
James Aquavella
Saveria Aquila
Victor Aquino
Jorge Aquino
Gabriella Aquino
Johan Åqvist
Marzieh Araban
Mohammad Arabestani
Michal Arabski
Enrique Aracil
Michael Arad
Filipa Aragão
Henio Aragão
Tomas Aragon
Virginia Aragon
Andrés Aragón Martínez
Brandon Aragona
Ayako Arai
Manabu Arai
Yoichi Arai
Lisa Arai
Richard Arakaki
Hirofumi Arakawa
Satoko Arakawa
Gayathri Arakere
Gururaj Arakeri
Shin-Ichi Araki
Marito Araki
Nobukazu Araki
Yoshihiko Araki
Yuya Araki
Eiji Aramaki
Adamantos Arampatzis
Christian Aran
Walter Arancio
Manuel Aranda
Agustin Aranda
Silvia Aranda
Federico Aranda
Mimi Arandjelovic
Amelia Aránega
Diego Arango
Vidya Arankalle
Ana Aransay
Tamas Aranyi
Toshikazu Araoka
Siddhesh Aras
Ramesh Arasaradnam
Hisashi Arase
Haghikia Arash
H. S. Arathi
Astolfo Araujo
Arturo Araujo
Ronaldo Araujo

Leonardo Araujo
Ivan Araujo
Alexandra Araujo
Aurigena Araújo
António Araújo
Joana Araújo
Gerson Araujo De Medeiros
Raimundo Araújo Júnior
Amir Arav
Ravit Arav-Boger
Andres Aravena
Vivekanandhan Aravindhan
Shigeki Arawaka
Sean Arayasirikul
Ozgur Araz
Armin Arbab-Zadeh
Yael Arbel
Caroline Arber Barth
Javier Arbizu
Adrià Arboix
Joseph Arboleda
Maria Lourdes Arbones
Jose Arbones-Mainar
Victoria Arbour
Benjamin Arbuckle
Patrick Arbuthnot
Gordon Arbuthnott
Luca Arcaini
Stefano Arcangeli
Alexandre Arcaro
Elsa Arcaute
Stephen Arce
Roger M. Arce
Patricio Arce-Johnson
Jon Arcelus
Juan Arcelus
Gerardo Arceo-Gomez
Elisa Arch
Karolina Archacka
Jacques Archambault
Annie Archambault
Simon Archer
Edward Archer
Debra Archer
G. Archer
Will Archer
Bruce Archibald
L. K. Archibald
Elizabeth Archie
Sotirios Archontoulis
Govindaraju Archunan
Biagio Arcidiacono
Nicola Arcilla
Vickery Arcus
Babak Ardekani
Reza Ardekani
David Ardell
Rosalind Arden
Chris Ardern
Amir Ardeshir
Amin Ardestani
Anna Ardevol
Daniel Ardia
Luca Ardigò
Alex Ardila-Garcia
Leopoldo Ardiles
Alexandria Ardissone
Moshe Arditi
Navid Ardjomand
Jeff Ardron
Arduino Arduini
Estela Area
Ana Paula Arêas
Francisco Areces
Antonio Arechar
Farzi Arefi-Khonsari
Badrul Arefin
Kathryn Arehart
Olatunde Aremu
Chris Arena
Carmen Arena
Jesús Arenas
Miguel Arenas
Francisco Arenas
Juan Arenas-Jimenez
Cesar Arenas-Mena
Douglas Arenberg
Danny Arends
Jan Arends
Josephine Arendt
Lisa Arendt
Rafael Arenhart
Peter Arensburger
Shawn Arent
Matthew Arentz
J. J. Arenzon

Gustavo Ares
Gaston Ares
Veronika Areskoug
Hermenegild Arevalo
Cynthia Arfken
Frank Arfuso
Shlomo Argamon
Martina Argano
Andrea Argentini
Maria Argos
Kelley Argraves
Maria Argudin
Debbie Argue
Juan-Carlos Argüelles
Martin Arguin
David Argyle
Panos Argyrakis
Anthoula Argyri
Thodoris Argyriou
Jason Argyris
Christos Argyropoulos
Kristopher Arheart
Stelios Arhondakis
Pablo Arias
M. Arias-Estévez
Tomoki Arichi
Tsutomu Arie
Frederic Ariey
Mohammad Arif
Muhammad Arif
Shabina Ariff
Sm Niaz Arifin
Hiroyoshi Ariga
Katsuhiko Ariga
Emma Arigi
Ingrid Arijs
Deniz Arikan
Bülent Arikan
Kentaro Arikawa
Jyothi Arikkath
Eugenio Arima
Hisatomi Arima
Taro Arima
Mary Arimond
Yoshiaki Arimura
Takuro Arimura
Helena Ariño
Ganiyu Arinola
John Aris
Anna Aris
Elif Damla Arisan
Marios Aristophanous
Kirill Aristovich
Minetaro Arita
Mikio Arita
Chikashi Arita
Miguel Ángel Ariza Gracia
Juan Arizaga
Vincenzo Arizza
Krishna Priya Arjunan
Elizabeth Arkema
Alexander Arkhipkin
Kimberly Arkin
Natalie Arkus
Mònica Arman
Samuel Arman
Ines Armando
Olivier Armant
Antonio Armario
Ioanna Armata
Peter Armbruster
Chris Armbruster
Jean Armengaud
M. Eugenia Armengod
Nigel Armfield
Paul Armistead
Charles Armitage
John Armour
Anthony Armson
Ian Armstead
Glen Armstrong
Scott Armstrong
Ben Armstrong
Philip Armstrong
Matthew Armstrong
Nicola Armstrong
Hilary Armstrong
Judith Armstrong
Lois Armstrong
Jane Armstrong
Stuart Armstrong
Sara Armstrong
Paul Armstrong
D. Armstrong Briley
Darius Armstrong-James
Arunmozhiarasi Armugam

Arunmoziarasi Armugam
Paul Arnaboldi
Isabelle Arnal
Alexei Arnaoutov
John Arnason
Ulfur Arnason
Philippe Arnaud
Sophie Arnaud-Haond
Filip Arnberg
Stepfan Arndt
Patrick Arndt
Michael Arndt
Volker Arndt
Jason Arndt
Alberto Arnedo
Miquel Arnedo
Todd Arnedt
Karen Arnell
Ezequiel Arneodo
Elias Arner
Erik Arner
Enrico Arneri
Isabelle Arnet
Tim Arnett
Norman Arnheim
Heinz Arnheiter
Gavin Arno
Steven Arnocky
Derek Arnold
Walter Arnold
Wolfgang Arnold
Emily Arnold
Paul Arnold
Lauren Arnold
Daniel Arnold
Forest Arnold
David Arnold
Suzanne Arnold
Solvi Arnold
Robert Arnold
Mark Arnold
John Arnold
Leonard Arnolda
Kathleen Arnolds
Maria Arnone
Danilo Arnone
Jon Arnot
Shelley Arnott
Damien Arnoult
Christophe Arnoult
Eric Arnoys
Paul Arnstein
Charles Arntzen
Isabelle Arnulf
Christopher Arnusch
Hannu Aro
Arja Aro
Jose Daniel Aroca-Aguilar
Luiz Aroeira
Serge Aron
Arthur Aron
Justin Aronoff
Stephen C. Aronoff
Stella Aronov
Bruce Aronow
Wilbert Aronow
Kristan Aronson
Myla Aronson
Ripla Arora
Karunesh Arora
Rishi Arora
Teresa Arora
Jyoti Arora
Rohit Arora
Rajeev Arora
Ritu Arora
Oneida Arosarena
Joe Arosh
Paolo Arosio
Ahmad Arouri
Grazia Arpino
Otso Arponen
Anni Arponen
Nailet Arraiz
Gloria Arratia
Armando Arredondo
Abdelilah Arredouani
Sergio Arregui
M. Arrese
Agnes Arrey
Protus Arrey Tarkang
Sonia Arriaga
Rodrigo Arriagada
Mari Carmen Arriaza
Marie Arrieta
André-Patrick Arrigo

Nicole Arrigo
Alessandro Arrigo
Teresa Arrigo
Roberto Arrigoni
Elise Arrivé
Sarah Arron
Juan Arroyo
Alicia Arroyo
Rossana Arroyo
David Arroyo
D. Arroyo
Jorge Arrubla
Eurico Arruda
Luciana Arruda
Paulo Arruda
Rafael Arruda
Andoni Arruti
Elisabet Ars
Marie Arsalidou
Joanne E. Arsenault
Florence Arsène-Ploetze
Hassan Arshad
Mehreen Arshad
Steve Arshinoff
Ruben Arslan
Ayca Arslan-Ergul
Marta Artal-Sanz
N. Sertac Artan
Gavin Arteel
Panagiotis Artemiadis
Antreas Artemiou
Gleb Artemov
Jeffrey Arterburn
Sylvaine Artero
Paul Artes
Hilana Artese
James Arthos
J. Simon Arthur
Agnes Arthur
Terrance Arthur
Janelle Arthur
Sit Arthur
Steven Arthurs
Mara Artibani
Antonio Artigas
Katharina Artinger
Guilherme Artioli
Katie Artis
Carol Artlett
Isabella Artner
Roberto Artoni
Ferruh Artunc
Per Artursson
Derek Artz
Marc Artzrouni
Augustine Arukwe
Bernard Arulanandam
Banu Arun
Vadivel Arunachalam
Wilson Aruni
Constandina Arvanitis
S. Arvedsen
Stefan Arver
Cindy Arvidson
Daniel Arvidsson
Akalpita Arvindekar
Ehsan Aryan
Marta Arzarello
Higinio Arzate
Boaz Arzi
Eduardo Arzt
Rene Arzuffi
Shahar Arzy
Sylvia Asa
Minoru Asada
Kamal Asadi
Niaz Asadullah
Hiroshi Asahara
Yasuhiro Asahina
Kenta Asahina
Kinji Asahina
David Asai
Akira Asai
Tetsuya Asakawa
Deanna Asakawa
Tomiko Asakura
Munehiro Asally
Katsura Asano
Yoshihiro Asano
Ryosuke Asano
Shinji Asano
Atsushi Asano
Katsuhiko Asanuma
Yu Asanuma
Kei Asayama
Hugues Aschard

Briseis Aschebrook-Kilfoy
Andy Aschenbrenner
Judith Ascher
Giuseppe Asciutto
Marina Ascunce
S. Asefi-Najafabady
Fredrika Asenius
Norberto Asensio
Sassan Asgari
Soheila Asgari
Hilmir Asgeirsson
Muhammad Asghar
Mohammad Asghar
Seyyed Mohsen Asghari
Hosseinali Asgharian
Arlene Ash
Jessica Ash
John Ash
Yacov Ashani
Andrea Ashbaugh
John Ashburner
Michael Ashby
Sarit Ashckenazi-Polivoda
David Asher
Roy Asher
Elad Asher
Ruth Ashery-Padan
Muhammad Ashfaq
Michael Ashford
Julian Ashford
Akira Ashida
Motoyuki Ashikari
Angel Ashikov
Muhammad Waseem Ashiq
Keyoumars Ashkan
Neal Ashkanasy
Jason Ashley
Laura Ashley
Patricia Ashley
Noah Ashley
Paul Ashley
Ramkumar Ashokkumar
Thanganadar Ashokkumar
Haitham Ashoor
Ahmed Ashour
Nicole Ashpole
Muhammad Ashraf
Nasheeman Ashraf
Mohammad Ashraf
M. Ashraf
Hamid Ashrafi
Manzar Ashtari
Amit Ashtekar
Thomas Ashton
Philip Ashton
Lee Ashton
Nick Ashton
Margaret Ashwell
Laureano Asico
Michael Asiedu
Marina Asiedu
Huma Asif
Mehar Asif
Benon Asiimwe
Brenda Asiimwe-Kateera
Timo Asikainen
Gershim Asiki
Mohammad Asim
Susana Asin
Ninitha Asirvatham-Jeyaraj
Kjetil Ask
Mohammad Sadegh Askari
Graham Askew
Ian Askew
Nikos Askitas
Maria Askmyr
Katie Askren
Muhammad Aslam
Alper Aslan
Parisa Aslani
Aaron Aslanian
Oleg Aslanidi
Stella Aslibekyan
Richard Aslin
Reto Asmis
David Asmuth
Anu Asnaani
Vahid Asnafi
Laura Asnaghi
Yoshimasa Aso
Taka-Aki Asoh
Ahmad Asoodeh
Eleni Asouti
Thor Aspelund
Lisa Asper
Patricia Aspichueta

Fiona Aspinal
Esther Aspinall
John Asplin
Linnéa Asplund
Johannes Asplund-Samuelsson
Terry Aspray
E. K. Asprodini
Mark Asquith
Nigel Asquith
Tufik Assad
Fariba Assadi-Porter
Suheir Assady
Frederic Assal
Bouchra Assarag
Shervin Assari
Shervin Assassi
Tarik Asselah
Gianni Assenza
Michael Assfalg
Guillaume Assie
Johanna Assies
Volker Assing
Rafael L. Assis
Lorenz Assländer
Peter Assmann
Alexander Assmann
Birgit Assmus
Richard Assoian
Sabrina Assoumou
Jamil Assreuy
José Afonso Guerra Assunção
Giuseppe Astarita
Ayalew Astatkie
Jon Aster
Margaret Astin
Susana Astiz
Laura Astola
Stefania Astolfi
Eric Aston
Anna Astorga
Todd Astorino
Elaine Astrand
Renato Astray
Sophie Astrof
Edwin Asturias
Giovanni Astuti
Sebastian Asurmendi
Erkin Asutay
Dana Aswad
Amit Aswasthi
Baris Ata
Kamran Atabai
John Atack
Ricardo Ataíde
Koray Atalag
Joel Atallah
Sergei Atamas
Michael Atamian
Kalina Atanasova
Santosh Atanur
Michael Atchison
Ruth Ann Atchley
Victoria Ateca
Bushra Ateeq
Jelle Atema
Kostantinos Athanasakis
Georgios Athanasiadis
Mohammad Athar
Davenport Athena
Sai Krishna Athuluri-Divakar
Vasilios G. Athyros
Jolene Atia
Yemil Atisha-Fregoso
Stephen Atkin
Andrew Atkin
Audrey Atkin
John Atkins
Gerald Atkins
Salla Atkins
Nigel Atkinson
Anthony Atkinson
Quentin Atkinson
Carl Atkinson
Prescott Atkinson
Jennifer Atkinson
Victoria Atkinson
Paul Atkinson
Greg Atkinson
Daphne Atlas
Ella Atlas
Akin Atmaca
Robert Atmar
Haruyuki Atomi
Sara Aton
Pradeep Atrey
Hanudatta Atreya

Chintamani Atreya
Barbara Atshaves
Yoshitaka Atsumi
Ikiru Atsuta
Giuseppe Attanasi
Roberta Attanasio
Laura Attanasio
Renuka N. Attanayaka
Anjika Attanayake
Elham Attaran
Hrayr Attarian
Arthur Attema
Marco Atteritano
Annarita Atti
Ahmed Attia
Engi Attia
Graeme Attwood
Prabhani Atukorale
Donald Atwood
Martin Atzmueller
Marco Atzori
Kin Fai Au
Man Ho Au
Shiu Lun Ryan Au Yeung
Nicholas Au Yong
Alexander Auad
Fernando Auat Cheein
Antoine Aubeneau
Geraldine Aubert
Maxime Aubert
Michael Aubertin
Thierry Aubin
Carl Eric Aubin
Nadia Aubin-Horth
Patrick Aubourg
Sylvain Aubry
Alexandra Aubry
Georg Auburger
Sarah Auburn
Francoise Auchere
Dominick Auci
Karine Auclair
Anne-Marie Aucour
Vincent Audard
Kris Audenaert
Julie Audet
Carolyn Audet
Laura Audi
Jon Audia
Francois Audibert
Stéphane Audic
Lívia Audino
Paolo Audisio
Michel Audran
Neil Audsley
Etienne Audureau
Asta Audzijonyte
Manfred Auer
Carol Auer
Reto Auer
Christian Auer
Johann Auer
Randy Auerbach
Victoria Auerbuch
Elza Auerkari
Prasert Auewarakul
Cédric Auffray
Christoph Aufricht
Charles Augarde
Josep M. Auge
Jacques Auger
Christopher Auger
Robert Aughey
Denis Augot
Claudia Augste
Martin Augsten
Albert Auguste
Salvador Augustin
Jorg Augustin
Erika Augustine
Antonios Augustinos
Rosângela Augusto
Laurent Augusto
Danillo Augusto
Yannick Aujard
Ikramuddin Aukhil
Vanessa Auld
Tony Auld
Stuart Auld
Sara Auld
Garry Auld
Alexander Aulehla
Anna Aulinas
Jerald Ault
Dagfinn Aune
Myo Aung

Robin Aupperle
Christiane Auray-Blais
Filippo Aureli
Didier Aurelle
Tibor Aurer
Salvatore Auricchio
Ferdinando Auricchio
Renata Auricchio
Christine Aurich
Bernard Auriol
Rajeev Aurora
Igor Aurrekoetxea
Gabriele Ausiello
Marcel Ausloos
Laurent Aussel
Gabriela Aust
Sabine Aust
Marie Austdal
Brian Austen
Robert Austin
Caroline Austin
Jeremy Austin
Eric Austin
Brian Austin
Judy Austin
Bryn Austin
Ryan Austin
Elena Austin
Gregory Austin
Jehannine Austin
Chris Austin
Richard Auten
I. B. Autenrieth
Stella Autenrieth
Chantal Autexier
Matthieu Authier
Ludovic Autin
Matthew Auton
Flavia Autore
Brigitte Autran
Joseph M. Autry
Taina Autti
Bertran Auvert
Edward Auyang
Bice Avallone
Esperanza Avalos-Díaz
Mauro Avanzi
Aurore Avargues-Weber
Mehmet Avcar
Selmir Avdic
Thiago Avelino-Silva
Allen Aven
Alessio Avenanti
Leonel Avendaño-Reyes
Bruno Averbeck
Michelle Averill
Diana Averill-Bates
Marc Avey
Mark Aveyard
Konstantinos Avgoustakis
Anshul Avijit
Andrea Avila
Daiana Avila
Viridiana Avila
Maria Ávila-Arcos
Luis Avila-Cabadilla
Federico Avila-Moreno
German Avila-Sakar
Malcolm Avison
Daniele Avitabile
Avi Avital
Richard Aviv
Limor Avivi-Arber
Alberto Avolio
Jérôme Avouac
Karen Avraham
Russell Avramenko
Stevan Avramov
Stephane Avril
Natalia F Avrova
Anna Avrova
Dimitrios Avtzis
Uma Mahesh Avula
Rasmi Avula
Tiong Gim Aw
Lara Awad
Muhammad Awais
Ahmed Awaisu
Reda Awali
Tahir Awan
Mehnaz Awan
Khalijah Awang
Amit Awasthi
Anjali Awasthi
Sanjay Awasthi
Sita Awasthi

Suyash Awate
Rajeshwar Awatramani
Midori Awazu
Alexander Awgulewitsch
Phyllis Awor
M. Rabiul Awual
Hibah Awwad
Roers Axel
Kevin Axelrod
Jonas Axelsson
Jan Christoph Axmacher
Eva Axner
Robert Axtell
Cihan Ay
Recep Ay
Ferhat Ay
Koichiro Aya
Abdel Ayadi
Hiyoshi Ayako
Julio Ayala
Yaneri A. Ayala
Marcela Ayala
Sylvette Ayala-Peña
Amir Ayali
Korcan Ayata
Hasan Ayaz
Muhammad Ayaz
Ozge Aydemir
Suleyman Aydin
Yusuf Aydogdu
Aydogan Aydogdu
Sergul Aydore
Myint Aye
Naziha Ayeb
Adejumoke Ayede
Don Ayer
John Ayers
Banu Aygun
Cyrus Ayieko
Philip Ayieko
Tutku Aykanat
Juan Ayllon
Frank Aylward
Maria Aymerich
Gizem Ayna
Godwin Ayoko
Guadalupe Ayora-Talavera
Pierre Ayotte
Nabieh Ayoub
Olalekan Ayo-Yusu
C. F. J. Ayres
Matthew Ayres
Ozer Ayse Belin
Scott Ayton
Lauren Ayton
Qasim Ayub
Muhammad Ayub
Angel Ayuso-Sacido
Napatkamon Ayutyanont
Srinivas Ayyadevara
Radha Ayyagari
Rajeev Ayyagari
Abdel Azab
Eric Azabou
Abul Azad
N. Azad
Abul Kalam Azad
Kayhan Azadmanesh
Muluken Azage
Dan Azagury
Hela Azaiez
Ameer Azam
Mohammad. Azam
Dany Azar
Hamed Azarbad
Taj Azarian
Rosario Azcón
Abdul Azeez
Samy Azer
Vasco Azevedo
Nuno Azevedo
Liane Azevedo
Ruben Azevedo
Luis Azevedo
Hatylas Azevedo
A. Azevedo
Vania Azevedo
Marcelo Azevedo Costa
Ali Azghani
Azheng Azhang
Nabil Azhar
Shafquat Azim
Syed Aziz
Moammir Aziz
Monowar Aziz
Yang Faridah Aziz

Mariam Aziz
Fereidoun Azizi
Bahareh Azizi
Reha Azizoglu
Azizullah Azizullah
Azrina Azlan
Andrew Azman
Syed Khurram Azmat
Jose Aznar-Moreno
Abderrahim Azouani
Élie Azoulay
Elie Azria
Jean-Philippe Azulay
Noriyuki Azuma
Toshifumi Azuma
Mizuki Azuma
Akifumi Azuma
Naoto Azuma
Kenichi Azuma
Chieko Azuma
Iskandar Azwa
Zaher Azzam
Arianna Azzellino
Ricardo Azziz
Eduardo Azziz-Baumgartner
Denis Azzopardi
George Azzopardi
Marc Baaden
Jan Baak
Laura Baams
Gudveig Baarli
Hoeke Baarsma
Esther Baart
Antonius Baartscheer
Peter Baas
Dolgor Baatar
Harald Baayen
Tadashi Baba
Hideo Baba
Yuki Baba
Sachiko Baba
Kazuyoshi Baba
Kenkichi Baba
Amir Babaeian
Lamine Baba-Moussa
Sankhiros Babapoor
Ali Babar
José Babarro
Opeyemi Babatunde
Akira Babazono
Shashi Babbar
Gregory Babbitt
Elizabeth Babcock
Molly Babel
Przemyslaw Babel
Andrea Babelova
Igor Babiak
Victor Babich
Harriet Babikako
Hamza Babiker
Doreen Babin
Patrick Babinger
Parker Babington
E. Babirekere-Iriso
Jodie Babitt
Igor Babkin
Jeff Babon
L. Charles Babout
Flurin Babst
Subash Babu
Giridhara R Babu
P. Babu
Petr Babula
Sergio Bacallado
Silviu-Alin Bacanu
Luiz Baccalá
Silvia Bacci
M. Baccini
Adriana Bacelo
Horacio Bach
Michael Bach
Leon Bach
Dominik Bach
Lars Bach
Lis Bach
Eviatar Bach
Lennart Bach
Veronika Bachanova
Robin Bachelder
Nathan Bacheler
Annabell Bachem
Jeff Bacher
Daniela Bacherini
Claus Bachert
Nandita Bachhawat
Maria Bächle-Haas

Andreas Bachmair
Michael Bachman
Daniel Bachman
Martin Bachman
Nathan Bachman
Sebastian Bachmann
Laura Bachmann
Till Bachmann
Asaf Bachrach
Markus Bachschmid
Adam Bachstetter
Emmanuel Bachy
Sung Hoon Back
Jantien Backer
Steffen Backert
Johannes Backs
Peter Backx
Guillaume Bacle
Michael Baclig
Albino Bacolla
Charles Bacon
Attila Bacsi
T. Baczek
Michael Bada
Hamid Badali
Asha Badaloo
Hussain Badani
Aldo Badano
Jose Badano
Jerome Badaut
Ahmed Badawy
Roland Baddeley
Natalie Baddour
Franz Badeck
Guney Bademci
Lindsey Baden
Joel Bader
Regine Bader
Brigitte Bader-Meunier
Arnaud Badets
Behnam Badie
Suguna Badiga
Ramesh Badisa
Judith Badner
Anna Badner
Andre Bado
Christal Badour
Vladimir Badovinac
Abdelfattah Badr
Stephen Badylak
Taeok Bae
Jin-Woo Bae
Hyunsu Bae
In Hwa Bae
Eun Ju Bae
Sun Hyun Bae
Sejong Bae
Kwang-Hak Bae
Wan Ki Bae
Christer Baeck
Wolfgang Baehr
Victoria Bae-Jump
Seung Baek
Kyungim Baek
Daehyun Baek
Seungik Baek
Veerle Baekelandt
Cristina Baena
José M. Baena-Cañada
Maria Baer
Matthew Baer
Felix Baerlocher
Scott Baerson
Erin Baerwald
Melissa Baese-Berk
Miriam Baeta
Jared Baeten
Kristin Baetz
Dieter Baeyens
Luc Baeyens
Ana Báez
Selene Báez
Andres Baeza
Mona Bafadhel
Georges Baffet
Sumit Bag
Sudeep Bag
Sukantadev Bag
Chiara Bagattini
Sarah Bagby
Robert Bagchi
Anindya Bagchi
Sabahat Cigdem Bagci
Ulas Bagci
Paramjeet Bagga
Herman Bagga

Jacopo A. Baggio
Hugo Baggio
Jacopo Baggio
Matthew Baggott
Ziad Baghdadi
Kamran Bagheri Lankarani
Emmanouil Bagkeris
Demetrius Bagley
Serena Bagnasco
Michel Bagnat
Emilia Bagnicka
Rosemary Bagot
James Bagrow
Andrew Bagshaw
Kenneth Bagstad
Thomas Baguley
Catherine Bagwell
Davis Bagyaraj
Csaba Bagyinka
Ayse Baha
Ranjit Bahadur
G. Bahadur
Krishna Bahadur
Ahmed Bahammam
Ofir Bahar
Mohd Yusof Baharuddin
Gulistan Bahat
Badrulhisham Bahazador
Maria Bahia
Mehdi Bahiraei
Ali Bahkali
Martin Bahl
Christie Bahlai
L. Bahler
Jürg Bähler
Ferdinad Bahlmann
Melanie Bahlo
Martin Bahls
Hisham Bahmad
Yong-Sun Bahn
Geonho Bahn
David Bahr
Soheyl Bahrami
Bahador Bahrami
Emad Bahrami-Samani
Lorraine Bahrick
Archana Bahuguna
Paluku Bahwere
Yawen Bai
Feng-Yan Bai
Guihua Bai
Ming Bai
Zhi Yi Bai
Fan Bai
Shu-Nong Bai
Xuefeng Bai
Hua Bai
Xiaoyan Bai
Gang Bai
Xiyuan Bai
Guangzhen Bai
Lu Bai
Wei Bai
Yuntao Bai
Zhen Bai
Haiqing Bai
Edith Bai
Bing Bai
Bin Bai
Haibo Bai
Yong-Rui Bai
Yingchen Bai
Xuemei Bai
Zhu Bai
Wenjia Bai
Songling Bai
Lijun Bai
Yonghong Bai
Peng Bai
I. Baiardini
Martina Baiardo Redaelli
Shakuntala Baichoo
Jennifer Baidal
Frank Baiden
Michael Baier
Gerold Baier
David Baier
Soon Koo Baik
Gwang Ho Baik
Kasia Bail
Julian Bailes
Vanessa Bailey
J. Mike Bailey
Charles Bailey
Shannon Bailey
Shara Bailey

Heather Bailey
Mark Bailey
Travis Bailey
Matthew Bailey
Daniel Bailey
L. Charles Bailey
Scott Bailey
Megan Bailey
Zinzi Bailey
James Bailey
Dylan Bailey
Sean Bailey
Kira Bailey
Michael Bailey
Chris Bailey-Kellogg
Joan Bailey-Wilson
Daniel Bailis
Howard Bailit
Renée Baillargeon
Frederic Bailleul
Benjamin Bailleul
Stéphanie Baillif
Sandrine Baillon
Xavier Bailly
Sebastien Bailly
Raquel Bailon
Barbara Bain
Paul Bain
Daniel Bain
Angus Bain
David Bainbridge
Richard Baines
Christopher Baines
Isabelle Bairati
Kevin Baird
Andrew Baird
J. Baird
C Noel Bairey Merz
Franz Bairlein
Harsh Bais
Niranjan Baisakh
Vineeta Bajaj
Abhay Bajaj
Renu Bajaj
Mona Bajaj-Elliott
Sandra Bajjalieh
Sofia Bajocco
Monika Bajorek
Peter Bajorski
Vivek Bajpai
Francis Bajunirwe
Søren Bak
Jin Seop Bak
Margit Bak Jensen
Zakaria Baka
Petros Bakakos
Muideen Bakare
Roger Bakeman
David Baker
Susan Baker
Michael Baker
Stuart Baker
William Baker
Brett Baker
Jennifer Baker
Darren Baker
Barry Baker
Jason Baker
Julien Baker
Mary Baker
Sarah Baker
Joel Baker
Joseph Baker
Brian Baker
Christopher Baker
Joanna Baker
Ld Baker
Olga Baker
Laurie Baker
Joshua Baker
Matthew Baker
Ann-Marie Baker
Cindy Baker
Rosalind Baker
Mark Baker
Mei Baker
Luisa Baker
Hooman Bakhshi
Zuzsa Bakk
Christopher Bakkenist
Erik Bakker
Martha Bakker
Jan Bakker
Guus Bakkeren
Jeroen Bakkers
Jamie Bakkum-Gamez

Péter Bakonyi
Giorgos Bakoyannis
Ioannis Bakoyiannis
Tatjana Bakran-Petricioli
Abhijeet Bakre
George L Bakris
Shairaz Baksh
Avijit Bakshi
Chandra Shekhar Bakshi
Richard Bakst
Murray Bakst
Wojciech Bal
Naresh Bal
Shashi Bala
Jay Bala
Evan Balaban
Carey Balaban
Yanina Balabanova
Siddharth Balachandran
S. Balachandran
Premalatha Balachandran
Kartik Balachandran
Ashwin Balagopal
Francesc Balaguer
Jacint Balaguer Coll
Sergio Balaguera-Reina
Yoga Balagurunathan
Paulchamy Balaiah
Kithiganahalli Balaji
Petety Balaji
Kethanapatti Balaji
Pachamuthu Balakrishnan
Sundar Balakrishnan
T. Balamugesh
Krishnaswamy Balamurugan
Murugabaskar Balan
Christiana Brigitte Balan
Venkatesh Balan
Bartosz Balana
Deepak Balani
Amy Balanoff
Hemalatha Balaram
Marly Balarin
Benjamin Balas
Valentina Balas
Nicole Balasco
Cheslav Balash
Natasha Balashova
Konstantinos Balaskas
Krishnan Balasubramaniam
Dorairajan Balasubramanian
Madhusudhanan Balasubramanian
Sowmya Balasubramanian
Priya Balasubramanian
Poonkuzhali Balasubramanian
Sureshkumar Balasubramanian
Shankar Balasubramanian
Vimalkumar Balasubramanian
Udeni Balasuriya
Anna Balato
Guillaume Balavoine
Stefan Balaz
Miroslav Balaz
Matthew Balazik
Andre Balbi
Graciela Balbin
Luciane Balbinot
Luciana Balboa
Susana Balcells
William Balch
Taye Balcha
Darius Balciunas
Kelvin Balcombe
Carsten Balczun
Enrico Baldan
Carlo Baldari
Chris Baldassano
Claire Baldeck
Kayla Balderson
Torsten Baldeweg
Alfonso Baldi
Elisabetta Baldi
Regina Baldini
Antonio Baldini
Chiara Baldini
Giulia Baldini
Matheus Baldissera
Guilherme Baldo
Laura Baldo
Juliana Baldo
Clair Baldock
Anne Baldock
Luciana Baldoni
Gerald Baldridge
Megan Baldridge
Luigi Balducci

Alessandra Balduini
Walter Balduini
Cynthia Baldwin
Scott Baldwin
Robert Baldwin
Austin Baldwin
John Baldwin
K. Baldwin
Darren Baldwin
David R Baldwin
Sherri Bale
Bachir Balech
William Balee
Biljana Balen
Sivacarendran Balendhran
Pablo Balenzuela
Graciela Balerio
Giorgio Balestra
Alma Balestrazzi
Raffaella Balestrini
Damien Balestrino
Tanjore Balganesh
Kiran Bali
Christos Baliatsas
Sebastian Balibar
Ravi Balijepalli
Eva Balint
Bela Balint
Zoltán Bálint
Peter Balint-Kurti
Dolly Baliunas
Wayne Balkan
Cecile Balkema
Zita Balklava
Cheryl Balkman
Suzanne Balko
Jonathan Ball
Gareth Ball
Felix Ball
David Ball
Keir Balla
Praveen Ballabh
Dov Ballak
Meltem Ballan
Pierre-Alexandre Balland
Benedicte Ballanger
Ford Ballantyne
Jimmy Ballard
Guy Ballard
Olivia Ballard
Sarah-Blythe Ballard
Natalia Ballardini
Paolo Ballarini
Samir Ballas
Esteban Ballestar
Coralio Ballester
A. Ballester
Soledad Ballesteros
Christoph Ballestrem
Stefano Ballestri
Daniel Ballhorn
D Balliet
Megan Ballinger
Karla Ballman
Manfred Ballmann
Piercarlo Ballo
Flavia Ballocca
Anne Balloch
Robert Ballotti
Jean-Marie Ballouard
Lora Ballweber
Jane Balme
John Balmes
Gabriel Balmus
Aamna Baloch
Adalbert Balog
Robert Balog
Zsolt Balogh
István Balogh
Mehmet Cengiz Baloglu
Mobolanle Balogun
Dimitrios Balomenos
Sahil Balotra
Vinohar Balraj
Eva Balsa-Canto
Maria Balsamo
Michele Balsamo
Sandor Balsamo
Andrea Balsari
Henrik Balslev
Sevket Balta
G. Baltaci
Fátima Baltazar
Sharon Balter
Kristen Baltgalvis
Cláudio Balthazar

Jacques Balthazart
Eric Balti
Svetlana Baltic
David Baltrus
Jay Baltz
Kimberly Baltzell
Pascal Baltzer
Dimitrios Baltzis
Niranjan Balu
Ramani Balu
Michiel Balvers
Irina Balyasnikova
Ryan Balzan
Kiran Bam
Makarem Bamatraf
Rameshwar Bamezai
Dennis Bamford
Christina Bamia
Giorgos Bamias
Shernaz Bamji
Zebalda Bamji
Karin Bammann
Bert Bammens
Fabrizia Bamonti
Yoshiyuki Ban
Natalie Ban
Jong-Wook Ban
Yusuke Ban
Hiroshi Ban
Maciej Banach
Niaz Banaei
Srinivas Banala
John Banas
Miroslaw Banasik
Michael Bancks
Germana Bancone
I. Bancos
Gregory Bancroft
Elizabeth Bancroft
Vimla Band
Leah Band
Daniel Bandarra
Abdul Rouf Banday
Antonio Bandeira
Christianne Bandeira De Melo
Klaus Bandel
Claudio Bandi
Stelvio Bandiera
Gretchen Bandoli
Rina Bandopadhyay
Hana Bandouchová
Oliver Bandschapp
Robert Bandsma
Gk Bandyopadhyay
Arun Bandyopadhyay
Debasish Bandyopadhyay
Amal Bandyopadhyay
Anita Bane
Kartik Bane
Sharmistha Banerjee
Rajat Banerjee
Sushanta Banerjee
Sanjeev Banerjee
Debabrata Banerjee
Debarshi Banerjee
Priyam Banerjee
Mayukh Banerjee
Jyotirmoy Banerjee
Sreeparna Banerjee
Anirban Banerjee
Arup Banerjee
Debasish Banerjee
Indroneal Banerjee
Dibyajyoti Banerjee
Rahul Banerjee
Rebecca Banerjee
Anita Banerjee
Santanu Banerjee
Arindam Banerjee
Versha Banerji
Christopher Banerji
Botond Bánfi
Marie-Louise Bang
Duhee Bang
Shashi Banga
S. Banga
Matthew Banghart
Max Bangs
Filipe Banha
Aria Baniahmad
Gouri Banik
Michael J. Banissy
Manoj Banjara
Roxanne Banker
Troy Bankhead
Stephanie Bankhead-Dronnet

William Banks
Peter Banks
Mattew Banks
Jonathan Banks
Wiliam Banks
Cristina Banks-Leite
James Bann
Darrin Bann
David Bann
John Bannantine
Danika Bannasch
Roger Bannister
Melanie Bannister-Tyrell
E. Banon-Maneus
Rosa María Baños
Manju Bansal
Anju Bansal
Dipika Bansal
Ruchi Bansal
Kushagra Bansal
Nisha Bansal
Vishal Bansal
Sheel Bansal
Nick Bansback
Scott Banta
Heike Bantel
Leonidas Bantis
Eleonor Bantry White
Brent Bany
Krisztian Banyai
Ashley Banyard
Vanessa M. Banz
Rita Banzi
Xiaoyong Bao
Shaowen Bao
Yongping Bao
Yan-Yuan Bao
Bo-Ying Bao
Zhenmin Bao
Changjun Bao
Bin Bao
Yongbo Bao
Yuping Bao
Jinsong Bao
Nirong Bao
Yan-Ping Bao
Xiaomin Bao
Jianqiang Bao
Yan Bao
Yongli Bao
Li Bao
Kunshan Bao
Shaowu Bao
Xiao-Yuan Bao
Rui Bao
Aihua Bao
António Baptista
Maurício Baptista
Maria Baptista
Pedro Baptista
Paula Baptista
Jayapalli Bapuraj
Shahida Baqar
Georges Baquet
Haim Bar
Katharine Bar
Himadrinandan Bar
Moshe Bar
Ido Bar
Gil Bar Sela
Jennifer Bara
Gyorgy Barabas
Frederic Barabe
David Baracchi
Vickie Baracos
Sarah Baracz
David Barad
Hamid Reza Baradaran
Pierre Baraduc
Ivano Baragetti
Andrea Baragetti
Pankaj Barah
Isabel Barahona
Paula Baraitser
Miguel Barajas
Hector Barajas-Martínez
Meir Barak
Khaled Barakat
Lamia Barakat
Pankaj Baral
Nabin Baral
Himlal Baral
Randolph Baral
Roberto Barale
Angela Baralla
Marco Baralle

Tallie Baram
Jane Baran
Ismet Baran
Yoav Bar-Anan
Gytis Baranauskas
Miroslav Barancik
Lisa Baranik
James Baraniuk
Ancha Baranova
Tatiana Baranovich
Tom Baranowski
Catherine Baranowski
Piotr Baranowski
Vikas Baranwal
Sergio Baranzini
Gian Marco Baranzoni
Va Barao
Edwine Barasa
Jonathan Barasch
Josep M. Barat
Mariella Baratti
Adam Barb
Ignasi Barba
Ap Barba De La Rosa
Ignazio Barbagallo
Mario Barbagallo
Nicola Barban
Veronique Barban
Facundo Barbar
Chirullo Barbara
William Barbaresi
Luc Barbaro
Nuria Barbarroja
Guy Barbato
Scott Barbay
Claudio Barbeito
Daniel L. Barber
Pa Barber
Thomas Barber
Bonnie Barber
Asa Barber
Tiziano Barberi
Luigi Barberini
Massimo Barberis
Shannon Barber-Meyer
Andrea Barbero
Jacques Barbet
Anthony Barbet
Fabrizio Barbetti
Elke Barbez
Michelle Barbi De Moura
Olivier Barbier
Pierre Barbier De Reuille
Joseph Barbieri
Chiara Barbieri
Judite Barbosa
Angela Barbosa
Andres Barbosa
A. Marcia Barbosa
Helene Barbosa
Marconi Barbosa
Leandro Barbosa
Fabiana Barbosa
Tiago Barbosa
Sandra Barbosa-Silva
Alan Barbour
Boris Barbour
Emily Barbour
Pascal Barbry
Adrian Barbu
Corrado Barbui
Adrian Barbul
José Alexandre Barbuto
Francesco Barca
Gianni Barcaccia
Leonardo Barcellos
Juan Barceló
Luciola Barcelos
Carlos Barcenas
Daniele Barchiesi
Guido Barchiesi
Julieta Barchiesi
Francesco Barchiesi
Aaron Barchowsky
Angel Barchuk
Marcello Barcinsky
Stephen Barclay
Roman Barczyk
Mira Barda-Saad
Pradip Barde
Janine Barden-O'Fallon
Maria Barderas
Sougata Bardhan
Benjamin Bardiaux
Amélie Bardil
Marc Bardin

Babara Maria Bardoni
Marco Bardoscia
Marc Bardou
Isabelle Bardou
Edouard Bardou-Jacquet
Sonia Bardy
Sonja Bareiss
Akshay Bareja
Monique Barel
Oshri Barel
Michael Bar-Eli
Luiz Barella
Elena Barengolts
Michael Barer
Luca Bargelloni
Steven Barger
John Bargh
Daniel Bargieri
Jill Bargonetti
Thereza Bargut
Ausaf Bari
Helene Baribault
Sailen Barik
Saroj Barik
Arnab Barik
Anandamay Barik
Patrick Baril
Lucio Barile
John Barile
Francis Barin
Michael Barish
Marin Barisic
Andrea Barison
Joseph Bariyanga
Isabel Barja Nunez
Christina Barja-Fidalgo
Charles Bark
Rosalind Bark
David Bark Jr.
Ran Barkai
Antonia Barke
Nigel Barker
Christopher Barker
Clive Barker
Diane Barker
Daniel Barker
Jason Barker
Bridget Barker
Alex Barker
Jocelyn Barker
John Barker
Amanda Barker
Laurie Barker
Basam Barkho
Shari Barkin
Andrew Barkley
Eric Barklis
Irit Bar-Kochva
Stephanie Barksdale
Jon Barlett
Raul Barletta
Linda Barlow
Peter Barlow
Jay Barlow
James Barlow
Fiona Kate Barlow
Miriam Barlow
John Barlow
J. Barlow
Judit Barna
Vincenzo Barnaba
Ruanne Barnabas
Jean-Yves Barnagaud
Donald Barnard
Gavin Barnard
Emma Barnard
Monia Barnat
Satish Barnawal
Luis Barneo
Anna Barnes
Tamsin Barnes
Andrew Barnes
Caroline Barnes
Gregory Barnes
David Barnes
Ralph Barnes
Deborah Barnes
Chris Barnes
Susan Barnett
Lionel Barnett
Whitney Barnett
Anna Barnett
Lisa Barnett
Adrian Barnett
Tracey Barnett
Carlton Barnett

Debora Barnett Foster
Jill Barnholtz-Sloan
D. Barnidge
Maxwell Barnish
Joaquín Barnoya
Francesc Baró
Victor Barocas
Elisabetta Barocelli
Jean-Claude Baron
Ellen Baron
Rebecca Baron
Roland Baron
Silvère Baron
Kelly Baron
Thierry Baron
Andrew Baron
Murray Baron
Yotam Bar-On
Lynn Bar-On
Marco Baron Toaldo
Andrea Baronchelli
Simona Baronchelli
Simon Baron-Cohen
David Barondeau
Maria Vittoria Barone
Francesca Barone
Mark Barone
Rosario Barone
Gianluca Baroni
Silvia Baronti
Giovanni Barosi
Robert Barouki
Guy Bar-Oz
Indrani Barpujari
Al Baha Barqawi
Elias Barquero-Calvo
Rachel Barr
Martin Barr
Richard Barr
Dale Barr
Gordon Barr
Jeremy Barr
Donald Barr
Frederic Barr
Adriano Barra
Laure Barrabé
Timothy Barraclough
Nick Barraclough
Aurélie Barrail-Tran
Jose Barral
Jérôme Barral
Manoel Barral-Netto
Yurena Barrameda-Medina
Victor Barranca
Rodolphe Barrangou
Neus Barrantes-Vidal
Frederic Barraquand
Alain Barrat
Joel Barratt
Jonathan Barratt
Simon Barratt-Boyes
Ivan Barreda
Tiago Barreira
Noelia Barreira
Rosa Barreira Da Silva
Andre Barreira Da Silva Rocha
Esther Barreiro
Anxo Barreiro
Antón Barreiro-Iglesias
Roberto Barrera
Lourdes Barrera
Valentina Barrera
Ana Barrera
Ernest Barreto
Cristine Barreto
Emiliano Barreto
Eliana Barreto-Bergter
Maria Luiza Barreto-Chaves
Jose Barreto-Filho
Andre Barreto-Vianna
Alan Barrett
Bruce Barrett
Timothy Barrett
Kim Barrett
John Barrett
Angela Barrett
Frederick Barrett
Tyler Barrett
Graham Barrett
Julie Barrette
Fernando R. Barri
Kelle Barrick
Nancy Barrickman
Antoni Barrientos
Yves Barrière
Guillermina Barril Cuadrado

Jessica Barrington-Trimis
Concepcion Barrio
Amy Barrios
Alfred Barritt
Andrew Barron
Francisco Barros
Aluisio Barros
Mario Barros
Mário Barros
Víviam Barros
Vinicius Barros
Alfonso Barrós-Loscertales
Eliane Barroso
Gilberto Barroso
Margarida Barroso
Paulo Barroso
Colin J. Barrow
Kevin Barrow
Alyssa Barry
Kathryn Barry
Peter Barry
Pennan Barry
Joseph Barry
Sarah Barry
Cornelius Barry
Jeremy Barry
Amanda Barry
Sheila Barry
Lisa Barry
Janina Barsiene
Henry Bart
David Bart
Jiri Barta
Bonnie Bartel
Sabine Bartel
Peter H. Bartels
Randy Bartels
Meike Bartels
Ronald Bartels
Tim Bartels
Mette Bartels
Devra Barter
Renata Bartesaghi
Giulio Barteselli
Susanne Barth
Holger Barth
Brian Barth
Kerstin Barth
Andreas Barth
Matthew Barth
Roos Barth
Benjamin Barthel
Brandon Barthel
Marc Barthelemy
Philippe Barthélémy
Ronaldo Barthem
Daniella Bartholomeu
Geoffrey Bartholomeusz
Jerri Bartholomew
Bruce Bartholow
James Bartles
Stephen T. Bartlett
Laura Bartley
Emily Bartley
Timothy Bartley
Salomon Bartnicki
Ewa Bartnik
Thomas Bartnikas
Joshua Bartoe
Soraya Bartol
Erzsebet Bartolak-Suki
Mark Bartold
Michele Bartoletti
Marc Bartoli
Manuela Bartoli
Claudia Bartoli
Francesco Bartoli
Paolo Bartolomeo
Alessandro Bartolomucci
Elena Bartoloni
Al Bartolucci
Ignasi Bartomeus
Anne Barton
Jennifer Barton
Huw Barton
Hazel Barton
John Barton
Ludek Bartos
Michael Bartos
Laszlo Bartosiewicz
Rafal Bartoszewski
Eva Bartova
Eveline Bartowsky
Sarah Bartsch
Guillermo Barturen
Jason Bartz

Jerry Bartz
Subit Barua
Rajat Barua
Dominique Baruch
André Baruchel
Enrico Baruffini
Shirish Barve
Narayani Barve
Vijay Barve
Sonali Barwe
Yaneer Bar-Yam
Joshua Barzilay
Dudy Bar-Zvi
Ehsan Basafa
Jolly Basak
Trayambak Basak
David Basanta
M. R. Basar
Randall Basaraba
Allan Basbaum
Ulrike Baschant
Christiane Baschien
Jorg Basecke
Kamran Ul Baset
Margaret Bash
Yoav Bashan
Meredith Bashaw
Aneesh Basheer
Khurram Bashir
Ilham Bashir
Syed Muhammad Arsalan Bashir
Ishtiaq Bashir
Mikhail Bashkurov
Murali Bashyam
Marijana Basic
Michael Basil
Juan Basile
Franco Basile
Stefania Basili
James Basilion
Abdul Basit
Nagarajan Baskaran
David Basketter
Tobias Baskin
Darrell Baskin
Arnaud Basle
Ram Basnet
Purusotam Basnet
Venkatesha Basrur
Joseph Bass
James Bass
Pier Paolo Bassareo
Per Basse
Patricia Bassereau
Patrick Basset
Kenneth Bassett
Julie Bassett
Emma Bassett
Scott Bassett
Christopher Bassey
Roberto Bassi
E. Bassil
Craig Bassing
James Bassingthwaighte
Alicia Basso
Stefano Basso
Bruno Basso
Michela Bassolino
Abigail Basson
Alexander Bassuk
Felix Bast
Nicole Basta
Dietmar Basta
Salim Bastaki
Francois Bastardie
Ulrike Basten
Stuart Basten
Javier Basterra
Björn Bastian
Kerry Bastian
Fernando Bastida
Guillaume Bastille-Rousseau
Philippe Bastin
Mark Bastin
Jean-François Bastin
Emanuela Bastonini
Francisco Bastos
Armanda Bastos
João Bastos
Marco Bastos
Rogerio Bastos Craveiro
Ashis Basu
Utpal Basu
Devraj Basu
Debashree Basu
Sanjib Basu

Arpita Basu
Aparna Basu
Surupa Basu
Analabha Basu
Anirban Basu
Chandana Basu Mallick
Robindra Basu Roy
Soumik Basuray
Upal Basuroy
Euguenia Basyuc
Nizar Batada
Ken Batai
Ibrahim Batal
Dafnis Batalle
Ramon Bataller
Peter Batcheller
Matthew Batchelor
Frances Batchelor
Moses Bateganya
Joseph Bateman
Heather Bateman
Bill Bateman
Amin Bateni
John Bates
Paul Bates
Mark Bates
Matthew Bates
Jon Bates
Amanda Bates
Nathaniel Bates
Kieran Bates
Kevin Bath
Erik Bathoorn
Mona Batish
Wagner Batista
Rui Batista
Dora Batista
Hunt Batjer
Jacqueline Batley
Diego Batlla
Henri Batoko
Arvind Batra
Janendra Batra
Ranjan Batra
Nidhi Batra
Sanjay Batra
Cécile Bats
Eric Batsche
Glaivy Batsuli
Carl Batt
Ryan Batt
Manuela Battaglia
Salvatore Battaglia
Sebastiano Battaglia
Ricardo Battaglino
Dilek Battal
Jonathan Batten
Alan Batterham
Laura Batterink
Anna Battersby
Frederic Batteux
Elisabeth Battinelli
Giovanna Battipaglia
Andrea Battisiti
Anna Battista
Antonio Battisti
Stefano Battiston
Fabia Ursula Battistuzzi
Michele Battle
Cynthia Battle
Venkata Battula
Kevin Batty
Neha Batura
Michael B. Batz
Haim Bau
Helene Baubichon-Cortay
Chris Bauch
Laurent Baud
Peter Baudains
Gabriel Baud-Bovy
Mara Baudena
Linnea Baudhuin
Marc Baud'Huin
Sylvie Baudino
Luc Baudouin
Jean-Yves Baudouin
Stéphane Baudry
Jerome Baudry
Kenneth Bauer
Brent Bauer
Jürgen Bauer
Alison Bauer
Elizabeth Bauer
Greta Bauer
Stephanie Bauer
Patricia Bauer

Daniel Bauer
Martin Bauer
Reinhard Bauer
Johann Bauer
Axel Bauer
Paul Bauer
Isabelle Bauer
Eva Bauer
Petra Bauer-Kreisel
Tobias Bäuerle
Fernando Bauermann
Jose Bauermeister
Günther Bauernfeind
Stefan Bauersachs
Jerome Baufreton
Lee Baugh
Alexander Baugh
Anthony Baughn
Jürgen Bauhus
Alain Baulard
Johannes Baulmann
Buzz Baum
Linda Baum
Erika Baum
Michael Baum
Michel Baum
Kristen Baum
Dana Baum
Sarah Baum
Christina Baum
Melissa Bauman
Andrew Bauman
Whitney Bauman
Michael Baumann
Oliver Baumann
Arnd Baumann
Francine Baumann
Nicolas Baumard
Kyle Baumbauer
Ben Baumberg
Philipp Baumeister
Phillipp Bäumer
Thomas Baumert
Mathias Baumert
Johannes Baumgart
Bernhard Baumgartner
Rupert Baumgartner
Joy Noel Baumgartner
Christina Baumgartner
Karine Baumstarck
Daniel Baumunk
Hansjörg Baurecht
Andreas Bausch
Dirk Bausch
Karin Bausenhart
Denise Bauser
Christophe Bauters
Wendy Bautista
Tara Bautista
Ivan Bautmans
Hermann Bauwe
Frederick Bauzon
Keith Baverstock
Giorgio Bavestrello
Patrik Bavoil
Vassiliy Bavro
Anthony Bavry
Ad Bax
Heather Bax
Bonnie Baxter
Mark Baxter
Leslie Baxter
Greg Baxter
David Baxter
Bagora Bayala
Elsa Bayart
Sam Bayat
Ahmet Baydin
Joe Bayer
K. Bayer
Martin Bayer
Marina Bayeva
Jude Bayham
Betul Baykan-Baykal
Priscilla Bayle
Sandrine Bayle
Kenneth Bayles
Sarah Bayless
Howard Baylis
Christine Baylis
Oliver Baylis
Christopher Bayliss
Philip Bayly
Emine Bayman
Stephen Bayne
Alice Baynes

Javier Bayod
Hasan Bayram
Jagadeesh Bayry
Melissa Baysari
Rachid Baz
Maher Baz
Catalina Bazacliu
J. Fernando Bazan
Haydee Bazan
Jose Bazan
Yaser Bazargani
Mahdi Bazarghan
Corinna Bazelet
Ra Bazemore
Dmitry Bazhanov
Adam Bazinet
Ivonne Bazwinsky-Wutschke
Dmitry Bazyka
Marya Bazzi
Eda Bcer
Nicholas Be
Sílvia Beà
James Beach
Thomas Beach
Michael Beach
Kirk Beach
Terry Beacham
Joanna Beachy
Robert Beaglehole
Jessica Beagley
Mathew Beale
Ian Beales
Clifford Beall
Stower Beals
Jake Beam
Lesa Beamer
Gillian Beamer
Bruce Bean
Tim Bean
Travis Bean
Wendy Beane
Elena Beani
Philippa Beard
John Beardall
Carrie Bearden
Alessandra Bearz
David Beasley
James Beasley
Rodrigo Beas-Luna
Claude Beata
Margaret Beaton
Scott Beatson
Lynette Beattie
Geoff Beattie
Christopher Beatty
William Beatty
Brian Beatty
Terri Beaty
Lynne Beaty
Michael Beauchamp
Kathy Beaudette
Hughes Beaufrere
Michael Beaulieu
Carole Beaulieu
Melanie Beaulieu
Christophe Beauloye
Bruno Beaumelle
Martin Beaumont
Steven Beaupre
Christian Beausejour
Ngaio Beausoleil
Thomas Beaver
William Beavers
Daniel Beavers
Melanie Beazley
Marketa Bebarova
Piotr Bebas
Gwyn Bebb
Penelope Bebeli
Marek Bebenek
Thomas Beblo
Francisco Beca
Laia Bécares
Cecilia Becattini
Alejandro Beceiro
Jose Becerra
Per Bech
Meagan Bechel
Ronan Becheler
Heiko Becher
Francesca Becherucci
Alessio Bechini
Lars Bechmann
Thea Bechshoft
Christopher Beck
Laurent Beck

Harald Beck
Evelin Beck
Felix Beck
Jürgen Beck
Andreas Beck
Ilse Beck
Daniel Beck
Laurence Beck
Maurie Beck
Thilo Beck
R.W. Beck
Belinda Beck
Peter Becker
Stan Becker
Catherina Becker
Christian Becker
Marissa Becker
Sören Becker
Claude Becker
Pamela Becker
Wolfgang Becker
Richard Becker
Nina Becker
Karsten Becker
Stephen Becker
Jesse Becker
Ingebor Becker
Daniel Becker
Lewis Becker
Sara Becker
Heather Becker
Nicolas Becker
Jerome Becker
Donna Becker
D. Vaughn Becker
María Becker
Thomas Becker
Therese Becker
Stephen Beckerman
Christoph Becker-Pauly
Gabriel Beckers
Erik Beckers
Stephen Beckett
Yvonne Beckham
Leontine Becking
Alfred Becking
Diane Beckles
Nick Beckloff
Paolo Beck-Peccoz
Robert Beckstead
Michael Beckstead
Kyle Beckwith
Colin Beckworth
Frederic Becq
Aditi Bector
Nicolas Becu
Antonio Bedalov
Donna Bedard
Patrick Bédard
Aurélie Bedel
Harold Bedell
Branka Bedenic
Michael Bedford
Gillinder Bedi
Updesh Bedi
Jeffery Bednark
Nina Bednarsek
Nicolas Bedo
Barbara Bedogni
Christine Bedore
Renesh Bedre
Isabelle Bedrosian
Sharifah Bee
David Beebe
Dean Beebe
Nigel Beebe
Stephen Beebe
Aaron Beedle
Sashidhar Rao Beedu
Marian Beekman
Patrick Beeler
Karen Beemon
L. Beenen
Ronny Beer
Hans-Dietmar Beer
Philip Beer
Avraham Be'Er
James Beerens
Kim Beernaert
David Beers
Jody Beers
Aidan Beers
Gilad Be'Ery
Katja Beesdo-Baum
Amer Beg
Daniela Begandt

Gerrit Begemann
Friedrich Begemann
Maria Beger
Dominik Begerow
John Beggs
Clive Beggs
Ettore Beghi
Cesare Beghi
John Beghin
Philippe Begin
David Begley
Christoph Beglinger
Maria Begnami
Josip Begovac
Julien Beguin
Rasheedunnisa Begum
Ferdouse Begum
Khadija Begum
Alexander Begun
Katarina Begus
Samuel Behar
Jaideep Behari
Erica Behling-Kelly
David Behm
Spencer Behmer
Ehsan Behnam
Martin Behnisch
Sascha Behnk
Spencer Behr
Björn Behr
Jurgen Behrens
Sven-Erik Behrens
Maik Behrens
Jane Behrens
Martin Behrens
Johanna Behringe
David Behringer
Julia Behrman
Kevin Behrns
Weicheng Bei
Jin-Xin Bei
Karsten Beiderwellen
Paul Beier
Frank Beier
Margaret Beier
Ulrik Beierholm
Elizabeth Beierle
Claudia Beiersmann
John Beigel
Anne-Sophie Beignon
Oscar Beijbom
Ulla Beijer
Omid Beiki
Robert Beiko
Tatsiana Beiko
Michael Beil
Kurt Beil
Andreas Beilhack
Traude Beilharz
Euro Beinat
Jean-Claude Béïque
Kostantinos Beis
Kirk Beisel
Auke Beishuizen
Tim Beißbarth
Christoph Beisswenger
Daniel Beiting
Alvin Beitz
Dietrich Beitzke
Albano Beja Pereira
Ismail Bejia
T. Bek
Laurent Bekale
Wubishet Bekele
Kimon Bekelis
Yehonadav Bekenstein
Isabelle Bekeredjian-Ding
Stefan Bekiranov
Arzu Beklen
Gabriel Bekö
Robin Bekrater-Bodmann
Lynn Bekris
Yolanda Bel
Nathalie Belanger
Anna Belardinelli
Juan Belardinelli
Robert Belas
Daniel Belavý
Alexandra Belayew
Olivia Belbin
John Belcher
William Belcher
Britany Belcher
Ricardo Beldade
Lisa Belden
Guido Beldi

Pablo Beldomenico
Laurent Belec
Ashok Belegundu
Cristiano Belem Da Silva
Enrique Belenguer
Enrique Belenguer-Balaguer
Peter Belenky
Sandra Beleza
Anna Belfer-Cohen
Antonino Belfiore
Marlene Belfort
Tildon Belgard
Tom Belhorn
Eleni Beli
Alexander Beliaev
Georgios Belibasakis
Srinivas Belide
Pascal Belin
Dominique Belin
Eric Belin De Chantemèle
Boris Belitsky
Darrell Belke
Charles Bell
Derek Bell
Howard Bell
Kayce Bell
Jason Bell
Susan Bell
Christopher Bell
Joshua Bell
Andrew Bell
F. Wayne Bell
David Bell
Natasha Bell
Rebecca Bell
Phil Bell
Adrian Bell
Richard Bell
Maria Belladonna
Felipe Belladonna
Bryan Bellaire
Steve Bellan
Leon Bellan
Paul Bellan
Francesco Bellanti
Anthony Bellantuono
C. Bellard
Srikanth Bellary
Evangelia Bellas
Adriana Bellati
Andrea Bellavia
Sybelle Bellay
Janeil Belle
Mino Belle
Ashwin Belle
Clémence Belleannée
Laure Bellec
Barbara Bellei
Giuseppe Bellelli
Simon Bellemare
Carsten Beller
Marina Bellet
Robyn Bellet
Alessandro Belletti
John Bellettiere Bellettiere
Claudio Bellevicine
Silvia Bellezza
Alfonso Bellia
Teresita Bellido
Jérémy Bellien
Bertrand Bellier
Marco Bellinzoni
Susan Bellis
Veronica Bellisario
Gonzalo Bello
Silvia Bello
Salvador Bello
Felio Bello
Murilo Bello
Martiniano Bello Ramirez
Luis E. Bello-Espinosa
Vivian Bellofatto
Elisa Bellomo
Juan Bellón
Rebecca Bellone
Stefano Bellosta
Vittorio Bellotti
Tony Bellotti
Arianna Bellucci
Elisa Bellucci
Ilaria Belluomo
Soumaya Belmecheri
John Belmont
Matthew Belmonte
Vittorio Belmonti
Vinícius Belo

H. Beloeil
Artem Belopolsky
Apostolos Beloukas
Assila Belounis
Katherine Belov
George Belov
Tony Belpaeme
Aniruddha Belsare
Jessica Belser
Denise Belsham
Graham Belsham
Daniel Belsky
Jay Belsky
Antonio Paolo Beltrami
Carlo Alberto Beltrami
Cristina Beltrami
Marie-Claude Bel-Venner
Aditya Belwadi
Peter Belwood
Alexa Bely
Olga Belyaeva
Igor Belykh
Clara Belzer
Luc Belzunces
Moufida Ben Nasr
Nouri Ben Zakour
Karim Benabdellah
Alim Louis Benabid
Jorge Benach
Mohamed Benahmed
Manjunatha Benakanakere
Shani Ben-Amitay
Alexandre Benani
Christian Benar
Charaf Benarafa
Michael Benard
Philippe Benaroch
Mariagrazia Benassi
Barbara Benassi
Solomon Benatar
Ricardo Benavente
Alexandra Benavente-Perez
Mar Benavides
Sandra Benavides
Andres Benavides-Serralde
Yehuda Benayahu
Stefano Benazzi
Massil Benbouriche
Doris Benbrook
Charles Benbrook
James Bence
Sompop Bencharit
Mojca Bencina
Péter Bencsik
Norbert Benda
Andrew Bendall
Linda Bendall
Merav Ben-David
Reina Bendayan
William Bendena
Andreas Bender
Dorothea Bender
Michael Bender
Kevin Bender
Jackie Bender
David Benditt
Ivo Bendix
Alexandra Bendixen
Casper Bendixsen
Michele Bendoni
Irene Benech
György Benedek
Andrea Benedetti
Sara Benedetti
Celso Benedetti
Lisandro Benedetti-Cecchi
Simone Benedetto
Lauryn Benedict
Agnes Benedict
Kaitlin Benedict
Stefano Benedini
Luciano Beneduce
Brenda Benefit
Giovanni Benelli
Itzhak Benenson
Joyce Benenson
Abdulbari Bener
Fabrício Benevenuto
Miriam Benezra
Mara Benfato
Philip Benfey
Laurentiu Benga
Susanne Bengesser
Mohammed Benghezal
Enrique García Bengoechea
Andrew Bengsen

Sarah Bengston
Stefan Bengtson
David Bengtson
Ewert Bengtsson
Boel Bengtsson
Helen Benham
Dan Benhamou
Mohamed Benharouga
Jean Benhattar
Tal Ben-Horin
Guy Benian
Ariela Benigni
Romualdo Benigni
Elisa Beninca
Joscha Beninde
Karen Beningo
Vicente Benites
Bruno Benitez
Guillermo Benitez
Maria Soledad Benitez
Alfonso Benitez-Paez
Manuel Benito
Antonio Benito
Jon Benito
Rosa María Benito
Marta Benito Garzon
Sara Benjamin Neelon
Jade Benjamin-Chung
Yuval Benjamini
S. Janine Benjamins
Soumaya Benjelloun
Kálmán Benke
Dietmar Benke
N. Benkhedah
Craig Benkman
Kamel Benlagha
Jose Maria Benlloch
Peter Benn
Christine Benn Christiansen
Idriss Bennani-Baiti
Antonio Bennasar
Mar Bennasar
Miquel Bennasar-Veny
Lori Bennear
Steven Benner
Ina Benner
Seico Benner
S. Christopher Bennett
Anton Bennett
Carol Bennett
Matthew Bennett
Alison Bennett
William Bennett
Brian Bennett
David Bennett
Ian Bennett
Gordon Bennett
Nathan Bennett
Eric Paul Bennett
Scott Bennett
Vann Bennett
James Bennett Jr.
Rachel Bennetts
Kevin Bennewith
Richard Benninger
Abby Benninghoff
Douglas Bennion
Emily Bennitt
Gerard Benoit
Joshua Benoit
Roland Benoit
Joseph Benoit
Kelly Benoit-Bird
Hilla Ben-Pazi
Olivier Bensaude
Bernadette Bensaude-Vincent
Kimberley Benschop
Mohammed Bensellam
Dorit Ben-Shachar
Yoram Ben-Shaul
Gil Ben-Shlomo
Andrew Benson
Constance Benson
Valerie Benson
Jill Benson
Ashleen Benson
Stacey Benson
John Benson
Philip Benson
Elizabeth Bent
Smadar Ben-Tabou De-Leon
Bastian Bentlage
Stephen Bentley
Peter Bentley
Margaret Bentley
Paul Bentley

Katie Bentley
Amy Bentley
John Kelley Bentley
Isabel Bento
Michael Benton
Paul Benton
Gabriel Benton
Matthew Benton
Håvard Bentsen
Christian Bentz
Ronald Benveniste
Brian Benway
Sigal Ben-Yehuda
Roland Benz
Adele Benzaken
Sigal Benzaken
Dror Ben-Zeev
Karen Benzies
Athanasios Beopoulos
Subir Bera
Carolina Beraldo Meloto
Michael Beran
Roy Beran
Simone Berardi
Aileen Berasategui
Guillaume Béraud
Nicolas Berbari
Tanya Berbasova
Mary Berbee
Friederike Berberich-Siebelt
Guy Am Berbers
Marika Berchicci
Cristina Berchio
Nadège Bercovici
H. Bercovier
Fred Bercovitch
Aikaterini Berdiaki
Georg Berding
Luca Berdondini
Miguel Berdugo
Evgeny Berdyshev
Alfred Bere
Elling Bere
Yoella Bereby-Meyer
Vesna Berec
Michael Berenbrink
Evelien Berends
Bettina Berendt
Juan Berenguer
Jose Berenguer
Christian Berens
Ali Berens
Nina Berentzen
Stephen Beres
Viktoria Beres
Alexander Berestetski
Giovanni Beretta
Sabina Beretta
Giangiacomo Beretta
Alexey Berezhnoy
Maxim Berezovski
Daniela Berg
Stefan Berg
Carla Berg
Ole Berg
Gabriela Berg
Christine Berg
Einar Berg
Björn Berg
Karl Berg
Alvaro Berg Soto
Matteo Bergami
Carlo Bergamini
Elena Bergamini
Gabriele Berg-Beckhoff
Basti Bergdahl
Lillian Berge
Eivind Berge
Jeffrey Bergelson
Elika Bergelson
Arthur Bergen
Dessa Bergen-Cico
Johannes Berger
Klaus Berger
William Berger
Philipp Berger
Joachim Berger
Howard Berger
Swetlana Berger
Zoltan Berger
Sarah Berger
Frank Berger
Madeleine Berger
Roberta Bergero
Patrick Bergeron
Pierre-Jérôme Bergeron

Benjamin Bergerot
Cristiane Bergerot
Ron Bergevoet
Elizabeth Bergey
Wendy Berg-Foels
Vincenzo Berghella
Herman Berghuijs
Philip Bergin
Steven Bergink
Jutta Bergler-Klein
Eva Berglund
David Berglund
Peter Bergman
Patrick Bergman
Eric Bergman
Andreas Bergmann
Lothar Bergmann
Carsten Bergmann
Christina Bergmann
Gaddy Bergmann
Till Bergmann
Julie Bergmann
Philip Bergmann
Uri Bergmann
Audrey Bergouignan
Niels Bergsland
Clare Bergson
Ted Bergstrom
Anna Bergström
Lena Bergström
Jason Bergtold
Clemens Bergwitz
Alamin Berhanu
Ioana Berindan-Neagoe
Stine Bering
Tiziana Beringhelli
Alexander Beristain
Johannes Berkhof
Ben Berkhout
James Berkley
Elliot Berkman
Nadia Berkova
Bruce Berkowitz
Natacha Berkowitz
David Berkowitz
Richard Berl
Joel Berletch
Gloria Berlier
Ivan Berlin
Jacob Berlin
Maud Berlincourt
Léo Berline
Sebastian Berlit
Stewart Berlocher
Eric Berlow
Giovanni Berlucchi
Hal Berman
Marc Berman
Nancy Berman
Yonatan Berman
Jesse Berman
J Michael Berman
Marvin Berman
Javier Bermejo
Azucena Bermejo
Pablo Bermejo-Alvarez
Mairead Bermingham
Luiz Bermudez
Kalina Bermudez Torres
Federico Bermúdez-Rattoni
Fabrice Berna
Daniel Bernabe
Eduardo Bernabé
Abel Bernadou
Kristel Bernaerts
Blanca Bernal
Moises Bernal
Fa Bernal
Diego Bernal
David Bernal-Estévez
Ernesto Bernal-Mizrachi
Bruno Bernard
Hans-Ulrich Bernard
Ernest Bernard
Karen Bernard
Cora Bernard
Kristin Bernard
Amelie Bernard
Laurence Bernard
Kathryn Bernard
Kristen Bernard
Christina Bernardes
Juliana Bernardes
Gonçalo Bernardes
Jean Francois Bernardet
Francesco Bernardi

Luciano Bernardi
Stella Bernardi
Sara Bernardi
Juliana Bernardi
Danilo Bernardo
Mark Bernards
Sandra Bernards
A. Bernasconi
Sebastian Bernasek
Pascal Bernatchez
Nikolaus Berndt
Alexander Berndt
Daniel Berner
Michael Berney
Jürgen Bernhagen
Frank Bernhard
Wolfgang Bernhard
Scott Bernhardt
Anne Bernhardt
Paul Bernhardt
Boris Bernhardt
Roberto Berni Canani
Max Berniker
Franco Bernini
Stine Bernitz
Daniel Bernoulli
Gustav Bernroider
Harold Bernstein
Helene Bernstein
Joseph Bernstein
Sanford Bernstein
Kyle Bernstein
Daniel Bernstein
Galina Bernstein
Jonathan Bernstein
Matthias Bernt
Erik Berntsen
Adam Bero
Rachel Berquist
Bastien Berret
Marcelo Berretta
Wade Berrettini
Felix Berrigan
Sonia Berrih-Aknin
Alain Berro
Veronica Berrocal
Arturo Berrones
Giovanna Berruti
Fred Berry
Gerard Berry
Jarett Berry
Colin Berry
Elaine Berry
Kristin Berry
William Berry
Peter Berry
Michael Berry
Matthew Berryman
Donald Bers
Ferdinando Bersani
Ines Bersch
Andrew Bersten
Fabrizio Bert
Annalisa Berta
Temugin Berta
Assunta Bertaccini
Stéphane Bertani
L. E. Bertassoni
Sergio Bertazzo
Olivier Berteau
Jean-Philippe Berteau
Dominique Berteaux
Mario Bertella
Claire Bertelli
Thomas Bertelmann
Mette Bertelsen
Michael Berthaume
Karine Berthelot
Laureline Berthelot
Pierre Berthet
Francois Berthiaume
Jessica Berthiaume
Marcelo Berthier
E. Berthier
Franz Berthiller
Hans-Rudolf Berthoud
Francois Berthoux
Sylvie Berthoz
Matteo Berti
Fabrice Bertile
Stefan Bertilsson
Francesca Bertinetto
Caterina Bertini
Juan Berto
Leandro Bertoglio
Monica Bertoia

Laura Bertola
Andrea Bertoldi
A. Bertoletti
Laurent Bertoletti
Simona Bertoli
Giovanni Bertolini
Robert Bertolo
Elizabeth Bertone-Johnson
Giovanni Bertoni
Giorgio Bertorelle
Tulio Bertorini
B. Bertosa
Mirjam Bertoune
John Bertram
Ralph Bertram
Kateri Bertran
Pascale Bertrand
Stéphanie Bertrand
Daniel Bertrand
Luc Bertrand
William Bertrand
Arnaud Bertrand
Julien Bertrand
Sophie Bertrand
Alexandra Bertron
Katja Bertsch
Alison Bertuch
Rômulo Bertuzzi
Jaime Berumen
Daniel Berwick
Alfredo Berzal-Herranz
Chrystel Besche-Richard
Katharina Besemer
Urban Besenfelder
Kerim Beseoglu
Thor Besier
Julien Besle
Stephane Besnard
Francois Besnier
Marie Besnier
Daniela Besozzi
Filipa Bessa
Lucinda Bessa
Alysson Bessani
Alban Bessede
George Besseris
Barbara Bessette
Tadayoshi Bessho
Kazuhisa Bessho
Mireille Besson
Galina Besstremyannaya
Lyle Best
John Best
Jan Best
Oriol Bestard
Dany Beste
Christian Beste
Sébastien Besteiro
Dirk Bester
Aletta Bester-van der Merwe
Timothy Bestor
Guru Betageri
Tatiana Betakova
Monica Betancor
Alexander Betekhtin
Swati Betharia
Christina Bethell
Kathleen Bethin
Richard Bethlehem
Jeffrey Bethony
Eriola Betiku
Michiel Betjes
Fay Betsou
Glenna Bett
Josette Bettany-Saltikov
Raul Bettencourt
Viviana Betti
Pete Bettinger
Maria Bettini
Matthew Bettini
Victoria Betton
Michael Betts
Jonathon Betts
Alison Betts
Saverio Bettuzzi
Oliver Betz
Richard Betzel
Alan Beu
Jan Beucke
Olivier Beuf
Wouter Beukema
Anita Beukes
Joline Beulens
Thijs Beuming
Eleonore Beurel
Rainer Beurskens

Gisela Beutner
Annie Beuve
Romina Bevacqua
David Bevan
James Bever
Levi Beverly
David Beversdorf
Catherine Bevier
Antonio Bevilacqua
Alan Bevington
Annamaria Bevivino
Joerg Bewersdorf
Carole Bewley
Jeffrey Bewley
Peter Bex
Blake Bextine
Erik Bey
Jan Beyea
Haluk Beyenal
Martin Beyer
Wolfgang Beyer
Tobias Beyer
Frederike Beyer
Eric Beyer
Katrin Beyer
Niklas Beyersdorf
Jolly Beyeza-Kashesya
Natalie Beylis
Nicolas Bez
Daniela Bezemer
Jorge Bezerra
Andréia Bezerra
Vivien Beziat
Connie Bezzina
Viaji Bhadauria
Subramanyam Bhadriraju
Sanjay Bhagani
Ashok Bhagwat
Jong Bhak
Mehul Bhakta
Utpal Bhalala
Gopalkrishna Bhalerao
Needhi Bhalla
Devender Bhalla
Savita Bhalla
Ishir Bhan
Urvashi Bhan
Gp Bhandari
Ruchi Bhandari
Sunil Bhandari
Seema Bhangar
Venkanna Bhanothu
V Bhanuprakash
Amarjit Bhanwer
Mausumi Bharadwaj
Swati Bharadwaj
Shrikant Bharadwaj
Uddalak Bharadwaj
C. Bharadwaj
Anil Bharath
Arun Bhardwaj
Vikas Bhardwaj
Anshu Bhardwaj
Neeru Bhardwaj
Aditi Bhargava
Kapil Bharti
Santosh Bharti
Arora Bharti
Omesh Bharti
Deeksha Bhartiya
Sangeeta Bhaskar
Arun Bhaskar
Radika Bhaskar
Ramachandra Bhaskara
Santosh Bhaskaran
Subha Bhassu
Hari Bhat
Harish Bhat
Prashant Bhat
Ramesh Bhat
Vijayalakshmi Bhatia
Anuj Bhatia
Dhiraj Bhatia
Swapnil Bhatia
Aruni Bhatnagar
Raj Bhatnagar
Sushant Bhatnagar
Sandhya Bhatnagar
Rakesh Bhatnagar
Deepak Bhatnagar
Pooja Bhatnagar-Mathur
Apoorva Bhatt
Vijaya Bhatt
Jay Bhatt
M. Ramachandra Bhatt
Abinash Bhattachan

Surajit Bhattacharjee
Atanu Bhattacharjee
Subhadeep Bhattacharjee
Ashish Bhattacharjee
Niloy Bhattacharjee
Sudha Bhattacharya
Joydeep Bhattacharya
Sanjoy Bhattacharya
Shibani Bhattacharya
Resham Bhattacharya
Sohinee Bhattacharya
Bhaskar Bhattacharya
Shelley Bhattacharya
Arup Bhattacharya
Jaydeep Bhattacharya
Sanmitra Bhattacharya
Jayanta Bhattacharya
Sourav Bhattacharya
Madan Bhattacharyya
Swati Bhattacharyya
Nitai Bhattacharyya
Suvendra Bhattacharyya
Krishnendu Bhattacharyya
Moitrayee Bhattacharyya
Samit Bhattacharyya
Sudeepa Bhattacharyya
Somanth Bhattacharyya
Manpreet Bhatti
Attya Bhatti
Ashay Bhatwadekar
Siddhartha Bhaumik
Mrinal Bhave
Ujjal Bhawal
Louis Bherer
B Bhhatarai
Jabeene Bhimji
Neha Bhise
Rahul Bhola
Pranav Bhounsule
Deb Bhowmick
Rudra Bhowmick
Bishwajit Bhowmik
Jahar Bhowmik
Manoshi Bhowmik-Stoker
Mejbah Bhuiyan
Alauddin Bhuiyan
Zaver Bhujwalla
Rajinder Bhullar
Adisak Bhumiratana
Anirban Bhunia
Alok Bhushan
Lokesh Bhushan
Jaikrit Bhutani
Yujing Bi
Xiaoning Bi
Yuhai Bi
Yanchao Bi
Lijun Bi
Weimin Bi
Huiquan Bi
Hongsheng Bi
Rui Bi
Xiaolin Bi
Xin Bi
Taiyong Bi
Pengpeng Bi
Tiago Biachi De Castria
Fantahun Biadglegne
Italo Biaggioni
Federico Biagi
Paolo Biagi
Philippe Biagini
Giuseppe Biagini
Bernard Bialecki
Suzanne Bialek-Davenet
Stephen Bialkowski
Andrea Bialocerkowski
Ronald Bialozyt
Michal Bialy
Ellen Bialystok
Zhuan Bian
Jiang Bian
Tao Bian
Ying Bian
Jian-Min Bian
Zhaoxiang Bian
Rongwen Bian
Xiaofang Bian
Yang Bian
Wei Bian
Shaomin Bian
Qian Bian
Carlo Bianca
Paolo Biancheri
Matt Bianchi
Riccardo Bianchi

Giacomo Bianchi
Francesca Antonella Bianchi
Renzo Bianchi
Marino Bianchin
Bianca Bianco
Piero Bianco
Antonino Bianco
Giuliana Bianco
Luigi Biancone
Ginestra Bianconi
Clotilde Biard
Alexandre Biasi
Emiliano Biasini
Fred Biasini
Deborah Biasoli
Matteo Biasotto
Daniele Biasucci
Constanza Biavaschi
Jean-Emmanuel Bibault
Kyle Bibby
Thomas Bibby
Patrick Biber
Peter Biberthaler
Nikolay Bibikov
Frederic Bibollet-Ruche
Rodrigo Bicalho
Goran Bicanic
Thomas Bice
Terry Jo Bichell
Delphine Bichet
Clara Bicho
Manuel Bicho
Balthasar Bickel
Jerome Bickenbach
David Bickford
Paula Bickford
Derek M Bickhart
Timothy Bickmore
Stephen Bickston
François-Clément Bidard
Mallikarjun (Arjun) Bidarimath
Sílvia Bidarra
John Biddlestone
Gavin Bidelman
Aurelie Bidet Caulet
Christel Bidet-Ildei
Tobias Bidon
Stefan Bidula
Aurelian Bidulescu
Christopher Bidwell
Joseph Bidwell
Kathleen Biebel
Erhard Bieberich
Peter Biedermann
Madeleine Bieg
Bonita Biegalke
James Bieker
Helle Bielefeldt-Ohmann
Diane Bielenberg
Christian Bieli
Robin Biellik
Katie Biello
Maximilian Bielohuby
Barbara Biemans
Frank Bienaimé
Przemyslaw Bienkowski
Rachelle Bienstock
Riccardo Bientinesi
Gabriele Bierbaum
Marc Bierkens
Joanna Biernacka
Bernhard Biersack
Jeremy Biesanz
Sander Biesbriek
Jan Bieschke
Leslie Biesecker
Karolien Biesheuvel-Leliefeld
Geert Biessels
Erik Biessen
Franck Biet
Francesco Bifari
Albert Bifet
Edward Biffin
Stefano Biffo
Maurizio Bifulco
Peggy Biga
Boris Bigalke
Anna Bigas
Tim Bigdeli
Naïke Bigé
Matthew Biggerstaff
Sarah Biggs
Manus Biggs
Adam Biggs
Greg Biging
Marcelo Bigliassi

Jean Joel Bigna
Elena Bignami
Darell Bigner
Christophe Bignon
Davide Bigoni
Maria Bigoni
Sarah Bigot
Subhash Bihani
Kelly Bijanki
Philippe Bijlenga
Maarten Bijlsma
Esmée Bijnens
S. D. Biju
Elisabeth Bik
Holly Bik
Boris Bikbov
Andreas Bikfalvi
Benjamin T. Bikman
András Bikov
Vadim Biktashev
Erhan Bilal
Philip Bilan
Antonio Bilancio
Jose Ramon Bilbao
Itxarone Bilbao
R. Bilder
Nazan Bilgel
Bahar Bilgen
Berkin Bilgic
Jacob Bilhartz
Tiago Bilhim
Robyn Bilinski
Cemil Bilir
Roslyn Bill
Aude Billard
Patrick Billard
Elisabeth Billard
Marie Billaud
Marc Billaud
Andrea Bille
Johan Billen
Adrian Billeter
Henny Billett
Philippe Billiald
Sylvain Billiard
Annelies Billiet
J. Billimeck
Martin Billinger
Sharon Billings
Molly Billings
James Michael Billingsley
Charlotte Billington
Charalambos Billinis
Konstantinos Billis
Pierre-Edouard Billot
Laurent Billot
Maxime Billot
Pierre Billuart
Henk Bilo
Grzegorz Bilo
Osman Bilsel
Dan Bilsker
Gonapenuwala Bimba
Sabrina Bimonte
Yue Bin
Yang Bin
Azizah Bin Mubayrik
James Bina
Olivier Binda
Markus Bindemann
Robert Binder
Marco Binder
Vera Binder
Ranjit Bindra
Francois Binet
Xiaoli Bing
Ulrike Bingel
Geoffrey Bingham
Daniel Bingham
Wu Bingli
Tobias Bingold
James Binley
Sandra Binning
Colin Binns
Bryce Binstadt
Amrei Binzer
Marcello Biocca
Andreia Biolo
Andrea Biondi
Antonio Biondi
Giuseppe Biondi-Zoccai
Silvia Bione
Leann Birch
James Birchler
Rainer Birck
Doug Bird

Chris Bird
Victoria Bird
Tannaz Birdi
Dawn Birdsell
Kelly Birdwell
Brigitte Birebent
Ruthie Birger
Claudia Birkemeyer
Andreas Birkenfeld
Edmund Birkhamshaw
Klaus Birkhofer
Irina Birman
Brenda Birmann
Rasmus Birn
Rebecca Birnbaum
Yochai Birnbaum
Teresa Birngruber
David Birnkrant
Inanc Birol
David Biron
Deirdre Birtles
Andrew Birtles
Konstantin Birukov
Praveen Birur
Marco Bisaglia
Martin Bisaillon
Filippo Biscarini
Federico Biscetti
Gian Battista Bischetti
Josef Bischofberger
Manuel Biscoito
Roberta Bisconti
Joseph Bisesi Jr
William Bishai
David Bishai
Mahendra Bishnoi
Kumar Sanjeev Bishnupuri
Alexander Bishop
Barney Bishop
Jeffrey Bishop
Martin Bishop
Nicholas Bishop
Christine Bishop
Melanie Bishop
Justin Bishop
Tom Bishop
Justin Avery Bishop
Anthony Bishopp
Nanette Bishopric
Naveen Bisht
Tatiana Bisi
Patrizia Bisiacchi
Marcia Bissaco
Ute Bissels
Sylvie Bisser
Patrick Bissett
Marie-Josee Bisson
Etienne Bisson
Ioannis Bistinas
Bruce Bistrian
Akshaya Biswal
Bichitra Biswal
Manisha Biswal
Tapan Biswas
Sumi Biswas
Kajal Biswas
Swati Biswas
Roopa Biswas
Arijit Biswas
Sampa Biswas
Sagarika Biswas
Manosh Biswas
Saswati Biswas
Animesh Biswas
Asim Biswas
Ashis Biswas
Tanuka Biswas
Partha Biswas
Sumalika Biswas
Khalil N Bitar
Youszef Bitar
Edward Bitarakwate
Davide Bitetto
Michael Bithell
Elena Bitocchi
Marcia Bitondi
Jacob Bitoun
Constantine Bitsaktsis
Douglas Bittel
Joerg Bittenbring
Wilbert Bitter
Alan Bittles
Eric Bittman
Stefan Bittner
Ava Bittner
Trever Bivona

Walter Bixby
Ryan Bixenmann
Edward Bixler
Necmi Biyikli
Pierre Bize
Mina Bizic-Ionescu
Benoit Bizimungu
Boris Bizumic
Mariano Bizzarri
Joe Bizzarro
Dan Bizzotto
Thomas Bjarnsholt
Anders Bjartell
Hein Bjerck
Jorgan Bjerggaard Jensen
Silje Bjerknes
Pernilla Bjerling
Mette Bjerre
Lise Bjerregaard
Morten Bjerregaard-Andersen
Peter Bjerring
Lena Björck
Jan Bjordal
Kathe Bjork
Linda Björkhem-Bergman
Peyman Björklund
Heidi Björklund
Geir Bjørklund
Anne Bjorkman
Anders Björkman
Christer Björkman
Olivia Bjorkquist
Johanna Bjorkroth
Niklas Björkström
Dale Bjorling
Lars Bjørndal
Hakan Björne
Toni Björninen
Ingunn Björnsdottir
Petter Bjornstad
Louis Bjostad
Ellen Blaak
Dieter Blaas
Bert Blaauw
Elizabeth Blaber
Jan Blacher
François Blachier
James Blachly
Agnieszka Blachnio-Zabielska
Robert Black
Lindsay Black
Brian Black
Graeme Black
Maureen Black
Dennis Black
John Black
Stephen Black
Christopher Black
Jennifer Black
Peter Black
Andrew Black
Georgia Black
Joyce Black
William Black Iv
Pat Blackall
Patrick Blackall
Jason Blackard
Jason Blackburn
David Blackburn
Adele Blackler
Chris Blackman
Daniel Blackmore
Emma Blackmore
Murray Blackmore
Perry Blackshear
Bradley Blackwell
Susanna Blackwell
Keith Blackwell
Nigel Blackwood
Tjeerd Blacquiere
Mark Blades
Joanna Bladowska
Phillip Blaen
Franz Blaes
Nadja Blagitko-Dorfs
Mg Blagitz
Marcus Blagrove
Jan Blaha
Roman Blaheta
Roger Blahnik
Bastien Blain
Harry Blair
David Blair
Ian Blair
Paul Blair
Carol Blair

Steven Blair
Jaime Blair
Allison Blair
Nat Blair
Karina Blair
Lauren Blair
Norman Blair
Cindy Blair
Peter Blair
Claudia Blais
Chris Blais
Daniel Blake
Garry Blakely
April Blakeslee
Eric Blanc
Francois-Xavier Blanc
Miguel Blanca
Gabriel Blanca
Elison Blancaflor
Anne Blanchard
Frederic Blanchard
Nicolas Blanchard
Caroline Blanchard
Stephane Blanche
Christophe Blanchet
Claudio Inostroza Blancheteau
Paola Blanchette
Christopher Blanchette
Patrick Blanco
Carlos Blanco
Fernando Blanco
Cynthia Blanco
Antonio Blanco
Carolina Blanco
Gerardo Blanco
Mar Blanco
Leocadio Blanco-Bercial
Maria Blanco-Prieto
Javier Blanco-Rivero
Giovanni Blandino
William Blaner
Christopher Blanford
Marta Blangiardo
Volker Blank
Fabian Blank
Robert Blank
Arthur Blank
James Blankenship
Terry Blankenship-Paris
Marco Blanker
Peter Blankestijn
Joseph Blankinship
Paul Blankman
Francois Blanquart
Ronald Blanton
Robert Blanvillain
Olivier Blarquez
José-María Blasco
Vincent Blasco-Baque
Francesco Blasi
Damian Blasi
Udo Bläsi
Janusz Blasiak
David Blask
Kristina Blaslov
Maria Blassioli-Moraes
Gregory Blatch
Marek Blatný
Rumsaïs Blatrix
Joseph Blattman
Christine Blattner
Justin Blau
Michael Blaut
Thomas Blauwblomme
Sergio Blay
Maame Blay
Jeremy Blaydes
Charlotte Blease
Jens Blechert
Christoph Bledowski
J. Bledsoe
Stefan Bleeck
Sacha Bleeker
Alexandre Bleibtreu
Nienke Bleijenberg
Konstantinos Blekas
Cherie Blenkiron
Andreas Blennow
Javier Blesa
Elisabeth Blesbois
Inger Bleskestad
Molly Bletz
Josefa Bleu
Sophie Bleves
Lynn Blewett
Yannick Bleyenheuft

Ronald Bleys
Theo Blick
Ray Blick
Reinhard Blickhan
S Bliddal
Kevin Blighe
Belinda Blignaut
Klaus Blischke
James Bliska
Jackie Blissett
Bradley Blitvich
Dawn Blitz
Nikolay Bliznyuk
Michael Bloch
Michelle Block
Hannah Block
Geoffrey Block
Jon Block
Marc Block
Christophe Blockeel
Oswald Bloemen
Elisabeth Bloemena
Sandra Bloemendal
Jos Bloemers
Vivian Blok
Melanie Blokesch
Harry Blokhuis
Elma Blom
Anders Blomberg
Erik Blomberg
Eric Blomme
Anders Blomqvist
Eva Blomstrand
Marc Blondel
Danielle Blondel
Carlos Blondel
Stacy Blondin
Brittany Bloodhart
Nathaniel Bloodworth
Paul Bloom
Michael Bloom
Frank Bloomfield
Gerald Bloomfield
Susan Bloomfield
Robert Bloomfield
Jeffrey Bloomquist
Frank Bloos
Gerrit Bloothooft
Mark Blostein
Nicolas Blot
Hervé Blottière
Dieter Blottner
Jean Blouin
Jean-Sebastien Blouin
Paul Blount
Peter Blouw
Matthew Blow
Frances Blow
Matthias Bluemke
Burton Bluhm
Martin Blum
Alan Blum
Kenneth Blum
Christian Blum
Andrew Blumberg
Jochen Blumberger
Martin Blume
Margaret Blume-Kohout
Miroslav Blumenberg
Olga Blumenfeld
Efrat Blumenfeld-Lieberthal
Deena Blumenkrantz
Justin Blumenstiel
Anna Blumental-Perry
Scott Blumenthal
Przemyslaw Blyszczuk
Benjamin Blyth
Hazel Blythe
Michaela Blyton
Simona Bo
Marzia Bo
Yongli Bo
Chen Bo
Lai Bo
Zhang Bo
Sally Boa
Lisa Boardman
Jason Boardman
William Boarman
Adeline Boatin
Jeffrey Boatright
Alessio Boattini
M. Bobbert
Gerd Bobe
Larissa Bobermin
Magdalena Bobowik

Kirsten Bobrow
Stefano Boccaletti
Franck Boccara
Gabrielle Bocchese Da Cunha
Daniele Bocchiola
Giuseppe Boccignone
Marie-Luce Bochaton-Piallat
Tomasz Bochenek
Herve Bocherens
Thomas Bochynek
Jan Bocianowski
Christoph Bock
Hans Bock
C. Thomas Bock
Michael Bock
John Bock
Christian Bock
Susan Bock
Dan. G. Bock
Linda Bockenstedt
Carlos Bocos
Christian Bocti
László Bodai
Laszlo Bodai
Olaf Bodamer
Manish Bodas
Dhananjay Bodas
Sandeep Bodduluri
Lynne Boddy
Louis Boddy
Antonio Bode
Michael Bode
Nikolai Bode
Clara Bodelon
Lisa Boden
Joseph Boden
Olivier Bodenreider
Rogier Bodewes
Loys Bodin
Sue Bodine
Benedetta Bodini
Robert Bodizs
Rolf Bodmer
Lisa Bodnar
Glen E. Bodner
Martin Bodner
Levente Bodrossy
A. Bodzenta-Lukaszyk
Lucas Boeck
Michael Boeckh
Brigitte Boeckmann
Carsten Boehler
Michael Boehm
Manfred Boehm
Christine Boehmer
Jan Boehnke
Jef Boeke
Kim Boekelheide
Wilbert Boelens
Ronald Boellaard
Sofia-Natalia Boemi
Marianne Boeni-Schnetzler
Jolanda Boer
Christa Boer
Diana Boer
Derek Boerboom
Joel Boerckel
Wout Boerjan
Ties Boerma
Julie Boerner
Olaf Boernsen
James Boers
Philipp Boersch-Supan
Dirk-Jan Boerwinkel
Claudia Boesmueller
Sanne Boesveldt
Sylvain Boet
Pieter Boets
Angelika Boettger
Charlotte Boettiger
David Boettiger
Tobias Boettler
Philippe Boeuf
Michael Boffa
Jean-Jacques Boffa
Jody Boffa
Amy Bogaard
Harm Bogaard
Sergé Bogaerts
Eliene Bogaerts
Alemtsehay Bogale
Jonathan Bogan
Clifton Bogardus
Jim Bogart
G. Bogat
Peter Bogaty

Fausto Bogazzi
Ryan Bogdan
Volodymyr Bogdanov
Anna Bogdanova
Wieslaw Bogdanowicz
Sara Bögels
Daniel Bogema
Heather Boger
Emma Boger
Carsten Böger
Toine Bogers
Vijay Boggaram
M.M. Boggess
Jose Boggia
Nancy Boghossian
Marija Bogic
Giuseppe Bogliani
Paul Bogner
Franz Bogner
Lydia Bogomolnaya
Marina Bogomolov
Roman Bogorad
Bjarte Bogstad
Magdalena Boguta
Johannes Bohacek
Carolyn Bohach
Michelle Bohan Brown
Richard Bohannon
Jonathan Bohbot
Holger Bohlmann
Stephan Böhm
Björn Bohman
Benjamin Bohman
Stefan Böhmdorfer
Frank D. Böhmer
Ana Böhmer
Erwin Bohn
Rebecca Bohn
Jens Bohne
Delwayne Bohnenstiehl
Vanessa Bohns
Tanggis Bohnuud
Gil Bohrer
Lynn Bohs
Sean Bohun
Costin-Anton Boiangiu
Michele Boiani
Didier Boichard
Eric Boilard
Adrien Boillot
Alexandra Boing
Krishna Boini
Denis Boire
Gilles Boire
Adrienne Boire
Pascal Boireau
Yves Boisclair Boisclair
Lawrence Boise
Mogens Boisen
Detlev Boison
Marie-Christophe Boissier
Catherine Boisson-Vidal
Isabelle Boisvert
Maude Boisvert
Alice Boit
Luigi Boitani
Carla Boitani
Scott Boitano
Felix Boivin
Ingo Bojak
Rafael Bojalil
Mirela Bojan
Piotr Bojarski
Andrzej Bojarski
Ognjen Bojovic
Maria Bokarewa
Hatice Boke
Reinoud Bokkers
Kamila L. Bokszczanin
Roland Bol
C. Boland
C. Richard Boland
Julie Boland
Torrey Boland
Pauline Boland
Sorana Bolboaca
Christopher Bolch
Kata Bolcskei
Ingrid Boldin
Fernas Boldizsar
Renzo Boldorini
Blaise Boles
Eckhard Boles
Albert Bolhuis
Nomesh B. Bolia
Saikat Boliar

Davide Bolignano
Valerie Bolivar
L. Bolkun
Matthias Boll
Wendy Bollag
Mariela Bollati-Fogolin
Johan Bollen
Christian Bollensdorff
Bethany Bolling
Kathryn Bollinger
Elfriede Bollschweiler
Deborah Bolnick
Matteo Bologna
Monique Bolotin-Fukuhara
Toby Bolsen
Elyse Bolterstein
James Bolton
Kirsty Bolton
Kristy Bolton
Carolyn Bolton-Moore
Valerie Boltz
Elisabeth Bolund
Hanno Bolz
Christiano Bombardi
Aureliano Bombarely
Jennifer Bomberger
Yannick Bomble
Arne Bomblies
Barbara Bomfim
Ananth Bommakanti
Sébastien Bommart
Richard Bomphrey
Morgane Bomsel
Robert Bonacci
M. Bonaccio
Juan Bonachela
Fabrizia Bonacina
Marino Bonaiuto
Damien Bonal
Martin Bonamino
Carla Denise Bonan
Carl Bonander
Enrica Bonanni
Joseph Bonanno
Laura Bonati
Mario Bonato
Anthony Bonato
M. Bonato
Marcel Bonay
Bruno Bonaz
Matteo Bonazzi
Danail Bonchev
Jason Bond
Robert Bond
Christine Bond
Virginia Bond
Alexander Bond
Chelsea Bond
Carol Bond
Michelle R. Bond
Eduardo Bondan
Ana-Nicoleta Bondar
Vladyslav Bondarenko
Robert Bonde
Isabel Bondia Pons
Catherine Bondonno
Anna Bone
Timothy Bonebrake
Charlotte Bonefeld
Eva Bonefeld-Jorgensen
Francesco Bonella
Mara Bonelli
Pierluigi Bonello
Marc Bonenberger
Bruno Bonetti
Lynda Bonewald
R. Bonfil
Patricia Bonfim-Mendonça
Stephan Bongard
Lennart Bongartz
Raoul Bongers
Massimo Bongiovanni
Lars Bongo
Peta Bonham-Smith
Francois Bonhomme
Paolo Bonilauri
Vera Bonilha
Carolina Bonilla
Luis Bonilla
Francisco Bonilla-Escobar
Robert Bonin
Luca Bonini
Claudia Bonini-Domingos
Ferruccio Bonino
Andrea Bonisoli Alquati
Marco Bonizzoni

Stefan Bonn
Camille Bonneaud
Jean-Francois Bonnefon
Amélie Bonnefond
Amelie Bonnefond
Mathilde Bonnefond
Tyler Bonnell
Guusje Bonnema
Matthew Bonner
Caroline Bonner
Daniel Bonner
Susan Bonner-Weir
Dominique Bonnet
Xavier Bonnet
Aurélie Bonnet
Pascal Bonnet
Shilah Bonnett
Marc Bonneville
Kristin Bonnie
Remy Bonnin
Céline Bonnyaud
James Bono
Silvia Bono
Hernán Bonomi
Marco Bonomi
Francesca Bonomini
Robert Bonomo
Adriana Bonomo
Marisa Bonsignore
Carmelo Bonsignore
Jean-Daniel Bontemps
Marc Bonten
David Bonter
Sébastien Bonthoux
Reza Bonyadi
Istvan Bonyhay
Neeltje Boogert
Robert Booher
Linda Booij
Geert Booij
Jan Booij
Thijs Booiman
Adam Book
Squire Booker
Sam Booker
Fred Bookstein
Mieke Boon
Reinier Boon
Wouter Boon
Charles Boone
John Boone
Jan Boone
Janne Boone-Heinonen
Harrie Boonen
Chanchai Boonla
Rudy Boonstra
André Boonstra
Tjitske Boonstra
Peter Boor
Kenneth Boorom
Margarete Boos
Anja Boos
Walter Boot
Arnoud Boot
Jonathan Boote
David Booth
Martin Booth
Warren Booth
Sarah Booth
Tom Booth
Jean Paul Booth
Ross Booth
Mark Booth
R. Booth
Thomas Boothby
Lynda Boothroyd
Martin Bootman
Didier Boouhassira
Robert Booy
George Booz
Jean Chrisostome Bopassa
Andrea Bor
Emre Bora
Puran Bora
Samudragupta Bora
Namrata Bora
Diana Boraschi
Alisdair Boraston
Iman Borazjani
Manuel Borca
Peter Borchardt
Sangeeta Borchatai
David Borchelt
Christoph Borchers
Annette Borchers
Glen Borchert

Katherine Borden
Victor Borden
Antonio Borderia
Magnus Bordewich
Christophe Bordi
Philippe Bordie
Rémy Bordonné
Albert Bordons
Ingrid Borecki
Rajeev Boregowda
Amy Borenstein
Elhanan Borenstein
Katarina Borer
Susan Boretius
Justin Borevitz
Melissa Borg
Michael Borg
Javier Borge-Holthoefer
Luca Borger
Ricardo Borges
Karin Borges
Adolfo Borges
Helena Borges
Atila Borges
Bárbara Borges
Boniek Borges
Paulo Borges
Nica Borgese
Márcio Borges-Martíns
Christian Borghesi
Anna Borghi
Claudio Borghi
Elisa Borghi
Andrea Borghini
Audrey Borghi-Silva
Marta Borgi
Anders Borgkvist
Christine Borgman
Stefan Borgwardt
Hossein Borhan
Giuseppe Boriani
Michael Borich
Eduardo Borie
Sara Borin
Calderon Boris
Jeffrey Boris
Alex Borisenko
Judith Borja
Mark Borja
Steven Borkan
Christopher Borkent
Megan Borkum
Barry Borlaug
Joerg Bormann
Jörg Bormann
Helge Bormann
Giacomo Bormetti
Yan Borné
James Borneman
Boris Bornemann
Helmar Bornemann-Cimenti
Christoph Borner
Jan Börner
Martin Bornhäuser
Stefan Bornholdt
Daniela Börnigen
Ina Bornkessel-Schlesewsky
Lutz Bornmann
Mark Borodovsky
Florentino Borondo
Mihaly Boros
Ilya Borovok
James Borowiec
Richard Borowsky
Alexander Borowsky
Ronald Borra
Pedro Borralho
Ester Borras
Jm Borràs
Carles Borrego
Eli Borrego
Jose Borreguero
Víctor Borrell
Ferran Borrell
Joseph Borrelli
Juan Borrero Del Pino
Adriana Borriello
Steffen Borrmann
Riccardo Borroni
Dasiel Borroto Escuela
Ray Borrow
Luigimaria Borruso
Pascal Borry
Elisa Borsani
Marlene Borschel
Simone Borsci

Alessandra Borsetti
Terry Borsook
Jan Willem Borst
Jelmer Borst
Luke Borst
Mark Borsuk
Ari Borthakur
Lee Borthwick
Carl Bortner
Marta Bortoletto
Raul Bortolin
Catherine Bortolon
Stefania Bortolotti
Renata Bortolus
Luca Bortolussi
Stefania Bortoluzzi
Eduardo Bortoluzzi
Judit Bort-Roig
Eric Bortz
Stanislav Borysov
Peter Bos
Martine Bos
Johan Martijn Bos
Lieuwe Bos
Daniel Bos
Arend Bos
Arjan Bos
Elisabeth Bos
Silvano Bosari
Pedro Boscan
Silvia Boscardin
Elisa Boscari
Jackie Bosch
Laura Bosch
Jurgen Bosch
Xavier Bosch
Assumpcio Bosch
Thijs Bosch
Pablo Bosch
Mark Boschen
Federica Boschetti
María Laura Boschiroli
Ciril Bosch-Rosa
Francesca Boscia
Alejandra Bosco
Francis Boscoe
Giulano Boscutti
Jizzo Bosdriesz
Michael Bose
Susmita Bose
B. Bose
Jayakumar Bose
Jeff Bose
Thomas Bose
Cristina Bosetti
Valentina Bosetti
Helena Boshoff
Marcello Bosi
Federico Bosia
Steven Bosinger
Catharine Bosio
Adele Boskey
Zoran Boskovic
Jasminka Boskovic
Maarten Bosland
Paul W. Bosland
Madeleen Bosma
Andrea Bosman
Giel Bosman
Fredrik Bosman
Samantha Bosman
Darko Bosnakovski
Cynthia Bosquillon
Iris Bosschem
Marianne Bosscher
Jonathan Bossenbroek
Patrick Bosshart
Antonio Bossi
Peter Bossier
A. Bossios
Cara Bossley
Tanja Bossmann
Luciano Bosso
Xavier Bossuyt
Franky Bossyut
Moriah Bostian
Chris Bostick
Kristina Bostrom
Pontus Bostrom
Stephanie Bostrom
Kim Boström
Elisabeth Boström
William Bosu
Hayden Bosworth
Rain Bosworth
Anja Bosy-Westphal

Icaro Boszczowski
Zsuzsanna Bosze
Arjan Bot
Simona Bota
Constantin Bota
Vladimir Botchkarev
Revaz Botchorishvili
Ana Botelho
Hugo Botelho
Maria João Botelho
Jose Botella
Juan Botero
Fidel Botero-Castro
Mona Botezatu
A. J. Both
Anna-Maria Botha
Graham Bothamley
Brian Bothner
Alfred Lm Bothwell
Cristina Botías
Johan Botling
Luis Boto Lopez
Istvan Botos
Diomidis Botsikas
David Botstein
Nicholas Bott
Maurizio Botta
Federico Botta
Amy Botta
Roberto Botta
Giovanni Bottari
Yvonne Böttcher
Sindy Böttcher
Giovanni Bottegoni
Françoise Botterel
Evelyn Böttger
Sara Botti
Emmanuel Bottieau
Marco Bottino
Walter Bottje
Alex Bottle
Florencia Botto
Matthew Bottomley
Isadora Botwinick
Germán Bou
Raed Bou Matar
Jean-David Bouaziz
Laura Boucai
Muriel Boucart
Pavel Bouchal
Jean-Philippe Bouchara
Josee Bouchard
Charles Boucher
Jeremie Boucher
Etienne Boucher
Georgios Bouchouras
Drion Boucias
Kimberley Bouckaert
José Boucraut
Emmanuel Boucrot
Megan Boudewyn
Naïla Boudiaf
Brendon Boudinot
Francois Boudreau
Stephen Boue
Guenaelle Bouet
David E. Boufford
Soufiane Boufous
Julia Boughner
Yacine Bouguezza
Kamal Bouhadir
Didier Bouhassira
Eric Bouhassira
Roger Bouillon
Steven Bouillon
Jamal Bouitbir
David Boukal
Chantal Boulanger
John Boulanger
Pierre Boulanger
Elizabeth Boulding
Salix Boulet
Pascale Boulet
Claire Bouleti
Thomas Boulin
Maria Boulina
Thierry Boulinier
Daniel Boullosa
Etienne Boulter
Michel Boulvain
Yap Boum Ii
Gerrit Bouma
Esther Bouman
Dimitrios Boumpas
Kyria Boundy-Mills
Vassiliki Bountziouka

Jerome Bouquet
Mathieu Bourbonnais
Emmanuel Bourdon
Estelle Bourdon
Myriam Bourens
Anne Bourgarit Durand
Cyril Bourgeois
Sacha Bourgeois-Gironde
Sylvain G. Bourgoin
Olivier Bouriaud
Jason Bourke
Liam Bourke
Virginie Bourlier
Stylianos Bournazos
Rupert Bourne
Barbara Bournet
Lydia Bourouiba
Charles Bourque
Francois Bourque
Melanie Bourque
Pierre Bourquelot
Franck Bourrat
Olivier Bourron
Tamar Boursalian
Kostas Bourtzis
Latifa Bousarghin
Aziz Bousfiha
Mekki Boussaha
Hamouda Boussen
Alex Boussioutas
Andre Boustany
Nada Boustany
Stan Boutin
Jean Boutin
Marc Boutry
Helene Bouvaist
Daniel Bouvard
Romaric Bouveret
Michael Bouvet
Benjamin Bouvier
Marlene Bouvier
Stefan Bouwense
Jan Bouwknecht
Hindrik Bouwman
Jessica Bouwmeester
Jérémy Bouyer
Thierry Bouyssou
Karim Bouzakri
Anne-Karine Bouzier-Sore
Elena Bouzinova
Dana Howard Bovbjerg
Paola Bovolenta
Richard Bowater
Saman Bowatte
Christopher Bowd
Nikola Bowden
Rachel Bowden
David Bowden
Anton Bowden
Henrietta Bowden-Jones
Dawn Bowdish
Scott Bowdridge
Steven Bowe
Timothy Bowen
David Bowen
Scott Bowen
Gabe Bowen
Ashley Bowen
Holly Bowen
Mary Bowen
Justin Bower
Kelly Bower
K. Bowering
Robert M. Bowers
Barbara Bowers
Albion Bowers
Michael Bowes
Rauri Bowie
William Bowie
Abdalla Bowirrat
Kristina Bowles
Robert Bowles
David Bowles
Terry Bowlin
Nathan Bowling
B. Bowling
Alan Bowman
Dwight Bowman
Howard Bowman
Aaron Bowman
Barry J Bowman
Gregory Bowman
Doug Bowman
Nicholas Bowman
Timothy Bowman
Andrew S Bowman

Teresa Bowman
James Bown
Hannah Bowrey
Julia Bowsher
Joanna Bowtell
R. Bowyer
Adam Boxer
Patricia Boya
Kevin Boyack
Maxim Boyanov
Ross Boyce
Robert Boyce
Heather Boyd
Charlotte Boyd
Norman Boyd
David Boyd
Brian Boyd
Russ Boyd
Mette Boye
Catherine Boyen
Paul Boyer
Laurent Boyer
James Boyer
Michael Boyer-Guittaut
Doug Boyette
Emma Boyland
Stephen Boyle
Joseph Boyle
Jon Boyle
Keith Boyle
Sarah Boyle
Patrick Boyle
Kathleen Boyle
Alice Boyle
Theresa Boyle
Fran Boyle
Michelle Boyle
James Boyle
Onur Boyman
Michael Boyne
Murat Boysan
Gunnar Boysen
Gergely Boza
Panayiotis Bozanis
G. Bozdag
Mithat Bozdayi
Yves-Marie Bozec
Ibrahim Bozgeyik
Ioannis Boziaris
Tanja Bozic
Alper Bozkurt
Latife Bozkurt
Joel Bozue
Tom Bozza
Patricia Bozza
Marco Bozzali
Fiammetta Bozzani
Federico Bozzetti
Yuri Bozzi
Ineke Braakman
Barbara Braams
Daren Brabham
Leslie Braby
Giorgia Bracaglia
Annalisa Bracco
Chloe Bracis
Ken Bracke
Cameron Bracken
John Brackenbury
Gilles Brackman
Doug Brackney
Ruth Brack-Werner
Chiara Braconi
Alexander Braczkowski
Nicola Bradbear
Charles Bradberry
Elizabeth Bradbury
Janet Bradbury
Neil Bradbury
Kathryn Bradbury
Oliver Braddick
Peter Bradding
Thomas Brade
Barry Bradford
Rod Bradford
Cortney Bradford
Daniel Bradford
Steven Bradfute
Brian Bradke
Monika Bradl
Kenneth Bradley
Peter Bradley
Daniel Bradley
Elizabeth Bradley
Mark Bradley
Declan Bradley

John Bradley
William Bradley
Barbara Bradley
Paul Bradley
Elspeth Bradley
Sean Bradley
Darcy Bradley
Shelton Bradrick
Amy Bradshaw
Patrick Bradshaw
Nicholas Bradshaw
Elizabeth Bradshaw
Pamela Bradshaw
Bruce Bradtmiller
L. Jeannine Brady
Michael Brady
Matthew Brady
Oliver Brady
Jeff A. Brady
Carrie Brady
Kevin Braeckmans
Senne Braem
Toon Braeye
Walter Braga
J. Braga
Alfésio Braga
Jefferson Braga Silva
Allison Brager
Marie Bragg
Jennifer L. Bragg-Gresham
Esteban Braggio
Alberto Braghiroli
Anatol Bragin
Anastasia Bragina
Luca Braglia
Michel Brahic
Mayur Brahmania
Heena Brahmbhatt
Sheryl Brahnam
Janice Brahney
Fulvio Braido
George Brainard
Daniel Brainard
Julii Brainard
Victoria Braithwaite
David Braithwaite
Vickie Braithwaite
Francois-Xavier Brajot
Scott Brakenridge
Axel Brakhage
Lea Brakier-Gingras
Barbara Bramanti
Vincenzo Bramanti
Viviana Brambilla
Naomi Bramhall
Kate Bramham
Marc Bramkamp
Pablo Brañas-Garza
Antoine Branca
Rosa Tamara Branca
Paola Brancaccio
Pedro Henrique Brancalion
Andrea Branch
Marc Branch
Pascal Branchereau
Simone Branchini
Ricardo Branco
Rita Branco
Brett Branco
Claudio Brancolini
Michael Brand
Stephan Brand
Denys Brand
Matthias Brand
Serge Brand
Judith Brand
Tilman Brand
Luise Brand
Sinval Brandão Filho
Margaret Brandeau
Jean-Philippe Brandel
Vincent Brandenburg
Klaus Brandenburg
Kenneth Brandenburg
Claire Brandenburger
Elke Brandes
Sebastian Brandhorst
Maria Luisa Brandi
Cristina Brandileone
Simon Brandl
Eva Brandl
Stephanie Brändlein
Talia Brandman
Pietro Brandmayr
Johanna Brandner
Scott Brandon

Beate Brand-Saberi
Ivan Brandslund
Joost Brandsma
Alexander Brandt
Curtis Brandt
Marilyn Brandt
Eric Brandt
Ulrich Brandt
Laura Brandt
Mark Brandt
Adam Brandt
Paul Brandt-Rauf
Marc Branham
Wojciech Branicki
Jessica Brann
Richard Bransford
Ryan Branski
Richard Branson
Sara Brant
Susan Brantley
Jose Bras
Dawn Brasaemle
Jan Brascamp
Christopher Bräsen
Matthew Brashears
James Brasic
Pedro Brasil
Patrice Brassard
Susan Brasser
Kevin Brasseur
Charlotte Brassey
Rachel Brathwaite
Scott Bratman
Sebastian Brauchi
Veronique Braud
Stanton Braude
Fred Brauer
Juliane Bräuer
Thomas Braulke
Jeff Brault
Marie Brault
Ilka Braumann
Jonathan Braun
Hans Braun
Werner Braun
Andrew Braun
Thomas Braun
Rosemary Braun
David Braun
Armin Braun
Daniel Braun
Clait Braun
Camrin Braun
Frank Braun
Juergen Braun
Danielle Braun
Matthias Braun
Christina Braun
Ralf Braun
Josephine Braun
Jerome Braun
Julia Braungart-Rieker
Andreas Bräuninger
Christina Braunsdorf
Miriam Braunstein
A. Braunstein
Gerhard Braus
J. Braverman
Alejandra Bravo
Jeronimo Bravo
Ignacio Bravo
Elena Bravo
Jose Bravo-Cordero
George Bray
Timothy Bray
Molly Bray
Nicholas Bray
Jon Bray
Gloria Braz
F. Braza
Václav Brázda
Derek Brazil
James Brazill-Boast
Adriana Breaban
Gloria Brea-Calvo
Emmanuel Breard
Lieven Brebels
Nicholas Brecha
Andre Brechmann
Jeffrey Brecht
Stewart Breck
Broder Breckling
Karine Breckpot
Annette Breckwoldt
Michael Breckwoldt
Laura Breda

Marc Brede
Cato Brede
Nicolas Bredeche
Thomas Bredow
Andrew Breed
Martin Breed
Odalisca Breedy
Ronan Breen
Leigh Breen
Conor Breen
Clarissa Breen
Elizabeth Breeze
Klaus Brehm
Byron Brehm-Stecher
Achim Breiling
Ari Breiner
Samuel Breit
Andreas Breit
Sarah Breitbach
Mya Breitbart
Michael Breitenbach
Luana Cassandra Breitenbach Barroso Coelho
Hans-Georg Breitinger
Katja Breitkopf-Heinlein
Rainer Breitling
Rolf Brekken
Graciela Brelles-Mariño
Björn Brembs
Corinna Bremer
Erhard Bremer
Viviane Bremer
Katharina Bremer
Anja Bremm
Victor Brena-Medina
Rachel Brenchley
Andrea Brenciani
Jeffrey Brender
Heather Brenhouse
Simone Frédérique Brenière
Bertram Brenig
J. Brenna
Patrick Brennan
Marian Brennan
Timothy Brennan
Lisa Brennan
Meadhbh Brennan
Adrian Brennan
Irina Brennan
Rex Brennan
Martin Brennan
Tracy Brennand
Mark Brennan-Ing
Christian Brenneis
Charles Brenner
Eli Brenner
Robert Brenner
David Brenner
Alina Brenner
Christoph Brenner
Erich Brenner
Thorsten Brenner
Chad Brenner
Darren Brenner
Roger Brent
Lauren Brent
Benjamin Brent
Andrew Brent
Colin Brent
Marília Brescia
Guilherme Bresciani
Roberto Bresciani
Jerome Breslin
Fabiana Bressan
Silvia Bressan
Laurence Bresson-Bepoldin
Stephane Bretagne
Anne Bretagnolle
Lionel Bretillon
Michael Brett
Marianne Brett
Alexis Bretteville
Alison Brettle
David Brett-Major
Christian Brettschneider
Eefjan Breukink
Robert Breunig
Marianne Breuninger
Abraham Breure
Sebastien Breurec
Ingrid Breuskin
Rossella Breveglieri
Jason Breves
Gerhard Breves
Diego Breviario
Tiziana Brevini
Bruce Brew

David Brewer
Warrick Brewer
Michael Brewer
Tom Brewer
Rachel Brewer
Tom D. Brewer
Judson Brewer
Chris Brewin
Robert Brewin
Liz Brewster
William Brey
Leonid Breydo
Rachel Breyta
Antoine Brezin
Rachel S. Brezis
Emilio Bria
Nadege Briancon
Serge Briançon
Anne Briancon-Marjollet
Enora Briand
Chiara Briani
Norton Brianna
Laurence Briant
Ana Bribian
Jane Brice
G. Brichetto
Genevieve Bricheux
Ian Bricknell
Brian Bride
Claire Bridel
Pierre Bridevaux
Tom Bridge
Diane Bridge
Eli Bridge
Dave Bridges
Francis Bridges
Laura Bridgewater
Arika Bridhikitti
Kim Bridle
David Bridwell
Jason Bried
Elodie Briefer
William Brieger
Jürgen Brieger
David Brieger
Matthias Briel
Aenne Brielmann
Andre Briend
Yves Briers
Benny Briesemeister
Jorge Brieva
Michael Briga
Tanja Brigadski
John Brigande
Ryan Brigante
Alberto Briganti
Derek Briggs
Winslow Briggs
Farren Briggs
Scott Briggs
Robert Briggs
Adam Briggs
Melissa Briggs
R. Mark Brigham
Christopher Brigham
Filippo Brighina
Jen Bright
Michael Bright
Molly Bright
Manfred Brigl
Maurizio Brigotti
Antonino Briguglio
Volker Briken
Vera Bril
David Briles
Ilene Brill
Richard Brill
Thomas Brill
Stefano Brillanti
Matteo Brilli
Mitchell Brin
Jan Brinchmann
Ingar Brinck
Nicholas Brindle
Isabele Bringhenti
Marisa Brini
Kirstin Brink
Rene Brink
Gunnar Brinkmalm
Don Brinkman
Ashley Brinkman
Benjamin Brinkmann
Folke Brinkmann
Ralf Brinkmann
Candice Brinkmeyer-Langford
Ralph Brinks

Martin Brinkworth
Emma Brint
Jean-Pierre Brion
Christian Brion
Ana Briones
Óscar Briones
Marco Briones-Orta
Karine Briot
Cynthia Brisac
Meredith Brisco
Antonio Briseño
Francesca Brisigelli
Olivier Brissaud
Sylvain Brisse
Normand Brisson
Dustin Brisson
Vania Brissos
Claire Bristow
Carlos Brites
José Brito
Paulo Brito
Daniel Brito
Leandro Brito
Fabiane Brito
Andre Brito
Rosangela Brito
Steven Britt
Rodney Britt
David Britt
Evan Brittain
Katie Brittain
Marcelo Britto
Kate Britton
Adam Britton
Jennifer Britton
Oliver Britton
Eric Britzke
Ali Brivanlou
Maurizio Brivio
Kevin Brix
Carina Brixval
Maria Felice Brizzi
Gavin Broad
Kev Broad
Kelsy Broadaway
Jillian Broadbear
Adam Broadhead
Gloria Broadwater
Patricia Brocardo
Stefania Brocca
Egidio Brocca-Cofano
Francoise Brochard-Wyart
Bruno Brochet
Christian Brochmann
Christopher Brochu
Roland Brock
Patrick Brock
Marianna Brock
James Brock
Neil Brockdorff
Stefan Brocke
Warren Brockelman
Thomas Brocker
Chad Brocker
Susan Brockerhoff
Anne Brockhaus
Inka Brockhausen
Mark Brockman
Axel Brockmann
Kathrin Brockmann
Marc Brockmann
Timo Brockmeyer
David Brockwell
Arndt Bröder
Nichole Broderick
Hugh Broders
Scott Brodie
Jon Brodie
Bertha Brodin
Patrik Brodin
Edward Brodkin
Thomas Brodnicki
Tim Brodribb
Alexander Brodsky
Cláudia Brodskyn
Pnina Brodt
Lori Brody
Corey Broeckling
Olivier Broennimann
Stefan Broer
Inge Broer
Heike Broetz-Oesterhelt
Søren Brofeldt
María Noel Brogger
Juli Broggi
Massimo Broggini

Simone Brogi
Ariane Brogliato
Steven Broglio
Karim Brohi
Eran Brokovich
Karl A. Brokstad
Kristina Broliden
Susana Brom
Timothy Bromage
Karl Broman
Jennifer Bromberg-White
Michael Bromley
Thomas Bromley
Alain Bron
Peter Bron
Ruben Brondeel
Jean-Marc Brondello
Elizabeth Brondolo
Francisca Bronfman
David Broniatowski
Katarzyna Broniowska
Christian Bronner
Jean-Pierre Bronowicki
David Brook
Ryan Brook
Barry Brook
Benjamin Brooke
Greg Brooke
Rohan Brooker
Matthew Brookes
James Brooks
Brian Brooks
David Brooks
Thomas Brooks
Dana Brooks
Ellen Brooks
Amanda Brooks
Cassandra Brooks
Mark Brooks
Ellen Brooks-Pollock
Deirdre Brophy
Thomas Broquet
Robert Brosh
Amanda Brosnahan
M. J. Brosnan
Sebastien Brosse
Christine Brostjan
Witold Brostow
Kyle Brothers
Julia Brotherton
Marco Brotto
Margit Brottveit
Eugenia Broude
Richard Broughton
Emmanuel Brouillet
Jonathan Brouillette
Gueorgui Broukhanski
Benedicte Brounais
Josiane Broussard
Emmanuel Broussolle
Elizabeth Brouwer
Miranda Brouwer
Melissa Brouwers
Mariya Brovchenko
Andrea Brovelli
Howard Browman
Eric Brown
Grant Brown
William Brown
Culum Brown
C. Titus Brown
Judith Brown
Wendy Brown
Charles Brown
Jeremy Brown
Emery Brown
Christopher Brown
Janine Brown
Alan Brown
Mary Brown
Kevin E. Brown
Steven Brown
Matthew Brown
Celeste Brown
Stuart Brown
Susan Brown
Heidi Brown
Kevin Brown
Gordon Brown
Deborah Brown
Lindsay Brown
Justin Brown
James Brown
Biobele Brown
Kathleen Brown
Ann Brown

Lee Brown
Richard Brown
Adam Brown
Peter Brown
Lou Ann Brown
Andrew Brown
Calum Brown
Christian Brown
Lisa Brown
Katherine Brown
Federico Brown
Adrian Brown
Jill Brown
Cecil Brown
Casey Brown
Allan Brown
A. C. Brown
Jesslyn Brown
Julie Brown
Lawrence Brown
Pamela Brown
Helen Brown
Lydia Brown
Graham Brown
Monique Brown
Rachel Brown
Ian Brown
Timothy Brown
Juanita Brown
Paul Brown
Daniel Brown
Veronica M Brown
Edwina Brown
Gregory Brown
Andre Brown
Anthony Brown
Cathleen Brown Crowell
Sigal Brown Horowitz
Holly Brown-Borg
Richard Browne
Nicola Browne
Liam Browne
Matthew Browne
Duncan Browne
Mark Anthony Browne
Dillon Browne
Cameron Browne
Isaac Brownell
Anna-Liisa Brownell
Jess Brownell
Jeffrey Browning
Robert Browning
Jeremy Brownlie
Roger Brownsey
Michael Brownstein
Ryan Broxterman
Joseph Brozinick
Thomas Brozoski
Anna Brozyna
Julius Brtko
John Brubacher
Vicky Bruce
Barry Bruce
Ian Bruce
David Bruce
Toby Bruce
Heather Bruce
Volker Bruchert
Marcel Bruchez
Jason N. Bruck
Thomas Brück
Robert Brucker
Tim-Allen Bruckner
Katja Brückner
Reinhold Brückner
Dunja Bruder
Kristoffer W. Brudvik
Wolfgang Brueckl
Michael Bruegger
Doerthe Brueggmann
Annette Bruehl
Jason Bruemmer
Meg Bruening
Ronny Bruffaerts
Michael Bruford
Doug Brugge
Leslie Bruggeman
Jeroen Bruggeman
Richard Bruggeman
Frank Bruggeman
Holger Bruggemann
Hermann Brugger
Rossa Brugha
Daniel Brugman
Laura Brugnara
Radan Bruha

Heike Bruhn
Alejandro Bruhn
Sjoerd Bruijn
Jojanneke Bruins
Karsten Bruins Slot
Sylvia Bruisten
Jonathan Brumberg
Henrik Brumm
Nathan Brummel
Tim Brümmendorf
Stacey Brummer
Paola Brun
Yuriy Brun
Jean-Frederic Brun
Maria Bruna
Anna Maria Brunati
Geir Brunborg
Patrik Brundin
Andreas Brune
Roman Brunecky
Jean Michel Brunel
Cheryl Brunelle
Matteo Brunelli
Maria Brunello
Henri Brunengraber
Emiliano Bruner
Frédéric Brunet
Jean-Luc Brunet
Isabelle Brunet
Enrico Brunetti
Natale Brunetti
Beniamino Brunetti
Celso Brunetti
Nicola Brunetti-Pierri
Maurizia Brunetto
Liam Brunham
Oliviero Bruni
Tommaso Bruni
Sascha Brunke
Shelby Brunke
Robert Brunkhorst
Eric Brunner
Peter Brunner
Frédéric Brunner
Thomas Brunner
Romuald Brunner
David O Brunner
Christian Brunner
Philip Brunner
Ivano Brunner
John Bruno
Stefania Bruno
Rafaela Bruno
Vincent Bruno
Aurelio Bruno
Odemir Bruno
D. Bruno
Ferry Bruno
José Bruno-Bárcena
M. Bruno-Galarraga
André Brunoni
Linda Brunotte
M. Brunotto
Helge Bruns
Mary Ann Bruns
Luc Brunsveld
Bingni Brunton
Attila Brunyanszki
Tad Brunye
Berit Brurok
Silvio Brusaferro
Fabrizio Bruschi
Thomas Brüser
Øystein Bruserud
Lorenzo Brusetti
Corina Brussaard
Timothy Brusseau
Nele Brusselaers
Guy Brusselle
Peter Brust
Eric Brustad
Nickolay Brustovetsky
Andreas Brutemark
Jesper Bruun
Birgitte Bruun
Jo Bruusgaard
Olivier Bruyère
Angela Bruzzaniti
Jeffrey Bryan
Brett Bryan
Lawrence Bryan
Juliet Bryant
Helen Bryant
Litticia Bryant
Josephine M. Bryant
Katarzyna Bryc

Donna Bryce
Mikkel Brydegaarde
Christopher Brydges
Josef Bryja
Michal Brylinski
Mark Brynildsen
Ola Brynildsrud
Marc Brysbaert
Robert Bryson-Richardson
Pawel Brzek
Edyta Brzoska
Tomasz Brzozowski
P. Brzuzan
Wenjun Bu
Wen Bu
Heng-Fu Bu
Rongyan Bu
Fengxiao Bu
Xiangning Bu
Matthew Buas
John Buatti
Lukas Bubendorf
Annalisa Bucchi
Lauro Bucchi
Fabio Bucchieri
Maria Pia Bucci
Vanni Bucci
David Bucci
Monica Bucciarelli
Laura Buccini
Javier Buceta
Thorsten Buch
John Buchanan
Cara Buchanan
Helen Buchanan
Gordon Buchanan
Sergio Bucharles
Marisa Bucheli
Roman Bucher
Sherri Bucher
René Buchet
Emily Buchholtz
Stephanie Buchholtz
Ursula Buchholz
Malte Buchholz
Daniel Buchholz
Bruno Buchholz
Angela Buchholz
Vladimir Buchman
Tobias Buchmann
Denise Buchner
David Buchner
Maciej Buchowski
Eric Buchser
Peter Buchwald
David Buchwalter
Gregory Buck
Andreas Buck
Hudson Buck
James Buck
Dorothea Buck
Julia Buck
Bela Buck
Bill Buck
Laura Buck
Wolfgang Buckel
Jay Buckey
Phillip Buckhaults
Robert Buckheit Iii
Gavin Buckingham
Bethany Buck-Koehntop
Malcolm Buckle
Nicholas Buckley
Hannah Buckley
Katherine Buckley
Michael Buckley
Laura Buckley
Stephen Buckley
David Buckley
Hallie Buckley
Thomas Buckley
Charlotte Buckley
Jenni Buckley
Ann Bucklin
David Bucklin
Jennifer Buckman
Gerhard Buck-Sorlin
Matthew Buckthorpe
Grzegorz Buczkowski
Sandy Buczynski
Sergey Budaev
Hikmet Budak
Ann Budd
William Budd
Matthew Budde
Katharina Budde

Bryce Buddle
K. Buder
Sagar Budhe
Pooja Budhiraja
Anuradha Budhu
Henna Budhwani
G. R. Scott Budinger
Mauricio Budini
Sarah Budischak
Hector Budman
Bruce Budowle
Marilena Budroni
Steven Budsberg
Luke Budworth
Mikalai Budzevich
Berno Buechel
Christa Buechler
Evan Buechley
Andreas Buechner
Else Buenemann
Susan Bueno
Valquiria Bueno
Primitiva Bueno
Carolina Bueno
Juan Bueno
Alfonso Bueno-Orovio
Jan Buer
Javier Buesa
Lars Buesing
Garry Buettner
Christoph Buettner
Falk Buettner
Florian Buettner
Ilaria Bufalari
Matthew Buffington
Annalisa Buffo
Barbara Buffoli
Mariano Buffone
Ingo Bugert
Timothy Bugg
Marcus Buggert
Leandro Bugoni
Beáta Bugyi
Jerome Buhl
Michael Buhnerkempe
Christoph Bührer
Elisabeth Bui
Ngoc Bui
Tuan Bui
J. M. Buick
Peter Buijnsters
Ruud Buijs
Tom Buijse
Brian Buijsse
Jane Buikstra
Lorraine Buis
Yves Buisson
Kinga Bujakowska
Emmanuel Bujold
Zdenek Buk
Feliksas Bukauskas
Alexander Bukreyev
Michael Bukrinsky
Cristina Bulai Livideanu
Alexandr Bulatov
Joseph Bulbulia
Mariana Bulgarella
Marie Bulgin
Michael Bull
Carolee Bull
Caroline Bull
Joan Bull
Jan Bulla
Kai Bullard
Fabio Bulleri
Mark Bullimore
Monika Bullinger
Eric Bullinger
Alex Bullock
Timothy Bullock
Ben Bullock
Donald Bullock
Malcolm R. Bullock
Bronson Bullock
Pedro Bullón
Dennis Bulman
Judith Bulmer
Mariano Bulos
Leif Bulow
Nico Buls
Erwin Bulte
Isabelle Bülthoff
Geert Bultynck
Tomislav Bulum
Maria Bulzacchelli
Brian Buma

Namandje Bumpus
Timothy Bunchman
Madeleine Bunders
Brad Bundy
Robert Bunet
Silvia Bunge
Jorg Bungert
Melanie Bungert
Takashi Bungo
Barry Bunin
Boyke Bunk
Richard Bunker
Nikolay F. Bunkin
Andy Bunn
Ismael Buño
Erin Bunting
Fred Bunz
Jean-Claude Bunzli
Luigi Buonaguro
Pasqualina Buono
Mónica Buono
Camila Buono
Danilo Buonsenso
Nathalie Buonviso
Luiz Augusto Buoro Perandini
Xabier Buque
Syamala Buragadda
Bradley Buran
Alexander Burashnikov
Samuele Burastero
Peter Burbelo
Rémy Burcelin
Kathleen Burchhardt
Scott Burchiel
Irene Burckhardt
Martin Burd
Nicholas Burd
Adrian Burden
Sarah Burdett
Christopher Burdett
Jonathan Burdette
David Burdige
Marie Burdine
Paula Burdisso
Tricia Burdo
Kathryn Burdon
Catriona Burdon
Michal Burdukiewicz
Dominique Bureau
Esther Buregyeya
Theresa Burg
Matthew Burg
Gaetan Burgaud
Jeffrey Burgdorf
Russel Burge
Pierre-Régis Burgel
Danielle Burger
David Burger
Alan Burger
Helena Burger
Huibert Burger
Christoph Burger
Martin Burger
Benjamin Burger
Thomas Burger
Paul T. P. W. Burgers
Shawn Burgess
Sean Burgess
Helen Burgess
Janette Burgess
Harold Burgess
Kevin Burgess
Scott Burgess
Treena Burgess
Brandy Burgess
Angela Burgess
Neil Burgess
Nesha Burghardt
Gaetan Burgio
Patrick Burgon
Jose Burgos
Louise Burgoyne
Gerald Burgstaller
Katalin Burián
Mark Burkard
Robert Burkard
Volker Burkart
Judith Burkart
Robert Burke
Allan Burke
Mark Burke
Adam Burke
Marshall Burke
Ian Burke
Robert E. Burke
Suzanne Burke

Deron Burkepile
Nathan Burkett-Cadena
Matthew Burkey
Janis Burkhardt
Ralph Burkhardt
Pawel Burkhardt
Brant Burkhardt
Juergen Burkhardt
Timothy Burkhart
Dean Burkin
Mark Burkitt
Frederick Burkle, Jr.
Linda Burkly
Andre Burkovski
Scott Burks
Deborah Burks
Christopher Burlak
David Burley
Sandeep Burma
Pradeep Kumar Burma
Oliver Burman
Thorsten Burmester
Robert Burnap
Owen Burnell
Arthur Burnett
Kathryn Burnett
Karen Burnett
Michel Burnier
M. Burnley
Anne-Françoise Burnol
Sylvie Burnouf
Jane Burns
Mark Burns
Richard Burns
James Burns
Timothy Burns
Fredric J. Burns
Siobhan Burns
Cara Burns
S. Burns
Ted M Burns
Jonathan Burns
Michael Burns
Lucinda Burns
Christopher Burns
Ryan Burns
Meike Burow
David Burr
Kevin Burrage
Pamela Burrage
James Burrell
Brian Burrell
Reto Burri
Raghunadha Burri
Keith Burridge
Paul Burridge
Christopher Burridge
Robert Burriss
Kelly Burrowes
Mike Burrows
Neil Burrows
Hugh Burrows
Jirí Burša
Christina Bursill
Sumner Burstein
Timo Burster
Trevor Burt
Janis Burt
Sara Burt
John Burt
Bryan Burt
Jenny Burt
Gordon Burtch
Emilie Burte
Stéphane Burtey
Mary Burtnick
Ron Burton
A. Burton
Jeremy Burton
Britt Burton-Freeman
Doris Burtscher
Martin Burtscher, Dmed
Ingunn Burud
Benjamin Burwitz
Kamil Bury
Oleksandr Burylko
Sakib Burza
Michael Busa
George Busby
Michele Busby
Ben Busby
Post Busby
Louis Buscail
Michael Busch
Jonah Busch
Hauke Busch

Theresa Busch
Andrew Busch
Wolfgang Busch
Hans Jörg Busch
Robert Busching
Anne Buschmann
Alejandro Buschmann
Andreas Buser
Thomas Buser
Thomas Busey
Ronald Bush
Kathryn Bush
William Bush
Stephen Bush
Khalaf Bushara
Martin Bushell
Andrew Bushell
Mary Bushman
Wade Bushman
John Bushweller
Julia Busik
Doreen Busingye
Edward Buskey
Leo Buss
Karen Bussard
Jan Busschbach
Dietrich Busselberg
Harmen Bussemaker
Giovanni Bussi
Rainer Bussmann
Carlos A. Busso
Juan Busso
Gianni Bussolati
Ovidio Bussolati
Federico Bussolino
Cyrill Bussy
Juan Bustamante
M. A. Bustamante
Eduardo Bustamante
Michael Bustin
Lill-Tove Busund
Anna But
Sachit Butail
Matej Butala
Azeez Butali
Patrick Butaye
Jonathan Butchar
Stuart Butchart
Matthew Butchbach
Geoff Butcher
Jonathan Butcher
Joshua Butcher
Saulius Butenas
Melinka Butenko
Robert Butera
Luigi Butera
Donald Buth
Samantha Butler
Peter J. Butler
John Butler
J. Butler
Mark Butler
Matthew Butler
Noah Butler
Mary Butler
Janet Butler
Robert Butler
Gillian Butler-Browne
Clare Butler-Ellis
Mark Butlin
John Butman
Cécile Butor
Marina Butovskaya
Javier Butragueño
Ghazwan Butrous
M. Butt
Craig Butt
Gabriele Buttafuoco
Frances Buttelmann
Colin Butter
Thomas Butterfield
Isabella Buttino
Mathias Buttmann
Chris Button
Mirko Buttrini
Carter Butts
Ian Butts
Kim Butts Pauly
Vincent Butty
Ralph Buttyan
Alexander Butwick
Nicholas Butzin
Malte Butzlaff
Anne Buve
Laura Buyan-Dent
Fulden Buyukozturk

Ozlem Buyuktanir
G. Buzas
Bruno Buzatto
Walter Buzina
Oleksiy Buznyk
Stefan Buzoianu
Dominique Buzoni
Denis Bwesigye
Peter Byass
Seth Bybee
Stine Byberg
Winston Byblow
Sergio Bydlowski
Christopher Bye
Bruce Byers
Lauren Byers
John Byers
Derek Byers
Kelsey Byers
William Bynum
Siddappa Byrareddy
Thomas Byrd
R. Andrew Byrd
Nick Byrd
Barbie Byrd
Todd Byrem
Gerald Byrne
Scott Byrne
Richard Byrne
Maria Byrne
Jason Byrne
Kimberly Byrnes
Kenneth Byron
Caughey Byron
Craig Byron
Adam Byron
Chris Bystroff
Yong-Soo Byun
Tatiana Byzova
Jorge Caamano
Mario Caba
Teresa Cabaleiro
Cesar Caballero
Susana Caballero
Santiago Omar Caballero Morales
Juan Caballero-Pérez
Nicolas Cabaton
Hugo Cabedo
María Pilar Cabezas
Oscar Cabezón
Manuela Cabiati
Andrea M Cabibbe
Marcelo Cabido
Deborah Cabin
Sara Cabodi
Ryan Cabot
Florence Cabot
Horacio Cabral
Maria Clara Cabral
Luis Cabral
Patricia Cabral
Lucélia Cabral
Antonio Cabrales
Adoracion Cabrera
Delia Cabrera Debuc
Hector Cabrera-Fuentes
Luca Cabrini
Nadia Caccamo
Laura Cacciani
Barbara Cacciari
Francesco Cacciatore
Fabio Caccioli
Alfredo Caceres
Omar Cáceres
Edward Cachay
Fidel Cacheda
Arnaud Cachia
Begona Cachon-Gonzalez
Anne Caclin
Patrice Cacoub
Dan Cacsire Castillo-Tong
Gemma Cadby
Enrique Cadenas
Giovanna Caderni
Jean Lud Cadet
Alessandra Cadete Martini
Noel Cadigan
Hervé Cadiou
Eduardo Cadore
Jean Charles Cadoret
Ruggero Cadossi
David Cadotte
Monique Cadrin
Renee Cadzow
Catia Caeiro
Daniel Caetano

Derek Caetano-Anolles
Kelsey Caetano-Anolles
Joana Caetano-Lopes
Laurence Caeymaex
Donata Cafasso
Maria Caffarel
Carla Caffarelli
William Cafferty
Alida Caforio
Jonathan Cagan
Maria Cagetti
Humeyra Caglayan
Alexandro Cagliari
Angelo Cagnacci
Luciano Cagnolo
Patrick Cahan
Alex Cahana
Katelyn Cahill-Rowley
Wiepke Cahn
Elizabeth Cahoon
Edgar Cahoon
Monika Cahova
Chantal Cahu
James Cai
Jingli Cai
Yu Cai
Li Cai
Yu-Dong Cai
Zongwei Cai
Lu Cai
Lifeng Cai
Jianming Cai
Jiye Cai
Weidong Cai
Liming Cai
Hong Cai
Daguang Cai
Weibo Cai
Xin Cai
Hui Cai
Xuehui Cai
Dongming Cai
Wanzhi Cai
Yiyong Cai
Yuan-Jun Cai
Jianfeng Cai
Fei Cai
Yong Cai
Baiyan Cai
Shanshan Cai
Hongmei Cai
Ruichu Cai
Jianfang Cai
Zhengxin Cai
Wenfeng Cai
Shi-Min Cai
Hongwei Cai
Shengguan Cai
Ling Cai
Yan Cai
Jian-Chun Cai
George Cai
Liang Cai
Lijun Cai
Yuexin Cai
Zhao-Hua Cai
Zhiqiang Cai
Dachuan Cai
Xianfeng Cai
Jing Cai
Hongning Cai
Benzhi Cai
Guorong Cai
Sanjun Cai
Qian Cai
Xiong Cai
L. M. Cai
Paola Caiafa
Anne Caignard
Pascal Caillet
Fabien Cailliez
Jocelyne Caillon
Melissa Caimano
Kevin Cain
Kelvin Cain
Stephen Cairns
Matthew Cairns
John Cairns
David Cairns
Rob Cairns
Dana Cairns
Cristiana Cairo
Pietro Caironi
Miren Cajaraville
Ahmet Çakir
Bulent Cakmak

Dimitrios Cakouros
De Cal
Steven Cala
Francesco Calabrese
Vittorio Calabrese
Evan Calabrese
Aurelie Calabrese
Laura Calabresi
Rodrigo Calado
Riccardo Calafiore
Matthew Calamia
Elana Calandre
Sara Calatayud
Karin Calaza
Federico Calboli
Andrea Calcagno
Luis Calcaterra
Rebecca Calcott
Mick Calcutt
Nigel Calcutt
Camila Caldana
Roberto Caldara
Guido Caldarelli
Joana Caldeira
Isabelle Caldelari
Bobby Calder
Simone Calderano
Tina Calderon
Roger Calderon
Rosario Calderón
Lilian Calderon -Garciduenas
Luz Irina Calderon Villalobos
Richard Calderone
Amaia Calderón-Larrañaga
Eva Calderon-Sanchez
Audrey Calderwood
Chris Caldow
Charles Caldwell
Janalee Caldwell
Stephen Caldwell
Gary Caldwell
Brent Caldwell
Michael Caldwell
Catherine Caldwell-Harris
Richard Calendar
Miguel Calero
C Calero
Peter Caley
Rodrigo Calheiros
William Calhoun
Pietro Caliandro
Rudy Calif
Andrea Califano
Sabrina Calil-Elias
George Calin
Anda-Alexandra Calinescu
Mariafe Calingacion
Jan Calissendorff
Kelly Calisto Lima
Paolo Calistri
Cornelis Calkhoven
David Calkins
Amanda Callaghan
Damien Callahan
Ann M. Callahan
Ewa Callahan
Daniel Callan
Mitch Callan
Akiko Callan
Myrasol Callaway
Clifton Callaway
Leonardo Calle
Patrick Callery
Henrik Callesen
Geert Callewaert
Joseph Callicott
Viviane Callier
Myriam Callier
Judy Callis
Virginie Callot
Bernard Callus
Lido Calorini
Enrica Calura
Mario Calus
Edmundo Calva
Graciano Calva Calva
Vincenzo Calvanese
Vincenza Calvaruso
Aleix Calveras
P. Calverley
Matt Calverley
Peter Calvert
John Calvert
D. Calvet
Laura Calvet Mir
Juan Calvete

Vincent Calvez
Sébastien Calvignac-Spencer
Dustin Calvillo
Katherine Calvin
Maria Calviño-Cancela
Cinzia Calvio
Diego Calvisi
Eric Calvo
Cristina Calvo
Estefanía Calvo Álvarez
Javier Calvo Gariido
Alejandra Calvo-Diaz
Beatriz Calvo-Merino
Monica Calvo-Polanco
Laura Calza
Carlos Eduardo Calzavara-Silva
Enrico Calzia
Laurence Calzone
Hakan Cam
Vitaliano Cama
Ana Camacho
Juan Pedro Camacho
Morelia Camacho
Andres Camacho-Gonzalez
Jean-Michel Camadro
Simonetta Camandola
Amadou Camara
Estela Camara
Niels Câmara
Alessandra Camarca
Grazia Camarda
Valentina Camarda
Roman Camarda
Constanza Camargo
Raphael Camargo
Luiz Sérgio Camargo
Joíza Camargo
Arely Camargo
Maria Izabel Camargo-Mathias
Miller Camargo-Valero
Emmanuelle Cambau
Lorne Cambell
Alessandra Cambi
Valentina Cambiano
Marie Anne Cambon-Bonavita
Gilles Cambonie
Oscar Cambra-Moo
Geraldine Cambridge
Eric Cambronne
Serge Camelo
Emanuela Camera
Marina Camera
Donny Camera
Anne-Linda Camerini
Heather Cameron
Caroline Cameron
Elissa Cameron
Adrian Cameron
D. Cameron
David Cameron
Stephen Cameron
Robin Cameron
Chris Cameron
Jennifer Cameron
Paul Cameron
Delroy Cameron
Scott Cameron
Daniel Cameron
Trudy Cameron
Peter Cameron
Tim Cameron
Mary Cameron
Nicole Cameron
Géraldine Camilleri
Cyril Caminade
Jorge Caminos
Marta Camino-Serrano
Antoni Camins
Irina Caminschi
Rodney Camire
Calogero Cammà
Giancarlo Cammarota
Laura Camoni
Blanca Camoretti-Mercado
Joanne Camp
Gabriella Campadelli-Fiume
Davide Campagna
Giuseppe Campagna
Leo Campagna
Pascal Campagne
Aurélie Campagne
C. Sylvie Campagne
Paola Campagnolo
Joseph Campanale
Juan Campanario
Lee Ann Campbell

Kenneth Campbell
Douglas Campbell
Karen Campbell
Peter Campbell
Lesley Campbell
Corey Campbell
Thomas Campbell
Daniel Campbell
Jos Campbell
Grant Campbell
Patricia Campbell
B. Todd Campbell
Jacquelyn Campbell
W Campbell
Duncan Campbell
Alexandra Campbell
Arezoo Campbell
B. Campbell
Colin Campbell
Archie Campbell
Polly Campbell
Robert Campbell
Stuart Campbell
Matthew Campbell
Ian Campbell
John Campbell
Moray Campbell
Scott Campbell
Marilyn Campbell
Hamish Campbell
Lisa Campbell
P. Campbell
Emily Campbell
Angus Campbell
Kirk Campbell
Jay Campbell
Ruth Campbell
Lindsay Campbell
Ashley Campbell
Ellsworth Campbell
Paul Campbell
Martha Campbell-Thompson
Esther Camp-Dotlic
Matthew Campen
Umberto Campia
Natalia Campillo
Lena Campino
Elena Campione
Andrea Campisano
Patrizio Campisi
Orlando Campolo
Rodolfo Campos
Maria Campos
Paula Campos
Erika Campos
Angélica Cristine Campos
Marcia Campos
Joaquin Campos
Raquel Campos-Herrera
Ramón Campos-Olivas
Giulia Campostrini
Cristina Campoy
Carme Camps
Julia Camps
Ulas Camsari
Seyit Camtepe
Lamberto Camurri
Anne Camus
Frank Can Meurs
Stéphane Canaan
Askery Alexandre Canabarro
Ana Canadas
David Canaday
Maria Canal
Angelo Canale
Donaldo Canales
Erick Canales-Rodríguez
Ernesto Canalis
Thomas Canam
Paula Canas
Guillaume Canaud
Maureen Canavan
Ali Canbay
Sule Canberk
Carlos Cançado
André Cançado
Giovanni Cancarini
Ranieri Cancedda
Juan Cancino-Diaz
Leopoldo Cancio
Eva Candal
Demet Candas
Kate Candelario
E. Candi
Simona Candiani
Giovanni Candiano

Matteo Candidi
Olivia Candini
Ermanno Candolfi
Marianela Candolfi
Sophie Candon
Guseppina Candore
John Candy
Rossella Canese
Laura Canesi
Nicola Canessa
Daniela Canestrari
Pietro Canetta
Claudio Canetti
Giulia Caneva
Loredana Canfora
Jianhua Cang
Angelo Cangelosi
Maria Giulia Cangi
Alberto Cangiano
Manuela Canica
Carolina Caniffi
Isabella Caniggia
Laetitia Canini
Michel Canis
Samuel Canizales-Quinteros
Mayilee Canizares
Barbara Canlon
David Cannatella
Margherita Cannillo
Brendan J Canning
Carlovittorio Cannistraci
Carlo Cannistraci
Renato Cannizzaro
Barbara Cannon
Paula Cannon
Mary Cannon
Steven Cannon
Richard Cannon
Judy Cannon
Charles Cannon
John Cannon
Kristine Cannon
Robert Cannon
Phil Cannon
David Cano
Carlos Cano
Liliana Cano
François Canonne-Hergaux
Francisco Canovas
David Canovas
Manuel Canovas
Dexter Canoy
Ugur Canpolat
Vincenzo Cantaluppi
Francesco Cantatore
Roberto Cantello
Edouard Cantin
Fabrizio Cantini
Francesco Cantini
Massimiliano Cantinotti
Carlos Canto
Efrain Canto-Lugo
Gador Canton
Yolanda Cantón
Scott Cantor
Margherita Cantorna
Charles Cantrell
Dario Cantu
Lisette Cantú-Salazar
Marie Cantwell
John Canty, Jr.
Luigi Canullo
Rosa Canuto
Vincenzo Canzonieri
Jian-Ping Cao
Liang Cao
Jay Cao
Jia Cao
Junli Cao
Huijuan Cao
Yilin Cao
Yiping Cao
Xu Cao
Zhiwei Cao
Ruifeng Cao
Qi Cao
Ji-Min Cao
Qingjiu Cao
Siyan (Stewart) Cao
Xiao-Lin Cao
Quang V. Cao
Weibiao Cao
Bo Cao
Lin Cao
Dianjun Cao
Sha Cao

Lei Cao
Guangchun Cao
Yue Cao
Yin Cao
Jianping Cao
Hongnan Cao
Jin Cao
Muqing Cao
Yanguang Cao
Chengqi Cao
Zubing Cao
Guangzhong Cao
Tie-Sheng Cao
Jian Cao
Huansheng Cao
Wanjun Cao
Yan Cao
Ruibing Cao
Shuqing Cao
Hongbo Cao
Wei Cao
Jin-Lin Cao
Qian Cao
Bin Cao
Xinping Cao
Miao Cao
Hung Cao
Moju Cao
Xiaohua Cao
Jinshan Cao
Qing Cao
Tian Cao
Xiaoyan Cao
Qiufen Cao
Yanping Cao
Shujuan Cao
Linlin Cao
Fangbin Cao
Feilin Cao
Salvatore Caorsi
Andrew Cap
Marinela Capanu
Serge Caparos
Mario Capasso
Clemente Capasso
Neus Capdeferro
Delphine Capela
Manuel Luis Capelas
Marzia Capelletti
Cristian Capelli
Giovanni Capelli
Isabella Capellini
Patrice Capers
Ana Caperta
Encarnación Capilla
Cesar Capinha
Catherine Capio
José Capitán
Nazzareno Capitanio
Dario Capizzi
Jeremy Caplan
Michael Caplan
Louis Caplan
Paul Caplat
G. Caplovitz
Virginia Capmourteres
Enrico Capobianco
Marco Capocasa
Maurizio Capogrossi
Doug Capone
Andrea Caporali
Maria Nieves Capote Mainez
Yvan Capowiez
Vittorio Capozzi
Stefano Cappa
Angélica Cappellari
Gianluca Cappelleri
Gianni Cappelli
Germana Cappellini
Maria Cappello
Ezio Cappello
Mike Cappo
Valeria Capra
Piera Capranzano
Valerio Capraro
Giovanni Capretti
Michael Caprio
Daniele Caprioli
Flavio Caprioli
Joseph Caprioli
Annalisa Capuano
Ana Capuano
Claudia Capurro
Gabriele Capurso
Viviana Capurso
Cristina Capusa

Fabio Caputo
Paolo Caputo
Barbara Caputo
Maurizia Capuzzo
Luis Caraballo
Rey Carabeo
Hélène Carabin
Barbara Caracciolo
Alfonso Caracuel
Paul Caradonna
Michel Carael
Anthony Caragiulo
Michele Caraglia
Martin Caraher
Iris Caramalho
Paola Caramaschi
Doretta Caramaschi
Carla Caramella
Paulo Caramelli
Gaetano Caramori
Aaron Carass
Laure Carassou
Fuensanta Caravaca
Fernando Caravaggio
Marion Carayol
Pau Carazo
Rafael Carazo-Salas
Anibal Carbajo
Marta Carballa
Claus-Christian Carbon
Emilio Carbone
David Carbone
Anna Carbone
Salvatore Carbone
Agostino Carbone
Franck Carbonero
Nicholas Carbonetti
Lucia Carboni
Etienne Carbonnelle
Ivan Carcamo-Orive
Enric Carcereny Costa
Francesco Carcò
Daren Card
Roderick Card
Laura Cardador
Ring T. Carde
Michelle Cardel
Francesc Cardellach
Manuel Cardenas
Edwin Cardenas
Cesar Cardenas
Julio Cardenas-Rodriguez
Valentina Cardi
Mario Cardiel
Carmine Cardillo
Alessio Cardillo
Jessica Cardin
Sophie Cardin
Eric Cardinale
Vincenzo Cardinale
Massimiliano Cardinale
Yudith Cardinale
Giorgia Cardinali
Ulisse Cardini
Pere-Joan Cardona
Genis Cardona
Gloria Patricia Cardona-Gomez
Luciana Cardoso
Pedro Cardoso
Tereza Cardoso
Marlon Cardoso
Cynthia Cardoso
Rosilene Fressatti Cardoso
Danon Cardoso
Cr Cardoso
Sandra Cardoso
Juan Andrés Cardoso
Rhanderson Cardoso
Jose Maria Cardoso Da Silva
Rui Cardoso-Pereira
Timothy Cardozo
Christopher Cardozo
Alessandra Carè
Faizal Careem
Thomas Carell
John Carethers
James (Jim) Carey
Thomas Carey
Gregory Carey
Alison Carey
Gerardo Carfagno
Andrea Carfi
Alessia Cariani
Cristina Carias
Anna Cariboni
Alex Carignan

Rita Carini
Paul Carini
Guillaume Carissimo
Dan Cariveau
Carsten Carlberg
Georges Carle
Ryan Carle
Melanie Carless
Jean Carlet
Marco Carletti
Giancarlo Carli
Aurélie Carlier
Johan Carlin
Paul Carlin
Joseph Carlin
Gregory Carling
Celia Carlini
Alessandro Carlini
Matteo Carlino
Jay Carlisle
John Carlisle
Mckenzie Carlisle
Gretchen Carlisle
Waldemar Carlo
Simona Carlomagno
Chiara Carlomagno
Paolo Carloni
Schenck Carlos
Mar Carlos
Carmelo Carlo-Stella
John Carlquist
Annelie Carlsbecker
Anthony Carlsen
Anja Carlsohn
Sandra Carlson
Jordan Carlson
Steve A Carlson
Cathy Carlson
Bradley Carlson
Kyle Carlson
Elwood Carlson
Anders Carlsson
Kathy Carlstead
Peter Carlton
Susan Carlton
James Carlton
Marcos Carlucci
Erberto Carluccio
Jason Carlyon
Chris Carman
Claudia Carmassi
Liran Carmel
Mar Carmena
Thomas Carmichael
Owen Carmichael
Marco Carmignani
Adriana Carmona
Carlos Carmona
Erico Carmona
Carmelo Carmona-Rivera
Monica Carmosino
Heather Carnahan
Nicole Carnegie
Ana Carneiro
Everardo Carneiro
Miguel Carneiro
Bruno Carneiro
Benedito A Carneiro
Regina Carneiro
Susan Carnell
Amancio Carnero
Andrés Carnero
Cynthia Carnes
Gianluca Carnevale
Luca Carnevali
Greg Carney
Paul Carney
Simon Carney
Adam Caro
Valeria Carola
James Carolan
Cristina Carollo
Alexandre Caron
Robert Caron
Rafaela Caron-Lienert
Geoffrey Caron-Lormier
Emanuele Carosati
Erik Caroselli
Ylenia Carotenuto
Francesco Carotenuto
Luciana Carotenuto
Angelo Carotti
Berit Carow
Francesca Carozzi
Armando Carpaneto
Roger Carpenter

Matthew Carpenter
Guy Carpenter
Ryan Carpenter
Lewis Carpenter
Sebastien Carpentier
Arnaud Carpentier
Giovanna Carpi
Guido Carpino
Darren Carpizo
Lindsay Carpp
Antony Carr
Christopher Carr
J. A. Carr
Thomas Carr
Richard Carr
Jillian Carr
Joel Carr
Catherine Carr
Michael Carr
Rotonya Carr
Brittany Carr
Benjamin Carr
Serena Carra
Andrea Carra
Arkaitz Carracedo
Matthew Carrano
Andrés Carrano
Maria Laura Carranza
Stefania Carrara
Francesco Carrara
Nicola Carraro
Raffaele Carraro
Julia Carrasco Valiente
Gonzalo Carrasco-Avino
Concepcion Carratala-Munuera
Pierluigi Carratù
Justin Carre
Clément Carré
Matthieu Carré
Madeleine Carreau
Adriano Carregaro
Valeria Carreira
Maria Angela Carreira
João Carreiras
Margaret Carrel
Douglas Carrell
Marco Carrer
Helaine Carrer
Ana Carrera
Inés Carrera
Enric Carrera-Gallissa
Elena Carreras
Oscar Carretero
Stephanie Carretero
Lorenzo Carretero-Paulet
Luis Carretié
Hunter Carrick
Danielle Carrick
Adam Carrico
João Carriço
Julie Carrier
Erica Carrier
Alice Carrier
Yves Carriere
Maria Vincenza Carriero
Tiago Carrijo
Juli Carrillo
Yolima Carrillo
Catherine Carrillo
Jose Luis Carrillo Estrada
Enrique Carrillo-De-Santa-Pau
Maria Carrillo-Sepulveda
Mary Carrington
Lauren Carrington
José A. Carrión
Jerome Carriot
Juan Carrique-Mas
Gabriela Carriquiry
Mariana Carriquiry
Michael Carrithers
Antonio Carroccio
Martin Carroll
Allan Carroll
Robert Carroll
Marta Carroni
Philip Carrott
Alessandro Carrozzo
Vern Carruthers
Glen Carruthers
Jane Carr-Wilkinson
Rita Carsetti
Richard Carson
James Carson
Dean Carson
William Carson Iv
Wolff Carsten

Maren Carstensen
Chris Carswell
Anna Carta
Mario Carta
Angelino Carta
Paulo Cartaxana
Valentina Cartei
Gerald Carter
K. C. Carter
Adrian Carter
Michael Carter
Stacy Carter
Cedric Carter
William Carter
Tandrea Carter
Evan Carter
David Carter
Lawrence Carter
Anna Carter
A. Brent Carter
Gillian Carter
Morgan Carter
Cathy Carter-Snell
Maria Teresa Carthery-Goulart
Oiliver Cartiaux
Matt Cartmill
Pierre-François Cartron
Paulyn Cartwright
Rufus Cartwright
Jennifer Cartwright
Matthew Caruana
John Carucci
Pedro Caruso
Claudio Caruso
Antonio Caruz
Alfonso Carvajal
Jaime Carvajal
Micaela Carvajal
Daniel Carvajal
Luis Carvajal-Carmona
Cristina Carvalheiro
Karina Carvalho
Luisa Carvalho
Ana Luisa Carvalho
Leonardo Carvalho
Sandra Carvalho
Agostinho Carvalho
Daniel Carvalho
Claudia Carvalho
Andre Carvalho
Jorge Carvalho
Susana Carvalho
Natalie Carvalho
Monica Carvalho
Eugenia Carvalho
Joana Carvalho
Livia Carvalho
Jose Carlos Carvalho
Brendan Carvalho
Matheus Carvalho De Carvalho
Alessandra Carvalho Goulart
Roberto Carvalho-Filho
Michael Carvan Iii
Wayne Carver
Scott Carver
Leslie Carver
Anne-Ruxandra Carvunis
Josie Carwardine
Jeffrey Cary
Fiona Caryl
Anna Casabianca
Elena Casacuberta
Josep Casadesús
David Casadevall
Rita Casadio
Nicola Casagli
Daniela Casagrande
Paolo Casali
Enrique Casalino
Ricardo Casalino
Nuria Casals
Amelia Casamassimi
Christian Casanova
Jim Casanova
Bonaventura Casanova
Daniel Casanova
Rubén Casanova-Sáez
Fernando Casares
Jose Casaretto
Marco Casari
Dulce Casarini
Ricardo Casaroli-Marano
Simona Casarosa
Nicoletta Casartelli
François Casas
Eduardo Casas

Irene Casas
Laura Casas Castano
Oscar Gabriel Casas Monroy
Sergio Casas-Flores
Paula Casati
Joseph Casazza
Krista Casazza
Rita Cascão
Raffaella Cascella
Dan Casciano
Carissa Cascio
Alessandro Cascioferro
Ingolf Cascorbi
Elizabeth Case
Laura Case
Jenn Caselle
Chiara Caselli
Christina Caselli
Martina Casenghi
Robert Casero
Rodolfo Casero
Paolo Caserotti
Ilaria Casetta
Joseph Casey
Keith Cash
David Cash
Lucinda Cash- Gibson
Amanda Cashen
Anil Cashikar
Neil Cashman
Cara Cashon
Antonino Casile
Araní Casillas-Ramirez
Georges Casimir
Sandra Casimiro
Federica Casiraghi
Michael D. Casler
Elizabeth Casman
Antonella Casola
Vincent Casoli
Laura Casorzo
Raffaella Casotti
Thomas Caspari
Tamara Caspary
Corey Casper
Charlotte Casper
Brandon Casper
Luis Caspeta
Avshalom Caspi
Jonathan Caspi
Geovanni Cassali
Fabricio Cassan
Paulo Cassano
Sandra Cassard
James Cassat
Flemming Cassee
Michele Cassetta
Pamela Cassiday
Joseph Cassidy
Rachel Cassidy
Deby Cassill
Jean-Jacques Cassiman
Renata Cristina Cassimiro De Lemos
Patricia Cassina
Noriko Cassman
Antonio Carlos Cassola
Juliana Cassoli
Robert Casson
Vincent Cassone
Paola Cassoni
Brooke Casssell
Antonella Castagna
Vincent Castagné
Stefania Castagnetti
Bastien Castagneyrol
Nicoletta Castagnino
Jacqueline Castagno
Massimo Castagnola
Elio Castagnola
Clotilde Castaldo
Benoit Castandet
Cristina Castañé
Tamara Castaneda
Homero Castaneda-Lopez
Carlos Castañeda-Orjuela
Susette Castañeda-Rico
Raúl Castanera
Mariana Castanheira
Andréa Castanho
Justo P. Castaño
Lina Castano-Duque
Daniela Castanotto
Helene Castel
Guillaume Castel
Alan Castel
Mickael Castelain

Rene Castelein
Monica Castelhano
Linda Castell
Stefanie Castell
Margarida Castell
Alberto Castella
Isabella Castellano
Leandro Castellano
Giuseppe Castellano
Joseph Castellano
Milagros Castellanos
Cindy J. Castelle
Luigi Castelli
Lorys Castelli
Cesare Castellini
Mauro Castello
Lana Castellucci
Marcela Castelo
Frederico Castelo Branco
Miguel Castelo-Branco
Patricia Castelucci
Umberto Castiello
Riccardo Castiglia
Stefano Castiglione
Isabella Castiglioni
Alessandra Castiglioni
Rogerio Castilho
Rita Castilho
Aurea Castilho
Jessica Castilho
Paula Castilho
Lucio Castilla
Pablo Castillo
Ana-Maria Castillo
Jesús Castillo
Jorge Castillo
Andrea Castillo
Miguel Castillo
Santiago Castillo
Andres Castillo
Ricardo Castillo-Galvan
Javier Castillo-Olivares
Alan Castle
Erica Casto
Martin Castonguay
Yves Castonguay
Frank Castora
Armando Castorena
Carlos Castorena
Gabriella Castoria
Maija Castren
Javier Castresana
Carmen Castresana
Daniele Castro
Patricia Castro
Newton Castro
Andrea Castro
Marcia Castro
L. Filipe Castro
Patrícia Castro
Jason Castro
Nadia Castro
Jose Castro
Jorge Castro
Rui Castro
Sílvia Castro
Rosario Castro
Marta Castro
Laura Castro De La Guardia
Antonio Jesus Castro Lopez
Eduardo Castro Nallar
Maria Dolores Castro Rojas
David Castro-Diaz
Javier Castro-Hernandez
Alejandro Castro-Luna
Susana Castro-Obregón
Hayo Castrop
Itandehui Castro-Quezada
Javier Castro-Rosas
Enrique Castro-Sánchez
Manuela Casula
Patrick Caswell
Clayton Caswell
Mathieu Catala
Raquel Català
Mariana Catalano
Mauro Cataldi
Annalisa Cataldi
Alessandro Cataliotti
Daniele Catalucci
Daniele Cataneo
Marcos Catanho
Maria Vincenza Catania
Viviana Catania
Antonino Catanzaro
Vittoria Catara

Raquel Catarino
Brian Catchpole
Ken Catchpole
Andrés Catena
Cristiana Catena
Daniel Catenacci
Alessandro Catenazzi
Chris Cates
Dominique Cathelin
Claire Catherall
Arnaud Catherine
Zulma Catherine
Dodds Catherine
Emilie Catherinot
Gieri Cathomas
Mark Catley
Caroline Catmur
Douglas Cato
Paul Caton
Anca Irinel Catrina
Sergiu-Bogdan Catrina
Teresa Catry
Boudewijn Catry
Isabella Cattadori
Antonino Cattaneo
Zaira Cattaneo
Cristina Cattaneo
Riccardo Cattaneo-Vietti
Kirsten Catthoor
Vincent Cattoir
Vibeke Catts
Silvia Catuogno
Andrea Cau
Corrado Caudek
William Caudle
Page Caufield
Byron Caughey
Gillian Caughey
George Caughey
Melissa Caughey
Adrian Caulfield
Carlos Caulin
James Cauraugh
Paola Causin
Lisa Cavacini
Afonso Cavaco
Artur Cavaco-Paulo
Andrea Cavagna
Vincent Cavaillès
Vincent Caval
Viviana Cavalca
Leny Cavalcante
Jeferson Cavalcante
Gisele Cavalcanti
Elisabetta Ada Cavalcanti-Adam
Jean François Cavalier
Francesca Cavalieri
Sebastiano Cavallaro
Rodrigo Cavallazzi
Andrea Cavallo
David Cavanagh
Pauline Cavanagh
Jane Cavanaugh
Kyle Cavanaugh
Andrea Cavanna
Mariangela Cavarelli
Alberto Cavazza
Andrea Cavazzoni
Matthew Cave
Matthew Cavender
José Cavero
Stephen Cavers
Joseph Caverzasio
Itzue Caviedes Solis
Enrique Caviedes-Vidal
Sonia Cavigelli
Lionel Cavin
Regiane Cavinato
Kara Cavuoto
Kaelin Cawley
William Cawthorn
María Cayuela
Maria Cayuela
Jean-Michel Cayuela
Hugo Cayuela
Remy Cazabet
Sophie Cazanave
Maxime Cazorla
Marta Cazzaniga
Valentina Cazzato
Dario Cazzato
Ben Cazzolato
Michele Cea
Ana Ceballos
Natalie Ceballos
Claudia Ceballos

Gerardo Cebrian
Colleen Cebulla
Mauro Ceccanti
Gregorio Ceccantini
Simona Ceccarelli
Michele Ceccarelli
Francesca Ceccherini-Silberstein
Michele Cecchini
Maurizio Cecconi
Sandra Cecconi
Gulsah Cecener
Jacopo Cecere
Nadja Cech
Michelangelo Ceci
Adriana Ceci
Charlotte Cecil
Fabrizio Ceciliani
Jens Ceder
Tomas Cedhagen
Susana Cedres
Maciej Cedzynski
Jose Cegoñino
Filipe Ceia
Ana Ceia-Hasse
Cornelia Ceianu
Mirnova Ceide
Jitka Cejková
Francisco Cejudo
Maria Grazia Celani
Antonio Celani
Giuseppe Celano
Emanuele Celauro
Nora Celebi
Peter Celec
Jose Celedon
Benedetto Celesia
Angela Celetti
Alessandro Celi
Leo Celi
Smith Celia
Ayla Çelik
Beamer Celine
Marina Cella
Laura Cella
Eleonora Cella
Jean Celli
Jonathan Celli
Gilles Cellier
Sonia Cellot
Connie Celum
Maja Cemazar
Osman Cen
Hellas Cena
Angela Cenci
Massimo Cenciarini
Chiara Cencioni
Fernando Cendes
Maysa S Cendoroglo
Mari Carmen Cenit
Edina Cenko
Nunzio Cennamo
Federica Censi
Tracy Centanni
Luna Centifanti
Valeria Cento
Georgina Cepeda
Chloe Cera
Jocelyn Céraline
Carla Cerami
Antonio Cerasa
Maria Ceravolo
Leonardo Ceravolo
Nuno Cerca
Salvatore Cerchio
Claudio Cerchione
Jorge Cerda
Alvaro Cerda
Joan Cerdà
Sebastian Cerdan
Pablo Cerdán
José Miguel Cerdá-Reverter
Lage Cerenius
Brian Ceresa
Richard Cerione
Aldo Ceriotti
Chiara Cerletti
Caterina Cerminara
Ibolja Cernak
Tomislav Cernava
Jasmina Ziva Cerne
Julian Ceron
José Cerón
Francesca Ceroni
Nuno Cerquiera
Maria Cerra
Jose Cerrato

Matteo Cerri
Ronaldo Cerri
Milan Certík
Pierpaolo Cerullo
Stefania Ceruti
Bernard Cerutti
Miguel Cervantes
Jorge Cervantes
Luisa Cervantes-Barragan
Tiziana Cervelli
Carlos Cervera
Ronald M. Cervero
Laura Cervi
Renate Cervinka
Aline Cesar
Roberto César Pereira Lima
Paolo Cesare
Mario Cesaretti
Paola Cesari
Annaelle Cesaro
Corrado Cescon
Paola Cescutti
Daniela Cesselli
Tomris Cesuroglu
Mine Cetinkaya-Rundel
Miguel Angel Cevallos
Lucia Cevidanes
Ratnasekhar Ch
Chen Ch
Wonjae Cha
Dong Seok Cha
Hala Chaaban
Sarah Chabal
Anne-Lise Chaber
Béatrice Chabi
Bruce Chabner
Agnieszka Chacinska
Bryce Chackerian
George Chacko
George Chaconas
Carlos Chacón-Díaz
Marucia Chacur
Heramb Chadchankar
Kris Chadee
V. Chadha
Pooja Chadha
Chadwick Chadwick
Hiun Suk Chae
Benjamin Chaffee
Marcos Chagas
Alexandre Chagnes
Takemore Chagomoka
Monica Chagoyen
Nadeen Chahine
Javaan Chahl
Yang Chai
Li Chai
Toby Chai
Shua Chai
Yimin Chai
Siew Chai
Renjie Chai
Sheau Ching Chai
Tuan-Yao Chai
Zhenhua Chai
Jianfang Chai
Liang Chai
Guohong Chai
Chenglin Chai
Elias Chaibub Neto
Kaisorn Chaichana
Thanapong Chaichana
J. Richard Chaillet
Anaïs Chailleux
Sarawuth Chaimool
Benny Chain
Patrick Chain
Lelia Chaisson
Mark Chaisson
Alan Chait
Parin Chaivisuthangkura
Sage Chaiyapechara
Donald Chakeres
Shigeyuki Chaki
Murali Chakinala
Joe Chakkalakal
Ela Chakkarapani
Rafael Chakr
Subrata Chakrabarti
Debopam Chakrabarti
Jayprokas Chakrabarti
Subhabrata Chakrabarti
Ratna Chakrabarti
Abhijit Chakrabarti
Rahul Chakrabarti
Prosanta Chakrabarty

Paramita Chakrabarty
Debasis Chakrabarty
Anirban Chakraborti
Trinad Chakraborty
Nitya Chakraborty
Debapriyo Chakraborty
Subhankar Chakraborty
Saikat Chakraborty
Subrata Chakraborty
Debojyoti Chakraborty
P Chakraborty
Syandan Chakraborty
Avishek Chakraborty
Sourav Chakraborty
Damayanti Chakraborty
Venkatesan Chakrapani
Srinandan Chakravarthy
Balu Chakravarthy
M. Chakravarty
Runu Chakravarty
Dipshikha Chakravortty
Soumitesh Chakravorty
Dhruva Chakravorty
Paulina Chalan
Sreekanth Chalasani
Joe Chalfoun
Tigran Chalikian
Alistair Chalk
Victoria Chalker
Suresh Challa
Stephen Challacombe
Etienne Challet
Damien Challet
Gaelle Challet
John Challis
John Challiss
Jeffrey Chalmers
James Chalmers
Peter Chalmers
Jenny Chalmers
Sylvie Chalon
Helena Chaloupková
Laura Chalup
Krzysztof Chalupka
Candace Cham
Srikar Chamala
Jamshidkhan Chamani
Mahmood Chamankhah
Andres Chamarro
Kerry Chamberlain
Connie Chamberlain
Joel Chamberlain
Philip Chamberlain
Brent Chamberlain
Andrew Chamberlain
James Chamberlain
Maxime Chamberland
Christina D Chambers
Benedict Chambers
Stephen Chambers
James Chambers
Jeremy Chambers
Valerian Chambon
Venkateswarlu Chamcha
Jean Christopher Chamcheu
Gabriel Chamie
Supat Chamnanchanunt
Jose Manuel Chamorro
Eric Chamot
Danny Chamovitz
Colin Champ
Donald Champagne
Cory Champagne
Emilie Champagne
Julie Champion
Patricia Champion
Matthew Champion
Hassan Chamsi-Pasha
Steven Chamuleau
Robert Chamuleau
Paul Chan
Nei-Li Chan
Danny Chan
Allen Chan
Christina Chan
Wood Yee Chan
Anthony Chan
Edward K.L. Chan
K. Arnold Chan
Rebecca Chan
Raymond Chan
Jennifer Chan
Kuan-Rong Chan
David Chan
Jerry Chan
Keith Syson Chan

Ming-Tsair Chan
Kok Gan Chan
Eric Chun Yong Chan
Sam C. C. Chan
Agnes Chan
Stephen Chan
Ting-Fung Chan
Benny Chan
Chi-Ping Chan
Yoke Fun Chan
Ding-Cheng Chan
Julie Chan
Shiao Chan
Che-Chang Chan
Gary Chan
Ta-Chien Chan
Andrew Chan
Christian Chan
Edward Chan
Hing Chan
Michael Chan
Daniel Chan
Wah-Kheong Chan
Kui Ming Chan
Siu Hung Joshua Chan
Wing Chan
Wing Keung Chan
Pei-Chun Chan
Chi Bun Chan
Jason Chan
Dick Chan
Raymond Honfu Chan
Kevin Chan
Piu Chan
Chee Hon Chan
Rachel Chan
Tommy Chan
Dylan Chan
Ming Chan
Ann Chan
Alexandre Chan
Philip Chan
Kwan Nok Chan
Chih-Chiang Chan
Christine Chan
King Chong Chan
Yu Ki Chan
Alex Chan
Xin Hui Chan
Yoke Hwee Chan
Hon Fai Chan
Yee Kwan Chan
Martin Chan
Kun-Ming Chan
Hsiao Yun Chan
Wai Sum Chan
Wallace Chan
James Chan
Chi Ngai Chan
Amy Chan Hilton
Anastasios Chanalaris
Mark Chance
Sourabh Chand
Ramesh Chand
Diptiman Chanda
Giriraj Chandak
Apoorva Chandar
Nalini Chandar
Prataap Chandie Shaw
Michael Chandler
L Judson Chandler
Dawn Chandler
Christopher Chandler
Jesse Chandler
Richard Chandler
Paula Chandler-Laney
Sudhir Chandna
Murali Chandra
Vikash Chandra
Joya Chandra
Namas Chandra
Shanmuganathan Chandrakasan
Parthasarathy Chandrakesan
Rajesh Chandramohanadas
Kartik Chandran
Bharath Chandrasekaran
Sriram Chandrasekaran
Natarajan Chandrasekaran
Bindu Chandrasekharan
K. Chandrashekar
S. Chandrashekara
Bhagwan Chandravanshi
Mammen Chandy
Cassandra Chaney
Guillaume Chanfreau
Ta-Yuan Chang

Yen-Pei Chang
Yongchang Chang
Eric Chang
Kuo-Wei Chang
Caren Chang
Hsing-Yi Chang
Yung-Fu Chang
Ming-Ling Chang
Jun Chang
Anne Chang
Jeff Chang
Belinda Chang
Ching-Fong Chang
Stephanie Chang
Seon Hee Chang
Wen Chang
Gee-Chen Chang
Chun-Che Chang
Anne-Marie Chang
King-Jen Chang
Wen-Tsan Chang
Jinhong Chang
Hong Chang
Wen-Chi Chang
Deh-Ming Chang
Ding-Kao Chang
Tien-Hsien Chang
Andrew Chang
Benny Chang
Sulie Chang
Yuan-Jen Chang
Ing-Feng Chang
Suzanne Chang
Anthony Chang
Chee-Jen Chang
Yu-Chao Chang
Xiao Chang
Kai-Chih Chang
Jung-Su Chang
Young-Cheol Chang
Shwu-Jen Chang
Ke-Vin Chang
Chih-Long Chang
Siow-Wee Chang
Ching-Chien Chang
Wei-Chiao Chang
Junlei Chang
Hao-Yuan Chang
Wei-Chou Chang
Je-Ken Chang
Yih-Leong Chang
Yuxiao Chang
Yaqing Chang
Yu-Kang Chang
Shiou-Chi Chang
Chia-Chuan Chang
Kwanghyeon Chang
Sheng Chang
Jongwook Chang
Hun Soo Chang
Jae-Hoon Chang
Sun-Young Chang
Pahn-Shick Chang
Fengshui Chang
Sheng-Kai Chang
Victor Chang
Woo-Suk Chang
Zhao-Yang Chang
Chi-Yao Chang
Po-Cheng Chang
Chun-Yuan Chang
Hyesang Chang
Chih-Hao Chang
Che-Chang Chang
Soo-Eun Chang
Tammy Chang
Hung Ju Chang
Steven Chang
Franklin Chang
Jerry Chang
Jianxia Chang
Jo-Shu Chang
Jer-Chia Chang
Lijun Chang
Li-Yun Chang
Nien-Tzu Chang
Herbert Chang
Sandy Chang
Angela Chang
Yang-Chi Chang
Wen-Hsin Chang
Jung-Chen Chang
Liang Chang
Warren Chang
Tara Chang
Kuo-Chin Chang

Kuan-Cheng Chang
Chung-Te Chang
Ching-Chun Chang
Hui-Hua Chang
Chih Chang
Jenny Zc Chang
Eugene Chang
Wei-Hau Chang
Chien-Chung Chang
Theresa Chang
Pei-Ching Chang
Shuhua Chang
Shiow-Ru Chang
Malraju Changal Raju
Alastair Channon
Jacqueline Channon Smith
Penradee Chanpiwat
Collins Chansa
Marc Chanson
Tracey Chantler
Guillermo Chantre
Andrew Chantry
Nadia Chanzu
Moses Chao
Nelson Chao
Lin Chao
Anne Chao
Day-Yu Chao
K. S. Clifford Chao
Dai-Yin Chao
Tang Chao
Yanjie Chao
Jane Chao
Huhe Chao
Tiffany Chao
Pen-Hsiu Chao
Shi-Bin Chao
Shashi Chaoudhary
Wanpracha Chaovalitwongse
Tanawat Chaowasku
Prem Chapagain
Florence Chapeland-Leclerc
Robert Chapin
Aaron Chapla
Richard Chapleau
Eddie Chaplin
Becky Chaplin-Kramer
Paul Chapman
Matthew Chapman
Gretchen Chapman
Kent Chapman
Demian Chapman
Scott Chapman
Jason Chapman
Samantha Chapman
Deborah Chapman
Christopher Chapman
William Chapman
Eli Chapman
Kenneth Chapman
Audrey Chapman
Hazel Chapman
Jeremy Chapman
Graham Chapman
Cole Chapman
David Chapman
James Chapman
Virginie Chapon
Catherine Chapon
Svetlana Chapoval
Daniel Chappard
Christine Chappard
Mark Chappell
Patrick Chappell
John Chappell
Dreux Chappell
Catherine Chappell
Maryse Chappin
Paul Chapple
Taylor Chapple
Hélène Chappuy
Jean-Louis Chapuis
Marie-Pierre Chapuis
Benoit Chaput
Brian Chaqour
Bharat R. Char
Ruby Charak
Esmita Charani
Godefroid Charbon
Fadi Charchar
Ayoub Charehbili
Nikhil Chari
A. Charidimou
Marie-Laure Charkaluk
Nisha Charkoudian
Trevor Charles

Cyril Charles
Lucie Charles
Nicolas Charles
Jac Charlesworth
Gavin Charlesworth
Nicolas Charlet-Berguerand
Keyne Charlot
Nikki Charlton
Rachel Charlton
Samuel Charlton
George Charlton
Neil Charman
Gary Charness
Alexander Charney
D Stephen Charnock-Jones
Charlotte Charpentier
Myriam Charpentier
Jean-Paul Charpentier
Isabelle Charrier
Guillaume Charrier
Marie-Christine Chartier-Harlin
Dionysios Chartoumpekis
Katie Chartrand
Nicolas Chartrel
Sofía Charvel
Gilles Charvin
Kandala Chary
Anita Chary
Olga Charyeva
Steve Chase
Christine Chase
Elissa Chasen
Stuart Chaskes
Alex Chaskopoulou
Deborah Chasman
Carine Chassain
Benoit Chassaing
Pauline Chaste
John Chaston
Kaywalee Chatdarong
Pierre-Alexandre Château
Jean-Marc Chatel
Laurent Chatel-Chaix
Lucienne Chatenoud
Liliane Chatenoud
Keith Chater
Mark Chatfield
Talal Chatila
Arnaud Chatonnet
Varanuj Chatsudthipong
Mitali Chatterjee
Subhadeep Chatterjee
Subroto Chatterjee
Nabendu Chatterjee
Anirvan Chatterjee
Sidharta Chatterjee
Somdev Chatterjee
Satabdi Chatterjee
Chiradip Chatterjee
Piyali Chatterjee
Sumantra Chatterjee
Sujoy Chatterjee
Tanaya Chatterjee
Anindita Chatterjee
Jayanta Chatterjee
Arjun Chatterjee
Debashree Chatterjee
Jyotirmoy Chatterjee
Dipankar Chatterji
James Chatters
Robert Chatterton
Mary Lou Chatterton
Nipon Chattipakorn
Siriporn Chattipakorn
Bruno Chatton
Naibedya Chattopadhyay
Krishnananda Chattopadhyay
Sharmila Chattopadhyay
Debprasad Chattopadhyay
S. Chattopadhyay
Debasis Chattopadhyay
Subhasis Chattopadhyay
Debaleena Chattopadhyay
Munmun Chattopadhyay
Pratip Chattopadhyay
Partho Chattoraj
Nagendra Chaturvedi
Vandana Chaturvedi
Sumit Chaturvedi
Eleni Chatzi
Aikaterini Chatzidionysiou
Afroditi Chatzifragkou
Antonios Chatzigeorgiou
Arion Chatziioannou
P. H. Chau
Gar-Yang Chau

Nearkasen Chau
K. W. Chau
Gyaneshwer Chaubey
Abhijit Chaudhari
Vikas Chaudhary
Mamoona Chaudhry
Shehzad Chaudhry
Jayanta Chaudhuri
Abhijit Chaudhuri
Tapas Kumar Chaudhuri
Ovijit Chaudhuri
Roy Chaudhuri
Shubho Chaudhuri
Minu Chaudhuri
Deba Chaudhuri
Arindam Chaudhury
Muhammad Chaudhury
Kamlesh Chauhan
Virander Chauhan
Ashok Chauhan
Subhash Chauhan
Abhay Chauhan
Ashvini Chauhan
Ravendra Chauhan
Vinita Chauhan
Harsh Chauhan
Arun Chauhan
Suriyan Cha-Um
Maximilien Chaumon
Catherine Chaumontet
Arnaud Chaumot
Pratima Chaurasia
Amit Chaurasia
Akhilanand Chaurasia
Catherine Chaussain
Michael Chaussee
Sophie Chauvet
Sylvain Chauvette
Arvind Chavali
Sangeeta Chavan
Hemant Chavan
Stéphane Chavanas
Laia Chavarria
Jorge Chavarro
Pascale Chavatte-Palmer
Marie Chavent
Gloriana Chaverri
Madalena Chaves
Raquel Chaves
Alexandre Chaves
Anderson Chaves
Lais Chaves
Maria Luiza Chaves
Robert Chavez
Noberto C. Chavez
Andres Chavez
De Chavez
Candice Chavez
Sebastián Chávez
Roberto Chávez
Martin Chavez Hoffmeister
Kenneth Chavin
Ali Reza Chavshin
Mamta Chawla-Sarkar
Kazuaki Chayama
Michael Chazan
Bénédicte Chazaud
Gregorio Chazenbalk
Lianqiang Che
Dongsheng Che
Pulin Che
Chris Cheadle
William Cheadle
Jacob Cheadle
Phaik Yeong Cheah
Peh Yean Cheah
Yoke-Kqueen Cheah
Bentley Cheatham
Daniel-Robert Chebat
Mary Chebib
Ivan Chebib
Frederic Checler
Abbas Cheddad
Amrita Cheema
Joseph Cheer
Ian Cheeseman
Michael Cheetham
Marcus Cheetham
Timothy Cheetham
Novel Chegou
Farid Chehab
Hassib Chehade
Nor Chejanovsky
Timofey Chekalkin
Lucy Cheke
Martha Chekenya

Linda Chelico
Prashen Chelikani
Meenakshi Chellaiah
Sarah Chellappa
Karthi Chellappa
Riccardo Chelli
Ivo Chelo
Shekar Chelur
Lucia Chemes
Isabelle Chemin
Joseph Chemler
Jonathan Chemouny
Daniel Chemtob
Abel Chemura
Weisan Chen
David Chen
Min Chen
Cathy Chen
Chien-Hsiun Chen
Feng-Chi Chen
Kow-Tong Chen
Yan Chen
Xin Chen
Yiping Chen
Ching-Yi Chen
Shu Chen
Xuewei Chen
Nansheng Chen
Ye-Guang Chen
Xin Jie Chen
Xiangmei Chen
Pau-Chung Chen
Jaw-Wen Chen
Jiandong Chen
Zeliang Chen
Antao Chen
Rui Chen
Liwei Chen
Ruoling Chen
Su-Shing Chen
Xiu-Lan Chen
Shuang Chen
Benjamin Chen
Chang-Han Chen
Hsin-Wei Chen
Songlin Chen
Xiang-Sheng Chen
Jason Chen
Guo-Bo Chen
Shih-Pin Chen
Yin Chen
Jinyun Chen
Chaomei Chen
Xing-Zhen Chen
George Chen
Hai-Feng Chen
Yanfang Chen
Hui Chen
Yung Chen
Liang Chen
Guo-Qiang Chen
Zhe-Sheng Chen
Yan Ping Chen
Peter Chen
Keping Chen
Yaosheng Chen
Chien-Chung Chen
S.R. Wayne Chen
Liang-Kung Chen
Haoyu Chen
Yu-Guo Chen
Chu Chen
Chiao-Chi Chen
Chang Yan Chen
Ruey-Hwa Chen
Bin Chen
Ming-Shun Chen
Xiao-Ya Chen
Yasheng Chen
Chang-Shi Chen
Chien-Cheng Chen
Yee-Chun Chen
Yau-Hung Chen
Jianchi Chen
Jin Chen
Yu Chen
Yu-Chun Chen
Haijun Chen
Guan Chen
Hsiao-Huei Chen
Qi Chen
Zigui Chen
Bor-Sen Chen
Limei Chen
Weijun Chen
Chiu-Ying Chen

Yu Wai Chen
Lanming Chen
Chiung-Mei Chen
Chi-Shuo Chen
Feng Chen
Jyh-Cheng Chen
Qin Chen
Henry Chen
Shangbin Chen
Chien-Yu Chen
Gong-You Chen
Jiann-Chu Chen
Jianquan Chen
Hsuan-Yi Chen
Chengrong Chen
Yanguang Chen
Jing Chen
Jonathan Chen
Wensheng Chen
Chider Chen
Ming Chen
Huei-Wen Chen
Xiaoping Chen
Li-Song Chen
Jinping Chen
Bao-Ming Chen
Chao Chen
Haiquan Chen
Zhilei Chen
Chi Chen
Shi-You Chen
Mark Chen
Zheyu Chen
Xiao-Ping Chen
Yung-Chang Chen
Cheng-Yu Chen
Xing Chen
Xiaojie Chen
Li-Mei Chen
Hung-Chi Chen
Jun Chen
Jang-Yi Chen
Jeremy J. W. Chen
Xi Chen
Xuhai Chen
Wei Chen
Qianqian Chen
Pao-Yang Chen
Hanbo Chen
Ceshi Chen
Ssu-Ching Chen
Chunhong Chen
Haibing Chen
Fang Chen
Xiaoxin Chen
Xianming Chen
Wei-Sheng Chen
Jun-An Chen
Tzu-An Chen
Jen-Chih Chen
Yen-Hao Chen
Charles Chen
Taosheng Chen
Huai Chen
Kun-Ming Chen
Wenhao Chen
Lizhang Chen
Yang-Ching Chen
Rong Chen
Yen-Hsu Chen
Yung-Che Chen
Ruei-Ming Chen
Chi-Ling Chen
Wilfred Chen
Yuxin Chen
Lingyi Chen
Jyh-Yih Chen
Jyh-Ping Chen
Chen Chen
Chuan-Yu Chen
Zhuo (Adam) Chen
Yi-Jane Chen
Hungwen Chen
Yong Chen
Xinbo Chen
Li-Sheng Chen
Dima Chen
Huey-Ling Chen
Zijiang Chen
Shuqing Chen
Chien-Yuan Chen
Dar-Ren Chen
Shih-Ann Chen
Ben-Kuen Chen
Swaine Chen
Likwang Chen

Yongsheng Chen
Chu-Huang Chen
Ku-Fan Chen
Tai-Been Chen
Xuelong Chen
Yuh-Lien Chen
Yueh-Sheng Chen
Mingxing Chen
Yigen Chen
Yongbin Chen
Mingzhou Chen
Hsi-Chung Chen
Peijie Chen
Li-Tzong Chen
Yizhou Chen
Ya-Lei Chen
Maohua Chen
Chun-An Chen
Xiao-Ren Chen
Cheng-Sheng Chen
Xiaofeng Chen
Wenbiao Chen
Xiaoyun Chen
Fu-Sheng Chen
Jin-Shuen Chen
Jen-Hau Chen
Suning Chen
Xuesong Chen
Zheng Chen
Weizao Chen
Kun Chen
Ying Chen
Yuh-Fung Chen
Yi-Jen Chen
Ji-Long Chen
Tianmu Chen
Yun Chen
Tao Chen
Yung-Tai Chen
Eunice Chen
Yi-Lin Chen
Wei-Ta Chen
Shih-Chu Chen
Chin-Yi Chen
Chung-Ming Chen
Tian Chen
Grace Chen
Chunxian Chen
Hong Chen
Mei-Huei Chen
Qingfeng Chen
Qingxia Chen
Wei-Yu Chen
Xiuping Chen
Limin Chen
Rong-Jane Chen
Shuo Chen
Yun-Ju Chen
Chien-Hung Chen
Zhanghua Chen
Mei Chen
Qiang Chen
Yi-Chen Chen
Yunfeng Chen
Hsiang-Yin Chen
Shun-Hua Chen
Elizabeth Chen
Fanglin Chen
Weiguo Chen
Xiaoji Chen
Chun-Ku Chen
Ping-Chung Chen
Weiwei Chen
Nai-Hong Chen
Jie Chen
Rachel Chen
Lei Chen
Dongshi Chen
Li Chen
Baihua Chen
Joyce Chen
Xiaoshu Chen
S. Chen
Xiaoguang Chen
Guangping Chen
Xiaoying Chen
Yahong Chen
Han Chen
Chiung-Ling Chen
Wen Chen
Chien-Tzung Chen
Yingxi Chen
Mei-Lien Chen
Yongheng Chen
Xiangjun Chen
Shiping Chen

Junping Chen
Gin-Shin Chen
Hua Chen
Zhen Chen
Ping-Jen Chen
Jauer Chen
Youzhou Chen
Chiehfeng Chen
Xianhong Chen
Ray-Jade Chen
Fuyi Chen
Youxin Chen
Shee-Uan Chen
Miao-Hsueh Chen
Zhichang Chen
Kai Chen
Chun Chen
Yi-Qiang Chen
Yulin Chen
Zhao Chen
Bang-Bin Chen
Jian-Ye Chen
Aimin Chen
Mu Chen
Da Chen
Yi-Cheng Chen
Janice Chen
Jian Chen
A. Chen
Xiaolei Chen
Honglei Chen
Yunji Chen
Roy Chen
Robert Chen
Yeng-Long Chen
Yi-Ting Chen
Litong Chen
Yinghu Chen
Cynthia Chen
Xinjian Chen
Xiwen Chen
Jian-Jun Chen
Claire Chen
Jin-Hua Chen
Qiong Chen
Wen-Pin Chen
Lihong Chen
Yongbo Chen
Jaqueline Chen
Diyi Chen
Alan Chen
Lang Chen
Cheng-Ci Chen
Roger Chen
Youjun Chen
Jih-Jung Chen
Joanna Y-F Chen
Wanping Chen
Hongying Chen
Jiande Chen
Jiansheng Chen
Rongsheng Chen
Juine-Ruey Chen
Po Chuan Chen
Yi-Hsin Chen
Yung-Yi Chen
Xiaomin Chen
Xuwei Chen
Li-Chia Chen
Pei-Chun Chen
Chunli Chen
Zhiyuan Chen
Huisheng Chen
Cheng Chen
Gang Chen
Shuhang Chen
Jianjiao Chen
Hung-Hsuan Chen
Xuehao Chen
Huifang Chen
Zhiyong Chen
Runsheng Chen
Xiaowen Chen
Duan-Bing Chen
Shunde Chen
Songbiao Chen
Hailong Chen
Wei-Chuan Chen
Guangdi Chen
Wei-Neng Chen
Long-Sheng Chen
Mingjie Chen
Lih Chen
Allen Chen
Wan Chen
Ru Chen

I-Ping Chen
Mike Chen
Jingyuan Chen
Ran Chen
Yi-Min Chen
Shu-Hui Chen
Hsuan-Yu Chen
Tianle Chen
Xueni Chen
Hsin-Hua Chen
Shu-Ching Chen
Jianjun Chen
Kailiang Chen
Eric Chen
Lijie Chen
Nihong Chen
Chunhua Chen
Elaine Chen
Han-Yang Chen
Hanying Chen
Geng Chen
Yabin Chen
Jiguang Chen
Weiqian Chen
Yunching Chen
Maogen Chen
Oliver Chen
Juan Chen
Songhai Chen
Wei-Ju Chen
Zhiguo Chen
Yao-Hui Chen
Fujun Chen
Hongwei Chen
Yingyeh Chen
Lien-Cheng Chen
Bo Chen
Linxiao Chen
Wenbo Chen
Tony Chen
Shaohua Chen
Yi-Pu Chen
Try Xi Chen
Chunyan Chen
Kong Chen
Tianwen Chen
Shaojie Chen
Jinpeng Chen
Wei-Hsiang Chen
Jingchang Chen
Yalin Chen
Pingkun Chen
Jian-Ning Chen
Zhenmin Chen
Weitian Chen
Zhiliang Chen
Jixin Chen
Tsung-Ming Chen
Shuyan Chen
Guangjie Chen
Kuan-Hui Chen
Jingchun Chen
Yuhui Chen
Lijing Chen
Yousheng Chen
Bingkun Chen
Peng Chen
Shao-Hua Chen
Weile Chen
Liming Chen
Yunliang Chen
Yi-Tien Chen
Tenghui Chen
Yixin Chen
Junhong Chen
Zhongsheng Chen
You Chen
Trevor Chung-Ching Chen
Xiaoli Chen
Chiao-Yun Chen
Fa-Jun Chen
Congying Chen
Kui Chen
Ping-Hsien Chen
Young-Mao Chen
Jenny Chen
Ching-Hsein Chen
Pin-Yu Chen
Taolin Chen
Siyu Chen
Ping Chen
Shen Liang Chen
Yuchun Chen
Guoqian Chen
Sheng Chen
Xiangdong Chen

Zhiyun Chen
Hsin-Jen Chen
Kunsong Chen
Ying-Chu Chen
Yanxi Chen
Jeng-Haur Chen
Yu-Cheng Chen
Huanhuan Chen
Guangwen Chen
Alexandre Chenal
Ezhilarasi Chendamarai
Arnaud Chene
Richard Cheney
Qin Cheng
Keding Cheng
Jing Cheng
Keith Cheng
Tingcai Cheng
Alfred Cheng
Chi-Lien Cheng
Bin Cheng
Ji-Yen Cheng
Tong Cheng
C Yan Cheng
Liting Cheng
Yi-Chuan Cheng
G. Cheng
Pin-Nan Cheng
Kang Cheng
Ziqiang Cheng
Yuchung Cheng
Qijin Cheng
Xiang Cheng
Weixin Cheng
Ching-Feng Cheng
Lu Cheng
Feixiong Cheng
Qing Cheng
Wen-Fang Cheng
Alan Cheng
Anchun Cheng
Yuxiang Cheng
Gong Cheng
Yu-Ling Cheng
Shou-Hsia Cheng
Caroline Cheng
Iona Cheng
Hong Cheng
Kian-Kai Cheng
Hongmei Cheng
Hai-Ying Cheng
Yuqi Cheng
Liang Cheng
Huai Cheng
Jack Cheng
Shih-Hsun Cheng
Oumei Cheng
Heung-Chin Cheng
Yingduan Cheng
Jian Cheng
Hao Cheng
Jiuqing Cheng
Steven Cheng
Zhang Cheng
Ying Wu Cheng
Xiaoqian Cheng
Wen-Hsing Cheng
R. Holland Cheng
Nikki Cheng
Feng Cheng
Chao-Wen Cheng
Chia-Hsiung Cheng
Robert Cheng
Yongzhong Cheng
Tiejun Cheng
Ke Cheng
Ya Cheng
Tao Cheng
Jya-Wei Cheng
Ren-Chung Cheng
Yunfeng Cheng
Jun Cheng
Long Cheng
Jimin Cheng
Yuwei Cheng
Ao Cheng
Chunming Cheng
Dongliang Cheng
Shuiping Cheng
Sam Cheng
Shaoan Cheng
Xuansheng Cheng
Xianwu Cheng
Po Jen Cheng
Zi-Xi Cheng
Bi-Hua Cheng

Brian Cheng
Shiyuan Cheng
Kai-Chun Cheng
Zhangrui Cheng
Fa-Juan Cheng
Junzhou Cheng
Wen-Hsi Cheng
Han Cheng
Jianzhong Cheng
Zhongyang Cheng
Mei-Ling Cheng
Shunfeng Cheng
Aixin Cheng
B. Cheng
Qin-Bo Cheng
Yuanyuan Cheng
Yang Cheng
Li-Hua Cheng
Gang Cheng
Suk Hang Cheng
Hsueh-Ling Cheng
Tina Cheng
Dong Seon Cheng
Zhang Chengcui
Jin Cheng-Qiang
Alice Chen-Plotkin
Yulia Chentsova Dutton
Chantal Chenu
Anne Chenuil
Dong-Joo Cheon
Gi Jeong Cheon
Hyae Gyeong Cheon
Young Koog Cheon
Jaehun Cheong
Siew Ann Cheong
Jeanie Cheong
Alex Cheong
Taesu Cheong
Kartikeya Cherabuddi
Bobby Cherayil
Farid Cherbal
Claire Cherbuy
Yves Cherel
George Cherian
Tom Cherian
Alhaji Cherif
Ameur Cherif
Vino Cheriyan
Artem Cherkasov
Yijuang Chern
Phern Chern Tor
Nikita Chernetsov
David Cherney
Howard Chernick
Jonathan Chernoff
Ariel Chernomoretz
Vladislav Chernov
Igor Chernyavsky
Anatoly Chernyshev
Anas Cherqui
Gary Cherr
James Cherry
Julia Cherry
Elizabeth Cherry
Matthew Chersich
Thomas Chertemps
Beata Chertok
Daniel Chertow
Paolo Cherubini
Andrea Cherubini
Antonio Cherubini
Frank Chervenak
Eric Cheshin
Alessandra Chesi
Megan Chesin Chesin
Alex Chesler
David Chesler
Alessandro Chessa
Manuela Chessa
Stefania Chessa
Harrell Chesson
Julia Chester
David Chester
Hugh Chester-Jones
Douglas Chesters
Tara Chestnut
Rose Chesworth
Basundhara Chettri
Ellen. Chetwynd
Daniel Cheuk
Gordon Cheung
Po-Yin Cheung
Olivia Cheung
Allen Cheung
Stephen Cheung
Man Kit Cheung

Him Cheung
Carol Cheung
Wing-Hoi Cheung
Ching-Lung Cheung
Roy Cheung
Chi-Wai Cheung
Celeste Cheung
S. T. Cheung
Mei-Chun Cheung
Alice Cheung
Doug Cheung
King-Ho Cheung
Rocky Cheung
Tom Cheung
Ivy Cheung
Martin Cheung
Dorothy Cheung
Francis Cheung
S. Cheung
Wisit Cheungpasitporn
Boris Cheval
Christian Chevalier
François Chevalier
Mathieu Chevalier
Philippe Chevalier
Frédéric Chevalier
Xavier Chevalier
Stéphane Chevaliez
Frederic Chevessier
Eric Chevet
Christophe Chevillard
Mordechai Chevion
Nathalie Chevre
Sylvie Chevret
Emily Chew
Lock Chew
Eng Hui Chew
Claire Chewapreecha
Benjamin Cheyette
Raghav Chhetri
Netra Chhetri
Purna Chhetri
Jen-Tsan Chi
Hongbo Chi
Yuling Chi
Xiao Chi
Junwook Chi
Chih-Lin Chi
Shau-Chi Chi
Yanling Chi
Godfrey Chi Fung Chan
Nicholas Chia
Jean-San Chia
Jonathan Chia
Brian Chia
John Chia
Fulvio Chiacchiera
Cristiano Chiamulera
Ri-Cheng Chian
Chiara Chianese
Chin Chiang
Y. Jeffrey Chiang
Hui-Ling Chiang
Chen-Yuan Chiang
Yung-Hsiao Chiang
Yen-Sheng Chiang
Yu-Chung Chiang
Jui-Kun Chiang
Chun-Te Chiang
John Chiang
Samuel Chiang
Chun-Pin Chiang
An-Na Chiang
Charleston Chiang
Hsiu-Mei Chiang
Yao-Yi Chiang
Salvatore Chiantia
Maria Vincenza Chiantore
Mariachiara Chiantore
Chuan-Chin Chiao
Paul Chiao
Elizabeth Chiao
Marco Chiapello
Gennaro Chiappetta
Geneviève Chiapusio
Ferdinando Chiaradonna
Marcos Chiaratti
Adriano Chiarello
Mario Chiariello
Helio Chiarini-Garcia
Giuseppe Chiarioni
Ornella Chiavola
Peter Chiba
Shuichi Chiba
Rie Chiba
Seiichi Chiba

Chikafumi Chiba
Kelly Chibale
Alexander Chibalin
Ravindra Chibbar
Carla Chibwesha
Adam Chicco
Adriana Chicco
Laurent Chiche
Vitaly Chicherov
Chris Chickadel
Vidya Chidambaran
Peter Chidiac
Wei-Chu Chie
Eui Kyu Chie
Eusebio Chiefari
Paolo Chieffi
Ming-Hsien Chien
Rong-Nan Chien
Hua-Hong Chien
Li-Chien Chien
Yin-Hsiu Chien
Chun-Wei Chien
Arturo Chieregato
Jose Artur Bogo Chies
G. Chiesa
Flaminia Chiesa
Yeong Shiong Chiew
Elise Chiffoleau
Yuna Chiffoleau
Cecilia Chighizola
Norio Chihara
Dai Chihara
Obinna Chijioke
Anton Chikaev
Yoshimura Chikara
Yoshito Chikaraishi
Shailaja Chilappagari
Stephanie Child
Dylan Childs
Emma Childs
Jason Childs
Martin Chilvers
Sylvester Chima
Sergio Chimenti
Isotta Chimenti
Patricia Chimin
Wei-Chun Chin
Kazuo Chin
Michael Chin
Andrew Chin
Arnold Chin
Weng Yee Chin
Marcello Chinali
Mai Chinapaw
V. Gregory Chinchar
Zaida Chinchilla-Rodriguez
Leonid Chindelevitch
Wn Ching
Siok Siong Ching
Andrea Chini
Marco Chini
Sreenivasa Chinni
Yuzo Chino
Christos Chinopoulos
Hector Chinoy
Shankar Chinta
Sreenivasulu Chintala
Adriano Chiò
Roberto Chiocchetti
Gilles Chiocchia
Paolo Chiodini
Rodrick Chiodini
Mario Chiong
Edmund Chiong
Arthur Chiou
Pinwen Chiou
Hung-Yi Chiou
Chiuan-Chian Chiou
Shyh-Horng Chiou
Yi-You Chiou
Jeng-Fong Chiou
Pinwen Peter Chiou
Luca Chiovato
Shubhada Chiplunkar
Ariel Chipman
Lindsay Chipman
Abigail Chipman
Jean-Philippe Chippaux
Matthias Chiquet
Johanne Chiquette
Abhilash Chiramel
Gianpaolo Chiriano
Giuseppe Chirico
Valeria Chirico
Andrea Chiricozzi
Lucian Chirieac

Gregory Chirikjian
Maurizio Chiriva-Internati
Guy Chiron
Carmelo Chisari
Meg Chisolm
Ayman Chit
Pasquale Chitano
Walter Chitarra
Fabrizio Chiti
Ritu Chitkara
Teodor Chitlaru
Chetan Chitnis
Yacine Chitour
Kumaraswamynaidu Chitrala
Tom Chittenden
Prashant Chittiboina
Lars Chittka
Lyn Chitty
M. Chitwood
Susan Chiu
Charles Chiu
Cheng-Hsun Chiu
Joanna Chiu
Yi-Yuan Chiu
Chong-Chi Chiu
Ya-Wen Chiu
Hui-Wen Chiu
Chih-Yu Chiu
Wei Che Chiu
Hao Chiu
Yen-Cheng Chiu
Wei-Yih Chiu
Chee Chiu
Y-Wen Chiu
Nai-Chi Chiu
Valerio Chiurchiù
Miguel Chiurillo
Susan Chivers
Meredith Chivers
Tawanda Chivese
Venkat Keshav Chivukula
Patrick Chiyo
Howard Chizeck
Vyacheslav Chizhevsky
Carlo Chizzolini
Katerina Chlichlia
George Chlipala
Helena Chmura Kraemer
Agata Chmurzynska
Hervé Chneiweiss
Kern Rei Chng
Eng Siong Chng
Choon Peng Chng
Eugene Ch'Ng
Nam-Hyuk Cho
Judy Cho
Yong-Gu Cho
Young Min Cho
Nam Hoon Cho
Chunghee Cho
Myung-Haing Cho
Won Kyong Cho
Joo-Youn Cho
Hongsik Cho
Dong-Hyung Cho
Seok-Goo Cho
Steve Cho
Michael Cho
Je-Yoel Cho
Sung Yong Cho
Younghak Cho
Seung-Woo Cho
Min Chul Cho
Hye Sun Cho
Won Yong Cho
Nam-Joon Cho
Yeoungjee Cho
Yoon-Kyoung Cho
Patricia Cho
Jaehyung Cho
Young I Cho
Eunyoung Cho
Seungho Cho
Hyoung Jin Cho
Hwi-Young Cho
Er-Chieh Cho
Sukki Cho
Hy Cho
Clifford Cho
Cheorun Cho
Seong Beom Cho
Wookyung Cho
Sheryl Chocron
Yoonsuck Choe
Keith Choe
Seong-Kyu Choe

Kwang-Wook Choi
Kang-Yell Choi
Byung Tae Choi
Murim Choi
Jae Young Choi
Young Ki Choi
Eun-Kyoung Choi
Kyung Mook Choi
Je-Yong Choi
Jeong-Hyeon Choi
Yung Hyun Choi
Horace Choi
Young Min Choi
Si Ho Choi
Hyung-Kyoon Choi
Jaeyoung Choi
Inho Choi
Ki Choon Choi
Joon Young Choi
Inyong Choi
Hongkyu Choi
Hyo Geun Choi
Hoon Young Choi
Yu Suk Choi
In-Geol Choi
Yoon Kyung Choi
Michael Choi
Jae-Hyeok Choi
Seungbum Choi
Young-Joon Choi
Sang-Il Choi
Inchul Choi
Jae-Hyuk Choi
In-Suk Choi
Jung-Hye Choi
Jay Chol Choi
Sugy Choi
Joong Sub Choi
Sang Ii Choi
Yoonjoung Choi
Tae Young Choi
Y. Choi
Jin-Seung Choi
Cheol Yong Choi
Young-Kil Choi
Chang Won Choi
Seokheun Choi
Jonghoon Choi
Serah Choi
Seon Young Choi
Kelvin Choi
Hyung Jin Choi
Hyong Woo Choi
Yongwook Choi
Se-Woong Choi
Marc Choisy
David Chojnacky
Rubens Chojniak
Kenneth S. H. Chok
Kashyap Choksi
Elena Choleris
Brenna Cholerton
Lukas Choleva
Jacek Cholewicki
Evangelos Cholongitas
Michael Choma
Theodore Choma
Chao Cho-Ming
Su-Hyoun Chon
Kowit-Yu Chong
Kang Chong
Sang Chul Chong
Victor Chong
Ving Ching Chong
Zechen Chong
Sook Yee Chong
Huihui Chong
Siang Yew Chong
Luke Chong
Hao Chongqing
Karen Chong-Seng
Virasak Chongsuvivatwong
Virasakdi Chongsuvivatwong
Myung-Soo Choo
Young Kug Choo
Sarvenaz Choobdar
Babak Choodari-Oskooei
Yit-Heng Chooi
Yuh Min Chook
Mahesh Choolani
Denis Chopera
Ashok Chopra
Kanwaljit Chopra
Shauhrat Chopra
Vikas Chopra
Teena Chopra

Rémi Choquet
Helene Choquet
Benny Chor
Nikos Chorianopoulos
Brian Chorley
Joanna Chorostowska-Wynimko
Chang-Hung Chou
Wen-Chien Chou
Shen-Ju Chou
Che-Yi Chou
Joshua Chou
Li-Shan Chou
Yu-Ting Chou
Wenchi Chou
Wen-Ying Chou
Michelle Chou
Loke Ming Chou
Chinmei Chou
Kee Lee Chou
Ruey-Hwang Chou
Shih-Feng Chou
Bessem Chouaia
Chiang-Ting Choud
Sanjeev Choudary
Dharamainder Choudhary
Pratik Choudhary
Alok Choudhary
Omar Choudhri
Mashkoor Choudhry
Mahua Choudhury
Claire Chougnet
Jyoti Chouhan
Silvina Choumenkovitch
Valerie Choumet
Natalia Chousou-Polydouri
Louise Chow
Yen-Hung Chow
Eric Chow
Keng-See Chow
Seinen Chow
Robert Chow
Louis Chow
Daniel Chow
Wang-Ngai Chow
Chin Moi Chow
Li Sze Chow
Edward Chow
Chi Kin Chow
K. W. Chow
Balram Chowbay
Anuradha Chowdhary
Rukhsana Chowdhury
Parimal Chowdhury
Shafiqul Chowdhury
Sanjib Chowdhury
Mahbub Chowdhury
Soumitra Chowdhury
Ehsan Chowdhury
Tamjid Chowdhury
Souma Chowdhury
Gerardo Chowell
Itay Chowers
Michal Chowers
David Choy
Henry Choy
Edwin Choy
Mary-Margaret Chren
Jean-Paul Chretien
Denis Chretien
Cantwell Chris
Bruno Christ
Emanuel Christ
Torsten Christ
Urania Christaki
Nicholas Christakis
George Christakos
Philippe Christe
Robert Christelle
Beat Christen
Peter Christen
Nicholas Christenfeld
Erik Christensen
Henrik Christensen
Jørn Christensen
Cory Christensen
Brock Christensen
Terje Christensen
Brian Christensen
Tue Christensen
Hannah Christensen
Garret Christensen
Stephanie Christenson
Olivier Christiaens
James Christian
Natalie Christian
Uwe Christians

Jesper Riis Christiansen
Bo Christiansen
Blaine Christiansen
Danny Christiansen
Hanna Christiansen
Gunna Christiansen
Christian Christiansen
Julie Christianson
Tatiana Christides
John Christie
Joshua Christie
Schindler Christina
Brandenberger Christina
Rob Christley
Karen Christman
Stephen Christmas
Manolis Christodoulakis
Myron Christodoulides
Vincent Christoffels
Michael Christoffers
Christina Christoffersen
Gustavo Christofoletti
John Christoforidis
Nicolas Christoforou
Habas Christophe
Olivier Christophe
Solomon Christopher
Kenneth Christopher
Michael Christopher
Mark Christopher
Elliott Christopher
Ndoleriire Christopher
Theodore Christopher
Lisa Christopher-Stine
Katerina Christopoulos
Theodore Christopoulos
A. Christopoulos
Marta Christov
John Christy
Annette Christy
Nicolas Chromerat
Veronika Chromikova
Ioannis Chronakis
Zissis Chroneos
Elisabeth Chroni
Eva Chronowska
Ewa Chrostek
George Chrousos
Maksymilian Chruszcz
Caitlin Chrystoja
Zofia Chrzanowska-Lightowlers
Hamdi Chtourou
Yacine Chtourou
Jhih-Wei Chu
Xiang-Ping Chu
Zhaohui Chu
Chi-Ming Chu
Yiwei Chu
Zhaoqing Chu
Mingxing Chu
Wen-Ming Chu
Justin Jang Hann Chu
Tzong-Shinn Chu
Hong Wei Chu
Xiaowen Chu
Hongyuan Chu
Yiu Wai Chu
Kon Chu
Dong Chu
Jia-Qi Chu
Min Kyung Chu
Pei-Yi Chu
Wanshun Chu
Jaime Chu
Fang-Hua Chu
Amanda Chu
Chishih Chu
Janet Chu
Ch Chu
Lee Chu
James Chu
Binh Chu
Thuan Chu
Chun-Yen Chu
Weihua Chu
Annie Chu
Delphine Chu Miow Lin
Fook Kee Chua
Melvin Lee Kiang Chua
Anita Chua
Siew Siang Chua
Felix Chua
Jacqueline Chua
Seng-Kee Chuah
Aaron Chuah
Ts Chuah

Gwo-Yu Chuang
Pao-Tien Chuang
Yung-Jen Chuang
Lee-Ming Chuang
Chiou-Fen Chuang
Yao-Chi Chuang
Kai-Jen Chuang
Kai-Hsiang Chuang
Shih-Sung Chuang
Jiin-Haur Chuang
Tsung-Hsien Chuang
Yi-Ping Chuang
Shuang-En Chuang
Kuo-Hsiang Chuang
Wen-Yu Chuang
Shu-Chun Chuang
Yi-Fang Chuang
Wen-Po Chuang
Jian-Ying Chuang
Chia-Chi Chuang
Yung-Yu Chuang
Ming-Chin Chuang
Huai-Hu Chuang
Po-Heng Chuang
Vladimir Chubanov
Lon Chubiz
Ana Chucair-Elliott
Shih-Chieh Chueh
Jane Chueh
Diane Chugani
Bilal Chughtai
Dehua Chui
Hiroshi Chuman
Jerold Chun
Tae-Hwa Chun
Kyung-Hee Chun
Taehoon Chun
Jong Ahn Chun
Yvonne Chun
Rumi Chunara
I-Fang Chung
Moo Chung
Kwang Chung
Kian Chung
Chun Kee Chung
Ren-Hua Chung
Benjamin Chung
Hun-Tag Chung
Ming-Yi Chung
Fu-Tsai Chung
Henry Chung
Brian Chung
Hsiao-Wen Chung
Doo Hyun Chung
Chi-Jung Chung
Yong Eun Chung
Min-Huey Chung
Kyungsoon Chung
Won-Yoon Chung
Woo Jin Chung
Suyoun Chung
Sungjin Chung
Joanne Chung
Wen-Yu Chung
Frances Chung
Sochung Chung
Kuo Fang Chung
Ka Fai Chung
Sun Sook Chung
Al Chung
Yeon-Ho Chung
Yeun Goo Chung
Donghoon Chung
Sang-Hyuk Chung
Taijoon Chung
Wendy Chung
Jae Chung
Hae-Sun Chung
J. Sook Chung
Ki Wha Chung
Ren-Jei Chung
Cheng-Ming Chuong
Michael Chuong
George Church
Timothy Church
Candice Church
Tim Churches
Nathan Churchill
Morgan Churchill
Alice Churchill
Jessica Church-Lang
Tyler Churchward-Venne
Andrew Churg
Alexander Churkin
Daniella Chusyd
Anil Chuturgoon

Chih-Pin Chuu
Emilio Chuvieco
Michael Chvanov
Charng-Cherng Chyau
Tomasz Ciach
Lynn Cialdella-Kam
Giovanni Luca Ciampaglia
Andrea Ciampalini
Paolo Ciana
Giovanni Ciana
Carlo Cianchetti
Fiona Cianci
Claudia Cianci
Bruno Ciancio
George Cianciolo
Katherine Cianflone
Elena Ciani
Oriana Ciani
Lorenzo Ciannelli
Maria Ciaramella
Aitor Ciarreta-Antuñano
Andrea Ciavattini
Pawel Ciborowski
Claudia Cicala
Cinzia Ciccacci
Olga Ciccarelli
Daniela Ciccarelli
Francesco Ciccarese
Sonia Ciccazzo
Jessica Cicchino
Francesco Ciccia
Rachele Ciccocioppo
Giovannino Ciccone
Marco Ciccone
Giovanni Ciccotti
Massimo Ciccozzi
Alessandro Cicerale
Arrigo Cicero
David Cicero
Frank Cichocki
Marilena Ciciarello
Stefano Ciciliot
Lucia Ciciolla
Sanja Cicko
Helena Cicková
Fabio Cicoira
Giancarlo Cicolini
Maria Cid
Marzena Ciechomska
Marta Ciechonska
Przemyslaw Ciepiela
Marek Cieplak
Christian Cierpka
Kristina Ciesielski
Grzegorz Cieslar
Marcin Cieslik
Carlo Cifani
Ingrid Cifola
Víctor Cifuentes
Liliana Cifuentes
S. Cigarran
Secundino. Cigarran Guldris
Jan Ciger
Andrea Cignarella
Angelo Cignarelli
A. Cihat Baytas
Roberto Cilia
Elisa Cilia
Corrado Cilio
Rodrigo Cilla
Anthony Cillo
Nicholas Cilz
Andrea Cimarelli
Helena Cimarosti
Thomas Cimato
Raffaello Cimbro
Antonio Cimellaro
Phillip Cimiano
Giulio Cimini
Roberta Cimmaruta
Alessio Cimmino
Veronica Cimolin
Anca Maria Cimpean
Ali Cinar
Resat Cinar
Melih Ertan Cinar
Mehmet Ulas Cinar
Juan Cinca
Aude Cincotta
Luca Cindolo
Tereza Cindrova-Davies
Michael Cinelli
Antonella Cingolani
Joshua Cinner
Raphael Cinotti
Saverio Cinti

Jessica Cioffi
Emmanuel Ciolac
Marco Ciolfi
Gabriele Cioni
Matthew Ciorba
Joseph Ciorciari
Alexander Ciota
Marco Ciotti
Ana Cipak Gasparovic
Amaia Cipitria
Marilyn Cipolla
Marilena Cipollaro
Amanda Ciprandi Pires
Pietro Cipresso
Gabriele Cipriani
Matheus Cipriano
Laura Cirelli
Daniela Cirillo
Giuseppe Cirino
Maria Ciriolo
Connie Cirrincione
Elizabeth Cirulli
Francesca Cirulli
Josh Cisler
Gerardo Cisneros
Irma Cisneros
Badara Cisse
Guéladio Cissé
Luca Cistrone
Peter Cistulli
Brittney Ciszek
Annarita Cito
Antonio Cittadini
S. Citterio
Paolo Ciucci
Carlos Ciudad
Alessio Ciulli
Marina Ciullo
Adrian Ciurea
Claudia Civai
John Civale
Peter Civán
Roberto Civitelli
Carrie Cizauskas
Jenny Clack
Nancy Cladel
Stephan Claes
Nathalie Claes
Heiner Claessen
Dennis Claessen
Frank Claessens
Geert Claeys
Corentin Claeys Bouuaert
Sylvie Claeysen
Jessica Clague
Audrey Claing
Eric Clambey
Alexander Clanachan
Colleen Clancy
Kathryn Clancy
David Clancy
Paul Clapham
Hannah Clapham
Pascal Claquin
Elizabeth Clare
Christian Clarenbach
Marc Claret
Mark Clarfield
Diane Claridge
Andrew Claridge
Lee Claridge
Jordi Clarimon
David Clark
John Clark
Melody Clark
Timothy Clark
Jesse Clark
J. Clark
Rulon Clark
Theodore Clark
Karl Clark
Clifford Clark
Abbot Clark
William Clark
Christopher Clark
Deborah Clark
Nathan Clark
Richard Clark
Chris Clark
Maggie Clark
Corinna Clark
Brian Clark
Carrie Clark
Susan Clark
Lorraine Clark
Jennifer Clark

Andrea Clark
Luke Clark
Adam Clark
Robert Clark
Mary Clark
Anne B. Clark
Alex Clark
Josephine Clark-Curtiss
Ian Clarke
Duncan Clarke
Steven Clarke
Robert Clarke
Thomas Clarke
Murray Clarke
Christopher Clarke
Stephen Clarke
Aaron Clarke
David Clarke
Ed Clarke
Angus Clarke
Neil Clarke
Siobhan Clarke
Nigel Clarke
John Clarke
Amy Clarke
Kenneth Clarke
Simon Clarke
J. Clarke
John Clarkson
Jill Clarridge 3Rd
Lorna Clarson
Thomas F. Clasen
Joseph Classen
Thomas Classen
Philip Clatworthy
Erika Claud
Lardicci Claudio
Ralf Claus
Harald Claus
Eric Claus
Tine Clausen
Frederik Clausen
Morten Clausen
Kevin Clausen
Malene Clausen
Preben Clausen
Marcus Clauss
Matthias Clauss
Sebastian Clauss
Eva Clausson
Daniel Clauw
Thomas Clavel
Christine Clavel
Antonio Clavenna
Gary Clawson
David Claxton
Zanna Clay
Julie Claycomb
Jonathan Clayden
John Claydon
Pascal Clayette
Els Clays
Christine Clayton
Richard Clayton
Sue Clayton
Daniel Cleary
Ondine Cleaver
C. Clech
Susanne Clee
Peter Clegg
Deborah Clegg
John Cleland
Elsa Cleland
Timothy Cleland
Catherine Clelland
Rollie Clem
Kelly Clemens
Stephan Clemens
Mark Clemens
Christophe Clement
Charles Clement
Wendy Clement
Matthew Clement
Jade Clement
Christian Clement
Yves Clément
Alberto Clemente
Cj Clemente
Diego Clemente
Vicente Clemente-Suárez
Kendall Clements
David Clements
Gopalasamy Reuben Clements
Mark Clements
Nicholas Clements
Mark Clementz

Philippe Clerc
Aldo Clerico
Gilles Clermont
Olivier Clermont
Beth Cleveland
David Clewett
Geremy Cliff
Martin Clift
Eric Clifton
Kenneth Clifton
Andreu Climent
Jason Climer
Hollis Cline
Kenneth Cline
Mark Cline
Christopher Cline
Gisele Clofent-Sanchez
Marylene Cloitre
Samuel Clokie
Alex Clop
Gerald Clore
Daniel Closa
Jacqueline Close
Shane Close
Sophie Clot
Peter Clote
Brian Clough
Steve Clouse
Sean Clouston
Jasmin Cloutier
Gavin Clowry
Megan Clowse
Robert Clubb
Tyler Cluff
Mt Clunes
William Clusin
Hans Clusmann
Avner Cnaani
Fokie Cnossen
Sean Coady
Cristian Coarfa
Ernestina Coast
Juliet Coates
Daniel Coates
Joel Coats
Brittany Coats
Neslihan Coban
Oksana Coban
Aurélie Cobat
Fernanda Cobayashi
George Cobb
Stuart Cobb
Jason Cobb
Cory L. Cobb
Mia Cobb
Stephen Cobbold
Elroy Cober
Kelly Cobey
Steve Cobley
Teresa Cobo
Enrique Cobos Del Moral
Steven Coca
Emil Coccaro
Eliana Coccia
Lucio Cocco
Federico Coccolini
Flavio Coceani
Clement Cochain
Hervé Cochard
Stephen Cochi
Brent Cochran
Amy Cochran
Ethan Cochrane
Silvia Cocito
J. Mark Cock
Ian Cockburn
Lynn Cockburn
Gillian Cockerill
John Cockrem
Amanda Cockshutt
Paul Cockwell
Claudio Cocozza
Claudia Cocozza
Rachel Codd
Brian Codding
Claudia Codeço
Muhammed Yasin Çodur
Dianna Cody
Marco Coelho
Eduardo Coelho
Miguel Coelho
Luis Coelho
Francisco Coelho
Lara Coelho
Lia Coelho
Silvia Patrícia Coelho

Rui Coelho
Manuel Coelho-E-Silva
Yann Coello
C. Coello
Jessica Coertse
Jenny Coetzee
William Coetzee
Theresa Coetzer
Willem Coetzer
Michael Coeurdassier
Remy Coeytaux
Emily Coffey
Sean Coffey
Patricia Coffey
Allison Coffin
James Coffman
Clark Coffman
Donna Coffman
L. Eduardo Cofré Lizama
Jay Coggan
Craig Cogger
David Coggon
Anna Coghill
Brett Coghlan
Massimo Cogliati
Anthony Cognato
Michel Cogne
Paola Cogo
Angel Cogolludo
Karina Cogo-Müller
Mary Cogswell
Aurélie Cohas
Lauren Cohee
Myron Cohen
Jerry Cohen
Mark Cohen
Jacqueline Cohen
Henri Cohen
Mitchell Cohen
Aaron Cohen
Joachim Cohen
Anne Cohen
Robert Cohen
Jessica Cohen
Yigal Cohen
Jonathan Cohen
Matt Cohen
Adam Cohen
Trevor Cohen
Jeremy Cohen
Lawrence Cohen
Chad Cohen
Susan Cohen
Ethan Cohen
Andrew Cohen
Deborag Cohen
Emily Cohen
Joel Cohen
A. D. Cohen
Jacqueline M Cohen
Helen S Cohen
Julien Cohen-Adad
Mirri Cohen-Cinder
Devora Cohen-Karni
Martine Cohen-Salmon
Karine Cohen-Solal
Douglas Cohn
Avery Cohn
Michael Cohn
Randall Cohrs
Valentina Coia
Elaine Coimbra
Lachlan Coin
Frederic Coin
Alessandra Coin
Catherine Coirault
Annibale Cois
Ana Coito
Darren Coker
Ben Colagiuri
Tarah Colaizy
Yasar Colak
Vittorio Colantuoni
Antonio Colantuoni
Antonio Colaprico
Claudia Colasante
Stefano Colazza
Francesca Colazzo
Sheri R. Colberg
Matthew Colbert
Robert Colbert
Tim Colbourn
Fred Colbourne
Robb Colbrunn
Geoffrey Colby
Donn Colby

Fernando Colchero
Francesca Colciaghi
Kelly Cole
Nelson Cole
James Cole
Victoria Cole
Marsha Cole
John Cole
Jonathan J. Cole
Matthew Cole
Michael Cole
Joanne Cole
Richard Cole
Tim Cole
Laura Cole
Maurizio Colecchia
Kathleen Colegrove
Pasqualina Colella
Raymond Colello
William Coleman
Annette Coleman
Jamie Coleman
Harold Coleman
David Coleman
Michael Coleman
Joanna Coleman
Maureen Coleman
Brenda Coleman
Jason Coleman
Craig Coleman
Rivka Colen
Claudia Colesie
Alexander Colevas
Lissy Coley
Phyllis Coley
Carol Colfer
Michelle Colgrave
John Colicelli
Alain Colige
Thierry Colin
Herve Colinet
Olympia Colizoli
Marta Coll
Mar Coll
Maria Carmen Collado
Jean-Francois Collard
Rose Collard
Licio Collavin
James F. Collawn
Andres Collazo
Jérôme Collemare
Jonas Collén
Bertrand Collet
Tom Collett
Helen Colley
Licia Colli
Leandro Colli
Rebecca Collie
Nigel Collier
Jason Collier
Scott Collier
Melissa Collier
Quinten Collier
Ann Collier
Peter Collignon
Jerome Collignon
E. Collin
Francesca Collina
William Collinge
Maddalena Collini
Federica Collino
James Collins
Scott Collins
Richard Collins
Brett Collins
David Collins
Steven John Collins
Colm Collins
Andrew Collins
Sheila Collins
Marissa Collins
Natalie Collins
Jeannie Collins
Catherine Collins
Michael Collins
Adam Collison
Luana Colloca
Martine Collumbien
Andrew Colman
Ian Colman
Brian Colman
Ricki Colman
Juan D Colmenero
Luiz Colnago
John Cologne
Danny Colombara

Raffaella Colombatti
Sophie Colombié
Giorgio Colombo
Arnaldo Colombo
Gualtiero Colombo
Barbara Colombo
Dolors Colomer
Mª Àngels Colomer
Vincenza Colonna
Giuliano Colosimo
Claudio Colosio
Jan Colpaert
Tonya Colpitts
Gabriela Colpo
Ian Colrain
Benoit Colsh
Philippe Colson
Serge Colson
Natalie Colson
Brett Colson
Veronika Coltheart
Rocchina Colucci
Antonio Coluccia
Luca Colucci-D'Amato
Amedeo Columbano
Ivan Coluzza
Louise Colville
Jonathan Colville
Christopher Colvin
Charlotte Colvin
Christopher Colwell
Frederick Colwell
Edoardo Colzani
Lorenza Colzato
Maria Comănescu
Diana Coman
Manola Comar
Iñaki Comas
Merce Comas
Erika Comasco
Christophe Combadière
Yves Combarnous
Christian Combe
Valery Combes
Jean-Damien Combes
Christophe Combet
Emilie Combet Aspray
David Combosch
Colin Combs
Etienne Côme
André Comeau
Jeannette Comeau
Aaron Comeault
Alejandro Comellas
Diego Comerci
Alison Comfort
Hannie Comijs
Clarissa Comim
Begoña Comin-Anduix
Elizabeth Comino
Tara Cominski
Francesco Comiti
John Commito
Paolo Comoglio
Fabrice Compain
Stéphane Compant
Angelo Compare
Leonida Compostella
Laurie Comstock
Philippe Comtois
Gavin Conant
Gleice Conceição
Catarina Conceição
Teresa Conceição
Waldo Concepcion
Luis Concha
Javier Conde
Alberto Conde
Carlos Conde
Artur Conde
Carlos Conde-Glez
Antonio Condino-Neto
Richard Condit
Jennifer Condon
Tony Condon
Paul Condon
Catriona Condon
Gianluigi Condorelli
Annie Conery
Christine Conesa
Massimo Conese
Viktoriya Coneva
Fabrice Confalonieri
Laura Conforti
Feng Cong
Yingzi Cong

Le Cong
Yang Cong
Hengjiang Cong
Gao Cong
Ling Cong
Fengyu Cong
Nathan Congdon
Scott Conger
Davide Conigliaro
Douglas Conklin
Daniel Conklin
Andrew Conlan
Wayne Conlan
Daniel Conley
Terri Conley
Frank Conlon
Thomas Conlon
Beth A. Conlon
Michael Conlon
J. Michael Conlon
Jan Conn
David Conn
Paul Conn
Kristen Conn
Ian R Connell
Kim Connelly
John Connelly
Shane Connelly
William Conner
Sean Conner
Caitlin Conner
Tamlin Conner
Joann Conner
Alana Conner
Alex Conner
M Conner
Bradley Conner
David Conner
Philippe Connes
Grant Connette
Mark Connick
Anne Connolly
Jennifer Connolly
Eric Connolly
Darragh Connolly
Brian Connolly
Andrew Connolly
E. Sander Connolly Jr.
James Connor
Jason Connor
Barry Connors
Geoffrey Connors
Matthew Conomos
Cheryl Conover
Susan Conrad
Jacinta Conrad
Donald Conrad
Jack Conrad
Henk Jan Conradi
John Conran
Nicola Conran
Paul Conroy
Simon Conroy
Nathan Consedine
Robert Considine
Pierpaolo Consoli
Fernando Cônsoli
Filippo Consolo
Roberto Consonni
Christos Consoulas
Paul Constable
Gabriela Constantin
Norman Constantine
Stefan Constantinescu
Cris Constantinescu
Christos Constantinou
Annamaria Conte
Caterina Conte
Luciano Conti
Bruno Conti
Fiorenzo Conti
Alfredo Conti
Heather Conti
Alana Conti
Michele Conti
Vincent Conticello
Silvestro Conticello
Davide Contini
Cristina Contreras
Lydia Contreras
Marco Contreras
Rubén Contreras Patiño
Carlos Contreras-Martel
Bruno Contreras-Moreira
Jose Luis Contreras-Vidal
Alexander Converse

Matteo Convertino
Andrew Conway
Bernard Conway
Brian Conway
James Conway
Bryan Conway
Damian Conway
Jessica Conway
Richard Conway
F. Conway
Andrew Conway Morris
Constance Coogle
Nancy Cook
Steven Cook
Erik Cook
Jackie Cook
Katherine Cook
Katrina Cook
Jeanette Cook
Richard Cook
Mark Cook
Audrey Cook
Gary Cook
John Cook
Linda Cook
Brian Cook
Daniel Cook
Douglas Cook
Steven Cooke
Graham Cooke
Marcus Cooke
Gerard Cooke
Julia Cooke
Paul Cooke
Brian Cooke
Jessica Cooke Bailey
Mark Cookson
Adrian Cookson
Sarah Jane Cookson
Marcel Coolen
Marco Coolen
Lynn Cooley
Toon Cools
Pieter Cools
Joanne Coombe
Brenda Coomber
Jason Coombes
Kevin Coombs
Jeff Coombs
Steve Coombs
David Coomes
Elizabeth Coon
Greg Cooney
Austin Cooney
David Cooper
Ben Cooper
Curtis Cooper
Philip Cooper
Katrina Cooper
Vaughn Cooper
Jonathan Cooper
Geoffrey Cooper
Tim Cooper
Melanie Cooper
Di Cooper
Chris Cooper
Nigel Cooper
Lee Cooper
Ellis Cooper
Matthew Cooper
Sandra Cooper
Trevor Cooper
Edward Cooper
Caren Cooper
Scott Cooper
Crystal Cooper
Jon Cooper
Richard Cooper
Kerry Cooper
Christopher Cooper
Ziva Cooper
Rachel Cooper
Lauren Cooper
Seth Cooper
Christine Cooper-Vince
Gerald Cooray
Emily Cope
William Copeland
William Copen
Michael Copenhaver
Massimiliano Copetti
Mhairi Copland
Lawson Copley
Alessandro Coppe
Fabio Coppedè
Isabelle Coppens

Ken Coppieters
Geraldine Coppin
Helene Coppin
Raffaele Coppini
Paul Coppo
Mauricio Coppo
Amy Coppock
Vincenzo Coppola
Roberto Coppola
Gianluca Coppola
T. Coppus
Leslie Copstein
Jeremy Coquart
Hélène Coqueugniot
Davide Cora
Karunakaran Coral
Nadia Corazza
Lanfranco Corazzi
Vincent Corbel
John Corbett
Jennifer Corbett
Laura Corbett
Amanda Corbett
Jo Corbett
Russell Corbett-Detig
Nicoletta Corbi
Marine Corbin
William Corbin
Vincenzo Corbo
Massimo Corbo
Vanessa Corby-Harris
Angela Corcelli
Lynn Corcoran
Jacob Corcoran
Leyre Corcuera
Anna Cordeiro
Hugues Cordel
Pierre Cordelier
Heather Cordell
Barbara Cordella
Mathilde Cordellier
Guilherme Cordenonsi Da Fonseca
Adolfo Cordero
Oscar Cordero
Erik Cordes
Sara Cordes
Martine Cordier-Bussat
Ricardo Cordioli
Miguel Cordoba
Sarai Córdoba
Jose Cordoba-Chacon
Giada Cordoni
Adriana Cordova
Luis Cordova
Ana Lucia Cordova-Kreylos
Alana Cordy-Collins
Esther Coren
Lawrence Corey
Seth Corey
Eva Corey
Ian Corfe
Anthony Corfield
Carlos Coriano
Marie-France Corio-Costet
H. Corke
Barbara Corkey
Alexandru Corlan
Luca Corlatti
Scott Corlew
Anne Corlu
Canan Corlu
Lawrence Cormack
Estelle Cormet-Boyaka
Stephania Cormier
Vernon Cormier
Luigi Cormio
David Cormode
Jacob Corn
Markus Cornberg
David Cornblath
Divi Cornec
Amy Corneli
Tom Cornelis
Pierre Cornelis
Dawn Cornelison
Christopher Cornelison
Bart Cornelissen
Tatiana Cornelissen
Gert Cornelissen
Laura Cornelissen
Jan Cornelius
Cintia Cornelius
Sean Cornelius
Rosemary Cornell
Robert Cornell
Stephen Cornell

Sarah Cornell
Morna Cornell
Bruce Cornell
Oliver Cornely
Leigh Corner
François Cornet
Raphael Cornette
David Corney
Nancy Cornick
Marc-Andre Cornier
Emmanuel Cornillot
Astrid Cornils
Jillian Cornish
Bruce Cornish
Jennifer Cornish
Robert Cornman
Gianluca Corno
Tom Cornsweet
Gail Cornwall
Christopher Cornwall
Julien Corny
Miguel Corona
Benjamin Corona
Ligiana Corona
Ismael Coronado
Minas Coroneo
Claudia Coronnello
Armelle Corpet
Maripat Corr
Susan Corr
Massimo Corradi
Marco Corradi
Javier Corral
Jesus Corral
Mi Corral-Baqués
Vicente Corrales-Medina
Giovanni Corrao
Marife Corre
Pelayo Correa
Ricardo Correa
Isabel Correa
Regis Correa
Bendito Correa
Margarita Correa
Rodrigo Correa
Daniel Corrêa
Paulo Corrêa
Thiago Corrêa
Alberto Corrêa
Vania Maria Correa Da Costa
Jose Correa-Basurto
Ricardo Correa-Rotter
Maria Almira Correia
Marta Correia
Teresa Correia
João Correia
Ilídio Correia
Antonio Correia
Cristina Correia
Rion Correia
Paulo Correia-De-Sá
Margarida Correia-Neves
Xavier Correig
Joshua Correll
Marialaura Corrente
Jesus Corria Osorio
Laura Corrigan
Kathleen Corriveau
Sherryse Corrow
David Corry
Robert Corry
Maria Michela Corsaro
Ilaria Corsi
Angelo Corsico
Alberto Corsini
Gaetano Corso
Stephen Corson
Timothy Corson
Paul Corstjens
Oscar Cortadellas
Andrea Cortegiani
Gabriella Cortellessa
Pietro Cortelli
Adrian Cortes
Jorge Cortés
Andrés Cortés
Miriam Cortese-Krott
Pa Cortesi
Fabio Cortesi
Bernard Cortet
Mauro Cortez
Melissa Cortez
Helena Cortez-Pinto
Donnamaria Cortezzo
Blaise Corthesy
Claudia Corti

Monica Cortinovis
Cristina Cortis
Pierluigi Cortis
P. Cortoos
Gino Cortopassi
Ronald Cortright
Curtis Corum
Jenny Cory
Gerardo Corzo
Ilaria Coscia
Michele Coscia
Mireia Coscolla
Carlo Cosentino
Jose Cosgaya
Lisa Cosgrove
Gregory Cosgrove
Antonio Cosma
Benilde Cosmi
Camila Cosmo
Omar Coso
Pascale Cossart
Jack-Christophe Cossec
Stephanie Cossette
Angelia Cost
Jose Costa
Manuel Costa
Daniel Costa
José Costa
Max Costa
Joana Costa
Maria Do Carmo Costa
Rodrigo Costa
Luis Costa
Filipe Costa
Tommaso Costa
Albert Costa
Marcio Costa
Kevin Costa
Patrício Costa
Susana Costa
José Hélio Costa
Helio Costa
Felipe Costa
Marcos Costa
Angelo Costa
M. Costa
Barbara Costa
Corrado Costa
Maria Cecilia Costa
Gabriel Costa
Aida Costa
Elisiio Costa
Cristina Costa
Danielle De Souza Costa
F. Costa
Frederico Costa
Roberto Costa
Blaise Costa
Catarina Costa
Taciana Costa Dias
Julia Costa-Cruz
Ciro Costagliola
David Costalago
Ef Costalonga
Leticia Costa-Lotufo
Claudio Costa-Neto
Carlo Costantini
Marcello Costantini
Maria Costantini
Federica Costantini
Todd Costantini
Laura Costantini
David Costantini
Antonio Costanzo
Katie Costanzo
Paola Costanzo
Sandra Costanzo
Pierluigi Costanzo
Jon Costanzo
João Costa-Rodrigues
Rodrigo Costas Comesaña
Sébastien Costedoat
Paola Costelli
Eithne Costello
Derek Costello
Michael Costello
Sylvain Costes
Craig Costion
Xurxo Costoya
Ernesto Cota
Vinicius Cota
Douglas Cotanche
Kimberly Cote
Michele Cote
Gilbert Cote
Rick Cote

Julien Cote
Helene Cote
Julie Cote
Michel Cote
Isabelle Côté
Patrice Côté
E. Cothran
Ioana Cotlarciuc
Sehoya Cotner
Antonella Cotoia
Ana Paola Cotrim
George Cotsarelis
Menino O. Cotta
Michiel Cottaar
Charles Cotten
Karl Cottenie
Joshua Cotter
Fenton Cotterill
Luis Cottet
Fabien Cottier
Clémentine Cottineau
Theresa Cotton
Elizabeth Cottrell
Stuart Cottrell
Maria Luz Couce
Thérèse Couderc
Jerome Coudert
Amelie Coudert
Steven Coughlin
Isabelle Couillin
Carolyn Coulam
Jennifer Coull
Pierre Coulombe
Jasmin Coulombe-Huntington
Aurélie Coulon
Marjorie Coulon
Graeme Coulson
Yvette Coulson-Thomas
Leigh Coultas
Alison Coulter
David Coulter
Sarah Coulthurst
Simon Coulton
Pascal Coumailleau
Dim Coumou
Lou Counil
Serena Counsell
Christopher Counter
Brian Counterman
Christopher Counts
Bérengère Coupé
Kevin Couper
David Couper
Julien Courchet
Jannelle Couret
Mathieu Coureuil
Laurent Cournac
Isabelle Cournu-Rebeix
Donald Court
François Courtemanche
Cari Courtenay-Quirk
Jason Courter
Gregoire Courtine
Susan Courtney
Cornelius Courts
Denis Cousineau
Scott Cousins
Francoise Coussen-Choquet
Paul Coussens
Anna Coussens
Christine Coustau
Sarah Cousty
Marc Coutanche
Régis Coutant
Sheryl Coutermarsh-Ott
Pedro Coutinho
Patrícia Coutinho
Luiz Coutinho
Artur Coutinho
Marcos Coutinho Schechter
Francisco Couto
Isabel Couto
Anna Coutsoudis
Philippe Couttet
Aaron Coutts-Smith
Daniel Coutu
Réjean Couture
Christine Couturier
Lydie Couturier
Eduardo Couve
David Couvin
Alain Couvineau
Olivier Coux
Alejandra Covarrubias
Manuel Covarrubias
Mihai Covasa

Anabella Covazzi Harriague
Maureen Covelli
Peter Coventry
Domenico Coviello
Kristen Covino
Thiago Covoes
Luciene Covolan
Douglas Cowan
Nelson Cowan
Frances Cowan
Ann Cowan
James Cowan
Don Cowan
Lauren Cowan
Dan Cowan
Juthaporn Cowan
Charles Cowan
Robert Cowan
Ronald Cowan
Kiriana Cowansage
Ashley Cowart
Beverly Cowart
Charles Cowden
Rita Cowell
Leah Cowen
Eileen Cowey
Robert Cowie
Dorothy Cowie
Julie Cowie
Nathan Cowieson
Chris Cowled
Jane Cowles
Siobhan Cowley
David Cowley
Elizabeth Cowley
Daniel Cowley
Benjamin Cowling
Victoria Cowling
Wallace Cowling
Michael Cox
Jonathan Cox
Gregory Cox
Gary Cox
Adrienne Cox
Timothy Cox
Brian Cox
Phillip Cox
Lynne Cox
Stephen Cox
Eileen Cox
James Cox
Anthony Cox
Dianne Cox
Andrew Cox
John Cox
Dennis Cox
Marc Cox
Samantha Cox
Stephanie Cox
Tarnya Cox
Rachel Cox
Carmen Coxon
James Coxon
Janet Cox-Singh
Pilar Coy
Ericsson Coy-Barrera
Douglas Coyle
Vernon Coyne
Mark Coyne
Daniel Coyne
James Coyne
Melanie Cozad
Wendy Cozen
Yvette Cozier
Mario Cozolino
Emanuele Cozzani
Domenico Cozzetto
Luca Cozzi
Renata Cozzi
Alessandro Cozzi-Lepri
Daniel Cozzolino
Brendan Crabb
Bryan Crable
Margaux Crabtree
Jeffrey Crabtree
Matt Craddock
Jason Craggs
Jordan Crago
Aimee Crago
Jeffrey Craig
James Craig
Oliver Craig
Dawn Craig
Justin Craig
Ian Craig
Paul Craig

Robert Craigie
Charles Craik
Gordon Cramb
Gilles Crambert
Michael Cramer
Grant Cramer
Claudia Cramer
Joel Cramer
Amelia Crampin
Ian Crandall
David Crandall
Brian Crane
Paul Crane
Benjamin Crane
Andrew Crane
Nicole Crane
Laura Crane
Melissa Crane
John Crane
Arryn Craney
Colleen Crangle
John Cranmer
Peter Cranston
Alexandro C. W. Craparo
Corentin Cras-Méneur
Peter Crauwels
Benjamin Cravatt
Dylan Craven
Pedro Cravo
Robert Crawford
Bryan Crawford
Gary Crawford
Kerri Crawford
Dolly Crawford
D Crawford-Brown
Angela Crawley
Francis Crawley
Antonio Craxì
James Cray
Carolyn Cray
Roman Crazzolara
Pasquale Crea
Franceso Crea
Teresa Crease
Robert Creath
Carmine Crecchio
Daniel Credeur
Ian Cree
Lynsey Cree
Douglas Creedon
Scott Creel
Sarah Creel
John Creemers
Christopher Creevey
Mathew Creighton
Pau Creixell
Enrico Crema
Lazaro Cremades
Julie Cremeans-Smith
Marcel Cremene
Thomas Cremer
Pascal Crenn
Thomas Crenshaw
Edward Crenshaw
N. Crepaz
Franci Crepeau-Hobson
Valérie Crepel
Elvira Crescenzi
Silvia Crescioli
Mabel Crescioni
Emmanuele Crespan
Gonzalo Crespo
Isaac Crespo
Andre Crespo
Jesus Crespo Cuaresma
Anne Cress
Peter Cressey
Erin Cressman
John Cressman
Will Cresswell
James Cresswell
Bruno Crestani
Maurizio Crestani
Carlos Crestani
Fabio Cresto Aleina
Kasey Creswell
Patrice Crété
Benjamin Cretin
Carole Creuzenet
Frédéric Crevecoeur
Gilles Crevel
Richard Crevenna
Tara Crewe
Douglas Crews
Sarah Crews
Ernesto Criado-Hidalgo

Ivor Cribben
Matthew Cribbet
Neil Crickmore
Krista Crider
Theresa Crimmins
Gerard Criner
Lucio Crino
Swee Cripe
Kent Crippen
Richard Cripps
Mihaela Crisan
Charles Criscione
Michael Criscitiello
Samuel Crish
Pierre-Alex Crisinel
Catherine Crisp
Paul Crispen
Stefania Crispi
Daisy Crispim
Sandra Crispim
Max Crispin
Alison Criss
Thomas Crist
Antonia Cristaudo
Ioana Cristea
Matthieu Cristelli
Bogdan Cristescu
Viviane Cristiano
Xavier Cristina
Vittorio Cristini
Luigia Cristino
Gael Cristofari
Vivian Cristofaro
Luca Cristofolini
David Criswell
Hugo Critchley
Christine Critchley
Alexander Crits-Christoph
Elena Critselis
Alyssa Crittenden
Federica Crivellaro
Lorenzo Crocco
Fabio Crocetta
Melissa Crocker
Julie Crockett
Belinda Crockett
Julio Croda
Tatjana Croenlein
Kevin Croft
James Croft
Jean-Claude Croizet
Timothy Croley
Roger Croll
Erica Crome
Deborah Cromer
Walter Cromer
Robin Crompton
Randy Cron
Joakim Crona
John Cronan
Matty Crone
Candace Croney
Colleen Croniger
Tom Cronin
Adam Cronin
Matthew Cronin
Robert Cronin
Mark Cronin
Bruce N. Cronstein
Darren Crook
Helen Crooke
Daniel Crooks
Casey Crooks
Lizzie Croose
Jennifer Cropley
Vanessa Cropley
Cheryl Cropp
Simon Cropper
Rachelle Crosbie
Alfred Crosby
Vincent Croset
Paolo Crosetto
Adrienne Crosier
Patrick Croskerry
Paul Cross
Janet Cross
Troy Cross
Timothy Cross
Benjamin Cross
Megan Cross
David Crossman
Lisa Crossman
Sean Crosson
Damien Croteau-Chonka
Jimmy Crott
Elena Crotti

Felicity Crotty
Peter Crouch
Nicholas Croucher
Adrian Croucher
Pedro Crous
Marta Crous-Bou
William Crow
Sidney Crow
David Crowder
Suzanne Crowe
Francesca Crowe
Andrew Crowe
Brenda Crowe
Trevor Crowell
Andrew Crowl
William Crowley
Thomas Crowley
David Crowley
Michael Crowley
Kevin Crowston
Damian Crowther
Mathew Crowther
Paula Croxson
Ilona Croy
Anne Croy
Bertrand Crozatier
Ottavio Croze
Sarah Crozier
Giorgio Cruccu
Aline Crucello
Pablo Cruces
Brian Crucian
Céline Cruciani-Guglielmacci
Alan Cruess
Rob Cruickshank
Nancy Crum-Cianflone
Byron Crump
Clyde Crumpacker
Jan Crusius
Rik Crutzen
Elaine Cruz
Celia Cruz
Antonio Cruz
Miguel Cruz
Dianne Cruz
Fernanda Cruz
Vinicius Cruzat
Andreas Cruz-Herranz
Monica Cruz-Lemini
Rogelio Cruz-Martinez
Pablo Cruz-Morales
Ariovaldo Cruz-Neto
Lillian Cruz-Orengo
Luis Cruz-Vera
Paul Cryan
Colin Cryer
Miklos Csala
László Csanády
Robert Csapo
Péter Csermely
Laszlo Csernoch
E. Csernok
Anita Cservenka
Gabor Csifcsak
Julia Csikar
Balazs Csoka
Csaba Csontos
Sándor Csosz
Gabor Csucs
María Cuacos
Diego Cuadros
Jaime Cubero
Francisco Javier Cubero
Cankut Cubuk
Brett Cucchiara
Francis Cucinotta
Domenico Cucinotta
H. S. Cuckle
Andrea Cuconati
Phillip Cuculich
Carla Cuda
Suresh Cuddapah
Will Cuddy
Predrag Cudic
Mauricio Cuello
Nicolás Cuenca
Magdalena Cuenca-Garcia
Valentin Cuervas-Mons
Ana Cuervo
Luis Gabriel Cuervo
Marta Cuervo
Henar Cuervo
Miguel Cuesta
Eduardo Cuesta
Marc Cuesta
Isabel Cuesta

Pascale Cuet
Ramon Cueto
Victor Cueto
Carlos Cueva
E. Cuevas
Kimberly Cuevas
Maria Cuevas
Katarina Cufar
Andrew Cuff
Marc Cuggia
Massimo Cugno
Yan Cui
Liwang Cui
Yuehua Cui
Qinghua Cui
Juan Cui
Fang Cui
Hongchang Cui
Jianmin Cui
Xiaoyong Cui
Zongbin Cui
Yuhai Cui
Jin-Jie Cui
Zhaoxia Cui
Hengmi Cui
Taixing Cui
Guohong Cui
Long Cui
Zhenling Cui
Bianxiao Cui
Rutao Cui
Xiang-Shun Cui
Peixin Cui
Jiwei Cui
Ziyou Cui
Jinhui Cui
Gaochao Cui
Jixin Cui
Wei Cui
Yibin Cui
Yuqi Cui
Julia Cui
Mei-Zhen Cui
Xinyi Cui
Jiujie Cui
Dangqun Cui
Ruqiang Cui
Weiguo Cui
Baoshan Cui
Xiaobing Cui
Pim Cuijpers
Iva Cukic
Judith Cukor
Jennifer Culbertson
Heather Culbertson
Jennifer Culhane
Ognjen Culic
Stéphane Cullati
Bryan Cullen
Kathleen Cullen
Mark Cullen
Richard Culleton
Dominik Cullmann
Maxime Culot
Valeria Culotta
Patricia Culp
Thibaut Culty
Dave Culver
Kelly Culwell
Neil Cumberlidge
Andrea Cumino
David Cummiing
Sean Cumming
David Cumming
Geoff Cumming
Richard Cummings
Brian Cummings
Kevin Cummings
Michael Cummings
Eoin Cummins
Philip Cummins
David Cundiff
Rodrigo Cunha
Alexandre Cunha
Marina Cunha
Cristina Cunha
Angela Cunha
Thiago Cunha
Fernanda Cunha
Carla Cunha
Eugénia Cunha
Dênis Cunha
Diogo Cunha
Nicolay Cunha
Dayse Cunha
Joana Cunha-Cruz

João Sabino Cunha-Filho
Teresa Cunha-Oliveira
Toni Cunillera
Stephen Cunnane
Patrick Cunningham
Mark Cunningham
Lisa Cunningham
Mark O. Cunningham
Melissa Cunningham
Adam Cunningham
John A Cunningham
George Cunningham
Christopher Cunningham
Michael Cunningham
Timothy Cunningham
Fraser Cunningham
Charlotte Cunningham-Rundles
Aubrey Cunnington
Vincenzo Cunsolo
Gilles Cuny
Ornella Cuomo
Gerald Cupchik
Andrea Cupp
Lilian Cuppari
Michele Curatolo
Antonietta Curci
John Curci
Branislava Curcic-Blake
Christine Curcio
Giuseppe Curcio
Giuseppe Curcurù
Giulia Curia
Vladimir Curic
Giuseppe Curigliano
Ino Curik
Millaray Curilem
James Curley
Chester Curme
Paul Curmi
Antonio Currà
Sean Curran
Jerry Curran
Joseph Curran
Sara Curran
Sabrina Curreli
John D. Currey
Phillip Currie
Kevin Currie
Craig Currie
David Currie
Dianne Currier
Gemma Currrie
Fitz-Roy Curry
Stephen Curry
Silvia Curteanu
Stefan Curth
Jeffrey Curtis
Val Curtis
Nigel Curtis
Lisa Curtis
Kevin Curtis
Patrick Curtis
Abigail Curtis
Maggie Cusack
Jeremy Cusack
Sarah Cusick
John Cusick
Christine Cuskley
Chrissy Cuskley
Cesare Cuspidi
Victoria Cussen
Michel Cusson
Deborah Custance
James Custis
Anna Custo
Ceci Castilho Custódio
Ana Cusumano
Hattie Cutcliffe
Innes Cuthill
Luisa Cutillo
Mary Lou Cutler
Chris Cutler
Paul Cutrufello
Gary Cutter
Michael Cutter
Michael Cuttica
Frank Cuttitta
Ann Cuypers
Jack Cuzick
Verginia Cuzon Carlson
Maria Cvach
Erin Cvejic
Ales Cvekl
James Cybulski
Melissa Cyders
Miroslaw Cygler

Aaron Cypess
Douglas Cyr
Clemens Cyran
Jason Cyster
Marcus Czabanka
Tibor Czabany
Peter Czabotar
Tomer Czaczkes
Mark Czaja
K. Czaja
Laszlo Czako
Peter Czarnecki
Ewa Czarnobilska
Michael Czech
Christian Czech
Boldizsar Czeh
Elena Czeizler
Rakefet Czerninski
Marta Czerska
Mike Czervinske
Marcin Czerwinski
Sergiusz Czesny
Andras Czirok
Henryk Czosnek
Cezary Czosnek
Marek Czosnyka
Michael Czubryt
Urszula Czyzewska
Traci Czyzyk
Maria Czyzyk-Krzeska
Eric D’Hoore
Christophe D’Hulst
Matthias D’Huyvetter
Xavier D’Journo
G. D’Orta
Yuwei Da
Guillaume Da
Milton Da Costa
Gonçalo Da Costa
Bruno Da Costa
Luís Da Costa
Renata Da Costa
Ronaldo Da Costa
Sandra Da Costa
Ana Maria Da Costa Ferreira
Eduardo Da Cruz
Aline Da Silva
Wilmar Da Silva
Fernando Da Silva
Flavio Da Silva
Albérico Da Silva
Gabriela Jorge Da Silva
Rodrigo Da Silva
Henrique Da Silva
Weber Da Silva
Jane Da Silva
Aleksandro Da Silva
Antonio Da Silva Ferreira
Michelly Geórgia Da Silva Marinho
Itabajara Da Silva Vaz Jr
Gabriela Da Silva Xavier
Juliano Da Silveira
Hein Daanen
Sarah Dababnah
Ali Dabaja
Ali Dabbagh
Vahid Reza Dabbagh Kakhki
H Dabbah
Geoff Dabelko
Jacek Dabert
Neha Dabral
Andrei Dabravolski
Wojciech Dabrowski
Matthieu Dacher
Shimrit Daches
Jean Louis Dacheux
Jordi Dachs
R. J. Dachs
Armond Daci
Michelle Dacosta
Rosalia Dacosta Aguayo
Valerie D'Acremont
Matthew Dacso
Laura Dada
Gilles Dadaglio
Luciano D'Adamio
Marco Dadda
Beatriz Dáder
Abel Dadi
Harold Dadomo
Soheil Dadras
Arezoo Dadrasnia
Alessandro Daducci
Curtis Daehler
Dorothea Daentzer
Georg Daeschlein

Daniele Daffonchio
Katherine Dafforn
Sirlei Daffre
Arnon Dag
Ruben Dagda
Hanne Dagfinrud
Rada Dagher
Magda Bou Dagher Kharrat
Stefania Daghino
Mohsen Daghooghi
Maria Lucia Dagli
Lina Dagnino
Adan Dagnino-Acosta
Samuel Dagogo-Jack
Gregorio D'Agostino
Paul D'Agostino
Harel Dahari
Silvia Daher
Elizabeth Daher
Neetu Dahiya
Kris Dahl
Geoffrey Dahl
Edgar Dahl
Morten Dahl
John Dahl
Richard Dahl
Espen Dahl
Jordi Dahl
Jeremy Dahl
Maria Dahle
Roland Dahlem
Thomas Dahlgren
Linda Dahlgren
Rainer Dahlhaus
Frank Dahlke
Kimberly Dahlman
Burkhardt Dahlmann
Franziska Dahlmann
Karin Dahlman-Wright
Christina Dahm
Souhayl Dahmani
Hans-Uwe Dahms
Nabila Dahodwala
Mohammad Dahrouj
Wei Dai
Chang-Feng Dai
Shao Dai
Qi Dai
Chia-Yen Dai
Zhengjia Dai
Jiapei Dai
Tianhong Dai
Dao-Qing Dai
Limin Dai
Caili Dai
Zhongquan Dai
Yue Dai
Huaping Dai
Zhen-Dong Dai
Silan Dai
Jian Dai
Shuan Dai
Zhiyu Dai
Ning Dai
Hong-Jie Dai
Zhi-Cong Dai
Yakang Dai
Haiming Dai
Dong Dai
Xin Dai
Hongji Dai
Andreas Daiber
Vincent Daien
Claire Daien
Stephen Daiger
Courtney Daigle
Remi Daigle
Harry Dailey
Hemant Daima
Lidia Daimiel-Ruiz
Makoto Daimon
Benjamin Dainat
Vidyadhar Daithankar
Dina Dajani
Gavin Daker-White
Helen Dakin
Vasilis Dakos
Gajalakshmi Dakshinamoorthy
Martina Dal Bello
Francesco Dal Grande
Sarang Dalal
Koustuv Dalal
Ashwin Dalal
Lokesh Dalasanur Nagaprashantha
Nejat Dalay
Vincent Dalbo

Paul Dalby
Philip Dale
Nicholas Dale
Russel Dale
Brian Dale
Ann Marie Dale
Adam Dale
Jonas Dalege
George Dalekos
Love Dalen
Jelle Dalenberg
Yuri D'Alessandra
Esther Dalfo
Olav Dalgard
Clifton Dalgard
Kenneth Dalgarno
Marianela Dalghi
Tina Dalianis
Nawar Dalila
Carole Dalin
Mohammad Reza Daliri
Deniz Dalkara
Riccardo Dalla Volta
Enrico Dall'Ara
Erica Dall'Armellina
Frederic Dalle
Stephane Dalle
Tobias Dallenga
Glenn Dallerac
Simon Dalley
Martin Dallimer
Joan Dallinga
Geesje Dallinga-Thie
Reinhard Dallinger
Tim Dallman
Robert Dallmann
Florence Dallo
Luigi Dall'Olmo
Eduardo Dalmarco
Marco D'Alonzo
Danilo Daloso
Maeli Dal-Pai-Silva
Daniel Dalquen
Rafael Dal-Ré
Brian Dalrymple
Véronique Dalstein
John Dalton
Craig Dalton
Patricio Dalton
Brian Dalton
Pranjali Dalvi
Manish Dalwani
Norelle Daly
Martina Daly
Ian Daly
Janis Daly
Barbara Daly
Daniel Daly
Seth Daly
Julie Dalziel
Kim Dalziel
Hans Dam
Dimitrios Damalas
Eswar Damaraju
Domenico D'Amario
Mahendra Damarla
Bruno Damásio
Renato Damatta
Jiri Damborsky
Michael D'Ambrosio
Stefano D'Amelio
Christopher Dames
Shale Dames
Nailê Damé-Teixeira
Christian Damgaard
Giovanna Damia
Fabrizio Damiano
Andreas Damianou
Simonetta D'Amico
Gennaro D'Amico
Dennis D'Amico
Platten Damien
Nadja Damij
Daniel Damineli
Dragan Damjanovic
Sashko Damjanovski
Karim Damji
Peter Damm
Oliver Damm
Olaf Dammann
Markus Damme
Belle Damodara Shenoy
Murali Damodaran
Shima Damodaran
Chendil Damodaran
Bruce Damon

Scott Damon
Elsa Damonte
Fredrick Damron
Siriporn Damrongsakkul
Edward Damrose
Hancai Dan
Danhui Dan
Zhang Dan
Syamal Dana
Claude Danan
Siva Danaviah
Kelsey Dancause
David Dance
Antoine Danchin
Etienne Danchin
Nicolas Danchin
Andrew Dancis
James Danckert
Debashish Danda
Tong Dandan
Abhijit Dandapat
Collet Dandara
Prasad Dandawate
Thomas Dandekar
Samantha Dando
Luisa Dandolo
Lalit Dandona
Paresh Dandona
Marco Maria D'Andrea
Luca D'Andrea
Maura Dandri
Antonio D'Andrilli
Rickard Danell
Jenny Ann Danell
Christophe Danelon
Silvio Danese
Nazila Daneshjou
Roxana Daneshjou
Kaveh Daneshvar
Shaonong Dang
Weiwei Dang
Yan Dang
Zhi Dang
Rolando D'Angelillo
Maria Grazia D'Angelo
Daniela D'Angelo
Sara D'Angelo
Stefania D'Angelo
Lydia Danglot
Carlo Dani
Nika Danial
Heide-Marie Daniel
Potter Daniel
Mat Daniel
Jaiyanth Daniel
Catherine Daniel
Nathalie Daniel
Maria Giovanna Danieli
Alberto Danielli
Salvatore D'Aniello
Jane Daniels
Ranjit Daniels
Savel Daniels
Karen Daniels
Stephen Daniels
Mark Daniels
Anthony Daniels
Joseph Daniels
Troy Daniels
Amy Daniels
Rachel Daniels
Finn Danielsen
E. Michael Danielsen
Jared Danielson
Delia Danila
Valery Danilack
Laure Danilo
Sergei Danilov
Alexey Danilov
Tatiana Danilova
Elena Danilova
Maximilien Danisch
Patricia Dankers
Ellen Danneels
H. Kathleen Dannelly
Dirk Dannenberger
Antonella D'Anneo
Omar Danner
Udo Dannlowski
Ng Danny Wang-Kit
Davide Danovi
Judith Danovitch
Silvana Dans
Jan Danser
Mark Danta
Marcio Dantas

Ana Paula Dantas
Raymund Dantes
Anthony D'Antoni
Nico Dantuma
Francoise Dantzer
Benjamin Dantzer
Heather Danysh
Michael Danziger
Ming Dao
Georges Daoud
Ziad Daoud
Luciana D'Apice
Maria Rosaria D'Apice
Alexandros Daponte
Michael Dapp
Leonardo Dapporto
Elena Daprati
Masoud Dara
Mohammad Darainy
Shrinivas Darak
Behrooz Darbani
Dawood Darbar
Ian Darby
Manolo D'Arcangelo
Marie-Laure Darde
Hugues Dardente
Bernard Dardzinski
Mohammad Daremipouran
Helen Dargahi
Arnaud D'Argembeau
Giuseppe D'Argenio
Corinna Darian-Smith
Marco D'Arienzo
Kristin Darin
A. B. Dario
Rakus Dariusz
Chris Darke
Charles Darkoh
Tristan Darland
Victor Darley-Usmar
Eric Darling
Meghan Darling-White
Nissar Darmani
Antonella D'Arminio Monforte
Nicole Darmon
Sally Darney
Blair Darney
Gerald Darnis
Amir Darooneh
Salvatore D'Arpa
Chantelle D'Arquenne
Sébastien Darras
Guillaume Darrasse-Jeze
Christelle Darrieutort-Lafitte
Michelle Darrieux
Majid Darroudi
Amelia Darrouzet-Nardi
Véronique Dartois
Darlene Dartt
Brian Darvell
William Darwall
Ara Darzi
Aparup Das
Undurti Das
Atze Das
Arup Das
Sulagna Das
Kalyan Das
Tirtha Das
Amit Das
Sabyasachi Das
Santasabuj Das
Arundhati Das
Anath Das
Bhudev Das
Kiranmoy Das
Theerthankar Das
Utpal Das
Sarit Das
Surajit Das
Sushmita Das
Srijit Das
Payel Das
Shamik Das
Parimal Das
Prasanta Das
Raj Das
Subhasis Das
Arupratan Das
Bibhu Das
Bhaskar Das
Vaskar Das
Akash Das
Chittaranjan Das
Marco Das
Samar Das

Amitava Das
Samir Das
T. P. Das
Subbha Das
Jayashankar Das
Saumya Das
Ashok Kumar Das
Archita Das
Alok Das
Sourav Das
Subir Das
Dibash Das
Nibaran Das
Sujatha Das Gollapalli
Sujoy Das Gupta
José Das Neves
Krishna Das Saha
Srinivasan Dasarathy
Venkata Ramesh Dasari
Bhanu Dasari
Nathan Dascal
Fabrizio D'Ascenzo
Gregory Dasch
Pierre Dasen
Piyali Dasgupta
Partha Dasgupta
Subhamoy Dasgupta
Biplab Dasgupta
Indranil Dasgupta
Santanu Dasgupta
Subhajit Dasgupta
Srikanta Dash
Ranjan Dash
Rupesh Dash
Pradyot Dash
Paban Dash
Padmanava Dash
Ebony Dashiell-Aje
Luis Dasilva
Edson Dasilva
Silvia Da-Silva
Marina Daskalopoulou
Tim Daskivich
Maheshi Dassanayake
Paul Dassonville
Sana Dastgheyb
Mehran Dastmalchi
Daniel Datiko
Manuel Datiles
Kasturi Datta
Malabika Datta
Pratik Datta
Atin Datta
Nabanita Datta
Prasun Datta
Kamal Datta
Karabi Datta
Rhea Datta
Sandipan Datta
Arnab Datta
Alexandre N. Datta
Subhojit Datta
Esha Datta
Wesley Dáttilo
Raymond Dattwyler
Nicolas Dauchot
David Daude
Eric Daudé
Nicolas Daudet
Joseph Dauer
Alan Daugherty
Patrick Daugherty
Matthew Daugherty
John Daugirdas
Jean Daugrois
Robert Daum
Günther Daum
Martin Daumer
Yannicke Dauphin
Francois Dauphin
Marco Dauriz
Martin Daus
François Dautry
Yves Dauvilliers
Brigitte Dauwalder
Liliana Davalos
Joel Dave
Vrushank Dave
Maneesh Dave
Utpal Davé
Domenico D'Avella
Adam Davey
Gail Davey
Peter Davey
Olivier David
Wishart David

Stephen David
Jean-Pierre David
John David
Sascha David
Jacques David
Michael David
Laurent David
Valentin David
Robert David
Aaron David
Ray David
Matthias David
Alexandre David
Jenny David
Preiss David
Tamas David-Barrett
Sandra Davidge
Maria Davidich
A. M. Davidoff
Bill Davids
Alan Davidson
Andrew Davidson
Fordyce Davidson
Kathryn Davidson
Doug Davidson
Julie Davidson
Jacob Davidson
Steve P. Davidson
Judy Davie
Neil Davies
Jamie Davies
Kelvin Davies
Christopher Davies
William Davies
Simon Davies
Wayne Davies
Sarah Davies
Janet Davies
Jaime Davies
Toby Davies
Joanna Davies
David Davies
Nathaniel Davies
Andrew Davies
Nicholas Davies
Kate Davies
Barry Davies
Charlotte Davies
Claire Davies
Clive Davies
Jill Davies
Jaime Davila
Joana Da Costa D'Avila
José Dávila
David Dávila De León
Marina Davila Ross
Claudia D'Avila-Levy
Jose Davila-Velderrain
Salvatore Davino
William Davis
George Davis
Randall Davis
Joshua Davis
Aaron Davis
David Davis
Darryl Davis
Frank Davis
Phil Davis
Keith Davis
Robert Davis
Thomas Davis
Paul Davis
Adrian Davis
Faith Davis
Catherine M. Davis
Josh Davis
Leyla Davis
Michael Davis
Alissa Davis
Brian Davis
Elysia Davis
Darrell Davis
Alex Davis
Anthony Davis
Timothy Davis
Jillian Davis
Mckale Davis
Simon Davis
Ann Davis
Naomi Davis
Matthew Davis
Susan Davis
Jeremy Davis
Harry Davis
Linda Davis
Mark Davis

F. Caroline Davis
Barbara Davis Goldman
Jon Davison
Raziel Davison
Patt Davitt
Bazbek Davletov
Roberta Davoli
Antonio D'Avolio
Norouzi Davood
Horvatic Davor
Dominique Davoult
Alice Davy
Iakov Davydov
Mohamed Daw
Amy Dawel
Piers Dawes
Halima Dawood
Scott Dawson
Lisa Dawson
Alistair Dawson
Marcia Dawson
Michelle Dawson
Jesse Dawson
Wayne Dawson
Sally Dawson
John Dawson
Angela Dawson
Sarah-Jane Dawson
Carol Dawson Rose
Bess Dawson-Hughes
Egon Daxbacher
Anil Day
Christopher Day
Richard Day
Brad Day
Regina Day
John Day
J. Day
Alexander Day
Jeremy Day
Cody Day
Robert Day
Carolyn Day
Joseph Day
Lukejohn Day
Govindan Dayanithi
Loic Dayon
Paul Dayton
Carolyn Joy Dayton
Jean-Eudes Dazard
Kamila Dázio De Souza
Paola Dazzan
Frank B. Dazzo Dazzo
Rajat De
Wei De
Alok De
Abhijit De
Prithwiraj De
Bratati De
Emmanuelle Dé
Marilda Aparecida Milanez Morgado de Abreu
Marcus de Aguiar
Cesar de Almeida Neto
Guillermo De Anda-Jaúregui
Mariza de Andrade
Vincent De Andrade
Sara Adrian de Andrade
Katia De Angelis
Patricia de Araujo Souza
Lucilla de Arcangelis
Adriano de Assis
João de Azevedo
Soledad de Azevedo
Elfride De Baere
Mark de Baets
Kenneth De Baets
Miguel de Barros Lopes
Arnout Jan de Beaufort
Dirk de Beer
Tjaart de Beer
Maria de Beer
Giuseppe De Benedetto
Anna De Benedetto
Ana de Bettencourt-Dias
Dario de Biase
Ricardo de Bin
Birgitte de Blasio
Onno de Boer
Anthonius de Boer
Sietse de Boer
Bart de Boer
Albertus de Boer
Hans de Boer
Patrick De Boever
Adolfo De Bold
Xavier De Bolle

Raquel De Boni
Mario de Bono
Karolien De Bosscher
Elise de Bree
Anna de Breij
Lycia de Brito-Gitirana
Nancy De Briyne
Ellen de Bruijn
Marleen de Bruijne
Marika de Bruijne
Eling de Bruin
Angela De Bruin
Eduard J. de Bruin
Paul De Bruin
F.T. de Bruine
Guy de Bruyn
Marien de Bruyne
Jeroen De Buck
Juan De Carlos
Luiz Pedro de Carvalho
Mamede de Carvalho
Aline de Castilho
Christophe De Champs
Alain de Cheveigne
Cesira de Chiara
Loretta De Chiara
Munmun De Choudhury
Olivier De Clerck
Patrick De Clercq
Laurens De Cocker
Barbara De Coninck
Barbora de Courten
Marieke De Craemer
Kaat De Cremer
Raimondo De Cristofaro
Marco de Curtis
Ivan de Curtis
Laure de Decker
Steven De Decker
Ruth de Diego Balaguer
Isabel de Dios
Ulf de Faire
Giulia De Falco
Claudio De Felice
Massimo De Felici
Aldo De Ferrari
Maria Laura De Filippis
Bianca De Filippis
Henrik de Fine Licht
Lucia De Florio
Stefan de Folter
Vittorio de Franciscis
Paulo de Freitas
Sergio Tonetto de Freitas
Sergio de Frutos
Bart De Geest
Sabina De Geest
Beatrice de Gelder
Ugo De Giorgi
Marco De Giovanni
Giovanni de Girolamo
Chiara De Girolamo
Juliana De Gobbi
David de Gorter
Miranda De Graaf
Dirk de Graaf
Gert de graaf
Simon de Graaf
Laura de Graff
Wim de Grip
Bert De Groef
Arjan de Groot
Bert de Groot
J. F. de Groot
Natalia S. De Groot
Sandra De Groote
Frank de Gruijl
Judy de Haan
Lucia De Haene
Emile de Heer
Marc De Hert
Sybren de Hoog
Natascha de Hoog
Esther de Hoop
Robb de Iongh
Geoffry De Iuliis
Alberto de J. Oliveros-Bastidas
Celeste de Jager
Magdia De Jesus
Hans de Jong
Frank de Jong
Mart de Jong
Pim de Jong
Koert de Jong
Trynke de Jong
David De Jong

Marcus de Jong
Bert de Jong
Hugo de Jonge
Nicolaas de Jonge
Lilian De Jonge
Anoek A. E. de Joode
Annelien C. de Kat
Peter de Keizer
Ellen De Keyser
Willem de Keyzer
Yves de Keyzer
Jorrit de Kieviet
Peter de Knijff
Christiaan de Kock
Tanja de Koeijer
Luit J. De Kok
Jason de Koning
Jos de Koning
Dirk-Jan de Koning
Harry de Koning
Jolanthe de Koning
Tania de Koning-Ward
Saula de Kreutzenberg
Silvia de la Barrera
Adela de la Campa
Berta De la Cerda
Albert de la Chapelle
Fernando de la Cruz
Jesus de la Cruz
José de la Fuente
Jesus De la Fuente
Cynthia de la Fuente
Gabriel de la Fuente
Esther de la Fuente
Leonardo De La Fuente
Ana Laura de la Garza
Pierre de la Grange
Nicole De La Mata
Luis De la Maza
John de la Parra
Ike de la Pena
Alejandro de la Peña-Moctezuma
Jose Luis de la Pompa
Raul de la Rosa
Stephan de la Rosa
Jorge Luis de la Rosa-Arana
Ivana de la Serna
Ignacio de la torre
Isabel de la Torre-Diez
Irmgard de la Vega
Jan De Laet
Primal de Lanerolle
Stijn De Langhe
Victoire De Lastours
Paul De Lay
Els De Leenheer
Joshua de Leeuw
Peter De Leeuw
Laura de Lellis
Natasha I De Leon-Rodriguez
Nico De Leu
Irma De Ley
Carlo De Lillo
Luiz Henrique de Lima Araujo
Giselle de Lima Peixoto
Antonino De Lorenzo
Paolo De Los Rios
Teresa de los santos
Andrea De Luca
Antonio De Luca
Paul De Luca
V. de luca
Manuela de Lucas
Martin de Luis
Ruud de Maagd
Moniek De Maat
Michiel de Maat
Cristiana de Macedo
Flavio De Maio
Örjan de Manzano
Laura De Marco
Patrizia De Marco
Renata De Maria
Nicola De Maria
Filippo de Marinis
Maurizio de Martino
Samantha De Martino
Luisa De Martino
Herbert de Matos Guedes
Ricardo de Matos Simoes
Monica De Mattei
Rita De Matteis
Willy De Mattia
Luiz de Mattos
Sebastiao Freitas de Medeiros
Ingrid De Meester

Hester de Melker
Gerard de Melo
Bernadette de Melo Franco
Osvaldo Pompilio de Melo Neto
Lorena de Mena
Ilse De Mesel
Tim De Meyer
Marc De Meyer
Veronica de Micco
María Victoria Pendón-Ruiz DE MIER
Carmen De Miguel
Samuele De Minicis
Joachim de Miranda
Noel de Miranda
Marcia Terezinha de Moraes
Augusto César de Moraes
Tania Beninga de Morais
Alejandra de Moreno de Leblanc
René De Mot
Leandro de Moura
Vera de Moura Azevedo Farah
Miguel de Mulder
Diane De Neubourg
Wim De Neys
Luca De Nicola
Gilberto De Nucci
Camila de Oliveira
Rodrigo de Oliveira
Antonio de Oliveira
Túlio de Oliveira
Stephan de Oliveira
Luiz de Oliveira
Amanda de Oliveira
Elaine de Oliveira
Mariana C. de Oliveira
Juliana de Oliveira
Cassia de Oliveira
Lariza Laura de Oliveira
Jarbas Rodrigues de Oliveira
Airton de Oliviera Manoel
Tiago Costa de Padua
Raffaele De Palma
Michele De Palma
G. De Palma
Stéphane De Palmas
Marian M. De Pancorbo
Francesca De Pandis
Donatella de Pascale
Vilfredo De Pascalis
Erich De Paula
Edwin De Pauw
Pieter De Pauw
Saskia De Pee
Vito De Pinto
Gonzalo de Polavieja
David de Pomerai
Kevin de Queiroz
Valli De Re
Nick De Regge
Christelle de Renty
Dirk De Ridder
Denise De Ridder
Mark De Ridder
Karin De Ridder
Luz Maria de Rigil
Juan Pablo de Rivero Vaccari
Hugues de Rocquigny
Jaap de Roode
Rafael de Roodt
Johan de rooij
Steve de Rosa
Mario De Rosa
Anita De Rossi
Alexandra de Rotte
Silvia De Rubeis
Marina De Rui
Dirk De Ruysscher
Bert De Rybel
Paulo de Sá Júnior
Tainá de Sandes-Freitas
Amalia De Santo
Vincent De Sapio
Dominique de Seny
Shermin de Silva
Rohan de Silva
Alfonso De Simone
Ana Carolina de Siqueira Couto de Oliveira
Luis de Sisternes
Enrico De Smaele
Lina De Smet
Riet de Smet
Stefaan De Smet
Adam de Smith
Patrícia de Soárez
Giuseppe De Socio
Jeanlex de Sousa

Paulo de Sousa
Gustavo De Souza
Mary Jane De Souza
Alessandra De Souza
André de Souza
Flavio de Souza
Russell de Souza
Raphael de Souza
Sonia de Souza
Roberto Antonio de Souza
Cristiane De Souza Carvalho
Bart De Spiegeleer
Nicola De Stefano
Johan De Sutter
Rik de Swart
Vincenzo De Tata
Anthony De Tomaso
Pieter de Tombe
Maria de Toro
Carmen de Torres
Marleen De Troch
Miguel de Vega
Manuel De Vega
Natasha de Vere
Luciana De Vero
Fabrizio de Vico Fallani
Jean-Pierre de Villartay
Willem de Villiers
Melgardt M. de Villiers
Deidre de Villiers
Jill De vis
Fernando De Vita
Pasquale De Vita
Marco De Vivo
Steven de Vleeschouwer
Elly De Vlieghere
Dirk De Vos
Paul De Vos
Maarten De Vos
Alex de Voux
Carlie de Vries
Rory de Vries
Ronald de Vries
Esther de Vries
Jantina de vries
Haitze de Vries
Sieta de Vries
Frank de Vries
Gerben Klaas Dirk De Vries
Jo De Vrieze
André de Waal
Jan De Waele
Gerdien de Weert
Roel de Weger
Letty de Weger
Rosemarie De Weirdt
Aaike De Wever
Rene de Wijk
Leon de Windt
Benedicte De Winter
Joost de Winter
Emmie de Wit
Nicole de Wit
Rutger De Wit
Joris de Wit
Maarten de Wit
Peter de Witte
Martina de Zwaan
Bruce Deagle
Silvia Deaglio
Janine Deakin
Paba Dealwis
Caroline N Dealy
Christopher Dean
Wendy Dean
Michael Dean
Matthew Dean
Jesse Dean
Elizabeth Dean
John Dean
Melinda Dean
David Dean
Brian Dean
Kevin Deane
Robert Deaner
Donald L. Deangelis
Greg Deangelis
Don Deangelis
Margaret Deangelis
Monica Deangelis
Andrew Deans
Tara Deans
James Dear
Laura Dearden
Ian Deary
Aimée Deaton

Jane Deayton
Rajib Deb
Michel Debaar
Koenraad Debackere
Vincent Debari
Florence Debarre
Jean Debarros
Guillaume Debaty
Martin Debbane
Lucie Debeffe
Rudolf Debelak
Justine Debelius
S. Debette
Melanie Debiais-Thibaud
Melissa Debiasse
Thierry Debillon
Choi Deblieck
Anjan Debnath
Jane Debode
Vincent Deboer
Mark Deboer
Amber Debono
Patrick Deboosere
Alain Debrabant
Thomas Debray
Anik Debrot
Jennifer Debruyn
Philip Debruyne
Adeb Debska-Slizien
Bettina Debû
Jonas Debusscher
Zeger Debyser
Koen Decancq
Julius Decano
James Decaprio
Anthony Decaprio
Nicola Decaro
Guarionex Decastro
Oscar Decco
Agnes Dechartres
Thomas Dechat
Alexander Dechene
Peter Dechent
Paul Dechow
Thomas Decker
Brooke Decker
Summer Decker
Yves Declerck
Steven Declerck
Wim Declercq
Eugene Declercq
Anne Emilie Decleves
Xavier Declèves
Anne-Laure Decombeix
Frederik Deconinck
Lucimary Deconto
Lore Decoster
Axel Decourtye
Karin Dedek
Simon Dedeo
Stephane Dedieu
Dan Dediu
Elena Dedkova
Francesca De'Donato
Fabrice Dedouit
Michael Dee
Anne Dee
Scott Dee
Cornelia Deeg
Mark Deeg
Rosalia Deeken
Shelley Deeks
John Deeks
Dinesh Deelchand
Michael Deem
Denis Deeming
Jacqueline Deen
Sarah Deeny
Gagan Deep
Giovanni Defazio
Tony Defazio
Omar Defeo
Kenneth Deffenbacher
Xavier Deffieux
Guillaume Deffuant
Armin Deffur
Paola Defilippi
Christopher Defillipi
Louis Defrate
Ruth Defrin
Jason Defuria
Francis Degache
Carolin Degener
Franziska Degenhardt
Howard Degenholtz
Giulia Degiacomi

Michael Degiorgio
Cyril Degletagne
Alessia Deglincerti
Sandie Degnan
Patrick Degnan
Andrew Degnan
David Degraff
Joseph Degraft-Johnson
Gloria Degrandi-Hoffman
Francesca Degrassi
Fred Degraves
James Degregori
Michael Degregorio
Sven Degroeve
Jun-O Deguchi
Lilia Deguzman
Kofi Deh
Patrick Dehail
Eric Deharo
Pieter Dehaseth
Benjamin Dehay
Mohammed Dehbi
Cristina Dehelean
Dimitri Deheyn
Hesam Dehghani
Mohsen Dehghani
Matthias Dehmer
Katharina Dehnen-Schmutz
Louis Dehner
Martin Dehnhard
Nina Dehnhard
Yves Dehouck
Ahmad Dehpour
Manuela Deidda
Stacie Deiner
Thomas Deisboeck
Florian Deisenhammer
Heidrun Deissler
Annemie Deiteren
George Deitrick
Kirk Deitsch
Elisabetta Dejana
Jerome Dejardin
Emmanuel Dejardin
Alain Dejean
Gregory Dekaban
Benjamin Dekel
Serhiy Dekhtyar
Aldo Dekker
Teun Dekker
Andre Dekker
Olaf Dekkers
Bas Dekkers
Erik Dekwaadsteniet
Julia Del Amo
C. Del Amo
Rosa Maria Del Angel
Altair Del Bel Cury
Luiz Eduardo Del Bem
Rosa Del Campo
Lorenzo Del Castello
I. Del Castillo
Ignacio Del Castillo
Marco Del Chiaro
Kleber Del Claro
Maria Del Curto
Silvia Del Din
Stefano Del Duca
Andrea Del Fattore
Carlos Del Fresno
Federico Del Gallo
Charo Del Genio
Deborah Del Junco
Annarosa Del Mistro
Federica Del Monte
Claudio Del Percio
Miguel A Del Pozo
Jesús Del Pozo Cruz
Borja Del Pozo Cruz
Lucian Del Priore
Dominic Del Re
Mónica Del Rey
Eduardo Del Rio
Rodrigo Del Rio
Maria Del Rocío Reyes-Montes
Mario Del Rosso
Silvia Del Ry
Giannino Del Sal
Sara Del Valle
Catherine Del Vecchio
Erin Dela Cruz
Jean Delabar
Jacques Delabie
Douglas Delahanty
Fabien Delahaye
Eamonn Delahunt

Paul Delamater
Alexandre Delamou
Pierre Delanaye
Kevin Delaney
Sven Delaney
Deborah Delaney
Meghan Delaney
Nathaniel Delaney-Busch
Gabrielle Delannoy
Paul Delano
Matthew Delano
John Paul Délano-Frier
Isabel Delany
Anne Delany
Pierre Delaplace
Keith Delaplane
Cecile Delarasse
Jacques Delarue
Carla Delatorre
Benoit Delatour
Hélène Delatte
Thomas Delattre
Franck Delaunay
Pascal Delaunay
Ronald Delaune
Ro Delaune
Dawn Delay
Luis Delaye
Frederic Delbac
Lea Delbridge
Alex Delbridge
Claude Delcayre
Kleber Del-Claro
George Delclos
Pablo Delclós
Anne Delcour
Johann Delcourt
Louise Deldicque
Henri-Jacques Delecluse
James Delehanty
Michela Deleidi
Olivier Delelis
Lucie Delemotte
Teresa Deleon
Ronak Delewi
Loic Deleyrolle
Massimo Delfabbro
Frederick Delfin
Kristin Delfino
Massimo Delfino
Victoria Delgado
Cynthia Delgado
Juan Luis Delgado
Elena Delgado
A. V. Delgado
Edgar Delgado-Eckert
José Delgado-García
Guillermo Delgado-Lamas
Juan Delgado-Sanmartin
Glenn Delgiudice
Laurence Delhaes
Patric Delhanty
Gustavo Delhon
Gabriele Delia
Boris Delibašic
Céline Delierneux
Marie Laure Delignette-Muller
Mario Milco D'Elios
Christine Delire
Jean-Sébastien Delisle
Brian Delisle
Marcello Delitala
Evangelos Delivopoulo
Evangelos Delivopoulos
Gary Dell
Silvia Della Bella
Maria Della Chiesa
Helvécio Della Coletta-Filho
Claudia Della Corte
Chiara Della Libera
Maria Elena Della Pepa
Matteo Della Porta
Giorgia Della Rocca
C. J. Della Valle
Raffaele Dellaca'
Flavio Dell'Acqua
Mark Dell'Acqua
Alia Dellagi
Mario Dell'Agli
Bianca Della-Guardia
David Della-Morte
Giorgio Dellarocca
Robert Dellavalle
Gianfranco Delle Fave
Simona Delle Monache
Gemini Delle Vedove

Santo Dellegrottaglie
Stephanie Dellicour
Thanh Dellinger
Olivier Dellis
Persio Dello Sbarba
A-Lee Dellon
Evan Dellon
Daniele Dell'Orco
Valentina Dell'Oste
Volker Dellwo
Christine Delmaire
Bernard Delmas
Robert Delmas
Eric Delmelle
Pieluigi Delmonte
Daniela Delneri
Giovanni Delogu
Allison Delong
John Delong
Kristine Delong
Laura Delorenzo
Darren Delorey
Maria Deloria-Knoll
Yann Delorme
Philippe Deloron
Regine Delourme
Marion Delous
Francis Delpeyroux
Lynda Delph
Nicolas Delpierre
Sylvain Delplanque
Jérôme Delroisse
Serge Delrot
Michel Delseny
Eric Delson
Andrew Delton
Angelo Delucia
Jean-François Delvenne
Michael Delves
Marianne Delville
Edgard Delvin
Eric Delvin
Jeannine Delwiche
Christophe Délye
George Demakis
Maurice Demanou
Vincent Demarco
Flávio Demarco
Bernice Demarco
David Demarini
Thomas Demarse
Angelo Demarzo
Marcelo Demarzo
Gregory Demas
Nicolas Demaurex
Deeptankar Demazumder
Shadmehr Demehri
Joe Demer
Caroline Demeret
Cecilia Demergasso
Vincent Demery
I. Demesteer
Michael Demetriou
Lloyd Demetrius
Michael D'Emic
Eugene Demidenko
Sylvie Demignot
Marie Demion
Claire Demiot
Ihsan Ekin Demir
Alican Demir
Selami Demir
Emrah Demirci
Ufuk Demirel
Plamen Demirev
Margaret Demment
Ryan Demmer
Matty Demont
Amanda Demopoulos
Sharon Demorrow
William R. Demott
Jean-Baptiste Demoulin
Eugene Dempsey
Harriet Dempsey-Jones
Taku Demura
Angelo Demuro
Elisa Demuru
Donald Demuth
Svitlana Demyanets
Henk Den Bakker
Femke Den Boon
Nicoline Den Breems
Wijnand Den Dekker
Arjan Den Dekker
Anne Den Heijer
Wouter Den Hollander

Erick Denamur
Katherine Denby
Laura Denby
Nandini Dendukuri
Adam Denes
Balazs Denes
Caroline Denesvre
Jeremiah Deneve
Wu-Min Deng
Wen Deng
Xuemei Deng
Xin Deng
Kuiying Deng
Chao Deng
Sophie Deng
Xiuxin Deng
Jianming Deng
Tao Deng
Haiteng Deng
Fei Deng
Zhi-Hong Deng
Wen-Hong Deng
Nan Deng
Chunhua Deng
Lin Deng
Min Deng
Han-Bing Deng
Qihong Deng
Xinyang Deng
Zeyi Deng
Daiyong Deng
Youcai Deng
Qi Deng
Cecilia Deng
Wenjun Deng
Xiang-Wen Deng
Yingfeng Deng
Meng Deng
Fenglin Deng
Xiangzheng Deng
Yun-Fei Deng
Zhaohong Deng
Jun Deng
Hongxin Deng
Xiaoling Deng
Demao Deng
Guohong Deng
Qing Deng
Weiwei Deng
Changwang Deng
Zifa Deng
Xuliang Deng
Ziniu Deng
Jim Denham
Emma Denham
Petra Denig
Cecile Denis
Vianney Denis
Michel Denis
Marc Denis
Hémon Denis
Lionel Denis
Pierre Denise
Boris Denisov
Jeremy Denizot
Johannes Denk
Eric Denkers
Nathaniel Denkers
Stuart Denman
John Dennehy
Sven Dennerlein
Jonathan Dennis
James Dennis
John Dennis
Siobhan Dennison
Jonathan Denniss
Mark Denny
Thomas Denny
Bill Denny
Alexandre Denoyer
Béatrice Denoyes
Jerker Denrell
Llewellyn Densmore
Alexander Dent
Sharon Dent
Joseph Dent
Francesco Dentale
Francesco Dentali
Patrizia Dentelli
Kate Denton
Paul Denton
John Denu
Martin Denvir
Paul Denys
Atul Deodhar
Amit Deokar

Poonamjot Deol
Yadwinder Deol
C. Deom
Rajendar Deora
R. Deora
Sebastian Deorowicz
Baptiste Depalle
Claude Depamphilis
R. William Depaolo
Antoine Depaulis
Frantz Depaulis
Christopher Deperno
Adrien Depeursinge
Michael Depew
Thomas Depner
Marcel Deponte
Inge Depoortere
Antje-Christin Deppe
Stephen Deppen
Reinhard Depping
Chris Deppmann
Michael Depriest
Brendan Depue
Bertrand Deputte
Pieter Depuydt
Clio Der Sarkissian
Mohammad Derakhshan
Therese Deramaudt
Vincent Deramecourt
Colin Deransart
Wim Derave
Andrei Derbenev
Jonathan Derbridge
Stuart Derbyshire
Katarína Dercova
Cynthia Derdeyn
Colin P. Derdeyn
Colin Derdeyn
Imre Derenyi
Brian Derenzi
Massimo Derenzini
Assefa Deresse
Zygmunt Derewenda
Tobias Derfuss
Kebede Deribe
Amare Deribew
Kathryn Deriemer
Marco Deriu
Daniel Derivois
Katja Derkow
Eleonora Derlindati
Celine Dermardirossian
Wannes Dermauw
Veronique Dermauw
Kurt Dermen
Birgit Derntl
Mickael Deroche
Andrew Derocher
Giuseppe Derosa
Mathieu Derouet
Philippe Derreumaux
Jeremy Derrick
Steven Derrick
Philippe Deruelle
Li Derui
Stacy Deruiter
Christos Dervenis
Don Des Jarlais
Shyamal Desai
Sachin Desai
Tanvi Desai
Ankit Desai
Ankita Desai
Ankur Desai
Tara Desai
Dattesh Desai
Larisa Desantis
Morgan Desantis
Stacia Desantis
Anthony Desbien
Christele Desbois-Mouthon
Diane Descamps
Sébastien Descamps
Karl Desch
Jacqueline Deschamps
Thibault Deschamps
Reginald Deschepper
Stephane Descorps-Declere
Albert Descoteaux
Vianney Descroix
Yves Desdevises
Kobe Desender
John Desesso
Jessy Deshane
David Deshazer
Sachin Kumar Deshmukh

Manjeet Deshmukh
Umesh Deshmukh
Rajesh Deshmukh
Michael Deshotel
Uday Deshpande
Ani Deshpande
Siddharth Deshpande
Deepak Deshpande
Poonam Deshpande
Giovambattista Desideri
Vincenzo Desiderio
Justin A. Desimone
Paolo Desimone
Christopher Desjardins
Danielle Desjardins
Annie Deslauriers
Berthony Deslouches
Didier Desmaele
Christophe Desmet
Ann Desmet
Renee Desmond
Franck Desmoulin
Michel Desmurget
F. Desomer
Guillaume Desoubeaux
Danielle Desouza
Thomas Desplantez
Charles Després
Viviane Després
Annie Desrochers
Nicolas Desroy
Mahalia Desruisseaux
Scott Dessain
Patrick Dessein
Jean-Luc Desseyn
Daniele Dessi
Mireille Dessimoz
Clio Dessinioti
Christopher Destache
Olivier Destaing
Catherine D'Este
Sébastien Destercke
Giovanni Destro Bisol
Amélie Desvars
Mickaël Desvaux
Renu Deswal
Alexandre Detappe
Renaud Detayrac
Roger Detels
Rogier Determann
Richard Deth
Linda Detman
Michael Detmar
Emmanuel Detournay
John Detre
Christine Detrembleur
Jon Detterich
Robert Dettman
David Dettman
Amanda Dettmer
C Dettmers
M. L. Dettori
Patrizia D'Ettorre
Christopher Detzel
Susan Deuchars
Ruud Deurenberg
Mark Deurinck
Michael Deuschle
Andreas Deussen
Marcus-André Deutsch
Alexander Deutsch
Anne Deutsch
Mark E Deutschlander
Douglas Deutschman
Sandesh Dev
Marie Devaine
Kishor Devalaraja-Narashimha
Harsha Devalla
Sriram Devanathan
Joseph Devaney
Bharat Kumar Devapatla
Karthik Devarajan
Niveditha Devasenapathy
Jean-Marc Devaud
Yvan Devaux
Frédéric Devaux
Jerome Devaux
Ranjan Devbhandari
Pallavi Devchand
Brian Deveale
Jenni Deveau
Linda Deveaux
Ralph Devere White
Barry Devereux
Massimo Devescovi
Lakshmi Devi

Rukumani Devi
Sapna Devi
Anandita Devi
Martine Devic
Jessy Dévieux
Alessandro Devigili
Sébastien Devillard
Pierre Deville
Steven Devine
Alexandra Devine
Gregor Devine
Theresa Devine
Katie Devine
Ram Devireddy
Brecht Devleesschauwer
Sarne Devliegher
Angela Devlin
Joanne Devlin
B. Devlin
Holli Devon
Mark Devonald
Nick Devoogdt
Jayna Devore
Ben Devries
Marieke Devries
Carolyn Dewa
Laurence Dewachter
Oliver Dewald
Benjamin Dewals
Puneet Dewan
Adam Dewan
Genevieve Dewar
Meagan Dewar
Chiara Dewaure
Mieke Dewerchin
Michael Dewey
Marc Dewey
Floyd Dewhirst
Sylvia Dewilde
Jamie Dewitt
Antoine Dewitte
Andre Dewolf
J. Dewoody
Jeroen Dewulf
Sharmistha Dey
Ranadhir Dey
Bijan Dey
Sanjit Dey
Pranab Dey
Prasenjit Dey
Tanujit Dey
Nupur Dey
Feza Deymeer
Guillaume Dezecache
Dome Dezsi
Colette Dezutter-Dambuyant
Charlene Dezzutti
Maarten Dhaenens
Namariq Dhahir
Maxime Dhainaut
Ajay Dhaka
Santosh Dhakal
Monica Dhakar
Rafael Dhalia
Sanjay Dhall
Naranjan Dhalla
Nafeesa Dhalwani
Mukesh Dhamala
M Dhanasekaran
Saravana Mohan Dhanasekaran
Navneet Dhand
Nisha Dhanda
Sivashanmugam Dhandapani
Subramanian Dhandayuthapani
Shireesha Dhanireddy
Om Dhankher
Neeraj Dhar
Animesh Dhar
Ratan Dhar
Surbhi Dhar
Rajagopal Dhar Chakraborty
Animesh Dhara
Nila Dharan
Ramamurthy Dharani
Dharani Dharani Hapangama
Sheetal Dharia
Ashwin Dharmadhikari
Shyamali Dharmage
Anak Dharmapatni
Kumar Dharmarajan
Arun Dharmarajan
Sunethra Dharmasiri
Vikas R Dharnidharka
Archana Dhasarathy
Dhruva Dhavale
Keertan Dheda

Frederic Dhermain
Waljit Dhillo
Samjot Dhillon
Paul Dhillon
Sunil Dhiman
Sanjiv Dhingra
Yatin Dholakiai
Stijn Dhondt
R. D'Hooge
William D'Hoore
Sahajal Dhooria
Gurtej Dhoot
Mehul Dhorda
Emily Dhurandhar
Yuanpu Peter Di
Zengru Di
Da Di
Chongzhi Di
Xin Di
Zhao Di
Angela Di Baldassarre
Vitantonio Di Bello
Fabio Di Bello
Anna Maria Di Betta
G Di Bonaventura
Paola Di Bonito
Giuseppe Di Caprio
Enrico Di Cera
Mariachiara Di Cesare
Patricia Di Ciano
Riccardo Di Clemente
Luisa Di Costanzo
Tomás Di Domenico
Maikon Di Domenico
Jeremy Di Domizio
Pietro Di Fazio
Mirko Di Febbraro
Vittorio Di Federico
Alfredo Di Filippo
Martina Di Fonzo
Giancarlo Di Gennaro
Riccardo Di Giminiani
Nicola Di Girolamo
Biagio Di Iorio
Vincenzo Di Lazzaro
Margherita Di Leo
Fabio Di Lisa
Francesco Di Lorenzo
Giulia Di Lullo
Massimo Di Maio
Lucia Di Marcotullio
Michael Di Maria
Bendetto Di Martino
Vincenzo Di Marzo
Francesco Di Nardo
Paolo Di Nardo
Javier Di Noia
Alessandro Di Nuovo
Thérèse Di Paolo
Giovanni Di Pasquale
Giuseppe Di Pasquale
Giuseppe Di Pellegrino
Fabio Di Pietrantonio
Attillio Di Pietro
Flavia Di Pietro
Francesco Di Raimondo
Gianfranco Di Renzo
Laura Di Renzo
Antonio Di Sabatino
Martino Di Salvo
Thomas G Di Salvo
Elia Di Schiavi
Katie Di Sebastiano
Francesco Di Serio
Nicoletta Di Simone
Stephanie Di Stasi
Antonino Di Stefano
Giuseppina Di Stefano
Luca Di Tommaso
Francesco Di Virgilio
Agustina Di Virgilio
Dolores Di Vizio
Erika Di Zazzo
Giovanni Di Zenzo
Ibrahima Dia
Vasile Diaconu
Vasilios Diakonis
Ivan Diakonov
George Diallinas
Ariel Diamant
Zuzana Diamant
Evanthia Diamanti-Kandarakis
Athanasios Diamantopoulos
Scott Diamond
Alan Diamond
Michael Diamond

Gill Diamond
Tony Diamond
Navid Dianati
Aaron Diantonio
Irma Dianzani
Xianmin Diao
Honglu Diao
Honyang Diao
Brian Dias
João Miguel Dias
Teresa Dias
Pedro Dias
George Dias
Olivia Dias
Cícero Dias
Magnus Dias Da Silva
Marcos Dias De Assuncao
Jose Diaz
Bruno Diaz
Patricia Diaz
Francisco Diaz
Julio Diaz
Francisca Diaz
Maria Alejandra Diaz
Gabriel Diaz
Pilar Diaz
Mario Diaz
Esperanza Diaz
Gerardo Diaz
Aaron Diaz
Isabel Díaz
Paula Díaz
Begoña Díaz
G. Díaz
Antonio Diaz Espejo
María Díaz Roldán
Guillermo Diaz-Araya
Ramon Diaz-Arrastia
Isis Arlene Díaz-Carrión
Josue Díaz-Delgado
Daniel Diaz-Gil
Albert Diaz-Guilera
Ana R. Diaz-Marrero
Ramon Diaz-Orejas
Alberto Díaz-Ruiz
Carla Díaz-Tielas
Katharine Dibb
Kimberly Dibble
Hamdi Dibeklioglu
Sulayman Dib-Hajj
Charna Dibner
Wolfgang Dichtl
Tobias Dick
Warren Dick
Alec Dick
Matthew Dick
Joseph Dickens
William Dickens
Adam Dicker
Ian Dickerson
Erin Dickerson
Chad Dickey
Aaron Dickey
Jeffrey Dickhout
David Alexander Dickie
Thomas Dickins
David Dickinson
Tim Dickinson
Chris Dickinson
Christopher Dickman
Ahmadou Dicko
Robert Dickson
David Dickson
Ruth Dickstein
Nagasa Dida
Raphael Didham
Romain Didier
Faye Didymus
Roberto Dieci
Maria Vittoria Dieci
Ralf Dieckmann
Kai Diederich
Adele Diederich
Marc Diederich
Andreas Diefenbach
Julia Diegelmann
Ferran Diego
Xavier Diego
Louis Diehl
Philipp Diehl
Robert Diehl
William Diehl-Jones
Maximilian Diehn
Chris Diehnelt
Jurgen Dieker
Rabea Diekmann

Roland Diel
Joseph Dieleman
Nikki Dieleman
Francesca Diella
Ricarda Diem
Jeremy Diem
Igor Diemberger
David Diemert
Stewart Diemont
Ed Diener
Rodrigo Dienstmann
Cuong Diep
Andreas Diepold
Ron Diercks
Hans Dierckx
Brenda Diergaarde
Herman Dierick
Kai Dierkes
Thomas Dierks
Christine Dierks
Sarah Diermeier
Martin Diers
Anne Diers
Mara Dierssen
Vincent Dietemann
Kevin Dieter
Doug Dieterich
Paul Dietl
Gregory Dietl
Ursula Dietrich
Fred Dietrich
Dalton Dietrich
Lars Dietrich
Dimo Dietrich
Alexander Dietrich
Olaf Dietrich
Tobias Dietrich
Thomas Dietrich
Alia Dietsch
Maurine Dietz
Noella Dietz
Markus Dietz
Thomas Dietz
Michael Dietze
Ralf Dietzgen
Anke Dietzsch
Bernhard Dietzschold
Javier Diez
Claudius Diez
Iratxe Diez-Delgado
Ana Diez-Fernandez
Ricardo Diez-Valle
Cristina Diez-Vives
Joseph Roland Difranza
Florence Digennaro Reed
Susan Digiovanni
Maurizio Digiuni
Michelle Digman
Joseph Digrazia
Gianni Diguglielmo
Rafael Dihl
Lubbert Dijkhuizen
Rick Dijkhuizen
Jan-Willem Dik
Willem Dik
Sergey Dikalov
Jimmy Dikeakos
Michele Dileone
Anna Dilger
David Dilillo
Christina Dillahunt-Aspillaga
Maria Dillard
Stephanie Dille
Johanna A. Dille
Tom Dillehay
Marie-Agnès Dillies
Andrew Dillin
Allissa Dillman
Joakim Dillner
Rod Dillon
John Dillon
Shannon Dillon
Harvey Dillon
Michael Dillon
Gregory K. Dillon
Moira Dillon
Stephanie Dillon
Thomas Dilts
Giancarlo Dimaggio
Marie-Thérèse Dimanche-Boitrel
Helen Dimaras
Patrick Di-Martino
Jacqueline Dimatelis
Paul Dimayuga
Wayne Dimech
Raffaella Dimicco

Sona Dimidjian
Nicola Dimitri
Georgis Dimitriadis
Slavica Dimitrieva
Gabriel Dimitriou
Jordan Dimitrov
Borislav Dimitrov
Julia Dimitrova
Krste Dimitrovski
Henrk Dimke
James Dimmock
Stavros Dimopoulos
Christian Dimpka
Goberdhan Dimri
Muhammad Din
Aumreetam Dinabandhu
Courtney Dinardo
Pietro Dindo
Grete Dinesen
Andrii Dinets
Guosheng Ding
Xiaoyan Ding
Mei Ding
Chunming Ding
Xiangdong Ding
Wen-Xing Ding
Yali Ding
Fei Ding
Nai Ding
Qiang Ding
Yuchuan Ding
Shaoxiong Ding
Hu Ding
Ke-Feng Ding
Chuanqing Ding
Xiao Ding
Shujun Ding
Xi-Qin Ding
Jingxian Ding
Husheng Ding
Zhaojun Ding
Xianfeng Ding
Yun Ding
Yan Ding
Zeng-Bo Ding
Ying Ding
Yu Ding
Shaojun Ding
Gangqiang Ding
Cheng Ding
Weixin Ding
Ling Ding
Sheng Ding
Yousong Ding
Yingyding Ding
Shuai Ding
Jie Ding
Jingzhen Ding
Ye Ding
Xianjun Ding
Aidong Ding
Zhi-Ming Ding
Fangbao Ding
Ning Ding
Shiyou Ding
Yezhang Ding
Wei Ding
Guowei Ding
Xia Ding
Xueli Ding
Dale Ding
Cody Ding
Feng-Hua Ding
Lili Ding
Guangda Ding
Wj Ding
Tao Ding
Mark Dingemanse
Michael Dingkuhn
David Dingli
Douglas Dingman
Minh Dinh
Rj Dinis-Oliveria
Carmen Diniz
Mário Diniz
Bruno Diniz
Pedro Paulo Diniz
Julien Dinkel
Lisa Dinkler
Jonathan Dinman
Jacqueline Dinnes
Achmad Dinoto
Ivo Dinov
Anuwat Dinudom
Gniesha Dinwiddie
Maria José Diogenes

Marina Diomedi
Daniele Dionisio
Alan Diot
Quentin Diot
Jorge Dipaola
Ottavia Dipasquale
Nicholas Dipatrizio
Keith Dipetrillo
Luisa Dipietro
Ludovico Dipineto
Leila Dirani
Bruno Direito
Haner Direskeneli
Gianfranco Diretto
Daniel Dirkmann
Ron Dirks
Wendy Dirks
Ellen Dirkx
Ermias Diro
Giulio Disanto
Michelle Discacciati
Rodney Dishman
Clara Dismuke
Anita Disney
Salvatore Disomma
John Disterhoft
Larry Distiller
Ottmar Distl
Eleonora Distrutti
Elke Dittmann
Michael Dittmar
Thorsten Dittmar
Dirk Dittmer
Neal Dittmer
Ian Dittmer
Anna Dittrich
Henrik Ditzel
Pradeep Divakar
Sara Divall
Maziar Divangahi
Vincent Dive
Chaitanya Divgi
Dario Diviani
Christina Divne
Richard Dix
Vishva Dixit
Narendra Dixit
Priyanka Dixit
Linda Dixon
Dan Dixon
Ian Dixon
Roger Dixon
Dave Dixon
Philip Dixon
J. Brandon Dixon
Peter Dixon
Katie Dixon-Gordon
Barnaby Dixson
Danielle Dixson
Luwis Diya
Bekir Dizdaroglu
Janine Dizon
Ali Djalilian
Madava Djearaman
Dagbegnon C. Sohoulande Djebou
R. Djeribi
Philippe Djian
Valentin Djonov
Jelena Djordjevic
Sergej Djuranovic
Zora Djuric
Ouria Dkhissi-Benyahya
Darryl D'Lima
Monika Dmitrzak-Weglarz
Ivan Dmochowski
Roger Dmochowski
Tri Do
Michael Do
Ha Do
S. H. Do
Elizabeth Do
An Do
Frederic Do
Yi-Yin Do
Andre Luiz Do Nascimento
Elizabeth Do Nascimento
José Clóvis Do Prado
Jean Claude Do Rego
Elizanilda Ramalho Do Rêgo
Thomas Doak
Stacey Doan
Charles Doarn
Carlota Dobano
Chelsea Dobbins
Fred Dobbs
Matthew Dobbs

Sylvia Dobbs
Joannie Dobbs
Christian Dobel
Claudia Dobler
Martina Doblin
Karen Dobos
Katalin Dobra
Marian Dobranschi
Sergey Dobretsov
Dobromir Dobrev
Mario Dobrilovic
Ulrich Dobrindt
Alexander Dobrovic
Oxana Dobrovinskaya
Hana Dobrovolny
Ricardo Dobrovolski
Gabriella Dobrowolny
Stephen Dobson
Renwick Dobson
Adam Dobson
A. Docherty
Denitsa Docheva
Margaret Docker
Sean Docking
Samantha Dockray
Paul Dockree
Mark Dockrell
Pilar D'Ocon
Jean-Denis Docquier
Tamas Doczi
Ian Dodd
Peter Dodd
Justin Dodd
Peter Dodds
Elizabeth Dodds Ashley
Luca Dodero
Kara Dodge
George Dodge
Somayeh Dodge
Kimberly Dodge-Kafka
Dejan Dodig
Syam Dodla
Peter Dodson
Chad Dodson
Matthew Dodson
Sabine Doebel
Philipp Doebler
Stefanie Doebler
James Doecke
John Doench
Torsten Doenst
Hendrik Doering
Johanna Doerr
Sam Doesburg
Volker Doetsch
Andreas Doetsch
Michiel Doff
Jean D'Offay
Mehmet Doganay
Tamara Dogandžic
Zoran Dogas
J. Doggett
Karl Doghramji
Preeti Dohare
Emer Doheny
Tim Doherty
Phil Doherty
Andrew Doherty
Shinya Dohgu
Gert Dohle
Pascal Dohmen
C. Dohna-Schwake
Nobuhide Doi
Kent Doi
Kazuyuki Doi
Hideyuki Doi
Yohei Doi
Kazutaka Doi
Lawrence Doi
Andrew Doig
Gordon Doig
Amber Doiron
Gilad Doitsh
Hisham Dokainish
Serhat Doker
Ivana Dokic
Karol Dokladny
Daniel Doktor
Maxim Dokukin
John Dolan
Patrick Dolan
Christopher Dolanc
Pavlina Dolashka-Angelova
Pasquale Dolce
Antonina Dolei
Jurij Dolensek

Tomas Dolezal
Adam Dolezal
Amalia Dolga
Alain Dolla
Laurent Dollé
Matthias Dollinger
Günther Dollinger
Oliver Dolly
Marie-Madeleine Dolmans
Igor Dolmatov
Stephanie Dolrenry
Helmut Dolznig
Kenji Doma
Teresa Domagala
Isabelle Domaizon
Frederick Domann
Robert Domaoal
Alice Domar
József Dombi
Alan Dombkowski
Aviv Dombrovsky
Stephan Dombrowski
Keith Dombrowski
Christian Dombrowski
Ronald Domen
Xavier Domene
Laia Domenech
Maria Rosaria Domenici
Luciano Domenici
Prattichizzo Domenico
Lorenzo Domenis
Pb Dominelli
Pere Domingo
Pedro Domingos
Ana Domingos
Jose Domingos
Michael Domingue
Francisco Domingues
Carla Domingues
Juan Dominguez
Francisco Dominguez
Ligia Dominguez
Marisol Dominguez
Angela Domínguez
Jorge Domínguez
Jose Domínguez
Estefanía Domínguez-Martínez
Manuel Domínguez-Rodrigo
Fernando Dominici
Massimo Dominici
Zachary Domire
Henrik Dommisch
Daryl Domning
Ferenc Domoki
Hisanori Domon
Maria Gabriella Donà
Xavier Donadeu
Stefano Donadio
Carlo Donadio
C. Donadio
Michael Donahoe
Henry Donahue
Manus Donahue
Megan Donahue
Lyall Donald
Paul Donaldson
Julie Donaldson
Janet Donaldson
W. Donarchie
Cornelius Donat
Lars Donath
Marc Donath
Giuseppe Donati
Chiara Donati
Marcello Donati
Abele Donati
Giacomo Donati
Rosario Donato
Jose Donato
Tony Donato
Celeste Donato
Nicholas Donato
José Donázar
Frank Dondelinger
Francesco Dondero
Timme Donders
Luca Dondini
Michael Dondrup
Sarah Donegan
Nathan Donelson
Chuanhui Dong
Hansong Dong
Suomeng Dong
Jie Dong
Min Dong
Cun-Jian Dong

Shuang-Lin Dong
Hui Dong
Hengjin Dong
Shikui Dong
Jian Dong
Yufeng Dong
Yue Dong
Liyao Dong
Bi-Cheng Dong
Zheng Dong
Yuan Dong
Hongpo Dong
Zaijie Dong
Jing Dong
Yupeng Dong
Hezhong Dong
Yifei Dong
Yulei Dong
Xiaoling Dong
Yueping Dong
Yi Dong
Xiang Dong
Junchao Dong
Li-Hua Dong
Zhiping Dong
Bing Dong
Xuran Dong
Guanpeng Dong
Zhongyun Dong
Li Dong
Jing-Ming Dong
Yinghui Dong
Ming Dong
Yalin Dong
Guanghui Dong
Wei Dong
Gaogao Dong
Xiaoli Dong
Hongyuan Dong
Ruoyu Dong
Yongli Dong
Shuangling Dong
Lijin Dong
Jingcheng Dong
Yan Dong
Annibale Donini
Ruben Donis
Jeroen Donkers
Peter Donkor
Rodney Donlan
Jeffrey Donlea
Maureen Donlin
L. Donlin
Cam Donly
M. Metin Donma
Tiziano Donnarumma
Karsten Donnay
M. Brent Donnellan
Ryan Donnelly
Sheila Donnelly
Alison Donnelly
John Donnelly
Roarke Donnelly
Alan Donnelly
Martin Donnelly
Philippe Donnen
Michael Donnenberg
Daniel Donner
Olivier Donnez
Nicole Donofrio
Mirko D'Onofrio
Giuseppe D'Onofrio
Helen Donoghue
Daniel Donoghue
Deborah Donoghue
Sarah Donohue
Paulina Donoso
Mark Donowitz
Evi Dons
Curtis Donskey
Frede Donskov
David Donze
Sean Doody
Tanya Doody
Keith Dookeran
Serena Dool
Frederick Dooley
Alton Dooley
Kevin Dooley
Jenalee Doom
Mark Dooner
Denis Doorly
Sören Doose
Xaquin Dopico
Kathrin Doppler
Taraneh Dor Mohammadi Toosi

Soofia Dorabati
Pedro Dorado
Rajkumar Dorajoo
Michael Doran
Olena Doran
Juan José Dorantes-Aranda
Robert Dorazio
John D'Orazio
Gregory Dore
Simone Dore
Sylvain Dorel
Michael R. Dores
Elodie Dorey
Joanne Dorgan
Anca Dorhoi
Andrea Doria
Fabrizio Doricchi
Oliver Dorigo
Thomas Döring
Yvonne Döring
Thomas Dorlo
Susan Dorman
Karin Dorman
Michael Dorman
Dirk Dormann
Nathan Dormer
Olivier Dormond
Paulo Dorneles
Elaine Dorneles
Marcus Dorner
Pierre Dorny
Fabio Doro
Ginsberg Doron
Michael Dorothy
Sergey Dorozhkin
Michael Dorr
Jan Dörr
Adrienne Dorrance
Michael Dorrell
Lucy Dorrell
Richard Dorrell
Rob Dorrington
Carlos Dorronsoro
Maria Dorrucci
Travis Dorsch
Susan Dorsey
E. Ray Dorsey
Ivan D'Orso
Johannes Dorst
Pablo Dorta-González
Alan Dorval
Dinis Dos Reis Miranda
Nuno Dos Santos
Luara Isabela Dos Santos
A. S. Dos Santos
Carolina Dos Santos Passos
Derek Dosdall
Andrea I. Doseff
Strahinja Dosen
Prashant Doshi
Franco Dosio
Mert Döskaya
George Dosreis
C. George Priya Doss
Michael Xavier Doss
George Priya Doss Doss
Washington Dos-Santos
Aaron Dossey
Laure Dossus
David Dostal
Petr Dostal
Zsuzsanna Dosztányi
Javier Dotor
Dobromir Dotov
Ellen Dotson
Cedrick Dotson
Stefan Dötterl
Ivan Dotu
Tracy Doty
Q. Ping Dou
Daolong Dou
Huanyu Dou
Weibei Dou
Dejing Dou
Hongen Dou
Yuhong Dou
Horng-Yunn Dou
Zhicheng Dou
Marc Douaisi
Veronique Douard
Luc Douay
Zoe Doubleday
Vincent Doublet
Silvie Doublie
Gillian Douce
Alain Doucet

Nicolas Doucet
Christine Doucet
Souleymane Doucoure
David Douda
Anthony Doufas
David Dougan
Patrick Dougherty
Denise Dougherty
David Doughty
Ron Douglas
Marlis Douglas
Pamela Douglas
Tania Douglas
Timothy Douglas
Karen Douglas
Matthew Douglass
Mathieu Douhard
Vladimir Douhovnikoff
Francois Douillard
Katerina Douka
Agapi Doulgeraki
Paschalis-Thomas Doulias
Jean Douliez
Andreas Doulis
Patrick Doumas
Claudie Doums
Edmond Dounias
Natalia Dounskaia
Bruno Douradinha
Pierre Dourlen
Gael Dournes
Andrew Doust
Isabel Douterelo
Linda Douw
Chrysostomos Dovas
Athanassios Dovas
Sinisa Dovat
Martin Dovciak
Lynn Dover
Natalia Dovlatova
Constantine Dovrolis
John Dow
Steven Dow
Christopher Dow
Dorothy Dow
Sue Dow
Wes Dowd
Michael Dowd
Steven Dowdy
David Dowdy
Richard Dowell
Ann Dowker
Damian Dowling
Thomas C Dowling
Daniel Dowling
Ian Down
Thomas Down
Barbara Downes
Michael Downey
Laura Downie
Colleen Downs
Julie Downs
Stephen Downs
Peter Downs
Harold Dowse
James Dowty
Andrew Doxey
Stephen Doxsey
J. Doyden
Stephen Doyle
Scott Doyle
Elena Dozio
Mikhail Dozmorov
Jirí Drábek
Finn Drablos
Maxime Drabo
Cinthia Drachenberg
Annette Draeger
Rika Draenert
Dejan Dragan
Marko Dragas
Antonio Drago
Lorenzo Drago
Davide Dragone
Eduard Dragut
Paul Drain
Alison Drake
Alistair Drake
John Drake
Bettina Drake
Abby Drake
Robert E. Drake
Jeana Drake
Richard Drake
Mark Drakesmith
Papa Drame

Shaynoor Dramsi
Michel Drancourt
Mark Dranias
Brian Dranka
Ian Dransfield
Pierre Drapeau
Michelle Drapeau
Simon Draper
David Draper
Stéphane Dray
Matthew Dray
Cédric Dray
Dennis Drayna
Jean Drayton
Jeffrey Drazen
Sinead Drea
Anna Dreber
Sten Dreborg
Maik Drechsler
Susanne Drechsler
Alex Dregan
Steffen Dreger
Thomas Dreher
Robert Dreibelbis
Stephanie Dreier
Jacob Dreiher
Amelie Dreiss
Fotios Drenos
Clemens Drenowatz
Joost Drenth
Martin Dres
Markus Dresel
Stephen Dreskin
Mark Drever
Joël Drevet
Pascal Drevet
Pavel Drevinek
Christian Drevon
Mark Drew
Joshua Drew
Robert Drew
Trafton Drew
Paul Drew
David Drew
Julian Drewe
Lukaszola Drewniak
H. A. Drexhage
Jan Felix Drexler
Gideon Dreyfuss
Daniela Dreymueller
Jean-Michel Drezen
Sami Dridi
Denis Drieghe
Terri Driessen
Steven Driever
William Driggers
Sybren Drijfhout
Marc Drillich
Maria Drincovich
Petros Drineas
Rachid Dris
Paul Driscoll
Barbara Driscoll
Carlos Driscoll
Amy Driskell
Tarak Driss
Rachid Drissi
Karl Drlica
Drmoonss Drmoonss
Francis Drobniewski
Igor Drobyshev
Sam Droege
Marc Drolet
Mithilesh Dronavalli
Daniela Droppa
Itiel Dror
Vivian Drory
Konstantinos Drosatos
Agnieszka Drosdzol-Cop
Elefterios Drosinos
Garyfalia Drossopoulou
Dirk Droste
Christian Drosten
Guy Drouin
Michalis Drouvelis
David Drover
James Drover
Vladimir Drozdovitch
Stanislaw Drozdz
Martin Drucker
Dorothee Drucker
Thomas Druetz
Kirk Druey
Jan Drugowitsch
Sabine Druillennec-Rodière
Marjan Drukker

Todd Druley
Heidi Drummer
Frank Drummond
Rodrigo Drummond
Frances Drummond
Claire Drurey
John Drury
George Drusano
Irina Druzhinina
Irina S Druzhinina
Scott Dryden-Peterson
Stuart Dryer
Rachel Dryer
Tom Drysdale
Melissa Dsouza
Roshan D'Souza
Roshni D'Souza
Gina D'Suze
Shao Jun Du
Jiulin Du
Rose Du
Yansheng Du
Lixin Du
Pufeng Du
Min Du
Jing Du
Haijuan Du
Jiaxin Du
Aifang Du
Wei Du
Meijun Du
Zhenggui Du
Shihong Du
Jianchang Du
Huaidong Du
Jinming Du
Daolin Du
Jiang Du
Wen-Bo Du
Li-Lin Du
Yu-Zhou Du
Xin Du
Xuewen Du
Meng Du
Yuhui Du
Xiangjun Du
E. Du
Ruofei Du
Peng Du
Dongliang Du
Yi Du
Ning Du
Wenwen Du
Fuliang Du
Changzheng Du
Zhi-Qiang Du
Dan Du
Jian Du
Xueling Du
Yuchen Du
Lidan Du
Y. Du
Ximing Du
Xiuyuan Du
Ping Du
Wenbin Du
Xiong Du
Fei Du
Bastiaan Du Pre
Xianming Du Prel Carroll
Joss Du Toit
Caigan Dua
Tarun Dua
Dayue Duan
Zhao-Jun Duan
Ranhui Duan
Yongping Duan
Xin Duan
Chaojun Duan
Zhijun Duan
Susu Duan
Deyi Duan
Yuxi Duan
Jicheng Duan
Guangcai Duan
Lian Duan
Kangmin Duan
Qi Duan
Lei Duan
Hongtao Duan
Juan Duarte
Sofia Duarte
Simone Duarte
Elizabeth Duarte
Ana Rita Duarte
M. Duarte

Tiago Duarte
Djane Duarte
Noélia Duarte
Antonio Duarte
Angelo Duarte
Dena Dubal
Anna Dubaniewicz
Kateri Dubay
Erik Dubberke
Peter Dube
Anuradha Dube
Michael Dube
Timothy Dube
John Dube
Louis J. Dubé
Eve Dubé
Herbert Duber
Jean-Frederic Dubern
Christophe Dubessy
Sylvain Dubey
Rama Kant Dubey
David Dubnau
Melvin Dubnick
Margarita Dubocovich
Bertrand Dubois
Ludwig Dubois
Valérie Dubois
Stanislas Dubois
Emilie Dubois-Deruy
Julie Duboscq
Edward Dubovi
Olena Dubovyk
David Dubowitz
Sandrine Dubrac
Jacob Dubroff
Alexandra Dubrovina
Sasha Dubrovsky
Julien Dubrulle
Sylvie Dubuc
Sylvain Dubucquoi
Jean Dubuisson
Laurent Dubuquoy
Frederic Ducarme
Richard Ducatelle
Jordi Duch
Carsten Duch
Simon Ducheix
Jean-Bernard Duchemin
Sebastian Duchene
Michael Duchêne
Arnaud Duchon
Stephen Duckett
Hugh Ducklow
Jean-Charles Duclos-Vallee
Anne Ducros
Andrew Ducruet
Lorenzo Ductor
Georg Duda
Robert Duda
Piotr Duda
Katarzyna Duda
Jarrod Dudakov
Lubica Dudakova
Amel Dudakovic
Rachael Dudaniec
Tom Dudding
William Duddy
Pradeep Dudeja
Jan Dudek
Steve Dudgeon
Jeroen Dudink
Ekaterina Nailyevna Dudinskaya
Kevin Dudley
Jonathan Dudley
Anette Duensing
Macro Duering
Breck Duerkop
Felix Duerr
Rebecca Duerr
Daniel Duerschmied
Nicholas Duesbery
Mike Duever
Aaron Dufault
David Dufeau
M. C. Duff
Michael Duff
Putu Duff
Lynsey Duffell
Lisa Duffett
James Duffin
Stephen Duffull
Charles Duffy
Patrick Duffy
David Duffy
Michael Duffy
Carol Duffy

Craig Duffy
Lesley Duffy
Sandra Duffy
Lc Duffy
Nicolas Duforet-Frebourg
Sylvie Dufour
Antoine Dufour
Suzanne Dufour
Anne-Beatrice Dufour
Denis Dufrane
Christophe Dufresnes
Christina Duftner
Martin Dufva
Michael Dugan
Eric Dugan
Sreekantha Reddy Dugasani
Julie Dugdale
Ian Duggan
Raj Duggavathi
Brittany Dugger
Andrew Duggins
Srikant Duggirala
Mihai Duguleana
Dagne Duguma
Beatriz Duguy Pedra
S Duhachek-Muggy
Todd A. Duhamel
Arnaud Duhoux
Pascal Duijf
Saskia Duijts
Sjoerd Duim
Jorge Duitama
Ashley Duits
Tijs Duivenvoorden
Bernard Dujon
Reuven Dukas
Stephen Duke
Joshua Duke
Chris Duke
Jozef Dulak
Violaine Dulau-Drouot
Angela Dulhunty
Joanna Dulinska-Litewka
Ehsan Dulloo
Nicholas Dulvy
Jennifer Dumaine
Jan Dumanski
Guillaume Dumas
Nicolas Dumaz
Eric Dumbaugh
Guillaume Dumenil
Claudia Dumitru
Roger Dumke
Charles Dumke
Krishna Dummula
Tim Dumonceaux
Helene Dumond
Henri Dumont
Maitena Dumont
Nancy Dumont
Iroise Dumontheil
Hélène Dumortier
B. Dunais
Christophe Dunand
Carole Dunand
Yves Dunant
Robin Dunbar
Stephanie Dunbar
Michael Dunbar
Steven Duncan
Joseph Duncan
Robert Duncan
Elizabeth Duncan
Jacque Duncan
W. Duncan
Scott Duncan
Sylvia Duncan
Niall Duncan
Randall Duncan
David Duncan
Ryan Duncan
Mara Duncan
Ekrem Dundar
Christopher Dungan
Hans-Dirk Düngen
Jason Dunlop
Alex Dunlop
Patrick Dunlop
Rebecca Dunlop
Katherine Dunn
Graham Dunn
Adam Dunn
James Dunn
David Dunn
Michael Dunn
Louise Dunn

Gulayse Dunn
Sarah Dunn
Kelly Dunn
Joel Dunn
Margaret Dunne
Nicholas Dunne
James Dunne
Susana Dunner
Jake Dunning
Julie Dunning Hotopp
Gary Dunny
Meagan Dunphy-Daly
Sarah Dunphy-Lelii
Ayelet Dunsky
Joseph Dunsmoor
Yu Duonan
Le Duong
Isabelle Dupanloup
Stephan Duparc
Stéphane Dupas
Daphné Dupéré-Richer
Sandra Düpjan
Nicole Duplaix
Pascale Duplay
Mignon Duplessis
Anne Duplouy
Geneviève Dupont
Patrick Dupont
Sirio Dupont
Lise Dupont
Sreen Dupont
Christophe Dupont
Esther Dupont-Versteegden
Catherine Duport
Jean Dupouy-Camet
Loic Dupre
Denis Dupré
Aurélien Dupré
Nicolas Duprey
Delphine Duprez
Christelle Duprez
Paul Dupuis
Corinne Dupuy
Gael Dur
Jean-Maurice Dura
Salvador Dura-Bernal
Cecília Durães
Geetha Durairaj
Michael Durako
Jean-Baptiste Durand
Nicolas Durand
Miquel Duran-Frigola
Salvador Duran-Nebreda
Kathleen Durant
W. Durante
Kristina Durante
Emanuele Durante Mangoni
Edgardo Durantini
Francesca Duraturo
Matej Durcik
Stephen Durham
Andrew Durham
Tessa Durham Brooks
Nicolas Durier
Marcel Durieux
Pierre Durieux
Olivier Duriez
Magdalena Durlik
Michelle Durling
Ertan Durmusoglu
Jörg Durner
Joke Durnez
Alexandra Durr
Peter Durr
Timothy Durrett
Russell Durrrett
Ralf Dürrwald
Lisa Durso
Erdinc Dursun
Adil Duru
Carrie Durward
Jonathan Dushoff
Carlo Duso
Christian Dussault
Heiko Dussmann
Greg Dussor
Audrey Dussutour
Azger Dusthackeer
Michael Dustin
Donald Duszynski
Laura Dutca
Janice Dutcher
Frédéric Dutheil
Malcolm Duthie
Bernadette Dutia
Marie Dutreix

Sheetij Dutta
Noton Dutta
Shanta Dutta
Bhaskar Dutta
Samikshan Dutta
Tumpa Dutta
Ritaban Dutta
Anindita Dutta
Shruti Dutta
Arpan Dutta
Tushar K. Dutta
Gera Dutta
Asim Duttaroy
Susan Dutton
Rachel Dutton
Gareth Dutton
Ken Dutton-Regester
Jörg Dutz
Dawn Duval
Raphael Duval
Gilles Duverlie
Umamaheswar Duvvuri
Pierre Duyck
Jacques Duysens
Anthony Dvarskas
Zdenek Dvorak
Harsh Dweep
Michael Dwinell
Varun Dwivedi
Anila Dwivedi
S. F. Dworkin
Karen Dwyer
Johanna Dwyer
John Dwyer
Laura Dwyer-Lindgren
Michel Dy
Kristen Dybala
Mark Dybdahl
Cecil Dybowski
Jeff Dyche
Richard Dyck
P. James Dyck
Jason Dyck
John Dye
Justin Dye
Mike Dyer
George Dyer
William Dyer
Fiona Dyer
Robin Dyers
Gary Dykes
Brad Dykstra
Derek Dykxhoorn
Maya Dymova
Lyudmyla Dymytrova
Jan Dyr
Martin Dyrba
Lars Dyrskjot
Michele Dyson
Rebecca Dyson
Petter Dyverfeldt
Rhonda Dzakpasu
Susie Dzakpasu
Yvonne Dzal
Nicolas Dzamko
Cathy Dzerefos
Rosemary Dziak
Edward Dzialowski
Roman Dziarski
Agnieszka Dziduszko
Lukasz Dziewit
Wojciech Dzwolak
Bogdan Dzyubak
Oleksandr Dzyubak
