# Supplementary material for: PLOS ONE 2015 Reviewer Thank You
Source: PLoS One. 2016 Feb 23;11(2):e0150341. doi: 10.1371/journal.pone.0150341 (PMC4764340; doi:10.1371/journal.pone.0150341)
Supplement: S2 Reviewer List — (PDF) [file pone.0150341.s002.pdf]

PLOS ONE would like to thank all those who reviewed on behalf of the journal in 2015:

Hang-Korng Ea
Shaun Eack
Poul Eady
Anne Eady
Eric Eager
Patricia Earl
Ashlee Earl
Julia Earl
J. Mason Earles
Fergus Earley
Scott Earley
Cole Easson
Marion East
Andrew East
Alan Eastman
Joseph Eastman
Alison Eastwood
Mary Patrice Eastwood
Gillian Eastwood
William Eaton
Kathryn Eaton
Philip Eaton
David Eaton
Carla Eaton
Simon Eaton
Gareth Eaton
Colby Eaton
Michael Eaton
Nicholas Eaton
Tonya Eaves-Pyles
Abd El-Halim Ebaid
Sue Ebanks
Gregory Ebel
Werner Ebeling
Jessica Eberhard
B. Anne Eberhard
Ralf Eberhardt
Meghan Eberhardt
Robert Eberhart
Ivano Eberini
Stefan Eberl
Jeffrey Ebersole
Paul Ebert
Steven Ebert
Thomas Ebert
Natalie Ebert
Birgitta Ebert
David Eberth
Kristie Ebi
Masahito Ebina
Martin Ebinger
Sjoerd Ebisch
Ulrich Ebner-Priemer
Andreas Ebneter
Mansour Ebrahimi
Talin Ebrahimian
Esmaeil Ebrahimie
John Ebright
Richard Ebstein
Michael Eccles
David Eccles
R. Ecco
Aude Echalier
Marcela Echavarria
Miriam Echevarría
Karen Echeverri
Maria Echeverria
Valentina Echeverria Moran
Elizabeth Echoka
Reinalyn Echon
Matthias Echternach
Livia Echternacht
Walter Eckalbar
Kristin Eckardt
Thorsten Ecke
Juergen Eckel
Sandrah Eckel
Kristin Eckel-Mahan
Roderic Eckenhoff
Maryellen Eckenhoff
Gerhard Ecker
Ullrich Ecker
Timo M. Ecker
Patricia Eckerdal

Michelle Eckerle
Erin Eckert
Danny Eckert
Ester Eckert
Leopold Eckhart
Philip Eckhoff
Sidonia Eckle
Delrae Eckman
Doris Eckstein
Torsten Eckstein
Lutz Eckstein
Dieter Eckstein
Claude Ecoffey
Tassos Economou
Nicholas-Tiberio Economou
Shigetoshi Eda
Gilles Edan
Saadia Eddahibi
David Eddington
Tyler Eddy
Kamryn Eddy
Matthew Eddy
Obaghe Edeghere
Alison Edelman
Robert Edelman
Brad Edelman
Winfried Edelmann
Allan Edelsparre
John-Sebastian Eden
Maximilian Eder
Klaus Eder
István Édes
Dumessa Edessa
Robert Edgar
Graham Edgar
Chris Edge
Michael Edge
V. Reggie Edgerton
Mira Edgerton
Dale Edgerton
Jamie Edgin
Benoni Edin
Eunice Edison
Paul Edison
Majonga Edith
Myriam Edjlali
Adrienne Edkins
Andrea Edlow
Sarah Edmonds
Mark Edmondson-Jones
Peter Edmunds
Matthew Edmunds
Alemayehu Edo
Ruangelie Edrada-Ebel
Thomas Edrington
Sheikh Edrisi
Kathryn Edwards
Owain Edwards
Robert Edwards
Richard Edwards
Claire Edwards
Holly Edwards
Will Edwards
Andrew Edwards
Genea Edwards
Jessie Edwards
Nicholas Edwards
Kate Edwards
Morven Edwards
Aurelie Edwards
Claudia Edwards
Glenn Edwards
S. Gareth Edwards
Katie Edwards
Dan Edwards
Vijay Kumar Eedunuri
Iris Eekhout
Douglas Eernisse
Valerie Eertwegh
Yvonne Efebera
Alfred Effenberg
Jimmy Efird
Dimitar Efremov
Julia Efremova
Nathan Efron
Sol Efroni
Eleni Efstathiou
Stacey Efstathiou
Saeed Eftekhar Azam
Eftekhar Eftekharpour
Suhelen Egan
Susan Egan
Scott Egan
James Egan
Sarah Egan
Mary Egan

Kensuke Egashira
Nobuaki Egashira
Naohiro Egawa
Markus Ege
Gustavo Egea
Pascal Egea
Louise Egerton-Warburton
Arne Egesten
Howard Egeth
Elianne Egge
Torbjørn Eggebø
Adam Eggebrecht
Matthias Eggel
Jan Egger
Boris Egger
Marcel Egger
Jos Egger
Julia Eggermann
Jos Eggermont
Lori Eggert
Thomas Eggert
Anja Eggert
Waltraud Eggert-Kruse
Linda Eggertsen
Hafsteinn Eggertsson
Dirk Eggink
Karen Eggleston
Paul Eggleton
Lindstrom Egholm
Pirooz Eghtesady
Marcel Egli
David Eglin
Natalia Egorova
Tewodros Eguale
Satoru Eguchi
Tomoharu Eguchi
Yuichiro Eguchi
Akiko Eguchi
Yutaka Eguchi
Kazuo Eguchi
Takashi Eguchi
Hisashi Eguchi
Yawara Eguchi
Víctor Eguíluz
Oluwaseun Egunsola
Akira Ehara
Matthias Ehebauer
Reinhard Eher
Jan Ehlers
Frederick Ehlert
Petra Ehling
J. Ehling
Monika Ehling-Schul
Jens Ehmcke
Måns Ehrenberg
Ann Ehrenhofer-Murray
Sam Ehrenreich
Miriam Ehrensaft
Vera Ehrenstein
Günter Ehret
Anita Ehret
Christina Ehrhardt
Anja Ehrhardt
Harald Ehrhardt
Frédéric Ehrler
Garth Ehrlich
Stefan Ehrlich
Sabine Ehrt
Jan Ehses
Rudolf Ehwald
Matthias Eiber
Maryna Eichelberger
Ursula Eichenlaub-Ritter
Pieter Eichhorn
Götz Eichhorn
Sabine Eichinger
Jerry Eichler
Wolfram Eichler
Jessica Eichmiller
Meri Eichner
Dirk Eick
Sigrun Eick
Michael Eickermann
Carsten Eickhoff
Jesse Eickholt
Oliver Eickmeier
Michael Eid
Assaad Eid
Ali Eid
Mohamed R Eid
David Eide
Arne Eide
David Eidelberg
Martin Eiden
Maribeth Eiden
Lee Eiden

Pernille Eidesen
Satoshi Eifuku
Felix Eigenbrod
Rachel Eiges
Sato Eiichi
Morten Eike
Fritz Eilber
Alexander Eiler
Jens Eilers
Martin Eimer
Einar Einarsson
Gisli Einarsson
Baldvin Einarsson
Hermann Einsele
Mark Einstein
Daniel Einstein
Sigurd Einum
Alvaro Eiras
Sonia Eiras
Anne Eischeid
Ellen Eisen
Kathleen Eisenach
Michael Eisenbach
Jonathan D. Eisenback
Michael Eisenberg
Carol Eisenberg
Daniel Eisenberg
Naomi Eisenberger
Steffen Eisenhardt
Dorothea Eisenhardt
Nico Eisenhauer
Graeme Eisenhofer
Michael Eisenhut
Arik Eisenkraft
Toby Eisenstein
Tzipora Sarah Karin Eisinger
Andreas Eisingerich
Manuel Eisner
Brian Eisner
Wolfgang Eisterer
Daniel Eitzman
Elisenda Eixarch
Georgia Eizenga
Emmanuel Ejim
Keisuke Ejima
Aki Ejima
Anna Ek
Weronica Ek
Uwemedimbuk Ekanem
Solvig Ekblad
Robert Ekblom
Elin Ekblom-Bak
Veena Ekbote
Karl Ekdahl
Andras Eke
Christine Ekenga
Tahir Ekin
Panteleimon Ekkekakis
Stephen Ekker
Marc Ekker
Johan Eklof
D. Magnus Eklund
Simon Ekman
Inger Ekman
Francis Eko
Erika Eksioglu
Jorgen Ekstrom
Marie Ekstrom
Daisuke Ekuni
Elie El Agha
Sahar El Aidy
Hicham El Alaoui
Alison El Ayadi
Jamila El Baghdadi
Charbel El Bcheraoui
Regina El Dib
Khaled El Emam
Zaki El Fiky
Alicia El Haj
Nady El Hajj
Chaker El Kalamouni
Imen El Karoui
Marc El Khoury
Malek El Muayed
Abdelfattah El Ouaamari
Nihal El Rouby
Adil El Taghdouini
David Elad
Elahe Elahi
Varalakshmi Elango
Moustafa El-Araby
Martyna Elas
Dorraya El-Ashry
Mohamed Elasri
Zvulun Elazar
Tatiana El-Bacha

Hossein Elbadawy
Medhat Elbadry
Husni Elbahesh
Nabila El-Bassel
Michael Elbaum
Rivka Elbaum
Mohamad Elbaz
Ali Elbehery
Ulf Elbelt
Abd El-Fattah El-Beltagy
Paul W. Elbers
Thomas Elbert
Randolph Elble
J. Stuart Elborn
Samir El-Dahr
John Elder
James Elder
James Elderfield
Eric Eldering
Ann Eldh
Bjarki Eldon
Hamza El-Dorry
Felice Elefant
John Elefteriades
Florent Elefteriou
Ioannis Eleftherianos
Zoltán Elek
Coen Elemans
Bassam El-Eswed
Anna Maria Eleuteri
Mohammed Elfaramawi
Samia Elfekih
Cristiane Elfes
Henk Elffers
Marlies Elfrink
Marcus Elfstrom
Frank Elgar
Mohamed El-Gazzar
Mohamed Elgendi
Mohamed Elgendy
Bahaa El-Gendy
Ahmed El-Geneidy
Christian Elger
Ghassan El-Haddad
Mohamed Elhadidy
Nazira El-Hage
Jeff Elhai
Eran Elhaik
Karin Eli
Nadia Elia
Leonardo Elia
Theodore Eliades
Damian Elias
Peter Elias
Rosilene Elias
Riad Elias
Harold Elias
Marek Elias
Jorge Elias Jr
Montserrat Elías-Arnanz
Veronica Eliasson
Nicole Eling
Elaine Elion
Roman Eliseev
Irina Eliseeva
Rossella Elisei
Celina Elissondo
Nabiwemba Elizabeth
Abdallah Elkhal
Karen Elkins
Carrie Elks
Philip Elks
R. Ellahi
K. El-Laithy
Timothy Ellam
Anne Ellaway
Ali Ellebedy
Marianne Ellegaard
Lea Ellegaard-Jensen
Susan Ellenberg
Tom Ellenberger
Mark Ellenbogen
Johanne Ellenbroek
Michael Eller
Kathrin Eller
Philipp Eller
David Ellerby
Sandrine Ellero-Simatos
Christina Ellervik
Adam Ellery
Skander Elleuche
Enrique Elli
Lesley Ellies
Helga Ellingsgaard
Andrew Ellington
Simon Elliot

Marie Elliot
Andrew Elliot
Joshua Elliot
Digby Elliott
Richard Elliott
James Elliott
Steve Elliott
Kyle Elliott
David Elliott
Jennifer Elliott
Chris Elliott
Natalie Elliott
Sean Elliott
Doug Elliott
Paul Elliott
Robert Elliott
Daniel W. Elliott
Lee Ellis
Ronald Ellis
Jeff Ellis
James Ellis
Tom Ellis
Julie Ellis
Chris Ellis
Terry Ellis
Vincenzo Ellis
David Ellis
Jessica Ellis
Carla Ellis
Wendy Ellis
Richard Ellis
Peter T Ellison
Amanda Ellison
Amy Ellison
Matt Ellison
David Ellison
James A. Ellison
Stephen Ellner
David Ellsworth
Buffy Ellsworth
Patrick Ellsworth
Peter Ellsworth
Sriramulu Elluru
Nadia El-Mabrouk
Ahmed Elmarakby
Sammy Elmariah
Mahmoud El-Mas
Moamen Elmassry
Stefan Elmer
Andrew Elmore
Lynne Elmore
Adel El-Naggar
Adel El-Nashar
Wassim El-Nemer
Mohammad N. Elnesr
Laura Elnitski
Yusra Elobaid
J. Eloff
Arne Elofsson
Marc Eloit
Aino-Maija Eloranta
Felix Elortza
Marko Elovainio
Micah Elovitz
Sabine Elowe
Christophe Eloy
Ani Eloyan
Marwa Elrakaiby
Mona Elrefai
Azza El-Remessy
John Elrod
Marwan El-Sabban
Hs Elsarraj
Hend Elsayed
Salah Elsayed
Abdulrahman El-Sayed
Yasser El-Sayed
Julia Sarah El-Sayed Moustafa
Hassan El-Sayyad
Mattijs Elschot
Manuela Elsen
Hatem Elshabrawy
Mostafa Elshahed
Wael Elshamy
Ahmed El-Shamy
Omar El-Sherif
Ayman El-Shibiny
Christoph Elsing
Gerrit Elsinga
Paul Elsinghorst
Ann Elsner
Bernhard Elsner
Deborah Elstein
Marcus Elstner
Lindsay Eltis
Sakina Eltom

Viswanathan Elumalai
Naveen Eluru
Vinod V. Elverfeldt
Dominik V. Elverfeldt
Christoper Elvidge
Chris K. Elvidge
Rune Elvik
C Elvira
Rani Elwy
Ramze Elzahrany
Bernet Elzinga
Masatsugu Ema
Peter Emanuel
Linda Emanuel
Enzo Emanuele
Tatiana Emanuelli
Monica Embers
Lauren Emberson
Lonnie Embleton
Marina Emborg
Michael Emch
Luni Emdad
Michele Emdin
Connor Emdin
John Eme
Felix Emele
Stanislav Emelianov
Emmanuel S. Emelogu
Jonathan Emens
Guillaume Emeriaud
Charles Emerson
Patrick Emery
Paul Emery
Ben Emery
Neil Emery
Vince Emery
Rob Emery
Ann Emery
Derek Emery
Richard Emes
Sherif Emil
Jean Francois Emile
Chimusa Emile Rugamika
Sabine Eming
Guenova Emmanuella
Thomas Emmel
Tamara Emmenegger
B. J. Emmer
Louise Emmerson
Kirsten Emmert
Michael Emmert-Buck
Frank Emmert-Streib
Emily Emmott
Stephane Emond
Jennifer Emond
Stephane Emonet
Noriaki Emoto
Nuno Empadinhas
Scott Emrich
Teri Emrich
Theresa Emser
Eve Emshwiller
Gunilla Enblad
Jorge Encarnação
Teresa Encinas
Juan Encinas
Ignacio Encío
Gabriele Ende
Heiko Enderling
Leah Enders
Ulrike Endesfelder
John Endler
Wagner Endo
Itaru Endo
Satoshi Endo
Tomoyuki Endo
Keigo Endo
Zoltan Endre
Bryan Endress
Janice Endsley
Bogdan Ene-Iordache
Eric Enemark
Meric Enercan
Morten Enersen
Christian Enevold
Charis Eng
Ralf Engbert
David Engblom
Andreas Engel
Astrid Engel
Michael Engel
Jerome Engel
Elisabeth Engel
Pablo Engel
James Engel
Paul Engel

Felix Engel
Stephanie Engel
Christoph Engel
Kurt Engeland
Hanna Engelberg-Kulka
Jurgen Engelberth
Daniel Engelbertsen
Jill Engel-Cox
Martin Engelhard
Brabara Engelhardt
Julia Engelhorn
Alan Engelman
Julia Engelmann
Jan Engelmann
Robby Engelmann
Susanne Engelmann
Yvonne Engels
Tim Engels
Bevin Engelward
Richard Engeman
Denis-Alexander Engemann
Haakon Engen
Florian Engert
Richard Engh
James Engle
James Englehardt
Eric Engleman
Jan Engler
Coralie English
Karen English
Megan English
Dario Englot
David Engman
Francisco Enguita
Alfredo Enguix-Armada
Grigori Enikolopov
Jacob Enk
Perenlei Enkhbaatar
Mats Enlund
Irene Ennis
Richard Ennos
Murray Enns
Eva Enns
Hideki Enokida
Noboyuki Enomoto
Hirayuki Enomoto
Daniel Enquobahrie
Delia Enria
Pablo Enriori
Jose Antonio Enriquez
Amalia Enríquez-De-Salamanca
Christian Enriquez-Olguin
Emilia Entcheva
Peter Enticott
Sally Entrekin
Sonja Entringer
Yeetey Enuameh
Volker Enzmann
Young-Ho Eom
Ralph Epaud
Elissa Epel
Boris Epel
Anne Ephrussi
Mariastella Epifanio
Sabrina Epiphanio
Vasso Episkopou
Willemijn Eppenga
Michael Epperly
Anna Epperson
Eric Epping
Ben Eppinger
Hans Joerg Epple
John Eppley
Patience Epps
Suzanne Epstein
Jonathan Epstein
Leonard Epstein
David Epstein
Paul Epstein
Yoram Epstein
Kai Epstude
Tracy Epton
Tze-Kiong Er
Satish Eraly
Marcel Eras
Rajiv Erasmus
Peter Erb
Klaus Erb
Matthias Erb
Tobias Erb
Laurie Erb
Holger Erb
Tomris Erbas
Raimund Erbel
Wendy Erber
Slaven Erceg

Ae Ercin
Barbaros Erdal
Ebru Erdemir
Ozlem Erden
Péter Érdi
Erland Erdmann
Nathan Erdmann
Friederike Erdmann
Deana Erdner
Suna Erdogan
N. Erdol
Mesut Erdurmus
Metin Eren
Tunc Eren
Jekaterina Erenpreisa
Deniz Erezyilmaz
Gisela Erf
Paul Erftemeijer
Marc Erhardt
Robert Erhardt
Rajaraman Eri
Harvill Eric
Ghigo Eric
Robert Erickson
David Erickson
Brian Erickson
Jeff Erickson
Pamela Erickson
Per Ericson
Göran Ericsson
Jason Eriksen
Erik Fink Eriksen
Renée Eriksen
Niclas Eriksson
Anders Eriksson
Lars E. Eriksson
Charli Eriksson
Kimmo Eriksson
Sofi Eriksson
Joakim Eriksson
Ulf Eriksson
Ivan Erill
Derek Erion
Brad Erisman
Mert Erkan
H. Verda Erkizan
Cihan Erkut
Armin Erlacher
Martin Erlandson
Kristine Erlandson
Judith Erlen
Janine Erler
Sten Erm
Miikka Ermes
Ulrich Ermler
Megan Ermler
Vladimir Ermolayev
Thomas Ernandez
Paul Erne
Philipp Erni
Oliver Ernst
Raffael Ernst
Ulrich Ernst
Alexandra Ernst
Robert Ernst
Kacey Ernst
Cagla Eroglu
Almila Erol
Boban Erovic
Gunay Erpul
Roberto Erro
Nilüfer Ersan
Hege Ersdal
Robert Erskine
William Erskine
Jennifer Erskine-Ogden
Elif Erson
Elhan Ersoz
Gokhan Ertaylan
Adam Ertel
Cesim Erten
Jenni Ervasti
Lars-Peter Erwig
W. Mark Erwin
Erica Erwin
Christopher Erwin
Yigitcan Eryaman
Serpil Erzurum
Andrew Esbaugh
Anna Esbensen
Ananias Escalante
Bruno Escalante
Pablo Escandell-Montero
Patricia Escandon
Julia Escandon
Ricardo Escarcega

Carole Escartin
Romain Eschalier
Michael Eschbaumer
Thomas Eschenhagen
Steven Eschrich
Michael Esco
Carolina Escobar
Ana Escobar
José Arturo Escobar
Federico Escobar
Jorge Escobar Camargo
Alejandro Escobar-Gutiérrez
Héctor Escobar-Morreale
Jorge Escobedo
Carlos Escobedo
Nicolas Escoffier
Joan Carles Escola-Gil
Sylvie Escolano
David Escors
Julio Escribano
José Escribano
Joaquin Escribano
Gema Escribano-Avila
Marcial Escudero
Daniel Escudero
Alejandro Escudero
Renaud Escudie
Chikezie Eseonu
Ehsan Esfahani
M Esfahanian
Manal Eshelli
Ladan Eshkevari
Emily Eshleman
Mehrzad Eskandari
Simon Eskildsen
Changiz Eslahchi
Mohammed Eslam
Medi Eslani
Guy Eslick
Philippe Esling
Saber Esmaeili
A. Esmaoglu
Gamal Esmat
Sean Esmende
Charles Esmon
Stephane Esnault
Vincent Esnault
Edgar Espana
N. Joseph Espat
Björn Espedido
Marianne Espeland
Chris Espelin
Luca Espen
Annette Esper
Mariano Esperatti
Toomas Esperk
Eduardo Espeso
Antonio Espín
Virginia Espina
Jesús Espinal-Enríquez
Eduardo Espinar-Escalona
Ana Espinel-Ingroff
Ana Espinola
Manuel Espinosa
Alexander Espinosa
Carlos Espinosa
Diego Espinosa
Laura Espinosa
Javier Espinosa-Aguirre
Carmen Espinosa-Gongora
Georgina Espinosa-Lopez
Ricardo Espinosa-Tanguma
Luis Espinoza
Bertha Espinoza
Andreas Espinoza
Alejandro Espinoza Orías
Ramón Espinoza-Lewis
Ana Espirito-Santo
Clara Espitia
Luciana Esposito
Carla Lucia Esposito
Federica Esposito
Roberto Esposito
Antonio Esposito
Elga Esposito
Irene Esposito
Maria Grazia Esposito
Gianluca Esposito
Giovanni Esposito
Marcello Esposito
Alfonso Esposito
Emanuela Esposito
Roberto Esposti
Mark Espy
Jeremy Esque
Aurora Esquela-Kerscher

Charles Ess
Musthafa Essa
Ahmed Essaghir
Adib Essali
Kobina Essandoh
Jake Esselstyn
Marissa Esser
Charlote Esser
Marieke A.G. Essers
M. Essex
Marie-Louise Essink-Bot
Jeffrey Essner
Edward Essuman
Joan Estany
Candelaria Estavillo
Sergio Estay
José Esté
David Esteban
Maria Angeles Esteban
Jaime Esteban
Irene Esteban
Jean-Pierre Estebe
Marcos Estecio
Mark Estelle
Joan Estelrich
Jeremie Estepp
Bradley Estes
Cristina Esteva Font
Pilar Esteve
Jordi Esteve
Maria Esteve-Gassent
Enrique Estevens-Navarro
Joaquim Esteves Da Silva
José Estevez
Charles Estill
Ana Estrada
Teresa Estrada-Garcia
Agustin Estrada-Pe±A
Michelle Estrella
Anthony Estrera
Dario Estrin
Catherine Etchebest
Adolfo Etchegaray
Andrew Etchell
Heather Etchevers
Kenji Etchuya
Mohammad Etemadi
Paula Eterovick
Steen Ethelberg
Iryna Ethell
Christopher Etherton-Beer
Stephen Ethier
Thomas Ethofer
Sandrine Etienne-Manneville
Dominik Ettlin
Rüdiger Ettrich
Paul Eubig
François Eudes
Eliseo Eugenin
Sung Eum
Young Gyu Eun
Eva Eva Schmelzer
Maggie Evan
Andrew Evan
Constantinos Evangelinos
Adriane Evangelista
Luis Evangelista
Dominic Evangelista
Stefano Evangelista
Iordanis Evangelou
Evangelos Evangelou
Ben Evans
Karl Evans
Karen Evans
Sylvia Evans
David Evans
Jennifer Evans
Jay Evans
Carlton Evans
Kate Evans
Karla Evans
Peter Evans
Denise Evans
Robert Evans
Christian Evans
Sam Evans
Gareth Evans
Luke Evans
Charles Evans
Richard Evans
John Evans
Elizabeth Evans
Ellen Evans
Sarah Evans
Simon Evans
Kelly Evans

Tom Evans
Caroline Evans
Ra Evans
Carmella Evans-Molina
Artem Evdokimov
Patrick Even
Maya Evenden
Pieter Evenepoel
Ann Evensen
Jonas Everaert
Paul H Everest
Gabriel Everett
Eric Everett
Helen Everett
Charles Everett
Thomas Everett, Iv
Sydney Everhart
S. Everingham
Carol Everson
Alison Every
Sebastian Eves-Van Den Akker
Michael Evgen'Ev
Antonio Evidente
A. Eden Evins
Alexander Evins
Sophie Evison
Stephane Evoy
Cuneyt Evren
Denis Evseenko
Sarah Ewald
Katie Ewer
Michael Ewers
Rob Ewing
James Ewing
Holly Ewing
Filippos Exadaktylos
Laurent Excoffier
Vernat Exil
Christopher Exley
Elodie Ey
Jonathan Eya
Gal Eyal
Sana Eybpoosh
Claire Eyers
Patrick Eyers
Willard Eyestone
Lisa Eylerd
Nir Eynon
Toshihiko Ezashi
Tariq Ezaz
Exequiel Ezcurra
Martin Ezcurra
Christian Ezeala
Vanessa Ezenwa
Nicole Ezer
Tal Ezer
Lara Ezquerra
Laura Ezquerra
Leila Ezzat
Mohamed Ezzelarab
Hanane Ezzouine
Claudio F Donner
Rui Fa
Mauro Fa'
Gavino Faa
Kay Faaberg
Guido Faas
Marijke Faas
Marco Faasse
Elsa Fabbretti
Alessia Fabbri
Elisa Fabbri
Carlo Fabbri
Francesco Fabbri
Cornelius Faber
Milosz Faber
Richard Fabes
João Fabi
Francois Fabi
Claudia Fabiani
Madeleine Fabic
Melodina Fabillo
Adriana Fabra
Nidia Fabre
Jorge Fabres
Mario Fabri
Anthony Fabricatore
Katharina Fabricius
Gabriel Fabricius
Luca Fabris
Laura Fabris
Zeri Fabrizio
Ben Fabry
Bonto Faburay
Arnoldo Facanha
Angelo Facchiano

Andrea Facchinetti
Livia Facchinetti
Angelo Facchini
Lúcia Faccioli
Marc Facciotti
Massimo Faccoli
Laura Fachal
Angela Fachel
Oliver Fackler
Pam Factor-Litvak
Elisa Fadda
Andrey Fadeev
Mohamed Fadel
Hind Fadel
Marianela Fader
João Fadista
Amin Fadl
James M. Fadool
Martin Faehling
Mario Faenza
Gary Faerber
Marina Faerman
Misagh Faezipour
Susanna Fagerholm
Michel Fages
Anne Fages
Serena Faggiano
Clovis Faggion
Mohammad Faghihi
Angela Fago
Sharmila Fagoonee
Nelson Fagundes
Rodrigo Fagundes Braga
Sheena Faherty
Robert Fahey
Saman Fahimi
Ashkaan Fahimipour
William Fahl
Richard Fahlman
Suhaib Fahmy
Aly Fahmy
Margaret Fahnestock
Astrid Fahrleitner
Gregory Fahy
Siu Fai Lee
Jozsef Fail
Cristina Failla
Osvaldo Failla
Dorothée Faille
Arnaud Faille
Paola Failli
Roy Faiman
Patricia Fair
Trudee Fair
Jeanne Fair
Sue Fairbanks
Will Fairbrother
Christoffer G Fairburn
Stuart Fairclough
Rick Fairhurst
Merle Fairhurst
Anna Marie Fairhust
Christopher Fairley
Neil Fairweather
Delisa Fairweather
Stefano Fais
Syed Faisal
Fazle Faisal
Francesco Faita
Dan Faith
Alain Faivre-Chauvet
Dennis Faix
Inmaculada Fajardo
Gorazd Fajdiga
Alexander Faje
Lenka Fajkusová
Ireti Fajolu
Merritt Fajt
Frida Fåk
Stylianos Fakas
Fadi Fakhouri
Ahmad Fakhoury
Elham Fakhrejahani
Rawil Fakhrullin
F. Fakhry
Mohamad Fakih
Giannoulis Fakis
David Fakunle
Patrizia Falabella
A. G. Falade
Monica Falautano
Jack Falcon
Maria Inez Falcon
Rosse Mary Falcon-Antenucci
Franco Falcone
Germana Falcone

Erin Falcone
Emilia Falcone
Stuart Falconer
Massimo Falconi
Howard Falcon-Lang
Elizabeth Falendysz
Hanne-Lise Falgreen Eriksen
Tiago Falk
Roni Falk
Jamie Falk
Ronald Falk
Falk Falk Müller-Riemenschneider
Joseph Falke
Jeffrey Falke
Malin Falkenmark
Peter Falkingham
Joseph Falkinham
Torbjörn Falkmer
Daniel Falkstedt
Tove Fall
Magnus Fall
Mamadou Fall
Deborah Falla
Mazyar Fallah
Nefer Fallico
Petra Fallier-Becker
Maria Fallman
James Fallon
Stewart Fallon
Pål Falnes
Matthieu Falque
James Falter
Andras Falus
Daniel Falush
Michael Falvo
Dennis Falzon
Barbara Fam
Mary Familari
Hannah Family
Nicholas Famoso
Thomas Famula
Chia Fan
Longjiang Fan
Jin Fan
Hugh Fan
Jie Fan
Wen-Lang Fan
Bao Jian Fan
Qi-Wen Fan
Timothy Fan
Xiujun Fan
Chenguang Fan
Chunhai Fan
Yuchen Fan
Zhen Fan
Hong-Jie Fan
Ze-Xin Fan
Daping Fan
Shengjun Fan
Huizhou Fan
Xiaobing Fan
Xingjun Fan
Yunpeng Fan
Chuandong Fan
Guoqiang Fan
Shufang Fan
Fan Fan
Chuchuan Fan
Songqing Fan
Zaifeng Fan
Tung-Yung Fan
Qiao Fan
Andy Fan
Ying Fan
Yue Fan
Meiyun Fan
Xiucheng Fan
Jingyao Fan
Jun Fan
Lin Fan
Hui Wen Fan
Hanqing Fan
Qiyong Fan
Xinxin Fan
Zhenxin Fan
Suohai Fan
Ling Fan
Yong Fan
Qipeng Fan
Youran Fan
Peilei Fan
Xiaorong Fan
Zhiqiang Fan
Pi-Chuan Fan
Gaoqiong Fan
Tinglun Fan

Jian-Gao Fan
Ke Fan
Jibiao Fan
Dayong Fan
Victoria Fan
Dong S Fan
Wenyi Fan
Roberto Fancellu
Bronwyn Fancourt
Yana Fandakova
Joachim Fandrey
Carmine Fanelli
Vito Fanelli
Caterina Fanello
Francesco Fanfulla
Shengyun Fang
Fang Fang
Bingliang Fang
Chi-Tai Fang
Weiyi Fang
Jianwen Fang
Rong Fang
Yue Fang
Jia Fang
Xiaoguang Fang
Qiang Fang
Haoshu Fang
Jun Fang
Tiegang Fang
Zhang Fang
Zhuo Fang
Boping Fang
Gang Fang
Ying Fang
Bin Fang
Huasheng Fang
Lei Fang
Rui Fang
Qingming Fang
Wenwen Fang
Shona Fang
Congbing Fang
Xiuqi Fang
Wei Fang
Yanjun Fang
Xiangling Fang
Angela Fang
Hua-Chang Fang
Yaping Fang
Yongliang Fang
Pu Fang
Wen Fang
Meiying Fang
Haitong Fang
Ma Fangrui
Sarah Fankhauser
Cathy Fann
Séamus Fanning
Kent Fanning
Manoussa Fanny
Dimitrios Fanourakis
Jeremie Fant
Charles Fant
Paolo Fanti
Maria Pia Fantini
Maria Livia Fantini
Pier Lorenzo Fantozzi
Alessandro Fanzani
Jessica Fanzo
Helisson Faoro
Cristiano Farace
Mary Farach-Carson
Frank Faraci
Giuseppe Faraco
Mohamed Farag
Ehab Farag
Eshel Faraggi
Eric Faragher
Sándor Faragó
Rami Farah
Mansour Farahani
Farzam Farahmand
Davide Faranda
Nicoletta Faraone
David Faraoni
Thomas Faraut
Norman Farb
Steven Farber
Mark Farber
Marie-Laure Fardeau
Laurence Fardet
David Fardo
Silva Fare
Jawad Fares
Diego Farfan-Arribas
Juan Fargallo

Laura Fargas
Kyle Fargen
Olivier Farges
Silvia Fargion
S. M. Farhad
Maha Farhat
T. Farhat
Rita Faria
Nuno Faria
Christina Faria
Amanda Faria
Izeni Farias
Amro Farid
Mohammad Hafeez Faridi
Erik Farin
Lorenzo Farina
Nicholas Farina
Antonio Farina
Edgardo Farinas
Jose Fariñas-Franco
Paulo Farinatti
Carlos Farinha
Donatella Farini
Alessandro Farini
Justin Faris
Robert Faris
Piero Fariselli
Robert Fariss
Vinicius Farjalla
Krysten Farjo
Laszlo Farkas
Illés Farkas
Michael Farkas
Imre Farkas
Igor Farkas
Andrew Farke
Farzad Farkhooi
Tim Farley
Jason Farley
Edward Farley
Roger Farley
Theodora Farmaki
Evangelia Farmaki
C. G. Farmer
Cg Farmer
Cristan Farmer
Brad Farmilo
Hanna Farnelid
Anna Färnert
Mark Farnham
Amir Farnoud
Hamidreza Farnoush
Jose Faro
J. Farokhzadian
Matthew Faron
Amjad Farooq
Muhammad Farooq
I. Farooq
Sebastian Farquhar
Tracy Farr
Lee Farrand
David Farrar
Genevieve Farrar
Mark Farrar
Eva Farre
Ramon Farre
Magí Farré
Marta Farré
Tony Farrell
Geoffrey Farrell
Lindsay Farrell
Lindsay Farrer
Ken Farrington
Darise Farris
Alton Farris
Emmanuele Farris
Paul Farrow
John Farrow
Nicolas Farrugia
Konstantinos Farsalinos
Mazda Farshad
Amir Farshchi
Sandra Helena Farsky
Kelly Farwell
Robyn Fary
Faranak Farzan
Antonio Fasanella
Mauro Fasano
Laurent Fasano
Olufunke Fasawe
Andrea Fascetti
Folorunso Fasina
Cristina Fasolato
Dionysia Fasoula
Thijs Fassaert
Matteo Fassan

Anna Fassio
Jan Fassler
Patricia Fast
Rosamaria Fastuca
Marc Fatar
Enrique Fatas
Suzanne Fateh-Moghadam
S. Hossein Fatemi
Human Mousavi Fatemi
Ramzi Fatfouta
Brian Fath
Amir Fathi
Sumana Fathima
Yaghoub Fathollahi
Mar Fatjo Vilas
George Fatseas
Quek Fatt
Julien Fattebert
Liana Fattore
Lanfranco Fattorini
Lorenzo Fattorini
Sébastien Faucher
Nathalie Faucheux
Oliver Faude
Eric Faudry
Christian Faul
Anna Faul
Andrew Faulkner
Sarah Faulwetter
Patricia Fauque
Christophe Faure
Sebastien Faure
Adrien Faure
Daniel Faurholt-Jepsen
Jean-Michel Faurie
Gilles Faury
Sascha Fauser
Alexander Faussner
Karoline Faust
Rudolf Faust
Annunziata Faustini
Massimiliano Faustino
André Faustino
E. Vincent S. Faustino
Maria Amparo F. Faustino Faustino
Elaine Faustman
François Fauteux
Michael Fautsch
Xavier Fauvergue
Cristiano Fava
Luca Fava
Giammarco Fava
Bartolo Favaloro
Angela Favaro
Lorenzo Favaro
Phelipe Favaron
Paulo Jc Favas
Loic Favennec
Benoit Favier
François B. Favier Favier
Geraldine Favrais
Ophelie Favrod
Gloria Fawcett
Janet Fawcett
Christine Fawcett
Amani Fawzi
Elisabeth Faxelid
Michael Fay
Zahi Fayad
Albert Faye
Nathalie Faye
Franz Fazekas
Seena Fazel
Alireza Fazeli
Pk Fazeli
Serafino Fazio
Pietro Fazzari
Thomas Fazzio
Antonio Fea
Linda Feagins
Mark Fear
Howard Fearnhead
Rachel Fearns
Philip Fearnside
Ursula Fearon
Jeffrey Fearon
David Feary
Thomas Feasby
Nicholas Feasey
Mark Featherstone
Helen Featherstone
Mark Febbraio
Maria Febbraio
Gérard Febvay
Benedikt Fecher
Michael Fedak

George Fedak
Alessandro Fedele
Carmine Fedele
Ugo Fedeli
David Feder
Lisa Federer
Augusto Federici
Maurizio Federico
Paolo Federico
Lorenzo Federico
Michael Federle
Linda Fedigan
Inna Fedorenko
Ken Fedorka
Andriy Fedorov
Vadim V. Fedorov
Artur Fedorowski
Pawel Fedurek
Bradley Fedy
Alan Fedynich
Martin Feelisch
Kristen Feemster
Margaret Feeney
Eoin Feeney
Bronwyn Fees
Gregory Fegan
Narelle Fegan
Brent Fegley
Michael Fehlings
Todd Fehniger
Anthony Fehr
Jill Fehrenbacher
Lars Fehren-Schmitz
Shui-Zhang Fei
Xiang Fei
Gao Fei
Qiang Fei
Robert Feidenhans'L
Larry Feig
Heather Feigelson
Sara W. Feigelson
Andrea Feigl
Li Fei-Ka
Edward Feil
Hannes Feilhauer
Irwin Feinberg
Adam Feinberg
Jeremy Feinberg
Michelle Feinberg
John Feiner
Ofer Feinerman
Alan Feingold
Beth Feingold
Insa Feinkohl
Adam Feist
Matheus Feitosa
Gyorgy Fejer
Laura Fejerman
Imre Fejes
Marlena Fejzo
Abebaw Fekadu
Donna Fekete
Csaba Fekete
A. Fekete
Arnaud Fekkar
Horst Felbeck
Jordan Feld
Paul Feldblum
Michael Felder
Robin A. Felder
Marie-Paule Felder-Schmittbuhl
Kevin Feldheim
David Feldheim
Marita Feldkaemper
Marcus Feldman
Henry Feldman
Laurie Beth Feldman
Raisa Feldman
George Feldman
Inna Feldman
Yuri Feldman
Steven R. Feldman
Oriel Feldmanhall
Kyriacos Felekkis
Michel Feletou
M. Feletou
Denis Feliers
María Felipe-Lucia
Paulo Felisberto
Janine Felix
David Fell
Jack Fell
Daniel Felleman
Klaus Fellermann
John Fellers
Emanuela Felley-Bosco

Martin Fellner
Gidon Felsen
Diane Felsen
Pedro Femia Marzo
Forough Fendereski
Wolfgang Fendler
Liane Fendt
Sarah-Maria Fendt
Peter Feng
Gen-Sheng Feng
Mingqian Feng
Youjun Feng
Jian Feng
Rui Feng
Qili Feng
Wei Feng
Wenke Feng
Gu Feng
Yan Feng
Bo Feng
Yulong Feng
Shi-Qing Feng
Yucheng Feng
Dai Feng
Tingyong Feng
Chunliang Feng
Bin Feng
Biao Feng
Yaoyu Feng
Yi Feng
Ling Feng
Baomin Feng
Qianjin Feng
Shi-Ting Feng
Zhiyong Feng
Yuanming Feng
Ying Feng
Hai-Zhong Feng
Youzhi Feng
Xiaomei Feng
Hao Feng
Zhengzhi Feng
Lihua Feng
Pingfu Feng
Shengbo Feng
Song Feng
Wen-Hai Feng
L. Feng
Liangbing Feng
Liu Feng
Changchun Feng
Nan Feng
Zongdi Feng
Hailan Feng
Hu Feng
Congjing Feng
Jiang-Hua Feng
Hua Feng
Chenglian Feng
Xin Feng
Zeny Feng
Zhanchun Feng
Gang Feng
Yuxiong Feng
Chao Feng
Jing Feng
Jianmeng Feng
Xiao Feng
Long Feng
Rentian Feng
Huai L. Feng
Tao Feng
Shipeng Feng
Ma Fengwang
Kevin Fennelly
Lukas Fenner
Jack Fenner
Bob Fennis
Maria Fenoglio
Martina Fenske
Katherine Fenstermacher
Brock Fenton
Susan Fenton
Giuseppe Fenu
Eva Fenwick
Eva Fenyvesi
Francesco Feo
Cyrille Feray
Peter Ferdinandy
S. M. Ferdous
Farzad Fereidouni
Peter Ferenci
Thomas Ferenci
Nelli Ferenczi
Shingairai Feresu
Tom Fergus

David Ferguson
Heather Ferguson
Adam Ferguson
Andrew Ferguson
Christopher Ferguson
Carrie Ferguson
Mark Ferguson
Stephen Ferguson
Tanya Ferguson
Deborah Ferguson
Paige Ferguson
Kelly Ferguson
Nicole Ferguson
Peter Ferguson
Susan Ferguson
Alesia Ferguson
Pamela Fergusson
Teresa Feria Arroyo
Guido Ferilli
Gregory Ferl
Edoardo Ferlazzo
Walter Ferlin
Cristiano Ferlini
Pierre Olivier Fernagut
Russell Fernald
Maria Fernanda Laus
Maria Fernandes
Rosa Fernandes
Jorge Fernandes
Elizabeth Fernandes
Natalia Fernandes
Cathy Fernandes
Janaina Fernandes
Carlos Fernandes
Karlette Fernandes
Alexandra Fernandes
Susana Fernandes
João Fernandes
Ana Fernandes
Christopher Fernandes
Sergio Fernandes
Hugo Fernandes
Isabel Fernandes
Carmen Fernandez
Esteve Fernandez
Jose Fernandez
Ana I. Fernandez
Catherine Fernandez
Christian Fernandez
Julian Fernandez
David Fernandez
Pedro Fernandez
Ignacio Fernandez
Monica Fernandez
Oscar Fernández
Luis Ángel Fernández
Victoria Fernández
Jose Fernández
Juan Miguel Fernandez Alvira
Emma Fernández Covelo
Francisco Fernández De Miguel
Ana Fernandez Gonzales
Roberto Fernández Lafuente
Fernando Fernandez Mendoza
Daniel Alejandro Fernandez Velasco
Marcelo Fernandez Vina
Fernando Fernández-Aranda
Daniel Fernandez-Ayala
Maite Fernández-Barrena
Jesualdo Tomás Fernández-Breis
Alfonso Fernandez-Canteli
Albert Fernandez-Chacon
Miguel Fernandez-Del-Olmo
Narcis Fernandez-Fuentes
Borja Fernandez-Gauna
Barbara Fernandez-Going
Covadonga Fernandez-Golfin
Francisco J Fernandez-Gomez
Rodrigo Fernandez-Gonzalez
Nuria Fernandez-Hidalgo
Anabel Fernandez-Iglesias
Esteban Fernández-Juricic
Alfredo Fernandez-Lara
José Antonio Fernández-López
Pablo Fernández-Marcos
Joseph Fernandez-Moure
Eduardo Fernández-Pascual
Cristina Fernández-Portero
Eduardo Fernandez-Rebollo
Delmiro Fernandez-Reyes
Lindsay Fernandez-Rhodes
Daniel Fernandez-Ruiz
Pedro Fernandez-Salguero
María José Fernández-Serrano
Ana Fernandez-Sesma
Enrique Fernández-Taboada

Denise Fernandez-Twinn
Rodrigo Fernandez-Valdivia
Danilo Fernando
Samodha Fernando
Johan Ferno
Philip Fernside
Olivier Feron
Stefano Ferraina
Giovanna Ferraioli
Ludovic Ferrand
Arny Ferrando
Pasquale Ferrante
Maria Ferrante
Andrea Ferrante
Eliseo Ferrante
Katherine Ferrara
Gerardo Ferrara
Maria Antonietta Ferrara
Emilio Ferrara
Lisa Ferrara
Massimo Ferrara
Giuseppe Ferrara
Cleber Ferraresi
Mariano Ferraresso
Annapia Ferraretti
Rhuanito Ferrarezi
Matthew (Matt) Ferrari
Robert Ferrari
Serge Ferrari
Giovanni Ferrari
Daniel Ferrari
Marco Ferrari
Renata Ferrari
Benoit Ferrari
Merari Ferrari
Filippo Ferrario
Joan Ferraris
Elisabetta Ferraro
Angus Ferraro
Angelo Ferraro
Pietro Ferraro
André Ferraz
Maria Ferraz
Ivan Ferraz-Amaro
Enrico Ferrazzi
Sergi Ferre
Elisa Ferre
Juan Ferré
Catterina Ferreccio
Fatima Ferreira
Leonardo Ferreira
Julio Cesar Ferreira
Arthur Ferreira
Daniela Ferreira
Adilson Kleber Ferreira
Andrea Claudia Freitas Ferreira
Vivana Ferreira
Beatriz Ferreira
Danielle Ferreira
Ludmila Ferreira
L. Ferreira
Adaliene Ferreira
Andrêa Jacqueline Ferreira
Carlos Ferreira
Lara De Noranha Ferreira
Arnaldo Ferreira
Carlos Eduardo Ferreira
Pedro Ferreira
Helena Ferreira
Jorge Ferreira
Maria Ferreira
José Alexandre Ferreira
Joseli Ferreira
Eva Ferreira
Marta Ferreira Bastos
Marcelo Ferreira Da Costa Gomes
Augusto César Ferreira De Moraes
José Miguel Ferreira De Oliveira
Patricia Ferreira Monticelli
William Ferrell
Jorge Ferrer
Isidre Ferrer
Elizabeth Ferrer
Ramon Ferrer I Cancho
Luca Ferreri
Laura Ferreri
Richard Ferrero
Miguel Ferrero
Paola Ferrero
Victoria Ferrero
Sergio Ferrero
Ileana Ferrero
Antonio Ferretti
Francesco Ferretti
Elisabetta Ferretti
Todd Ferretti

Stefano Ferretti
Ceres Ferretti
Renata Ferretti-Rebustini
Cleusa Ferri
Nicola Ferri
Claudio Ferri
Gianmarco Ferri
Abigail Ferrieri
Christine Ferrier-Pagès
Maite Ferrin
Deborah Ferrington
Francesco Ferrini
María Ferriol
Michael Ferris
Howard Ferris
William Ferris
S. Ferrite
Noel Ferro
Frank Ferrone
Patrizia Ferroni
Leah Ferrucci
Alberto Ferrus
John Ferry
Allison Ferry-Abee
Anna Fertonani
Beatrice Fervers
Joshua Fessel
Gion Fessel
James Fessenden
Mike Fessing
Thierry Fest
Richard Festa
Marco Festa-Bianchet
Victor Fet
Constantin Fetecau
Lawrence Feth
Jackie Fetherston
J. Fetherston
Sergueï Fetissov
Anna Rita Fetoni
Christopher Fetsch
Anne-Kathrin Fett
Jessica Fetterman
Chris Fettig
Jennifer Fettweis
Gudrun Feuchtner
Ronny Feuer
Joseph D. Feuerstein
Michael Feuerstein
Jean-Paul Feugeas
Georg Feulner
Jamie Feusner
Paul Feustel
Davina Fevery
Lauren Few
David Fewer
Malin Fex
Paul Fey
Garumma Feyissa
Peter Feys
Brendan Ffrench
Charles Ffrench-Constant
Enrico Fiaccadori
Ivan Fiala
Christian Fiala
Milan Fiala
Massimo Fiandaca
Patricia Michelle Fiander
Davide Fiaschi
Francesco Ficetola
Marco Fichera
Raina Fichorova
Claudia Fichtel
Carl Fichtenbaum
Andreas Fichter
Jörns Fickel
Darren Ficklin
Stephen Ficklin
Thiago Fidalgo
Felipe Fidalgo
Morry Fiddler
Paul Fidel, Jr.
Astrid Fidika
Sarah Fidler
Justin Fidock
John Fieberg
Bernd Fiebich
Mark Fiecas
Martin Fieder
Wolfgang Fiedler
Konrad Fiedler
Goeran Fiedler
Joanna Fiedor
Katja Fiehler
Jens Fiehler
Oliver Fiehn

Mark Field
Iain Field
Katie Field
Joshua Field
Richard Fielding
David Fielding
Gregg Fields
Patricia Fields
David Fields
Paul Fields
Aaron Fields
Theodore Fields
David Fiellin
Alessandra Fierabracci
Noah Fierer
Fernando Fierro
Olga Fierro
Mallory Fiery
Rainer Fietkau
Luciano Fietto
Daniela Fietz
Brian Fife
Mario Fific
William Figg
Marc Figge
Matteo Figini
Federico Figliolini
Will Figueira
Maria Helena Figueiral
Camila Figueiredo
Ricardo Figueiredo
Pedro Figueiredo
Patricia Figueiredo
Gustavo Figueiredo
Tatiana Figueiredo
Ana Elisabeth Figueiredo
Mariana Figueiro
Maria-Jose Figueras
Antonio Figueras
Joan Figueras
Cristina Figuerido
Maria Figueroa
Christian Figueroa
Maximiliano Figueroa
Jordi Figuerola
Natale Figura
Andrea Figus
Remond Fijneman
Edith Filaire
Vicenzo Filardi
Ludmila Filaretova
Gregory Filatov
János Filep
Andrew Filer
Clark Files
Luciano Filgueiras
Vyacheslav Filichev
Kristian Filion
Marina Filip
Szymon Filip
João Filipe
Sergio Filipe
Ana Filipa Filipe
Catalin Filipeanu
Aleksandra Filipovic
Milos Filipovic
Vlatka Filipovic Marijic
Maria Laura Filippetti
Massimo Filippi
Marie-Dominique Filippi
Sevasti Filippidou
Antonio Filippin
Nicola Filippini
Reno Filippo
Simon Fillatreau
Aaron Filler
Lukas Filli
Melanie Fillios
Jorge Filmus
Marcel Filoche
Giuseppe Filomeni
Stefania Filosa
Aldo Filosa
Matthias Filter
Katharina Filz
Patrick Finan
Dario Finazzi
Karin Finberg
Caleb Finch
Emma Finch
Brian Finck
Christine Finck
Ola Fincke
Mark Findeis
John Findlay
Michael Findlay

Keisha Findley
Helen Findlow
Paul Fine
Jason Fine
Neil Finer
Philippe Fines
Murray Fingeret
Volker Fingerle
Barbara Fingleton
Chiara Fini
Michael Finiguerra
Veronica Finisguerra
Katja Fink
Valeria Fink
Mitchell Fink
Gregory Fink
Kyle Fink
Susan Finkbeiner
Kathrin Finke
Stefan Finke
Mark Finke
John Finke
Carsten Finke
Zoe Finkel
Alexei Finkelstein
Jacob Finkelstein
Iris Finkemeier
M. Finken
Florian Finkernagel
Anders Fink-Jensen
Michal Finklestein
Barbara Finlay
David Finlay
Brian Finlayson
Anna Finley
Brian Finlin
Aloke Finn
Stephen Finn
Bridgid Finn
Christine Finn
Anna Finnane
Jukka Finne
John Finnell
Silvia Finnemann
Renee Finnen
Constance Finney
Lydia Finney
Mark Finney
Sarah Finocchario Kessler
Eric Finot
Enrico Finotti
Findley Finseth
Jürgen Finsterbusch
Andres Finzi
Claudio Fiocchi
Alexandra Fiocco
Xavier Fioramonti
Emilio Fiore
Arlene Fiore
Christine Fiorello
Marion Fiorentino
Teresa Vanessa Fiorentino
Susana Fiorentino
Francesca Fiori
Antonio Fiorino
Ferdinando Fiorino
Mirta Fiorio
Graziano Fiorito
Romina Fiorotto
Stefano Fiorucci
Nicola Fiotti
Bonnie Firestein
Simon Firestone
Rebecca Firestone
Cyril Firmat
Gábor Firneisz
Cynthia Firnhaber
Cadhla Firth
Roser Fisa
Claudia Fischbach
Colin Fischbacher
Jorge Fischbarg
Gilles Fischer
Egil Fischer
Kael Fischer
Wolfgang Fischer
Ronald Fischer
Julie Fischer
Valentin Fischer
Sabine Fischer
Julia Fischer
Lutz Fischer
Brian Fischer
Joern Fischer
Michael Fischer
William Fischer

Adrian Fischer
Andreas Fischer
Kathleen Fischer
Håkan Fischer
Mareike Fischer
Marlene Fischer
Rebecca Fischer
Dominik Fischer
Kerstin Fischer
Bernard Fischer
Anthony Fischer
Bernd Fischer
Milos Fischer
Rico Fischer
Margaret Fischl
Martin Fischlechner
Susan Fiscus
Sylvain Fiset
Celine Fiset
Alexandre Fisette
Frank Fish
Mark Fishbein
Kenneth Fishbein
Melissa Fishel
Paul Fisher
Shannon Fisher
Diana Fisher
Edward Fisher
Rosemary Fisher
Andrew Fisher
Derek Fisher
Anat Fisher
Jason Fisher
Steve Fisher
Simon Fisher
Lee Fisher
Matthew Fisher
Oliver Fisher
Cynthia Fisher
Matt Fisher
Eric Fisher
Rebecca Fisher
Judith L. Fisher
Pollyanna Fisher-Pool
Joel Fishman
Marco Fisichella
Nicholas Fisichelli
Amy Fiske
Ane Fisker
Iztok Fister
J. Scott Fites
William Fitt
Stephen Fitter
Sylvia Fitting
Laura Fitton
Susan Fitzer
Paul Fitzgerald
J. Ross Fitzgerald
Michael Fitzgerald
Thomas Fitzgerald
David Fitzgerald
Melissa Fitzgerald
Colleen Fitzgerald
Edward Fitzgerald
Annette Fitzpatrick
Meghan Fitzpatrick
Emer Fitzpatrick
Sarah Fitzpatrick
Carolyn Fitzsimmons
Clare Fitzsimmons
Sean Fitzwater
Elisa Fiume
Anthony Fiumera
Elizabeth Fixman
Jon Fjeldså
Ola Fjellström
Maria Elena Flacco
Susanne Flach
Peter Flach
Shelly Flagel
Lee Flagg
Véronique Flamand
Nicolas Flamand
Martin Flamant
Avi Flamholz
Vittoria Flamini
Josef Flammer
J. Flammer
Lisa Flanagan
Katie Flanagan
Neal Flanagan
Timothy Flanigan
John Flannery
Brendan Flannery
Mike Flannigan
Klas Flardh

Aline Flatz
William Flavahan
Jonathan Flavell
Valerie Flax
Abraham Flaxman
Seth Flaxman
S. Flaxman
Patricia Flebbe
Holger Flechsig
Gerd-Uwe Flechsig
Christian Fleck
Johannes Fleckenstein
Paul Flecknell
Tiffany Fleet
Katherine Flegal
Jaroslav Flegr
Abby Fleisch
Shelby Fleischer
Thomas Fleischer
Mario Fleischer
W. Wolfgang Fleischhacker
Monika Fleischhauer
Diana Fleischman
David Fleischman
Robert Fleischmann
Andreas Fleischmann
Sarel Fleishman
John Fleishman
Karen Fleming
Tom Fleming
Robert Fleming
Jessica Fleming
Ronan Fleming
Angeleen Fleming
Patricia Fleming
Roland Fleming
Sherry Fleming
Chris Fleming
Padhraig Fleming
Paul Fleming
Sheila Fleming
Nicole Fleming
David Fleming
Joy Fleming
Stephen B. Fleming
Rachel Fleming
Erik Flemington
Monica Fleshner
Brian Flesner
Steffen Flessa
Paul Fletcher
Jamie Fletcher
Erica Fletcher
Helen Fletcher
M. J. Fletcher
Francis Fleurat-Lessard
Anthony Fleury
Ghozlane Fleury-Bahi
Jaume Flexas
Diego Flichman
Stephanie Fliedner
Antje Flieger
Ulrike Flierl
Klaus Fliessbach
Barry Flinn
Paul Flint
Melanie Flint
Sherry Flint-Garcia
Sylwia Flis
Tatiana Flisikowska
Kevin Floate
D. T. Tyler Flockhart
Monique Floer
Bernhard Floerchinger
Sergio Floeter
Phyllis Flomenberg
Shannon Flood Nichols
Urszula Florczyk
Francisco Florencio
Margarita Florencio
Enrique Flores
Elsa Flores
Eduardo Flores
Gilberto Flores
Oscar Flores
Francisco Flores
Elaine Flores
Carlos Flores
Eugenia Flores Figueroa
F. Flores-De-Santiago
Sergio Flores-Hernández
Carlos Flores-Mir
Amilcar Flores-Morales
Laurence Flori
Timothy Florin
Esther Florin

Monica Florin-Christensen
Igor Florinsky
Alessandro Florio
Antonia Florio
Michelle Floris-Moore
Esther Florsheim
Harald Floss
Jean-François Flot
Vidar Flote
Andres Floto
Christodoulos Floudas
Sarah Flowers
Bruce Floyd
Bernhard Flucher
Christa Fluck
Leif Fluehe
Tyrel Flügel
David Fluharty
Ad Fluit
Sabine Flury
Joel Fluss
Nicholas Flyger
Lawrence J. Flynn
James Flynn
Charles Flynn
Jacqueline Flynn
Theodore Flynn
Heather Flynn
Aaron Flynn
Simon Foale
Aaron Fobian
Stefano Focardi
Margit Focke-Tejkl
Andreas Focks
Vito Fodera
Matthias Foellmer
Frank Foerster
Steffen Foerster
Ismael Fofana
Mariam Fofana
Paolo Fogagnolo
Michael Fogarty
Laurel Fogarty
Leonardo Fogassi
Elizabeth Foglia
Vincenzo Fogliano
Agnes Fogo
Marta Fogolari
Federico Fogolari
Alessandro Foi
Wilhelm Foissner
Kin Lam Ellis Fok
Joseph Fokam
Marjolein Fokkema
Joel Fokom Domgue
Morenike Folayan
Albert Folch
Hernan Folco
Morten Foldager
Michael Foley
John Foley
Brian Foley
Janet Foley
Debra Foley
Steven Foley
William Foley
Joao Folgado
Alonzo Folger
Marco Folini
James Folk
Ryan Folk
Carl Folke
Antonia Follenzi
Clifford Folmes
Heike Folsch
Timothy Folsom
Daniel Foltz
Wd Foltz
Evgeny Fominskiy
Adrien Fonagy
Daniel Fonceka
Guillaume Fond
G Fondazione
Constantino Fondevila
Marco Fondi
Mun Yik Fong
Ka Wing Fong
Kam Weng Fong
Ted Fong
Laura Fonken
Virginia Fonner
Maria Fonoberova
Marko Fonovic
Gustavo Fonseca
Dina Fonseca
Simone Fonseca

Joao Fonseca
Cesar Fonseca
Miguel Fonseca
Allex Fonseca
Francina Fonseca
Leandro Fonseca
Eduardo Fonseca-Pedrero
Ruvani Fonseka
Albert Font
Guy Fontaine
Bertrand Fontaine
Kevin Fontaine
Benedicte Fontaine-Bisson
Luis Fontana
Angelo Fontana
Andrea Fontana
Ariel Fontana
Jake Fontana
Marianna Fontana
Caterina Fontanella
Cynthia Fontanella
Luca Fontanesi
Francesc Font-Clos
Gustavo Fontecha
Elizabeth Fontes
Cor Jesus Fontes
Marcos Fontes
Ricardo Fontes
Anna Font-Gonzalez
Maria Font-I-Furnols
Beatriz Fontoura
Nelson Fontoura
Ling Foo
Hooi Ling Foo
Giles Foody
Anthony Fooks
Gary Fooks
Rachel Foong
Ivo Foppa
J. W. Foppe Van Mil
Ronja Foraita
Randi Foraker
Christy Foran
Francesco Forastiere
Ricardo Forastiero
Neil Forbes
Shari Forbes
Sean Forbes
Elizabeth Forbes-Blom
Jennifer Forbey
Aaron Forbis-Stokes
Anja Forche
Karl Forchhammer
Nathan Ford
Judith Ford
Michael Ford
James Ford
Caroline Ford
Dianne Ford
Alex Ford
Anthony Ford
Adam T. Ford
John Ford
Ruth Ford
Adam Ford
Amanda Ford
Laura Ford
Kevin Ford
Nancy Forde
Niamh Forde
Damien Fordham
Jiri Forejt
Bo Foreman
Taylor Foreman
Roberto Forero
F. Forest
Carlo Foresta
Roberta Foresti
Christiane Forestier
Nicolas Forestier
Antoine Forget
Patrice Forget
Joachim Forget
Eric Forgoston
Francesca Forini
Torbjörn Forkby
Nils Daniel Forkert
Johannes Forkman
Thomas Forkmann
Maria Forlenza
Giovanni Forleo
Leena Forma
Ewa Forma
Stuart Forman
Pavel Formanek
Julie Forman-Kay

Craig Formby
Pietro Formisano
Rita Formisano
Catherine Formolo
Cécile Formosa
Pau Formosa-Jordan
Anahí Formoso
Christen Fornadel
Cristina Fornaguera
Francesco Fornai
Francesca Fornari
Mara Fornaro
Maria Fornasari
P. Fornasiero
Marco Fornazieri
Maria Fernanda Forni
Gianluca Forni
Lui Forni
Erick Forno
Juan Fornoni
Alessia Fornoni
Letizia Foroni
Robert Foronjy
Mohammad Forouzanfar
Michael Forrest
J. Forrest
Graeme Forrest
Lisa Forrest
John Forrester
Terrence Forrester
John Forsayeth
Bertil Forsberg
Joana Forsea
Ola Forslund
Kristoffer Forslund
Zac Forsman
Mats Forsman
Sonja Forss-Petter
Christian Forst
Peter Forster
Michael Forster
Martin Forster
Gina Forster
Michael Forsting
David Forsyth
Christopher Forsyth
Stephen Forsythe
Paul Forsythe
Philippe Fort
Patrice Fort
Nicolas Fortane
Daniel Forte
Juan Fortea
Nikolaus Fortelny
Yolanda Fortenberry
Ana Fortes
Zuleica Fortes
Donald Forthal
Tomaso Fortibuoni
Anne-Marie Fortier
Robert Forties
Claude Fortin
Daniel Fortin
Jennifer Fortin
Mathieu Fortin
Anny Fortin
Carole Fortin
Maria Grazia Fortina
Lucas Fortini
Paola Fortini
Kimberly Fortner
Brad Fortune
Suzanna Forwood
Maria Foschino Barbaro
Geoffrey Fosgate
Andrew Foskett
Iosifina Foskolou
Brynjar Foss
Alexander Foss
Catherine Foss
Carl Gunnar Fossdal
Sabrina Fossette
Caroline Fossum
Jeremy Foster
Timothy Foster
Mark P. Foster
Warren Foster
Paula Foster
Jane Foster
Gary Foster
Scott Foster
Adriana Foster
Mark Foster
Jeffrey Foster
Matthew Foster
Becky Foster

Angel Foster
Tina Foster
Rosemary Foster
Jan Fostier
Alexis Fostier
John Fosu-Nyarko
Maria Foti
Dimitrios Fotiadis
Carmen Fotino
Anastasios Fotiou
Edward Fottrell
Philippe Foubert
Alice Foucart
Julien Foucaud
Brooke Foucault Welles
Sophie Fouchécourt
Agnes Fouet
Daryl Fougnie
Theodoros Foukakis
Sébastien Foulquier
Tom Foulsham
Nabilla Founounou
Samuel Fountain
Konstantinos Fountoulakis
Elise Fouquerel
Antoine Fouquet
Yoan Fourcade
Bertrand Fourcade
Alexandre Fouré
Giselle Foureaux
Emmanuelle Fourme
Daryl Fourney
Alyson Fournier
Auriel Fournier
George Foussias
Maria Fousteri
Georgia Fousteri
Belen Fouz
Sotirios Fouzas
Freya Fowkes
Velia Fowler
Carol Fowler
Ashley Fowler
John Fowler
Gregory Fowler
Glenn Fowler
Amy Fowler
Ashley Fowlkes
Nathan Fox
Stanley Fox
Michael Fox
Ashley Fox
Gregory Fox
M. Fox
Jefferson Fox
Aaron Fox
Jessica Fox
Emily Fox
James Fox
Richard Fox
Edward A. Fox
Jerome Fox
Alison Fox-Robichaud
Brian Foy
John Fozard
Elizabeth Fozo
Anna Fra
Anna Fracanzani
Charles Fracchia
Richard Frackowiak
José María Frade
Chantal Fradin
Chris Fradkin
Cornel Fraefel
Cesar Fraga
Helder Fraga
Tatiana Fraga
Gorka Fraga González
Tiziana Fragasso
Gabriele Fragasso
Dorothy Fragaszy
Ellen Fragaszy
Filippo Fraggetta
Enric Frago
Rodrigo Rocha Fragoso
Hilda Fragoso-Loyo
Renerio Fraguas
Christiane Frahm
André Frainer
Jacqueline Frair
Alessandro Fraldi
R. Chris Fraley
Stephanie Fraley
Elizabeth Fraley
Timothy Frana
Nathalie Franc

Pelliccia Franca
Marcondes França Jr
Paolo Francalacci
Marc Francaux
Emma France
Daniel France
Christine France
Renny Franceschi
Francesco Franceschi
Silvia Franceschi
Emilie Franceschini
Anne Francez-Charlot
Harold Franch
Claudine Franche
Alessandro Franchi
Nicola Franchi
Lorenzo Franchi
Massimo Franchini
Antonio Franchitto
Gianluigi Franci
Flavio Francini
M Pilar Francino
Diego Franciotta
Charles Francis
Gregory Francis
Matthew Francis
Joseph Francis
Fiona Francis
Mark Francis
Heather Francis
Joel Francis
Isolde Francis
Daniel Francis
David Francis
Peter Francis
Alexandre Francisco
Rita Francisco
Romeu Francisco
Thierry Franck
Christopher Francklyn
Jose R. Franco
Augusto Franco
Octávio Franco
José Carlos Franco
Christopher M. M. Franco
Octavio Franco
Renato Franco
Valentina Franco
Walfre Franco
Lara Franco
Alessandra Franco
Fm Franco
L Franco
Carlos Franco Abuín
Arnaud François
Christopher J. François
Tiago Francoy
Sven Francque
Nikolaos Frangogiannis
Sophia Frangou
Constantin Frangoulis
Rick Franich
Michael Frank
Uri Frank
Kristi Frank
Dale Frank
Stuart Frank
Philippe Frank
Craig Frank
James Frank
Arthur Frank
Matthew Frank
Steve Frank
C. Andrew Frank
Morgan Frank
Christian Frank
Cornelia Frank
Molly Franke
Linus Franke
Paul Frankel
Timothy Frankel
Adam Frankel
Paul Franken
Julia Frankenstein
Richard Frankham
Dennis Frank-Ito
M.D. Frank-Kamenetskii
Paul Frankland
Jeremy Franklin
F. Chris Franklin
Michael Franklin
Wilbur Franklin
Craig Franklin
Bernardo Franklin
Peter Franklin
Sarah Franklin

Jessica Franklin
Scott Franklin
Robert Franklin
Alastair Franklin
Gary Franklin
Nigel Franks
Stephen Franks
Paul Franks
James Franks
Andrew Frank-Wilson
Charles, H. M. J. Fransen
Paul Fransen
Maurice Franssen
Peter Fransson
Per-Anders Fransson
Paul Fransz
Patrick Frantom
Kyle Frantz
Adrien Frantz
Matthias Franz
Eelco Franz
Nico Franz
Katherine Franz
Marcus Franz
Annamaria Franze
Erika Franzen
Ornella Franzese
Andrea Franzetti
Maria Franzini
Tamara Franz-Odendaal
Raul Franzolin
Chiara Franzoni
Lori Frappier
Mirella Fraquelli
Loredana Frasca
Marianna Frascarelli
Elisabetta Frascaroli
Manfred Frasch
Simonetta Fraschetti
John Fraser
Claire Fraser
James Fraser
Paul Fraser
Hamish Fraser
Lauchlan Fraser
Erin Fraser
Patricia Fraser
Rupsha Fraser
Tim Fraser
David Fraser
Kevin Fraser
Sarah Fraser
Donna Fraser
Clarissa Fraser
Kait Frasier
Elisa Frasnelli
Jonna Frasor
Filip Fratev
Rutilio Fratti
Francesca Frau
Sally Frautschy
Shawn Fraver
Martin Fray
Richard Frayne
Jacqueline Frayne
Maryann Frazier
Valerio Frazzini
Jeverson Frazzon
Silvia Fre
Rosanne Freak-Poli
Rachel Freathy
Ivo Frébort
Jitka Frébortová
Lenin Fred
Nicolas Freda
Jeffrey Fredberg
Thomas Freddo
Bruno Frederich
Barbara Frederick
Megan Frederickson
Patrick Frederix
David Fredman
Barbara Fredrickson
Gunilla Fredrikson
Maria Fredriksson-Ahomaa
Stephen Free
Meghan Free
Todd Freeberg
John Freebody
Carrie Freed
Daniel Freedberg
Bruce Freedman
Mark S Freedman
Neal Freedman
Stephen Freedman
Jane Freedman

David Freedman
Sara Freedman
Thomas Freeman
Scott Freeman
David Freeman
Christine Freeman
Lauren Freeman
Robert Freeman
Robin Freeman
Michael Freeman
Amanda Freeman
Stephen Freeman
Sara Freeman
Joseph Freeman
Tom Freeman
Jacob Freeman
Willard Freeman
Emily Freeman
Nicole Freene
Megan Freeth
Rosa Fregel
Nevis Fregien
Karl Frei
Michael Frei
Brian Freibaum
Tomas Freiberger
Wolfgang Freidl
Richard Freifelder
Jessica Freiherr
Moti Freiman
Katia Freire
Maristela Freire
Jose Freire Da Silva Neto
Célio Freire-De-Lima
Christian Freise
Eva Freisinger
Heinz Freisling
Nancy Freitag
Jenny Freitag
Dalial Freitak
Michael Freitas
Loreta Freitas
André Victor Lucci Freitas
Flavia Freitas
Flavio Freitas
Lucas Freitas
Carolyn Freiwald
Jan Freiwald
Thibaut Fréjaville
Jeffrey Frelinger
Stephen Fremes
Pim French
Martyn French
Andrew French
Kristine French
Neil French
Dustin French
Pierre Frendo
Jean-Benoit Frenette Charron
Cedric Frenette-Dussault
Lisa Frenkel
Dan Frenkel
Milana Frenkel-Morgenstern
Francesca Frentiu
Harald Frenz
Giada Frenzilli
Jean-Marie Frère
Julien Frère
Marc Frerix
Paula Fresco
Laura Frese
Jonathan Fresnedo-Ramírez
Massimo Fresta
Aharon Freud
John Freudenstein
Ramiro Freudenthal
Conrad Freuling
Patrick Freund
Joel Freundlich
Ute Frevert
Emma Frew
Paul A. Frewen
Sharon Frey
Erwin Frey
Joachim Frey
Brigitte Frey
Andreas Frey
Seth Frey
Lewis Frey
Alexander Frey
Peter Frey
Laura Frey Law
Saskia Freytag
Christian Frezza
Anna Maria Frezza
Ana Friães

Sylvie Friant
Sonia Frias
Vinicius Frias De Carvalho
Jorge Frias-Lopez
Natalia Frias-Staheli
Urban Friberg
Ingrid Friberg
H. Friberg
A. Fricano
Julia-Stefanie Frick
Matthias Frick
Michael Frick
Andrea Frick
Claudia Fricke
Evan Fricke
Stanley Fricke
Eva-Maria Frickel
Eva Frickel
Lloyd Fricker
Hagen Frickmann
Daniel Fridberg
Micha Fridman
Hermann Frieboes
Eiko Fried
Christoph Friedburg
Godehard Friedel
Ilona Frieden
Christina Frieder
Hendrik Friederichs
Noah Friedkin
Ari Friedlaender
Alan Friedlander
Marc Friedlander
Wolfgang Friedlmeier
Harvey Friedman
Alon Friedman
Katherine Friedman
Thomas Friedman
Samuel Friedman
Wilma Friedman
Alan Friedman
Matt Friedman
Peter A Friedman
Jed Friedman
Elliot Friedman
Rick Friedman
Mark Friedman
Alexander Friedman
Bruce Friedman
Matthew Friedman
Theodore Friedman
Joel Friedman
Jennifer Friedman
Dan Friedman
Markus Friedrich
Thomas Friedrich
Alex Friedrich
Johannes Friedrich
David Friel
David Friend
Peter Friend
Brant Fries
Gabriel Fries
Martin Friess
Daniel Friess
Daniel Frigo
Dominic Frigon
Johan Frijns
Luc Frimat
Niels Frimodt-Moller
Emmanuel Frimpong
Michael Frink
Jean-Pol Frippiat
Teresa Frisan
Ashley Frisch
Dmitrij Frishman
Laura Frishman
Linda Frisman
Ilona Friso-Van Den Bos
Rikard Fristedt
Karl Friston
Jens Frisvad
Rena Friswell
Jordan Frith
Lars Fritsche
Jörg Fritz
Gayle Fritz
Hervé Fritz
Heather Fritz
Uwe Fritz
Helena Fritz
Bernd Fritzsch
Carlos Fritzsche
Ray Frizzell
Nadia Fröbisch
Tania Frode

Thomas Frodl
Kerstin Froeber
Michael Froehner
Brett Froelich
Martijn Froeling
Lutz Froenicke
Tilde Froes
Bernd Froessler
Mathy Froeyen
Nicolas Froger
Elke Fröhlich-Reiterer
Michael Frohman
Helmut Frohnhofen
Jorgen Frokiaer
Christian Frokjaer-Jensen
Gregory Frolenkov
Roman Frolov
Romy Frömer
Michael Fromm
Bastian Fromm
Klaus Frommer
Karl-Heinz Frommolt
Berengere Fromy
Edward Frongillo
Fabrizio Frontalini
Antonio Frontera
Andrea Frontini
Rémi Fronzes
Steve Frost
Douglas Frost
Andra Frost
Michael Frost
Kenneth Frost
Gerald Frost
Stephen Frost
Anna Frostegård
Robert Froud
Jan Frouz
Hannah Froy
Doriana Fruci
Pedro Fruet
Giovanna Frugis
Gema Frühbeck
Luca Frulloni
Rebecca Frum
Idan Frumin
Jesse Frumkin
Michael Frumovitz
Donald Frush
Marcus Fruttiger
William E. Fry
Christopher Fry
Christopher Fryars
Dorota Frydecka
Mark Frye
Richard Frye
Victoria Frye
Tim Fryer
Philip Frykman
Tong-Ming Fu
Wenjiang Fu
Freddie Fu
Feng Fu
Li-Wu Fu
Mao Fu
Wen-Mei Fu
Zhen F. Fu
Bingmei Fu
Xiang-Dong Fu
Xianghui Fu
Qing-Ling Fu
Xiuju Fu
Xiaohua Fu
Jianping Fu
Chuanhai Fu
Binying Fu
Yang-Chih Fu
Liyong Fu
Zhongjie Fu
Guifang Fu
Hongtuo Fu
Sau Nga Fu
Jian Fu
Jun Fen Fu
Sun Fu
Yong-Bi Fu
Yingmei Fu
Wei Fu
Qiang Fu
Ci Fu
Grace Fu
Fuyou Fu
Aicun Fu
Ying Fu
Chaofeng Fu
Kai-Yuan Fu

Dafang Fu
Chunhua Fu
Zheng Fu
Weijun Fu
Audrey Qiuyan Fu
Hangfei Fu
Yucheng Fu
Feng-Ling Fu
Daolin Fu
Jing Fu
Tao Fu
Yue Fu
Jie Fu
Chun Fu
Chi-Yu Fu
J. Fu
Zhengqing Fu
Peifen Fu
Qiushi Fu
Yiling Fu
Riqiang Fu
Daqi Fu
Li Fuchang
Flávio Fuchs
Marc Fuchs
Bruno Fuchs
Tobias Fuchs
Oliver Fuchs
Heidi Fuchs
Beate Fuchs
Peter Fuchs
Lynn Fuchs
Jerome Fuchs
Kerstin Fuchs
Sandra Fuchs
Armin Fuchs
Ota Fuchs
Rudolf Fuchshofer
Maria Fuciarelli
Karolina Fucikova
Becky Fuda
Rodrigo Fuentealba
Teresa Fuentes
Ernesto Fuentes
Blanca Fuentes
A Fuentes
Mariana Fuentes
Elisa Fuentes-Montemayor
John Fuerst
Elaine Fuertes
Susanne Fuessel
Adriane Fugh-Berman
Gwenny Fuhler
Alexander Fuhrmann
Naoko Fuji
Kevin Fuji
Yasuhisa Fujibayashi
Yoshitaka Fujihara
Kohtaro Fujihashi
Takao Fujii
Yoshiharu Fujii
Wataru Fujii
Hiroaki Fujii
Naoto Fujii
Naohiko Fujii
Takeshi Fujii
Kazumichi Fujii
Shin-Ichiro Fujii
Nobuharu Fujii
Mineko Fujimiya
Maiko Fujimori
Tsuguto Fujimoto
Shigeru Fujimoto
Koji Fujimoto
Robert Fujinami
Keishi Fujio
Takako Fujioka
Yoshio Fujioka
Tomoyuki Fujisawa
Yasuyuki Fujita
Naoya Fujita
Mayumi Fujita
Mitsugu Fujita
Ryo Fujita
Ricardo Fujita
Masayuki Fujita
Naoto Fujita
Jun Fujita
Misuzu Fujita
Matthew Fujita
Tetsuji Fujita
Hideaki Fujita
Hirofumi Fujita
Takeo Fujiwara
Toshiyoshi Fujiwara
Hiroshi Fujiwara

Esther Fujiwara
Keiichi Fujiwara
Naoto Fujiwara
Masayuki Fujiwara
Toshimichi Fujiwara
So-Ichiro Fukada
Sandra Fukada
Tohru Fukai
Junya Fukai
Kei Fukami
Hironobu Fukami
Kiyoko Fukami
Maki Fukami
Hirotaka Fukasawa
Keita Fukasawa
Francois Fuks
Takeo Fukuchi
Minoru Fukuda
Shinji Fukuda
Takeshi Fukuda
Noboru Fukuda
Yusuke Fukuda
Tetsuya Fukuda
Naoya Fukuda
I Fukuda
Eriko Fukuda
Daiju Fukuda
Masafumi Fukuda
Shin Fukudo
Masahide Fukudo
Masayo Fukuhara
Koji Fukui
Yoshihiro Fukumori
Kumiko Fukumura
Mizuho Fukunaga-Kalabis
Hiroyuki Fukuoka
Shuetsu Fukushi
Atsushi Fukushima
Michio Fukushima
Masami Fukushima
Atsuki Fukutani
Masashi Fukuzawa
Lewis Fulcher
Valerio Fulci
Stephany Fulda
Peter Fulé
A. J. Fulford
Richard Fulford
Domenico Fulgione
W. Fulham
Richard Fullagar
Adam Fullenkamp
Gerald Fuller
Claire Fuller
Peter Fuller
Deborah Fuller
Nathan Fuller
Robert Fuller
Kevin Fuller
Joel Fuller
Cindy Fuller
Shannon Fuller
Heidi Fuller
Stephanie Fullerton
Aimee Fullerton
Morgan Fullerton
Gavin Fullstone
Livia Fülöp
Roberta R. Fulthorpe
Elizabeth Fulton
David Fulton
John Fulton
Kelly Fulton
Tara Fulton
Benjamin Fulton
Keertik Fulzele
Luca Fumagalli
Roberto Fumagalli
Laura Fumanelli
Claudia Fumarola
Giorgio Fumera
Renata Fumis
Nobutaka Funa
Masayuki Funaba
Daisuke Funabara
Katsu Funai
Makoto Funaki
Satoru Funamoto
Tetsuro Funato
Kimito Funatsu
Noriko Funayama
Peter Funch
Nicholas Funderburg
Teresa Fung
Winky Wing Ki Fung
Christiane Funk

Richard Funk
Klaus Funke
Barbara Funnell
Martha Funnell
Andras Furedi
Terrence Furey
Nathan Furey
Jennifer Furin
Luciano Furlanetti
Clem Furlong
Laura I. Furlong
Edward Furlong
Gianluca Furneri
Pio Furneri
Bob Furness
David Furness
Nicholas Furnham
Clemens Fürnsinn
Debra Furr-Holden
Matthias Fürst
Axel Fürstberger
Sara Furuhagen
Kengo Furuichi
Koichi Furukawa
Yoichi Furukawa
Emi Furukawa
Takuya Furukawa
Hiroshi Furukawa
Satoshi Furukawa
Yutaka Furukawa
Takayuki Furumatsu
Fumikazu Furumi
Chikara Furusawa
Toshi Furuuchi
Yoichi Furuya
Norihiko Furuya
Luis Furuya-Kanamori
Maria Fusaro
Riccardo Fusaroli
Patrizia Fuschiotti
Maria Alice Fusco
Nobuo Fuse
Shinya Fushinobu
Marco Fusi
Andrea Fuso
Barbara Fussi
Ryo Futahashi
Takashi Futamura
Bruce Futcher
Matthias Futschik
Johannes Futterer
Abby Fyer
Jackson Fyfe
Beth Fylan
Nick Fyson
Tamas Gaal
Douglas Gaasterland
Carmen Gabaldón
Laurel Gabard-Durnam
Steven Gabardi
Chiara Gabbi
Sarah Gabbott
R. Gabdoulline
Christopher Gabel
Frank Gabel
Sandra Gabelli
Davide Gabellini
Chiara Gabellini
Thomas Gaberel
Yankel Gabet
Jean-Charles Gabillard
Nicholas Gabler
Katharina Gabriel
Mourad Gabriel
Andrea Gabrielli
Karin Gabriels
Britt Gabrielsson
Galina Gabriely
Amanda Gabster
Jana Gaburjakova
Alessandra Gabutti
Johannes Gach
Frédéric Gachon
Simon Gächter
Mar Gacias
Márta Gácsi
Raffaella Gadaleta
Shahinaz Gadalla
Nahla Gadalla
Yiorgos Gadanakis
Shiva Keshava Gaddam B
Shobhan Gaddameedhi
Dana Gaddy
Jennifer Gaddy
Joaquin Gadea
Crystal Gadegbeku

Bart Gadella
Shirish Gadgeel
Changdev Gadhe
Sanket Gadhia
Ivana Gadjanski
Mihaela Gadjeva
Klaus Gadow
Katrien Gaens
Lowell Gaertner
Justin Gaetano
Holly Gaff
Sarah Gaffen
Michelle Gaffey
Douglas Gaffin
Sarig Gafny
Jacek Gagala
Heather Gage
Elizabeth Gage-Bouchard
Sarah Gagliano
Maria Cristina Gagliardi
Dominique Gagliardi
Frank Gagliardi
Gabriella Gaglio
Matteo Gagliolo
Andre Gaglioti
Jean-Philippe Gagne
Raymond Gagne
Pascal Gagneux
Robert Gagnon
Lea Gagnon
Daniel Gagnon
Jorge Gago
Akhilesh Gaharwar
Manuel Gahete
Andreas Gahlmann
Carl Gahmberg
Junyi Gai
Xiaowu Gai
Yunchao Gai
Thais Gaiad
Jonas Gaiarsa
Nicolas Gaidet
David Gailani
Luc Gailhouste
Raphaël Gaillard
Jean-Michel Gaillard
Vinciane Gaillard
Jean-François Gaillard
Harold Gainer
Todd Gaines
Robert Gaines
Raul Gainetdinov
Robert Gaiser
Thomas Gaisl
Catherine Gaitanaki
Luis-Alberto Gaitán-Cepeda
Angelo Gaitas
Larry Gaither
Sarah Gaither
Patrick Gajewski
Raj Gaji
Prabu Gajjeraman
Georgios Gakis
Foram Gala
Katya Galactionova
Carmen Galan
Roberto Galán
José Galán
Dennis Galanakis
Jean-Philippe Galanaud
Pierre Galand
Elenis Galanis
Jennifer Galanis
Aristea Galanopoulou
Pedro A. F. Galante
William Galanter
Marc Galanter
Vasil Galat
Gabriela Galateanu
Alexia Galati
Ferruccio Galbiati
Maurizio Galderisi
Stefania Galdiero
Stephen Gale
Liisa Galea
Jerome Galea
Bennett Geoff Galef
Sujay Galen
Philippe Galera
Luis Gales
Pedro Galetti
Maricla Galetti
Luciana Galetto
Annick Galetto
Giovanni Galfano
Jose Galgani

Anne Galgon
Massimo Galia
Gigi Galiana
Francis Galibert
Pierre Galichon
Alessandra Galie
Luis Galietta
Daniela Galimberti
Andrea Galimberti
Hala Gali-Muhtasib
Veronica Galindo-Cuspinera
Claudia Galindo-Martínez
Caridad Galindo-Romero
Jorge Galindo-Villegas
Adam Galinsky
Matteo Galizzi
Cory Gall
Seana Gall
Hans-Joachim Galla
Katie Gallacher
Thomas Gallagher
William Gallagher
Martin Gallagher
Grant Gallagher
Stuart Gallagher
Iain Gallagher
Catherine Gallagher
Austin Gallagher
Suzanne Gallagher
Daniel Gallaher
Nathan Gallant
Maxime Gallant
Rodrigo Gallardo
Antonio Gallardo
Guillermo Gallardo
Cristian Gallardo-Escarate
Jason Gallas
Philippe Gallay
Robert Galle
Alejandro Gallego
Juan Álvaro Gallego
Beatriz Gallego
Angel Gallego Lázaro
Irene Gallego Romero
Alejandro Gallego-Roji
Maurizio Galleni
Stefan Galler
Rachel Gallery
Craig Gallet
Roberta Galletti
Bernard Gallez
Luisa Galli
Claudio Galli
Andrea Galli
Manela Galli
Loris Galli
Alvaro Galli
Vanessa Galli
Gary Gallick
Daniel Gallie
Maurizio Gallieni
Emanuela Galliera
Arianna Galliera
James Galligan
Warren Gallin
Alessio Gallina
Giorgio Gallinella
Elena Gallitto
Jiri Gallo
Paolo Gallo
Ciro Gallo
Angela Gallo
Pedro Gallo
Juan Gallo
Luigi Gallo
Stephen Gallo
Paul Gallo
James Gallo
Simone Gallo
David Gallo
Valentina Gallo
Antonia Gallo
Sarah Gallois-Montbrun
Susan Gallon
Lazaros Gallos
Riccardo Gallotti
Renee Galloway
Phillipe Gallusci
Fabienne Gally
David Gally
Alessandra Galmonte
Brook Galna
Anat Galor
Ana Galov
Benjamin Galper
Emilia Galperin

Jacob Galson
Aram Galstyan
Peter Galton
Ben Galuardi
Veronica Galvan
Laurie Galvan
Silvia Galvan
Christel Galvani
Felipe Galvan-Magana
Ma. De La Luz Galván-Ramírez
Klibs Galvão
Fernando Galvez
Patricia Galvez
Julio Galvez
Veronica Galvez
Antonio Galvez
Shannon Galvin
Edouard Galyov
Dumas Gálzvez
Patricia Gama
Adelina Gama
Alyssa A. Gamaldo
Amira Gamal-Eldeen
Carlos Gamazo De La Rasilla
Gerardo Gamba
Marco Gamba
Cristina Gamba
Gennaro Gambardella
Roberto Gambari
Alexandra Gambaryan
Maria Cristina Gambetta
K Gambetta-Tessini
Cristina Gambi
Maria Cristina Gambi
Alessandra Gambineri
Elisa Gambini
Giorgio Gambino
Carrol Gamble
Tony Gamble
T. Chris Gamblin
Michael Gamborg
Frances Game
Daniel Gamermann
Junaid Gamieldien
Alex Gamma
Don Gammon
Walter Gams
Yiqun Gan
Yunn-Hwen Gan
Ning Gan
Guojun Gan
Anne Kathleen Ganal-Antonio
Barry D. Ganapol
Bela Ganatra
J Ganczarek
G Gandaglia
E Gandarac
Paolo Gandellini
Phillip Gander
Fabian Gander
R. Gander
Mihir Gandhi
Ujjawal Gandhi
Shekar Gandhi
Gurpreet Gandhoke
Sara Gandini
Edson Gandiwa
Sheetal Gandotra
Sumanth Gandra
Doina Ganea
Tzvi Ganel
Anand Ganesan
Latha Ganesan
Subramaniam Ganesh
Shonraj Ganeshrao
Feng Gang
Bevin Gangadharan
Bagirath Gangadharan
Arunakumar Gangaplara
Subba Rao Gangi Setty
Umesh Gangishetti
Monique Gangloff
Sophie Gangloff
Debabani Ganguly
Parthasarathi Ganguly
Rita Gangwani
Alexander Ganin
Douglas Ganini Da Silva
Austen Ganley
Peter Gann
Sreenivas Gannavaram
Victor Gannon
Ioannis Ganopoulos
Yuval Ganot
Bergita Ganse
Thomas Ganslandt

Carolina Ganss
Bernhard Ganss
Andre Ganswindt
John Gant
Ilse Gantois
Lesya Ganushchak
Vitaly Ganusov
Tomas Ganz
Holly Ganz
Patricia Ganz
Lars Ganzert
Giulia Ganzetti
Jörg Ganzhorn
Michael Gänzle
Bin Gao
Feng Gao
George Gao
Xiaolian Gao
Xiaohu Gao
Huajian Gao
Guangping Gao
Wei Gao
Lei Gao
Fei Gao
Jimin Gao
Zaifeng Gao
Ge Gao
Dengfeng Gao
Xin Gao
Lin Gao
Shujing Gao
Junping Gao
Zhihong Gao
Xiangdong Gao
Xiangyun Gao
Jian Gao
Xue Gao
Hongchang Gao
Wenyuan Gao
Zheng Gao
Yi-Qin Gao
Hongbo Gao
Song Gao
Xiao-Ming Gao
Jinhao Gao
Zhong-Shan Gao
Zexia Gao
Weidong Gao
Feng Guang Gao
Jianxi Gao
Yurui Gao
Huijiang Gao
Xian-Hui Gao
Fen-Fei Gao
Xi-Wu Gao
Zhong-Ke Gao
Xiaodong Gao
Yu Gao
Jianbo Gao
Jinlong Gao
Nan Gao
Ming Gao
Jin Gao
Yang Gao
Hongjian Gao
Ying Gao
Yue Gao
Xianoqing Gao
Xinyan Gao
Jay Gao
Mingming Gao
Jun Gao
Lianghui Gao
Mingang Gao
Xuemei Gao
Man Gao
Mengqiu Gao
Fei Philip Gao
Lexuan Gao
Jianhua Gao
Qian Gao
Zhihai Gao
Chunxu Gao
Zan Gao
Xiaohua Gao
Yaozong Gao
Lianming Gao
Jinpeng Gao
Xiaorong Gao
Lu Gao
Yong Gao
Tong Gao
Lihong Gao
Xiao Gao
Yandi Gao
Jianzhao Gao

Liang Gao
Bilwaj Gaonkar
Laurent Gapin
Paolo Garagnani
Marion Garai
Stavros Garantziotis
Antonios Garas
Gianpiero Garau
Michael Garavito
Francesca Garbarini
Matteo Garbelotto
David Garber
Michael Garber
Christoph Garbers
Samantha Garbers
Nichola Garbett
David Garboczi
Sandra Garces
Patricia Garcez
Hector Garcia
Celia Garcia
Antonio Garcia
Jose Garcia
Sylvie Garcia
Gabriela Garcia
David Garcia
Pilar Garcia
Sonia Garcia
Danilo Garcia
Jair Garcia
Claude Garcia
Guilherme Garcia
A. Denise Garcia
Amaya Garcia
Jonathan Garcia
C. Garcia
Josefina Garcia
Franklin Garcia
Luis Garcia
Kevin Garcia
Idoia Garcia
Brandon Garcia
G. Garcia
Jesús García
José Luis García
Luis García
Luis Ángel García
Federico García
Rosario García
Carlos García
Pilar Beatriz Garcia- Allende
Jose Manuel Garcia Aznar
Andrea Garcia Bravo
Cristina García Cáceres
Rodolfo Garcia Contreras
Pablo Garcia De Frutos
José García De La Asunción
Daniel Garcia De La Serrana
Carlos Garcia De Leaniz
Francisco J. García De León García De León
Darío Garcia De Viedma
Javier Garcia Enriquez
Guillermo Garcia Garcia
Marina Garcia Garrido
Alfredo Garcia Iii
Jose Garcia Manteiga
Jose Garcia Martin
Jorge Garcia Molinos
Pablo A. Garcia Parisi
Luis Garcia Rodriguez
Maria Teresa Castilho Garcia Santana
Xabier Garcia-Albeniz
Monica Garcia-Alloza
Luz Garcia-Alonso
A. García-Álvarez
Itsaso Garcia-Arcos
Alberto Garcia-Basteiro
Ignacio García-Basterra
María Laura García-Bermejo
Héctor Garcia-Caldero
Javier Garcia-Campayo
Pilar Garcia-Canela
Francisco Garcia-Cozar
Miguel Garcia-Diaz
Diego Garcia-Diaz
César García-Díaz
Guillermo Garcia-Effron
Vega García-Escudero
Antonio Garcia-España
José García-Fernández
David García-Fresnadillo
Guillermo Garcia-Garcia
Manuel Garcia-Garcia
José-María García-García
Lourdes García-García
José García-Giménez

Juan Garcia-Gomez
José Carlos García-Gómez
Juan García-Gómez
Marta Garcia-Granero
Ricardo García-Herrera
Guillermo García-Manero
Teresa Garcia-Marques
Juan Antonio Garcia-Martin
E. García-Martín
Mauricio García-Mateu
Jose Garcia-Mazcorro
Jose Javier Garcia-Medina
Carmelo García-Monzón
Marcos García-Ojeda
Luis Garcia-Ortiz
Daniel García-Ovejero
Pablo García-Palacios
Lidia Garcia-Perez
Abel Garcia-Pino
Joan Garcia-Porta
Anthony Garcia-Prats
Juan-Carlos García-Ramirez
Isabel García-Real
Susana Garcia-Recio
Francisco García-Río
Juan Luis García-Rodriguez
Pablo Garcia-Roves
Carmen Garcia-Ruiz
J. Adolfo García-Sáinz
Adolfo Garcia-Sastre
Alfonso Garcia-Sosa
Javier Garcia-Tirado
Juan J. Garcia-Vallejo
Ignacio Garcia-Verdugo
Angel García-Villalón
Angel Garcimartín
Dominique Garcin
Christophe Garcion
Stephen Garczynski
David Gard
Gwenn Garden
Sara Gardenghi
Laurent Garderet
Susana Gardete
Stephanie Gardham
Katheleen Gardiner
David Gardiner
Chris Gardiner
Bruce Gardiner
Natalie Gardiner
Sue Gardiner
Jayne Gardiner
Barry Gardiner
Kevin Gardner
David Gardner
Paul Gardner
Beth Gardner
Lytt Gardner
Allison Gardner
Benjamin Gardner
Matthew Gardner
Iain Gardner
Ian Gardner
Thomas Gardner
Charlie Gardner
Fabrizio Gardoni
Lucia Gardossi
Jennifer Gardy
Melanie Gareau
Mahdi Garelnabi
David Garfield
Sara Garfield
D. Garfinkel
Prabha Garg
Nisha Garg
Himanshu Garg
Vidu Garg
Shikha Garg
Puneet Garg
Rohini Garg
Seema Garg
Rajat Garg
G. K. Garg
Luna Gargani
Andrea Gargas
Caroline Gargett
Claudia Gargini
Floriana Gargiulo
J. Jay Gargus
Gerhard Garhöfer
Manuela Gariboldi
Tara Gariepy
Genevieve Gariepy
Krishna Garikipati
Venkata Garikipati
Eduardo Garin

Vincent Garitey
Kimberly Garland-Campbell
Gustavo Garlet
Anders Garm
Junkal Garmendia
Lana Garmire
Holger Garn
Jeffrey Garnas
Teresa Garnatje
Malgorzata Garnczarska
Sylvie Garneau-Tsodikova
Warren Garner
Harold Garner
Ethan Garner
Duane Garner
Joseph Garner
Brian Garner
Anne Garnier
Romain Garnier
Peter Garnsey
Daniela Garone
Richard Garrad
Olivier Garraud
Victoriano Garre
Peter Garred
Wiebke Garrels
Melissa Garren
Michael Garrett
Jinnie Garrett
Nigel Garrett
Scott Garrett
Christopher Garrett
Elizabeth Garrett-Mayer
Lee Ann Garrett-Sinha
R. Garrick
Lucia Garrido
Marcelo Garrido
Patrick Garrigan
Didier Garriguet
Holly Garringer
Kathleen Garrison
Jered Garrison
Gregory Garrison
David Garrod
Maité Garrouste-Orgeas
Robert Garry
Mary Garry
David Garry
Kathrin Garschall
Mark Garside
Hom Gartaula
Philippe Garteiser
John Garthwaite
Anton Gartner
Jutta Gartner
Fátima Gärtner
Annette Gärtner
Fleur Garton
Jim Garvey
Peter Garvin
Michael Garvin
Russell Garwood
Thomas Gary
J. Brannon Gary
Sarah Garza
Jacob Garza
Ulises Garza-Ramos
Jessica Garzke
Carol Garzon Lopez
Tomas Garzon-Muvdi
Janvier Gasana
Antonio Gasbarrini
Audrey Gasch
Caley Gasch
Christoph Gasche
Geraldine Gascoin
Mireia Gascon
Juan J. Gascón
Duncan Gascoyne
Danijela Gasevic
Anatoliy Gashev
Daniel Gasic
Brianna Gaskill
Peter Gaskill
Jeremy Gaskins
Luciane Gaspar
Augusta Gaspar
Tania Gaspar
Flavio Gaspari
Gail Gasparich
Julien Gasparini
Stefano Gasparini
Hrvoje Gasparovic
Giuseppe Gasparre
Fabio Gasparri
Antonio Gasparrini

Lawrence D. Gaspers
Justin Gass
Pamela Gasse
Susan Gasser
Thomas Gasser
Brigitte Gasser
Christian Gasser
Maria Gasset
Natalie Gassman
Katja Gassner
Diana Gassó
Amalia Gastaldelli
Hill Gaston
Daniel Gaston
Anca Gaston
Amir Gat
Thibault Gateau
Jose Gatell
Robert Gatenby
Soeren Gatermann
Andrew Gates
Terry Gates
Daniel Gates
Kathleen Gates
François-Joël Gatesoupe
John Gatesy
Stephen Gatesy
David Gathara
Grace Gathungu
Anne Gatignol
Jesse Gatlin
Miriam Gatt
Blandine Gatta-Cherifi
Valter Gattei
Norbert Gattermann
Jean-Luc Gatti
Giulia Gatti
Luca Gattinoni
M. P. Gatto
Will Gattrell
Stefan Gaubatz
Hugh Gauch
Gérôme Gauchard
Jean-Francois Gauchat
Melvin Gauci
Nicolas Gaudenzio
Yves Gaudin
Amélie Gaudin
Mario Fulvio Gaudino
Luciano Gaudio
Benoit Gaudou
Véronique Gaudreault
Sylvie Gaudron
Quentin Gaudry
Phillip Gauger
Andrea Gaughan
Ingrid Gaugler-Senden
James Gauld
Cassandra Gauld
Victor Gault
Jane Gaultney
Laurence Gaume
Michael Gaunt
Stephen Gaunt
Deepak Gaur
Rajiv Gaur
Ajay Gaur
Pascale Gaussem
Rajeev Gautam
Uma Shankar Gautam
Vivek Gautam
Vikas Gautam
Shalini Gautam
Rekha Gautam
Gregory Gauthier
Emilie Gauthier
Baptiste Gauthier
Simon Gauthier
Olivier Gauthier
Sidney Gauthreaux
Virginie Gautier
Amandine Gautier-Stein
Jacques Gautrais
Pascale Gautret
Mary Gauvain
Laetitia Gauvin
Cindy Gauvreau
Paulo Gavaia
Julie Gavard
Evripidis Gavathiotis
David Gaveau
Donald Gaver
Maria Gaviao
Kathleen Gavin
Felicity Gavins
Zohar Gavish

Nirit Gavish
Anna Gavrieli
Mikhail Gavrilin
Marija Gavrovic-Jankulovic
Alexandra Gavryushkina
Meinrad Gawaz
Matthias Gawlitza
Timothy Gawne
Cynthia Gay
P. Gay
Alan Gay
Denise Gay
Marie-Claire Gay
Ghislaine Gayan-Ramirez
Etienne Gayat
Charlotte Gaydos
Srimonta Gayen
Charlotte Gayer-Anderson
Surya Gayet
Angèle Gayet-Ageron
Brian Gaylord
Eugenia Gayo
Sheena Gayomba
Elodie Gazave
William Gaze
David Gaze
Nurun Nahar Gazi
Tom Gaziano
Raúl Gazmuri
Maria Gazouli
Stéphane Gazut
Laura Gazza
Elisabetta Gazzerro
Maria Gazzinelli
Mattia Gazzola
Maria Gazzotti
Uwe Gbureck
Xuejun Ge
Jian Ge
Jianqiao Ge
Wei Ge
Yubin Ge
Xue-Jun Ge
Feng Ge
Shuping Ge
Yuan Ge
Xin Ge
Zhao-Jia Ge
Huiming Ge
Jung Ge
Ying Ge
Guang-Bo Ge
Xiuli Ge
David Gealy
Po-Wu Gean
Ashley Gearhardt
John Gearhart
Jeff Gearhart
Marla Gearing
Emma Gearon
Timothy Geary
David Geary
Steven Geary
Konstanze Gebauer
Florian Gebauer
Niklas Gebauer
Jonny Geber
Rolf Gebhardt
Christoffer Gebhardt
Stefan Gebhardt
Christof Gebhardt
Connie Gebhart
Usman Gebi
Bas Geboers
Mekdes Gebremariam
Wondwossen Gebreyes
Ayman Gebril
Teklemichael Gebru
Kerry Gedan
John Geddes
Abeje Gedefaw
Christopher Geden
Stephen Gee
Julie Gee
Mitch Geel
Alexander Geen
Vincent Geenen
Annemie Geeraerd
Sofie Geeraerts
Arie Geerlof
Geert-Jan Geersing
Bart Geerts
Dirk Geerts
Maria Geffen
Matthew Gegg
Gernot Geginat

Paul Geha
Marcelo Gehara
M. Gehling
Kurt Gehlsen
Ricardo Gehrau
Mario Gehri
Niels Gehring
Adam Gehring
U. Gehring
Stanley Gehrt
Scott Geib
Christian Geib
Richard Geider
Susanna Geidne
Hartmut Geiger
Eva-Maria Geigl
Christoph-Martin Geilfus
Inga Geipel
Janet Geipel
Christian Geis
Roland Geisberger
Stefan Geisen
David Geiser
Fritz Geiser
Dawn Geiser
Martial Geiser
Tobias Geisler
Katrin Geisler
Martin Geisler
Alexander Geissler
Katja Geissler
Paul Wenzel Geissler
Quentin Geissmann
Andrea Geisz
Tessarolo Geiziane
Rafael Gelaleti
Tesfaye Gelanew Taye
Bizu Gelaye
Rondi Gelbard
Shari Gelber
Hans Gelderblom
Mathias Gelderblom
Peter Geldhof
Jonas Geldmann
Katrin Geleijns
Stephan Geley
Jeffrey Gelfand
Eric Gelhaye
Maria Isabel Geli
Roselle Gélinas
David Gell
Christopher Gell
Laxmi Gella
Stacie Geller
David A. Geller
Andrew Geller
Gail Geller
Gary Gellerman
Jean-Christophe Gelly
Simon Gelman
Carlo Gelmetti
Dambala Gelo
Alessandro Geminiani
Sandra Gemma
Robert Gemmill
Emmanuel Gempp
Jens Gempt
Suzuki Gen
Kursad Genc
Salih Gencer
Caroline Genco
Robert Genco
Dominique Gendrel
Maria Gendron
Nicholas Generous
Elke Genersch
Lorenzo Genesio
Jacques Genest
Anne-Marie Genevière
Daoying Geng
Liying Geng
Jinju Geng
Yupeng Geng
Tuoyu Geng
Yanquan Geng
Junping Geng
Shuo Geng
Xin Geng
Jie Geng
Santhana Gengiah
Samuel Genheden
Grigory Genikhovich
Guy Genin
Emmanuelle Génin
Davide Genini
Massimo Gennarelli

Luigi Gennari
Massimo Gennaro
Arne Gennerich
Jean-Luc Gennisson
Kimball Geno
Mathieu Génois
Helen Genova
Simonetta Genovesi
Andrea Genre
Rudiger Gens
Thomas Gensch
John Gensel
Lianne Gensler
David Gent
Fernando Genta
Harald Genth
Gabriele Gentile
Giorgio Gentile
Fabrizio Gentile
Luciana Gentile
Carla Gentile
Rodolphe Gentili
Fred Gentili
Maurizio Gentilucci
Matthew Gentle
Nikki Gentle
Matthew Gentry
Glen Gentry
Amy Gentzler
Martina Gentzsch
Diego Genuário
Birgit Geoerger
Jemma Geoghegan
Joan Geoghegan
Steve Georas
Birgitte Georg
Philip Georgakakos
Alex Georgakilas
Nikolaos Georgantzis
Michael George
Olivier George
Ronnie George
Sophie George
Sophia George
Andrew George
Eric George
Sheila George
Johnson George
Christine Marie George
Biju George
Sheeja George
James George
Brandon George
Philippe Georgel
Nikolaos Georgelis
Elias Georges
François Georges
Bogdan Georgescu
Adriana Georgescu
Nicholas Georgiadis
Janniko Georgiadis
Michael Georgiadis
Panagiota Georgiadou
George Georgiou
Christos Georgiou
Andrew Georgiou
Lucy Georgiou
Pantazis Georgiou
Marianthi Georgitsi
Urania Georgopoulou
Susanne Georgsson
Smitha Georgy
Nimish Gera
Max Geraedts
Adam Geraghty
Sandrine Geranton
Nadine Gerard
Philippe Gérard
Edward Gérardeaux
Patrick Gérardin
Charles Gerardo
Rita Gerardy-Schahn
Oleg Gerasimenko
Mantzaris Gerasimos
Maria Gerbase-Delima
Jean-Frederic Gerbeau
Bertram Gerber
Yariv Gerber
Alexander Gerber
Naamit Gerber
Madelyn Gerber
Peter Arne Gerber
David Gerberry
Jasper G Gerbers
Gunter Gerbeth
Susan Gerbi

Fernando Gerchman
Gregory Gerdeman
A. Martin Gerdes
Jantje Gerdes
Norbert Gerdes
Marco Gerdol
Volker Gerdts
Roberto Geremia
Chris Geremia
Akos Gerencser
Sarah Gergel
Markus Gerhard
Rene Gerhard
Joachim Gerhold
Francesco Geri
Justin Gerke
Gabriele Gerlach
Richard Gerlach
Patrick Gerland
Denis Gerlier
Offer Gerlitz
Nancy Gerloff
Hayley Germack
Anne Germain
Charlotte Germain-Aubrey
Donovan German
David Germanaud
Ana Germano
Menno Germans
Anastasios Germenis
Pierre Germon
Alison Gernand
G. Gerold
Vasilis Gerovasileiou
Gilberto Gerra
Mariel Gerrard Wheeler
Philippe Gerrienne
Karin Gerritsen
Hans Gerritsen
Edward Gershburg
Marvin Gershengorn
Elliot Gershon
Michael Gershon
Laurel Gershwin
Lisa-Ann Gershwin
Alexander Gerson
Tobias Gerstenberg
Bernard Gerstman
Jason Gerstner
Jan Gerstoft
Peter Gerszten
Eske Christiane Gertje
George Gerton
Hermann-Josef Gertz
Will Gervais
Cristina Gervasoni
Ana Gervassi
Kristina Gervin
Klaus Gerwert
Igal Gery
Franck Gesbert
Daniel Geschwind
Eric Gese
Laura Geselbracht
Sandra Gesing
Dionne Gesink
Moshi Geso
Antoine Gessain
Amanuel Gessessew
Marco Gessi
Bradford Gessner
Denise Gessner
Tarsis Gesteira
Ignacio Gestoso
Jason Gestwicki
Neil Gesundheit
Fentie Ambaw Getahun
Kyle Gettler
Thomas Gettys
Wayne Getz
Stefan Getzin
Claudia Geue
Sebastian Geukes
Changiz Geula
Stefano Geuna
Jan Geuns
Aron Geurts
Rene Geurts
Lucie Geurts
Michael Geusz
Sharon Geva
Olivier Gevaert
Leontien Geven
Pierre Alain Gevenois
Jana Gevertz
Richard Gevirtz

Goar Gevorkian
Jennifer Gewandter
Christina Gewinner
David Gewirtz
Felipe Geyer
Christopher Geyer
David Gfeller
Seyyed Abolghasem Ghadami
Nesreene Ghaddar
Foad Ghaderi
Samir Ghadiali
Anas Ghadouani
Mahboobe Ghaedi
Saghi Ghaffari
Seyed Ghaffari
H. O. Ghaffari
Shahab Ghafghazi
Bijar Ghafouri
Ketan Ghaghada
Aziz Ghahary
Rohit Ghai
Shahram Ghanaati
Mahmoud Ghandi
Hiba Ghandour
Aghareed Ghanim
Puya Gharahkhani
Mamoun A. Gharaibeh
Ali Gharavi
Sina Gharib
Payam Gharibani
Asghar Ghasemi
Younes Ghasemi
Behnam Ghasemzadeh
Jaleh Ghashghaie
Marzyeh Ghassemi
Subhadip Ghatak
Saeid Ghavami
Irfan Ghazi
Kamelia Ghazi
Keyan Ghazi-Zahedi
Hazem Ghebeh
Rahel Ghebre
Giulia Ghedini
Tarik Gheit
Tamer Gheita
Adrian Gheorghe
Giovanni Gherardi
Frank Gherardini
Mihaela Gherghiceanu
Stefan Cristian Gherghina
Sergio Ghersevich
Roberto Gherzi
Pietro Ghezzi
Lorenzo Ghiadoni
Homayon Ghiasi
Gabriel Ghiaur
Lina Ghibelli
Alessandro Ghidini
Michele Ghielmini
Veronica Ghiglieri
Giorgio Ghigliotti
Claudia Ghigna
Reza Ghiladi
Megha Ghildiyal
C. Ghimire
Alessandra Ghinato Mainieri
Maria Ghirardi
François Ghiringhelli
Giovanna Ghirlanda
Rafik Ghobrial
Yogita Ghodke-Puranik
Hazem Ghoneim
Jaba Ghonghadze
Ratna Ghosal
Geoffrey Ghose
Aditya Ghose
Tandra Ghose
Gourisankar Ghosh
Asish Ghosh
Debashis Ghosh
Saurabh Ghosh
Souvik Ghosh
Siddhartha Ghosh
Paramita Ghosh
Chandra Ghosh
Jagadananda Ghosh
Sukla Ghosh
Samiran Ghosh
Sujoy Ghosh
Moumita Ghosh
Chaitali Ghosh
Sumantra Ghosh
Arkasubhra Ghosh
Romi Ghosh
Zhumur Ghosh
Debraj Ghosh

Saikat Kumar Ghosh
Sumit Ghosh
Rajeshwary Ghosh
Dhimankrishna Ghosh
Sudip Ghosh
Debadyuti Ghosh
Suma Ghosh
Saswata Ghosh
Sudeshna Ghosh
Manik Ghosh
Mallika Ghosh
Santosh Ghosh
Sanjoy Ghosh
Modhumita Ghosh Dasgupta
Subhasis Ghoshal
Suparna Ghosh-Jerath
Saurabh Ghoshroy
Jade Ghosn
Taoufik Ghrairi
Othman Ghribi
Sachin Ghude
Prachi Ghule
Norbert Ghyselinck
Philippe Giabbanelli
Mauro Giacca
Gabriele Giachin
Juliana Giacomazzi
Daniele Giacoppo
Sylvaine Giakoumi
Gino Gialdini
Rebecca Giallo
Evangelos Giamarellos-Bourboulis
Barbara Giambene
Anna Giammanco
Giovanni M. Giammanco
Roberto Gianani
Elisabetta Gianazza
Sara Gianella
Claudia Gianelli
Annunziata Giangaspero
Adriana Giangrande
Silvia Gianì
Chiara Giannarelli
Sergio Giannattasio
Ilias Giannenas
Tatiana Gianni
Tereza Giannini
Richard Giannone
Alberto Giannoni
Eric Giannoni
George Giannopoulos
Nick Giannoukakis
Marianna Giannoulaki
Paschalis Giannoulis
Giuliana Giannuzzi
Daniel Gianola
Valentina Gianotti
Andrea Giansanti
Maria Grazia Giansanti
Eleonora Gianti
Efstathios Giaouris
Antonino Giaquinta
Irene Giardina
Traber Giardina
William Giardino
Benoit Giasson
Athanassios Giatropoulos
Hannah Giauque
Davide Giavarina
Raffaella Giavazzi
Martin Gibala
Robbin Gibb
Zamira Gibb
Kathrin Gibbert
Derrick Gibbings
Bryan Gibbon
James Gibbons
Mark Gibbons
Henry Gibbons
Sean Gibbons
Chris Gibbons
G. Gibbons
Allen Gibbs
Melanie Gibbs
Julie Gibbs
Susan Gibbs
John Gibbs
Lisa Gibbs
Davide Gibellini
Dominique Gibert
Yann Gibert
Benjamin Gibert
Robin Giblin-Davis
Greg Gibson
Wendy Gibson
Peter Gibson

Spencer Gibson
Todd Gibson
Laura Gibson
William Gibson
Edward Gibson
Deanna Gibson
Joel Gibson
Claire Gibson
Bradley Gibson
Douglas Gibson
Mark Gibson
Daniel Gibson
Matt Gibson
Luke Gibson
Matthew Gibson
Jerry Gibson
Frank Gibson III
Katherine Gibson-Corley
Hannah Gideon
Thomas Gidlewski
Mike Gidley
Nicholas Gidmark
Albert Gidon
Jadwiga Giebultowicz
Maciej Giefing
Jason Gien
Nina Gierasimczuk
R. Gierczyñski
Klaus Giersiepen
Karl Giese
Philip Giffard
David Gifford
Rene Gifford
Katie Gifford
Andrew Gifford
Paul Gifford
Tom Gift
Sabir Giga
Valeria Gigante
Vincenzo Gigantino
Sylvie Giger-Reverdin
Alexander Gigler
Elena Gigli
Robson Giglio
Francis Gigliotti
Christopher Gignoux
Sylvain Gigout
Vincent Giguere
Marion Gijbels
Tom Gijssels
Mark Gijzen
Helen G. Gika
Ana Gil
Fernando Gil
Antonio Gil
Hyo-Wook Gil
Federica Gilardi
Taly Gilat-Schmidt
Tim Gilberger
Lawrence Gilbert
Nicolas Gilbert
Clare Gilbert
Eric Gilbert
Martin Gilbert
James Gilbert
Robert Gilbert
Sarah Gilbert
Caroline Gilbert
Ryan Gilbert
Sophie Gilbert
Stephen Gilbert
Jeremy Gilbert
Amy Gilbert
Robert Gilbertson
Asaf Gilboa
Eva Gilboa-Schchtman
Simon Gilbody
Sabine Gilch
Michael Gilchrist
George Gilchrist
Mark Gilchrist
Jennifer Gilda
Donald Gilden
Jeffrey Gildersleeve
Kelly Gildersleeve
Teba Gil-Diaz
Hezi Gildor
Krista Gile
Michael Gilead
Opher Gileadi
Alex Gileles-Hillel
Lee Giles
Wayne Giles
Keith Giles
Jon Giles
Ian Giles

Carla Giles
Erin Giles
Kurt Giles
Giorgio Gilestro
Jeffrey Gilger
Matthias Gilgien
Amos Gilhar
Javier Gil-Humanes
Sebastiano Gili
Stacey Gilk
Melissa Gilkey
Christopher Gill
Steven Gill
Clare Gill
Anthony Gill
Sean Gill
Harvinder Gill
Andrew Gill
Jonathan Gill
Tiffany Gill
Wayne Gill
Navdeep Gill
Saar Gill
Brian Gill
Prit Gill
Jason Gill
Erin Gillam
Christopher Gillberg
Nanna Gillberg
Christian Gille
Wendy Gilleard
Annick Gilles
Daniel Gillespie
Laura Gillespie
Iain Gillespie
Joseph Gillespie
Sandra Gillespie
Daniel Gillet
Laurent Gillet
Joris Gillet
Cheryl Gillett
Gifford Gillette
Melissa Gilliam
Robert Gillies
Michel Gilliet
Matthew Gilliham
Melanie Gillingham
Michael Gillings
Nicolas Gillis
Patricia Gillis
Osnat Gillor
Mark Gillrie
Robert Gilman
Evan Gilmer
Joanna Gil-Mohapel
Thomas Gilmore
Brendan Gilmore
Stuart Gilmour
Kathleen Gilmour
Emmanuelle Gilot-Fromont
Nicholas Gilpin
Deirdre Gilpin
Sarah Gilpin
Vicente Gilsanz
Janet Gilsdorf
Eric Gilson
Erik Giltay
Andrew Giltrap
Sarah Gimbel
Jeffrey Gimble
Fabricia Gimenes
Luis Gimenez
Francisco Gimenez
Selena Gimenez-Ibanez
Anne-Paule Gimenez-Roqueplo
Lucia Gimeno
Debra Gimlin
John Gimnig
Phyllis Gimotty
Matthew Ginder-Vogel
Silvia Ginés
Pere Ginès
Valentina Gineviciene
Michael Ginger
Anne Gingery
Malin Gingnell
Florent Ginhoux
Catarina Ginja
Roman Ginnan
Gary Ginsberg
Ken Ginsburg
Carren Ginsburg
Laura Giojalas
Folco Giomi
Julia Giora
Philippe Giordanengo

Edgardo Giordani
Frank Giordano
Tom Giordano
Thomas Giordano
Silvia Giordano
Antonio Giordano
Livia Giordano
Bruno Giordano
Rosanna Giordano
Kyriaki Giorgakoudi
Alejandro Giorgetti
Mario Giorgi
Marco Giorgio
Antonio Giorgio
Paolo Giorgirossi
Rebecca Giorno
Ioannis Gioulbasanis
Stefano Giovagnoli
Guillermo Giovambattista
Marta Giovanetti
Luciana Giovannetti
Massimo Giovannotti
Federico Giove
Bruno Giraldes
Teresa Giraldez
Fernando Giráldez
Rafael Giraldo
Patricia Giraldo
Timothy Girard
Alexandre Girard
Benoît Girard
Nicolas Girard
Delphine Girard
Michael Girard
Pierre Marie Girard
Marco Girardello
Cesar Girardi
Massimo Girardis
Tatiana Giraud
Eric Giraud
Etienne Giraud
Andrew Giraud
Christophe Giraud-Carrier
Guillaume Girault
Domenico Girelli
Olivier Gires
Maria Teresa Giret
Mahasweta Girgenrath
Adel Girgis
Ashok Giri
Shailendra Giri
Jitender Giri
Ayush Giri
Archana Giri
Gonzalo Giribet
Sanal Girija
Senthil Balaji Girimurugan
Santhosh Girirajan
Ayça Giritligil
Mariangela Girlanda
Eshetu Girma
Leonard Girnita
Jorge Giron
Rosina Girones
Hermann Girschick
Raja Giryes
Andrej Gisbrecht
Storz Gisela
Robert Gish
Moshe Gish
Christian Giske
Gunnar Gislason
Pascale Gisquet
Conor Gissane
Paul Gissen
Mika Gissler
Darren Gitelman
Leonid Gitlin
Berenice Gitomer
Gitsios Gitsioudis
Adriana Gittenberger-De Groot
Rolando Gittens
Aryn Gittis
Yorick Gitton
Robert Gitzen
Nick Giudice
Rosalba Giugno
Alessandro Giuliani
Alessandra Giuliani
Gregory Giuliani
Piero Giulianini
Marina Giuliano
Silvana Giuliatti
Sara Giunti
Martin Giurfa
Sorin Giusca

Paola Giussani
Chad Giusti
Pietro Giusti
Barbara Given
Patricia Givisiez
Thomas Givnish
Srikanth Givvimani
Ian Gizer
Alessio Gizzi
Leornardo Gizzi
Albert Gjedde
Ruth A. Gjerset
Jan Gjershaug
Petter Gjersvik
Nikolaos Gkantidis
Spyros Gkelis
Alexandra Gkemitzi
Effrossyni Gkrania-Klotsas
Dominika Glabska
Pierre Gladieux
Evgeny Gladilin
Angela Glading
Dafna Gladman
Candece L. Gladson
Rebecca Gladstone
William Gladstone
Douglas Gladue
Thomas Gladwin
Stefanie Glaeser
Jessie A. Glaeser
Tibor Glant
Stanton Glantz
Michael Glascock
Philippe Glaser
Benjamin Glaser
Keith Glaser
Tom Glaser
Shannon Glaser
Amy L. Glaser
Eric Glasgow
Anna Glasier
Kathryn Glass
John Glass
Kimberly Glass
Thomas Glass
Harold Glass
Richard Glassock
Karl Glastad
Jean Glaszmann
Aharona Glatman-Freedman
Manuela Glattacker
Markus Glatzel
Rainer Glauben
Volkmar Glauche
Ingmar Glauche
Vlad Glaveanu
Courtney Glavis-Bloom
Frank Glaw
Christopher Glaze
Elizabeth Glaze
Cris Glazebrook
Douglas Glazier
Jennifer Gleason
Catherine Gleason
Lani Gleason
Scott Gleason
Dieter Glebe
Jason Gleditsch
Padraig Gleeson
Nigel Gleeson
Tobias Gleich
Norbert Gleicher
Amy Gleichman
Pablo Gleiser
Christian Gleissner
Merav Gleit Kielmanowics
Alain Gleizes
Al Glen
John Glendinning
Anthony Glendinning
D. M. Glenn
Jeffrey Glennon
Maria Tarcela Gler
Isaias Glezer
Daniel Glez-Peña
Patricia Glibert
Adam Glick
Thomas Gliddon
Anton Glieder
Teodora Gliga
Theodora Gliga
Gennadi Glinsky
Stephen Gliske
Elizabeth Glisky
Sabine Glock
Gernot Glöckner

John Glod
Michael Glodek
Mael Glon
Peter Gloor
Antonio Gloria
David Gloriam
Griet Glorieux
Joseph Glorioso
Tracey Gloster
Mark Glover
Anita Glover
Kira Glover-Cutter
Grzegorz Glowacki
Ashley Gluchowski
Marci Gluck
Judith Glück
Eliane Gluckman
Ronnie Glud
Stefan Glüge
Alexey Glukhov
Katey Glunt
Svetlana Glushakova
Rebecca Gluskin
Nicholas Glykos
Judith Glynn
Laura Glynn
Peter Gmeiner
Markus Gnädinger
Timo Gnambs
Divya Prakash Gnanadhas
Vaisnavi Gnanasekaran
Olivier Gnankiné
Robert Gniadecki
Davide Gnocchi
Pål Goa
Cyrille Goarant
Christopher Goatley
Keith Goatman
Eva Göb
Gabriella Gobbi
Marco Gobbi
Mariaida Gobbini
Glenda Gobe
Kerstin Göbel
Benjamin Gobet
Erika Gobet
Carole Goble
Christopher Gobler
Johan Gobom
Miriam Gochin
Ines Gockel
Annette Gockele
Gabriela Godaly
Amar Godar
Swetha Godavarthi
Madan Godbole
Julie Godbout
William Goddard
Ben Godde
Mariette Goddijn
Simon Godecharle
Axel Godecke
Markus Gödel
Rolf Godelmann
Tanja Godenschwege
Geert Goderis
Laurent Godet
Laurie Godfrey
Catherine Godfrey
Jacques Godfroid
William Godinez
Colin Goding
Rosely Godinho
Francisco Godinho
Veronique Godot
Pere Godoy
Patricio Godoy
Alice Godoy
Veronica Godoy
Antonia Godoy-Lorite
Sylvain Godreuil
Adrienne Godschalx
Ian Godwin
Ryan Godwin
Ulrich Goebel
Florian Goebels
Miriam Goebel-Stengel
Franz Goecke
Jeremy Goecks
Julia Goedecke
Lisa Goehle
Lisa Goehler
Sudhir Goel
Sandeep Goel
Apollina Goel
Sanjay Goel

Shailendra Goel
Pranay Goel
Vijay Goel
Gunjan Goel
Krystyna Goembiowska
Sevan Goenezen
Darla Goeres
Craig Goergen
Matthew Goering
Holger Goerlitz
Fernando Goes
Heidi Goethert
Thomas Goettert
Claudia Goettsch
Ernest Goetz
Georges Goetz
Jens Peter Goetze
Andreas Goetzenich
James Goff
Vincent Goffin
Jerome Goffin
Eric Goffin
François Goffinet
Bernard Goffinet
Carl Goforth
Parikshit Gogate
William Goggins
Joseph Gogos
Nj Gogtay
Vladimir Gogvadze
Vicky Goh
Boon-Cher Goh
Kian Mau Goh
Lay Hoon Goh
Mahmoud Gohari
Jacob Goheen
Suril Gohel
Shalini Gohil
Yoshiaki Goi
Isabel Goicolea
Andrea Goijman
Beatrice Goilav
Scott Going
William Goins
Pedro Henrique Gois
Cumali Gokce
Omer Gokcumen
Alexander Golberg
Scott Gold
Michael Gold
Stefan Gold
Lisa Gold
David Gold
Maike Gold
Joseph Gold
Maria Gold
Daniel Goldberg
Joanna Goldberg
Tony Goldberg
Terry Goldberg
Burt Goldberg
Amy Goldberg
Michel Goldberg
Gary Goldberg
Matthew Goldberg
Mark Goldberg
Richard Goldberg
Mark Goldblatt
Ashley Golden
Miriam Golden
David Goldenberg
Robert Goldenberg
Fernando Goldenberg
Lucy Golden-Mason
David Goldfarb
Sharon Goldfeld
Lawrence Goldfinger
Sabine Goldhahn
Andrea Goldin
Jon Golding
Jean Golding
Stephen Goldinger
Barry Goldman
Daniel Goldman
Jeremy Goldman
Torsten Goldmann
Emily Goldmann
Lisa Goldman-Rosas
Denis Goldobin
Mary Goldring
Imeke Goldschmidt,
Pascal Goldschmidt-Clermont
David Goldsmith
Jeff Goldsmith
Paul Goldspink
Tracey Goldstein

Allan Goldstein
Stuart Goldstein
Aaron Goldstein
Adam Goldstein
Christina Goldstein
Abby Goldstein
Jed Goldstone
David Goldstone
Blanka Golebiowski
Greg Golenia
Delia Goletti
Srinivas Goli
Yvonne Golightly
John Golin
Mary Goll
Yulia Golland
Hero Gollany
Huw Golledge
Henrik Gollee
Sandra Gollnick
Andrea Gollucke
Dennis Golm
Beatrice Golomb
Rieta Gols
Sajjad Golshannavaz
Vassilij Goltsev
David Goltzman
Jonathan Golub
Sarit Golub
Jordan Golubov
Veronika Golygina
Olga V. Golyshina
S. Mojtaba Golzan
P. Golzio
Fatma Gomaa
Noha Gomaa
Jesús Gomar
Monica Gomaraschi
Aurelie Gombault
Leonard Gomella
Richard Gomer
Charles Gomer
João Gomes
José Alvaro Gomes
Yara Gomes
Maria Salomé Gomes
Rachel Gomes
Manuela Gomes
Pedro Gomes
Edgar Gomes
Ana Gomes
Cynthia Gomes
Aldrin Gomes
Aline Gomes
Samirah Gomes
Felipe Gomes
Helena Gomes
Gabriela Gomes
Eric Gomès
Sérgio Gomes Da Silva
Eduarda Gomes Neves
Mário Gomes-Pereira
Maria Gomes-Solecki
Africa Gomez
R. Ariel Gomez
Hernando Gomez
Javier Gomez
Gabriela Gomez
Romel Gomez
Maria Adelaida Gomez
James Gomez
Ricardo Gomez
Esther Gomez
Alice Gomez
Andres Gomez
Pierrick Gomez
Sandra Gomez
Jesus Gomez
Francisca Gomez
Cécile Gomez
Olga Gomez
Juan Gomez
Maria Gomez
Ana Gomez
Daniel Gomez
María Gómez
Juan-Carlos Gómez
Ana Gómez
Jordi Gómez
Miguel-Ángel Gómez
Ana Rosa Gómez Cano
Maria Teresa Gomez Casares
Diego Gomez De Barreda
Pilar Gomez Ruiz
Antonio Gomez Sal
Vicente Gomez-Alvarez

Jose Gomez-Arroyo
Marian Gómez-Beldarrain
Maria Jose Gomez-Benito
Alba Gómez-Cabello
Marta Gomez-Chiarri
Elena Gomez-Diaz
Juan Jose Gomez-Doblas
Juan-Pablo Gomez-Escribano
Jesus Gomez-Gardenes
Manuel Gomez-Gonzalez
Armando Gómez-Guerrero
Froylan Gomez-Lagunas
María José Gómez-Lechón
Andres Gomez-Lievano
Carolina Gómez-Llorente
Baltazar Gomez-Mancilla
Candelaria Gomez-Manzano
Pilar Gomez-Ramirez
Aida Gomez-Robles
Paulina Gomez-Rubio
Beatriz. Gomez-Sala,
Norma Gomez-Viquez
Jesús Gómez-Zurita
Fumi Gomi
Kenji Gomi
Tiziano Gomiero
Margarita Gomila
Ana Gomis
F. Xavier Gomis-Ruth
Anne Gompel
Philimon Gona
Alexandra Goncalves
Sebastián Gonçalves
Anthony Gonçalves
Natalia Gonçalves
Anders Goncalves Da Silva
María Gonçalves-Ageitos
Elena Goncharova
Tamas Gonda
Shakuntla Gondalia
Christopher Gondi
Julien Gondin
Florence Gondret
Cedric Gondro
Elena Gonella
Mehmet Gonen
Zhiyuan Gong
Cheng-Xin Gong
Michelle Gong
Peng Gong
Chengliang Gong
Hong-Yi Gong
Rujun Gong
Feng Gong
Haipeng Gong
Xun Gong
Jun Gong
Yiwei Gong
Li Gong
Jian Gong
Zhenping Gong
Yilei Gong
Xiangyang Gong
Hai-Qing Gong
Guoshu Gong
Daozhi Gong
Guanyu Gong
Ai Hua Gong
Xiao Gong
Kerui Gong
Weili Gong
Joshua Gong
Yiping Gong
Chenguang Gong
Zhenwei Gong
Yiming Gong
Heng Gong
Xiaoying Gong
Jirui Gong
Yuanzheng Gong
Haijun Gong
Nian Gong
Binsheng Gong
Hui Gong
Joaquín Goñi
Pilar Goñi
Takehisa Gono
Wilson Gonsalves
Tanja Gonska
Paolo Gontero
Brigitte Gontero-Meunier
Corentin Gonthier
Eric Gontier
Lessando Gontijo
Vygintas Gontis
Bruno Gonzales

Lorenzo Gonzales
Eric Gonzales
Octavio Gonzales
Luis Gonzales
Jeffrey Gonzales
Jose Gonzales
Laura Gonzales Bosc
Antonio Gonzalez
Michael Gonzalez
Susana Gonzalez
Claudia Gonzalez
Andres Gonzalez
Pedro Gonzalez
Graciela Gonzalez
Norberto Gonzalez
Deyarina Gonzalez
Iveth Gonzalez
Anjelica Gonzalez
Nathalie Gonzalez
Jesus Gonzalez
Sonia Gonzalez
Aitor Gonzalez
Claudio Gonzalez
Darinka Gonzalez
Camille Gonzalez
Stevan Gonzalez
Jose Gonzalez
Jorge Gonzalez
José Gonzalez
C. R. Gonzalez
Esther Gonzalez
Javier Gonzalez
Alberto Gonzalez
Mirna Gonzalez
John Gonzalez
Elizabeth Gonzalez
Georges Gonzalez
Bernardo González
Aridane G. González
José González
Antonio Paz González
María José González
Gabriela Gonzalez Aleman
Natalia González Benítez
Claudia Gonzalez Brambila
Gustavo Gonzalez Cuevas
Jose-Luis Gonzalez De Aguilar
Elvira Gonzalez De Mejia
Africa Gonzalez Fernandez
Jose Efren Gonzalez Monroy
Rafael Gonzalez Redondo
Jose Gonzalez Ros
Daniel González Tokman
José Gonzalez-Alonso
Marcela González-Araya
Gloria Gonzalez-Aseguinolaza
Ivan Gonzalez-Bergonzoni
Sara M. González-Betancor
Lucia Gonzalez-Buendia
Guillermo González-Burgos
Fernando Gonzalez-Candelas
Javier Gonzalez-Castillo
Maria González-Domínguez
Mauricio González-Forero
Manuel Gonzalez-Garay
Ismael González-García
Miguel A González-Gay
Mario Gonzalez-Gronow
Javier Gonzalez-Maeso
Fernando Danilo Gonzalez-Nilo
Héctor González-Ocampo
Dolores González-Pacanowska
Ana M Gonzalez-Pinto
Arturo Gonzalez-Quintela
Emilio González-Reimers
José A. González-Reyes
Manuela González-Suárez
Daniel González-Tokman
Jose Gonzalez-Valdez
Romer Gonzalez-Villalobos
Emiliano González-Vioque
Alejandro Gonzalez-Voyer
Mercedes Gonzalez-Wanguemert
Bruno Gonzalez-Zorn
Peter Good
Phillip Good
Matthew Good
Sara Goodacre
Greg Goodall
Jonathan Goodall
Mark Goodarzi
Rose Goodchild
Traci Goodchild
Bruce Goode
Adam Goode
Maureen Goodenow

Michael Goodisman
Kim Good-Jacobson
Howard Goodkin
Catherine Goodman
Joel M. Goodman
Steven Goodman
Stuart Goodman
Robert L Goodman
Mark Goodman
Rosa Goodman
James Goodman
Adrian Goodman
April Goodman Hall
Beverly Goodman Tchernov
Philip Goodney
Charles Goodnight
Bret Goodpaster
John Goodpaster
Steven Goodreau
Lisa Goodrich
Laurie Goodrich
Robin Goodrich
J Max Goodson
Adam Goodwill
David Goodwin
Douglas Goodwin
Victoria Goodwin
Arthur Goodwin
Amy Goodwin
Laura Goodwin
Roger Goody
Paul Goodyer
Debra Gook
Paula Goolkasian
Maartje Goorden
Lucas Ma Goossens
Luc Goossens
Ellen Goossens
Nicolas Goossens
Purva Gopal
Shubha Gopal
K. Gopal
S. Gopala Krishnan
Ramakrishnan Gopalakrishnan
Narendran Gopalan
Vinod Gopalan
Ashok Gopalarathnam
Martin Göpfert
Uri Gophna
Vijayaprasad Gopichandran
Bamini Gopinath
Ajay Gopinathan
Unni Gopinathan
Anupama Gopisetty
Santhi Gorantla
Alexander Gorbalenya
A. Gorban
Steven Gorbatkin
Marina Gorbatyuk
Stefania Gorbi
Maxim Gorbunov
N. Gorbunov
Vera Gorbunova
Anna Gorbushina
Victor Gordeuk
John Gordon
David Gordon
Stephen Gordon
Karen Gordon
Michael Gordon
Catherine Gordon
Jonathan Gordon
Morris Gordon
Scott Gordon
Grant Gordon
Cristopher Gordon
Keith Gordon
Leo Gordon
Amy Gordon
Christopher Gordon
Eric Gordon
Florencia Gordon
Brian Gordon
Phillip Gordon-Weeks
Claire Gordy
Jeff Gore
Milind Gore
Charlotte Gore
Fred Gorelick
Root Gorelick
David Gorelick
Gabriela Gorelik
Amir Goren
Markus Gorfer
André Görgens
Martin Gorges

Tobias Gorges
Ashraf Gorgey
Chris Gorgolewski
Ronald Gorham
Simone Gori
Stanislas Goriely
H. Ulrich Göringer
Nigel Goring-Morris
Giorgio Gorini
Francesca Gorini
Marga Goris
Tobias Goris
Marnix Gorissen
Marat Gorivodsky
Priyatham Gorjala
Renata Gorjão
Olga Gorlanova
Roy Gorman
Shelley Gorman
Michael Gorman
Thomas Gorman
Jamie Gorman
Isobel Gormley
Eamonn Gormley
German Gornalusse
Stacey Gorniak
Sebastian Gornik
Krzysztof Gornik
Elise Gornish
Nico Görnitz
Ekaterina Gornung
Nikolay Gorobets
Oksana Gorobets
Guy Gorochov
Raphael Gorodetsky
Jorg Goronzy
Myriam Gorospe
Sven Gorr
Matt Gorr
Juan Gorraiz
Josef Görres
Simone Gorressen
Andrew Gorringe
Kylie Gorringe
Juan Górriz
Paul Gorry
Magdalena Gorska
Magdalena M Gorska
Malgorzata Gorska-Ciebiada
L. Gorski
Andrzej Górski
Bgh Gorte
Marko Gosak
Shachi Gosavi
Taco Gosens
Harry Goshgarian
Samuel Gosling
Martin Gosling
Erica Goss
Kara Goss
Amy Goss
Julie Gosse
Laure Gossec
Frederic Gosselin
Nadia Gosselin
Louis Gosselin
Frédérick Gosselin
Philippe Gosset
Achim Gossler
Stefan Gossling
Martin Gossner
Olivier Gossner
Elena Gostjeva
Prabhat Goswami
Moloy Goswami
Kiyoko Gotanda
André Gotardo
Yoav Gothilf
Jean Gotman
Naohisa Goto
Shin Goto
Kazushige Goto
Katsumasa Goto
Kaku Goto
Tetsuhisa Goto
Hideo Goto
Sharon Goto
Takanari Gotoda
Osamu Gotoh
Hiroki Gotoh
Takaufmi Gotoh
Nami Goto-Yamamoto
Cara Gottardi
Nicole Gottdenker
Martin Gotte
Matthias Götte

Martin Götte
Eric Gottenberg
Michael Gottfert
Cecilia Gotti
Stephan Göttig
Martin Göttlich
Yuval Gottlieb
Jeffrey Gotts
Alan Gottschalk
Stephen Gottschalk
Jinger Gottschall
Paul Gottschall
Marc Gottschling
Eva Gottwein
Silke Götze
Xiaohua Gou
Xiaoping Gou
Mingyue Gou
Leonid Goubergrits
Maryam Goudarzi
Benjamin Goudey
Basavana Goudra
Anneke Goudriaan
Christopher Gough
Leonie Gough
Patricia Gough
David Goukassian
Luis F Goulao
Sven Gould
Doug Gould
Billie Gould
Francois Gould
Lisa Gould
George Goulielmos
Ioannis Goulis
Sacha Goultiaev
Marie Jose Goumans
Fotini Gounari
Prabhu Gounder
Mj Gounis
Matthew Goupell
Guillaume Gourcerol
Robert Gourdie
Samudrala Gourinath
Benjamin Gourion
Annabelle Gourlay
Ivan Gout
Sonia Gouvea
Alexandra Gouveia
Mieke Gouwy
Elvire Gouze
Alexandre Gouzy
Nir Gov
Ureshnie Govender
Srini Goverdhan
Jose Govers-Riemslag
Jerome Govin
Chhabi Govind
Byju Govindan
Govindjee Govindjee
Elena Govorkova
Chien-Hung Gow
Elizabeth Gow
D. Channe Gowda
Siddarame Gowda
Manje Gowda
Charitha Gowda
Kymberly Gowdy
Humaira Gowher
Kavitha Gowrishankar
Félix Goyache
Sidhartha Goyal
Madhav Goyal
Rakhee Goyal
Amit Kumar Goyal
Claudia Goyer
Aymeric Goyer
Joaquin Goyret
David Gozal
Evelyne Gozal
Roberto Gozalbo-Rovira
Pedro Gozalo
Eric Goze
Davood Gozli
Raffaella Gozzelino
Yacine Graba
Natalia Grabar
Reingard Grabherr
Manfred Grabherr
Chad P. Grabner
Marcia Grabowecky
Przemyslaw Grabowicz
Marcin Grabowski
Paul Grabowski
Luis Graca
James Grace

Angela Grace
Enrique Gracia
Luis Gracia
Carlos Gracia-Lazaro
Carolina Gracitelli
Yonatan Grad
Janez Grad
Kim Gradel
Philippe Gradidge
Sergio Gradilone
Luigi Gradoni
Gerard Gradwohl
Cheryl Grady
Sue Grady
Daniel Graeber
George Graef
Sarson Graeme
Friedrich Graesser
Steffen Graether
Peter Graf
Daniel Graf
Rolf Graf
Erika Graf
Amanda Graf
Christine Graf
Ralph Gräf
Mariaelisa Graff
Christian Graff
Jan Graffelman
Ariel Graff-Guerrero
Robert M Graham
Terry Graham
Stephen Graham
Todd Graham
Catherine Graham
Scott Graham
Stuart Graham
James Graham
Stewart Graham
Emily Graham
Laura Graham
Cynthia Graham
Conor Graham
Eva Graham
Nora Grahl
Jessica Grahn
Wolfgang Graier
David Grainger
Alan Grainger
Richard Gralla
Beata Grallert
Jeffrey Gralnick
Lone Gram
Klaus Gramann
Laura Gramantieri
Steven Grambow
Greta Gramig
Michael Gramlich
Susanne Gramlich
Oliver Gramlich
Dimitris Grammatopoulos
Manuel Grana
Elisa Graña
Urs Granacher
Miriam Granado
Vinicio Granados-Soto
Bill Granath
Daniel Granato
Ann Grand
Stephanie Grand
Jonathan Grandaubert
Michel Grandbois
Nicolas Grandchamp
Joseph Grande
Andrew Grande
Gunn Grande
Paola Grandi
Umberto Grandi
Valérie Grandjean
Louis Grandjean
Jorg Grandl
Michael Grandner
Sergei Grando
Danilla Grando
Elvira Grandone
Raquel Granell
Roser Granero
Robert Grange
Cristina Grange
Antoneta Granic
Sebastian Granica
Reuben Granich
Marie-Axelle Granié
Carolyn Granier-Deferre
Winfried Graninger
Mats Granlund

Fred Grannis
Maria Grano
Hanna Granroth-Wilding
Barth Grant
William Grant
Steven Grant
Chris Grant
Andrew Grant
Taran Grant
Robyn Grant
Merida Grant
W Grant
Maria Grant
Isha Grant
Kyle Grant
Nancy Grant
Catherine Grant
Don Grant
Iain Grant
Stephen Grant
Liz Grant
Gerald Grant
Francesca Granucci
Cristina Granziera
Alexander Graphodatsky
Alessandro Grapputo
Luuk Gras
Maria Del Mar Grasa
L. Grasa
Felix Grases
Juris Grasis
Johannes Gräske
Torbjörn Gräslund
Raoul Grasman
Beatriz Gras-Miralles
Ingo Grass
Gregor Grass
Fabian Grass
Susanne Grässel
Claudio Grassi
Massimo Grassi
Gabriele Grassi
Lorenzo Grassi
Emanuela Grassilli
Stanislas Grassin Delyle
Rodrigo Grassi-Oliveira
Guntram Grassl
Lon Grassman
Felix Grassmann
Catherine Grasso
Salvatore Grasso
Giovanni Grasso
Michal Grat
Frauke Gräter
Maya Gratier
David Grattan
Paolo Gratton
Iris Gratz
Veronika Grau
Marijke Grau
Cai Grau
Niels Graudal
Linda Graudins
Anne Grauer
Christian Graugaard
Peter Graumann
Jordi Grau-Moya
Jakob Grauslund
Claudia Gravekampa
Denise Gravel
Edward Graves
Lee Graves
Steven Graves
Michael Gravett
Cinzia Gravili
Maria Flavia Gravina
Nick Gravish
Frederik Graw
Jochen Graw
Russell Gray
Andrew Gray
Jeremy Gray
Nicola Gray
Juliet Gray
Joe Gray
Rob Gray
Stuart Gray
Steven Gray
Robert Gray
Kurt Gray
Stephen Gray
Richard Gray
Ryan Gray
Alana Gray
Elin Gray
Michael Gray

Suzanne Gray
Katie L. H. Gray
Claudia Gray
Larry Gray
Miranda Gray
Michelle Gray
Kishonna Gray
Warren Grayson
Rebecca Graziani
Giuseppe Graziano
Madeleine Grealy
Maura Grealy
Erin Greaves
Urs Greber
Daryna Grechyna
Luigi Greco
Steven Greco
Teresa Greco
A. Greco
Frank Greco
Francesca Greco
Kim Green
Peter Green
William Green
Richard Green
Adam Green
James Green
Stefan Green
Dannielle Green
Ann Green
Kent Green
Edward Green
Paul Wc Green
Chris Green
Michael Green
Jennifer Green
Jonathan Green
C. Shawn Green
Philip Green
Jason R. Green
Lauri Green
Timothy Green
Charlotte Green
Julianne Green
Katharine Greenaway
David Greenberg
Eyal Greenberg
Joel Greenberger
Shoshana Greenberger
Matthew Greenblatt
David J Greenblatt
Sharon Greenblum
Harry Greene
Catherine Greene
Michael Greene
Lewis J Greene
Tom Greene
Michelle Greene
Jeffrey Greene
Scott Greene
Nicholas Greene
Michael Greenfield
Ben Greenfield
Sheldon Greenfield
William Greenhalf
David Greenhalgh
Sharon Green-Hennessy
Andrew Greenhill
Stuart Greenhill
Neil Greening
Sander Greenland
Justin Greenlee
Michael Greenlief
Yona Greenman
Mark Greenough
Anne Greenough
Robert Greenstein
Matt Greenstone
Aaron Greenville
Frank Greenway
John Greenwood
Alex Greenwood
Darren Greenwood
Sharlene Greenwood
Charles Greer
Alexander Greer
Susanna Greer
Frank Greer
Darren Greetham
Dale Gregerson
Robert Gregg
Anthony R Gregg
Ewa Gregoraszczuk
Dario Gregori
Jesse Gregory
Steve Gregory

Andrew Gregory
Philip Gregory
Stephen Gregory
Carl Gregory
Nicola Gregory
Anthony Gregory
John Gregory
Richard Gregory
Diane Gregory
Chris Gregory
Nigel Greig
Emma Greig
Ellen Greimel
Dale Greiner
Michelle Greiver
Timothy Greives
Sasha Grek
Anna Greka
Christina Gremel
Thomas Gremmel
Hendrik Gremmels
Agrena Gren
Ryszard Grenda
Ruth Grene
James Grenert
Pier Luigi Grenga
Daniel Grenier
Cécile Grenier
Paola Grenni
Laura Grenville-Briggs
Jodi Gresack
Paolo Gresele
Nohad Gresh
Melissa Gresle
Pierre Gressens
Peter Gresshoff
Tim Greten
Norbert Gretz
Michael Grever
Parwinder Grewal
Peter Grewe
Felix Grewe
Christoph Grewer
Finn Grey
Giorgia Gri
Giorgio Gribaudo
Paul Gribben
Elizabeth Grice
Joseph Grice
Marcos Gridi-Papp
Daila Gridley
Melanie Grieb
Paolo Grieco
Julie Grieco
Tina Grieco-Calub
Gerhard Gries
Oliver Griesbeck
Scott Grieshaber
Frank Griesinger
Christoph Griessinger
Kenneth Grieve
David Grieve
Alessandra Griffa
Ann Griffen
Gregg Griffenhagen
Patricia Griffin
Stephen Griffin
Darren Griffin
Marie Griffin
Christine Griffin
Gilly Griffin
Andrea Griffin
Russell Griffin
Courtney Griffin
John Griffin
Claire Griffin
Kurt Griffin
Michael Griffin
Karen Griffin
Jim Griffin
Amy Griffin
Kristen Griffin
Leslie Griffith
Brad Griffith
Boyce Griffith
May Griffith
Malachi Griffith
Derek Griffith
Mike Griffith
Ronald Griffith
Andrew Griffith
Sandra Griffith
John Griffith
James Griffith
D Griffith
Paul Griffiths

Richard Griffiths
Huw Griffiths
Michael Griffiths
Bryan Griffiths
Anthony Griffiths
Emily Griffiths
Helen Griffiths
Kristi Griffiths
Andrew Griffiths
Robert Griffitt
Alba Grifoni
Kristina Grigaityte
John Grigg
Carol Griggs
Francesco Grignani
Michelangelo Grigni
Stefano Grignolio
Ulrich Grigoleit
Paolo Grigolini
Elena Grigorenko
Boyan Grigorov
Tracy Grikscheit
Jacopo Grilli
Renato Grillo
Rachel Grillot
Clara Grilo
Ramon Grima
Annalisa Grimaldi
Benedicte Grimard
Nicolas Grimault
Bodo Grimbacher
Jack Grimes
David Robert Grimes
Volker Grimm
Dirk Grimm
Daniela Grimm
Sabine Grimm
Stefan Grimme
Neil Grimsey
Alexei Grinbaum
Jon Grinnell
Steven Grinspoon
Josef Grinyo
Paul Grippo
Denis Gris
Enrico Grisan
Salvatore Grisanti
Cameron Grisdale
Blake Grisham
Anatoly Grishin
Olaf Grisk
Andrea Grisold
Valeriya Gritsenko
Konstadina Griva
Jean-Charles Grivel
Michal Grivna
Nadja Grobe
Gerhard Gröbner
Martin Grobusch
Alex Groce
Anna Grochot-Przeczek
Thomas Grochtdreis
John Groeger
Albert Groen
Iris Groen
Wim Groen
Ewout Groen
A. B. Johan Groeneveld
Linn Groeneveld
Ronny Groenteman
James Grogan
Dorothy Grogono
Jennifer Groh
Alexander Groh
Janos Groh
Ursula Grohmann
Dina Grohmann
Elisabeth Grohmann
Olli Grohn
Sergei Grokhovsky
Alexei Grom
M. Michael Gromiha
Hermann-Josef Grone
Piotr Gronek
Detlef Gronenborn
Petra Gronholm
Sebastian Grönke
Gloria Gronowicz
Emily Gronseth
Wolfram Gronwald
Leif Groop
John Groopman
Joris Groot
Astrid Groot
Jan Groothuis
Floris Groothuis

Olivier Gros
Robert Gros
Stephanie Gros
Rita Grosch
Rebecca Groschwitz
Henri Grosjean
Julie Gros-Louis
David Gross
Garrett Gross
Alecia Gross
Briana Gross
Catharina Gross
Eduardo Gross
Jeff Gross
Harald Gross
Anika Gross
Josef Gross
Alan Gross
Jeferson Gross
Thomas Gross
Erin Gross
Melissa Gross
Alexander Gross
Joshua Gross
Tom Gross
Hans-Peter Grossart
Ian Grosse
Robert Grosse
Constantino Grosse
Scott Grosse
Dena Grossenbacher
Andree-Anne Grosset
Jean-Francois Grosset
Moritz Grosse-Wentrup
Ewald Grosse-Wilde
Paolo Antonio Grossi
Maria Fatima Grossi-De-Sa
Elena Grossini
Jasper Grosskurths
Zehava Grossman
Ashley Grossman
Robert Grossman
William Grossman
Ruth Grossman
Patrick Grossmann
Giuseppe Grosso
Michela Grosso
Gerard Grosveld
Chad Grotegut
Karin Groten
Mike Grotewiel
Georg Groth
Paul Groth
Shawn Groth
Claudia Grothe
Hinrich Grothe
Itamar Grotto
Andrea Grottoli
Michael Grotzer
Huw Groucutt
Christian Grov
Elizabeth Grove
Amy Grove
Olya Grove
Sunita Grover
Abhinav Grover
Liam Grover
Sandeep Grover
Sonam Grover
Colin Groves
Andy Groves
Patrick Groves
Allison Groves
Mitchell D. Groves
Ian Groves
Christina Grozinger
Gerd Grözinger
G. Gruartmoner
Matthew Grubb
Martin Grube
Susanne Grube
Christian Gruber
Thibaud Gruber
Monica Gruber
Renee Gruber
Karl Gruber
Mariella Gruber-Filbin
Martin Grübler
Marvin Grubman
Jeremy Gruel
Sonja Gruen
Marcus Grueschow
Marion Gruffaz
Paolo Grumati
Isabella Grumbach
Stéphane Grumbach

Alexey Grum-Grzhimaylo
Ruth Grümmer
Birgit Grund
David Grundy
Tilman Grune
Klaus Grunert
Niels Grunnet
Roland Grunow
Anne Grunseit
Ugis Gruntmanis
Niklaus Grunwald
Thomas Grunwald
Gerald Grunwald
Ilona Grunwald Kadow
Philip Gruppuso
Michael Grusch
Alexandra Gruss
Arnaud Gruss
Gerald Grütz
John Gruzelier
Ignacy Gryczynyski
Mark Grygier
Ryszard Grygorczyk
Charlotte Gryseels
Nina Grytten Torkildsen
Monika Grzesiak-Feldman
Tomasz Grzybowski
Michael Gschwandtner
Maher Gtari
Jian Gu
Jieruo Gu
Xingfang Gu
Guoqiang Gu
Ian Gu
Fangyi Gu
Bin Gu
Hongcang Gu
Dongsheng Gu
Xinbin Gu
Xiang Gu
Xingyou Gu
Peili Gu
Yong Gu
Haiyong Gu
Jianxin Gu
Zhen Gu
Bon-Mi Gu
Feng Gu
Jin Gu
Ben Gu
Zhimin Gu
Linlin Gu
Hong Gu
Lianfeng Gu
Yian Gu
Jinghua Gu
Yumei Gu
Lei Gu
Junlian (Julia) Gu
Ruo-Xu Gu
Zhiyong Gu
Qihai Gu
Keyu Gu
Ruolei Gu
Shun Gu
Haitao Gu
Chao Gu
Xiaosong Gu
Yuan Gu
Fei Gu
Wenyi Gu
Qilin Gu
Weiyong Gu
Haiwei Gu
Dongfeng Gu
Ning Gu
Tian-Xiang Gu
Rodrigo Guabiraba
Amelia Guadalupe-Grau
Victor Guaiquil
Antoni Gual
Bruno Gualano
Stanislao Gualdi
Pilar Guallar-Castillon
Fabio Gualtieri
C. Thomas Gualtieri
Yuanfang Guan
Bin Guan
Rongzhan Guan
Weihua Guan
Tao Guan
Xiangmin Guan
Hanfeng Guan
Haiyan Guan
Min-Xin Guan
Tianjia Guan

Jianfeng Guan
Jingjiao Guan
Yingjie Guan
Guijun Guan
Lihong Guan
Xie Guan Lin
Frederico Guanais
Shouhong Guang
Xiao-Hong Guang
Song Guangjia
Mao Guangyun
Li Guanwu
Giovanni Guaraldi
Pietro Guaraldi
Jean Guard
Blake Guard
Daniele Guardavaccaro
Cristina Guardia-Laguarta
Christine Guardino
Leonor Guariguata
Alfredo Guarino
Fabio Guarino
Riccardo Guarino
Maria Marta Guarna
Alba Guarné
Marco Guarneros
Michael Guarnieri
Vito Guarnieri
Biancamaria Guarnieri
Carol Guarnizo-Herreno
Fay Guarraci
Fabio Guarracino
Antonio Guasch
Ann Guassora
Luigina Guasti
Silvia Guatimosim
Jean-Pierre Guay
Marco Guazzi
Matt Gubbins
Ernesto Guccione
Henk-Jan Guchelaar
Prasenjit Guchhait
Hasan Guclu
Chittibabu Guda
Kishore Guda
Lorraine Gudas
Natalie Gude
Tore Gude
Severin Gudima
Aparna Gudlur
Kristbjorn Gudmundsson
Rakesh Guduru
Daniel Guebel
Georg Guebitz
Fitsum Guebre-Egziabher
Jerome Guechot
Raul Narciso Guedes
Nelsa Guedes
Alexandra Guedes
Vivian Guedes
Joana Guedes
Sara Guediche
Jeremie Guedj
Eric Guedj
Yann Guédon
Miguel Gueimonde
Scott Guelcher
Isabelle Guellec
Nils Guelzow
Sylvia Guendelman
Sylvie Guenette
Gelena Guens
Sebastian Guenther
Rainer Guenther
Matthias Guenther
Arndt Guentsch
Onur Guentuerkuen
M. Guentzel
Marina Guenza
Katherine Guérard
Dennis Guergen
Marcelo Guerin
Greg Guerin
Jean-Luc Guérin
Engin Guerlevik
Raphael Guerois
Raphaël Guérois
Javier Guerra
Eliete Guerra
Miguel Guerra
Marcelo Guerra
Ugo Paolo Guerra
Mateus Guerra
Richard Guerrant
Michel Guerraz
Angel Guerrero

Ricardo Guerrero
Jose Guerrero
Antonieta Guerrero-Plata
Rafael Guerrero-Preston
Fernando Guerrero-Romero
Michele Guerreschi
Dominique Guerrot
Patricia Guerry
Kristin Guertin
David Guertin
James Guest
Claire Guest
Steven Guest
Lisbeth Guethlein
Michael Guetschow
Rosalinda Guevara-Guzmán
Nicolas Guex
Francois Gueyffier
Maximilien Gueze
Alessandro Guffanti
Markus Gugatschka
Alfred Gugerell
Pascal Guggenbuhl
William Guggino
Roberto Gugig
Simone Guglielmetti
Valeria Guglielmi
Christopher Guglielmo
Letterio Guglielmo
Alice Guh
Chandan Guha
Jian-Fang Gui
Jiang Gui
Zhongzheng Gui
Renyi Gui
Dingkun Gui
Gerald Gui
Kai Gui
Silviu Guiasu
Elisa Guida
Paolo Guidetti
Laura Guidetti
Sandra Guidi
Francesca Guidobono Cavalchini
David Guidot
Alessandro Guidotti
Jeffrey Guidry
Bruno Guigas
Christophe Guignabert
Alain Guignandon
Emmanuel Guigon
Yann Guiguen
Marguerite Guiguet
Amélie Guihot
Jeroen Guikema
Francisco Guil
Gaël Guilhem
Alain Guillaume
Thomas Guillaume
Matthew Guille
Francois Guillemette
Marie-Laure Guillemin
Pablo Guillen
Carlos Guillén
Gurutzeta Guillera-Arroita
Lauren Guillette
Remy Guillevin
Quentin Guillon
Carole Guillonneau
Clement Guillot
Yannick Guilloux
Christophe Guilluy
Joao Guimaraes
Marcos Guimaraes
Denise Guimaraes
Rafael Guimaraes
John Guinan
Claire Guinat
Michele Guindani
Jesús Guinea
Joan Guinovart
Silvana Guioli
Caterina Guiot
Peng Guiqing
Evelyn Guirado
Khadidiatou Guiro
Nicole Guiso
Stéphane Guitet
Pascale Guiton
Daniel Guitton
Matthieu Guitton
Marta Guix
Nicolas Guizard
F. Gujam
Shashi Gujar
Ahmet Gul

Taza Gul
Roman Gula
Per Guldberg
Bernt Guldbrandtsen
Michael Gülden
Ulrich Güldener
Bayasi Guleng
Reto Guler
Rakeshwar Guleria
Anton Guliaev
Javier Gulías
Tod Gulick
Arthur Guljamow
Penelope Gullan
Donald Gullberg
S. Guller
Joshua Gulley
Arne Gullich
Vince Gullo
Pedro Gullón
Martin Gullstrom
Naveed Gulzar
James Gumbart
Abba Gumel
Abubakkar Gumi
Sathyanarayana Gummadi
Jacob Gump
Jaromir Gumulec
Pinar Gumus
Aldy Gunawan
Sharmini Gunawardena
Shermali Gunawardena
Venugopal Gunda
Naga Siva Kumar Gunda
Bence Gunda
Sushma Reddy Gundala
Nina Gunde-Cimerman
Vidar Gundersen
Daniel Gundersen
Gregory Gundersen
Alex Gunderson
Ozan Gundogdu
Utku Güner
Mehmet Gunes
Mustafa Gunes
Monica Gunetti
Emre Guney
Sven Gunia
Robert Gunier
Akash Gunjan
Megu Gunji
Yurii Gun'Ko
John Gunn
Alistair Gunn
Jane Gunn
Lachlan Gunn
David Gunnell
Gregg Gunnell
Yanni Gunnell
Emma Guns
Claudia K. Gunsch
John Gunstad
Bahar Güntekin
Thomas Gunter
Jennifer Gunter
Lisa Gunter
Peter Güntert
Jack Gunther
Ulrich Günther
Andreas Günther
Frauke Günther
Sreenvasulu Gunti
Orlando Guntinas-Lichius
Jenny Gunton
Anyonya Guntur
Dorothee Günzel
Matthias Gunzer
Douglas Gunzler
Zheng Guo
Feng-Biao Guo
Jun-Tao Guo
Wangzhen Guo
Nancy Guo
Ju-Tao Guo
Qinghua Guo
Fei Guo
Wei Guo
Hong-Xiong Guo
Yuming Guo
Xueliang Guo
Shiwei Guo
Zhiyong Guo
Feifan Guo
Xiong Guo
An-Yuan Guo
Xiuqing Guo

Wenwu Guo
Yalong Guo
Na Guo
Yige Guo
Xiaojuan Guo
Chao-Yu Guo
Ke Guo
Li-Tao Guo
Shuzhen Guo
Maozu Guo
Mingzhou Guo
Peiguo Guo
Wan-Jun Guo
Chunyan Guo
Chuanyong Guo
Qiyong Guo
Da-Long Guo
Yue-Leon Guo
Xingqi Guo
Ying Guo
Mingruo Guo
Longbiao Guo
Zhongren Guo
Yunqian Guo
Xiangqian Guo
Grace Guo
Hui Guo
Fangjian Guo
Xijie Guo
Yunliang Guo
Shengli Guo
Chunming Guo
Wenbin Guo
Zhiqiang Guo
Baocheng Guo
Jiannan Guo
Hongbin Guo
Zhaojiang Guo
Minglei Guo
Feng Guo
Yongli Guo
Yan-Yan Guo
Anyuan Guo
Xiao Guo
Haitao Guo
Kejun Guo
Xinxing Guo
C. Guo
Bin Guo
Yanrong Guo
Peng Guo
Chun Guo
Jun Guo
Songchang Guo
Donghua Guo
Anchen Guo
Yirui Guo
Qingqu Guo
Hou-Fu Guo
Xiaolong Guo
Shuliang Guo
Hao Guo
Youhao Guo
Yanjun Guo
Song Guo
Hai-Chao Guo
Zigu Guo
Xuemin Guo
Xinyu Guo
Xin Guo
Weichao Guo
Ren-Yong Guo
Cindy Guo
Mei-Hui Guo
Weidong Guo
Jixun Guo
Jianping Guo
Diansheng Guo
Xiaojun Guo
Hongxia Guo
Liang Guo
Jin Guoliang
Kalpna Gupta
Amita Gupta
Sanjay Gupta
Shailendra Gupta
Sanjeev Gupta
Sudhir Gupta
Sandeep Gupta
Gopal Gupta
Sudhiranjan Gupta
Sarita Gupta
Ashwani Gupta
Nimesh Gupta
Subash Gupta
Gaurav Gupta

Pushpendra Gupta
Disha Gupta
Rishein Gupta
Ajay Gupta
Madhulika Gupta
Vikrant Gupta
Rajinder Gupta
Santosh Gupta
Srishti Gupta
Aditi Gupta
Santosh Gupta
Digant Gupta
Anil Gupta
Sanchita Gupta
Arvind Gupta
Kanupriya Gupta
Manoj Gupta
Nath Gupta
Suresh Gupta
Kamal Gupta
Punit Gupta
Kajal Gupta
Meetu Gupta
Phalguni Gupta
Vikas Gupta
Rajiv Gupta
Parul Gupta
Kuldeep Gupta
Ravindra Gupta
Samir Gupta
Sumit Gupta
Manish Gupta
Rajeev Gupta
Anisha Gupte
Jeffrey Guptill
Bala Gur Dedeoglu
Tulay Guran
Zachary Gurard-Levin
Mehmet Gürbüzel
Brendon Gurd
Ali Gure
Ekrem Gürel
Erdem Güresir
Ide Gomes Dantas Gurgel
Richard Gurgel
Carlos Frederico Gurgel
Roi Gurka
Matthew Gurka
Tugba Gurkok
Susan Gurley
Kurtis Gurley
Christina Gurnett
Kevin Gurney
D. Gurney
Katerina Gurova
Werner Gurr
Olga Gursky
Ulvi Kahraman Gursoy
Ozge Gursoy-Yuzugullu
Channabasavaiah Gurumurthy
David Gurwitz
Esteban Gurzov
Irina Guschina
Aaron Gusdon
Brian Gushulak
Narcis Gusi
Roberto Gusinu
Michael Gusmano
Sander Gussekloo
Markus Gusset
Lori Gustafson
Clay Gustafson
Kathleen Gustafson
Kathryn Gustafson
Deborah Gustafson
Jan-Ake Gustafsson
Lars Gustafsson
Jeff Gustin
Kurt Gustin
Ian Gut
David Gute
Ryan Gutenkunst
Elan Guterman
Arvid Guterstam
Yoram Gutfreund
Beatriz Guth
Nagesha Guthalu Kondegowda
Eva Gutheil
William Gutheil
Reinhard Guthke
Kathleen Guthrie
Sally Guthrie
O'Neil Guthrie
Crisanto Gutierrez
Juan Pablo Gutierrez
Orlando Gutierrez

Rm Gutierrez
Jose Gutierrez
Gregory Gutierrez
Javier Gutierrez
Janet Gutierrez
Ricardo Gutierrez
Alvaro Gutierrez
Maximiliano Gutierrez
Félix Gutiérrez
Juan Gutiérrez
Ricard Gutiérrez
Carlos Gutiérrez
José Gutiérrez
Santiago Gutierrez Martin
Cayetano Gutiérrez-Cánovas
Carmina Gutiérrez-González
Laila Gutiérrez-Kobeh
Jose Gutierrez-Maldonado
José Gutiérrez-Pabello
Fabian Gutjahr
Gabriel Gutkind
Ivan Gutman
Kelly Gutpell
Thomas Gutsmann
Mary Guttieri
Emma Guttman
Aline Guttmann
Tolga Güvenç
David Guwatudde
R. Guy
Franck Guy
Jodie Guy
Rebecca Guy
Gordon Guyatt
Joseph Guydish
Soazig Guyomar'Ch Guyomar'Ch
Frederic Guyon
Thomas Guyondet
Stephane Guyot
Eda Celik Guzel
Irina Guzhova
Tomasz J. Guzik
Monica Guzman
Raul Guzman
Camilo Guzman
Efrain Guzman
Miguel Guzmán
Alerie Guzmán De La Fuente
Carmen Guzmán-Bracho
Enrique Guzman-Gutierrez
Ernesto Guzman-Novoa
Mariangela Guzzardi
Giorgio Guzzetta
Gianpaolo Guzzi
Rosa Guzzo
Marya Gwadz
Geum-Youn Gwak
David Gwatkin
Le Teuff Gwenael
Carole Gwizdek
Wencke Gwozdz
Chaubey Gyaneshwer
Lorenz Gygax
Klara Gyires
Alexandra Gyllenberg
Tibor Gyokeres
Marian Gyongyosi
Sandor Gyorke
David Gyorki
Valerie Gyselinck
Conny Gysemans
Attila Gyucha
Patrick Ha
Joon Ha
Mina Ha
Hyunjung Ha
Sang-Jun Ha
Kwon-Soo Ha
Ki-Tae Ha
Suk-Jin Ha
Jeong Hyub Ha
James Ha
Nam-Chul Ha
Brian Haab
Robert Haack
Rainer Haag
Karen Haag
David Haaga
Bart Haagmans
Wolfgang Haak
Koen Haak
David Haak
Daniel Haak
Jurgen Haanstra
Rita Haapakoski
Minna Haapalainen

Jarrod Haar
Regine Haardoerfer
Alexander Haas
David Haas
Bernd Haas
Karen Haas
Mark Haas
Carl Haas
Verena Haas
Joel Haas
Ashley Haase
Hajo Haase
Volker Haase
Bianca Haase
David Haase
Elisabeth Haase
Elaine Haase
Astrid Haase
Kristina Haase
Tina Haase
Ylva Haasum
Kaisa Haatainen
Marc Habash
Antje Habekuss
Jan Christian Habel
Laurel Habel
Hasem Habelhah
James Haber
Elaine Haberer
Daniel Haberman
Nagy Habib
Abdulrazaq Habib
Bilal Habib
Aida Habib
Rima Habib
Matthias Habjan
Gregor Habl
Caroline Habold
Angelita Habr-Gama
James Habyarimana
Orçun Haçariz
Abderrahman Hachani
Abigail Hackam
David Hackam
Benjamin Hackel
Leor Hackel
Jorg Hackermueller
Maree Hackett
Murray Hackett
Martine Hackett
David Hackney
T. Hackney
Ted Hackstadt
Holger Hackstein
Stéphane Hacquard
Batsheva Hadad
Farzad Hadaegh
Ashok Hadapad
Yitzhak Hadar
Karine Hadaya
Juliette Hadchouel
Nick Haddad
Luciana Haddad
Ella Haddad
Dana Haddad
Steven Haddock
Alice Haddy
Donat Häder
Stephen Hadfield
Naila Hadi
Hamid Hadi-Alijanvand
Oliver Hädicke
Fadila Hadj-Bouziane
Molouk Hadjibabaie
Christos Hadjichristodoulou
Kostas Hadjidimitrakis
Maria Hadjifrangiskou
Costas Hadjipanayis
Smail Hadj-Rabia
James Hadler
Craig Hadley
Darryl Hadsell
Jeff Hadwiger
Yavor Hadzhiev
Dusan Hadzi-Pavlovic
Udo Haecker
Annelies Haegeman
Dieter Haemmerich
Marisa Haenni
Hiroshi Haeno
Beate Haertel
Thomas Haertle
S. M. Mansour Haeryfar
Steven Haeseker
Marcel Haesler
Martin Haeusler

Julius Clemence Hafalla
Gaudenz Hafen
Doris Hafenbradl
Hafez Hafez
Gregory Haff
Doug Haffner
Michael Haffner
David Hafler
Marc Hafner
Lena Hafrén
Raphael Haftka
Susanne Haga
Cindy Hagan
John Hagan
Jose Hagan
Lars Hagander
Gisela Hagberg
Steffen Hage
Peter Hagell
Martin Hagemann
Christoph Hagemeyer
Ferry Hagen
Thilo Hagen
Wilfred Hagen
Edward Hagen
Thomas Hagenaars
Megan Hagenauer
Bruno Hagenbuch
Henrik Hager
Randi Hagerman
Ariadne Hager-Theodorides
Patrick Haggard
Gareth Hagger-Johnson
Aliakbar Haghdoost
M Haghi
Shahpar Haghighat
Aiden Haghikia
Daisuke Hagiwara
Cornelia Hagl
Lena Haglin
Lisbet Haglund
Henning Hagmann
Seifu Hagos
Angelica Hagsand
Steve Hague
Nobuhiro Hagura
Eva-Maria Hahler
Hyeouk Hahm
Ji-Sook Hahn
Judith Hahn
Mchael Hahn
Daniel Hahn
Amanda Hahn
Tim Hahn
Andreas Hahn
Sinuhe Hahn
Sei Kwang Hahn
Deirdre Hahn
Mariah Hahn
Sebastian Hahnel
Eric Hahnen
Stephanie Hahner
Amy Hahs
Debbie Hahs-Vaughn
Rong Hai
Chi-Ming Hai
Hossam Haick
Malak Haidar
Constantine Haidaris
Sylvia Haider
Neena Haider
Syed Haider
Sandra Haider
Donald Haider-Markel
Joerg Haier
Ives Haifig
Susan Haig
Brian Haig
Missak Haigentz
Catherine Haighton
Candace Haigler
Demewoz Haile
James Haile
Frank Hailer
Ariel Haimovici
Tim Hain
Tomoyuki Haishi
Nitai Hait
Adrian Haith
Gertruud Haitsma
Wang Hai-Ying
Behzad Hajarizadeh
E. Hajdarbegovic
Eric Hajduch
André Hajek
Peter Hajek

Masoud Hajiakbari
Mohammad Hajihosseini
George Hajishengallis
Inaya Hajj Hussein
Mohammadreza Hajjari
Alex Hajnal
Norbert Hajos
Mihaly Hajos
Laura Hak
Hak Hak
Ramin Hakami
Sebastian Håkansson
Oliver Hakenberg
Leona Hakkaart
Lari Häkkinen
Helinä Hakko
Mariai Halabalaki
Wissam Halabi
Anna Halama
Joseph Halamek
A. Halaris
Veronika Halas
Natasha Halasa
Ali Halawi
Andrew Halayko
Thanos Halazonetis
Richard Halberg
Adam Halberstadt
Amy Halberstadt
Steffen Halbgebauer
Yaroslav Halchenko
Julian Halcox
Justin Haldar
Jayanta Haldar
Amit Haldar
Lauren Haldeman
Rolf Halden
Laura Hale
Ben Hale
Amanda Hale
Lauren Hale
Rachel Hale
Nagaraj Haleagrahara
Buck Hales
Guillaume Halet
Gali Halevi
Andreana P. Haley
Kathleen Haley
William Haley
M. Ryan Haley
Nicholas Haley
John Haley
Marc Halfon
William Halford
Oday Halhouli
Adna Halilovic
Nafisa Halim
Pauliina Halimaa
Cornelia Halin
Mark Halko
Arja Halkoaho
Wayne Hall
Randy Hall
Ailsa Hall
Frank Hall
H. Irene Hall
Robert Hall
Alex Hall
Aron Hall
Rebecca Hall
Cameron Hall
Ruth Hall
Timothy Hall
Andrew Hall
Per Hall
Matthew Hall
Molly Hall
Stephen Hall
W. Hall
Gerod Hall
Alix Hall
Nathaniel Hall
Charles Hall
Kara Hall
Tom Hall
Carina Hall
Chad Hall
David Hall
Daniel L. Hall
Gunnstein Hall
Deborah Hall
Roy Hall
Mathew Hall
Casey Hall
Lawrence O. Hall
Ronald G Hall, Ii

Nadim Hallab
Asma Hallab
Rami Hallac
Dennis Hallahan
Joelle Hallak
Jorge Hallak
Pedro Hallal
Dean Hallam
Vipin Hallan
Pille Hallast
Mryka Hall-Beyer
Finn Hallböök
Helene Hallböök
Gunnel Hallden
Thorhildur Halldorsdottir
Thor Halldorsson
Bertil Halle
Gustaaf Hallegraeff
Ann Hallemans
Gregory Hallen
Patrick Hallenbeck
Dirk Haller
Florian Haller
Heidemarie Haller
Eric Hallerman
Bernard Hallet
Paul Hallett
Julianne Halley
Denise Hallfors
Benedikt Hallgrímsson
David Halliday
Joel Halligan
Mikko Hallman
Kenneth R Hallows
Jose Halloy
Jason Hall-Spencer
Teal Hallstrand
Timothy Hallstrom
John Hallsworth
Luke E. Hallum
Rachel Hallum-Montes
Richard Hallworth
Robert Halmosi
Sandra Halonen
Rebecca Halperin
Melissa Halpern
Casey Halpern
Lewis Halsey
Eric Halsey
Kimberly Halsey
Scott Halstead
Brian Halstead
Neal Halstead
Simon Halstead
Murrelet Halterman
Dietmar Haltrich
Lucyna Halupka
Marc Halushka
Kim Halvorsen
Jong Hyun Ham
Anthony Ham
Phillip Ham
Dennis Hamacher
Yoji Hamada
Hiroki Hamada
Issam Hamadeh
Masatsugu Hamaji
Af Hamam
Shioka Hamamatsu
Yoshinori Hamamoto
Takeshi Hamamura
Kazunori Hamamura
C. Hamani
Lutz Hamann
Andrea Hamann
Mark Hamann
Tsuyoshi Hamano
Takayuki Hamano
Péter Hamar
Pierre-Jacques Hamard
Kei Hamazaki
Peter Hamback
Natalie Hambalek
Dolores Hambardzumyan
Michael Hamblin
Karleigh Hamblin
K. Hambright
Anne Hamburger
Sami Hamdeh
Mohamed Hamdy Doweidar
Ahmed Hamed
Sahar M. Hamed
Sandra Hamel
Richard Hamelin
Jérôme Hamelin
Sarah Hamer

Mark Hamer
Kazanna Hames
Raymond Hames
Mohamad Shariff A Hamid
Mash Hamid
Josiph Hamill
Ruth Hamill
Thomas Hamilton
Scott Hamilton
Douglas Hamilton
Richard Hamilton
Robert Hamilton
Derek Hamilton
Paul Hamilton
Emily Hamilton
David Hamilton
Stuart Hamilton
Alison Hamilton
Justin Hamilton
Carl Hamilton
Trinity Hamilton
Matthew Hamilton
Charmain Hamilton
Fred Hamker
Christina Hamlet
J. Kiley Hamlin
Adam Hamlin
Heidi Hamm
Robert Hamm
Alison Hamm
Samar Hammad
Saoussen Hammami
Rasha Hammamieh
Christian Hammann
Peter Hammarsten
Per Hammarstrom
Lennart Hammarstrom
Anne Hammarström
Michal Hammel
Kenneth Hammel
Jörg Hammel
Daniel Hammenfors
Michael Hammer
Brian Hammer
Tobin Hammer
Malte Hammer
J. Hammer
Cornelius Hammer
John Hammer 3Rd
Dustin Hammers
Neil Hammerschlag
Kurt Hammerschmidt
Sven Hammerschmidt
Richard Hammersley
Sara Hammerstad
Frederik Hammes
Theodore Hammett
Bruce Hammock
Harald Hammon
Chrissy Hammond
Sarah Hammond
John Hammond
Thomas Hammond
Billy Hammond
Sean Hammond
Geoff Hammond
Evelynn Hammonds
Nadjib Hammoudi
Stavros Hamodrakas
Thierry Hamon
Rifat Hamoudi
Regina Hampel
Ulrike Hampel
Daniela Hampel
Shalaka Hampras
David Hampson
Ian Hampson
Elizabeth Hampson
Mark Hampton
Cheri Hampton
Ghassan Hamra
Kristin Hamre
J. Hamrick
Shannon Hamrick
James Hamrick
Melvyn Hamstra
Eliakim Hamunyela
Iqbal Hamza
Kamel Hamzaoui
Min Han
Jin Han
Xiaoxu Han
Xiaobing Han
Myung-Kwan Han
Xiao Han
Ying Han

Buhm Han
Weiping Han
Yefei Han
Ke-Li Han
Kap-Hoon Han
Chunsheng Han
Xuesong Han
Guichun Han
Zhiyong Han
Zhenhai Han
Bong-Kwan Han
Zhiming Han
Heyou Han
Chenggui Han
Seung Hyeok Han
Eun-Taek Han
Peng Han
Jian-Lin Han
Fangpu Han
Weiguo Han
Xiaozhe Han
Xiao-Pu Han
Byung Hee Han
Mei Han
Guangxuan Han
Danxiang Han
Xinwei Han
Shengtong Han
Yoo-Jeong Han
Jae Woo Han
Yiping Han
Li-Zhong Han
Dai Hoon Han
Lixing Han
Heon-Seok Han
Yuhan Han
Jeffrey Han
Qingxia Han
Jungong Han
Zhe Han
Bing Han
Kyu-Tae Han
Shuling Han
Xue Han
Shuguang Han
Lei Han
Sang-Wook Han
Tianfu Han
Joan Han
Kyu-Ho Han
Jonghee Han
Xin Han
Taewon Han
Tianxu Han
Dongmei Han
Qinghong Han
Yong Han
Shiying Han
Shuting Han
Kihwan Han
Jeong-Yeob Han
Tao Han
Fei Han
Songyan Han
Seunghee Han
Yuanhuai Han
Renzhi Han
Hyun Ho Han
Xuemei Han
Sushan Han
Peidong Han
Kin-Lan Han
Chihab Hanachi
Kousuke Hanada
Ahmad Ali Hanafi-Bojd
Norio Hanafusa
Hiroshi Hanafusa
Khalid Hanafy
Ken-Ichi Hanaki
Hiroshi Hanamoto
Jim Hanan
Niall Hanan
Menachem Hanani
Abdulsamie Hanano
Hironari Hanaoka
Masayuki Hanaoka
David Hanauer
Haruo Hanawa
David Hanbury
Lynn Hancock
William Hancock
Peter Hancock
Jeff Hancock
James Hancock
Matthew Hancock
Osamu Handa

Robert Handa
Ayse Handan Baysal
Nils Olav Handegard
Andreas Handel
Janine Handforth
Zafar Handoo
Yves Handrich
Joseph Handsaker
Daniel Handwerker
Francis Hane
Justin Hanes
Jonathan Hanes
Marc Hanewinkel
Steven Haney
Bernd Hänfling
Liang-Wen Hang
Jun Hang
Alexandru Hanganu
Jürgen Hänggi
Daniel Hänggi
Kashif Hanif
Muzlifah Haniffa
Muz Haniffa
Charles Hanifin
Christin Hanigan
Franz-Georg Hanisch
Tomas Hanke
Wolf Hanke
Katja Hanke
Michael Hanke
Kurt Hankenson
Jane Hankins
Oliver Hankinson
Emily Hankosky
Brent Hanks
Anthony Hanley
Kathryn Hanley
Luke Hanley
Hie-Won Hann
Katrina Hann
Philip Hanna
David Hanna
Nasser Hanna
Michael Hanna
Amy Hanna
Thomas Hannagan
Lee Hannah
Jennifer Hannah
Anthony Hannan
Nicholas Hannan
Johanna Hannan
David Hannapel
Ewald Hannappel
Wendy Hanna-Rose
Anne Hanneken
Sridhar Hannenhalli
Edouard Hannezo
J. Thomas Hannich
Angelos Hannides
Gregory Hannigan
Uta Hanning
Mark Hannink
Eilis Hannon
Erin Hannon
Claude Hannoun
Jarna Hannukainen
Yusuf Hannun
Xavier Hanoulle
Colleen Hanrahan
Donna Hanrahan
Chetan Hans
Philip Hansbro
Thomas Hänscheid
Eric Hanse
Anna Hansell
Narelle Hansell
Marc Hansen
David Hansen
Peter Hansen
Lars Hansen
Kristian Hansen
Jonathan Hansen
Loren Hansen
Bruce Hansen
P. Hansen
Tom Hansen
Mette Hansen
Malin Hansen
Birgita Hansen
Kasper Hansen
Haakon Hansen
Karen Hansen
Christian Hansen
Jacob Hansen
Bendek Hansen
Egon Hansen

Jason Hansen
Sonja Hansen
Hans Hansen
Anders Hansen
Michael Hansen
Ulrik Jes Hansen
Marlen Hansen
Christoph Hansis
Thomas Hanson
Charles Hanson
Bradley Hanson
Chad Hanson
Erik Hanson
Claudia Hanson
Magnus Hansson
Regina Hanstein
Olivier Hantz
Heather Hanwell
Felicia Alexandra Hanzu
Chuan-Ming Hao
Chunyi Hao
Ning Hao
Bing Hao
Fan Hao
Jiejie Hao
Jia-Sheng Hao
Feng Hao
Chongqing Hao
Hongxun Hao
Lingyun Hao
Han Hao
Chun Hao
Nan Hao
Jia-Jie Hao
Jian Hao
Yu-Jin Hao
Ma Hao
Pei Hao
Chang-Ning Hao
Zheng Haopeng
James Haorah
Alexander Hapfelmeier
Thomas Happe
Izhar Haq
Abedul Haque
U Haque
Syed Haque
Mainul Haque
Kazuo Hara
Takafumi Hara
Naozumi Harada
Ken Harada
Kazutoshi Harada
Masaru Harada
Nahoko Harada
Mariko Harada-Shiba
Hiroaki Harai
Erin Haramoto
Ali Harandi
Mariann Harangi
Oscar Harari
Avital Harari
Ai Harashima
David Harasti
Maciej Harat
Hayat Harati
Omar Harb
Elise Harb
Stephan Harbarth
Simon Harbarth
Robert Harbert
Alf Harbitz
Alastair Harborne
Brian Harcourt
Jennifer Harcourt
Sarah Harcum
Sveinn Hardarson
Alison Hardcastle
Ian Hardcastle
Jörg Hardege
Nicholas Harden
Timm Harder
Kenneth Harder
Daniel Harder
Jens Harder
Justin Hardick
Roger Hardie
Grahame Hardie
Kim Hardie
Paul Hardiman
Gary Hardiman
Jo Hardin
Pamela Harding
Scott Harding
Cary Harding
Charles Harding

Joel Harding
Ian Harding
Louisa Harding
Tim Hardingham
W. Hardman
Jonathan Hardman
Martin Hardmeier
Pablo Hardoim
Oliver Hardt
J. Marie Hardwick
James Hardwick
Robert Hardwick
Philip Hardwidge
John Hardy
Jeanne Hardy
Ronald Hardy
Joshua Hare
Brian Hare
Matt Hare
Jonathan Hare
Greg Hare
Tomomi Haremaki
Job Harenberg
Jaroslaw Harezlak
Brian Harfe
Alan R. Hargens
Sara Hargrave
Levi Hargrove
Ljubica Harhaji-Trajkovic
Michael Harhay
Jesper Harholt
Renata Hari
Svasti Haricharan
Abishek Harihar
Nirmala Hariharan
Kb Harikumar
Kaleeckal Harikumar
Robin Haring
Moustafa Hariri
Kusum Harjai
Kishore Harjai
Carmen Harjoe
Alex Harkess
Damien Harkin
Pirkko Härkönen
Richard Harland
Nicholas Harland
Diane Harland
Alexandre Harle
Avi Harlev
Vincent Harley
David Harley
Guy Harling
Barbara Harlizius
Halil Harman
Romain Harmancey
Sonia Harmand
Jean-Georges Harmelin
Frank Harmon
Luke Harmon
Thalia Harmony
Lauren Harms
Hauke Harms
Paul Harms
Marcelo Haro
M.J. Haro-Cruz
Spencer Harpe
Sam Harper
Cynthia Harper
Scott Harper
Diane Harper
Elizabeth Harper
Nathan Harper
Matthew Harper
Michael Harper
James Harper
Sherilee Harper
Gordon Harper
Dörte Harpke
Brock Harpur
Balázs Harrach
Vanessa Harrar
Frank Harrell
Joshua Harrell
Paul Harrigan
George Harrigan
Robert Harrigan
Whitney Harrington
Jesse Harrington
Matt Harrington
Peter Harrington
Reuben Harris
Reid Harris
Mark Harris
Laurence Harris
Tajie Harris

Sarah Harris
John Harris
Steven Harris
Rebecca Harris
Fiona Harris
David Harris
James Harris
Stephen Harris
Melissa Harris
J. Harris
Linda J. Harris
Andrew Harris
Ricci Harris
Jenine Harris
Edward Harris
Daniel Harris
Keith Harris
Irina Harris
Lois Harris
Nigel Harris
Lynne Harris
Ashley Harris
Kameron Harris
M. Brennan Harris
Nathaniel Harris
Craig Harris
Michael Harris-Love
Rhett Harrison
Paul Harrison
Abby Harrison
Rene Harrison
Neil Harrison
Wendy Harrison
Joe Harrison
Mark Harrison
Xavier Harrison
Jayne Harrison
Steven Harrison
Laura Harrison
Adrian Harrison
Reema Harrison
Abigail Harrison
Lisa M. Harrison
Alistair Harrison
Hugo Harrison
Lisa Harrison-Bernard
Katherine Harrisson
Marni Harris-White
Kevin Harrod
Chris Harrod
James Harrop
Issifu Harruna
Shimon Harrus
Alastair Harry
Lisa Harryson
I Harsch
Griffith Harsh
Lawrence Harshman
Stephen Hart
Joshua Hart
Amy Hart
John Hart
Prue Hart
Michael Hart
William Hart
Sarah Hart
Daniel Hart
Nicolas Hart
Jamie Hart
Jane Hart
Kathyrn Hart
Jo Hart
Trevor Hart
Tibor Hartel
Nienke Hartemink
Sarah Harten
Volker Hartenstein
Klaus Hartfelder
Tom Hartgill
Sean Hartig
Andreas Hartig
Frank Hartig
Philip Hartigan
Axel Hartke
Dominik Hartl
Sylvia Hartl
Chris Hartleb
Rebecca Hartley
Carol Hartley
Glen Hartman
William Hartman
Thomas Hartmann
Anton Hartmann
Nils Hartmann
Boris Hartmann
William Hartmann

Christian Hartmann
Wiebke Hartmann
Till Hartmann
Erica Hartmann
Daniel Hartmann
Ulrich Hartmann
Matthias Hartmann
Petra Hartmann
Andreas Hartmann
Sonja Hartnack
Chris Hartnady
Mary Elizabeth Hartnett
Peter Hartsough
Adam Hartstone-Rose
Robert Hartsuiker
John Hartung
Matthew Hartwig
Gesa Hartwigsen
John Harty
James Hartzell
Megumi Haruna
Yoshiaki Harushima
Ashot Harutyunyan
Kevin Harvatine
Kirsten Harvey
Natasha Harvey
Philip Harvey
Alexandra Harvey
Mark Harvey
Danielle Harvey
Jeffrey Harvey
Brian Harvey
Erin Harvey
Steven Harvey
Lara Harvey
Brandon Harvey
Ben Harvey
Alistair Harvey
"Barrett Harvey "
Michelle Harvie
John Harwood
Adrian Harwood
Gyan Harwood
Colin Harwood
Anne-Wil Harzing
Rumina Hasan
Zahra Hasan
Tayyaba Hasan
Nur Hasan
Syed Saif Hasan
Md. Kamrul Hasan
Alkomiet Hasan
David Hasan
Baktiar Hasan
Sadaf Hasan
Syed Khizer Hasan
Mirza Hasanuzzaman
Takashi Hasebe
Abdul Haseeb
Junichi Hasegawa
Hitoki Hasegawa
Hiroo Hasegawa
Midori Hasegawa
Takehisa Hasegawa
Hiroshi Hasegawa
Jan Hasenauer
Mike Hasenberg
Karl Hasenstein
Jonathan Hasford
Kazuo Hashido
Yoshihisa Hashiguchi
Yumi Hashim
Tomoki Hashimoto
Takashi Hashimoto
Diogo Hashimoto
Hideki Hashimoto
Mamoru Hashimoto
Junichiro Hashimoto
Koshi Hashimoto
Masahiro Hashizume
Hiroshi Hashizume
Hafiz Hashmi
Syed Hashsham
György Hasko
Nick Haslam
David W Haslam
Richard Haslam
Martin Haslbeck
Udo Hasler
David Hasler
Sandra E. Hasmann
Gal Haspel
Nurit Haspel
Fabienne Haspot
Christopher Hassall
Imran Hassan

Ferdaus Hassan
Mohammad Hassan
Amin Hassan
Kamal Hassan
Mahmoud Hassan
Syed Hassan
Sergio Hassan
Norul Hassan
Hossein Hassani
Masoud Hassanpour
Zaki Hassan-Smith
Gholamreza Hassanzadeh
Barbara Hasse
David Hassel
Martin Hasselmann
Christiane Hassenrueck
Eileen Hasser
Robert Hasserjian
Angela Hassiotis
Shayne Hassler
Dan Hasson
Christopher Hasson
Paul Hassoun
Houria Hassouna
Jason Hassrick
Michael Hast
Johanna Hastbacka
Eric Hastie
Michelle Hastings
Philip Hastings
Mardi Hastings
Paul Hastings
Hatice Hasturk
Elizabeth Haswell
Yutaka Hata
Jun Hata
Atsuko Hata
Hiroto Hatabu
Kiyohiko Hatake
Katsunori Hatakeyama
M. Hatami
Jun Hatazawa
Maureen Hatch
Abigail Hatcher
Heather Hatcher
T. Hatchette
Hiroyasu Hatekayama
Kimberly Hatfield
Gary Hatfield
Graham Hatfull
Jon Hathaway
Gareth Hathway
Anne Hatløy
Shin Hatou
Anthony Hatswell
Hideo Hatta
Taku Hatta
Christine Hatte
Theresa Hattenrath
Maximilian Hatting
Elke Hattingen
Wallisen Hattori
Ricardo Hattori
Noboru Hattori
Toshio Hattori
Angelos Hatzakis
Nicholas Hatzirodos
Vassilla Hatzitaki
Maria Hatzoglou
Polydefkis Hatzopoulos
Stavros Hatzopoulos
Peter Hau
Sebastian Haubitz
Bernard Haubold
Ruediger Hauck
Dominik Haudenschild
Martin Hauer-Jensen
Thierry Hauet
Sven Haufe
Ingeborg Haug
Joachim Haug
Ulrike Haug
Line Haugaard
Gorill Haugan
Bryan Haugen
Thomas Haugen
Kjetil Haugen
Matthew Haugh
Sylvie Hauguel-De Mouzon
Kjersti Haugum
Anna Haukioja
David Haukos
Cecile Haumaitre
Jered Haun
Andrew Haun
Robin Haunschild

Larisa Haupt
Michael Hauptmann
Jacob Haus
Sunna Hauschildt
Ben Hause
Philippe Hauser
Hansjörg Hauser
Kurt Hauser
Allen Hauser
Michael Hauser
Carl Hauser
Frank Hauser
Lorenz Hauser
David Hauser
Peter Hauser
Oliver Hauser
Pierre Hausfater
Dorothy Hausman
Jean-Francois Hausman
Jürgen Hausmann
Daniel Hausmann
Elke Hausner
Thomas Hausner
Helena Hauss
Diogo Haussen
Susanne Häussler
Stefanie Haustein
Mark Hauswald
Anne Hauswald
Sampsa Hautaniemi
Guillaume Hautbergue
Richard Hautmann
Hubert Hautmann
H. Hautzel
Viktoria Havas
Andrea Havasi
Alexandra Havdahl
Robbert Havekes
Jon Havenhand
Kathryn Havens
Thomas Haverkamp
Manon Haverkate
Bjarte Havik
Jeannette Haviland-Jones
Izhak Haviv
Jan Havlicek
Eva Havrdova
Stefan Hawelka
Joseph Hawes
Stephen Hawes
Ian Hawes
Heather Hawk
David Hawke
Bradford Hawkins
Kenneth Hawkins
Jared Hawkins
Shannon Hawkins
Carlee Hawkins
Anthony Hawkins
Meredith Hawkins
Clare Hawkins
Misty Hawkins
Guy Hawkins
Hal Hawkins
Alicia Hawkins
Simon Hawley
P. Hawley
Joshua Haworth
Simon Haworth
Violetta Hawro
Gregory Hawryluk
Jonathan Haws
Boris Haxel
Ron Hay
Mark Hay
David Hay
Roderick Hay
William Hay
Dean Hay
Phillipa J. Hay
Kazuhide Hayakawa
Yoshihiro Hayakawa
Mikito Hayakawa
Takehito Hayami
Koichi Hayano
Abdallah Hayar
Tetsuya Hayashi
Jun-Ichi Hayashi
Takuya Hayashi
Naoyuki Hayashi
Mirian Hayashi
Toshio Hayashi
Hidetaka Hayashi
Kanako Hayashi
Kazohiko Hayashi
Yuko Hayashi

Noriyuki Hayashi
Mutsuharu Hayashi
Ken Hayashi
Takeharu Hayashi
Shinichiro Hayashi
Shin-Ichiro Hayashi
Tatsuya Hayashi
Mikio Hayashi
Kentaro Hayashi
Mitsuhiro Hayashibe
Masakazu Hayashida
Morihiro Hayashida
Tomoko Hayashida
Tassawar Hayat
Malik Hayat
Johannes Haybaeck
Courtney Haycraft
Michael Hayden
Matthew Hayden
Brian Hayden
Cari-Ann Hayer
Patrick Hayes
Joseph Hayes
David Hayes
John Hayes
C. Nelson Hayes
Daniel Hayes
Matthew Hayes
Dave Hayes
Louise Hayes
Heather Hayes
Taylor Hayes
Boris Hayete
K Hayford
Kent Hayglass
Emma Hayiou-Thomas
Shawn Hayley
John L Haylor
David Hayman
Laura Hayman
Jean-Philippe Haymann
Harlene Hayne
Lia Haynes
Joel Haynes
Katelin Haynes
El Akrem Hayouni
Soren Hayrabedyan
Sohan Hayreh
Graeme Hays
William Hayton
William Hayward
Simon Hayward
Steven Hayward
Bruce Hayward
Christina Hayward
Joel Hayworth
Rachel Hazan
Puja Hazari
Khaled Hazaymeh
Kaoru Hazeki
William Hazelton
Samuel Hazen
Karsten Hazlett
Majda Haznadar
Rupenangshu Hazra
B. Hazra
Xionglei He
Fuchu He
Yuke He
Tong-Chuan He
Meian He
Xin He
Xi He
Ningjia He
Wei-Ming He
Cynthia He
Xiaosong He
Na He
Chuan He
John He
Bin He
Xinqiang He
Wei He
Ping He
Xiang He
Jiang He
Zhonghu He
Yu He
Jianguo He
Lin He
Biao He
Jun He
Zhimin He
Qigai He
Haibin He
Ji He

Yuqing He
Zengyou He
Bing He
Zhen He
Zuping He
Zhengyu He
Xiaoming He
Shaoqing He
Junxian He
Jin He
Jing He
Chaoying He
Yuxian He
Debiao He
Ji-Huan He
Daihai He
Junfeng He
Shun He
Kun He
Haiqi He
Dan He
Yipling He
Yong-Ming He
Chunguang He
Congcong He
Dalin He
Pei He
Hongbo He
Shuning He
Li He
Zhiheng He
Guofu He
Ben He
Lan He
Yao He
Yuanqing He
Bulang He
Jianxing He
Xiangling He
Shun-Min He
Kai He
Zangdong He
Yi He
Mingyue He
Jiankui He
Qin He
Tongli He
Xuesong He
Ying He
Yi-Feng He
Jian-Guo He
Dongxiao He
Qing He
Jiayuan He
Fei He
Yayi He
Cheng He
Chunyan He
Pei Min He
Guangyuan He
Yuchi He
Ximin He
Zheng He
Tianyu He
Yonghan He
Hongming He
Saike He
Weichun He
Ye He
Zhi He
Weilue He
Mark Head
Stewart Head
Megan Head
John Headrick
Jonathon Headrick
James Heaf
Shannon Heald
Simon Heales
Patrick Healey
Sue Healy
Genevieve Healy
Maureen Heaman
Christopher Heaney
Stephen Heard
Isabelle Heard
Sonya Heath
Gregory Heath
Jill Heathcock
James Heathers
Elizabeth Heath-Heckman
Robert Heaton
Nicholas Heaton
Lisa Heaton
Howard Heavner
Lionel Hebbard

David Heber
Marianne Heberlein
Lee Hebert
Kathy Hebert
Courtney Hebert
Sebastian Hebert
Katja Hebestreit
Paul Hebinck
Thomas Hecht
Kathryn Hecht
Robert Hecht
J. R. Hecht
Julia Heck
Ronald Heck
Douglas Heckathorn
Scott Heckathorn
Frank Heckel
Reinhard Heckel
Robert Heckendorn
Michael Hecker
Arnaud Hecker
Manfred Hecking
Michael Heckman
Manfred Heckmann
Stefan Heckmann
Stephen Hecnar
Andrew Hector
Christoph Heddergott
Jakob Hedegaard
Katrine Hedengran
Peter Hedera
Lars Hederstedt
Ulf Hedin
Radim Hedl
Pete Hedley
Klaus Hedman
Eva Hedman
Christophe Hedon
Laetitia Hédouin
Christian Hedrich
Dagmar Hedrich
Sabrina Hedrich
Phil Hedrick
Ann Hedrick
Philipp Heeb
Niels Heegaard
Peter Heeger
Hannelore Heemers
Sylvia Heeneman
Katrin Heer
Joerg Heeren
Amarins Heeringa
Nico Heerink
Dieter Heermann
Ralf Heermann
John Heesakkers
C. Heesen
Christopher Heffelinger
Jane Heffernan
Michael Heffernan
Henry Heffner
Sean Heffron
Katrin Hefler-Frischmuth
Nathan Heflick
P. Scott Hefty
Matthew Hegarty
Nagendra Hegde
Pushpa Hegde
Zoltan Hegedus
Richard Hegele
Harlad Hegen
Michal Heger
Tina Heger
Ulrich Hegerl
Rainer Hegselmann
Adriana Heguy
Thomas Hehlgans
Yoriko Heianza
Zahra Heidar
Kazem Heidari
Soroush Heidari Pahlavian
Gena Heidary
Warren Heideman
Johann Heider
Timo Heidt
Peter Heiduschka
Christoph Heier
David F Heigener
Richard Heijink
Irene Heijink
Wim Heijman
Jordi Heijman
Bastiaan Heijmans
Amber Heijne
M. Heijnen
Oskari Heikinheimo

Katriina Heikkila
Tuomas Heikkinen
Sarah Heilbronner
Urs Heilbronner
Mike Heilemann
Charles Heilig
Christine Heilmann
Stefanie Heilmann
Albert Heim
Julia Heiman
Lisa Heimbauer
Douglas Heimburger
Gaston Heimeriks
George Heimpel
Bernd Heimrich
Annkristin Heine
Martin Heine
Jay Heinecke
Thorsten Heinekamp
Eva Heinen
Monika Heiner
Matthias Heinig
Claire Heinitz
Stefan Heinl
Margit Heinlaan
Mikko Heino
Jani Heino
Seppo Heinonen
Krista Heinonen
Jussi Heinonsalo
Norbert Heinrich
Henriette Heinrich
Jochen Heinrichs
Elizabeth Heinrichs-Graham
Robert Heinsohn
Martin Heintzelman
Hendrik Heinz
Ruth A Heinz
Stanley Heinze
Annette Heinzel
Katharina Heinzelmann
Robert Heinzen
Svenja Heischmann
Mark Heise
Rebecca Heise
Thomas Heise
Laura Heiser
Peter Heisig
Margit Heiske
Donald Heistad
Nora Heisterkamp
Joseph Heitman
Stewart Heitmann
Rebekka Heitmar
Christine Heitsch
Mary Heitzeg
James Hejtmancik
Anne-Maarit Hekkala
Sophie Helaine
Harri Helajärvi
Heikke Helantera
Marco Helbich
Ingo Helbig
A. Katharina Helbig
Caren Helbing
Peter Helbling
Andreas Helck
Christoph Held
Paraskevi (Evi) Heldin
Dustin Heldman
Caryn Heldt
Bartosz Helfer
Miep Helfrich
Gérard Helft
Julie Helft
Björg Helgadóttir
Gudmundur Helgason
Jorn Helge
Luisa Helguero
Jean-Marie Helies
Armin Helisch
Johannes Hell
Aslaug Helland
Augustino Hellar
Christopher Hellen
Garrett Hellenthal
H. Heller
Stefan Heller
Richard Heller
Jane Heller
Rebecca Heller
David Heller
Aaron Heller
Markus Heller
Claus Hellerbrand
Walter C Hellinger

Chris Helliwell
Lars Hellman
Jens Hellmann
Richard Hellmich
Mario Hellmich
Rebecka Hellsten
Wayne Hellstrom
Kerstin Hellwig
Mark Helm
Lothar Helm
Rebecca Helm
Francoise Helmbacher
Catherine Helmer
Stefanie Helmer
Eva Helmerhorst
Matthew Helmers
Christian Helmers
Brian Helmke
Volkhard Helms
John Helms
Stephen Helms Tillery
Moritz Helmstaedter
Brian Helmuth
Claudia Helou
Christopher Helps
Jeffrey Helton
Jon Helvik
Peter Helwig
L. P. Madhubhani Hemachandra
Divakar Hemadri
Peiman Hematti
Myriam Hemberger
Maged Hemida
H. Coenraad Hemker
Martin Hemler
Bernhard Hemmer
Helmut Hemmer
Peter Hemmerich
Ann Hemmerle
Karla Hemming
Alan Hemming
Hugh Hemmings
Anna Hemnes
Stefan Hempel
Nadine Hempel
Moritz Hempen
Carl-Hermann Hempen
Liesbeth Hempenius
Jean-Louis Hemptinne
O. Henao
James Hench
Christian Hendershot
Brian Henderson
Deborah Henderson
Jeffrey Henderson
Graeme Henderson
Janet Henderson
Gemma Henderson
Lisa Henderson
Marlone Henderson
Andrew Henderson
Elizabeth Henderson
Ken Henderson
Kirsten Henderson
Linda Henderson
Arun Hendi
Anne Hendrich
Jonathan Hendricks
Nathan Hendricks
Christopher Hendrickson
Frederik Hendrickx
Antoni Hendrickx
Wouter Hendriks
Hendrikus Hendriksen
Tijn Hendrikx
David Hendrix
Petr Heneberg
Henry Heng
Keith Hengen
Tomislav Hengl
Ulrich Hengst
Katrin Henke
Christiaan Henkel
R. Henkelman
Jane Henley
Karsten Henne
Maciej Henneberg
Claudine Hennessey
Annemarie Hennessy
Dwight Hennessy
Erin Hennessy
Holger Hennig
Frederico Henning
Lars Henning
Arne Henningsen
Gwenn Hennon

Roger E Hennriken
A. K. Henras
Sandrine Henri
Charles Henri Malbert
Birgit Henrich
Natalie Henrich
Amy Henrici
Patrick Henriet
Roger Henriksen
Lena Henriksen
Denise Henriques
Jose Henriques
Alexandre Henriques
Fiona Henriquez
Bernard Henrissat
Thomas Henry
Christopher Henry
Keith Henry
Michael Henry
David Henry
Pierre-Yves Henry
Molly Henry
Kevin Henry
Jaymie Henry
Chuck Henry
Robert Henry
Niel Hens
Philipp Henschel
Nicholas Henschke
Sabrina Hense
Michael Hensel
Devon Hensel
David Henshall
Jonathan Henshaw
Erin Henshaw
Scott Hensley
Lisa Hensley
Michael Henson
Joseph Henson
Anton Henssen
Darren Henstridge
François Hentges
Eric Hentges
Kyu Heo
Lee Heow Pueh
David Hepburn
Jussi Hepojoki
Gary Hepp
Scott Heppell
P. Hepper
Guor Mour Her
Benji Heran
Priyantha Herath
John Heraty
Jean-Michel Heraud
Georges Herbein
Andre Herbelin
F. A. Herbella
Marie Herberstein
Andrew Herbert
Neil Herbert
Garth Herbert
James Herbert-Read
Peter Herbison
Craig Herbold
Martin Herbordt
Andreas Herbst
David Herbst
Julie Herbstman
Klaus Herburger
David Hercher
Andrzej Herczynski
Pamela Herd
James Herdegen
Maria Teresa Herdeiro
Christian Herder
Anna Nele Herdina
Michael Herdman
Thomas Herdt
Katrina Heredia
José Heredia-Guerrero
Hubertine Heremans
Lydie Herfort
Andreas Hergovich
Alexander Hergovich
Karl Herholz
Veronica Herias
Iveta Herichova
Paul Herijgers Herijgers
Nicolae Herisanu
Miles Herkenham
Eva Herker
Marco Herling
Eliot Herman
Pawel Herman
Jennifer Herman

Melissa Herman
Alexander Herman
Katya Herman
Sarah Herman
Dirk Hermann
Andrea Hermann
Brian Hermann
Nv Hermann
Sara Hermann
Norbert Hermanns
Joseph Hermanowicz
Sabine Hermans
Emmanuel Hermans
Katleen Hermans
Cecilia Hermans
John Hermans
Ola Hermanson
Daniel Hermens
Frouke Hermens
José Hermida
Dominique Hermier
Juan Hermoso
Jochen Herms
Roberto Hernán Gonzalez
Nouria Hernandez
Alvaro Hernandez
Fabiola Hernandez
Elena Hernandez
Jesus Hernandez
Marcela Hernandez
Brenda Hernandez
Bernardo Hernandez
Gonzalo Hernandez
Alfredo Hernandez
Teri Hernandez
Manuel Hernandez
M. Hernandez
Diana Hernandez
Felix Hernández
Maria Hernández
Alejandro Hernández
Penélope Hernández
Cristina Hernández
Luis Hernández
Dolores Hernández
Manuel Hernandez Fernandez
Edgar Hernandez-Andrade
Claudia Hernandez-Camacho
Manuel Hernández-Córdoba
Fidel Hernandez-Hernandez
Ana Hernández-Hernández
Blanca Hernandez-Ledesma
Ismael Hernández-Lucas
Salvador Hernandez-Martinez
Gabriela Hernandez-Molina
Esteban Hernandez-Vargas
Rosa Hernansaiz Ballesteros
Jose Luis Hernanz
Dennis Hernaus
Nic Herndon
Elizabeth Herndon
Sophie Hernot
Betsy Herold
Kevan Herold
Marco Herold
Dorrit Herold
Elizabeth Heron
Sarah Heron
M Héroux
Beate Herpertz-Dahlmann
Wolfgang Herr
Joshua Herr
Nadine Herr
Deron Herr
Zaida Herrador
Claudia Herrea
Anthony Herrel
Sarah Herremans
Luis Herrera
Emilio Herrera
Eder Herrera
Santiago Herrera
Raul Herrera
Antonio Herrera
Fabiany Herrera
Ileana Herrera
Virginia Aurora Herrera Valencia
Andrea Herrera-Solis
Jose Ignacio Herrero
Miguel Herrero
Laura Herrero
José Herrero
David Herrero Martin
Ana Herrero-Fresno
Víctor Herrero-Solana
Jason Herrick

Brant Herrin
Susan Herring
David Herring
John Herrington
Georg Herrler
Thomas Herrmann
Andreas Herrmann
Jean Louis Herrmann
Christian Herrmann
Michael Herrmann
Ken Herrmann
Martin Herrmann
Diana Herrmann
Karl-Heinz Herrmann
Christoph Herrmann-Lingen
Maria Del Carmen Herrojo Ruiz
Todd Herron
Patrick Herron
Christophe Herry
Jolyn Hersch
Alan Herschtal
Florian Herse
Michelle Hersh
Ray Hershberger
Israel Hershkovitz
Carlton Hershner
Laura Hertel
Dirk P. Herten
Gunilla Herting
Veronique Hertrich
Christian Hertweck
Stefan Hertwig
Tomer Hertz
Jens Michael Hertz
Andreas Hertz
Ronna Hertzano
Jean Hertzberg
Denis Hervé
Maxime Hervé
Rodolphe Hervé
Jactel Hervé
Loreen Herwaldt
Genevieve Hery-Arnaud
Rachel Herz
Mohammad Herzallah
Osnat Herzberg
Hanspeter Herzel
Sharon Herzka
Grit Herzmann
Roland Herzog
Walter Herzog
Harold Herzog
Sereina Herzog
Peter Herzsprung
Dale Hesdorffer
Allison Heskes
Kathryn Hesketh
Anthony Hesketh
Robert Hess
Rex Hess
David Hess
Ursula Hess
Ellen Hess
Steven Hess
Michael Hess
Dean R Hess
Lisa Hess
Elisabeth Hessmann
D. J. Hetem
Gyorgy Hetenyi
Nicholas Hette-Tronquart
Steven Hetts
Sébastien Hétu
Claudio Hetz
Katja Heubel
Adam Heuberger
Sabine Heublein
Rainer Heuchel
Dagmar Heuer
Herbert Heuer
Holger Heuer
Rachael Heuer
Jorg Heukelbach
Patrick Heun
Brandon Heung
Lena Heung
Valérie Heurgué-Hamard
Gerd Heusch
Michael Heuser
Nicole Heussen
Ep Heuvelink
Nikolaus Heveker
Tim Hewett
James Hewett
Alex Hewitt
Nicola Hewitt

Sandra Hewlett
Miles Hewstone
Jody Hey
Ahlke Heydemann
Robert Heyderman
Stanley Heydrick
Tobias Heye
Andreas Heyland
Elisabeth Heylen
Beate Heym
Gene Heyman
Anthony Heymann
Marc Heyndrickx
Jeffrey Heys
Scott Heysell
Charles Heyser
Anina Heystek
Anita Heywood
Yoichi Hiasa
Akihide Hibara
Paul Hibbard
Julian Hibberd
Carina Hibberd
Matthew Hibbs
Ryan Hibbs
Toshifumi Hibi
Terumasa Hibi
Taizo Hibi
Hiroshi Hibino
Lex Hiby
Paul Hick
Michael Hicke
Valerie Hickey
William Hickey
Anthony Hickey
Graham Hickling
Js Hickman
Matthew Hickman
Noreen Hickok
Stephen Hicks
Lanis Hicks
Joshua Hicks
Matthew Hicks
J. Kevin Hicks
Rosangela Hickson
Gilles Hickson
K. Hida
Michio Hidaka
Andres Hidalgo
Manuel Hidalgo
Bertha Hidalgo
M. Hidalgo
Alfredo Hidalgo-Miranda
Diana Hide
Yoshida Hideki
Robert C Hider
Kohzaki Hidetsugu
Pieter Hiemstra
Hervé Hien
Tobin Hieronymus
Brian Hiestand
Jari Hietanen
Peter Hietz
Elisa Higa
Yukihito Higashi
Nobuaki Higashi
Yusuke Higashi
Youichirou Higashi
Haruhiro Higashida
Tomomi Higashide
Tadahisa Higashide
Norihisa Higashihori
Mariano Higes
Megan Higgie
Des Higgins
N. Higgins
Paul Higgins
Lucy Higgins
John Higgins
Adam Higgins
Pennilyn Higgins
Christopher Higgins
Patricia Higgins
Stephen Higgs
Elizabeth Higgs
Dennis Higgs
Nicholas Higgs
Megan Higgs
Thomas Higham
Timothy Higham
James Higham
Charles Higham
Dean Higham
Wesley Highfield
Sarah Highlander
Lisa Hightow-Weidman

Victoria Higman
Sei Higuchi
Youichi Higuchi
Chieko Higuchi
Fatima Higuera
Carlos Higuera
Jaako Hiidenhovi
Diego Hijano
Zlad Hijazi
Karolin Hijazi
Robert Hijmans
Yasufumi Hikichi
Kenji Hikosaka
Amaya Hilario
Christian Hilbe
Angelika Hilbeck
Martin Hilbert
Kevin Hilbert
David Hilbert
Luuk Hilbrands
Ashley Hilchie
Kevin Hildebrand
Frank Hildebrand
Herbert Hildebrandt
Tom Hildebrandt
Gerhard Hildebrandt
Michelle Hildebrandt
Jørgen Hilden
Allan Hildesheim
David Hildick-Smith
Andres Hilfiker
Roger Hilfiker
Joseph Hilgard
Ingo Hilgendorf
Anne Hilgendorff
Rolf Hilgenfeld
Yvonne Hilhorst
Frederic Hilke Meier
William Hill
Philip Hill
Russell Hill
Peggy Hill
Andrew Hill
Warren Hill
David Hill
Jaclyn Hill
Janet Hill
Theresa Hill
Anne-Marie Hill
Vincent Hill
Bradford Hill
Nichola Hill
Matthew Hill
Allan Hill
Camilla Hill
Steven Hill
Mathew Hill
Jessica Hill
Robert Hill
Terence Hill
Christopher Hill
Jenny Hill
Erica Hill
Deborah Hill
Cecilia Hillard
George Hillas
Louise Hiller
Joel Hillhouse
Julia Hilliard
Marisa Hilliard
Susan Hillier
Sheila Hillier
John Hillier
Argye Hillis
Noah Hillman
Aubrey Hillman
Edna Hillmann
Falk Hillmann
Ansel Hillmer
Robert Hills
Thomas Hills
Peter Hills
Danny Hills
Nathan Hillson
Steven A. Hillyard
Julian Hillyer
Clayton Hilmert
Mark Hilsenroth
Emma Hilton
Ivan Hiltpold
Mikko Hiltunen
Markus Hilty
Juha Himanen
Sayed Himatt
Tommi Himberg
Toshiki Himeda

Seiichiro Himeno
Misako Himeno
Austin Himes
Uwe Himmelreich
Xavier Hinaut
Dirk Hincha
Monique Hinchcliff
Kenneth Hinchcliff
Cody Hinchliff
Doug Hinchliffe
Erica Hinckson
M. Hind
Katherine Hinde
Anne Hinderliter
Sajedah Hindi
Sofi Hindmarch
Mark Hinds
Rupert Hinds
Terry Hinds, Jr.
Ellen Hines
Patrick Hines
Melissa Hines
Yaniv Hinits
Kim Hinkelmann
Patricia Hinkle
Jun Hino
Takao Hinoi
Melanie Hinojosa
Andrea Hinojosa-Azaola
Norihide Hinomoto
Winfried Hinrichs
Chris Hinrichs
Jenny Hinshaw
Damien Hinsinger
Shelley Hinsley
Peter Hinterdorfer
Christoph Hintermueller
David Hinton
Barry T. Hinton
Devon Hinton
Tina Hinton
Elanor Hinton
Sara Hintze
Rogier Hintzen
Niels Hintzen
Shuji Hinuma
Boris Hinz
Catarina Hioe
Kevin Hiom
John Hipp
Mark Hipp
James Hipp
Daniel Hippe
Franz Hippler
Christy Hipsley
Tohru Hira
Masahiro Hirai
Hiroki Hirai
Satoshi Hirakawa
Akihiro Hirakawa
Hideki Hirakawa
Y. Hirakawa
Yoshihisa Hirakawa
Masayuki Hiramatsu
Roberto Hiramoto
Tsutomu Hirano
Yukinori Hirano
Shinji Hirano
Katsuya Hirano
Toshihide Hirao
Takashi Hirasawa
Kensuke Hirasawa
Makoto Hirasawa
Akihiro Hirashiki
Ken-Ichi Hirata
Masayuki Hirata
Dai Hirata
Hiromi Hirata
Hiroaki Hirata
Ayumu Hirata
Kazuyuki Hiratsuka
Harukazu Hiraumi
Kenji Hirayama
Jun Hirayama
Yoshitaka Hirayama
Yositaka Hirayama
Bertrand Hirel
Kirsten Hirneisen
Shunsei Hirohata
Taniguchi Hirokazu
Ogata Hiromitsu
Moritoshi Hirono
Ikuo Hirono
Yoshitaka Hirooka
Kazuyuki Hirooka
Masamichi Hirose

Atsumi Hirose
Simon Hirota
Jeremy Hirota
Takeshi Hirota
Akatsu Hiroyasu
Munehara Hiroyuki
Johannes Hirrlinger
Alec Hirsch
Lawrence Hirsch
Oliver Hirsch
Cory Hirsch
Elizabeth Hirsch
Dania Hirsch
Ben Hirsch
Brad Hirsch
Raimund Hirschberg
Matthew Hirschey
Gideon Hirschfield
Lisa Hirschhorn
Jay Hirsh
Petra Hirsova
Martin Hirst
Andrew Hirst
Theodore Hirst
Robert Hirt
Lena Hirtler
Shizuko Hiryu
Hajime Hisaeda
Tadakazu Hisamatsu
Takashi Hisamatsu
Tamotsu Hisamatsu
Shin-Ichi Hisanaga
Shin-Ichi Hisasue
Peter Hiscock
Alexandra Hiscox
Andrew Hislop
Peter Hitchcock
Sara Hitchman
Patrick Hitchon
Taro Hitosugi
Wilbur Hitt
Mary Hitt
Akio Hiwatashi
Stefanie Hixson
Mahmut Hiz
Anita Hjelmeland
Anders Hjern
Jesper Hjortdal
Tim Hla
Wolfgang Hladik
Stephen Hladky
Kristen Hladun
Vladimir Hlady
Valentina Hlebec
Jaroslav Hlinka
Abdelkrim Hmadcha
Zakaria Hmama
Jay Hmielowski
Gabriel Hmimina
Jill Hnatiuk
May Ho
Peggy Ho
Quang Tri Ho
Sai-Yin Ho
Derek Ho
Shinn-Ying Ho
Paul Ho
Philip Wing-Lok Ho
Hon Ho
Wen-Chao Ho
James Ho
Roger Ho
Mandy Ho
Sheng Yow Ho
Ming-Chih Ho
Chun-Te Ho
Margaret S. Ho
Thien Ho
Chai-Ling Ho
Ye-Shih Ho
Hua David Ho
Roger Chun-Man Ho
Chen-Hsun Ho
David Ho
Mae-Wan Ho
Chen-Lung Ho
Brian Ho
Jessica Ho
Joshua Ho
Christopher Hoagstrom
Quyen Hoang
Minh Hoang Pham
Erik Hobbie
Michael Hobbins
Jamie Hobbs
Tom Hobbs

Suzanne Hobbs
Joanne Hobbs
Jean-Paul Hobbs
Matthew Hobbs
Alistair Hobday
Calvin Hobel
Sabrina Höbenreich
Oliver Hobert
Markus Hobert
Keith Hobson
David Hobson
Charles Hobson
John Hobson
Rick Hochberg
Natasha Hochberg
Andreas Hochhaus
Katharina Hochheiser
Alejandro Hochkoeppler
Hartwig Henry Hochmair
Sarah Hochman
Guy Hochman
Sonja Hochmeister
Binyamin Hochner
Christian Hochstim
Andreas Hochwagen
Howard Hock
Karlo Hock
Cord Hockemeyer
Joel Hockensmith
Gregory Hockerman
Gregory H. Hockerman
Carinna Hockham
Jane Hocking
Anne Hocking
Jean-Francois Hocquette
Moshe Hod
Ladislav Hodac
Mojgan Hodaie
Natasha Hodas
Emma Hodcroft
Mark Hoddle
Rebecca Hodes
James Hodge
Christopher Hodge
Andrew Hodge
Ian Hodge
Kristin Hodge
Kathie Hodge
Anne-Marie Hodge
Rebecca Hodge
Robert Hodges
Sara Hodges
David Hodgins
Stephen Hodgins
James Hodgkinson
Louis Hodgson
Timothy Hodgson
Emma Hodgson
Amr Hodhod
John Hodsoll
Emma Hodson-Tole
Emir Hodzic
Victor Hoe
Berthold Hoeckner
Imo Hoefer
Julia Hoefer
Thomas Hoefer
Sebastian Hoefert
Manfred Hoefle
Ulrich Hoeger
Sandra Hoegl
Richard Hoehn
Robert Hoehndorf
Annemieke Hoek
Janet Hoek
Bert Hoeksema
Hopi Hoekstra
Edward Hoekstra
Rosa Hoekstra
Timothy Hoellein
Christoph Hoeller
Thomas Hoellinger
Kim Hoelmer
Michael Hoelscher
Caroline Hoemann
Miriam Hoene
Thomas Hoenen
Janet Hoenicka
Margarethe Hoenig
Martin Hoenigl
M. Hoenigl
Pia Hoenscheid
Luke Hoeppner
Daniel Hoeppner
Nicolas Hoertel
Stefan Hoerzer

Corinne Hoesli
Peter Hoet
Jennifer A. Hoeting
Andrew Hoey
Anouschka Hof
Ivo Hofacker
Philipp Hofemeier
Markus Hofer
Stefan Hofer
Tim Hofer
Christoph Höfer
Soren Hoff
Erika Hoff
Uwe Hoff
Jesse Hoff
Barry Hoffer
Kenneth Hoffer
Lee Hoffer
Sabine Hoffjan
Matthew Hoffman
Charles Hoffman
Robert Hoffman
Dax Hoffman
Paula Hoffman
Brad Hoffman
Marian Hoffman
Brenton Hoffman
Gloria Hoffman
Noah Hoffman
Rhonda Hoffman
Nolan Hoffman
Andrew Hoffman
Jay Hoffman
Melissa Hoffman
Ryan Hoffman
Christy Hoffman
Keith Hoffman
Alexander Hoffmann
Wolfgang Hoffmann
Ary Hoffmann
Klaus Hoffmann
Petra Hoffmann
Heinrich Hoffmann
Frauke Hoffmann
Matthias Hoffmann
M. Hoffmann
Andrea Hoffmann
Hans Hoffmann
Till Hoffmann
Kathryn Hoffmann
René Hoffmann
Christopher Hoffmann
Markus Hoffmann
Rudiger Hoffmann
Michael Hoffmeister
Daniel Hofius
Jan Hofland
Johannes Hofland
Manfred Höfle
Courtney Hofman
Joerg Hofmann
Rainer Hofmann
Laurie Hofmann
Tobias Hofmann
Thomas Hofmann
Mathias Hofmann
Ulrich Hofmann
Wilhelm Hofmann
Alexander Hofmann
Marion Hofmann Bowman
Martin Hofmann-Apitius
Kirsten Hofmockel
Benjamin Hofner
Ivan Hofsajer
Wayne Hofstetter
Christoph Hofstetter
Robert Hofstra
Daniel Hoft
Romana Höftberger
Monica Höfte
Daniel Hogan
Deborah Hogan
Robert Hogan
Quinn Hogan
Ben Hogan
Catherine Hogan
Beth Hogans
Lee Hogarth
Pancras Hogendoorn
Hinze Hogendoorn
Saskia Hogenhout
Janneke Hogervorst
Eef Hogervorst
Philip Hogg
Robert Hogg
Ruth Hogg

J. Robert Hogg
Russell Hogg
Andy Hogg
Melissa Hogg
Benjamin Hoggan
Clive Hoggart
Günter Höglinger
Jacob Hoglund
Johan Hoglund
Johanna Höglund
Jean-Yves Hogrel
Todd Hogue
Ambika Hoguet
Peter Hohenstein
Jörg Höhfeld
Martin Hohmann-Marriott
Marilene Hohmuth Lopes
Herbert Hoi
Egbert Hoiczyk
Erling Hoivik
Z Hojabri
Ole Højberg
Johan Höjesjö
Aki Hoji
Radovan Hojs
Diego Hojsgaard
David Hokey
Heikki Hokkanen
Akishige Hokugo
Paul Johan Høl
Scott Holaday
Matthew Holahan
Tad Holak
Steve Holbrook
Michael Holbrook
Peter Holbrook
Luke Holbrook
Corley Holbrook
Mike Holcombe
Georgina Hold
Nicola Holden
Arun Holden
Matthew Holden
Richard Holden
Ronald Holden
Anthony Holder
Mark Holder
Benjamin Holder
Graham Holder
Peter Holder
Alexander Holderied
Friederike Holderried
Ricardo Holdo
Stephen Holdsworth
Lesca Holdt
Johannes Holfeld
Nick Holford
África Holguín
Andrij Holian
Michael Holick
Mike Holinstat
Karen Holl
Heather Holl
Sahana Holla
Carla Hollak
Martin Holland
Nicholas Holland
Brenden Holland
Michelle Holland
Nina Holland
Doron Holland
Claire Holland
Margaret Holland
Lisa Holland
Linda Holland
Richard Holland
Kristen Hollands
Gareth Hollands
Guillaume Hollard
Andrew Hollenbach
Christopher S. Hollenbeak
Morly Hollenberg
Walter Holleran
Matthew Holley
Casey Holliday
Jason Holliday
Andrew Hollingworth
Liz Hollingworth
Bruce Hollis
Karen Hollis
James Hollis
Brian Hollis
John Hollman
Mg Hollomon
Alison Holloway
Kathleen Holloway

Tanya Holloway
Per Höllsberg
Langston Holly
Lars Holm
Soren Holm
Astrid Holm
Charlotte Holm
Eric Holman
Peter Holmans
Scott Holmberg
Hans Christer Holmberg
Hans-Christer Holmberg
Rikard Holmdahl
Inger Holme
Marie Holmefur
Haya Holmegard
Wendy Holmes
Susan Holmes
Mark Holmes
Robert Holmes
Philip Holmes
Gregory Holmes
Charles Holmes
Bev Holmes
Ann Holmes
Clive Holmes
Gregory Holmes-Hampton
Margaret Holmes-Rovner
Robert Holmgren
Arne Holmgren
Irma Holopainen
Joseph Holoshitz
Pascal Hols
Dirk Holscher
Anders Holsgaard-Larsen
R. M. Damian Holsinger
Peter Holst
Jens Holst
Thomas Holstein
Daniel Holstein
Christine Holt
Matthew Holt
Vance Holt
Daniel Holt
Carl Holt
Kathleen Holt
Martin Holt
Chet Holterman
Aixuan Holterman
Harry Holthofer
Monique Höltig
Derald Holtkamp
Anthony Holtmaat
Helmut Holtmann
Lori Holtz
Tim Holtz
David Holtzman
Nick Holtzman
Kristin Holvik
Gregory Holwell
Erik Holy
Olaf Holz
Konstantin Holzapfel
Boris Holzapfel
Erika Holzbaur
Martin Holzenberger
Alison Holzer
David Holzer
Dirk Holzinger
Emily Holzinger
Iris Holzleitner
Gregg Homanics
Jihane Homann-Ludiye
Michael Hombach
Hayden Homer
Caroline Homer
Krisztian Homicsko
Bernhard Hommel
Carola Hommerich
László Homolya
Eduardo Homsi
Jaco Homsy
Gary Hon
Shigeru Honda
Masao Honda
Makoto Honda
Kazufumi Honda
Kazuhisa Honda
Kohsuke Honda
Yoshitomo Honda
Sumihisa Honda
Robert Hondal
David Hondula
Jan Hondzinski
David Hone
Thibault Honegger

Alois Honek
Johannes Hönekopp
Isobella Honeyborne
Amanda Honeycutt
Allen Honeyman
Peter Honeyman
Chuanxue Hong
Jiang Hong
Ling Hong
Chi-Chen Hong
Xinru Hong
Yi Hong
Keum-Shik Hong
Chwan-Yang Hong
Sunghoi Hong
Zhang Hong
Kyung Hong
Deli Hong
Lin Hong
Seongjin Hong
Hyun Seok Hong
Ni Hong
Shiyuan Hong
Seong-Yun Hong
Chen Hong
Yuling Hong
Jinkee Hong
Zhou Hong
Jiong Hong
Tzung-Pei Hong
Ye Hong
Sok Chul Hong
Jiaxu Hong
Gilbert Hong
Jing-Song Hong
Yiguo Hong
Kyung Sue Hong
Zhongkui Hong
Sun-Mog Hong
Peiying Hong
Sung-Ha Hong
Han Hongbin
Wei Hongjiang
Li Hongli
Wen Hongling
Kenichi Hongo
Lydia Hönig
Saul Honigberg
Henkjan Honing
Ken-Ichi Honjoh
Koichi Honke
Anna Honko
Patrick Honore
Partick Honore
N. Honzikova
Chantelle Hood
Rebecca Hood-Nowotny
Danny Hooftman
Ignace Hooge
Mia Hoogenboom
Bart Hoogenboom
Martine Hoogendoorn
Casper C Hoogenraad
Niek Hoogervorst
Govert Hoogland
Willem Hoogmoed
Shelley Hoogstraten-Miller
Erik Hooijberg
Magnus Hook
Sarah Hook
Sascha Hooker
Stephanie Hooker
Bryan Hooks
Merrilyn Hooley
Mark Hoon
Mrinalini Hoon
Koung Hoon Kook
John Hooper
Nigel Hooper
D. Craig Hooper
Cornelia Hooper
Tony Hooper
Daniel Hoops
Ewout Hoorn
David Hoos
Shiran Hooshmand
Donald Hoover
Joe Hoover
Anne Hoover-Miller
Paul Hooykaas
Marco Hoozemans
Ian Hope
Thomas Hope
Jayne Hope
Philip Hopewell
Lan Ho-Pham

Lan T. Ho-Pham
Robert Hopkin
Will Hopkins
John Hopkins
Richard Hopkins
Jason Hopkins
Nicholas Hopkinson
Joost Hopman
Béla Hopp
Kathryn Hoppe
Adam Hoppe
Thorsten Hoppe
Lydia Hopper
Waheeta Hopper
Christiane Hoppmann
Jessica Hoppstädter
James Hopson
Matthew Hoptman
Paul Hopwood
Muhammad Hoque
Chee Peng Hor
Jamila Horabin
Ivan Horácek
Martin Horak
John Horan
Michael Horberg
Craig Horbinski
P. Hore
Danny Horesh
Masatoshi Hori
Hiroaki Hori
Etsuro Hori
Masakazu Hori
Shigeo Horie
Yoshinori Horie
Rie Horie
Arata Horii
Takuro Horii
Daiki Horikawa
Momoko Horikoshi
Bjoern Horing
Yoshiyuki Horio
Masaru Horio
Takafumi Horishita
Nobuyuki Horita
Keisuke Horiuchi
Shiro Horiuchi
Sandrine Horman
Sheriar Hormuzdi
Sally Horn
Anselm Horn
Lisa Horn
Andrew Horn
Troy Hornberger
John Hornberger
M. Hornberger
Andrew Horne
Jon Horne
Benjamin Horne
Malcolm Horne
David Horne
Mary Horne
John Horner
Paddy Horner
Aidan Horner
Mark Horner
Alexander Horner-Devine
Willi Horner-Johnson
Jennifer Horney
Gwo-Jiun Horng
Claudia Horn-Hoffmann
Francis Hornicek
Jane Hornickel
Kurt Hornik
Peter Hornsby
Matthew Hornsey
Thomas Hornyak
Ioana Horodnic
David Horohov
Michal Horowitz
Arie Horowitz
Tzipi Horowitz-Kraus
Frank Horrigan
Nicholas Horrocks
Ben Horrocks
Andreas Horsch
Nelson Horseman
Jacquelyn Horsington
Alex Horsley
William Horsnell
David Horst
Maya Horst
Olaf Horstick
Eric Horstick
Rüdiger Horstkorte
Alexander Horswill

Craig Horswill
Bernardo Horta
Hugo Horta
Joaquín Hortal
Stefan Hortensteiner
Marcus Hortmann
Tibor Hortobagyi
John Horton
Jonathan Horton
Susan Horton
Julie Horton
William Horton
Milena Horvat
Gabor Horvath
Peter Horvath
Zsuzsanna Horvath
Keith Horvath
Martin Horvath
David Horwitz
Marc Horwitz
Richard Horwitz
Mitchell Horwitz
Nicole Horwood
L. Horwood
Hans-Peter Horz
Sean Hosein
Seyed Hossein Hoseinifar
Md. Hosen
Katrine Hoset
H Dean Hosgood
Moshe Hoshen
Takashi Hoshiba
Satoshi Hoshide
Masahiko Hoshijima
Tatsuhiko Hoshino
Robert Hoskin
Jay Hosking
Jason Hoskins
Paul Hoskisson
Peter Hosner
Hajime Hosoi
Masako Hosoi
Nobuko Hosokawa
Shinya Hosokawa
Jun Hosomichi
Takayuki Hoson
Satoyo Hosono
Jonas Hosp
Shahed Hossain
Md. Iqbal Hossain
Sk Tofajjen Hossain
Shahdat Hossain
Faisal Hossain
Mohammad Hossain
Firoz Hossain
Sheikh Hossain
Hany Hossam Eldien
Laure Hossard
Parviez Hosseini
Afshin Hosseini
Morteza Hosseini
Ahmad Hosseini-Safa
Sima Hosseinverdi
Leila Hosseinzadeh
Eric Hoste
H. Hoste
Caroline Hostetler
Camelia Hostinar
Morten Hostrup
Eric Hosy
Swetansu Hota
Richard Hotchkiss
Stefan Hotes
Markus Hoth
Benoît Ho-Tin-Noé
Carlos Hotta
Harumi Hotta
Michael Hottiger
Eugenio Hottz
Christian Hotz
Tingjun Hou
Zhuocheng Hou
Wei Hou
Weikun Hou
Ya-Ming Hou
Jinlin Hou
Ming-Feng Hou
Lin Hou
Zhonge Hou
Chunyan Hou
Youming Hou
Xiaohua Hou
Hong Hou
Hailong Hou
Yongqing Hou
Tiesheng Hou

Liping Hou
Shengping Hou
Fujun Hou
Pingping Hou
Zhanming Hou
Jiayi Hou
Jun Hou
Rein Houben
Michael Houbraken
Courtney W. Houchen
Claude Houdayer
Jonathan Houdmont
Nadine Houédé
Gunnar Houen
Catherine Hough
John Hough
Jon (Belfast) Houghton
Conor Houghton
Michael Houghton
Odette Houghton
Lauren Houghton
David Houghton
Peter Houk
Kathryn Houk
Ashley Houlden
Daniel Houle
Nicolas Houlie
Emilie Houliez
Laetitia Houot
Eric Houpt
Katherine Houpt
Thomas Houpt
Joseph Houpt
Dennis Hourcade
Christophe Hourde
Nicolette Houreld
Walid Houry
Reniqua House
Karen Houseknecht
Jonathan Houseley
Gregory Houseman
Stan Houston
Douglas Houston
James Houston
Michael Hout
Jon Houtman
Adriaan Houtsmuller
Peter Houweling
Roderick Houwen
Elisa Houwink
Kevin Hovel
Phillip Hövel
Nadine Hövelmeyer
Peter Hovenkamp
Iris Hovens
Erella Hovers
Sara Hoverter
Rc Hovey
Stirling G. Hovieson
Joppe Hovius
Martin How
Jonathon Howard
Kirsten Howard
Beatrice Howard
Ian Howard
Michelle Howard
Matthew Howard
Eric Howard
Scarlett Howard
Amber Howard
Brian Howard
Clive Howard-Williams
Gordon Howarth
Sam Howarth
Jennifer Howcroft
Rachel Howcroft
Philippa Howden-Chapman
Charles Howe
Alan Howe
Franklyn Howe
Amanda Howe
Laurence Howe
Martha Howe
Daniel Howe
Bruce Howe
Leann Howe
Gareth Howell
Hunt Howell
P. Lynne Howell
Viive Howell
Michael Howell
Nicholas Howell
Brittany Howell
Kate Howell
James Hower
Rosalind Howes

Melanie-Jayne Howes
Duncan Howie
Susan Howitt
Julia Howitt
Shanshan Howland
John Howland
M Howlett
Teuta Hoxha
Kate Hoy
Angela Hoye
Siguard Hoye
Bimba Hoyer
Wolfgang Hoyer
Marc Hoylaerts
Larry Hoyle
Lesley Hoyles
Cathrine Hoyo
Juan Hoyos
Joseph Hoyt
Anne Höytö
Estelle Hrabak
Sami Hraiech
Ivan Hrdy
Grzegorz Hreczycho
Michael Hristov
A. N. Hristov
Pavel Hrouzek
Terry Hrubec
Daniel Hruschka
Keith Hruska
Paul Hruz
Martina Hruzova
Michelle Hsiang
Nei-Yuan Hsiao
George Hsiao
Liang-Tsai Hsiao
Hsi-Min Hsiao
William Hsiao
Ying-Hen Hsieh
Yi-Hsien Hsieh
Michael Hsieh
Shi-Tong Hsieh
J. T. Hsieh
Chung-Cheng Hsieh
Ching-Hua Hsieh
Hui-Min Hsieh
Chia-Ling Hsieh
William Hsieh
Shie-Liang Hsieh
Yi-Chen Hsieh
Gillian Hsieh
Teng-Fu Hsieh
Ching-Lin Hsieh
Hsu-Liang Hsieh
Li-Chun Hsieh
Chi-Yuan Hsu
Wen-Lian Hsu
Yau Heiu Hsu
Mei-Yu Hsu
Chih-Cheng Hsu
Shih-Ming Hsu
Wei-Yen Hsu
Chun-Hua Hsu
Tsai-Ching Hsu
Ching-Sheng Hsu
Kuang-Hung Hsu
Yi-Hsin Hsu
Chung-Yao Hsu
Ming-Jen Hsu
Shu-Hao Hsu
Yao-Chun Hsu
Chun Liang Hsu
Pang-Hung Hsu
Yi-Chiang Hsu
Wei-Hsiu Hsu
Pin-I Hsu
Jonathan Hsu
Yeh-Liang Hsu
Hui-Chuan Hsu
Jue-Liang Hsu
Chia-Yang Hsu
Po-Lin Hsu
Jung-Jiin Hsu
Ching-Chi Hsu
Yao-Wen Hsu
Dong-Bo Hsu
Yung-Ho Hsu
Li-Chi Hsu
Chia-Chi Hsu
Kean Hsu
Janice Hsu
Shao-Jung Hsu
Jeffrey Hsu
Jason Hsu
Denise Hsu
Chia-Ling Hsu

Sylvia Hsu
Chao-Wei Hsu
Juchun Hsu
Douglas Hsu
Aaron Hsueh
Rui-Yuan Hsueh
Tai-Chiu Hsung
Guanjing Hu
Kun Hu
John Hu
Yanmin Hu
Dewen Hu
Xiaoxiang Hu
Peter Hu
Jianjun Hu
Bin Hu
Shijun Hu
Zihua Hu
Zhihong Hu
Qinghua Hu
Yanqing Hu
Xiaowen Hu
Wenhui Hu
Chang-Deng Hu
Zhibin Hu
Shengshou Hu
Zhenghui Hu
Hongzhen Hu
Dan-Ning Hu
Yin-Gang Hu
Qingzhong Hu
Guoqing Hu
Zhiguo Hu
Huijuan Hu
Yijuan Hu
Bo Hua Hu
Shao-Ji Hu
Chunhong Hu
Cheng-Jun Hu
Anyi Hu
Dan Hu
Xinhua Hu
Hui Hu
Hao Hu
Zhongli Hu
Shengwu Hu
Zhongmin Hu
Jiankun Hu
Jun Hu
Wei Hu
Gang Hu
Xiaoping Hu
Quanjun Hu
Jianping Hu
Yong Hu
Yifei Hu
Zhuo-Wei Hu
Bang-Chuan Hu
Hu Hu
Xiaotong Hu
Guo-Fu Hu
Li-Fang Hu
Jiehui Hu
Marian Hu
Kaiwen Hu
Weiping Hu
Jiandong Hu
Jie Hu
Jianxin Hu
Shijia Hu
Pingzhao Hu
Yuedong Hu
Qiao Hu
Mao-Bin Hu
Hao-Yuan Hu
Xiaosu (Frank) Hu
Tom Hu
Yaodong Hu
Xiaoke Hu
Qiyang Hu
Yuzheng Hu
Guang Hu
Yanbo Hu
Lun Hu
Hongli Hu
Jianzhong Hu
Li-Min Hu
Houxiang Hu
Zheng Hu
Lisong Hu
Huijian Hu
Junli Hu
Ming Hu
Yanqiu Hu
Yanfeng Hu
Dechang Hu

Wenhuo Hu
Changwei Hu
Haitao Hu
Baolan Hu
Yu-Lan Hu
Yue Hu
Zhen Hu
Xianglong Hu
Jiaxi Hu
Nien-Jen Hu
Qinhong Hu
Linden Hu
Che-Ming Hu
Ruifeng Hu
Nan Hu
Houchun Hu
Xiaogang Hu
Kelin Hu
Chaur-Jong Hu
Hongxia Hu
Xiao Hu
Mengqi Hu
Ai-Qun Hu
Jian Hu
Wei-Wen Hu
Xin-Sheng Hu
Zhen Hua Hu
Zhaonong Hu
Ping Hu
Xiao-Yang Hu
Xiaoqing Hu
Jin Hu
Rongbin Hu
Han-Hwa Hu
Min Hu
Xiuli Hu
Gene Hu
Qinglong Hu
Claire Hu
Yi Hu
Hongyu Hu
Xianxin Hua
Yuejin Hua
Jinping Hua
Jinlian Hua
Baojin Hua
Jing Hua
Wei Hua
Changchun Hua
Ting Hua
Yuchao Hua
Zonglu Hua
Yanjun Huan
Juan Huan
Sui Huang
Shengfeng Huang
Wendong Huang
Ying Huang
Chi-Ying Huang
Po-Hsun Huang
Ngan Huang
Tao Huang
Yu-Xia Huang
Yu Huang
Xuehui Huang
Yunda Huang
Ming-Shyan Huang
Yi-Shuian Huang
Wei-Chien Huang
Chang-Bing Huang
Xu-Feng Huang
Jingfei Huang
Yining Huang
Cheng-Yang Huang
Yhu-Chering Huang
Jee-Fu Huang
Zachary Huang
Jirong Huang
Jun Huang
Ruiwang Huang
Wan-Ting Huang
Hui Huang
Shile Huang
Yan Huang
Xuemei Huang
Chung-Feng Huang
Kunlun Huang
Xiaqin Huang
Cai Huang
Chao-Li Huang
Weishan Huang
Xingxu Huang
Bu-Miin Huang
Samantha Huang
Jian Huang
Guan-Hua Huang

Shau-Ku Huang
Andrew Huang
Yadong Huang
Felix Huang
He Huang
Hailiang Huang
Fangneng Huang
Tzung-Chi Huang
Xiaoqi Huang
Shu-Pin Huang
Chien-Ning Huang
Bing Huang
Zheng-Wei Huang
Wei Huang
Chang-Ming Huang
Junzhou Huang
Li-Tung Huang
I-Chueh Huang
Emma Huang
Cho-Ying Huang
Lili Huang
Xuqing Huang
David Huang
Huaxiong Huang
Zirui Huang
James Huang
Jiansheng Huang
Yijun Huang
Meng-Chuan Huang
Danfeng Huang
Yao-Wei Huang
Hanwen Huang
Chiung-Yu Huang
Yong Huang
Lu Huang
Hui-Chun Huang
I-Hsiu Huang
Changzhi Huang
Run-Yue Huang
Chao-Yuan Huang
Ya-Yi Huang
Hao-Jen Huang
Xin Huang
Jing Huang
Li-Rung Huang
Jenq-Wen Huang
Jeffrey Huang
Zhiying Huang
Haojie Huang
Steven Huang
Dong Huang
Chun-Jen Huang
Haochu Huang
Liang Huang
Zi-Gang Huang
Yaling Huang
Changjin Huang
Xiuzhen Huang
Teng-Yi Huang
Peng Huang
Angela Huang
Ji Huang
Chengmin Huang
Yuan Huang
Jingjing Huang
Yu-Jei Huang
Ruijie Huang
Ting-Zhu Huang
Xuelian Huang
Kuang-Tzu Huang
Shaobin Huang
Qifang Huang
Kai-Wen Huang
Chiung-Kuei Huang
Lin Huang
Jinsong Huang
Hao Huang
Yun Huang
Keke Huang
Yongmei Huang
Shih-Chia Huang
Jianbin Huang
Chao-Cheng Huang
Hsiang-Wen Huang
Bin Huang
Qiaojia Huang
Jian-Zhi Huang
Taosheng Huang
Yufeng Huang
Chen Huang
Fei Huang
Zhaofeng Huang
Xiaoming Huang
Guangbin Huang
Ying-Hsien Huang
Linshan Huang

Yi-Shin Huang
Fangjin Huang
Mengtian Huang
Fang Huang
Chin-Tser Huang
Chien-Sheng Huang
Qinlong Huang
Po-Chia Huang
Jason Huang
Ting Huang
Lan Huang
Libin Huang
Shaodan Huang
Guomin Huang
Zhongwei Huang
Kevin Huang
Yanyi Huang
Wenjing Huang
Jian-Guo Huang
Linfeng Huang
Yin Huang
Zonghai Huang
Qiaoqiao Huang
Tzu-Chou Huang
Yen-Hua Huang
Jiajin Huang
Chi-Chang Huang
Yongxiang Huang
Jianguo Huang
Qinghui Huang
Chang-Wen Huang
Jiun-Lang Huang
Rui Huang
Juan Huang
C. W. Huang
Haishui Huang
Lijie Huang
Junming Huang
Pintong Huang
Yan-Jang Huang
Wenhan Huang
Jingyu Huang
Jihong Huang
Shi-Wei Huang
D. Huangfu
Feng Huashan
Klaus Hubacek
Jaroslav Hubacek
Monica Hubal
Jean-Pierre Hubaux
Stevan Hubbard
Timothy Hubbard
Rebecca Hubbard
Jeffrey A. Hubbell
Ryan Hubble
Ludwig Huber
Peter Huber
Thomas Huber
Alexa Huber
Michael Huber
Robert Huber
David H. Huber
Christian Huber
Roland Huber
Brian Huber
Gilles Huberfeld
Markus Huber-Lang
Elisabeth Huber-Sannwald
Pierre Hubert
Jan Hubert
Stefan Huber-Wagner
Michael Hübler
Jean-Jacques Hublin
Marc Hübner
Claudia Hübner
Elise Huchard
Megan Huchko
Jane Huckerby
Rachel Huckfeldt
Anke Huckriede
Luis Huckstadt
Mark Huckvale
Ahmed Huda
Sophie Huddart
Thomas S. Huddle
Jennifer Huddleston
Daniel Huddleston
Michael Hudecek
Csilla Hudek
Anthony Hudetz
Louanne Hudgins
Tara Hudiburg
Clifford Hudis
Teresa Hudock
Catherine Hudon
Kristelle Hudry

Quanah Hudson
James Hudson
Matthew Hudson
Joanna Hudson
Joanne Hudson
Isabelle Hue
Kay Huebner
Johannes Huebner
Gesche Huebner
David Huebner
Lindsay Huebner
Markus Huebscher
Tania Huedo-Medina
Julianne Huegel
Martin Huellner
Michael Huen
A. Huerta
Alicia Huerta-Chagoya
Rodrigo Huerta-Quintanilla
Sara Huerta-Yepez
Marilyn Huestis
Markus Huettel
Fabian Huettig
Falk Hüettmann
Joel Huey
Harold Huff
Mark Huff
Emily Huff
Christine Huffard
Damien Huffer
Jeff C Huffman
Derek Huffman
Matt Huffman
Gary Huffnagle
Matthew Hufford
Laura Hug
Christopher Hug
Sylvain Hugel
Christina Hugenschmidt
Jonathan Huggins
Christopher Hughes
James Hughes
Austin Hughes
Diarmaid Hughes
Robin D. Hughes
Ieuan Hughes
William Hughes
Grant Hughes
John Hughes
Jane Hughes
Colin Hughes
Maria Hughes
Derralynn Hughes
Gethin Hughes
Cris Hughes
Joel Hughes
Alun Hughes
Margaret Hughes
Michael Hughes
Bob Hughes
David Hughes
Lyndsay Hughes
Mark Hughes
Stephen Hughes
Alicia Hughes
Patrick Hughes
Adam Hughes
Kerri-Ann Hughes
Elizabeth Hughes
Myra Hughey
Martin Hugh-Jones
Richard Hughson
Julien Hugon
David Huguenot
Elisabeth Huguet
Gemma Huguet
Florence Huguet
Jimmy Huh
Jin Hoe Huh
Winston Huh
Dongeun Huh
Min Huh
Jun Huh
Ragnar Huhn
Konstantin Huhn
Ilpo Huhtaniemi
Chi-Chung Hui
Kenrie Hui
Zhang Hui
Zhao Hui
Francis Hui
Edward S. Hui
Lisa Hui
Elliot Hui
S. Hui
Zhouguang Hui

Suzanne Huijts
Kristi Huik
Heikki Huikuri
Eugénie Huillet
Barbara Huisamen
Geertjan Huiskamp
Thierry Huisman
Monique Huisman
John Huisman
Edgar Huitema
Jan Huizinga
Philippe Hujoel
Maciej Huk
Sabine Huke
Scot Hulbert
Jiri Hulcr
Mark Hull
Pincelli Hull
Elizabeth Hull
Mark Hulme
Charles Hulme
Florence Hulot
Matthias Hüls
Jan Hulscher
Janina Hülsebusch
Frank Hülsemann
Hilleke Hulshoff Pol
H Hulst
Dan Hultmark
Michael Hultstrom
L. A. Hulvershorn
Ryan Hum
Robert Hum
Hannelie Human
Sajid Humayun
James Humble
Joseph Humble
David Hume
Kelly Hume
Tatyana Humle
Thomas Hummel
Hans E. Hummel
Jurgen Hummel
Edith Hummler
Christian Humpel
Jay Humphrey
Sean Humphrey
Mike Humphreys
Lee Humphreys
R. Keith Humphries
Romney Humphries
Mark Humphries
Jan Humplik
Thomas Hund
Kris Hundertmark
Vanora Hundley
Tábita Hünemeier
Chien-Ching Hung
Kuo-Hsiang Hung
Tai-Ho Hung
Clark Hung
Rayjean Hung
Ivan Fn Hung
Chao-Hung Hung
Man-Hsin Hung
Chi-Chih Hung
Chin-Sheng Hung
Pei-Hsuan Hung
Yu-Chiang Hung
Jia-Jang Hung
Albert Hung
Hsiao-Chun Hung
Shu-Chen Hung
Hung–Hsu Hung
Ying Hung
Hsin-Yi Hung
Lin Hung
Shih-Han Hung
Chi Hung
Carol Hunja
Tracy Hunley
Iva Hunova
Mauricio Hunsche
Peter Hunt
David Hunt
Arthur Hunt
W. Hunt
Gillian Hunt
Alan Hunt
Amelia Hunt
Martin Hunt
Natalie Hunt
Sally Hunt
Kathleen Hunt
Mary C. Hunt
Ray Hunt

Ann Hunt
Anne Hunt
Nicola Hunt
Peter Hunter
Wayne Hunter
David Hunter
Luke Hunter
Gary R Hunter
Ryan Hunter
Margaret Hunter
Aimee Hunter
Robert Hunter
Stacy Hunter
Starling Hunter
Paul Hunter
Catherine Hunter
Mitchell Hunter
Blake Hunter
Marycarol Hunter
George Hunter
Felicity Huntingford
James Huntington
Byki Huntjens
John Huntriss
Lynn Huntsinger
Ran Huo
Qiang Huo
Li-Jun Huo
Teh-Ia Huo
Yunlong Huo
Jinhai Huo
Heqiang Huo
Sylvain Huon
Michael Hupfer
Stacey Huppert
Berthold Huppertz
Anna Huppler
Katherine Huppler Hullsiek
Muhammad Huq
Rumana Huque
Yoonkang Hur
Ji-Won Hur
Seung-Ho Hur
Yun Kyoung Hur
Pilwon Hur
Mirna Hur
Hilary Hurd
Yasmin Hurd
Peter Hurd
Toby Hurd
Anya Hurlbert
John Hurlbert
James Hurley
Jane Hurley
David Hurley
Jennifer Hurley
Samuel Hurley
Christof Hurschler
Robert Hurst
Douglas Hurst
Thomas Hurst
Mark Hurst
Luis Hurtado
Reyna Hurtado
Uriel Hurtado Páez
Jorge Hurtado-Gonzales
Matthew Hurteau
Jørn Hurum
Jerard Hurwitz
Richard Hurwitz
Matloob Husain
Brian Husband
Geir Huse
Gunnar Reksten Husebø
Holger Husi
Sally Huskinson
Knut Husmann
Mikael Huss
Anke Huss
Volker Huss
Tahziba Hussain
Mahmood Hussain
Tahir Hussain
Sazid Hussain
Saira Hussain
Ahm Enayet Hussain
Muhammad Hussain
Mohammad Hussaini
Jamal Hussaini
Khaled Hussein
Almontaser Bella Hussein
Mohamed Hussein
Tracy Hussell
Michael Hust
John Hustad
Christopher Huston

Wilhelmina Huston
Michael Huston
Rolf Hut
Cendri Hutcherson
Steven Hutcheson
Joshua D Hutcheson
Jack Hutcheson
Michael Hutchings
Michael Hutchins
Angela Hutchinson
Edward Hutchinson
Mark Hutchinson
Ian Hutchinson
Jim Hutchinson
Shirley Hutchinson
William Hutchison
R. Matthew Hutchison
Brian Hutchison
Cornelia Huth
Hendrik Huthoff
Andreas Hutloff
Dietmar Hutmacher
Thomas Hutson
Peter Hutson
Alan Hutson
Lara Hutson
Stefan Hüttelmaier
Maik Huttemann
Alexander Hüttenhofer
Stephan Hutter
Birgit Hutter-Paier
Alexandra Huttinger
David Hutton
Brian Hutton
Virginia Hutton-Estabrook
Gyorgy Hutvagner
Mara Hutz
Hugo C. Huurdeman
Charlie Huveneers
A. Huvet
Julie Huxley-Jones
Adrianne Huxtable
Kris Huygen
Flavia Huygens
Jean-René Huynh
Bao Lam Huynh
Geert Huys
Quentin Huys
Raoul Huys
Ann Huysseune
Todd Huzar
Snehalata Huzurbazar
Lars Hviid
Jenn-Kang Hwang
Ming-Jing Hwang
Pung-Pung Hwang
Bing-Fang Hwang
Kyu-Baek Hwang
Peter Hwang
Nathaniel Hwang
Eric Hwang
Juey-Jen Hwang
Jason Hwang
Jeong-Min Hwang
Wen-Li Hwang
Dong-Uk Hwang
Priscilla Hwang
Jun Eul Hwang
Hau-Hsuan Hwang
Heungsun Hwang
Tzung-Jeng Hwang
Janice Hwang
Ki-Chul Hwang
Daw-Yang Hwang
Ji-Young Hwang
Yu Kyeong Hwang
Hyonson Hwang
Y. S. Hwang
Andrew Hwang
Shen-An Hwang
Wei-Ting Hwang
Tsong-Long Hwang
Chiimin Hwu
Doug Hyatt
Kevin Hybiske
Daniel Hyde
Melissa Hyde
R. Katherine Hyde
Jenny Hyde
Embriette Hyde
Fahmeed Hyder
Ayaz Hyder
Omar Hyder
John Hyett
Sarah Hykin
Michael Hyland

Emily Hyle
Elaine Hylek
Anthony Hyman
Weston Hymas
Michael Hynes
Wayne Hynes
Kim Hynes
Allison Hyngstrom
Tuulia Hyötyläinen
Stephen Hyslop
Paul Hyslop
Natalie Hyslop
Richard Hyson
Jukka Hytönen
Jari Hyttinen
Sang-Hwan Hyun
Seung Hyup Hyun
Kim Hyun Koo
Timo Hyypia
Margherita Iaboni
Guido Iaccarino
Leonardo Iaccarino
Matthew Iacchei
Gianluca Iacobellis
Maurizio Iacobone
Giuseppe Iacomino
Gwenllian Iacona
Roberto Iacovelli
Daniela Iacoviello
Costantino Iadecola
Pietro Iaffaldano
Stefania Iametti
Adriana Iamnitchi
Luca Iani
Giuseppe Ianiri
Gianluca Ianiro
Paola Iannello
Leopoldo J. Iannone
Luciana Iannuzzi
Robert Iansek
Raffaella Iantomasi
Kelly Iarosz
Felice Iasevoli
Alessandra Iavello
Ivo Iavicoli
Lourdes Ibanez
Agustin Ibanez
Juan Jose Ibanez
Javier Ibañez
Inés Ibáñez
Juan D. Ibanez-Alamo
Alejandro Ibañez-Costa
Jean-Pierre Ibar
Fernando R Ibarra
Beatriz Ibarra-Molero
Ximena Ibarra-Soria
Elena Ibarz
Michael Ibbotson
Tobias Ibfelt
Samad Ibitokou
Morufu Ibitoye
Mohamed Ibrahem
Muntaser Ibrahim
Nizar Ibrahim
Nahla Ibrahim
Ahmed Ibrahim
George Ibrahim
El Cherif Ibrahim Ibrahim
Mohamed Izham Ibrahim
Elmira Ibrahim
N. Ibrahim
Salam Ibrahim
George M. Ibrahim
Joseph Icenogle
Tomoko Ichibangase
Yasunori Ichihashi
Hidenori Ichijo
Naotsugu Ichimaru
Yoshinobu Ichimura
Genki Ichinose
Masao Ichinose
Koji Ichinose
Takao Ichioka
Rebecca Ichord
Tomomi Ide
Satoru Ide
Takeshi Ide
Tatsuya Ide
Trey Ideker
Evgeny Idelevich
Marco Idiart
Alexander Idnurm
Yasuo Ido
Fernando Idoate
Miguel Angel Idoate
Travis Idol

Omar Idrissi
William Idsardi
Masaki Ieda
Motoyuki Iemitsu
Riccardo Ientile
Marianthi Ierapetritou
Demian Ifa
Imran Iftikhar
Abir Igamberdiev
Makoto Igarashi
Jotaro Igarashi
Hirotaka Igarashi
Tsutomu Igarashi
Ernesto Igartua
Takeshi Igawa
Peter Igaz
Herrade Igersheim
Branislav Igic
Joseph Igietseme
Mar Iglesias
José Roberto Iglesias
Alberto Iglesias
Sergio Iglesias
Isabel Iglesias
Maria Isabel Iglesias Diaz
Alejandro Iglesias Linares
Raquel Iglesias-Fernández
Isabel Iglesias-Platas
Cifre Ignacio
Anita Ignatius
Taisen Iguchi
Hidekatsu Iha
Masafumi Ihara
Kaori Ihida-Stansbury
Kate Ihle
Michaela Ihle
Michael Ihnat
Anna Ihnatowicz
Shinsuke Iida
Koji Iihara
Hideki Iijima
Masamitsu Iino
Antti Iivanainen
Hisashi Iizasa
Yoko Ijima
Shigeho Ijiri
Auke Ijspeert
Richard Ijzerman
Yoshinori Ikarashi
Akihiro Ikeda
Stephen Ikeda
Nayu Ikeda
Hideki Ikeda
Yoshio Ikeda
Koji Ikeda
Kei Ikeda
Joanne Ikeda
Kota Ikeda
Yasumasa Ikeda
Yoichiro Ikeda
Ryoukichi Ikeda
Tempei Ikegame
Tetsuro Ikegami
Taro Ikegami
Shiro Ikegawa
Yuji Ikegaya
Hidetoshi Ikeno
Yukinobu Ikeya
Karen Ikin
Ignatios Ikonomidis
Moses Ikpeme
Salima Ikram
Mitsu Ikura
Tohru Ikuta
John Ikwuobe
Lena Ilan
Kumaravel Ilangovan
Beatriz Ilari
Marianne Ilbert
Adeodat Ilboudo
Will Iles
Winfried Ilg
Harun Ilhan
Maria Ilheu
Jung Ilhyo
George Iliakis
Aris Ilias
Dusko Ilic
Dragan Ilic
Jasminka Ilich
Iliyan Iliev
Jeffrey Iliff
Thomas Iliffe
Elena Ilina
Olga Ilinskaya
Othon Iliopoulos

Dimitrios Iliopoulos
Zubairu Iliyasu
Miriam Illa
Sebastián Illanes
Andres Illanes
Peter Illes
Harald Illges
Robert Illing
Katharina Ilm
Tanja Ilmarinen
Titilayo Ilori
Natalia Ilyushina
Wan-Taek Im
Seung-Soon Im
Hiroshi Imai
Yutaka Imai
Enyu Imai
Yuuki Imai
Yumi Imai
Kohsuke Imai
Kazunori Imaizumi
Satoshi Imaizumi
Fumiaki Imamura
Yutaka Imamura
F. Imamura
Takeshi Imamura
Yuichi Imanaka
Tetsuji Imanaka
Kyoko Imanaka-Yoshida
Alex Imas
Isabelle Imbert
Anne Imberty
Andrey Imbs
Gwenaël Imfeld
Roland Imhoff
John D. Imig
Jim Imlay
Swapna Immani
Stephan Immenschuh
Franz Immer
Richard Immink
Maarten Immink
Matthias Imöhl
Genji Imokawa
Issei Imoto
Daniela Impellizzeri
Francesco Imperi
Barbara Imperiali
Daniel Impoinvil
Asma Imran
Mudassar Imran
Suzan Imren
Maria Imtiaz
Mohammad Imtiaz
Takeshi Inagaki
Masaki Inagaki
Masatoshi Inagaki
Tristen Inagaki
Naoki Inagaki
Naoyuki Inagaki
Hiroyuki Inagawa
Kunihiro Inai
Shinako Inaida
Behcet Inal
Tina In-Albon
Joji Inamasu
Maneesha Inamdar
Mandar Inamdar
Omer Inan
Daniel Inaoka
Johji Inazawa
D. Inbakandan
Aida Inbal
Yoel Inbar
Can Ince
Sara Incera
Guido Incerti
Patrik Inderbitzin
Giuseppe Indolfi
Ciro Indolfi
Sabarish Indran
Nibaldo Inestrosa
E. Infusino
Alberto Inga
Puraskar Ingale
Brian Ingalls
Robin Ingalls
Maria Ingaramo
Susham Ingavale
Sven Ingebrandt
Jorgen Ingebrigtsen
F. Ingegnoli
Magnus Ingelman-Sundberg
Pablo Ingelmo
Kurt Ingeman
Roger Ingham

Patrick Ingiliz
Atul Ingle
Matthew Inglis
Holly Ingraham
James Ingram
Maia Ingram
Jennifer Ingram
Wendy Ingram
Katherine Ingram
Diego Ingrosso
Supawadee Ingsriswang
Gerardo Iñiguez
Nicole Iniguez-Ariza
Ahmet Inkaya
Kyung-Soo Inn
Michele Innangi
John Innes
Steve Innes
Hamish Innes-Brown
Alessandro Innocenti
Naohiro Inohara
Masanao Inokoshi
Luis Inostroza
Kimiko Inoue
Satoshi Inoue
Masahiro Inoue
Hiroki Inoue
Machiko Inoue
Ken Inoue
Kazuo Inoue
Akiomi Inoue
Ken-Ichiro Inoue
Shigeaki Inoue
Taku Inoue
Manabu Inoue
Hiroyasu Inoue
Shintaro Inoue
Naoki Inoue
Yuki Inoue
Masayoshi Inoue
David Inouye
Michael Inouye
Robert Insall
Heribert Insam
Beverly Insel
Nathan Insel
Hazel Inskip
Ryan Insolera
Marcos Intaglietta
Kiao Inthavong
Ali Intizar
Tom Inui
Shigeki Inui
Gaetano Invernizzi
Pietro Invernizzi
Thomas Inzana
Jason Inzana
Babis Ioannou
John Ioannou
Francesca Iodice
Luisa Iommarini
Andrei Ionescu
Maksim Ionov
Laura Iop
Giulia Iori
Alfonso Iorio
Lorenzo Iorio
Matthew Iorio
Marco Iosa
Nicola Iotti
Nicole Iovine
Radu Iovita
Y. Tony Ip
Nancy Ip
Dennis Ip
Philip Ip
John Ipsen
Syed Iqbal
M. Iqbal
Mohammad Perwaiz Iqbal
Atif Iqbal
Zafar Iqbal
Ramsha Iqbal
Hafiz Iqbal
Maria Iraburu
Concetta Irace
Armin Iraji
Pouya Iranmanesh
Alex Iranzo
Jaime Iranzo
Fuad Iraqi
Ahmed Iraqi Ahmed Iraqi
Carlos Irarrazabal
Shahriar Iravanian
Alex Ireland
Keith Ireton

M. T. Irfan
M. Okan Irfanoglu
Xabier Irigoien
Maria Cláudia Irigoyen
Nerea Irigoyen
Manuel Irima
Jose Irimia
Andrey Irintchev
Uwe Irion
Joel Irish
Leah Irish
Jean-Olivier Irisson
Ryosuke Iritani
Marcello Iriti
Takanori Iriuchishima
Stephan Irle
Randall Irmis
Duncan Irschick
Humayun Irshad
Muhammad Irshad
Silvia Irusta
Risha Irvin
Darrell Irvine
Katharine Irvine
Brian Irving
Hyacinth Irving
Samantha Irving
Michael Irwig
David Irwin
Lourdes Isaac
Methvin Isaac
Mathew Isaac
Sibren Isaacman
Stuart Isaacs
John T Isaacs
Jennifer Isaacs
Aaron Isaacs
Petros Isaakidis
Dragan Isailovic
Shuji Isaji
Yoshitaka Isaka
Amin Isanejad
Rosario Isasi
Scott Isbell
Wolf Isbert
Geoffrey Isbister
Sachiko Iseki
Ryuta Iseki
Catherine Isel
Berend Isermann
Sharon Isern
Joan Isern
Kimberley Isett
Ivana Išgum
Anuar Ishak
Waguih Ishak
Sauid Ishaq
Yoshitaka Ishibashi
Kenji Ishibashi
Shuta Ishibe
Susumu Ishida
Andrew Ishida
Yuko Ishida
Takafumi Ishida
Miho Ishida
Yasuhiro Ishiga
Yasushi Ishigaki
Hiroshi Ishiguro
Akira Ishihama
Y. Ishihara
Hirofumi Ishihara
Masae Ishihara
Hisamitsu Ishihara
Yasutoshi Ishihara
Shunsuke Ishii
Ken Ishii
Akira Ishii
Isao Ishii
Masaru Ishii
Hideki Ishii
Kuniaki Ishii
Takeshi Ishii
Kojiro Ishii
Hiroki Ishii
Sumio Ishijima
Yuichi Ishikawa
Yoshihiro Ishikawa
H. Ishikawa
Yoshiki Ishikawa
Goro Ishikawa
Tetsuya Ishikawa
Toshizo Ishikawa
San-E Ishikawa
Atushi Ishikawa
Tokiro Ishikawa
Kiyotake Ishikawa

Takaki Ishikawa
Naoko Ishikawa
Rieko Ishima
Yoshiro Ishimaru
Yoshiko Ishimi
Takuya Ishimoto
Tomoko Ishino
Shoichi Ishiura
Shin'Ichi Ishiwata
A. Ishiwata
Nobukazu Ishizaka
Atsuko Ishizuya-Oka
Alexander Ishov
Andrea Isidori
Emanuele Isidori
T. Isik
Yasuko Isikawa
Marcus Ising
Dalia Iskander
Mozaffarul Islam
Salim Islam
Md Islam
Farzana Islam
Shahidul Islam
Tanvir Islam
Lucrezia Islam
Silvia Islam
Shams Ul Islam
Ahsan Islam
Ishrat Islam
Syed Islam
Karin Isler
Anthony Isles
Nahed Ismail
Salmah Ismail
Wael Ismail
Murray Isman
Bob Isman
Leyla Ismayilova
Jean-Charles Isner
Satoshi Isobe
Sachiko Isobe
Jun Isoe
Toshiaki Isogai
Masanori Isogawa
Yoichiro Isohama
Miriam Isola
Daniela Isola
Lori Isom
Clay Isom
Antti Isomäki
Michael Ison
Nicole Ison
Mak Ison
Risa Isonaka
Philippe Isope
Esther Isorna
Ayako Isotani
Theocharis Ispoglou
Mark Israel
Emmanuelle Issakidis-Bourguet
Manish Issar
Rachele Isticato
Czigler Istvan
Vera Istvanovics
Haruhiko Itagaki
Takashi Itahashi
Keiji Itaka
Shoji Itakura
Hirotaka Itakura
Keiichi Itatani
Roxane Itier
Harri Itkonen
Taisuke Ito
Yasuhiro Ito
Shinya Ito
Yoshihiro Ito
Masaya Ito
Junya Ito
Yuri Ito
Takashi Ito
Yasuki Ito
Yu Ito
Shigeki Ito
Hikaru Ito
Minami Ito
H. Ito
Hideki Ito
Satoko Ito
Keisuke Ito
Kouta Ito
Yoko Ito
A. Ito
Ken Itoh
Kyoko Itoh
Kanako Itoh

Shiro Itoi
Gail Itokazu
Richard Ittenbach
Wannaporn Ittiprasert
Iñigo Iturbe-Ormaetxe
Yasser Iturria Medina
Miren Iturriza-Gomara
Luigi Iuliano
Norberto Iusem
Mircea Ivan
Cristina Ivan
Lacramioara Ivanciu
Renata Ivanek
Viatcheslav Ivanenko
Fabrice Ivanes
Andrada Ivanescu
Alexey Ivanov
Alexander Ivanov
Dmitri Ivanov
Ivan Ivanov
Nikolai Ivanov
Andrei Ivanov
Alex Ivanov
Milan Ivanov
Vadim Ivanov
Bobi Ivanov
Elena Ivanova
Saska Ivanova
Alla Ivanova
Diana Ivanova
Juraj Ivanyi
Juliana Ivar Do Sul
Emma Ivarsson
Angela Ivask
Domagoj Ivastinovic
Richard Ivell
Rebecca Ivers
Kevin Ivers
John Iversen
Rasmus Iversen
Marjolein Iversen
Michael Iverson
Vladimir Ivezic
Maria Lucia Ivo
Benjamin Ivorra
Sarah Ivory
Kazuya Iwabuchi
Kazuhisa Iwabuchi
Toshiyuki Iwahori
Naoharu Iwai
Leo Iwai
Moriya Iwaizumi
Yasuko Iwakiri
Atsushi Iwama
Tomotada Iwamoto
Jun Iwamoto
Takayuki Iwamoto
Ritsuko Iwanaga
Adam Iwanicki
Andrew Iwaniuk
Megumi Iwano
Luke Iwanowicz
Erik Iwarsson
Yuka Iwasaki
Mayumi Iwasaki
Yoshiaki Iwasaki
Masanori Iwasaki
Kenta Iwasaki
Kengo Iwasaki
Tae Iwasawa
Yoshio Iwashima
Masaya Iwashita
Atsushi Iwata
Takeshi Iwata
Koichi Iwata
Hiroyoshi Iwata
Hiroshi Iwata
Takashi Iwata
Junichi Iwata
Minoru Iwata
S. Iwata
Keiko Iwata
Hirotsugu Iwatani
Keiichi Iwaya
Juliet Iwelunmor
Azuka Iwobi
Sushma Iyengar
Sujatha Iyengar
Nandini Iyer
Arun Iyer
Jyoti Iyer
Parameswar Iyer
Easwar Krishna Iyer
Parameswaran Iyer
Aarti Iyer
Swami Iyer

Sunao Iyoda
Reza Izadpanah
Ana Izcue
Bérengère Ize
David Izhaky
Ruvim Izikson
Jacques Izopet
Javier Izquierdo
Maria José Izquierdo Rico
David Izquierdo-Garcia
Keise Izuma
Yuichi Izumi
Yukitoshi Izumi
Yasuhiko Izumi
Hirohisa Izumi
Yasuhiro Izumiya
Takehiro Izumo
Francesca Izzi
Angelo Izzo
Bradley J Roth
William Ja
Lindsay Jaacks
Laura Jaakola
Sairam Jabba
Abdul Jabbar
Mbemba Jabbi
Randa Jabbour
Kausar Jabeen
Tarek Jaber
Piotr Jablonski
Nina Jablonski
Mary Ann Jabra-Rizk
Martin Jaburek
Rodrigo Jacamo
Karen Jaceldo-Siegl
Ewa Jacewicz
David Jachowski
Alessandro Jacinto
Estela Jacinto
Rachael Jack
Helen Jack
Goldstone Jack
William Jackman
Suzanne Jackowski
Stefan Jackowski
Christian Jackowski
Alan Jackson
Mary Jackson
Robert Jackson
Frankie Jackson
David Jackson
Malcolm Jackson
Christopher Jackson
Meyer Jackson
Wesley Jackson
Craig Jackson
Nicki Jackson
Colin Jackson
Michelle Jackson
Melissa Jackson
Charles Jackson
Glen Jackson
John Jackson
Elizabeth Jackson
James Jackson
Charlene R. Jackson
Brian Jackson
Jeffrey Jackson
Philippa Jackson
Rodney Jackson
Daral Jackwood
Christopher Jacob
Naduparambil Jacob
Francis Jacob
Suma Jacob
Stefan Jacob
Yael Jacob
Donna Jacob
Joseph Jacob
Merle Jacob
Heidi Jacobe
Frank Jacobi
Judith Jacobi
William Jacobs
Enno Jacobs
Louis Jacobs
Richard Jacobs
Wilco Jacobs
J. Jacobs
David Jacobs
Robert Jacobs
W. Bradley Jacobs
Jan Jacobs
Jon Jacobs
Jonathan Jacobs
Michael Jacobs

Laura Jacobs
Jelle Jacobs
Reinhilde Jacobs
Douglass Jacobs
Chris Jacobs
Doris Jacobs
David Jacobs, Jr.
Ilse Jacobsen
Frank Jacobsen
Dean Jacobsen
Kathryn Jacobsen
Joyce Jacobsen
Marcelo Jacobs-Lorena
Jeffrey Jacobson
Matthew Jacobson
Karen Jacobson
Kathryn Jacobson
Bailey Jacobson
Deborah Jacobs-Sera
Anders Jacobsson
Mathys Jacobus Redelinghuys
David Jacoby
Mathieu Jacomy
Alain Jacot
Vincent Jacquemet
Patrick Jacquemin
Guillaume Jacquemin
Vincent Jacquemond
Sebastien Jacquemont
Mario Jacques
Hélène Jacques
Constanza Jacques
Claire Jacques
Timothee Jacquesson
Jennifer Jacquet
Alain Jacquet
Arnaud Jacquier
Evelyne Jacqz-Aigrain
Hervé Jactel
Sudarshan Jadcherla
Priyanka Jadhav
Amir Jadidi
Pooja Jadiya
Nauman Jadoon
Michel Jadoul
Will Jaeckle
Gerhard Jaeger
Susanne Jaeger
Susanne Jaeggi
Anja Jaeschke
Tazeen Jafar
Peyman Jafari
Amirhossein Jafari Bidhendi
Klaus Jaffe
Elaine Jaffe
Sagi Jaffe-Dax
Samie Jaffrey
Nicole Jaffrezic-Renault
Aravindakshan Jagadeesan
Bharathi Jagadeesan
Pradeep Babu Jagadeesh Reddy
Guru Jagadeeswaran
Ramasamy Jagadeeswaran
J. Jagadish
Devan Jaganath
Saravana Kumar Jaganathan
Narasimhan Jagannathan
Martine Jager
Franc Jager
Henriette Jager
Benedikt Jäger
Jonathan Jaggar
Ann Jagger
Pamela Jagger
Meena Jaggi
Amteshwar Jaggi
Krzysztof Jagla
Susan Jaglal
Sadaf Jahan
Younes Jahangiri
Hamid Jahed
Md Jamiul Jahid
Albrecht Jahn
Regine Jahn
Klaus Jahn
Daniela Jahn
Ullrich Jahn
Alex Jahn
K. Jahn
Reinhold Jahn
Inge Jahn
Marlene Jahnke
Lisa Jahns
Frode Jahnsen
Enrique Jaimovich
Mukesh Jain

Vivek Jain
Pooja Jain
Maneesh Jain
Neeta Jain
Abhinav Jain
Vikas Jain
Kavita Jain
Shushant Jain
Arsh Jain
Tarun Jain
Rajeev Jain
Sarika Jain
Rohit Jain
Aditya Jain
Yogesh Jain
Anshika Jain
Richard Jaine
Tang-Her Jaing
Stephanie Jainta
Dinesh Jaishankar
Deb Jaisi
Frederic Jaisser
Stephane Jaisson
Pundrik Jaiswal
Mamta Jaiswal
Vandana Jaiswal
Richa Jaiswal
Ranjana Jaiswara
Pawan K. Jaiwal
Anjana Jajoo
Andras Jakab
Attila Jakab
Timo Jakkola
Jennifer Jakobi
Tatjana Jakobs
Ruth Jakobs
Asgeir Jakola
Mihajlo Jakovljevic
Vladimir Jakovljevic
Mike Jakowec
Viljar Jaks
Lars Jakt
Jan Jakubík
Aleksandra Jakubowski
Venkatakrishna Jala
Diana Jalal
Beenu Jalali
Sarika Jalan
Sridhar Jaligama
Estelle Jaligot
Hamid Jalilvand
Anahita Jalilvand
Sirpa Jalkanen
Archana Jalligampala
Vincent Jallu
Ana Rebeca Jaloma Cruz
Tiffany Jamann
Helena Jambor
Melissa Jamerson
W Philip James
S. Jill James
Margaret James
Thomas James
Rathinanadar James
Eddie James
Leighton James
Llana James
William James
Andrew James
Taryn James
Paula James
Gary James
Kelsey James
Garth James
Judy James
Phil James
Lisa James
Laura James
Edward James
Stephen Jameson
Geoffrey Jameson
Julie Jameson
Anne Jamet
Jean-Louis Jamet
Sarra Jamieson
Muhammad Jamil
Sreenivasa Rao Jammalamadaka
Krzysztof Jamroziak
Shazia Jamshed
Neema Jamshidi
Ahmedreza Jamshidi
Athanasios Jamurtas
Gwenaël Jan
Gräfe Jan
Ren-Long Jan
Hamid Jan Jan Mohamed

Naveena Janakiram
Anuradha Janakiraman
Rajiv Janardhanan
Guilhem Janbon
Michel Jancloes
Monika Janczarek
Tibor Janda
Katerina Jandová
S. E. Jandricic
Sarah Jandricic
Martine Jandrot-Perrus
David Jandzik
Esther Jane
Chung-Chen Jane Yao
Stefan Janecek
Michael Janech
Baptiste Janela
Shorena Janelidze
Holly Janes
James Janetka
Hak Chul Jang
Jae Young Jang
Kyu Yun Jang
Hee-Chang Jang
Jae-Won Jang
Hyung-Kwan Jang
Cheol Seong Jang
Min Jang
Kamlesh Jangid
Tim Janicke
Piotr Janicki
Marc Janier
Gabor Janiga
Damir Janigro
Vincent Janik
David Janik
Marc Janin
Michael Janitz
Zorica Janjetovic
Naveed Janjua
Eshetu Janka
Karel Janko
Robert Jankov
William Jankowiak
Marta Jankowska
Marek Jankowski
Maciej Jankowski
Piotr Jankowski-Mihulowicz
Stefano Jann
Martin Jannot
Florence Janody
Claire Janoir
David Janos
Pavel Janoš
Alexis Janosik
Miroslaw Janowski
David Jans
Jan Jansa
Vincent Jansen
Gert Jansen
Jacobus Jansen
Ana Jansen
Teunis Jansen
Peter Jansen
Maurice Jansen
Cassie Jansen
Anja Jansen
Henning Jansen
Steven Jansen
Petrus Jansen Van Vuren
Andreas Janshoff
Nomdo Jansonius
Magdalena Jansová
Ian Janssen
Patricia Janssen
Edith Janssen
Paul Janssen
Marco Janssen
Matthijs Janssen
Emiel Janssen
Saskia Janssen
Frans Janssens
Roland Jansson
Kaemwich Jantama
Richard Jantz
Tiffany Jantz
Fraser Januchowski-Hartley
Christopher Janus
Lukasz Januszkiewicz
Philippe Janvier
Katy Janvier
Tavan Janvilisri
David Janz
Christian Janzen
Bonnie Janzen
Samir Jaoua

Maelle Jaouannet
Uta Jappe
Sebastien Jaquemet
Alfonso Jaramillo
Maritza Jaramillo
Nicolas Jaramillo-O
Benjamín Jarcuška
Ludwig Jardillier
Armando Jardim
Christophe Jardin
Elliott Jardin
Paul Jardine
Alan Jardine
Carlos Jared
Sergei Jargin
Ivan Jaric
David P. Jarmolowicz
Malgorzata Jarmuz-Szymczak
William Jarnagin
Johanna Järnegren
Catherine Jarnevich
Ernst Jarosch
Beata Jarosiewicz
David Jarrard
Mohamed Jarraya
Ken Jarrell
Stuart Jarrett
Ruth Jarrett
Michael Jarstfer
Gail Jarvik
Kirsi Jarvinen
Tero Jarvinen
Erich Jarvis
Joseph Jarvis
Paul Jarvis
Wolfgang Jaschinski
Scott Jasechko
Marie Jasienuk
Michal Jasinski
Bernard Jasmin
Aaron Jasnow
Jacek Jassem
Ricardo Jasso-Chavez
Kris Jatana
Devcharan Jathanna
Santosh Jatrana
Jean Jaubert
Edward Jauch
Mauren Jaudal
Sameer Jauhar
Annikki Jauhiainen
Matti Jauhiainen
Damia Jaume
Kathrine Jauregui-Renaud
Cecile Jauzein
Pasha Javadi
Amir-Homayoun Javadi
Farzam Javadpour
Narjes Javaheri
Marco Javarone
Tariq Javed
Asma Javed
Zeeshan Javed
Delphine Javelaud
Babak Javid
Pouya Javidpour
Dymphna Javier
Etelvina Javierre
Michal Javorka
James Jawitz
Coline Jaworski
Jennifer Jay
Philippe Jay
Gregory Jay
Panneer Selvam Jayabal
Muthuvel Jayachandran
P. Jayamurthy
Jabadurai Jayapaul
Kannamannadiar Jayaprakasan
Ciriyam Jayaprakash
Vasanthi Jayaraman
Arul Jayaraman
N. Jayaraman
Arunachalam Jayaraman
Dhileepkumar Jayaraman
Shiva Jayaraman
Muralidharan Jayashree
Upali Jayasinghe
Nishad Jayasundara
Ranil Jayawardena
David Jaye
Assan Jaye
L. A. Jaykus
David Jayne
Bruce Jayne
Aurelie Jayol

E. A. Jayson
Seyed Mehdi Jazayeri
Agnieszka Jazwa
Hyunsoo Je
Dominique Jean
Melanie Jean
Didier Jean
Pascale Jeannin
Petersen Jeannine
Jed Jebali
Paul Jedlicka
Sun Ha Jee
Rajesh Jeewon
Jo Jefferies
John Jefferies
Roy Jefferis
Jonathan Jeffers
Kevin Jefferson
Glen Jeffery
Ian Jeffery
Louisa Jeffery
Austin Jeffery
Meghan Jeffres
Wade H Jeffrey
Brett Jeffrey
Matlock Jeffries
Ken Jeffries
Michael Jeffries
Penny Jeggo
Timothy Jegla
Elizabeth Jeglic
Jana Jeglinski
Bernard Jegou
Jeremie Jegu
Frederic Jehan
Nico Jehmlich
Megan Jehn
John Jeka
Katrin Jekel
Elissa Jelalian
Sven Jelaska
Aline Jelenkovic
Linda Jelicks
Jaroslav Jelinek
Herbert Jelinek
Wilhelm Jelkmann
Rick Jellen
Scott Jellish
Kristen Jellison
Casey Jelsema
Wojciech Jelski
Jennifer Jelsma
Albert Jeltsch
Philip Jen
Ian Jen
Chun-Ping Jen
Satya Jena
Josef Jenewein
Jiann-Shing Jeng
Yung-Ming Jeng
Jiiang-Huei Jeng
Seng-Feng Jeng
Wen-Juei Jeng
Clinton Jenkins
Cheryl Jenkins
Helen Jenkins
Michael Jenkins
Timothy Jenkins
Frank Jenkins
Adrianna Jenkins
Gregory Jenkins
Herman Jenkins
Darlene Jenkins
William Jenkins
Emma Jenkins
Louis Jenkins
Richard Jenkins
Howard Jenkinson
Ian Jenkinson
Claire Jenkinson
Ronald Jenner
Lukas Jenni
Susanne Jenni-Eiermann
Thomas Jennings
Barbara Jennings
Patrick Jenny
Nancy Jenny
Matt Jenny
Henrik Jensen
Mark Jensen
Paul Jensen
Thomas Jensen
Michael Jensen
Majken Jensen
Taylor Jensen
Gitte Jensen

Pablo Jensen
Randy Jensen
Jørgen Jensen
Kai Jensen
Annette Jensen
Poul Jensen
Greg Jensen
Klavs Jensen
Esther Jensen
Carsten Jensen
Elizabeth Jensen
Kristin Jensen
Jacoib Jensen
Chad Jensen
Brian Jensen
Natasja Jensen
Kathe Jensen
Eric Jensen
Just Jensen
Kevin Jensen
Evelyn Jensen
Erika Jensen-Jarolim
Matthias Jentschke
Synne Jenum
Noo Li Jeon
Byeonghwa Jeon
Jong-Seong Jeon
Daejong Jeon
Christie Jeon
Joo Jeon
Byeong Hwa Jeon
Hong Jin Jeon
J. Jeon
Jaeseung Jeong
Soon-Chun Jeong
Byeongmoon Jeong
Jae-Wook Jeong
Kwang Cheol Jeong
Joon Jeong
Chang Wook Jeong
Soo-Jin Jeong
Seong Jin Jeong
Jae-Hoon Jeong
Suh Young Jeong
Kyu-Shik Jeong
Soung Won Jeong
Seungwon Jeong
Tobias Jeppsson
Anders Jeppsson
Paul Jepson
Christopher Jerde
Travis Jerde
Lars Jerdén
Mcbride Jere
Nadia Jeremiah
Susanna Jernelöv
Robert Jernigan
David Jernigan
George Jeronimidis
Bertus Jeronimus
George Jerums
Lori Jervis
Marc Jeschke
Nathaniel Jeske
Vicky Jespers
Thomas Jespersen
Rolf Jessberger
Cristiano Jesse
Nadia Jessel
Niels Jessen
Sarah Jessen
Ryan Jessup
James Jester
Christopher Jesudason
Pamela Jeter
Anton Jetten
Jolanda Jetten
Espen Jettestuen
Thomas L. Jetton
Marc Jeuland
Yong-Chull Jeun
Eui-Bae Jeung
Ben Jeuris
Yadava Jeve
Vesna Jevtovic-Todorovic
Derek Jewell
Oliver J.D. Jewell
Michael Jewett
Katarina Jewgenow
Rachel Jewkes
Kandiah Jeyaseelan
Anand Jeyasekharan
Joe Jez
Rajesh Kumar Jha
Gopaljee Jha
Ayan Jha

Awadesh Jha
Shshmita Jha
Sumit Jha
Vivek Jha
Uday Jha
Prabhat N. Jha
Rajesh Jha
Guarang Jhala
Amit J. Jhala
Sungchul Ji
Minjun Ji
Hongbin Ji
Yewei Ji
Li-Jun Ji
Yinduo Ji
Zhi-Liang Ji
Hong-Long (James) Ji
Yanhong Ji
Peng Ji
Jinchao Ji
Na Ji
Junfeng Ji
Fei Ji
Jim Ji
Y Ji
Ruijun Ji
Pingsheng Ji
Wei Jia
Weiping Jia
Yulin Jia
Ting Jia
Haibo Jia
Ji-Dong Jia
Sujuan Jia
Rong Jia
Xiaobing Jia
Zhansheng Jia
Xiaoxuan Jia
Xiaopeng Jia
Ze Jia
Lin Jia
Zhenyu Jia
Guang Zheng Jia
Junjing Jia
Xiaofei Jia
Bin Jia
Tao Jia
Mary Jia
Haiyan Jia
Xiuqin Jia
Peizeng Jia
Shaowei Jia
Bingrui Jia
Zhongtian Jia
Peng Jia
Zhenquan Jia
Ming Jian
Hou Jian
Xiaoying Jian
Xueqiu Jian
Zhou Jianbin
Ning Jiang
Jiming Jiang
Shibo Jiang
Taijiao Jiang
Zhihua Jiang
Yun-Jin Jiang
Xuejun Jiang
Lin-Hua Jiang
Shuye Jiang
Xiaoyan Jiang
Zide Jiang
Haobo Jiang
Chun Jiang
Hui Jiang
Yang Jiang
Hu-Lin Jiang
Zi-Feng Jiang
Xingyu Jiang
Peihua Jiang
Songshan Jiang
Mali Jiang
Hong Jiang
Pingping Jiang
Yi Jiang
Jean Jiang
Dong Jiang
Haisong Jiang
Shijin Jiang
Guo-Fang Jiang
Xiaoqian Jiang
Shi-Wen Jiang
Chunlei Jiang
Joy Jiang
Xiaoqing Jiang
De-An Jiang

Mingxi Jiang
Dianhua Jiang
Dianming Jiang
Xiaodong Jiang
Rui Jiang
Jicai Jiang
Hongbing Jiang
Feng Jiang
Baoguo Jiang
Fan Jiang
Qin Jiang
Cuncang Jiang
Houshuo Jiang
Guihua Jiang
Ling Jiang
Libing Jiang
Weijie Jiang
Yuelu Jiang
Lei Jiang
Sizun Jiang
Tingbo Jiang
Yiwei Jiang
Zongliang Jiang
Lingxi Jiang
Chao Jiang
Luo-Luo Jiang
Xuezhi Jiang
Guo-Liang Jiang
Lili Jiang
Yong Jiang
Rong-San Jiang
Zhengxuan Jiang
Jiansen Jiang
Jiang Jiang
Jheng-Jie Jiang
Jianping Jiang
Bo-Le Jiang
Jingfeng Jiang
Xia Jiang
Kun Jiang
Rongrong Jiang
Minlin Jiang
Zhi-Qiang Jiang
Zhiguo Jiang
Yiliang Jiang
Yunyun Jiang
Yuan-Qing Jiang
Wei Jiang
Hanchao Jiang
Weizhe Jiang
Yujun Jiang
Yizhang Jiang
Yajun Jiang
Xi Jiang
Lin Jiang
Baichun Jiang
Xiaoyu Jiang
Tingting Jiang
Gangyi Jiang
Xiao Guang Jiang
Heng Jiang
Li-Yun Jiang
Yulei Jiang
Jinjin Jiang
Jun Jiang
Guangming Jiang
Peiyong Jiang
Lixi Jiang
Ziyan Jiang
Yuanyuan Jiang
Yuzhen Jiang
Shiguo Jiang
Chunsun Jiang
Ming Jiang
Xiaoming Jiang
Shengyang Jiang
Rays Jiang
Zhongyuan Jiang
Liao Jianglin Jianglin
Wang Jianguang
Yu Jianjun
Wei-Wei Jiao
Ping Jiao
Yang Jiao
Yongjun Jiao
Xiaoguo Jiao
Shuliang Jiao
Jingjing Jiao
Yun Jiao
Glen Jickling
Zhijun Jie
Dongmei Jie
Biao Jie
Shi Jie-Hua
Nikolaus Jilg
Feng Jiliang

Bernd Jilma
Belinda Jim
Ryo Jimbo
Masahito Jimbo
Wladimiro Jime ´Nez
Fabio Jimenez
Gerardo Jimenez
Rafael Jimenez
M. Angeles Jimenez
Juan Jimenez
Juan J Jimenez
Alejandro Jimenez
Carlos Jimenez
Natalia Jiménez
Juan Jiménez
Blanca Jiménez Cisneros
María Jiménez De Bagüés
Veronica Jimenez Ortis
Alejandro Jiménez Sosa Jiménez Sosa
Mónica Jimenez-Castro
Rodrigo Jiménez-Garcia
José Jiménez-Heffernan
Sergi Jimenez-Martin
Susana Jiménez-Murcia
Joohi Jimenez-Shahed
Lai Jimmy
Dezhe Jin
Qiming Jin
Guangfu Jin
Byung Rae Jin
Dong Il Jin
Jingbo Jin
Xiaoli Jin
J. P. Jin
Xiaohua Jin
Jing Jin
Yong-Su Jin
Mingjuan Jin
Zixue Jin
Faguang Jin
Zhiqiang Jin
Jianping Jin
Di Jin
Yu Jin
Peiyao Jin
Hongfang Jin
Jun-O Jin
Chengcheng Jin
Meng Jin
Sheng Jin
Jianhua Jin
Zhi-Min Jin
Yuan-Ting Jin
Zi-Bing Jin
Jianxue Jin
Liya Jin
Yan Jin
Xin Jin
Chenwang Jin
Yi Jin
Chunyu Jin
Weibo Jin
Ling Jin
Ran Jin
Bo Jin
Guangze Jin
Cheng Jin
Nange Jin
Wei Jin
Xun Jin
Zuo-Lin Jin
Devin Jindrich
Goodwin Jinesh
Huaiqi Jing
Yongkui Jing
Hai-Chun Jing
Shuqian Jing
Fan Jing
Xiaojuan Jing
Hongmei Jing
Shaojiao Jing
Yan Jing
Jiang Jingkun
Zhuo Jing-Schmidt
Kentaro Jingushi
Clare Jinks
Wang Jinlin
Shi Jinlong
Masafumi Jinno
Umesh Jinwal
Jiri Jiracek
Aigars Jirgensons
Katerina Jirsova
Karin Jirström
Mark Jit
Daisuke Jitoku

Aliya Jiwani
Patrick Jjemba
Hanjoong Jo
Junghyo Jo
Hang-Hyun Jo
Jun-Ichiro Jo
You Hwan Jo
Anja Joachim
Christoph Joachim
Broderick Joan
Olivier Joannes-Boyaux
Michael Joannidis
Dominique Job
Blair Jobe
Malcolm Jobling
Andrew Jobling
Simon Jobson
Richard Jobson
Bertram Jobst
Samia Joca
Christoph Jochum
Ralf Jockers
Meritxell Jodar
Eun-Hye Joe
Mark Joffe
Aaron Joffe
Mateus Joffily
Michel Joffres
Mandar Jog
Ulrich Joger
Annika Jögi
Guri Johal
Lavaud Johann
Susana Johann
Ludger Johannes
Bernd Johannes
Johannes Johannes
Sigrun Alba Johannesdottir Schmidt
Asgeir Johannessen
Aud Johannessen
Kerstin Johannesson
Neil Johannsen
Kerri Johannson
Christoffer Johansen
Mark Johansen
Jacob Johansen
Peter Johansen
Stein Johansen
Flemming Johansen
Anders Johansson
Martin Johansson
Michael Johansson
Pia Johansson
Therese Johansson
Frank Johansson
Petter Johansson
Klara Johansson
Roger Johansson
Jan Johansson
Hans Johansson
Daniel Johansson
Hanna Johansson
Orjan Johansson
Carolina Johansson
Henrik Johansson
Adam Johansson
Ann-Christin Johansson
Karin Johansson Blight
Amit Joharapurkar
Danielle Johinke
Oommen John
Ulrik John
Jacob John
Peter John
Annie John
Dolly John
Leslie K. John
Reimar Johne
Bastian Jöhnk
Roger Johns
Gerrit John-Schuster
Sonke Johnsen
Hans Johnsen
Carl Johnson
Kenneth Johnson
Marc Johnson
Paul Johnson
Eric Johnson
Mark Johnson
James Johnson
Jane Johnson
Edward Johnson
Arlen Johnson
Pieter Johnson
Jeffrey Johnson
Jill Johnson

Timothy Johnson
Nathalie Johnson
Robert Johnson
David Johnson
Leigh Johnson
Loretta Johnson
J. Johnson
Bruce Johnson
Giles Johnson
Pauline Johnson
Christopher Johnson
Shannon Johnson
Devin Johnson
W. Evan Johnson
Reed Johnson
Elizabeth Johnson
Mark A Johnson
Rodney Johnson
Casonya Johnson
Raymond Johnson
Maribeth Johnson
William Johnson
Douglas Johnson
Jeremy Johnson
John Johnson
Brian Johnson
Simon Johnson
Hope Johnson
Lara Johnson
Ian Johnson
Sally Johnson
Susan Johnson
Warren Johnson
Michael Johnson
Chris Johnson
David Samuel Johnson
Patricia Johnson
Matthew Johnson
K. Ian Johnson
Jed Johnson
Marilyn Johnson
Matt Johnson
Xenie Johnson
Blair T. Johnson
Ronald Johnson
Mats Johnson
Steve Johnson
Sarah Johnson
Aaron Johnson
R. J. Johnson
Julene Johnson
W. Johnson
Kim Johnson
Barbara Johnson
Nicholas Johnson
Jodi Johnson-Maynard
Nils Johnsson
Brent Johnston
Andrew Johnston
James Johnston
Carol Johnston
Susan Johnston
Matthew Johnston
Peter Johnston
Richard Johnston
Lucy Johnston
Brian Johnston
Stuart Johnston
Samantha Johnston
Jarmila Johnston
Michael Johnston
Stephen Johnston
Jill Johnstone
Daniel Johnstone
Scott Johnstone
Marianne Johnstone
Manu Johny
Olaf Jöhren
Sören Johst
Mei-Ling Joiner
Ian Joint
Jukka Jokinen
Vilija Jokubaitis
Jaap Joles
Jacob Jolij
Alain Joliot
Jukka Jolkkonen
Jolle Jolles
Kate Jolly
Emmitt Jolly
Jasleen Jolly
Cornelia Jol-van der Zijde
Simon Joly
David Joly
Erik Joly
Pierre-Benoit Joly

Philippe Joly
Jean-Christophe Jonas
Kai Jonas
Peter Jonason
Lena Jonasson
Jon Jonasson
Nira Jonathan
Inge Jonckheere
M. Joner
Alan Jones
Jonathan Jones
Lesley Jones
Kevin Jones
Gareth Jones
Arwyn T. Jones
Michael Jones
Wendell Jones
Nicola Jones
Neil Jones
Joanne Jones
Bryan Jones
Jeffery Jones
Nigel Jones
Richard Jones
Clinton Jones
T. Todd Jones
Peter Jones
David Jones
Jeremy Jones
Luke Jones
Anne Jones
Roger Jones
Brian Jones
Rebecca Jones
Miranda Jones
Douglas Jones
Gregory Jones
Lara Jones
Ryan Jones
Steven Jones
Pete Jones
Des Jones
Helen Jones
Andrew Jones
Adam Jones
Melanie Jones
Byron Jones
Brad Jones
Nicole Jones
Melvyn Jones
Stuart Jones
Sylwia Jones
Daniel Jones
Meaghan Jones
Nick D Jones
Scott Jones
John E Jones
Terry Jones
Linda Jones
Sarah Jones
Sande Jones
Matthew Jones
Anita Jones
A. Jones
Trevor Jones
Eleanor Jones
Deborah Jones
Thomas C. Jones
Michelle Jones
Christopher Jones
Patricia Jones
Desiree Jones
Cerith Jones
Emily Jones
Catherine Jones
Warren Jones
Cassandra Jones
Paul Anthony Jones
Tamekia Jones
Jana Jones
Carol Jones
James Jones
Devin Jones
Glynis Jones
Ben Jones
Katherine Jones
Walton Jones
Jessica Jones-Smith
Jonathan Jong
Ilse Jongerius
Somchai Jongwutiwes
Marcel Jonkman
Ian Jonsen
Bror Jonsson
Elisabeth Jonsson
Lage Jonsson

Birgitta Jonsson
Zophonías Jónsson
Jón Einar Jónsson
Helmut Jonuleit
Rocio Joo
Beuy Joob
Katerina Jood
Catherine Jopling
Chris Jopling
Eilin Jopp
Lucas Joppa
Gerhard Jordaan
Bjarte Jordal
Peter Jordan
Kimberly Jordan
Gregory Jordan
Timothy Jordan
Susan Jordan
Mark Jordan
Brian Jordan
Irmgard Jordan
Jillian Jordan
Rebecca Jordan
Janeen Jordan
David Jordan
Heather Jordan
Chris Jordan
Bryen Jordan
Kelvin Jordan
Ferenc Jordán
Manel Jordana
Anna Jordanous
Sandra Jordao
Alceu Jordão
Lynn Jorde
Per Erik Jorde
Lars Jordheim
Joerg Jores
Schubert Jörg
Stephan Jörg
Vivanco Jorge
Veronique Jorge
Marco Jorge
Christian Jorgensen
Murray Jorgensen
Helle Jorgensen
Kirsten Jorgensen
Matthew Jorgensen
Sebastian Jorgensen
Peter Jørgensen
Even Jørgensen
Louise Jørgensen
Andrew Jorgenson
Vanda Jorgetti
Bernard Joris
Robert Jorissen
Anthony Jorm
Vejo Jormalainen
François Jornayvaz
Patricia Jorquera
Rudolf Jörres
Wiard Jorritsma
Pedro Jose
Salgado-Borges Jose
Ricardo Jose
Sforcin Josè Mauricio
Alf Josefson
Jami Josefson
Emma Josefsson
Patrice Joseph
Leo Joseph
Agnel Joseph
Anna-Maria Joseph
Andreas Joseph
Yeboah Joseph
Jomon Joseph
Donald Joseph
Alex Joseph
Maxwell Joseph
Holly Joseph
Nelly Joseph-Mathurin
Cassandra Josephson
David Josephy
Rajendra Joshi
Pheroze Joshi
Shantaram Joshi
Sadhana Joshi
Jasmin Joshi
Santa Ram Joshi
Hrishi Joshi
Arun Joshi
Yogendra Kumar Joshi
Rohina Joshi
Chaitanya Joshi
Shalaka Joshi
C Joshi

Rohit Joshi
Shubhada Joshi
Satish Joshi
Shashank Joshi
Bharat Joshi
Amit Joshi
Pradeep Joshi
Atul Joshi
Smita Joshi
Manish Joshi
Beena Joshi
Deepika Joshi
Suhasini Joshi
R. Joshi
Archana Joshi Saha
Kaumudi Joshipura
Laurence Josset
Stephanie Jost
Christian Jost
Philipp Jost
Anna Jöud
Paula Jouhten
George Jour
Fred Jourdan
François Jouret
Laure Journet
Stéphane Jouve
Zivko Jovanovic
Borko Jovanovic
Branko Jovcic
Mariona Jové
J. Joven
Ramiro Jover
Jelena Jovic
Sandra Jovic
Thomas Jovin
Ion Jovin
Ljiljana Jowitt
Paul Jowsey
Sheila G Jowsey
Jeffrey Joy
Domino Joyce
Michael A. Joyce
Andrea Joyce
Christopher Joyce
Michael Joyner
William Joyner
Andrew Joynert
Thomas Jozefiak
Damian Józefiak
Alicja Jozkowicz
Mihály Józsi
Mihaly Józsi
Matthieu Jozwiak
Jihang Ju
Bensheng Ju
Weimin Ju
Haiyue Ju
Joon Ching Juan
Laura Juan
Chun-Jung Juan
Javier Juan-Albarracín
Ana Juan-Garcia
Michelle Juarez
Antonio Juárez
Herbert Juch
Zachary Jud
Walter Judd
David Jude
Habu Judeh
Stefan Judex
Andrew Judge
Sarah M. Judge
Kevin Judge
Cavalcante Judney
Richard Judson
Leandro Juen
Freimut Juengling
Klaus Juergens
Verena Juergens
Norbert Juergens
Alexander Jueterbock
Begoña Jugo
Mithila Jugulam
Gabriella Juhasz
Gabor Juhasz
Angela Juhasz
Csaba Juhasz
Steven Juhn
Sirkku Juhola
Martti Juhola
Laura Juignet
Lucienne Juillerat
Pascal Juillerat
Ana Juknat
Justin Julander

Seyfert Julia
Timothy Julian
Stuginski-Barbosa Juliana
Celina Juliano
Claire Julian-Reynier
Margarida Julià-Sapé
Vincent Julien
Steven Julious
Ulrich Julius
Ilkka Julkunen
Petro Julkunen
Elizabeth Juma
Hassan Jumaa
Donald Jump
Ari Jumpponen
Gyungah Jun
Liu Jun
Jae Yeoul Jun
Qiu Jun
Sung Jun
Flora Junca
Howard Junca
Won Hee Jung
Klaus Jung
Younghun Jung
Yong-Sam Jung
Kwang-Woo Jung
Hyungtaek Jung
Kwonil Jung
Friedrich Jung
Woo-Sung Jung
Andreas Jung
Kyu Sik Jung
Jae-Joon Jung
Il Lae Jung
C. Jung
Camille Jung
Chang Hee Jung
Ho Won Jung
Esther Jung
Thomas Jung
Hyun Joon Jung
Yong Woo Jung
Tae Sung Jung
Byung Hwa Jung
Ki-Hong Jung
Kyuwhan Jung
Sean Jungbluth
Claudia Junge
Harald Junge
Wolfgang G. Junger
Bill Jungers
Jacob Jungers
Alexandra Jungert
Karl-Heinz Jungfer
Doerte Junghaenel
Ralf Jungmann
Berit Jungnickel
Marie-Pierre Junier
Kim Juniper
Kerstin Junker
Robert Junker
Anders Junker
Juliana Junqueira
Inacio Junqueira De Azevedo
Ana Paula Junqueira-Kipnis
Suh-Hang Juo
Markus Juonala
Amalia Jurado
Maria Jurado
Alicia Jurado Acosta
Sandra Jurado Sanchez
Juan Luis Jurat-Fuentes
Georgiana Juravle
Barbara Jurczyk
Raja Jurdak
Jon Jureidini
Joanna Jurewicz
David Jurgens
Ivan Juric
Melissa Jurica
Abdo Jurjus
Kerstin Jurk
Edouard Jurkevitch
Dominika Jurkovic
Mario Juruena
Jocelyne Just
Ramon Juste
Amy Justice
Sheryl Justice
Zuzana Justinova
Jessica Justman
Ignacio Jusue Torres
Marko Jusup
Brandon Jutras
Eric Jüttler

Artti Juutinen
Marta Juvany
Seppo Juvela
Jaana Juvonen
Haoumi Jyonouchi
