# Supplementary material for: PLOS ONE 2015 Reviewer Thank You
Source: PLoS One. 2016 Feb 23;11(2):e0150341. doi: 10.1371/journal.pone.0150341 (PMC4764340; doi:10.1371/journal.pone.0150341)
Supplement: S3 Reviewer List — (PDF) [file pone.0150341.s003.pdf]

PLOS | ONE

PLOS ONE would like to thank all those who reviewed on behalf of the journal in 2015:

Hakhyun Ka
Shuk-Man Ka
Nadeem Kaakoush
Joel Kaar
Tommi Kääriäinen
Kai Kaarniranta
Achim Kaasch
Ron Kaback
Mehdi Kabani
Christoph Kabbasch
Basirudeen Syed Ahamed Kabeer
David Kabelik
Dieter Kabelitz
Omer Kabil
Ali Kabir
Parijat Kabiraj
Markus Kächele
George Kachergis
Scott Kachlany
S. Patrick Kachur
Puneet Kacker
Alina Kacperska
Joanna Kacprzyk
Ina Maria Kacso
Bogumil Kaczkowski
Agnieszka Kaczor
Sarah Kada
Madhavi Kadakia
Shilpa Kadam
Rameshwar Kadam
Sandeep Kadam
John Kaddis
Reto Kaderli
Aras Kadioglu
Masood Kadir
Jan Kadlec
Karl Kadler
Mahita Kadmiel
Martina Kadmon
Cigall Kadoch
Kenji Kadomatsu
James Kadonaga
Narendra Kadoo
David Kadosh
Kyuichi Kadota
Norimitsu Kadowaki
Slawomir Kadrow
Jaspal Kaeda
Bertrand Kaeffer
Peter Kaempfer
Annemarie Kaesbohrer
Tobias Kaeser
Michael Kaess
David Kaetzel
O. Kaewboonchoo
Jaranit Kaewkungwal
Joseph Kagaayi
George Kagadis
Maiko Kagami
Shin-Ichiro Kagami
Jonathan Kagan
Ron Kagan
Daniel Kaganovich
Gibson Kagaruki
Larry Kagemann
Minna Kahala
Bashar Kahaleh
Joel Kahane
Yaacov Kahanov
Maria Kahar-Bador
Mika Kähkönen
Barbara Kahl
Hans-Peter Kahle
Waltraud Kahle
Joanne Kahlenberg
David Kahler
Timo Kahles
Sonja Kahlmeier
James Kahn
Henry Kahn
Jeremy Kahn
Daniel Kahn
Andrew Kahn
Shulamit Kahn

Thorsten Kahnt
Abdullah Kahraman
Peter Kahrilas
Alem Kahsai
Hirofumi Kai
Michiaki Kai
Minako Kaido
Toshimi Kaido
Shinjiro Kaieda
Darnell Kaigler
Evangelos Kaimakamis
Junya Kaimori
Pinky Kain
B. Kaina
Angela Kaindl
Jana Kainerstorfer
Heikki Kainulainen
Brad Kairdolf
Rolf Kaiser
Ursula Kaiser
Markus Kaiser
William Kaiser
Jan Christian Kaiser
Martin Kaiser
Christina Kaiser
Frank Kaiser
Daniel Kaiser
Elsi Kaiser
Armin Kaiser
Kathryn Kaiser
Stefan Kaiser
Pekka Kaitaniemi
Tu'Uhevaha Kaitu'U-Lino
Karl Kaiyala
Eloise Kaizar
Taisei Kaizoji
Murali Kaja
Simon Kaja
Jessica Kajfasz
Hideko Kaji
Hiroshi Kaji
Yuya Kajikawa
Stephen Kajiura
Hiroshi Kajiya
Adriana Kajon
Lukasz Kajtoch
Henry Kajumbula
Othman Kakaire
Gopal Kakani
Sham Kakar
Yoshihiro Kakeji
Kazuhiro Kakimi
Yu Kakimoto
Yoshihiko Kakinuma
Yutaka Kakizoe
Samata Kakkad
Poonam Kakkar
Ashok Kakkar
Fatima Kakkar
Mayumi Kako
Masao Kakoki
Foteini Kakulas
Hiroki Kakuta
Chandra Prakash Kala
Petr Kala
Roshni Kalachand
Tamas Kalai
Panagiotis Kalaitzis
Freddie Kalaitzis
Predrag Kalajdzic
Hazem Kalaji
Hanna Kalamarz-Kubiak
Spyros Kalams
Ammie Kalan
Kamyar Kalantar-Zadeh
Eyal Kalanthroff
Kriton Kalantidis
Venkat Kalapatapu
John Kalapurakal
Nandakumar Kalarikkal
Wojciech Kalas
Suresh Kalathil
Ravi Kalathur
Siripen Kalayanarooj
Sita Kalayanarooj
Miriam Kalbitz
Andreas Kalckert
Matina Kalcounis-Rueppell
Lee Kalcsits
Jonas Kalderstam
Philipp Kaldis
Jeffrey Kalenak
Ruslan Kalendar
Charlene Kalenkoski
Tobias Kalenscher
Bindu Kalesan

Theodosia Kalfa
Sameer Kalghatgi
Satish Kalhan
Amy Kalia
Suneil Kalia
Sam Kalibala
Seth Kalichman
Kyriaki Kalimeri
Antigoni Kaliontzopoulou
Katarzyna Kalita
Stiliyan Kalitzin
Efstathios Kaliviotis
Emma Kalk
Sinan Kalkan
Markus Kalkum
Lukas Käll
Asgar Kalla
Bhaskar Kallakury
Meri Kallasvuo
Riddhika Kalle
Roland Kallen
Bengt Kallen
Karl Josef Kallen
Neville Kallenbach
Klaus Kallenbach
A. S. Kallimanis
Tuomo Kalliokoski
Dorina Kallogjeri
Eric R. Kallwitz
Eleftheria Kalogera
Theodore Kalogeris
Lalit Kalra
Bhawna Kalra
Alon Kalron
Andries Kalsbeek
Sukhvinder Kalsi-Ryan
Bernhard Kaltenboeck
Anna Kaltenboeck
Holger Kalthoff
Gregory Kaltsas
Antonia Kaltsatou
Joanna Kaluza
Ananth Kalyanaraman
Ramki Kalyanaraman
Shanker Kalyana-Sundaram
Lisa Kalynchuck
Tae-In Kam
Moshe Kam
Jason Kam
Yusuke Kamachi
Yoshihiro Kamada
Masaki Kamakura
A. Kamal
Shahmir Kamalian
Nazila Kamaly
Tunku Kamarul
Dmitri Kamashev
Hideaki Kamata
Takaaki Kamatani
Ganesh Kamath
Binita Kamath
Ambika Kamath
Mary Kamb
Sekhar Kambakam
Naotomo Kambe
Joseph Kambeitz
Robert Kambic
Pramod Kamble
Mini Kamboj
Colince Kamdem
Hideto Kameda
Yasutomi Kamei
Asuka Kamei
Rina Kamenetsky
Helen Kamens
Julia Kamenz
Hidehiro Kamezaki
Alan Kamhi
Julian Kamhieh-Milz
Masahiro Kami
Kei Kamide
Masamichi Kamihira
Yoshi-Ichiro Kamijo
Kenya Kamimura
Shinji Kamimura
Hironori Kaminaka
Nina Kaminen-Ahola
Alexandra Kamins
Dorota Kaminska
Karol Kaminski
David Kaminsky
Kubra Kamisoglu
Akihide Kamiya
Shigeru Kamiya
Yoshimi Kamiyama
Mehran Kamkar

Monika Kamkuemah
Suchitra Kamle
Christian Kamm
Thomas Kammer
Peter Manuel Kammer
Melanie Kammerer
Masahiro Kamouchi
Timothy Kamp
Marcel Kamp
Siri-Maria Kamp
Jörg Kämper
Thomas Kampf
Gunter Kampf
Lars Kamphuis
Sandra Kamping
Dora Kampis
Ulla Kampmann
Sotirios Kampranis
Biao Kan
Xianzhao Kan
Bahadir Kan
Chi-Chuan Kan
Anita Kan
Bavesh Kana
Rajesh Kana
Mona Kanaan
Yasamin Kanaan
Rajapandian Kanagaraj
Nobuhisa Kanahara
Ryota Kanai
M. Kanai
Nobuo Kanai
Surapathrudu Kanakala
Ioannis Kanakis
Arun Kanakkanthara
Venkateswarlu Kanamarlapudi
Satoru Kanamori
Keizo Kanasaki
Kumiko Kanatani
S. Kanatous
Rushed Kanawati
Hiroshi Kanayama
Shigeyuki Kanazawa
Tetsufumi Kanazawa
Masato Kanazawa
Akira Kanazawa
Motoyori Kanazawa
Takeharu Kanazawa
Vijaya Kancherla
Waldemar Kanczkowski
Takashi Kanda
Yoshinobu Kanda
Tatsuo Kanda
Hiroko Kanda
Naoko Kanda
Prabodh Kandala
Ngianga Ii Kandala
Susan Kandarian
Rama Kandasamy
Matheswaran Kandasamy
Abraham Kandathil
Eugene Kandel
Rita Kandel
Leonid Kandel
Ben Kandel
Ramesh Kandimalla
Anne Kandler
Pramod Kandoth
Mustapha Kandouz
Konstantin Kandror
Namratha R. Kandula
Patricia Kane
Lawrence Kane
Maureen Kane
Daniel Kane
Adam Kane
Sarah Kane
Erin Kane
Atsushi Kaneda
Norio Kaneda
Kazue Kanehara
Masaru Kanekiyo
Yoshikatsu Kaneko
Akira Kaneko
Osamu Kaneko
Takehito Kaneko
Satoshi Kaneko
Yuji Kaneko
Yuko Kaneko
Shinya Kaneko
Angelos Kanellis
Jean Kanellopoulos
Asako Kaneoka
Kengo Kanetaka
Hannah Kanety
Un-Jung Kang

Gyeong Hoon Kang
Sang-Moo Kang
Min Kang
Chulhee Kang
Hyun Ah Kang
Jaewoo Kang
Zhensheng Kang
Chunsheng Kang
Shin-Wook Kang
Myungshim Kang
Wei Kang
A. Kang
Xiang-Yang Kang
Yun Kang
Youmin Kang
Sung Ung Kang
Hunseung Kang
Byoung-Cheorl Kang
James Kang
Congbao Kang
Muyi Kang
Hakmook Kang
Ho Chul Kang
Seok Hui Kang
Peter Kang
Jae Kang
Keon Wook Kang
Shuli Kang
Shehee H Kang
Kyung-Il Kang
Min-Jong Kang
Hou-Yong Kang
Young Jun Kang
Jeonghyun Kang
Jung Kang
Zhouqing Kang
Hojeong Kang
Jin-Su Kang
Kevin Kang
Sung Kang
Se Woong Kang
Se Chan Kang
Chi-Dug Kang
Jian Kang
Minglei Kang
Meng-Zhen Kang
Daeseok Kang
Kristopher Kang
Hara Kang
Bokyu Kang
Jong-Sun Kang
Shaozhong Kang
Chounghun Kanga
Lauri Kangas
Mari Kangasniemi
Madhuri Kango-Singh
Beth Kangwana
Artur Kania
Urša Kanjir
Jiri Kanka
Hannu Kankaanranta
Maaria Kankare
Hyacinthe Tchewonpi Kankeu
Phyllis Kanki
Yasuharu Kanki
Georgette Kanmogne
Oliver Kann
Mathur Kannan
Sivakumar Kannan
Natarajan Kannan
Srinivasaraghavan Kannan
Stephen Kanne
Caroline Kannengiesser
Kasturi Kanniah
Sridhar Kannurpatti
Fumihiro Kano
Flora Kano
Yutaka Kano
Deepak Kanoja
Deepak Kanojia
Scott Kanoski
Michael Kanost
Kazuyuki Kanosue
Aswin Kansakar
Outi Kanste
Melda Kantar
Alpdogan Kantarci
Steve Kanters
Tim Kanters
Phillip Kantharidis
Anumantha Kanthasamy
Sreetharan Kanthaswamy
Jimut Kanti Ghosh
Marc Kantorow
Jyotshna Kanungo
Suman Kanungo

Yashpal Kanwar
Jagat Kanwar
Karl-Georg Kanz
Hung-Ying Kao
Rowland Kao
Wei-Chun Kao
Jia-Horng Kao
Winston Kao
Cheng-Fu Kao
Chienhui Kao
John Kao
Katy Kao
Ching Huei Kao
Yu-Hsun Kao
Wei-Yu Kao
Hung-Wen Kao
Chien-Min Kao
Tze-Wah Kao
Mubbasir Kapadia
Dharmi Kapadia
Pankaj Kapahi
Ali Kapan
Vsevolod Kapatsinski
Aliki Kapazoglou
Flavio Kapczinski
Amy Kapczynski
Maria Kapetanaki
Yvonne Kapila
Vikram Kapila
Allen Kaplan
David Kaplan
Ian Kaplan
Warren Kaplan
Gilaad Kaplan
Noam Kaplan
Jeremy Kaplan
Daniel Kaplan
Gary Kaplan
Jennifer Kaplan
Lee Kaplan
Katherine Kaplan
Allan Kaplan
Ozgur Kaplan
Atya Kapley
Pashalia Kapli
Nicholas Kaplinsky
Stan Kaplowitz
Dimitrios Kapogiannis
Rohit Kapoor
Rupam Kapoor
Kapil Kapoor
Avnish Kapoor
Anil Kapoor
Meenu Kapoor
Valerie Kapos
Andras Kaposi
Zoi Kapoula
Steven Kapp
Ilias Kappas
Stefan Kappe
Jasper Kappen
Ulrike Kappler
Maxim Kapralov
Philipp Kapranov
Efstathia Kapsogeorgou
Stephen Kaptoge
Jagadis Kapuganti
Andras Kapus
Nestor Kapusta
Alexander Kapustin
Premashis Kar
Sitanshu Sekhar Kar
Firat Kara
Ezgi Karaca
Bilge Karacali
Niki Karachaliou
Paraskeui Karachle
Spyros Karadimas
Abdullah Karaer
Levente Karaffa
Jim Karagiannis
Panagiotis Karagiannis
Apostolos Karagiannis
Nikoletta Karaiskou
Huseyin Caglar Karakaya
Ioannis Karakikes
Petros Karakousis
Giorgos Karakousis
Gökhan Karakülah
Sana Karam
Lisa Karam
Pascale Karam
Dimitrios Karamichos
Timokratis Karamitros
Athanasios Karampeazis
Mike Karampelas

Ratna Karan
Narasimha Kumar Karanam
Pankaj Karande
Nitin Karandikar
John Karanicolas
Andrew Karaplis
Alicia Karas
Alexander Karasev
David Karasik
Lana Karasik
Hilary Karasz
Ece Karatan
Ilia Karatsoreos
Georgios Karavas
Frini Karayanidis
Amir Karban
Jennifer Karberg
Musturay Karcaaltincaba
François Karch
Celeste Karch
Konrad Karcz
József Kardos
Gabor Kardos
Zsofia Kardos
Joseph Kardouni
Kevin Kardynal
Isabella Kardys
Hassen Kared
Patrik Karell
John Karemaker
Stephen Karesh
Georgy Karev
Fatemeh Kargarfard
Barry Karger
Andrei Karginov
Joanna Kargul
Zahida Karim
Zubair Karim
Zahra Karimaghaloo
Roxanne Karimi
Mahdi Karimi
Soheila Karimi-Abdolrezaee
Azar Kariminia
Hadi Karimzadeh
Kenji Karino
Piia Karisola
Symon Kariuki
Samuel Kariuki
Stephen Karl
Mike Karl
Thomas Karlas
Joel Karliner
Kinga Karlinger
Gennady Karlov
Petr Karlovsky
Elinor Karlsson
Erik Karlsson
Fredrik Karlsson
Ida Karlsson
Laila Karlsson
Jenny Karlsson
Brynjar Karlsson
Niclas Karlsson
Rakesh Karmacharya
Dibesh Karmacharya
Faisal Karmali
Wilfried Karmaus
Artashes Karmenyan
Konstantinos Karmiris
Christof Karmonik
Dimitrios Karmpaliotis
Dimitris Karnabatidis
Clara Karnebeek
Jason Karnes
Sasha Karnes
Daniel Karney
George Karniadakis
Eddy Karnieli
Kamil Karolczak
Nitsara Karoonuthaisiri
Evgenia Karousou
Peter Karp
Sarah Karpanty
Marcel Karperien
Darja Karpova
Jolanta Karpowicz
Manjula Karpurapu
Victor Karpyak
Nancy Karraker
Istvan Karsai
Marton Karsai
Aly Karsan
Sven Karstens
Barbara Karten
Peter Karth
Melanie Karthaus

Narayanan Karthikeyan
Joscha Kärtner
Tyler Kartzinel
Jordan Karubian
S. Karumanchi
Devarajan Karunagaran
Harin Karunajeewa
Chithra Karunakaran
Sheela Karunanithi
Indrani Karunasagar
Tilakavati Karupaiah
Senthilkumar Karuppagounder
S. Mohan Karuppayil
Carrie Karvonen-Gutierrez
Maciek Karwowski
Lamprini Karygianni
David Kasahara
Kota Kasahara
Mureo Kasahara
Emiko Kasahara
Fumio Kasai
Katayoon Kasaian
Parastu Kasaie
Eshvendar Kasala
Atsushi Kasamatsu
Zuzana Kasanova
Murad Kasap
Muzeyen Aytul Kasapoglu
Siddha Kasar
Sandor Kasas
Julie Kase
Daisuke Kase
Benjamin Kasenda
Arthur Kaser
Fabian Käsermann
Thomas Kash
Fatah Kashanchi
Jubin Kashef
Yechezkel Kashi
Naoki Kashihara
Saori Kashima
Yoshihisa Kashima
Ali Kashkouli
Michael Kashon
Rajpal Kashyap
Sabine Kasimir-Bauer
Manish Kasliwal
Enkelejda Kasneci
Michael Kaspari
Susan Kasper
Siegfried Kasper
Fred Kasper
Slawomir Kasperczyk
Araya Kassa
Theodoros Kassimatis
Georgios Kassimis
Zamaneh Kassiri
Judith Kassis
Alf Kastbom
Mary Jo Kasten
Jude Kastens
Panagiotis Kasteridis
Irida Kastrati
Christian Kastrup
Partha Kasturi
Sudhir Kasturi
Takao Kasuga
Akiyoshi Kasuga
Francis Xavier Kasujja
Takhar Kasumov
József Kaszaki
Beata Kasztelan-Szczerbinska
Masatoshi Katabuchi
Fumiaki Katagiri
Daisuke Katagiri
Jun Katahira
Vesa Kataja
Prasad Katakam
Naoto Katakami
Yoshinori Katakura
Vladimir Katanaev
Tatsuki Kataoka
Hiroharu Kataoka
Rajesh Katare
Glowacka Katarzyna
Naoki Katas
Takuya Katayama
Benjamin Katchman
Koutra Katerina
Pantelis Katharios
Sophia Kathariou
Anup Katheria
Nicholas Kathman
Katie Kathrein
Vassiliki Kati
Vittal Katikireddi

Athanasios Katis
Santosh Katiyar
Diksha Katiyar
Bhuvana Katkere
Kikuya Kato
Akiko Kato
Massuo Kato
Junichi Kato
Itaru Kato
Takahiro Kato
Hiroki Kato
Takuma Kato
Takashi Kato
Johji Kato
Masahiko Kato
Akihiko Kato
Shinichi Kato
Norihiro Kato
Masaharu Kato
Takafumi Kato
Koji Kato
Kenji Kato
Koichi Kato
Takehide Kato
Roberta Kato
Souchiro Kato
Kazuhisa Kato
Motohiro Kato
Hiroshi Kato
Rina Kato
Yasumasa Kato
Takamitsu Kato
Hironori Katoh
Munenori Katoh
Elizaveta Katorcha
Yuki Katou
Stylianos Katsanevakis
Konstantinos Katsanos
Paraskevi Katsaounou
Dimitrios Katsaros
Ulrich Katscher
Pavel Katsel
Ahmed Katsha
Panagiotis Katsonis
Klea Katsouyanni
Maki Katsuhara
Yasuhiro Katsumata
Yukako Katsura
Tomohiro Katsuya
Miyake Katsuya
Masata Katsuyama
Antonis Kattamis
Madhusudan Katti
Kalpana Katti
Harish Katti
Paul Katz
Mark Katz
Richard Katz
Donald Katz
Itamar Katz
Arieh Katz
Steven Katz
Curren Katz
Ben-Zion Katz
Albert Katz
Daniel Katz
Leila Katz
Hans Katzberg
Michael Katze
David Katzenstein
Frank Katzer
Helmut Katzgraber
Mandy Katz-Jaffe
Wendy Katzman
Peter Katzmarzyk
Steffen Katzner
Todd Katzner
Aris Katzourakis
Marcia Kauer-Sant'Anna
Norbert F. Käufer
Mathias Kauff
Matt Kauffman
Boone Kauffman
Jim Kaufman
Laura Kaufman
Jay Kaufman
Jordy Kaufman
Jean-Marc Kaufman
David Kaufman
Howard Kaufman
Erica Kaufman West
Thomas Kaufmann
Michael Kaufmann
Ines Kaufmann
Tobias Kaufmann
Pakieli Kaufusi

Rupert Kaul
Sunil Kaul
Marcus Kaul
Sanjana Kaul
Abhishek Kaul
Roland Kaunas
Klemens Kaupmann
Anu Kauppinen
Jasleen Kaur
Gunveen Kaur
Jasbir Kaur
Indu Pal Kaur
Sukhbir Kaur
Harleen Kaur
Kulbir Kaur
Kirandeep Kaur
Rupinderjeet Kaur
Prameet Kaur
Sukhwinder Kaur
Antti Kause
Edgar E. Kausel
Deepak Kaushal
Aradhana Kaushal
Charu Kaushic
Sadasivam Kaushik
Azad Kaushik
Gaurav Kaushik
Nagendra Kaushik
Neerja Kaushik-Basu
Max Daniel Kauther
Stefanie Kautz
John Kauwe
Nat Kav
Martin Kavaliers
Nickolas Kavallieratos
Kevin Kavanagh
John Kavanagh
Phillip Kavanagh
Musa Kavas
Andreas Kavazis
Irina Kaverina
Robert Kavet
Bodil Kavli
Parviz Kavoussi
Panos Kavvadas
Dinkar Kaw
Shigeyuki Kawa
Hiroyoshi Kawaai
Shun-Ichiro Kawabata
Soichiro Kawabe
Kenji Kawada
Takumi Kawaguchi
Shoichiro Kawaguchi
Mitsuyo Kawaguchiya
Jun Kawahara
Genri Kawahara
Tetsuya Kawahara
Yoshihiro Kawahara
Atsuo Kawahara
Ryouka Kawahara-Miki
Yoshichika Kawai
Fusako Kawai
Kenichiro Kawai
Keigo Kawaji
Yasuhiko Kawakami
Koji Kawakami
Hiroshi Kawakami
Tetsuya Kawakita
Kazumichi Kawakubo
Agata Kawalec
Ryuichi Kawamoto
Elisa Kawamoto
Shin-Ya Kawamoto
Tatsuro Kawamoto
Kensaku Kawamoto
Makoto Kawamukai
Shoji Kawamura
Hideki Kawamura
Ryo Kawasaki
R. Kawasaki
Tomoyuki Kawase
Masahiro Kawashima
Akihiro Kawashima
Koichiro Kawashima
Yukichika Kawata
Yohei Kawatani
Suguru Kawato
Takeshi Kawauchi
Lawrence Kawchuk
Thomas Kawula
Jolanta Kawulok
Steven Kawut
A. Barry Kay
Katherine Kay
Alan Kay
Jeremy Kay

Margaret Kay
Mehmet Kaya
H. Kaya
Umur Kayabasi
Ehsan Kayal
Mohsen Kayal
Ghazi Kayali
Guven Kayaoglu
Steve Kaye
Kenneth Kaye
Kassoum Kayentao
Nkomba Kayeyi
Felix Kayigamba
James Kayima
Tuba Kayman
Govender Kaymarlin
Ata Kaynar
Monica Kayo
Gbenga Kayode
Stanley Kays
George Kaysen
Manfred Kayser
Bengt Kayser
Karen Kayser
Hokto Kazama
Kemal Kazan
Ilias Kazanis
Mirdad Kazanji
Powel Kazanjian
Konstantin Kazankov
Anastasiya Kazantseva
Alan Kazdin
Majid Kazemian
Brenda Kazemier
Yukinori Kazeto
Mirajul Kazi
Julhash Kazi
Dhruv Kazi
Layla Kazkaz
Romas Kazlauskas
Victoria Kazmerski
Amir Kazory
Toshinobu Kazui
Santosh Kc
Yuehai Ke
Xiaoyan Ke
Yang Ke
Zun-Ji Ke
Bibo Ke
Yi-Ni Ke
Ruian Ke
Wenshan Ke
Sarah Keadle
Crystal Kean
Brian Keane
David Keane
Andrea Keane-Myers
Lim Tow Keang
Jennifer Kearney
Josephine Kearney
Lisa Kearney
Thomas Kearney
Michael Kearney
Hugh Kearney
Daniel Kearns
Robert Kearny
Stephen Kearsey
Rachael Keating
Aline Keating
Sheila Keating
Mark Keating
Karen Keating
Marie Keatley
Jacob Keaton
Susan Keay
Lisa Keay
David Keays
Melkam Kebede
Hedi Kebli
Marcel Bawindsom Kébré
Raouf Kechrid
James Keck
Tobias Keck
Bastian Keck
Tara Keck
Krisztina Kecskes-Kovacs
Katherine Kedzierska
Mariusz Kedzierski
Barbara Kee
Spencer Kee
Richard Keefe
Carol Keefer
Brian Keegan
Mark Keegan
Brandon Keehn
Jeffrey Keelan

Brandon Keele
Jon Keeley
Annika Keeley
Simon Keely
Carl Keen
Steve Keen
Phillip Keen
Thomas Keen
Kevin Keenan
Hillary Keenan
Jacqueline Keenan
Douglas Keene
Richard Keers
Daniel Keeser
John Keesing
Vladimir Kefalov
Haresh Keharia
Alexander Kehl
Shannon Kehle-Forbes
Elizabeth Kehoe
Corinna Kehrenberg
Michael Keidar
Joyce Keifer
Jaap Keijer
Guenther Keil
Julian Keil
Daniel Keil
Ludy Keino
Brett Keiper
Susanne Keipert
Greg Keir
Carl Nicolas Keiser
Jeff Keisler
Scott Keith
Philippe Keith
Matthew Keith
Elizabeth Keithley
Mp Keizer
Kiriaki Kekou
Uddhav Kelavkar
Almut Kelber
Christopher Kelble
Stijn Kelchtermans
Roya Kelishadi
Laurie Kell
Alison Kell
Nicholas Kellar
Sebastian Kelle
Kelly Kelleher
Ian Kelleher
Shannon Kelleher
Stephan Kellenberger
Evan Keller
Andreas Keller
Peter Keller
Ulrich Keller
Simon Keller
Alexander Keller
Kate Keller
Kathleen Keller
Matthieu Keller
Carmen Keller
Michael Keller
Thilo Kellermann
Maureen Keller-Wood
John Kellett
Thomas Kelley
Scott Kelley
Joanna Kelley
George Kelley
Patricia Kelley
Mark Kelley
Patrick Kelley
M. Lisa Kellogg
John Kellum
Kathleen Kelly
Dave Kelly
Heath Kelly
Charles Kelly
Ben Kelly
Robert Kelly
Ann Kelly
Diane Kelly
Brendan Kelly
William Kelly
Steven Kelly
Daniel Kelly
Sharon Kelly
Matthew Kelly
Angela Kelly
Gabrielle Kelly
Kevin Kelly
Simon Kelly
Emer Kelly
Fiona Kelly
Peter Kelly

James D. Kelly
Lesly Kelly
Malte Kelm
Sørge Kelm
Steven Kelsen
Karl Kelsey
Thomas Kelsey
Joel Kelso
David Kelvin
Jackelyn Kembro
Bertha Maria Lidwina Kemenade
Martin Kemler
David Kemlink
Markus Kemmelmeier
Andre Kemmling
Tom Kemp
Stephan Kemp
Helen Kemp
Paul Kemp
Kevin Kemp
David Kemp
James Kemp
Vivien Kemp
Graham Kemp
Anne Kempel
Björn Kemper
Hervé Kempf
Frank Kempken
Petri Kemppainen
Eben Kenah
Ellen Kenchington
Bruce Kendall
William Kendall
Ion Kendall
Katherine Kendall
Denise Kendrick
Tetyana Kendzerska
Tal Kenet
Dror Kenett
Andre Kengne
Marc Kenis
Tony Kenna
David Kennaway
Chris Kennedy
John Kennedy
Timothy Kennedy
Patrick Kennedy
David Kennedy
Nicholas Kennedy
Breandán Kennedy
Mark Kennedy
Lindsey Kennedy
Byron Kennedy
Kelsey Kennedy
Brian Kennedy
Stephen Kennedy
Jessica A. Kennedy
Eileen Kennedy
Linda Kenney
Anna Kenney
Scott Kenney
Cassandra Kenning
Glen Kenny
Lawrence Kenny
Paraic Kenny
Dermot Kenny
Stephen Kent
Jack Kent
Michael Kent
David Kent
Shia Kent
Seamus Kent
Brian Kent
Christopher Kent
Erin Kent
Jonathan Kentish
Joseph Kenworthy
Tsuneaki Kenzaka
Louise Keogh
Rebecca Keogh
Justin Keogh
Mandy Keogh
Kasper Kepp
Malcolm Kepping
Daniel Keppler
Eldad Kepten
Bernard Keraita
Mehdi Keramati
Angelo Keramidas
Tapeni Keränen
Antonios Kerasnoudis
Jeffrey Kerby
Garry Kerch
Yves Kerdraon
Nora Kerekes
Gyorgy Kerekes

Nir Keren
Attila Kereszt
Frédéric Kerff
Steven Kerfoot
Hugo Kerhervé
Scott Kerick
Savita Kerkar
Andrew Kerkhoff
Georg Kerkhoff
Annette Kerkhoff
Ioannis Kerkines
Jens Kerl
Robert Kerlan
Graham Kerley
Anna-Elodie Kerlo
Ali Kermanizadeh
Stephanie Kermorgant
Elsa Kermorvant-Duchemin
Winfried Kern
Michael Kern
Timothy S Kern
Matthias Kern
Peggy Kern
Colin Kern
Katharina Kerner
Ryan Kerney
Steven Kernie
Kemp Kernstine
J. Kero
Tom Kerppola
Ian Kerr
Bethany Kerr
Candace Kerr
Andrew Kerr
Benjamin Kerr
Peter Kerr
Steven Kerrigan
Talitha Kerrigan
Maiwenn Kersaudy-Kerhoas
Katharina Kerschan-Schindl
Hubert Kerschbaum
Mônica Kersch-Becker
Gilbert Kersh
Jeff Kershaw
Arik Kershenbaum
Christian Kerskens
Janko Kersnik
Sander Kersten
Phil Kersten
Christian Kersten
Attila Kertesz-Farkas
Marc Kery
Jerome Kerzerho
Tm Kesar
Santosh Kesari
Aparna Kesarwala
Bilgin Keserci
Behrang Keshavarz
Ali Keshavarzian
Shashank Keshavmurthy
Mahtab Keshvari
Dylan Kesler
Line Kessel
Jurg Kesselring
Ronald Kessler
Danny Kessler
André Kessler
Benedikt Kessler
Yoav Kessler
Thorsten Kessler
Ian Kessler
Barbara Kessler
Sonja Kessler
Jean-Pierre Kessler
Katharina Kessler
Viktor Kessler
Jason Kessler
Alison Kesson
Thomas Kesteman
Luc Kestens
Yan Kestens
Karen Kester
M. Kesting
Daniel Keszthelyi
James Ketchum
Daniel Ketelhuth
Hamit Keten
C. Ketonis
Markus Ketteler
Rene Ketting
Anthony Kettle
Johannes Kettunen
James Ketudat Cairns
Oliver Keuling
Olivier Keunen
Zela Keuylian

Peter Kevan
Christopher Kevil
Silverstein Kevin
Olen Kew
Felix Key
Sam Keyes
Nemat Keyhani
Chad Keyser
Joann Keyton
Dineo Khabele
Anuradha Khadilkar
Kapil Khadka
Anmar Khadra
Alka Khaitan
Sharukh Khajotia
Bekzod Khakimov
Annette Khaled
Mohammed Khalfallah
Syma Khalid
Asma Khalid
Mohamed Khalifa
Hind Khalifeh
Kadry Khalik
Asma Khalil
Michael Khalil
Andre Khalil
Naila Khalil
Hussein Khalil
Farah Khalil
Kamel Khalili
Hamed Khalili
Davood Khalili
Mohammad Khalilzadeh
Oleh Khalimonchuk
Saba Khaliq
Bouziane Khalloufi
Bavornlak Khamnamtong
Naveed Khan
Imtiaz Khan
Asif Khan
Mahmood Khan
Waliul Khan
Rizwan Khan
Imran Khan
Razib Khan
Muhammad Khan
Saleem Khan
M. Firoze Khan
Matiullah Khan
Sadaf Khan
Zeina Khan
Gausal Khan
Waqar Khan
Nooruddin Khan
Aarlenne Khan
Feroz Khan
Ilyas Khan
Muhammad Altaf Khan
Nasim Ahmed Khan
Awais Khan
Palwasha Khan
Jahangir Khan
Mohsin Khan
Taseer Khan
Mohammad Khan
M. Salman Khan
Michael Khan
Waqar Ahmad Khan
Sheraz Khan
Hamid Khan
Fahim H. Khan
Zohaib Khan
Mohammad Aslam Khan
Atif Khan
Meraj Khan
Meena Khan
Nadia Khan
Mi Khan
Kashif Aziz Khan
Seema Khan
Masuma Khanam
Daniel Khananshvili
Rajiv Khandekar
Lidita Khandeparker
Gilson Khang
Dongwoo Khang
Quach Thi Khanh Ngoc
Ashwani Khanna
Savita Khanna
Hemant Khanna
Sahil Khanna
Ryan Khanna
Aditya Khanna
Nimish Khanna
Vishesh Khanna
Neelam Khaper

Tanya Khara
Michael Kharas
Kusum K. Kharbanda
Anshuman Khardenavis
Garima Khare
Samer Kharroubi
Ravindra Nath Kharwar
Ali Khashan
Patricia Khashayar
Alireza Khatami
Ameneh Khatami
Firas Khatib
Ali Khatibi
Mehar Khatkar
Mahmoud Khattab
Shahid Khattak
Muhammad Khattak
Amina Khatun
Aurangzaib Khawaja
Leslie Khawli
Paul Khayat
Yasser Khazaal
Mohamad Khazaei
Maher Khdour
George Khelashvili
Mehdi Khellaf
Sahil Khera
Farrah Kheradmand
Ferath Kherif
Radhika Khetani
Salman Khetani
Jong Seong Khim
Ashraf Khir
Alvin Kho
Kamran Khodakhah
Nikolai Khodarev
Konstantin Khodosevich
Shakeel Khoja
Iskandar Kholmanov
Saye Khoo
U.S. Khoo
Edmund Khoo
Angela Khor
Reza Khorooshi
Sohail Khoshnevis
Chaitan Khosla
Christine Khosropour
S. M. Khot
Samar Khoury
Anthony Khoury
Colin Khoury
Michael Khoury
Sadik Khuder
Yury Khudyakov
Polyna Khudyakov
Jane Khudyakov
Madhu Khullar
Syed Khundmiri
Benjawan Khuntirat
Surender Khurana
Sawsan Khuri
Mohammad Khuroo
Asim Khwaja
A. Khwitshana
Mahesh Khyade
Chang-Seok Ki
Sung Hwan Ki
Lawrence Kiage
Arash Kialashaki
Jalil Kianfar
Mohammad Kiani
Suda Kiatkamjornwong
Zoha Kibar
Frederick Kibenge
Charles Kibert
Anton Kichev
Michal Kicinski
Satoshi Kida
Tetsuo Kida
Teruyo Kida
D. Kidawa
Kenneth Kidd
Gerald Kidd
Thomas Kidd
Michael Kidd
La Creis R. Kidd
Martin Kidd
Sarah Kidd
Sean Kidd
Lisa Kidd
Benjamin Kidder
Til Kiderlen
Khameer Kidia
Rachel Kidman
Yoshiaki Kido
Hiroyuki Kidokoro

Giora Kidron
Claudine Kieda
Friedemann Kiefer
Florian Kiefer
Meghan Kiefer
Andreas Kiefer
Adam Kiefer
James Kieffer
Jessica Kiefte
Pawel Kiela
Jennifer Kielczewski
Renata Kieling
Karina Kielmann
Jan Kielstein
Heike Kielstein
Lambertus Kiemeney
Kathrin Kienapfel
William Kiene
Pascal Kienlen-Campard
Ann Kier
Uwe Kierdorf
Andrzej Kierzek
Bernd Kieseier
Jan Kiesewetter
Matthias Kieslinger
Silke Kiessling
David Kietrys
Thomas Kietzmann
Manfred Kietzmann
Sebastian Kiewnick
Peter Kiffney
Takanori Kigawa
Gabriel Kigen
Daisuke Kihara
Takanori Kihara
Yasuki Kihara
Lars Kihm
James Kijas
Aize Kijlstra
Ushio Kikkawa
Masahide Kikkawa
Yoshiaki Kikkawa
Rainer Kiko
Shoshi Kikuchi
Taisei Kikuchi
Mitsuru Kikuchi
Toshiaki Kikuchi
Eiji Kikuchi
Kazu Kikuchi
Ryosuke Kikuchi
Colin Kikuchi
Haruhito Kikuchi
Takefumi Kikusui
Zaal Kikvidze
Ferenc Kilar
Sreenivasulu Kilari
Varun Kilaru
Angela Kilb
John Kilbane
Todd Kilbaugh
Kelly Kilburn
Kanchana Kildegaard
Liam Kilduff
William Kilembe
Mogens Kilian
Kristopher Kilian
Monique Kilkenny
Anthony Killard
Timothy Killingback
Nabil Killiny
William Kilpatrick
Charlotte Kilstrup-Nielsen
John Kiluk
Kami Kim
Uh-Hyun Kim
Deok Ryun Kim
Joomyeong Kim
Donghee Kim
Jeansok Kim
Tae-You Kim
Ki-Joong Kim
Sangsoo Kim
Beom Jun Kim
Bum-Joon Kim
In-San Kim
Kyun-Hwan Kim
Young Bong Kim
Sung-Hoon Kim
Sin-Yeon Kim
Sung Soo Kim
Peter Kim
Hong Jin Kim
Kwang Soo Kim
J. Julie Kim
Yonggyun Kim
Haesun Kim

Kyong-Tai Kim
Ha Won Kim
Joonhoon Kim
Jung-Jae Kim
Gwang Hoon Kim
Jin Cheon Kim
Jin Woo Kim
Ji-Eun Kim
Isaac Kim
Young-Bum Kim
Soo Mi Kim
Dong Ki Kim
Yon Su Kim
Jae-Sung Kim
Manho Kim
Jong-Il Kim
Seung Up Kim
Kyoung-Mee Kim
Paul Kim
Tae-Kyung Kim
Woo Taek Kim
Eung-Soo Kim
Haeryoung Kim
Ho Kim
Hong-Jin Kim
Sunyoung Kim
Jayoung Kim
Beom Kyung Kim
Dong-Eog Kim
Soo-Youl Kim
Anthony W Kim
Han Sang Kim
Nam Kyu Kim
Hyung Kim
Woo-Yang Kim
Jongphil Kim
Nam Keun Kim
Jenny Kim
Jae Ho Kim
Seungtaek Kim
Kisoon Kim
Soo Wan Kim
Chae-Yong Kim
Kevin Kim
Jeong-Ho Kim
Yangmee Kim
Tae-Bum Kim
Jin Kim
Jang Kim
K. Kim
Seok Jin Kim
Namkug Kim
Tae-Woo Kim
Soochong Kim
Hee-Sun Kim
Gwang-Jin Kim
Larsson Kim
Sangyun Kim
You-Jin Kim
Tae Il Kim
Inho Kim
Suil Kim
Hongtae Kim
Eugene Kim
Inki Kim
Yoon-Seong Kim
Youl-Ri Kim
Moon Young Kim
Yong-Mi Kim
Shin-Hee Kim
Weon Kim
Hyoung-Ryoul Kim
Daniel Kim
Ho Sung Kim
H. S. Kim
Sang Kyum Kim
Yun Kyung Kim
Stephen Kim
Jeonga Kim
Hyun Kyung Kim
Ji Hoon Kim
Yeon-Ki Kim
Jungsu Kim
Hyeonjin Kim
Taeyoon Kim
Youngwon Kim
Jae-Jin Kim
Kyoung-Nam Kim
Edward Kim
Jeong-Gyu Kim
Yangjin Kim
Jinwon Kim
Young-Hak Kim
Hyun-Woo Kim
Nayun Kim
Bo Hyun Kim

Chan-Duck Kim
Il-Ho Kim
James Kim
Yongkyu Kim
Dae Woo Kim
Jong-In Kim
Soo Rin Kim
Changkyun Kim
Seongho Kim
Sun Kwang Kim
Sun Kim
Il-Doo Kim
Jong Kyong Kim
Bo Kim
Mi Na Kim
Jihye Kim
Baek Il Kim
Hyunjee Kim
Dokyoon Kim
Seul Ki Kim
Hun Kim
Hyun Kim
Jungrack Kim
Aimee Kim
Myung Chul Kim
Yonghyun Kim
Young Kim
Sung Joon Kim
Young-Jae Kim
Hye Kyong Kim
Sun Kwon Kim
Min Hwan Kim
Jeong-Sun Kim
Seok-Won Kim
Seok-Ki Kim
Theresa Kim
Yong Bae Kim
Jung-Whan Kim
Seong Hwan Kim
Suhn Hee Kim
Kyoung-Han Kim
Joon Mo Kim
Min Joo Kim
Tae Hyun Kim
Young-Mo Kim
Jeong Han Kim
Helen Kim
Ja-Yeon Kim
Seok-Jo Kim
Brian Kim
Yong-Sung Kim
Hyekyeong Kim
Bojeong Kim
Sanha Kim
Dong-Yun Kim
Wk Kim
Hail Kim
Yuna Kim
Jung-Ae Kim
Tae-Jip Kim
Bum Jung Kim
J. G. Kim
Joonyyoung Kim
Sang Gyune Kim
Jee Hyun Kim
Jonghwa Kim
Su Young Kim
Woochul Kim
Yuri Kim
Jeong Kon Kim
Do-Hyun Kim
Jonathan Kim
Mansuck Kim
Cha Young Kim
Albert Kim
Michelle M. Kim
Jeffrey Kim
Chan K. Kim
Su-Kang Kim
Beob Gyun Kim
Ho Jin Kim
Jongkee Kim
Myoung Sook Kim
In Jung Kim
Wook Kim
Kwang Dong Kim
Minkyoung Kim
Eun Kim
Sunae Kim
Joo Seung Kim
Jinsang Kim
Il-Man Kim
Jin Kim
Jin-Hwan Kim
Sang-Ki Kim
Tae Hoon Kim

Nam Deuk Kim
Donghern Kim
Michelle Kim
Byeong Kim
Jaeyoun Kim
Jongoh Kim
Hye Young Kim
Jeong Ho Kim
Christopher Kim
Tania Kim
Yong Soo Kim
Dong-Ki Kim
Daejin Kim
Dong-Hyun Kim
Seungwon Kim
Hyunjae Kim
Hwi Young Kim
Cheolgi Kim
Hojun Kim
Jung-Yeul Kim
Hae Won. Kim
Seong-Jun Kim
Sun Tae Kim
Hyeun Bum Kim
Hoseong Kim
Peter Kima
James Kimani
Daniel Kimball
Jonathan Kimball
Melissa Kimber
Oliver Kimberger
Robert Kimberly
Zaneta Kimber-Trojnar
Kevin Kimbro
Ismael Aaron Kimirei
David Kimmel
Shokei Kim-Mitsuyama
Virginia Kimonis
Randall Kimple
Daniel Kim-Shapiro
Hiroshi Kimura
Tadashi Kimura
Tsuyoshi Kimura
Yoko Kimura
Nobuyuki Kimura
Cindy Kin
John Kinahan
Paul Kinchington
Zsigmond Tamas Kincses
Karen Kind
Pieter-Jan Kindermans
Robert Kinders
Detlef Kindgen-Milles
Thomas Kindler
Pavel Kindlmann
Gurpreet Kindra
Mark Kindy
Rhonda Kineman
Mary-Claire King
Michael King
Paul W. King
Mark King
Peter King
David King
Paul King
Aileen King
Charlotte King
Derek King
Malcolm King
Deborah King
Kevin King
Carina King
Richard King
Jessica King
Annie King
Laurie King
Stanley King
Suzanne King
Tommy King
Tisha King Heiden
Fred Kingdom
Wade Kingery
Paul Kingham
Peter Kingshott
J. Derek Kingsley
Danny Kingsley
Stephen Kingsmore
David Kingsmore
Joel Kingsolver
Dawn Kingston
Andrew Kingston
Alan Kingstone
R. Kini
Milan Kinkhabwala
Raimund Kinne
Stuart Kinner

Margie Kinnersley
Patrick Kinney
Tomoshige Kino
Masahiro Kinoshita
Sachiko Kinoshita
Hiroyuki Kinoshita
Tetsu Kinosita
Michael Kinsella
Paula. Kinsella
Todd Kinsella
Glynda J Kinsella
Elaine Kinsella
Steven Kinsey
Craig Kinsley
Audrey Kinter
Eugene Kinyanda
Damaris Kinyoki
Karin Kiontke
Thomas Kiørboe
Dimitrios Kiortsis
Anja Kipar
Kurt Kipfmueller
Aaron Kipp
Markus Kipp
Deborah Kipp
Benjamin Kipp
Frank Kipp
Anna Kipp
Nestor Kippes
Judy Kipping
Gokhan Kir
Russell Kirby
Karen Kirby
Lynn Kirby
Edward Kirby
Bronywn Kirby
Kate Kirby
Miranda Kirby
Amy Kirby
Frank Kirchhoff
Louis Kirchhoff
Anne Kirchhoff
C Kirchhoff
David Kirchman
Lena Kirchner Brahe
Bruce Kirenga
Kostas Kiriakoulakis
Girish Kirimanjeswara
Kohtaro Kirimura
Yohei Kirino
Erkan Kiris
Nathan Kirk
Jonathan Kirk
Adam Kirk
Julie Kirkby
Robert Kirkcaldy
Carsten Kirkeby
Karla Kirkegaard
Grant Kirker
Jamie Kirkham
Amy Kirkham
Pertti Kirkinen
Jackson Kirkman-Brown
Bruce Kirkpatrick
Jamie B. Kirkpatrick
Mike Kirkpatrick
Benjamin Kirkup
Les Kirkup
John Kirkwood
Thomas Kirkwood
Keith Kirkwood
Nameer Kirma
Andrea Kirmaier
Serdal Kirmizialtin
Antonis Kirmizis
Reinhard Kirnbauer
George Kirov
Dmitri Kirpotin
Peter Kirsch
Louise Kirsch
Alexander Kirsch
Matthew Kirschen
Susanne Kirschnek
Denise Kirschner
Femke Kirschner
Paul Kirshen
Lorrie Kirshenbaum
Kent Kirshenbaum
Christoph Kirst
Frank Kirstein
Mark Kirstein
Peggy Kirstetter
Adam Kirton
John Kirwan
Anna Kis
Bernhard Kis

Valerij Kiselev
Konstantin Kiselev
Anton Kiselev
Irina Kiseleva
Laszlo Kish
Tomoo Kishaba
Takuya Kishi
Taro Kishi
Shigeki Kishi
Satoshi Kishida
Masahiro Kishii
Toshihiko Kishimoto
Hiro Kishimoto
Taishiro Kishimoto
Takashi Kei Kishimoto
Akihiro Kishimura
Hirohisa Kishino
Takayoshi Kishino
Dmitry Kishkinev
Uday Kishore
Nand Kishore
Raj Kishore
Dagmara Kisiela
Zelma Kiss
Szilárd Kiss
John Kiss
Adrien Kissenpfennig
Peter Kissinger
S. Kissler
Grace Kissling
James Kistler
Andrea Kistner
Katalin Kis-Toth
Ichiro Kita
Kazuo Kitagawa
Hiroshi Kitagawa
Nobuyoshi Kitaichi
Kazuhiro Kitajima
Masaaki Kitajima
Kenji Kitajima
Shin-Ichiro Kitajiri
Keiichi Kitajo
Sabrina Kitaka
Masafumi Kitakaze
Yasunori Kitamoto
Katsuhiko Kitamoto
Toshio Kitamura
Kenichiro Kitamura
Kazuo Kitamura
Tomomi Kitamura
Hidemitsu Kitamura
Ken Kitamura
Yoshiichiro Kitamura
Hiroshi Kitani
Katsunori Kitano
Shigehisa Kitano
Akio Kitao
Azusa Kitao
Hiroyasu Kitashiba
Joji Kitayama
Haruki Kitazawa
Andrew Kitchener
Henry Kitchener
Maria Kitchen-Hosp
A. Kitchlu
Joanna Kitlinska
Shunsuke Kito
Hiroshi Kitoh
Maksim Kitsak
Paraskevi Kitsiou
Glenn Kitsune
Pattamaporn Kittayapong
Robert Kittel
J. Matthew Kittelberger
Todd Kitten
Daniel Kitterer
Padraig Kitterick
John Kittinger
Harald Kittler
James Kitts
Andrew Kitua
Manfred Kitzbichler
Yoshiaki Kiuchi
Sami Kivelä
Sonja Kivinen
Shaye Kivity
Stephanie Kivlin
Ryoiti Kiyama
Hideyasu Kiyomoto
Shigeki Kiyonaka
Toko Kiyonari
Tohru Kiyono
Hiroshi Kiyono
Rene Kizek
Caghan Kizil
Jasmin Kizilirmak

Alegrria Kizilova
Dimosthenis Kizis
Toshikazu Kizuka
Per Kjær
Michael Kjaer
K. D. Kjaergaard
Curtis Klaassen
Marcel Klaassen
Eline Klaassens
Cheryl Klaiman
Guenter Klambauer
Christian Klambt
Marian Klamer
Lidija Klampfer
Petr Klan
Barbara Klapcinska
Rebecca Klaper
Benjamin Klapholz
Holger Klapproth
Philipp Klaritsch
George Klarmann
André Klarsfeld
Sven Klaschik
Ursula Klaschka
Per Johan Klasse
Jonathan Klassen
Giseli Klassen
Nichole Klatt
David Klatzmann
Osmar Klauberg Filho
Jeffery Klauda
Sheila Klauer
Susanne Klaus
Bernd Klaus
David Klaus
Miriam Klausberger
Robert Klautz
Rolf Kleber
Marcus Kleber
Jan Klecka
Irena Kleckova
Leszek Kleczkowski
Steven Kleeberger
Nanne Kleefstra
Steven Kleene
Michiel Kleerebezem
Thomas Klefoth
Oliver Klefter
Dimitrios Kleftogiannis
Alexander Kleger
Andis Klegeris
Juliane Klehmet
Thomas Klei
Petra Kleiblová
Oded Kleifeld
David Kleijn
Birgit Kleim
Evan Kleiman
William Klein
Ulf Klein
E. K. Klein
Gerd Klein
Stanley Klein
Robyn Klein
David Klein
Reinhild Klein
Nicole Klein
Travis Klein
Richard Klein
Thomas Klein
Johannes Klein
Mark Klein
Eili Klein
Verena Klein
Matthias Klein
Kerenaftali Klein
Sabine Klein
David M. Klein
Jonathan Klein
Marlise Klein
Michael Klein
Samantha Kleinberg
Petra Kleinbongard
Christoph Kleineidam
David Kleiner
Markus Kleinewietfeld
Marisa Klein-Gitelman
Alex Kleinjan
Jens Kleinjung
Joel Kleinman
Mark Kleinman
Guy Kleinmann
Oded Kleinmintz
Christoph Kleinn
Johanneke Kleinnijenhuis
Iivari Kleino

Ruth Kleinpell
Christoph Kleinschnitz
Andres Klein-Szanto
Nina Kleint
Thomas Kleinteich
Annabelle Kleist
Ingo Kleiter
Sabina Kleitman
Karel Klem
Zalika Klemenc-Ketis
Giannoula Klement
Reija Klemetti
Konstantin Klemm
Robin Klemm
Friederike Klempin
Jochen Klenk
Ulrike Klenke
Anna Klenova
Werner Kleophas
Anna Klepikova
Rune Kleppe
Magne Kleppest
Konrad Kleszczynski
Gijs Kleter
Dimitris Kletsas
Alexa Klettner
Scott Klewer
Thomas Kleyman
Alexander Klibanov
Alan Klide
Claudia Klier
Catharina Klijn
Bart Klijs
Vaclav Klika
Lars Klimaschewski
Magdalena Klimek
Corrine Kliment
Florian Klimm
Pavel Klimov
Anna Klindworth
Kimberly Kline
Dan Kline
Christopher Kline
David Kline
Jeffrey Kline
Sabine Kling
Catherine Kling
Torkel Klingberg
Uwe Klinge
Carolyn Klinge
Karin Klingel
David Klinger
Peter Klink
Thorsten Klink
Barbara Klink
David Klinke
Eveline Klinkenberg
Kenneth Klinker
Florian Klinker
Dennis Klinman
Adi Kliot
Anna Klisinska-Kopacz
Alexander Klistoner
Alexander Klistorner
Kirstine Klitgaard
Sebastian Klobuch
Helmut Klocker
Amy Klocko
Lorie Kloda
Stefan Kloeppel
Laura Kloepper
Stefan Kloiber
Dennis Klomp
Bernard Klonjkowski
Kimberly Klonowski
Wlodzimierz Klonowski
Matthias Kloor
Wigard Kloosterman
Jan Kloosterman
Tomas Kloosterman
Robert Klopfleisch
Ann Klopp
Christophe Klopp
Raoul Kloppenborg
Peter Kloppenburg
Edda Kloppmann
Thomas Klopstock
Rafael Klorman
Karl Klose
Petra Klose
Ralf Klosgen
Janusz Kloskowski
Anita Kloss-Brandstätter
Andre Klostermann
Nora Klöting
Luisa Klotz

David Klotzkin
Vasily Klucharev
Ruth Kluck
Tim Klucken
Jochen Klucken
Anke Klueter
Bryan Kluever
Christian Klug
Dennis Klug
Jürgen Kluge
Bernhard Kluger
Keith Klugman
Joshua Klugman
Georgette Kluiters
Uli Klumper
Katja Klumpp
Barbara Klupp
Michael Klüppel
Enno Klussmann
Stephen Klusza
Johannes Kluwe
Eric Kmiec
Tomas Knapen
Leslie Knapp
Guido Knapp
Deborah Knapp
Michal Knapp
David Knapp
Michael Knapp
Susanne Knapp
Susanne Knappe
Paul Knappenberger
Steven Knapper
Marit Knapstad
Felix Knauer
Claude Knauf
Jeffrey Knauf
Felicia Knaul
Birgit Knebel
Erwin Knecht
Robert Kneller
Mark A. Knepper
Ulrich Kneser
Zorica Kneževic-Jugovic
Kali Kniel
Nina Kniel
Olaf Kniemeyer
Ellen Knierim
Kendall Knight
Marc Knight
Rob Knight
Gwenan Knight
Stefan Knight
Sarah Knight
Rod Knight
Jason Scott Knight
Theodore Knight-Jones
Jennifer Knight-Madden
Dan Knights
David Knipe
Marlies Knipper
Douglas Kniss
Joshua Knobe
M Knobf
Jürgen Knobloch
Tobias Knoch
Paul Knoepfler
Bernd Knoll
Nadja Knoll
Brian Knoll
Maximilian Knoll
Björn Knollmann
Volker Knoop
Michael Knop
Katrina Knope
Stefanie Knopp
Michael Knopp
Steve Knotek
Ben Knott
Cheryl Knott
Thomas Knott
P. Knott
William Knowler
Juliet Knowles
Joshua Knowles
Jessie Knowlton
Christine Knox
Dayan Knox
Beatrice Knudsen
Vibeke Knudsen
Lars Knudsen
Kelly Knudson
Cory Knudson
Helmut Knuepffer
Larissa Knüppel
Mitchell Knutson

Anders Knutsson
Heather Knych
Ya-Ping Ko
Gladys Ko
Chemyong Ko
Dennis Ko
Seong-Gyu Ko
Kwan Soo Ko Ko
Shun-Yao Ko
Hyun-Jeong Ko
Winne Ko
Sung-Youl Ko
Ja Kyong Ko
Ginger Ko
Shinji Koba
Jaroslaw Kobak
Darwyn Kobasa
Hidenaga Kobashi
Moptoi Kobashi
N. Kobayashi
Susumu Kobayashi
Katsunori Kobayashi
Shoko Kobayashi
Eiji Kobayashi
Yoshitsugu Kobayashi
Masayoshi Kobayashi
Donald Kobayashi
Kazuya Kobayashi
Hiroaki Kobayashi
Jun Kobayashi
Tamaki Kobayashi
Shunsuke Kobayashi
Lindsay Kobayashi
Takumi Kobayashi
Makito Kobayashi
Kazuyuki Kobayashi
Kentaro Kobayashi
Shigeru Kobayashi
Kazuo Kobayashi
Makoto Kobayashi
Miwako Kobayashi
Tetsuo Kobayashi
Daisuke Kobayashi
Firas Kobeissy
Martina Köberl
James Kobler
Beryl Koblin
Jennifer Koblinski
Jens Koblitz
Stephan Koblmueller
Marta Koblowska
Masuko Kobori
Hiroyuki Kobori
Katja Kobow
Lester Kobzik
Jaroslav Koca
Katherine Kocan
Benjamin Kocar
Jan Kocbach
Raphael Koch
Guus Koch
Iring Koch
Michael Koch
Wayne Koch
Marcus Koch
Frank Koch
Philipp Koch
Walter Koch
Christiane Koch
Sabine Koch
Katharina Koch
Robert Koch
Andre Koch
Hemant Kocher
Thomas Kocher
Jacob Kocher
Yuta Kochi
Bose Kochupurakkal
Krys Kochut
Matthew Koci
Robin Köck
Ferdinand Köckerling
Jens Kockskämper
Ingrid Kockum
Maaike Kockx
Kevin Kocot
Bernat Kocsis
G. Kocsy
Andreas Koczulla
Andreas Rembert Koczulla
Ryszard Koczura
Hiroki Koda
Masao Koda
Yusaku Kodaka
Toshio Kodama
Naoki Kodama

Maja Kodani
Gergana Kodjebacheva
Dwight Koeberl
Ralf Koebnik
Birgit Koechl
Uwe Koedel
Muriel Koehl
Michael Koehle
Sebastian Koehler
Philip Koehler
Reinhard Koehler
Jonathan Koehler
Lizette Koekemoer
Katja Koelkebeck
Roland Koelliker
Phil Koellinger
Leo Koenderman
Christian Koenecke
Rory Koenen
Inke Koenig
Daniel Koenig
Gabriele Koenig
Rainer Koenig
Melissa Koenig
Joyce Marie Koenig
Walter Koenig
Mike Koenigs
Joerg Koenigstorfer
Roger Koenker
Yvonne Koenraads
Constantianus Koenraadt
Tyler Koep
Cristian Koepfli
Deanna Koepp
Michael Koeppen
Hermann Koepsell
Richard Koerber
Leonardo Koerich
Henk Koerten
Gerhard Koertner
Doris Koesling
Dirk Koester
Diana Koester
Gerta Koester
Devin Koestler
Benjamin Koestler
Dagmar Koethe
Ad Koets
Jan Koetsenruijter
Peter Koetsier
Harald Köfeler
Alphonsine Koffi
Erik Koffijberg
Julia Kofler
Jiri Kofranek
Ryuichi Koga
Masatoshi Koga
Sivaramakrishna Koganti
Karl-Heinz Kogel
Lisette Kogelman
Karin Kogermann
Kentaro Kogure
Michael Kogut
Jung-Min Koh
Duk-Su Koh
Andrew Koh
Won-Jung Koh
Hee-Jong Koh
Woon-Puay Koh
Chang-Sung Koh
Seong-Ho Koh
Timothy Koh
Yasuhiro Koh
Young Ho Koh
Mariko Koh
Wan Koh
Victor Koh
Ho-Jin Koh
R. Sarah Kohansal
Katsuhiko Kohara
Takashi Kohda
Amnon Kohen
Ron Kohen
Matthias Kohl
Kevin Kohl
Axel Kohler
Hans-Peter Kohler
Mark Kohler
Annegret Kohler
Stefan Kohler
Gerwald Kohler
Connie Kohler
Jörn Köhler
Thilo Köhler
Jane Kohlhoff
Rahul Kohli

Ajay Kohli
Gurjeet Kohli
Rüdiger Köhling
Niko Kohls
Sepp Kohlwein
Michael Kohn
Andre Kohn
David Kohn
Takashi Kohno
Milky Kohno
Satomi Kohno
Mark Kohr
Josef Köhrle
Takahide Kohro
Shun Kohsaka
Jun Kohyama
Noriyuki Koibuchi
Shohei Koide
Roger Koide
Masayo Koide
Takao Koike
Masato Koike
Hideaki Koike
Teruhiko Koike
Shinsuke Koike
Yasuharu Koike'
Ari-Pekka Koivisto
Peppi Koivunen
Jarko Koivunen
Akio Koizumi
Nobuo Koizumi
Georg Kojda
Itaru Kojima
Seiji Kojima
Gotaro Kojima
Hisaya Kojima
Ilpo Kojola
Jen Kok
Chee Choy Kok
Philippe Kok
Gemma Kok
Gerjo Kok
Bethany Kok
Robin Kok
Sang-Heng Kok
Victor Kok
Peter Kok
Koichi Kokame
Arif Kokcu
Marcela Kokes
Michail Kokkoris
Efi Kokkotou
Zenon Kokot
Niels Kokot
Sulev Koks
Tugba Kok-Tas
Hiroki Kokubo
Norihiro Kokudo
Axel Kola
Bryan Kolaczkowski
Saeed Kolahian
John Koland
Filip Kolár
Daniel Kolarich
Andrew Kolarik
Joseph Kolars
Jurek Kolasa
Robert Kolasinski
Julia Kolata
Pappachan Kolattukudy
Hans-Jochem Kolb
E. Kolb
Jurgen Kolb
Frederic Kolb
Roland Kolbeck
Benedict Kolber
Morey Kolber
Jonathan Kolby
Crystal Kolden
Martin Kolditz
John Kolega
Tony Koleske
Despina Koletsi
Poornima Kolhar
Vassilis Koliatsos
Andrzej Kolinski
Randall Kolka
Miriam Kolko
Anne Kolko
Anup Kollanoor Johny
Attila Kollar
Nicole Kollars
Ingrid Koller
Monika Koller
Barbara Kollerits
Florian Kollert

Orit Kollet
Katja Kollewe
Laxmikanth Kollipara
Simon Kollnberger
Kathrin Kollndorfer
Jay Kolls
Nageswara Rao Kollu
Harald Kolmar
Carolin Kolmeder
Ilana Kolodkin-Gal
Gerald Kolodny
Kerstin Kolodzie
Alexander Kolovos
Joseph Kolowski
Svein Kolset
Charles Kolstad
Simon Kolstoe
Dhaval Kolte
James Koltes
Kairi Kolves
Oleksandr Kolyvushko
Krishna Komanduri
Nicholas Komar
Narayana Komaravelli
Martin Komarc
Anthony Komaroff
Andras Komaromy
Paul Komesaroff
Tamas Komives
Dragan Komljenovic
John Komlos
Ramakrishna Kommagani
Yuta Komoike
Michal Komorowski
Yanusobo Komoto
Christian Komposch
Kimiaki Komukai
Christian Komusiewicz
Elizaveta Kon
Kelika Konda
Vani Konda
Ganesh Kondabattula
Paturu Kondaiah
Divya Kondaveeti
Subramanyam Kondeti
Akihiko Kondo
Eisei Kondo
Naoki Kondo
Hirohito Kondo
Kazuhisa Kondo
Takashi Kondo
Keiichi Kondo
Yasuteru Kondo
Makoto Kondo
Hidemasa Kondo
Kazuya Kondo
Tadashi Kondo
Yasuhiro Kondoh
Ivan Kondov
Irena Kondova
Ganesh Konduri
Bruce Kone
Vladimir Konecni
Richard Kones
Richard Kong
Xiangyin Kong
Xiangrong Kong
Lan Kong
De-Xin Kong
Wei Kong
Jian Kong
Weijia Kong
Bo Kong
Lingbo Kong
Qingpeng Kong
Xiangpei Kong
Qiusheng Kong
Lingyi Kong
Xiangyu Kong
Fanna Kong
Shengchun Kong
Fanhua Kong
Fanjiang Kong
Mei Kong
Anthony Kong
Yong Lin Kong
Sinyi Kong
Qiang Kong
William Kong
Xingxing Kong
Linglong Kong
Yawei Kong
Fanchang Kong
Xiuying Kong
Jin Kong
Anine Kongelf

Warat Kongkitkul
Frank Konietschke
Maximilian Konig
Julia Konig
Peter König
Bettina König
William Konigsberg
Oliver Königsbrügge
Mark Konijnenberg
Jacob Konikoff
Kazuo Konishi
Justin Konje
Prasad Konkalmatt
Kotaro Konno
Masahiro Kono
Elisa Konofagou
Veronika Konok
James Konopka
Genevieve Konopka
Adam Konopka
Dorota Konopka-Postupolska
Marcel Konrad
Sara Konrath
Theocharis Konstantinidis
Efstathios Konstantinidis
George Konstantinou
Sergey Konstantinov
Igor Konstantinov
Maria Konstantopoulou
Tsuneo Konta
Maria Kontaridis
Roland Kontermann
Vasilis Kontis
Dimitris Kontodimas
Evangelos Kontopantelis
Theano Kontopoulou
Christopher Kontos
Kimmo Kontula
Anatol Kontush
Kevin Konty
Kishori Konwar
Malcolm Koo
Terry Koo
Jawoo Koo
Jason Koo
Michael Koob
Jalil Koohpayehzadeh
Edgar Eduard Kooijman
Judith Kooiman
Wiebe Kooistra
Wouter Kool
Marijn Koolen
Woong Sub Koom
Jeroen Kooman
John Koomen
Yen Ling Koon
David Koons
Demian Koop
René Koopman
Jacob Koopman
Cheryl Koopman
Sietse-Jan Koopmans
Klaas Pieter Koopmans
Joseph Koopmeiners
Elouise Koops
Susanne Koot
Neeltje Kootstra
R. Kooy
Petr Kopacek
Doris Kopahnke
Alexander Kopatz
Gelina Kopeina
Dmitry Kopelevich
Juliane Kopf
Rene Kopietz
Jurgen Kopitz
Jennifer Koplin
Helen Kopnina
Jeffrey Kopp
Michael Kopp
Matthias Kopp
Ulla Kopp
Helena Kopp Kallner
Janna Koppe
Georgia Koppe
Janet Koprivnikar
John Koprowski
Suzanne Koptur
Zeljka Korade
Günther Koraimann
Igor Koralnik
Lorrin Koran
Mohammed Korashi
Rüdiger Korbel
Philipp Korber
Márta Korbonits

Tamás Korcsmáros
Izabela Korczowska
Claus Kordes
Konrad Kording
Erez Koren
Ruth Koren
Ivan Korendovych
Andrej Korenic
Nina Koren-Karie
Daniel Korevaar
Ian Korf
Horst-Werner Korf
Katrina Korfmacher
Maarit Korhonen
Piyush Koria
Julia Koricheva
Netanel Korin
Carmi Korine
Birgit Korioth-Schmitz
Miikka Korja
Mert Korkali
Hasan Korkaya
Brice Korkmaz
Kostantinos Kormas
David Korn
Thomas Korn
Dieter Korn
Cornelia Kornblum
Martin Korndörfer
H Korner
Antje Körner
Heinrich Körner
André Körner
Irv Kornfield
Melanie Kornides
Olga Kornienko
Panagiotis Kornilios
Gyorgy Korniss
Birgitte Kornum
Zsuzsanna Környei
Przemyslaw Korohoda
Abraham Korol
Igor Korolev
Victoria Korolik
David Korones
Aniko Korosi
Grigorios Korosoglou
Konstantin Korotenko
Konstantin Korotkov
Katri Korpela
Helena Korpelainen
Sigrun Korsching
Catharina M. Korse
Robert Korst
Ron Korstanje
Peter Korsten
Hendrik Korswagen
Eric Kort
Marion C Kortekaas
Tanja Kortemme
William Korth
Nicoline Korthagen
Trond Kortner
Christina Kortsalioudaki
Anne Kortstee
Steven Korzeniewski
Viktor Korzun
Lidia Kos
Janko Kos
Martine Kos
Cara Kosack
Takayuki Kosaka
Herman Kosasih
Thomas Kosatsky
Matthias Koschorreck
Krzysztof Koscinski
Anne Kösem
Aneta Koseska
Sentaro Koshida
Naohiko Koshikawa
Jill Koshiol
Nadya Koshkina
Douglas Koshland
Anita Koshy
Ajeesh Koshy Cherian
Lana Kosi Trebotic
Michal Kosinski
Sonja Koski
Gary Koski
Spee Kosloff
Matthew Koslow
Ralf Kosma
Arkadiusz Kosmala
Daniel Kosman
Ioannis Kosmas
Vasiliki Kosmidou

Agnieszka Kosny
Simone Kosol
Klára Kosová
Michael Kosoy
Michael Koss
Nina Kossack
Fabian Kosse
Eric Kossoff
Eric Kostelich
Andreas Koster
Remco A Koster
Karel Kostev
Srdjan Kostic
Joel Kostka
Tomasz Kostka
Patty Kostkova
Leondios Kostrikis
Lidia Kostyniuk
Janaiah Kota
Hiroki Kotabe
Yojiro Kotake
S. Kotake
Toshihisa Kotake
Laura Koteen
Ayman A. Koteish
Manas Kotepui
Stephan Koter
Masaaki Kotera
Shiva Kotha
David Kothamasi
Naga Rama Kothapalli
Kumar S. Kothapalli
Ute Kothe
Linda Kothera
Madhuri Koti
Anja Kotiranta
Tadej Kotnik
Georgios Kotoulas
Kurt Kotrschal
Michalis Kotsyfakis
Kameswara Rao Kottapalli
Mark Kotter
Melissa Kotterman
Shyam Kottilil
Matt Kottmann
Leah Kottyan
M. Kotula-Balak
Sradha Kotwal
Eszter Kotyuk
Daniel Kotz
Andrew Kotze
Zhihua Kou
Yu Ru Kou
Azam Kouhkan
Banno Kouji
Jari Kouki
Artemis Koukounari
M. Koukourakis
Annette Koulakoff
Albert Koulman
Maria Koulmanda
Miroslav Koulnis
Lefteris Koumakis
George Koumbaris
Ilias Kounatidis
Petros Kountouris
Richard Koup
Dimitrios Kouretas
Robert Kourist
Yiannis Kourkoutas
Dimitrios Kourtis
Takayuki Kousaka
Anita Koushik
Evanthia Kousi
Konstantin Kousoulas
Konstantinos Koussis
Stavroula Kousteni
Petr Koutecky
Glykeria Koutina
Marek Koutny
Stella Koutros
Spyridon Koutroubas
Nikos Koutsias
Michael Koutsilieris
Dimitrios Koutsonanos
Eduardo Koutsoukos
Kostas Koutsoumanis
Vassilis Kouvelis
Fiona Kouyoumdjian
Motoki Kouzaki
Andrej Kovac
Megan Kovac
Stjepana Kovac
Dejan Kovac
Anthony Kovac
Michal Kovac

Milica Kovacevic
Aleksandar Kovacevic
Ryan Kovach
Jason Kovacic
Jozef Kovacik
Ilona Kovacs
Peter Kovacs
Krisztina Kovacs
Daniela Kovacs
Kornel Kovacs
Andrea Kovacs
K. Kovacs
Balazs Kovacs
Stephanie Kovacs
Gyula Kovacs
Katalin Kovacs
Denes Kovacs
Luciana Kovacs
Levente Kovacs
Mihály Kovács
Illés Kovács
Vilas Kovai
Peter Koval
Katya Kovalenko
Ilona Kovalszky
Petri Kovanen
Joy Kovar
Pavel Kovarik
Ales Kovarik
Jaromír Kovárík
Sari Kovats
Susan Kovats
Charles Koven
Nik Kovinich
Prashanthi Kovur
Rafal Kowalczyk
Michal Kowalewski
Marc Kowalkowski
Bernd Kowall
Kira Kowalska
Justyna Kowalska
Greg Kowalski
Markus Kowarik
Renu Kowluru
Meenal Kowshik
M Kox
Eisuke Koya
Alain Koyama
Tatsuki Koyama
Ryuta Koyama
Maki Koyama
Tetsuo Koyama
Akihiro Koyama
Yutaka Koyama
Yoshio Koyanagi
Jay Koyner
Leslie Kozak
Igor Kozak
Josef Kozak
Ashot Kozak
Genevieve Kozak
Maciej Kozak
Magdalena Kozakowska
Roland Kozdrowski
Beth Kozel
Tamas Kozicz
Henry Koziel
Marek Kozinski
Elizabeth Koziol
Vera Kozjak-Pavlovic
Tomasz Kozlecki
Julia Kozlitina
Ken Kozloff
Michael Kozlov
Andrey Kozlov
Stanislav Kozlovsky
Pamela Kozlowski
David Kozono
Michal Kozubek
Lukasz Kozubowski
Gurukumar Kr
Jeff Kraakevik
Peter Kraal
Claudine Kraan
Alexandra Kraberg
Ian Kracalik
Sören Krach
Rainer Kraehenmann
Fredric Kraemer
Richard Kraemer
Brian Kraemer
Alwin Kraemer
Roberto Kraenkel
Nicolle Kraenkel
Antje Kraft
Peter Kraft

Clifford Kraft
Robert Kraft
Beate Kraft
Joan Kraft
Chris Krageloh
Udo Kragl
Rudiger Krahe
Thomas Krahe
Rainer Krähenmann
Ekaphan Kraichak
Peter Kraiczy
Pavel Kraikivski
Dimitri Krainc
Maciej Krajcarz
Maja Krajinovic
Rosa Krajmalnik-Brown
Nir Krakauer
Deborah Krakow
Michelle Krakowski
Andrej Kral
John Kral
Stefan Kralev
Petr Kralik
Martina Kralinger
Simona Kralj-Fiser
Rodger Kram
Vardit Kram
Richard Kramer
Boris Kramer
Michael Kramer
Marcel Kramer
Arthur Kramer
Joseph Kramer
John Kramer
Holger Kramer
Adam Kramer
Oliver Krämer
Nicole Krämer
Andrei Kramerov
Florian Krammer
Henning Krampe
Indrikis Krams
Anna Kramvis
Kamil Kranc
Hendrik-Jan Kranenburg
Michael Krangel
Alexander Kranjec
Alexander Krannich
Julie Krans
Philip Kranzusch
Dario Krapf
Nikolaj Krarup
Anna Krasnodembskaya
Aleksei Krasnov
Mark Krasnow
Matthew Krasowski
Vessela Krasteva
Zoltan Krasznai
Dagmar Kratky
Martin Kratky
Lucas Kratochvil
Franz Kratochvill
Claudius Kratochwil
Anton Kratz
Karl Kratz
Wolfgang Kratzer
Ian Kraucunas
Jennifer Krauel
Alexander Kraupner
Johann Kraus
Johanna Kraus
Theo Kraus
Allison Kraus
Robert Kraus
Olaf Kraus De Camargo
David Krause
Jens Krause
Philip Krause
Peter Krause
Kirsten Krause
Johannes Krause
Stefan Krause
Sebastian Krause
Hans Joachim Krause
Robert Krause
Rui Krause
Annalinda Krause
Annegret Krause-Utz
Stefan Krauss
Sybille Krauß
Ulrich Krauss
Michael Krausz
A. Kraut
Ellen Krautkrämer
Andra Krauze
Jacqueline Kraveka

Alexxai Kravitz
Peter Krawczel
Przemek Krawczyk
Marcin Krawczyk
Stephen Krawetz
Peter Krawitz
Merle Krebber
Charles Krebs
Robert Krebs
Michael Krebs
Emanuel Krebs
Susan Krebs-Smith
Raymond Krediet
Cory Krediet
Pamela Kreeger
Jürgen Kreft
Henrik Krehenwinkel
Saskia Kreibich
Richard Kreider
Consuelo Kreider
Lothar Kreienbrock
Benjamin Kreifelts
Anat Kreimer
Robert Kreitman
Silke Kreitz
Lumir Krejci
Premysl Krejcí
Bart Krekelberg
Natali Krekeler
Gerald Krell
Maxwell M. Krem
William Kremen
David Krementz
Laurent Kremer
Natacha Kremer
Jan Kremers
Mira Krendel
Guido Krenning
Hartmut Krentz
Joan Krepinsky
Miklos Krepuska
Michaela Kress
Olga Kresse
Dieter Kressler
Mariska Kret
Karsten Kretschmer
Hildrun Kretschmer
Michael Kreuter
Urs Kreuter
Roger Kreuz
Jan Kreuze
Grigorios Krey
Sebastian Krey
Wojciech Krezel
Oleg Krichevsky
Jeffrey Krichmar
Rami Kridli
Alison Kriegel
Marco Krieger
Jan Krieger
Ana Krieger
John Krigbaum
Timothy Kring
Morten Kringelbach
Jens Kringelum
Sharon Krinsky-Mchale
Boris Krischek
Vera Krischik
Gintas Krisciunas
Kewal Krishan
Rebecca Krisher
Neel Krishna
Murali Krishna
Vibhor Krishna
Somashekar G Krishna
D. Krishna Kumar
Subramanian Krishnakumar
Vivek Krishnakumar
Janarthanan Krishnamoorthy
Raghu Krishnamoorthy
Nandini Krishnamoorthy
Ramesh Krishnamurthy
Karthik Krishnamurthy
Arvind Krishnamurthy
Prasanna Krishnamurthy
Natraj Krishnan
Suneeta Krishnan
Giri Krishnan
Anand Krishnan
Ramaswamy Krishnan
Archana Krishnan
Vengadesan Krishnan
Gopal Krishnan
Sathya Krishnasamy
Jagdish Krishnaswamy
Sriram Krishnaswamy

Patnam Krishnaswamy
Anita Krisko
Bernhard Krismer
Jukka Krisp
Milos Krist
William Kristan
Jean Kristeller
Erik Kristensen
Michael Kristensen
Lars Erik Kristensen
Bjarne Kristensen
Tibor Kristian
Karsten Kristiansen
Glen Kristiansen
Magnhild Kristiansen
Christopher Kristich
Arni Kristjansson
Ladislav Kristoufek
Stephen Kritchevsky
Darren Kriticos
Aristeidis Kritis
Delane Kritsky
Jürgen Kriwet
David Krizaj
Igor Krizaj
Danny Krizanc
Andrea Kroeger
Kristy Kroeker
Alexander Kroemer
Jeremy Kroemer
Christopher Kroenke
Floor Kroese
Edeltraut Kroger
Björn Kröger
Anders Krogh
Sheila Krogh-Jespersen
Nicole Krogstrup
Andreas Kroh
Kenneth Krohn
Jona Krohn
Georg Krohne
Anja Kroke
Anita Krokosz
Dawid Krokowski
Jacek Krol
Wojciech Krol
Judith Kroll
J. Simon Kroll
Jens Kroll
Alexandra Kroll
Katja Kröller
Bastiaan Krom
Katrin Kromeyer-Hauschild
Jacco Kromkamp
Irving Kron
Annelies Kroneman
Marcus Kronforst
Ian Kronish
Frederieke Kroon
Lee Kroos
Ian Krop
Yury Kropotov
Matthew Krosch
Susan Krown
Daniela Kroy
Janet Krska
Nicolas Krucien
Luise Kruckenhauser
Max Krucoff
Andreas Krueger
Dirk Krueger
Robert Krueger
Richard Krueger
Joachim Krueger
Peter Krug
Sebastian Krug
Dominik Krug
Susanne Krug
Carsten Kruger
Renate Krüger
Andrey Kruglov
Willem Kruijer
Marieke Kruip
Boudewijn Kruithof
Joanna Kruk
Cheryl Krull
Markus Krumbholz
Eva Krumhuber
Ralf Krumkamp
Brian Krumm
Matthew F. Krummel
Claude Krummenacher
Edward Krupat
Tuane Krupek
Olga Krupkova
Alexander Krupnick

Danika Krupp
Michael Kruppa
Karsten Kruse
Johannes Kruse
Elizabeth Krusemark
Dean Krusienski
Peter Krustrup
Jens Kruth
L. E. B. Kruuk
Peter Kruyen
Frank Kruyt
Petar Kruzic
Peter Kruzliak
Konstantin Krychtiuk
Per Kryger
Sergey Krylov
Vera Krymskaya
Angelos-Miltiadis Krypotos
Andriy Kryshtafovych
Mark Krystal
Pierre Krystkowiak
Urszula Krzych
Krzysztof Krzysztofjankowski
Wojciech Krzyzanski
Wlodzimierz Krzyzosiak
Malgorzata Krzyzowska
Maik Kschischo
Daniel Ksepka
Chuan Ku
Nam Su Ku
Meng Kiat Kuah
Yung-Shu Kuan
Chee Sian Kuan
Chia-Yi Kuan
Rui Kuang
Yang Kuang
Shihuan Kuang
Yan-Ping Kuang
Fei Kuang
Da Kuang
Jialiang Kuang
Keiji Kuba
Kenji Kuba
Jurgen Kuball
Jan Kubanek
Laura Kubatko
Jan Kubecka
Karen Kubena
Paul Kubes
Bruno Kubiak
John Kubie
Janet Kübler
Andrea Kübler
Kirsten Kübler
Joanna Kubler-Kielb
Akiharu Kubo
Mugino Kubo
Yoshiaki Kubota
Shosei Kubota
Kengo Kubota
Hana Kubová
Michal Kucera
Ernest Kuchar
Adam Kucharski
George Kuchel
Melanie Kucherlapati
Sergei Kuchin
Lars Kuchinke
Suresh Kuchipudi
Karl Kuchler
Sarah Küchler
John Kuchtey
Rachel Kuchtey
Giorgi Kuchukhidze
Selim Kuci
Ulrich Kück
Ulrike Kuckelkorn
Can Kucuk
Aaron Kucyi
Krzysztof Kuczera
Srikanth Kudithipudi
Ola Kudlicka
Akira Kudo
Takashi Kudo
Makoto Kudo
Takayuki Kudoh
Greg Kudray
Anatoliy Kudryavtsev
Indira Kudva
Chin Siang Kue
Wolfgang Kuebler
Meta Kuehn
Christian Kuehn
Esther Kuehn
Christine Kuehner
Tobias Kuemmerle

Lars Kuepfer
Ralf Kueppers
Stefanie Kuerten
Wilfried Kues
Ursula Kües
Irina Kufareva
Thomas Kufer
Jeffrey Kugelman
Dimitris Kugiumtzis
Anton Kühberger
Sarah Kuhl
Michael Kühl
Angela Kuhla
Jens Kuhle
Brian Kuhlman
Levin Kuhlmann
Tanja Kuhlmann
Martin Kuhlmann
Valerie Kuhlmeier
Cynthia Kuhn
Peter Kuhn
Hans Kuhn
Michaela Kuhn
Louise Kuhn
Eric Kuhn
Gustav Kuhn
Liisa Kuhn
Annegret Kuhn
Randall Kuhn
Kristine Kuhn
Bernhard Kuhn
Pierre Kuhn
Simone Kühn
Jens-Peter Kühn
Susanne Kühn
Mary Kuhner
Harriet Kuhnlein
Jochen Kuhse
P. P. F. M. Kuijer
Hugo Kuijf
Bram Kuijper
Mette Kuijpers
Anne Marie Kuijpers-Jagtman
Andre Kuijsters
J. Kuiper
Allison Kuipers
M. Kuipers
Jan Albert Kuivenhoven
Praveen Kujal
Omar Kujan
Elizabeth Kujawinski
Anna Kukekova
Iwao Kukimoto
Toshio Kukita
Kalev Kuklane
Pawel Kuklik
Veerapol Kukongviriyapan
Maria Kukuruzinska
Anna Kula
Ipek Kulahci
Carolina Kulak
Dominik Kulakowski
Asli Kulane
Sanjeewa Kularatna
Suryawanshi Kulbhushansingh Ramesh
Alanna Kulchak Rahm
Franceli Kulcheski
Josipa Kules
Akos Kulik
Margarete Kulik
Alexander Kulikov
Jaime Kulisevsky
Janina Kulka
Hemant Kulkarni
Abhaya Kulkarni
Savita Kulkarni
Sandip Kulkarni
Ashok Kulkarni
Sakil Kulkarni
Ritwij Kulkarni
Nachiket Kulkarni
Skadi Kull
Ravina Kullar
Brian Kullin
F. Aura Kullmann
Jenni Kulmala
Anna Kulminskaya
Dagmar Kulms
Bernard Kulohoma
Aino Kulonen
Marjean Kulp
Wantanee Kulpeng
Ritu Kulshreshtha
Jens Kultima
Anahid Kulwicki
William Kulyk

Hye-Chung Kum
Yoshito Kumagai
Etsushi Kumagai
Kathleen Kuman
Vipin Kumar
Rashmi Kumar
Devender Kumar
Dhiraj Kumar
Vijay Kumar
Ashok Kumar
Sanjeev Kumar
Shailendra Kumar
Pradeep Kumar
Supriya Kumar
Binay Kumar
Satish Kumar
Arvind Kumar
Ranjeet Kumar
Addanki Kumar
Sushil Kumar
Sunil Kumar
Sanjay Kumar
Santosh Kumar
Rajesh Kumar
Sandeep Kumar
Vivek Kumar
Ranjeet Ranjan Kumar
Ramaiah Kumar
Aundy Kumar
Mukesh Kumar
Prashant Kumar
Dilip Kumar
Ujendra Kumar
Rajneesh Kumar
Divya Kumar
Dhruv Kumar
Pramod Kumar
Rahul Kumar
Shiva Kumar
Amit Kumar
Subodh Kumar
Ram Kumar
Virendra Kumar
Ashwani Kumar
Ravi Kumar
Manoj Kumar
Manish Kumar
Amar Kumar
N. K. Krishna Kumar
Devendra Kumar
Ashish Kumar
Vinod Kumar
Parameet Kumar
Ajay Kumar
Ashutosh Kumar
P. Kumar
Mayank Kumar
Vibhor Kumar
Bhavna Kumar
Viksit Kumar
Nitish Kumar
Maya M. Kumar
Dharmendra Kumar
Bimlesh Kumar
Nithin Kumar
Anil Kumar
Praveen Kumar
Kvs Hari Kumar
Himanshu Kumar
Rajnish Kumar
Jitendra Kumar
Dileep Kumar
Sachin Kumar
Santhosh Kumar
Smita Kumar
Sunny Kumar
Ganesh Kumar
Veerendra Kumar
Biplob Kumar Kumar Modak
Honnavalli Kumara
Dinakantha Kumararatne
N Kumarasamy
Vasanthapuram Kumaraswami
Rasadurai Kumaravel
Jyothi Kumari
Suman Kumaria
Hirokazu Kumazaki
Sangamesh Kumbar
Toshiaki Kume
Sirinart Kumfu
George Kumi
Abera Kumie
Jolanta Kumirska
Ursula Kummer
Terrance Kummer
Noelle Kumpel

Emily Kumpel
Tilo Kunath
Marija Kundakovic
Paolo Kunderfranco
Michael Kundi
Vikas Kundra
Martin Kundrát
Manikuntala Kundu
Gopal Kundu
Chanakya Kundu
Anjan Kundu
Siddhartha Kundu
Pallob Kundu
Tanja Kunej
Hsing-Jien Kung
Chun-Chia Kung
Johannes Kung
C. T. Kung
Michal Kunicki
Mark Kuniholm
Hiroshi Kunikata
Shiho Kunimatsu-Sanuki
Shinji Kuninaka
Jun Kunisawa
Ramkumar Kunka Mohanram
Barbara Kunkel
Ian Kunkler
Sreenivasan Kunnatheeri
Florian Kunneman
Muthusamy Kunnimalaiyaan
Ajaikumar Kunnumakkara
Atsushi Kuno
Florentina Kunseler
Anton Kunst
Thomas Kunt
Guruprasad Kuntamallappanavar
Krushnamegh Kunte
Uta Kunter
Jennifer Kuntz
Ludwig Kuntz
Luciana Kuntze
Hiroshi Kunugi
Setor Kunutsor
Wolfram Kunz
Michael Kunze
Carol Kunzel
Arthur Kuo
Yu-Min Kuo
Shiu-Ming Kuo
Pao-Lin Kuo
Hsiao-Che Kuo
Sung-Hsin Kuo
Hann-Chorng Kuo
Caroline Kuo
Yung-Ting Kuo
Chang-Fu Kuo
John Kuo
Ching-Chuan Kuo
Li-Wei Kuo
Chia-Lam Kuo
Ya-Wen Kuo
Chia-Tung Kuo
Yu-Hung Kuo
I Fan Kuo
Yuan-Hung Kuo
Chin-Chi Kuo
Soong-Yu Kuo
Chih-Horng Kuo
Chao-Yang Kuo
Denise It Kuok
Christian Kupatt
Juozas Kupcinskas
Anne Kupczok
Maciek Kupczyk
Nuriye Kupeli
Hannah Kuper
Marcelo Kuperman
Ron Kupers
Silke Kuphal
Peter Kuppens
Toon Kuppens
Hendrik Küpper
Dhandapani Kuppuswamy
Dmitry Kuprash
Zoya Kurago
Lin Kurahara
Hiroki Kurahashi
Eiko Kuramae
Alexandr Kuranov
Kesava Rao Kurapati
Tetsuya Kurata
Yasutaka Kurata
Hirohiko Kuratsune
Mazen Kurban
Emila Kurbasic
Ekaterina Kurbatova

Siavash Kurdistani
Paul Kurdyak
Nagomi Kurebayashi
Joshua Kurek
Cemil Kurekci
Jeff Kuret
Reet Kurg
Boris Kurganov
Moni Kuriakose
Hidemi Kurihara
Gregorij Kurillo
Takeshi Kurita
Kenichi Kuriyama
Carolyn Kurle
Rainer Kurmayer
Irma Kurniawan
Nyoman Kurniawan
Hirotsugu Kurobe
Igor Kurochkin
Junya Kuroda
Makoto Kuroda
Marcelo Kuroda
Satoshi Kuroda
Kenichi Kuroda
Takeshi Kuroha
Mineo Kurokawa
Manabu Kurokawa
Kenji Kurokawa
Keiichi Kuroki
Makoto Kuro-O
Hitoshi Kurose
M Kurosu
Dorota Kurowicka
Mariola Kurowska-Stolarska
Shekar Kurpad
Peter Kurre
Christine Kurschat
Wolfram Kürschner
Florian Kurschus
Petri Kursula
Timothy Kurt
Birguel Kurt
Dilber Kurtboke
Vartan Kurtcuoglu
Andreas Kurth
Thomas Kurth
Christian Kurts
Bruce Kurtz
Armin Kurtz
Isaac Kurtzer
Cleutus Kurtzman
Shiro Kurusu
Ralf Kurvers
Tagried Kurwie
Patrick Küry
Kim Kurz
Mateusz Kurzawski
Teymuras Kurzchalia
Yuko Kusakabe
Yoichiro Kusakari
Souvik Kusari
Marion Kusche-Gullberg
Kristen Küsel
Toshihiro Kushibiki
Akifumi Kushiyama
Sidney Kushner
Mark Kushner
Elena Kushnerenko
Vitaly Kushnir
Sapana Kushwaha
Cheryl Kuske
Claudia Kusmic
Piotr Kusnierczyk
Oliver Kuss
Niels Kuster
Diederik Kuster
Adam Kustka
Sentaro Kusuhara
Shigeru Kusumoto
Masami Kusunoki
Szilvia Kusza
Ruth Kutalek
Zoltán Kutalik
Jason Kutch
Guruharsha Kuthethur Gururaj
Joseph Kuti
Anton Kutikhin
Sebla Kutluay
Bryan Kutner
Olaf Kutsch
Verena Kutschera
Joseph Kutzin
Ines Kutzner
Antti Kuuliala
Satu Kuure
Hitoshi Kuwabara

Hiroyuki Kuwahara
Atsukazu Kuwahara
Mieko Kuwahara
Ichiro Kuwahira
Tetsuo Kuwamura
Kazuo Kuwata
Jane Kuypers
Maria Kuyukina
Mehmet Kuyumcu
Muge Kuyumcu-Martinez
Craig Kuziemsky
Ivan Kuzmin
Konstantin Kuzmin
Ml Kuzmina
Alexey Kuznetsov
Nikita Kuznetsov
Andreas Kuznik
Kiyotaka Kuzushima
Marc Kvansakul
Tarja Kvist
Libor Kvitek
S. Kvolik
Andrea Kwa
Hau Kwaan
Bernard Kwabi-Addo
Kyubum Kwack
Sang-Soo Kwak
Su-Hwan Kwak
Linda Kwakkenbos
Johnny Kwan
Jennifer Kwan
My Kwan
Paul Wing Hing Kwan
Letty Kwan
Mei-Po Kwan
Grazyna Kwapiszewska
Douglas Kwazneski Ii
Robert Kwee
R. Kwekkeboom
David Kwiatkowski
Adam Kwiatkowski
Iwona Kwiecien
Marcin Kwissa
Henry Kwok
John Kwok
Jessica Kwok
Man Ki Kwok
Wai-Meng Kwok
Hang Fai Kwok
Young Kwok
Wingchi Kwok
Seong Keun Kwon
Hyug Moo Kwon
Sunghoon Kwon
Ja-Young Kwon
Seung-Hwan Kwon
Sohee Kwon
Hanna Kwon
Youngeun Kwon
Soonjo Kwon
Dongjin Kwon
Nayoung Kwon
Miyoung Kwon
Hyuk-Moo Kwon
Kyung Kwon-Chung
Jeffrey Kwong
Yok Lam Kwong
Jacky Kwong
Joseph Kwong
Tin Kyaw
Jennelle Kyd
Julia Kydd
Sunghyon Kyeong
Hana Kyjonkova
Leena Kylanpaa
Jennifer Kyle
Christopher Kyle
Anneli Kylliainen
Tina Kyndt
Peter M. Kyne
Makoto Kyougoku
Kypros Kypri
Charalambos Kyriacou
Kyriacos Kyriacou
Constantinos Kyriakis
Marinos Kyriakopoulos
Stavroula Kyriazi
Athanassios Kyritsis
Ioannis Kyrou
Vitaliy Kyryk
David Kysela
Pavel Kyslik
Vasileios Kyttaris
Anastasia Kyvelidou
Karol Kyziol
Giancarlo La Camera

Anne Camille La Flamme
Susanne La Fleur
Nicole La Gruta
Renaud La Joie
Gaetano La Manna
Silvia La Monica
Concettina La Motta
Vincenzo La Mura
Caterina La Porta
Giampiero La Rocca
Cristian La Rocca
Carmelo La Rosa
Jerome J. La Rosa
Camilo La Rota
Maria Teresa La Rovere
Mauro La Russa
Sophie La Salle
Valentina La Scaleia
Bernard La Scola
Giuseppe La Torre
Jeff Laake
Johanna Laakkonen
Maarit Laaksonen
Suman Laal
Maris Laan
Anne Laarman
Tiina Laatikainen
Tracey-Lea Laba
Joshua Labaer
Conrad Labandeira
Michael Labarbera
Daniel Labarbera
Mark Labarge
Angelo Labate
Joanne Labate
Vincent Labatut
Maurizio Labbate
Antoine Labbé
Sid Labdi
Thomas Labean
Bernard Labedan
Siegfried Labeit
Antje Labes
Luc Labey
Verena Labi
Karim Labib
Jim Labisko
Jessica Labonte
Christa Labouliere
Nathalie Labrecque
Fernand Labrie
Fotini Labropulu
Karien Labuschagne
Justyna Labuz
Krzysztof Labuzek
Isabel Lacau-Mengido
Norman Lacayo
A. C. R. Lacerda
Giuseppina Lacerra
Odilia Laceulle
Simon Lacey
Rebecca Lacey
Cameron Lacey
Melanie Lacey
Joseph Lachance
Gérard Lachapelle
Myriam Lacharité
Jean-Paul Lachaud
Christophe Lachaud
David Lacher
Thomas Lacher
Salil Lachke
Anusha Lachman
Herbert Lachman
Bernd Lachmann
Robin Lachmann
Nathan Lachowsky
Joël Lachuer
Lubica Lacinova
John Lackie
Christian Lackinger
Helmut Lackner
Michaela Lackner
Martin Laclaustra
Benoit Lacombe
Veronique Lacombe
Sandrine Lacombe
Ezio Laconi
H. Daniel Lacorazza
Deanna Lacoste
Thomas Lacour
Stephan Lacour
Rodney Lacret
Ludovic Lacroix
Romaric Lacroix
Nathan Lacross

María Lacruz
Paige Lacy
Dean Lacy
Mary Lacy
Hania Lada
Lara Ladage
Daniel Ladant
Elissa Ladd
Bob Ladd
Simone Ladeia-Andrade
Nimzing Ladep
Raj Ladher
Friedrich Ladich
Ana Ladio
John Ladisa
Daniel Ladley
Daniela Ladner
Joël Ladner
Anna Ladogana
Michael Ladomery
Alain Laederach
Cédric Laedermann
Theodore Laetsch
Oliver Laeyendecker
Xavier Lafarge
Giuseppe Lafauci
Pierre Lafaye
Harry Lafeber
Shawn Laffan
John Laffey
Luke Laffin
Michael Lafleur
Marni Lafleur
Benoit Lafleur
Mickael Lafond
Francois Lafond
Jerome Lafont
Rene Lafont
Eric Lafontaine
Jeffrey Lafranca
Audrey Lafrenaye
Anunciacion Lafuente
William Lafuse
Robert Lafyatis
Patricia Lagadec
Neil Lagali
Marina Laganaro
Bernard Lagane
Frederic Lagarce
Michel Lagarde
Alfonso Lagares
Guillaume Lagarrigues
Eric Lagasse
Ricardo Lage
Laurel Lagenaur
Vincent Lagente
Andrea Lagerche
Nina Lagerqvist
Martin Lagging
Fiorenzo Laghi
Jean-Christophe Lagier
Malgorzata Lagisz
Joao Lago
David Lagomasino
Carlos Lago-Pena
Dimitris Lagos
Francisco Lagos
Nelson A. Lagos
Doris Lagos-Kutz
Clément Lagrue
Tara Lagu
Michael Lague
Mariano Laguna
Pablo Laguna
Beatriz Lagunas
Yael Lahav
Christelle Lahaya
Ismail Laher
Timothy Lahey
Debrupa Lahiri
B. Lahoz
Kaarina Lähteenmäki
Leo Lahti
Jouni Lahti
Ming-Zong Lai
Luhua Lai
Eric Lai
Ren Lai
Zhi-Chun Lai
Wai-Lung Lai
Hsin-Chih Lai
Meng-Chuan Lai
Ming-Derg Lai
Zhongxiong Lai
Liang-Chuan Lai
Carlo Lai

Chao-Lun Lai
Timothy Lai
Alessia Lai
Yuping Lai
Xin Lai
Jiang-Shan Lai
Keane Lai
Henry Lai
Wf Lai
Chih-Cheng Lai
Ching-Shu Lai
Taavi Lai
Olimpia Lai
Zhibing Lai
Maoyi Lai
Jinping Lai
Zhongping Lai
Sarah Lai
Victor Lai
Kuan-Lin Lai
Hong-Chang Lai
Darong Lai
Lanxiu Lai
Huichuan Lai
S. C. Lai
Yukun Lai
Hung-Wen Lai
Fangnong Lai
Nai Ming Lai
Ming-Chih Lai
Yongxiu Lai
Wen-Fu Lai
Jonathan Lai
Wei Lai
Ya-Yun Lai
Ching Tat Lai
Rafael Laia
Evagelia Laiakis
Anita Laidlaw
Rebekah Laidsaar-Powell
Christian Laier
Markus Laimer
Margit Laimer
Anna Laine
Christopher Laine
Ken Laing
Nigel Laing
Ines Lains
Paola Laiolo
Michael Laiosa
Diana Laird
Brian Laird
Ite Laird-Offringa
Kirsi Laitinen
Dennis Lajeunesse
Patrick Lajoie
Jasna Lajtner
Itziar Laka
Melike Lakadamyali
Peter Lakatos
Seema Lakdawala
Jessica Lake
Douglas Lake
Robin Lake
Spencer Lake
Daniël Lakens
Kimberley Lakes
Nelly Lakestani
Yihunie Lakew
Laila Lakhal
Samira Lakhal-Littleton
Shaheen Lakhan
Akhlesh Lakhtakia
Milena Lakicevic
Bhaskar Lakkakula
Aparna Lakkaraju
Sirish Lakkaraju
Taras Lakoba
Jeffrey Lakritz
Peter Laks
Madepalli Lakshmana
Lakshminarayanan Lakshmanan
Imayavaramban Lakshmanan
Prakash Lakshmanan
P. V. Lakshmi
Shanthana Lakshmi
Sribalaji Lakshmikanthan
Kamakshi Lakshminarayan
Polavarapu Lakshminarayana
Satyan Lakshminrusimha
Uma Lakshmipathy
Sunil Lal
Rup Lal
Ratnesh Lal
Girdhari Lal
Shruti Lal

Ashutosh Lal
Peeyush Lala
Marc Lalande
Wim Laleman
Alex Laliberte
Prajna Lalitha
Marco Lalle
Jean-Paul Lalles
Enzo Lalli
Anand Lalli
David Lalush
Gaurav Lalwani
Ching-Wan Lam
Kong-Peng Lam
Brian Lam
Tak-Wah Lam
Tommy Tsan-Yuk Lam
Ernest Lam
Tommy Lam
Kin Bong Hubert Lam
Tania Lam
Wilbur Lam
Fong Lam
Benjamin Lam
Lap Po Lam
Hanh Lam
Tzeng Yih Lam
Catherine Lam
Shu Lam
Yukyan Lam
Yin Lam
Connie Lam
Thomas Lam
Daniel Lam
Sharon Lam
Phyllis Lam
Larry Lam
Amrita Lama
Theresa Lamagni
Gabriela Lamarca
B. Lamarca
Benoit Lamarche
Miles Lamare
Fotini Lamari
Trond Lamark
Laurent Lamarque
Alain Lamarre
Jerome Lamartine
José Ramón Lamas
J. C. Lamattina
Fabien Lamaze
Ned Lamb
Matthew Lamb
David Lamb
Janine Lamb
Doriano Lamba
Cornelis Lambalk
Xavier Lamballerie
Barrot Lambdin
Fernand Lambein
Anne-Marie Lambeir
James Lambers
Olivier Lambert
Patricia Lambert
Amy Lambert
Christian Lambert
David Lambert
Allison Lambert
Lukas Lambert
Nevin Lambert
Walter Lambert
W. Lambert
Sylviane Lambert
Patrizia Lamberti
Luca Lambertini
Regis Lamberts
Kate Lambertsen
Christian Lambertz
Mark Lambie
S.M. Lambies
Philippe Lambin
Eric Lambin
Renaud Lambiotte
Bert Lambooij
Sn Lambova
Julia Lambret-Frotte
Petar Lambrev
Christopher Lambrides
John Lambrinos
Maryou Lambros
Adriano Lameira
Marcelo Lamers
Claudiana Lameu
Tina Lamey
Jay Ram Lamichhane
Rajan Lamichhane

Claudia Lamina
Todd Lamitina
Mohamed Lamkanfi
Bouchaib Lamkhioued
Kathleen Lamkin-Kennard
Marilyn Lamm
Joris Lammers
Wim Lammers
Frank Lammert
Mikko Lammi
Patrick Lammie
Johanna Lammintakanen
Severine Lamon
Thomas Lamonerie
Iain Lamont
Richard Lamont
Richard Lamontagne
Elizabeth Lamos
Claudine Lamoth
Frederic Lamoth
Valerie Lamour
Francois Lamoureux
Ecosse Lamoureux
Guillaume Lamoureux
Angelika Lampert
Kirsten Lampi
Kathryn Lamping
Amit Lampit
Peter Lamptey
James Lamsdell
Daryl Lamson
Rosa Lamuela-Raventós
Thomas Lamy
Pierre-Jean Lamy
François Lamy
Ruiting Lan
Chung-Yu Lan
Weizhong Lan
Tsuo-Hung Lan
Xiqian Lan
Ganhui Lan
Feng Lan
Li Lan
Xianyong Lan
Guo-Cheng Lan
Yeqing Lan
Zhou Lan
Ping Lan
Ke Lan
Shiwei Lan
Susan Lana
David Lanar
Marcello Lanari
Claudio Lanata
Jack Lancaster
Steven Lancaster
Jarrett Lancaster
Phillip Lancaster
Stacey Lance
Brent Lance
Steve Lancel
Antonio Lancha
Tiziana Lanciano
Hovirag Lancioni
Rosalba Lanciotti
Ellen Lanckacker
Kenneth Land
Blanca Landa
Igor Landais
Aimee Landar
Barbara Landau
Ruth Landau
Yan Landau
Göran Landberg
John Landefeld
Melissa Landell
Merrill Landers
Scott Landfear
Matthias Landgraf
Rainer Landgraf
Dominic Landgraf
Carl Landhuis
Marco Landi
Paolo Landini
Maria Landini
Kerry Landman
Bennett Landman
Jaime Landman
Neil Landman
Joseph Landolph
Luca Landoni
Karl Landorf
Gary Landreth
Jean-Francois Landrier
Matteo Landriscina
Joseph Landry

Oriane Landry
Guillaume Landry
David Landsberger
Johannes Landsheer
Markus Landthaler
Bart Landuyt
Tim Lane
Richard Lane
Scott Lane
Andrew Lane
Steven Lane
Lydie Lane
James Lane
Shelly Lane
Brian Lane
Jerome Lane
Alison Lane
Gerolamo Lanfranchi
Luisa Lanfranco
Francesca Lanfranconi
Laurence Lanfumey
James Lang
Jochen Lang
Jenna Lang
Elmar Lang
Christine Lang
Britta Lang
Shelley Lang
Irene Lang
Andrew Lang
Sven Lang
Haoxiang Lang
Roland Lang
Elke Lang
Carol Lang
Ken Lang
Di Lang
Rongling Lang
Z. Lang
Taimour Y. Langaee
Øystein Langangen
Ellen Langballe
Jessica Langbaum
Jan Langbein
Jane Langdale
Reinhard Lange
Stephan Lange
Anke Lange
Lene Lange
Denise Lange
Dirk Lange
Priscila Lange
Holger Lange
Philippe Langella
Terence Langendoen
Harald Langer
Daniel Langer
Martin Langer
Nicolas Langer
Gero Langer
Oliver Langer
Susanne Langer
Annette Langer-Gould
Jan Langeveld
Helene Langevin
Scott Langevin
Christelle Langevin
Dianne Langford
Terry Langford
Berthold Langguth
Jakub Langhammer
Sigrid Langhans
Simone Langhans
Lisa Langhaug
Jean Langhorne
Peter Langhorne
Sabine Langie
Morgan Langille
Kathryn Langin
Tracy Langkilde
Philip Langlais
Ricky Langley
Chris Langley
Tessa Langley
Marc-André Langlois
Ryan Langlois
Craig B. Langman
Anika Langmann
Benjamin Langmead
Soenke Langner
Tanja Langsenlehner
Helge Langseth
Lisa Langsetmo
Gordon Langsley
Gernot Längst
Catherine Langtimm

Michele Lanham
Aditya Lankapalli
Kamran Lankarani
Brigitte Lankat-Buttgereit
Mareike Lankeit
Christa Lankes
Kaisu Lankinen
Pauliina Lankinen
Alexander Lankowski
Gisela Lannig
M. G. Lansberg
Peter Lansdorp
Van Lansingh
Joseph Lanska
Beate Lanske
Amy Lansky
Daniele Lantagne
Paul Lantos
Olivier Lantz
Laura Lantz
Sasha Lanyon
Rainer Lanz
Tobias Lanz
Bruno Lanz
Robert Lanza
Gaetano Lanza
Stefano Lanzi
Tatiana Lanzieri
Vanessa Lanziotti
Humberto Lanz-Mendoza
Terence Tzu-Hsi Lao
Kaiqin Lao
Oscar Lao
Malinee Laopaiboon
Olga Laosa
Dhafer Laouini
Olav Lapaire
Rosa Lapalombella
Luigi Lapalorcia
Luis Lapao
Valero Laparra
Paul Lapchak
Bertrand Lapergue
Liron Lapid
R. Lapid-Gortzak
Kyle Lapidus
Pascal Lapierre
Louis Lapierre
Hélène Lapillonne
Brittany Lapin
Stephen Lapinsky
Alexei Lapkin
Diego Laplagne
David Laplaud
Laurent Laplaze
Scott Lapoint
Réjean Lapointe
Gisèle Lapointe
Stephen Lapointe
Jacques Lapointe
Lauren Lapointe-Shaw
Rosaria Laporta
Stephane Laporte
Jyrki T Lappalainen
Rosamaria Lappano
Martha Lappas
Therese Lapperre
Otto Lappi
Terence Lappin
Joseph Lappin
Maria Lara
Ryan Laranger
Marta Laranjo
Muriel Larauche
Francisco Lara-Valencia
Thibaut Larcher
Michael Lardelli
David Largaespada
Mathew Large
Edward Large
Roddy Large
Etienne Larger
Raquel Largo
Miguel Larguinho
Dan Larhammar
Martina Lari
Vincent Larivière
Markku Larjavaara
Anthony Larkum
Jan Larmann
Petra Larmo
Maarten Larmuseau
S. Larney
Luigi Larocca
Daniel Laroche
Karine Laroucau

Mounir Laroussi
Amanda Larracuente
Ricardo Larrainzar
Adriana Larregina
Lionel Larribere
Gérald Larrouy-Maumus
Lesli Larsen
Peter Larsen
Jesper Larsen
Fin Stolze Larsen
Melinda Larsen
Ray Larsen
David Larsen
Mogens Larsen
Anders Larsen
Randy Larsen
Klaus Larsen
Magdalena Larska
Steven Larson
Timothy Larson
Erik Larson
Charles Larson
Bruce Larson
Andrew Larson
Derek Larson
Steve Larson
Nicholas Larson
Shawn Larson
Eric Larson
Amy Larson
Michael Larson
Ray Larson
Anders Larsson
Marie Larsson
Hans Larsson
Elin Larsson
Maria E. H. Larsson
Per-Göran Larsson
Michelle Larue
Amanda Larue
Bobby Larue
Tiziana Larussa
Nicholas Larusso
Carlota Las Hayas
Iñigo Lasa
Roberta Lasagna
Laura Lasagni
John Lasalle
Nadia Lascar
Kara Lascola
Kayla Laserson
Ahmed Lasfar
Lawrence Lash
Kameran Lashkari
Terra Lasho
Gordana Laskarin
Tamás Laskay
Howard Lasker
Paul Lasko
Tomasz Laskus
Jesse Lasky
Jessica Lasky-Su
Stephen Lasley
Edwin Lasonder
Cornelia Lass- Flörl
Jurgen Lassak
Alvaro Lassaletta
Amandine Lassalle
Andrew Lassar
William Lassek
Cecilia Lässer
Zohra Lassi
G. Daniel Lassiter
Hans Lassmann
Robert Last
Scott Laster
Isabel Lastres-Becker
Vieri Lastrucci
Zoltán László
Charu Lata
Matthieu Latapy
Maria Ujue Latasa
Jean-Paul Latge
Keith Latham
Justin Lathia
Richard Lathrop
Kira Lathrop
Sarah Lathrop
Quresh Latif
Bruce Latimer
Roberto Latini
Guillaume Latombe
Jorne Laton
Irene Latorre
Ramon Latorre
Antonio Latorre

Paulina Latos
Agnieszka Latosinska
Lawrence Latour
Drew Latta
Pallavi Latthe
Samantha Lattof
Antti Latvala
Susanna Kp Lau
Lester Lau
Eric Lau
Bryan Lau
Yun-Fai Chris Lau
Gee Lau
Christopher Lau
Chantal Lau
Loretta Lau
Wayne Bond Lau
Wei Ling Lau
Terrence Chi Kong Lau
Min-Yu Lau
John Lau
William Lau
Goeff Lau
Siong Hoe Lau
Boris Lau
Hang Lau
George Kk Lau
Calvin Ho-Fung Lau
Adeline Lau
Julia Laube
Christian Lauber
Daniel Laubitz
Romy Lauche
Michael Lauck
Agne Laucyte
Luca Laudani
Tsai-Ling Lauderdale
Vincent Laudet
Stephen Lauer
Miriam Laufer
Yocheved Laufer
Helmut Laufs
Kathryn Laughon
Charles Laughton
Charles Lauhon
Christina Laukaitis
Kris Laukens
Petri Laukka
Cornelia Laule
Raúl Laumann
Odile Launay
Elise Launay
Jacques Launay
Cristian Launes
Christine Lauren
Ted Laurence
Casini Laurence
Arian Laurence
Cato Laurencin
Louise Laurent
Gilles Laurent
Carine Laurent
Fabrice Laurent
Terradot Laurent
Sarah Laurent
Jose Laurent
Marcia Laurenti
Sandra Laurentino
Fulvio Lauretani
Valentina Lauria
Massimiliano Lauria
Karen Laurie
Nikia Laurie
Andrew Laurie
Michel Laurin
Gaia Vaglio Laurin
Kristin Laurin
Josef Laurincik
Mattia Lauriola
Marco Lauriola
Chiara Lauritano
Lotte Lauritzen
Davide Lauro
Brett Laursen
Kelly Laurson
José Laus
Berthold Lausen
Ludwig Lausser
Stefan Lautenbacher
Tim Lautenschlaeger
Ingmar Lautenschläger
Nick Lauter
Matthias Lauth
Sylvie Lautru
Dale Lauver
Bernard Lauwerys

Sebastiano Lava
Raul Lavado
Michael Lavagnino
Marc Lavaleye
Christina Lavallee
Jean Lavallee
Ewa Lavant
Shahram Lavasani
Francesca Lavatelli
Catharina Lavebratt
Mary Lavelle
Donald Lavelle
Aonghus Lavelle
Garry Laverty
James Lavery
Patrizia Lavia
Hugo Laviada
Fabrice Lavial
Alessandro Laviano
Carl Lavie
Eric Lavigne
Esteban Lavilla
Maurice Laville
Matt Lavin
Laura Lavine
Joel Lavinsky
Luigi Laviola
Victor Lavis
Jean-Pierre Lavoie
Julie Lavoie
Martin Lavoie
Anne-Violette Lavoir
E. Lavonas
Alfonso Lavorgna
Olga Lavrik
Inna Lavrik
Sergey Lavrov
Alexander Lavrov
Paul Lavy
Michael Law
Mansun Law
Brian Law
Yuen Kwan Law
Tsun Yee Law
Sean Law
Kara Law
Timothy Lawes
Michael Lawes
Sara Lawhon
Joshua Lawler
Sean Lawler
Blair Lawley
Linette Lawlor-Savage
Nicholas Lawn
Daniel Lawrence
Rachel Lawrence
Ryan Lawrence
Christopher Lawrence
Andrew Lawrence
B. Paige Lawrence
Claire Lawrence
Da Lawrence
David Lawrence
Krakoff Lawrence
Scott Lawrence
Kate Lawrenson
Nathan Lawrentschuk
Matthew Lawrenz
Evelyn Lawrenz
James Lawson
Sally Lawson
Becki Lawson
William Lawson
Paul Lawson
Jamie Lawson
Gregory Lawson
Michelle Lawson
Patricia Lawston
Simon Lax
Antonio Lax
D. Ross Laybutt
Ian Laycock
Gwenael Layec
Lester Layfield
Georg Layher
Laura Layland
Wanda Layman
Jack Layne
Charles Layne
Pierre Layrolle
Nicole Lazar
Cassandre Lazar
Alpar Lazar
Diane Lazard
Amparo Lazaro
Luisa Lázaro

Michael Lazarou
Eric Lazartigues
Jeffrey Lazarus
Helen Lazear
James Lazenby
John Lazo
Alejandro Lazo-Langner
Antigone Lazou
Maïna L'Azou
Eric Lazo-Wasem
Ahmed Lazrak
Lambros Lazuras
Claudio Lazzari
Lorenza Lazzari
Chiara Lazzari
Antonio Lazzarino
Giuseppe Lazzarino
Douglas Lazzaro
Chiara Lazzeri
Eros Lazzerini Denchi
Yun Le
Dung Le
Jie Le
Cheng Foh Le
Anh Le
Vien Le
Minh Le
Phuc Le
Trung Le
Phong Le
Isabelle Le Ber
Bernard Le Bonniec
Jacques Le Bot
Eric Le Bourg
Arnault Le Bris
Kim-Anh Lê Cao
David Le Couteur
Yann Le Cunff
Kirsty Le Doare
Julie Le Faouder
Joost Le Feber
Nathalie Le Floch
Séverine Le Gac
Sylvain Le Gall
Marion Le Gall
Maude Le Gall
Lionel Le Gallic
Gaëlle Le Goff
Ronan Le Goffic
Renaud Le Goix
Fabienne Le Guyadec
Simon Le Hello
Jacques Le Houezec
Cecile Le Lann
Yves Le Loir
Gwendal Le Masson
Olivier Le Meur
Yann Le Meur
Jean-Claude Le Mevel
Alain Le Moine
Vincent Le Moing
Hervé Le Moual
Gilles Le Moullac
Cécile Le Péchoux
Jacques Le Pendu
Bruno Le Pioufle
Marie-Frédérique Le Potier
Karine Le Roch
Virginie Le Rolle
Carel Le Roux
Bruno Le Ru
Elisabeth Le Rumeur
Francois Le Tacon
Paul Le Tissier
Maryline Le Vaillant
Michel Le Van Quyen
Stephen Lea
Marzia Leacche
Katie Leach
Liana Leach
Corinne Leach
Elizabeth Leadbetter
Jim Leafloor
Morgan Leah
Patricia Leahy-Warren
Andrew Leake
Élcio Leal
Fabio Leal
Laura Leal
Luciana Leal
Ernesto Cesar Leal-Junior
Douglas Leaman
Karin Leander
Maria Leandro
Paula Leandro
Sylvia Leão

Pedro Leão
Gavin Lear
Juan Leardi
Alberto Leardini
Peter Learn
Christopher Leary
Peter Leary
Andrew Leask
Richard Leask
Simon Leather
David Leavens
Silas Leavesley
Patrick Leavey
Camille Lebarbenchon
David Lebeaux
Mikhail Lebedev
Alexander Lebedev
Irina Lebedeva
Alexandre Lebel
Etienne Lebel
Sarah Lebel
Michael Lebens
Paul Leberg
Gerald Leblanc
Roger M. Leblanc
Stephen Leblanc
Allana Leblanc
Normand Leblanc
Paul Leblanc
Binnaz Leblebicioglu
Agnès Leblond
Jared Leboldus
Pierre Lebon
Vincent Lebot
Christophe Leboulanger
Mario Lebrato
Francois Lebreton
Mael Lebreton
Benoit Lebreton
Carlito Lebrilla
David Lebrun
Gretchen Lebuhn
Fernando Lecanda
Edouard Lecarpentier
Thibault Lecarpentier
David Lecchini
Jean Michel Lecerf
Christophe Lechauve
James Lechleiter
Terry Lechler
Walter Lechner
Beatrice Lechner
Andreas Lechner
Dorota Lechniak
Karl Lechtreck
Daniele Lecis
Mary Leck
Deborah Leckband
Fabrice Leclerc
Estelle Leclerc
Stephanie Leclerc-Mercier
Isabelle Leclercq
Alexandre Leclercq
Tamara Leclercq
Lucas Leclère
Virginie Lecomte
Frédéric Lecouvet
Johna Leddy
Kristien Ledeganck
Helmut Leder
Michael Lederman
Ruben Ledesma
Elizabeth Ledgerwood
Johan Ledin
Jonathan Ledoux
Magalie Leduc
Veronika Ledvenyiova-Farkasova
Yong Lee
Myung-Shik Lee
Eun-Jig Lee
Phyllis Lee
Patty Lee
Dong Gun Lee
Ji-Hyun Lee
Ki-Young Lee
Benhur Lee
Sung-Jae Lee
Myeong Soo Lee
Sheng-An Lee
Peter Lee
Shin-Seok Lee
Bok-Luel Lee
Jooyoung Lee
Shyh-Jye Lee
Wen-Chung Lee
Junghee Lee

Luke Lee
Chia Lee
Vincent Lee
I-Min Lee
Yoosook Lee
Charles Lee
Chang-Won Lee
Duk-Hee Lee
Dong-Yup Lee
Yin-Won Lee
Kong-Joo Lee
Sangyeoup Lee
Young Ho Lee
Yun-Shien Lee
Kyung-Ah Lee
Wei-Lih Lee
Seung-Jae Lee
Cheng-Chung Lee
Huei Lee
Steve Lee
Na Lee
Juliet Lee
Soo Chan Lee
Oscar Lee
Chul-Ho Lee
Jinhee Lee
Seung Hun Lee
Song Lee
Po-Shun Lee
Jeeyun Lee
Heung-Man Lee
Hyo Jeong Lee
Min Lee
Beth Lee
Won-Woo Lee
Kyungwon Lee
Gyoung-Ah Lee
Suzee Lee
Sang-Rae Lee
Wen-Ying Lee
Yi-Chung Lee
Hsuan-Shu Lee
Dean Lee
Jungkwan Lee
Cheng Lee
Choogon Lee
Youngsook Lee
Richard Lee
Cheolju Lee
Margie Lee
Sun-Gu Lee
Ching-Chih Lee
Alan Yueh-Luen Lee
Chih-Hsin Lee
Jeffrey Lee
Yong Yi Lee
Choong Hwan Lee
Yong-Ho Lee
Jong Min Lee
Jong Hoon Lee
Li-Ang Lee
Hee Jae Lee
Inyoul Lee
Sung Chul Lee
Jin-Ching Lee
Sang Jin Lee
Yunjong Lee
Sung Kuk Lee
Jae-Kyung Lee
Yi-Chia Lee
Kichoon Lee
Jung Pyo Lee
Jih-Hsiang Lee
Jae W. Lee
Tienwen Lee
Ho-Young Lee
Jae Lee
Mei-Hsuan Lee
Robert Lee
Tzong-Shyuan Lee
Hon-Cheung Lee
Sang-Myeong Lee
Fa-Yauh Lee
Gabsang Lee
Sangkyu Lee
Kwang Ho Lee
Mee-Young Lee
Albert Lee
Wonchul Lee
R. K. Lee
Hey-Kyoung Lee
Hak Kyo Lee
Sangheun Lee
Yie Hou Lee
Spike Lee
Kyoung Lee

Eun Ji Lee
Jeong-Hoon Lee
Jongmin Lee
Kyunghwa Lee
Chia-Hwa Lee
Junsoo Lee
Tsung-Han Lee
Hyun Soon Lee
Yoonkwang Lee
Ming-Ching Lee
Paul Lee
Jang-Ming Lee
Dongho Lee
Barry Lee
Eun Kyung Lee
Guinevere Lee
Sang Lee
Sanghoon Lee
Wan-Ping Lee
Hye Seung Lee
Hee Joo Lee
Guan-Chiun Lee
Jung Ryeol Lee
Wing-Kee Lee
Seunggeun Lee
Darren Lee
Seung-Hwa Lee
Do Yup Lee
Joo-Yong Lee
Nam Lee
Hye Won Lee
Terence Lee
Royce Lee
Rebekka Lee
Sang-Hee Lee
Ray Lee
Wei Lin Lee
Robyn Lee
Mi-Jung Lee
Teng-Yu Lee
Wu-Jung Lee
Sachiko Lee
Chang-Muk Lee
Mi Kyeong Lee
Sang Ho Lee
Patrick Lee
Zarraz Lee
Esther Lee
Ting-Yim Lee
Kyung-Min Lee
Changhan Lee
Kwan Hyi Lee
Seok-Yong Lee
S. Lee
Victor Ho-Fun Lee
Tai-Chi Lee
Ilsoon Lee
Jooa Julia Lee
Jun Hee Lee
Eunjoo Lee
Hamilton Lee
Kyeongjun Lee
Ellen Lee
Choonsik Lee
I-Cheng Lee
Ruey-Hua Lee
Benjamin Lee
Po-Hsien Lee
Jung Eun Lee
Elizabeth Lee
Seung-Ju Lee
Hyunhwa Lee
Myung Ah Lee
Jing-Huei Lee
Heon-Jin Lee
Jaetae Lee
Hwa Lee
Tsong-Hai Lee
Hyung Keun Lee
Peilin Lee
Chanhui Lee
Joon-Ho Lee
Sang-Won Lee
Janette Lee
Hye Yeon Lee
Hye Shin Lee
Yeonkyeong Lee
Chu-I Lee
Kang Dae Lee
Michael Lee
Sang Yeul Lee
Jungmin Lee
P. Lee
Ni-Chung Lee
Min Young Lee
Soo-Jeong Lee

Chii-Ming Lee
Nora Lee
Juhwan Lee
Yueh Lee
De-Hyung Lee
Lui Shiong Lee
Jae-Hyung Lee
Sungbok Lee
Adam Lee
Winson Lee
Stephen Lee
Howon Lee
Christopher Lee
Hyun-Sung Lee
Kung-Ta Lee
Jai-Wei Lee
Sunkyung Lee
W. M. Steve Lee
Jung-Kul Lee
Soojoon Lee
Kwang Pum Lee
Sylvia Lee
Cheng-Chi Lee
Kathy Lee
Minjin Lee
Ken Ka-Yin Lee
Mihwa Lee
Hwan Young Lee
Dongjin Lee
Jacky W. Y. Lee
Yun-Ju Lee
Jinseok Lee
Kwonmoo Lee
Chiachi Bonnie Lee
Kuen-Haur Lee
Regina Lee
Aline Lee
Kiho Lee
Seung-Tae Lee
Chien-Chang Lee
Daeyoup Lee
Tae Lee
Won-Chul Lee
Yuen Phin Lee
Ming Ta Michael Lee
Han Hong Lee
Moon Young Lee
Jintae Lee
Ihn Suk Lee
Joo Young Lee
Yu-Hsiang Lee
Wei-Ju Lee
Sung-Hoon Lee
Dong Ho Lee
Dae-Hee Lee
B. Lee
Kyu Sup Lee
Yun-Sil Lee
Jia-Jung Lee
Jaehwan Lee
Jong Seok Lee
David Lee
John Lee
Jin-Yong Lee
Sang Hoon Lee
Jeoung Soo Lee
S. D. Lee
Doo-Hyung Lee
Sun-Kyeong Lee
Edwin Leeansyah
Tosso Leeb
Martin Leeb
Frank Leebeek
Duane Leedell
Stephen Leeder
Mariska Leeflang
Peter Leegwater
Jong Han Leem
Rik Leemans
Alexander Leemans
Koen Leemput
William Leenders
Max Leenders
Frans Leenen
Luke Leenen
Nicholas Leeper
Thomas Leeper
Shelley Lees
Alexander Lees
Florian Leese
Henry Leese
Paul Leeson
S. Leeuwenburgh
Frances Lefcort
Tristan Lefebure
Kathi Lefebvre

Romain Lefebvre
David Lefebvre
Sébastien Lefebvre
Alice Lefebvre
Jennifer Lefever
Michael Lefevre
Carmen Lefevre
Jo-Anne Lefevre
Marie-Martine Lefevre-Colau
Alexander Leff
Hyam Leffert
Andreas Leffler
Jonatan Leffler
Avigdor Leftin
Megan Leftwich
Jean-Christophe Lega
Pierre Legagneux
Luc Legal
Bryan Legare
Antje Legatzki
Patrick Legembre
Christophe Legendre
Michelle Leger
Pierre Léger
Peter Leggat
Kevin Legge
Cristina Legido-Quigley
Justin Legleiter
Patricia Legler
Tobias Legler
Stéphane Legleye
Jerome Legoff
Benoit Legoff
Valery Legrain
Antoine Legrain
Valérie Legué
Christian Leguern
Mariana Leguia
Renaud Léguillette
Petri Lehenkari
Laurence Leherete
Aleksi Lehikoinen
Sidney Lehky
Tom Lehman
Michael Lehman
Joel Lehman
Dara Lehman
R. Lehman
Ordan Lehmann
Sune Lehmann
Helmar C Lehmann
Michael Lehmann
Hugo Lehmann
Irina Lehmann
Katja Lehmann
Birthe Lehmann
Manja Lehmann
Brian Lehmann
Anthony Lehmann
Klaus Lehmann-Horn
Angelika Lehner
M Lehner
Erik Lehnert
Lukas Lehnert
Klaus Lehnertz
Erik Lehnhoff
Thomas Lehrnbecher
Kaisa Lehti
Maria Lehtinen
Markku Lehto
Hanna Lehto
Sanna Lehtonen
Topi Lehtonen
Esko Lehtonen
Elissa Lei
Xu Lei
Hao Lei
Xin Gen Lei
Zhentian Lei
Guang-Hua Lei
Yuancai Lei
Zhenmin Lei
Tie-Chi Lei
Li Lei
Xia Lei
Tingwu Lei
Kai Lei
Shufeng Lei
Ruoh-Lih Lei
Xiaoying Lei
Bu Lei
Hansheng Lei
Zhen Lei
Chengwei Lei
Lei Lei
Yu Lei

Hetian Lei
Jikai Lei
Zhigang Lei
Merav Leiba
Adi Leiba
Rudolph Leibel
Eric Leibert
Joseph Leibovich
Arleen Leibowitz
Gregor Leibundgut
Lars Leichert
Christof Leicht
Petra Leidinger
Ernst Leidinger
Philip Leifeld
Cynthia Leifer
Mary Beth Leigh
Lucy Leigh
Andrew Leigh Brown
Terrance Leighton
Lindsey Leighton
Ralph Leijenaar
Jeroen Leijten
Kimberly Leiken
Silke Leimkühler
Mallorie Leinenger
Olof Leinhard
Päivi Leinonen
Claudia Leiros
Jonathan Leis
Jeffrey Leis
Alexander Leis
Roman Leischik
Tanya Leise
Jorgen Leisner
Paul Leisnham
Kirsten Leiss
Jorge Leitao
Alexandre Leitão
Harry Leitch
Katia R. M. Leite
Fernando Leite
Ricardo B. Leite
Fabio Leite
Nuno Leite
Lucas Leite Cunha
Maria Leite-De-Moraes
David Leitenberg
Michael Leitges
Katharina Leithner-Dziubas
I. Leitman
Suvi-Katri Leivonen
Susanna Leivonen
Marcis Leja
Tomás Lejarraga
Fabrice Lejeune
Karim Lekadir
Porntippa Lekcharoensuk
Malgorzata Lekka
Siri Leknes
Arne Lekven
Pushkar Lele
Sophie A. Lelievre
Brigitte Lelongt
A. Titia Lely
Juan Lema
Katleen Lemaire
Jean-Jacques Lemaire
Jean-François Lemaitre
Frederic Lemaitre
Matthew Lemay
Margus Lember
Jean-Michel Lemée
Virginie Lemiale
Louis Lemieux
Bruno Lemieux
Helene Lemieux
Simone Lemieux
Joseph Lemire
Kevin Lemley
Eshetu Lemma
Sebelewengel Lemma
Mekonnen Lemma Dechassa
Trudo Lemmens
Gunnar Lemmer
Nathan Lemoine
Maud Lemoine
Derek Lemoine
Sakari Lemola
Roger Lemon
Marc Lemonnier
Paula Lemons
Alberto Lemos
Henrique Lemos
Eliana Lemos
Paulo Lemos

Laetitia Lempereur
Meryem Lemrani
Willem Lems
Bert Lenaert
Metka Lenassi
Sergio Lence
Rebekka Lencer
Heather Lench
Mathias Lendner
Francoise Lenfant
Gareth Leng
Ping Leng
Chuan Leng
Theodore Leng
Claudia Lengerke
Erik Lenguerrand
Szabolcs Lengyel
Imre Lengyel
Boris Lenhard
Audrey Lenhart
Hunter Lenihan
Guy Lenk
Balint Lenkei
Robert Lenkinski
Rachel Lennon
Frances Lennon
Shannon Lennon-Edwards
Jeffrey Lennox
Matthieu Lenoir
Maxime Lenormand
Jennifer Lenow
Rhoshel Lenroot
Andy Lenssen
John Lenters
Pia Lentini
Jenny Lentz
Marcello Lenucci
Guido Lenz
Bernd Lenz
Henrike Lenzen
Jason Lenzo
Oberdan Leo
Fabrizio Leo
Patricia Leon
Carlos Leon
Lisa Leon
Ramon Leon
Paul Leon
Javier León
Sébastien Léon
Joshua Leonard
Anne Leonard
Rosemary Leonard
Paul Leonard
Hayley Leonard
Janet Leonard
Emanuela Leonardi
Stefano Leonardi
Ute Leonards
Margareta Leonardsson-Hellgren
Lorenzo Leoncini
Luigi Leone
Tina Leone
Fabio Leonessa
Michel Léonetti
Jo-Ann Leong
Mee-Mee Leong
Fong Yew Leong
Julian Leong
Christine Leong
Gary Leong
Howard Leong-Poi
Sara Leonhardt
Giovanni Leoni
Fidias E Leon-Sarmiento
Dario Leosco
Philippe Lepage
Jean-François Lepage
Mario Lepage
Kyle Lepage
Isabelle Leparc-Goffart
Edwin D. Lephart
Kathy Lepik
Ana Paula Lepique
J. C. Leple
Alice Leplongeon
Franco Lepore
Angelo Lepore
Domenico Lepore
Benedetto Lepori
Dino Leporini
Rebecca Lepping
Fabien Leprieur
Michael Lerario
Aymeric Leray
Jessica Lerch

Holger Lerche
Alexander Lerchl
Wondwossen Lerebo
Bernard Lerer
Edgar Lerma
Lilach Lerman
Kristina Lerman
Ulf Lerner
Seth Lerner
Derek Leroith
Tanya Leroith
Shawn Leroux
Sandrine Leroy
Grégoire Leroy
Amnon Lers
Unax Lertxundi
James Lesage
Frédéric Lesage
Claude Lesaux
David Lesbarrères
Julien Lescar
Félice Lê-Scherban
Emil Lesho
Clémence Lesimple
Tracey Leskey
Matthew Leslie
Kimberly Leslie
Andrew Leslie
Frances Leslie
Alasdair Leslie
Ayelet Lesman
Edward Lesnefsky
Julie Lesnik
Hervé Lesot
Renaud Lesourne
Justin Less
Marcos Lessa
Thais Lessa
Christopher Lessard
Samuel Lessard
Heidi M.B. Lesscher
Richard Lessells
Mark Lesser
Jason T. Lessl
Lawrence Lessner
Robert B. Lester
Patrick Lester
Katarzyna Leszczynska
Jacqueline Leta
Hend Letaief
Emmanuel Letavernier
Adrian Letchford
Michal Letek
Luc Letenneur
Stephane Leteurtre
Anne Lethaby
Thomas Leto
Gaetano Leto
Vincent Letouzey
Philippe Letteron
Thorsten Leucker
Nicolas Leuenberger
Stefan Leuko
Anskar Leung
Frederick Leung
Po Sing Leung
Melody Leung
Tinchung Leung
Ming-Ying Leung
Lawrence Leung
Ting Fan Leung
Polly Leung
Elaine Leung
Yiu Yan Leung
Henry Leung
Justin Leung
Patrick Leung
Man Yee Mallory Leung
Chi Hung Leung
Jacqueline Leung
Hon-Chiu Leung
June Leung
David Leung
Florian Leuschner
Steven Leuthner
Hartmut Leuthold
Dylan Levac
Erez Levanon
Beth Levant
Stuart Levenbach
Shulamit Levenberg
Alytia Levendosky
Anait Levenson
Robert Levenson
Marc Levenston
Ilya Levental

Maya Leventer-Roberts
Gabriel Leventhal
Aurora Levesley
Maxime Levesque
Crystal Levesque
Simon Lévesque
Paul Levett
Moshe Levi
Taal Levi
Edi Levi
Sonia Levi
Allan Levi
Benjamin Levi
Scott Levick
Barry Levin
Michael Levin
Petra Levin
Ann Levin
Netta Levin
Robert Levin
Adam Levin
Max Levin
Yafit Levin
Anna Levina
Alan Levine
Jon Levine
Steven Levine
Herbert Levine
Stephen Levine
Martin Levine
Joel Levine
Megan Levings
Randall Levings
James Levinson
Nick Levinson
Anna Levinsson
Jeffrey Levinton
Mark Levis
Emily Levitan
Yona Levites
Roy Levitt
Jonathan Levitt
Michael Levitt
Joseph Levitt
Bodo Levkau
Sue Levkoff
Zlatko Levkov
Zoran Levnajic
Shaul Lev-Ran
Frederic Levy
Emmanuel Levy
Colin Levy
Adrian Levy
Dino J Levy
Maggie Levy
Karen Levy
Brynn Levy
Ron Levy
Matthew Levy
S. Levy
Efrat Levy
Sharon Levy
Jason Levy
Michael Levy
Simcha Lev-Yadun
Daniel Levy-Bercowski
Einat Levy-Gigi
Andrew Lew
Stephan Lewandowsky
Gregory Lewbart
Lars Lewejohann
Sharon Lewin
Alfred Lewin
Daniel Lewin
Astrid Lewin
Andrew Lewington
Anna Lewinska
Oded Lewinson
George Lewis
Edwin Lewis
Sheri Lewis
Tim Lewis
Kim Lewis
Sheena Lewis
Margaret Lewis
Rohan Lewis
Janina Lewis
James Lewis
Martin Lewis
Michael Lewis
Simon Lewis
Rosamund Lewis
Ceri Lewis
David Lewis
Charlie Lewis
Russel Lewis

Dwight Lewis
Martyn Lewis
Jada Lewis
Kathleen Lewis
William Lewis
Jill Lewis
Lawrence Lewis
Grant Lewison
Jarrod Lewis-Peacock
Joan Lewis-Wambi
Eric Lewitus
Joseph Lewnard
Ala Lew-Tabor
Christiane Lex
Katrina Lexa
Joel Lexchin
Manfred Lexer
Anne Lexmond
Willem Lexmond
Klaus Ley
Alexandra Ley
Sylvia Ley
Ciara Leydon
Werner Leyh
Stefan Leyk
Alastair Leyland
Cedric Leyrat
Victor Leyva-Grado
Juan Leza
Claudio Lezama-Davila
Karina Lezirovitz
Frederic Lezot
Frank Lezoualc'H
Stefan Lhachimi
Thibault Lhermusier
Karl Lhotta
Congjun Li
Xiaohang Li
Xinhai Li
Yue-Ming Li
Ming Li
Baowen Li
Xuesen Li
Leping Li
Jun Li
Chiang-Shan Li
Qingshun Li
Fei Li
Huaixing Li
Guangpu Li
Benyi Li
Jia Li
Guangheng Li
Lang Li
Xia Li
James Li
Fang Li
Shisheng Li
Shao Li
Wei Li
Hongzhe Li
Xiang-An Li
Rong Li
Guohong Li
Xue-Jun Li
Erguang Li
Feng Li
Shi-Fang Li
Robert Li
Jinsong Li
Xiaoling Li
Menglong Li
Chunying Li
Pingping Li
Yi-Ping Li
Fan Li
De-Quan Li
Zhenyu Li
Mingzhou Li
Ying Li
Fenge Li
Chunhao Li
Su-Xia Li
Longchuan Li
Guoliang Li
Jianyuan Li
Jiana Li
Chenghua Li
Yan Li
Ganwu Li
Wan-Ju Li
Chien-Feng Li
Qi Li
Zihai Li
Yuanqing Li
Tsai-Chung Li

Guoqing Li
Zhaohai Li
Chaoyang Li
Hongfu Li
Qiao Li
Qi-Lang Li
Qi-Han Li
Fuli Li
Chengdao Li
Hua Li
Xiaoli Li
Daqing Li
Wenjing Li
Guohui Li
Fengzhi Li
Jieliang Li
Peifeng Li
Shou-Li Li
Guo Li
Zheng Li
Juan Li
Xiangjun Li
Songhai Li
Qinglei Li
Miao-Xin Li
Feng-Min Li
Ellen Li
Mingyuan Li
Quanxi Li
P. Andy Li
Min Li
Gordon Li
Ya-Tang Li
Xue-Bao Li
Jian Li
Daiqin Li
You Li
Chuan-Yun Li
Jing Li
Changgui Li
Yutao Li
Jing-Woei Li
Yanping Li
Wenbin Li
Tao Li
Hui-Jie Li
Jingyun Li
Hui Li
Sean (Xuguang) Li
Zhenqing Li
Yuan Li
Jinjun Li
Jih-Heng Li
Shao-Ping Li
Meng-Hua Li
Shoujun Li
Chang Xian Li
Wen-Shan Li
Ling Li
Dongmei Li
Liping Li
Xuexian Li
Chenghao Li
Kai Li
Linlin Li
Chaojun Li
Xingshan Li
Shanshan Li
Xiaogang Li
Bin Li
Xiaotian Li
Xiong Li
Huige Li
Yuanguang Li
Hongshuai Li
Yu Li
Hongliang Li
Li Li
Huating Li
Maoteng Li
Hui-Ping Li
Changchun Li
Qiuhong Li
Huabin Li
Suxia Li
Jianneng Li
Anan Li
Zhen Li
Hongyi Li
Luowei Li
Yanbing Li
Hongwei Li
Rui Li
Xixiang Li
Hang Wun Raymond Li
Dejun Li

Yinxin Li
Shuxin Li
Chun Wei Li
Lingjiang Li
Jianqiang Li
Xinrui Li
Shaohua Li
Weizhe Li
Ye Lennon Li
Zhenlin Li
Jianhong Li
Yong-Feng Li
Jin Li
Kaiming Li
Bichun Li
Donghai Li
Zhao-Liang Li
Jingya Li
Tian-Fang Li
Jing Jing Li
Wenqing Li
Zhongmin Li
Fangbai Li
Li-Xin Li
Ao Li
Yongqin Li
Qing Li
Yue-Zhong Li
Cong-Jun Li
Shi-Ming Li
Dong Li
Chunming Li
Lei Li
Liang Li
Meili Li
Na Li
Qiu Li
Xiaobai Li
Shiyou Li
Su Li
Tingqiang Li
Jiale Li
Xiaoqing Li
Dongye Li
Shaokun Li
Jianfeng Li
Yuanyou Li
Sheng Li
Gang Li
Zhongwu Li
Yang Li
Liangcheng Li
Shuangcheng Li
Changwei Li
Xiaochun Li
Peng Li
Xishan Li
Zhoujun Li
Yihang Li
Dezhi Li
Guorong Li
Feiyu Li
Baoguo Li
Bingjin Li
Jing-Nan Li
Chengfang Li
Kui Li
Qingguo Li
Boqiang Li
Yuanchun Li
Yujuan Li
Hua-Jung Li
Mingkun Li
Fuhong Li
Weiguang Li
Chao Li
Zhentian Li
Yuxin Li
Zhipeng Li
Zhanguo Li
Jian-Ming Li
Jin-Tao Li
Ya Li
Liu Li
Zhanzhan Li
Shu Li
Wen Li
Xiaoying Li
Chen Li
Xinmiao Li
Dongpei Li
Hu Li
Lixiang Li
Hai-Feng Li
Xuming Li
Yongjin Li

Xianglan Li
Tieshi Li
Yuanyuan Li
Zhiming Li
Qiang Li
Hongge Li
Aihua Li
Huihui Li
Lingjie Li
Xiaofeng Li
Maoyin Li
Xinyun Li
Bo Li
Mingyu Li
Aike Li
Xiaoshu Li
Xiwen Li
Xueyong Li
Guoan Li
Li-Hua Li
Hung-Yuan Li
Lu Li
Ke Li
Tianhong Li
Chong Li
Ming-He Li
Longhui Li
Weizhong Li
Shugang Li
Botong Li
Chunyan Li
Yingwei Li
Fuhua Li
Xiufeng Li
Sy Li
Aimin Li
Huiying Li
Yun Li
Daofeng Li
W. P. Li
Weifeng Li
Xiaojuan Li
Xinzheng Li
Yong Li
Shuwen Li
De-Wei Li
Hang Li
Zhongming Li
Shijia Li
Yizeng Li
Lingjun Li
Zili Li
Jianfa Li
Haitao Li
Lianhua Li
Pengfu Li
Yanzhi Li
Zhongwei Li
Liguo Li
Wenli Li
Yimei Li
Ting Li
Z. H. Li
Honghai Li
Siming Li
Ruijiang Li
Dong Ming Li
Zhuo Li
Xinxu Li
Yikang Li
Zhi Li
Huixin Li
Shaofan Li
Qingfeng Li
Xuan Li
Yiyin Li
Ming-Tao Li
L. Li
Qian Li
Gen-Liang Li
Yajuan Li
Dayong Li
Huijun Li
Sophie Li
Cong Li
Wan-Chun Li
Zhigang Li
Baikun Li
Junhua Li
Xiangning Li
Dengwang Li
Xuping Li
Ruifan Li
Jun-Hao Li
An-Wei Li
Zhuoming Li

Meng Li
Zhengbang Li
Pan Li
Junfeng Li
Zhaofei Li
Jinyao Li
Song Li
Zhenghua Li
Lulin Li
Yanfang Li
Jinjin Li
Run-Zhi Li
Jiawen Li
Steven Li
Shouli Li
Shumin Li
Yaning Li
Zhengke Li
Rongbai Li
Guodong Li
Wenjun Li
Chia-Cheng Li
Changxi Li
Weijuan Li
Jingui Li
Dihua Li
Muwang Li
Lin Li
Guanhan Li
Xin Li
Fuchuan Li
Chunhai Li
Yunqi Li
Yi-Chia Li
Yanjiao Li
Huihua Li
Xuejie Li
Junmin Li
Hong-Fu Li
Sai-Ping Li
Y. F. Li
Xiaopeng Li
Baoguang Li
Zhanjun Li
Henghong Li
Xiao-Ran Li
Zhongjun Li
Yibo Li
Emmy Li
Shi-Hua Li
Jian-Bin Li
Chun Li
Weidang Li
Yingyue Li
Xiantao Li
Chenghe Li
Heng Li
Zhongyu Li
Dawei Li
Jiali Li
Tingting Li
Xiuling Li
Guangye Li
Jue Li
Hedong Li
Zhe Li
Xinmin Li
Jiang Li
S. F. Li
Xuehui Li
Tianyuan Li
Chenhong Li
Leida Li
Hong-Li Li
Erchao Li
Jiong Li
Jisu Li
Xinya Li
Xin-Guo Li
Shihua Li
Minxing Li
Junxu Li
Zezhi Li
Yongfang Li
Ningjun Li
Baizhan Li
Yongfu Li
Huan Li
Yuxing Li
Xiaonan Li
Xueqi Li
Xinde Li
Zibo Li
Sergio Li Calzi
Sergio Li Petri
Giovanni Li Volti

Sophie Liabeuf
Tippawan Liabsuetraku
Bertrand Liagre
Chengjie Lian
Zhengxing Lian
Xiaojun Lian
Conglong Lian
Yi Liang
Guosheng Liang
Jiyong Liang
Wei Liang
Guodong Liang
Caihua Liang
Cher-Wei Liang
Guang Liang
Houjie Liang
Hao Liang
Yuan Liang
Eryuan Liang
Zhenglun Liang
Haiying Liang
Yuying Liang
Tingbo Liang
Jialiang Liang
Po-Huang Liang
Shangdong Liang
Huaping Liang
Cheng-Guang Liang
Xia Liang
Qiangrong Liang
Pei Liang
Hung Hua Liang
Wanqi Liang
Xiaoling Liang
Haihua Liang
Anna Liang
Chun Liang
Cheng-Chao Liang
Lingyi Liang
Naishen Liang
Zhifeng Liang
Yongheng Liang
Rui Liang
Yong Liang
Jun Liang
Michael Tc Liang
Aizhen Liang
Liang Liang
Aihua Liang
X. Liang
Peipeng Liang
Kaicheng Liang
Jing Liang
Deguang Liang
Xibin Liang
Fan-Rong Liang
Hong Liang
June Liang
Yajie Liang
Lu Liang
Xinghua Liang
Jian-Hui Liang
Zongsuo Liang
Dong Liang
Shuang Liang
Jingjing Liang
Yinli Liang
Xiaozhen Liang
Chih-Ming Liang
Xiao Liang Dong
Suthat Liangpunsakul
Jiayu Liao
Wanqing Liao
Jian-You Liao
Joseph Liao
Fang Liao
Wan-Jin Liao
Yung-Feng Liao
Shi Jun Liao
Guoyang Liao
You-Di Liao
Wei Liao
Shanhui Liao
Wei-Chih Liao
Pei-Chun Liao
Yu-Chien Liao
Jiunn-Der Liao
Hua Liao
Yu-Ping Liao
Ping Liao
Vivian Liao
Sumei Liao
Wen-Bo Liao
Lingjie Liao
Mingzhi Liao
Min Liao

Yulin Liao
Yuxing Liao
Yi-Chu Liao
Guo-Shiou Liao
Chia-Te Liao
Hong Liao
Baojian Liao
Hao Liao
Jichen Liao
Ge Liao
Liao Liao
Qian Liao
Jiangqun Liao
Felix Haifeng Liao
Yaping Joyce Liao
Daiqing Liao
Irfana Liaqat
Laurence Liaubet
Lucas Liaudet
Andy Liaw
Yun-Fan Liaw
Stephen Libby
Camilo Libedinsky
Nicola Lucio Liberato
David Liberles
Stephen Liberles
Sergiy Libert
Silvana Libertini
Klaus Libertus
Melissa Libertus
Alexander Liberzon
Alexandre Liborio
Daniel Libraty
Diana Libuda
Lísa Libungan
Hongmei Li-Byarlay
P. Licandro
Concetta Licciardello
Paul Licciardi
Eunice Li-Chan
Michael Lichtenauer
Wandy Lichtenthal
Nathanael Lichti
Ralf Lichtinghagen
Andrew Lichtman
Gaetano Licitra
Brian Lickel
Rachel Licker
Shane Liddelow
Joshua Liddy
Keith Lidke
Ofir Lidor
Charles Lidz
Dieter Lie
Pearl Lie
Klaus Lieb
Katja Liebal
Ulf Liebal
Richard Liebano
Stefan Liebau
Max Liebau
Manuel Liebecke
Michael Lieber
Justin Lieber
Raquel Lieberman
Jay Lieberman
Dan A Liebermann
Dario Liebermann
Benjamin Liebeskind
Jürgen Liebig
Johanna Liebl
Daniel Liebl
Dan Liebling
Matt Liebman
Larry Liebovitch
Michael Liebrenz
Jane Liebschutz
Felix Liechti
Matthias Liechti
Edward Liechty
Ramona Lieder
Astrid Liedert
Wolfgang Liedtke
Carole Liedtke
Christian Liedtke
Miriam Liedvogel
Justin Liefer
Victor Lieffers
Angela Liegey Dougall
Raffael Liegl
Thomas Liehr
Franz Liem
Ching-Ling Lien
Juliane Liepe
Aaron Liepman
Graham Lieschke

John Lieske
Knut Liestøl
Arthur Liesz
Daniel Lietha
Thomas Lietman
Tinamarie Lieu
Nicolas Lieury
Jean-Charles Lievens
Ritsaert Lieverse
Chong Wee Liew
Siaw-Cheok Liew
Yi Jin Liew
Jan Lifjeld
A. Ligaba
Samuel Light
David C. Lightfoot
Stafford Lightman
John Lighton
Felice C. Lightstone
James Lightwood
Yves Lignereux
Romain Ligneul
Constantine Lignos
Petros Ligoxygakis
Maarten Ligtenberg
Wilco Ligterink
Claudio Liguori
Marijn Lijffijt
Garrett Liles
Tang Lili
Guan Lili
Markus Lill
Roland Lill
Peter Lillehoj
Karen Liller
Catherine Lilley
Martin Lilley
Ian Lilley
Simon Lillico
Timothy Lillicrap
Mette Lillie
Christopher Lillig
Ashlee Lillis
Concepcion Lillo
Fabrizio Lillo
Brenda Lilly
Harvey Lillywhite
Sai Kiang Lim
Tit Meng Lim
B. L. Lim
Wei-Yen Lim
Rebecca Lim
Wai Lim
Yen Ying Lim
Jenson Lim
Lina Lim
Jung Yul Lim
Boon Lim
Changwon Lim
Haikel Lim
S.H. S. Lim
Do-Sun Lim
Y. K. Lim
Eunjung Lim
Kian Meng Lim
Hye-Sun Lim
Young-Suk Lim
Koeun Lim
Geunbae Lim
Seokbin Lim
Ilhan Lim
Rongxuan Lim
Howie Lim
Shen-Yang Lim
Sungkyun Lim
Kyungjoon Lim
Cynthia Lim
Chinten Lim
Christopher D. Lima
Fabio Lima
Steven Lima
Julio César Lima
Jose Bento Lima
Rui Lima
Marcelo De Oliveira Lima
Thais Lima
Flávia Lima
Karine Lima
Alexandre Lima
Cristiano Lima
Emerson S. Lima
Marian Limacher
Anis Limami
N. Liman
Matheus Lima-Ribeiro
Adriano Lima-Silva

Jacqueline K. Limberg
Morten Limborg
Sean Limesand
Kristen Limesand
Wu Limin
Thawornchai Limjindaporn
Agenor Limon
Maarten Limper
Xiaorong Lin
Philana Lin
Qingsong Lin
Wen-Chang Lin
Jue Lin
Ruo-Kai Lin
Henry C. Lin
Jian-Sheng Lin
Sue-Hwa Lin
Jun Lin
Shau-Ping Lin
Brenda Lin
Yong Lin
Shinn-Zong Lin
Fucheng Lin
Shih-Hua Lin
Xiaojing Lin
Chung-Yen Lin
Jiandie Lin
Yi-Ling Lin
Tsan-Piao Lin
Wenyu Lin
Hua Lin
Hai-Yan Lin
Chiou-Feng Lin
Jialing Lin
Jer-Sheng Lin
Xia Lin
Dong-Tsamn Lin
Choun-Sea Lin
Qing Lin
Jessica Lin
Chun-Yen Lin
Mei-Hui Lin
Shih-Yi Lin
Dongxu Lin
Chunliang Lin
Bin Lin
Hong Lin
Ching-Long Lin
Ying-Ju Lin
Michael Lin
Sheldon Lin
Hung-Yun Lin
Kwang-Huei Lin
Fuchun Lin
Rick Lin
Honghuang Lin
Hui Lin
Yi-Tsung Lin
Feng-Yen Lin
Ping-Ting Lin
Francis Lin
Yihan Lin
Zhongxu Lin
Shuei-Liong Lin
Xiangui Lin
Wan-Yu Lin
Chin-Yo Lin
Ching-Shwun Lin
Shu-Chun Lin
Chun-Yi Lin
Si-Min Lin
Yingsong Lin
Hung-Ru Lin
Chen-Yong Lin
Wey-Ran Lin
Jiunn-Lee Lin
Chih-Yuan Lin
Tsai-Lien Lin
Hsing-Juh Lin
Phoebe Lin
Ji-Fan Lin
I-Feng Lin
Nien-Tsung Lin
Hongfei Lin
Pan Lin
Li Lin
Dongmei Lin
Rongcheng Lin
Wei-Ning Lin
Hao Lin
Ching-Wen Lin
Been-Ren Lin
Dayu Lin
Chen Lin
Xingwu Lin
Xiao-Min Lin

Hui-Kuan Lin
John Lin
Yu-Ru Lin
Chung-Ying Lin
Da-Ting Lin
Lin Lin
Yuh-Feng Lin
Hung-Du Lin
Weei-Chin Lin
Ling-Yi Lin
Kai Lin
Fenfang Lin
Xiaochen Lin
Yuan-Pin Lin
Clifford Lin
Cheng-Hung Lin
Yi Lin
Qin Lin
Zhong Lin
Ruiting Lin
Honghui Lin
Feng-Huei Lin
Chen-Si Lin
Xianzhi Lin
Chih-Ta Lin
Tzu-Hao Lin
Jianhong Lin
Han-You Lin
Chen-Chun Lin
Chin-Tarng Lin
Yu-Wei Lin
Qun Lin
Qi Lin
Yuqing Lin
Xianming Lin
Weili Lin
Jintian Lin
Chun-Hung Lin
Moubin Lin
Jules Lin
Ming-Yen Lin
Jinxiu Lin
Y. C. Lin
Yuan Lin
Yi-Dong Lin
Chuyong Lin
Jingyi Lin
Zhigui Lin
Xiaoxi Lin
Xiao Lin
Nai-Chun Lin
Yu-Hung Lin
Xiaolong Lin
Zhuoling Lin
Li-Mei Lin
Chun-Yu Lin
Yin Lin
Yang Wei Lin
Yl Lin
Huaiying Lin
Dong Lin
Ashleigh Lin
Keng-Han Lin
Steven Lin
Huey-Juan Lin
Chia Lin
Lawrence Lin
Zhifen Lin
Yu-Pin Lin
Ting-Sheng Lin
Pao-Yuan Lin
Mao Lin
Duoru Lin
Chung-Ping Lin
Yucong Lin
Yu-Hsuan Lin
Zhiyong Lin
Chin-Kai Lin
Hsiu-Chin Lin
Wenwei Lin
Yen-Chih Lin
Jun-Fang Lin
Chii-Wann Lin
Chih-Lin Lin
Jay Lin
Chuan Lin
Hai Lin
Z. Lin
Jeen-Shang Lin
Tong-Jun Lin
Wei Lin
Yiwei Lin
C. Y. Lin
Patrick Lin
Chih-Jen (Lance) Lin
Yuan-Feng Lin

Catherine Linard
Corinne Linardic
Spiros Linardopoulos
Cristina Linares
Pedro Linares
Benjamin Linas
Stuart Linas
Joy Lincoln
Anne Lincoln
Nilton Lincopan
Penelope Lind
Erik Lind
Paul Lindahl
Tomas Lindahl
Manfred Lindau
Iris Lindberg
Mark S. Lindberg
Pia Lindberg
Matts Lindbladh
Klaus Linde
Joerg Linde
Staffan Lindeberg
Jan Lindeman
Ulrich Lindemann
Ariel Linden
Dan Linden
Martin Lindén
Brett Lindenbach
Patrik Lindenfors
David Lindenmayer
Stefan Linder
Jan Linder
Joshua Linder
Michael Linderman
Lisa Lindesmith
Dan Lindfield
Helena Lindgren
Jenny Lindh
S. R. Lindheim
Bengt Lindholm
Anna Lindholm
Elizabeth Lindley
Nic Lindley
Angelica Lindlöf
Axel Lindner
Daniel Lindner
Benjamin Lindner
Andreas Lindner
Gregor Lindner
Kirsten Lindner
Ewald Lindner
Alberto Lindner
Maren Lindner
Steven Lindow
Erin Lindquist
Derick Lindquist
Kristen Lindquist
Pelle Lindqvist
Steven Lindsay
D. Lindsay
Scott Lindsay
Everett Lindsay
Merry Lindsey
Peter Lindsey
James Lindsey
Marcus Lindskog
William Lindsley
Anna Lindstrand
Jon Lindstrom
Nils Lindstrom
Mikael Lindström
Tom Lindström
Bernt Lindtjon
Frida Lindwall
Sergio Line
Binhua Ling
Zhi-Qiang Ling
Chen Ling
Li Ling
Feng Ling
Qin Ling
Erjun Ling
Thomas Ling
King Hwa Ling
Xiang Ling
Shiqi Ling
Juan Ling
Chang Chun Ling
Gee Ling
Nicholas Ling
Dennis Ling
Annett Linge
Ole Lingjaerde
Agnes Linglart
Susan Lingle
Angelika Lingnau

Jerry Lingrel
Hester Lingsma
Guido Lingua
Chen Ling-Yun
Marcelo Linhares
Daniel Linhares
Markus Linhart
Andrew Link
Brian Link
Alexander Link
Hannes Link
Dan Link
Emma Link
Dirk Linke
Simon Linke
Sebastian Linke
Sally Linkenauger
Raphael Linker
Wayne Linklater
Faina Linkov
Matthew Links
Ezra Linley
Charles Linn
Kristin Linn
Jennifer Linn
Vesa Linnamo
Michael Linnebacher
John Linnell
Alexandra Linnemann
Elwood Linney
Valeria Lino
Bruce Linquist
Roberto Lins
Anna Carolina Lins
Paul Linser
Christiane Linster
Carole Linster
Piyada Linsuwanon
Gabor Linthorst
Kenneth Linton
Macrae F Linton
Dominik Linz
Peter Lio
Pio Lio
Geraldine Liot
Ruey-Fen Liou
Jun-Yang Liou
Jyh-Ming Liou
Chia-Wei Liou
Ming-Li Liou
Saou-Hsing Liou
Vasileios-Arsenios Lioutas
Fred Lipfert
Anna-Maria Liphardt
Ewelina Lipiec
Michael Lipinski
Marta Lipinski
Daniel Lipinski
Daniel Lipka
Alexander Lipka
Peter Lipke
Marc Lipman
Jeffrey Lipman
Darren Lipnicki
Erin Lipp
Frank Lippert
Adam Lippert
Arto Lipponen
Paul Lips
John Lipscomb
Kenneth Lipson
Howard Lipton
John Lipuma
Wendy Lipworth
Rosalia Lira
Kriengsak Lirdprapamongkol
Stefanie Lis
Patricia Lisboa
Thiago Lisboa
Luiz Lisboa
Heike Lischke
Christophe Liseron-Monfils
Giuseppe Lisi
Gina Lisignoli
Martin Liska
Adam Liska
John Lisman
Sarka Lisonkova
Simeon Lisovski
Steven Liss
Tom Lissauer
Gianluca Lista
James Lister
Conor Liston
James Litch
Glen Litchwark

Ivan Literak
Gary Litherland
Nicolle Litjens
Geert Litjens
Paloma Liton
Irene Litosch
A. Litster
Kate Littin
David Little
Jonathan Little
Richard Little
Peter Little
Dianne Little
Kristen Little
Holly Little
Cathy Little
Bertis Little
Jeri Little
Andrew Littlefield
Chris Littlewood
Jasper Littmann
Zachary Litvack
Irene Litvan
Anastasia Litvintseva
Brett Litz
Jianquan Liu
Jun Liu
Bing Liu
De-Pei Liu
Renyi Liu
Albert Liu
Hongyan Liu
Haoping Liu
George Liu
Ming-Tsan Liu
Qiang Liu
Yongsheng Liu
Bolin Liu
Xueduan Liu
Yufeng Liu
Shing Liu
Fei Liu
Shaojun Liu
Shih-Jen Liu
Xuefeng Liu
Hesheng Liu
Ji-Long Liu
Xiufan Liu
Taosheng Liu
Xiaoqi Liu
Shanrong Liu
Zhijin Liu
Philip Liu
Zhonghua Liu
Zhihui Liu
Xue-Song Liu
Sanzhen Liu
Nannan Liu
Zewen Liu
Xingzhong Liu
Aimin Liu
Wende Liu
Zexian Liu
Xibao Liu
Qing Liu
Bingqiang Liu
Dazhi Liu
Chang Liu
Andrew Liu
Qiaoquan Liu
Juan Liu
Yang Liu
Shikai Liu
Caigang Liu
Xiaorong Liu
Qing-Song Liu
Xin-Yuan Liu
Zuguo Liu
Baorui Liu
Yan Liu
Hongjie Liu
Dongjun Liu
Jian Liu
Yule Liu
Yusen Liu
Haiying Liu
Shu Q Liu
Zhihua Liu
Ju Liu
Jing Hua Liu
Ching-Ti Liu
Chun-Jen Liu
Jinhua Liu
Jiang-Qin Liu
Zhanjiang Liu
Xinhai Liu

I-Min Liu
Huanxiang Liu
Jue Liu
Chunyu Liu
Xun Liu
Keliang Liu
Xiang Liu
Pi-Hua Liu
Bindong Liu
Jian-Guo Liu
Sijin Liu
Cun-Zhi Liu
Xiaojun Liu
Tian Liu
Bao Liu
Jianghuai Liu
Zonghua Liu
Yu Liu
Jer-Yuh Liu
Xuyang Liu
Fudong Liu
Qizhan Liu
Hong Liu
Ho-Ling Liu
Jialing Liu
Bang Liu
Fengquan Liu
Zhao-Qian Liu
Shiguo Liu
Xingyue Liu
Hongbin Liu
Tianrun Liu
Ji-Bin Liu
Yongkui Liu
Zhi-Yong Liu
Guihua Liu
Qingdu Liu
Jia-Ren Liu
Xianyun Liu
Cheng-Tzu Liu
Huanliang Liu
Bei Liu
Jing Liu
Guiyou Liu
Huafeng Liu
Shao-Lun Liu
Xi Liu
Qi Liu
Song-Mei Liu
Dengcai Liu
Jin-Xian Liu
Yuru Liu
Yangyang Liu
Gang Liu
Yanxin Liu
Yanhong Liu
Qing-Xin Liu
Chen-Hua Liu
Xihui Liu
Canran Liu
Shuguang Liu
Zhipeng Liu
Wendy Liu
Yuying Liu
Shengyi Liu
Dongyou Liu
Ke Liu
Wenshe Liu
Chun-Chi Liu
Qin-Xue Liu
Ming-Cheh Liu
Shubing Liu
Ying Liu
Zhan-Lin Liu
Hong-Yi Liu
Hongbo Liu
I-Chung Liu
Jenny Liu
Juxiu Liu
Ju-Fang Liu
Chun-Feng Liu
Ko-Jiunn Liu
Lin Liu
Guoshi Liu
Shiliang Liu
De-Yi Liu
Qin Liu
Zhuoming Liu
Liwang Liu
Wei Liu
Aizhong Liu
Zhen Liu
Chien-Liang Liu
Qingguang Liu
Wusheng Liu
Bingya Liu

Zhandong Liu
Hao Liu
Yongchao Liu
Yong Liu
Lunxu Liu
Shiming Liu
Ya-Hong Liu
Bi-Cheng Liu
Ling-Zhi Liu
Hongli Liu
Yijun Liu
Man-Qing Liu
Hongrui Liu
Shuzhen Liu
Quan Liu
Xin-Yang Liu
Ming Liu
Chaohong Liu
Guoxiang Liu
Tong-Xian Liu
Huishu Liu
Xiaozhong Liu
Jie Liu
Fusheng Liu
Zhongwei Liu
Wanhong Liu
Xinshe Liu
Xiaochuan Liu
Run-Ran Liu
Wen Liu
Yadong Liu
Guangxu Liu
Jinny Liu
Chuang Liu
Xiao-Jun Allen Liu
Jun-Jen Liu
Zheng Liu
Kwang Hyeon Liu
Li Liu
Wen-Bin Liu
Dongfang Liu
Zhili Liu
Siqi Liu
Zhiyong Liu
Liyu Liu
Feng Liu
Xiaohua Liu
Baoming Liu
Yarong Liu
Yun Liu
Ting Liu
Zhenlan Liu
Luxiang Liu
Zhenghui Liu
Yin-Quan Liu
Chun-Yu Liu
Zhengxia Liu
Guanjun Liu
Yiqing Liu
Baodong Liu
Zeming Liu
Chen Liu
Kai Liu
Jiexin Liu
Junjun Liu
Ming-Lin Liu
Dungang Liu
Zhao Liu
Chunsheng Liu
Qili Liu
Guansheng Liu
Yuwei Liu
Han Liu
Chao Liu
Jiabin Liu
Yunlong Liu
Chenggang Liu
Feiyan Liu
Xiaolei Liu
Liang Liu
Huitong Liu
Jiacai Liu
Hu Liu
Yancheng Liu
Zippo Liu
Yong-Zhong Liu
Xiaohui Liu
Yao Liu
Tao Liu
Zhengtao Liu
Jingbo Liu
Chai-E Liu
Xia Liu
Yanna Liu
Yunen Liu
Qiangming Liu

Xiao-Jing Liu
Yuhong Liu
Yutao Liu
Shelan Liu
Stephanie Liu
Bo Liu
Xiaohong Liu
Jin-Hu Liu
Anmin Liu
I-Hsin Liu
Zhun-Zhun Liu
Xiangde Liu
Mingxia Liu
Nan Liu
Ruolin Liu
David Liu
Miao Liu
Yunhua Liu
Peng Liu
An Liu
Quanxing Liu
Xiu Liu
Zheng-Rong Liu
Chun-Hung Liu
Tie Fu Liu
Xiaoping Liu
Yaoru Liu
Runran Liu
Lei Liu
Chia-Chi Liu
Song Liu
Manqiang Liu
Yonghong Liu
Qingsheng Liu
Yuming Liu
Yuelu Liu
Jianguo Liu
Dianzhi Liu
Xinwang Liu
Fang Fang Liu
Vincent Liu
Wei-Jie Liu
Zhentong Liu
Di Liu
Jen Liu
Yaling Liu
Vanson Liu
Spencer Liu
Qingyun Liu
Xin Liu
Chengcheng Liu
Qinghang Liu
Shiguang Liu
Datong Liu
Kaiyu Liu
Ran Liu
Ziyi Liu
Xiaoli Liu
Ju-Chi Liu
Yongjian Liu
Shuang Liu
Zhongyuan Liu
Maoxing Liu
Fang Liu
James Liu
Wenhai Liu
Dongqiang Liu
Xiangguo Liu
Rui Liu
Fenwu Liu
Xinfeng Liu
Zi-Jun Liu
Dajiang Liu
Zhuannian Liu
Jane Liu
Tan Liu
Xiaoxiao Liu
Hua Liu
Debing Liu
Zhenzhen Liu
Jiajuan Liu
Minghui Liu
W. Liu
Yen-Nien Liu
Shao Hsien Liu
Liegang Liu
Yen-Wen Liu
Chengming Liu
Xiaolin Liu
Jushan Liu
Ping-Yen Liu
Yu-Chi Liu
Jen-Pei Liu
Wanqing Liu
Ning Liu
C. Liu

Xuan Liu
Keyan Liu
Hsuan Liu
Ximeng Liu
Jian-Jun Liu
Zhuo-Hao Liu
Man Liu
Meilian Liu
Yiwen Liu
Yen-Lin Liu
Zhi Liu
F. Liu
Qinde Liu
Ke-Zhi Liu
Che-Ming Liu
Xiaofeng Liu
Xunjun Liu
Pengcheng Liu
Hongming Liu
Jin-Ping Liu
Deli Liu
Zhimin Liu
Zhifeng Liu
Shuo Liu
Zhan-Ju Liu
Shuyu Liu
Xigang Liu
Jin Liu
Xiaomin Liu
Hongjun Liu
Xiaoming Liu
Chang Hong Liu
Shu-Man Liu
Po-Hong Liu
Xuanghong Liu
Qianing Liu
Xiaowen Liu
Tongran Liu
Peter Liu
Jingze Liu
Jing-Xia Liu
Shichao Liu
Bolan Liu
Yudong Liu
Xulei Liu
Fulai Liu
Shi Liu
Yi Liu
Lixin Liu
Changchun Liu
Suxia Liu
Bin Liu
Xiao-Qing Liu
Yifei Liu
Hu-Chen Liu
Ying-Hsang Liu
Dongfei Liu
Helene Minyi Liu
Marco Tullio Liuzza
Grazia Liuzzi
Raffaele Liuzzi
Britt Livak
Giacomo Livan
Gabriel Livera
Simon P. Liversedge
Helen Liversidge
Dennis Livesay
Sarah Livesay
Jean Livet
Valerie Livina
David Livingston
Alessandra Livraghi
Heather Liwanag
Patricia Liwang
Robert Liwski
Lisa Lix
Gerard Lizard
Omar Lizardo
Gregory Lizee
Nelson Lizier
Alexander Ljubimov
Milos Ljubisavljevic
Britt-Marie Ljung
Battuvshin Lkhagvasuren
Montserrat Llagostera
Bastien Llamas
Adana Llanos
Ann-Katrin Llarena
César Llave
Alberto Lleo
Martin Llewellyn
Josep M Llibre
Joel Llopiz
Oscar Llorca
Silvia Llorens
Briardo Llorente

Vicenta Llorente-Cortés
Josep Lloreta
Matxalen Llosa
Joyce A. Lloyd
Steven Lloyd
Marianne Lloyd
John Lloyd
Rhiannon Lloyd
Huw Lloyd
Donald Lloyd-Jones
Frederic Lluis
Laura Llull
Chu-Fang Lo
Su Hao Lo
Jeng-Fan Lo
Janice Y. C. Lo
Janet Lo
Bernice Lo
Yi-Chun Lo
Gin-Ho Lo
Chun-Liang Lo
Shih-Yen Lo
Yu-Shu Lo
Suzanne Lo
James Lo
Wen-Tseng Lo
Ying-Ru Lo
Warren Lo
Kenneth Lo
Benjamin Lo
Clive Lo
Lorenzo Lo Muzio
Alessandra Lo Presti
Andrea Lo Vecchio
Andrea Loayza
Lucas Lobato
Peter Lobel
Robert Lober
Guillaume Lobet
Alexander Lobkovsky
Janek Lobmaier
Jose Lobo
Francisco Lobo
Dileep Lobo
Sonja Lobo
Peter Lobo
Malgorzata Lobocka
Agnieszka Loboda
Donald Lobsien
Stephen Locarnini
Joseph Locascio
Francesco Locatelli
Lisa Locatello
Marc Lochbaum
Timothy Lochmann
Amanda Lochner
Richard Lock
Adam Locke
Joseph Locker
Shawn Lockhart
Samuel Lockhart
Steve Lockless
Shahin Lockman
Oksana Lockridge
Julie Lockwood
Rowan Lockwood
Julianne Lockwood
Brent Lockwood
Jocelyn Lockyer
Noelle Loconte
W. Lodder
Robert Lodder
Mary Lodén
Elizabeth Loder
Caroline Lodge
John Lodge
Andrew Lodge
Nilanjan Lodh
Rakesh Lodha
Irfan Lodhi
Stephen Lodmell
Melissa Lodoen
Claudia Lodovichi
David Lodowski
Leif Egil Loe
Lawrence Loeb
Stacy Loeb
Mitch E. Loeb
Lisa Loeb
Gerard Loeber
Mike Loeffelholz
Kati Loeffler
John Loehlin
Mary Loeken
Octavio Loera
Adrian Loerbroks

Volker Loeschcke
L. Sue Loesch-Fries
Richard Loeser
Daniela Loessner
Markus Loeven
Michael Loevinsohn
Mark Loewen
Nils Loewen
Andrea Loewendorf
Yonatan Loewenstein
George Loewenstein
Rachel Loewy
Lorenzo Loffredo
Brent Lofgren
Christian Löfke
Jennifer Loftis
Elisabeth Loftus
Maria Logacheva
Darren Logan
Richard Logan
Susan Logan
David Logan
Ryan Logan
Samantha Logan
Jeongok Logan
Kenneth Logan
Greig Logan
Tharani Loganathan
Ramiro Logares
Damien Logeart
David Logerstedt
Carmen Logie
Antonio Logrieco
Craig Logsdon
Catherine Logue
Gunnar Loh
Stewart Loh
Liyen Loh
Marie Loh
Kim Loh
Soh Kheang Loh
Richard Loh
Mohtashim Lohani
Gertrud Lohaus
Madelon Lohbeck
Hannes Lohi
Neha Lohia
Jan Lohmann
David Lohmann
Kenneth Lohmann
Asko Lõhmus
Szimonetta Lohner
Andrew Lohrer
Konrad Lohse
Noemi Lois
Nicolas Loiseau
Marisa Loitfelder
Ursula Loizides-Mangold
Bannakij Lojanapiwat
James Lok
Ker Zhing Lok
Josephine Lok
Yoon Loke
Mun Fai Loke
Eric Loken
Vinata Lokeshwar
Ellen Løkkegaard
Tapio Lokki
Mark Lokman
Anna Lokshin
Christina Loley
Pete Lollar
Donald Lollar
Nicholas J. Loman
Herve Lombaert
Marlize Lombard
Martani Lombard
Raul Lombardi
Augusto Lombardi
Lara Lombardi
Vincent Lombardi
Marco Lombardo
Michael Lombardo
Johanne Lomholt
Marek Lommatzsch
Miriam Lommen
Slawo Lomnicki
Sara Lomonaco
Patrick Lomonte
Olga Lomovskaya
David Lonard
Amedeo Lonardo
A. Lonardo
Dejan Loncar
Jadranka Loncarek
Stephanie London

Robert London
Kamala London
Maria Londoño
Richard Londraville
Patrick Lonergan
Steven Lonergan
Yan Long
Fanxin Long
Mian Long
David Long
Ruijun Long
Zachary Long
Xiaochun Long
Yong Long
Run Long
Hong Long
Wenxing Long
Joanna Long
Jinyi Long
Di Long
Jingyi Long
Marcus Long
Jed Long
Erping Long
Elizabeth Long
Gang Long
Scott Long
Jeff Long
Chunlin Long
Mingsheng Long
Huiling Long
Hao Long
Teri Longacre
Michael Longaker
Adhemar Longatto-Filho
Marieke Longcamp
Ben Longdon
Bernard Longdoz
Nick Longford
Maria Longhi
Daniel Longley
Chris Longmore
Benedetto Longo
Lawrence Longo
Francesca Longo
Gary Longo
Anna Longo
Alberto Longo
Sarah Longo
Patrizia Longone
Nicholas Longrich
Nick Longrich
Robert Lonigro
Jennie Lönnbro Widgren
Christina Lönnroth
Oona Lönnstedt
Tina Lonsdorf
Elizabeth Lonsdorf
Lit-Hsin Loo
Torey Looft
Marco Loog
Chung Yeng Looi
Katharine Looker
Todd Lookingbill
Holli Loomans
Brett R. Loomis
Mark Looney
Juan Loor
Huib Looren De Jong
Bruno Loos
Maarten Loos
Brendan Looyenga
Vladimir Loparev
Joseph Loparo
Andreas Lopata
Joyce Loper
Joao Lopes
Lucia Lopes
Carina Lopes
Marcos Lopes
Antonio Lopes
Carla Lopes
José Lopes
Luisa Lopes
Hedibert Lopes
Silvio Lopes
Ana Isabel Lopes
Ana Lopes
Fernando H Lopes Da Silva
José Lopes De Faria
André Lopes Fuly
Leila Lopes-Bezerra
Vitor Lopes-Dos-Santos
Loris R. Lopetus
Anna Lena Lopez
Alvaro Sanchez Lopez

Marta Lopez
Regulo Lopez
J. Lopez
Patricia López
María López
Maria Celeste Lopez Abbate
Raquel López -Antoñanzas
José López Bucio
Evangelina López De Maturana
Adolfo López De Munain
Martin Lopez Garcia
Guillermo López Lluch
Daniel López Malo
Víctor Eric López Y López
Sandra Lopez-Arana
Adriana López-Arbarello
Antonio Lopez-Beltran
Abel López-Bermejo
Al Lopez-Bueno
Alberto López-Bueno
Cesar López-Camarillo
J. L. López-Campos
Gloria López-Casado
Eduardo López-Collazo
Juan Carlos Lopez-Delgado
Nestor Lopez-Duran
Jose Lopez-Escamez
Rosina López-Fandiño
Jose Antonio Lopez-Guerrero
Jesus Lopez-Herce
Francisco Lopez-Hernandez
Luz Lopez-Hernandez
Marcos López-Hoyos
Patricio Lopez-Jaramillo
Pia López-Jornet
Liliana López-Kleine
F. Xavier Lopez-Labrador
Eduardo López-Larraz
Luis Lopez-Llorca
Lucia Lopez-Lopez
Giancarlo López-Martínez
R. López-Marure
Ana-Flor López-Millán
Jose Lopez-Miranda
Luis Lopez-Molina
Carlos Lopez-Molina
Rodrigo López-Muñoz
Horacio Lopez-Nicora
Jose Lopez-Olmeda
Jorge Ramón López-Olvera
Citlali Lopez-Ortiz
Mario Lopez-Perez
Julio Lopez-Picazo
Jose Lopez-Rubio
Pablo Lopez-Soto
Elisa Lopez-Varela
Hector Lopez-Vergara
Frantisek Lopot
Jorge Lora
Irene Lorand-Metze
Vincent Lorant
J. Lora-Tamayo
Haya Lorberboum-Galski
Gwendolen Lorch
Janet Lord
Christopher Lord
Kevin Lord
Anton Lord
Stephen Lord
Tessa Lord
Kathryn Lord
Olivier Loreal
Theo Lorenc
Mar Lorente
Nicolas Lorente
José A. Lorente
Aaron Lorenz
Kristina Lorenz
Klaus Lorenz
Teresa Lorenz
Jan Lorenz
Kent Lorenz
Tierney Lorenz
Matthias Lorenz
Johan Lorenzen
Julio Cesar Lorenzi
Christian Lorenzi
Tommaso Lorenzi
M. Nieves Lorenzo
Bramanti Lorenzo
Michele Lorenzo
Paula Lorenzo
Ramon Lorenzo-Redondo
Vittorio Loreto
Matthias-Claudio Loretto
Sylvain Loric

Rik Lories
Balazs Lorincz
Monicque Lorist
Stefan Loroch
Gauthier Loron
Hugues Lortat-Jacob
Aurélien Lorthioir
Philippe Lory
Irena Loryan
Marek Los
Dmitry Los
Giovanni Losano
Wolfgang Löscher
Julia Loseff-Silver
Mario Losen
Aba Losi
Naida Loskutoff
Thomas Losnegård
Guilherme Loss
Amy Lossie
Andreas Lossius
Kristina Lossow
Anabel Lostao
Tamar Lotan
Sebastian Loth
Katie Loth
Achim Lother
Anne Lotz
Christopher Lotz
Sebastian Lotz
Martin Lotze
Yonggen Lou
Zhenkun Lou
Hongxiang Lou
Jie Lou
Yilai Lou
Qing Lou
Thomas Louail
Eric Loucks
Janice Lough
John Loughlin
Zachary Loughman
Siobhan Loughna
Katherine Louie
Julien Louis
Petra Louis
Joe Louis
Abner Louissaint
Yannis Loukas
Eugen Lounkine
Gilles Louppe
Iñigo Loureiro
Anete Lourenco
Barbara Lourenco
Lillian Lourenco
Anália Lourenço
Wilson Lourenço
André Lourenço
Sebastian Lourido
Denis Loustau
Isabelle Louveau
A. Louw
Quinette Louw
Julia Louw
Rogier Louwen
Vitor Louzada
Yoram Louzoun
William Lovallo
Sandro Lovari
Maria Lovato
Karl-Olof Lovblad
Martin Lövdén
John Love
Nancy Love
Michael Love
Jason Love
Ian M. Love
Ryan Love
Marian Loveday
Gabor Lovei
Connie Lovejoy
Erica Lovelace
Katherine Loveland
Mark Lovell
Nancy Lovell
John Lovell
Vanessa Lovenburg
Andrew Lovering
Ruth Lovering
Susan Lovett
Michael Lovett
Peter Lovibond
Frank Lovicu
Timothy Loving
Jenny Low
David Low

Eng-Ti Low
Daren Low
Siew Kee Low
Dorrain Low
Sue Low
Chien Tat Low
Todd L. Lowary
Jan Lowe
Martin Lowe
Chris Lowe
Andrew Lowe
Rachel Lowe
Jennifer Lowe
Samantha Lowe
Jean Lowe
David Lowe
Rohan Lowe
Robert Lowe
Clifford Lowell
Mark Lowell
Anice Lowen
Charles Lowenstein
Pedro Lowenstein
Jerome Lowenstein
Elizabeth Lowenthal
Aoife Lowery
Noel Lowndes
Michael Lowry
Andrew Lowther
Judy Lowthian
Prashant Loyalka
Alejandro Loydi
Alejandra Loyola
Oscar Lozano
Sergi Lozano
Jesus Lozano
Yudi M Lozano
Omar Lozano
Miguel Lozano
Roberto Lozano
Jose Lozano-Torres
Olga Lozovskaya
Laura Lozza
Shan Lu
Tsung-Hsueh Lu
Ying-Jie Lu
Hua Lu
Sangwei Lu
Mengji Lu
Chun Lu
Wei Lu
Jianrong Lu
Shemin Lu
Yanli Lu
Jun Lu
Yanhui Lu
Wuyuan Lu
Zheming Lu
Hongzhou Lu
Houyuan Lu
Jang-Jih Lu
Hong Lu
Haojie Lu
Jinsong Lu
Ming-Wei Lu
Jiachun Lu
Wenfu Lu
Ling Lu
Lei Lu
Da-Wen Lu
Mengzhu Lu
Lin-Yu Lu
Chen Lu
Wange Lu
Yongbo Lu
Pei-Hua Lu
Shena Lu
Dah-Yuu Lu
Liang-Jing Lu
Sheng-Nan Lu
Ching-Hua Lu
Yong-Jie Lu
Gang Lu
Bo Lu
Liqun Lu
Aiping Lu
Cheng-Hsien Lu
Qingxian Lu
Liming Lu
Bin Lu
Tsai-Ching Lu
Jie Lu
Zhongxian Lu
Fan Lu
Zhong-Qiu Lu
Tao Lu

Ping Lu
Paul Lu
Chia-Chen Lu
Zheng-Feng Lu
Dan Lu
Xiaotao Lu
Guo-Dong Lu
Xiaochun Lu
Mingyang Lu
Kun Lu
Mujun Lu
Yingchang Lu
Zhijun Lu
Liwei Lu
Qing Lu
Yuan Lu
Weiguo Lu
Zhi-Xiang Lu
Huayu Lu
X. Lucas Lu
Jibao Lu
Yanrong Lu
Aitao Lu
Xiuling Lu
Qingjun Lu
Congming Lu
Jingqiao Lu
Peilin Lu
Rongxing Lu
Wang Lu
Yunfeng Lu
Zhe Lu
Yonggang Lu
Shun Lu
Lizhi Lu
Guojun Lu
Peng Lu
Tzu-Pin Lu
Changming Lu
Yin Lu
Rongwen Lu
Haitao Lu
Yi Lu
Chin-Song Lu
Yu Lu
T. J. Lu
Min Lu
Yi-Han Lu
Zhimin Lu
Caiyan Lu
Shien Lu
Yang Lu
Miao Lu
Xiao-Hong Lu
Wenhua Lu
Chun-Yi Lu
Jiqi Lu
Hongyun Lu
Lilin Lu
Gui-Ning Lu
Jianquan Lu
Shiang-Ru Lu
Feng Lu
Meng Lu
Nanxi Lu
Stephen Lu
Huimin Lu
N Lu
Ning Lu
Xiaoling Lu
Kai Lu
Zhengfei Lu
Chung-An Lu
He-Zuo Lü
Peijun Lü
Angelique Luabeya
Xianghong Luan
Shenghua Luan
Sheng Luan
Yun-Xia Luan
Junbo Luan
Shengji Luan
Nantasit Luangasanatip
Louis Luangkesorn
Sudjit Luanpitpong
Todd Lubart
Przemyslaw Lubiatowski
Steven Lubitz
Katrin Lübke
Anna Lubkowska
Ruben Lubowski
Stephen Luby
Antonio Lucacchini
Radek Lucan
Mark Lucanic
Sebastian Lucas

Gregory Lucas
Robert Lucas
Sophie Lucas
Michaela Lucas
Bruno Lucas
Richard Lucas
Edralin Lucas
Cathy Lucas
Andrew Lucas
Christine Lucas
Françoise Lucas
Brian Lucas
Leilani Lucas
Guilherme Lucas
Timothy Lucas
Ana M. Lucas
Andrea Lucas-Hahn
Paul Lucassen
Alessandro Lucchetti
Giancarlo Lucchetti
Chiara Lucchi
Juan Lucena
Livia Lucentini
Jean-Christophe Lucet
Patrick Lucey
Claudio Luchini
Anne-Marie Lüchtenborg
Dan Luciani
Michelle Luciano
Vanessa Lucieer
Julie Lucifora
Luigi Lucini
Diana Lucio-Arias
Marília Lucisano
Gaetano Lucisano
Gary Luck
Jayne Lucke
Till Luckenbach
Mark Luckenbach
Sebastian Lücker
Shirley Luckhart
Michaela Luconi
Tyrone Lucon-Xiccato
Miha Lucovnik
Magdalena Luczak
Aneta Luczkiewicz
Christina Ludema
Carsten Lüder
Ulrike Luderer
Eileen Luders
Jens Lüders
Ingo Ludolph
Jean Ludovic
Richard Luduena
Jonas Ludvigsson
Björn Ludwar
Arne Ludwig
Marie-Gabrielle Ludwig
Andreas Ludwig
Ralf Ludwig
Michelle Ludwig
Bernd Ludwig
Frank Ludwig
Jutta Ludwig-Mueller
Neal Lue
Aaron Luebbe
E. Luebeck
Laura Luebke
Katharina Lueck
Tom Luedde
Reinhard Luehrmann
Ulrike Lueken
Alison Luengen
Andreas Lueschow
Felix Luessi
Marc Luetgehetmann
Jose Ruben Luevano Enriquez
Andreas Luft
Friedrich Luft
Micah Luftig
Vipul Lugade
Fulgentius Lugemwa
Eva Luger
Geanncarlo Lugo
Joaquin Lugo
Jocemir Lugon
Luis Lugones
Heiko Luhmann
B. L. Luhovvy
Arthur Luhur
Su Lui
Kai Lui
Camillia Lui
Maartje Luijten
Alberto Luini
Nuno Miguel Luis

Luca Luiselli
Rosalie Luiten
Wilson Luiz
Marla Lujan
H. M. Luk
Martin Lukac
Aron Lukacher
Nicholas Lukacs
Paul M. Lukacs
Dieter Lukas
Marcus Lukas
Jan Lukas
Anna Lukasik
Katarzyna Lukasiuk
Henry Lukaski
Aaron Lukasweski
Anne-Claire Lukaszewicz
Cliff Luke
Timothy Luke
Nada Lukkahatai
Jasper Lukkezen
Slawomir Lukomski
Robert Lukowski
Gediminas Luksys
Sergey Lukyanov
Dorothée Lulé
Judy Lulich
Matteo Lulli
Julian J. Lum
Fok Moon Lum
Sooky Lum
Sarah Lummis
K. Lumniczky
Jim Lumsden
Mary Ann Lumsden
Saisamorn Lumyong
Zhao-Rong Lun
Wenhui Lun
Gian Luna
Elizabeth Luna
Beatríz Carely Luna
Stelio Luna
Jose D Luna Pinto
Alejandro Isabel Luna Luna-Maldonado
Oscar Luna-Martinez
M Lunar
Klaus Lunau
Elsebet Lund
Amie Lund
Gertrud Lund
Jay Lund
Sigrid Lundberg
Roger Lundblad
Ida Lunde
Taina Lundell
Mojca Lunder
Steinar Lundgren
Torbjorn Lundh
Knut Lundin
Lars Lunding
Andreas Lundqvist
Brian Lundstrom
Johan Lundstrom
John Lunec
Sebastian Lunemann
Jan Lünemann
Kathryn Lunetta
Maria Lung
Rodica Lung
Hl Lung
Tom Lung
Paul Lunn
Dan Lunney
Mariia Lunova
Ma Luo
Cheng Luo
Gaoxing Luo
Zhenge Luo
Zhijun Luo
Tao Luo
Jiankai Luo
Dahai Luo
Guangbin Luo
Xiaoying Luo
Wen-Jing Luo
Jianyuan Luo
Zhe-Xi Luo
Zhong-Cheng Luo
Ruoyu Luo
Jun Luo
Chunming Luo
Weijia Luo
Yi-Bo Luo
Yangmei Luo
Ruibang Luo
Na Luo

Meizhong Luo
Nan Luo
Zhibin Luo
Zhehui Luo
Shu-Jin Luo
Pei Luo
Hongli Luo
Chunling Luo
Yunhai Luo
Jingqin Luo
Xiaoyan Luo
Jiangyun Luo
Jie Luo
Qing Luo
Mingyue Luo
Xiong-Jian Luo
Ailin Luo
Lihui Luo
Dan Luo
Yinghua Luo
Qiong Luo
Xiao-Xing Luo
Shi-Xiao Luo
Qingfei Luo
Siyang Luo
Zisheng Luo
Chengwen Luo
Sheng Luo
Lixin Luo
Yu-Heng Luo
Weibo Luo
P. Luo
Haiwei Luo
Weijun Luo
Yuping Luo
Rongcheng Luo
Hongbin Luo
Yongfeng Luo
Yan Luo
Gang Luo
Xunda Luo
Yu Luo
Kaijun Luo
Xiaolin Luo
Keming Luo
Ruibai Luo
Cai Luo
Livio Luongo
Antonella Lupetti
Christopher Lupfer
Carl Lupica
Stephen Lupker
Vincenzo Lupo
Shawn Lupold
Mikulas Luptacik
Daniel Lupu
Marian Lupulescu
Gary Lupyan
Irene Luque
Daniel Luque
Francisco Luque
David Luque
Maria José Luque
Antoni Luque
A Luque-Suarez
Frederick Lurmann
Peter Lurz
Donal Luse
Lucy Lush
Gerald Lushington
Kurt Lushington
Marina Lusic
Aldons Lusis
Christopher Lusk
Jayson Lusk
Bethany Lusk
Matthew Luskin
Katherine Lust
Maryam Lustberg
Molly Lutcavage
Ken Lutchen
Glen Lutchman
Heidi Luter
Carsten Lüter
Lesley Lutes
Georges Lutfalla
Elizabeth Lutge
Esther Lutgens
Jodie Lutkenhaus
Dominique Luton
Svetlana Lutsenko
Olha Lutsiv
Erika Lutter
Carol Lutz
Norbert Lutz
Antoine Lutz

Thomas Lutz
Michael Lutz
Jens Lutz
Kai Lutz
Joseph Lutz
Chi Luu
Keijo Luukko
Jeffrey Luvall
Siro Luvisetto
Marc Luwel
Koen Luwel
Victoria Lux-Lantos
G.W. Gant Luxton
Valerie Luyckx
James Luyendyk
Sebastiaan Luyssaert
Paula Luz
Christian Luz
Mariana Luz
Agustin Luz Madrigal
Francesca Luziatelli
Yating Lv
Guohua Lv
Fubing Lv
Wenhua Lv
Bin Lv
Jinglei Lv
Cheng Ly
Hinh Ly
Sonny Ly
Vijay Lyall
Meghan Lybecker
Viveka Lyberg-Åhlander
Samantha Lycett
Stephen Lycett
David Lydall
Patrick Lyden
Ralph Lydic
Patrick Lydon
Leonard Lye
Craig Lygate
Johan Lyhagen
Amy Lykins
Edward Lyman
Lee Lyman
Anastasios Lymperopoulos
Antony Lynam
Jeremy Lynch
John Lynch
Christopher Lynch
Matthew Lynch
Conor Lynch
Michael Lynch
Iseult Lynch
Rebecca Lynch
Gerard Lynch
Lori Lynch
Thomas Lynch
Audrey Lyndon
Salvador Lyngdoh
Elsebeth Lynge
Denis H. Lynn
James Lynn
Geoffrey Lynn
Shina Caroline Lynn Kamerlin
Niels Lynnerup
Gholson Lyon
Caroline Lyon
Karen Lyons
Robert Lyons
Traci Lyons
Jeri-Anne Lyons
David Lyons
Ian Lyons
Scott Lyons
Sarah Lyons
Tim Lyons
Graham Lyons
Karen Lyons-Ruth
Monica Lypson
Martin Lysak
Inna Lysnyansky
Valeriya Lyssenko
Lisa Lyssenko
Grant Lythe
Theodore Lytras
Zhe Lyu
Haijun Lyu
Yuri Lyubchenko
Julia Lyubovitsky
Xiaojing Ma
Qi-Fu Ma
Stefan Ma
Changxing Ma
Lu Ma
Fei Ma

Averil Ma
Yongxin Ma
Bin Ma
Jie Ma
Xiaoguang Ma
Keping Ma
Xuefei Ma
Daqing Ma
Zhihai Ma
Weihua Ma
Qin Ma
Edmond S K Ma
Keyi Ma
Bing Ma
Yongqing Ma
Ke Ma
Jincai Ma
Yongjie Ma
Chuang Ma
Jin Yeul Ma
Xiaotu Ma
Zhiying Ma
Zhonghua Ma
Lina Ma
Hui Ma
Jianhua Ma
Li Jun Ma
Stephanie Ma
Jun Ma
David Ma
Hongyu Ma
Xin-Rong Ma
Chi Ma
Xinghong Ma
Peisong Ma
Yijie Ma
Jun-Yu Ma
Tianle Ma
Yibing Ma
Chao Ma
Xingyi Ma
Wei Ma
Qingguo Ma
Shizhan Ma
Qi Ma
Jinjin Ma
Chuanxi Ma
Huailiang Ma
Dongyun Ma
Liangsuo Ma
Hongming Ma
Hk Ma
Quanfu Ma
Yiming Ma
Yan-Qing Ma
Zhiqiang Ma
Alan Ma
Yan Ma
Ming-Chieh Ma
Zhenjun Ma
Xian-Cang Ma
Zhiwei Ma
Liang Ma
Ying Ma
Wen-Lung Ma
Tao Ma
Cindy S Ma
Guojia Ma
Wen Ma
Xiaolei Ma
Wei-Li Ma
Feng Ma
Shi-Liang Ma
Xufa Ma
Baojun Ma
Guolin Ma
Patrick Ma
Xin-Liang Ma
Yuanmei Ma
Shujie Ma
Dongming Ma
Nianhan Ma
Zhengliang Ma
Yitong Ma
Xuejun Ma
Zhijun Ma
Junfeng Ma
Yanling Ma
Wenxiu Ma
Christoph Maack
Steffen Maak
Thomas Maal
Reza Maali Amiri
Raoel Maan
Coen Maas
Renke Maas

Angela Maas
Rutger Maas
Karen Maass
A. Maat
Hadi Maazi
Neil Mabbott
David Mabey
Geraldine Mabille
Guillaume Mabilleau
Nicola Mabjeesh
Barbara Mable
Fazle Mabood
Feilim Mac Gabhann
Caoimhin Mac Giolla Phadraig
Tim Mac Gloughlin
David Mac Lean
John Mac Sharry
Filippo Macaluso
Michelle Macaraig
Heather Macarthur
Bernard Jonas Macatangay
Calum Macaulay
Robert Macaulay
Matthew Macauley
Angus Macbeth
Cora Macbeth
Paul Maccabee
Simone Maccaferri
Cristina Maccalli
Donna Maccallum
Matthew Maccani
Sarah Maccarthy
Thomas Maccarthy
Roberto Macchiarelli
Guido Macchiarelli
Nicolo Macciotta
Kelly Macdonald
Margaret Macdonald
Raymond Macdonald
Patrick Macdonald
Stuart Macdonald
Ian Macdonald
Ellen Macdonald
Suzanne Macdonald
Robert Macdonald
Justin Macdonald
Sara Macdonald
David Macdonald
Douglas Macdonald
Scott Macdougall-Shackleton
Ruth Mace
Camille Mace
Oliver Mace
Etienne Macedo
Antonio Filipe Macedo
Angela Macedo
Luis Macedo-Soares
Mark Macek
Sean Macevoy
Sarina Macfadyen
Janet Macfall
Todd Macfarlan
Anne Macfarlane
Nuria Mach
Andre Machada
Ednildo Machado
Roberto Machado
Paulo Machado
Fernanda Machado
Daniel Machado
Heather Machado
Rosangela Machado
Mariana Machado
T.D. Machado
José Machado
Gisele Machado
Glaucia Machado-Santelli
Rodrigo Machado-Vieira
Janet Macher
Marie-Hélène Macherel
O. Macherey
Edouard Machery
Cristina Machial
Keigo Machida
Curtis Machida
Ryuji Machida
Takuji Machida
Tatsuya Machida
David Machin
Karen Machin
Maria Teresa Machini
Raghu Machiraju
Irma Machuca-Gayet
Javier Macia
Diego Macias
Michelle Macias

Emma Del Carmen Macías-Cortés
Marina Macias-Silva
Marcin Maciejczyk
Mateusz Maciejewski
Kristine Maciejewski
Thiago Maciel
Rafael Maciel-De-Freitas
James Macinko
Janet Macinnes
Martin Macinnis
Catriona Macinnis-Ng
Andrew Macintosh
Bradley Macintosh
Kate Macintyre
Salvador Macip
Hugh Macisaac
Rachael Macisaac
Richard Macisaac
Malgorzata Maciukiewicz
M Bruce Maciver
J. Scott Macivor
Matthias Mack
David Mack
Natasha Mack
Andreas Mack
Gary Mack
Julia Mackaronis
Trudy Mackay
Donna Mackay
D. Scott Mackay
Alex Mackay
Dylan Mackay
Johan Mackenbach
Charles Mackenzie
Jason Mackenzie
Todd Mackenzie
Gerardo Mackenzie
Colin Mackenzie
Bryan Mackenzie
S.J. MacKenzie
Catrina MacKenzie
Alexander MacKerell
Stephen Mackessy
Tim Mackey
Rachel Mackey
Dawn Mackey
Roderick Mackie
Pawel Mackiewicz
Colum MacKinnon
Joan Mackintosh
Shylie Mackintosh
James Macklin
Paul Macklin
Mike Mackness
Stephen Macknik
Erich Mackow
Adam Mackridge
James MacLachlan
Liam MacLachlan
Julian Maclaren
Evan MacLean
Daniel MacLean
Kenneth Maclean
Sarah MacLean
Joanna MacLean
Michael MacLellan
Malcolm MacLeod
Stuart MacLeod
Bethany MacLeod
Kirsty MacLeod
Toran MacLeod
Matthew MacLeod
Alan MacLeod
Greg MacLeod
Frank MacMaster
Harriet MacMillan
Douglas Macmillan
Colleen MacMillan
Katherine MacNamara
Sheila MacNeil
Paul MacNeilage
Jill Macoska
L. Macovei
Catherine MacPhail
Paul MacPherson
Alison MacPherson
Peter MacPherson
Eleanor MacPherson
Kerry MacQuarrie
Chris MacQuarrie
Daniel Macqueen
Thomas MacRae
C. Neil Macrae
Merrin Macrae
Christopher Macraild
Peter Macreadie

Nathalie Macrez
Simone Macri
Slobodan Macura
Libor Macurek
Brian Macwhinney
Luke Macyszyn
Anant Madabhushi
Magbubah Madadha
Behrouz Madahian
Satish Madala
Regina Madalozzo
Vanesa Madan
Kaveh Madani
Nima Madani
Pradeepa Madapura
Chris Madden
Greg Madden
Andrew Madden
Jeanne Madden
Maxime Madder
Krishna Rao Maddipati
Todd Maddox
Ross Maddox
Keith Maddox
Brenna Maddox
Mohan Maddur
Kamesh Madduri
Jean-Yves Madec
Giordano Madeddu
Fabio Madeddu
Paolo Madeddu
Pedro Madeira
María Madeira
Luciana Madeira Da Silva
Simone Mader
Juan Madera
Sheshu Madhav
Deepak Madhavan
Ashraf Madhoun
Mohammad Madhoun
Mayank Madhra
Michele Madigan
S.R. Murthy Madiraju
Tobias Madl
M. Madlena
Stuart Madnick
David Madoff
Rosalinda Madonna
F. Madore
Joseph Madri
Irene Madrigal
Henning Madry
Sten Madsbad
Jens Madsen
Jesper Madsen
Soren Madsen
Mark Madsen
Anazoeze Madu
Francisco Madueno
Jeffry Madura
Anotida Madzvamuse
Norikazu Maeda
Shin Maeda
Tatsuya Maeda
Kazuya Maeda
Eijiro Maeda
Yoshinobu Maeda
Akiko Maeda
Ken Maeda
K. Maeda
Hidefumi Maeda
Yoshihiko Maehara
Rene Maehr
Yasuhiro Maejima
Shinya Maekawa
Toshihiko Maekawa
Emilia Maellaro
Koji Maemura
Pil Jae Maeng
Sumiaki Maeo
Andreas Maercker
Charline Maertens
Gregory Maes
Piet Maes
Michael Maes
Satoshi Maesawa
Masayoshi Maeshima
Elena Maestri
Michelangelo Maestri
Elena Maestrini
Anna Maestroni
Ceferino Maestu
Tobias Maetzig
Massimo Maffei
Francesco Maffessanti
Michele Maffia

Andrea Mafficini
Tania Maffucci
Nicola Maffulli
Valéria Mafra
Denise Mafra
Susana Magadan-Mompo
Nicolas Magain
Marco Magalhaes
Ana Magalhães
Fernando Magalhães
Sandra Magaña
Everett Magann
Rama Maganti
Jules Magda
Frédérique Magdinier
Jesse Mager
Melissa Mageroy
Aude Magerus-Chatinet
Mario Maggi
Elaine Maggi
Ivan Maggini
Nicola Maggio
Mayara Fernanda Maggioli
Marcello Maggiolini
Franco Maggiolo
Aldo Maggioni
Martina Maggioni
Umberto Maggiore
Jennifer Maggs
Ghassan Maghzal
Peter Magill
Thomas Magin
Parker Magin
Tara Maginnis
Gkikas Magiorkinis
Emmanouil Magiorkinis
David Magis
Angeliki Magklara
Adriano Magli
Dangelo Magliano
Nicholas Magliocca
Anthony Magliocco
Antoine Magnan
Mauro Magnani
Jared Magnani
Nicola Magnavita
David Magne
Ronald Magness
Fulvio Magni
Paolo Magni
Julie Magno Zito
Marco Magnoni
Brooke Magnus
David Magnuson
Costan Magnussen
Nils Magnusson
William Magnusson
Ulf Magnusson
Borgthor Magnusson
Hiroshi Magome
Lilia Magomedova
Stefano Magon
Sergey Magonov
Jordi Magrané
Vincent Magrini
Jean-Philippe Mague
Orla Maguire
Maureen Maguire
Gerald Maguire
Sarah Maguire
Laurent Magy
John Magyar
Christopher Mah
Allison Mah
Liviu-George Maha
S. Mahadev
Mani Mahadevan
W. F. Mahaffee
Nupam Mahajan
Vinit Mahajan
Raman Mahajan
Sidhartha Mahali
Suresh Mahalingam
Sundarasamy Mahalingam
John Mahan
James Mahan
Nitish Mahapatra
Bidhubhusan Mahapatra
Tanmay Mahapatra
Sebabrata Mahapatra
Dwarikanath Mahapatra
Sadhan Mahapatra
Praful Maharana
Bijesh Maharjan
Sushil Mahata
Ram Mahato

Martyn Mahaut-Smith
Kamal K. Mahawar
H. Mahdi
Mohammed Mahdy
Coline Mahende
Jaideep Mahendra
Ratha Mahendran
Eshwar Mahenthiralingam
Sean Maher
Carol Maher
Bill Maher
Stephen Maher
Marcus Maher
Hafiz Maherali
M. S. Mahesh
Akhil Maheshwari
Radha Maheshwari
Dinesh Maheshwari
Rajat Maheshwari
Samuel Maheswaran
Nadia Mahfoudh
Jacques Mahillon
Amit Mahipal
Anne-Katrin Mahlein
Yasser Mahmmoud
Riaz Mahmood
Iftekhar Mahmood
Ahmed Mahmoud
Barakat Mahmoud
Shereif Mahmoud
Akrama Mahmoud
Abou Alaiwa Mahmoud
Abdelrahman Mahmoud
Nadia Mahmoudi Khatir
Hossein Mahmoudvand
Abdullah Mahmud
Mastura Mahmud
Mehmet Mahmut
Alexander Mahnert
Hassan Mahomed
Fawzi Mahomoodally
Robin Mahon
My Mahoney
J. Matthew Mahoney
Meredith Mahoney
Omar Mahroo
Hai-Qiang Mai
Knut Mai
Xiao-Mei Mai
Shi-Juan Mai
Carla Maia
Ivan Maia
Cristiane Maia
Valéria Cid Maia
Mari Maia Da Silva
Juan Pablo Maianti
Howard Maibach
Martine Maibeche-Coisne
Daniel Maidana
Susannah Maidment
Christoph Maier
Marc Maier
Uwe Maier
Jeanette Maier
Manfred Maier
Berenike Maier
Larissa Maier
Andreas Maier
Martin Maier
Bernhard Maier
Markus Maier
Barbara Maier
Klaus Hermann Maier-Hein
Amelia Maika
Jean-Yves Maillard
Michel Maillard
Arnaud Mailleux
Robbie Mailliard
Ryan Mailloux
Eric Maimela
Russell Main
Daniel Maina
Gediminas Mainelis
Philippe Maingon
Mark C. Mainwaring
P.Stanely Mainzen Prince
Margherita Maioli
Sabatino Maione
Maria Maiorino
Johannes Mair
Gunnar Mair
Eva Maire
Klemens Mairer
Tim Maisch
Marion Maisonobe
Debasish Maiti

Panchanan Maiti
Kristen Maitland
Anke Maitland-van der Zee
Brian Maitner
Arindam Maitra
Anurupa Maitra
Nathalie Maitre
Sankar Maity
Bettina Maiwald
Marian Majchrzycki
Antonio Majdandzic
Omid Majdani
Jeannine Majde
Reza Majdzadeh
Barbara Majello
Tamsin Majerus
Matthias Majetschak
Ian Majewski
Martin Majewski
Aman Shah Abdul Majid
Shideh Majidi
Richard Major
Matthew Major
Edyta Majorczyk
Fiona Majorin
Shannon Majowicz
Ireneusz Majsterek
Subeer Majumdar
Subrata Majumdar
Shyamasree Majumdar
Gipsy Majumdar
Avijit Majumdar
Paromita Majumder
Rinku Majumder
Joseph Majzoub
Kin Cheung Mak
Margaret Mak
Kepher Makambi
Andrew Makanya
Helen Makarenkova
A. Makarevic
Cheryl Makarewicz
Jyrki Makela
Keijo Makela
Eugene Makeyev
Govind Makharia
Dennis Maki
Takakuni Maki
Seicho Makihira
Suwa Makiko
Ermei Mäkilä
Tamar Makin
Guy Makin
Stephen Makin
Kristiina Makinen
Ville-Petteri Makinen
Taija Makinen
Netta Mäkinen
Ayako Makino
Yuichi Makino
Shigeki Makino
T. Makino
Hideki Makinoshima
Hideki Makishima
Tomoko Makishima
Takeru Makiyama
Steve Makkar
M. Makki
Mokhantso Makoae
Peter Makovicky
Tal Makovski
Michael Makowsky
Guido Makransky
Konstantinos Makridis
Alexandros Makriyannis
Hernán Makse
Johanna Maksimainen
Geoffrey Maksym
Alexey Makunin
Asoke Mal
Juan Malacara
Katerina Malagari
Fabienne Malagnac
Talía Malagón
Ángeles Malagón-Amor
Roberta Malaguarnera
Lucia Malaguarnera
Udayabanu Malairaman
Willy Malaisse
Dhruba Malakar
Georgia Malamut
Laurence Malandrin
Noemi Malandrino
Umberto Malapelle
Lorenzo Malatino
Luc Malaval

Fabio Malavasi
Stéfano Malavasi
Marco Malavasi
Iran Malavazi
Gathsaurie Malavige
Marcella Malavolti
Luiz Malbouisson
Marzia Malcangio
Kenneth Malcolm
R. Karl Malcolm
Philippe Malcolm
Hamish Malcolm
Frank Maldarelli
Manuel Maldonado
Yolanda Munoz Maldonado
Vilma Maldonado
Maricela Maldonado
Joel Malek
Mojtaba Malek
Goldis Malek
Naveed Malek
Angela Malek
Moh Malek
Soheila Maleki
Afsaneh Maleki-Dizaji
Reza Malekzadeh
Charles Malemud
Gertrud Malene Hjorto
Francesca Malentacchi
Paola Malerba
Ken Maleta
Jozsef Maleth
Nicole Malfait
Fransiska Malfait
Anne-Marie Malfait
Peter Malfertheiner
Anneleen Malfliet
Assumpció Malgosa
Harmeet Malhi
Pawan Malhotra
Arun Malhotra
Indu Malhotra
Bansi D. Malhotra
Sony Malhotra
Samir Malhotra
Ajai Malhotra
Deepak Malhotra
Ivana Mali
Jarema Malicki
Tessy Maliekal
Ahmad Malik
Rohit Malik
Ausama Malik
Fayaz Malik
Amyn Malik
Rayaz Malik
Steven Malin
David Malinvaud
Robert Malison
Laurent Malisoux
Irina Maljkovic Berry
David Malkin
Dan Malkinson
Marcus Mall
Rajesh Mall
Kimberley Mallan
Surya Mallapragada
S. Mallavarapu
Ernst Malle
Bertram Malle
Yassine Mallem
Benoit Malleret
Robert T. Mallet
Frédérick Mallette
Roland Malli
Luca Mallia
Maria Malliarou
Thérèse Malliavin
Parag Mallick
Anastasia Mallidou
Prabodhika Mallikaratchy
Karthik Mallilankaraman
Rachel Mallinger
Meni Malliori
Dermot Mallon
David Mallon
Jordan Mallon
James Mallory
Mark Mallory
Hanspeter Mallot
Elizabeth Mallott
Athanasios Mallouchos
Philip Malloy
Michael Mallozzi
Christer Malm
Kalle Malmberg

Juanita Malmberg
Anders Malmström
Bettina Malnic
Mickael Malnoy
Noel Malod-Dognin
Jacob Malone
David Malone
Sparkle Malone
Christopher Maloney
Ryan Maloney
John Malouff
Alina Maloyan
Mats Målqvist
Monica Malta
Maira Malta
Jansen Malte
Emin Maltepe
Adam Maltese
Davide Maltoni
Victor Maltsev
Marcos Malumbres
E. Malusa
J. Mckim Malville
Boris Malyarchuk
Denis Malyshev
J. Malyszko
Khursheed Mama
Mamas Mamas
Adam Mamelak
Asgeir Mamen
Srinivas Mamidi
Irene Mammarella
Andrew L. Mammen
Caterina Mammina
Zaid Mammo
Akiko Mammoto
John Mamo
Hassen Mamo
Gianfranco Mamone
Tomasz Mamos
Hadii Mamudu
Diamanto Mamuneas
Si Ming Man
Jianguo Man
Kenneth Man
Yi Man
Ichiro Manabe
Donal Manahan
Celia Manaia
Emmanuel Manalo
Mark Manary
O. Manathip
Daniele Mancardi
Charles Manceau
Steven Manchester
Veronika Mancikova
Jorge Mancini
Flavia Mancini
Maria Vittoria Mancini
Patricia L. Mancini
Martina Mancini
Rita Manco
Massimo Mancone
Mauro Manconi
Antonella Mancusi
Cesare Mancuso
Patrizia Mancuso
Michelangelo Mancuso
Raivo Mänd
Nawajes Mandal
Diptasri Mandal
Chitra Mandal
Kousik Mandal
Chandi C Mandal
Bidisha Mandal
Asit Baran Mandal
Atin Mandal
Sema Mandal
A. Mandal
Subhrangsu Mandal
Abul Kalam Azad Mandala
Manolis Mandalakis
Sundhiya Mandalia
Reet Mändar
Carlos Mandarim-De-Lacerda
Lucinda Manda-Taylor
Yossi Mandel
David Mandel
Roi Mandel
Avigdor Mandelberg
Laura Mandelli
John Mandelman
Vera Manders
Kate Mandeville
Sandy Mandic
Pierre Mandin

Giuseppe Mandraffino
Mauro Mandrioli
Suzanna Mandruzzato
Antonia Manduca
William Mandy
Chitra Mandyam
Arati Mane
Loreen Mane
Adrian Manea
Jose Ramon Maneiro
Anna Manelis
Emanuel Manesis
Nicholas Maness
Pooja Maney
Angelo Manfredi
Fabio Manfredini
Chiara Manfrin
Olivia Manfrini
John Manful
Vijjulatha Manga
Lingegowda Mangala
Ionel Mangalagiu
Michael Mangan
Louis Manganas
Sara Manganelli
Stefano Mangani
Alberto Mangano
Carlo Mangano
Anne Mangen
Philippe Mangeot
Peter Mangesho
Harald Mangge
Alessandra Mangia
Fabio Mangiacapra
Giuseppe Mangialardi
Regina Mangieri
Paulo Mangini
Massimo Mangino
Martin Mangino
Marco Mangone
Annarosa Mangone
Maria Luisa Mangoni
Steve Mangos
Venkata Mangu
Jan Mangual
Sangeeta Mangubhai
Neelam Mangwani
Alex Manhães
Lisa Manhart
Michael Manhart
Eric Manheimer
Hélio Manhica
Shyamala Mani
Sridhar Mani
Ankur Mani
Baskaran Mani
Sendurai Mani
Helen Mani
Chinnadurai Mani
Kruti Maniar
Caroline Manicam
Chaysavanh Manichanh
Cordelia Manickam
Krishnan Manickam
Natesan Manickam
Tobias Manigold
Rishya Manikam
M. Manikandan
Sathya Manimunda
Giulia Manina
Paul Manis
Brian Maniscalco
John Maniscalco
Rajajeyakumar Manivel
Philippe Manivet
Elias Manjarrez
Masoud Manjili
K.R. Manjunath
K. Manjunath
Judith Mank
Paul Manka
Annette Mankertz
Joseph Mankin
Richard Mankin
Nancy Manley
Kezia Manlove
David Mann
John Mann
Jelena Mann
Fanny Mann
Koren Mann
Jaclyn Mann
Martin Mann
Francis Mann
Evelyne Mann
Jake Mann

Jim Mann
Prasenjit Manna
Inge Mannaerts
Upender Manne
Baboucarr Manneh
Daniela Mannel
Claudia Männel
Kaisa Mannerkorpi
Mattias Mannervik
Jean-Baptiste Manneville
Michelle Manni
Raffaele Manni
Sabrina Manni
Mark Mannie
Jaan Männik
Aki Manninen
Adrian Manning
Shannon Manning
Laurens Manning
John Manning
Pete Manning
Katie Manning
Catherine Manning
David Mannino
Gaia Mannino
Marcello Mannino
Philip Mannion
Martina Manns
Pier Mannucci
Edoardo Mannucci
Tadaaki Mano
Catherine Manoha
Aswin Manohar
Muthu Saravanan Manoharan
Muthusamy Manoharan
Palanikumar Manoharan
Lokeshwerand Manoharan
Irina Manokhina
Spililios Manolakopoulos
Morris Manolson
Paul Manos
Santi Mañosa
Weerawat Manosuthi
Ali Manouchehrinia
Nicholas Manoukis
Tereza Manousaki
Francisco Manresa-Manresa
Pablo Manrique Saide
Christoph Mans
Jonathan Mansbach
Tim Manser
Marta Manser
Timothy Manser
Linda Mansfield
Caroline Mansfield
Avril Mansfield
Kate Mansfield
Kim Mansky
Ulrich Mansmann
Michael Manson
Robert Manson
Lucia Manso-Silvan
Wael Mansour
Ahmed Mansouri
Kamran Mansouri
Shiva Mansouri
Alka Mansukhani
Vanya Mantareva
Eman Mantawy
Massimo Mantegazza
Francesco Mantegazza
Rosario Mantegna
Aukje Mantel-Teeuwisse
Daniel Manter
Franco Mantero
Jakob Manthey
Girolama La Mantia
Carlos Mantilla
César Mantilla
Nicholas Mantis
Giovanna Mantovani
Shrikant Mantri
Nate Mantua
Patrick Mantyh
Christopher Mantyh
Michail Mantzios
Peter Manu
Michaël Manuel
Remy Manuel
Suzanne Manugian
Paolo Manunta
Maria Manunta
Rudolf Manz
K. Manz
Miguel Manzanares
María Cristina Manzanares-Céspedes

Antonio Manzaneda
Antoine Manzanera
Rebeca Manzano
Eduardo Manzano Moreno
Alejandro Manzano-Marin
Christian Manzardo
Ann Manzardo
Alessandra Manzin
Ivan Manzini
Emiliano Manzo
Gianluca Manzo
Stephane Manzo
Lamberto Manzoli
Claudia Manzoni
Chuanbin Mao
Kangshan Mao
Hui Mao
Yuxin Mao
Long Mao
Yilei Mao
Chen Mao
Junhao Mao
Weiming Mao
Xiaobo Mao
Donghai Mao
Fenglou Mao
Xinliang Mao
Zhiyong Mao
Gaowei Mao
Limin Mao
Qing Mao
Huina Mao
Hongda Mao
Yaping Mao
Yijin Mao
Peisheng Mao
Liang Mao
Rong Mao
Yousheng Mao
Yinghui Mao
Qi Mao
Elad Maor
Seraya Maouche
Douglas Mapel
Francesca Mapelli
Terry Maple
Hannah Maple
Frederic Maps
Salwan Maqdasy
Raymond Mar
Rubiana Mara Mainardes
Anna Marabotti
Petros Maragkoudakis
Richard Maraia
Leonard Marais
Panagiotis Marakos
Avudaiappan Maran
Christa Marandino
Antonella Marangoni
Raul Maranhão
Emilio Marañón
Katie Maras
Felipe Maraschin
Daniela Marasco
Ramona Marasco
Valeria Marasco
Agnieszka Marasek-Ciolakowska
Barbara Maraux
Antonio Maraver
Pablo Maravilla
Aniko Maraz
Moreno Marazzi
M. Marazzi
Daniel Marbach
Ketan Marballi
Daniel Marc
Robert Marc
Guillaume Marcais
Marija Marcan
Matteo Marcantonio
Paolo Marcatili
Michael Marceau
Machteld Marcelis
La Noce Marcella
Marjory Marcello
Dror Marchaim
Luc Marchand
Stephane Marchand-Maillet
Veronique Marchand-Pauvert
Jonathan Marchant
Jeremy Marchant-Forde
Cinzia Marchese
Giulio Marchesini
Silvia Marchesotti
Bianca Marchetti
Giulia Marchetti

Piero Marchetti
Paolo Marchetti
Carlo Marchetti
Cristina Marchetti
Paolo Marchettini
Artur Marchewka
Nicola Marchi
Saverio Marchi
Damiano Marchi
Fabricio Marchini
Cristina Marchini
Timoteo Marchini
Caterina Marchio
L.L.M. Marchiori
Paola Marchisio
Mario Marchisio
Douglas Marchuk
Francine Marciano-Cabral
Stefan Marciniak
Pawel Marciniak
Alifano Marco
Maria Marco
Eva Marco
Del Lama Marco
Salvatore Marco
Jose Marco-Contelles
Francisco Marco-Jiménez
Alessandro Marcon
Luciano Marcon
Maria Cecilia Marcondes
Fernanda Marcondes
Richard Marconi
Susana Marcos
Ricard Marcos
Marcos Marcos
Bruce Marcot
Walter Marcotti
Olivier Marcou
Tania Marcourakis
Claude Marcus
Ulrich Marcus
Julia Marcus
Steven Marcus
Shoshana Marcus
Simone Marcuzzo
Helmi Mardassi
Christian Mardin
Adil Mardinoglu
Patrick Mardulyn
Edson Mareco
Hans Maree
Paul Marek
Erika Marek
Roger Marek
Manjula Marella
Stefano Marenco
Olga Mareninova
Julie Marentette
Kath Mares
Vittoria Maresca
Marc Maresca
Denis Mareschal
Lucile Marescot
Jennifer Maresh
Andre Marette
Thorsten Maretzky
Raffaele Marfella
Mary Marfori Christie
Jutta Marfurt
Paolo Margaria
Immaculada Margarit
Andriana Margariti
Paris Margaritis
M. Margarson
Peter Margetts
Marcia Margis-Pinheiro
William Margolin
Drew Margolin
Leonid Margolis
David Margolis
Elisa Margolis
Sandra Margriter
Samuel Marguerat
Guiguet Marguerite
Marguerite Marguerite Schneider
David Margulies
B. Margulies
Marko Marhl
Karol Marhold
Neus Mari Mena
Durvanei Maria
Herrera-Campos Maria
Maria Maria Del Pilar Crespo-Ortiz
Fabyana Maria Dos Anjos
Mahendra Mariadassou
Silvya Maria-Engler

Bernard Mariamé
Paolo Mariani
Massimiliano Mariani
Eduardo Mariano
Karina Mariante Monteiro
Thomas Marichal
Brian Maricle
Isabelle Maridonneau-Parini
Benjamin Marie
Dannette Marie
Paul Mariel
Carine Marie-Magdeleine
Mylene Mariette
Romain Marignier
Ionan Marigomez
Nicolas Marilleau
José Maria Marimon
Ben Hur Marimon Junior
Ganapathy Marimuthu
Frederic Marin
Jose Marin
Anelis Marin
Pedro J. Marin
Bóris Marin
Gonzalo Marin
Mauricio Marin
Terri Marin
Ethan Marin
Maria Marin
Clara Marin
Ignacio Marín
Francisco Marín
Daniele Marinazzo
Clara Marín-Briggiler
Michela Marinelli
Jessica Marinello
Jose Maring
Rui Marinho
Francesco Marini
Chiara Marini
Ruben Marin-Juez
Ksenija Marinkovic
Bruno Marino
Maria Marino
Fe Marino
Tiziana Marino
Silvia Marino
Angela Marino
Rodrigo Mariño
Osvaldo Marinotti
Georgi Marinov
Vesna Marinovic
Michael Marinovich
Luis Marins
Alberto Marin-Sanguino
Martin Marinus
Taffurelli Mario
Sabrina Marion
Céline Marionneau
Silvio Mariotti
Mauro Mariotti
Encarnita Mariotti-Ferrandiz
Sofia Mariotto
Sadie Marjani
Henju Marjuki
Dopson Mark
Patrick B Mark
Westneat Mark
Sophia Markantonis
Sheraz Markar
Melanie Marketon
Sebastian Markett
Iwona Markiewicz
Jürgen Markl
Erik Marklund
Karoly Marko
Lajos Marko
Mollie Marko
Athina Markou
Gabriel Markov
Slobodan Markovic
Milica Markovic
Tania Markovic
Omer Markovitch
Therese Markow
Morri Markowitz
Kenneth Markowitz
Steven Markowitz
Michael Marks
Angharad Marks
Klas Markström
Perkmann Markus
Nolf Markus
Thor Nygaard Markussen
Michal Markuszewski
Fritz Markwardt

Soma Sunder Marla
Ferdinand Marlétaz
Kevin Marley
W. Marlicz
Florence Marlow
Heather Marlow
Neil Marlow
Srinivas Marmamula
Meir Marmor
Michael Marmor
Philippe Marmottant
Alice Marmugi
Michael J. Marmura
Harvey Marmurek
Lawrence Marnett
Jennifer Marohasy
Michele Maroli
Nimrod Marom
Tal Marom
Erik Maronde
Tatiana Maron-Gutierrez
Katalin Maros
Luc Maroteaux
Gergely Maroti
Hubert Marotte
Mc Maroun
Nicole Marquardt
Mary Marquart
Antonio Marques
Rafael Elias Marques
Alexandra Marques
Márcia Maria Marques
João Tiago Marques
Miriam Marques
Joana Marques
Amélia Marques
Pedro Marques
Cláudia Marques
A. Marques
Adriana Marques
Adilson Marques
Idoia Marqués-Iturria
Roland Marquet
David Marquez
Eladio Marquez
Jodie Marquez
Rebecca Marquez
Manlio Márquez
Alfonso Marquez-Chamorro
Antonio Marquina
Linsey Marr
Michael Marr
Nico Marr
Giancarlo Marra
Fabio Marra
Maurizio Marra
Vannina Marrachelli
Philippa Marrack
Maurizio Marrale
Jeanne Marrazzo
Olivier Marre
Philip Marren
Stéphane Marret
Siewert Jan Marrink
Belen Marron
Federico Marrone
Laura Marroquí
James Marrow
Robert Marrs
Jet Mars
Sara Marsal
Karel Marsal
Francesco Marsano
Manfred Marschall
Ian Marschner
Islay Marsden
Alison Marsden
O. Marsenic
Terry Marsh
Christopher Marsh
Glenn Marsh
James Marsh
Joseph Marsh
Helene Marsh
Kim Marsh
Jessecae Marsh
Antoinette Marsh
Thomas Marsh
Samantha Marsh
David Marshak
Gad Marshal
Tara Marshall
John Marshall
Justin Marshall
Christian Marshall
Janice M. Marshall

Brandon Marshall
Gailen D. Marshall
Mark Marshall
Paul Marshall
Harry Marshall
William Marshall
Katie Marshall
Ariela Marshall
Jim Marshall
Brittney Marshall
Sonya Marshall-Gradisnik
Sarah Marshall-Pescini
Nicholas Marsh-Armstrong
Alyssa Marshell
Carmen Marsit
Maarten Marsman
David Marsolais
Frederic Marsolais
Laurent Marsollier
Lesley Marson
Lorna Marson
Alexander Marson
Adele Marston
Sean Marston
Marta Marszalek
Monica Marta
Muzio Marta
Marco Martalo
Jan Martel
Guillaume Martel
Paulo Martel
Marco Martel
Vito Martella
Andrea Martella
Valerie Martel-Laferrière
Alberto Martelli
Pier Luigi Martelli
Vyacheslav Martemyanov
Geert Martens
Alexandre Camargo Martensen
Lena Mårtensson
Shiva Marthandan
Hanspeter Marti
Sebastien Marti
Guillaume Marti
Joachim Marti
Hanna Marti
Hans-Peter Marti
Fernando Marti´Nez-Garci´A
Janne Martikainen
David Martill
Paul Martin
David Martin
Francois-Pierre Martin
Javier Martin
Thomas Martin
Frank Martin
James Martin
Juan Martin
Keith Martin
Lee Martin
Samuel Martin
Stefan Martin
Oliver Martin
Roland Martin
Graeme Martin
Alastair Martin
Rocio Martin
Arnaud Martin
Francis Martin
Tammy Martin
Camilia Martin
Thibaud Martin
Denis Martin
Seth Martin
Debra Martin
Charles Martin
Pascal Martin
William Martin
Allan Martin
Olivier Martin
Caroline Martin
Loren Martin
Amanda Martin
Dustin Martin
Mariana Martin
Laura Marti-N
Joanna Martin
Tara Martin
Rosemarie Martin
Christian Martin
Erika Martin
Gilbert Martin
Kirsty Martin
Adam Martin
Brent Martin

William Martin
Meghan Martin
Leonard Martin
Patricia Martin
Melissa Martin
Travis Martin
Julien Martin
Bryn Martin
Jérémy Martin
Kathy Martin
Diana Martin
Mauricio Martín
Carlos Martín Ardila
Joan Martin Montaner
Jose Martina
Luigi Martina
Boris Martinac
Miguel Martin-Acebes
Valeria Martin-Albarracin
Inmaculada Martín-Burriel
Pedro Martin-Cabrera
Sanda Martincic-Ipsic
Ana-Belen Martin-Cuadrado
Russell Martindale
Adrian Martineau
Yvan Martineau
Luiz Martinelli
Paola Martinelli
Pasquale Martinelli
Roberta Martinelli
Filippo Martinelli Boneschi
Laetitia Martinerie
Salvador Martinez
Juan Martinez
Octavio Martinez
Fernando Martinez
Ernest Martinez
Luis Martinez
Antigona Martinez
Alejandro Martinez
Vicente Martinez
Priscilla Martinez
Jose Martinez
Pierre Martinez
Gary Martinez
Ivan Martinez
Felipe Martinez
Eloy Martinez
Katherine Martinez
M. Carmen Martinez
Daniel Martinez
Neus Martinez
Gabriel Martinez
Leonardo Martinez
Jayson Martinez
Aline Martinez
José Martínez
Miguel Angel Martínez
Alexander Martínez
Lara Martínez
Maria L Martinez Chantar
Sara Martinez De Lizarrondo
Guillermo Martinez De Tejada
Miguel Martínez González
José Martínez González
Pilar Martínez Hidalgo
Agustin Martinez Molina
Javier Martinez Reina
Moisés Martínez Velázquez
Maria Del Pilar Martinez Viedma
S.F. Martínez_Díaz
Alejandro Martinez-Abrain
Alejandro Martínez-Abrain
María Del Carmen Martínez-Ballesta
Jesús Martínez-Barnetche
Roberto Martínez-Beamonte
Alberto Martinez-Castelao
Jaime Martínez-Castillo
Jose Martinez-Costas
Begoña Martínez-Crego
Juan Martinez-Cruzado
Victoria Martinez-Diaz
Ana Martinez-Donate
Ana Paola Martínez-Falcón
Monica Martinez-Fernandez
Antonio Martínez-Fuentes
Ma Martinez-Garcia
Pedro Martínez-Gómez
Maite Martinez-Granado
José Armando Martínez-Guarneros
Erik Martinez-Hackert
Antonio Martínez-Laborda
Elena Martinez-Lapiscina
Pablo Martinez-Legazpi
Luis Martinez-Lemus
Miguel Martinez-Lirola

Joaquín Martínez-López
Luis Martinez-Lostao
Pilar Martinez-Martinez
Margarita Martinez-Medina
Alberto Martínez-Ortí
Luisa Martinez-Pomares
Narcisa Martinez-Quiles
Daniel Martinez-Ramirez
David Martinez-Rego
Jose Martínez-Rodríguez
Encarnacion Martinez-Salas
Encarnación Martínez-Salas
Carlos Martinez-Salgado
Aida Martinez-Sanchez
José Martínez-Sanz
Maria Jose Martinez-Sebastian
Manuel Martinez-Selles
Jordi Martinez-Serra
Luis Martínez-Sobrido
Inigo Martinez-Solano
Julio Martinez-Trujillo
Naiara Martinez-Velez
Carmel Martin-Fairey
Enrique Martin-Gayo
Montserrat Martin-Hernandez
Olga Martinho
Claudia Martini
Rudolf Martini
Matteo Martini
Paolo Martini
L. Martini
José Martín-Nieto
Tami Martino
Davide Martino
Juan Martino
Mikaël Martino
Ashley Martino
Enrico Antonio Martino
David Martino
Alain Martinot
Jean-Claude Martinou
Enca Martin-Rendon
Yuri Martins
Allan Cezar Martins
Antonio Martins
Madalena Martins
Marco Martins
Ademir Martins
Eduardo Martins
Cesar Martins
Reinaldo Martins
Bruna Martins
Samuel Martins
Daniele Martins
Nelson Martins
Carolina Martins
Viviana Martins
Mariana Martins
Bianca Martins
Gilberto Martins
Leonardo Martins
Gustavo Martins
Ana Martins
David Martins De Matos
José Luiz Martins Do Nascimento
Ana Martins Sequeira
Sheryl Martin-Schild
Manuela Martins-Green
Elizabeth Martin-Silverstone
Vince Martinson
Jan Martinussen
Denis Martinvalet
Jose-Luis Martin-Ventura
Isabelle Martin-Verstraete
Adam Martiny
Jennifer Martiny
Christopher Martius
Patrick Martone
Francesca Martora
Alessandro Martorana
Juan Antonio Martos-Sitcha
Robert Marty
Antonino Marullo
James Maruniak
Hiroyuki Marusawa
Ana Marušic
Eizo Marutani
Shoichi Maruyama
Tadashi Maruyama
Jun-Ichi Maruyama
Yukio Maruyama
Kazuichi Maruyama
Reo Maruyama
Tetsuo Maruyama
Toru Maruyama
Antonino Marvuglia

Steven Marx
Nikolaus Marx
Michael Marx
Felix Marx
Andrey Maryandishev
Krzysztof Marycz
Tristan Mary-Huard
Lucy Marzban
Miriam Marzen
Emanuele Marzetti
Andrea Marzi
Tessa Marzi
Mario Marzilli
Mark Marzinke
Marco Marzioni
Francesca Marzo
Cm Marzocchi-Machado
Stefania Marzocco
Roberto Marzotto
Vincenzo Manuel Marzullo
Antonio Mas
Tali Mas
Sergi Mas
Shiyomi Masae
Katsuhiro Masago
Yoshifumi Masago
Naohiko Masaki
Haruhiko Masaki
Atsushi Masamune
Kei Masani
Tetsuji Masaoka
Michal Masarik
Mario Mascalchi
Jennifer Mascaro
Françoise Mascart
Martin Mascher
Tiziana Mascia
Luciana Mascia
Helene Masclaux
Damian Maseda
Vinod Maseedupally
Refiloe Masekela
Rosalinde Masereeuw
Be Maserti
Matteo Masetti
Deborah Mash
Vladimir Mashanov
Saidur Mashreky
Muriel Masi
Stefano Masi
Marco Masi
Moses Masika
Madhan Masilamani
Laura Masino
David Masip
Marisse Masis Solano
Daniel Masison
Virginia Masiulionis
Cheryl Maslen
Edward Masler
Sharmila Masli
Dana Maslovat
John Masly
Esther Mas-Martí
William Mason
Georgia Mason
Joel Mason
Robert Mason
Kevin Mason
Philip Mason
Christopher Mason
Olivia Mason
Stephen Mason
Jeremy Mason
Deborah Mason
Charlie Mason
Dan Mason
Peri Mason
Craig Mason
Mojgan Masoodi
Andrea Masotti
Luca Masotti
Mohamed Masoud
Ali Masoudi-Nejad
Pere Masque
Bruno Masquelier
Timothée Masquelier
Caroline Masquillier
Roberto Massa
Luisa Massaccesi
Angelo Massacci
Rodrigo Massara
Paola Massari
Serafina Massari
Emanuele Massaro
Julie Massart

Nicolas Masse
Christophe Masselon
Jorg Massen
Myosotis Massidda
Orietta Massidda
Allan Massie
Lourdes Massieu
Chandirasegaran Massilamany
Silvia Massironi
Alessandro Massolo
Patrick Masson
David Masson
Serge Masson
Florent Masson
Ewan Masson
Lindi Masson
Ahmed Massoud
Nicola Massy-Westropp
Fred Mast
Susan Masta
S.S. Mastana
Emma Master
Michal Masternak
William Masters
Jolene Masters Pedersen
Douglas Masterson
Francesca Mastorci
Rossana Mastrandrea
Anna Maria Mastrangelo
Valentina Mastrantonio
Alicia Mastretta-Yanes
Vic Mastro
Maddalena Mastrogiacomo
Claudio Mastroianni
D Mastropietro
Satohiro Masuda
Takahiko Masuda
Shinji Masuda
Muneyuki Masuda
Takashi Masuko
Howard Masuoka
James Masuoka
Hiroshi Masutani
Kenkichi Masutomi
Masahiro Masuya
Paola Masuzzo
Juan Mata
Ignacio Mata
Ale Mata Cabana
P Matafome
Joan Matamalas
Manuel Matamoros
Tetsuro Matano
G. M. Matar
Maria Matarazzo
Giuseppe Matarese
Daniela Matarrese
Carmen Matás
Sebastian Mate
Bogdan Mateescu
Raluca Mateescu
Daniela Matei
Jason Mateika
Jason Matejkowski
J. Matena
Jill Mateo
Rafael Mateo
Juan Mateo
Luciano Mateos
Ivana Matera
Stefan Materna
Lisa Matero
Silvia Matesanz
Mauricio Mateu
Guillermo Mateu
Michael Mathai
Ewy Mathe
Aleksander Mathe
Csaba Máthé
Ashley Matheny
Karen Mather
Amanda Mather
Kieren Mather
Alison Mather
Colin Mathers
Birgit Mathes
Melanie Matheson
Heath Matheson
Anuja Mathew
Joseph Mathew
Ashish Mathew
Bobby Mathew
T. Chacko Mathew
Sijo Mathew
David Mathews
Sarah Mathews

Clayton Mathews
Vikram Mathews
Andrew Mathews
Emily K. Mathey
Klaus Mathiak
Paulo Mathias
Richard Mathias
Clinton Mathias
Jean-Denis Mathias
Candace Mathiason
Joachim Mathiesen
Ian Mathieson
Chantal Mathieu
Patrick Mathieu
Jerome Mathieu
Veronique Mathieu
Kostas Mathiopoulos
Saroj Mathupala
Jaideep Mathur
Vani A. Mathur
Nb Mathur
Manu Mathur
Ramkumar Mathur
Miguel Matias
Pedro Matias
Ivan Matic
Bojana Bogovic Matijas
Babak Matinfar
Elizabeth Matisoo-Smith
Scot Matkovich
Nobuyuki Matoba
Satoaki Matoba
Antonio Teixeira Matos
Petr Matous
Tomas Matousek
Valeria Matranga
Antonios Matsakas
Rebecca Matsas
Michael Matschiner
Joerg Matschullat
Paul Matson
Amanda Matson
Terhuhiko Matsubara
Chieko Matsubara
H. Matsubara
Koichi Matsuda
Tetsuya Matsuda
Masayuki Matsuda
Morihiro Matsuda
Hiroshi Matsuda
Shinichi Matsuda
Michiyuki Matsuda
Osamu Matsuda
Hiroshi Matsui
Minami Matsui
Reiko Matsui
Toshihiro Matsui
Yoshihiko Matsui
Doreen Matsui
Toru Matsukawa
Chiaki Matsukura
Kunihiro Matsumoto
Takashi Matsumoto
Isao Matsumoto
Kunio Matsumoto
Yasuharu Matsumoto
Satoru Matsumoto
Takuya Matsumoto
Kazumasa Matsumoto
Daiki Matsumoto
Riki Matsumoto
Hiroyuki Matsumoto
Asako Matsumoto
Chota Matsumoto
Midori Matsumoto
Shuichi Matsumura
Noriomi Matsumura
Takeshi Matsumura
Sachihiro Matsunaga
Masahiro Matsunaga
Kenjiro Matsuno
Kenji Matsuno
Koichi Matsuo
Michinori Matsuo
Koji Matsuo
Ryu Matsuo
Masao Matsuoka
Atsushi Matsuoka
Michiya Matsusaki
Kazuyuki Matsushita
Hiroaki Matsushita
Hirokazu Matsushita
Eiji Matsuura
Hiroshi Matsuura
Katsuhisa Matsuura
Hideo Matsuura

Keiko Matsuura
Shigemi Matsuyama
Yusuke Matsuyama
Shigeru Matsuyama
Daisuke Matsuyoshi
Hideo Matsuzaki
Shigenobu Matsuzaki
Kentaro Matsuzaki
Tomohiko Matsuzawa
Kosuke Matsuzono
Ajay Matta
Benjamin Matta
Mario Matta
Francesco Mattace-Raso
Joseph Mattapallil
Rejane Mattar
Alberto Matteelli
Eugenio Mattei
Alessio Mattesini
Andrea Mattevi
Michael Matthay
Asha Matthew
Howard Matthew
Verghese Matthew
Mervin Matthew
Russell Matthews
Jonathan Matthews
Hugh Matthews
Slade Matthews
Daniel Matthews
Thomas Matthews
Joy Matthews
Julian Matthews
Vance Matthews
Luke Matthews
Allison Matthews
Stephen Matthey
Alexa Mattheyses
Diethart Matthies
Hans Matthijs
Suzette Matthijsse
Patrick Matthys
Donatella Mattia
Davide Mattia
Gianfranco Mattia
Heather Mattie
Silvana Mattiello
Lucia Mattiello
Heather Mattila
Raymond Mattingly
Chris Mattison
Frauke Mattner
Garrett Mattos
Rodrigo Mattos
Mark Mattson
Mats-Olof Mattsson
Pauline Mattsson
Jonas Mattsson
Radim Matula
Giuseppe Matullo
Anke Matura
H. Matusaki
Kai Matuschewski
Dusan Matusica
Robert Matusik
Ewa Matuszczak
Eugenia Matveeva
Tatiana Matveeva
Beata Matysioková
Karl Matz
Sandra Matz
Anastasios Matzavinos
Shannon Matzinger
Martin Matzuk
Marlene Mauch
Christine Mauck
Thais Maud
Rapeephan Maude
Audrey Maudoux
Michael Mauer
L. Maugendre
Teresa Luciana Maugeri
Marcello Maugeri-Saccà
Brendan Maughan-Brown
Jochen Maul
Maximilian Mauler
Jonathan Maupin
Joan Maurel
John Maurer
Matthew Maurer
Urs Maurer
Jochen Maurer
Carine Maurer
Uli Maurer
Peter Maurer
Joachim Maurer

Mathias Mäurer
Sebastian Maurer-Stroh
Pierluigi Mauri
Tangui Maurice
Donald Maurice
Michèle Maurice
Sean Maurice
Corinne Maurice
Dídac Mauricio
Thomas Maurin
Marguerite Mauritz
Michael Maurizi
Claudio Mauro
Pál Maurovich-Horvat
Wendy Maury
Julien Maury
Santosh Maurya
Kimberly Maute
Eva Mautner
Brian Mautz
Franck Mauvais-Jarvis
Nirmala Mavila
Patrick Mavingui
Brian Mavis
Irene Mavridis
Dmitri Mavrodi
Evgeny Mavrodiev
Eleni Mavrogonatou
Manolis Mavroidis
Zira Mavunganidze
Peter Maxfield
Molly Maxfield
Caio Maximino
Valdemar Maximo
Stephen Maxson
Taylor Maxwell
Sara Maxwell
Lauren Maxwell
Toby Maxwell
Michael May
Victor May
Catherine May
Celia May
Felicity May
Meghan May
Paul May
Peter May
Ross May
Stephen May
Michael L. May
May Lei May
Juan Maya
Christopher Mayack
Tanya Mayadas
Clarissa Maya-Monteiro
Anoop Mayampurath
Toufic Mayassi
Marc Mayberg
Peter Maye
Akila Mayeda
Bruce Mayer
Gert Mayer
Andrew Mayer
Emeran Mayer
Günter Mayer
Gaétan Mayer
Greg Mayer
Balazs Mayer
Veronika Mayer
Stephan Mayer
Susanne Mayer
Katrin Mayer-Barber
Peter Mayerhofer
Mariana Mayer-Pinto
William Mayes
Patrick Mayeux
R. Mayfield
Stephen Mayhew
Jerry Mayhew
Maskit Maymon
Jennifer Maynard
C. Maynard
John Maynard
Kevin H Mayo
Christie Mayo
Nancy Mayo
Karla Mayolo-Deloisa
Alfredo Mayor
Stephen Mayor
Thibault Mayor
Evan Mayo-Wilson
Aileen Maypa
Gerald Mayr
Thomas Mayr
Marie-Hélène Mayrand
Ralf Mayrhofer

Helen May-Simera
Rosie Mayston
Nicolas Maystre
Percy Mayta-Tristán
Nishi Mayumi
Fadia Mayyas
Oktawia Mazanowska
Mervyn Maze
Bruce Mazer
Fabienne Mazerolles
T.D. Mazgajski
Mikael Mazighi
Jean-Xavier Mazoit
Rachid Mazroui
Reiko Mazuka
Abhijit Mazumdar
Barsanjit Mazumder
Wojciech Mazur
David Mazurais
Vera Mazurak
Gerald Mazurek
Veronica Mazza
Tommaso Mazza
Davide Mazza
Edoardo Mazza
Giuseppe Mazza
Sante Mazzacane
Paulo Mazzafera
Luca Mazzarella
Claudia Mazzeschi
Dominique Mazzi
Stefania Mazzini
Adriano Mazziotta
Gianluigi Mazzoccoli
Emanuele Mazzola
Rosario Mazzola
Barbara Mazzolai
Marcelo Mazzolli
Patrizio Mazzone
Annamaria Mazzone
Pietro Mazzoni
Cristina Mazzoni
Valerio Mazzoni
Marco Mazzorana
Gabriella Mazzotta
Marc Mazzuca
Walter Mazzucco
Gabriel Mbalaviele
Leonard Mboera
Ralph Mbouna
Lawrence Mbuagbaw
John Mc Evoy
Jennifer Mc Sharry
Paul McAdam
Carrie McAdams
Ryan McAdams
Andrew McAinsh
John McAlaney
Colm McAlinden
Tim McAllister
James P mcallister
Sandra McAllister
Sean McAllister
Shane McAllister
Susan McAllister
Ken McAnally
Lisa McAndrew
Mary McAndrews
Joseph McArdle
Craig McArdle
Andrew McArthur
Justin Mcarthur
Kate McArthur
Simon McArthur
Jb McArthur
John McAteer
Julie McAuley
Annie McAuley
Paul McAuley
Julian McAuley
Fionnuala McAuliffe
Olivia McAuliffe
Gail McAvay
Andrew McBain
Hayley McBain
Heidi McBride
Marissa McBride
Alison McBride
Kim McBride
Kevin McBride
Shonna McBride
Jeffrey McBride
Alan McBride
Michael McBurney
Laura R McCabe
Paul McCabe

Ryan McCabe
Candy McCabe
James McCabe
Kira McCabe
Peter McCaffery
Rebecca McCaffery
Charles McCall
Kimberly McCall
Matthew McCall
Cade McCall
Grant McCall
Scott McCallum
Ian McCallum
Timothy McCalmont
Michael McCamy
Dennis McCance
Matthew McCann
Robert McCann
Honour McCann
Tyler McCann
Margaret McCarron
Michael McCarthy
David mccarthy
John McCarthy
William McCarthy
Kevin McCarthy
Heather McCarthy
Neil McCarthy
Fiona McCarthy
Elizabeth McCarthy
Andy McCarthy
Matthew J. McCarthy
Robert McCarthy
Nael McCarty
John McCarty
Douglas McCarty
Owen McCarty
Christopher McCarty
Jessica Mccarty
Michael McCaskill
John McCauley
Mark McClain
Tim McClanahan
Bruce McClane
Siobhan McClean
Paula McClean
Sally McClean
Amanda McCleery
Amie McClellan
Graham McClelland
Susan McClement
Tim McClintock
James McClintock
Eugene McCloskey
Karen McCloskey
Kevin McCluney
Michael McClung
Colleen McClung
Elizabeth McClure
Christopher McClure
Roderick Mcclure
Scott McClure
Urszula McClurg
James McCluskey
Declan McCole
Gawain McColl
Susanna McColley
Eric McCollum
Kim McConkey
Brendan McConkey
David McConkey
Roy McConkey
Michael McConnell
Kevin McConnell
Malcolm McConville
Matthew McCormack
Shana McCormack
John McCormick
Craig McCormick
Sheree McCormick
David Mccormick
Michael McCormick
Aleesha McCormick
Sean McCormick
Joy McCorriston
Mark McCourt
J. Philip McCoy
Karen McCoy
Rajiv McCoy
Annette M McCoy
Tony McCoy
Lance McCracken
Malcolm McCrae
Pierre McCrea
D. McCree
Brent McCright

Rory McCrimmon
Luke McCrohon
Paul McCrone
Jean McCrory
Megan Ann McCrory
Molly McCue
Marshall McCue
Rebecca McCulley
Christopher Ag McCulloch
Michael McCulloch
Richard McCulloch
Karen McCulloch
Michael McCulloch
Mac McCullough
Robert McCullumsmith
K McCully
Jenny McCune
Carrie McCurdy
Robert McCutcheon
John McCutcheon
Simon McDade
Larry McDaniel
Michael McDaniel
Marshall McDaniel
Lee McDaniel
Jonathan McDearmid
Joann McDermid
John C. McDermott
David McDermott
Allison McDermott
Sean McDermott
Suzanne McDermott
Kev McDermott
Lee McDermott
Paul McDermott
Shana McDermott
Ashley McDermott
James McDevitt
Elizabeth McDevitt
Adam McDiarmid
Marian McDonagh
Bruce McDonald
Gregory McDonald
Thomas McDonald
Fiona McDonald
John McDonald
Robbie McDonald
Kerrie McDonald
Andrew McDonald
Emily McDonald
Tami McDonald
Patrick McDonald
Birgitte McDonald
Stuart McDonald
Brenna McDonald
James McDonald
Chloe McDonald
Jay McDonald
Samantha McDonald
Neville McDonald
Suzanne McDonald
Peter McDonald
Alyson McDonald
Sheila McDonald
Karen A. McDonalds
Sue McDonnell
Timothy McDonnell
Alicia McDonough
Kathleen McDonough
Stefan McDonough
Molly McDonough
Ian McDonough
Alex McDougall
Carmel McDougall
Kenneth McDowall
Mary Ann McDowell
Andy McDowell
Rosie McEachan
Richard McEachin
Robert McElderry
Janet McElhaney
Lorraine McElhinney
Anthony M McElligott
James McElnay
Steven McElroy
Arthur McElroy
Donald McElwain
Jo McEvoy
Iain McEwan
Ryan McEwan
Alistair McEwan
Scott McEwen
Bradley J. McEwen
Catherine McFadden
Kristina McFadden
Brandon McFadden

Willi McFarland
Nikolaus McFarland
Craig McFarlane
Brian McFarlin
Melanie McField
Patrick McGah
Anita McGahan
Chris McGahan
John McGann
Patrick McGann
Donald McGarey
Matthew McGarry
Jeffery McGarvey
Suzanne McGaugh
Jill McGaughy
Kerry McGawley
Michael McGeachie
Lesley McGee
Meghan McGee Lawrence
William Mcgeown
James McGettigan
Carolyn McGettigan
David McGhie
Anne-Thea McGill
Janet McGill
Mitchell McGill
Amanda McGillivray
James McGinnis
Ann McGinty
Alexander McGirr
David McGivern
C. Jane McGlade
Edwina McGlinn
Chris McGlory
Joel McGlothlin
Dennis McGonagle
Imelda Mcgonnell
Ryan McGorty
James McGough
Kathryn McGovern
Patrick McGowan
Catherine McGowan
Victoria McGowan
Michael R. McGowen
Chris McGowin
Matthew McGrail
Devan McGranahan
Barbara McGrath
Christine McGrath
Margaret McGrath
Callie McGrath
Colman McGrath
Marie McGrath
Catherine McGrath
Tim McGraw
Rose McGready
Skye McGregor
Alison McGregor
Julie Anne G. McGregor
Ailsa McGregor
Glenn McGregor
Alison McGuigan
Dagmara McGuinness
Jimmy McGuire
John McGuire
Liam McGuire
Kathleen McGuire
Sarah McGuire
Jenny McGuire
Stephen McGuire
Anthony McHale
Andrew McHill
Timothy McHugh
Peter McHugh
Alan McHughen
Maureen McHugo
William McIlhagga
Marcia McInerney
Christopher McInerny
Kathleen McInnes
Alistair McInnes
Campbell McInnes
Thomas McIntosh
Marla McIntosh
Robert McIntosh
Shane McIntosh
Andrew McIntosh
Cathrine McIntyre
Neil McIntyre
Jeremy McIntyre
Kevin McIver
David McIver
Michael McKain
Gareth McKay
Derek McKay
Stephanie McKay

Colette McKay
Alex McKay
Jack McKay Fletcher
Vanessa Mckean
Roberta Mckean-Cowdin
Andrew McKechnie
Dorothy McKeegan
Ryan McKellar
Peter McKenna
Benjamin S. McKenna
Debbie McKenzie
David McKenzie
Sean McKenzie
Joanna McKenzie
Matthew McKenzie
Lisa McKenzie
Lara McKenzie
Andrew McKeon
Niall McKeown
Iain McKillop
Laura McKillop
Kim McKim
Eliot McKinley
Danette McKinley
Christopher McKinley
Michelle C McKinley
James McKinnell
Michael McKinney
Matthew McKinney
Shannon McKinney-Freeman
John McKinnon
Emily McKinnon
Lyle McKinnon
Timothy McKinsey
Kai McKinstry
Aine McKnight
Ursula McKnight
Alexander McLain
Donald McLaren
Zoe McLaren
Patricia McLaughlin
Richard McLaughlin
John McLaughlin
Marie McLaughlin
Daniel McLaughlin
Michael McLaughlin
Emily McLaurin
Mary-Louise McLaws
Susannah Mclean
Pamela McLean
Dianne McLean
Ailsa McLean
Carmen McLean
Robert McLean
Claire Mclean
William McLean
Michael McLeish
Alexander McLellan
Jason McLellan
Sandra McLellan
Gill McLellan
Roger McLendon
David S. McLeod
Donald McLeod
Adele McLeod
Linda McLoon
Philip McLoone
Philip McLoughlin
Grainne McLoughlin
Chris McMahan
Trina McMahon
James McMahon
Francis J McMahon
Gearoid McMahon
Kelton McMahon
Catherine McMahon
Taegan McMahon
Clive McMahon
James McManaman
Chris McManus
Georges McManus
Hamish McManus
David McManus
Ali McManus
Concepta McManus
Alexandra McManus
W. Robert McMaster
Tom McMeekin
Sarah McMenamin
Paul McMenamin
Geoff McMichael
Lindsay McMillan
Diana McMillan
Sally McMillin
Kenneth McMillin
Matthew McMIllin

P. McMinn
Terry McMorris
Tara McMorrow
Meredith McMorrow
Julie McMullen
Matthew McMurray
Aaron McMurtray
Adrian McNairn
Margaret McNairy
Elizabeth McNally
Gavan McNally
Kenneth McNally
Alan McNally
Richard McNally
James Dayre McNally
Paul McNamara
James McNamara
Shamus McNamara
Eoin McNamee
Phillip McNamee
Monica McNeal
Karen McNeal
Jeremy McNeil
Chris McNeil
Matthew McNeil
Tom McNeilly
Alister McNeish
Erin McNerny
Lance McNew
Archibald McNicol
Penelope McNulty
Chris McOwen
John McPeak
Michael McPhaden
Jamie S. McPhee
Kevin McPhee
Shannon McPherron
Mike McPherson
Stephen McPherson
Hannah McPherson
Kyle McQuade
Robert McQueen
Zoe McQuilten
Ryan Patrick Mcquinn
Rory McQuiston
Allan McRae
Annie-Laurie McRee
W. McShan
Helen McShane
Brian McSharry
William McShea
Daniel McSkimming
Stephen McSorley
Henry McSorley
Gavin McStay
Alistair McTaggart
John McTague
Paul McVeigh
F. McVerry
David McVey
Tim McVicar
Michael McVoy
Todd McWhorter
Sean McWilliam
Justin P McWilliams
Rennatus Mdodo
Elisabetta Meacci
Sarah Meachem
Julie Meachen
Simon Mead
Richard Mead
Ben Mead
Ross Mead
Kieran Meade
Mark Meadowcroft
Jennifer Meadows
Richard Meadows
Stryder Meadows
Justin Meager
Rebecca Meagher
Robert Meagher
Shawn Meagher
Matthew Meagher
Brian Mealor
Anna Means
Daniel Meara
Luisa Mearin
Vivien Measday
Thomas Measham
Alan Meca
Rosaria Meccariello
L. David Mech
Naguib Mechawar
Salaheddine Mécheri
Yehia Mechref
Sylvia Mechsner

Sergei Mechtcheriakov
Diana Mechtcheriakova
Tobias Meckel
Patrizia Mecocci
Donata Medaglini
Hugh Medal
Ohad Medalia
Zdravka Medarova
Mara Medeiros
Bruno C. Medeiros
Danielle Medek
Gertjan Medema
Benjamin Meder
Richard Medford
Katarina Medger
Araya Medhanyie
Enzo Medico
Guruprasad Medigeshi
Kay Medina
Carlos Medina
Loreta Medina
Daniel Medina
Antonio Medina
Rafael Medina
Raul Medina
Diego Medina
Nagore Medina
Freddy Medina
Margarita Medina
Jared Medina
Rafael Medina Silva
Maria Elena Medina-Mora
Rafael Medina-Navarro
Carlo Eduardo Medina-Solís
Sarah Medland
Kathryn Medler
Sara Mednick
Paula Medone
Michaela Medova
Francisco Javier Medrano
Mónica Medrano
Luis Medrano-González
Nora Medrano-Mercado
Patrik Medstrand
Milica Medved
Alexei Medvedev
David Medvigy
Hind Medyouf
Robyn Meech
Mary Meeham
David Meek
Mariah Meek
Shannon Meeks
Ramovatar Meena
Ram Swaroop Meena
Sreepriya Meenakshisundaram
Jochen Meens
Mohammed Meer
Mariana Meerhoff
Geert Meermans
Joanne Meers
Paul Meers
Melanie Meersch
Martijn Meeter
Ivan Meeus
Mira Meeus
Steven Meex
A. M. Megahed
Wout Megchelenbrink
Jean-Louis Mege
R.M. Mege
Hendrick-Jan Megens
Alberto Megías
Emese Meglécz
Igor Meglinski
Matteo Megna
Rosette Megnekou
Francis Mégraud
Timothy Megraw
Muthamilarasan Mehanathan
Chadi Mehanna
Caroline Meharg
Alem Mehari
Hamed Mehdipoor
Shawn Mehlenbacher
Rashid Mehmood
Obaid Mehmood
Thomas Mehner
Ramit Mehr
Smriti Mehra
Rohit Mehra
Simin Mehrabani Zeinabad
Arianeb Mehrabi
Reza Mehrazin
Neda Mehrdad
Saeid Mehrkanoon

Arne Mehrkens
Mohammad Mehrmohammadi
R. Mehrotra
Swarna Mehrotra
Atul Mehta
Payal Mehta
Mitul Mehta
Divya Mehta
Dolly Mehta
Anita Mehta
Raaj Mehta
Jodhbir Mehta
Pranjal Mehta
Tapan Mehta
Aditi Mehta
Suchita Mehta
Neil Mehta
Shwetal Mehta
Daryush Mehta
Bella Mehta
Ranjana Mehta
Rupal Mehta
S. Mehtar
Subhash Mehto
Jie Mei
Zubing Mei
Xiaohu Mei
Xiaodong Mei
Chuansheng Mei
Tieniu Mei
Yi Mei
Han-Wei Mei
Shu Mei Teo
Pascal Meier
Raphael Meier
Matthias Meier
Christoph Meier
Emily Meier
Uwe J. Meierhenrich
David Meierhofer
Elizabeth Meiering
Garrett Meigs
Lisa Meihls
Gerrit Meijer
Johanna Meijer
Annemarie Meijer
Wim Meijer
Onno Meijer
Joost Meijers
Björn Meijers
J. Meijs
Peter Meikle
William Meikle
Richard Meilan
Leo Meile
Jens Meiler
Svenja Meiler
Tobias Meilinger
Martina Meincken
Christoph Meinel
David Meinke
Holger Meinke
Elizabeth Meins
Pedro Meirelles
Lindolfo Meirelles
Hamutal Meiri
Dror Meirow
Jacques Meis
Kristine Meise
Peter Meisel
Jonathan Meisel
Richard Meisel
Markus Meissner
Christian A. Meissner
Franziska Meissner
Dominik Meissner
Melanie Meister
K. Meister
Brandt Meixell
Marina Meixner
Vlatka Mejaski Bosnjak
Rojelio Mejia
Juan Manuel Mejia
Anilena Mejia
Jorge Mejias
Asuncion Mejias
Sumiko Mekaru
K.H. Mekheimer
Igor Mekjavic
Armand Mekontso Dessap
Rachel Melamed
Michal Melamed
Eivind Meland
Christian Melander
Roberto Melano
Edward Melanson

Christian Melaun
Roberto Melcangi
Ulrich Melcher
Karsten Melcher
Karen Melcher
Peter Melcher
Marc Melcher
Martin Melchers
Maria Gabriella Melchiorre
Stefan Meldau
Maria Laura Mele
Giovanni Mele
Paula Meleady
Michael Meledeo
Juan Melendez
Barbara Melendez
Loyda Melendez
Miguel Angel Meléndez
Carmen Melendez-Vasquez
Jorge Melendez-Zajgla
Jose Melero
Remedios Melero
Stefano Meletti
César Melgar
Marcia Melhem
Rosaria Meli
Heather Melichar
Bohuslav Melichar
Lester Melie-Garcia
Rosa Marina Melillo
Paolo Melillo
Merit Melin
Patricia Melin
Amanda Melin
Giovanni Melina
Giovanni Melioli
Victoria Meliopoulos
René Melis
Claudia Melis
Roberta Melis
Marta Melis
Marcovalerio Melis
Davide Melisi
Girish Melkani
Mireille Melko
Stephanie Melkonian
Michael Melkus
Loren Mell
Joshua Mell
Valentina Mella
Christian Melle
Rob Mellecker
Matthew Mellema
Victoria Meller
Jarek Meller
Menachem Meller
Mario Melletti
Ingo Mellinghoff
Alexander Mellmann
Antonietta Mello
Maria Luiza Mello
Pamela Mello-Carpes
Pamela Mellon
Lisa Mellon
Ugo Mellone
Sabato Mellone
Harry Mellor
Duane Mellor
Amine Mellouk
Håkan Mellstedt
Shlomo Melmed
Michael Melner
Michael Melnychuk
Paulo Melo
Eduardo Melo
Adriano Sanches Melo
Ari Melo Mariano
Sandro Meloni
Antonella Meloni
Carlo Meloro
Phillip Melton
Jed Meltzer
Andrea Meltzer
Stephen Melville
Ann Melvin
Ryan Melvin
Itshak Melzer
Sandra Meme
Erdogan Memili
Matthew Memoli
Hongsheng Men
Nacho Mena
Alvaro Mena
Christine Ménager
Mehdi Menai
Benoit Menand

Jasmine Menant
Sandrine Ménard
Rima Menassa
Antonella Mencacci
Joy Mench
Jörg Menche
Chang Men-Chi
Silvia Menchon
Arianna Menciassi
Stine Mencl
John Mendelson
Alexander Mendelson
Alexander Mendenhall
Pedro Mendes
Lucas William Mendes
Renato Mendes
Cesar Mendes
Adelio Mendes
Poliana Mendes
Celso Mendes-Junior
Susana Mendez
Juan Mendez
Armando Mendez
Martin Mendez
Ian Mendez
Antonio Mendez
Constantino Méndez-Bértolo
Rosália Mendez-Otero
Christopher Mendias
Vincenzo Menditto
Enrica Menditto
Olivia Mendivil Ramos
Luca Mendler
Fernando Mendonça
Sergio Mendonça
Marcelo Mendonça
Jorge Mendoza
Laura Mendoza
Julia Mendoza-Perez
Vincent Mendy
Matthew Menear
Rachel Menegaz
Rogerio Meneghini
Guerrino Meneguzzi
Daniel Menendez
Margarita Menéndez
María Menéndez-Miguélez
Alfredo Meneses
Jerome Menet
Paulo Menezes
Renato Menezes
Gustavo Menezes
Charlene Menezes
Regina Menezes
Paulo Menezes Silva
Jin Meng
Songdong Meng
Xiangbing Meng
Zhipeng Meng
Xiangfei Meng
Xianxin Meng
Qi Meng
Xianmei Meng
Hailin Meng
Jianjun Meng
Yajing Meng
Fanyin Meng
Kyle Meng
Yan Meng
Pei-Jie Meng
Jing-Hui Meng
Zhang Meng
Le Meng
Zijun Meng
Zhuoxian Meng
Dechuan Meng
Hongdao Meng
Shan Meng
Fantao Meng
Stefan Meng
Christian Menge
Bruce Menge
Til Menge
Friederike Mengel
Eugen Mengel
Jonas Mengel-From
Catherine Mengelle
Elena Mengheri
Rossella Menghini
Meron Mengistu
Alessio Mengoni
Chiara Mengoni
John Mengshol
Lourdes Mengual
Ximo Mengual
Daniela Menichella

Francesco Menichetti
Nicolas Menjot De Champfleur
Douglas Menke
Ellen Menkhorst
Sue Menko
Rubem Menna-Barreto
Adèle Mennerat
Maarten Mennes
Doug Mennin
Samir Menon
Purnima Menon
Ramkumar Menon
Pradeep Menon
Vijay Menon
Smita Menon
Santosh Menon
Raj Menon
Marcelo Menossi
Fiona Mensah
Ayikoe Guy Mensah-Nyagan
Elena Menshchikova
Allen Mensinger
Romuald Mentaverri
Marielle Mentek
Joris Menten
Björn Menten
Andreas F. Mentis
Giovanni Mento
Alex Mentzer
Eline Menu
Arnaud Menuet
Karen Menuz
Myles Menz
Matthew Menza
Claudia Menzaghi
Michael Menze
Bjoern H Menze
Stephan Menzel
Dick Menzies
Robert Menzies
Allyson Menzies
Stefanie Menzies
Nick Menzies
A. Menzies-Gow
Giovanni Meola
Michele Meoli
Flavia Meotti
Maia Merabishvili
Nofel Merbahi
Sebastiano Mercadante
Davide Mercadante
Elena Mercadé
Nadia Mercader
Jesus Mercado
Frances Mercer
Kristina Mercer
John Mercer
Kristin Mercer
Aaron Mercer
Jason Mercer
Ryan Mercer
Andrew Mercer
Gildas Merceron
Fatima Merchant
Hugo Merchant
Aziz Merchant
Joffre Mercier
Olaf Mercier
Luc Mercken
Joanna Merckx
Vincent Merckx
Dan Mercola
Nicola Mercuri
Arthur Mercurio
Andreas Merdes
Timothy Meredith
Bertrand Meresse
Irmgard Merfort
Ján Merganic
Max Mergeay
Mario Mergelsberg
Mario Merialdi
Guillaume Meric
Mathias Mericksay
Cetin Mericli
Annabelle Merieau
Karine Merienne
Alberto Meriggi
Susan Mérillat
Aimee Merino
Agustín Merino
Andres Merits
Christian Merkel
Béla Merkely
Mathias Merker
Jerod Merkle

Alexander Merkler
Daphne Merkus
Maarten Merkx
Manuela Merli
Didier Merlin
Christophe Merlin
Christine Merlin
Giampaolo Merlini
Giorgio Merlo
Emiliano Merlo
Cláudia Mermelstein
Erszebet Mernyak
Antti Mero
Fabienne Merola
Dafna Merom
Aldo Merotto
D. Merrell
Remy Merret
William Merrick
Mike Merrick
Christien Merrifield
Matthew Merrifield
Peter Merrifield
Alfred Merrill
Scott Merrill
Ray Merrill
Amy Merrill-Brugger
Tony Merriman
Joseph Merriman
Allen Merritt
Humberto Merritt
Edward Merritt
Diane Merry
David Merryman
Samuel Merson
Bart Mertens
Haydyn Mertens
Jerome Mertz
Blake Mertz
Dominik Mertz
Elizabeth Merwin
Frederic Mery
Hans Merzendorfer
Hiltrud Merzenich
Joel Mesa Hormaza
Charis Mesaritakis
Annamaria Mesaros
Omar Mesarwi
Noah Mesbah
Gustavo Mesch
John Scott Meschke
Federica Mescia
Andrew Mesecar
Pablo Mesejo
Addisu Mesfin
Mohsen Mesgaran
David Mesher
Steven Meshnick
Charles Meshul
Sam Mesiano
Irute Meskiene
Thibault Mesplede
Pedro Mesquita
Andrea Mess
Irene Messana
Smail Messaoudi
Ilhem Messaoudi
Evangelos Messaris
Steven Messe
Arnaud Messé
Louisa Messenger
Martin Messerle
Geralyn Messerlian
Elisa Messina
Frank Messina
Louis Messina
Daniel Messinger
Laurent Messonnier
Oto Mestek
Ermes Mestroni
Ana Mestrovic
Istvan Meszaros
Balint Meszaros
Christian Metallo
Anna Metaxas
Jessica Metcalf
Kelly Metcalf Pate
Beverly Metchock
Katja Metfies
Mehdi Metheni
Ulrich Methner
Raghu Metpally
Matteo Metruccio
Bart Metselaar
Rodolfo Metulini
Samy K. Metyas

Hans Metz
Thomas Metz
Verena Metz
J.R. Metz
Klaus Metzeler
Eric Metzen
Michael Metzen
Jean-Paul Metzger
David Metzger
Cesar Metzger
Florian Metzger
Ralf Metzler
Bernhard Metzler
Walter Metzner
Karin Metzner
Sascha Meudt
Frederic Meunier
David Meunier
Patrice Meunier
François Meurens
William Meurer
Marie-Jean Meurs
Mark Meuth
Theo Meuwissen
Ralph Meuwissen
Vildan Mevsim
Inga Mewis
Nathan Mewton
Alexandra Mey
Patrick Meybohm
Axel Meyer
David Meyer
Georg Meyer
Joel Meyer
Justin Meyer
Ana-Claire Meyer
Ron Meyer
Jeffrey Meyer
Jaimie Meyer
Rikke Meyer
Wallace Meyer
Philipp Meyer
Britta Meyer
Anne Meyer
Thomas Meyer
Sebastian Meyer
Kacie Meyer
Dorothy Meyer
Mathias Meyer
Gretchen Meyer
Saharon Meyer
Carolyn Meyer
Rachel Meyer
E. Meyer
Evelyne Meyer
Julie Meyer
Tim Meyer
Richard Meyer
Joseph Meyer
Wallace Meyer Iii
D'Arcy Meyer-Dombard
Andreas Meyerhans
Dieter Meyerhoff
David Meyerholz
Helen Meyer-Martin
Victor-Benno Meyer-Rochow
Goswin Meyer-Rochow
Gregor Meyers
Craig Meyers
Kathrine Meyers
Jason Meyers
Philip Meyers
Catherine Meyer-Schwesinger
Laura Meyerson
Anna Meyer-Weitz
Nicolai Vitt Meyling
I.A. Meynaar
Delphine Meynard
Alison Meynert
David Meyre
Esther Meyron-Holtz
Stephane Meystre
Cesar A. Meza-Herrera
Takahiro Mezaki
Christine Mezard
Mihaly Mezei
Eva Mezey
James Mezhir
Ferhat Meziani
Brianna Mezuk
Briana Mezuk
Alceu Mezzalira
Alessandro Mezzani
Laura Mezzanotte
Massimo Mezzavilla
Josué Mfopou

Sungano Mharakurwa
Shengli Mi
Xiangcheng Mi
Sha Mi
Guohua Mi
Chao Mi
Gu Mi
Marta Miaczynska
Caterina Mian
M.A. Rouf Mian
Maria Giuseppina Miano
Joseph Miano
Silvia Miano
Wei Miao
Junying Miao
Yi-Liang Miao
Jun Miao
Xiaoping Miao
Xuexia Miao
Long Miao
Qing Miao
Chiyuan Miao
Jianwei (Jojhn) Miao
Shida Miao
Wen Miao
Lifeng Miao
Yansong Miao
Alfredo Miccheli
Cristina Miceli
Antonio Miceli
Francesco Miceli
Mh Miceli
Thomas Miceli
Corinne Miceli-Richard
Ivan Micetic
Hallman D. Michael
Ciesielski Michael
Drew Michael
Helen Michael
J. Michael
Petrascheck Michael
Michael Michaelides
Patrick Michaels
Larry Michaelsen
Stella Michaelsen
Jakob Michaëlsson
Adina Michael-Titus
Tonkonogi Michail
George Michailidis
Marek Michalak
Tomasz Michalak
Johannes Michalak
Matthias Michalek
Richard Michalet
Sean Michaletz
Anna Michalik
Sara Michaliszyn
David Michalk
George Michalopoulos
Nv Michalopoulos
Panayiota Michalopoulou
Katarzyna Michalska-Malecka
Christoph Michalski
Stefan Michalski
Fernanda Michalski
Dominique Michaud
Isabelle Michaud-Soret
Marcela Michaut
Grégoire Michaux
Andrew Micheal
Olivier Micheau
Sylvain Michée
Jean-Baptiste Michel
Tatiana Michel
Andrew Michel
Anita Michel
Martin Michel
Marcus Michel
Meunier Michel
Brady Michel
Daniel Michele
Manuele Michelessi
Jerome Micheletta
Robert Micheletti
Carla Micheli
Marcia Michelin
Lisete Michelini
Nathalie Michels
Wieneke Michels
Jan Michels
Paul Michels
Brigitte Michelsen
Murugi Micheni
Denis Michez
Alison Michie
Jan Michiels

Carine Michiels
Alessandro Michienzi
Toshimi Michigami
Patrick Michl
Gracjan Michlewski
Daniela Michlmayr
Konstantinos Michmizos
Anna Michnik
Frederic Michon
François Michonneau
Franziska Michor
Laetitia Michou
Alan Mickelson
Elizabeth Micks
Mihai Miclau
Florin Miculescu
Rutger Middelburg
Jaap Middeldorp
Christel Middeldorp
Jinte Middeldorp
Keren Middelkoop
Jessica Middlemis Maher
Deborah Middleton
Sandy Middleton
Jeremy Midgley
Claire Midgley
Rajiv Midha
David Midmore
Anne Midwinter
Piotr Mieczkowski
Frank Miedema
Mihai Mieila
Michal Mielcarek
Luca Miele
Catherine Miele
Mara Miele
Manfred Mielenz
Michelle Mielke
John Mielke
H. Mielke
Sebastien Miellet
Michael Mienaltowski
Jan A. Miernyk
Roger Miesfeld
Elizabeth Mietlicki-Baase
Roger Mieusset
Egidia Miftode
Hussein Migdadi
Matteo Migheli
Federica Migliardo
Francesco Migliavacca
Maria Pia Miglietta
Maria Angelica Miglino
Daniele Migliorati
Lucia Migliore
Marco Donald Migliore
Ana Mignaqui
Paolo Mignatti
Sandrine Mignon-Grasteau
Bernard Mignotte
Morgan Mignyard
Magdalena Migocka
Célia Miguel
Irene Miguel-Aliaga
David Miguez
Joaquin Miguez
Catalina Mihai
Preda Mihailescu
Jovan Mihajlovic
Rafael Mihalic
Jozsef Mihaly
Frane Mihanovic
Sabine Mihm
Nagasawa Miho
Steve Mihok
Seema Mihrshahi
Fumika Mi-Ichi
Risto Miikkulainen
Agnieszka Mika
Carmen Mikacenic
K. Mikami
Yu Mikami
Charles Mikell
Thomas Mikeska
Linetsky Mikhail
Evgeny Mikhaylov
Yasuhiro Miki
Takeshi Miki
Atsuya Miki
Jørn Mikkelsen
Irene Mikkelsen
Jens Mikkelsen
Kristin Mikkelson
Sofia Mikko
Marja Mikkola
Stanley Miklavcic

Moïra Mikolajczak
Rafael Mikolajczyk
Ivana Mikolasevic
Susan Mikota
Emmanuel Mikros
David Mikulis
Katarina Mikusova
Ralf Mikut
Christian Mikutta
Montserrat Mila
Jelica Milanovic
Joseph Milanovich
Marciane Milanski
Matto Mildenberger
Diane Milenic
Tijana Milenkovic
Pavle Milenkovic
Jeremy Miles
Donald Miles
John Miles
Mary Miles
Nathan Miles
James Miles
Paulo Milet-Pinheiro
Maria Elena Miletto Petrazzini
Catarina Milheiriço
Wilbur Milhous
Stavroula Mili
Yoamel Milián-García
Pietro Milillo
Goran Miljus
James Millam
Jose Millan
Javier Millan
Ivan Millan
Ana Millanes
Jocelyn Millar
John Millar
Neil Millar
Anthony Millar
Seán Millar
Sarah Kate Millar
Charles Millard
Peter Millard
Andrew Millard
Thomas Millat
Dominic Millenaar
Christopher Miller
Daniel Miller
Thomas Miller
Scott Miller
Gifford Miller
Veronica Miller
Steve Miller
Richard Miller
Benjamin Miller
Michael Miller
Rachel Miller
William Miller
Ryan Miller
A. Miller
Yury Miller
Loren Miller
Jason Miller
David Miller
Mark Miller
Joshua Miller
Jennifer Miller
Robert Miller
Austin Miller
Luke Miller
Kai Miller
Jeremy Miller
Allison Miller
Nicholas Miller
Courtney Miller
Luis Miller
Ted Miller
Jordan Miller
Edgar Miller
Kyle Miller
Ashley Miller
Laurie Miller
Ross Miller
Charlotte Miller
Adam Miller
Zachary Miller
Matthew Miller
Lisa Miller
George Miller
Brandon Miller
Bradley A. Miller
Vandana Miller
Lindsey Miller
Timothy Miller
Alison Miller

Gary Miller
Marshall Miller
Paul Miller
Holly Miller
Gabrielle Miller
Tracie Miller
Thaddeus Miller
Anthony Miller
Amalia Miller
Michele Miller
Ezra Miller
David Miller Iii
Emily Miller-Cushon
Oscar Millet
Grégoire Millet
Juan-Pablo Millet
Laurent Millet
Kenneth Millett
Jonathan Millett
Glenn Millhauser
Erin Milligan
Gemma Milligan
Allen Milligan
Sophia Millington-Ward
Mulugeta Million
Alexander Millner
Alexandre Millon
Michael-John Milloy
Kingston Mills
Daniel Mills
Edward Mills
Ken Mills
Julia Mills
Ian Mills
Candice Mills
Richard Mills
Harriet Mills
Roger Milne
Tom Milne
Alice Milne
Barry Milne
Joel Milner
Adrienne Milner
Danny Milner, Jr.
E.J. Milner-Gulland
Stasa Milojevic
Genevieve Milon
Walter Milon
Eric Milot
Julie Milstien
Rachel Milte
Abul Hasnat Milton
Eugene Milus
Barbara Milutinovic
Sophie Mimigkou
Wang Min
Jung-Joon Min
David Min
Kyung Hoon Min
Jun-Ki Min
Paul Min
Na Young Min
Ahrum Min
Woong-Ki Min
Lillian Min
Yoshio Minabe
Behrouz Minaei-Bidgoli
Chieka Minakuchi
Eiichi Minami
Ushio Minami
Naojiro Minami
Tohru Minamino
Tetsuo Minamino
Toshinari Minamoto
Elaine Minatel
Peter Minchella
James Minchin
Gabriella Minchiotti
Diego Minciacchi
Mark Minden
Yohei Mineharu
Alessandro Minelli
Cosetta Minelli
Annalisa Minelli
Jeffrey Miner
Michael Miner
Adrienne Minerick
Alec Miners
Giovanni Minervini
Fabio Minervini
Yann Mineur
Ray Ming
Liu Ming
Wai-Kit Ming
Qinglei Ming
Xun Ming

Hsieh Ming-Fa
Matteo Minghetti
Wang Minghui
Federico Mingozzi
Geltrude Mingrone
Michael Mingueneau
Roberto Miniero
Vladimir Minin
Chris Minion
Baruch Minke
Monique Minnema
Martina Minnerop
Jens Minnerup
Petter Minnhagen
Scott Minnich
Robert Minns
Mari Mino
Philippe Minodier
Conor Minogue
Emily Minor
Richard Minshall
Jeremy Minshull
Claire Minshull
Katja Mintenbeck
Justine Mintern
Bruno Minto
Allen Minton
Akiva Mintz
Angelo Minucci
Roberto Minutolo
Filippo Minutolo
Vincent Minville
Svjetlana Miocinovic
Maria Caterina Mione
Lisa Miorin
Eneida Mioshi
Paolo Miotto
José Miotto
Dale Miquelle
Pablo Mir
Snober Mir
Alex Mira
Mariana Mirabel
Massimiliano Mirabella
Giovanni Mirabella
Lucia Mirabella
Sylvain Mirade
Maria Miragaia
Michele Miragoli
Miguel Miranda
Susan Miranda
Rajesh Miranda
Nelson Miranda
Juan Jose Miranda
Regina Miranda
Marcos Miranda
Ruben Miranda
Rafael Miranda
Gustavo Miranda-Carboni
Jezid Miranda-Quintero
Marc Mirande
Haralampos Miras
Claudio Mirasso
Ursula Mirastschijski
Marc Miravitlles
Clotilde Mircher
Chad Mire
Paul Mireji
Andrew Mirelman
Anat Mirelman
Liviu Mirica
Mario Mirisola
Santiago Gabriel Miriuka
Gianluca Mirizzi
Dragan Mirkov
Arash Mirmohammadsadeghi
Masoud Mirmomeni
Paria Mirmonsef
Ali Mirnajafizadeh
Victoria Mironova
Talya Miron-Shatz
Paola Mirra
Mehdi Mirsaeidi
Jon Mirsalis
Wilfried Mirschel
Hengameh Chloé Mirsepasi-Lauridsen
Tooraj Mirshahi
Alireza Mirshahi
Christen Mirth
Veriko Mirtskhulava
Aashiq Mirza
Shama Mirza
Taher Mirzahasanloo
Razmik Mirzayans
Masaya Misaki
Mario Misale

John Misasi
Norihiko Misawa
Kiyoshi Misawa
Harald Mischak
David Mischoulon
Ali Miserez
Masaki Mishima
Yuji Mishina
Rakesh Mishra
Nagendra Mishra
Sandip Mishra
D. Mishra
Deepak Mishra
Kumud Mishra
Umakant Mishra
Avshesh Mishra
Avinash Mishra
Bibhuti Mishra
Sujata Mishra
Shiraz Mishra
Virendra Mishra
Dheerendra Mishra
Seema Mishra
Paras Mishra
Sweta Mishra
Sachidananda Mishra
Amarjit Mishra
Vani Mishra
Sudhanshu Mishra
Geetanjali Mishra
Shashwat Mishra
Anshuman Mishra
S. R. Mishra
Michele Mishto
Bratislav Misic
Stefan Miska
Sabina Misoch
Stephanie Misono
Hari Misra
Rajeev Misra
Ravi Misra
Vikram Misra
Kamana Misra
Jyoti Misra
Prashant Misra
A. Misra
Shivani Misra
Nisha Misra
Biswapriya Misra
Bayan Missaghi
Gabriele Missale
Dorothée Missé
Caterina Missero
Edoardo Missiaglia
Dominique Missiakas
Julia Missitzi
Chuck Mistretta
Nerges Mistry
Ole Arve Misund
Jaroslaw Miszczak
Toshihiro Mita
Delphine Mitanchez
Yoshihide Mitani
Namiko Mitarai
Aaron Mitchell
James Mitchell
Thomas Mitchell
Jude Mitchell
Cassie Mitchell
William Mitchell
David Mitchell
John Mitchell
Timothy Mitchell
Derek Mitchell
Peter Mitchell
Claire Mitchell
Beverly Mitchell
Brett Mitchell
Hugh Mitchell
Andrea Mitchell
Christopher Mitchell
Lewis Mitchell
Phil Mitchell
Suzanne Mitchell
Anna Mitchell
Piers Mitchell
Mark Mitchell
Colter Mitchell
Mike Mitchell
Caroline Mitchell
Paul Mitchell
Alice Mitchell
Robert Mitchell
Michael Mitchell
Marci Mitchell
E. D. Mitchell

Gordon Mitchell
Maria Miteva
Daniela Miteva
Gilles Mithieux
Tijana Mitic
Oriol Mitjà
Arnold Mitnitski
Mihail Mitov
Ashim Mitra
Kasturi Mitra
Arkadeep Mitra
Aniruddha Mitra
Ritendranath Mitra
Pralay Mitra
Adinpunya Mitra
Sara Mitri
Nico Mitro
John Mitrofanis
Steve Mitroff
Ioannis Mitroulis
Marija Mitrovic Dankulov
Andreea Mitrut
Nicholas Mitsakakis
Dimitrios Mitsotakis
Tetsuya Mitsudomi
Hiroshi Mitsuzawa
Aditya Mittal
Vinay Mittal
Jeetain Mittal
Manish Mittal
Mukul Mittal
Shuchi Mittal
Ravinder Mittal
Ashwani Mittal
Michel Mittelbronn
Maurice Mittelmark
Bettina Mittendorfer
Ellenor Mittendorfer Rutz
Birgit Mitter
Sayak Mitter
Holger Mitterer
Anthony Mittermaier
Philipp Mitteroecker
Thomas Mittmann
Luigi Mittone
Erik Mittra
Wayne Mitzner
Andrei Miu
Kazutoyo Miura
Kiyonori Miura
Takeshi Miura
Kenji Miura
Tetsuji Miura
Shin-Ichiro Miura
Tanya Miura
Shinji Miura
Masanobu Miura
Kenjiro Miura
Hiroto Miwa
Makoto Miwa
Masato Miwa
Masaki Miya
Yohei Miyagi
Motohide Miyahara
Yoshihiro Miyahara
Yasushi Miyahira
Tempei Miyaji
Tsuyoshi Miyakawa
Hitoshi Miyakawa
Yasunobu Miyake
C. Miyake
Sanae Miyake
Noriko Miyake
Cristina Yumi Miyaki
Yuichiro Miyamatsu
Shigeki Miyamoto
Hiroomi Miyamoto
Takeshi Miyamoto
Naokazu Miyamoto
Yohei Miyamoto
Hiroshi Miyamoto
Akio Miyao
Makoto Miyara
Naoyuki Miyasaka
Akinori Miyashita
Hiromitsu Miyata
Mutsumi Miyauchi
Katsumi Miyauchi
Takashi Miyauchi
Shouichi Miyawaki
Makoto Miyazaki
Tetsuro Miyazaki
Kentaro Miyazaki
Yoichi Miyazaki
Masao Miyazaki
Masaaki Miyazawa

Takayuki Miyazawa
Eiji Miyoshi
Noriyuki Miyoshi
Toru Miyoshi
Tohru Miyoshi-Akiyama
Ivana Mizikova
Atsushi Mizoguchi
Junya Mizoi
Yusuke Mizokami
Eshchar Mizrachi
Yaffa Mizrachi Nebenzahl
Kiyomi Mizugishi
Kenji Mizuguchi
Yoshiaki Mizuguchi
Hiroyuki Mizuguchi
Hiroki Mizukami
Kenji Mizumoto
Kazue Mizumura
Kensaku Mizuno
Carolina Megumi Mizuno
Masaki Mizuno
Y. Mizuno
Tsunehiro Mizushima
Shunsaku Mizushima
Katsumi Mizuta
Sayaka Mizutani
Michael Mizwa
Stefano Mizzaro
Ger Mjoes
Daud Mk
Milan Mladenovic
Snezana Mladenovic Drinic
Miranda Mladinic
Radoslaw Mlak
Godfree Mlambo
Koleka Mlisana
Wiktor Mlynarski
Kristin Mmari
Xiaokui Mo
Yin-Yuan Mo
Delin Mo
Jinhan Mo
Xiu-Lei Mo
Xingguo Mo
Phoenix Mo
Huan Mo
Gila Moalem-Taylor
Catherine Moali
William Moar
Helen Susannah Moat
Peter Moate
Rex Moats
Hossein Moayedi
Massieh Moayedi
Mehran Moazen
Mehdi Moazzez Lesko
Ali Mobasheri
Kenneth Moberg
Nadja Møbjerg
William Mobley
J. Mocco
Simone Mocellin
Fanny Mochel
Yoshiyuki Mochida
Nobuyoshi Mochizuki
Hideki Mochizuki
Maja Mockenhaupt
Robin Mockett
Attila Mocsai
Rahul Modak
Mohammad Modaressi
Marc Modat
Maria Grazia Modena
Letizia Modeo
Vengamanaidu Modepalli
Pietro Amedeo Modesti
Alessandra Modi
Girish Modi
Ankita Modi
Jaime Modiano
Gemma Modinos
Helmout Modjtahedi
Mike Modo
Sepideh Modrek
Susanne Modrow
Dominique Modrowski
Hanini Modugu
Karsten Mody
Orson Moe
Borge Moe
Luke Moe
Stein R. Moe
Angelica Moe
Carolyn Moehling
Patricia Moehlman
Amanda Moehring

Charles Moehs
Cathy Moelans
Dirk Moelants
Jesper Moelgaard
Alexander Moeller
Hanne Moeller
Scott Moeller
Christian Moellmann
Erika Moen
Thomas Moench
Cecilia Moens
Ugo Moens
Katrien Moens
Yves Moens
Siver Moestue
Jennifer Moffat
John Moffat
Scott Moffat
Sarah Moffitt
Mohammad Mofrad
Hammed Mogaji
Vittal Mogasale
Trine Mogensen
Stephen Moggach
Aldo Moggio
Seyed Moghadas
Alireza Moghisi
Jeffrey Mogil
Alex Mogilner
Axel Mogk
Daniel Mograbi
Christina Mogren
Majid Mohajerani
Mohd Saberi Mohamad
Hamid Mohamadi
Jamal Mohamed
Junaith Mohamed
Rozi Mohamed
Hossam Eldin Mohamed
Hamza Mohamed
Mohamed Mohamed
Muhanad Mohamed
Feroze Mohamed
Nik Mohd Izham Mohamed Nor
Abdul Khader Mohammad
Haroon Mohammad
Reza Ali Mohammadpour
Ata Ur Rasheed Mohammed
Altaf Mohammed
Abdulaziz Mohammed
Ibrahim Mohammed
Kamal Mohammed
Atif Mohammed
Abdelrahman Tarek Mohammed
Noor Adelyna Mohammed Akib
S. Venkata Mohan
Maradumane Mohan
Chilukuri Mohan
Sumit Mohan
Amita Mohan
C.D. Mohan
Viswanathan Mohan
Anita Mohan
Battini Mohan Reddy
Thalachallour Mohanakumar
Sanjeeva Mohanam
Manoj Mohanan
Tapan Mohanta
Debasisa Mohanty
Sanjay Mohanty
Chandan Mohanty
Kishore Mohanty
Bidyut Mohanty
Keshar Mohanty
Jogeswar Mohapatra
Satyabrata Mohapatra
Saeid Moharramipour
Akif Mohd
Farizah Mohd Hairi
Khayriyyah Mohd Hanafiah
Fauziah Mohd Jaafar
Mohd Kamal Mohd Nawawi
Norazmi Mohd Nor
Siti Mohd Ramli
Narazah Mohd Yusoff
Katia Mohindra
Vindhya Mohindra
Marghoob Mohiyuddin
Christoph Möhl
Peter Mohler
Betty Mohler
Mark Mohnac
Christine Mohr
Alicia Mohr
Ralf Mohrmann
Aaron Mohs

Kamran Mohseni
M. Mohsenii
Alexander Mohseny
Samantha Mohun
Muhammad Raheel Mohyuddin
Meng Ling Moi
Syed Moin
Amir Moin
Christophe Moinard
Anne Moir
Robert Moir
Robyn Moir
Gavin Moira
Nathalie Moiré
Marie-Pierre Moisan
Pia Moisander
Alexander Moiseev
Jennifer Moisi
Christine Moissl-Eichinger
Nazia Mojib
Marija Mojic
Slavko Mojsilovic
K. Hun Mok
Chi Chiu Mok
Hin-Kiu Mok
Ka Pun Mok
Vincent Mok
Clara Mok
Hyejung Mok
Aurelia Mok
Nicola Mok
Thabiso Mokotjomela
Andreas Mokros
Anna Mokrowiecka
Lauren Mokry
Sho Mokuda
Jan Mol
Ben Mol
Goudarz Molaei
Even Moland
Samat Moldakarimov
Lyle Moldawer
Daniel Molden
Florina Moldovan
Gomotsegang Fred Molelekwa
Peter Molenaar
Dylan Molenaar
Pascal Molenberghs
Gloria Molero
Alessio Molfino
Jérome Molimard
Antonio Molina
Jennifer Molina
Esther Molina
Anthony Molina
Rafael Molina
Samuel Molina
Rafael Molina Soriano
Alvaro Molina-Cruz
Henriette Molinari
Filippo Molinari
Marco Molinari
Sabrina Molinaro
Miguel-Angel Molina-Vila
Diana Molino
Markus Molis
Mark Molitch
John Molitor
Yaroslav Molkov
Markus Moll
Gianluca Molla
Diego Molla Aliod
Mehdi Mollapour
Freerk Molleman
Lucas Molleman
Brit Mollenhauer
Peter Moller
Andreas Möller
Sören Möller
Korbinian Möller
Anders Møller
Per Møller
Niels Møller
Christian Möllers
Luca Mollica
Helge Möllmann
Sabee Molloi
Peter Molloy
Anne M Molloy
Bonnie Molly
Andreea Molnar
Julia Molnar
István Molnár
Gerard Moloney
Carla Molthoff
Carolina Moltó-Puigmartí

Michelle Momany
Frank Momburg
Reza Momenan
Navneet Momi
Amin Momin
Claudia Momo
Eiichi Momotani
Anne Mette Momsen
Stefano Mona
Lucia Monacis
Denise Monack
Cristián Monaco
Alessandro Monaco
Fabrizio Monaco
Ara Monadjem
Andy Monaghan
Sean Monaghan
Paul Monahan
William Monahan
Manhanzva Monalisa
Gerald Monard
Remi Monasson
Octavio Monasterio
Jean-Marc Moncalvo
Roy Moncayo
Darren Monckton
Spencer Monckton
Bernard Moncla
Dinesh Mondal
Debrapriya Mondal
Tapan Mondal
Arijit Mondal
Partha Mondal
Sukanta Mondal
Kajari Mondal
Pinki Mondal
Stefania Mondello
Yuki Monden
Guillaume Mondesert
Mateus Mondin
Charles Mondo
Andrea Mondoni
Edward Mondor
Stanislas Mondot
Lucie Mondoulet
Bernardo Monechi
Thomas Monecke
Pablo Monedero
Ahmed Moneim
Rafael Moner
Marc Monestier
Bruce Monger
Philippe Monget
Gianluigi Mongillo
Marco Mongillo
Skorn Mongkolsuk
Pattanasak Mongkolwat
Monica Mongodi
Cojocaru Monica
Tom Monie
Lamers Monique
Marcin Moniuszko
Norihito Moniwa
Sonia Moniz
Arta Monjazeb
David Monk
Peter Monk
Jennifer Monk
Juha Mönkäre
Shannon Monnat
M.S. Monnazzi
Fanny Monneaux
Erik Monness
Xavier Monnet
Vincent Monnier
Philippe Monnier
Catherine Monnier
Evelyn Monnikhof
Thibaud Monnin
Rosa Monno
Michel Monod
Jaakko Mononen
Marco Monopoli
Cara Monroe
Emy M. Monroe
Courtney Monroe
Oscar Monroig
Octavio Monroy-Vilchis
Ute Mons
Rafael Monsanto
Stephen Monsell
Pablo Monsivais
Eva Monsma
Nancy Monson
Daniel Monson
Anne-Helene Monsoro-Burq

Michael A. Mont
Annika Montag
Cristina Montagna
Alexandra Montagner
Sara Montagnese
Michael Montague
Clara Montagut
Manuel Montalbán-López
Tiziana Montalcini
Giovanni Montana
Federica Montanaro
Lorenzo Montanaro
Joel Montane
Joan Montaner
Jean-Pierre Montani
Barbara Montanini
Gerard Montarou
Grégoire Montavon
Ernest Montbrio
Jin Kim Montclare
Mireille Montcouquiol
Martin Monte
Milena Monte
Enrique Monte
Andréa Monte Alto Costa
Adrienne Monteath-Van Dok
Martin Montecino
Alessandra Montecucco
Cesare Montecucco
Carlomaurizio Montecucco
Maria Montefinese
Mirco Montefiori
Arnaud Monteil
Alvaro Monteiro
Renato Monteiro
Mário Monteiro
Wuelton Monteiro
Lara Monteiro
Filipa Monteiro
Isabel Monteiro Dos Santos Pires
R.S. Monteiro-Junior
Claudia Monteiro-Vitorello
Daniel Montello
Marcelo Montemurro
Filippo Montemurro
M. Angeles Montero
Christopher Monterola
Monica Montero-Lomeli
John Monterosso
Pavel Montes De Oca Balderas
Roberto Montesano
Francesco Fabiano Montesano
Daniela Montesarchio
Marco Antonio Montes-Cano
Monica Montesi
Paula Montesinos
Pau Montesinos
Xavier Montet
Mark Montforts
Grant Montgomery
Stephen Montgomery
Ann Montgomery
Christopher Montgomery
John Montgomery
Catharine Montgomery
Susanne Montgomery
Jolynn Montgomery
Eugenio Monti
Fabiano Montiani
Marcela Montico
Silvia Monticone
Robson Q Montiero
Jean-Luc Montillet
Giovanni Montini
Rosario Montirosso
Lluis Montoliu
Kathleen Montone
Guillermo Montoya
Jose Montoya
Pedro Montoya
Estrella Montoya
Jaime Montoya
Ben Montpetit
Josep Maria Montserrat
Nunzia Montuori
Paolo Montuschi
Akm Monwarul Islam
Mark Mon-Williams
Makama Monyeki
Fabio Monzani
Lianet Monzote
Keymanthri Moodley
Dhayendre Moodley
Arshnee Moodley
Anand Moodley
Jayajothi Moodley

Leon Moodley
James Moody
Eric Moody
M. Moody
John Moody
Kelsey Moody
Christiane Moog
Frits Mooi
Simon Mooijaart
Piers Mook
Neeloffer Mookherjee
Wouter Moolenaar
Anne Moon
Richard Moon
Aree Moon
Rebecca Moon
Jee Youn Moon
Hee Sun Moon
M. Moon
Robert W Moon
Du Geon Moon
Hyejin Moon
Jin Moon Kim
Patrick Moonan
Justine Moonen
Jan-Renier Moonen
David Mooney
Rachael Mooney
Peter Mooney
Edward Mooney
Lieve Moons
Tim Moons
Helen Moor
Jon Moorby
Frank Moore
Robert Moore
David Moore
Lee Moore
Daniel Moore
Julie Moore
Holly Moore
Lorna Moore
Michael Moore
Christopher Moore
Edward Moore
Jean-Sebastien Moore
James Moore
Elizabeth Moore
Jeff Moore
Pippa Moore
Frederick Moore
Thomas Moore
Martin Moore
C. Mark Moore
Terry L Moore
Raeanne Moore
Justin Moore
Nicole Moore
Kerryn Moore
Ashlee Moore
Ignacio T. Moore
Don Moore
Roger Moore
Isabel Moore
Aubrey Moore
John Moore
Andrew Moore
Linda Moore
Alec Moore
Geromy Moore
Christopher Moorman
Randall Moorman
Bhagavatula Moorthy
Krishnan Moorthy
Ali A. Moosavi-Movahedi
Shirin Moossavi
Rajshree Mootanah
Henning Mootz
Tsafrir Mor
Nilly Mor
Cordula Mora
Conchi Mora
Isabel Mora
Freddy Mora
Norhashimah Morad
Tahereh Moradi
Mahmoud Moradi
Ghobad Moradi
Mehdi Moradi
Maziar Moradi-Lakeh
Farhad Moradpour
Milton Moraes
Christopher Moraes
Fernando Moraes
Thyago Moraes
Eder Moraes

Chris Moraes
Denis Altieri Moraes
Santiago Mora-Garcia
Andreas Moraitis
Pedro Moral
Raul Moral Herrero
Daniela Morale
Albert Morales
Marisela F Morales
Maria Aurora Morales
Jose Manuel Morales
Olivier Morales
Manuel Morales
Luis Orlando Morales
David Morales
Alfredo J. Morales
Noppawan Morales
Daniela Morales-Espinosa
José A. Morales-González
Julio Morales-Medina
Juan Morales-Ramos
A. Morales-Rivera
Giulliana Moralez
Vincenzo Moramarco
Hector Mora-Montes
Nancy Moran
Chris Moran
Colin Moran
Andrew Moran
Michael Moran
Julio Moran
Niamh Moran
Ellen Moran
Meghan Moran
Teresa Moran
Robert Moran
Paloma Moran
Verónica Morán-Barroso
Serge Morand
Alessandro Morandi
Anita Morandi
Joan Moranta
Silvia Morante
Javier Morante
José Carlos Morante-Filho
Raymond A. Moranz
Arturo Mora-Olivo
Andrea Morash
Telmo Morato
Chantal Moratz
Stephan Morbach
Dean Morbeck
Héctor Morbidoni
Glilciane Morceli
Erin Mordecai
Dana Mordue
Jennifer Mordue-Luntz
Herve Moreau
Caroline Moreau
Dimitri Moreau
Jerome Moreaux
Jan Moreb
Luciana Moredo
Andrea Morehouse
Andre Moreira
Leila Moreira
Monica Moreira
Helena Moreira
Irina Moreira
Xoaquin Moreira
P.I. Moreira
Pedro Moreira
João Moreira
Ernesto Moreira
Maria Aparecida Scatamburlo Moreira
Thereza Moreira
Rodrigo Moreira
Carlos Moreira-Filho
Sandrine Morel
Laurence M Morel
Franck Morel
Penelope Morel
Etienne Morel
Robert Morell
Patricia Morellato
Nicolas Morellet
Micaela Morelli
Lorenzo Morelli
Federico Morelli
Andrea Morelli
Laura Morello
Kelley Moremen
Constanza Moren
Maria Morena
Carlos Moreno
Yamir Moreno

Beatriz Moreno
Antonio Moreno
Juan Antonio Moreno
Ignacio Moreno
Fernando Moreno
David Moreno
Ernesto Moreno
Cristina Moreno Gutiérrez
Aida Moreno Moral
Ruben Moreno-Bote
Miguel Moreno-García
Gabriel Moreno-Hagelsieb
Laura Moreno-López
Miguel Moreno-Mateos
José María Moreno-Navarrete
Juan Moreno-Navas
Rubén Moreno-Opo
Ricardo Moreno-Rodríguez
Jose Manuel Moreno-Rojas
Raquel Moreno-Varcarcel
Rosamaria Moresco
David Moretti
Antonio Moretti
Riccardo Moretti
Matteo Moretto Zita
Leslie Morey
Mariza Morgado
Antonio Morgado
Ann Morgan
Ian Morgan
Dave Morgan
Victoria Morgan
William Morgan
Todd Morgan
John Morgan
Katy Morgan
Matt Morgan
Matthew Morgan
Thomas Morgan
Fraser Morgan
Stephanie Morgan
Phil Morgan
Drake Morgan
Ethan Morgan
Elaine Morgan
Brent Morgan
Rachael Morgan-Kiss
Mary Morgan-Richards
Francesca Morgante
Michelangelo Morganti
Timothy Morgenthaler
Floriana Morgillo
Tetsuya Mori
Hisashi Mori
Kiyoshi Mori
Scott Mori
Masayuki Mori
Rintaro Mori
Masaki Mori
Takefumi Mori
Akira Mori
Kanji Mori
Kazuhiko Mori
Takeshi Mori
Emiliano Mori
Koji Mori
Danilo Moriel
Clément Morier
Richard Moriggl
Marina Morigi
Takaya Moriguchi
Takashi Moriguchi
Kennichi Morikawa
M Mori-Kawabe
Dimitrios Morikis
Yasujiro Morimitsu
Richard Morimoto
Sachio Morimoto
Kozo Morimoto
Naoki Morimoto
Satoshi Morimoto
Fabrice Morin
Andrew Morin
Shai Morin
Benjamin Morin
Alain Morin
Timothy Morin
Benoit Morin
Olivier Morin
Karen Morin
Miguel Morinigo
Cedric Morio
Beatrice Morio Liondore
Ichiro Morioka
Marco Moriondo
Andrea Moriondo

Demetrios Moris
Takayuki Morisaki
Naho Morisaki
Kunio Morishige
Ryuichi Morishita
Yoshiaki Morishita
Christophe Morisseau
Mathieu Morissette
Kouichi Morita
Takashi Morita
Mitsuhiro Morita
Masaya Morita
Yasu Morita
Akio Morita
Toshisuke Morita
Daisuke Morito
Orson Moritz
Karen Moritz
Erin D. Moritz
Hisao Moriya
Toshiki Moriyama
Ignacio Moriyon
Markus Morkel
Gunnar Morken
Iwona Morkunas
Simon Morley
Neil Morley
Michael Morlock
Pierre Mormède
Roger Mormul
Laura Moro
Esteban Moro
Andrea Moro
Ana Moro
Loredana Moro
Dorian Moro
Matteo Moro
Massimo Moro
Ana Moro-Egido
Jose Moron
Renato Morona
Jose Morones-Ramirez
Rafael Mendes Moroni
Yuki Morono
Gota Morota
Susan Morpeth
Carla Morri
Sheldon Morris
Sidney Morris
Roger Morris
John Morris
Alison Morris
Margaret Morris
Joan Morris
Gilbert Morris
Shaun Morris
J. Morris
Katrina Morris
Brian Morris
Richard Morris
Ulrika Morris
Aaron Morris
Robert Morris
Meghan Morris
H. Douglas Morris
Andrew Morris
Gerwyn Morris
Paul Morris
Wayne Morris
Kirsty Morris
Lilian Morris
Joanna Morris
Katherine Morris
Christopher Morris
Meg Morris
Gerald Morris
David Morris
Donald Morrison
John Morrison
Trudy Morrison
Shaun Morrison
Lynda Morrison
Thomas Morrison
Christopher Morrison
Brett Morrison
Edward Morrison
Janna Morrison
Greg Morrison
Todd Morrison
Bob Morrison
Douglas Morrison
Gillian Morrison
Alexandra Morrison
Tiffany Morrison
Juliet Morrison
Kathryn Morrison

Rob Morrison
James Morris-Pocock
Michael Morrissey
Robert Morrissey
Ember Morrissey
James Morrissey
Amelia Morrone
Doralisa Morrone
Casey Morrow
Ardythe Morrow
Bernice Morrow
Kathleen Morrow
Jonathan Morrow
Kathy Morrow
Eric Morschhauser
Joachim Morschhäuser
Christian Morsczeck
Martin Mörsdorf
Randall Morse
David Morse
Cindi Morshead
Giulia Morsica
Mohamed Morsy
Renato Mortara
Behzad Mortazavi
Sara Mortaz-Hejri
Richard Mortensen
Eric Mortensen
Brent Mortensen
Erik Mortensen
Michelle Morters
Geert Mortier
Anneleen Mortier
Kevin Mortimer
Jenny Mortimer
Ann Mortimer
Susan Morton
Jennifer Morton
Douglas Morton
Cynthia Morton
David Morton
James Morton
Katie Morton
Sarah Morton
Gerard Morton
Katherine Morton
Ryan Morton
Alec Morton
Franck Mortreux
Kareem Mosa
Sameh Mosaed
Anisa Mosam
Roberto Mosca
Elena Mosca
Alessandra Mosca
Ettore Mosca
Federica Moschella
Monica Moschioni
Fabio Mosconi
Fermin Moscoso Del Prado Martin
Liza R. Moscovice
Michael Moseley
Gregory Moseley
Rainer Mosenthin
Muriel Moser
Martin Moser
Claus Moser
Gerhard Moser
Jürgen Moser
Florian Moser
Stephen Moses
Louis Moses
Eydie Moses-Kolko
Han Moshage
Morten Moshagen
Deane Mosher
Darius Moshfeghi
Ali Moshiri
Rabih Moshourab
Gr Moshtaghi-Kashanian
Valentina Mosienko
Emily Mosites
Mor Moskovitz
Judith Moskowitz
Andrew Moskowitz
Roxana Moslehi
Mohammad Kazem Moslemi
Jonathan Mosley
Ian Mosley
Rana Mosli
Bernard Moss
Stephen Moss
Paul Moss
Joel Moss
Jennifer Moss
Scott Moss

Stuart Moss
Tim Moss
Travis Moss
Georg Mossböck
Enrico Mossello
Anna Mosser
Andrew Mossholder
Valeri Mossine
Cristina O. Mosso
Joel Mossong
Corrine Moss-Racusin
Bonifacio Mostacedo
Heba Mostafa
Sarah Mostafavi
Arash Mostaghimi
Klara Mosterd
Lizel Mostert
Ivan Moszer
Luís Mota
Manuel Mota
Paula Mota
N. Motallebi
Mohammad Hosein Kalantar Motamedi
Ryosuke Motani
David Mota-Sanchez
Sei-Ichiro Motegi
Walther Mothes
Isabelle Mothe-Satney
Rajender Motiani
Ruta Motiejunaite
Vladimir Motin
Tasneem Motiwala
Robert Motl
Lukas Motloch
Norio Motohashi
Hozumi Motohashi
Yuri Motorin
Justin Mott
Alicia Motta
Hans Motte
Roberto Motterlini
Amy Mottl
David Motto
Marcella Mottolese
Laurent Mottron
Matti Mottus
Harvey Motulsky
Carol Anne Motycka
Katherine Motyl
Jin Mou
Xuanqin Mou
Corrina Moucheraud
Gastón Adolfo Mougabure Cueto
Faiza Mougari
Gregory Mouille
Judd Moul
John Moulder
Florent Mouliere
Thierry Moulin
David Moulin
Margaret Moulson
Simon Moulton
Catherine Mounier
Andrew Mount
George Mount
Adrian Mountford
Giorgos Mountrakis
John Mountz
Giannis Mountzios
Kostas Mountzouris
Hugo Mouquet
Raphael Mourad
Pierre Mourad
Paulo Mourão
André Mouraux
Zissimos Mourelatos
Gabriel Mourente
Jean-Baptiste Mouret
Johann Mourier
Eve Mourier
Rosa Mouriño-Pérez
Laurent Mourot
Rogier Mous
Paula Mouser
Zaki Moushira
Charbel Moussa
Fathi Moussa
Ahmed Moussa
Adel Moussa
Nabila Moussaoui
Dina Moustafa
Naima Moustaid-Moussa
Petros Moustardas
Heta Moustgaard
Joanne Mouthaan
Catia Moutinho

Laurence Mouton
Dimitrios Moutopoulos
Christina Moutsiana
Loukas Moutsianas
Jamileh Movassat
Liviu Movileanu
Allan Mowat
Garth Mowat
Danielle Mowery
Chris Mowry
Abbe Mowshowitz
Linda Moxey
John Moxnes
Joe Moxon
T. Moxon
Marilyn Moy
Patricia Moya
Natali Moyal
Francisco Moyano
Jennifer Moye
Jack Moye Jr
Craig Moyer
Cheryl Moyer
Alison Moyer
Scott Moye-Rowley
Bernard Moyersoen
David Moyes
Jennifer Moylan
Cynthia Moylan
Peter Moyle
Wendy Moyle
Sizulu Moyo
Rosa Moysés
Mahmood Mozaffari
Khyobeni Mozhui
Ioana Mozos
Andrea Mozzarelli
Tomáš Mrácek
Fatima Mraiche
Marinka Mravak-Stipetic
Patrik Mraz
Jan Mrazek
Andrea Mroginski
Pawel Mroz
Michal Mrug
Malgorzata Mrugacz
Philippe Msellati
Gerry Mshana
Yuguang Mu
Xiaodong Mu
David Mu
Wei-Ping Mu
Rong Mu
Qiwen Mu
Shengyu Mu
Yuming Mu
Donald Muccio
Giulio Muccioli
Nathan Muchhala
Antoine Muchir
Carla Mucignat-Caretta
Thomas Mücke
Divya Mudappa
Raksha Mudar
Simon Mudd
Joseph Mudd
Giridhar Mudduluru
Vivek Mudera
Agnieszka Mudge
Rajini Mudhasani
Amritpal Mudher
Anuradha Mudipalli
Maria Mudryj
Brian Muegge
Carolyn Muegge
Thomas Muehlbauer
Andreas Muehlberger
Daniel Muehlschlegel
Lukas Mueller
Jonathan Mueller
Karsten Mueller
Susette Mueller
Rachel Mueller
Jakob Mueller
Anne Mueller
Ann-Kristin Mueller
Roman-Ulrich Mueller
Rolf Mueller
Timo Mueller
Erik Mueller
Niklaus Mueller
Sebastian Mueller
Markus Mueller
Wendt Mueller
Julie Mueller
Robert Mueller

Gregory Mueller
Christian Mueller
Shane Mueller
Christine Mueller
Jacob Mueller
Pavel Mueller
Patricia W. Mueller
Karin Mueller-Decker
Wolfgang Mueller-Klieser
Frank Mueller-Langer
Gerald Muench
Stephen Muench
Marcus Muench
Maximilian Muenchhoff
Felix Muerdter
Karsten Muessig
Ryan Muetzel
Stefan Muetzel
Raja Mugasimangalam
Amin Mugera
Lucia Muggia
Lapo Mughini-Gras
Emmanuel Mugisha
Rogerio Mugnaini
Begoña Muguerza
Ferdinand Mugusi
Taseer Muhammad
Abdul Kadar Muhammad Masum
Heiko Mühl
Felix Muhlanga
M. Mühlau
Axel Mühlbacher
Christian Mühlfeld
Beverly Muhlhausler
Barbara Muhling
Virpi Muhonen
Khitam Muhsen
Balasingam Muhunthan
Joshua Muia
Paolo Muiesan
Christopher Muir
Keith Muir
Paul Muir
Andrew Muir
Kristy Muir
James Muir
Eric Muir
Vanessa Muirhead
Robin Muise-Helmericks
Mario Mujica-Mota
Elizabeta Mukaetova-Ladinska
Chinatsu Mukai
Takao Mukai
Naofumi Mukaida
Dana Mukamel
Eran Mukamel
Masafumi Mukamoto
David Mukanga
Sushmita Mukerjee
Bipasha Mukherjee
Krishnendu Mukherjee
Malay Mukherjee
Jogeshwar Mukherjee
Prasun Mukherjee
Pinku Mukherjee
Kuntal Mukherjee
A. Mukherjee
Konark Mukherjee
Shomen Mukherjee
Nabanita Mukherjee
Amit Mukherjee
Sunil Mukherjee
Rahul Mukherjee
Kanchan Mukherjee
Indrani Mukherjee
Abhijit Mukherjee
Animesh Mukherjee
Moumita Mukherjee
Shubhra Mukherjee
Raju Mukherjee
Sangita Mukhopadhyay
Asish Mukhopadhyay
Debashis Mukhopadhyay
Anirban Mukhopadhyay
Somshuvra Mukhopadhyay
Suman Mukhopadhyay
Kunal Mukhopadhyay
Dipta Kanti Mukhopadhyay
Sudipta Mukhopadhyay
Partha Mukhopadhyay
Arpita Mukhopadhyay
Hasan Mukhtar
Toru Mukohara
Amar Mukund
Ramanjaneya Mula
Marco Mula

Paolo Mulatti
Ajitkumar Mulavara
Daniel Mulcahy
Nicholas Mulcahy
Christian Mulder
Frans Mulder
Martijn Mulder
Kathleen Mulder
Laetitia Mulder
Petra Mulder
Mark Mulder
Gundula H Müldner
Katherine Muldoon
Leslie Muldoon
James Mule'
Miquel Mulero
José Mulet
Carly Muletz
Rita Mulherkar
Shalaka Mulherkar
Luke Mullany
Peter Mullany
Lisa Mullany
Caitlin Mullarkey
Isis Mullarky
Michael Mullen
William Mullen
Sean Mullen
Sylviane Muller
Daniel Muller
Claude Muller
Kenneth Muller
Yves Muller
Dominique Muller
Bernard Muller
Christian Muller
Martina Muller
Yunhua Muller
Alexandra Muller
Erinn Muller
Olivier Muller
Kayleigh Muller
Jean Muller
Eric Muller
Ulrike Muller
Sean Muller
François L L Muller
Elmi Muller
Michael Müller
Andreas Müller
Norbert Müller
Ferenc Müller
Martin Müller
Caroline Müller
Henning Müller
Henry Müller
Hans-Peter Müller
Matthias Müller
Wolfgang Müller
Christian Müller
Hermann Müller
Martijn Müller
Markus Müller
Barbara Müller
Alexandra Müller
Pavel Müller
Francuois Müller
Loretta Müller
Arndt-Christian Müller
Christina Andrea Müller
Thomas Müller
Gabriele Müller-Mundt
Ralf Müller-Xing
Patrick Mullie
Lois M Mulligan
R. Mulligan
Mark Mulligan
Stephen Mulligan
Jennifer Mulligan
Christopher Mullin
Conrad W. Mullineaux
Robert Mullins
Niamh Mullins
David Mullins
Benjamin Mullins
Michael Mullins
Joaquim Mullol
Brian Mulloney
James Mulloy
Paula Mulo
Melissa Mulraney
Gerd Multhaup
Gerhard Multhaup
Gabriele Multhoff
Don Mulvaney
Jason Mulvenna

Michael Mulvey
Minal Mulye
Joyce Mumah
Kosta Mumcuoglu
Elizabeth Mumford
John Mumford
C.L. Mumford
Rita Mumm
Christine Mummery
Joung Hwan Mun
Marcus Munafo
Masanori Munakata
Tsubasa Munakata
Carine Munaut
Jelena Muncan
Jan Munch
Inger Christine Munch
Elizabeth Munch
Daniel Münch
Philipp Münch
John Munday
Ulrike Munderloh
Roger Mundry
Adrian Mundt
William Mundy
Rachata Muneepeerakul
Asif Muneer
Ken Muneoka
Christopher Mungall
Kenneth Munge
Kassandra Munger
Joshua Munger
Carolina Munhoz
Arasambattu Kannan Munirajan
Ranganath Muniyappa
Graciela Muniz Terrera
Carsten Münk
Tamara Munkemuller
Sarah Munkholm
Lance Munn
Sergi Munné-Bosch
Jose Munoz
Francois Munoz
Manuel Munoz
William Munoz
Jessian Munoz
Alvaro Munoz
Angel Munoz
Ernesto Munoz
Alberto Muñoz
Miguel Muñoz
Purificación Muñoz
Ana Muñoz
Victor Muñoz
Pilar Muñoz
Luis Muñoz
M. Ángeles Muñoz-Fernández
Cesar Munoz-Fontela
Violeta Munoz-Fuentes
Felix Muñoz-García
Cristina Munoz-Pinedo
Dany Munoz-Pinto
Luisa Silvia Munoz-Price
Sara Muñoz-Vallés
Claudia Munoz-Zanzi
Carol Munro
Kevin Munro
Melissa Munroe
Samantha Munroe
Anupama Munshi
George Munson
Jennifer Munson
Vincent Munster
Stefan Munster
Andrea Münsterberg
Leonard Munstermann
Jordi Muntane
Thomas Münte
Danina Muntean
Andreea Munteanu
Shannon Munteanu
Else Munthe
Lea Munthe-Fog
Joseph Munyaneza
Kylie Munyard
Christian Munz
Thomas Münzel
Valeria Muoio
Miratul Muqit
Marieke Mur
Catherine Mura
Gioia Mura
Maurizio Muraca
Mohammad Hassan Murad
Koji Murai
Eric Muraille

Kazuma Murakami
Takashi Murakami
Hiroki Murakami
Makoto Murakami
Yoshitaka Murakami
Hiroshi Murakami
Masanao Murakami
Michio Murakami
Jessica Murakami
N. Murakami
Shin Murakami
Satoshi Muraki
Katsuhiko Muraki
A. Muraleedharan
Kasinathan Muralidharan
Sribalasubashini Muralimanohara
Eduard Murani
Wayne Muraoka
Daisuke Muraoka
Paolo Muraro
Elena Muraro
Claude Murat
Atsuhiko Murata
Shigeo Murata
Takahisa Murata
Hiroshi Murata
Takayuki Murata
Christina Muratore
Luigi Muratori
Prayag Murawala
Kelly Muraya
Yuichi Murayama
Shigeo Murayama
Kou Murayama
Alastair Murchie
Elizabeth Murchison
Pablo Murcia
Maria Antonia Murcia
Juan-Carlos Murciano
Roberto Murcio
Giuseppe Murdaca
David Murdoch
Courtney Murdock
Daniel Murdock
Vanessa Murdock
Minae Mure
M. Murea
Walter Murfee
Roberto Murgas
Nicola Murgia
Juan Murias
Delphine Muriaux
Debra Murie
Clemente Muriel Villoria
Genoveva Murillo
Eric Murillo-Rodriguez
William Murk
Andrew Murkin
B.V. Murlimanju
Chantelle Murnaghan
Silvia Muro
Manuel Muro
Yoshinao Muro
Florida Muro
Toyoaki Murohara
Sergio Murolo
Shigeyuki Murono
Susumu Muroya
Mandi Murph
Stephen Murphey
Robert Murphy
Timothy Murphy
Edward Murphy
Eileen Murphy
Gillian Murphy
Andrew Murphy
Paula Murphy
Shona Murphy
Cormac Murphy
Verena Murphy
Kevin Murphy
Gwen Murphy
George Murphy
Richard Murphy
Eain Murphy
Daniel Murphy
James Murphy
Madeline Murphy
Cristina Murphy
Bernadette Murphy
Eric Murphy
Erin Murphy
Jeffrey Murphy
Adam Murphy
Brenda Murphy
Michael Murphy

Dean Murphy
Graeme Murray
Megan Murray
James Murray
Richard Murray
Barbara Murray
Andrew Murray
Kristy Murray
Patricia Murray
Seth Murray
Alison Murray
Shauna Murray
Philip Murray
Rachael Murray
Emily Murray
Nick Murray
Lyndsay Murray
Alan Murray
Gemma Murray
Sarah Murray
Alexander G. Murray
Ben Murray
Brent Murray
Danielle Murray
Damian Murray
Laura Murray-Kolb
Colin Murray-Wallace
Colin Murrell
Ebony Murrell
Rita Murri
Mora Murri-Pierri
Fliss Murtagh
Michael Murtaugh
Muhammed Murtaza
Amy Murtha
P Murthi
Padma Murthi
Mathur Murthy
Vishnu Murthy
Sriyutha Murthy
Venkatesh Murthy
Ganti Murthy
Teemu Murtola
Brandon Murugan
Ramachandran Murugesan
Aliyu Musa
Gabriel Musante
Javed Musarrat
Marcelo Muscará
Maurizio Muscaritoli
David Muscatello
Neil Muscatiello
Mark Musch
Jorge Muschietti
Christian Muschitz
Giovanna Musco
Giovanna Muscogiuri
Carolina Muscoli
Janek Musek Musek
Eustasius Musenge
Philippe Musette
Ian Musgrave
Daniel Musher
Muhammad Mushtaq
Francesco Musiani
Erik Musiek
Linda Musil
Ismail Musirin
Gabrielle Musk
Eri Muso
Mirco Musolesi
Alessandro Mussa
Patricia Mussali
Michele Mussap
Christoph Müssel
Lytton Musselman
Kristin Musselman
Bret Musser
Margherita Mussi
Tofy Mussivand
S.M. Mussmann
Abu S Mustafa
Mohd Rais Mustafa
Devkumar Mustafi
Ananda Mustafiz
Mirna Mustapha
Mustapha Mustapha
Anne-Mari Mustonen
Angelika Mustroph
Rosario Musumeci
Maria Musumeci
Giuseppe Musumeci
Tami Musumeci-Szabo
Jude Musuuza
Andrew M. Musyoki
Artur Muszynski

Hideki Mutai
Nora Mutalima
Marko Mutanen
Onisimo Mutanga
David Mutch
Norris Muth
Zachary Muthamia
M. Muthamilarasan
Raja Mutharasan
Magesh Muthu
Veerappan Muthukkaruppan
Suresh Muthukumaraswamy
R. Muthurajan
Meenakumari Muthuramalingam
Muthuraman Muthuraman
Vivek Muthurangu
Hariharan Muthusamy
Saminathan Muthusamy
Kerim Mutig
Eugene Mutimura
Isaac Mutingwende
Apiwat Mutirangura
Haitham Mutlak
Ozal Mutlu
Tatsushi Mutoh
Daniel Mutonga
Ankur Mutreja
Chantal Mutsaers
Mousumi Mutsuddi
Raya Muttarak
George Mutter
Nico Mutters
Pavan Muttil
Gaudensia Mutua
Massy Mutumba
Ephantus Muturi
Harry Mutvei
Venkateshwar Mutyam
Rüdiger Mutz
Till S Mutzbauer
Tobias Mütze
R. Muwonge
Benoit Muylkens
Odwell Muzari
C. Muzyamba
Innocenzo Muzzalupo
Mirko Muzzi
Mulindi Mwanahamuntu
Charles Mwandawiro
Zebedee Mwandi
Benson Mwangi
Michael Mwaniki
Peter Mwitari
Mark Myatt
Alexis Mychajliw
Phillip Myer
Robert Myerburg
Chad Myers
Chris Myers
Jeffrey Myers
Janet Myers
Bronwyn Myers
Dean Myers
Valerie Myers
Candice Myers
Jim Myers
Alan Myers
Mary Myerscough
Merle Myerson
Nathan Myhrvold
Soe Myint
Ola Myklebost
Peter Myler
Paul Myles
Sean Myles
Ian Myles
V. Mylius
Hannu Myllykallio
Samy Myllymaa
Eleftherios Mylonakis
Randall Mynatt
Masako Myowa-Yamakoshi
Michael Myrick
Andriy Myronovych
Indira Mysorekar
Atle Mysterud
Nils Myszkowski
David Myung
Byoung-Kuk Na
Muzi Na
Kun Na
Guangshui Na
Muna Naash
Afia Naaz
Cristina Nabais
Patrice Nabbe

Pamela Nabeta
Shereen Nabhani-Gebara
Burt Nabors
Gert-Jan Nabuurs
Mark Naccarato
Raffael Nachbagauer
Werner Nachbauer
Parashkev Nachev
Subramanian Nachiappan
Vasanthi Nachiappan
Senthil Kumar Nachimuthu
Mark Nachtigal
Andrea Nackley
Philippe Nacry
Jean-Pierre Nadal
Marcos Nadal
Ernest Nadal
Lydie Nadal-Desbarats
Silvio Nadalin
Tibor Nadasdy
Pasqualina Naddeo
Jay Nadeau
Stephen Nadeau
S. Nadeem
M. Nadeem
Simon Nadel
Nader Nader
Ingo W. Nader
Thomas Naderer
Sobhan Naderi Parizi
Mercader Nadia
Rachel Nadif
Shivkumar Nadimpalli
Behzad Nadjm
Mangala Nadkarni
Abhijit Nadkarni
Nachiket Nadkarni
Jerry Nadler
Muhammad Naeem
Michael Naef
Hanspeter Naegeli
Shohreh Nafisi
Tapas Nag
Shalini Nag
Sathyamangla Naga Prasad
Seiho Nagafuchi
Hiroki Nagai
Yoshinori Nagai
Maria Nagai
Atsushi Nagai
Nobuhiro Nagai
Takashi Nagai
Jyothi Nagajyothi
Shushi Nagamori
Kisaburo Nagamune
Tokiko Nagamura-Inoue
Vasi Naganathan
Yukio Nagano
Seido Nagano
Atsuko Nagano-Saito
Shizuko Nagao
Kyoko Nagao
Tadahiro Nagaoka
Kentaro Nagaoka
T. Nagaoka
Bhushan Nagar
Aaron Nagar
Ramakrishnan Nagaraj
Srinivas Nagaraj
Amruthesh Kestur Nagaraj
Valakunja Nagaraja
Tavarekere N. Nagaraja
Sridevi Nagaraja
James Nagarajah
Srikantan Nagarajan
Uma Nagarajan
Rajaratinavelu Nagarajan
Rana Nagarkatti
Hideki Nagasaki
Toshiyuki Nagasawa
Kazue Nagasawa
Hiroki Nagase
Kei Nagashima
Hiroshi Nagashima
Yoji Nagashima
Hiroyuki Nagashima
Shigekazu Nagata
Shinji Nagata
Noriyo Nagata
Yosuke Nagata
Koichi Nagata
Yukihiro Nagatani
Ryoichi Nagatomi
Kazuyuki Nagatsuka
Shin Nagayama
Georg Nagel

Stefan Nagel
Bonnie Nagel
Rosemarie Nagel
Jonas Nagel
Nico Nagelkerke
Valentin Nagerl
Olivier Naggara
Ali Naghoni
Michael Nagler
Eve Nagler
Patricia Nagnan-Le Meillour
Tomohisa Nagoshi
Seema Nagpal
Sunitha Nagrath
Deepak Nagrath
Laszlo Nagy
György Nagy
Edit Nagy
Peter Nagy
Éva Nagy
Zsuzsanna Nagy
Gergely Nagy
Balint Nagy
Paul Nagy
Mate Nagy
T. Nagy
Nándor Nagy
Kyeongah Nah
George Nahas
Serafim Nahas
A. Naheed
Hareth Nahi
Rooban Nahomi
Pierre Nahon
Sergey Naidenko
David P Naidich
Saloshni Naidoo
Sanushka Naidoo
Shan Naidu
Sajo Naik
Abhijit Naik
Hironobu Naiki
David Naimark
Ashley Naimi
Isa Naina Mohamed
Harish Nair
Suresh Nair
Saidas Nair
Sreejayan Nair
Lakshmi Nair
Viswam Nair
Madhavan P. Nair
Dhana Nair
Nita Nair
Manoj Nair
Preeti Nair
Rajesh Nair
Nikhil Nair
Anilkumar Nair
Manfred Nairz
Robert Naismith
Yuji Naito
Kunihiko Naito
Yoshiro Naito
Ken Naito
Yuki Naito
Akira Naito
Hiroyoshi Naito
Bijan Najafi
Nicolas Najdovski
Mustapha Najimi
Sonia Najjar
Raymond Najjar
Hossein Najmabadi
Takashi Naka
Tetsuji Naka
Naoyuki Nakada
Susumu Nakae
Hironori Nakagami
Takayuki Nakagawa
Hidewaki Nakagawa
Tsuyoshi Nakagawa
Yoshimi Nakagawa
Atsuo Nakagawa
Osamu Nakagawa
Ichiro Nakagawa
Shigeki Nakagawa
Shinsuke Nakagawa
Kyoko Nakagawa-Goto
Akira Nakagawara
Shigeki Nakagome
Kazuyuki Nakagome
Osamu Nakagomi
Yousuke Nakai
Ryusuke Nakai
Yuji Nakai

Ayako Nakajima
Takahiro Nakajima
Hiroshi Nakajima
Mikiko Nakajima
Masanobu Nakajima
Katsuyuki Nakajima
Shunsuke Nakakura
Takashi Nakamae
Masayuki Nakamichi
Robert Nakamoto
Shingo Nakamoto
Kent Nakamoto
Keiko Nakamura
Toru Nakamura
Masahisa Nakamura
Motonobu Nakamura
Wataru Nakamura
Kazufumi Nakamura
Mary Nakamura
Hiroyuki Nakamura
Takeshi Nakamura
Yoshiki Nakamura
Yu Nakamura
Akihiro Nakamura
Michinari Nakamura
Miho Nakamura
Kensuke Nakamura
Fabio Nakamura
Kae Nakamura
Satoko Nakamura
Masato Nakamura
Kazuhiko Nakamura
Koshi Nakamura
Masatoshi Nakamura
Nobuhiro Nakamura
Shin-Ichi Nakamura
Takashi Nakamura
Tatsufumi Nakamura
Yoshimasa Nakamura
Tomomichi Nakamura
Hiroshige Nakamura
Mashio Nakamura
Hideyuki Nakane
Yoshinobu Nakanishi
Kuniaki Nakanishi
Takeo Nakanishi
Shuji Nakanishi
Hayao Nakanishi
Jun Nakanishi
Hiroaki Nakanishi
Makoto Nakanishi
Hiroyasu Nakano
Ryo Nakano
Daisuke Nakano
Masakazu Nakano
Kenji Nakano
Toshiaki Nakano
Takanari Nakano
Kiichiroh Nakano
Hideki Nakano
Eliana Nakano
Takeshi Nakano
Atsuhito Nakao
Minoru Nakao
Lia Nakao
Shinji Nakao
Kazuki Nakao
Christos Nakas
Kinichi Nakashima
Ichiro Nakashima
Kazuo Nakashima
Akio Nakashima
Isao Nakata
Yoshio Nakata
Masashi Nakatani
Masahiro Nakatochi
Hitoshi Nakatogawa
Yusuke Nakatsu
Haruaki Nakaya
Kazuhiro Nakayama
Masafumi Nakayama
Kenji Nakayama
Jiro Nakayama
Tomohiro Nakayama
Masanori Nakayama
Hideki Nakayama
Masayuki Nakayama
Shingo Nakayamada
Michiko Nakazato
Koichi Nakazato
Takefumi Nakazawa
Marcelo Nakazone
Hira Nakhasi
Carol Nakisige
Lydia Nakiyingi
Nienke Nakken

Joanna Nakonieczna
Teofil Nakov
Luigi Naldi
Aurelien Naldi
Giovanna Nalesso
Eugene Nalivaiko
Jayakrupakar Nallala
Brahmajee Nallamothu
Pratibha Nallari
Mike Nalls
Kenneth Nally
Nicolas Nalpas
Fred Nalugoda
Hawa Nalwoga
Jeong-Seok Nam
Kyoungphile Nam
Hyo Suk Nam
Jin Nam
Hyung Wook Nam
Jungho Nam
Gaewon Nam
Rami Namas
Gautham Namasivayam
Shigetou Namba
Toshiyuki Namba
Dhanya Nambiar
Dmitry Namgaladze
Ho Namkoong
Wan Namkung
Wail Nammas
Amine Namouchi
Nisana Namwat
Yongshan Nan
Xiaolin Nan
Mayank Nanavaty
Gayani Nanayakkara
Jeremy Nance
Rahul Nanchal
Vikas Nanda
Anil Nanda
Kutty Selva Nandakumar
Manu Nandan
Asoke Nandi
Ashis Nandi
Arijit Nandi
Sulakshana Nandi
Sumit Nandi
Vinay Nandicoori
Emeline Nandrot
Vijay Nandula
Dilip Nandwani
Tina Nane
Ei Ei Khaing Nang
M. Nangaku
Vinay Nangia
Keiko Nanishi
Som Nanjappa
Purushothama Nanjappa
Ian Nanjiani
Leo Nankervis
Toshihiro Nanki
Christian Nansen
Chanin Nantasenamat
Swarna Nantha
Zvi Naor
Supawadee Naorungroj
Takeyama Naoshi
Marina Naoumkina
Nadia Naous
Maria Napal
Yaakov Naparstek
Richard Napier
Adam Naples
Eleonora Napoli
Pietro Napoli
Marcelo Napoli
Francesco Napolitano
Giuliana Napolitano
Antonella Napolitano
Constanza Napolitano
Lars Napp
Armando Nappi
Solange Nappo
Nawazish Naqvi
Ravin Narain
Karthiek Narala
Vivek Naranbhai
Himanshi Narang
Steve Naranjo
Victoria Naranjo
David Naranjo
Hua Naranmandura
Sukanya Narasimhan
Balaji Narasimhan
Edward Narayan
Shalini Narayana

Sampath Narayanan
Aarthi Narayanan
Ramesh Narayanan
Manjith Narayanan
Chitra Narayanan
Divya Narayanan
Sareesh Narayanan
Raja Narayanan
Nandakumar Narayanan
Krishna Narayanan
Krishnamoorthy Narayanan
Balaji Narayanan
Mahesh Narayanan Nair
Franz Narberhaus
Michel Narce
Ed Nardell
Sheila Nardelli
Dean Nardelli
Emilio Nardi
Alessandra Nardi
Luisa Nardini
Simona Nardozza
Ann Nardulli
Kumar Narendra
Parth Narendran
Hisashi Narimatsu
Hiroaki Naritomi
Vihang Narkar
Joanna Narkiewicz
Steven Narod
Hema Narra
Kazim Narsinh
Masataka Narukawa
Kiyoshi Naruse
Veronica Narvaez-Padilla
Olga Narvskaya
Antonio Narzisi
Ana Nascimento
Eduardo Nascimento
Rafaella Nascimento
Luiz Fernando Nascimento
Andréa Nascimento
Cristiana Nascimento-Carvalho
Noman Naseer
Saleh Naser
Mahdi Naseri
Theodore Nash
David Nash
Kevin Nash
Rodney Nash
Kirsty Nash
Kelly Nash
Björn Nashan
Bjorn Nashan
Raad Nashmi
Aejaz Nasir
Arshan Nasir
Sazzad Nasir
Vahid Nasirian
Mohammad Nasiriavanaki
Radin Nasirudin
John Naslund
Joacim Näslund
Katie Nason
Rihab Nasr
Mohammed Nasr
Sharifa Nasreen
Najmunnisa Nasreen
Mohammad Hossein Nasr-Esfahani
Caitlin Nass
Giovanni Nassa
Nicolas Nassar
Antonio Paulo Nassar Junior
Dick Nässel
Christina Nassenstein
William Nasser
Mohd W Nasser
Bahman Nasseroleslami
Luigi Aurelio Nasto
Tiago Natal-Da-Luz
Natalja Natalja Fjodorova
Charles Natanson
Lisa Natanson
Raviraj Nataraj
Karaba N. Nataraja
Sathish Kumar Natarajan
Krishnamurthy Natarajan
Baker Nate
Urs Nater
Ricarda Nater-Mewes
Sankar Natesan
Utpal Nath
Pravendra Nath
Biswajit Nath
Aritro Nath
Amar Nath

Neetika Nath
Meena Nathan
Lucas Nathan
Derek Nathan
Ruvandhi Nathavitharana
Avery Nathens
M. Natic
Giovanni Natile
Ofer Nativ
Eugenia Natoli
Marvin Natowicz
Usha Natraj
Myrsini Natsopoulou
Jacob Nattermann
Barbara Natterson-Horowitz
Romain Nattier
Vaidehi Natu
Sonali Natu
Yosihiro Natuhura
Peter N. Nau
Thomas Nau
C. Nau
Michael Nauck
Richard Naud
Y. Naudé
Christopher Naugler
Gunnar Naulaers
Jean Charles Nault
Brian Nault
Eric Nauman
Ulrike Naumann
Christoph Naumann
Elena Naumova
Anna Naumova
Amarjit Naura
Fauzia Nausheen
Roya Navab
Alireza Navabi
Joaquin Navajas
Maria Navajas
Gabriel Navar
Kristen Navara
Eliano Navarese
Jordi Navarra
Michele Navarra
William Navarre
Duroy Navarre
Francisco Navarrete
Ferran Navarro
David Navarro
Xavier Navarro
Noemi Navarro
Vicente Navarro
Isabel Navarro
Fabrice Navarro
Victoria Navarro
Lucia Navarro De Lara
Adoración Navarro Torné
Fernando Navarro-Garcia
Nora Navarro-Gonzalez
Carlos Navas
Placido Navas
Maria-Cristina Navas
Ana Navas-Acien
Santiago Navas-Carretero
Jesus Navas-Castillo
Gideon Nave
Manuel Navedo
Shaik Naveed
Muhammad Naveed
Somanna Naveen
Magali Naville
Gerjan Navis
Hiroyuki Nawa
Martin Nawrot
Tim Nawrot
Roman Nawroth
Christian Nawroth
Francisco Naya
Debasis Nayak
Nihar Nayak
Jennifer Nayak
Ramesh Nayak
G.N. Nayak
Bhagabat Nayak
Dana Nayduch
Muhammad Ali Nayeem
Gavin Naylor
Claire Naylor
Richard Naylor
Scott Naysmith
Saeeda Naz
R. Naz
Ross Nazar
Ara Nazarian
Susanna Nazarian

Kianoush Nazarpour
Mona Nazeri
Kim Nazi
Mustafa Naziroglu
Filomena Nazzaro
Nicaise Ndam
Patrick Ndase
Pius Nde
Martial Ndeffo Mbah
Linus Ndegwa
Nicaise Ndembi
Chiratidzo Ndhlovu
W. Apoutou N'Djin
G. Ndrepepa
Andrada Neacsiu
Kevin Nead
Daniel Neafsey
Christopher Neal
Matthew D. Neal
James Neal
Benjamin Neal
Lori Neal
Chris Neale
Joseph Neale
Nick Neave
Carina Nebel
Andreas Nebenführ
Alex Nechiporuk
Jan Nechwatal
Anamaria Necsulea
Taku Nedachi
Mathieu Nedelec
Sophie Nedelec
Dobrin Nedelkov
Maiken Nedergaard
Jan Nedergaard
Aart Nederveen
Saharnaz Nedjat
Lee Nedkoff
David Needleman
Nicole Neef
Girish Neelakanta
Jaladhar Neelavalli
Aravind Neelavara Ananthram
Jaap Neels
Karel Neels
Sven Neelsen
Melody Neely
Kristina Neely
Mamidi Neeraja
Matthias Nees
Frauke Nees
Albrecht Neesse
Keith Neeves
Serge Nef
Tobias Nef
Kristin Neff
Roni Neff
Karl Neff
Lisa Neff
Patrick Neff
Anne Neff
Irina Neganova
Danilo Neglia
Priscilla Negraes
Daniela Negraia
Sonia Negrao
Deborah Negrão-Corrêa
Ana Negredo
Anne Negre-Salvayre
Oscar Negrete
E Negrete-Abascal
Armando Negri
Melyssa Negri
Lukas Negrin
Riccardo Negrini
Enrico Negrisolo
R Negro
Henok Negussie
Deborah Neher
Jonas Neher
Peter Neher
Eric Nehl
Ross Nehm
Z. Nehme
Volker Nehring
Matthew A Nehs
Holly Neibergs
Matthew Neidell
Eva Neidhardt
Jonathon Neidigh
Cornelia Neidlinger-Wilke
Joseph Neigel
Smart Neil
Amanda Neil
John Neill

John Neilson
Andrew Neilson
Roy Neilson
Alexander Neiman
Christoph Neinhuis
José Neira
Willie Neiswanger
Maureen Neitz
Jay Neitz
Kari Nejak-Bowen
Jens Nejstgaard
K. Nekaris
Sergei Nekhai
Deborah Neklason
Michael Nekludov
Vladimir Nekorkin
Andrt Nel
Ronel Nel
Natalie Nelissen
Jay Nelson
Martha Nelson
Glyn Nelson
Charles Nelson
Sherry Nelson
Heather Nelson
Rex Nelson
Emma Nelson
Andrew Nelson
David Nelson
Julie Nelson
Richard Nelson
Aimee Nelson
Christopher Nelson
George Nelson
Scott Nelson
Trisalyn Nelson
Eric Nelson
Deborah Nelson
Corwin Nelson
Michael Nelson
Timothy Nelson
Warrick Nelson
Candace Nelson
Nicole Nelson
Josiah Nelson
Peter Nelson
Jun Nelson
Greg Nelson
Mike Nelson
Flavia Nelson
Karin Nelson
Peggy Nelson
Kara Nelson
Houshang Nemati
M.A. Nematollahi
Lucie Nemcova
Rafik Neme
Pavel Nemec
Georges Nemer
Glen Nemerow
Peter Nemes
Elisa Nemes
Attila Nemeth
Edwin Nemoto
Tooru Nemoto
Eiji Nemoto
Igor Nenadic
Nuno Nene
Ioannis Nenekidis
Ilona Nenko
Sutapa Neogi
Charalampos Neophytou
Madhav Nepal
T. Nepolean
Pablo Nepomnaschy
Alexandre Nepomuceno
Johannes Nepp
Marco Neppi
Robert Nerem
Robert Nerenberg
Christian Neri
Bibiana Nerli
Tracy Nero
John Nerva
Clara Nervi
Bruno Nervi
Jeanne Nervina
Arthur Nery
Mariana Nery
Ragnhild Nes
Ragnhild Bang Nes
Darren Nesbeth
Sterling Nesbitt
Irina Nesmelova
Amen Ness
Kirsten Ness

J. A. Nessler
David Nestel
Steffen Nestler
Adrian Nestor
Yvonne Nestoriuc
Chris Netherton
Lauro Neto
Armando Neto
Jeniel Nett
Dirk Nettelbeck
Hans Netter
Daniel Nettersheim
Bo Netterstrøm
Paolo Netti
Thomas Netticadan
George Netto
Corey Neu
Simon Neubauer
Aljoscha Neubauer
Aljoscha Steffen Neubauer
Peter Neubauer
Arthur Neuberger
Reinhard Neubert
Marco Neubert
Gera Neufeld
Jörg Neufeld
Howard Neufeld
Hermann Neugebauer
Benjamin Neuhaeuser
Eva Neuhaus
Winfried Neuhaus
Hannelore Neuhauser
Duncan Neuhauser
Elisabeth Neuhauser
Christiane Neuhoff
Karin Neukam
Jeff Neul
Sven Neulinger
Keir Neuman
Mark Neuman
Jens Neumann
Elena Neumann
Detlef Neumann
Eric Neumann
Jessica Neumann
Dawn Neumann
Tobias Neumann
Kerstin Neumann
Anne Neumann
Frank Neumann
Yehuda Neumark
Veronique Neumeister
Christa Neumeyer
Ann Neumeyer
Joshua Neunuebel
Saraswoti Neupane
Susanne Neupert
C. Neutel
Cécile Neuvéglise
Maarit Neuvonen
Jennifer Neuwald
Edward Neuwelt
Catherine Neuwirth
Helena Nevalainen
Marja Nevalainen
Paul Neve
Joana Neves
Francisco Neves
Graça Neves
Maria Alice Neves
Leandro Neves
Veronica Neves
Valdirene Neves Monteiro
Cedric Neveu
Allan Nevill
Christian Nevill
Kathleen Neville
Helene Neville
Hannah Nevins
Yoram Nevo
Aaron New
Steve New
Ed Newbigin
Carrie Newbold
David Newburg
Sarah Newbury
Jennifer Newby
James Newcomb
George Newcombe
Benjamin Newcomer
Marie-Louise Newell
Fiona Newell
Ben Newell
Evan Newell
Robert Newell
Richard Newell

Karl Newell
Annie Newell-Fugate
Karen Newell-Litwa
Mark Newman
Debra Newman
John Newman
Christopher Newman
M. Sophia Newman
Greg Newman
Ian Newman
Roger Newman
Gale Newman
Robert Newman
Joan Newman
Michael Newman
Paula Anne Newman-Casey
Sara Newmann
Sean Newsom
Amanda Newsom
Timothy Newsome
Thomas Newsome
Jamie Newsome
Irene Newton
Ryan Newton
Tim Newton
Robert Newton
Peter Newton
Emma Newton
Jasmin Teresa Ney
Mitsuo Neya
Thomas Neyens
Samuel Neymotin
Joseph Nezgoda
Fugen Neziroglu
Yu Pong Ng
Lee-Ching Ng
Daniel Ng
Peter Ng
Simon Siu Man Ng
Tzi Bun Ng
Lai Guan Ng
Kevin Tak-Pan Ng
Victoria Ng
Kenneth Ng
Wan-Fai Ng
Terry Fei Fan Ng
Shu-Hang Ng
Sophia Ng
Kim Tien Ng
Aylwin Ng
Ting Hui Ng
Tze Siong Ng
Fu Siong Ng
Alison Ng
Wai-Leung Ng
Tat Ming Ng
Colin Ng
Lily Ng
Heok Hee Ng
Karl Ng
Hon Keung Tony Ng
Tsz Kin Ng
Manwa Ng
Jason Ng
Leo Ng
Sai-Ming Ngai
Yip Ngai-Ming
Elly Ngan
Umakanta Ngangkham
Korakot Nganvongpanit
R. Ngara
Joanne Ngeow
Oscar Ngesa
Doan Ngo
Van Ngo
Anthony Ngugi
Engelbert Mephu Nguifo
Tuck Ngun
Kenneth Ngure
Huu Phuc Nguyen
Tuan Nguyen
Hoan Nguyen
Thanh Nguyen
Quang Nguyen
Scott Nguyen
Nhu Nguyen
Nam-Trung Nguyen
Petr Nguyen
Cuong Nguyen
Nguyen Hong Nguyen
Van-Nui Nguyen
Nathalie Nguyen
Dong-Phuong Nguyen
Nam Nguyen
Thi-Phuong-Lan Nguyen
Jennifer Nguyen

Anthony Nguyen
Nam-Phuong Nguyen
Van Kinh Nguyen
Noel Nguyen
Tung Nguyen
Phu Nguyen
Bich Nguyen
Thu-Mai Nguyen
Lam-Son Nguyen
Tienhuy Nguyen
Hue Nguyen
Anthony Nguy-Robertson
Shepherd Nhamoyebonde
Joo Young Nho
Shane Nho
Rui Ni
Zhen Ni
Michael Ni
Weiming Ni
Ting Ni
Dongkui Ni
Andy Ni
Wu Ni
Liqiang Ni
Huangjing Ni
Xin Ni
Dong Ni
Hadi Nia
Hai Nian
Amir Niasari-Naslaji
Javed Niazi
Nathan Nibbelink
Ken-Ichi Nibu
Masashi Nibuya
Eimear Nic Lughadha
Katy Nicastro
Giampaolo Niccoli
Alan Nichol
Frank Nicholas
Lisa Nicholas
Richard Nicholas
Jennifer Nicholas
Robin Nicholas
John Nicholls
Stuart Nicholls
Stephen Nicholls
Kathy Nicholls
Benjamin Nichols
Colin Nichols
David Nichols
Frank Nichols
Melanie Nichols
Jeremy Nichols
Michael Nichols
Timothy Nichols
Wayne Nicholson
Garth Nicholson
Lindsay B. Nicholson
Scott Nicholson
Allen Nicholson
Jody Nicholson
Anne Nicholson-Weller
S. Nick
Annette Nicke
Bryce Nickels
Joseph Nickels
Georg Nickenig
John Nickerson
Kenneth W Nickerson
Kevin Nickerson
Dorothee Nickles
Thomas Nickl-Jockschat
Thomas Nickolas
André Nicola
Anthony Nicola
Gerry Nicolaes
Mogens Nicolaisen
Nicos Nicolaou
Anna Nicolaou
Marisa Nicolas
Sophie Nicole
Ferdinando Nicoletti
Adriana Nicoletti
Andrea Nicolini
Franck Nicolini
Giorgia Nicolini
Steven Nicoll
Andrea Nicolò
Sue Nicolson
Elisa Nicoud
Elena Nicu
Uday Nidumolu
Dawid Nidzworski
Pin Nie
Qinghua Nie
Jing Nie

Shinan Nie
Xinhua Nie
Weizhi Nie
Junsheng Nie
Feiping Nie
Da-Cheng Nie
Jingxin Nie
Song Nie
Haitao Nie
Xingju Nie
Caroline Nieberding
Mark Niebylski
Paula M. Niedenthal
Claus Niederau
Ellen Niederberger
Verena Niederberger
Steven Niederer
Florence Niedergang
Jerry Niederkorn
Thomas Niederkrotenthaler
Laura Niedernhofer
Karen Niederreither
Harald Niederstätter
Megan Niederwerder
Gerald Niedobitek
Janusz Niedojadlo
Matthias Niedrig
Inga Niedtfeld
Grzegorz Niedzwiedzki
Anke Niehof
Lee Niel
Michel Nielen
Morten Nielsen
Henrik Nielsen
Lars Nielsen
Einar Nielsen
Kristian Nielsen
Jeppe Nielsen
Jens Nielsen
Heber Nielsen
Carsten Nielsen
Kaare Nielsen
Brent Nielsen
Dennis Nielsen
Maja Nielsen
Birgitte Nielsen
Anders Nielsen
Lasse Nielsen
Jimmi Nielsen
Gunnar Lauge Nielsen
Henriette Nielsen
Vance Nielsen
Søren Nielsen
Karin Nielsen-Saines
Carrie Nielson
Anne Nielson
Dorien Nieman
Marvin Nieman
Lynnette Nieman
David Nieman
Heiner Niemann
Tilo Niemann
Barbara Niemeyer
Katarzyna Niemiec
Matthew Niemiller
Pekka Nieminen
Taina Nieminen
Angelika Niemz
Sandra Niendorf
Albert Nienhaus
William Nierman
Susan Niermeyer
Masja Nierop Groot
Dietrich Nies
Jan-Hendrik Niess
Wiro Niessen
Stijn Niessen
Christine Nießner
Alexander Niessner
Philipp Niethammer
Raquel Nieto
Francisco Nieto
Ramfis Nieto-Martínez
Alice Nieuwboer
Mark Nieuwenhuijsen
Karen Nieuwenhuijsen
Sander Nieuwenhuis
Monika Niewczas
James Nifong
Priyanka Nigam
Gordon Nigh
Norbert Nighoghossian
Kendra Nightingale
Norbert Nigoghossian
Jérôme Nigou
Mark Nigrini

Patrizia Nigro
Cecilia Nigro
Giovanna Nigro
Caetano Nigro Neto
Keishi Nihira
Takako Niikura
Yoshihito Niimura
Ülo Niinemets
Kristjan Niitepõld
Tomihisa Niitsu
Tom Nijenhuis
Rian Nijmeijer
Ron Nijs, De
Hiroshi Nikaido
Masato Nikaido
T Nikam
Takeshi Nikawa
Nik Nikbakht
Pablo Nikel
Mikko Nikinmaa
Dragana Nikitovic-Tzanakaki
Hooshang Nikjoo
Seppo Nikkari
Maryam Nikkhah
Mehdi Nikkhah
Somayeh Niknazar
Nikolas Nikolaidis
Michalis Nikolaidis
Basil Nikolau
Miroslav Nikolic
Dragana Nikolic
Mikeljon Nikolich
Nikolaos Nikolioudakis
Georgios Nikolopoulos
Zoran Nikoloski
Svetoslav Nikolov
Yulia Nikolova
Anna Nikonova
Ed Nikonowicz
Hassan Nikoueinejad
Kjell Nikus
Vani Nilakantan
Richard Niles
Kayzad Nilgiriwala
Matthew Nilles
Roy Niloptal
Nadra Nilsen
Tom Nilsen
Frank Nilsen
Peter Nilsson
Leif Nilsson
Bo Nilsson
Maria Nilsson
Lars Nilsson
Mats Nilsson
Emil Nilsson
Erik Nilsson
Louise Nilsson
Flemming Nilsson
Ashish Nimbarte
Kumar Nimit
Shahid Nimjee
Vamshi Nimmagadda
Graeme Nimmo
Leonardo Nimrichter
Ipe Ninan
Kang Ning
Baitang Ning
Tangyuan Ning
Chuanyi Ning
Tang-Yuan Ning
Zhiwei Ning
Daliang Ning
Shunbin Ning
Xia Ning
Jianchang Ning
Marisa Ninivaggi
Natalia Ninkina
Diego Nino
Elina Nino
John Niparko
David Nipperess
Jesse Nippert
B. Nirmala
Ran Nir-Paz
S. Niru Nirthanan
R. Ellen Nisbet
Richard E. Nisbett
Hans Dieter Nischalke
Sandra Nischwitz
Vicki Nisenblat
Akinori Nishi
Hiroshi Nishi
Daisuke Nishi
Toshiya Nishibe
Gohei Nishibuchi

Kunihiro Nishida
Atsushi Nishida
Ikuo Nishigaki
Koji Nishiguchi
Akihiro Nishiguchi
Masahiro Nishihara
Tatsuji Nishihara
Muneko Nishijo
Hiroki Nishikawa
Kanto Nishikawa
Haruka Nishikawa
Nobuyuki Nishikiori
Hiroshi Nishimaru
Stephen Nishimura
Ichiro Nishimura
Satoshi Nishimura
Wataru Nishimura
Reiki Nishimura
Hidekazu Nishimura
Takashi Nishina
Toru Nishinaka
Ichizo Nishino
Kunihiko Nishino
Seiji Nishino
Mizuki Nishino
Kazuto Nishio
Mizuho Nishio
Yasuhiko Nishioka
Hideki Nishitoh
Akiko Nishiwaki
Akira Nishiyama
Chiharu Nishiyama
Yusuke Nishiyama
Mari Nishizaka
Yoko Nishizawa
Corey Nislow
Guiomar Niso
Tracy Nissan
Noam Nissan
Mogens Nissen
Robert Nissen
Mikko Nissi
Itzhak Nissim
Anne Nissinen
Ravi Nistala
Harikiran Nistala
Estanislao Nistal-Villán
Ionut Nistor
Jun-Ichi Nitadori
Aleksandra Nita-Lazar
Narong Nitatpattana
Marloes Nitert
Jess Nithianantharajah
M.A. Nitsche
D. Patric Nitsche-Schmitz
Atsumi Nitta
Soniya Nityanand
Vivek Nityananda
Anjana Nityanandam
Bjoern Nitzsche
Anika Nitzsche
Marie Nitzschner
Deng-Ke Niu
Tianhua Niu
Yan Yang Niu
Gang Niu
Shi-Hui Niu
Jinzhi Niu
Xuyan Niu
Zhiyv Niu
H.S. Niu
Junqi Niu
Junfeng Niu
Suyan Niu
Wenxin Niu
Masha Niv
Hitoshi Niwa
Shimpei Niwa
Allan Nix
U. Nixdorff
Brett Nixon
Jessie Nixon
Sara Jo Nixon
Mark Nixon
Christopher Niyibizi
François Niyonsaba
Vandana Niyyar
Dean Nizetic
Dean Nizetic
Margaret Niznikiewicz
Antonio Nizza
Joseph Njau
M. Njenga
Sisse Njor
Bongani B Nkambule
John Nkengasong

Kabwe Nkongolo
Hermenegilde Nkurunziza
Jerome Noailly
Jennie Noakes
Angela Nobbs
Andrew Nobel
Clarissa Nobile
Luke Noble
Peter Noble
Mary Mar Noblezada
Kosuke Noborio
Vandack Nobre
R. Nobre
Giuseppe Nocella
Giuseppe Nocentini
Michela Nocetti
Kotaro Nochioka
Charles Nock
Masaki Noda
Mami Noda
Takashi Noda
Takeshi Noda
Takuji Noda
Nobuo Noda
Naonobu Noda
Koichi Node
Hiroyuki Nodera
Sabrina Noel
Pierre Noel
Danièle Noël
Peter Noël
Heidi Noels
Alvarez Nogal
Masayuki Noguchi
Eishi Noguchi
Takuya Noguchi
Norihisa Noguchi
Marcelo Nogueira
Cristiano Nogueira
Anselmo Nogueira
Regina Nogueira
Xesus Nogueira
Waldo Nogueira
Susan Noh
Katharina Nöh
Takehiko Nohmi
Josselin Noirel
Eisei Noiri
Masanori Nojima
Ippei Nojima
Takashi Nojiri
Marzieh Nojomi
Erica Nol
Christopher Nolan
Vikki Nolan
John Nolan
Michael Nolan
Jessica Nolan
Robert Noland
Gregory Noland
Maria Nolano
Dirk Nolf
Stefano Nolfi
Thomas Nolin
Fernando Noll
Marcelo Nöllmann
Dagmar Nolte
Arne Nolte
Guido Nolte
Jeffrey Nolz
K. Noma
Kensuke Noma
Cristina Nombela
Manuel Nombela
Jason Nomi
Kyriaki Nomikou
Nikolitsa Nomikou
Yves Nominé
T. Nomiya
Takashi Nomiyama
Christopher Nomura
A. Nomura
Amy Non
Peter Nonacs
Michael Nonet
Yibing Nong
Maria Noni
Alberto Nonis
Lourens Nonkes
Larisa Nonn
Michael Nonnemacher
Dan Nonneman
Ken-Ichi Nonomura
Michael Noonan
Douglas Noonan
Chadanat Noonin

Mohamed Noor
Abdisalan Noor
Rashed Noor
Mehwish Noor
Meine Van Noordwijk
Ursula Nopp-Mayr
Khairun Nor Aripin
Khairun Nain Nor Aripin
Gray Nora
Nathalie Norais
Dan Norback
Andreas Nord
Lars Nordenmark
Lars Nordenskiöld
Merete Nordentoft
Antoine Nordez
Verena Nordhoff
Jessica Nordlund
Robert Nordon
Rebecca Nordquist
Anne Nordrehaug
Kerstin N. Nordstrom
Carlos Nordt
D. Noreika
John Norelli
Karin Noren
Javier Nori
Fernando Noriega
Tommy Norin
Marina Noris
Torsten Norlander
Greg J. Norman
Beth Norman
Sharon-Lise Normand
Sébastien Normand
Nicola Normanno
Benjamin Normark
Takayuki Noro
Peter Norquest
Rikke Norregaard
Dennis Norris
David Norris
Karen Norris
Joel Norris
Abigail Norris Turner
Ivan Norscia
Thor Norstrom
Grant Norte
Carol North
Rachel North
Michael North
Hope Northrup
Joseph Northrup
J.L. Nortier
Raymond Norton
Samuel Norton
Gareth Norton
Lloyd Norton
Elizabeth Norton
William Norton
Robert Norwood
Andy Nosal
Joshua Nosanchuk
Chiara Nosarti
Michael Noseworthy
Hirokazu Noshiro
Katsuhiko Nosho
Sergei Noskov
Andrew Noss
Ralph Nossal
Hans Nossent
Marika Nosten-Bertrand
Janina Noster
Michitaka Notaguchi
Giuseppe Notarbartolo Di Sciara
Silvio Notari
Lies Notebaert
Wim Notebaert
Jennifer Noto
Caroline Nott
Ad Notten
Leif Nøttestad
Stephane Nottin
Angela Noufaily
Antoine Nougairede
Jean-Philippe Nougayrède
Anastasios Noulas
Aria Nouri
Pierre Nouvellet
Antonio Nouvenne
Régis Nouvian
Yuval Nov
Deborah Novack
Victor Novack
Ângela Novais
Renato Novais

Richard Novak
Vera Novak
Ivana Novak
Donald Novak
Gordon Novak
Elizabeth Novak
Paige Novak
Lenka Nováková
Eva Nováková
Antonio Novelli
Giacomo Novembre
Noa Novershtern
Daniela Novick
Olga Novikova
Len Novilla
Marko Novinec
Sergey Novitskiy
Beatriz Novoa
Adam Novobilský
Valentyn Novosad
Elena Novoselova
Vojtech Novotny
Rachel Novotny
Artem Novozhilov
Shane Nowack
Marc Nowaczyk
Thaddeus Nowak
Michael Nowak
Albina Nowak
Sabina Nowak
Grazyna Nowak
Markus Nowak
Patrycja Nowak-Sliwinska
Daniela Nowara
Kristen Nowell
Katja Nowick
Katarzyna Nowicka-Sauer
Bogdan Nowicki
Stella Nowicki
Norbert Nowotny
Bettina Nowotny
Ali Nowrouzi
Ariela Noy
Ilan Noy
Belkisyole Noya
Noelle Noyes
Tomoyoshi Nozaki
Hisayoshi Nozaki
H. Nozaki
Sylvie Nozaradan
Yoko Nozawa
Susan Nozell
Koizumi Nozomu
Tomoko Nozoye
Kandai Nozu
T. Nozu
Christian Nsanzabana
Georgia Ntani
Francis Ntumngia
Silke Nuber
Mariana Nucci
Paolo Nucci
Richard Nuccitelli
Gaetano Nucifora
Eric Nudleman
Eric Nuermberger
Timothy Nugent
Nicole Nugent
Scott Nugent
Maggy Nugues
Hitoshi Nukada
Nobuyuki Nukina
Clair Null
Shusuke Numata
Izaya Numata
Sirpa Nummela
José Pedro Nunes
Marta Nunes
Jacques Nunes
Francis Nunes
Vania Nunes
Altacílio Nunes
Kevin Nunes
Maria Nunes
Fernanda Nunes
Rita Nunes
Adriano Nunes-Nesi
Paul L. Nunez
Kenia Nunez
Sylvia Nunez
Angel Nuñez
Julio Nuñez
Jose Nuñez
Vitelbina Nuñez
Megan E. Núñez
Laure Nuninger

Brook Nunn
A. Nunn
Leonard Nunney
Juan Carlos Nuño
Hiroyuki Nunome
Nadav Nur
Alan Nurden
Osamu Nureki
Roza Nurieva
Michael Nurmohamed
Gertrud Nürnberg
Yuana Nurulita
Heather Nuske
Charlotte Nusman
Maury Nussbaum
Thomas Nussbaumer
Ruth Nussinov
Andreas Nüssler
Sabina Nuti
Stephen Nutt
John Nutt
Leta Nutt
Patricia Nuttall
Scott Nutter
Jari Nuutila
Iman Nuwayhid
Samer Nuwwareh
Anne Monique Nuyt
Sandra Nuyts
Cally Nwosu
Farai Nyabadza
Julius Nyalwidhe
Árpád Nyári
Mukesh Nyati
Fred Nyberg
Scott Nyberg
Laura Nyblade
Lars Nybo
Hilde Nybom
Verity Nye
Tyler Nygaard
Brendan Nyhan
Philip Nyhus
Ildiko Nyilasi
Miklos Nyitrai
Alan Nyitray
László Nyitray
Hannu Nykänen
Leena Nylander-French
Susanne Nylen
Tuula Nyman
Jeffry Nyman
Andy Nyman
Alinane Nyondo
Paul Nyquist
Johanna Nystedt
Thomas Nystrom
Laura Nyström
Myaing Nyunt
Jacinta Nzinga
Mary O' Brien
Michael O' Connor
Stephen O' Neill
Aifric O Sullivan
Beth O'Brien
Oliver O'Brien
Richard O'Connell
Ross O'Hara
Daniel O'Leary
Manoj Oak
Jessica Oakes
Patrick Oakes
John Oakeshott
Jane Oakey
Berl Oakley
Todd Oakley
Brian Oakley
Andrew Oates
Rd Oates
Jon Oatley
Keith Oatley
Takashi Obama
Akira Obana
M. Kerry O'Banion
Joshua Obar
Ilona Obara
Taku Obara
Sueli Oba-Shinjo
Toshihiro Obata
Darren Obbard
Glen Obear
Lina Obeid
Rima Obeid
Omar Obeid
Dietrich Ober
Assad Oberai

Daniel Oberfeld
Ann Oberhauser
Walter Oberhuber
Hannes Oberkofler
Volker Oberle
Nicholas Oberlies
Brandon Oberlin
Barbara Obermayer-Pietsch
Ernst Oberortner
Andreas Obersteiner
Zuzana Obertova
Timm Oberwahrenbrock
Ignacio Obeso
Yoshitsugu Obi
Walter Obiero
Satoshi Obika
Martin Obin
Elia Obis
Loraine Obler
Paula Oblessuc
James Obol
Uri Obolski
Ernest O'Boyle
Walter Obregon
Aleksandra Obrepalska-Steplowska
John O'Brien
Tim O'Brien
Timothy O'Brien
Charles O'Brien
Michael O'Brien
Louise O'Brien
Sarah O'Brien
Katherine O'Brien
Meagan O'Brien
Emily O'Brien
Kristin O'Brien
Oonagh O'Brien
Heath O'Brien
Terrence O'Brien
Katie O'Brien
Catherine O'Brien
Maureen O'Brien
Neil O'Brien-Simpson
Tom Obrig
Martin Obrist
Moira O'Bryan
John Obrycki
Martin Obschonka
Ingrid Obsuth
Ognjen Obucina
Ekwaro Obuku
David Obura
David O'Callaghan
Paolo Ocampo
Simon Ocarroll
Giovanni Occhipinti
Jeremi Ochab
Oksana Ocheretina
Takumi Ochiai
Josiah Ochieng
Begoña Ochoa
Maria Teresa Ochoa
Jesus Ochoa
Francisco Ochoa-Corona
Javier Ochoa-Reparaz
Eleanor Ochodo
Ben Ockert
Boyo Ockinga
Sebastian Ocklenburg
Redmond O'Connell
Jeffrey O'Connell
Mary O'Connell
Lauren O'Connell
Grace O'Connell
Kristen O'Connell
Timothy O'Connell
Craig O'Connell
Mary O'Connell Motherway
Patrick Oconnor
Helen Oconnor
David O'Connor
Patrick O'Connor
Timothy O'Connor
James O'Connor
Vincent O'Connor
John O'Connor
Kathleen O'Connor
Jingmai O'Connor
Kevin O'Connor
Mairead O'Connor
Teresia O'Connor
Roberta O'Connor
Christine O'Connor
Christopher O'Connor
Catherine O'Connor
Tracey O'Connor

Alexander O'Connor
Daniel O'Connor
Barry O'Connor
Peter O'Connor
Sophie Octavia
Helena Oczak-Woltman
Elizabeth Oczypok
Shinya Oda
Kenji Oda
Masato Oda
Ryo Oda
Tom Oda
Yuko Odagiri
Kenichi Odaka
Patrick Odawo
John Odden
Michelle Odden
Mauro Oddo
Paul Ode
Shirley O'Dea
Luke Odell
Margarete Odenthal
Alex Odermatt
Maria Odero
Shmuel Odes
Patrizio Odetti
Paul Odgren
Emmanuel Odic
Elena Odintsova
Dennis Odion
Jack Odle
Mark O'Doherty
Silvia Odolini
Stephen Odom
Max O'Donnell
Sean O'Donnell
Brian O'Donnell
Kerrie O'Donnell
Kieran O'Donnell
Ae O'Donnell
Amy Odum
Ayub Oduor
Cornelia Oedekoven
Peter Oefner
Vivian Oehler
Bettina Oehrle
Ju Lee Oei
Anika Oellrich
Viktoria Oelze
Christopher Oermann
Christoph Oesterreicher
William Oetting
Orit Oettinger-Barak
Konrad Oexle
Robert Ofenloch
Steven Offenbacher
Dietmar Offenhuber
P. Offerhaus
Bernard Offmann
Martin O'Flaherty
Cristian O'Flaherty
Michael Ofori
Opokua Ofori-Anyinam
Anthony Ofosu
Okechukwu Ogah
Joe Ogas
Toru Ogasawara
Michihiro Ogasawara
Haruhiko Ogasawara
Nahoko Ogata
Hiroaki Ogata
Toshiyasu Ogata
Seishi Ogawa
Emiko Ogawa
Yoshiko Ogawa
Takehiko Ogawa
Aiko Ogawa
Kelechi Ogbuehi
Matthew Ogburn
Betsy Ogburn
Rob Ogden
Nicholas Ogden
Javier Ogembo
Marco Oggioni
Steve Oghumu
Eric Ogier-Denis
Yuji Ogihara
Peter Ogilby
Akiyoshi Ogimoto
Shuji Ogino
Yasushi Oginosawa
S. Ogiso
Amanda Oglesby-Sherrouse
Mylène Ogliastro
Magdalena Ogluszka
Haluk Ögmen

Takeshi Ogo
Donal O'Gorman
David O'Gorman
José Oguiza
Babatunde Ogunbosi
Abiodun Ogunniyi
Olorunseun Ogunwobi
Yuji Ogura
Masatsune Ogura
Sayoko Ogura
Sae-Ock Oh
Seung-Ha Oh
Jong-Won Oh
Goo Taeg Oh
Deok-Kun Oh
Chang Sik Oh
S. Paul Oh
Heung-Bum Oh
Ding Yuan Oh
Jung Mi Oh
Daniel Oh
Myung-Min Oh
Duck-Won Oh
Il-Hoan Oh
Young Taik Oh
Seungdae Oh
Dong-Ha Oh
Jeong-Eun Oh
Edwin Oh
Sang-Yun Oh
Sung Oh Hwang
Justin O'Hagan
Damien O'Halloran
Naoya Ohara
Yuki Ohara
Bruce O'Hara
Timothy O'Hara
Bob O'Hara
Tom O'Hara
Patrick O'Hara
Joseph O'Hare
Louise O'Hare
Brain O'Hartaigh
Jun Ohashi
Koji Ohashi
Shinya Ohashi
Kenji Ohba
Kirsten O'Hearn
Robert Ohgami
Kazutaka Ohi
Masahiro Ohira
Mitsuru Ohishi
Yoshiyuki Ohkawa
Yasuyuki Ohkawa
Naohiko Ohkouchi
Takayoshi Ohkubo
Siew-Wan Ohl
Johan Ohlander
Kevin Ohlemiller
Annemarie Ohler
Carsten Ohlmann
Knut Ohlsen
Joyce Ohm
Jay Ohm
Dennis Ohman
Nobuko Ohmido
Akemi Ohmiya
Tsukasa Ohmori
Makoto Ohmoto
Masaki Ohmuraya
Takbum Ohn
Thomas Ohnesorg
Kouhei Ohnishi
Takeo Ohnishi
Kinji Ohno
Seiko Ohno
Michiya Ohno
Haruya Ohno
Paul Ohori
John O'Horo
Richard Ohrbach
Nitin Ohri
Yusei Ohshima
Issei Ohshima
Satoru Ohshima
Shigeo Ohta
Kumimasa Ohta
Aaron Ohta
Takeshi Ohta
Ryo Ohta
Akihide Ohta
Takayasu Ohtake
Naoto Ohtani
Misato Ohtani
Nobuyuki Ohte
Chikara Ohtsuki

Colm O'Huigin
Eric Ohuma
Yusuke Ohya
Pål Øian
Birgit Oidtmann
Hideaki Oike
Naoki Oishi
Kazunori Oishi
John Ojal
Miina Ojansivu
Henn Ojaveer
Jenifer Ojeda
Nkemcho Ojeh
Anil Ojha
Opeolu Ojo
Edward Ojuka
Masahiko Oka
Kyoko Oka
Koichiro Oka
Shinichi Oka
Yukinori Okada
Shinji Okada
Masayoshi Okada
Takashi Okada
Yuki Okada
Kazunori Okada
Annabelle Okada
Motohiro Okada
Tomo Okada
Miki Okada
Kazunari Okada
Hitoshi Okada
Morihito Okada
Hiroaki Okada
Maria-Theresa Okafor
Chika Okafor
Fumikazu Okajima
Hidehiko Okamoto
Kenichi Okamoto
Keith Okamoto
Kinya Okamoto
Maristela Okamoto
Yoshiharu Okamoto
Masato Okamoto
Toru Okamoto
Daniel Okamoto
K. Okamoto-Mizuno
Noboru Okamura
Katsutomo Okamura
Beth Okamura
Tsuyoshi Okamura
Orkan Okan
Hideyuki Okano
Mitsuhiro Okano
Akihiro Okano
Kamolnetr Okanurak
Sezer Okay
Ryuichi Okayasu
Yozo Okazaki
Shin Okazaki
Tobi Oke
Richard O'Kearney
F. Robin O'Keefe
Andy O'Keefe
Grant O'Keefe
Stephen O'Keefe
Denise O'Keefe
Edward Okeke
Samson Okello
Edward Okello
Fuad Oken
Leah Okenwa
Chioma Okeoma
Masaya Oki
Yasuhiro Oki
Shinya Oki
Takashi Okiji
Noriaki Okimoto
Peter Okin
Clarissa Hamaio Okino-Delgado
Atsutaka Okizaki
Klaus Okkenhaug
Iheanyi Okonko
Ogugua Okonkwo
Ikechi Okpechi
Evaezi Okpokoro
Allan Okrainec
Niels Oksbjerg
Ole Andreas Økstad
Ayse Oktay
Ozgur Oktem
Patricia Okubara
Takayoshi Okubo
Yoshiro Okubo
Takashi Okubo
Koji Okudela

Yoshinaga Okugawa
Mobolaji Okulate
Monika Okuliarova
Yasuyuki Okuma
Fredros Okumu
Kenji Okumura
Toshikatsu Okumura
Yasuyuki Okumura
Cheryl Okumura
Ken Okumura
Naoki Okumura
Michael Okun
Alec Okun
Paul Okunieff
Takafumi Okura
Mitsuharu Okutsu
Julianna Oláh
Íñigo Olalde
Hammed Olanrewaju
Ola Olapade
Eo Olapade-Olaopa
Victor Olariu
Mariefel Olarte
Ioana Olaru
Sergio Olate
Sebastian Olbrich
Natasha Olby
Monica Olcina
Guillaume Oldenhove
Karl Oldhafer
Mark Oldham
Carolyn Oldham
Ben Oldroyd
Brett Olds
Pedro Olea
Francisco Olea-Popelka
Caroline O'Leary
Stephen O'Leary
Nataly O'Leary
Mónica Oleastro
Jason Olejarz
Andrzej Oleksa
Ola Olen
Barbara Olendzki
Emma Olesen
Kirsten Oleson
Philipp Olias
Jose Olijnyk
Jill Olin
Tom Olino
Nicholas Oliphant
Francesco Oliva
Jonàs Oliva
Kevin Olival
Alberto Olivares
Manuel Olivares Grohnert
Rene Olivares-Navarrete
Aires Oliva-Teles
Pedro Oliveira
Fabiano Oliveira
Marcus Oliveira
Maria Leonor Sarno Oliveira
Carla Oliveira
Claudio Oliveira
Vinicius Oliveira
Sandra Oliveira
Paulo Oliveira
Maria Oliveira
Hugo Oliveira
Ubirajara Oliveira
Marcos Oliveira
Fernando Luiz Pereira De Oliveira
Halley Oliveira
André Oliveira
Miguel Oliveira
Clara Oliveira
Jta Oliveira
Ernna Oliveira
Gisele Oliveira
Cristieli Oliveira
Jorge Oliveira
Jhones Oliveira
Helinando Oliveira
Maria Oliveira Souza
João Luís Oliveira-Carvalho
Antonio Oliver
Francisco Oliver
Jeffrey Oliver
Brian Oliver
Richard Oliver
Donald Oliver
Paula Oliver
Jonathan Oliver
Gavin Oliver
Bonamy Oliver
Rema Oliver

Haley Oliver
Steven Oliver
Kathryn Oliver
Arnau Oliver
William Oliver
Verity Oliver
Carina Olivera
Alvaro Olivera-Nappa
Matteo Oliverio
Paola Olivero
Jesus Olivero Anarte
Eleonora Olivetta
Martin Olivier
Christoph Olivier
Magali Olivier
Abraham Olivier
Jocelien Olivier
Anna Olivieri
Oliviero Olivieri
Alejandro Olivieri
Antonio Oliviero
Bettina Olk
Satu Olkkola
Vesa Olkkonen
Martta Olkkonen
Henri Olkoniemi
Miina Ollikainen
Juliane Ollinger
David Ollis
Cheryl Olman
Ettore Olmo
Roberta Olmo Pinheiro
Gemma Olmos
Andrea Olmstead
Jack Olney
Erik Olofsen
Jonas Olofsson
Mana Oloomi
Jennifer Olori
Samantha O'Loughlin
Andrea Olschewski
Horst Olschewski
Sonja Olsen
David Olsen
Jorn Olsen
Steven C. Olsen
Thale Kristin Olsen
Catharina Olsen
Ole Olsen
Rikke Olsen
Robert Olsen
Mark Olsen
Flemming Olsen
Brian Olshansky
Richard Olshen
Ann Olson
Link Olson
Jb Olson
Deanna Olson
Matthew Olson
Timothy Olson
Michael Olson
Donald Olson
Robert Olson
Jonathan Olson
Zachary Olson
David Olson
Åke Olson
Erik Olson
Mats Olsson
Peter Olsson
Håkan Olsson
Magnus Olsson
Michal Olszewski
Deborah Olszewski
Krzysztof Olszynski
James Oltjen
Mette Olufsen
Fawole Olufunmilayo
Bode Olukolu
Oluyemi Olumolade
Olufemi Olumuyiwa Desalu
Oluyinka Olutoye
Christine Olver
Ian Olver
Heidi Olze
Claudia Omachi
Siobhain O'Mahony
Ronan O'Malley
Grace O'Malley
Robert O'Malley
Michelle O'Malley
Khairuddin Omar
Megan O'Mara
Keliana O'Mara
Perera Omaththage

Larsson Omberg
Stefano Omboni
Michael Ombrello
Denis O'Meally
Gilbert Omenn
Jean Ometto
James Omichinski
Arash Omidi
Reza Omidi Varmezani
Elisa Omodei
Samuel Omokhodion
Emmanuel Omondi
Miyuki Omori-Miyake
Olayemi Omotade
Hiroshi Omote
Celso Omoto
Ebun Omoyinmi
Anders Omsland
Colm O'Muircheartaigh
Elizabeth Omukunda
Olusegun Onabajo
Mark Onaitis
Adebola Onanuga
Altan Onat
Frankline Onchiri
Lucas Onder
Pascale Ondoa
Brian Ondov
Elen Oneal
Matt O'Neal
Serena O'Neil
Shawn O'Neil
Rachel O'Neill
Geraldine O'Neill
Joseph O'Neill
Charles O'Neill
Malcolm O'Neill
Liam O'Neill
Maria Giuseppina Onesti
Albert Ong
Peck Ong
Jason Ong
Loke Ong
Zhi Ong
Eugenia Ong
Lee-Ling Ong
S. Tiong Ong
John Ong'Echa
Greg Ongie
Ennio Ongini
Masayuki Onishi
Michael Onken
Itay Onn
Jukka-Pekka Onnela
Tetsuya Ono
Koh Ono
Yusuke Ono
Masahiro Ono
Takahiro Ono
Satoshi Onoda
Hiroaki Onoe
John Onofrey
Andrea Onofri
Paolo Onori
Ikushi Onozaki
Daisuke Onozuka
Mark Onslow
Renske Onstein
Luiz Onuchic
Monica Onyango
Isaac Onyango
Ye Oo
Eng Eong Ooi
Joshua Ooi
Keith Ooi
Mark Ooi
Kenneth Ooi
Cees Oomens
Antonius Oomens
Marcel Ooms
Youko Oono
Chris Oostenbrink
Rianne Oostenbrink
Robert Oostendorp
Gerard Oostermeijer
Rony Oosterom-Calo
Tony Oosterveen
Mariska Oosterveld-Vlug
Mirjam Oosterwerff
Youichirou Ootsuka
Michiel Op De Beeck
Vera Opatova
Juan Opazo
Anders Opdal
Ghislain Opdenakker
Stanley Opella

John Opfer
Ron Ophir
Bastian Opitz
Bertram Opitz
Christiane Opitz
Philipp Opitz
Christian Opländer
Steffen Oppel
Federico Oppenheimer
Mark Oppenlander
Charles Opperman
Brenda Oppert
Pablo Oppezzo
Raymond Oppong
Tanja Opriessnig
Emanuelle Opsommer
Trine Opstad
Katrijn Opstoel
Lance Optican
Michael O'Quinn
Charles C.F. Or
Yizhar Or
Hiroki Ora
Tamer Oraby
Federica Oradini
Berk Orakcioglu
Elif Oral
Mike Oram
Babak Orandi
J.B. Orange
Zita Oravecz
Peter Orazem
Marc Orbach
Dara Orbach
Jean-Christophe Orban
Levente Orban
Sheina Orbell
Sandra Orchard
Paul Orchard
Mariano Ordano
Jose Orden
Jaume Ordi
Francesc Ordines
Celestino Ordonez
Alejandro Ordonez
Pedro Ordunez
Stefania Orecchioni
Richard Oreffo
Ruth O'Regan
Kathleen O'Reilly
Michael O'Reilly
Paul O'Reilly
Francisco Orejuela
Elena Orekhova
Pablo Orellano
Mark Oremus
Michael Orendurff
W. Orenstein
Stylianos Orfanos
Alberto Orfao
Jason Organ
Jose Orgaz
Michael Orger
Vasiliki Orgeta
Prisca Oria
Jacqueline Orian
Véronique Orian-Rousseau
Eniyou Oriero
Francesco Origgi
Kanami Orihara
Carlos Orihuela
Pedro Orihuela
Adebola Orimadegun
Koichi Orino
Silvia Orisio
Kenji Oritani
Jérome Orivel
Claudio Orizio
Pamela Orjuela-Sánchez
Augusto Orlandi
Rosaria Orlandi
Andres Orlandini
Giuseppe Orlando
Mohammed Orloff
Sergei Orlov
Yuriy Orlov
Elzbieta Orlowska
Mehmet Orman
David Ormandy
Ian Orme
Elena Ormeño
Kate Ormerod
Tom Ormorod
Mihály Ormos
Christopher Ormsby
Juan Francisco Ornelas

Claudia Patricia Ornelas García
Fernanda Ornellas
Uri Oron
Daniel Oros
José A. Orosa
Dennis O'Rourke
Peter O'Rourke
Gisela Orozco
Aurea Orozco
Esther Orozco
Alan Orpin
Bernardo Orr
Joseph Orr
Teri Orr
Catherine Orrel
Christina Orru
Luigi Orrù
Stefania Orrù
Anna-Leena Orsama
Simone Orsenigo
La'Or Orshan
Angela Orshinsky
William Orsi
Mario Orsi
Francesco Orsini
Frank Orson
Manuel Luis Orta
Hugo Ortega
Felipe Ortega
Victor Enrique Ortega
Enrique Ortega
Francisco J Ortega
Ruben Ortega-Alvarez
Javier Ortego
Joaquin Ortego
Norberto Ortego-Centeno
Per Örtenwall
Anthony Orth
Kim Orth
Michael Orth
Juncal Orth
Guillermo Orti
Tania Ortiga-Carvalho
Pavel Ortinski
Juan Carlos Ortiz
Rodomiro Ortiz
Marco Ortiz
Jorge Ortiz
Carolina Ortiz
J. Bryce Ortiz
Abigail Ortiz
Julio Ortiz Canseco
Dario Ortiz De Orue Lucana
Irene Ortolani
Christian Ortolf
David Orton
Frances Orton
Elena Ortona
Anne Ortqvist
Francesco Ortuso
Francis Orvain
Raoul Orvieto
David Orwig
Paul Orwin
Virginie Ory
Marek Orzechowski
Naoki Osada
Kazumi Osada
Hiroyuki Osada
Shigehiro Osada
Tadeusz Osadnik
Remus Osan
Motomi Osato
Tsuyoshi Osawa
Hiroyuki Osawa
Takahiro Osawa
Nosayaba Osazuwa-Peters
Olivia Osborn
Megan Osborne
James Osborne
Danny Osborne
Amy Osborne
José María Osca-Lluch
Jan Oscarsson
Jon Oscherwitz
Isaac Osei
Douglas Osei-Hyiaman
Patrick Osei-Owusu
Mohammad Oshaghi
Peter O'Shaughnessy
K Sue O'Shea
Nir Osherov
Junko Oshima
Tadayuki Oshima
T Oshima
Yusuke Oshima

Yoko Oshima-Franco
Mitsuo Oshimura
John Oshinski
Atsushi Oshio
Hitoshi Oshitani
Faith Osier
Artur Osikowski
Evgeny Osin
Carla Osiowy
Michael Osland
Mary Osley
Roman Osman
Mahasin Osman
A Osman
Abdimajid Osman
Stephen Osmani
Todd Osmundson
Natalia Osna
Mehdi Osooli
Daniel Osorio
Fernando Osório
Flávia Osório
Alfred Osoti
Caroline Ospelt
Marco Ospelt
Maria Ospina
Andrés Ospina-Alvarez
Gustavo Ospina-Tascón
Peter Ossenkopp
Krystyna Ossowska
Lars-Goran Ost
Michael Ostacher
Valeriy Ostapchenko
Teresa Ostaszewska
Anna-Lena Ostberg
Tammo Ostendorf
Henrik Oster
Alexandra Oster
Harriet Oster
Kris Osterberg
Leif Østergaard
Jonas Osterloff
Marlies Ostermann
Marc Ostermeier
Katherine Osteryoung
Katherine Ostevik
Adam Osth
Michael Osthoff
Max Ostinelli
Bjørn Østman
Julia Ostner
Elena Osto
Sergej Ostojic
Ivika Ostonen
Erin Ostrem
Harry Ostrer
Kostya (Ken) Ostrikov
Lisa Ostrin
Rachel Ostroff
Luis Ostrosky-Zeichner
Yan Ostrovski
Olga Ostrovsky
Sisse Ostrowski
Dirk Ostwald
Marcin Osuchowski
Daniel O'Sullivan
Justin O'Sullivan
Orla O'Sullivan
Matthew O'Sullivan
Robert Oswald
Fred Oswald
Motonori Ota
Erika Ota
Miho Ota
Shuhei Ota
Tsuguhito Ota
Shingo Ota
Yuko Ota
Kenneth Otabil
Joji Otaki
Niels Otani
Hajime Otani
Juan Oteiza
Jose Oteo
Rafael Otero
Carol Otey
Rozana Othman
Sreekumar Othumpangat
Martin Oti
Perrie O'Tierney-Ginn
Timo Otonkoski
John O'Toole
Mai Ots-Rosenberg
Eigo Otsuji
Motoyuki Otsuka
Atsushi Otsuka

Toshiaki Otsuka
Yasumasa Otsuka
Takemi Otsuki
Michael Ott
Jorgelina Ottado
Cristina Ottaviani
Jan-Michel Otte
Karen Ottemann
Hans Martin Otten
Coen Ottenheijm
Andreas Otterbeck
Leo Otterbein
Ronald Otterstetter
Elizabeth Ann Ottesen
Deborah Otteson
Penelope Ottewell
Oliver Otti
Eleonora Ottina
Christian Ottmann
Michael Otto
Clint Otto
Gordon Otto
Caitlin Otto
Giovanni Ottoboni
Claudio Ottoni
Easmon Otupiri
Daniel Otzen
Hong-Yu Ou
Henry Ou
Jing-Song Ou
Xiaokun Ou
Ming-Chiu Ou
Jingxing Ou
Jodie Ouahed
Noriyuki Ouchi
Mamoru Ouchida
Martin Oudega
Ruud Oudega
Raoul Oudejans
Scot Ouellette
Nadine Ouellette
Nicholas Ouellette
Andre Ouellette
Dennis Ougrin
Allal Ouhtit
Filip Oulehle
Abderrahim Oulhaj
Peter Ouma
Alain Ourry
Merry Oursler
Tim Oury
Cécile Oury
Diana Outlaw
Stephanie Ouvrard
Odile Ouwe Missi Oukem-Boyer
Arthur Ouwehand
D. Margriet Ouwens
Diane Ouwerkerk
Hongsheng Ouyang
Minhui Ouyang
X. Ouyang
Min Ouyang
Wen Ouyang
Fang Ouyang
Bo Ouyang
Meric Ovacik
Zehava Ovadia-Blechman
Pamela Ovadje
Rupert Overall
Geertjan Overbeek
Ilse Overdevest
Simon Overduin
Christopher Overend
Will Overholt
Oyvind Overli
Edgar Turner Overton
Thomas Overton
Ed Overton
Krista Overvliet
Nestor Oviedo
Norma Oviedo
Cristina Ovilo
Johan Ovrevik
Yan Xiang Ow
Richard Owczarzy
Adrian Owen
Gareth Owen
Micheal Owen
David Owen
Caroline Owen
Shawn Owen
Nick Owen
Gary Owens
Douglas Owens
Trevor Owens
Raymond Owens

Hannah Owens
Jacob Owens
Norman Owen-Smith
Patrick Owili
Peter Owira
Doug Owsley
Leif Oxburgh
Andrew Oxenham
David Oxley
Charles Oxnard
Mototsugu Oya
Itziar Oyagüez
Tomoko Oya-Ito
Lila Oyama
Fumitaka Oyama
Esteban Oyarzabal
Patricio Oyarzun
Carlos Oyarzun
Ganiyu Oyetibo
Adewale Oyeyemi
Shefali Oza
Fatih Ozaltin
Kazunari Ozasa
Koichiro Ozawa
Yoko Ozawa
Hitochi Ozawa
Ertugrul Ozbudak
Aydogan Ozcan
Oguz Ozcelik
Engin Ozcivici
Derya Ozdemir
Burhanettin Ozdemir
Metin Ozdemir
Betul Ozdilek
Mutlu Ozdogan
Mehmet Ozdogan
Cenap Ozel
Margareth Ozelo
Nesrin Ozer
Egon Ozer
Murat Ozeren
Andrew Ozga
Riza Koksal Ozgul
Ferda Ozkinay
Elif Ozkirimli Olmez
Tammy Ozment
Keiichi Ozono
Hiroki Ozono
Fatih Ozsolak
Savas Ozsu
M Ozturk
Kadir Ozturk
Enis Ozyar
Tuna Ozyurekoglu
