# Supplementary material for: PLOS ONE 2015 Reviewer Thank You
Source: PLoS One. 2016 Feb 23;11(2):e0150341. doi: 10.1371/journal.pone.0150341 (PMC4764340; doi:10.1371/journal.pone.0150341)
Supplement: S4 Reviewer List — (PDF) [file pone.0150341.s004.pdf]

PLOS ONE would like to thank all those who reviewed on behalf of the journal in 2015:

Peter Paal
Ken Paap
Gerrit Paasche
Noel Pabalan
Reddy Pabbidi
Sudheer Kumar Pabbisetty
Christina Pabelick
Ingrid Pabinger
Stephan Pabinger
Krzysztof Pabis
Jose L. Pablos
Thomas Pabst
Ann Pabst
Karel Pacak
Daniele Pacaud
M. Paccalin
Betty Pace
Leonardo Pace
Noemi Pace
Lauren Pacek
Elena Pacella
Matthew Pachai
Beatriz Pacheco
Aldo Pacheco
Mario Pacheco
Felipe Pacheco
Alvaro Pacheco-Silva
Jesús Pacheco-Torres
Yakov A. Pachepsky
Pal Pacher
Jiri Pachernik
Steffen Pacholak
Eugenio Paci
Maurizio Pacifici
Roberto Pacifici
Krishna Pacifici
Michela Pacifici
Giovanni Pacini
Davide Pacini
Davide Pacitti
Rene Packard
Craig Packer
Laurence Packer
Rowena Packer
Muralidhar Padala
Jasdeep Padaria
Nesri Padayatchi
Christopher Paddock
Irina Pader
Kerry Padgett
Mark Padgham
Dianna Padilla
Norma Padilla
Gabriel Padilla Maldonado
Jaya Padmanabhan
Sandosh Padmanabhan
Parasuraman Padmanabhan
Venkat Padmanabhan
Nora Padola
Elisabetta Padovan
Francesco Paduano
Kyung-Hee Paek
Ki Young Paek
Jin Chul Paeng
Bosco Paes
Roberto Paes-De-Carvalho
Mark Paetzel
David Paez
Marcelo Paez-Pereda
Alessandra Paffi
Panayiotis Pafilis
One Pagan
Fernanda Paganelli
Elisabetta Pagani
Luca Pagani
Marco Pagani
Ayelen Pagani
Joseph Pagano
Marcela Pagano
Patrick Pagano
Marc Pagano
Robert Page
Rebecca Page
Anne-Laure Page

Lawrence Page
Guylène Page
Clive Page
Amanda Page
Kimberly Page
Amy Page
Matthew Page
Gerald Page
Jean-Christophe Pages
Liesl Page-Shipp
David Pagliaccio
Chiara Pagliarani
Silvia Pagliardini
David Pagliarini
Pasquale Pagliaro
Marco Pagliazzi
Fabio Paglieri
Alistair Pagnamenta
Andrea Pagnani
Valeria Pagnin
Ugo Pagnini
Cristiano Pagnini
Marco Pagnoni
Fernanda Arnhold Pagnussatt
Mariana Pagotto
Madeleine Pahl
Sabine Pahl
Sandra Pahr
Mark Pahuta
Ramdas Pai
Anand Pai
Mirko Paiardini
Alessandro Paiardini
Judith Paice
Sarah Paige
Johanna Paik
Yong-Han Paik
Taejong Paik
Eric Pailhoux
Sharon Pailler
Arnab Pain
Bertrand Pain
Debkumar Pain
Timothy Paine
Tracie Paine
Dean Paini
Francesca Paino
I. Painter
Coro Paisan-Ruiz
John Paisley
Samuel Paiva
Vitor Paiva
Sandra Paiva
Tatiane Paixao
Fauzia Paize
Krisztián Pajer
Marina Pajic
Rolando Pajon
Ahdeah Pajoohesh-Ganji
Utpal Pajvani
Daniel Pak
On Shun Pak
Mikko Pakarinen
Trevino Pakasi
Poungrat Pakdeechote
Farzad Pakdel
Mariya Pakharukova
Serguei Pakhomov
Henna Päkkilä
Sunandan Pakrashi
Utpal Pal
Biswajoy Pal
Ranajit Pal
Sikander Pal
Rahul Pal
Jayanta Pal
Tuya Pal
Csaba Pal
Magda Pal
Amit Pal
Rajesh Pal
Harish Pal
Chiranjib Pal
Kuntal Pal
Maria Pala
Adrian Palacios
Jose Palacios
Ricardo Palacios
Gustavio Palacios
Daniela Palacios
Jorge Palacios
Emanuel Palade
Fabiana Paladini
Dario Paladini
Maria Paola Paladino
Elisabetta Palagi

Laura Palagini
Srinath Palakurthi
Kara Palamountain
Raghavan Palaniappan
Latha Palaniappan
Malliya Gounder Palanichamy
Viswanathan Palanisamy
Arun Palanisamy
Thirumoorthy Palanisamy
Jayakumar Palanisamy
Ravishankar Palanivelu
Kanagaraj Palaniyandi
Suresh Palaniyandi
Senthilkumar Palaniyandi
Nades Palaniyar
Shajesh Palantavida
Gualtiero Palareti
Dawn Palaszewski
Javier Palatnik
Victoria Palau
Azhahianambi Palavesam
Santiago Palazón
Antonio Palazón-Bru
Alberto Palazzuoli
Krzysztof Palczewski
Andras Paldi
Sean Palecek
Anita Palepu
Laura Palermo
Tia Palermo
Giulia Palermo
Claire Palermo
Ferran Palero
Yuko Palesch
Luigi Palese
Gabriela Palestino
Yuval Palgi
Joana Palha
Miltiadis Paliouras
Krishna Palipudi
James Palis
Preeti Paliwal
Oleg Paliy
Eleftheria Palkopoulou
Antonella Palla
Gergely Palla
Ray Pallab
Mark Pallansch
Pierlorenzo Pallante
Virginia Pallante
Federico Pallardo
Roman Pallares
Jordi Pallarès
Vicente Pallares-Carratala
Alberto Pallavicini
Lucia Pallecchi
Mark Pallen
Claire Palles
Nicolas Pallet
S. Pallikkuth
Angelo Pallini
Deborah Palliser
Thomas Pallone
Johannes Pallua
Estelle Palluel
Günther Palm
Fredrik Palm
Carla Palma
Mário Palma
Dieter Palmberger
Karin Palmblad
Ann Palmenberg
Tracy Palmer
Robert Palmer
Caroline Palmer
Colin Palmer
Gregory Palmer
Nicholette Palmer
Kelli Palmer
R. Palmer
Matthew Palmer
Suetonia Palmer
Lucy Palmer
Lachlan Palmer
Michael Palmer
Stuart Palmer
Mark Palmer
Jeffrey Palmer
Debbie Palmer-Green
Tom Palmeri
Gaby Palmer-Lourenco
Michael Palmgren
Gianna Palmieri
Nicola Palmieri
André Palmini
Stefano Palminteri

Alida Palmisano
Marie Palmnas
Francisco Palomares
Mª Lourdes Palomares
Melanie Palomares
Oscar Palomares
Juan Palomares-Rius
Gabriela Palomo
Jennifer Palomo
C Palomo-Alvarez
Riikka Paloniemi
Bernhard Palsson
Arjan Palstra
Yniv Palti
Saverio Paltrinieri
Peter Palukaitis
Rocco Palumbo
Letizia Palumbo
Satu Palva
Vilmos Palya
Sushmita Pamidi
Frederic Pamoukdjian
Francesco Pampaloni
João Pamphile
Roger Pamphlett
Olatz Pampliega
Gülsüm Pamuk
Weiqing Pan
An Pan
Guoqing Pan
Kai-Feng Pan
Yuchun Pan
Xiaochuan Pan
Junmin Pan
Genxing Pan
Zhenwei Pan
Weihong Pan
Jay Pan
Jing Pan
Yu Pan
Min-Hsiung Pan
Szu-Hua Pan
Wei Pan
Yue Pan
Huipeng Pan
Yanyun Pan
Lizhi Pan
Jianji Pan
Liping Pan
Shuming Pan
Meng Pan
Songqin Pan
Xiaofei Pan
Ming-Ju Pan Pan
Xiangqing Pan
Yulin Pan
Yong-Bao Pan
Feifei Pan
Yuan Pan
Xunyu Pan
Haiyun Pan
Jianjun Pan
Chuxiong Pan
Ay-Woan Pan
Zui Pan
Yunfeng Pan
Qinghua Pan
Weiheng Pan
Xueliang Pan
Bing Pan
Zy Pan
Jiayi Pan
Yuepeng Pan
Theodora Panagaki
Costas Panagiotakis
Demosthenes Panagiotakos
Hanna Panagiotopoulou
Olga Panagiotopoulou
Grigorios Panagiotou
Orestis Panagiotou
Periklis Panagopoulos
Efstathios Z. Panagou
Mehdi Shafa Shariat Panahi
John Panaretos
Maria Serena Panasiti
Ganna Panasyuk
Vijay Pancholi
Preeti Pancholi
Radoslaw Panczak
Subrat Panda
Chinmay Panda
Binay Panda
Koustubh Panda
Sanjib Panda
Chethan Pandarinath
Raghvendra Panday

Abhay Pande
Akhilesh Pandey
Subhash Pandey
Amit Pandey
Satish Pandey
Virendra Pandey
Amit Pandey
Vishal Pandey
Janmejay Pandey
Ajay Pandey
Preetanshu Pandey
Alessandro Pandini
S.R. Pandi-Perumal
Nikolaos Pandis
Sandeep Pandit
Maharaj Pandit
Jaideep Pandit
Tej Pandita
Pushpa Pandiyan
Assunta Pandolfi
Silvia Pandolfi
Victoria Pando-Robles
Arturo Panduro
Sapan Pandye
Jessica Pane
Jun Panee
Jaime Paneque-Galvez
John Panetta
Isabella Panfoli
Yuan-Ping Pang
Elizabeth Pang
Yu Pang
Xiaodong Pang
Junxiong Pang
Lisa Pang
Yong Pang
Jinsong Pang
Xueyong Pang
Dalong Pang
Zhiqian Pang
Melinda Pang
Xiaoming Pang
Ka-Lai Pang
Zhifeng Pang
Stephanie Pangas
Galen Panger
Biswaranjan Pani
Beatriz Paniagua
Vincenzo Panichi
Mauro Panigada
Carol Panis
Andre Panisson
Frank Panitz
Alberto E Paniz-Mondolfi
Peter Panizzi
Subrata Panja
Mojtaba Panjehpour
Pankaj Pankaj
Sven Panke
Ned Pankhurst
Louise Pankhurst
Helga Pankoke
Michael Pankratz
Nathan Pankratz
Jaak Panksepp
Jules Panksepp
Tom Pannabecker
Pia Pannaraj
Kerstin Pannek
Yvonne Pannekoek
Nicolas Pannetier
Simona Panni
Angela Pannier
Evan Pannkuk
Giuseppe Pannone
Venkatesu Pannuru
Georgios Panos
Vily Panoutsakopoulou
Tanate Panrat
Harish Pant
Chaitanya Pant
Vandana A. Pant
Deepak Pant
D V Krishna Pantakani
Sophie Pantalacci
Vitantonio Pantaleo
Francesco Pantano
Spiro Pantazatos
Dionysios Pantazatos
Periklis Pantazis
Nikos Pantazis
Dimitrios Pantazis
Charalampos Pantazopoulos
Eric Pante
Michael Pante
Alexander Pantelyat

Mary Pantin-Jackwood
Costas Pantos
Annalisa Pantosti
Roman Pantucek
Gabriella Panuccio
Sneh Panwar
Bharat Panwar
Jitendra Panwar
Irina Panyushkina
Francesco Panza
Valentina Panzarin
Ute Panzenboeck
Ursula Panzner
Hwalin Pao
Francia Di Celle Paola
Gabriele Paolacci
George Paoli
Antonio Paoli
Chiara Paoli
Elisa Paolicchi
Francesca Paolini
Giuseppe Paolisso
Boccacci Paolo
Marco Paoloni
Daniela Paolotti
Mario Paolucci
Gregorino Paone
Akos Pap
Michele Papa
Linda Papa
Evan Papa
Roberto Papa
Gianpaolo Papaccio
Federica Papaccio
George Papadakis
Christos Papadelis
Evangelia Papadimitriou
Konstantinos Papadimitriou
Vassilios Papadopoulos
Antonios Papadopoulos
Vassilis Papadopoulos
Nestoras Papadopoulos
Barbara Papadopoulou
Nickolas Papadopulous
Vassiliki Papaevangelou
Tassos Papageorgiou
Eleni Papageorgiou
Dimitrios Papageorgiou
Silvana Papagerakis
Andriana Papaioannou
Eleni Papakonstantinou
Marianthi Papakosta
Elena Papale
Elena Papaleo
Emmanuel Papamichael
Nikolaos Papanas
Seraphim Papanikolaou
Praveen Papareddy
Domenico Paparella
Eric Papas
Panagiotis Papasaikas
Yannis Papastamatiou
Panagiotis Papastamoulis
Vasilios Papastergiou
Evangelos Papathanasiou
George Papatheodoridis
Panagiotis Papatheodorou
Stefania I. Papatheodorou
Dmitri Papatsenko
Venizelos Papayannopoulos
Panagiota Papazafiri
Stefano Papazian
Diana Papazova
Ellen Pape
Hans-Christoph Pape
L. Pape
Jesse Papenburg
Theodore Papenfuss
Monica Papes
Natalia Papeta
Chiara Papetti
Alessio Papi
Enrica Papi
Esther Papies
Padmasayee Papineni
Nico Papinutto
Sarah Papiorek
Roger Papke
Garegin Papoian
Ilona Papousek
Balázs Papp
Gabor Papp
István Papp
Federico Pappalardo
Salvatore Pappalardo
Peter Pappas

Christos Pappas
Vladislav Papper
Hanu Pappu
Rajeev Pappuru
Igor Paprotny
Jordi Paps
Paul Paquet
Dominic Paquin Proulx
Dominic Paquin-Proulx
Michael Para
Mayte Parada
Adriana Parada Silveira
Maria Parages
Kim Paraiso
Galina Paramei
Raveendran Paramesran
Teresa Paramio
Jorge Paramo
Augusto Paranhos
Mateus Paranhos Da Costa
Shruti Paranjape
Ramesh Paranjape
Shantini Paranjothy
Seetharaman Parashuraman
Manolis Paraskakis
Efrosyni Paraskeva
Steven Paraskevas
Hadi Parastar
Tamara Paravicini
Marta Parazzini
Luca Parca
Angela Parcesepe
Francois Parcy
Milosz Parczewski
Panos Pardalos
Luc Pardanaud
Daniel Pardi
Melissa Pardi
Matteo Pardini
Juan Pablo Pardo
Fernando Pardo
Thiago Pardo
Heath Pardoe
Els Pardon
Laura Parducci
Mary-Lou Pardue
Machelle Pardue
Douglas Pardue
Paul Pare
Maxime Paré
Roger Paredes
Daniel Paredes-Sabja
Ashawani Pareek
Sajid Pareeth
Martín Pareja
Helios Pareja-Galeano
Anant Parekh
Kalpaj Parekh
Samir Parekh
Jean-Marie Parel
Jiri Parenica
Carole Parent
Lucie Parent
Romain Parent
Boris Parent
Christine Parent
Daniella Parente
Astrid Parenti
Madalin Parepa
Mathews Paret
Christian Paret
Gaynor Parfitt
Peter Parham
James Parham
Ishwar Parhar
Keshab Parhi
Klaus Parhofer
Swarup Parida
Shreemanta Parida
Federico Parietti
Paolo Parigi
Robin Parihar
Urvi Parikh
Vinay Parikh
Pranav Parikh
Mansi Parikh
Mathilde Paris
Daniel Paris
Claire Paris
Christophe Paris
Carol Parise
Patricia Parise-Maltempi
Gustavo Parisi
Daniel Parisi
Christian Parisod
Amadeo Parissenti

Vani Pariyadath
Margriet Park
Jongsun Park
Hansoo Park
Su-Chan Park
Jongbae Park
Taesun Park
Chiwook Park
Frank Park
Myung Hee Park
Yoonseong Park
Soyoung Park
Woojun Park
Sung Kyun Park
June-Soo Park
Yongjin Park
Hee Soo Park
Yoonkyung Park
Hun-Kuk Park
Sunghyouk Park
Yongkeun Park
Jin Kyung Park
Jong Kuk Park
Changwon Park
Jun Yong Park
Christopher Park
Jeehye Park
Myung-Jin Park
Joonghoon Park
Tea Soon Park
Jino Park
Marcelo Park
Jaewon Park
Sung Mi Park
Hyun Park
Kyoung Chan Park
Jong-Wan Park
Ji Wan Park
Ki Ho Park
Steven Park
Sang Woo Park
Yong-Moon Park
Min Hae Park
Hee-Moon Park
Sungha Park
Won Sang Park
Mijung Park
Eun-Cheol Park
Sang Min Park
Pyo-Jam Park
Chan Kee Park
Choul Yong Park
Paul Park
Sang Ki Park
Sam-Yong Park
Gyungsoon Park
Clara Park
Joong Yull Park
Sungchul Park
Dong Park
Daeho Park
Jae-Woo Park
Ki Duk Park
Sung Chul Park
Soyeun Park
Jong-Won Park
Seong-Wook Park
Seong Yong Park
Jung-Eun Park
Chang-Jin Park
S. Park
Juyong Park
Hana Park
Su-Hyung Park
Bernadette Park
Eun-Woo Park
Chang-Hwan Park
Lani Park
Sanghyun Park
Seung Ha Park
Junyoung Park
Sang Myun Park
Hyung J. Park
Jin-Byung Park
Suk Young Park
Woo Jin Park
Young-Jun Park
Jong-In Park
Jaihyun Park
Jin Woo Park
Ji Park
Jerry Park
Yoonjung Park
Chong-Wook Park
Ji Yeon Park
Hyun-Ju Park

Susanna Park
Sangtae Park
Jong-Seung Park
B. Park
Kyong Soo Park
Deric Park
Jung Joon Park
Kyungtae Park
Kwangsung Park
Jaesung Park
Sung-Hwan Park
So-Young Park
Ian Parker
Gordon Parker
Andrew Parker
Dane Parker
William Parker
Brian Parker
Josie Parker
Tony Parker
Stephen Parker
Matthew Parker
Lisa Parker
Daniel Parker
Joel Parker
John Parker
Stacey Parker
Heather Parker
Laura Parker
Glendon Parker
Darren Parker
David Parker
Mary Parker
Laurie Parker
Mark Parker
Julian Parkhill
Isobel Parkin
Alison Parkin
John Parkington
Michael Parkins
John Parkinson
Brian Parkinson
Kathryn Parkinson
David Parkinson
William Parks
Griffith D Parks
Robin Parks
Susan Parks
Craig Parks
Nathan Parks
Onur Parlak
Valentina Parma
Pietro Parma
Kalindi Parmar
Simrit Parmar
Benoit Parmentier
Christopher Parmeter
E. Parmley
Ingela Parmryd
Grant Parnell
Laurence Parnell
Winsome Parnell
Jerome Parness
Helena Paro
Maurizio Parola
Alessandro Parolari
Victor Paromov
Maria Paola Paronetto
Federico Paroni
Vladimir Parpura
Maria Parr
Gabriel Parra
Valentina Parra
Eduardo Parra
Roberto Parra
R. Gonzalo Parra
Mauro Parra-Cordero
Grace Parraga
Isabel Parraga
Victor Parraguez
Isela Parra-Rojas
Miguel Parra-Saavedra
Cristina Parravano
Valeriano Parravicini
Paula Parreira
Lucas Parreiras-E-Silva
Paul Parren
Deborah Parris
Julia Parrish
Jean-Luc Parrou
Eldryd Parry
Christopher M Parry
Geraint Parry
Christopher Parry
Afshin Parsa
Ben Parslew

Walther Parson
Simon Parson
Larry Parsons
Henrique Parsons
Ryan Parsons
Will Parsons
Christine Parsons
Pirjo Partanen
Kaushik Parthasarathi
Sampath Parthasarathy
Seshacharyulu Parthasarathy
Ranga Parthasarathy
Mark Parthun
Santiago Partida-Sanchez
Sally Partridge
Martyn Partridge
Lynda Partridge
Sailaja Paruchuri
Avi Parush
Giridhar Parvatam
M.K. Parvez
Faruque Parvez
Suhel Parvez
Bahram Parvin
Jeffrey Parvin
Parviz Parvizi
Stesha Pasachnik
Sirichai Pasadhika
Marina Pasca Di Magliano
Géraldine Pascal
Steven Pascal
Pierre-Yves Pascal
Stefano Pascarella
Emilie Pasche
Katharina Paschinger
Ana Maria Paschoal
Nikolaos Paschos
David Pasco
Mellissa Pascoe
Peter Pascoe
Anna Roberta Pascom
David Pascual
Marta Pascual
J. Pascual
Jose Pascual
Alvaro Pascual
David Pascucci
Luisa Pascucci
Manijeh Pasdar
Matthew Pase
Josh Pasek
Anastas Pashov
Stefan Pasiakos
Laura Pasin
Piera Pasinelli
Giulio Pasinetti
Erica Pasini
Jotam Pasipanodya
Jantsje Pasma
Frank Pasmans
Massimo Pasqualetti
Jean-Louis Pasquali
Giancarlo Pasquali
Lorenzo Pasquali
Francesco Pasqualini
Marcia Pasqualini
Claudia Pasqualini
Emanuele Pasqualotto
Cristian Pasquaretta
Lucia Pasquato
Marcelo Pasquini
Pankaj Pasricha
Francesco Passafaro
Desiderio Passali
Yale Passamaneck
Luca Passamonti
Carlos Passarelli
Giuseppe Passarino
Fabio Passetti
Alberto Passi
Daniela Passilongo
Claudio Passino
Jo-Ann Passmore
Geraldo Passos
Maria Rita Passos-Bueno
Franck Pasta
Stephen Pastan
Pablo Pasten
Fernando Pasteran
R. Jeroen Pasterkamp
Jeffrey J. Pasternak
Anna Pasternak
Jesus Pastor
Fernando Pastor
Marçal Pastor-Anglada
Agata Pastorczak

Annalisa Pastore
Lucio Pastore
Alvin Pastore
David Pastor-Escuredo
Ricardo Pastori
Roberta Pastorino
Luigi Pastormerlo
Nuria Pastor-Soler
Pantazis Pastras
Alexander Pastukhov
Kalyan Pasupathy
Nagarekha Pasupuleti
Francesca Pasutto
Matthew Paszek
Cindy Paszkowski
Árpád Patai
Todd Pataky
Solenn Patalano
Swati Patankar
Manish Patankar
Manuel Patarroyo
Smita Patel
Mahomed Patel
Ketan Patel
Manish Patel
Amit Patel
Manisha Patel
Biren Patel
Hemal Patel
Vanash Patel
Mainak Patel
Jaymin Patel
Hiten Patel
Ankita Patel
Ravi Patel
Niketa Patel
Vinood Patel
Mrinali Patel
Nimesh Patel
Vyomesh Patel
Rashmi Patel
Asish Patel
Nirali Patel
Divyang Patel
Jitesh Patel
Rohit Patel
Sona Patel
Shyam Patel
Anant Patel
Meera Patel
Riyaz Patel
Kepal Patel
Pragna Patel
Molini Patel
N. Patel
Sangram Patel
Kaushik Patel
Mackenzie Pater
Marco Paterni
David Paterson
Bev Paterson
Helen Paterson
Iain Paterson
Narendra Pathak
Arvind Pathak
Jyotishman Pathak
Amit Pathak
Rambha Pathak
Varun Pathak
Simanta Pathak
Preeti Pathela
Roger Patient
Lawrence Patihis
Prabhu Patil
Rameshwar Patil
Kiran Patil
Naeem Patil
Asawari Patil
Avinash S Patil
Ujwal Patil
B. Patil
Sumeet Patil
Prasad Patil
Etienne Patin
Jairo Patino
James Paton
Chad Paton
Carl Paton
Joanne Paton
Chittaranjan Patra
Barunava Patra
Sisir Patra
Jaakko Patrakka
Rina Patramanon
Marianna Patrauchan
Vandana Patravale

Amee Patrawalla
Kristin Patrick
Rhonda Patrick
Steve Patrick
Kent Patrick
Bernadeta Patro
Rob Patro
Cesare Patrone
Enrico Patrono
Damiano. Patrono
Philippos Patsalis
Daniel Patschan
Eleonora Patsenker
Theofania-Sotiria Patsiou
Lucy Patston
Joshua Patt
Antonia Patt
Padmanabhan Pattabiraman
Sitakanta Pattanaik
Cristian Pattaro
Sivakumar Pattathil
Cheryl Patten
Gabriela Patten
Anna Patten
Bruce Patterson
Roy Patterson
Andrew Patterson
Brent Patterson
William Patterson
John Patterson
Kara Patterson
Adam Patterson
Mary Elizabeth Patti
Francesco Patti
Giuseppe Patti
Chris Pattillo
Bramhadev Pattnaik
John Patton
James Patton
Stephanie Patton
Jon Patton
Thomas Patton
Marcos Pattussi
Md. Mostofa Ali Patwary
Mark Patzkowsky
Cindy Pau
Yannick Pauchet
Alfredo Pauciullo
Khum Paudayal
Shishir Paudel
Krishna Paudel
Deepak Paudel
Priyamvada Paudyal
Friedemann Paul
Sudhir Paul
Sangeeta Paul
Ketema Paul
Mical Paul
Soumen Paul
Sushmita Paul
Dennis Paul
Michael Paul
Matthew Paul
Suman Paul
Mandira Paul
Surojit Paul
Sinu Paul
Jean-Francois Paul
Krausman Paul
Jacob Paul
Paramita Paul
David Paul
Lynn Paul
Darren Paul
Rajib Paul
Adelzon Paula
Gustav Paulay
Juraj Paule
Paolo Pauletto
Perrine Paul-Gilloteaux
Jonathan Pauli
Wolfgang Pauli
Laura Paulin
Roxane Paulin
Ariana Paulina Carabajal
Luciana Paulino
Glaucio Paulino
Hipolito Paulino-Neto
Gianni Paulis
Timothy Paulitz
Silke Paulmann
Ramasamy Paulmurugan
Ana Cristina Paulo
Ryne Paulose-Ram
Fernando Paulovich
Steffen Pauls

Goran Paulsen
Julie Paulsen
Robert Paulson
Achim Paululat
Martin Paulus
Walter Paulus
Jeremiah Paulus
Yannis Paulus
Diana Pauly
Fabienne Paumet
Christian Paumi
Tatjana Paunesku
Katarina Paunovic
Ingvild Paur
Hubert Pausch
Thomas Pausch
Silke Paust
Marco Pautasso
Alex Pauvolid-Corrêa
Patrick Pauwels
Ruben Pauwels
Kim Pauwels
Shruti Pavagadhi
Fredrick Pavalko
Gianni Pavan
Mauro Sergio Pavao
Ana Pavasovic
Gaspare Pavei
Petr Pavek
Lobachevsky Pavel
Norman Pavelka
Giulio Pavesi
Chris Pavey
Nicole Pavio
Giulia Paviotti
Sue Pavitt
Meda Pavkov
David Pavlacky
Peter Pavlidakey
Nena Pavlidi
Paul Pavlidis
Michail Pavlidis
Nicholas Pavlidis
Philip Pavlik
Boris Pavlin
Patricia Pavlinac
Gabriela Pavlinkova
Georgios Pavlopoulos
Evgeny Pavlov
Luigi Michele Pavone
Lorenzo Pavone
Snehalata Pawar
Rajendra Pawar
Graham Pawelec
Janusz Paweska
Andrzej Pawlik
Jan Pawlowski
Michal Pawlowski
Jodi Pawluski
William Paxton
Lynn Paxton
Robert Paxton
Alexandra Paxton
Kristina Paxton
Mehrdad Payandeh
Didier Payen
Celia Payen
Jessica Payne
Collin Payne
Keith Payne
Beth Payne
Brendan Payne
Mark Payne
Christopher Payne
Thomas Payne
Matthew Payne
Bernard Payrastre
Thomas Payton
Cristina Paz
Gabriela Paz-Bailey
Adam Pazda
Katarzyna Pazdzior-Czapula
Valerio Pazienza
Anna Paziewska
Florencio Pazos
Marni Pazos Espejel
Carmen Pazos-Moura
Gregory Pazour
Francesca Pazzaglia
Carlo Pazzani
Neal Peachey
Elizabeth Peacock
David Peacock
Justin Peacock
Warren Pear
Simon Pearce

Timothy Pearce
Grant Pearce
Elizabeth Pearce
J. Pearce
William Pearce
Alison Pearce
Stephen Pearce
David Pearl
Ronald Pearl
John Pearl
Daniel Pearlman
Rachel Pearlman
Catherine Pears
Ian Pearse
Talima Pearson
Angela Pearson
Frances Pearson
Amy Pearson
Amber Pearson
Yanthe Pearson
William Pearson
Melanie Pearson
James Pease
Bruno Peault
Michael Pecaut
Anna Pecchinenda
Jean Peccoud
Jan Pechenik
Kate Pechenkina
Diane Pecher
Eve-Isabelle Pecheur
Marta Pecina
Sun Peck
Kyung Peck
Robert Peck
Octavia Peck Palmer
Michelle Peckham
Lorenzo Pecoraro
Jacques Pecreaux
Tetyana Pedchenko
Marisa Pedemonte
Martin Pedersen
Lea Pedersen
Palle Pedersen
Bartholomew Pederson
Neil Pederson
Kerry Pedley
Emilia Pedone
Claudio Pedone
Rosetta Pedotti
Joao Pedra
Alisa Pedrana
José Luis Pedraz
Juan Manuel Pedraza
Salvador Pedraza
José Pedraza-Chaverri
Mario Pedrazzoli
Alessandro Pedretti
Gianni Pedrizzetti
Nuria Pedrol
Jorge Pedrosa
Andre Pedrosa
Adriana Pedroso
Peter Peduzzi
Christie Peebles
Richard Peek
Niels Peek
Jennifer Peel
Marius Peelen
Linda Peelen
Jaap Peen
Mark Peeples
Angelika Peer
Shaqil Peermohamed
Wim Peersman
Michael Pees
Marijke Peetermans
Nemo Peeters
Christian Peeters
Ben Peeters
Silvy Peeters
Eveline Peeters
Violeta Peeva
Dafni Pefani
Melanie Peffer
Manuel Pegalajar Cuéllar
Marie Pegelow
James Peggins
David Peggs
Alan Pegna
Ana Paula Pêgo
Gianluca Pegoraro
Anna Peguero
Javier Peguero-Pina
Gary Peh
Jimin Pei

Yu-Cheng Pei
Ming Pei
Xin-Wu Pei
Honglei Pei
Haiyan Pei
Sen Pei
Desheng Pei
Katja Peijnenburg
Juan Peinado
Anna Peired
Wiebke Peitsch
Sarah Peitzmeier
Amalia Peix
Carolina Peixinho
Claudia Peixoto
Luiz Antônio Peixoto
Antonio Peixoto
Zhang Peixun
Liba Pejchar
Stanislav Pekar
Miloslav Pekar
M Pekarova
Yuri Pekarsky
Kerem Pekkan
Minna Pekkinen
Jure Peklar
Dorothy Pekmezi
Sarah Pekny
Beatriz Pelacho
Carmen Pelaez-Moreno
Pawel Pelczar
Konstantinos Pelechrinis
Yoav Peleg
Pablo Pelegrin
Simone Peletto
Vladimir Pelicic
Adrian Pelin
Antoine Pelissolo
Peter Pelka
Philip Pell
Lucia Pellanda
Catherine Pellat-Deceunynck
Marc Pellegrini
Barbara Pellegrini
Roseli Pellens
Jean-Luc Pellequer
Jerry Pelletier
Johanne Pelletier
Laurent Pelletier
Julien Pelletier
Guillaume Pelletier
Philip Pellett
Sabine Pellett
Rinaldo Pellicano
Antonio Pellicer
Achille Pellicioli
Christine Pelligrini
Jean-Philippe Pellois
Claire Pellot-Barakat
Herve Pelloux
Yann Pelloux
Jennifer Pellowski
Paolo Pelosi
Gina Peloso
Pedro Peloso
Lorraine Pelosof
Perrine Pelosse
Kevin Pelphrey
Andre Pelser
Bernd Pelster
Sergey Peltek
Gretel Pelto
Mikko Peltola
Elina Peltomaa
Juha Peltonen
Clément Peltre
Gary Peltz
Karl Peltzer
Susana Peluc
Kirsten Pelz-Stelinski
J. M. Pemberton
Janusz Pempkowiak
Lindomar Pena
Javier Pena
Michelle Pena
Carlos Pena
Elena Pena
Estefania Peña
Fernando Peña
Fransisco Pena Pereira
Jose Penades
Francisco Peñagaricano
Olga Peñagarikano
Pablo Penaloza
Claudia Peñaloza
Luiz Penalva

Joana Peñarrubia
Brandt Pence
Brian Pence
Mario Pende
David Pendergast
John Penders
Gurudutt Pendyala
Frederic Pene
Cecilia Penedo
Jinrong Peng
Jun Peng
Xuan-Xian Peng
Shao-Lin Peng
Gang Peng
Hongli Peng
Ji-Bin Peng
Cheng-Yuan Peng
Dongjun Peng
Hsin-Hsin Peng
Ziwen Peng
Junjie Peng
Junhua Peng
Chang-Lian Peng
Hua Peng
Daihui Peng
Ting Peng
Jing Peng
Yong Peng
Guangneng Peng
Shushi Peng
Jamy Peng
Weiwei Peng
Mian Peng
Hanwei Peng
Zhikang Peng
Yun Peng
Hao Peng
Lei Peng
Shichun Peng
Shaobing Peng
Dingxiang Peng
Hanchuan Peng
Ping'An Peng
Kuan-Po Peng
Zongju Peng
Yisheng Peng
G.H. Peng
Chao Peng
Qian Peng
Ching-I Peng
Haipeng Peng
Rafael A. C. Penha Filho
Lucie Penin
Catherine Penington
Krisjna Penmetcha
Arthur Penn
Alex Penn
Antonella Penna
Claudia Penna
Mario Penna
Aubin Penna
Angela Pennacchio
Patchareewan Pennangpetch
E. Pennarun
James Pennebaker
Craig Pennell
Iris-Katherina Penner
Johannes Penner
Orion Penner
Andrew Penner
Chiara Pennesi
Cédric Pennetier
Trevor Penning
Steven Pennings
Giovanni Pennisi
Giuseppe Penno
Jo Pennock
Maria Pennuto
William Penny
Gordon Pennycook
Keith Pennypacker
Caterina Penone
Deepak Pental
C. Ryan Penton
Pasi Penttinen
Stefan Pentzold
Silvia Penuela
Erika Penz
Thomas Penzel
Hervey Peoples
Olimpia Pepe
Pietro Pepe
Alessia Pepe
Lieke Peper
Louis Peperzak
Pierre Pepin

Carl Pepine
Joanna Pepkezaba
Jenny Peplies
Melissa Pepling
Antonella Peppe
Maikel Peppelenbosch
Marion Pepper
Gillian Pepper
Irene Pepperberg
Rainer Pepperkok
Gert-Jan Pepping
Pedro Acl Pequeno
Pandelis Perakakis
Javier Peral
José Perales
Carmen Peralta
Alex Perálvarez-Marín
Alan Perantoni
Mark Perazella
Sandro Perazzio
Luciane Perazzolo
Matjaz Perc
Olivier Perche
Gaetano Perchiazzi
Vincenzo Perciavalle
Nikki Percival
Alan Percy
Andrew Percy
Gary Perdew
Christopher Perdue
Bonnie Perdue
Manuel Perea
M. Perea
Anna Perea
J.M. Perea Sanchez
Felipe Perecin
Javier Pereda
Marta Perego
Ugo Perego
Paola Perego
Michele Perego
Alessia Perego
Lenore Pereira
Lygia Pereira
Alexandre Pereira
Carina Pereira
Roberto Pereira
Manuel Pereira
Stephen Pereira
Pedro Pereira
Paulo Pereira
Telmo Pereira
Eugênia Pereira
Tiago Pereira
Rosangela Pereira
Felipe Pereira
Sofia Pereira
Maria Lourdes Pereira
G. Pereira
Eulália Pereira
Ulysse Pereira
Luis Pereira De Almeida
Adalberto Pereira Filho
Cecelia Pereira-Stabile
Eli Perencevich
Dominic Pérennou
Jason Perepelkin
David Perera
Minoli Perera
Subashan Perera
Ranawaka Perera
Rushika Perera
Raphael Peres
Marco Peresani
Pedro Peres-Neto
Alfredo Peretti
Jackye Peretz
Sabine Pereyre
Daniel Perez
Luis Perez
Paloma Perez
Jesus Perez
Sonia Perez
Sylvia Perez
Alberto Perez
Marco Perez
Larry Perez
Viviana Perez
Agustin Perez
Ma Perez
Juan Jesús Pérez
Francisco Pérez
M. A. Pérez
Maira Pérez
Thierry Pérez
Luz Pérez

Analía Pérez
Jessica Perez Alquicira
Teodosio Perez Amaral
T. Perez Cervera
Gonzalo Pérez De Lis Castro
Luis Pérez De Sevilla Mueller
Bernat Pérez De Val
Juan Manuel Pérez García
Guillem Pérez I De Lanuza
Juliana Perez Laspiur
Gaspar Pérez Martínez
Francisco Jose Perez Reche
Tania Perez Sanchez
Miguel Pérez-Amador
Soledad Pérez-Amodio
Martin Perez-Andres
Miguel Angel Perez-Angon
Amaya Perez-Brumer
David Perez-Callejo
Rafael Pérez-Cambrodi
Tomás Pérez-Contreras
Daniel Perez-Cremades
Belen Perez-Dueñas
Koraly Pérez-Edgar
Miguel Perez-Enciso
Alfonso Perez-Escudero
Miguel Perez-Fontan
M. Teresa Perez-Garcia
Jordi Perez-Gil
Jorge Perez-Gomez
Marcos Pérez-Losada
Leonor Perez-Martinez
Xochitl Perez-Martinez
P. Pérez-Matute
Valentín Pérez-Mellado
Oscar Pérez-Méndez
Manuel Perez-Molina
Mirna Perez-Moreno
Rogelio Perez-Padilla
Rogelio Pérez-Padilla
Eduardo Pérez-Palma
Danilo Pérez-Pantoja
Guillermo Perez-Perez
José Manuel Pérez-Pérez
Antoni Perez-Poch
Regino Perez-Polo
José Pérez-Pomares
Edward Perez-Reyes
Yasset Perez-Riverol
Ana Perez-Ruiz
L. Pérez-Salcedo
Pau Perez-Sales
Ricardo Pérez-Sánchez
Jaume Pérez-Sánchez
Josue Perez-Santiago
Jose Perez-Simon
Emilio Pérez-Trallero
Javier Perez-Tris
Mario Pérez-Zepeda
Jose Perez-Zoghbi
John Perfect
Christoph Perger
Claudio Peri
Maria Periago
Julien Périard
Muthu Periasamy
Tania Perich
Norberto Perico
Luca Perico
Eva Pericolini
Marlene Perignon
Bruno Perillo
Laura Perin
Alessandro Perina
Emmanuel Perisse
Palsamy Periyasamy
Joep Perk
Susan Perkins
Stephen Perkins
Timothy Perkins
Brian Perkins
Alex Perkins
Neil Perkins
Axel Perkonigg
Vlado Perkovic
Mario Perl
Gabor Perlaki
Vincent Perlbarg
Stefano Perlini
Stanley Perlman
Steven Perlman
Eran Perlson
Irina Perminova
Andrea Perna
Cyril Pernet
Fabrice Pernet

Olivier Pernet
Guey Chuen Perng
Alessandra Pernis
Ana Carolina Pero
Clara Peron
Georgia Perona-Wright
Daniela Perotti
Juan Perotti
Silvia Perotto
Olga Perovic
Milka Perovic
Nicola Perra
Michael Perrais
Adrien Perrard
Anne-Laure Perraud
Victoria Perreau
Claude Perreault
Jean-Pierre Perreault
Leigh Perreault
Aurel Perren
Stéphanie Perret
David Perrett
Stéphane Perrey
Eduardo Perri
Tamar Perri
Francesco Perri
Carlo Perricone
Roberto Perricone
Emeline Perrier-Groult
Richard Perrin
Philippe Perrin
Benjamin Perrin
Shane Perrine
John Perrine
Cria Perrine
Susan Perrine
Laurent Perrinet
Sebastio Perrini
Karl Perron
Giuseppe Perrone
Sandro Perrone
Xavier Perrot
Julie Perroy
Helen Perry
Linnea Perry
Conrad Perry
Linda Perry
Gavin Perry
Roland Perry
James Perry
Samantha Perry
Ben Perry
Lin Perry
Roger Perry
Jacques-Olivier Pers
Carol Persad
Luca Persani
Krishna Persaud
Navindra Persaud
Nav Persaud
Pedro Persechini
Gianluca Perseghin
Suzann Pershing
Yuri Persidsky
Michael Persinger
Derek Peršoh
Amanda Persons
Lars Åke Persson
Jenny Persson
Karina Persson
Patrik Persson
Rutger Persson
Björn Persson
Ylva Persson
Darlene Persuhn
Francesco Perticone
Maria Pertl
Cino Pertoldi
Arkady Pertsov
Inna Pertsovskaya
Fernando Peruani
Piero Perucca
Antonella Peruffo
Omathanu Perumal
Venkatachalam Perumal
Senthilkumar Perumal Kuppusamy
Ramar Perumal Samy
Janice Perussi
Francesca Peruzzi
Denis Peruzzo
Konstantin Pervushin
Olivia Perwitasari
Guy Peryer
Patricia Pesavento
David Pescador
Lorenzo Pesce

Caterina Pesce
Maurizio Pesce
Beate Pesch
Andreas Peschel
Angelo Peschiaroli
Everett Pesci
Milica Pešic
George Pess
Eliano Pessa
Antonello Pessi
Mauro Pessia
Alan Pessier
Augusto Pessina
Luiz Pessoa
Bruno Pessoa
Diogo Pestana
Manuel Pestana
Mario Pestarino
Mariana Petaccia
Toni Petan
Fiona Petchey
Davorina Petek
Karlheinz Peter
Trevor Peter
Miklos Peterfy
Christoph Peterhänsel
B. Matija Peterlin
Borut Peterlin
Thomas Peterman
Joel Peterman
Scott Peterman
Cindy Peternelj-Taylor
Heiko Peters
Richard Peters
Nathan Peters
Jay Peters
Harm Peters
Jan Peters
Christopher Peters
Ralph Peters
Brian Peters
Reuben Peters
Valerie Peters
Ryan Peters
Francesc Peters
Christian Peters
David Peters
Anna-Lena Peters
Owen Peters
Inga Peters
Megan Peters
Kitt Petersen
Christine Petersen
Kyle Petersen
Esben Petersen
Jessica Petersen
Bjoern Petersen
Christian Petersen
Alexander Petersen
Chris Petersen
Inge Petersen
Frank Petersen
Fernanda Petersen
John Petersen
Eskild Petersen
Asa Petersen
Sibylle Petersen
Jens Petersen
Bent Petersen
Matt Petersen
Gitte Petersen
Sesilje Petersen
Jens Kjerulf Petersen
Svend Petersen-Mahrt
Marc Peters-Golden
Townsend Peterson
Randall Peterson
Martha Peterson
Ellena Peterson
Kenneth Peterson
Mark Peterson
David Peterson
Christopher Peterson
Scott Peterson
Jonathan Peterson
Stephen Peterson
Daniel Peterson
Dwight Peterson
Ib Krag Peterson
Catherine Peterson
Dana Peterson
James Peterson
Gabriel Peterson
La Peterson
Zoe Peterson
Anya Peterson Royce

Wolfgang Peti
Patrick Petignat
Jean-Marc Pétillon
Marie-Agnès Petit
Odile Petit
Isabelle Petit
Jean-Michel Petit
Amelie Petitclerc
Pierre Petitgas
Eleni Petkari
Nikolai Petkau
Trevor Petney
Maxim Petoukhov
Lisanne Petracca
Felice Petraglia
Melissa Petrakis
Panagiotis Petrantonakis
Václav Petráš
Antonello Petrella
K. Petrides
Dennis Petrie
Marco Petrillo
Karin Petrini
Thomas Petro
Nicola Petrocchi
Andrea Petróczi
Basil Petrof
David Petroff
Andy Petroianu
W. Petroll
Walter Matthew Petroll
Pierpaolo Petrone
Linda Petrone
Katia Petroni
Isabelle Petropoulos
George P. Petropoulos
Ioannis Petropoulos
John Petros
Laura Petrosini
Agne Petrosiute
Katherina Petrou
Alex Petrov
Georgi Petrov
Constantinos Petrovas
Predrag Petrovic
Mriko Petrovic
Isidora Petrovic
Natasa Petrovic
Andreas Petrovic
Goran Petrovski
Dimitri Petrovykh
Hilda Petrs-Silva
Elisa Petruccioli
Michele Petruzzelli
Clive Petry
Franck Petry
Anna Petryk
Tracey Petryshen
Nikolaos Petsas
Sarah Pett
Tristan Pett
Salvatore Petta
D. Pettay
Davide Pettener
Matthew Pettengill
James Pettengill
Alexander Petter-Puchner
Sven Pettersson
Filippa Pettersson
Carloalberto Petti
Melinda Pettigrew
Paul Pettitt
Frank Petzke
Axel Petzold
Gabor Petzold
Thomas Petzoldt
Thomas Peulen
Paul Pevet
Jonathan Pevsner
Payam Peymani
Adrien Peyrache
Jean-Marc Peyrat
Marisa Peyre
Eric Peyretaillade
Olivier Peyruchaud
Vsevolod Peysakhovich
Carole Peyssonnaux
Shelly Peyton
John Paul Pezacki
Hamid Pezeshk
Behnaz Pezeshkpoor
Sophie Pezet
Isabelle Pezron
Roberto Pezza
Leo Pezzementi
Raffaele Pezzilli

Lorenzo Pezzoli
Annalisa Pezzolo
Patrizio Pezzotti
Luciano Pezzullo
Francesca Pezzuto
Florian Pfab
Jim Pfaendtner
Alexander Pfaff
Peter Pfaffelhuber
Michael Pfaller
George Pfaltzgraff
Kenneth Pfarr
Stefan Pfattheicher
Doreen Pfau
Sebastian Pfautsch
Sebastien Pfeffer
Lawrence Pfeffer
Ulrich Pfeffer
Jennifer Pfeifer
Yvonne Pfeifer
Bernhard Pfeifer
Michael Pfeiffer
Norbert Pfeiffer
Christian Pfeiffer
Friedhelm Pfeiffer
Deirdre Pfeiffer
Ronald Pfeiffer
Ferris Pfeiffer
Waltraud Pfeilschifter
Karen Pfennig
Pascal Pfiffner
Thorsten Pfirrmann
Riccardo Pfister
Jean-Pascal Pfister
Donald Pfister
Cathie Pfleger
Hans Pflueger
Annette Pflugfelder
Francoise Pflumio
Peter Pfordrescher
Carmen Pfortmueller
Timo-Kolja Pförtner
Michael Pfreundschuh
Gert Pfurtscheller
David Phalen
Mirko Pham
Christine Pham
Tam Pham
Tuan Pham
Lan Pham
Son Pham
Thinh Pham
Sem Phan
An-Tuan Phan
Tri Phan
Gregg Phares
Paul Pharoah
Anastasia Pharris
Carmen Pheiffer
Paul Phelan
John Philbeck
Robert Philibert
Pierre Philip
Sairu Philip
Melanie Philipp
Andrew Philippides
N. Philips
Sjaak Philipsen
Casandra Philipson
Julie Philley
Veit Phillip
Helen Phillippou
Patrick Phillips
Colin Phillips
Gregory Phillips
Christopher Phillips
Wayne Phillips
Susan Phillips
Joanna Phillips
Mark Phillips
Robert Phillips
Ruth Phillips
Matthew Phillips
Jacqueline Phillips
Andrew Phillips
Caleb Phillips
Elizabeth Phillips
Lisa Phillips
Rs Phillips
Alison Phillips
Tina Phillips
Glenn Phillips
James F Phillips
Craig Phillips
Bonnie Phillips
Julie Phillips

Kathryn Phillips
Cynthia Phillips
Bob Phillips
Thomas Phillips
J. Phillips
Anna C Phillips
Penelope Phillips-Howard
Andrea Phillott
Benjamin Philpot
Caroline Philpott
Stuart Phinn
Donald Phinney
Angkoon Phinyomark
Richard Phipps
Amanda Phipps
Colin Phipps
Helen Phipps
William Phipps
Amornrat Phongdara
Yu Phua
Vorapong Phupong
Lorenzo Pia
Giorgio Piacentini
Maria Piacentini
Andy Piacsek
Alberto Piaggesi
Giulia Piaggio
Antoinette Piaggio
Eliane Piaggio
Jean-Philippe Pialasse
Mariagrazia Piancino
Timothy Pianta
Steven Piantadosi
Donald Pianto
Zhongyun Piao
Renaud Piarroux
Cyrille Piatecki
John Piatt
Adriano Piattelli
M. Blanca Piazuelo
Paolo Piazza
Francesco Piazza
Stephen Piazza
Jennifer Piazza
Roxane Piazza
Gabriella Piazzesi
Manuela Piazzi
Ana Picado
Didier Picard
Martin Picard
Franck Picard
Fabien Picard
Brigitte Picard
Vincent Picard
Ernesto Picardi
Angelo Picardi
Mauro Picardo
Gianluca Picariello
Nathalie Picault
Pier Paolo Piccaluga
Simone Picchi
Carla Piccinato
Camila Piccinin
Giuseppe Picciolo
David Piccioni
Alessandra Piccirillo
Cristiana Picco
Tommaso Piccoli
Renata Piccoli
Stefano Piccolo
John Piccolo
Pietro Picerno
Jean-Baptiste Pichancourt
Muriel Pichavant
Thierry Piche
Marc Picheral
Michael Pichichero
Renate Pichler
Sylvie Pichon
Aurelien Pichon
Samuel Pichon
Michel Pichon
Jacques Pichon
Sabrina Pichon
Kathy Pichora-Fuller
Jean-Francois Picimbon
Mark Pickard
John Pickard
Paul Pickell
Martin Pickering
Amy Pickering
Anthony Pickering
John Pickering
Rm Pickering
Will Pickett
Kristen Pickett

Perry Pickhardt
Peter Pickkers
Jana Pickova
David Pickup
Yolanda Pico
Belén Picó
Delia Picone
Antonia Picornell
Miguel Picornell
Stephane Picot
Sandrine Picq
Paul Picton
Anabela Picton
Laura Piddock
Toby Piddocke
Nataliya Pidkovka
Sacha Pidot
Pedro Piedra
Erika Piedras-Renteria
Michael Piehler
Gary Pielak
Lorenzo Piemonti
Alison Pienciak
Martin Pienkowski
Rembert Pieper
Robert Pieper
Sofie Piepers
Hans-Peter Piepho
Massimo Piepoli
Alessandra Pierani
Riccardo Pierantoni
Denis Pierard
Susan Pierce
Raymond Pierce
Graham Pierce
G.N. Pierce
Annick Pierce
Christine Pierce Campbell
William Pierceall
Jonathan Pierce-Shimomura
Julius Piercy
Jean-Yves Pierga
Angela Rose Piergiovanni
Carlo Piermarocchi
Marco Pieroni
Laurent Pierot
Theunis Piersma
Theodore Pierson
Elizabeth Pierson
Jennifer Pierson
Mariusz Pierzchala
Jacob Piet
Catherine Pietanza
Gabriella Pietra
Filippo Pietrantonio
Maria Chiara Hiara Pietrogrande
A. Pietroiusti
Davide Pietropaoli
Susanna Pietropaolo
Michael Pietrusewsky
Maciej Pietrzak
Uwe Pietrzyk
Tim Pietsch
Corinna Pietsch
Jakob Pietschnig
Jacques Piette
Laurent Pieuchot
Paul Piff
Simone Pifferi
Gwenael Piganeau
Jon Piganelli
Ivan Pigarev
Wilfred Pigeon
Marie Pigeyre
Kathleen Pigg
Hugh Piggins
Jeremy Piggott
Sandro Pignatti
Emanuele Pignoli
Vincent Piguet
Olivier Piguet
Frank Pigula
Stephen Pihlaja
Lasse Pihlstrøm
Catherine Pihoker
Youry Pii
Jyrki Piilo
Albrecht Piiper
Gorben Pijlman
Daniel Pijnappels
Jeremy Pike
Alison Pike
Tom Pike
Maria Pikilidou
Barbora Piknova
Bettina Pikó

Arkady Pikovsky
Christian Pilarsky
Ivan Pilas
Adrian Pilatz
Andrew Pilecki
Henriette Pilegaard
Kim Pilegaard
Cynthia Pileggi
Stefano Pileri
Nicholas Pilfold
David Pilgrim
Erik Pilgrim
Roberto Pili
Aleksandr Pilipenko
Adrian Piliponsky
Natasha Pilkauskas
F. Pilkington
Nikolaj G. Pillai
Girinath G. Pillai
Satish Pillai
Ramesh Pillai
Radhakrishna Pillai
Rashmi Pillai
Dylan Pillai
Sreekumar Pillai
Valério Pillar
Micheli Pillat
Vincent Pillaud
Viness Pillay
Evangéline Pillebout
Elena Pilli
Manohar Pilli
David Pilliod
Sandra Pillon
Argenia Paola Pilloni
Julien Pilme
Marc Pilon
Nicolas Pilon
Louise Pilote
Sara Pilotto
Paul Pilowsky
Karin Pilz
Luiz Pimenta
Gustavo Pimentel
Dulce Pimentel
David Pimentel
Genaro Pimienta
Catalina Pimiento
Jose Pimiento
Stuart Pimm
Christopher Pin
Chueh Pin Ju
Cristina Pina
Ileana Pina
Elisabeth Pinart
Juan Pablo Pinasco
Sylvain Pincebourde
William E. Pinchak
Yang Pinchen
Seth Pincus
Díaz-Jaimes Píndaro
Kirit Pindolia
Pascal Pineau
Nicolas Pineault
Jose Pineda
Federico Pineda
Miguel Pinedo
Elisabeth Pinel
Fabio Pinelli
Fulvio Pinelli
Miguel Pineros
Jesse Pines
Florence Pinet
Pablo Pineyro
Alessandro Pingitore
Melissa Pingree
Antônio Pinheiro
Ana Acacia Pinheiro
Hudson Pinheiro
Leonardo Pinheiro
Rejane Pinheiro
Fabiano Pinheiro Da Silva
Armando Pinho
Vanessa Pinho
Andreia Pinho
Greg Piniak
Abdul Pinjari
Michael Pinkawa
Amy Pinkham
Elmar Pinkhardt
Graziano Pinna
John Pinney
Susan Pinney
Gavin Pinniger
Bradley D. Pinno
Pietro Pinoli

Gregory Pinon
Nikos Pinotsis
Maria Pino-Yanes
Martin Pinquart
Paul Pinsky
Michael Pinsky
Jose Pintado
John Pintar
Lionel Pintard
Emmanuel Pinteaux
Lancelot Pinto
Yigal Pinto
Miguel Pinto
Rogerio Pinto
Caroline Pinto
Joseph Pinto
Joana Pinto
Jose Pinto
Marcelo Pinto
Gustavo Pinto
Perpétua Pinto-Do-Ó
Roberto Pinton
Giorgio Pintore
Filipa Pinto-Ribeiro
Carlos Pinzón-Flórez
Marco Piola
Daniele Piomelli
Augen Pioszak
Niels Piot
Jeff Piotrowski
Andrea Piotti
Elisa Piovano
Damiano Piovesan
Daniel Pipeleers
Karen Piper Hanley
Pantelis Pipergias Analytis
Dolores R. Piperno
Alberto Piperno
G.C. Teg Pipes
Matthew Pipkin
Jose Piqueras
Jacques Pir
Stefano Piraino
Beth Piraino
Antonio Piralla
José Pirani
Federica Piras
Fabrizio Piras
Mahendra Piraveenan
Marco Pirazzini
Joachim Pircher
Jose Pires
Camilla Pires
Douglas Pires
José C.M. Pires
Jouko Pirhonen
Grisha Pirianov
Flavia Pirih
Angela Pirillo
Eija Pirinen
Christian Pirk
Robert Pirker
Giuseppe Pirlo
Jean-Paul Pirnay
Rosario Piro
Edyta Pirog
Carlos Pirola
Luciano Pirola
Cary Pirone-Davies
Loris Pironi
Carlos Pirovani
Romain Pirracchio
Angelo Pirrone
Patrick Pirrotte
Pertti Pirttiniemi
Antonio Pisabarro
Elizabeth Pisani
Antonio Pisani
Didier Pisani
Luciana Pisani
Eva Pisano
Kasia Pisanski
Gideon Pisanty
Irene Pisanty
Fabio Piscaglia
Sven Pischke
Tobias Pischon
John Pisciotta
Addolorata Pisconti
David Pisetsky
Pramod Pisharady
Trairak Pisitkun
Gilles Pison
Alberto Pisoni
Mauro Pistello
Emidio Pistilli

Marco Pistis
Vito Pistoia
Francesca Pistoia
Fernando Piston
Wojciech Pisula
Ewa Pisula
Lucía Pita
Salvador Pita-Fernández
Teerat Pitakrat
Nayla Pitangui
Ramasamy Pitchappan
Graham Pitcher
David Pitcher
Brandelyn Pitcher
Mark Pitcher
Benjamin Pitcher
Frédérique Pitel
Fiabo Pitella Silva
Raymond Pitetti
Patrick Pithua
Kaisu Pitkala
Asla Pitkanen
Nigel Pitman
Dario Pitocco
Leonardo Pitombo
Yannis Pitsalidis
Werner Pitsch
Andrew Pitsillides
Bruce Pitt
Geoffrey Pitt
Kylie Pitt
Christopher Pittenger
Sharon Pitteri
Andre Pittig
Mauro Pittiruti
Simon Pittman
Alan Pittman
Jamie Pittock
R. Pitts
David Pitts
Virginia Pitzer
Chiara Piubelli
Roberta Piva
Francesco Piva
Pascal Piveteau
Irina Pivneva
Lara Pivodic
Theresa Pizarro
Luis Pizarro
Cristina Pizarro-Irizar
Fabio Pizza
Diego Pizzagalli
Massimo Pizzato
Elisabetta Pizzi
Salvatore Pizzo
Lisa Pizzol
Roberto Pizzolotto
Clara Pizzuti
Teresa Pizzuti
Trenton Place
Sandra Plachta-Danielzik
Bodo Plachter
Fabio Placidi
David Plackett
Patrick Pladys
Scott Plafker
Léon Plaghki
Karsten Plamann
Helene Plamondon
Stephen Planck
Emmanuel Planel
Vicente Planelles
Serge Planes
Rebeca Plank
Michael Plank
Maximilian Plank
Gregory Plano
Stephanie Planque
Robert Planque
Pedro Plans
Ewan Plant
Giles Plant
Richard Plant
Elena Plante
Yves Plante
Courtney Plante
Laurent Plantier
Laura Plantinga
Domenico Plantone
Bryce Plapp
Dietrich Plass
Jeremiah Plass-Johnson
Carlos Platas Iglesias
Diana Platas-Neri
Jonathan Platkiewicz
Michael Platow

Daniel Platt
William Platt
Roy Platt
Jennica Platt
Harald Platta
Timothy Platts-Mills
Uwe Platzbecker
Edward Platzer
Jessica Plavicki
Kevin Plaxco
Julio Plaza-Diaz
W. Plazak
Giuseppe Plazzi
David Pleasure
Mario Plebani
Milan Plecas
Barbara Plecko
Jürgen Pleiss
Richard Plemper
Paul Plener
Robert Plenter
Nancy Pleshko
Ole Pless
Robert Pless
Charles Plessy
Sergei Pletnev
Jonathan Plett
Mathias Pletz
Belinda Pletzer
Michael Plevin
Thorsten Plewan
Dariusz Plewczynski,
Christos Pliatsikas
Michael Plichta
Rainer U Pliquett
Danuta Plisko
Steve Pliszka
Jeff Plochocki
Rutger Ploeg
Till Ploenes
Birgit Ploier
Robert Plomin
Jaap Plomp
Till Plönes
Przemyslaw Plonka
Jonathan Ploski
Lilian Plotkin
Claudia Plottel
Anne Plotto
Ingrid Plotton
Brian Plouffe
Louis Plough
Stephane Plourde
Lydie Ploux
Edward Plow
Jeff Plowman
Mateusz Plucinski
Michael Pluess
Mark Plumbley
Jacqueline Plumbridge
Paul Plummer
Kim Plummer
Andrew Plunk
Malcolm Plunkett
Tomáš Pluskal
Wojciech Pluskiewicz
Barbara Plytycz
May Ee Png
Graham Poage
Leonid Pobezinsky
Ana Paula Poblacion
Radhika Pochampally
Derek Pociask
Katherine Pocius
Alan Pockley
Miguel Pocovi
Istvan Pocsi
István Pócsi
Benjamin Podbilewicz
Maria Podda
Juan Poderoso
Appa Rao Podile
Daniele Podini
Natraj Kumar Podishetty
Petar Podlesniy
Boris Podobnik
Vedran Podobnik
Jewel Podratz
Gina Poe
Jonathan Poe
Ella Poels
Klaas Poelstra
Steven Poelzing
T.L. Poepping
Timm Poeppl
Angelika Poesel

Christian Poets
Maya Poffenberger
David Poger
Lucia Poggi
Paolo Poggio
Santiago L. Poggio
Eleonora Poggiogalle
Gennady Pogorelko
Igor Pogribny
Robert Pogue
Chit Laa Poh
Kian Keong Poh
Michal Pohanka
Raimo Pohjanvirta
Sandra Pohl
Petra Pohl
Martin Pohl
Paula Pohlmann
Stefan Pöhlmann
Rolf Pohmann
Georg Pohnert
Shivaram Poigai Arunachalam
George Poinar
Laurent Poirel
Steve Poirier
Marc Poirot
Timothée Poisot
Vincent Poitout
S. Poitras
David Poitz
Martina Pokorná
Mieczyslaw Pokorski
Marta Pokrywczynska
Marta Pola
Norbert Polacek
Julio Polaina
Michal Polak
Marta Polak
Rani Polak
Kinga Polanska
Jaroslaw Polanski
Thomas Polasek
Uri Polat
Bart Polder
Tatyana Polenova
Lubos Polerecky
Jerry Polesel
Piero Poletti
Venerino Poletti
Barbara Poletti
Michele Poletti
Chiara Poletto
Joanna Polewska
Gianluca Polgar
Graeme Polglase
Philip Polgreen
Linnea Polgreen
Valeria Poli
Andrea Poli
Sven Poli
Muriel Poli
Daniela Poli
Carlos Poli De Figueiredo
Ellen Poliakoff
Eugenia Poliakov
Andrew Poliakov
Maria-Cristina Polidori
Rafael Polidoro
Alexios Polidoros
Jean-Baptiste Poline
Damon Polioudakis
Kishore Polireddy
John Polisar
Alessandra Polissi
Pierluigi Politi
Luis Politi
Ioannis Politikos
Stan Politis
Angelo Polito
Anastasia Politou
Mario Poljak
Zvonimir Poljak
D. Brent Polk
Linda Polka
Jessica Polka
Adam Polkinghorne
Ben Polkinghorne
Aleksandra Polkowska
Harold Pollack
Anna Pollack
Harvey Pollack
Victor Pollak
Martin Pollak
Georgios Pollakis
Michael Pollanen
Eija Pöllänen

Justin Pollara
Andrew Pollard
Susanna Pollastri
Martina Pollastrini
Juergen Polle
Andrea Polle
Spencer Polley
Frank Pollick
Howard Pollick
Toni Pollin
Peter Pollner
Pamela Pollock
Alex Pollock
Raphael Pollock
Kristian Pollock
Tresa Pollock
Kevin Pollock
Richard Pollok
Karen Pollok
Erqi Pollom
Luca Pollonini
Petra Pollux
Nuria Polo
Victoria Polonis
David Pols
Andrew Polson
Annikka Polster
Stephan Polterauer
Fabio Polticelli
Elisabetta Poluzzi
Patrizia Polverino De Laureto
Lina Polvi Sjöberg
David Polya
Stephen Polyak
Maksym Polyakov
Constantin Polychronakos
Magdalini Polymenidou
Ines Polzer
Constança Pomba
Jean-François Pombert
Marco Pombi
Jeremy Pomeroy
V. Pomeroy
Kimball Pomeroy
Régis Pomès
Yves Pommier
Celine Pompeia
Eugenio Pompeo
Assunta Pompili
Maurizio Pompili
Denis Pompon
María Ponce
Ninez Ponce
Adrian Ponce-Alvarez
Julia Poncela
Pascal Poncet
Aurore Ponchon
Dimity Pond
Richard Pond
Santhi Pondugula
Alexandra Ponette
Raymond Pong
Chatlert Pongchaiyakul
Jean-Francois Ponge
Drazenka Pongrac
Peter Pongracz
Evgeni Ponimaskin
V.K. Chaithanya Ponnaluri
Sreenivasan Ponnambalam
Moorthy Ponnusamy
Senthil Kumar Ponnusamy
Mikhail Ponomarenko
Igor Ponomarev
Vladimir Ponomarev
Eugene Ponomarev
Marisa Ponpuak
Xavier Pons
Miquel Pons
Ferran Pons
Thijs Pons
Siriluck Ponsuksili
Alicia Ponte Sucre
Andrew Ponter
Antonio Ponti
Stephanie Pontier
Patrizia Pontisso
Jordi Pont-Tuset
Maurilio Ponzoni
Mikhail Pooggin
Martin Pool
John Pool
Eva-Maria Pool
Daniele Poole
David Poole
Daniel Poole
Chaithep Poolkhet

Leona Poon
Kwun Yee Poon
Selvamuthu Poongulali
Simandeep Poonian
Stephen Poor
A. Poore
Jalal Poorolajal
Marijn Poortvliet
Wirulda Pootakham
Shabnam Pooya
Ioan Pop
Calin Popa
Andreea Popa
Vlad Tudor Popa
Roman Popat
Hashmat Popat
Sudeep Popat
Aurel Popa-Wagner
Janet Pope
Welkin Pope
Carey Pope
Phillip Pope
Lisa Pope
Bernard Pope
Rachel Popelka-Filcoff
Viorel Popescu
Gabriel Popescu
Ana Maria Popescu
Mihail Popescu
David Popham
Frank Popham
Lukasz Popiolek
Daniel Popkin
Stephen Popkin
Tony Pople
Michel Popoff
Steven Popoff
Patrizia Popoli
Serguei Popov
Tzvetan Popov
Lucy Popova
Phillip Popovich
Jürgen Popp
Julius Popp
Magnus Popp
Walter Popp
Arthur Popper
Nurith Porat
Francesca Porcellati
Fernando Porcelli
Raphaël Porcher
David Porciani
Vittorio Porciatti
Marko Porcic
Travis Porco
Rachel Poretsky
Maurizio Porfiri
Filippos Porichis
Owen Pornillos
Stephen Poropat
Matteo Porotto
Valeriy Poroyko
Vincent Porphyre
Thibaud Porphyre
Gustavo Porpino
Guillaume Porraz
Enzo Porrello
Daniele Porretta
Michael Porrier
Esteban Porrini
Danilo Porro
Renato Porrozzi
J Porszasz
Alberto Porta
Miquel Porta
Erja Portegijs
Jeanell Portelli
Todd Porter
Warren Porter
Richard Porter
Christopher Porter
Nada Porter
Lisa Porter
Lauren Porter
Leila Porter
Weston Porter
Alan Porter
Brenda Porter
George Porter
Jason Porter
Teresita Porter
James Porter
Augustine Porter
Ryan Porter
Melanie Porter
Megan L Porter

Chad Porter
Claire Porter
Pierre Portero
Matthew Porteus
Christine Portfors
Ilga Porth
Hugues Portier
Javier Portilla
Carlos Portillo-Quintero
Piero Portincasa
Michael Portman
Alla Portnychenko
Garca Porto
José Portoles
Laercio Porto-Neto
Isabel Portugal
Silvia Portugal
José Portugal
Steve Portugal
Raquel Portugal
Kevin J Portune
Stefan Porubsky
Andrea Porzionato
Klaas Pos
Inmaculada Posadas
Anna Posadino
Martin Posch
Florian Posch
Charlotte Poschenrieder
Claudia Pösel
Matthew Posewitz
James Posey
Vincenzo Positano
Nikki Posnack
Andreas Pospischil
Lourival Possani
Rory Post
Frank Post
Steven Post
Gerald Post
Max Post van der Burg
Pieter Postema
Michael Posthumus
Catherine Postic
Guillaume Postic
Thomas Postler
John Postlethwait
Arnold Postlethwaite
Joeke Postma
Maarten Postma
Johannes Postma
Tom Postmes
Judy Postmus
Svetlana Postnova
Dragos Postolache
Maurizio Postorino
Vita Postuvan
Bruno Pot
Zuzana Potacova
Peter Potapov
Cristhian Potes
Andries Potgieter
Jens Poth
David Pothier
Charalabos Pothoulakis
Delphine Potier
Prasanth Potluri
Lakshmi-Prasad Potluri
Neha Potnis
Samuel Potolichio
Tatjana Potpara
Heidrun Potschka
Christian Pott
Stephane Potteaux
Hans Pottel
Kathleen Potter
Philip Potter
Lesley Potter
Barry Potter
Nicholas Potter
Jaime Potter
Matthew Potthoff
Nicolas Pottier
Tom Pottinger
Jonathan Potts
Malia Potts
Raghava Potula
Harihara Potula
Corey Potvin
Patrice Poubelle
Krishna Poudel
Durga Poudel
Hemant Poudyal
Mahmoud Pouladi
Pierre Poulain
Bernard Poulain

Dimos Poulikakos
Robert Poulin
Ray Poulin
Gino Poulin
Diane Poulin-Dubois
Florence Poulletier De Gannes
Michael Poulsen
Lars Poulsen
Bo Poulsen
Mads Poulsen
Michael Poulter
Russell Poulter
Joanna Poulton
George Poultsides
Catherine Pound
Michael Pound
Dean Pountney
Rodolphe Poupardin
Aidin E. Pour
Nader Pouratian
Farshad Pourmalek
Spyros Pournaras
Philippe Pourquier
Frédéric Pouzoulet
Eva Poveda
Michael Povelones
William Powderly
William Powell
Frank Powell
Don Powell
Timothy Powell
Saul Powell
Scott Powell
Joann Powell
Daniel Powell
Rebecca Powell
Matthew Powell
Jennifer Powell
Terrinieka Powell
Kevin Powell
Jonathan Powell
Emily Powell
Tiffany Powell Avila
Mary Power
Christopher Power
Carl Power
Deborah Power
Robert Power
Anne Marie Power
David Power
Robert Powers
Jenny Powers
Jennifer Powers
Daniel Powers
John Powers
Simon Powis
John Powles
Mary Pownall
Claire Poyart
D.R. Poyner
Mary Poynten
Jenny Poynter
Hannu Pöysä
Alex Pozhitkov
Haralampos Pozidis
Carlo Pozilli
Edoardo Pozio
Svetlana Poznanovic
Anton Pozniak
Daniel Pozza
Alberto Pozzebon
Bruno Pozzetto
Ambra Pozzi
Nicola Pozzi
Federico Pozzi
Paolo Pozzilli
Lucas Pozzo-Miller
Christian Pozzorini
Meredith Praamsma
Bellur Prabhakar
Rajeev Prabhakar
Chandra Prabhakar
Janani Prabhakar
K. Prabhu
K. Sandeep Prabhu
Varun Vijay Prabhu
Suijt Prabhu
Asmita Prabhune
Patrícia Prada
Carlos Prada
Joaquin Prada
Pierre-Francois Pradat
Wagle Pradeep
Appukuttan Pradeep
Rajendra Pradeepa
P.I. Pradeepkumar

Rebecca Pradeilles
Jean Philippe Pradere
Bérengère Pradet-Balade
Abani Pradhan
Jalandhar Pradhan
Gautam Pradhan
Florence Pradillon
Jerome Prado
V. Prado
Carla Prado
José Prado Martin
Jeppe Praetorius
Helle Praetorius
Mark Praetorius
Manuel Praga
Sean Prager
Katherine Prager
Franz Prager
Alexa Pragman
Melanie Prague
Sivaraman Prakasam
Y.S. Prakash
Siddharth Prakash
Prem Prakash
Gaurav Prakash
Megha Prakash Bangalore
Niki Prakoura
Vincent Praloran
Pairot Pramual
Francesca Prandi
Fabio Pranovi
C. Pranteda
Daniel Prantner
Catharina Praptiningsih
Hanumanthappa Prasad
Rajendra Prasad
Vibhore Prasad
Pottumarthi Prasad
Durbaka Vr Prasad
Manoj Prasad
Sahdeo Prasad
Kalika Prasad
Saurabh Prasad
Lakshman Prasad
Soumya Prasad
Rishi Prasad
Anamika Prasad
Krishna Prasad
Vivek Prasad
Ramesh Prasad
Ram Prasad
Ramakrishna Prasad
P. Prasad
Shreya Prasanna
L.C. Prasanna
S.P. Prasanna
Aleix Prat
Joan Prat
Maria Prat
Siddharth Pratap
Bhim Pratap
Jayant Pratap
Morgan Pratchett
Annamari Pratelli
Renato Prates
Ivan Prates
Randall Prather
Jonathan Prather
Aric Prather
Arun Pratihast
Christine Pratilas
Mauro Prato
Rosa Prato
Carlo Prato
Emanuela Prato-Previde
Clara Prats
Elena Prats
Harris Pratsinis
Anna Prats-Puig
Stephen Pratt
Wayne Pratt
Jessica Pratt
Kathleen Pratt
Daniel Pratt
Steven Pratt
Arthur Pratt
Christopher Pratt
Guillem Pratx
Jean-Paul Praud
Nicole Prause
Shelly Praveen
Michal Pravenec
Vladimir Pravosudov
Dirk Prawitt
Josef Prchal
Vesna Prchkovska

Damiano Preatoni
Ezio Preatoni
Matthew Prebus
József Prechl
Bill Precht
Jessica Preciado
Bogdan Preda
Irene Predazzi
Rui Prediger
David Preen
Ranjan Preet
Raman Preet
Federico Prefumo
Jochen Prehn
Christine Preibisch
Johannes Preiner
Tobias Preis
Christian Preisinger
Anette Preiss
David Preiss
Evan Preisser
Rytis Prekeris
Roman Prem
Elsie Premereur
Zul Premji
Isabella Premoli-Silva
Richard Premont
Garreth Prendergast
Brian Prendergast
Mark Prendergast
Andrew Prendergast
Luke Prendergast
Hans Prenen
Andrew Prentice
Michael Prentice
Stephen Prentice
Heather Prentice
Fabio Presaghi
Mark Prescott
John F. Prescott
Hallie Prescott
Natalie Prescott
Steven Presley
Gerry Presley
Clare Press
Frances Press
Daniel Pressnitzer
Cyrille Prestianni
Clive Prestidge
Ioana Preston
Kenzie Preston
Christopher Preston
Daniel Preston
Jonathan Preston
Nancy Preston
Etheresia Pretorius
Mias Pretorius
Paolo Pretto
Daniel Prevedello
Jayme Prevedello
Roberto Prevete
Stephen Previs
Charlotte Prevost
Francisco Prevosti
Vincent Prevot
D. Rebecca Prevots
Matthew Prewett
Nicolas Preyat
P. Prezerakos
John Priatel
Peter Pribis
Alena Pribyl
David Price
Matt Price
Theodore Price
Richard Price
Ric Price
Erin Price
Nicholas Price
Neil Price
Owen Price
Michael Price
Karen Price
Stephen Price
Melissa Price
Paul Price
Catherine Price
Jennifer C Price
Allan Price
Matthew Price
Rumi Price
Wayne Price
Joshua Price
Jeremy Prichard
Frances Priddy
David Pride

Dominique Prié
Stefan Priebe
Imants Priede
Reid Priedhorsky
Feliciano Priego-Capote
Avi Priel
Esther Priel
Axel Pries
Viola Priesemann
Naomi Priest
Dolores Prieto
Victor Prieto
Minolfa Prieto
Pilar Prieto
Albert Prieto Marquez
Alejandra Prieto-Davó
Angeles Prieto-Fernandez
Mario Prieto-Velasco
Xavier Prieur
Kostas Priftis
Claude Prigent
Sylvain Prigent
Claire Prigent-Combaret
Alessandro Prigione
Valeria Prigione
Boris Prilutsky
Charlotte Primard
Florian Primavesi
Stefany Primeaux
Massimo Primignani
Craig Primmer
Ana Ligia Primo
John Primrose
Alice Prince
Silvas Prince
Lawrence Prince
Jane Prince
Karine Princé
Mary Princip
Nicola Principi
Alessandro Prinetti
Elizabeth Pringle
Anne Pringle
Gail Prins
Theo Prins
Jan-Bas Prins
Els Prinsen
Marco Prinz
Christian Prinz
Immo Prinz
Andreas Prinzing
Suzette Priola
Philippe Prior
Lynda D. Prior
Anders Prior
Juergen Pripfl
Domenico Prisco
Kevin Prise
Michael Prislin
Catarina Prista
P. Pristas
Leighton Pritchard
Kirkwood Pritchard
Michele Pritchard
Rachel Pritchard
James Pritchett
Kathleen Pritchett-Corning
Martin Privalsky
Alain Privat
Smriti Priya
Padma Priyadarsini
U. Deva Priyakumar
Aashish Priye
Andreas Prlic
Lesley Probert
Alexander Probst
Christian Probst
Daniele Procissi
Darwin Prockop
Bogdan Procopet
Giuseppe Procopio
Richard Proctor
Darby Proctor
Carole Proctor
Heather Proctor
Michael Proctor
Helen Proctor
Emmanuel Procyk
Ric Procyshyn
Paulo Prodöhl
Roger Prodon
Simone Proemel
Vania Proenca
Diogo Proença
Catherine Proenza
Willem Proesmans

Antonios Proestakis
Paolo Profice
Jaime Prohens
Laszlo Prokai
Polina Prokopovich
Jeanine Prompers
Vasilis Promponas
Leanne Proops
Friedrich Propst
Ewgenij Proschak
Thomas Pröschold
Katarina Prosenc
Witold Stanislaw Proskura
Chiara Prosperetti
Simone Prospero
Sean Prosser
R. Scott Prosser
Audrey Prost
Feliciano Protasi
Natacha Protopopoff
Alexandra Protopopova
Dario Protti
Alessandro Protti
Ulrike Protzer
Judith Proudfoot
Christopher Proudman
Stéphanie Proulx
Jim Provan
Ignacio Provencio
José Provenzano
Paolo Provenzano
Mauro Provinciali
Federica Provini
Patrick Provost
Sylvain Provot
Nikola-Michael Prpic
Miguel Prudencio
Benjamin Prud'Homme
Joan Prudic
Jens Pruessner
Timothy Pruett
Christin Pruett
Menno Pruijm
Raimon Pruim
Jonathan Pruitt
Basil Pruitt
Steven Prus
Dov Prusky
Reeta Prusty Rao
Andrew Pruszynski
David Pruyne
Christopher Pryce
Anna Przekoracka-Krawczyk
Grabowicz Przemyslaw
Wojtek Przepiorka
Rachel Przeslawski
Jude Przyborski
Andrew Przybylski
Peter Psaltis
Theodora Psaltopoulou
Caterina Psarropoulou
Roland Psenner
Androniki Psifidi
Leszek Pstras
Mina Psychogiou
Lamprini Psychogiou
Dimitrios Psyrakis
Grazyna Ptak
Christopher P. Ptak
Maurice Ptito
Xiong-Ming Pu
Sheng-Yan Pu
Cunlai Pu
Xuemei Pu
Jeffrey Pu
Yong Hao Pua
Steve Publicover
Annibale Puca
Paolo Puccetti
Giacomo Pucci
Rosana Puccia
Aina Puce
Michel Puceat
Adam Puche
Philip Pucher
Eduardo Pucheta
Carlo Pucillo
Haridas Pudavar
Paolo Emilio Puddu
Raghavendra Pudupakam
Sébastien Puechmaille
Olivier Puel
Carlos Puente
Lus Puente-Maestu
Chloe Puett
Esther Pueyo

Elena Pugacheva
Subbiah Pugazhenthi
Anna Maria Puglia
Maura Pugliatti
Giuseppe Pugliese
Steven Pugliese
Livia Pugliese
Jo Puglisi
Orazio Puglisi
Francisco Ignacio Pugnaire
Pedro Puig
Manel Puig Domingo
Pere Puigdomenech
Xavier Puig-Montserrat
Giulia Puja
Sanjay Pujari
Flor Pujol
Jean-Louis Pujol
Benoit Pujol
Ricardo Pujol Borrell
Jose Martin Pujolar
Rüdiger Pukall
Budhan Pukazhenthi
Anderson Puker
Read Pukkila-Worley
Céline Pulcini
Bali Pulendran
Srinivas Puli
Oorvashi Roy Puli
Francisco Pulido
Vinesh Kumar Puliyappadamba
Jacob Puliyel
Phani Pullela
Nicholas Pullen
Wim Pullen
Eleanor Pullenayegum
Tonu Pullerits
Christopher Pulliam
Alessandra Pulliero
Helen Pullisaar
Geoffrey Pullum
Steven Pulos
Jerome Pulpytel
Dianne Pulte
Alfredo Pulvirenti
Michael J. Puma
René Pumain
Denise Pumain
Kate Pumpa
Sher Pun
Sachin Pundhir
Gabija Pundziute
Anna Punga
Somahekhar Punnuri
Andre Punt
Peter Punt
Filomena Puntillo
Rosalda Punturo
Chamindie Punyadeera
Claudio Punzo
Mirja Puolakkainen
Michelino Puopolo
Naveen Puppala
Francesca Puppo
Witoon Purahong
Swati Puranik
Sampada Puranik
Damian Purcell
Steven Purcell
Jeremy Purcell
Kevin Purdy
Pie Lorenzo Puri
Raj Puri
Sanjeev Puri
Puneet Puri
Ajit Puri
Nitin Puri
Dhivya J. Puri
Enroca Purisima
Jubilee Purkayastha
Zita Purkrtova
Devina Purmessur
Kasyap Purna
Rituraj Purohit
Zachary Pursell
Autun Purser
Harry Purser
Louise Purtell
Kelly Purtell
Dale Purves
Tertia Purves-Tyson
Preeti Purwaha
Endang Purwantini
Benjamin Purzycki
Snigdhasmrithi Pusalavidyasagar
Markus Puschenreiter

Bernd Puschner
Smruti Pushalkar
Yulia Pushkar
Sathnur Pushpakumar
Kaur Pushpinder
Marc Pusztaszeri
Chaturong Putaporntip
Nirupama Putcha
Pilaipan Puthavathana
Manojkumar Puthenveedu
Theresa Puthussery
Lorenza Putignani
Rory Putman
David Putnam
Shawn A. Putnam
Shan Putnam
James Putney
Diane Putnick
David Puts
Katrin Pütsep
Radhika Puttagunta
Puttaswamy Puttaswamy Manjunath
Nancy Puttkammer
Francis Putz
Felix Putze
Sasima Puwunan
Xuehua Puyang
Ana Puyo
Geoffrey Puzon
Béatrice Py
Elizabeth Pyatak
Prashant Pyati
Nadya Pyatigorskaya
Timothy Pychyl
Dohun Pyeon
Simon Pyke
Richard Pyle
Anna Pyle
April Pyle
Richard Pyles
Magdalini Pylli
Larysa Pylyp
Robert Pyron
Alexandre Pyrrho
Peter Pytel
Leah Pyter
Lisa Pytlikzillig
Jae-Chul Pyun
Sampo Pyysalo
Firdausi Qadri
Ishtiaq Qadri
Matin Qaim
Uzma Qaisar
Raheel Qamar
Arman Qamar
Khaled Qanud
Atif Qasim
Waseem Qasim
Muhammad Qasim
Amal Qattan
Robert Qi
Qingsheng. Qi
Jianxun Qi
Ruomei Qi
Xiaoquan Qi
Chao Qi
Yin Qi
Ruifeng Qi
Xin Qi
Yitao Qi
Dunwu Qi
Meng Qi
Yanbing Qi
Rongfeng Qi
Ji Qi
Sharon Qi
Wei Qi
Jiayin Qi
Xinshuai Qi
Jin Qi
Yang Qi
Wen-Hua Qi
Yingying Qi
Yuan-Yuan Qi
Yiping Qi
Fuqqiang Qi
Jifa Qi
Zhi Qi,
Hong Qian
Men-Bao Qian
Wei Qian
Cheng Qian
Shi Qian
Bingjun Qian
Yuan Qian
Chao-Nan Qian

Song Qian
Wenfeng Qian
Guoqing Qian
Li Qian
Xi Qian
Kun Qian
Hua Qian
Zhikang Qian
Guoliang Qian
Weijun Qian
Zhu Qian
Zhong Qian
Xiaoning Qian
Ziqing Qian
Xu Qian
Jingjing Qian
Feng Johnson Qian
Jinze Qian
Jianjun Qian
Mei Qiang
Fang Qiang
Ya-Wei Qiang
Tie Qiao
Li-Ya Qiao
Lei Qiao
Jun Qin
Xiang Qin
Ling Qin
Zhihai Qin
Zhao Qin
Gangjian Qin
Qiwei Qin
Hongwei Qin
Zhaohui Qin
Bolin Qin
Jian Qin
Boqiang Qin
Feng Qin
Shaozheng Qin
Cheng-Feng Qin
Zhangcai Qin
Lili Qin
Guozheng Qin
Zhiqiang Qin
Wen Qin
Yufang Qin
Liu Qin
Bo Qin
Xuebo Qin
Wei Qin
Dajun Qin
Shucun Qin
Xuebin Qin
Qiaoping Qin
Hong Qin
Yingying Qin
Jing Qin
Pengmin Qin
Yu Qin
Nannan Qin
Yu-Feng Qing
Kun Qing
Li Qingjie
Yin-Long Qiu
Yi Qiu
Jiang Qiu
Chunfang Qiu
Changjian Qiu
Fan Qiu
De You Qiu
Weiliang Qiu
Xiangyun Qiu
Xiangguo Qiu
Jian-Ding Qiu
Yingwei Qiu
Tian Qiu
Xiao Qiu
Bensheng Qiu
Liyan Qiu
Tao Qiu
Yong Qiu
Yang Qiu
Shanhu Qiu
Zhifang Qiu
Weibao Qiu
Deqiang Qiu
Fuming Qiu
Yafeng Qiu
Yan Qiu
Yongqiang Qiu
Yu Sheng Qiu
Gaofeng Qiu
Chao Qiu
Huan Qiu
Xin Qiu
Luo Qiulan

Zhou Qixing
Mostafa Qorbani
Hui-Qi Qu
Rongda Qu
Zhilin Qu
Yi Qu
Chaoling Qu
Mingjing Qu
Le Qing Qu
Zhe Qu
Feng Qu
Jin Qu
Lujiang Qu
Xiaobo Qu
Lianghuan Qu
Junfeng Qu
Xia Qu
Chen Qu
Joachim Quack
Mohammed Quader
Sadiqa Quadri
Joe Quadrilatero
Alberto Quaglia
Luca Quagliata
Lorenzo Quaglietta
Alison Qualtrough
Lei Quan
Ruidang Quan
Zhe-Xue Quan
Taihao Quan
Cheng Quan
Ming Quan
Xianyue Quan
Lijun Quan
Melvyn Quan
Philip Quanjer
Luciano Quaranta
C. Quarles
Angelo Quartarone
Rodolfo Quarto
Luca Quartuccio
Peter Quashie
Pascale Quatresooz
Mattia Quattrocelli
Walter Quattrociocchi
Aldo Quattrone
Paul Quax
Jianwen Que
Qiudeng Que
Piotr Quee
Lurdes Queimado
Felisbina Queiroga
Ana Queiros
Glória Queiroz
Bernardo Queiroz
Frederick Quelle
Annaïk Quémard
Siobhan Quenby
Delphine Quenet
Michaël Quentin
Troy Querec
Damien Querlioz
Pascal Querner
Jose Quero
Amparo Querol
Xavier Querol
Ivan Quesada
Antonio Quesada
Enrique Quesada-Moraga
Lina Quesada-Ocampo
Valerie Quesniaux
Andrew Quest
Erin Questad
Christophe Queval
Jorge Quevedo
Mario Quevedo
Sophie Quevillon-Cheruel
François Queyroi
Sergio Quezada
Sandra Quezada
Martha Quezado
Yongzhi Qui
Jim Quigley
Matthew Quigley
Pedro Quijón
María Quiles
Thibaut Quillard
Thelma Quince
André Quincozes-Santos
John Quindry
Muriel Quinet
Sylvie Quiniou
Elizabeth Quinlan
Roy Quinlan
Mark Quinlivan
Conrad Quinn

Frederick Quinn
Janet Quinn
Cheryl Quinn
Julian Quinn
Graham Quinn
T Alexander Quinn
Jennifer Quinn
Kimberly Quinn
Kylie Quinn
Gwendolyn Quinn
Shannon Quinn
Emma Quinn
Patrick Quinn
Ashlinn Quinn
Andrew Quinn
Rupert Quinnell
Ana Quinones
Beatriz Quiñones
Martha Quiñones
Miguel Quinones-Mateu
Carlos Quiñonez
Eva Quinque
Giuseppe Quintaliani
Francisco Quintana
Flavio Quintana
Jose Quintana
Daniel Quintana
S Quintana
Ester Quintana-Rizzo
Franciane Quintanilha Gallego
Cristina Quintavalle
Chantal Quinten
Lina Quintieri
Jessica Quintin
Loic Quinton
Federico Quinzi
Joselito P. Quirino
Lucia Rita Quitadamo
Alexandra Quittner
Robert Quivey
Fabio Quondamatteo
Sadeq Quraishi
Rahat Qureshi
Yvonne Qvarnstrom
Tavs Qvist
Nair R Harikumaran
Song-Gyu Ra
Markus Raab
Nicolas Raab
Dierk Raabe
Philip Raake
Sadaf Raana
Victoria Rabago
Christopher Rábago
Sarah Rabau
Gulam Rabbani
Naila Rabbani
R. Rabbitt
Christian Rabeling
Ton Rabelink
Ligia Rabello
Ananza Rabello
Carlos Rabelo
Jacob Raber
Thierry Rabilloud
Leonard Rabinow
Paul Rabinow
Joshua Rabinowitz
Alan Rabinowitz
Peter Rabinowitz
Felicia A. Rabito
Alison Rabosky
Peter Racay
Alessandra Raccichini
Susan Racette
Brad Racette
Lyne Racette
Satyanarayana Rachagani
P.Sivaramakrishna Rachakonda
Lyudmila Rachek
Reinhard Rachel
Milene Rachid
Howard Rachlin
Sarah Racine
Caroline Racine Belkoura
Gregory Raciti
Lorraine Racusen
Balazs Rada
Jody Rada
Zsolt Radak
Christian Radauer
A. Radbruch
Alexander Radbruch
Volodymyr Radchuk
Nathan Radcliffe
Noura Raddadi

Nicole Radde
Katherine Radek
Volker Radeloff
Jessica Rademacher
Aleksandra Radenovic
Christoph Rader
Janet Rader
Peter Radermacher
Sheena Radford
Craig Radford
Ian Radford
Prakash Radhakrishnan
Vinod Kumar Radhakrishnan
S.R. Radhika Rajasree
Marco Radi
Zoran Radic
Marko Radic
Filippo Radicchi
Kathryn Radigan
Madeleine Rådinger
Anita Radini
Milica Radisic
Vanja Radišic Biljak
Viviane Radl
Andrew Radley
Franz Radner
Tyler Radniecki
Justin Radolf
Julien Radoux
Jelena Radovanovic
Sally Radovick
Tamás Radovits
Markus Radsak
Joaquim Radua
Ernst Radue
Anca Radulescu
Ovidiu Radulescu
François Radvanyi
Jacek Radwan
Przemyslaw Radwanski
Robbie Rae
Dale Rae
Kevin Raehtz
Lori Raetzman
Pierre Raeven
Alex Rafacho
Jimenez-Flores Rafael
Jill Rafael-Fortney
Eshkol Rafaeli
Hershel Raff
Jennifer Raff
Amanda Raff
Ken Raffa
Tommaso Raffaello
Peter Raffalt
Stéphane Raffard
Thomas Raffay
Thomas Raffel
David Raffelt
John Rafferty
Janet Rafferty
Mohd Y. Rafii
Mohd Y. Rafii
Mohammed Rafii-Elidrissi Benhnia
Kazi Rafiq
Ismael Rafols
Natasha Rafter
Daniel Raftery
Rosanne Raftery
Vasilios Raftopoulos
Timothy Ragan
Arthur Ragauskas
Hermann Ragg
Lorenzo Raggi
Alberto Raggi
Jose Raggio
Gajendra Raghava
Sathees Raghavan
Malini Raghavan
Ram Raghavan
Sukanya Raghavan
Agapeti Raghavendra
Pongali Raghavendra
Krishnan Raghavendran
Kozhiparambil Gopalan Raghu
Sheshagiri Raghukumar
Manchala Raghunath
Vijay Krishna Raghunathan
Karthik Raghunathan
P. Raghupathy
Ramesh Raghupati
Kavarthapu Raghuveer
Jayna Raghwani
J. Ragland
Andre Ragnauth
Laura Ragona

Andreas Ragoschke-Schumm
Ioannis Ragoussis
Michael Ragozzino
Erik Ragsdale
Subramanyam Ragupathy
Jan Raguse
Robert Raguso
Jee Rah
Elias Rahal
Irfan Rahaman
Abdul Hakkim Rahamathullah
Afsar Rahbar
Nuh Rahbari
Frederic Rahbari-Oskoui
Yvan Rahbé
Javad Rahebi
Aneel Rahim
Farid Rahimi
Ossi Rahkonen
Brian Rahm
Anisur Rahman
Mahfuzar Rahman
Mosiur Rahman
Mustafizur Rahman
Ruman Rahman
Muhammad Aziz Rahman
Maryam Rahman
Mohammad Rahman
Habibur Rahman
Mohammed Rahmatullah
Anne Rahn
Dobromir Rahnev
Kaja Rahu
Teddie O. Rahube
Iyad Rahwan
Muhammad Farooq Rai
Rhitu Rai
Niraj Rai
Rajivnandan Rai
M.K. Rai
Lal Rai
Florian Raible
James Raich
Shane Raidal
Azad Raiesdana
Julia Raifman
Robert Raike
Gaurav Raikhy
Jens Raila
Adalbert Raimann
Kaitlin Raimi
Francesco Raimondi
Francesca Raimondi
Sara Raimondi
Stacey Raimondi
Domenico Raimondo
Stefania Raimondo
Ramesh Raina
Pascal Rainard
Timothy Rainer
Olivier Raineteau
Paul Rainey
Jan Rainey
Petrie M. Rainey
George Rainger
Glen Rains
Pierre Rainville
Sari Räisänen
Geoffrey Raisman
John Raison
Olli Raitakari
Martin Raithel
Neeraj Raizada
Srilakshmi Raj
Anand Raj
Kenneth Raj
S. K. Raj
Dominic Raj
Srikumar Raja
Edwin Raja
Huzefa Raja
Yusuf Rajabally
Mehdi Rajabi
Lakshmi Rajagopal
Raman Rajagopal
Govindarajan Rajagopalan
Kumaravel Rajakumar
Tuomas Rajala
Satish Rajamani
Govindan Rajamohan
Ramesh Rajan
Rakhi Rajan
Krishnamurthy Rajanikant
Venkatesh Rajapurohitam
Murugesan Rajaram
Narasimhan Rajaram

Krishna Rajarathnam
Julie Rajaratnam
Kanniah Rajasekaran
Namakkal-Soorappan Rajasekaran
Parthiban Rajasekaran
Kamalakannan Rajasekaran
Naren Rajasekaran
Johnson Rajasingh
Yogendra Rajawat
Istvan Rajcan
Mario Rajchenberg
Mangalathu Rajeevan
Singh Rajender
Jeyaprakash Rajendhran
Yashas Rajendra
Vazhaikkurichi Rajendran
Ganeshkumar Rajendran
P. Rajendran
Joseph Rajendran
Mythilipriya Rajendran
Jacob Rajfer
Mukaila Raji
Swaraj Rajkhowa
Vladislav Rajkovic
Luis Rajmil
Singh Rajni
Om Rajora
Shashi Rajput
Charu Rajput
Sandeep Rajput
Jyutika Rajwade
Allah Rakha
Gábor Rákhely
Vahid Rakhshan
Alexander Rakin
Miroslava Rakocevic
Hannes Rakoczy
Sam Rakover
Alexandra Rak-Raszewska
Mariann Rakszegi
Randeep Rakwal
Michael Raleigh
Glenn Rall
Katharina Rall
Stephen Ralph
Brandon Ralph
Markus Ralser
David Ralston
Arthur Ram
Dinesh Ram
Luis Rama
Nagaraja Reddy Rama Reddy
Vijay Ramachandani
Srinivasan Ramachandran
Dhanya Ramachandran
Shyam Ramachandran
Satya Krishna Ramachandran
Ranjani Ramachandran
Rithwik Ramachandran
Satish Ramachandrarao
Bojan Ramadanovic
Swetha Ramadesikan
Giuliano Ramadori
Andrew Ramage
Sudha Ramaiah
Wusirika Ramakrishna
Suresh Ramakrishna
Rohan Ramakrishna
Girija Ramakrishnan
Vijay Ramakrishnan
Chandra Ramakrishnan
Satish Ramalingam
Ayyalusamy Ramamoorthy
Mahesh Ramamoorthy
Sammanda Ramamoorthy
Chandra Ramamoorthy
Indira Raman
Rajiva Raman
Rajiv Raman
Venkata Ramana
Sasanka Ramanadham
Vikram Ramanarayanan
Murali Ramanathan
Arvind Ramanathan
Sheela Ramanathan
Rajesh Ramanathan
Rangasamy Ramanathan
Dhakshin Ramanathan
Arunas Ramanavicius
Sasirekha Ramani
Georgia Ramantani
Srinivasan Ramasamy
Yasodha Ramasamy
Ravichandran Ramasamy
Ranjan Ramasamy
Parthasarathy Ramaseshadri

Anand Ramasubramanian
Mani Ramaswami
Girish Ramaswamy
Stefano Ramat
Alessandro Rambaldi
Daniela Rambaldini
Clotilde Rambaud Althaus
Robert Rambo
Rajeev Ramchandran
Sahienshadebie Ramdas
Tennore Ramesh
Mathan Ramesh
N. Ramesh
Divya Ramesh
Kirti Ramesh
Ganesan Ramesh
Gopal Ramesh Kumar
Mohammad Ramezani
Michael Ramharter
M. S. Rami´Rez
Mirana Ramialison
Mário Ramirez
José Luis Ramirez
Servio Ramirez
Darioc. Ramirez
Juan David Ramirez
Paula Ramirez
Raul Ramirez
Kelly Ramirez
Maria Laura Ramirez
Desmond Ramirez
Álvaro Ramírez
Victoria Ramírez
Rafael Ramirez Morales
Patricio Ramírez-Correa
Elena Ramirez-Parra
Alfredo Ramírez-Reveco
Mauricio Ramirez-Rodriguez
Ricardo Ramirez-Romero
Hugo Ramirez-Saad
Ramiro Ramirez-Solis
Hugo Ramírez-Tobías
Jose Ramirez-Valiente
Gita Ramjee
Vickram Ramkumar
Nirupama Ramkumar
Hema Ramkumar
Rahizar Ramli
Steven Ramm
Stefan Rammelt
Raina Ramnath
Doreen Ramogola-Masire
Meike Ramon
Eva Ramon
María Ángeles Ramón Jerónimo
Raül Ramos
Celso Ramos
José Ramos
Marcos Ramos
Sonia Ramos
Alexandre Ramos
Yolande Ramos
Clécio Souza Ramos
Paula Ramos
Flavio Ramos
Carlos Ramos
Irma Ramos
Hugo Ramos
Gustavo Ramos
Maria João Ramos Pereira
Mikael Ramos-Casals
Gabriel Ramos-Fernandez
Francisco Ramos-Morales
Yuval Ramot
Daniel Ramp
Angela Rampa
Elena Rampanelli
Raja Rampersaud
Ermanno Rampinini
Giordano Rampioni
Cristof Rampitsch
M. Rampling
Luca Rampoldi
Heribert Ramroth
Jennifer Ramsay
Kyle Ramsey
Mike Ramsey
Matthew Ramsey
Tod Ramsfield
Salah Ramtani
Pradeep Ramulu
Sophia Ran
Chongzhao Ran
Yong Ran
Basabi Rana
Majeed Rana

Ujala Rana
Neha Rana
Surinder Rana
Paola Mv Rancoita
Derrick Rancourt
Raymond Rancourt
David Rand
Tatyana Rand
Kasper Rand
Richard Randall
Cameron Randall
Carly Randall
Elissa Randall
Paul Randazzo
Antonio Randazzo
Rebecca Randell
Winfried Randerath
Harpal Randeva
Parmjeet Randhawa
Haseeb Randhawa Randhawa
Christophe Randin
Tara Randis
Bharat Randive
Melanie Randle
Gianpaolo Rando
Timothy Randolph
Theodore Randolph
Gregory Randolph
John Randolph
Marie Randoux
Rindra Randremanana
Sushil Rane
Petter Ranefall
Kevin Raney
Gary Raney
Molebogeng Rangaka
Padmini Rangamani
Anna Rangan
Venkatesh Rangarajan
Srikant Rangaraju
Sampathkumar Rangasamy
Ryan Range
Elizabeth Rangel
Drauzio Rangel
Juliana Rangel
Maria Rangel
Claudia Rangel-Escareño
Héctor Rangel-Villalobos
Andrea Ranghino
Mario Rango
Erik Ranheim
Vibha Rani
Marco Ranieri
Elena Ranieri
Girolamo Ranieri
Ravi Ranjan
Aashish Ranjan
Rakesh Ranjan
Louis Ranjard
Nalini Ranjit
Roger Rank
Melanie Rank
Sara Rankin
Erin Rankin
Kenneth Rankin
Kristiina Rannikmae
Brigitte Ranque
Emma Ransome
Hilary Ranson
Jonas Ranstam
Tomi Rantamäki
Barbara Rantner
Kalliopi Rantsiou
Marco Ranucci
Otavio Ranzani
Andrea Ranzi
Xiancai Rao
Venigalla Rao
Rajini Rao
Balaji Rao
Li-Lin Rao
Hai Rao
Chinthalapally Rao
Simita Rao
Vivek Rao
Arvind Rao
Guillaume Rao
Sheng-Xiang Rao
Radhakrishna Rao
Jaladanki Rao
Koteswara Rao
H. Raghav Rao
Dhananjai Rao
S.S. Rao
Vadlamudi Raghavendra Rao
Panduranga Rao

Christopher Rao
Rajesh Rao
Desirazu Rao
Govind Rao
Xiang-Jun Rao
Uma Rao
Vassili Raos
David Raper
Yehoash Raphael
William Raphael
David Rapoport
Fábio Raposo Do Amaral
Kilian Rapp
Anna Rapp
Francesca Rappa
Jay Rappaport
Noa Rappaport
Wouter-Jan Rappel
Chad Rappleye
Rino Rappuoli
Kristina Rapuano
Kevin Rarick
Katja Räsänen
Troy Rasbury
Björn Rasch
Volker Rasche
Ericka Rascon
M. Rasenick
Hector Rasgado-Flores
Natalie Rasgon
Carla Rash
Asia Rashed
Khaled Rasheed
Muhibur Rasheed
M.K. Rasheeda
Asif Rashid
Barnaly Rashid
Mohammad Mehdi Rashidi
Mahnaz Rashidi
Jean-Philippe Rasigade
Mladen-Roko Rasin
Mathias Rask-Andersen
Johannes J Rasker
Christian Rask-Madsen
David Rasko
Sergio Rasmann
Theodore Rasmussen
Magnus Rasmussen
Line Rasmussen
David Rasmussen
Andrew Rasmussen
Finn Rasmussen
Richard Rasmussen
Keith Rasmussen
Heather Rasmussen
Susan Rasmussen
Dennis Rasmussen
Soren Rasmussen
Tim Rasmussen
Zeba Rasmussen
Randall Rasmusson
Mark Rasnake
Ghulam Rasool
Mario Raspanti
Marco Rasponi
Tienush Rassaf
Francois Rassendren
Jens Rassweiler
Jonathan Rast
Mojgan Rastegar
Sepand Rastegar
Rita Rasteiro
Giulio Rastelli
Alessandra Rastelli
Ashutosh Rastogi
Gurdeep Rastogi
Linda Rasubala
Norman Ratcliffe
Timo Rath
Narayan Rath
N. Rath
Gayatri Rath
Daniel Rathbun
Phil Rather
Yogesh Rathi
Bala Rathinasabapathi
Thenmalarchelvi Rathinavelan
Fabian Rathke
Michael Rathleff
Keerti Rathore
Anurag Rathore
Felix Ratjen
John Ratliff
Oliver Ratmann
Wickneswari Ratnam
Milind Ratnaparkhe

Rinki Ratnapriya
Lee Ratner
Nan Bernstein Ratner
Catarina Rato
Edward Ratovitski
A. Ratsch
Jonah Ratsimbazafy
Carlo Ratti
Antonia Ratti
Pietro-Luca Ratti
Barnett Rattner
Amir Rattner
Alexander Ratushny
Franz Ratzinger
Domenico Rau
Raghavendra Rau
Kristofer Rau
Christopher Raub
Andri Rauch
Alexander Rauch
Marjatta Raudaskoski
Terje Raudsepp
Diana Raufelder
Daniel Rauh
Michael Rauh
Frédéric Raulier
Martina Rauner
Bent Raungaard
Navin Rauniyar
Michael Raupach
Michael Rauscher
Estrella Rausell
Mark Rausher
Ashwin Raut
Stefan Rautenbach
Silke Rautenschlein
Georg Rauter
Susanne Rautiainen
Milla Rautio
Paul Rauwolf
Saaeha Rauz
Matteo Ravaioli
Siamak Ravanbakhsh
Lara Ravanetti
Viola Ravasio
Giuliano Ravasio
Vineesh Raveendran
Anthony Ravel
Michel Ravelonandro
John Raven
Jose Raventos
Nuria Raventos
Yazhini Ravi
Kodi Ravichandran
Andrea Ravignani
Sowmya Ravikumar
Thulasiraj Ravilla
Rahul Ravilla
Vijayalakshmi Ravindranath
Kundapura Ravishankar
Sadhana Ravishankar
Alfredo Ravizza
Maria Ravo
Jorgen Ravoet
Matthew Ravosa
Bhupendra Rawal
Shruti Rawal
Lal Rawal
Oliver Rawashdeh
Rahul Rawat
Nidhi Rawat
Siddhartha Rawat
Harshadrai Rawel
Kerri Rawson
Patrick Rawstorne
Krishanu Ray
Pradipta Ray
David Ray
Stuart Ray
Pierre Ray
Monika Ray
Sidhartha Ray
Supratim Ray
Justina Ray
Pritha Ray
Deepak Ray
Partho Sarothi Ray
Debolina Ray
Patricio Ray
Devin Ray
Alpana Ray
Lane Rayburn
Siba Raychaudhuri
Syamal Raychaudhuri
Emily Rayfield
Graydon Raymer

Vincent Raymond
Jane Raymond
Elizabeth Raymond
Lynn Raymond
Melissa Raymond
Jason Raymond
Patrick Raynal
Jean-Philippe Raynaud
Julian Rayner
Brian Rayner
Katey Rayner
Hugh Rayner
Theo Raynor
Hollie Raynor
Edward Raynor
Catherine Rayon
Holly Rayson
Gary Rayson
Assaf Raz
Shaan Raza
Mahdi Razafsha
Karina Razali
Raimundas Ražanskas
Shahnaz Razavi
Joseph Razik
Sergey Razin
Olga Razorenova
Y. Razvodovsky
Fabio Re
Daniel Re
Mark Rea
Elizabeth Rea
Ilaria Rea
Andrew Read
Laurie Read
Jenny Read
Tim Read
Scott Read
Clare Reade
Arran Reader
Jonathan Ready
Michaela Reagan
John Reager
Joseph Reagle
Marjorie Reaka
Maria Dolores Real
Luis Real
Francisco Real
Eusebio Real
Lara Reale
Walt Ream
Joseph Reardon
Melissa Reardon-Robinson
Drury Reavill
Krzysztof Rebala
Ana Rebane
Amanda Rebar
Abdelhadi Rebbaa
Robyn Rebbeck
Richardson Rebecca
Clark Rebecca
Boddicker Rebecca
Vito Rebecca
Mario Rebecchi
Joseph Rebehmed
Johanna Rebel
Mauro Rebelo
Rui Rebelo
Ana Rebelo
Rolf Reber
Gabriel Rebick
Maija Reblin
Eduardo Rebollar
Rolando Rebolledo
M.C. Rebolledo
Angelita Rebollo
Breno Reboucas
Julien Reboud
David Reboutier
Ginger Rebstock
David Reby
Mariana Recamonde Mendoza
Fabio Recchia
Maria Cristina Recchioni
Veronique Receveur-Brechot
Dino Rech
Megan Rech
Jason Rech
Wolfgang Recheis
Félix Recillas-Targa
Ma Carmen Recio
Martin Reck
Jane Reckelhoff
Mario Recker
Ana Recober
Alan Rector

Sergio Recuenco
Richard Redak
Sam Reddington
Hudson Reddon
Sakamuri V. Reddy
Geereddy Bhanuprakash Reddy
Hemachandra Reddy
Sanjay Reddy
Malireddy Reddy
Timothy Reddy
Sekhar Reddy
Narsa Reddy
Nageshwar Reddy
Bindu Madhava Reddy
Elizabeth Reddy
Priscilla Reddy
Madhavi Reddy
Pavankumar Reddy
Arubala Reddy
Samba Reddy
Vasudha Reddy
Vasudevi Reddy
Umesh Reddy
Jagedeshwar Reddy
Benjaram Reddy
Nancy Redeker
John Redell
Michele Redell
Gil Redelman-Sidi
Mathieu Rederstorff
Simon Redfern
R. Redfield
Bjorn Redfors
Jessica Redgrave
Thomas Redick
Roxana Redis
Ronny Redlich
Joy Redman
T. Michael Redmond
Josep Redon
Carmen Redondo
Maria Redondo
Simon Redwood
Zoran Redzic
Rick Ree
Carol Reeb
E. Albert Reece
James Reecy
Danielle Reed
Douglas Reed
Robert Reed
Rolf Reed
Kent Reed
Sarah Reed
Damon Reed
Phil Reed
Derek Reed
Floyd Reed
Andrew Reed
Catherine Reed
Miranda Reed
Brandon Reeder
Clare Reeder
Joanne Reekie
Buket Reel
Robert Rees
Sian Rees
Paul Rees
Kate Rees
William D Rees
James Rees
Benjamin Reese
Peter Reese
Michael Reese
Gerhard Reese
Torsten Reese
Jennifer Reeve
Johnmarshall Reeve
Peter Reeves
W. Brian Reeves
Stuart Reeves
Stephen Reeves
Andrew Reeves
R. Keith Reeves
Roger Reeves
Tamer Refaat
Roberto Refinetti
P. Refolo
Matthieu Réfrégiers
Kent Refsal
Nicolas Regamey
David Regan
Markus Regauer
Birgitte Regenberg
Christian Regenbrecht
Luciana Regitano

Luca Regli
Pramod Regmi
Binod Regmi
Veronique Regnault
Kevin Regner
A. Rego
Eduardo Rego
Ryan Rego
Giuseppe Regolisti
Juan Reguera
Senda Reguera
Virender Rehan
Sophie Rehault-Godbert
Vincent Rehder
Eva Rehfuess
David Rehkopf
Thomas Rehle
Markus Rehm
Ateequr Rehman
Holger Rehmann
Thomas Reiberger
David Reich
Adam Reich
Heather Reich
Stephen Reich
Holger Reichardt
Ina Reiche
Janine Reiche
Jürgen Reichenbach
Michael Reichenheim
Andreas Reichert
Brian Reichert
Florian Reichmann
Colleen Reichmuth
Jörg Reichrath
Gregor Reid
Ian Reid
Tony Reid
Steven Reid
Chris Reid
Marvin Reid
Russell Reid
Kathryn Reid
Gail Reid
Sean Reid
Andrew Reid
Paul Reier
Jaques Reifman
Carla Reigada
Heide Reil
Jan-Christian Reil
Norbert Reiling
Steve Reilly
Peter Reilly
Michelle Reilly
Christopher Reilly
Colin Reily
Frank Reimann
Thomas Reimchen
Raylene Reimer
James Reimer
Paula Reimer
Michell Reimer
Stian Reimers
Jeffrey Reimers
Arne Reimers
Laura Reimers Ladeluca
Alan Rein
Richard Reina
Ester Reina-Romo
Jeff Reinbolt
Joerg Reinders
Marion Reindl
Thomas Reinecke
Andreas Reinecke
David Reiner
Robert Reiner
Gesine Reinert
Eyal Reingold
Jurgen Reingruber
Karl Reinhard
Chris Reinhard
Klaus Reinhardt
Didier Reinhardt
Timothy Reinhardt
E. Reinhardt
Richard Reinhardt
Jan Reinhardt
Todd Reinhart
Kurt Reinhart
Cynthia Reinhart-King
Finn Reinholt
Ulrich Reininghaus
Lovisa Reinius
Hans Reinke
Lester Reinke

Andrew Reinmann
Peter Reinthal
Lufs Reis
Celso Reis
Alexandre Reis
Ricardo Reis
Mitermayer Reis
Joice Reis
Flavio Reis
Rui Reis
Bernardo Reis
Leonardo Reis
Pedro Reis
Roberto Reis
Joice Neves Reis
Aramys Reis
Rosana Reis
William Reisen
Georg Reiser
Jochen Reiser
Ingrid Reiser
Parham Reisi
Dominic Reisig
Ricardo Reisin
Michael Reiskind
David Reisman
Julia Reiss
Lawrence Reiter
Karl Reiter
Maarten Reith
Marc Reitman
Marcel Rejmanek
Jose Del Carmen Rejon-Orantes
Islem Rekik
Ole Peter Rekvig
Ole Rekvig
Mohamed Rela
Piotr Religa
Rajko Reljic
Norman Relkin
Mary Relling
Christian Rellstab
Angela Relógio
Claire Remacle
Alan Remaley
K. C. Remant
Edmond Remarque
Anthony Remaud
Mats Remberger
Christopher Rembold
Silje Reme
Marlene Remely
Anne Remes
Daniel Remick
Ra Remigio-Baker
Melissa Remis
Marc Remke
Henriette Remmer
Noelle Remoué
Giuseppe Remuzzi
Andrea Remuzzi
Elisabeth Remy
Jun Ren
Dacheng Ren
Gang Ren
Yuhong Ren
Zhong Ren
Jian Ren
Pengyu Ren
Hai Ren
Huiying Ren
Shuyu Ren
Shuxin Ren
Quan Ren
Yi Ren
Fei Ren
Tao Ren
Mingwu Ren
Fazheng Ren
Xiang Ren
Long Ren
Ren Ren
Guoyu Ren
Shen Ren
Jinchang Ren
Lili Ren
Yonglin Ren
Ting-Ting Ren
Kate Ren
Hongjun Ren
Jinfeng Ren
Zhuoming Ren
Jianfeng Ren
Jiangong Ren
T.T. Ren
Changhong Ren

Jiaoyan Ren
Yingxue Ren
Hongqi Ren
Haiyan Ren
Ze-Fang Ren
Shifang Ren
Zong-Xin Ren
Zhenhua Ren
Laurence Renard-Schild
Paul Renaud
Sabrina Renaud
Stephen Renaud
Michael Renaud
Paul Renauer
Elizabeth Rendina-Ruedy
Justin Rendleman
Katy Renfro
Aravind Kumar Rengan
Deivendran Rengaraj
Jyothi Rengarajan
Kannan Rr Rengasamy
Katrien Rengerink
Gianluigi Reni
Laurent Rénia
Alessandra Renieri
F. Reniers
Ortwin Renn
Elisha Renne
Thomas Renné
Jennifer Rennels
Susanne Renner
Bertold Renner
Marianne Renner
Swen Renner
Christoph Renner
Robert Rennie
Heidi Renninger
François Renoz
M. Rensel
Patrick Rensen
Christopher Rensing
Sa Rensing
Adam Renslo
Erik Renstrom
Frida Renstrom
Robert Renthal
William Renthal
Cyrill Rentsch
Gourapura Renukaradhya
Hanna Renvall
Bill Renwick
Julie Renwick
Neil Renwick
Jean-Luc Reny
Karen Renzaglia
Anastasia Renzi
Tiziana Renzi
Elizabeth Repasky
Maria Repolles
Tiina Reponen
Jennifer Requejo
Jose Maria Requejo-Isidro
Natalia Requena
Juan Requena-Mullor
Lucio Requião-Moura
Grégory Resch
Jacob Resch
Ursula Rescher
Matthieu Resche-Rigon
Maria Rescigno
Renato Resende
Angela Resende
Yakir Reshef
April Reside
Michael Resl
Fredrik Resman
Andrew Resnick
James Resnick
David Resnik
Gesine Respondek
Kerry Ressler
Habtom Ressom
Olivier Restif
Silvia Restrepo
Blanca Restrepo
Juan Restrepo
Domenico Restuccia
Joseph D. Restuccia
Gregory J. Retallack
Patricio Retamal
Arthur Retnakaran
Leslie Retnam
Scott Retterer
Mallik Rettiganti
Ruben Retuerto
Johanna Reudler Talsma

Karen Reue
Matthias Reumann
Gislaine Réus
Thorsten Reusch
Sebastian Reuter
Björn Reuter
Werner Reutter
Nina Reuven
Boris Reva
Gunturu Revathi
Ariel Revel
Florent Revel
Peter Revell
William Revelle
Massimo Reverberi
David Reverter
Benjamin Reves
Dora Revesz
Crawford Revie
Michael Revitt
Tomasz Rewicz
Tonia Rex
Brent Rexer
Gregoire Rey
Guillaume Rey
Martial Rey
Corsino Rey
Olivier Rey
Federico Rey
Pedro Rey
Andres Camilo Rey Sanchez
Pedro Rey-Biel
Christopher Reyer
Jose Reyes
Victor Reyes
Bernardo Reyes
Elija Reyes
Héctor Reyes Bonilla
Rodrigo Reyes Lamothe
Marjorie Reyes-Díaz
Victoria Reyes-García
Carlos Reyes-Moreno
Elsa Reyes-Reyes
Arturo Reyes-Sandoval
Horacio Reyes-Vivas
Gabriel Reygondeau
Philippe Reymond
Nicky Reynaert
Luz Reynales-Shigematsu
Olivier Reynard
Anna Reyners
Harmen Reyngoudt
Chandra Reynolds
Todd Reynolds
Don Reynolds
Richard Reynolds
Julian Reynolds
Gavin Reynolds
Clare Reynolds
Harmony Reynolds
Joseph Reynolds
Charles Reynolds
Joanna Reynolds
Olivia Reynolds
Peggy Reynolds
Julie Reynolds
James Reynolds
Paula Reynolds
Mark Reynolds
Paul Reynolds
Andrew Reynolds
Bert Reynvoet
Ana Rey-Rico
Mandana Rezaei
Farhad Rezaei
Nima Rezaei
Iman Rezaeian
Gustavo Rezende
Leandro Rezende
Enrico Rezende
Flavia Rezende
Christopher Reznich
Sergey Reznik
Marina Reznik
Andrew Rezvani
Amelie Rezza
Rita Rezzani
Koon Ho Rha
Nour-Eddine Rhaleb
Christopher Rhea
Soo-Yon Rhee
Dong-Kwon Rhee
Kunsoo Rhee
Kyu Rhee
Sung-Keun Rhee
Eugene Rhee

Handoo Rhee
Sylvain Rheims
Cosima Rhein
Maikel Rheinstadter
Mijke Rhemtulla
Mikael Rhen
Jennie Rheuban
Allison Rhines
Mina Rho
Gyu-Jin Rho
Seungmin Rho
Jaerang Rho
Harmony Rhoades
Jon Marc Rhoads
Douglas Rhoads
Robert Rhoads
Dale Rhoda
Jonathan Rhodes
Olin Rhodes
Andrew Rhodes
Theo Rhodes
Andrew Rhyne
Dario Riascos-Bernal
Andri Riau
Summaira Riaz
Sheila Riazi
S. Amer Riazuddin
Laia Ribas
Judit Ribas
Marta Ribases
Patricia Ribaud
David Ribble
Karen Ribbons
José Ribeiro
Antonio Ribeiro
Andre Ribeiro
Paula Ribeiro
Bergmann Ribeiro
Carlos Ribeiro
Ana Isabel Ribeiro
Dimas Ribeiro
Cristiéle Ribeiro
Sabina Ribeiro
Martha Ribeiro
Rafael Ribeiro
Natasha Ribeiro
Fabiano Ribeiro
Admilson Ribeiro
Pedro Ribeiro
Fabiana Ribeiro
Karina Ribeiro
Fernando Ribeiro
Guilherme Ribeiro
Maria Cristina Ribeiro De Castro
Maria Ribeiro Vieira
Anderson Ribeiro-Carvalho
Alfredo Ribeiro-Da-Silva
Aida Ribera
Pedro Ribera
Maurizio Ribera D'Alcala
Christian Riberholt
Werner Ribitsch
Julie Ribot
François Ric
Stefano Ricagno
Jean-Damien Ricard
Daniel Ricard
Ana Ricardo
Laiss Ricardo
Ezio Ricca
Valdo Ricca
Giovanna Riccardi
Gabriele Riccardi
Nicoletta Riccardi
Vincent M. Riccardi
Roberta Riccelli
Fulvio Ricceri
Paolo Ricchi
Zaccaria Ricci
Giulia Ricci
Irene Ricci
Beatrice Ricci
Raffaela Ricci
Giovanna Ricci
Stefano Ricci
Lila Ricci
Carmela Ricciardelli
Fabrio Ricciardolo
Andrea Ricci-Maccarini
Charles Rice
Scott Rice
Robert Rice
Kelly Rice
David Rice
Danielle Rice
Benjamin Rice

Lyndi Rice
Kenneth Rice
Simon Rice
Jeremy Rich
Thomas Rich
Josiah Rich
Peter Rich
Vincent Richard
Peter Richard
Denis Richard
Mathilde Richard
Freddie-Jeanne Richard
Hillary Richard
Gerkin Richard
Patrick Richard
Tom Richard
John Richard
Marion Richard
Florence Richard
Dave Richard
Vaea Richard
Aline Richard
Martin Richards
Corinne Richards
Jeffrey Richards
Christopher Richards
J. Brent Richards
Carl Richards
Paul Richards
Catherine Richards
Jack Richards
Adam Richards
Jim Richards
Peter Richards
Winston Richards
Anne Richards
Jerry Richards
Rose Richards
J.R. Richards
Sean Richards
W Richards
Rickelle Richards
Jennifer Richards
Kathryn Richards-Hrdlicka
Rebecca Richards-Kortum
Michael Richardson
Dave Richardson
Samantha Richardson
Claire Richardson
Daniel Richardson
Kris Richardson
Heather Richardson
Mark Richardson
Stephen Richardson
Paul Richardson
Sarah Richardson
Des Richardson
Jason Richardson
Ann Richardson
Laurie Richardson
Peter Richardson
Rodney Richardson
Philip Richardson
Philippe Richebe
David Richens
Martin Richer
Jennifer Richeson
John Richey
Ashutosh Richhariya
Thomas Richie
Jerome Richie
Fabio Richlan
Joy Richman
David Richman
Ann Richmond
Brian Richmond
Helen Richmond
Juergen Richt
Linda Richter
Claus-Peter Richter
Alex Richter
Elihu D Richter
Kai-Florian Richter
Ronny Richter
Alexander Rickard
John Rickard
Alissa Rickborn
Christopher Ricketts
Mariana Rickmann
Jenna Rickus
Beatriz Rico
Iván Rico
Mario Rico
Camillo Ricordi
Carlo Ricotta
Calogero Ricotta

Emily Ricotta
Vanessa Ridaura
Valéry Ridde
Valery Ridde
Gregory Riddick
Mark Riddle
Ryan Riddle
Daniel Riddle
Aline Rideau Batista Novais
Mark Rider
Perry Ridge
Karen Ridge
John Ridge
Neale Ridgway
Hayley Ridgway
Tom Ridler
Lisa Ridnour
Lorenzo Ridola
Julia F. Ridpath
Katharina Riebel
Robert Rieben
Nikolaus Rieber
Dean Riechers
Matthias Rieckher
Thomas Ried
Klaus Riede
Tobias Riede
Thomas Riedel
Marc Riedel
Christian Riedel
Claudia Riedel
Ch Riedel
Steffi Riedel-Heller
Michael Rieder
Anita Rieder
Ingo Riederer
John Rieffel
Siegbert Rieg
Timo Rieg
Heiko Rieger
Gerulf Rieger
Bernd Rieger
Johannes Rieger
Sandra Rieger
Markus Riegler
Simone Riehl
Kimberly Riehle
Kira Riehm
Wayne Riekhof
Tapani Riekki
Lasse Riemann
Angelika Riemer
Stefanie Riemer
Matthias Riepe
Jorge Riera
Marta Riera
Maria Riera
A. Riera-Mestre
Jonas Ries
Jana Ries
Kristian Riesbeck
Alison Rieser
Lynne Rieske-Kinney
Olaf Riess
Thomas Riess
Matthias Riess
Markus Riessland
Scott Riester
Jens Rietdorf
Kees Rietmeijer
Arne Rietsch
Ernst Rietzschel
Adrien Rieux
Mahfuza Rifat
Dalin Rifat
Brittany Rife
Sabine Riffault
Jeff Riffel
Jeffrey Riffell
Helena Riffkin
Scott Rifkin
François Rigal
Francois Rigal
Stamatis Rigas
Silvia Rigato
Marcello Riggio
Blake Riggs
Nathaniel Riggs
Francesca Righetti
David Righton
David Riglar
Isidore Rigoutsos
Lionel Rigoux
Coen Rigtering
Ali Rihani
Zoltan Rihmer

Hans Ulrik Riisgard
Emile Rijcken
Dick Rijken
Huub Rijnaarts
Rv Rikard
Wavne Rikkers
Jouko Rikkinen
Thomas Riley
Edward Riley
Michael Riley
Sean Riley
Anne Riley
David Riley
Tracy Riley
James Rilling
Bertus Rima
Gerald Rimbach
Rebecca Rimbach
Mendell Rimer
Frederic Rimet
Joseph Rimland
Guus Rimmelzwaan
Lars M. Rimol
Arja Rimpela
Espen Rimstad
Barbara Rinaldi
Carlos Rinaldi
Andrea Rinaldo
Gonzalo Rincon
Diego Rincon-Limas
Eli Rinde
Thomas Rinderer
Heiner Rindermann
Laura Rindi
Francois Rineau
Claire Rinehart
Andrei Rineiski
Matthew Rinella
Daniel Rinella
Tamar Ringel-Kulka
Adrian Ringelstein
Steffen Ringgaard
Matthew Ringuette
Maria Ringvall
David Rinker
Frank Rinkevich
Petteri Rinne
Luke Rinne
Markus Rinschen
John Rinzel
Natalia Riobo
Joëlle Riond
Philip Riordan
Rodrigo Rios
Gabino Ríos
Emmanuel Rios Velazquez
Luis Daniel Ríos-Barrera
Rafael Riosmena-Rodriguez
Catherine Riou
Julien Riou
Giancarlo Ripabelli
Ugo Ripamonti
Maria Giovanna Riparbelli
Andrea Ripoli
Pablo Ripolles
Bengt Rippe
Jnrgen Ripperger
Julie Ripplinger
Shannon Risacher
Makarand Risbud
Denise Risch
Martin Risch
David Risco
Patrizia Rise
Nils Risgaard-Petersen
Kathryn Risher
Arun Rishi
Sebastian Risi
Mauro Risio
Kimberly Risma
Theo Rispens
Sarah Risse
Chris Rissel
Howard Rissen
Olivia Rissland
Emilie Rissman
Adena Rissman
Valeria Risso
Manuela Rist
G. Ristagno
Sandra Ristori
Viviana Ritacco
Mark Ritchie
Stephen Ritchie
Stuart Ritchie
Tiarney Ritchwood

Nicolas Riteau
Lauren Riters
Koert Ritmeijer
Teresa Rito
Andreas Ritsch
Lucas Ritschl
Chadwick Rittenhouse
Tracy Rittenhouse
Sylvia Ritter
Uwe Ritter
Gerd Ritter
Laure Rittié
Simon Rittmann
Daniel Rittschof
Clare Rittschof
Thomas Ritz
Markus Ritz
Roy Ritzmann
Ramona Ritzmann
Giuseppe Riva
Silvia Riva
Casto Rivadulla
Alain Rivard
Valeria Rivarola
Donato Rivas
M. Fernanda Rivas
Albert Rivas-Ubach
D.F. Riveiro
Francisco Rivera
Ajna Rivera
Gabriel Rivera
Jesus Rivera
Maria Rivera
Maite Rivera Gorrin
Jesus Rivera-Nieves
Crisalejandra Rivera-Perez
Francisco Rivero
Brian Rivers
Jack Rivers-Auty
Emily Rivest
Elisabeth Riviello
Michel Riviere
Guillaume Riviere
Rafael Rivilla
Eleonor Rivindelcampo
Laura Rivino
Carlo Rivolta
Inger Maren Rivrud
Margarit Rizea
Rahim Rizi
Nabil Rizk
Salwa Rizkalla
Jacques Rizkallah
Dimitris Rizos
Raheela Rizvi
Sumera Rizvi
Wasia Rizwani
Federica Rizzi
Menico Rizzi
Marco Rizzi
Manuela Rizzi
Victor Rizzo
Manfredi Rizzo
Giovanni Rizzo
Renata Rizzo
Maria Giulia Rizzo
Claudia Rizzo
Elizete Rizzo
Michael Rizzo
Gabrielle Rizzuto
Daekyun Ro
Eunyoe Ro
Juan Roa
Rob Roach
Robert Roach
Vanessa Roach
Mack Roach
Eric Roalson
Timberley Roane
Brendan Roark
Nikos Robakis
Shannon Robalino
Bernard Robaye
Claudio Robazza
Frank Robb
David Robbe
Suelee Robbe-Austerman
Catherine Robbe-Masselot,
Melissa Robbiani
Paul Robbins
Hilary Robbins
Gwen Robbins Schug
Stanley Robboy
Bruno Robbs
Michael Robek
Carlos Robello

Horst Robenek
Eyal Robenshtok
Allison Rober
Debi Roberson
Paula Roberson
Annie Robert
Kylie Robert
Michael Robert
Isabelle Robert
Katleen Robert
Meyer Robert
Verdijk Robert
Marinella Roberti
Joshua Roberti
Cazzato Roberto
David Roberts
Michael Roberts
Craig Roberts
Darren Roberts
Sally Roberts
Stephen Roberts
Steven Roberts
S. Craig Roberts
Ron Roberts
Dar A Roberts
Jason Roberts
Seán Roberts
John Roberts
Sara Roberts
Gordon Roberts
Dan Roberts
Keith Roberts
Chris Roberts
Robert Roberts
Jennifer Roberts
Hannah Roberts
Het Roberts
Lance Roberts
Jacqueline Roberts
Lynette Roberts
Patrick Roberts
Lindsay Roberts
Brent Roberts
William Roberts
Eugene Roberts
Borre Robertsen
Erle Robertson
Hugh Robertson
Brian Robertson
Janice Robertson
Sarah Robertson
Anne Robertson
John Robertson
Danielle Robertson
Gregory Robertson
Eleanor Robertson
Judy Robertson
Stephen Robertson
D. Ross Robertson
Lindsay Robertson
Suzanne Robertson
Michael Robeson
Scott Robeson
Jacques Robidoux
Rebecca Robillard
Charles Robin
S. Robin
Libby Robin
Jean-Marie Robine
Nicolas Robine
Peter Robins
Maxi Robinski
Scott Robinson
Richard Robinson
Rob Robinson
James Robinson
Mark Robinson
Elva Robinson
Karen Robinson
Victoria Robinson
Joan Robinson
Beren Robinson
Terry Robinson
Colin Robinson
Scott R. Robinson
Phyllis Robinson
Eric Robinson
Margaret Robinson
Paul Robinson
Kate Robinson
Dudley Robinson
Jennifer Robinson
Matthew Robinson
Esther Robinson
Nicolas Robinson-Garcia
Marc Robinson-Rechavi

Nicola Robinson-Smith
Christopher Robison
Gerardo Robledo
Juan Jose Robledo-Arnuncio
Pedro Robles
Nicolas Robles
Ted Robles
Eduardo Robles-Belmont
Alan Robock
Paul Robson
Michael Robson
Robert Robson
Christine Robson-Doucette
John Robst
Daniel Roby
Katherine Roby
Joaquim Roca
Xavier Roca
Miquel Roca
Arantxa Roca-Feltrer
Maria Rocca
Aldo Roccaro
Phillippe Rocca-Serra
Iara Rocchetta,
Marcella Rocchetti
Stéphane Rocchi
Duccio Rocchini
Andrea Rocco
Gaetano Rocco
Patricia Rocco
Fernando Roch
Marie Roch
Luiz Rocha
Eduardo Rocha
Edson Rocha
Rafael Rocha
Oscar Rocha
Maria José Rocha
Dominique Rocha
Joana Rocha
Juan Rocha
Flavio Rocha
Ricardo Rocha
Fred Rocha
Leticia Rocha-Zavaleta
Katherine Roche
Joelle Roche
Philippe Roche
Colette Roche
Benjamin Roche
Jenny Roche
Gael Y. Rochefort
Nathalie Rochefort
Christian Rocheleau
Emma Rochelle-Newall
Petra Rocic
Charles Rock
Jason Rock
Christoph Rocken
Peter Rockers
Daniel Rockey
Kathy Rockland
Joacim Rocklöv
Stanley Rockson
Patricia Rockwell
Cheryl Rockwell
Robert Rockwell
Neesha Rockwood
Edward Rockwood
Barry Rockx
Xavier Roda
Avital Rodal
Celia Rodd
Matthew Rodda
Wojciech Rode
David Rodeberg
Matthew Rodeheffer
Franz Rödel
Luigi Rodella
Richard Roden
Eric Roden
Daniel Roden
Anja Roden
Heinrich Roder
Christoph Roderburg
Hans-Reimer Rodewald
Amanda Rodewald
Helen Rodger
Alison Rodger
Karla Rodgers
James Rodgers
Torrey Rodgers
Giverny Rodgers
Eli Rodgers-Melnick
Ana Rodiles Guerrero
Rocio Rodiles-Hernandez

Emmanouil Roditakis
Isabel Roditi
George Rodney
Marina Rodnina
Xavier Rodo
Georgios Rodolakis
D. Rodri´Guez De La Cruz
Chamira Rodrigo
Elaine Rodrigues
Gabriela Rodrigues
Márcia Rodrigues
Debora Rodrigues
Eduardo Rodrigues
Pedro Rodrigues
Joao Rodrigues
Ema Rodrigues
Everton Nei Lopes Rodrigues
Lidiany Rodrigues
Aline Rodrigues Hoffmann
Lurdes Rodrigues-Duarte
Luis Rodriguez
Santiago Rodriguez
Edgardo Rodriguez
Monica Rodriguez
Mariano Rodriguez
Fausto Rodriguez
Jaime Rodriguez
Adrian Rodriguez
Jan Rodriguez
L Rodriguez
Natalia Castano Rodriguez
Cristina Rodriguez
Diana Rodriguez
Fernando Rodriguez
Nuria Rodriguez
Marcela Rodriguez
Christophe Rodriguez
Brian Rodriguez
Airam Rodríguez
Amaia Rodríguez
Rafael Rodríguez
María Del Carmen Rodríguez
Jonathan Rodríguez
Laura Rodríguez
Rosa Rodríguez
Ricardo Rodríguez
Fernando Rodriguez De Fonseca
Francisco Jose Rodríguez Hernandez
Santiago Rodriguez Lopez
Luis Rodríguez Lorenzo
Javier Rodriguez Martinez
Cesar Rodriguez Sanchez
Beatriz Rodriguez Vega
Manuel Rodriguez-Achach
Jose Rodriguez-Alvarez
Antonio Rodriguez-Ariza
Jesús Rodríguez-Baño
Alicia Rodríguez-Barbero
Isabel Rodriguez-Barraquer
Elisabeth Rodriguez-Bies
Carmen Rodriguez-Blazquez
Enrique Rodriguez-Boulan
R.C. Rodríguez-Caro
Javier Rodriguez-Carrio
Kryssia Rodriguez-Castro
Isaac Rodriguez-Chavez
Francisco Rodriguez-Covarrubias
Ignacio Rodriguez-Crespo
Susana Rodríguez-Echeverría
Sara Rodríguez-Enríquez
Naiara Rodríguez-Ezpeleta
Francisco Rodriguez-Frias
Pablo Rodríguez-González
Gaston Rodriguez-Granillo
Bernardo Rodriguez-Iturbe
Mauricio Rodriguez-Lanetty
Ismael Rodriguez-Lara
Joaquin Rodriguez-Leon
José Manuel Rodríguez-Llanes
Victor Rodríguez-Moreno
Antonio Rodríguez-Núnez
Alexander Rodriguez-Palacios
Dolors Rodríguez-Pardo
F. Rodríguez-Pascual
Manuel Rodríguez-Perálvarez
Martin Rodriguez-Porcel
Federico Rodriguez-Porcel
Diego Rodriguez-Pujol
Diego Rodriguez-Puyol
Noe Rodriguez-Rodriguez
Rosalia Rodriguez-Rodriguez
Sonia Rodríguez-Ruiz
Francisco Rodriguez-Sanchez
Manuel Rodriguez-Vallee
Mayela Rodríguez-Violante
Jose Rodriguez-Zavala

Ursula Rodríguez-Zúñiga
Timothy Rodwell
Jung-Hye Roe
Simon Roe
Elke Roeb
Urte Roeber
Kathryn Roecklein
James Roede
Robert Roeder
Thomas Roeder
Falk Roeder
Mario Roederer
Ben Roediger
Will Roeffen
Diana Roeg
Nicholas Roehner
Patrick Roehrdanz
Philip Roelandt
Corné Roelen
Helene Roelofs
Paul Roepe
Luiz Roesch
Veit Roessner
Francois Roets
Leonardo Roever
Henrique Rofatto
George Roff
Steve Roffler
Ofer Rog
Anita Rogacs
Emily Rogalski
Marcelo Rogalski
Slavko Rogan
Mark Rogan
Elizabeth Rogawski
Claire Rogel-Gaillard
Boris Rogelj
Lars Rogenmoser
Sébastien Roger
Jerome Roger
Clemence Roger
Amino Rogerio
Thomas Rogers
Michael Rogers
Jeffrey Rogers
James Rogers
Elizabeth Rogers
Mary-Louise Rogers
Maximillian Rogers
Angela Rogers
Kerrylee Rogers
Natasha Rogers
Alice Rogers
Chris Rogers
Bonnie Rogers
Z. Rogers
Juan Rogers
Peter Rogers
Connie Rogers
Laura Rogers-Bennett
Stephen Rogerson
Peter Rogerson
Michelle Rogerson
Dirk Roggenbuck
Michael Roggenbuck
Victor Roggli
Robert Roghair
Mehrdad Roghani
Eric Rogier
Vera Rogiers
Bernard Rogister
Gerhard Rogler
Didier Rognan
Odd Harald Rognerud Jensen
Giulio Rognini
Carla Rognoni
Marta Rogowska
Isabelle Rogowski
Miin Roh
Mark Roh
Tae-Young Roh
Tibor Rohacs
Lisa Rohan
Kelly Rohan
Maryam Rohani
Mohammad Rohban
Kyle Rohde
Marieke Rohde
Holger Rohde
Christian Rohde
Paul Rohde
Benjamin Rohe
Gustavo Rohenkohl
Rajesh Rohilla
Sreevathsa Rohini
Aarti Rohira

Nadin Rohland
Norbert Rohlf
Ursula Rohlwink
Françoise Rohner-Jeanrenaud
Sussanne Rohrbach
Bärbel Rohrer
Bernd Röhrle
Pierre Simon Rohrlich
Sabine Rohrmann
Anna Roik
Emmanuel Roilides
Sergio Roiloa
Timo Roine
Bernard Roitberg
Jamie Roitman
Mitchell Roitman
David Roiz
Mauricio Rojas
Roxana Rojas
Clemencia Rojas
Ximena Rojas
Norma Rojas-Avelizapa
David Rojas-Rueda
Ewa Rojczyk
Mumtaz Rojiani
Rosa Rojo
Carmen Rojo
David Rojo Arjona
Slawa Rokicki
Theodore Rokkas
Anton Roks
Kenneth Roland
Teboh Roland
Eduardo Roldan
Teresa Roldán-Carrillo
Andreas Rolf
Christian Rolfo
Stephane Rolland
Udo Rolle
Chalotte Rolle
Richard Roller
Hardy Rolletschek
Eleonora Rolli
Pierre Rollin
Kim Rollings
Jeffrey Rollins
Lee Rollins
Louise Rollins-Smith
Asya Rolls
Josep Roma
Guglielmo Roma
Sarah Romac
Tania Romacho
Jean-Baptiste Romagnan
Alessandra Romagnoli
Renato Romagnoli
Micaela Romagnoli
Stefano Romagnoli
Jesus Romalde
Francesco Roma-Marzio
Jesse Roman
Elaine Roman
Sabiniano Roman
Adrienne Roman
Lara Roman
Marc Romana
Bruna Romana-Souza
Alessandra Romanelli
Nikolaus Romani
Andrea Romani
Aldo Romani
Christoph Romanin
Helena Romaniuk
Marco Romano
Marta Romano
Maurizio Romano
Tracy Romano
Maria Fiammetta Romano
Giulia Romano
Giovanna Romano
Daniele Romano
Simona Romano
Salvatore Mario Romano
Nicla Romano
Megan Romano
Mario Romano
Vito Romano
Barbara Romano
Eric Romanoswki
Julia Romanova
Andrej Romanovsky
Charles Romanowski
Rubén Román-Ramos
Hanna Romanska
Alexander Romanyukha
Antonino Romanzo

Luisa Romao
Pedro Romao
Jörg Römbke
Simona Rombo
Simona E. Rombo
Eric Rombokas
Adamo Rombolà
Ans Rombout
Serge Rombouts
Keith Rome
Pierre Rome
Vincenzo Romei
Jörg Romeis
Maksym Romensky
Giovanni Romeo
Stefania Romeo
Orazio Romeo
Lewis Romer
Antonio Romero
Francisco Romero
Javier Romero
Jaime Romero
Ignacio Romero
Nuria Romero
Irene Romero
Anibal Romero
Angel Romero
Esperanza Romero
Guillermo Romero
Heather Romero
Juanita Romero-Diaz
Manuel Romero-Gómez
Maria C. Romero-Puertas
Ethan Romero-Severson
Charles Romieu
Giuseppe Romito
Nanda Rommelse
Tania Romo-González
Gergely Róna
Katharina Ronacher
Paola Roncada
Vittoria Roncalli
Antonella Ronchi
Chiara Ronchini
Nicoletta Ronda
Philippe Rondé
Jorge Ronderos
Matthew Rondina
Itamar Ronen
Catherine Ronet
James Roney
Charles Roney
Junkang Rong
Libin Rong
Jun Rong
Zhili Rong
Shisong Rong
Cindy Gh Rönnau
Bent Ronnestad
Sissel Rønning
Kjersti Ronningen
Graziella Ronsein
Monique Roobol
Julian Rood
Kara Rood
Farzin Roohvand
Maarten Rookmaaker
Ilse Rooman
Neil Rooney
Peter Roopnarine
Christian Roos
Michael Roos
Per Roos
Naana Roos
Martin Röösli
Marilyn Roossinck
Allen Root
Robert Root-Bernstein
Holly Root-Gutteridge
Daniel Rootman
Bob Roozenbeek
Stefan Ropele
Steven Roper
David Roper
Randall Roper
Rachel Roper
Marcus Roper
Michael Roper
Athina Ropodi
Angelique Rops
Antonio Roque
Helio Roque
Matheus Roque
Fo Roque
Joana Roque De Pinho
Anne-Marie Roque-Afonso

Alain Roques
Cristina Roquet
Joanna Rorbach
Ellen Rorke
Maria Ros
Carlos Ros
Ivo Ros
Robert Ros
Alessandro Rosa
Daniela Rosa
Angelo Rosa
Marilin Rosa
Cristina Rosa
João Rosa
Maria Rosa
Andre Henrique Rosa
Adriane Rosa
Juan Rosado
Bruno Rosado
Paulo Henrique Rosado De Castro
Carlos Rosales
Sergio Rosales-Mendoza
Sergio Rosales-Rodriguez
Jesus Rosales-Ruiz
Eduardo Rosa-Molinar
Gus Rosania
Mario Rosanova
Gracy Rosario
Scott Rosas
Ulises Rosas
Antonio Rosas
Paola Rosas
Alessandra Rosati
Carlo Rosati
Antonio Rosato
Jason Rosch
Hamilton Roschel
Christiane Roscher
Federica Roscioni
Lori Roscoe
Paul Roscoe
Thomas Roscoe
Noel Rose
Mary Rose
Ray Rose
Jonathan Rose
Adam Rose
Jason Rose
Aaron Rose
Amy Rose
Michael Rose
C.F. Rose
Charles Rose
Devin Rose
Warrick Roseboom
Kathryn Rosecrans
Stefan Rose-John
Patricia Rosel
Anna Rosell
Laura Rosella
Francesco Roselli
Leonilde Roselli
Daniele Rosellini
Anthony Rosellini
Charles Roseman
Stuart Rosen
Hugo Rosen
David Rosen
Rochelle Rosen
Hannah Rosen
Steven Rosen
Ran Rosen
Charlotte Rosenbach
Jean Rosenbaum
Tamara Rosenbaum
James Rosenbaum
Simon Rosenbaum
Michael Rosenberg
Sharon Rosenberg
Yvonne Rosenberg
Nora Rosenberg
Molly Rosenberg
Jessica Rosenberg
Abby Rosenberg
Helene Rosenberg
Andrew Rosenberg
Jens Rosenberg
Mona Rosenberg
William Rosenberger
Adam Rosenblatt
Tova Rosenbloom
David Rosenblueth
Michael Rosenblum
Jonas Rosendahl
Mikkel Rosendahl
Thomas Rosendal

Amy Rosenfeld
Steven Rosenfeld
Mark Rosenfield
Matthew Rosengart
Rhonda Rosengren
Andreas Rosenhagen
Ulf Rosenhall
Brad Rosenheim
Jessica Rosenholm
Peter Rosenkranz
Alexander Rosenkranz
Gerd Rosenkranz
Jeremy Rosenkranz
Hanna Rosenmann
Robert Rosenson
Ruth Rosenstein
Philip Rosenstiel
Stephen Rosenstiel
Tatiana Rosenstock
Tatiana Rosenstok
Kenneth Rosenthal
Adam Z. Rosenthal
Samantha Rosenthal
Rita Rosenthal
Meagen Rosenthal,
Lanny Rosenwasser
Ivana Rosenzweig
Connie Roser-Renouf
Florencia Rosetti
Alex Rosewell
Hendrik Rosewich
Roger Ros-Freixedes
Marcel Rosinger
Calvin Roskelley
Marieke Roskes
Krishna Roskin
Beverly Roskos-Ewoldsen
Robert Roskoski
Nicholas Roskruge
Frank Rösl
Tomas Roslin
Johanna Rosman
Marsha Rosner
Judah Rosner
Sabine Rosner
Christopher Ross
Kenneth Ross
Michael Ross
Nancy Ross
Callum Ross
Bernhard Ross
James Ross
Joshua Ross
Beth Ross
Michelle Ross
Stephen Ross
Caroline Ross
Pablo Ross
Jennifer Ross
Carlos Rossa Junior
Jan Rossaint
Federico Rossano
José Luis Ros-Santaella
Bruno Rossaro
Mateus Rossato
Davi Rossatto
Amy Ross-Davis
Filippo Rosselli
Lauren Rossen
John Rossen
Anne Rosser
Charles Rosser
Stefano Rossetti
Simona Rossetti
Carlos Rossetti
Ornelle Rossetto
Cyprian Rossetto
Alyssia Rossetto
Pellegrino Rossi
Antonio Rossi
Luca Rossi
Carlo Rossi
Simona Rossi
Michael Rossi
Valentina Rossi
Gian Paolo Rossi
Noreen Rossi
Francesca Rossi
Jean-Pierre Rossi
Giulio Rossi
Vittorio Rossi
Sergio Rossi
Esther Rossi
Michele Rossi
Livia Rossi
Jeffrey Ross-Ibarra

Ombeline Rossier
Julien Rossignol
Peter Rossing
Walter Rossing
Paolo Rossini
Moritz Rossner
Gertrud Rössner
Antonietta Rosso
Gian Maria Rossolini
Wilfried Rossoll
Theresa Rossouw
Heidi Rossow
Lionel Rostaing
Mehran Rostami
Vittorio Rosti
Susana Rostro-Garcia
Martin Rosvall
Rhonda Rosychuk
Antal Rot
Paul A Rota
Rossella Rota
Christopher Rota
Paolo Rota
Matteo Rota
Omar Rota-Stabelli
Susan Rotenberg
Alexander Rotenberg
Michael Roth
Marie-Paule Roth
Steven Roth
Camille Roth
Patrick Roth
Zvi Roth
Daniel Roth
Gregory Roth
Olivia Roth
Sebastien Roth
Elliot Roth
Anjali Röth
Brad Rothberg
Amy Rothberg
Nicolas Rothen
Barbara Rothen
Dietrich Rothenbacher
Marc Rothenberg
Stephen Rothenberg
Sarah Rothenberg
Martina Rothenbühler
Anya Rothenbuler
Nils Rother
Joachim Röther
Kathrin Rothermich
Carl Rothfels
Till Rothig
Kai Rothkamm
Holger Rothkegel
Matthias Roth-Kleiner
Hermann-Josef Rothkötter
David Rothman
Jessica Rothman
Micol Rothman
M. J. Rothmann
T. Rothmund
Anita Roth-Nebelsick
Hannah Rothstein
Daniela Rotin
Alice Rotini
Noemi Rotllan
Oren Rotman
Joris Rotmans
Dino Rotondo
Veerle Rots
Wolfgang Rottbauer
Shlomo Rottem
Stephen Rottgers
Giulia Rotundo
Brice Rotureau
Vanessa Rouach
Hatem Rouached
Jean-Max Rouanet
Maria Roubelakis
David Roubik
Eric Rouchka
Yasser Roudi
Annette Roug
Jacques Rougemont
Nicolas Rougier
Hélène Rougier
Guillermo Rougier
Geneviève Rougon
Nicolas Rouhier
Vahid Rouhollahi
Yves Rouillé
Dimitrios Roukos
Alexandre Roulin
Anne-Lyse Roulin Ducrest

Carli Roulston
T'Ai Roulston
Kathryn Roulston
Lubka Roumenina
Robert Rounbehler
Sharon Rounds
Adam Rountrey
Jim Rouquette
Eugeni Roura
Nuria Roura-Pascual
Bryan Rourke
Kasper Rouschop
Rodney Rouse
Ronald Rousseau
Eric Rousseau
Karine Rousseau
Francois Rousseau
David Rousseau
Antoine Rousseau
Clotilde Rousseau
Marc Roussel
Alain Roussel
Guillaume Rousselet
Patricia Rousselle
Matthieau Roustit
Matthieu Roustit
Namita Rout
Pramod Rout
Sylvain Routier
Christina Routsi
Philippe Roux
Saartjie Roux
Perrine Roux
Stanley Roux
Jean-Christophe Roux
Camille Roux
Christine Rouzioux
Lucio Rovati
Anne Rovelet-Lecrux
Alessio Rovere
Francesco Rovero
Pietro Roversi
Giandomenico Roviello
Nikoletta Rovina
David Rowat
Wade Rowatt
J. Marcus Rowcliffe
J. Rowe
Brian Rowe
Jacob Rowe
Heather Rowe
Aaron Rowe
Gene Rowe
Christie D. Rowe
Nick Rowe
Helen Rowe
Fiona Rowe
Suzanne Rowe
A. Rowe
Sylvia Rowe
Hannah Rowe
Mark Rowland
Bosco Rowland
Sarah Rowland-Jones
Stella Rowlands
Christopher Rowley
Erik Rowley
James Rowley
Peter Rowley-Conwy
Campbell Roxburgh
Sabita Roy
Scott Roy
Polly Roy
Denis-Claude Roy
Siddhartha Roy
Paul Roy
Helen Roy
Partha Roy
Chad Roy
Denis Roy
Snehashis Roy
Indrajit Roy
Sujoy Roy
Joy Roy
Shovonlal Roy
Romain Roy
Richard Roy
Sib Roy
Lise Roy
Ambrish Roy
Pratik Roy
Sunanda Roy
Pierre-Marie Roy
Herve Roy
Bishnupada Roy
Kingshuk Roy Choudhury

Piklu Roy Chowdhury
Biswajoy Roy-Chaudhuri
R. Roychoudhuri
Pavitra Roychoudhury
Jayanta Roy-Chowdhury
Christophe Royer
Jimmy Royer
Todd Royer
Brigitte Royer-Pokora
Stephen Royle
Andrew Royle
Luca Roz
Ignacio Rozada
Malgorzata Rozanowska
Malgorzata Rózanowska
Emmanuel Roze
Daniel Rozen
Michal Hason Rozenstein
Jean Michel Rozet
Timofey Rozhdestvensky
Andrii Rozhok
Gary Rozier
Jan Rozman
Peng Ru
Diyun Ruan
Sheng-Yuan Ruan
Dike Ruan
Jianhua Ruan
Jianbin Ruan
Yuhong Ruan
Leilei Ruan
Neil Ruane
Rodrigo Ruano
Jorge Ruas
Martial Ruat
Enrico Rubaltelli
Speranza Rubattu
Dennis Rubbenstroth
Andrea Rubboli
Leonid Rubchinsky
Peter Ruben
Peter Rubenstein
Dan Rubenstein
Leonard Rubenstein
Marie Rubér
Nicolas Rubido
Marta Rubiera
Joshua Rubin
Brian Rubin
Gary Rubin
Benjamin Rubin
Bruce Rubin
Sebastien Rubin
Dvora Rubinger
Michele Rubini
Mauro Rubini
Tiziana Rubino
Corrado Rubino
Guita Rubinsky-Elefant
Eric Rubinstein
Jack Rubinstein
Adolfo Rubinstein
Hector Rubinstein
Angel Rubio
Marie-Thérèse Rubio
Vicente Rubio
Luis Rubio
Ignacio Rubio
Isabel T Rubio
Justin Rubio
Luis Ángel Rubio
Marta Rubio-Codina
Paula Rubio-Fernández
Diego Rubolini
Nicole Rübsamen
Nadia Rucci
Patrick Ruch
Marek Ruchala
Antoni Rucinski
Robert Rucker
Edmund Rucker
Christian Rückert
John Rudan
Jérémie Rudant
Serge Rudaz
Merrill Rudd
Murray Rudd
Vidar Ruddox
Richard Ruddy
Douglas Ruden
Avima Ruder
Neil Ruderman
James Rudge
Jennifer Rudgers
Knut Rudi
Assaf Rudich

Seitz Rüdiger
Mario Rüdiger
Bieler Rüdiger
Gary Rudnick
Deborah Rudnick
Wieslawa Rudnicka
Michael Rudnicki
Geir Rudolfson
Kara Rudolph
Shivaprakash Rudramurthy
Lukas Rueber
Matthias Ruebner
Angelica Rueda
Manuel Ruedi
Stefan Ruediger
Nicole Ruedy
Franziska Rueff
Markus Ruegg
Urs Ruegg
Kristen Ruegg
L Bruno Ruest
Maja Ruetten
Dennis Ruez
Thomas Ruf
Alessandra Rufa
S. Emil Ruff
Ilana Ruff
Enrico Ruffini
Piero Ruggenenti
Franco Ruggeri
Gregory Ruggerone
Michael Ruggieri, Sr.
Robert Ruggiero
Anand Rughani
Paul Rugman-Jones
Stefan Ruhl
Ralph Rühl
Heiko Rühl
Frank Rühli
Mohammed Ruhman
Martina Ruhmland
Mark Ruhnke
Liangyou Rui
Junpeng Rui
Zhang Ruifu
Tom Ruigrok
Ynte Ruigrok
Matthijs Ruiter
Marilyn Ruiz
John Ruiz
Teresa Ruiz
Juan Ruiz
Carolina Ruiz
Facundo Ruiz
Natividad Ruiz
Maria Jose Ruiz Martos
Jesus Ruiz-Cabello
Jordi Ruiz-Camp
Ana Ruiz-Casado
Javier Ruiz-Castillo
Maria Ruiz-Echevarria
Jose Francisco Ruiz-Fons
Manuel Ruiz-García
Mariano Ruiz-Gayo
Mario Ruiz-Gonzalez
José Ruiz-Herrera
Maria Ruiz-Lopez
Jorge Ruiz-Medrano
Edward Ruiz-Narvaez
Martin Ruiz-Ortiz
Eduardo Ruiz-Pesini
Magdalena Ruiz-Rodriguez
Francisco Ruiz-Ruano
Jesus Ruiz-Suarez
Rosa M Ruiz-Vázquez
Ursula Ruiz-Vera
Håkon V Rukke
Françoise Rul
H. Earl Ruley
Kristiina Rull
Juan Rull
Kendra Rumbaugh
Martin Rumbo
Alice Rumbold
M.A. Rumi
Franklin Rumjanek
Vivian Rumjanek
Christian Rummel
Jan Rummel
Nikol Rummel
Samantha Rumschlag
Anna Rumshisky
John Runcie
Mark Runco
Gabriella Rundblad
Andrew Rundle

Francesco Rundo
Alessio Rungatscher
Jørgen Rungby
Kurt Runge
Wanilada Rungrassamee
Jos Runhaar
Kadri Runnel
Jonathan Runstadler
Michelina Ruocco
Christin Ruoff
Kalle Ruokolainen
Margherita Ruoppolo
Jani Ruotsalainen
Shivani Ruparel
Stefan Rupf
Stefan Ruping
Maja Rupnik
Rick Rupp
André Rupp
Rüdiger Rupp
Oliver Rupp
Jan Rupp
Etienne Ruppe
Gregg Ruppel
Charles Rupprecht
Eszter Ruprecht
Jairos Rurinda
Horea Rus
Guillermo Rus
Hannes Rusch
Travis Rusch
Laura Rusche
Massimiliano Ruscica
John Ruscio
Michael G. Ruscio
Rasa Ruseckaite
Clark Rushing
Paul Rushton
Simon Rushton
Marco Rusnati
Garry Russ
David Russ
Steven Russell
Paul Russell
Scott Russell
Anthony Russell
Jane Russell
Rodney Russell
Jacob Russell
Bruce Russell
Amy Russell
Steven Russell
Richard Russell
Tanya Russell
George Russell
Robin Russell
Margaret Russell
Mark Russell
Keith Russell
Matthew Russell
Maria Russell
Ann Russell
Marion Russier
Giandomenico Russo
Antonio Russo
Thomas Russo
Danilo Russo
Tommaso Russo
Domenico Russo
Giorgio Ivan Russo
Paul Russo
Valentina Russo
Ilaria Russo
Gabrielle Russo
Antonietta Russo
Manuela Russo
Isa-Rita Russo
Francesco Russo
Emilio Russo
Nino Russo
Michael Rust
Mike Rust
Neeti Rustagi
Alison Rustagi
Ingo Rustenbeck
Sachin Rustgi
Mauro Rustici
Alina Rusu
Mugurel Rusu
Marcello Ruta
Claude Rutanga
Matt Rutar
Bone Ruth
Martin Ruthardt
Edward Ruthazer
Derek Ruths

Justin Ruths
Heloisa Rutigliano
Ervan Rutishauser
Bastiaan Rutjens
Seward Rutkove
Tomasz Rutkowski
Adam Rutland
Shea Rutstein
Guy Rutten
Erica Rutten
Marcel Rutten
Michael Rutter
Ricardo Rüttimann
Daniel Rutz
Kenneth Ruud
Olli Ruuskanen
Suvi Ruuskanen
Gary Ruvkun
Peter Ruvolo
Maria Ruz
Erik Ruzek
Daniel Ružek
Evžen Ružicka
Manuela Ruzzoli
Srikanth Ryali
Krista Ryall
Edward Ryan
Michael Ryan
Una Ryan
Peter G Ryan
Elizabeth Ryan
Joanne Ryan
Aideen Ryan
Colm Ryan
Cormac Ryan
Matthew Ryan
John Ryan
Rita Ryan
Patrick Ryan
Terence Ryan
Robert Ryan
David Ryan
Tatiana Ryba
Fanny Rybak
Leonard Rybak
Daria Rybakova
Sergei Rybalkin
Ed Rybicki
Nancy Rybicki
Krystyna Rybka
Joshua Rychak
Ivan Rychlik
Malgorzata Rychlowska
Kelli Ryckman
Linda Rydén
Mark Ryder
Chris Ryerson
Bernhard Ryffel
Ryan Rykaczewski
Jamie Rylance
Chris Rylander
Grzegorz Rymarczyk
Zev Rymer
Tasmin Rymer
Agnieszka Rynda-Apple
Lene Ryom
Hyung Don Ryoo
Michael Ryoo
Andrew Rypel
Irina Rypina
Bart Rypma
Wojciech Rypniewski
Ann Rypstra
Eduard Ryschich
Erik Rytting
Sangryeol Ryu
Euijung Ryu
Stephen Ryu
William Ryu
Wang-Shick Ryu
Kwon-Yul Ryu
Jay Ryu
Young Hoon Ryu
Jongseong Ryu
Nina Rzechorzek
Arni S.R. Srinivasa Rao
Eduardo Sa
João Carlos Sa
Joana Sá
Paula Saa
Sammy Saab
Khaled Saad
Mohamad Saad
Ehsan Saadat
Debora Saade
Michael Saag

Hannes Saal
Tiina Saanijoki
Seppo Saarelainen
Antti Saari
Juha Saarikangas
Kimmo Saarinen
Urmas Saarma
Annika Saarto
Fahri Saatcioglu
Kathryn Saatman
Ingrid Saav
Carlos Saavedra
Juan Saavedra
Emma Saavedra
Sheena Saayman
Alessandro Saba
Omar Sabah
Valeria Sabaj
Sead Sabanadzovic
Charumathi Sabanayagam
Kalpana Sabapathy
Benedikt Sabass
Pablo Sabat
Grzegorz Sabat
Artur Sabat
Raimon Sabate
Patrizia Sabatelli
Sebastia Sabater
Gerardo Sabater-Grande
Maria Sabater-Lleal
Umberto Sabatini
Francesco Sabatini
Denise Sabatino
Juan Sabatte
Maurizio Sabatti
Laurent Sabbagh
Heba Sabbagh
Bernhard Sabel
Hamidreza Saberkari
Mario Sabetelli
Wilber Sabiiti
Caroline Sabin
Lora Sabin
Grosch Sabine
Hussein Sabit
Suraj Sable
Gaurav Sablok
Chris Sablynski
Roy Sabo
Ivan Sabol
Ivan Sabolic
Luc Sabourin
Nirmeen Sabry
Sarven Sabunciyan
Ahmet Sacan
Sergio Saccà
Matthew Sacchet
Massimo Sacchetti
Gianni Sacchetti
Nicoletta Sacchi
Veronica Sacchi
Randy Sacco
Alessandra Sacco
James Sacco
Patrick Saccone
Leonardo Sacconi
Ajanta Sachan
Sonya Sachdeva
Rohan Sachdeva
Lucia Maria Sacheli
Ravi Sachidanandam
Rainer Sachs
Laurent Sachs
Ingolf Sack
Markus Sack
David Sack
Dan Sackett
David Sacks
Gavin Sacks
Emma Sacks
Rachel Sacks-Davis
Todd Sacktor
Subash Sad
Ken-Ei Sada
Sepideh Sadaghiani
Kaori Sadakane
Makiko Sadakata
Anguraj Sadanandam
Manish Sadarangani
Shankar Sadasivan
Sivalal Sadasivan
Norihiro Sadato
Robert Sade
Wolfgang Sadée
Hassan Sadeghi Naeini
Asaf Sadeh

Naomi Sadeh
Hesham Sadek
Michel Sadelain
Provash Sadhukhan
Abbas Sadikot
Meredith Sadinski
Kashif Sadiq
Masoud Sadjadi
Michael Sadler
Brooke Sadler
Yoel Sadovsky
Ewa Sadowska-Krepa
Anna Sadowska-Rociek
Martin Sadowski
Hooman Sadri-Ardekani
Salim Sadruddin
Marat Sadykov
Mohsan Saeed
Muhammad Saeed
Fahad Saeed
Hidehisa Saeki
Oystein Saele
Xavier Saelens
Christoph Saely
Nelly Saenen
María Elena Sáenz
Pablo Saenz-Agudelo
Elizabeth Saewyc
Juan Saez
Fabrice Saez
Pablo Sáez
Gema Sáez Rodriguez
Asier Sáez-Cirión
Javier Sáez-Valero
Fayez Safadi
Mohammad Reza Safaei
Mahboobeh Safaeian
Ilgar Safak
Marwa Safar
Roya Safari
Dodi Safari
Karolina Safarzynska
Adeel Safdar
Stephen Safe
Martin Safer
Hugh Safford
Barbara Safiejko-Mroczka
Robert Safirstein
Steven Safren
Adam Safron
Audrey Saftlas
Tsuneo Saga
Ikuko Sagami
Sandrine Sagan
Skyler Sagarese
Alvaro Sagasti
Atiene Sagay
Julien Sage
Andrew Sage
Georg Sager
Charles Sagerström
Miguel Saggese
Isabella Saggio
Mohammad Ali Saghiri
Mehrnoosh Saghizadeh
Ramesh Sagili
Murat Saglam
Sejal Saglani
Francis Sagliocco
Evangelista Sagnelli
Kwamena Sagoe
Konstantinos Sagonas
Gurdeep Sagoo
Francesc Sagues
Robert Sah
Sukanta Saha
Pradip Saha
Asish Saha
Margaret Saha
Manujendra Saha
Chabita Saha
Sudipto Saha
Prasenjit Saha
Achinto Saha
Saikat Saha
Dipnarayn Saha
Chandan Saha
Supriya Saha
Subhrajit Saha
Swati Saha
Sibu Saha
Ekrem Sahan
Goutam Sahana
Naruhiko Sahara
Anagh Sahasrabuddhe
Seema Sahay

Satya Sahay
Vaidurya Sahi
Orhan Sahin
Ismet Sahin
Mustafa Sahin
Hacer Sahin
Ozgur Koray Sahingoz
Jason Sahl
Julie Sahler
Cecilia Sahlgren
Ullrika Sahlin
Christian Sahlmann
Shannon Sahlqvist
Debashis Sahoo
Susmita Sahoo
Satyaprakash Sahoo
Malaya Sahoo
Nirakar Sahoo
Sanghamitra Sahoo
Surinder S Sahota
Amrik Sahota
Afaf Sahraoui
Praveen K Sahu
Anshuman Sahu
Sitanshu Sahu
Ignasi Sahun
Elvira Sahuquillo
Sergio Saia
Rafael Saia
Adolfo Saiardi
Neveen Said
Zuzana Saidak
Abdul Saied
Tabish Saifee
Romesh Saigal
Kumiko Saika
Shinji Saiki
Vesa Saikko
Pothana Saikumar
Steve Sain
Vikram Saini
Ekjyot Saini
Vaibhav Saini
Sharanjot Saini
Upendra Sainju
Amanda Sainsbury
Magali Saint-Geniez
Gaelle Saint-Hilary
Pauline Saint-Martin
Pedro Saint-Maurice
Bruno Sainz
Teresita Sainz-Espuñes
Yoshifumi Saisho
Sheila Sait
Takehiko Saito
Yoshiro Saito
Shigeru Saito
Mayuko Saito
Masaya Saito
Suzue Saito
Akira Saito
Takeshi Saito
Masayuki Saito
Tsuyoshi Saito
Isao Saito
Tais Saito
Hiroshi Saito
Kazuki Saito
Priscila T.M. Saito
Yuki Saito
Atsushi Saito
Tetsuichiro Saito
Tatsuya Saitoh
Kazuyoshi Saitoh
Shigeaki Saitoh
Leonor Saiz
Margarita Sáiz
Ronald Sajarinov
Hideo Saji
Hisashi Saji
Naoki Saji
Muhammad Sajid
Ravi Sajja
Umadevi Sajjan
Mamta Sajwan-Khatri
Masakiyo Sakaguchi
Shota Sakaguchi
Shuichi Sakaguchi
Lynn Sakai
Juro Sakai
Akinori Sakai
Toshiyuki Sakai
Hideki Sakai
M. Sakai
Takao Sakai
Takashi Sakai

Shunsuke Sakai
Daisuke Sakai
Ryuichi Sakai
Kumi Sakai-Kato
Taisuke Sakaki
Iori Sakakibara
Wataru Sakamoto
Shuji Sakamoto
Taiji Sakamoto
Jeffery Sakamoto
Kensuke Sakamoto
Masahiro Sakamoto
Koji Sakamoto
Elza Sakamoto-Hojo
Rama Murthy Sakamuri
Ikuo Sakane
Yukinori Sakao
Ichiro Sakata
Yasushi Sakata
Asuka Sakata
Yoichi Sakata
Khashayar Sakhaee
Amul Sakharkar
Susumu Sakimoto
Kenji Sakimura
I. Sakinofsky
M. Sakiroglu
George Sakoulas
Nitin Saksena
Priyanka Saksena
Sunil Saksena Raj
Nicole Saksens
Brit I. Saksvig
Sadayappan Sakthivel
Tetsushi Sakuma
Yoh Sakuma
Yasuhito Sakuraba
Takeshi Sakurai
Takuya Sakurai
Fuminori Sakurai
Takashi Sakurai
Akira Sakurai
Samuel Sakyi
Maria Sal Moyano
Claudia Sala
Evis Sala
Gessica Sala
David Salac
Srinivas Saladi
Nick Salafsky
Ali Salajegheh
Babatunde Salako
Omolola Salako
Roser Sala-Llonch
Muhammad Salam
Alan Salama
Suzy Salama
Therese Salameh
Justin Salamon
Daniel Salamone
Asaf Salamov
Ali Salanti
Monica Sala-Rabanal
M.J. Salar-García
Keyan Salari
Safia Salaria
Arash Salarian
Diego Salariato
Leonardo Salas
Rodrigo Salas
Dolores Salas-Trejo
Kinga Salat
Cristiano Salata
Mathieu Salaün
Diego Salazar
Gloria Salazar
Gerardo Salazar
J. Michael Salbaum
Adriana Salcedo
Suzana Salcedo
D.D. Salcido
Carlota Saldanha
Paulo Saldiva
Alessandro Sale
Michele Sale
Raquel Sá-Leão
Paul Saleeb
Mohamed Saleem
Shadi Saleh
Marwah Saleh
Elham Salehi
Leili Salehi
Hamid Salehiniya
Mogjiborahman Salek
Shahram Salek-Ardakani
Ghasem Salekdeh

Tamer Salem
Mohammed Salem
Mohammad Salem
Shireen Saleque
Mariacarolina Salerno
Gianandrea Salerno
Loredana Salerno
Verônica P. Salerno
Gabriele Sales
M.C. Sales
João Sales
Sílvia Sales-Peres
Jared Saletin
Meredith Saletta
Roberto Salgado
Paula Salgado
Guillermo Salgado-Maldonado
Wilmara Salgado-Pabon
Padmini Salgame
Matthew Salganik
Maxim Salganik
Salizawati Muhamad Salhimi
Michela Sali
Kevin Saliba
Faouzi Saliba
Elie Saliba
Bhargav Saligram
Vehid Salih
Jeremiah Saliki
Elena Salillas
Samina Salim
Saad Salim
Asmat Salim
Vahid Salimi
Irene Salinas
Santiago Salinas
Roberto Salinas
Manuel Salinas-Navarro
Miguel Salinero-Fort
Mariolina Salio
Andrew Salisbury
Adam Salisbury
Daniel Salisbury
Irving Salit
Henrik Salje
Glenn Salkeld
Tamer Sallam
Jose Maria Sallan
Jean-Michel Sallenave
Salam Salloum-Asfar
Jorge Salluh
Maria Anice Sallum
Fabio Sallustio
Fabrizio Sallustio
Nico Salmaso
Elina Salmela
Javier Salmeron
Marko Salmi
Anu Salminen
Adam Salmon
Brian Salmons
Vsevolod Salnikov
Markku Saloheimo
Reinaldo Salomao
Daniel Salomon
Roy Salomon
Carlos Salomon
Alberto Salomone
Sauro Salomoni
Brendan Saloner
Tuire Salonurmi
Haroon Saloojee
Jacqueline Salotti
Kristin Salottolo
Emmanouil Saloustros
Hannu Salovaara
Anne-Virginie Salsac
Jerome Salse
Chad Saltikov
Marcus Salton
Kristin Saltonstall
Fréderik Saltré
Tim Saltuklaroglu
Ashok Saluja
Gabriela Salvador
Raymond Salvador
Liliana Salvador
Bonifacio Salvador
Luis Salvador-Carulla
Maria Salvato
Domenico Salvatore
Veronica Salvatore
Marco Salvemini
Kjell Salvesen
Maria Salvetat
Sundeep Salvi

Daniele Salvi
Silvio Salvi
Gionata Salvietti
Alessandra Salvioli
Deborah Salvo
Sarah-Jeanne Salvy
Stephanie Salyer
Giulia Salzano
Mark Salzer
Michel Salzet
Zainab Samaan
M. Constantine Samaan
Rodney Samaco
Abdul Samad
Nadia Sam-Agudu
Ravi Samala
Afshin Samani
Horacio Samaniego
Saheli Samanta
Amalesh Samanta
Timir Samanta
Supriti Samantaray
Amali Samarasinghe
Chameen Samarawickrama
Marijana Samardzija
Eric Samarut
Lobelia Samavati
Usha Sambamoorthi
Nisha Sambamurthy
Ramkumar Sambasivan
Fabio Sambataro
Andrea Sambri
Gianmario Sambuceti
Ota Samek
Jonathan Samet
Diogo Samia
Arun Samidurai
Marcin Samiec
Ahmed Samir
Florence Samkange-Zeeb
Kimberley Samkoe
Jonathan Sammartino
Steffen Sammet
Rachel Sammons
R. Douglas Sammons
Tarik Sammour
Ian Sammy
Dorit Samocha-Bonet
Andriy Samokhvalov
Alexandre Samouilov
Jaime Samour
Paula Sampaio
José Sampaio
Elizabeth Sampaio
Francisco Sampaio
Jaime Sampaio
Tara Sampalli
Eric Sampane-Donkor
Maurilio Sampaolesi
Bhaven Sampat
Venkatesh Sampath
Deepa Sampath
Srinivasa-Gopalan Sampathkumar
Sruthi Sampathkumar
Luis Sampedro
Sofia Samper
Oltea Sampetrean
Jeffery Sample
Christopher Sampson
John Sampson
Robert Samson
Scott Samson
Chandramathi Samudi Raju
Michael Samuel
Didier Samuel
Gabrielle Samuel
Stephen Samuel
Labarge Samuel
D. Scott Samuels
T. Alafia Samuels
Joshua Samuels
David Samuels
Anne Samuelsen
John Samuelson
Jim Samuelson
Anne-Maj Samuelsson
R. Samulski
Abhilash Samykutty
Jun San Juan
Marta San Luciano
Alejandra San Martin
Gilles San Martin
Guillermo San Martín
Phillip San Miguel
Alvaro San Millan
Waldy San Sebastian-Ramirez

Thibault Sana
Daniel Sanabria
Yasser Sanad
Shoji Sanada
Yukihiro Sanada
Hironobu Sanada
Nader Sanai
Kumar Sanam
Yolanda Sanchez
Ignacio Sanchez
Ana Sanchez
Pascal Sanchez
Elena Sanchez
Sophie Sanchez
Mairena Sanchez
Aranzazu Sanchez
Elly Sanchez
Bruno Sanchez
Cecilia Sanchez
Diego Sanchez
Jaime Sanchez
Anthony Sanchez
Olga Sanchez
David Sanchez
Gloria Sanchez
Mar Sanchez
Sergio Sanchez
Ana Maria Sanchez
Gerardo Sánchez
Maria Sanchez Arago
Eugenio Sanchez Arreola
Gonzalo Sanchez Duffhues
Pilar Sánchez Gómez
Adela Sánchez Moreiras
Rosa Sanchez Panchuelo
Francisco Sánchez Vázquez
Eduardo Sanchez Vila
Jose Antonio Sánchez-Alcázar
Antonio Sanchez-Amat
Maria Teresa Sanchez-Ballesta
Francisco Sanchez-Bayo
Raudel Sánchez-Campusano
Tilman Sanchez-Elsner
Carlos Felix Sanchez-Ferrer
M. Victoria Sánchez-Gómez
Fermin Sanchez-Guijo
Pedro Sanchez-Lara
Alicia Sanchez-Mazas
Adrián Sánchez-Montalvá
Maria Dolores Sanchez-Niño
Santiago Sanchez-Pages
Raquel Sánchez-Pérez
Ricardo Sanchez-Prieto
Federico Sanchez-Quinto
Pablo Sanchez-Salcedo
Javier Sanchez-Sanchez
Alfredo Sánchez-Tójar
José Sánchez-Tomero
Andrea Sanchez-Vallet
Raquel Sanchez-Varo
Santiago Sánchez-Vicente
Jose Sanchez-Zapata
Fabian Sanchis
Joaquin Sanchis-Moysi
Pau Sancho-Bru
Giulio Sancini
Takaomi Sanda
Boe Sandahl Sørensen
Kristian Sandberg
L. Anders Sandberg
Michael Sandel
Linda Sandell
Lisa Sandell
Wil Sanden
Kerstin Sander
Sylvia Sander
Uwe Sander
Jamie Sanderlin
Ian Sanders
Nathan Sanders
Eduard Sanders
Jennifer Sanders
James O Sanders
Laurie Sanders
Shaun Sanders
Stephen Sanders
Lena Sanders
John Sanders
Kerrie Sanders
Stacey Sanders
Taren Sanders
Steven Sanders
William Sanders
Ralph Sanderson
Eric Sanderson
Michael Sanderson

Claire Sanderson
S. Sanderson
Anton Sandhofer
Kuljeet Sandhu
Gurjit Sandhu
Milap Sandhu
Sankaran Sandhya
Vicky Sandilands
Giulio Sandini
Anna Sandionigi
Maria Sandkvist
Netanya Sandler
Anthony Sandler
Wendy Sandler
Pascale Sandmann
David Sando
Leonidas Sandoval
J. Sandoval
Jonathan Sandoval-Castillo
German Sandoya
Jean-Christophe Sandoz
Marco Sandri
Mauro Sandrin
Christoph Sandrock
Joshua Sandry
Mark Sands
Gillian Sandstrom
Ulf Sandström
Ioanna Sandvig
Aniruddha Sane
Yves-Henri Sanejouand
Joshua Sanes
Rosemary Sang
Qing-Xiang Sang
Qingbing Sang
Nan Sang
Sabina Sangaletti
Samir Sangani
Felix Sangari
Joseph Sanger
Tina Sanghvi
Susana Sangiao
Giuseppe Sangiorgi
Federica Sangiuolo
Dominique Sanglard
Kazunori Sango
Taiye Sangoyomi
Mark Sangster
Francesca Sanguedolce
Vinod Sangwan
N. Sangwan
Naseer Sangwan
Davison Sangweme
Gabriele Sani
Marc Sani
Abbi Saniabadi
Shomyseh Sanjabi
Mohammed Sanjak
Archana Sanjay
Srinivasan Sanjay
Miguel A. F. Sanjuán
Alejandra Sanjuan Pla
Adam Sanjurjo
Pamela Sankar
Saikolappan Sankaralingam
Anoop Sankaranarayanan
Ganesh Sankaranarayanan
Ahter Sanlioglu
Isabel Sanmartin
Patricia Sanmartín
Marcelo Sanmartin-Fernandez
Vanna Sanna
Anna Sannino
W. Sannita
Teruo Sano
Hideto Sano
Motoaki Sano
Eirini Sanoudaki
Despina Sanoudou
Gerard Sanroma
Nathalie Sans
Lauren Sansing
Gordon Sanson
Clementina Sansone
Valeria Sansone
Andrea Sant
Lucia Santacruz
Scott Santagata
Carlo Santaguida
Alba Santaliestra Pasias
Alfredo Santalla
Marta Santalla
Fidel Santamaria
David Santamaria
Alberto Santamaria
Paolo Santambrogio

Miguel Santana
Maxwell Santana
Sharlene Santana
Nalini Santanam
Javier Santander
Elisa Santandrea
Andrea Santangeli
Tom Santangelo
Giovani Santangelo
María De La Paz Santangelo
Enrica Santarcangelo
Michael Santare
Andrea Santarelli
Emiliano Santarnecchi
Joshua Santarpia
Libero Santarpia
Carmen Santarpia
Xen Santas
Marco Santello
Indu Santhanagopalan
Arvind Santhanakrishnan
Balaji Santhanam
Jose Luis Santiago
Julio Santiago
Ana Raquel Santiago
Brendaliz Santiago
Tania Santiago
Joel Santiago Junior
Marie Laure Santiago-Raber
Juan Santibanez
J. Alfredo Santibáñez-Salgado
Marina Santic
Moisés Santillán
Mauricio Santillana
Francesca Santilli
Alexander Santillo
C. Santín
Emanuela Santini
Talitha C. Santini
Luca Santini
George Santis
Parankusam Santisree
Debora Santo
Michal Santocki
Matteo Santoni
Giorgio Santoni
Jarrod Santora
Rosaria Santoro
Mario Santoro
Domenico Santoro
Maria Santoro
Sónia A.O. Santos
Mauro Santos
Ana Santos
Leonilda Santos
Marinilce Santos
Pedro Santos
Javier Santos
Leonardo Santos
Udson Santos
Ubiratan Santos
Fran Santos
Adalberto Santos
Xavier Santos
Ar Santos
José Santos
Luísa Santos
Marcos Veiga Santos
Eduarda Santos
Mercedes Santos
Hélder A. Santos
Monica Santos
Marcio Santos
Rute Santos
Mudjekeewis Santos
Ana Lúcia Santos
Lara Lívia Santos Da Silva
Juan Santos Garcia
Lucas Santos Zambon
Ailiana Santosa
Enrique Santos-Bueso
Mathuram Santosham
Alejandro Santos-Lozano
Carlos Santos-Ocaña
Donato Santovito
Apurba Santra
Tapesh Santra
Swadeshmukul Santra
Peter Santschi
Valérie Santschi
Annalisa Santucci
Luca Santucci
Giulia Santulli
Gaetano Santulli
Borja Sañudo
Naoko Sanuki

Anderson Sa-Nunes
Suparna Sanyal
Subhabrata Sanyal
Anushree Sanyal
Esther Sanyé-Mengual
Jesus Sanz
Nuria Sanz
Libia Sanz
Juan José Sanz
Ana Sanz-Aguilar
Magdalena Sanz-Cortes
Victoria Sanz-Fernandez
Victoria Sanz-Moreno
Abdelhadi Saoudi
Murat Saparbaev
Clifford Saper
Przemyslaw (Mike) Sapieha
Piotr Sapiezynski
Lidar Sapir-Hen
Achyut Sapkota
Federica Saponaro
Gustavo Saposnik
Bernard Sapoval
Alexander Sapozhnikov
Martin Sapp
Sameer Saproo
Mahesh Saqcena
Vedat Sar
Funda Sar
Nejc Sarabon
James Saracco
Valeria Saraceni
Andrey Sarafanov
Stefan Sarafianos
Pantelis Sarafidis
H. Saragovi
Patricia Saragüeta
Joseph Saragusty
Anderson Sarah
Anni Saralahti
Jari Saramaki
Anya Sarang
Yehoshua Saranga
N. T. Saraswathi
Sujata Saraswat-Ohri
Gautam Sarath
Thanos Saratzis
Claire Saraux
Muthupandian Saravanan
Fernando Saravi
P. Sardar
Abhijit Sardesai
Jitender Sareen
Dhruv Sareen
Gabriele Saretzki
Shanta Sarfare
Marika Sarfati
Fred Sarfo
James Sargent
Frank Sargent
Daniel Sargent
Jennifer Sargent
Jane Sarginson
Saman Sargolzaei
Bruno Sargueil
Mohammed Sarhan
Ibrahim Sari
Vaibhav Saria
Elhadi Sariali
Lo Sarian
Amir Sariaslan
Dimosthenis Sarigiannis
Antonio Sarikas
Shiv Sarin
Hannu Sariola
Dipak Sarkar
Surojit Sarkar
Joy Sarkar
Sagartirtha Sarkar
Kishor Sarkar
Rajabrata Sarkar
Siddik Sarkar
Rupa Sarkar
Mohona Sarkar
Keka Sarkar
Monica Sarkar
Sovan Sarkar
Pralay Sarkar
Santosh Kumar Sarkar
Chinmoy Sarkar
Aloke Sarkar
Bidyut Sarkar
Mahfuzur Sarker
Shah-Jalal Sarker
Malabika Sarker
Latifur Sarker

Mosharraf Sarker
Abeed Sarker
Tiina Särkinen
Marianna Sarkissyan
Sakari Sarkkola
Fabrice Sarlegna
Peter Sarlin
Jayanta Sarma
S.S.S. Sarma
Birinchi Sarma
Bruno Sarmento
Olga L Sarmiento
Sanjeev Sarmukaddam
Tadeusz Sarna
Harvey Sarnat
Barbara Sarnecka
Euzenir Sarno
Malisa Sarntinoranont
Zoltán Sarnyai
Pablo Sarobe
Marco Saroglia
George Sarosi
Derek Sarovich
Demba Sarr
Jean Philippe Sarrette
Maria-Rosa Sarrias
Panagiotis Sarris
Jerome Sarris
Salvador Sarró
Elena Sarropoulou
Prashant Sarswat
Prashanat Sarswat
Marcello Sartarelli
Laura Sartiani
Mary Sartor
Maureen Sartor
Andrea Sartore Bianchi
Luisa Sartori
Massimo Sartori
Samantha Sartori
Benn Sartorius
Tina Sartorius
Gordon Sarty
Adelaida Sarukhan
Marinko Sarunic
Sarvaiya Jayrajsinh Sarvaiya
Minnie Sarwal
John Sarwark
David Sarwer
Marcella Sarzotti-Kelsoe
Tsukasa Sasaki
Takeshi Sasaki
Tsuyoshi Sasaki
Takao Sasaki
Joni Sasaki
Hitomi Sasaki
Ryo Sasaki
Yuko Sasaki-Sekimoto
Toshiyasu Sasaoka
Kazuhiro Sase
Benjamin Sasko
David Saslowsky
Hidenori Sassa
Laura Sasse
Kai Sassenberg
Jennifer Sasser
Davide Sassera
Christopher Sassetti
Oscar Sasso
E. Sasso-Cerri
Aaron Sasson
Magdalena Sastre
Jaume Sastre-Garriga
Akash Sastri
Masataka Sata
Venkata Satagopam
Karla Satchell
Milan Satcher
Norbert Satchivi
Premnandhini Satgunam
Vidiya Sathananthan
Prianka Sathe
Santosh Sathe
Noah Sather
William Sather
Brijesh Sathian
Gnanasekar Sathishkumar
Thozhukat Sathyapalan
Jitendra Satija
Leslie Satin
Kajana Satkunendrarajah
Hiroki Sato
Joao Sato
Shigeharu Sato
Akihiro Sato
Masamitsu Sato

Masa Sato
Ryuichiro Sato
Toshiro Sato
Hiroshi Sato
Koichi Sato
Shinji Sato
Takahiko Sato
Noriko Sato
Yukihiro Sato
Yukie Sato
Takeshi Sato
M. Sato
Shuichi Sato
Yoshifumi Sato
Yasunori Sato
Hiroe Sato
Koji Sato
Hirotaka Sato
Douglas Sato
Ayano Satoh
Kennichi Satoh
Akira Satoh
Michihiro Satoh
Hiroaki Satoh
Shigeru Satoh
Noriko Satoh-Asahara
Reetta Satokari
Anjali Satoskar
Yorifumi Satou
Jack Satsangi
Hideo Satsu
Naveed Sattar
Sampurna Sattar
Abdus Sattar
Glen Satten
Thomas Sattler
Sebastian Sattler
Frank Sattler
Sarina Saturn
Kapaettu Satyamoorthy
Ande Satyanarayana
Catherine Satzke
Laura Saucedo-Cuevas
Zahangir Alam Saud
Patrick Saudan
Aurore Saudemont
Michael Sauer
Karsten Sauer
Heinrich Sauer
John Sauer
Sven Sauer
Tom Sauer
John-Demian Sauer
Igor Sauer
Peter Sauer
Willi Sauerbrei
Andreas Sauerbrei
Alexis Sauer-Budge
Ola Saugstad
Jenny Sauk
Katherine Saul
Simon Saule
Denis Saulnier
Jean-Sébastien Saulnier-Blache
Philippa Saunders
Rhodri Saunders
Norman Saunders
Travis Saunders
Thomas Saunders
Bernadette Saunders
Darren Saunders
Timothy Saunders
Michael Saunders
Manu Saunders
Ashley Saunders
Patricia Saunders-Hao
Rachel Saunders-Pullman
Yogen Saunthararajah
Amy Saupe
Dieter Saur
Josep Saura
Thomas Sauter
Daniel Sauter
Craig Sauter
Christopher Sauvage
Alain Sauvanet
Anthony A. Sauve
Laura Sauve
Nathalie Sauvonnet
Peter Savadjiev
Anna Savage
David Savage
Sara Savage
Christina Savage
Lisa Savage
Dennis Savaiano

Niramol Savaraj
Serge Savary
Alfonso Savastano
Cosmin Saveanu
Barry Saver
Stephen Saville
Paul Saville
William Saville
Jonathan Savin
Olga Savinova
Handanahal Savithri
Alexander Savitsky
Vuk Savkovic
Carmine Savoia
Jean-Michel Savoie
Eriika Savontaus
Jelena Savovic
Yoland Savriama
Anca Savulescu
George Savva
Renos Savva
Hitoshi Sawa
Akira Sawa
Hirofumi Sawa
Yoshihiro Sawa
Zimi Sawacha
Naoki Sawada
Ikumi Sawada
Kenichi Sawada
Tetsuji Sawada
Akira Sawada
Amr H. Sawalha
Yvonne Sawall
Kazunobu Sawamoto
Mohamad Sawan
Samir Sawant
Devendra Sawant
Michael Sawaya
Ricardo Sawaya
Stephen Sawcer
Robert Sawers
S.J. Sawiak
Grzegorz Sawicki
Kenneth Sawin
Birgit Sawitzki
Nathaniel Sawtell
Nancy Sawtell
Michael Sawyer
Hall Sawyer
Alexandra Sawyer
Alyssa Sawyer
Dov F. Sax
Shailendra K Saxena
Anjana Saxena
Smita Saxena
Lynora Saxinger
Andrew Saxon
Tamsin Saxton
Yee-How Say
Ahmed Sayadi
David Sayah
Okay Saydam
Abdelrahman Sayed
Blayne Sayed
Mohamed Sayed-Ahmed
Aejaz Sayeed
John Sayer
Ian Sayers
Ayca Sayi-Yazgan
Gregory Sayuk
Eleni Sazakli
Daniele Sblattero
Andrea Sboner
Cristiana Sbrana
Paola Sbriccoli
Patrizia Sbriglia
F. Scaglia
K. Scaglione
Simona Scaini
Stefania Scala
Antonio Scala
Enrico Scala
Simone Scalabrin
Elisa Scalabrin
Enrico Scalas
I.C. Scaletsky
Monica Scali
Joshua Scallan
Andy Scally
Giancarla Scalone
Andrea Scaloni
Maurizio Scaltriti
Peter Scanlon
Martin Scanlon
Frank Scannapieco
Morris Scantlebury

Claire Scantlebury
Giuseppe Scapigliati
Sandra Scapin
Guido Scarabelli
Patricia Scaraffia
Tais Scaramucci
Andrea Scaramuzza
Fábio Scarano
Peter Scarborough
Giuliano Scarcelli
Damian Scarf
Giovanna Scarfone
Joy Scaria
Simone Scarlata
Vincenzo Scarlato
Gabriella Scarlatti
Eugenio Scarnati
Maurizio Scarpa
Vera Margarete Scarpassa
Cristina Scarpazza
Samuel Scarpino
C. Margaret Scarry
Rodrigo Scattone
Paola Scavone
Simon Scerri
H. Martin Schaaf
Anne Schaafsma
Sara Schaafsma
Heiner Schaal
Beatriz Schaan
Pauline Schaap
Matthew Schabath
Jodee Schaben
Paul Schaberg
Till Schäberle
Wolf Schäbitz
Anja Schablon
Manuel Schabus
Joseph Schacherer
Emma Schachner
Gregson Schachner
Adena Schachner
Sven Schade
Andreas Schadschneider
Michael Schaefer
H. Martin Schaefer
Arne Schaefer
Beat Schaefer
Liliana Schaefer
Sabine Schaefer
Saul Schaefer
Jerremy Schaefer
Anthony Schaeffer
Nicolas Schaeffer
Robert Schaeffer
Scott Schaeffer
Walter Schaeken
Joanna Schaenman
Yolanda Schaerli
Roland Schaette
Christa Schafellner
Ingmar Schafer
Karina Schafer
Zach Schafer
Karl-Herbert Schafer
Sabrina Schafer
Johanna Schafer
Marissa Schafer
Andreas Schäfer
Thorsten Schäfer
Thomas Schäfer
Daniela Schäfer
Matthias Schäfer
Jennifer Schaff
David Schaffer
Andrea Schaffer
Kirsten Schaffer
Bruce Schaffer
Erik Schäffer
Donald Schaffner
Steven Schafrik
Undraga Schagdarsurengin
Ulrich Schaible
Helmut Schaider
Enrico Schalk
Jack Schalken
Casper Schalkwijk
Joseph Schall
Teilo Schaller
Michael-Paul Schallmo
Nils Schallner
Karin Schallreuter
Renata Schama
Andrea Schamberger
Marie-Claire Schanne-Klein
Alessandra Schanoski

Dena Schanzer
Daniel Schardosim Calovi
Christopher Scharer
Michael Scharf
Birgit Scharf
Constance Scharff
Walter Ernesto Schargel
Mathias Scharinger
Michael Scharl
Benedikt Scharnagl
Andrea Scharnhorst
Robert Scharpf
Marion Scharpfenecker
Joern Scharsack
Volker Schartinger
Manfred Schartl
Helmut Schaschl
Heather Schatten
Jörn Schattenberg
A. Schattner
David Schatz
Bertrand Schatz
Michael Schatz
Hermann Schätzl
Michael Schaub
Gunter Schaub
Monika Schaubeck
Douglas Schaubel
Jürgen Schauber
Sascha Schäuble
Roland Schauer
Gabriele Schaumann
F. Schaumburg
Robert Schaut
Nils Schebb
Alan Schechter
Adrienne Scheck
David Scheel
Troels Scheel
Pauline Scheelbeek
Dirk Scheele
Hubertina Scheepers
Frank Scheer
Justin Scheer
Rene Scheeringa
Hans Scheers
Todd Scheetz
Dirk-Jan Scheffers
Juergen Scheffran
Susanne Scheibe
Andrew Scheibe
Carmen Scheibenbogen
Morten Scheibye-Knudsen
Marion Scheider
Ricarda Scheiner
Mindy Scheithauer
Anne Schel
Michael Schell
Lawrence Schell
Harriët Schellekens
Huub Schellekens
E. Glenn Schellenberg
John Schellenberg
Samuel Schellenburg
Henrik Scheller
Jeffrey Schelling
Martin Schels
Mark Schembri
Erik Scheme
Mirle Schemionek
Francesco Schena
Leonardo Schena
Martin Schencking
Simon Schenk
Peer Schenk
Sergio Schenkman
Jean Schensul
Nicolien Schepers
Inga Maren Schepers
Victor Schepkin
Andre Scherag
Rachel Scheraga
Stefan Scherbaum
Arnaud Scherberich
Steven Scherer
Guenther Scherer
Philipp Scherer
Reinhold Scherer
Ronald Scherer
Laura Scherer
Stefan Scherer
Luciene Scherer
Randall Scheri
Sicco Scherjon
Fulco Scherjon
Rhoda Scherman

Carol Schermer
Gerit Schernthaner
Alexandra Scherrer
Benoit Scherrer
Marielle Scherrer-Crosbie
Kurt Schesser
Marc Schetelig
F. Schettini
Giuseppe Schettino
Stefanie Scheu
Todd Scheuer
Karine Scheuermaier
Dietram Scheufele
Marina Scheumann
Simon Scheuring
Ben Scheven
Lawrence Scheving
Lawrence Schiamberg
Benedeta Schiavetti
Giampietro Schiavo
Marion Schiavone
Martin Schicht
Vanessa Schick
Udo Schickhoff
Karin Schiecke
Bernhard Schieffer
Drew Schield
William Schiemann
Cordelia Schiene-Fischer
Günter Schiepek
Alexander Schier
Kristina Schierenbeck
Carl Schiesser
Matt Schiewer
Gordon Schiff
Davide Schiffer
Joshua Schiffer
Jarad Schiffer
Dieter Schifferli
Helmut Schiffl
Joshua Schiffman
Ingo Schiffner
Tamara Schikowski
Nathan Schilaty
Karin Schilbach
Gernot Schilcher
Stefan Schild
Laurent Schild
Anne Schild
Hannah Schildberg-Hörisch
Jonathan Schildcrout
Matthijs Schilder
Mark Schildhauer
Caroline Schild-Poulter
Daniela Schiller
Thomas Schilling
Karl Schilling
Oliver Schilling
Arndt Schilling
Tobias Schilling
Martin Schilling
Jonathan Schilling
Maarten Schim van der Loeff
Lisa Schimmenti
Aaron Schimmer
Victor Schinazi
Aaron Schindeler
Christian Schindelhauer
Daniel E. Schindler
Karin Schindler
Stefan Schindler
Sheila Schindler-Ivens
Rafaela Schinegger
Valérie Schini-Kerth
Bernhard Schink
Thorsten Schinke
Tiziana Schioppa
Ernestina Schipani
Matthew Schipma
Louis Schipper
Marc Schipper
Axel Schippers
Uwe Schippmann
Chiara Schiraldi
Jan Schirawski
Michael Schirmacher
N.F. Schirmbeck
Annett Schirmer
Kristin Schirmer
Clemens Schirmer
Lyn-Rouven Schirra
Thomas Schirrmann
Sofia Schiza
Natalia Schlabritz-Lutsevich
Thomas Schlacher
David Schlaepfer

Florian Schlagenhauf
Stefan Schlager
Luregn Schlapbach
Markus Schläpfer
Verena Schlaphoff
Amber Schlater
Stefan Schlatt
Uwe Schlattner
Robert Schlauch
Elizabeth Schlaudecker
Gottfried Schlaug
Jnrgen Schlegel
Todd Schlegel
Florence Schleich
Ulrike Schleicher
Frank-Michael Schleif
Enrico Schleiff
Dorit Schleinitz
Nicolas Schleinitz
Mark Schleiss
Christian Schlenstedt
Georg Schlieper
Patrick Schlievert
Ulrich Schliewen
Fernando Schlindwein
Barbara Schlingmann
David Schlipalius
Andreas Schlitzer
Mathias Schlogl
Arran Schlosberg
Karen Schloss
Michael Schloter
Nadine Schlueter
Andreas Schlüter
C. Schmaderer
Alvin Schmaier
Kathleen Schmainda
Achim Schmalenberger
Karen Schmaling
Gerd Schmalisch
Eric Schmelz
Denes Schmera
Leopold Schmetterer
Christoph Schmid
Amy Schmid
Florian Schmid
Erwin Schmid
Thomas Schmid
Axel Schmid
Kara Schmid
Stefan Schmid
Simon Schmidbauer
Philippe Schmidely
Robert Schmidhammer
Geert Schmid-Schoenbein
M. Schmidt
Marc Schmidt
Gudula Schmidt
Konrad Schmidt
Martin Schmidt
Herbert Schmidt
Barbara Schmidt
Martina Schmidt
Nathan Schmidt
Stacy Schmidt
Benedikt Schmidt
Laura Schmidt
Peter Schmidt
Bela Schmidt
Enno Schmidt
Daniela Schmidt
Antje Schmidt
Boris Schmidt
Joshua Schmidt
Krzysztof Schmidt
Christopher Schmidt
Robert Schmidt
Eric Schmidt
Walter Schmidt
Ralph Schmidt
Alexander Schmidt
Morten Schmidt
Anne Schmidt
Heath Schmidt
Ty Schmidt
Michael Schmidt
Wiebke Schmidt
Kerstin Schmidt
Burkhard Schmidt
Artur Schmidtchen
Ursula Schmidt-Erfurth
Peter Schmidtke
Heike Schmidt-Posthaus
Dietrich Schmidt-Vogt
Miriam Schmidt-Wack
Carsten Schmidt-Weber

Klaus Schmierer
Brian Schmit
Vincent Schmithorst
Daniel Schmitt
Anthony Schmitt
Ulrich Schmitt
Fernando Schmitt
Heike Schmitt
Christine Schmitt
Lutz Schmitt
Antje Schmitt
François Schmitt
David Schmitt
Sebastian Schmitter
Marc Schmitter
Thomas Schmittgen
Michel Schmitt-Ney
Gerold Schmitt-Ulms
Oswald Schmitz
M. Lienhard Schmitz
Gerd Schmitz
Ruth Schmitz
Jörn Schmitz
Volker Schmitz
Lars Schmitz
Norbert Schmitz
Ulf Schmitz
Alexander Schmitz
Silke Schmitz
Florian Schmitz
R Schmitz
Berndt Schmitz-Drager
Bernd Schmitz-Dräger
Stephan Schmitz-Esser
Tanja Schmitz-Hübsch
Carsten Schmitz-Peiffer
Mirco Schmolke
Monika Schmoll
Gm Schmolzer
Jan Schmoranzer
Michael Schmueck-Henneresse
Michael Schmuker
Michaela Schmull
Gabriel Schmunis
Sheila Schmutz
Andrew Schnabel
Renate Schnabel
Uta Schnabel
H. Schnaper
Fabiana Schneck
Jörg Schnecker
Daniel Schneditz
Christian Schnedl
Korbinian Schneeberger
Marc Schneeberger
Stefan Schneeberger
Gerald Schneeweiss
Hanna Schneeweiss
David Schneider
Bernard Schneider
Jutta Schneider
Tanja Schneider
Sabine Schneider
Ian Schneider
Gisbert Schneider
Jurgen Schneider
Maria Cristina Schneider
Barbara Schneider
Erich Schneider
Kristan Schneider
Marielle Schneider
Jay Schneider
Sven Schneider
Helen Schneider
Hans Gerhard Schneider
Marcela Schneider
Torben Schneider
Carlos Schneider
Dana Schneider
Marlon Schneider
Frederic Schneider
Francine Schneider
Tomasz Schneider
Andreas Schneider
Stefan Schneider
Marc Schneider
Gary Schnell
Bernhard Schnetger
Esther Schnettler
Karin Schnetz
Roman Schniepp
Susanne Schnittger
Christine Schnitzler
David Schnoerr
Joseph Schober
Michael Schober

Barbara Schober
Patrick Schober
Wolfgang Schobersberger
Michael Schocke
Dagmar Schoder
Martin Schoeberl
Dale Schoeller
Ludger Schoels
James Schoelz
David Schoema
Corrie Schoeman
Brad Schoenfeld
Paul Schoenhagen
Timm Schoening
Margaret Schoeninger
Stefan Schoenland
Antoinette. Schoenthale
Randal Schoepp
Anja Schoeps
Anna Schoettle
Christopher Schofield
Timothy Schofield
Gail Schofield
Pamela Schofield
Andrew Schofield
Gabriele Schoiswohl
Dirkjan Schokker
Sebastian Schölch
Amy Scholik
Felix Scholkmann
Francisco Scholl
Stephanie Schollaert Uz
Steffen Scholpp
H. Steven Scholte
Denise Scholtens
Herman Scholthof
Holger Scholz
Glen Scholz
Michael Scholz
Lutz Schomburg
Karin Schon
Chris-Carolin Schön
Daniele Schön
Jennifer Schön
Christian Schönbach
David Schonberg
Torsten Schöneberg
Michael Schönenberg
Gregor Schoner
Dustin Schones
Kai Schönig
Felix Schönrath
Tim Schoof
Robert Schooley
C. Mary Schooling
Ethan Schoolman
Erwin Schoonderwaldt
Menno Schoonheim
Jon Schoonmaker
Veronika Schöpf
Francisco Schopfer
Doris Schopper
Nestor Schor
Allan Schore
John Schorge
Melanie Schori
Dominik Schori
Gregory Schorr
Benedikt Schoser
Alexandra Schosser
Evert Schothorst
Michael Schotsaert
Eline Schotsmans
Björn Schott
Ulf Schott
Heiko Schotte
Elizabeth Schotter
Leo Schouls
Jennifer Schrack
Elizabeth Schrack
Jürgen Schrader
Laura Schrader
Lars Schrader
Stephanie Schrag
Matthew Schrager
Arthur Schram
Maarten Schrama
Adrien Schramm
Oana Schramm
Michael Schramm
Michaela Schratzberger
Michael Schredl
Gideon Schreiber
Rainer Schreiber
Renate Schreiber
Barbara Schreiber

Michael Schreiber
Karin Schreiber
Günter Schreier
Nadja Schreier
Harold Schreier
Harald Schrem
Michiel Schreuder
Nicola Schreurs
Joseph Schrevel
Jonas Schreyögg
Lynn Schriml
Claudia Schrimpf
Jan Schripsema
Brian Schrire
Nadja Schroder
Ulrich Schröder
Helmut Schröder
Katrin Schröder
Kathrin Schrödl
Jens Schroeder
Hannes Schroeder
Julia Schroeder
Bjoern Schroeder
Henri Schroeder
Indra Schroeder
Lee Schroeder
Thies Schroeder
F. Schroeper
Tracy Schroepfer
Maya Schroevers
Erich Schröger
Jeppe Schroll
Gerhard Schroth
Martine Schroyen
Peter Schu
Ingo Schubert
Florian Schubert
Veit Schubert
Andras Schubert
Thomas Schubert
Jonathan Schubert
Martin Schubert
Edward Schuchman
Nicolas W. Schuck
Sabrina Schuck
Rudi Schuech
Stephan Schuele
Georg Schuele
Ora Schueler-Furman
Oliver Schuelke
Tobias Schuerholz
Andreas Schuering
David Schuermann
Wiebke Schuett
Katharina Schuett
Paul Schuette
Michael Schuetz
Martin Schuetz
Norbert Schuff
Stephan Schug
Kevin Schug
Klaus Schughart
Jane Schuh
Michael Schuhmann
Frans Schuit
Ewoud Schuit
Nathan Schuld
Andreas Schuldt
Cornelius Schüle
Steffen Schüle
Ben Schuler
Krysten Schuler
Hannes Schuler
Gerhard Schuler
Hildegard Schuller
Kathy Schuller
Brenda Schulman
Carl Schulman
Ira Schulman
Martin Schulman
Anne Schulp
Bruce Schulte
Lisa Schulte
Erica Schulte
Christian Schulte
Gunnar Schulte
Rogier Schulte
Ulrich Schulte
Karina Schulte
Albrecht Schulte-Hostedde
Wilhelm Schulte-Mattler
Michael Schulte-Mecklenbeck
Patrick Schultheiss
Constance Schultsz
Marcus Schultz
Tom Schultz

Joachim Schultz
Wesley Schultz
Michael Schultz
David Schultz
Krisann Schultz
Hd Schultz
Jeffrey Schultz
Eric Schultz
Stacey Schultz-Cherry
Daniel Schultze
Zach Schultzhaus
Reiner Schulz
Wolfgang Schulz
Peter Schulz
Rüdiger Schulz
David Schulz
Robert Schulz
Richard Schulz
Alexander Schulz
Rainer Schulz
Margot Schulz
Jan-Niklas Schulz
Carla Schulz
Michael Schulz
Laura Schulz
Sabine Schulz
Ernst-Detlef Schulze
Almut Schulze
Thomas Schulze
Mark Schulze
Manuel Schulze
Arne Schulze
Julian Schulze Zur Wiesch
Gundula Schulze-Tanzil
Sven Schulzke
Tanja Schulz-Mirbach
Fredrick Schumacher
Maria Schumacher
Anne Schumacher
Stefan Schumacher
Johannes Schumacher
Mark Schumacher
Nathan Schumaker
Joel Schuman
Michael Schumann
John Schuna
Heribert Schunkert
Gertraud Schüpbach-Regula
Marcus-Oliver Schupp
Jonas Schupp
Hans-Christian Schuppe
Markus Schuppler
Nick Schurch
Mark Schurdak
Timo Schürg
Jan Jacob Schuringa
Michael Schurr
Benjamin Schusser
Stefan Schuster
Alexander Schuster
Carolin Schuster
Christoph Schuster
Mathieu Schuster
Brian Schutte
Stefan Schütz
Alexander Schütz
Christian Schütz
Nadine Schuurman
Noemi Schuurman
Carol Schuurmans
Tobias Schuwerk
Jan Schwab
Yannick Schwab
Helmut Schwab
Lars Schwabe
Justus Schwabedal
Martin G Schwacha
Lori Schwacke
Andrew Schwaderer
Wilhelm Schwaeble
Juerg Schwaller
Jon-David Schwalm
Sabine Schwamberger
Jens Schwamborn
Ralf Schwanbeck
Markus Schwaninger
Jason Schwans
Robert Schwarcz
Henry Schwarcz
Olivier Schwartz
Eli Schwartz
Elisabeth Schwartz
Gary Schwartz
Larry Schwartz
Joel L. Schwartz
Robert Schwartz

Steven Schwartz
Janice Schwartz
Jeremy Schwartz
Barbara Schwartz
Stanley Schwartz
Sheree Schwartz
Noa Schwartz
Joseph Schwartz
Myron Schwartz
John Schwartz
Andrew Schwartz
Reinhard Schwartz-Albiez
Judith Schwartzbaum
M. Schwarz
Edward Schwarz
Ulrich Schwarz
Guenter Schwarz
Norbert Schwarz
Adam Schwarz
Thomas Schwarz
Stefan Schwarz
Jodi Schwarz
Kathleen Schwarz
Julie Schwarz
Emanuel Schwarz
Christoph Schwarz
Flavio Schwarz
Karlheinz Schwarz
Sascha Schwarz
Jaclyn Schwarz
Jennifer Schwarz
Jean Schwarzbauer
Jürgen Schwarze
Guido Schwarzer
Adrian Schwarzer
Michael Schwarzer
Dietrich Schwarzkopf
Larissa Schwarzkopf
Thomas Schwarzl
Markus Schwarzlander
Kathy Schwehr
Michal Schweiger
Jake Schweigert
Nicolas Schweighofer
George Schweitzer
Reinhard Schweitzer-Stenner
Ulrich Schweizer
Tom Schweizer
Falk Schwendicke
Marc Schweneker
Friedhelm Schwenker
Aleksandra Schwenk-Ferrero
Christian Schwentner
Lukas Schwentner
Angela Schwering
Christian Schwerk
Kathryn Schwertfeger
Andreas Schwertfeger
Ferdinand Schweser
Caspar Schwiedrzik
Jim Schwiegerling
Andreas Schwiertz
Andiara Schwingel
Andreas Schwingshackl
Susanne Schwinning
Amanda Schwint
Amee Schwitters
Jean Paul Schwitzguebel
Steven Schwulst
Joost Schymkowitz
Virginia Sciacca
Michele Sciacca
Angela Sciacqua
Roberto Sciagrà
Denisse Sciamarella
Sebastiano Sciarretta
Andrea Sciarretta
Emma Sciberras
Marija Sciberras
Nicola Scichilone
Cristina Scielzo
Franco Scinicariello
Giuseppe Scionti
Raffaele Sciorsci
Alessandra Sciutti
Robert Sclafani
Anthony Sclafani
Valentina Sclafani
R. Hal Scofield
Peter Scogings
John Scolaro
Glen Scoles
Giancarlo Scopettuolo
Diana Scorpio
Marco Scortichini
Valeria Scortichini

Antonella Scorziello
Federico Scossa
Kristin Scott
Jeffrey Scott
Christopher Scott
Andrew Scott
Mark Scott
Bill Scott
David Scott
Maxwell Scott
Robert Scott
Suzanne Scott
Rebecca Scott
Melanie Scott
Daryl Scott
Stuart Scott
John Scott
Ian Scott
Paul Scott
Dawn Scott
Stephen Scott
Donald Scott
Paul T. Scott
Harvey Scott
Ryan Scott
Nicole Scott
G. Richard Scott
Evan Scott
Karen Scott
James Scott
Clare Scott
Veras Scott
Graham Scott
Hyman Scott
David Scott,
Marcelo Scotta
Emma Scotter
Claudia Scotti
Lorenza Scotti
Marco Scotti
Riccardo Scotti
Thomas Scott-Phillips
Nicholas Scott-Samuel
Ivana Scovassi
Robert Scragg
Katherine Scranton
Thomas Scriba
Kim Scribner
Richard Scribner
Marie Scully
Eileen Scully
Erin Scully
Nicholas Sculthorpe
Nicholas Scurich
Marco Scutari
Angelo Scuteri
Charles Scutt
Greg Scutt
Prodromos Sdiropoulos
Eric Seaberg
André Seabra
Stephen Seah
Rebecca Seal
Judith Sealy
Matthew Seaman
Giorgio Seano
Ana Seara Cardoso
Dennis Searcy
Kate Searle
Dorothy Sears
Daniel Seaton
Elaine Seaver
Natalie Sebanz
Laura Sebastiá
Maria-Teresa Sebastià
Alexandra Sebastian
Siby Sebastian
Giada Sebastiani
Luca Sebastiani
Federico Sebastiani
Guido Sebastiani
Yuri Sebastião
Mireille Sebbag
Florent Sebbane
Mustapha Sebbane
Joy Sebe
Peter Sebo
Adriano Sebollela
R. Secades-Villa
Eduardo Secchi
Paola Secchiero
Teresa Maria Seccia
David Secco
Thomas Secher
Niels Secher
Annalisa Sechi

Roger Seco
Timothy Secomb
Chris Secombes
Agnese Secondo
Ahmad Sedaghat
Philip Seddon
Christopher Seder
Lauriane Sèdes
Lisa Sedger
Mamdouh Sedhom
James Sedinger
Brian Sedio
Ruth Sedlak
William Sedley
Martin Sedlmair
Peter Sedlmayr
Robert Sedmak
Violaine See
Alfred See
Paul Seear
Michael Seear
J. E. Seeb
Hannah Seeba
Hanno Seebens
B. Seeber
Marco Seeber
Jens Seeberg
Patrick Seed
Amanda Seed
Michael Seeds
Teresa Seefeldt
Christian Seegelke
Christoph Seeger
Werner Seeger
Michael Seeger
Martin Seeger
Valerie Seegers
Ole Seehausen
Claudine Seeliger
Dominik Seelow
Ego Seeman
Torsten Seemann
Klaus Seemann
Barry Seemungal
Victoria Seewaldt
Michael Sefton
Mark Segal
Gad Segal
Cristina Segalin
Jeffrey Segall
Simon Segar
Alfons Segarra
James Segars
William Segars
Ludovica Segat
Bo Segerman
Ronen Segev
Udi Segev
G Segev
Chiara Seghieri
Nereo Segnan
Michel Segonzac
Ayellet Segrè
Luca Segreti
Philippe Séguéla
Cheryle Séguin
Ana Segura
Remedios Segura
Angel Segura
Miguel Segura
Cristina Segura-Garcia
Laure Segurel
Deepmala Sehgal
Mary Sehl
Frantisek Sehnal
Archana Sehrawat
Brad Seibel
Markus Seibel
Sebastian Seibold
Leonard Seibold
John Seibyl
Andrada Seicean
George Seidel
Philipp Seidel
Matthias Seidel
Rachel Seidler
Michael Seidman
J. Seidman
Mark Seielstad
Therese Seierstad
Alexander Seifalian
Erin Seifert
Frank Seifert
Ludovic Seifert
Marva Seifert
Vanadin Seifert-Klauss

Moritz Seiffert
Ali Seifi
Abiy Seifu
David Seigler
Tom Seijkens
Guillermo Seijo
Masahiro Seike
Christian Seiler
Gerald Seiler
Iban Seiliez
John Seinfeld
Yutaka Seino
Ken-Ichiro Seino
Maayke Seinstra
Ryan Seipke
Isabel Seiquer
Jochen Seissler
Hervé Seitz
Jochen Seitz
Frank Seitz
Daniel Seitz
Dallas Seitz
Susana Seixas
Goncalo Seixas
Marcel Seiz-Rosenhagen
Ramalingam Sekar
Padmapriya Sekar
Bülent Sekerel
Konjeti Sekhar
Rajandeep Sekhon
Ekihiro Seki
Naohiko Seki
Tsugio Seki
Motoaki Seki
Atsushi Sekiguchi
Masakazu Sekijima
Akira Sekikawa
Miho Sekimoto
Osamu Sekine
Adekemi Sekoni
Michael Sela
David Sela
Hanan Sela
Gloria Selabe
Erik Selander
Pål Selbo
Peter Selby
Duygu Selcen
Paul Selden
Thomas Selden
Yvonne Selecki
Mona Selej
Majlinda Selenica
Jana Selent
Ivan Selesnick
Matt Self
William Self
Jacob Selhub
Emre Seli
Irena Selicharová
Herve Seligmann
Vitaly Selivanov
Galina Selivanova
Ana Maria Sell
Henrike Sell
Francesco Sella
Adnane Sellam
Jacobo Sellares
Roberta Sellaro
Finn Sellebjerg
Lorenz Sellin
Manuela Sellitto
Michael Sellix
Thorsten Selmer
Carlo Selmi
Laura Selmic
Devarshi Selote
Joshua Selsby
Michael Selsted
Andrei Seluanov
Gennaro Selvaggi
Gopalan Selvaraj
Senthil Selvaraj
Dinesh Selvarajah
Rangaraj Selvarangan
Linda Selvey
Katherine Selwood
Markus Selzner
Daniel Sem
Jonathan Sembrano
Christina Semeniuk
Alexander Semenov
Svetlana Semenova
Gregg Semenza
Jan Semenza
Francesco Semeraro

Marc Semigran
Gün Semin
Elena Seminati
Ornella Semino
Thomas Semkow
Matthew Semler
Ray Semlitsch
Ben Semmekrot
Brice Semmens
Oliver Semmes
Torsten Semmler
Roshanak Semnani
Zhaleh Semnani-Azad
Marie Sémon
José Sempere
Lorenzo Sempere
Sean Semple
Susan Semple-Rowland
Grégory Sempo
Makeda Semret
Á.F. Semsei
Serafima Semyenova
Subrata Sen
Chandan Sen
Shamik Sen
Pradip Sen
Suvajit Sen
Shaunak Sen
Priyankar Sen
Aditya Sen
Urmimala Sen
Utpal Sen
Pritha Sen
Sevket Sen
S. Sen
Payel Sen
Sayan Sen
Emily Sena
David Senaeve
Mirna Senaha
Shantibhusan Senapati
Gerald Sendlhofer
Marcelo Seneda
Makbule Senel
Emilie Sénèque
Roberto Senesi
Aruni Seneviratna
Christina Senft-Batoh
Piseth Seng
Tan Tiow Seng
Takeshi Senga
Yukiko Senga
Manju Sengar
Ryan Senger
Laddawan Senggunprai
Guerkan Sengoelge
Sarah Sengstake
Anirvan Sengupta
Sadhak Sengupta
Prabuddha Sengupta
Neelanjana Sengupta
Srikumar Sengupta
Jyoti Sengupta
Debanti Sengupta
Utpal Sengupta
Souvik Sengupta
Filip Šenigl
Peter Senior
Satoru Senju
John Senko
Helen Senn
Irene Senna
Nathan Senner
E. Senneville
Florian Sennlaub
Christie Sennott
Takeharu Seno
Ladislav Šenolt
Maria Sensen
Paulo Cesar Sentelhas
Phil Senter
Sirisha Senthil
Muthappa Senthil-Kumar
Sang Beom Seo
Keun Seok Seo
Sang Heui Seo
Min Seo
Hak Soo Seo
Sangwon Seo
Jang-Kyun Seo
Han-Seok Seo
Young Su Seo
Ho Kyung Seo
Naohiro Seo
Ho Seong Seo
Yeon Seok Seo

Ssang-Hee Seo
Dong-Woo Seo
Jose A. Seoane
Patricia Seoane-Collazo
Jae Young Seong
Je Kyung Seong
Moon-Woo Seong
Chun Seow
Frances Separovic
Kristina Sepcic
Romina Sepe
Tuul Sepp
Miia Seppänen
Christophe Seppey
Seth Septer
Edwards Septimus
Endang Septiningsih
Jorge Sepulcre
Nuno Sepulveda
Ana Sepulveda
Fabiola Sepulveda
Andrea Sequeira
Bozena Sera
Rodrigo Serafim
Gianluca Serafini
Nick Serão
Rudolph Serbet
Magdalena Sereda
Tamas Seres
Chanuki Seresinhe
Laszlo Seress
Irini Sereti
Benjamin Serfas
Albrecht Serfling
Inna Serganova
Arnauld Sergé
Nicolas Sergeant
Mark Sergeant
Kjell Sergeant
Evan Sergeant
Stylianos Serghiou
Consolato Sergi
Manuel Sergi
Gianluigi Sergiacomi
Che Serguera Serguera
Guido Serini
Andrea Serino
Natalie Serkova
Walter Sermeus
Nathalie Sermondade
Ignacio Seropian
Ann Séror
Ary Serpa Neto
James Serpell
Fabio Serpiello
Esteban Serra
Raffaele Serra
Laura Serra
Ja Serra Rexach
Patricia Serradas
María Serrano
Jose Serrano
Carlos Serrano
Emmanuel Serrano
Oscar Serrano
Antonio Serrano
Emmanuel Serrano Ferron
Carlos Serrano-Cinca
Ignacio Serrano-Pedraza
Ester Serrao
Jose Eduardo Serrao
Mariano Serrao
Jordi Serrats
Frank Serrecchia
Bryan Serrels
Alan Serrels
Margrethe Serres
Gregor Sersa
Francesco Serti
Elisavet Serti
Erdim Sertoglu
Palle Serup
Vito Servedio
Denis Servent
Susan Service
Gaetano Serviddio
Gaetano Servidido
Bertrand Servin
Joan Servitja
Thomas Serwold
Thea Sesardic
Guillaume Sescousse
Chetan Seshadri
Vasudevan Seshadri
Mukund Seshadri
Sridhar Seshan

Janakiram Seshu
Ertugrul Sesli
Ane Sesma
Emily Sessa
Donna Session
October Sessions
Ricardo Sesso
Karol Sestak
I. Sestak
Nenad Sestan
David Sester
Giorgio Sesti
F. Sesti
Francesca Seta
Jiraporn Setakornnukul
Mamoudou Sétamou
Tesfaye Setegn
Philip Setel
Ajay Seth
P. Seth
Sanjay Sethi
Varun Sethi
Gaautam Sethi
Rajiv Sethi
Navil Sethna
Vaheshta Sethna
Kandan Sethumadhavan
Praveen Sethupathy
Swaminathan Sethuraman
Peter Setlow
Karen Seto
Shintaro Seto
Michael Seto
Jane Seto
Daiki Setoyama
Claudio Sette
Paola Sette
Ethan Settembre
Francesco Setti
Ahmed Settin
Lori Setton
Bhuvana Setty
Joao Setubal
William Setzer
Torsten Seuberlich
Manfredo Seufferheld
Thomas Seufferlein
Laurent Seugnet
Ruth Seurinck
Sabina Sevcikova
Nick Sevdalis
Jae Sevelius
Maelle Sevellec
Sanja Sever
David Sever
Childerick Severac
Alexandra Séverac Cauquil
Stefano Severi
Valeria Severino
Eva Sevick-Muraca
Carolyn Sevier
Noemí Sevilla
Iker Sevilla
Elena Seviour
Igor Sevostianov
Mary Sewell
Andrew Sewell
David Sewell
Daniel Sewell
Wade Sexton
Amber Sexton
Masud Seyal
Virginia Seybold
Karl Seydel
Cheikh Seye
Seyed Mohammad Seyedmehdi
Hans-Martin Seyfert
Thomas Seyfried
Armin Seyfried
Florian Seyfried
Gregory Seymann
Roger Seymour
Justin Seymour
Thomas Sferra
Petros Sfikakis
Chiarella Sforza
Susanna Sforzini
Pedro Sfriso
Paolo Sgarbossa
Haïtham Sghaier
Andrea Sgoifo
George Sgouros
Gregory Sgueglia
Zhenxia Sha
Hamdy Shaban
Farzin Shabani

Nancy Shackell
Mark Shackleton
Luciana Shaddox
Nickolai Shadrin
Lora Shadwick
Robert Shafer
William Shafer
Leigh Anne Shafer
Nathan Shaffer
Scott Shaffer
Hadi Shafiee
M. Najeeb Shafiq
Anum Shafiq
Rebecca Shaftel
Nigam Shah
Mala Shah
Nirao Shah
Prediman Shah
Raju Shah
Rupal Shah
Neeraj Shah
Payal Shah
Gul Shah
Prakeshkumar Shah
Zahoor Shah
Alok Shah
Ankit Shah
Gulzar Shah
Devendra Shah
Kalpit Shah
Rehan Ali Shah
Dhara Shah
Dilip Shah
Arti Shah
Rashed Shah
Prithvi Shah
Nishant Shah
Santosh Shah
Maya Shaha
Nahid Shahabadi
Payman Shahabi
Shohreh Shahabi
Shai Shaham
Ron Shahar
Yuval Shahar
Forooz Shahbazi
Mohammad-Ali Shahbazi
Hussam Shaheen
Humma Shahid
Muhammad Shahid
Shabbir Shahid
Mohommad Shahid
Mehdi Shahideh
Mohammad Shahnawaz
I. Shahramian
Vahid Shahrezaei
Rifat Shahriyar
Yechiel Shai
Adam Shai
Zahir Shaikh
Sumaiya Shaikh
Fnu Shailesh
Kathy Shair
Abdallah Shaito
Faiyaz Shakeel
Nicholas Shakeshaft
Mehdi Shakibaei
Basheer Shakir
Vikram Shakkottai
Akbar Shakoor
Alex Shalek
Stephen Shalet
Shaher Shalfawi
Sophia Shalhout
Joel Shalowitz
Yuk Sham
G. Shama
Jeffrey Shaman
Reuben Shamir
Mohammed Shamji
Elana Shamji
Rany Shamloul
Shihab Shamma
Judy Shamoun-Baranes
Larissa Shamseer
Shahaboddin Shamshirband
Mohammad Shamsudduha
Simukai Shamu
Yibing Shan
Chunlei Shan
Xueyan Shan
Baoci Shan
Jian Shan
Jingxuan Shan
Yan-Shen Shan
Bin Shan

Jing Shan
A.S. Shan
Letian Shan
Jie Shan
Yan Shan
Gongbing Shan
Catherine Shanahan
Hugh Shanahan
Lilly Shanahan
Fiona Shand
Robin Shandas
Matthew Shane
Andi Shane
Frank Shane
R. Andrew Shanely
Yu Shang
Shaobin Shang
Jing Shang
Hui-Fang Shang
Guofeng Shang
Yilun Shang
Nan Shang
Ce Shang
Ming-Sheng Shang
Limin Shang
Sylvia Shangani
Zhouping Shangguan
Yaniv Shani
Daniel Shank
Esaki M. Shankar
Premlata Shankar
Bhavani Shankar
Jay Shankar
Eswar Shankar
Sunita Shankar
Sunita Shankaran
Asheesh Shanker
Julia Shanks
Erin Shanle
Daryl Shanley
Jenelle Shanley Chatham
Srinivasan Shanmugam
Mala Shanmugam
K. Shanmuganandan
Shobana Shanmugasundaram
Santhanam Shanmughapriya
Oonagh Shannon
Lynne Shannon
Thomas Shannon
Katie Shannon
Jeffrey Shannon
Graeme Shannon
Jerry Shannon
Claire Shannon-Lowe
Tourangbam Shantibala
Saran Shantikumar
Qing Shao
Hongbo Shao
Kwang-Tsao Shao
Jian-Yong Shao
Changliang Shao
Ning-Yi Shao
Rong Shao
Changwei Shao
Xinqing Shao
Guofan Shao
Ying Shao
Fengjing Shao
Junfei Shao
Ming Shao
Lei Shao
Guifang Shao
Dan Shao
Feng Shao
Hui Shao
Junjiong Shao
Yaming Shao
Di Shao
Lin Shao
Michael Shapira
Michal Shapira
Steven Shapiro
Howard Shapiro
David Shapiro
Joseph Shapiro
Daniel Shapiro
David Shapiro-Ilan
Carrie Shapiro-Mendoza
Albulena Shaqiri
Igor Sharakhov
Rajeshwar Sharan
Mohammed Sharawy
Bianca Share
Jafar Sharif
Adnan Sharif
Azim Shariff

Zalilah Mohd Shariff
Salma Shariff-Marco
Nima Sharifi
Vandad Sharifi
Tanaz Sharifnia
Iraida Sharina
Michael Sharkey
Andrew Sharkey
Michele Sharkey
Alexandra Sharland
Elizabeth Sharlow
Kumar Sharma
Rakesh Sharma
Savitri Sharma
Vijay Sharma
Rameshwar Sharma
Yagya Sharma
Manu Sharma
Sherven Sharma
Vishwas Sharma
Cynthia Sharma
Jyotika Sharma
Pawan Sharma
Prince Sharma
Pushkar Sharma
Suresh Sharma
Surendra Sharma
Manoj Sharma
Rohini Sharma
Arun Sharma
Sandeep Sharma
Nutan Sharma
Arati Sharma
Abhishek Sharma
Sunita Sharma
Shyam Sharma
Pradeep Sharma
Namrata Sharma
Rita Sharma
Shvetank Sharma
Mukul (Mike) Sharma
Neeraj Sharma
Vineet Sharma
Aman Sharma
Akshay Sharma
Shilpi Sharma
Tarun Sharma
Blanka Sharma
Ram Sharma
Rajendra Sharma
Amrish Sharma
Shiva Sharma
Ritin Sharma
Pratyush Sharma
Shiv Sharma
Sai Kiran Sharma
Amit Sharma
Rajiv Sharma
Shivali Sharma
Nikhil Sharma
Rajesh Sharma
Mrityunjai Sharma
Ajay Sharma
Sushil Sharma
Shraddha Sharma
Mithum Sharma
Mona Sharma
Shilpa Sharma
Shivani Sharma
Girdhar Sharma
Vaneet Sharma
Sangeeta Sharma
Ashwani Sharma
Sunny Sharma
Abhinav Sharma
Pankaj Sharma
Umender Sharma
Surbhi Sharma
James Sharman
Vidyadaran Sharmili
Md Golam Sharoar
Amir Sharon
Paul Sharp
Paul Sharp
Burt Sharp
Tyler Sharp
Duncan Sharp
Warren Sharp
Claire Sharp
Paul Sharpe
Simon Sharpe
Richard Sharpe
Amanda Sharpe
Adam Sharples
Norman Sharpless
Andrew Sharrocks

Allison Sharrow
Ajit Shasany
Emad Shash
Rangaiah Shashidharamurthy
Swakkhar Shatabda
Robert Shatters
Steven Shattuck
Tan Shau Hwai
Pradeep Shaukla
Philip Shaul
Kimberlee Shauman
Jordan Shavit
Yuri Shavrukov
Pang-Chui Shaw
Albert Shaw
Brian Shaw
Andrey Shaw
James Shaw
Jonathan Shaw
Gary Shaw
Joseph Shaw
Lindsey Shaw
Edward Shaw
Lisa Shaw
Victoria Shaw
Kristi Shaw
David Shaw
Richard Shaw
Elizabeth Shaw
John Shaw
Christiana Shaw
Tanya Shaw
Paul Shaw
Cindy Shaw
Rachel Shaw
Mohamed Shawarby
Matthew Shawkey
Renat Shaykhiev
Halyna Shcherbata
Natalia Shcherbik
Ruiping She
Qing-Bai She
Weifeng She
Stephen Shea
Patrick Shea
Colin Shea
Gregory Shearer
Jane Shearer
Kate Shearer
Keith Shearwin
Andrew Shedlock
Vasu Sheeba
Frederick Sheedy
John P. Sheehan
Vivien Sheehan
Kate Sheehan
Timothy Sheehan
Michael Sheehan
Ann Sheehy
Fangmiin Sheen
H. David Sheets
Sandra J. Shefelbine
Efrat Sheffer
William Sheffield
Cory Sheffield
Sterling Sheffield
Amy Sheflin
Alex Sheftel
Kareem Shehab
Michael Shehata
Wasim Shehzad
Sabir Shehzad
Nader Sheibani
Aubrey Sheiham
Kazim Sheikh
Bilal Sheikh
Haroun Sheikh
M. Sheikholeslami
Douglas Sheil
Orla Sheils
Kiran Shekar
G.S. Shekhawat
Samuel Shelburne III
Kent Shelby
Eric Shelden
Frederick (Fred) Sheldon
Wendy Sheldon
Ekaterina Shelest
Mack Shelley
Druyan Shelly
G Shelton
Anthony Shelton
Noula Shembade
Einat Shemesh Mayer
Ian Shemilt
Lirim Shemshedini

Chen-Yang Shen
Jianhua Shen
Zhi-Qiang Shen
Weijun Shen
Xu Shen
Tang-Long Shen
Li Shen
Xuetong Shen
Liang Shen
Jiantong Shen
Jianfeng Shen
Xiaoying Shen
Jingshi Shen
Wenbiao Shen
Wei Shen
Hui Shen
Chengxing Shen
Jianxiong Shen
Hongmei Shen
Kunwei Shen
Zhenyao Shen
Yufeng Shen
Xihui Shen
Yinzhong Shen
Miaogen Shen
Yuan Shen
Zhangqi Shen
Shihao Shen
Huawei Shen
Zhenguo Shen
Lucy Shen
Lin Shen
Xinlian Shen
Hong Shen
Huanfeng Shen
Kui Shen
Jin-Ming Shen
Dong-Yan Shen
Bo Shen
Yuying Shen
Kezhen Shen
Zehao Shen
Bin Shen
Chwan-Li Shen
Zhesi Shen
Yanguang Shen
Haihong Shen
Wenjiang Shen
Chaofeng Shen
Aimee Shen
Yubang Shen
Xiaoyan Shen
Yihang Shen
Chengyong Shen
Dong Shen
Jia Shen
Zhi Yang Shen
Jiayun Shen
Juan Shen
Yi Shen
Chiayi Shen
Fei Shen
Megan Shen
Zhigang Shen
Yin Shen
Kang-Ning Shen
Yang Shen
Liquan Shen
Dan Shen
Jincheng Shen
Jun Shen
Shihua Shen
Yao Shen
John Shen
Qiang Shen
Xiaoyan Sheng
Yongwei Sheng
Feng Sheng
Changsheng Sheng
Yubang Sheng
Amitai Shenhav
Andrew Shennan
Stephen Shennan
Sheela Shenoi
Shirish Shenolikar
Chetan Shenoy
Avinash Shenoy
Abhineet S. Sheoran
Emily Shepard
Bryan Shepherd
Kennie Shepherd
Trevor Shepherd
Samantha Shepherd
Jason Shepherd
James Shepherd
Donald Sheppard

Christine Sheppard
Karen Sheppard
Paula Sheppard
Lauren Sheppard
Chris Sheppard
Haynes Sheppard
Yuh-Pyng Sher
Nathan Sherer
Robert Sherertz
Brian Sheridan
Nicolette Sheridan
Zaki Sherif
Jawaad Sheriff
Lori Sherlock
Philip Sherman
Michael Sherman
Aleksandra Sherman
Stephanie Sherman
Ryne Sherman
Stanton Keith Shernan
Michelle Shero
Emma Sherratt
Rosemary Sherriff
Tom (Thomas) Sherry
David Sherry
Michael Sherry
Howard Shertzer
Catherine Sherwin
Graham Sherwood
Edward Sherwood
James Sherwood
Tanuja Shet
Prameet Sheth
Ashok K. Shetty
Sreerama Shetty
Amith Shetty
Meei Sheu
Chau-Chyun Sheu
Ming-Thau Sheu
Gwo-Tarng Sheu
Shwu-Jiuan Sheu
Manjunath Shevgoor
Maxim Shevtsov
Hemant Shewade
Patrica Shewmaker
Patricia Shewokis
Weibin Shi
Suhua Shi
Yun-Bo Shi
Lei Shi
Zhengli Shi
Min Shi
Yongshen Shi
Yongyong Shi
Pei-Yong Shi
Yulin Shi
Yuguang Shi
Zhuanghua Shi
Haifei Shi
Feng Shi
Ling Shi
Dongmei Shi
Qing Shi
Liyun Shi
Xianming Shi
Kerong Shi
Weiwei Shi
Huijing Shi
Shaolin Shi
Weiming Shi
Jun Shi
Xianglin Shi
Yufang Shi
Huidong Shi
Rui Shi
Yongtang Shi
Yu Shi
Zhongjie Shi
Yuyan Shi
Hon-Yi Shi
Gongle Shi
Chuan Shi
Tujin Shi
Yanfeng Shi
Kun Shi
Peiqing Shi
Benkang Shi
Yusheng Shi
Zhou Shi
Ainong Shi
Libin Shi
Xiaoli Shi
Anbing Shi
Junping Shi
Chanjuan Shi
Zhumei Shi

Shepo Shi
Yinghuan Shi
Lihui Shi
Guixiu Shi
Hong Shi
Cheng-Ming Shi
Jiangfeng Shi
Jian-Yu Shi
Jianbo Shi
Junfeng Shi
Hang Shi
Peili Shi
Z.H. Shi
Shine-Gwo Shiah
Shin-Hong Shiao
Yugo Shibagaki
Youssef Shiban
Koji Shibasaki
Shigenobu Shibata
Takehiko Shibata
Tatsuo Shibata
Hiroshi Shibata
Masahiko Shibata
Midori Shibata
Junko Shibayama
Youtaro Shibayama
Reiko Shibazaki-Yorozuya
Jamil Shibli
Caroline Shiboski
Akira Shibuya
Yoshinori Shichida
J. Shiea
Chi-Chang Shieh
Adrian Shieh
Tzong-Ming Shieh
Sheau-Yann Shieh
Kun-Ruey Shieh
Kevin Shield
Ryan Shields
Vonnie Shields
Don Shields
J. Shields
Kate Shields
Cleveland Shields
Alan Shiell
Alan Shiels
Paul Shiels
Amde Selassie Shifera
Yohannes Shiferaw
Saul Shiffman
Sakiko Shiga
Hideaki Shiga
Jun Shigemura
Katsumi Shigemura
Kitajima Shigetaka
Kunitoshi Shigeyasu
Shin-Ru Shih
Chiaho Shih
Tiffany Ting-Fang Shih
Vivianne Shih
Neng-Yao Shih
Sophy Shih
Wei-Chuan Shih
Masaaki Shiina
Yumi Shiina
Teiichiro Shiino
Kazuhiro Shiizaki
Jonathan Shik
Ariella Shikanov
Cecilia Shikuma
Daniel Shilhavy
Rebecca Shilling
Lisa Marie Shillito
Richard Shilton
Byoung-Shik Shim
Ju Hyun Shim
Minsub Shim
Hojae Shim
Jae-Jun Shim
Joong-Youn Shim
Michio Shimabukuro
Masayuki Shimada
Michiko Shimada
Takashi Shimada
Nobuo Shimamoto
Kyoko Shimamoto
Masako Shimamura
Hitoshi Shimasaki
Jiro Shimazaki
Taichi Shimazu
Hiroaki Shime
Jeff Shimeta
Takao Shimizu
Hiroshi Shimizu
Noriaki Shimizu
T. Shimizu

Akira Shimizu
Satoshi Shimizu
Yasutake Shimizu
Makoto Shimizu
Katsuhiko Shimizu
Ken Shimizu
Yoshihiro Shimizu
Tatsuya Shimizu
Toshiyuki Shimizu
Yuko Shimizu-Motohashi
Tetsuo Shimmura
Tsuyoshi Shimo
Kazuya Shimoda
Hidetoshi Shimodaira
Takayoshi Shimohata
Yasuyuki Shimohigashi
Kayoko Shimoi
Naoki Shimojima
Naoki Shimojo
Hiroaki Shimokawa
Iichiro Shimomura
Joshua Shimony
Takeshi Shimosato
Tamaki Shimose
Hiroki Shimura
Tsutomu Shimura
Young Kee Shin
Dong Hoon Shin
Eui-Cheol Shin
Aesun Shin
Dong Shin
Sung Jae Shin
Young Joo Shin
Hwa Kyoung Shin
Dong Wook Shin
Heungsoo Shin
Damian Shin
Ho-Joon Shin
Jooho Shin
Su-Ryon Shin
Hoen-Oh Shin
Hyu-Dong Shin
Min-Kyung Shin
Daehwan Shin
Shunichiro Shinagawa
Arti Shinde
Nikki Shindo
Akihiro Shindo
Richard Shine
James Shine
R. Shiner
Prashant Shingate
K Shinlapawittayatorn
Douglas Shinneman
Tetsuro Shinoda
Akira Shinohara
Mari Shinohara
Kazuyuki Shinohara
Yasuaki Shinohara
Toshiharu Shinoka
Shigeru Shinomoto
Youichi Shinozaki
Takahiro Shintani
Miho Shinzawa
Tatsuo Shioda
Ichiro Shiojima
Nobuyoshi Shiojiri
Kozue Shiomi
Masahiro Shiomi
Goshi Shiota
Seiji Shiota
Takahiro Shiotsuki
Stacey Shiovitz
Kazuhiro Shiozaki
Atsushi Shiozaki
Bill Shipley
Martin Shipley
Pat Shipman
Tetyana Shippee
Zachary Shipstead
Ceri Shipton
Ken Shirabe
Hidenori Shiraha
Nobu Shirai
Yuichiro Shirai
Koji Shiraishi
Akihiro Shirakabe
Hisashi Shirakawa
Behrouz Shiran
Tomoaki Shirao
Kenta Shirasawa
Katsuhiro Shiratake
Akiko Shiratsuchi
Yukihiko Shirayama
Karen Shires
Abbas Shirinifard

Ronit Shiri-Sverdlov
Paul Shirk
Pramod Shirke
Susan Shirley
Matthew Shirley
Marina Shirmanova
Eric Shiroma
Yuichiro Shirota
Andrey Shiryaev
Tetsuro Shishido
Sagari, Shitalkumar
Cheng-Shi Shiu
David Shiuan
Ivy Shiue
Yow-Ling Shiue
S. Shivaji
Sisinthy Shivaji
Muthugounder Shivakumar
Amol Shivange
Ahlawat Shivani
Roger Shivas
Vijay Shivaswamy
Kalyanam Shivkumar
Peter Shizgal
Nikoloz Shkriabai
Eli Shlizerman
Amir Shlomai
Maya Shmulevitz
Antony Shmygol
Larissa Shnayder
Saeed Shoar
Yugo Shobugawa
M. Shochat
Ian Shochet
Jay Shockey
Keith Shockley
Charles Shoemaker
Craig Shoemaker
Kevin Shoemaker
Yehuda Shoenfeld
Scott Shofer
David Shoham
Yuval Shoham
Esty Shohami
Jason Shohet
Ali Shojaie
Hideo Shojaku
Maxim Shokhirev
Shadi Shokralla
Wuraola Shokunbi
Lynette M. Sholl
Lauren Shomaker
Yasuhito Shomura
Addmore Shonhai
Nicole Shonka
Suzanne Shontz
Robin Shook
Natalie Shook
Shahin Shooshtari
Bo Shopsin
Steven Shoptaw
Stephanie Shore
Richard Shore
Anna Shore
Stephen Shore
Jesse Shore
Ekaterina Shorohova
Frederick Short
Sarah Short
James Shorter
J. Shortt
Varda Shoshan-Barmatz
Chengchao Shou
Huixia Shou
Guofa Shou
Yogesh Shouche
Samir Shoukry
Harel Shouval
Dror Shouval
Marian Showell
Michael Shoykhet
Nahum Shpigel
David Shprecher
Om Prakash Shrama
Sadeep Shrestha
Sourya Shrestha
Uttam Shrestha
Roman Shrestha
Mani Shrestha
Milan Shrestha
Mark Shrime
Daniel Shriner
Kalpita Shringarpure
Amit Shrira
Indira Shrivastava
Shubham Shrivastava

Ashutosh Shrivastava
Sanjeev Shroff
Rachna Shroff
Martha Shrubsole
Jwu-Ching Shu
Jiang Shu
Chang Shu
Chin Shu
Jun Shu
Qiang Shu
Xiaokang Shu
Xin Shuai
Ling Shuai
Daniel Shub
Veronica Shubayev
Kevin Shufran
Lui Shuhai
Hao-Ai Shui
Jr-Wen Shui
Fukuoka Shuichi
Barbara Shukitt-Hale
Deepak Shukla
Ravi Shukla
Dinesh Shukla
Rakesh Shukla
Diwakar Shukla
Sanjeev Shukla
Shivendra Shukla
Abhay Shukla
Shirish Shukla
Devesh Shukla
Animesh Shukla
Girish Shukla
Rituraj Shukla
Sudhanshu Shukla
Girja Shukla
Surendra Shukla
Garima Shukla
Peter Shull
Lester Shulman
Dorothy Shulman
Julia Shulman
David Shultis
Kato Shum
Jeffrey Shum
Elena Shumskaya
Maria Shumskaya
Martha Shumway
Shiow Ching Shun
Dmitry Shungin
Shibata Shunichi
Michael Shurin
Igor Shuryak
S. Shushruth
Stephen Shuster
Janis Shute
Dave Shutler
Shade Shutters
Show-Ling Shyng
Yu Shyr
Wei Shyy
Ming-Sing Si
Tian-Mei Si
Huaijun Si
Tong Si
Nikolaos Siafakas
Ahmed Siah
Siem Doo Siah
Popluechai Siam
David Siaussat
Matt Sibbald
D. Sibbing
Tennille Sibbritt
Elizabeth Sibert
Maria Sibilia
Carol Sibley
Chris Sibley
Shalamar Sibley
David Sibley
Olivier Sibony
Mathieu Sicard
Pierre Sicard
Nadine Sicard
Wen Sicheng
Frank Sicheri
Laura Sichero
Gabriele Siciliano
Jason Sicklick
Cosmin Sicora
Elisa Sicuri
Souraya Sidani
Nura Sidarus
Basavaraj Siddalingappa
Sridhar Siddharth
Kamran Siddiqi
Hifzur Siddique

Shahid Siddique
Zahra Siddique
Afzal A. Siddiqui
Maqsood Siddiqui
Shadab Siddiqui
M. Rizwan Siddiqui
Mohd Siddiqui
Adnan Siddiqui
Fouzia Siddiqui
Saad Siddiqui
Hannah Siddle
Theodora Sideri
Kuldip Sidhu
Sb Sidhu
Pritam Sidhu
Kiki Sidiropoulou
D. Sidjanin
Emil Sidky
Ludek Sidlo
Ekaterina Sidorchuk
Carlo Sidore
Svetlana Sidorenko
David Sidransky
Estelle Sidze
Daoud Sie
Ulrike Siebeck
Fritz Sieber
Michael Sieber
Christine Sieberg
Nadja Sieber-Ruckstuhl
Ursula Siebert
Paul Siebert
Stefan Siebert
Leslie Sieburth
Derek Sieburth
Gary Sieck
Solvej Siedler
Lynn Siefferman
Scott Sieg
Marc Siegel
Rebecca Siegel
Allan Siegel
S. Siegelaar
Deborah Siegele
Markus Siegelin
Bob Siegerink
Ingo Siegert
Blair Siegfried
Aaron Siegler
Jason Siegler
Claire-Anne Siegrist
Michael Siegrist
Daria Siekhaus
Martin Siemann-Herzberg
Ilias Siempos
Sonja Siennick
Timo Siepmann
Cornelis Sier
Jennifer Sieracki
Frederick Sierles
Eric Siéroff
Helge Sierotzk
Beatriz Sierra
Angels Sierra
Reyes Sierra-Alvarez
Juan Sierra-Madero
Petra Sierwald
Peter Siesjö
Wolfgang Siess
Christina Siettou
Michael Sieweke
Roland Siezen
Jean-Pierre Siffroi
Sibusiso Sifunda
Ron Sigal
Ian Sigal
Luca Sigalotti
Erwin Sigel
Laura Sigg
Trevor Siggers
Robert Siggins
Owen Siggs
Sara Sigismund
Eric Sigler
Mariano Sigman
Curt Sigmund
Eric Sigmund
Margaret Signorella
Luciana Signorelli
Salvatore Signorelli
Giuseppe Signoriello
Dimitri Sigounas
Monica Sigovan
Lara Sigurdardottir
Alice Sigurdson
Einar Sigurdsson

Gardar Sigurdsson
Virginie Siguret
Ólafur Sigurjónsson
Julia Sigwart
Fred Sigworth
Paul Sijens
Alice Sijts
Marianna Sijtsema
Puran Sijwali
Izukanji Sikazwe
Derek Sikes
Bob Sikes
Mile Šikic
Paul Sikkel
Andrew Sikora
Anna Sikorska
Katarzyna Sikorska
Johannes Sikorski
Aleksander Sikorski
Angela Sikorski
Yandisa Sikweyiya
Parames Sil
Cornelia Silaghi
Asli Silahtaroglu
Olin Silander
Philippe Silar
Ariel Silber
Sherman Silber
Gilad Silberberg
Ellen Silbergeld
Jeffrey Silberman
Jordan Silberman
Rebecca Silbermann
Stephen Silberstein
Claudia Silberstein
Lauren Silbert
Mary Silcox
Tadas Sileika
Ulrich Siler
José Siles
Sheri Silfies
Joan Silk
John Silke
Bernard Silke
Heinz Sill
David Sillam-Dussès
Niko Sillanpaa
Neftalí Sillero
Fabiano Sillo
Erin Sills
Miles Silman
Nigel Silman
Michael Silosky
André E. Silva
Eduardo A. Silva
Jerson Silva
Francisco Silva
Matthew Silva
Afonso Silva
Adriana Silva
Susana Silva
João Silva
Denise Silva
Michael Silva
Daniel Silva
Rogerio Silva
Fatima Regina Silva
Roberto Silva
Lucia Silva
Tomé Silva
Jonathan Silva
Tarcilia Silva
Alexandre Silva
Pedro Silva
Marta Silva
Gabriela Silva
Elisabete Silva
Josie Silva
João Renato Silva
Felipe Silva
Floriano Silva
Adrian Silva
Samuel Silva
Maria Silva
Augusto Silva
Denise A.K. Silva
Vania Silva
Margarida Silva
Neuza Silva
Rodrigo Silva
Julie Silva
Danilo Silva
Stefania Cc Silva
Albená Silva
George Silva
Ana Silva

Cristina Silva Pereira
Catarina Silva-Costa
Marcio Silva-Filho
Nicholas Silvaggi
Alessandro Silvani
Juha Silvanto
Cristina Silvar
Rafael Silva-Rocha
Mariana Silveira
Juliana Silveira
Luis Silveira
Patricia Silveira
Fernanda Schmidt Silveira
Gianmaria Silvello
Lynn Silver
Richard Silver
Heidi Silver
Debra Silver
Donald Silverberg
Jonathan Silverberg
M. Caterina Silveri
Michael Silverman
Marni Silverman
Andrea Silverman
Gary Silverman
William Silverman
Kevin Silverstein
Marnie Silverstein
Douglas Silverstein
Peter Silverstone
Jean-Sébastien Silvestre
Jean-Sebastien Silvestre
Romano Silvestri
Laura Silvestri
Franco Silvestris
Nicola Silvestris
Massimo Silvetti
Paul Silvia
Silvia Silvia Lai
Olivier Silvie
Cheolho Sim
Judith Sim
Sergey Simakov
Oleg Simakov
Jesús Simal Gándara
Miguel Angel Simancas Pallares
Amanda Simanek
Charles Simanjuntak
Irina Simanova
Cinira Simao
Marc Simard
Martin Simard
Leickness Simbayi
Kenneth Simbiri
Karen Sime
Jerry Simecka
Télesphore Sime-Ngando
Aline Simen-Kapeu
Charles Simenstad
Carmen Simeon
Marco Simeone
Umberto Simeoni
Dina Simes
Jay Simhan
Tatjana Simic
Domagoj Simic
Filippo Simini
Constantine Simintiras
Maria Regina Lorenzetti Simionato
Dan Simionescu
Paolo Simioni
Warren Simison
Rachel Simister
Maria Simitsopoulou
Siro Simizu
Myrtill Simko
Jeffry Simko
Andreas Simm
Friedrich Simmel
Karen Simmer
Thomas Simmet
Anna Simmonds
Michael Simmonds
Andrea Simmons
Craig Simmons
Mark Simmons
Joe Simmons
Victoria Simms
Patricia Simner
Nelson Simoes
Zilá Simoes
Elisabetrh Simoes
Maria Luisa Simoes
Filipe Simoes
Herbert Simões
Augusto Simoes-Barbosa

Daniel Simola
Scott Simon
Gary Simon
Anne Simon
Michel Simon
Raphael Simon
Arne Simon
Marc Simon
Steve Simon
Nicholas Simon
Corey Simon
Melanie Simon
Noa Simon-Delso
Cristiano Simone
Benedetto Simone
Martin Simoneau
Michael Simone-Finstrom
Martine Simonelig
Joseph Simonett
Geninatti Crich Simonetta
Manuela Simoni
Jane Simoni
Giampaolo Simonini
Gabriele Simonini
Frank Simonis
Anne Simonis
Maria Simonova
Kai Simons
Andrean Simons
Laura Simons
Rachel Simons
Guus Simons
Claudia Simons
Lone Simonsen
Ulf Simonsen
Jacob Simonsen
Marianne Simonsen
Eleanor Simonsick
Uri Simonsohn
Thomas Simonson
Dean Keith Simonton
Kristina Simonyan
Tamas Simor
Peter Simor
George Simos
Jose-Enrique Simo-Ten
Stefan Simovic
Kim Simpfendorfer
Julie Simpson
Wayne Simpson
Angela Simpson
T. J. Simpson
Peter Simpson
Trefford Simpson
Brent Simpson
Shannon Simpson
Nigel Simpson
David Simpson
Jeremy Simpson
Elizabeth Simpson
Michael Simpson
Stephen Simpson
Nuala Simpson
Steven Simpson
Daniel Simpson
PJ Simpson-Haidaris
Peter Sims
Andrew Sims
Suat Simsek
Sunder Sims-Lucas
Laurent Simula
Michelo Simuyandi
Emanuele Sinagra
Anthony Sinai
Roberta Sinatra
Gale Sinatra
Ilya Sinayskiy
Alison Sinclair
Martha Sinclair
Andrew Sinclair
Stephen Sinclair
David Sinclair
Alan Sinclair
Karin Sinclair
Robert Sinden
Suzanne Sindi
Shireen Sindi
Ak Singal
Amit Singal
Kai Singbartl
Sofie Singbrant
Andrew Singer
Michael Singer
Steven Singer
Randall Singer
Harold Singer

George Singer
Philipp Singer
Florian Singer
Alon Singer
Ellen Singer
Benjamin Singer
Neena Singh
Yogendra Singh
Shree Ram Singh
Neeloo Singh
Pradeep Singh
Shiva Singh
Balram Singh
Sarman Singh
Urvashi Singh
Karam Singh
Sandeep Singh
Madhu Singh
Jaswinder Singh
Prashant Singh
Neeru Singh
Sanjeev Singh
Hardeep Singh
Kamlendra Singh
Rajesh Singh
Ajay Singh
Ugra Singh
Narendra Singh
Pankaj Singh
Saurabh Singh
Neetu Singh
Lalit Singh
Divyendu Singh
Shailesh Singh
Bashisth Narayan Singh
Ramandeep Singh
Abhishek Singh
Sanjay Singh
Kiran Singh
Pratibha Singh
Amardeep Singh
Brijesh Singh
Ashok Singh
Anshuman Singh
Rakesh Singh
Mahavir Singh
Balvinder Singh
Sudhir Singh
Sasha Singh
Vir Singh
Ravi Singh
Braj Singh
Harpreet Singh
Mohar Singh
Prachi Singh
Tripti Singh
Vijay Singh
Anil Singh
Sushma Singh
Bhagat Singh
Ankur Singh
Kamaleshwar Singh
Satyendra Singh
Archana Singh
Moganavelli Singh
Aparna Singh
Anne Singh
Meera Singh
Anil Kumar Singh
Rajeev Pratap Singh
Amar Singh
Ruchira Singh
Nandita Singh
R.P. Singh
Ram Singh
Harshpal Singh
Amit Singh
Mewa Singh
Arvind Singh
Lakhan Singh
Rishikesh Singh
Nathan Singh
Tirath Raj Singh
Nagendra Singh
Anup Singh
Poonam Singh
J. Singh
Ravinder Singh
Amareshwar Singh
Anju Singh
Saranjit Singh
Gurbir Singh
Sunil Singh
Shripriya Singh
Nk Singh
Brahmanand Singh

Raj Kumar Singh
Harnam Singh
Chandra Singh
Thakur Gurjeet Singh
Manpreet Kaur Singh
Nirbhay N. Singh
Kuljit Singh
Anurag Singh
J.S. Singh
Mandeep Singh
Kunwar Singh
Sheelendra Singh
Rajvir Singh
Rajendra Singh
Gurpreet Singh (Calgary)
Tarunpreet Singh Virk
Pravin Singhal
Sharad Singhal
Amit Singhal
Prabhat Singhal
Aatur Singhi
Pooja Singhmar
Praful Singru
Saurabh Sinha
Satrajit Sinha
Himanshu Sinha
Sitabhra Sinha
Alok Sinha
Debasish Sinha
Anusha Sinha
Neeraj Sinha
Amit Sinha
Ashish Sinha
Anju Sinha
Sunil Sinha
Anushua Sinha
Abhijit Sinha Roy
Marcello Siniscalchi
Antonio Sinisi
Marianne Sinka
Steven Sinkins
Ralph Sinkus
David Sinn
Dong Hyun Sinn
Scott Sinnett
Bharathalingam Sinniah
Judith Sinnige
Frederic Sinniger
Steffen Sinning
Christoph Sinning
Walter Sinnott-Armstrong
Ulrich Sinsch
Daniela Sint
David Sinton
Christian Sinzger
Isabelle Sioen
Chrissa Sioka
Matthias Sipiczki
Dorothy Sippo
Pentti Sipponen
Rachel Sippy
Marinez Siqueira
André Siqueira
Herbert Siqueira
Jair Siqueira-Neto
Mustafa Sir
Mauro Siragusa
Helmy M Siragy
Maria Sirakov
Clelia Sirami
Ravi Sirdeshmukh
Punnarai Siricharoen
Sema Sirin
Korsuk Sirinukunwattana
A. Siriphorn
Sunee Sirivichayakul
Jean Sirois
Pierre Sirois
Maurizio Sironi
Lucia Sironi
Laura Sirri
Daniel Sirtes
Cesare Sirtori
Giorgio Sirugo
Jm Sirvent
Maria Josè Sisalli
Mitike Sisay
Meghan Sise
Balindiwe Sishi
Marco Sisignano
Daniella Sisniega
Seeta Sistla
Ramakrishna Sistla
Lea Sistonen
Divya Sitaraman
Ioannis Sitaras

Cassian Sitaru
Freddy Sitas
Jack Sites
Patchima Sithisarn
Stephanie Sitnick
Evgenia Sitnikova
Harald Sitte
Beathe Sitter
Michael Sitvarin
L. Kristopher Siu
Parco Siu
Gilman Siu
Timothy Siu
Joanna Siuda
Roma Siugzdaite
Hari Sivakumar
Thillaiapalam Sivakumar
Rathinam Sivakumar
Jaichandran Sivalingam
E Sivamani
Jothilingam Sivapackiam
Suthesh Sivapalaratnam
Sobha Sivaprasad
J Sivaraman
Sridhar Sivasubbu
Jose Siverio
Stephen Michael Siviy
Stefan Siwko
Diana Six
Bruno Sixou
Katie Sizeland
Jacques Sizun
Gottfrid Sjödahl
Bengt Sjogren
Klara Sjögren
Elisabet Sjökvist
Jesper Sjökvist
Anita Sjölander
Tea Skaaby
Joel Skaistis
Bjørn Steen Skålhegg
Helen Skaltsa
Alexios–Leandros Skaltsounis
Panayiotis Skandalakis
Spyridon Skandalis
Panagiotis Skandamis
Stephen Skaper
George Skaracis
Katia Skarbek
Jacek Skarbinski
Aleksander Skardal
Jon Skare
Astrid Skarp
Dariusz Skarzynski
Sheila Skeaff
Paul Skehel
Michael Skeide
Guri Skeie
Sune Skeldal
Christopher Skelly
Jennifer Skelly
Matthew Skelton
Joshua Skelton
Christine Skerka
Arne Skerra
Timothy Skerry
Ingrid Sketris
Jeff Skevington
Karolina Skibicka
David Skibinski
Leif Skibsted
Randal Skidgel
Andrew Skidmore
Paula Skidmore
Michael Skinner
Susan Skinner
Heath Skinner
Brian Skinner
Eila Skinner
Matthew Skinner
Jared Skinner
Daniel Skinner
Robin Skinner
Benjamin Skinner
Andy Skinner
Finn Egil Skjeldestad
Theodorus Sklaviadis
Cezary Skobowiat
Steve Skoda
Tore Skodvin
Pontus Skoglund
Sten Skogmar
Helen Sköld
Anders Sköldunger
David Skole
Jeffrey Skolnick

Nadine Skoluda
Greg Skomal
Michele Skopec
Joanna Skorko-Glonek
Oleksii Skorokhod
Katherine Skorupski
Leif Skot
Jan Skotheim
Brian Skotko
Efthimios Skoulakis
Robert Skov
Ole Skovgaard
Sissel Skovgaard
Kerstin Skovgaard
Henrik Skovgaard
Soren Skovlund
Gry Skovsted
Mt Skowronski
Dorota Skowyra
Laura Skrip
Thomas Skripuletz
K. Skriver
Nikolai Skrynnikov
Grzegorz Skrzypek
Daniel Skuk
Svein Magne Skulstad
David Skurnik
Thomas Skutella
M. Skutsch
Pavel Skutschas
Jerod Skyberg
Catherine Slack
Mary Slack
Linda Slack-Smith
Tim Slade
Ronald Sladky
Christopher Slagle
Robert Slany
Christopher Slape
Mel Slater
Joel Slaton
Marc Slattery
Martha Slattery
Matthew Slattery
Susan Slatyer
James Slauch
Thomas Slaughter
James Slaughter
Monica Slavin
Joanne Slavin
Robert Slavin
Irma Slavutsky
Jean Slawinski
Ov Slayden
William Slayton
Willem Sleegers
James Sleigh
Patrick Sleiman
Sama Sleiman
Marit Sletmoen
Dennis Slice
Hamdy Sliem
Stephen Sligar
Wendy Sligl
Ilja Sligte
Predrag Slijepcevic
David Sliney
Annabelle Slingerland
Bernard Slippers
Richard Sliuzas
Daniel Sliva
Karen Sliwa
Richard Sloan
Erica Sloan
Nancy Sloan
Chantel Sloan
Elizabeth Sloand
Bonnie Sloane
Matthew Sloat
Helena Slobodskaya
Semyon Slobounov
Alexander Slocum
Amy Slogrove
Ivan Sloma
Andrzej Slominski
Joan Slonczewski
Michelle Slone
Michel Slotman
Burton Slotnick
Zdenek Slouka
Robert Sloviter
Diewertje Sluik
Judith Sluimer
Kathleen Sluka
Igor Slukvin
Carolyn Slupsky

David Slusky
Wendy Slutske
Marc Slutzky
Vincent Sluydts
Ronald Sluyter
Laura Sly
Jennifer Slyker
David Smadja
Stephen Smagula
David Šmajs
Marc Smaldone
Stephen Smale
C. Smales
Neil Smalheiser
Pamela Small
Ian Small
Dana Small
Christopher Small
Michael Small
Rhonda Small
Eric Small
Alex Small
Susan Small
Jan Smalle
Renate Smallegange
Michael Smanski
David Smart
Deedee Smart
Damian Smedley
Bard Smedsrod
Delbert Smee
Melanie Smee
Nicholas Smeeton
Jeroen Smeets
Bert Smeets
Bart Smeets
Erland Smeland
László Smeller
Mark Smeltzer
Annemieke Smet
Judith Smetana
Barth Smets
Richard Smeyne
Craig Smibert
Scott Smid
Jerusa Smid
Timo Smieszek
Richard Smiley
Jeff Smiley
Marko Smiljanic
Despina Smirlis
Stelios Smirnakis
Theo Smit
Izak Smit
Linda Smit
Filip Smit
Pieter Smit
Suzanne Smit
Jesper Smit
Suchi Smita
Tvrtko Smital
Thomas Smith
Arnold Smith
Corey Smith
Jeffrey Smith
Tara Smith
Brian Smith
Gregory Smith
Eric Smith
Tanya Smith
Barry Smith
Val Smith
Allan Smith
Jennifer Smith
Steven Smith
David Smith
Nicholas Smith
C. Smith
Douglas Smith
A. Smith
Sinead Smith
Abigail Smith
Lynette Smith
Dawn Smith
Amber Smith
Michael Smith
P Brian Smith
Kirsty Smith
Clyde Smith
Ken Smith
Kimberley Smith
Lee Smith
Graham Smith
Peter Smith
Shad Smith
Susan Smith

Martin Smith
Lachlan Smith
Maree Smith
Nigel Smith
Andrew Smith
George Smith
Stephanie Smith
Samuel Smith
Wade Smith
Mark Smith
Krister Smith
Justin Smith
Lindsay Smith
Richard Smith
Theresa Smith
Kevin Smith
Adam Smith
Alistair Smith
Colton Smith
Robert Smith
Megan Smith
Matthew Smith
Alan Smith
Chris Smith
Rebecca Smith
Darren Smith
Lincoln Smith
R. Theodore Smith
Noel Smith
James Smith
Joel Smith
Jeff Smith
John Smith
Michelle Smith
Alex Smith
Kimberly Smith
Rex N Smith
Gordon I. Smith
Kenneth Smith
Steve Smith
Brad Smith
Tina Smith
Jeannina A. Smith
Vanessa Smith
Kara Smith
Annabel Smith
Elizabeth Smith
William Smith
Leann Smith
Shavannor Smith
Dean Smith
Dave Smith
Sakima Smith
Anne Smith
Rosamund Smith
Phillip Smith
Gary Smith
Dirk Smith
Alexandra Smith
Kathlyn Smith
Brennan Smith
Briar Smith
Erica Smith
Stephen Smith
Patrick Smith
A. Peyton Smith
Tyler Smith
Alice Smith
Webb Smith
Ashley Smith
Chelsea Smith
Jessica Smith
Daphne Smith Marsh
Robert Smith?
Sophie Smither
T.A. Smitherman
Peter Smithers
Thomas Smithgall
Karen Smith-Mccune
Abbie Smith-Ryan
Erica Smithwick
Henk Smits
Hermelijn Smits
Marion Smits
Anja Smits
Anke Smits
Wiep Klaas Smits
Iris Smits
Adrianne Smits
Nathalie Smitz
Ewelina Smoktunowicz
John Smol
Lee Smolen
Albert Smolenski
Ryszard Smolenski
James Smoliga

Agnieszka Smolinska
Marcus Smolka
Katherine Smollett
Yossi Smorgick
Abdelaziz Smouni
P.E. Smouse
Olya Smrkovski
Tom Smulders
Yvo Smulders
A. Smulian
Nikolaos Smyrnis
Pavlo Smyrnov
Christopher Smyser
Gordon Smyth
Susan Smyth
Jeremy Smyth
David Smyth
Davida Smyth
Ron Smyth
Carolyn Smyth
Tyson Smyth
Rene Snacken
Erik Snapp
Santha Kumari Snatkunam
Lynne Sneddon
Rodlescia Sneed
Ton Snelder
Paul Snelgrove
Robert Snelgrove
Warren Snelling
Timothy Snelling
Kim Sneppen
James Sneyd
Ban Sng
Ashley Snider
Martin Snider
Harold Snieder
Pamela Sniezhkin
Soham Snih
Peter Snijders
Antoine Snijders
Lysanne Snijders
Anke Snijders
Evelien Snippe
Eric Snively
Jasper Snoek
Kristin Snopkowski
Ásta Snorradóttir
Allison Snow
Meradeth Snow
Andrew Snow
Dean Snow
Robert Snowden
Abraham Snyder
Christopher Snyder
Kenneth Snyder
Amanda Snyder
Robert Snyder
Bruce Snyder
Richard Snyder
Joyce So
Euiyoung So
Winnie So
Wing Chee So
Cristina Soares
Irene Soares
Ana Soares
Maria Soares
Nelson Soares
Luisa Soares-Miranda
Krzysztof Sobczak
Noam Sobel
Jack Sobel
David Sobel
Ken Sobel
Saul Soberanes
Roy Soberman
Jorge Soberon
Jan Sobesky
S. Sobhani
Eric Sobie
André Sobiecki
Pawel Sobkowicz
Kathryn Sobocinski
Boris Sobolev
Stanislav Sobolevsky
Jonathan Soboloff
Daniel Sobota
Fabian Sobotka
Roman Sobotka
Rita Sobral
Luis Sobrevia
Lucia Sobrin
Francisco Sobrino
Tomas Sobrino
Stephanie Socher

Kei Sochi
Taha Sochi
Laura Sockol
Jonathan Sockolosky
Renato Socodato
Priscila Socolowski
Silvia Socorro
Didier Socquet-Juglard
Yao Sodahlon
Beate Sodeik
Kathy Soder
Daniel Soderberg
Kenneth Söderhäll
Martin Söderholm
Maria Söderlund-Venermo
Ann Charlott Soderpalm
Komal Sodhi
Sumeet Sodhi
M. Sodhi
Donald Sodora
Kent Søe
John Soechting
Oliver Soehnlein
Maria De Nazaré Soeiro
Takeshi Soeki
Christian Soeller
Peter Soema
José Soengas
Karline Soetaert
Djora Soeteman
Marc Soethout
Nidhi Sofat
Aytul Sofu
Satoshi Sofue
John Soghigian
Muhammad Sohail
Zahra Sohani
Md. Mahmodul Hasan Sohel
Terry Sohl
Yoshiro Sohma
Jae-Cheon Sohn
Elliott Sohn
Ju-Tae Sohn
Jae-Kyung Sohng
Siavash Sohrab
Abbas Sohrabpour
Uri Soiberman
Codruta Soica
Mustafa Soilak
Raija Soininen
Eeva Soininen
Mariano Soiza-Reilly
Nancy Soja
Abraham Sojan
Richard Sojda
Samuel Sojinu
Zbynek Sokol
Rebeccah Sokol
Arseny Sokolov
Igor Sokolov
Sasha Sokolov
Yuliva Sokolova
Jeremy Sokolove
Marla Sokolowski
Evgeni Sokurenko
Ion Sola
J. Sola
Vicky Solah
Zakaria Solaiman
Daniel Solaiman
Bogdan Solaja
Cuneyt Solak
Tiina Solakivi
Alberto Sola-Landa
Amolkumar Solanke
Sohan Lal Solanki
Angel Solano Garcia
Gloria Solano-Aguilar
Santiago Solares
Alessandra Solari
Florence Solari
Jan Helge Solbakk
Monica Solberg
Svein Solberg
Eric Solberg
Laurence Solberg
Leah Solberg Woods
Giorgio Soldani
Larisa Soldatova
Mirco Solé
Montserrat Solé
David Solecki
Mohsen Soleimani
Hassan Soleimanpour
Francisco Soler
Xavier Soler

Javier Solera
Alfonso Soler-Bistue
Jo Solet
Gaye Soley
Ole Solheim
Sylvain Soliman
Hatem Soliman
Emad Soliman
Angelo Solimini
Antonio Solinas
M. Solis
V. Soljic
David Soll
Dieter Söll
Ludvig Sollid
Stina Therese Sollid
Wolfgang Söllner
Marco Solmi
Jay Solnick
Joseph Solomkin
Sorin Solomon
Michael Solomon
Brad Solomon
George Marty Solomon
Sonja Solomon
Abraham Solomon
Barry Solomon
Tessa Solomon-Lane
Noel Solomons
Elena Solomou
Alicia Solorzano
Mark Soloski
Ilia Solov'Yov
Olga Solovyova
Andrew Solow
Kamran Soltani Arabshahi
Ali Reza Soltanian
Leellen Solter
Alex Soltermann
Doug Soltis
Peter Solymos
Sudipta Som
Takeshi Soma
E. Somanathan
Jason Somarelli
Kumaravel Somasundaram
Konstantinos Sombolos
Jerome Some
Raz Somech
Nami Someya
Tamás Somfai
Luba Sominsky
Avril Somlyo
Francesco Somma
Claire Sommargren
Ralf Sommer
Gunhild Sommer
Sebastian-Patrick Sommer
Ulrike Sommer
Ulrich Sommer
Julia Sommerfeld
Mark Sommerfeld
Mitchell Sommers
Rami Sommerstein
Jessica Sommerville
Ildiko Somorjai
Ma Somsouk
Seung-Woo Son
Deok-Soo Son
Hokyoung Son
Jae-Kyoung Son
Weon-Young Son
Gi Hoon Son
Diego Sona
Mahendra Sonawane
Morgan Sonderegger
Jonas Søndergaard
A. Sonderlund
Mark Sonderup
Neal Sondheimer
Toshimasa Sone
Samir Soneji
Nahum Sonenberg
Geir Sonerud
William Sones
Emily Sonestedt
Wenxia Song
Jiuzhou Song
Long-Sheng Song
Yiqing Song
Lijiang Song
Jiangning Song
Jiasheng Song
Changxu Song
Chang-Hwa Song
Fengming Song

Jianxun Song
Xue-Jun Song
Yajun Song
Bing Song
Cunjiang Song
Song-Quan Song
Fengju Song
Yong Song
Byoung-Joon Song
Yan Song
Jie Song
Ki Jun Song
Jae Kwang Song
Hojun Song
Haengseok Song
Conghe Song
Je Seon Song
Xinzhang Song
Haifeng Song
Jae Song
Kyo Young Song
Rui Song
Chang-Seon Song
Botao Song
Linsheng Song
Tao Song
Lei Song
Min Song
Bin Song
Huan Song
Jinlin Song
Dar-Kyu Song
Lixin Song
Liang Song
Jikui Song
Jingyuan Song
Xianliang Song
Xianjun Song
Wenjun Song
Changcheng Song
Liyan Song
Sunbin Song
Myeong Jun Song
Yang Song
Giltae Song
Rong Song
Pengfei Song
Zewei Song
Brian Song
Wei Song
Ai Song
Xingshun Song
Xiao-Yan Song
Ken Song
Zilin Song
Youhong Song
Benben Song
Gaoyuan Song
Xiao-Peng Song
Guohua Song
Yufang Song
Hai-Tao Song
Jun Song
Jim Song
Mina Song
Yuanjian Song
Zhen Song
Yong-Ak Song
Yuanyuan Song
Thomas Songer
Nucharin Songsasen
Sanjeev Soni
Eppurathu Soniya
Tolga Taha Sönmez
Pam Sonnenberg
Anton Sonnenberg
Sandro Sonnino
Chiara Sonnino
Alois Sonnleitner
Kai Sonntag
Koh-Hei Sonoda
Kintake Sonoike
Tad Sonstegard
Sadanand Sontakke
Po-Chi Soo
Valerie Soo
Bulbul Sood
Rashmi Sood
Sunhapas Soodvilai
Sajid Soofi
Raju Soolanayakanahally
Lynn Soong
Keryea Soong
Yoke Lim Soong
Jennifer Soong
Sada Soorapanth

Harini Sooryanarain
Ian Soosay
Fiona M. Soper
Antonia Sophocleous
Mohan Sopori
Jörg Soppa
Amuchou S. Soraisham
Andrea Soranno
Alma Sörberg
Sveinung Sørbye
Ingvil K Sørbye
Gabriele Sorci
Mary Sorci-Thomas
Paolo Sordino
Michael Sorensen
Mathew Sorensen
Flemming Sorensen
Peter Sörensen
Uffe Sørensen
Karen Sørensen
Christine Sorenson
Hermona Soreq
Christian Sorg
Joseph Sorg
Rüdiger Sorg
Christan Sorg
Olivier Sorg
Robert Sorge
Carlos Sorgi
Bernat Soria
Guadalupe Soria
Jeannette Soria
Edgar Soria-Gomez
Vincent Soriano
Jordi Soriano
Sergi Soriano
Miguel Soriano
Carles Soriano-Mas
Hiroyuki Sorimachi
Barbara Sorkin
Maria Pia Sormani
Marjorita Sormunen
Liliana Soroceanu
Lydia Sorokin
Dimitry Sorokin
Agnieszka Sorokowska
Piotr Sorokowski
Shahryar Sorooshian
Michail Sorotos
Reza Soroushmehr
Mohammad Sorower
Patrik Sörqvist
Valeria Sorrenti
Brian Sorrentino
Dario Sorrentino
Vincenzo Sorrentino
Maria Angela Sortino
Caryl Sortwell
Ana Luisa Sosa
Victoria Sosa
Miguel Sosa
Felipes Sosai
Margherita Sosio
Jacob Sosnoff
Lynn Sosnoskie
Tomasz Sosnowski
Wayne Sossin
Tamara Sotelo
Rogerio Sotelo-Mundo
Giovanni Sotgiu
Javier Sotillo
Aristeidis Sotiras
Ilka Sötje
Claudio Soto
Martha Soto
David Soto
Ileana Soto
Sara Soto
Jose Luis Soto
Gabriela Soto
Alejandro Soto-Gutierrez
Shinichi Sotome
Andres Soto-Varela
Jane Sottile
Andrea. Sottoriva
Maud Soty
Lina Soualmia
Philippe Soubeyran
Emmanuel Soubies
Julien Soubrier
Guillaume Souchay
Céline Souchay
Serhiy Souchelnytskyi
Hugo Soudeyns
Nadejda A. Soudzilovskaia
Assem Soueidan

J. Souframanien
Sami Souissi
Alexander Soukas
Laura Soul
Valérie Soulard
Pauline Soulas-Sprauel
Didier Soulat
Denis Soulet
Tewfik Soulimane
Kyriakos Souliotis
Zervoudaki Soultana
Panos Soultanas
Vassili Soumelis
Andrew Soundy
Carole Sourbier
Nicole Souren
Masayoshi Souri
Harald Sourij
Victor Sourjik
Joao Sousa
Tais Sousa
Daniela Sousa
Maria João Sousa
Ana Sousa
Aretuza Sousa
Magda Sousa
Carla Sousa
Diana Sousa
Konstantinos Sousounis
Lior Soussan-Gutman
Mikle South
Andrew South
Tony Southall
Anne Southerland
Peter Southern
Danielle Southern
Brian Southern
Melissa Southey
Colin Southwell
André Souto
Eliana B. Souto
Leandro Souto
David Souto
Valeria Souza
Rhonda F. Souza
Diogo O Souza
Danielle Souza
Marcy Souza
Cleverson Souza
Silvia Souza
Ana Carolina Souza
Rodrigo Souza
Julio Souza
Tharsis Souza
Bryan Souza
Carol Souza Da Silva
Vicente Souza-Dantas
Vanessa Souza-Mello
Jayme Souza-Neto
Flavia Souza-Smith
Graça Soveral
Mikhail Sovershaev
Bruno Sovran
Marianne Sowa
Ramanathan Sowdhamini
James Sowers
Pawel Sowinski
Paul Sowman
Mukhles Sowwan
Akihiko Soyama
Adedamola Soyibo
Ireneous Soyiri
Florian Soyka
Alejandro Soza
Adnan Sözen
Shanmuga Sozhamannan
Erica Spackman
Davide Spadaro
Oliver Spadiut
Filippo Spadola
Andrea Spaeth
Paolo Spagnolo
Matteo Spagnolo
Paul Spagnuolo
Martin Spahn
Anne Spain
David Spain
John Spainhour
Lena Spallek
Gianfranco Spalletta
Francesco Spallotta
Paul Span
Antonio Spanevello
Rainer Spang
Espen Spangenburg
Christopher Spankovich

Andrew Spann
Herbert Spapen
Courtney Sparacino-Watkins
Joseph Sparano
Jackson Sparks
Corey Sparks
Caroline Sparks
Michael Sparks
Marco Sparro
Janet Sparrow
Laurent Sparrow
Nicole Spartano
Helena Spartz
Irena Spasic
Peter Spath
Stephen Spatz
Stephen Spaulding
Luca Spazzapan
Greg Spear
Brett Spear
Peter Speare
Paul Spearman
Norah Spears
Nicola Specchio
Karsten Specht
Sabine Specht
Hanno Specht
Jim Specht
Anne Speckens
Carsten Speckmann
Denise Spector
Alexander Spector
Geoff Spedding
Simon Spedding
Reinhart Speeckaert
Marijn Speeckaert
Conrad Speed
James Speed
Doug Speed
Maarten Speekenbrink
Nicolas Spegazzini
Peter Spegel
Marc Spehr
Walter Speidl
William Speier
P.W. Speiser
C. Arnold Spek
Jason Spence
Alexa Spence
Dana Spence
Melissa Spencer
Patrick Spencer
John Spencer
James Spencer
Rebecca Spencer
Joseph Spencer
Sarah Spencer
Lisa Spencer
Netanya Spencer
Lillian Spencer
Benjamin Spencer
Ian Spencer
Megan Spencer-Smith
Friedrich Spener
Ryan Spengler
Ulrich Spengler
Dietmar Spengler
Christina Spengler
Sarah Spengler
Dimitrios Spentzos
M Sperandeo
Markus Sperandio
Felipe Sperandio
Alessandra Sperduti
Jm Spergel
Joachim Spergser
Felix Sperling
Silke Sperling
Claudia Sperling
Or Sperling
Ann Sperry
Jason Sperry
Steven Sperry
Cornelia Spetea
Mariana Spetea
Cornelia Speth
Robert Speth
Karina Speziale
Silvia Spezzaferri
Robert Spicer
Julie Spicer
Leon Spicer
Corinne Spickett
Žiga Špiclin
Paul Spiegel
Daniel Spiegel

Kai Spiegelhalder
Bruce Spiegelman
Jeffrey Spielberg
Seth Spielman
Wolfgang Spielmeyer
Andrew Spiers
Huub Spiertz
Claudia Spies
M. Spies
Andrej-Nikolai Spiess
Ewa Spiez
Francesca Spiga
Enrico Spiga
Lisa Spiguel
Stavros Spiliopoulos
Iris Spiliopoulou
Elias Spiliotis
Maria Grazia Spillantini
Robin C Spiller
Frank Spinale
Dominik Spinczyk
Katherine Spindler
Meredith Spindler
Sherry Spinelli
Francesca Romana Spinelli
Gaia Spinetti
Christoph Spinner
Helder Spinola
Michael Spiotto
Shalom Spira
Bruno Spire
Carlo Spirlì
Stephen Spiro
Emma Spiro
Christina Spiropoulou
Freek Spitaels
Andrea Spitaleri
Pietro Spitali
Ryan Spitler
Jan Spitsbergen
Ken Spitze
Martin Spitzer
Philip Spitzer
Rebecca Splan
Angela Spleen
Gary Splitter
Richard Splivallo
Kurt Spokas
Marialuisa Spoletini
Jeff Spooner
Thomas Spoorenberg
Victor Spoormaker
Michael Sporn
Georgina Sposetti
Richard Sposto
Belinda Spoto
Erica Spotswood
John Spouge
Cassandra Spracklen
David Spragg
Heidi Spratt
Neil Spratt
Daniel Spratt
David Spray
Armand Sprecher
Thees Spreckelsen
Rolf Sprengel
Andreas Sprenger
Christian Sprenger
Preston Sprenkle
Janet Sprent
Bryan Spring
Henriet Springelkamp
Mark Springer
Anne Springer
Sandra Springer
Poli Mara Spritzer
Gudrun Sproesser
Julann Spromberg
Robert Sprung
Karen Spruyt
John Spudich
Simone Spuler
David Spurgeon
Anne Spurkland
Robert Spurney
Maria Spychalska
Dionysios Spyratos
Cristiane Squarize
Andrea Squartini
Alessio Squassina
Flaminio Squazzoni
Nicola Squillace
Fabio Squina
Iain Squire
Christopher Squire

Geoffrey Squire
Malgorzata Srebniak
Ewald Srebotnik
Vishnu Sreekumar
Vidhyapriya Sreenivasan
Chandrashekhar Sreeramareddy
Srinand Sreevatsan
Sargur Srideshikan
Saranya Sridhar
Sri Sridhar
Vijayalakshmi Sridharan
Rupa Sridharan
Divya Srikumaran
Sadeesh Srinathan
Miduturu Srinivas
Sangly Srinivas
S. Srinivas
Niranjan Srinivas
Swaminath Srinivas
Manoj Srinivasan
Shanthi Srinivasan
Sundar Srinivasan
Sujatha Srinivasan
Vijaya Bharathi Srinivasan
Rajagopalbabu Srinivasan
Dayalan Srinivasan
Mythily Srinivasan
Mukund Srinivasan
Divya Srinivasan
Lalitha Srinivasan
Varun Srinivasan
R. Srinivasan
Pavuluri Srinivasu
Srinivasa Srinivasula
Venkateswara Sripathi
Dinesh Sriramulu
Manjula Sritharan
Sudesh Srivastav
Deepak Srivastava
Lalit Srivastava
Satish Srivastava
Rohit Srivastava
Amit Srivastava
Sanjay Srivastava
Jyoti Srivastava
Ajay Srivastava
Kamna Srivastava
Hari Shanker Srivastava
Gaurav Srivastava
Mansi Srivastava
Raghvendra Srivastava
Sanjeev Srivastava
Sarika Srivastava
Indresh Srivastava
Alok Srivastava
Vikas Srivastava
Sudhakar Srivastava
C.N. Srivastava
Ruchi Srivastava
Anuj Srivastava
Sanvesh Srivastava
Priyanka Srivastava
Pratap Srivastava
Anand Srivastava
Ritesh Srivastava
Prashant Srivastava
Komandoor Srivathsan
Anjana Srivatsan
Jacek Sroka
Miroslav Srutek
Agnes Ssali
John Ssempebwa
James St John
Daniel St Johnston
Benoit St Pierre
Brad St. Clair
Judy St. Leger
Anna St. Swierzko
Michael Staab
Jeffrey Staab
Claudia Staab-Weijnitz
Johan Staaf
Frank Staal
Herman Staats
Richard Staba
Judith Stabel
Edward Stabler
Alessadra Stacchiotti
Michael Stacey
Christopher Stach
Matthew Stachler
Peter Stachon
Jeanne Stachowiak
John Stachowicz
Steven Stack
Austin Stack

Sharon Stack
Samantha Staddon
Miguel Stadecker
Ralph Stadhouders
Vanessa Stadlbauer
Krisztian Stadler
Jonathan Stadler
Andreas Stadler
Julia Stadler
L. Stadtmauer
Martin Staege
Bart Staels
An Staes
Iain Staffell
Lorenzo Stafford
Megan Rose Stafford
Ryan Stafford
Graham Stafford
Massimo Stafoggia
Frank Stahl
Daniel Stahl
Eli Stahl
Rolf Stahl
Yvonne Stahl
James Stahl
Steen Stahlhut
Shauna Stahlman
Gunilla Ståhls
Amanda Staiano
Harald Staiger
Christopher Staiger
Helen Stain
Henry Staines
Richard Staines
Joseph Stains
Douglas Stairs
Bashar Staitieh
Eleanor Stalenberg
Kevin J. Staley
Christopher Staley
Kate Stalin
Ilse Stalis
Michael Stallcup
Kyle Staller
Sjoerd Stallinga
Christopher Stallings
Sericea Stallings-Smith
Ingeborg Stalmans
Lukas Stalpers
Remco Stam
Eric Stam
Jonny St-Amand
Efstathios Stamatatos
Nikiforos Stamatiadis
Iva Stamatova
Vuk Stambolic
Peter Stambrook
Maria Stamelou
Marija Stamenkovic
W. Daniel Stamer
Rio Stamler
Christof Stamm
Tanja Stamm
Lola Stamm
Cordula Stamme
Katie Stammler
Michael Stamos
Dimitrios Stamou
Marianna Stamou
Boryana Stamova
Rose Stamp
Lincon Stamp
Radu Stan
Michael Stanchina
Stefan G. Stanciu
Gabi Stancu
Edward Stanek
Ralf Stanewsky
Janet Stanford
William Stanford
Kristin Stanford
Elaine Stanford
Fatima Cody Stanford
Michael Stanford
Joseph Stanford
Andreas Stang
Catherine Stanger
Gabriele Stangl
Michael Stanhope
John Stanifer
Fernanda Staniscuaski
Lee Stanish
Charles Stanish
Magdalena Staniszewsa
Bruno Stankoff
Milan S. Stankovic

Theodore Stankowich
Margaret Stanley
Pamela Stanley
Jennifer Stanley
Christina Stanley
Damian Stanley
Nicola Stanley-Wall
Pasquale Stano
Maja Stanojevic
Olivera Stanojlovic
Stephen Stansfeld
Kirstie Stansfield
Giorgio Stanta
T. Stantchev
Patric Stanton
Bruce Stanton
Peter Stanton
Margaret Stanton
Mariana Stanton
Daniel E. Stanton
Tasha Stanton
David Stanton
John Stanturf
Boris Stanzel
Heather Stapleton
Jessica Stapley
Kristina Star
John Starbuck
Marjanca Starcic Erjavec
Katrin Starcke
John Stark
Benjamin C. Stark
Walter Stark
Renee Stark
Thomas Stark
Mitchell Stark
Diana Stark Ekman
Robert Starke
Joel Starkopf
Melissa Starling
Patrick Starlinger
Maud Starmans
René St-Arnaud
Iris Starnberger
John Starr
Alison Starr
Timothy Starr
Katarzyna Starska
Carla Startin
Alexander Staruschenko
Timothy Stasevich
Sara Stasik
Claudio Stasolla
Ruth Stassart
Michael Stastny
Lukas Staub
Fabian Staubach
William Stauber
Claudia Staubert
Roland Staud
Dawid Staudacher
Didier Staudenmann
Jeffrey Staudinger
Thomas Staudinger
Katharina Staufer
Natalie Stauffer
Glenn Stauffer
Harald Stauss
Marios Stavridis
Menelaos Stavrinides
David Stawarczyk
Stanislaw Stawicki
Philipp Stawowy
Tonje Stea
Lindsay Stead
Kathryn Steadman
Luca Steardo
Duncan Stearns
Keenan Stears
Charles Stebbins
David Stec
Boguslaw Stec
Patrizia Steca
Barbara Stecca
Carla Stecco
Antonio Stecco
Bärbel Stecher
Todd Steck
Susan Steck
Ulrike Steckelings
G. Christopher Stecker
Eric Stecker
Sophie Steculorum
Douglas Steeber
Clemens Steegborn
Régine Steegers-Theunissen

John Steel
Christina Steel
Craig Steel
Christopher Steel
Mark Steele
Joshua Steele
Terry Steele
James Steele
Val Steele
Latina Steele
Luc Steels
Hanno Steen
Christel Steen
Andrew Steen
David Steen
Laura Steenpass
Matt Steensma
Ivan Steenstra
Penelope Steer
Jeremy Steeves
Charikleia Stefanaki
Irene Stefanaki
Michael Stefanek
Horia Stefanescu
Stefania Stefani
Alessandro Stefani
Gabor Stefanics
Jörg Stefanie
Federico Mattia Stefanini
Leonidas Stefanis
Pawel Stefanoff
Michael Stefanone
Detelin Stefanov
Nadia Stefanova
Neda Stefanovic
Maja Stefanovic-Racic
Jeanine Stefanucci
Clara Stefen
Shawn Steffan
Will Steffen
Melanie Steffens
Darrell Steffensmeier
Arjan Stegeman
Dick Stegeman
Elizabeth Stegemoller
Alexander Stegh
Barbara Stegmann
David Stegner
Jimmy Stehberg
Jörg Stehle
Juan Steibel
Brian S. Steidinger
Christian Steidl
Patrick Steigemann
Sandra Steiger
Frank Steigerwald
Katrina Steiling
Wolfgang Stein
Markus Stein
Aryeh Stein
Barry Stein
Donald Stein
Jason Stein
Ellen Stein
Thor Stein
Anke Stein
Anna Stein
Mark Stein
Katharina Stein
Katharina Viktoria Stein
Sheman Stein
William Steinbach
Joachim Steinbach
Falko Steinbach
Derek Steinbacher
Peter Steinbacher
Martin Steinbauer
Doron Steinberg
Gregory Steinberg
Daniel Steinberg
Fabian Steinberg
Peter Steinberger
Yosef Steinberger
Holger Steinbrenner
Alexander Steinbuchel
Mário Steindel
Barbara Steiner
Kerstin Steiner
Adrian Steiner
Stephan Steiner
Joern Steinert
Konrad Steinestel
Eirikur Steingrimsson
Helen Steingroever
Radjin Steingrover
Karsten Steinhaeuser

Dieter Steinhagen
Yael Steinhart
David Steinhauer
Marco Steinhauser
Heinz-Juergen Steinhoff
Mara Steinkamp
Alexander Steinle
Nanette Steinle
Jena Steinle
Ortrud Steinlein
Lawrence Steinman
Eike Steinmann
Ivo Steinmetz
Adolf Steinrigl
Cari Stek
Jeffrey Steketee
Lukasz Stelinski
Rike Stelkens
Giulia Stella
John Stelling
Steven Stellman
Konstantinos Stellos
Julian Stelzer
Valter Stemberga
Johan Stenberg
Elisabet Stener-Victorin
Robert Stengel
Benedicte Stengel
Andreas Stengel
Drake Stenger
Magnus Stenhagen
Ronald Stenkamp
Deborah Stenkamp
Julie Stenken
Ulf-Hakan Stenman
Göran Stenman
Matti Stenroos
Karin Stensjo
Marcus Stensmyr
Peter Stenvinkel
Nikola Stenzel
Holger Stepan
Visnja Stepanic
Irina Stepanov
Christoph Stephan
Roger Stephan
Paula Stephan
Anastasis Stephanou
Catherine Stephen
Coles Stephen
Jacqueline (Jackie) Stephens
David Stephens
Edward Stephens
Scott Stephens
Brent Stephens
Charles Stephensen
Sally-Anne Stephenson
Jessica Stephenson
Emmanuel Stephen-Victor
Mary Ann Stepp
Claire Steppan
Jaroslav Sterba
Martin Šterba
Lieven Sterck
Nir Sterer
Peter Sterk
Timothy Sterling
Charles Sterling
David Stern
Hal Stern
Mariana Stern
Emily Stern
Paula Stern
Andrew Stern
Naftali Stern
Joel Nh Stern
Marc Stern
Marcelo Sternberg
Jared Sterneckert
R. Sternglanz
Fabio Sterpone
Gaetana Sterrantino
Silke Stertz
Torsten Sterzenbach
Florian Sterzing
Elisabeth Stes
Cinnamon Stetler
Manuela Stets
Karen Steudel-Numbers
Lara Stevanato
Stefan Stevanovic
Oliver Stevanovic
Marija Stevanovic
Sharon Stevelink
Akiyama Steven
Mark Stevens

Alexander Stevens
Richard Stevens
Martin Stevens
Peter Stevens
Craig Stevens
Katherine Stevens
Mf Stevens
Adam Stevens
Joanne Stevens
Hanna Stevens
Adrienne Stevens
Stanley M. Stevens Jr.
Pablo Stevenson
Mark Stevenson
Robert Stevenson
James Stevenson
William Stevenson
Rob Stevenson
Edward Stevenson
Gayle Stever
Andrew Steward
Dominic Stewardson
Alexandre Stewart
George Stewart
Elizabeth Stewart
C. Neal Stewart
Don Stewart
Russell J. Stewart
Robert Stewart
Duncan Stewart
Lamonica Stewart
Philip Stewart
Cameron Stewart
James Stewart
Frank Stewart
Michael Stewart
Grant Stewart
Clinton Stewart
Kathlyn Stewart
Jane Stewart
Abigail Stewart
S. Stewart
Jay M Stewart
Jennifer Stewart
Alex Stewart
Rpdney Stewart
Susan Stewart
Heather Stewart
Ian Stewart
Andrew Stewart
Josh Stewart
Anna Stewart Ibarra
Steve Stewart-Williams
Sam Steyaert
Ewout Steyerberg
Peter Steyger
Frederik Steyn
Adrie Steyn
Mark Steyvers
Blanka Stiburková
Alex Stiby
Eric Stice
Roger Stich
Ryan Stidman
Daniel Stieber
Stefan Stieger
Bruno Stieger
Philipp Stiegler
Michaela Stieglmeier
Audun Stien
Rinke Stienstra
Marc Stift
Anne Stiggelbout
Nikola Stikov
Bangyan Stiles
Brad Stiles
Nikolaos Stilianakis
Chris Still
Kathy Stiller
Adriano Stinca
O. Stine
Alexander Stine
Timothy Stinear
James Stinear
J. Stinear
Reinhard Sting
Florence Stinglhamber
Francesco Stingo
Sandra Stinnett
Monique Stins
John Stins
Kathryn Stinson
Joseph Stinziano
Julian Stirling
Paola Stiuso
Martin-Hugues St-Laurent

Ben Stobart
Ellen Stobberingh
Maciej Stobiecki
Nino Stocchetti
C Stocco
Ann Stock
David Stock
Jay Stock
Jeff Stock
Daniela Stock
Patricia Stock
Sarah Stock
Jennifer Stock
Michael Stock
Laura Stockdale
Claire Stocker
Michael Stocker
Martin Stocker
Philipp Stockhammer
Norbert Stockhofe
Silvia Stockinger
Heinz Stockinger
Emily Stockings
Jason Stockmann
Anders Stockmarr
Steven Stoddard
James Stoeckel
Barbara Stoecker
Mark Stoeckle
Tobias Stoeger
Angela Stoeger
Nicole Stoesser
Gijsbert Stoet
Kilian Stoffel
John G. Stoffolano
Thomas Stoffregen
Thomas Stöggl
Stephen Stohlman
Sabine Stöhr
Bogdan Stoica
Natasa Stojanovic
Monika Stojek
Caroline Stokes
Andrew Stokes
Barry Stokes
Alexander Stokes
Patrick Stokes
Dennis Stokes
Leanne Stokes
Michael Stokesbury
Jakub Stoklosa
Viktor Stolc
Paul Stolee
Megan Stolen
Justin Stoler
Beatriz Stolf
Stefan Stoll
Eckart Stolle
James K. Stoller
John Stolz
U. Stolz
Katharina Stölzel
Rachael Stolzenberg-Solomon
Elijah Stommel
M. Stommel
Tjeerdjan Stomph
Graham Stone
David Stone
Geoffrey Stone
Emma Stone
Laura Stone
Jonathan Stone
Arthur Stone
William Stone
Scot Stone
Chris Stone
Michael Stone
Mike Stone
Forrest Stonedahl
Nicola Stonehouse
Marie-Pierre St-Onge
Paul Stoodley
Mike Stoolmiller
Ruedi Stoop
Arkadiusz Stopczynski
Pavel Stopka
Laura Stoppelbein
Marco Storace
Eric Storch
Kai-Florian Storch
Jan Storek
Paola Storici
Nigel Stork
Erik Storkebaum
Aurora Storlazzi
Fabio Storm

Janet Storm
Gary Stormo
Gary D. Stormo
Helen Storr
Brian Storrie
Brad Story
Jay Storz
Peter Storz
Fabio Stossi
Paul Stothard
Christina Stothard
George Stothart
Alistair Stott
Shannon Stott
Michael Stotz
Peter Stougaard
Laura Stough
S. Stoulos
Jane Stout
Dietrich Stout
Michael Stout
Jeffrey Stout
Richard Stouthamer
John Stover
Kristin Stover
Henrik Støvring
Craig Stow
James Stowe
Kathryn Stowell
Dan Stowell
Lisa Stowers
Paul Stoy
Radka Stoyanova
Mary Ellen Stoykov
Asbjørn Støylen
Nikolay Stoynov
Marco Straccia
Eric D. Strachan
Stefan Strack
Sylvia Stracke
Lucia Strader
Leslie Stradiot
Masja Straetemans
Uwe Strähle
Dietmar Straile
Hans Straka
Tracey Straker
Rita Strakovsky
Peter Strålfors
Daniel Stram
Sebastiano Stramaglia
Jeanette Strametz-Juranek
Allan Strand
Michael Strand
Tor Strand
Eva Strand
Erik Strandberg
Ariana Strandburg-Peshkin
Blair Strang
Eric Sträng
Saverio Stranges
Michael Strano
Maria Strano
Christoph Stransky
Carly Strasser
Douglas Strathdee
Gordon Strathdee
Lane Strathearn
Efstratios Stratikos
Richard Stratton
Kelly Stratton
Christian Stratz
Tobias Straub
Cory Straub
Beate Straub
Sebastian Straube
Martin Strauch
Suzana Straus
Lawrence Straus
Phyllis Strauss
Roland Strauss
Erick Strauss
Johannes Strauss
Rupert Strauss
Richard Strauss
Alfred Strauss
Andrew Straw
Rebecca Strawbridge
Stephen Stray
Daniel Strbian
Stephen Streatfield
Witold Streb
Daniel Streblow
Emilio Streck
Charles Streckfus
Maryann Street

Tamsyn Street
Ricardo Strefezzi
Jeffrey Streicher
Daniel Streicker
Andrea Streit
Merle Streitberger
Tatyana Strekalova
Sergei Strelkov
Stefan Stremitzer
Caroline Stremnitzer
Nicola Strenzke
Arlette Streri
Gillian Stresman
Enrica Strettoi
Berthold Streubel
Laura Streyffeler
Reiner Strick
Thomas Stricker
Marc Strickert
Deborah Strickland
Dan Strickman
Helene Strick-Marchand
Lidia Strigari
Joeri Strijk
Siobhan Strike
Pontus Strimling
Jeffrey Stringer
Kathleen Stringer
Rebecca Stringwell
Barry Stripp
Nicola Strisciuglio
Franc Strle
Nadica-Maltar Strmecki
Tilo Strobach
Scott Strobel
Gary Strobel
Philipp Ströbel
Todd Strochlic
Uwe Stroeher
Nathalie Stroeymeyt
Sabine Ströfer
Albrecht Stroh
Ron Strohmeyer
Terry Strom
Samuel Strom
Sara Strom
Stephen Strom
Marin Strøm
Bodil Strom Holst
Susanna Strömberg
Carmen Stromberger
Staffan Stromblad
Judith Strong
Michael Strong
Allan802 Strong
Karien Stronks
Jörg Strotmann
John Strouboulis
Robert Stroud
Donna Stroup
Nicholas Stroustrup
R. Strowd
Diederik Strubbe
Torsten Struck
Eva Strucken
Lisa Strug
Jan Strugnell
Christine Strullu-Derrien
Erik Štrumbelj
Miriam Strumia
Dirk Strunk
Alex Strunnikov
Paul Strutton
Patrick Strutzenberger
Sofie Struyf
Artur Struzik
Natalie Strynadka
Michal Strzelecki
Patrick Stuart
David Stuart
Gary Stuart
Bryan Stuart
Ben Stuart
Alastair Stuart
Amy Stuart
Andrew Stuart
Joe Stubbersfield
Michael Stubbington
Andrew Stubbs
Peter Stubbs
Brendon Stubbs
Lisa Stubbs
Johannes Stubert
Audun Stubhaug
Amber Stubler
Brian Stucky

Michael Studdert
Adam Studebaker
Romain Studer
Michele Studer
Harald Studer
Deborah Studer
Bettina Studer
David Studholme
George Studzinski
Dennis Stuehr
Wolfgang Stuerzl
Tod Stuessy
Babs Stuiver
Jan-Bernd Stukenborg
Gert Stulp
Collin Stultz
Collin M. Stultz
Philip Stumbles
Jason Stumhofer
Walter Stummer
Craig Stump
Laura Stunz
Dwayne Stupack
Roger Stupp
Christopher Sturdy
Richard Sturm
Ekkehard Sturm
Edward Sturrock
Jackie Sturt
Joy Sturtevant
Barbara Stussman
Peter Roy Stutchfield
Simon Stutz
Aaron Stutz
Olaf Stuve
Mark Styczynski
Iain Styles
Triantafyllos Stylianopoulos
Elena Stylianou
Eleni Stylianou
Maya Styner
Jenny Stynoski
Tin Tin Su
Ih-Jen Su
Xiao Su
Chunlei Su
Zhen Su
Changqing Su
Zheng Su
Hua Su
Ying-Hsiu Su
Yeu Su
Ming-Wei Su
Po-Hsuan Su
Dong-Ming Su
Xiaohua Su
Yi-Hsien Su
Shuo Su
Yunchao Su
Xinming Su
Jianmin Su
Jianya Su
Chien-Wei Su
Qi Su
Yu-Wen Su
Tung-Hung Su
Emily Chia-Yu Su
Huanxing Su
Wei-Juin Su
Yongchao Su
Jen-Liang Su
Huabo Su
Guanfang Su
Lei Su
Chun-Li Su
Wenru Su
Hongyan Su
Linlin Su
Huei-Jiun Su
Ninghu Su
Yanrui Su
Shaoyong Su
Hongxin Su
Zhangjie Su
Richard Su
Shihbin Su
Riqi Su
Yi Su
Zhengan Su
Jingtan Su
Zhenwei Su
Xiaoling Su
Chen-Ming Su
Zheng-Yuan Su
Hang Su
Song-Zhi Su

Dan Su
Lianghu Su
Sylvia Suadicani
Paula Suares-Rocha
Felipe Suarez
Andrew Suarez
Blanca Suarez
Jose Suarez
Martha Suarez Mutis
Abel Suárez-Fueyo
Maria Eugenia Suarez-Ojeda
Aroa Suarez-Vega
Sargurunathan Subashchandrabose
Angela Subauste
Ramnath Subbaraman
Shaila Subbarao
Chris Subbe
Sam Subbey
Vivek Subbiah
Kalidas Subedi
Manil Subesinghe
Tk Subhawong
Yousif Subhi
Andrew Subica
Damien Subit
Dharmalingam Subramaniam
Ajit Subramaniam
V. Nathan Subramaniam
Nathan Subramaniam
Renuka Subramaniam
Gopal Subramaniam
Sarada Subramanian
Ramaswamy Subramanian
Sandeep Subramanian
Vijayalakshmi Subramanian
Senthil Subramanian
Prem Subramanian
Sathish Subramanian
Arohan Subramanya
A. Subramanyam
S.H. Subramony
Agathe Subtil
Prasanta Subudhi
Andrew Subudhi
Dominika Suchá
Boris Suchan
Robert Suchland
Timothy Suchomel
Frederick Suchy
Sonja Sucic
Arthur Suckow
Henry Sucov
Jan Suda
Mateus Sudano
Sanjana Sudarshan
Mysore Sudarshana
A. B. Sudeep
Staci Sudenga
Kumar Sudesh
Yakkanti Sudhakar
M Sudhakara Reddy
Babu Sudhamalla
Walter Sudhaus
Peter Suedfeld
Garret Suen
Hans-Dieter Sues
Luis Suescún Bolívar
Noriyuki Suetsugu
Atsushi Suetsugu
Piotr Suffczynski
Anthony Suffredini
Kazuyuki Sugahara
Takuya Sugahara
Takayoshi Suganami
Michael Sugarman
Jeffrey Sugarman
Norio Sugawara
Hiroko Sugawara
Yasuhiko Sugawara
I Sugawara
Sumiko Sugaya
Bill Sugden
David Suggett
Michael Sughrue
Yukiko Sugi
Piotr Sugier
George Sugihara
Genichi Sugihara
S Sugihara
Jonathan Sugimoto
Cassidy Sugimoto
Shinya Sugimoto
Mitsuhiko Sugimoto
Hiroyuki Sugimoto
Ken Sugimoto
Mitsushige Sugimoto

Kenji Sugio
Akiko Sugio
Mamoru Sugita
Iwao Sugitani
Kenichiro Sugitani
S. Sugiura
Koji Sugiura
Mayumi Sugiura-Ogasawara
Minetaka Sugiyama
Haruo Sugiyama
Aravind Sugumar
Lonchin Suguna
Ap Sugunan
Moo-Jin Suh
Chang-Hee Suh
Alexander Suh
Sunghwan Suh
Hoonkyo Suh
Dae-Yeon Suh
Pann-Ghill Suh
Klaus Suhling
Hendrik Suhling
Andreas Suhrbier
Karsten Suhre
Feng Sui
Jing Sui
Hongyan Sui
Meihua Sui
Dawen Sui
Wawan Sujarwo
Heung-Il Suk
Young Suk Park
Katerina Sukacová
Theresa Sukal-Moulton
Chonlaphat Sukasem
Sergiy Sukhanov
Sergei Sukharev
Denis Sukhodolsky
Igor Sukhotnik
Galina Sukhova
Bhagyalaxmi Sukka Ganesh
Maria Sukkar
Jana Suklan
Kom Sukontason
Jirapornchai Suksaeree
Naeti Suksomboon
Saraswati Sukumar
Pramod Sukumaran
Hei Suk Sul
Ken Sulak
Preeti Sule
Karolina Sulek
Muhamad Suleman
Hagir Suliman
Sara Suliman
Cameron Sullards
Kevin Sullivan
Lori Sullivan
John Sullivan
Breandan Sullivan
Robert Sullivan
Brian K Sullivan
Sheena Sullivan
Shannon Sullivan
Craig Sullivan
Richard Sullivan
Kelly Sullivan
Joseph Sullivan
Clare Sullivan
Charles Sullivan
James Sullivan
Justin Sullivan
Jon Sullivan
David Sullivan
William Sullivan Jr.
G. Sulo
Ronan Sulpice
Soriano Sulpicio
Benjamin Sultan
Sherif Sultan
Papia Sultana
Hameeda Sultana
Istvan Sulykos
Gerlind Sulzenbacher
David Sulzer
Ernest Sumaili
Shubhankar Suman
Saulius Sumanas
Surahyo Sumarsono
Vadim Sumbayev
Rachita Sumbria
Masayuki Sumida
Yoshio Sumida
Akio Sumioka
Carolyn Summerbell
Kyle Summers

Jesse Summers
Matthew Summers
Keith Summerville
Roger Summons
Michael Sumner
Rick Sumner
Jennifer Sumner
Colin Sumners
Robert Sümpelmann
David Sumpter
Colin Sumpter
Xiao-Hong Sun
Ren Sun
Yan Sun
Zhifu Sun
Hongmei Sun
Jiang Sun
Liangdan Sun
Der-Shan Sun
Jianxin Sun
Fenyong Sun
Lei Sun
Weining Sun
Zhonghua Sun
Hong Sun
Yi Sun
Genlou Sun
Sheng Sun
Xuecheng Sun
Chao Sun
Peiqing Sun
Fei Sun
Guang Sun
Jianzhong Sun
Yicheng Sun
Joseph Sun
Tung-Tien Sun
Jun Sun
Qi Sun
Xiaofei Sun
Qinghua Sun
Hongpeng Sun
Yuqiang Sun
Xi-Qing Sun
Hong-Shuo Sun
Xiaodong Sun
Xingmin Sun
Li Sun
Hang Sun
Jianbo Sun
Zhaoli Sun
Hongying Sun
Zhan-Li Sun
Shucun Sun
Jin Sun
Ge Sun
Hui Sun
Kai Sun
Yangbo Sun
Gang Sun
Dandan Sun
Yang Sun
Lixian Sun
Dongxiao Sun
Jiayuan Sun
Wei-Zen Sun
Dong Sun
Charlotte Sun
Yuhao Sun
Lihua Sun
Daochun Sun
Jinsheng Sun
Jiandong Sun
Ping Sun
Cheng Sun
Yuyang Sun
Fu-Lin Sun
Fanyue Sun
Xinchen Sun
Yuan Sun
Ye-Huan Sun
Yubing Sun
Wei Sun
Keer Sun
Geng Sun
Wenmin Sun
Mingming Sun
Dianjianyi Sun
Ju Sun
Xiaoshai Sun
Jimin Sun
Shiwen Sun
Bo Sun
Hongyan Sun
Gui-Quan Sun
Wenchao Sun

Ying Sun
Lena Sun
Jingbo Sun
Zhenjun Sun
Yaning Sun
Jianlong Sun
Hong-Qiang Sun
Xin Sun
Shi Sun
Yu-Xin Sun
Yanan Sun
Yun Sun
Xiao Sun
Wenxiang Sun
Zhen Sun
Yingpu Sun
Qun Sun
Bai-Nian Sun
Mei Sun
Hokeun Sun
Chunwen Sun
Xiaojun Sun
Yu Sun
Peng Sun
Aixin Sun
Tongxing Sun
Yunzhang Sun
Xin-Yang Sun
Di Sun
Xiaoshuai Sun
Xuemei Sun
Jim Sun
Huili Sun
Hyeon-Jin Sun
Yujun Sun
Junkui Sun
Jing Sun
Lun-Quan Sun
Xiaoqiang Sun
Dingzhong Sun
Ji Sun
Yixiao Sun
Shuying Sun
Dan Sun
Haipeng Sun
Zhiyi Sun
Pei Sun
Guihong Sun
Miao Sun
Desheng Sun
Zhengda Sun
Jiashu Sun
Michelle Sun
Bao-Liang Sun
Dongbo Sun
Zhaogui Sun
Yuanming Sun
Qiang Sun
Li-Ping Sun
Jianwei Sun
Shiquan Sun
Chandru Sundaram
Raman Sundaram
Paul Sundaram
Vijaya Sundararajan
T. Sundararaman
Nagalingam Sundaresan
Gobalakrishnan Sundaresan
Eric Sundberg
Svein Sundby
Marianne Sunde
Svein Sunde
Kevin Sunde Oppegaard
Caroline Sunderland
Matthew Sundermann
Simon Sundermann
Perola Sundin
Premanand Sundivakkam
Mark Sundrud
Paula Sundstrom
Selim Suner
Li-Ying Sung
Shian-Ying Sung
Junne-Ming Sung
Joohon Sung
Jong-Hyuk Sung
Kim Sunggil
Venkata Sunkesula
Susan Sunkin
Katharina Sunnerhagen
Paul Sunnucks
Xun Suo
Zhenhe Suo
Sakari Suominen
Tarja Suominen
Arho Suominen

Fran Supek
Charles Super
Virginie Supervie
Gerald Supinski
Philip Supply
Claudiu Supuran
Enrico Surace
Madhuri Suragani
G.K Suraishkumar
Roel Suralta
Krishna Mohan Surapaneni
Thilina Surasinghe
Prashanth Suravajhala
Wilson Suraweera
Simona Surdu
Camille Sureau
Sripathi Sureban
Xisca Sureda
M. Suresh
Aneesha Suresh
M.A. Sureshkumar
Michael Surette
Sunjay Suri
Fernanda Surita
Joachim Surm
Jennifer Surtees
Kadek Agus Surya Dila
M H Suryanarayana
Gajendra Suryawanshi
Bambang Suryobroto
Antonia Susca
Maciej Suski
Cory Suski
Rodolphe Suspène
Rossana Sussarellu
Ezra Susser
Mark Sussman
Dafna Sussman
Abraham Susswein
Robynne Sutcliffe
Steven Suter
Kelly Suter
Sílvia Maria Suter Correia Cadena
Manikkam Suthanthiran
Colin Sutherland
Ian Sutherland
Greg Sutherland
Andy Sutherland
Kate Sutherland
Tara Sutherland
Marcia Sutherland
Scott Sutherland
Andrew Sutherland
Ben Sutherland
Iain Suthers
Gerd Sutter
David Sutter
Matthias Sutter
Glenn Sutter
Hedwig Sutterlüty-Fall
Fayyaz Sutterwala
Sutas Suttiprapa
Jirí Suttnar
Tina Sutton
Nora Sutton
Jennifer Sutton
Richard Sutton
Gerard Sutton
Jolene Sutton
Jane Sutton
Melanie Sutton-Mcdowall
Tom Sutula
Jolien Suurmond
Larry Suva
Susmit Suvas
Murari Suvedi
Jaana Suvisaari
Yasushi Suwazono
Zhang Suxia
Kazutomo Suzue
Noboru Suzuki
Makuto Suzuki
Hiromu Suzuki
Katsuaki Suzuki
Takashi Suzuki
Iwane Suzuki
Osamu Suzuki
Nao Suzuki
Motoi Suzuki
Keiji Suzuki
Yuko Suzuki
Hideaki Suzuki
Yuichiro Suzuki
Toru Suzuki
Takayoshi Suzuki
Yuriko Suzuki

Yoshiyuki Suzuki
Hitoshi Suzuki
Michio Suzuki
Harumi Suzuki
Takeshi Suzuki
Haruo Suzuki
Ritsuro Suzuki
Etsu Suzuki
Masataka Suzuki
Ippei Suzuki
Shigeaki Suzuki
Ken-Ichi T Suzuki
Satoru Suzuki
Miwa Suzuki
Masaharu Suzuki
Hironori Suzuki
Kohta Suzuki
Gen Suzuki
Hidekazu Suzuki
Yoshihito Suzuki
Go Suzuki
Yoshihiro Suzuki-Karasaki
Ramesh Sv
Walter Svagelj
Richard Svanbäck
Catharina Svanborg
Eva Svanborg
Staffan Svard
Kurt Svärdsudd
Marta Svartman
Annika Svedholm-Häkkinen
Signe Sveegaard
Baldur Sveinbjornsson
Erik Svendsen
Emma Svennberg
Jens-Christian Svenning
Sine Svenningsen
Ola Svenson
Johan Svensson
Birte Svensson
Viktoria Svensson
Jannet Svensson
Mikael Svensson
Marcus Svensson-Frej
Yuriy Sverchkov
Aaron Sverdlov
Vesna Svetlicic
Valentina Svicher
Terezinha Svidzinski
Dmitri Sviridov
Vitaliy Sviripa
Mario Svirsky
Sergio Svistoonoff
Hanna Svitina
Harlan Svoboda
Jana Svobodová
Kayleigh Swaggart
Mark Swain
Amanda Swain
James Swain
Dilip K Swain
Edward Swain
Martin Swain
Catherine Swales
Rasmus Swalethorp
Hermann Swalve
Viren Swami
V. Swami
Gayathri Swaminath
Soumya Swaminathan
Sanjay Swaminathan
Meenupriya Swaminathan
Shankar Swaminathan
Musti Swamy
Mallikarjuna Swamy
Geeta Swamy
Lourens Swanepoel
Todd Swannack
Maurice Swanson
David Swanson
Brett Swanson
Kendall Swanson
Clarence J. Swanton
Helen Swarbrick
Katherine Sward
Karl Swärd
Snehasikta Swarnakar
Supriya Swarnkar
Alison Swartz
Johnna Swartz
Richard Swartz
Ann Swartz
Talia Swartz
Sanjay Swarup
Ghanshyam Swarup
Brooke Swash

Ronald Swatzyna
Joann Sweasy
Hugh Sweatman
Kristin Swedish
Jon Sweeny
Matthew Sweet
Michael Sweet
Kevin Sweet
Lawrence Sweetman
Neil Sweezey
Nadera J. Sweiss
Fadi Sweiss
Tony Swemmer
Dallas Swendeman
Jennifer Swenson
Russell Swerdlow
Elfie Swerts
Luc Swevers
Donald Swiderski
Izabela Swiecicka
Jakub Swiercz
Frauke Swieringa
Szymon Swiezewski
Simon Swift
Damon Swift
Larry Swift
David Swigon
William Swindell
Jean Swings
Susan Swithers
Marek Switonski
Neil Switz
Christopher Switzer
Steven Swoap
Jim Swoger
Gregory Sword
W. Edward Swords
Sherwin Sy
Ming Hui Sy
Kirtimaan Syal
Adam Sybilski
Vanphanom Sychareun
Zainulabeuddin Syed
Khajamohiddin Syed
Mansoor Ali Syed
Sheyum Syed
Mansoor Syed
Zain Syed
Zeeshan Syed
Josef Syka
Catherine Sykes
Peter Sykora
Sergiy Sylantyev
Francesca Sylos-Labini
Jason Sylvan
Richard J Sylvester
Francisco Sylvester
Michel Sylvestre
Allison Sylvetsky-Meni
Christos Symeonides
David Symer
Andrew Symes
Lene Symes
Lorraine Symington
Matthew Symmonds
Michael Symonds
V. Symonds
Celia Symons
Anne Synnes
Kostas Syrigos
Ilya Sysoev
Vladimir Sytnyk
Ann-Christine Syvänen
Marte Syvertsen
Laszlo Szabados
Florian Szabados
Judit Szabo
Aniko Szabo
Kornélia Szabó
Krzysztof Szade
Szymon Szafranski
Arpad Szallasi
Ivett Szalma
Ewa Szalowska
Szabolcs Számadó
André Szameitat
Zoltan Szantoi
Sarah Szanton
Agnieszka Szarkowska
Ben G. Szaro
Tibor Szarvas
Janelle Szary
Eörs Szathmáry
Jin Szatkiewicz
Lisa Szatkowski
Sara Szczepanski

Agnieszka Szczepek
Izabela Szczerbal
Danuta Szczesna-Cordary
Spencer Szczesny
Daniel Sze
Judit Szecsi
Pal Szecsi
Eva Szegezdi
Zoltan Szekanecz
Jidapa Szekely
Eszter Szekely
Tamás Székely
Michael Szell
Otto Szenci
Zsolt Szendro
Julia Szendroedi
Zoltán Szentesi
Laszlo Szereday
Andrew Szeri
Nicholas Szerlip
Cheuk-Chun Szeto
Myron Szewczuk
Tim Szewczyk
Cristina Szigyarto
Andrew Szilagyi
Amir Szitenberg
Damian Szklarczyk
Marek Szklarczyk
Grazyna Szklarz
Igal Szleifer
Stephanie Szobota
István Szokodi
Michael Szostak
Moriah Szpara
Krzysztof Szpila
Elizabeth Sztul
Alexander Szubert
Pawel Szulc
Robert Szulcek
Dávid Szüts
Gregor Szycik
Aneta Szymanska
Monika Szymanska-Czerwinska
Boleslaw Szymanski
Piotr Szymor
Thomas Szyperski
Paul Szyszka
Mieczyslaw Szyszkowicz
Garden Tabacchi
Joel Tabak
Benjamin Tabak
Valentina Tabanelli
Yasuharu Tabara
Vincent Tabard-Cossa
Lucia Tabares
Antoine Tabarin
Thibault Tabarin
Bruce Tabashnik
Samiya Tabassum
Yasuhiko Tabata
Takako Tabata
Niloofar Tabatabai
Louisa Tabatabai
Karsten Tabelow
F. Robert Tabita
Nathaniel Tablante
Giselle Taboada
Maria Taboada
Whitney Tabor
Holly Tabor
Barbara Taborsky
Michael Taborsky
Rodolphe Tabuce
Takahiro Tabuchi
Hiroko Tabunoki
Anne-Marie Taburet
Carlo Tacchetti
Fabio Taccone
Souvenir Tachado
Karine Tache
Ruth Tachezy
Nana Tachibana
Ken Tachibana
Tetsuya Tachibana
Hirokazu Tachikawa
Masashi Tachikawa
Cees Tack
Frank Tacke
Björn Tackenberg
Alan Tackett
Jennifer Tackett
Paul Tacon
Joanne Tactikos
Yoshifumi Tada
Yuichi Tada
Stefano Taddei

Maria Taddei
Tamar Hamosh Taddei
Getachew Tadesse
Vanja Tadic
Arbel Tadmor
Yaakov Tadmor
Mariane Tadros
Elizabeth Tadros
Dirk Taeger
Miriam Taegtmeyer
Carolina Tafalla
Mary Anne Tafuri
Agostino Tafuri
Gentaro Taga
Elya Tagar
Harry Tagbor
Ashujit Tagde
Michael Taggart
Mansour Taghavi Azar Sharabiani
Mansoureh Taghavinia
Tom Taghon
Magnus Tagil
Elda Tagliabue
Roberto Tagliaferri
Lidia Tagliafierro
Maurizio Taglialatela
Jared Taglialatela
Orazio Taglialatela-Scafati
Corinne Tagliarina
Franco Tagliaro
Enzo Tagliazucchi
Satoru Taguchi
Toshitsugu Taguri
Toru Tagushi
Muhamed-Kheir Taha
Rachida Tahar
Hidetoshi Tahara
Shahrad Taheri
Muhammad Tahir
Tahir Tahirov
Andrew Tai
Hsin-Hsiung Tai
Changfeng Tai
Phillip Tai
Chi-Ming Tai
Dar-In Tai
George Tai
Amos Tai
Guihua Tai
Redha Taiar
Nur Taib
Ludovic Tailleux
Vahid Taimouri
You-Lin Tain
Marko Tainio
Joseph Tainter
Hanna Taipaleenmaeki
Alan Tait
Babafemi Taiwo
Temitope Taiwo
Wu Taixiang
Rhim Taiyoun
Naoki Tajiri
Yuji Tajiri
Heidar-Ali Tajmir-Riahi
Lucia Tajoli
Homa Tajsharghi
Himanshu Tak
Teruhiro Takabe
Karoly Takacs
Ayato Takada
Shinji Takada
Yoshikazu Takada
Koji Takada
Toyoyuki Takada
Yoshikazu Takaesu
Kentaro Takagi
Hiroshi Takagi
Haruto Takagishi
Teruhiko Takahara
Mitsuyoshi Takahara
Akinori Takahashi
Hidehiko Takahashi
Takuya Takahashi
Tsutomu Takahashi
Tomoyuki Takahashi
Yusuke Takahashi
Mizuki Takahashi
Toshimitsu Takahashi
Nobuyuki Takahashi
Masanobu Takahashi
Masafumi Takahashi
Hidetoshi Takahashi
Daniel Takahashi
Yoshio Takahashi
Hideyuki Takahashi

Jun Takahashi
Kyo Takahashi
Toshiyuki Takahashi
Ryoji Takahashi
Aki Takahashi
Shigeru Takahashi
Kyoko Takahashi
Hiroko Takahashi
Satoshi Takahashi
Ken Takahashi
Miyako Takaki
Akinobu Takaki
S. Takaki
Kaoru Takakusaki
Tetsuya Takakuwa
Amol Takalkar
Tadashi Takamizo
Iseki Takamoto
Noboru Takamura
Eriko Takano
Elena Takano
Hitoshi Takano
Takehito Takano
Keisuke Takano
Tomoko Takano
Camila Takáo Lopes
Machimura Takashi
Inotsume Takashi
Shogo Takashiba
Fugo Takasu
Fuyuko Takata
Fusako Takayama
Koji Takayama
Shin'Ichi Takeda
Makio Takeda
Makoto Takeda
Minoru Takeda
Atsushi Takeda
Shin-Ichi Takeda
M. Takeda
Hidetoshi Takedatsu
Takeshi Takegaki
Kaoru Takegawa
Nori Takei
Nobuhiro Takemae
Ken-Ichi Takemaru
Hiromu Takematsu
Hiroshi Takemori
Kosuke Takemura
Chisato Takenaka
Shinsuke Takeno
Emura Takeshi
Haruo Takeshita
Junko Takeshita
Yui Takeshita
Osamu Takeuchi
Masaru Takeuchi
Tsutomu Takeuchi
Kengo Takeuchi
Kenji Takeuchi
Tohru Takeuchi
Naoko Takezaki
Toshiro Takezaki
Sukhjit Takhar
Faten Taki
Hirofumi Taki
Eiki Takimoto
Ken Takiyama
Adam Takos
Salma Taktek
Era Takumi
Toru Takumi
Michael Tal
Adel Talaat
Mohammad Talaei
Andrew Talal
Jason Talanian
Jamsheer Talati
Megha Talati
Karel Talavera
Paul Talbert
Erin Talbert
Jhimmy Talbot
Guylaine Talbot
Nick Talbot
Mahshid Talebi-Taher
François Talfournier
Pedro Talhinhas
Paola Talia
Farid Talih
Ambrose Talisuna
Stefan Talke
Chantal Tallaksen
Douglas Tallamy
Miikka Tallavaara
Tiphaine Tallec

Costellia Talley
Karen Tallman
Tatjana Tallo
Catherine Tallon-Baudry
James Talmadge
David Talmy
Marina Taloyan
Moshe Talpaz
Jaya Talreja
Dana Talsness
Eldin Talundzic
Gursaran Talwar
Cheuk-Ming Tam
Yat Hung Tam
Charmaine Tam
Emily Tam
Yosuke Tamada
Koji Tamada
Tetsuro Tamaki
Yasunobu Tamaki
Kenichi Tamama
Rakesh Tamang
Suzanne Tamang
Mayumi Tamari
Jordi Tamarit
Daniel Tamarit
Markus Tamás
Viola Tamási
Rita Tamayo
Teresa Tamayo
Craig Tambling
Victor Tambone
Cecilia Tamborindeguy
Rosemary Tambouret
Jerome Tamburini
Paul Tambyah
Luigi Tamé
Michele Tameris
James Tamerius
Berhanu Tameru
Marco Tamietto
Lynda Tamine Lechani
Jelmer Tamis
Motohiro Tamiya
Michael Tamkun
Ernst Tamm
Toomas Tammaru
Tuomas Tammela
Martin Tammemagi
Imke Tammen
Markku Tammi
Manu Tamminen
Jakke Tamminen
Manuele Tamo
Yoshikatsu Tamori
Gintautas Tamulaitis
Hiroshi Tamura
Yuichi Tamura
Ken-Ichi Tamura
Koutarou Tamura
Masahito Tamura
Yasuaki Tamura
Nguan Soon Tan
Aik Choon Tan
Shumin Tan
Ming Tan
Suet-Mien Tan
Iain Tan
Gene Tan
Zhiqun Tan
Haidong Tan
Wenjie Tan
Min-Han Tan
Lay Poh Tan
Kar-Chun Tan
Shu-Ping Tan
Jiangning Tan
Wenchang Tan
Ching-Ting Tan
Darrell Tan
Wenyong Tan
Cheemeng Tan
Roderick Tan
Chalet Tan
Xiaoyue Tan
Judy Tan
Lan Tan
Yuliang Tan
Xiaodong Tan
Dunxian Tan
Yunhao Tan
Xiao-Li Tan
Bee Kang Tan
Bruce Tan
Xungang Tan
Rui Zhen Tan

Poh Seng Tan
Tao Tan
Xiahui Tan
Timothy Tan
Tony Tan
Shukui Tan
Dan Tan
Li Tan
Xuhua Tan
Wenfeng Tan
Min Han Tan
Anjiang Tan
James Tan
Jacqueline Tan
Cher Heng Tan
Andrew Tan
Xiaohong Tan
Zheng-Hong Tan
Peng Tan
Ken Tan
Jane Tan
Hui Shan Tan
Yuying Tan
Ek Kia Tan
Chong Tan
Xiaojun Tan
Chen Tan
Fei Tan
Poh Tan
Yaw Sing Tan
Kay Sin Tan
Maw Pin Tan
Zhenning Tan
Dawn Tan
Irene Tan
Seiji Tanabe
Kouichi Tanabe
Akifumi Tanabe
Nobuhiro Tanabe
Mark Tanaka
Sakae Tanaka
Keiji Tanaka
Tsuyoshi Tanaka
Yasuhito Tanaka
Seiji Tanaka
Yasuaki Tanaka
Kayoko Tanaka
Masaki Tanaka
Minoru Tanaka
Masato Tanaka
Eiji Tanaka
Shinji Tanaka
Ryouichi Tanaka
Martin Tanaka
Eiichi Tanaka
Hidekazu Tanaka
Kenji Tanaka
Lilian Tanaka
Koji Tanaka
Junko Tanaka
Fumiaki Tanaka
Hiroaki Tanaka
Kiyoshi Tanaka
Toshihisa Tanaka
Kanji Tanaka
Radu Tanasescu
Babasaheb Tandale
David Tandberg
Anita Tandle
Francesco Tandoi
Radhika Tandon
Nitin Tandon
Mini Tandon
Pavankumar Tandra
Akito Taneda
Bhupesh Taneja
Atsushi Tanemura
C. Burcin Taner
Lisa Taneyhill
Christoph Tang
Yi-Wei Tang
Yuhong Tang
Bor Luen Tang
Shuang Tang
Yaoliang Tang
Hongzhi Tang
Hengli Tang
Binwu Tang
Jinsong Tang
W. H. Wilson Tang
Chih-Hsin Tang
Hua Tang
Fei Tang
Sen-Lin Tang
Hongwei Tang
Ni Tang

Alice Tang
Sung-Chun Tang
Qizhu Tang
Xian-Liang Tang
Jen-Yang Tang
Yang Tang
Ming Tang
Chengchun Tang
Yinjie Tang
Jie Tang
Weiming Tang
Chengwei Tang
Dingzhong Tang
Dalin Tang
Xiaoying Tang
Feng-Yao Tang
Chaorong Tang
Tingting Tang
Zhenghong Tang
Xianying Tang
Jun Tang
Yi Tang
Yi-Yuan Tang
Vera Tang
Wei-Hua Tang
Gang Tang
Tie-Shan Tang
Simon Tang
Xiangdong Tang
Sha Tang
Shuang-Yan Tang
Haiyang Tang
Xing Tang
Kwan Ho Tang
Huizhen Tang
Kam Tang
X Tang
Kevin Tang
Sanyi Tang
Leihan Tang
Guang-Da Tang
Qirong Tang
Lingli Tang
Xiaojia Tang
Lei Tang
Fen Tang
Elaine Tang
Tieqiao Tang
Hao Tang
Cuong Tang
Qiongyao Tang
Alex Tang
Zhengzheng Tang
Wenwu Tang
Xueying Tang
Lan Tang
Zhenyu Tang
Maoxue Tang
Chong Tang
Yu Tang
Juliet Tang
Shaoting Tang
Yaohui Tang
Buzhou Tang
Weiqing Tang
Minghua Tang
Wanxin Tang
Wenjuan Tang
B.L. Tang
Xiaoqing Tang
Yuanyuan Tang
Jianhua Tang
Qiongying Tang
Andrea Tangari
Subra Tangirala
Mark Tangney
C.C. Tangney
Vin Tangpricha
Jun Tani
Naoki Tani
Yukinori Tani
Satoshi Tanida
Mamoru Tanida
Manabu Tanifuji
Leandro Taniguchi
Koji Taniguchi
Akito Taniguchi
Hiroya Taniguchi
Wataru Taniguchi
Kouzou Taniguchi
Shinichi Tanihara
Heikki Tanila
Keiji Tanimoto
Naoyuki Tanimoto
Hiroshi Tanimoto
Jun Tanimoto

Martin Tanis
Toshihiro Tanizawa
Angelo Tanna
Franziska Tanneberger
Kyle Tanner
Julian Tanner
Nathan Tanner
Bertrand Tanner
Jared Tanner
Bobo Tanner
Dionne Tannetta
Jun Tanno
Luciana Tanno
Lisa Tannock
Jason Tanny
F Tanriverdi
Petri Tanska
Topi Tanskanen
Arnaud Tanti
Jean-François Tanti
Milos Tanurdzic
Bahattin Tanyolac
Robert Tanz
Rudolph Tanzi
Li Tao
Weng Tao
Feng Tao
Mi-Hua Tao
Fangbiao Tao
Ai-Lin Tao
Xiaorong Tao
Dacheng Tao
Fulu Tao
Xiang Tao
Renchuan Tao
Han Tao
Weixin Tao
Yuezhi Tao
Ling Tao
Haiying Tao
Pan Tao
Guo-Zhong Tao
Jun Tao
Wendong Tao
Bachir Taouli
Ken Tape
Donald Taphorn
Francisco Tapiador
Avraam Tapinos
Soile Tapio
Miika Tapio
Marta Tapparo
Elliot Tapper
Paramjit Tappia
Hannah Tappis
Adriana Tapus
Maxime Taquet
Maxime Tarabichi
Albert Taraboulos
Vera Tarakanova
Margaret Tarampi
Cristina Tarango
Giovanni Tarantino
Arnaud Tarantola
Takashi Tarao
Theodore Taraschi
Eustachio Tarasco
Melissa Tarasenko
Ariel Tarasiuk
Aleksej Tarasjev
Stavros Taraviras
John Tarbell
Kristin Tarbell
Adi Tarca
James Tardio
Florence Tardy
Rahul Tare
Meghana Tare
Corrado Tarella
Em Targarona
Kimara Targoff
Bozena Targonska-Stepniak
Ahmad Tarhini
Simson Tarigan
Sergey Tarima
Joseph Tariman
Munshi Tariq
Serhan Tarkan
Ahti H.A. Tarkkanen
David Tarlinton
Rachael Tarlinton
Ioannis Tarnanas
Der-Cherng Tarng
Joel Tarning
Mark Tarnopolsky
Paolo Tarolli

Aaron Tarone
Paola Taroni
Andrea Tarozzi
David Tarpy
Robert Tarran
Ilaria Tarricone
Pedro Tarroso
Arnaud Tarroux
Elena Tartaglia
Carmen Tartari
Stefano Tartarini
Armando Tartaro
Ariana Tart-Zelvin
Liila Taruffi
Valentina Tarzia
Haruko Tashiro
Donald Tashkin
Gregory Tasian
Sarah Tasian
Aurélie Tasiemski
Robert Tasker
Marja-Riitta Taskinen
Peter Tass
Anchalee Tassanakajon
Laura Tassi
Louis Tassinary
Chrysoula Tassou
Mathew Tata
Souzan Tatari
Fedor Tatarinov
Christopher Tate
Jacqueline Tate
Edward Tate
Eric Tate
Matthew Tate
Charlotte Tate
Chemen Tate
Shunsuke Tatebe
Ryosuke Tateishi
Norifumi Tateishi
Yoko Tateishi
Hiroaki Tateno
Andrew Tatham
Amanda Tatler
Denis Tatone
Christophe Tatout
Tomohide Tatsumi
Masami Tatsuno
Avery Tatters
Peter Tattersall
Glenn Tattersall
James Tattersall
Martin Tattersall
Pierre Tattevin
Utpal Tatu
Clifford Tatum
Nick Taub
Christian Taube
Wolfgang Taube
Anna Taubenberger
Pedro Tauler
Riccardo Taulli
Gerardo Tauriello
Naomi Taus
Mohammad Tauseef
Daniel Taussky
Norbert Tautz
Amye J. Tavaarwerk
Jahan Tavakkoli
Norma Tavakoli
Hossein Tavana
Francesca Tavano
Ivan Tavares
Fernando Tavares
Andre Tavares
Joao Manuel R.S Tavares
Mahvash Tavassoli
Giacomo Tavecchia
Nuno Taveira
Ej Tavender
Constanza Taverna
Nektarios Tavernarakis
Marco Taviani
John Tavis
Alessandro Tavoni
Gaia Tavosanis
Anna Tavridou
Hesham Tawfeek
Dan Tawfik
Bill Tawil
Rima Tawk
Christof Taxis
Sun Tee Tay
Hock Tay
Ywee Chieh Tay
Chandrakant Tayade

Jun Tayama
Ichiro Tayasu
Susan Taylor
Emmanuel Taylor
Rachael Taylor
Colin Taylor
Hugh Taylor
Stephen Taylor
Diane Taylor
Jordan Taylor
Michael Taylor
Graham Taylor
Gregory Taylor
Steve Taylor
J. Taylor
Kira Taylor
Peter Taylor
Matthew Taylor
Subhashni Taylor
Veronique Taylor
Scott Taylor
Caroline Taylor
Nicolas Taylor
Lee Taylor
Michelle Taylor
Lynn Taylor
Renea Taylor
Emily Taylor
Eric Taylor
Anne Taylor
Mark Taylor
Angela Taylor
Naomi Taylor
Joy Taylor
Richard Taylor
Paul Taylor
Andrew Taylor
Brett Taylor
Ann Taylor
Sabrina Taylor
Janice Taylor
Jennifer Taylor
John Taylor
Charlotte Taylor
Dennis Taylor
Brandie Taylor
Mark S. Taylor
Christopher Taylor
Nicholas Taylor
David Taylor
Alan Taylor
Melanie Taylor
Zachary Taylor
Dawn Taylor
Tonya Taylor
Samuel Taylor
Ulrike Taylor
Thomas Taylor-Clark
Jennifer Taylor-Cousar
Leanne Taylor-Smith
Bamidele Tayo
Amin Tayyebi
Irenus Tazisong
Guillaume Tcherkez
Andre Tchernof
Nicolas Tchitchek
J. Michel Tchuenche
Dennis Te Beest
Marinus Te Pas
Gregory Teague
Chloe Teasdale
Jan Tebben
Andrew Tebbenkamp
Marc Tebruegge
Mario Tecce
Suchila Techawongstien
Matthew Tector
Phil Tedbury
Thomas Tedder
Leho Tedersoo
Anna Tedeschi
Gabriele Tedeschi
Francesco Tedesco
Ryan Tedford
Nancy Tee
Andrew Tee
Justin Teeguarden
Bela Teeken
Jamie Teer
Teemu Teeri
Julia Teerling
Nicholas Teets
Solomon Teferra
Riitta Tegelberg
Robert Tegg
George Tegos

Bin Tean Teh
Ruth Teh
Aik-Hong Teh
Cindy Shuan Ju Teh
Daniel Teh
Oksana Tehlivets
Jamshid Tehrani
Beverly Teicher
Kristine Teichman
Markus Teige
Jean-Luc Teillaud
Jonas Teilmann
Martin Teintze
David Teis
Pierre-Louis Teissedre
Andrzej Teisseyre
Justin Teissie
Milt Teitler
Santuza Teixeira
Mauro Teixeira
Lúcia Teixeira
Henrique Teixeira
Cristina Teixeira
Natércia Teixeira
La Teixeira
Paula Teixeira
Ana Teixeira
Gerlinde Teixeira
Samantha Teixeira
Celia Teixeira
Renan Teixeira
Paul Teixeira
Cláudia Teixeira
Fatima Teixeira-Clerc
Armando Teixeira-Pinto
Cristina Teixido
Julian Tejada
Miguel Tejedo
Jesus Tejero Bravo
Gergely Tekes
Zeki Tekgul
Ayse Tekinay
Maria Tektonidou
Aparna Telang
Flavia Teles
Luciano Telesca
Stéphane Téletchéa
Frank Telewski
Janice Telfer
Sam Telford Iii
José Tella
Olivier Telle
Oiana Telleria
Noelia Tellez
Guillermo Tellez
Timothy Tellinghuisen
Marina Telonis-Scott
Ariel Telpaz
Langa Tembo
Philip Temby
Johnna Temenoff
Lesly Temesvari
Kevin Temeyer
Nora Temme
Marleen Temmerman
Dennie Tempel
Christian Temperli
Shelby Temple
Thomas Templeton
Douglas Templeton
Matthew Templeton
Steven Templeton
Michael Templeton
William Templin
Andre Tempone
Simone Temporal
Jean-Jacques Temprado
Vadim Ten
Hugo Ten Cate
R. Ten Cate
Arina Ten Cate-Hoek
B. Ten Haken
Rogier Ten Hoopen
Gavin Ten Tusscher
Alejandro Tena
Klaus Tenbrock
Michaela Tencerova
Scott Tenenbaum
Alexander Tenenbaum
Arthur Tenenhaus
Lida Teneva
Ching-Hao Teng
Zhongzhao Teng
Ying Teng
Ru-Jeng Teng
Sheng Teng

Jessica Teng
Lisong Teng
Onno Teng
Hsiang-Ling Teng
Tjoon-Tow Teng
Michael Teng
Santani Teng
Weiping Teng
Margareta Tengberg
Bernd-Alois Tenhagen
Mirja Tenhunen
Fitsum Sebsibe Teni
Dan Tennant
David R. Tennant
Scott Tenner
Jeffrey Tenney
Meredith Tennis
Martin Tenniswood
John Tentler
Nikolas Tentolouris
Adrian Teo
Koon Teo
Teck Hui Teo
Serena Teo
Miang Chneh Teo
Aaron Teo
Wei-Peng Teo
Mihaela Teodorescu
Kinneret Teodorescu
Grazielle Teodoro
May Teoh
Sachin Teotia
Raffaele Teperino
Alan Tepley
Max Teplitski
Roby Teply
Nadiya Teplyuk
Beverly Tepper
James Tepper
Rob Tepper
Tom Ter Bogt
Arjan Ter Horst
Feiko Ter Kuile
Lance Terada
Shuji Terai
Masanori Terajima
Masaru Teramoto
Takao Terano
Joaquín Terán-Santos
Junji Terao
Yasuo Terao
Chikashi Terao
Takeshi Terao
Eriko Terao
Mutsumi Teraoka
Ei Terasawa
Yasuo Terauchi
Shin-Ichi Terawaki
Wilma Terblanche
David Terburg
Ana Tercero
Dimitrios Terentes-Printzios
Andrew Terentis
Dmitry Terentyev
Larisa Tereshchenko
Devin Terhune
Theron Terhune
John Terhune
Fatiha Terki
David Terman
Nigel Ternan
Javier Terol
Mishka Terplan
Nicole Terpolilli
Paula Terraciano
Xavier Terradas
Jorge Terrados
Nicolás Terrados
Paul Terranova
Christopher Terranova
Nora Terrasini
César Terrazas
Luis Terrazas
Kathryn L. Terry
Robert Terry
John Terry
Jefferson Terry
Anna Terry
Christi M Terry
Josephine Terry
Ivarne Tersariol
Mari Tervaniemi
Osmo Tervonen
Tommi Tervonen
Stefan Terzer
Valeria Terzi

Ana Terzian
Greg Tesch
Andrew Teschendorff
Kay Teschke
Carolyn Teschke
Giuseppina Tesco
Markos Tesfaye
Dawit Tesfaye
Samuel Teshome
Chiara Tesi
Vera Tesic
Yasvir Tesiram
Theresa Teslovich
Giovanni Tesoriere
May-Britt Tessem
Fasil Tessema
Philippe Tessier
Antonio Tessitore
Claudio Tessone
Claudia Testa
Stephen Testa
Fernando Testai
Lara Testai
Pilar Testillano
Mario Testini
Daniel Teta
Frank Tetart
Jens Tetens
Ken Teter
Diana Teti
Anna Teti
Ian Tetlow
Sotirios Tetradis
Marie-Pier Tetreault
Lindsay Tetreault
Tetsuhiro Tetsuhiro Tanaka
Ciro Tetta
Gianluca Tettamanti
Mauro Tettamanti
Raymond Tetteh
Martin Teufel
Robin Teufel
Jacques Teulon
Alexander Teumer
Marcel Teunissen
Charlotte Teunissen
Paul Teunissen
Miguel Teus
Cory Teuscher
Friedrich Teuscher
Sergei Tevosian
Kenneth Tew
Asheesh Tewari
Rita Tewari
Shivendra Tewari
Deepanker Tewari
Haile Tewolde
Daniel Tews
Erik Tews
Paula Texeira
Stephen Textor
Faik Tezcan
Sophie Tezenas Du Montcel
Ayumi Tezuka
Sara Tezza
Anil Thachil
Jecko Thachil
Bart Thaci
Christine Thacker
Tyler Thacker
Larissa Thackray
Jose Thaiparambil
Keshari Thakali
Juilee Thakar
Monica Thakar
Charuhas Thakar
Vidhu Thaker
Katharine Thakkar
Diren Thakker
Ammarin Thakkinstian
Seema Thakore Meloni
Preston Thakral
Jitendra Thakur
Mukesh Thakur
Jarnail Thakur
Harshad Thakur
Lore Thaler
Andrew Thaler
Roman Thaler
Lea Thaler
Usha Thamattoor
Madhav Thambisetty
Douglas Thamm
Cindy Thamrin
Nandor Gabor Than
Khoi Than

Vipa Thanachartwet
Rajarajan Thandavarayan
Saravanan Thangamani
Shankar Thangamani
Kumarasamy Thangaraj
Lakshmipriya Thangavel
Raman Thangavelu
Kr Thankappan
Radhakrishnan Thankappan
Victor Thannickal
Solon Thanos
Maria Thanou
Subash Thapa
Roopa Thapar
Binu Tharakan
Pierre-Louis Tharaux
Nishanth Tharayil
Sujeenthar Tharmalingam
Wolfgang Thasler
Thomas Thatcher
Louise Thatcher
Prachi Thatte
Kristina Thayer
Julie Thayer
Jonathan Thayn
Katherine Theall
Maria Theas
Elisa Thebault
Elitza Theel
Nipon Theera-Umpon
Steven Theg
Diethilde Theil
Gregor Theilmeier
Tun-Linn Thein
Hla-Hla Thein
Michael Theisen
Guenter Theissen
Paul Thelen
Antonia Thelen
Eric Thelin
Mike Thelwall
Jason Themanson
Maria Themeli
Markus Themessl-Huber
Florian Then Bergh
Thenappan Thenappan
V. Thenmozi
Sathan Thennarasu
David Theobald
Martin Theobald
Theocharis Theocharides
Achilleas Theocharis
Georgios Theodoridis
Sarah Theodoroff
Athina Theodosiou
Ulrich Theopold
Guy Theraulaz
Nathalie Théret
Sebastien Theriault
Alex G. Therien
Nina Therkildsen
Grant Theron
Gerhard Theron
Guillaume Theroux-Rancourt
Eric Thervet
Clotilde Thery
Khin Thet Wai
Frédéric Theunissen
Leslie Theunissen
Sebastiaan Theuns
Markus Theurl
Michelle Theus
Sundararajah Thevananther
Frank Thevenod
Sophie Thevenon
Catherine Thevenot
Catherine Theves
J. G. M. Thewissen
Dominic Thewlis
Mia Thi
Patrick Thiam
Patrick Thiaville
Olivier Thibault
Helene Thibault
Aurore Thibaut
Susan Thibeault
Jacques Thibodeau
Patrick Thibodeau
Aye Thida
Michel Thiebaut De Schotten
Nathalie Thieblemont
Bernd Thiede
Brian Thiede
William Thiel
Martin Thiel
Christiane Thiel

Jens Thiel
Andra Thiel
Alexander Thiel
Volker Thiel
Alexander Thiele
Christoph Thiele
Todd Thiele
M Thiele
Geoffrey Thiele
Kris Thielemans
Peter Thielen
Jordy Thielen
David Thieltges
Christoph Thiemermann
Renate Thienel
Guillaume Thierry
Bernard Thierry
Benjamin Thierry
Thevenot Thierry
Frank Thies
Vincent Thijs
Bart Thijs
Willemien Thijs
Sofie Thijs
Basky Thilaganathan
Michelle Thill
Aw Thille
Nanda Thimmappa
Marco Thines
Eckhard Thines
Keneuoe Thinyane
Chloe Thio
Marc Thioux
Thimmasettappa Thippeswamy
Parthasarathy D. Thirumala
Soumya Thirumoorthy
Nepolean Thirunavukkarasu
Muthusamy Thiruppathi
Ronald Thisted
David Thistle
Pham Thi-Thanh-Hien
May Thitisaksakul
Mike Thiv
Venkatesan Thiyagarajan
Aaron Thode
Charles Thodeti
Ellen 'Thoen
Wayne Thogmartin
Ernst Tholen
D. Tholl
Matthew Thom
Jeanette Thom
Michael Thom
Myriam Thoma
Götz Thomalla
Wayne Thomas
Gary Thomas
Robert J Thomas
Sean Thomas
Gethin Thomas
Michael Thomas
Kyle Thomas
Gavin Thomas
Christopher Thomas
Emmanuel Thomas
Mª Carmen Thomas
Walter Thomas
Sara Thomas
Nikhil Thomas
Ranjeny Thomas
David Thomas
Pious Thomas
Muriel Thomas
Isabelle Thomas
Dierk Thomas
S. Thomas
Stephanie Thomas
Roger Thomas
Anish Thomas
Ajith Thomas
Christian Thomas
Bernadette Thomas
Miranda Thomas
Florence Thomas
Douglas Thomas
Alan Thomas
Charles Thomas
Stephen Thomas
Valsa Thomas
Jonathan Thomas
Ryan Thomas
Owain Thomas
Philipp Thomas
Mridul Thomas
Christoforos Thomas
Richard Thomas

Jason Thomas
James Thomas
Robert Thomas
Susan Thomas
Brian Thomas
Kathryn Thomas
Barbara Thomas
Daniel Thomas
Elizabeth Thomas
Veena Thomas
Philip Thomas
Frank Thomas
Margaret Thomas
Scott Thomas
Roland Thomaschke
Lynn Thomason
Mads Thomassen
Mary Thomassen
Sidinei Thomaz
Sara Thomee
Sebastien Thomine
Stavros Thomopoulos
Christopher Thompson
Richard Thompson
Kimberly Thompson
Arthur Thompson
William Thompson
Corinne Thompson
Graham Thompson
Jonathan Thompson
Stuart Thompson
Cristiane Thompson
Philip Thompson
Fabiano Thompson
Helen Thompson
Caroline Thompson
Deborah Thompson
C. Thompson
Jennifer Thompson
Scott Thompson
Peter Thompson
Dominic Thompson
Brian Thompson
Henry Thompson
Aiko Thompson
Robert Thompson
Neil Thompson
Beti Thompson
Michael Thompson
Bill Thompson
Allan Randrup Thomsen
Philip Thomsen
Karen Louise Thomsen
Louiza Thomsen
Jörn Thomsen
Trine Thomsen
Peter Thomson
Ashley Thomson
Maria Thomson
Michael Thomson
Axel Thomson
Cynthia Thomson
Jordan Thomson
Bruce Thomson
Murray Thomson
Keith Thomson
Rachel Thomson
Rebecca Thomson
Michael Thon
P.S.P. Thong
Jean-Louis Thonnard
Andri Thorarinsson
James Thorburn
John Thoresen
Wallace Thoreson
Eric Thorin
Henrik Thorlacius
Andrew Thorley
Ulrich Thormann
Kurt Thorn
Simon Thorn
Paul Thornalley
Jennifer Thorne
Stephen Thorne
Peter Thorne
Lesley Thorne
Curtis Thorne
Kevin Thorneloe
Michael Thorner
Randy Thornhill
John Thornhill
Graham Thornicroft
David Thornton
Catherine Thornton
Timothy Thornton
Gail Thornton

Lukar Thornton
Daniel Thornton
Bill Thornton
Chris Thornton
Daniel Thorogood
Margaret Thoroughgood
Kurt Thoroughman
Colin Thorpe
Susannah Thorpe
Jim Thorson
Anne-Luise Thorsteinsson
Christopher Thorstenson
Kristian Thorup-Kristensen
Steinar Thorvaldsen
Priyaleela Thota
Ramya Thota
Umeshkanta Thounaojam
Brian Thrall
James Throne
Edwin Thrower
Mahender Thudi
Barbara Thuer
Padmamalini Thulasiraman
Sarath Thulasiraman
Ryan Thum
Philip Thuma
Vivek Thumbigere-Math
Ryan Thummel
Inger Thune
Lena Thunell
Kimhan Thung
Erik Thunnissen
Maria Thunström
Andrew Thurber
Katherine Thurber
Stefan Thurner
Philipp Thurner
Matthew Thurtell
Gregor Thut
Baskaran Thyagarajan
Bharat Thyagarajan
John Thyfault
Gregory Thyssen
Lianping Ti
Hanna Tiainen
Changhai Tian
Xiaolin Tian
Xiao-Li Tian
Junce Tian
Pu Tian
Xing Tian
Rong Tian
Jianjun Paul Tian
Ling Tian
Lining Tian
Xiaoyu Tian
Ying Tian
Zhixi Tian
Mingxing Tian
Chunjie Tian
Yantan Tian
Chenxi Tian
Jie Tian
Daike Tian
Zhijun Tian
Wei-Min Tian
Longlong Tian
Ye Hong Tian
Meijuan Tian
Hongjun Tian
Yin Tian
Hui Tian
Ye Tian
Chengliang Tian
Lin Tian
Bo Tian
Zhen-Jun Tian
M. Tian
Chuan Tian
Yonghong Tian
Geng Tian
Suqing Tian
Yan Tian
Tianhai Tian
Wu Tian Shung
Guido Tiana
Joseph Tiano
Wang Tianqi
Greg Tiao
Ariadna Tibau
Helen Tibboel
Mario Tiberi
Luca Tiberi
Gianpaolo Tibolla
Roni Tibon
J. Tice

Petr Tichavský
Vladimir Tichelaar
Muriel Tichit
Ales Tichy
Luca Ticini
Dave Tickner
Patrick Tidball
Emmanuele Tidoni
Lu Tie
Yanmei Tie
Jeanne Tie
David Tiede
Erin Tiedeken
Ralph Tiedemann
Henning Tiemeier
Leopold Tientcheu Djomkam
William Tierney
Adam Tierney
Cassandra Tierney
Carla Tiesler
Pam Tietz
Erhard Tietze
Tony Tiganis
Cezar Tigaret
Jesse Tigner
Peter Tiidus
Betty Tijms
Peter Tijssen
Chris Tikellis
Ruben Tikidji-Hamburyan
Kaja Tikk
Ritva Tikkanen
Tizta Tilahun
Marcel Tilanus
Bronte Tilbrook
Michael Tildesley
Laurence Tiley
Vallo Tilgar
Esmerina Tili
Benedikt Till
Kevin Till
Douglas Tilley
Wayne Tilley
Dipti Tillu
Sam Tilsen
Fred Tilton
Robert D. Tilton
Jozsef Timar
Bogdan Timar
Laura Timares
Lubov Timchenko
Dennis Timlin
Stefan Timm
Janne Timm
Reinier Timman
Dagmar Timmann
Christiane Timmel
Kalinka Timmer
Nienke Timmer
Jennifer Timmer
Bert Timmermans
Harry Timmermans
Molly Timmers
J Timmers
John Timms
Vladimir Timoshevskiy
Sebastian Tims
Yoav Timsit
Jean-Francois Timsit
Adrienne Tin
Andrew Tindall
Paulina Tindana
Angela Ting
Kang Ting
Chen-Hung Ting
Chien-Kun Ting
Fan Ting
Wei Ting
Wei-Hsin Ting
Hou Ting-Jun
Morgan Tingley
Reid Tingley
Yotsawan Tinikul
Nicholas Tinker
Paul Tinkler
Peter Tino
Ignacio Tinoco
Mark Tinsley
Hugo Tinto
Cristina Tintori
George Tipoe
Richard Tipping
Brett Tipple
Megan Tipps
Laura Tipton
Judit Tirado Muñoz

Khajohn Tiranathanagul
Carlos Tirapelli
Engin Tiras
Laurence Tiret
Claudio Tiribelli
Virginia Tirino
Venkataswarup Tiriveedhi
Klaus Tiroch
Franck Tirode
Oren Tirosh
C. Tiruppathi
Suma Tiruvayipati
Laurie Tis
Daniel Tisch
Anna Tischler
James Tisdale
Jean-Luc Tison
Juliette Tison-Rosebery
Marie Tisserand
Mathilde Tissier
László Tiszlavicz
Silvia Titan
Kehmia Titanji
Alan Titchenal
Andrew Titman
Andrew Titmus
Luigi Titomanlio
Vladimir Titorenko
Esther Titos
Nickolai Titov
Nina Titova
Derek Tittensor
Kai Tittmann
Alexander Titz
Jens Titze
Alexei V. Tiunov
Amanda Tivnan
Vaibhav Tiwari
Sirish Tiwari
Kapil Tiwari
Siddharth Tiwari
Chetan Tiwari
Manish Tiwari
R. Tiwari
Fung Yee Tiwari
Ashok Tiwari
Harinarayan Tiwari
Vijay Tiwari
Aude Tixier
Michèle Tixier-Boichard
Michele Tizzoni
Sotirios Tjamos
Bosco Tjan
Nico Tjandra
Julius Tjelele
Ruzena Tkacova
Michael Tlauka
E Tlelo-Cuautle
Mustapha Tlidi
Michael Tlusty
Yasuo To
Kelvin To
Sabrina To
Masako To
Raquel Tobes
Dror Tobi
Aaron Tobian
Craig Tobias
Shozo Tobimatsu
Mary K. Tobin
Ursina Tobler
Philippe Tobler
Mathias W. Tobler
Kurt Tobler
Carlo Tocchetti
Glauco Tocchini-Valentini
Jack Tocco
Shin Tochinai
Shiro Tochitani
M. Antonio Todaro
John Todd
James Todd
Jim Todd
Richard Todd
Jamie Todd
Alexandra Todd
Juanita Todd
Rebecca Todd
Andrew Todd
Peter Todd
Michael Todd
Anne Todgham
Sokol Todi
Giancarlo Todiere
Mihail Todiras
Yasushi Todoroki

Todor Todorov
Zoran Todorovic
Stephen Todryk
Alexander Toet
Fahmida Tofail
Rosanna Tofalo
Sergio Tofanelli
Shahnaz Tofangchiha
Lorenzo Tofani
Giuseppe Toffoli
Adam Tofilski
Soren Toft
Søren Toft
Henrik Toft Simonsen
Padma Priya Togarrati
Marcelo Tognelli
Massimiliano Tognolini
Fumiharu Togo
Tetsuhiro Togo
Junya Toguchida
Cheng Hock Toh
Dorothy Toh
Cheng-Hock Toh
Alicia Toh
Chihiro Tohda
Takayuki Tohge
Jussi Tohka
Rania Tohme
Chiharu Tohyama
Gabriele Toietta
Yuji Toiyama
Akihiro Tojo
Akihiko Tojo
Hakan Toka
Andrey Tokarev
Rafal Tokarz
Yaman Toket
Seiichi Toki
Takashi Tokino
Makoto Tokuda
Kazuhiro Tokuda
Fuyuki Tokumasu
Jim Tol
Blanton Tolbert
Stefano Toldo
Magdalena Tolea
Debora Toledo Ramos
Nick Tolimieri
Sue Tolin
Jorge Tolivia
Edward Toll
Douglas Tollefsen
Deanna Tollefson
Mari Mette Tollefsrud
David Tollervey
Krystal Tolley
Keith Tolley
Betsy Tolley
Daniel Tollin
Ralph Tollrian
Eleni Tolma
Marcelo Tolmasky
Vicki Tolmay
Manlio Tolomeo
Eduardo Tolosa
Emanuela Tolosano
Genrich Tolstonog
Valerio Tolva
Sarah Tom
Shigeto Toma
Marieta Toma
Leny Toma
Francesco Tomaiuolo
Pavel Tomancak
Dhanendra Tomar
Andrew Tomaras
Orazio Tomarchio
Stanislav Tomarev
Nobuhiro Tomaru
Hummel Tomas
Jesus Tomas
Josefa Tomás
Terry Tomasek
Agostino Tomasello
Jorge Tomasevic
Dardo Tomasi
Thomas Tomasi
Adam Tomašových
Alice Tomassini
Daniele Tomassoni
John Tomaszewski
Krzysztof Tomaszewski
Analia Tomat
Shunji Tomatsu
Carlos Tomaz
Diana Tomback

Enrico Tombetti
Mario Tombini
Bruce Tomblin
Huseyin Tombuloglu
Seth Tomchik
Sara Tomczyk
Hudson Tomé
João Tomé
Rachel Tomer
Alexandru Tomescu
Maja Tomicic-Christmann
Yasuhiko Tomino
Haruaki Tomioka
Luciana Tomita
Masaki Tomita
Goji Tomita
Shinichiro Tomitaka
Asahi Tomitaka
Susumu Tomiya
Takami Tomiyama
Hirofumi Tomiyama
Kazuhito Tomizawa
Keith Tomlins
Brian Tomlinson
Sean Tomlinson
Michael Tomlinson
Simon Tomlinson
Giovana Tommaso
Joke Tommelein
Mark Tommerdahl
Giuseppina Tommonaro
Tatsuya Tomo
Hiroshi Tomoda
Akemi Tomoda
Oyewale Tomori
Cecilia Tomori
Andrija Tomovic
Ichiba Tomoyuki
Stephen Tompkins
Phillip Tomporowski
Tt Tompuri
Robert Tomsak
Sara Tomzyk
Claudio Tondo
Marcello Tonelli
Fiorella Tonello
Michela Tonetti
Hoang Tong
Xia-Jing Tong
Qingchun Tong
Jingou Tong
Xiaoyong Tong
Haiyan Tong
Wenyong Tong
Yunxia Tong
Allison Tong
Shuping Tong
Chunfa Tong
Jonathan Tong
Yiping Tong
Huichun Tong
Chao-Yang Tong
Xiuhong Tong
Tiezheng Tong
Peter Tonge
Daniel Tonge
Enrico Tongiorgi
Alexandre Tonin
Raffaella Tonini
Giuseppe Tonini
Daniela Toniolo
Pierluigi Toniutto
Tone Tonjum
Christopher Tonkin
Jonathan Tonkin
Sarah Tonkin-Crine
Henri Tonnang
Peter Tonner
Espen Tønnessen
Stephen Tonsor
Serena Tonstad
Pietro Tonutti
John Tooker
Michael Toole
Reuben Tooze
Bob Tooze
Jozsef Topal
Christopher M Topham
Ivan Topisirovic
Cem Topkaya
Hermann Toplak
Sascha Topolinski
Alain Topor
Edward Topp
Kairsty Topp
Jorma Toppari

David Topping
Umut Toprak
Ibrahim Toprak
Elena Torban
Bruce Torbett
D. Torchia
Mark Torchin
Andrew Torda
Atilla Tordai
Adrian Tordiffe
Joan Tordjman
Jan Tordoir
Daniele Torella
Michele Torella
Ruurd Torensma
Jeffrey Toretsky
Troy Torgerson
Paul Torgerson
Benno Torgler
Maria Toribio
Jenny-Ann Toribio
Toshihiko Torigoe
Åsa Torinsson-Naluai
Oivind Torkildsen
Zakary Tormala
Silvia Tornaletti
Maria Lina Tornesello
Brett Tornwall
Nicolás Toro
Daniel Torocsik
Kathryn Torok
Peter Török
Beverly Torok-Storb
John Torous
Natalia Torow
David Torpy
Salvatore Torquato
Andrea Torrão
Vincent Torre
Peter Torre
Iratxe Torre
Antoine Torre
Maria Torrecilla
Laurent Torregrosa
Jordi Torrelles
Montserrat Torremorell
Christopher Torrens
Marta Torrens
Clara Torrento
Miguel Torres
Alfredo Torres
Joaquin Torres
Victor Torres
Claudio Torres
Antoni Torres
Jaume Torres
Rosa Torres
Fernando Torres
David Torres
Alex Torres
Rita Torres
Susan Torres
Tatiana Torres
Jiram Torres
Jeremy Mo Torres
Adriana Torres
Diego Torres
Viviane Torres
Jose Torres Costa
Verônica Torres Da Costa E Silva
Ramon Torres Ruiz
Maria Torres Spicer
Nora Torres-Carrillo
Karoll Torres-Cordido
Almudena Torres-Cornejo
Gloria Torres-Cortés
Julian Torres-Dowdall
Enelio Torres-Garcia
Eduardo Caio Torres-Santos
Dino Torri
Sandra Torriani
Lluís Oviedo Torró
Laura Torroja
Antonio Torroni
Ignasi Torruella
Anders Torstensson
Lluis Tort
Diana Torta
Mauro Torti
Domenico Tortorella
Fartein Torvik
Silvio Tosatto
Benjamin Toscano
Nicola Toschi
Andrew Toseland
Vladimir Toshchakov

Mizobuchi Toshiaki
Hiroshi Toshida
Nobuyuki Toshikuni
Luigi Tosi
Simone Tosi
Laura Tosi
Beau Toskich
Annalisa Tosoni
Elisabetta Tosoni
Alexander Tossi
Umut Tosun
Tibor Tot
Bruno Tota
Gunilla Toth
Peter Toth
Viktor Toth
Attila Tóth
Szilvia Z Tóth
Zsuzsanna Tóth
Vaso Totsika
Pierangela Totta
Patricia Totten
Eliette Touati
Elias Toubi
Julie Toubiana
Brant Touchette
Chafia Touil-Boukoffa
Giota Touloumi
Anestis Touloumis
Aminata Toure
David Touretzky
Ana Lucia Tourinho
Regis Tournebize
Jean-Nicolas Tournier
Donald Tournier
Yannick Tousignant-Laflamme
Emmanuel Toussaint
Eric Toussirot
Hazem Toutounji
Daan J Touw
Maria Touz
Antoine Touze
E. Touze
Sulay A. Tovar
Armando Tovara
Andrey Tovchigrechko
Erica Towle
Terrence Town
Natavudh Townamchai
Jonathan Townend
Jonathan Towner
Ellen Townes-Anderson
Dewayne Townsend
Ellen Townsend
Simon Townsend
Raymond Townsend
Aloen Townsend
Mary Townsend
Tim G Townshend
Randall Toy
Sophie Toya
Tadashi Toyama
Hidenori Toyoda
Hiroo Toyoda
Jun Toyohara
Shinya Toyokuni
Douglas Tozer
Vincent Traag
Maret Traber
Daniah Trabzuni
Terence Tracey
Kalliopi Trachana
Andreea Trache
Daniel Trachsel
Samantha Tracht
Elizabeth Trachtenberg
Howard Trachtman
Kathleen Tracy
Saoirse Tracy
Derek Tracy
Melissa Tracy
Chris Tracy
Jens-Martin Träder
Lara Traeger
Birgit Traeuble
Sarah Tragesser
Lisa Trahan
Giovanna Traina
Heather Traino
Brian Trainor
Zlatko Trajanoski
Eftihios Trakakis
Dmitry Traktuev
Af Tralhao
Irene Tramacere
Marc Tramier

Clinton Trammel
Scott Trammell
Enzo Tramontano
Robert Trampel
Phuoc Tran
Ulrich Tran
Quang-Kim Tran
Thach Tran
Antoine Tran
Cuong Tran
Guy Tran Van Nhieu
Fabrice Tranchida
Lisbeth Tranebjaerg
Tuan Trang
Joan Tranmer
Mark Transtrum
Michael Tranter
Christopher Tranter
Michael Tranulis
Sabrina Trapp
Claudia Trappetti
Louisa Traser
Aaron Trask
Siegfried Trattnig
Mary Traub
Harald Traue
Clay Trauernicht
R. Alberto Travagli
Leonardo Travassos
Ana Traven
Carlo Traverso
Maria Luz Traverso
David Travieso
Gabriel Travis
Pavel M Trávnícek
Sophie Trawalter
Paul Trayhurn
Nikki Traylor-Knowles
Bryan Traynor
Martin Trbusek
Mohamed Trebak
Jonel Trebicka
Anett Trebitz
Corinna Trebst
Donna Treby
Edward Tredgett
Bradley Treeby
Moritz Treeck
Nathan Treff
Giorgio Treglia
John Tregoning
Indi Trehan
Gareth Treharne
Matthias Treiber
Isabelle Treilleux
Emmanuel Treiner
Roi Treister
Sarah Treit
Sven Trelle
Yann Tremblay
Leon Tremblay
Luc Tremblay
Nicolas Tremblay
Jonathan Treml
Martin Tremmel
Elena Tremoli
Stephanie Trend
M. Stephen Trent
Edmondo Trentin
Andrea Trentin
Catherine Trepanier
Lauren Trepanier
Francesco Trepiccione
Marco Trerotola
Martin Tresguerres
Flavia Trettel
Laszlo Tretter
Natalia Tretyakova
Colwyn Trevarthen
Ben Trevaskis
James Trevaskis
Wenda Trevathan
Helen Trevena
Adrian Treves
Sarah Treves-Kagan
Jose Trevino
Maurizio Trevisan
Sara Trevisan
Lucia Trevisi
Viviana Trezza
Omar Triana-Chávez
John Triantafillidis
Kathy Triantafilou
David Tribble
Gena Tribble
Jeffery Triblehorn

Andreas Tribsch
Elena Tricarico
Domenico Tricarico
Elizabeth Triche
Valérie Trichet
Antonia Trichopoulou
Hervé Tricoire
André Tricot
Paula Trief
Steven Triezenberg
Monica Trif
Youssef Trifa
Gianluca Trifirò
Vladimir Trifonov
Oleg V. Trifonov
Dragana Trifunovic
Bernardo Trigatti
Mark Trigg
Chris Triggle
Robert Trigiano
Cesar Trigueros
Thomas Trikalinos
Maria Angeles Trillo
Jeffrey Trimarchi
Virginia Trimble
Mounir Trimeche
Jamma Trinath
Alexandre Trindade
Thu Le Trinh
Benjamin Trinite
Vickery Trinkaus-Randall
Anubhav Tripathi
Manish Tripathi
Rudra Tripathi
Amit Tripathi
Avnish Tripathi
Vishal Tripathi
Garima Tripathi
Kaushlendra Tripathi
Ashok Tripathi
Sandeep Tripathi
Dinesh Tripathi
D. Tripathi
Markandey Tripathi
Baishnab Tripathy
Sucheta Tripathy
Julien Tripette
Jason Triplett
Gilbert Triplett
Lindsay Triplett
N. Triplett
Salvatore Tripodi
Dominique Tripodi
Vilma Tripodoro
Vincenzo Trischitta
Mcclure-Begley Tristan
Cordier Tristan
Stephen Tristram
Luigi Tritapepe
Jennifer Trittmann
Tiziana Triulzi
Stefania Triunfo
Arun Trivedi
Prabodh Trivedi
Hargovind Trivedi
Subrata Trivedi
Alfréd Trnka
Stefan Troche
Iñaki Troconiz
Linda Troeberg
Larissa Troesch
Justin Trogdon
Josef Troger
Ourania Trohatou
Angelo Troia
Teresa Troiani
Christian Troidl
Jörg Trojan
Diana Trojaniello
Maria Trojanowska
Patrick Trojer
Ylva Trolle Lagerros
François Trompier
Laure Tron
Luiz Troncon
Marcos Tronconi
Xoana Troncoso
Sophie Tronel
Arne Tronsmo
Anna Marte Tronsmo
Nicholas Troop
Esther Troost
Michael Tropak
Yaacov Trope
Daniela Tropea
Vincent Tropepe

Scott Troppy
James Trosko
Julia R. Trosman
Andrew Trotman
Ioannis Trougakos
Meredith Troutman-Jordan
Guglielmo Trovato
Jone Trovik
Alex Trowbridge
John Trowsdale
Karen Troy
Erin Troy
Marita Troye-Blomberg
Todd Troyer
Ray Truant
Andrea Trubanova
Massimo Trucco
Elisa Trucco
Geoffrey Truchetti
Jean-Francois Truchon
Johannes Trück
Louis-Éric Trudeau
Francois Trudeau
Lloyd Trueblood
Kristina Trujillo
Andrea Trujillo
Felipe Trujillo-Romero
Tom Trull
Ramon Trullas
James Truman
Andrew Truman
John Trumble
Stephen Trumble
Stephen Trumbo
Christoph Trumm
Walter Trump
Ben Trump
Pavel Trunecka
Hong-Ha Truong
Trong-Kha Truong
Vu Truong-Le
Valentina Truppa
Erkki Truve
Roberto Truzoli
V. Trygonis
Piotr Tryjanowski
Joshua Trzasko
Krzysztof Trzcinski
Stephen Trzeciak
Naomi Tsafnat
Avi Tsafrir
Athanasios Tsaftaris
Fuu-Jen Tsai
Ray Tsai
Shih-Jen Tsai
Chi-Chu Tsai
Eing-Mei Tsai
Yuan-Hsiung Tsai
Hui-Ju Tsai
Kelvin Tsai
James Tsai
Keng-Chang Tsai
Wei-Bor Tsai
Wen-Chieh Tsai
Ying I. Tsai
Yu-Huan Tsai
Ching-Piao Tsai
Chung-Fen Tsai
Jack Tsai
Rong-Kung Tsai
Ming-Ju Tsai
Shang-Ru Tsai
Pei-Chien Tsai
Kuen-Horng Tsai
Henghsiu Tsai
Chen-Gia Tsai
Shin-Fu Tsai
Siu Tsai
Sang-Bing Tsai
Wen-Chan Tsai
Chang-Youh Tsai
Chia-Fen Tsai
Kuen-Jer Tsai
Jie-Li Tsai
Jaw-Ji Tsai
Tsung-Yuan Tsai
Tsung-Yu Tsai
Hsin-Lin Tsai
Isheng Tsai
Chia-Liang Tsai
Wei-Lun Tsai
Chieh-Chih Tsai
Peter P. Tsai
Konstantinos Tsakalis
Panagiotis Tsakanikas
Athanassios Tsakris

Yu-Tse Tsan
Stephen Tsang
Michael Tsang
Suk Ying Tsang
Kwok Yeung Tsang
Chi-Ching Tsang
Jo-Ann Tsang
Wang Tsang-En
Jean Tsao
Kimberly Tsao
Henry Tsao
Anastasios Tsaousis
Konstantina Tsaparas
Chia Jung Tsay
Petra Tschakert
Stefan Tschanz
Thomas Tschernig
Rea Tschopp
Emanuel Tschopp
Daniel J. Tschumperlin
Herman Tse
Hubert Tse
Gary Tse
Brian Tse
Harley Tse
Chun-Yu Tse
Yuk-Ching Tse-Dinh
Wayland Tseh
Kostas Tselios
Hung Tseng
Shun-Fu Tseng
Ching-Ping Tseng
Chien-Te Kent Tseng
Yufeng Jane Tseng
Zhijie Jack Tseng
Tai-Chung Tseng
Kevin Tseng
Ping-Huei Tseng
Kuo-Kun Tseng
Chih-Wei Tseng
Deng-Yu Tseng
Li-Chun Tseng
Kuo-Chih Tseng
Fan-Gang Tseng
George Tserpes
Konstantinos Tsetsos
Aspasia Tsezou
Kevin Tsia
Evangelos Tsiambas
George Tsiamis
Evangelia Tsiani
George Tsianos
Chryssa Tsiara
Georgios Tsiavaliaris
John Tsibris
Anne Tsicopoulos
Dimitros Tsikas
Konstantinos Tsilidis
Karl Tsim
Nikolaos Tsirikos Karapanos
Stella Tsirka
Margarita Tsiros
Elena Tsitsami
Dimitrios Tsitsigiannis
Daniel Tso
Geoffrey Tso
Luke Tso
Lai Sze Tso
Emmanuel Tsochatzis
Oleg Tsodikov
Alexander Tsodikov
Benjamin Tsofa
Lam Tsoi
Sophia Tsoka
Christos Tsokos
Apostolos Tsolakis
Lungiswa Tsolekile
Renée Tsolis
Theodore Tsotsis
Ming-Hsiang Tsou
Hsiao-Hui (Sophie) Tsou
Pantelis Tsoulfas
Georgios Tsounis
Tatiana Tsoutsman
Sugawara Tstsuya
Takeshi Tsubata
Takafumi Tsuboi
Takuya Tsuchihashi
Kenji Tsuchiya
Naoyuki Tsuchiya
Naoto Tsuchiya
Ken Tsuchiya
Masao Tsuchiya
Makoto Tsuda
Leo Tsuda
Yoshiaki Tsuda

Akira Tsuda
Soichiro Tsuda
Hiroyuki Tsuda
Naoto Tsuda
Shinya Tsuda
Takanori Tsuda
Kin Ming Tsui
Nancy Tsui
Stephen Kwok-Wing Tsui
Ting Tsui
Venus Tsui
Shoji Tsuji
Yoshiaki Tsuji
Takemasa Tsuji
Atsushi Tsuji
Akitaka Tsujikawa
Takahiro Tsujikawa
Tetsuro Tsujimoto
T Tsujimura
Ichizo Tsujino
Naohisa Tsujino
Eiji Tsujita
Kenichi Tsujita
Hideo Tsukada
Tomohide Tsukahara
Takashi Tsukamoto
Tetsuya Tsukamoto
Satoshi Tsukamoto
Mitsutoshi Tsukimoto
Noriyuki Tsumaki
Daisuke Tsumune
Masayuki Tsuneki
Andrew Tsung
Kanami Tsuno
Susan Tsunoda
Oren Tsur
Masato Tsurudome
Kazuhiko Tsuruya
Hidekazu Tsutsui
Kimiko Tsutsui
Masato Tsutsui
Rie Tsutsumi
Mikihiro Tsutsumi
Yutaka Tsutsumi
Tsvetan Tsvetanov
Alexander Tsygankov
Tetyana Tsykun
Vassiliy Tsytsarev
Jumin Tu
Qichao Tu
Xiaolin Tu
Wen-Jun Tu
Lili Tu
Cong Tu
Shen Tu
Huakang Tu
Feiyun Tu
Jianhua Tu
Samson Tu
Jeffrey Tuan
Balwant Tuana
Roberto Tuberosa
Shea Tuberty
Tracy Tuberville
Eszter Tuboly
Valter Tucci
Franca Maria Tuccillo
Tiziano Tuccinardi
Mihran Tuceryan
Valery Tuchin
Janusz Tucholski
Abigail Tucker
Priscilla Tucker
Budd Tucker
Aimee Tucker
Jalie Tucker
Elena Tucker
Nicholas Tucker
Abraham Tucker
Conrad Tucker
Haley Tucker
Kylie Tucker
Anita Tucker
Robert Tuckey
Edward Tuddenham
Megan Tudor
Paul Tudzynski
David Tuerlinckx
Vincenzo Tufarelli
Alda Tufro
Sharof Tugizov
Cagla Tukel
Aiman Tulaimat
David Tuller
Jonathan Tullis
Robert Tulloh

Damien Tully
Erin Tully
Mary Tully
Florin Tuluc
Madalina Tuluc
Hayrettin Tumani
Semih Tumen
Santa Tuminia
James Tumlin
James Tumlinson
Shyam Sunder Tummanapalli
Michele Tumminello
Innocent Tumwebaze
Elizabeth Tunbridge
Nurcan Tuncbag
Pürhan Tuncer
Smanla Tundup
Gisela Tunes Da Silva
Jenny Tung
Chun-Wei Tung
Yu-Chi Tung
Bui Thanh Tung
Chih-Wei Tung
Chih-Kuan Tung
Su Tung-Hung
Gro Tunheim
José Tuñón
Tomi-Pekka Tuomainen
Helena Tuomainen
Elaine Tuomanen
Jouni Tuomi
Jaakko Tuomilehto
Raimo Tuominen
Hanna Tuomisto
Jouni Tuomisto
Delphine Tuot
Rossella Tupler
Josep Tur
Carmen Tur
Kiran Turaga
Angela Turalba
Janet Turan
Bulent Turan
Ilker Turan
Ferit Turanli
Tamás Turányi
Chiara Turati
Graziella Turato
Massimo Turatto
Guillaume Turc
Gerardo Turcati
Andreia Turchetto Zolet
John Turchi
Gleb Turchinovich
Ronald Turco
Elena Turco
Lucia Turcoková
Borbala Turcsan
Gustavo Turecki
Michael Turell
Nicolas Turenne
Kürsad Turgay
Robert Turgeon
Julie Turgeon
Ali Turhan
Zsolt Turi
Charles Turick
Maurizio Turiel
Emanuela Turillazzi
Massimo Turina
Vito Turk
Boris Turk
Serdar Turkarslan
Baris Türkbey
Levent Türkeri
Anne Turkova
Mustafa Turkyilmazoglu
M. Turkyilmazoglu
Matt Turley
Glen Turley
Ted Turlings
Katarzyna Turnau
Jeremy Turnbull
Matthew Turnbull
Colin Turnbull
Isaiah Turnbull
Andrew Turnell
Dennis Turner
David Turner
Neil Turner
Ray Turner
Alan Turner
Paul Turner
Steve Turner
Raymond Turner
Andrew Turner

Martin Turner
Jefferson Turner
Karly Turner
Dennis C. Turner
Nancy Turner
Wendy Turner
Tony Turner
Robin Turner
Bradley Turner
Sheila Turner
Neill Turner
Shane Turner
Katie Turner
Annie Turner
Kendrick Turner
Lesley-Anne Turner
James Turner
Tychele Turner
Lauren Turner-Brown
Esther Turnhout
Conny Turni
Xavier Turon
Konstantin Turoverov
Tomasz Turowski
Bernd Turowski
Williams Turpin
Cuauhtemoc Turrent
Francesca Turroni
Anna Turska-Szewczuk
Ossi Turunen
Hannele Turunen
Mikko Turunen
Jason Turuwhenua
Michael Turvey
Carolyn Turvey
Teresa Tusie-Luna
Gabor Tusnady
Nicholas Tustison
George Tuszynski
Yusuf Tutar
Geetu Tuteja
James Tutor
Mike Tuttle
Antonino Tuttolomondo
Juha Tuukkanen
Jetro Tuulari
Michael Tuvim
Beyhan Tuysuz
Pernille Tveden-Nyborg
Gard Frodahl Tveitevåg Svingen
Sean Tweedy
Jean Twenge
Robert Tweyongyere
Gilad Twig
Michael Twigg
Jimmy Twin
Martin Twiste
Claire Twose
Robert Twycross
Peter Tyack
Jaya Tyagi
Pradeep Tyagi
Rahul Tyagi
Mudit Tyagi
Wricha Tyagi
Nikhil Tyagi
Alexander Tyakht
Joshua Tybur
Benjamin Tycko
Coralee Tye
Jens Tyedmers
Ronald Tykoski
Robert Tykot
Brett Tyler
Seth Tyler
Kevin Tyler
Damian Tyler
Rich Tyler
Thorkild Tylleskär
Przemko Tylzanowski
Elizabeth Tymczyszyn
Peter Tymms
Joel Tyndall
Julian Tyne
Angela Tyner
Jeffrey Tyner
William Tyor
Jean-Robert Tyran
Pippa Tyrrell
Sarah Tyson
Alison Tyson-Capper
Eric Tytell
Henna Tyynismaa
Taina Tyystjärvi
Alexander Tzabazis
Charidimos Tzagarakis

Antonios Tzamaloukas
Emmanuel Tzanakakis
Georgios Tzanakakis
Nikolaos Tzanakis
Evangelos Tzanatos
Ioannis Tzanetakis
Ifigeneia Tzannou
Socrates Tzartos
Shean-Shong Tzean
Jung-Ying Tzeng
Shun-Fen Tzeng
Yu-Chieh Tzeng
Maria Tzetis
Oren Tzfadia
Anastassios Tzingounis
Athina Tzinia
Konstantinos Tziomalos
Athanasios Tzioufas
Ioanna Tzoulaki
Charalampos Tzoulis
Argyrios Tzouvelekis
Barbara Tzschentke
Tristan Tzschichholz
Hsieh Tzung-Bao
