# Supplementary material for: PLOS ONE 2015 Reviewer Thank You
Source: PLoS One. 2016 Feb 23;11(2):e0150341. doi: 10.1371/journal.pone.0150341 (PMC4764340; doi:10.1371/journal.pone.0150341)
Supplement: S5 Reviewer List — (PDF) [file pone.0150341.s005.pdf]

PLOS ONE would like to thank all those who reviewed on behalf of the journal in 2015:

Dirk Ubbink
Paloma Ubeda
Carles Ubeda
Peter Ubel
Olga Ucar
Mitsuhiro Uchiba
Naoya Uchida
Yukiko Uchida
Shusaku Uchida
Yoshikazu Uchida
Shunya Uchida
Motoharu Uchida
Mitsuo Uchida
Kimiko Uchii
Shigehiko Uchino
Ken Uchino
Koji Uchiyama
Manoel Uchôa
Ambros Uchtenhagen
Rifki Ucler
Joshua Udall
Iris Udasin
Bjarne Udd
Moin Uddin
Aftab Uddin
Riaz Uddin
Muhammad Jasim Uddin
Jashim Uddin
Jasim Uddin
Belinda Udeh
Klas Udekwu
Andrej Udelnow
Venkatachalam Udhayakumar
Nikolina Udikovic-Kolic
Rachanee Udomsangpetch
Heiichiro Udono
Zarir Udwadia
Victor Uebele
Beatrix Ueberheide
Natsuo Ueda
Kazumitsu Ueda
Koji Ueda
Tadashi Ueda
Takanori Ueda
Masashi Ueda
Mitsuharu Ueda
Hironori Uehara
Yuki Uehara
Thiers Uehara
Kazumasa Uehara
Mariko Uehara
Yasuyoshi Ueki
Shigeharu Ueki
Thor Ueland
Yoshinobu Uemoto
H. Uemoto
Kazuhiko Uemura
Hirohide Uenishi
Yoshiyuki Ueno
Naoto Ueno
Takashi Ueno
Massaro Ueti
Masayoshi Uezono
Akiyoshi Uezumi
Rafael Ufret-Vincenty
Antonio Ugalde
Edgardo Ugalde
Durdica Ugarkovic
Victor Ugaz
Giuseppe Ugazio
Kenneth Ugen
Piero Ugliengo
Sophie Ugolini
Carlos Ugrinowitsch
Barkan Ugurlu
Gabriele Uhl
Dieter Uhl
Anne-Catrin Uhlemann
Per Uhlen
Stefan Uhlig
Ondrej Uhlik
Yalda Uhls
Han Sup Uhm
Markus Uhrberg
Kathryn Uhrich
R. Glen Uhrig

Raivo Uibo
Remko Uijlenhoet
Jouni Uitto
Sya Ukena
Christian Ukena
Antti Ukkonen
Ukoha Ukoha
Kingsley Ukwaja
Kandasamy Ulaganathan
Amy Ulappa
Ilya Ulasov
Sebastian Ulbert
Karin Ulbrich
Elke Ulbricht
Szymon Ulenberg
Glen Ulett
Jay Ulfelder
Silvia Uliana
Tim Ulinski
Paola Ulivi
Kahraman Ülker
Miguel Ulla
Ghanim Ullah
Ekkehard Ullner
Mauricio Ulloa
Alfredo Ulloa-Aguirre
Rolando Ulloa-Gutierrez
Oliver Ullrich
Simone Ullrich
Evelyn Ullrich
Sarah Ullrich-French
Antigona Ulndreaj
Magda Ulrich
Helle Ulrich
Silvia Ulrich
Andreas Ulrich
Reiner Ulrich
Robert Ulrich
Daniela Ulrich
Katja Ulrich
Werner Ulrich
Natalie Ulrich
Bauer Ulrike
Sukru Ulusoy
Peter Ulvskov
Soo-Jong Um
Shanmugasundaram Uma
Román Umaña-Peña
Samuil Umansky
Brian Umberger
Claudia Umbreit
Hiroyuki Umegaki
Shigeki Umemura
Junji Umeno
Michihisa Umetani
Masakazu Umezawa
David Uminsky
Lars Umlauf
Ramesh Ummanni
Irusta Unai
Hamiyet Unal
Robert Unckless
Akif Undar
Anetta Undas
Gottfried Unden
Darrell Underhill
Kristen Underhill
Fiona Underwood
Martin Underwood
Tony Underwood
Eivind Undheim
Ashiwel Undieh
Eduardo Undurraga
Andrea Ungar
Daniel Ungar
Elizabeth Unger
Tamar Unger
Annemarie Unger
Rodolfo Ungerfeld
Emilio Ungerfeld
Tamas Ungi
Zoltan Ungvari
Jim Uniacke
Leanne Unicomb
Peter Unmack
Thomas Unnasch
Suraj Unniappan
Ashwin Unnikrishnan
Tatsuya Unno
Zolt Unoka
Jon Unosson
Nicolas Unsain
Christine Unson
Costin Untaroiu
Leonie Unterholzner
Joseph Unthank

Sathya Unudurthi
Derya Unutmaz
Frederick Unverzagt
Juanjo Unzilla
Natalie Uomini
Rohit Upadhyay
Arun Upadhyay
Piyush Upadhyay
Swapna Upadhyay
Chandan Upadhyay
Paul Upchurch
Gilbert Upchurch
John Updegraff
Jeffrey Upperman
Susan Uprichard
Carole Upshur
Takao Urabe
Lianne Urada
Fumihiko Urano
Fikriye Uras
Karen Uray
Constantin Urban
Carl Urban
Josef Urban
Jeffry Urban
Dan Urban
Daniel Urban
Beata Urban
Vilma Urbancic
Margrit Urbanek
Petr Urbanek
Jacek Urbanek
Sandra Urbanelli
Malvina Urbani
Michael Urbaniak
Javier Urbano
Amparo Urbano
Breeanna Urbanowicz
Rolf Urbanus
Diemo Urbig
Julio Urbina
Maria Elena Urcelay Garcia
Philip Uren
Aykut Üren
Pablo Ureña-Torres
Tamsyn Uren-Webster
Iker Uriarte
Phillip Uribe
Jean-Louis Uribelarrea
Santiago Uribe-Lewis
Tim Urich
Vicente Urios
Vlada Urlacher
Oleg Urminsky
Gerald Urquhart
Julio Urrets-Zavalía
Nikhil Urs
Robert Ursano
Sylvain Ursenbacher
Matilde Valeria Ursini
Cinzia Lucia Ursini
Raquel Urtasun
Peter Urwin
Clara Urzí
Eugen Urzica
Muhammad Usama Khanzada
Masahide Usami
Peter Usatyuk
Karen Usdin
Jakob Usemann
Valentina Usenik
Crystal Usenko
Konstantin Usevich
Irina Ushach
Edward Usherwood
Takahiro Ushida
Toshikazu Ushijima
Takashi Ushimaru
Masuko Ushio-Fukai
Siegfried Ussar
David Ussery
Joseph Usset
Julnar Usta
Vera Usuelli
Yoshihiko Usui
Torsten Utescher
Rene Utianski
Igor Utochkin
Akira Utsumi
Antonio Uttaro
Katarina Uttervall
Ingo Uttner
Paolo Uva
Vladimir Uversky
Brigitte Uwimana
Korkut Uygun

Georges Uzan
Svetlana Uzbekova
Gunes Uzer
Jude Uzonna
Takashi Uzu
Mehmet Uzumcu
Sami Väänänen
Veli-Matti Väänänen
Anusha Vable
Astrid Vabret
Michele Vacca
A. Vacca
Alberto Vaccheri
Matteo Vacchi
Caterina Vacchi-Suzzi
Vidula Vachharajani
Csaba Vadadi-Fülöp
Gianluca Vadala
Susan Vadaparampil
Sumeet Vadera
Jaydutt Vadgama
Miguel Vadillo
Ratna Vadlamudi
Krishna Vadrevu
Viveka Vadyvaloo
Maarten Vaessen
Harald Vaessin
Michael Vaezi
Marina Vafeiadi
Alejandro Vagelli
Panagiotis Vagenas
Federico Vaggi
Augusto Vaglio
Paola Vagnarelli
Bahareh Vahabi
Nasim Vahabi
Maryam Vahdaninia
Shahabeddin Vahdat
Ali Vahdati
Karim Vahed
Antti Vaheri
Ali Vahidnia
Wilfried Vahjen
Anders Vahlne
Varpu Vahtera
Stefania Vai
Bijay Vaidya
Shantashri Vaidya
Bhuvaneshwar Vaidya
Ravi Vaidyanathan
Srinivas Vaidyanthan
David Vailancourt
Cathy Vaillancourt
Andrew Vaillant
Nathalie Vaillant-Gaveau
Daniel Vaiman
Milena Vainieri
Seppo Vainio
Maria Helena Vaisbich
Iosif Vaisman
Anne-Charlotte Vaissière
Rathna Vaithilingam
Claire Vajdic
Peter Vajkoczy
Efsevia Vakiani
Keyvan Vakili
Ady Vaknin
Ilya Vakser
Andrew Vakulin
Pierre Val
Aurore Val
Samuel Valable
Joseph Valadez
Alan Valaperti
Stephanie Valberg
Gustavo Valbuena
Ruben Valbuena
Mihai Valcu
Adriana Valcu
Joaquin Valderrama
Alessandro Valderrama
Luis Valdes
Ja Valdes
Alicia Valdes
Jesús Valdés
Sergio Valdés-Ferrer
Gregorio Valdez
Rodolfo A Valdez
Alireza Valdiani
Gudrun Valdimarsdottir
Hector H. Valdivia
Hugo Valdivia
Cândida G. Vale
Giampiero Valè
Claudia Valeggia
Hein Valenberg

Catalina Valencia
Herbert Valensise
Peter Valent
Thomas Valente
Marco Valente
Rubia Valente
Giovanna Valenti
Luca Valenti
Helene Valentin
Eulogio Valentin
Stephen Valentine
Elia Valentini
Alessio Valentini
Luzia Valentini
Rita Valentino
Gaetano Valenza
Michael Valenzuela
C. Fernando Valenzuela
Olivia Valenzuela
Antonia Valenzuela
Sofia Valenzuela
Fabiana Valera
Francisco Valera
Massimiliano Valeriani
Clara Valero
Francisco Valero-Cuevas
Luca Valgimigli
Taufik Valiante
Ranjini Valiathan
Francisco J Valiente-Soriano
Clarissa Valim
Liora Valinsky
Mohammad Valipour
Priit Väljamäe
Sofie Valk
Sándor Valkai
O. Valkenburg
Jon Valla
Manuel Valladares-Ayerbes
Jose Vallarino
Francesc Vallderiola
Denise Valle
Jaione Valle
Mario Valle
Mario Vallejo
Edgar Vallejo
C. Eduardo Vallejos
Eric Vallender
Angelo Valleriani
Alain-Jacques Valleron
Soraya Valles
Antonio Vallesi
Francois Vallette
Matthew Valley
Marta Vallino
Michael Vallis
Daniela Vallone
Giorgio Vallortigara
Josep Valls-Sole
Joan-Carles Vallvé
Josep Vallverdú-Poch
Anna Vallverdu-Queralt
Lucia Valmaggia
Damien Valour
Victoriano Valpuesta
Nakul Valsangkar
Matias Valsecchi
Matteo Valsecchi
Sergio Valsecchi
Deltcho Valtchanov
Nadejda Valtcheva
Marco Valtorta
Nicole Valtorta
Anna Valujskikh
Olga Valverde
Ángela Valverde
Sergi Valverde
Diana Valverde
Federico Valverde
Angel Valverde
Nelson Valvidia
György Vámosi
Markus van Ackeren
Robbie van Aert
Jason Van Allen
Jacques van Alphen
Lieke van Alphen
Leentje van Alphen
Mike Van Amburgh
Pierre van Antwerpen
Pleun Van Arensbergen
Thea van Asselt
Marcel van Assen
Susanne Van Asten
Nienke van Atteveldt
Geraldine van Aubel

Lisa van Baarsen
Françoise Van Bambeke
Eduard Van Beeck
Hans van Beek
Frank van Bel
Marco van Belkum
Simon van Bellen
Chris van Beneded
Jeroen van Bergen
Jeroen van Bergenhenegouwen
Theo van Berkel
Jop van Berlo
Steven van Beurden
Heleen van Beusekom
Nico van Beveren
Jolanda van Bilsen
Erin Van Blarigan
Marka van Blitterswijk
Mieke Van Bockstal
Inge Van Bogaert
Claudia van Borkulo
Wim Van Bortel
Cornelis van Breemen
Gerard Van Breukelen
Ariena van Bruggen
Bram van Bunnik
Jaap van Buul
Ben Van Calster
Eve van Cauter
Jelle Van Cauwenberg
Fabien Van Coppenolle
Reinout van Crevel
Hendrik van Dalen
Nicole van Dam
Daphne van de Bongardt
Martijn van de Bunt
Mark Van de Casteele
Maarten van de Guchte
Nicole Van de Kar
Madelon van de Kerk
Gijs van de Kuilen
Rick van de Langenberg
Ingrid van de Leemput
Sebastian van de Linde
Pierre-Francois Van de Moortele
Philippe Van De Perre
Frank van de Veerdonk
Cornelis van de Velde
Vincent van de ven
David van de Vijver
Fons Van De Vijver
Dedmer Van de Waal
Gerlinde Van de Walle
Thomas Van de Water
Cornelia van de Weg
Rogier van de Wetering
Johannes van Delden
Joost van den Aardweg
Jan Van Den Abbeele
Guido Van den Ackerveken
Johannes N. van den Anker
Henry van den Bedem
Marius van den Beek
L Van den bemt
Timo van den Berg
Albert van den Berg
Bert Van Den Berg
Bernard van den Berg
Jacob van den Berg
Janneke van den Bergen
Peter van den Besselaar
Ellen van den Bogaard
Bartholomeus Van den Bogert
Dick van den Boomen
Jacob van den Born
Ludo Van Den Bosch
Jan van den Brand
Sybille van den Brule
Hendrik van den Bussche
Maarten van den Buuse
Stephen Van Den Eeden
Gert Van den Eynde
Martijn van den Heuvel
Maurice van den Hoff
Jose van den huevel
Peter van den Hurk
Josien van den Noort
Jan Van den Stock
Ignatia Van den Veyver
Cees van den Wijngaard
Tim Van den Wyngaert
Axel Van Denwalle
Bart Van der Bruggen
Chloe van der Burg
Bram van der Eerden
Elise van der Elst

Arie van der Ende
Mark van der Gaag
Kornelis van der Geest
Johanneke Van der Harst
Yuri van der Heijden YF
Lieke van der Helm
Annette van der Helm-van Mil
Julie Van der Hoop
Geertje van der Horst
Mathieu Van der Jagt
Fiona van der Klis
Anja van der Kolk
Michael van der Kooij
Mia van der Kop
Peter van der Kraan
Laura van der Laan
Lijckle van der Laan
Andries Van der Leij
Peter Van Der Ley
Mark van der Linden
Sander van der Linden
Aad van der Lugt
Nicoline van der maas
Marieke van der Maaten-Theunissen
Pieter F. van der Meer
Jan van der Meer
Marcel T. J. van der Meer
Frans van der Meer
Gro Van der Meeren
Henny C. van der Mei
Arie van der Meijden
Paola van der Meijden
Margaretha Van der Merwe
Saskia Van der Oord
Wim van der Poel
David van der Poorten
Daniel van der Post
Tineke van der Pouw Kraan
Han van der Rhee
Marianne van der Sande
Job van der Schalk
Yvonne van der Schouw
Frans van der Sluis
Tetje van der Sluis
Jozef (Sjef) van der Steen
Nathan van der stoep
Jacques Van der Straeten
Ariane van der Straten
Rosalie van der Vaart
Elske van der Vaart
Monique van der Veen
Daan Van der Veen
Eric van der Veer
Marijn van der Velde
Jos van der Velden
Peter G. van der Velden
Adrianus van der Velden
Leo van der Ven
Johan van der Vlag
Albert van der Vliet
René van der Vlugt
Monique van der Voet
Emiel van der Vorst
Annemieke van der Wal
Walter van der Weegen
Patrick van der Wel
Tjip van der Werf
Ysbrand van der Werf
Christien van der woude
Johannes van der Wouden
Julie van der Zee
Mikael van Deurs
Ioana van Deurzen
Allen Van Deynze
Eric van Diessen
Mike van Diest
Patrick Van Dijck
Marie van Dijk
Koene Van Dijk
Jan Maarten van Dijl
Karine Van Doninck
Paul van Donkelaar
H. Rogier van Doorn
Donald Van Doornik
Neeltje van Doremalen
Ian van Driel
Joram van Driel
Karel van Duijvenboden
Adri Van Duin
Luca van Duren
Hans Van Dyck
Linda van Dyk
Gerhild van Echten-Deckert
Fred van Eeuwijk
Michiel van Eijk
James Van Etten

Hanneke van Ewijk
Eric van Furth
Hans van Gasteren
Ingeborg van Geijlswijk
Teun van Gelder
Peter van Gelderen
Lisette van Gemert
Arend Van Gemmert
Dik van Gent
Marjolein van Gent
Jay Van Gerpen
Marit van Gils
Frederik van Ginkel
Nadja van Ginneken
Coen van Gool
Harry van Goor
Eric van Gorp
Martijn van Griensven
Guido Van Hal
Sander van Haperen
Andrew Van Hateren
Karen van Hedel
Stephen Van Hedger
Waander van Heerde
Vincent Van Hees
Jacques Van Helden
Mieke Van Hemelrijck
Eldert J. van Henten
Jo Van Herwegen
Caroline van Heugten
Marieke Van Heugten
Walter van heuven
Brian Van Hezewijk
Matthias Boris Van Hiel
Richard van Hillegersberg
Monique van Hoek
Ambro van Hoof
Madelon Van Hooff
Matthew Van Hook
John Van Horn
Amanda Van Horne
Rob Van Houdt
Rene van Hout
Kyle Van Houtan
Elijah Van Houten
Marc Van Hulle
Paul Van Hummelen
Wim van Ieperen
Marinus Van IJzendoorn
Christina Van Itallie
Heyo Van Iten
Martin Van Ittersum
Cornelia van Jaarsveld
Sanne van Kampen
Julia Van Kessel
Freya van Kesteren
Joshua van Kleef
Esther van Kleef
Yvette van Kooyk
Joost van Kordelaar
Linda van Laake
Colette Van Laar
Kristel Van Laethem
Jacques van Lankveld
Geert van Leenders
Frank van Leeuwen
Thomas Van Leeuwen
Thed van Leeuwen
Florian Van Leeuwen
Abram van Leeuwen
Iman van Lelyveld
Erik J. van Lieshout
Carine Van Lint
Marcela Van Loo
Geert van Loo
Andy Van Loocke
Joop van Loon
Luc van Loon
Mark van Loosdrecht
Jorg Van Loosdregt
Judith van Luijk
Menno van Lummel
Carine Van malderen
Wouter van Marken Lichtenbelt
Harm Van Marwijk
Kimberly Van Meter
Tim Van Mieghem
Inge Van Molle
R.J. Joost van Neerven
Jaap Van Netten
Martine van Nierop
Geerten van Nieuw Amerongen
Cornelis Van Noorden
Saskya van Nouhuys
Michael Van Nuland
Nicolai van Oers

M van Oijen
Peter van Ooijen
Joep van Oorschot
Pepijn A.J. van Oort
Patricija van Oosten-Hawle
Sandra van Oostrom
Thijs van osch
Xaveer Van Ostade
Mannis van Oven
Willem Van Panhuis
Steven Van Passel
Melissa van Pel
Ans van Pelt
Wilfrid van Pelt
R. Van Pelt
Vincent van Pesch
Filip Van Petegem
Cyma Van Petten
Brett van Poorten
Henriette Van Praag
Jan-Willem van Prooijen
Gijs van Puijvelde
Ingrid Van Putten
Daniel van Raalte
Jeremy Van Raamsdonk
Anthony van Raan
Don van Ravenzwaaij
Nicolien van Ravesteyn
Franka van Reekum
Kees Van Reenen
Jacco Van Rheenen
Ildiko Van Rhijn
Bas van Rhijn
Johan van Rhijn
Annelies Van Rie
Debbie van Riel
Piet van Rijn
Jaap van Rijn
Leonie van Rijt
Willeke van Roon-Mom
Jacques van Rooy
Floor van Rosse
Marion van Rossum
Rufin Van Rullen
Dimitri Van Ryckeghem
Emer Van Ryswyk
Hjalmar van Santvoort
Dirkjan van Schaardenburg
Willem van Schaik
Erin van Schaik
Christian van Scheve
Natasja van Schoor
Erik Van Sebille
Alper van Sijl
Douwe van Sinderen
Esther van Sluijs
Jeroen van Smeden
Knoek Van Soest
Arthur van Soest
Anneloes van Staa
Henk van Steenbergen
Henk van Stel
Jan Van Strien
Bruno van Swinderen
Celine Van Themsche
Christopher van Tilburg
Brian Van Tine
Sandra van Tongeren
Hans C van Trijp
A.S. van Trotsenburg
Marleen Van Troys
Janet van Uem
Frans Van Valen
Blaire Van Valkenburgh
Irene G.M. van Valkengoed
Franciscus van Veen
Paul Van Veldhoven
Robin van Velzen
Marcel Van Verk
Pieter Van Vlierberghe
Sandra van Vliet
Jasper van Vliet
Liesbeth van Vliet
Bart van Vlijmen
Dave Van Voorhees
Brad Van Voorhis
David Van Vranken
Saskia van Vugt
Mark van Vugt
Dean Van Vugt
Marieke van Vugt
Nicholas Van Wagoner
Willem van Wamel
Daniel van Wamelen
Aleid van Wassenaer
Sam Van Wassenbergh

Isabel van Waveren
Rene van Weeren
Maarten van Wijhe
Klaas van Wijk
Erwin van Wijk
Els Van Wijngaarden
Guido van Wingen
Robert van Woesik
Nelleke van Wouwe
Hannes van Wyk
Jean Van Wyk
Arthur van Zanten
Menno van Zelm
Catherine van Zelst
Wieske van Zoest
Martijn van Zomeren
Mirjam van Zuiden
Arjan van Zuilen
Brigitte van Zundert
Jelle van Zweden
Gert van Zyl
Riann van Zyl
Peter Vanable
Jean-Marc Vanacker
Nicola Vanacore
Maddi Vanaja
Jairam Vanamala
Éva Vanamee
Murugendra Vanarotti
Jan Vanaverbeke
Glenn Vanblaricom
Collin Vanburen
David Vance
Eric Vance
Ivana Vancurova
Milène Vandal
Mark Vandam
Peter Vandamme
Christine Vande Velde
Robert Jan Vandebriel
Justin Vandehey
Corneel Vandelanotte
Andrew Vandemark
George Vandemark
An Vanden Broeck
Jamie Vandenberg
Thierry Vandenberg
Robert Vandenberg
John Vandenberg
David Vandenbergh
Rik Vandenberghe
Joshua Vandenbrink
Koen Vandenbroeck
Tineke Vandenbroucke
Roosmarijn Vandenbroucke
Christopher Vandenbussche
Eric Vanden-Eijnden
Élodie Vandenhaute
Hadewijch Vandenheede
Philippe Vandenkoornhuyse
Patrick Vandeputte
Kathy Vander Werff
Laura Vanderdrift
Dieter Vanderelst
Barbara Vanderhyden
Paul Vanderlaan
Elien Vandermarliere
John Vandermeer
Johan Vandermeer
Louk Vanderschuren
Anthony Vandervoort
Mark Vanderwel
Eric Vanderwerf
Karen Vanderwolf
Karen Vandevelde
Pamela Vandevord
Gilles Vandewalle
Antonius Vandongen
Jennifer Vandooren
Bertrand Vandoorne
Ryan Vandrey
Moriel Vandsburger
Kate Vandyke
Evelien Vanekert
Renata Vanelli
Lowie Vanfleteren
Jagadish Vangipurapu
Lynn Vanhaecke
Guido Vanhal
Petr Vanhara
Bartel Vanholme
Filiep Vanhonacker
Boudewijn Vanhoudenhove
Daan Vanhove
Janaki Vani
Lavanya Vanjari

Hugo Vankelecom
Radomira Vankova
Dana Vanlandingham
Sarah Vanlieferinghen
Jill Vanmassenhove
Steffen Vanneste
Tazio Vanni
Ester Vanni
Ayyasamy Vanniarajan
Edouard Vannier
Stephane Vannitsem
Tommaso Vannocci
Alessandro Vannucchi
Maria Giuliana Vannucchi
Jeroen Vanoirbeek
Maria Antonietta Vanoni
Jennifer Vanos
Paul Vanraden
Michael Vansaun
Gregory Vanstavern
Vincent Vantrepotte
Jan Vanzundert
Olli Vapalahti
Javier Vaquero
Kulandaiappan Varadaraj
Sudhahar Varadarajan
Navin Varadarajan
Pritish Varadwaj
Jasmina Varagic
Vijaya Varanasi
Gabriele Varani
Zeno Varanini
Chiara Varazzani
Greta Varchi
Briony Varda
John Vardakis
Michael Vardaro
G. Vardar-Schara
Ricardo Vardasca
Assaf Vardi
Yoav Vardy
Cristian Varela
Sara Varela
Ruben Varela
Miguel A. Varela
Luis Miguel Varela
Ruben Varela-Calviño
Isabel Varela-Nieto
Armando Varela-Ramirez
Maria Varela-Silva
Guillaume Vares
Cristina Varese
Zsuzsanna Varga
Franz Varga
Gabor Varga
Zoltan Varga
Pablo Vargas
Sergio Vargas
Jose Alberto Vargas
Ashley Vargas
Gilberto Vargas-Alarcón
Mariana Vargas-Caballero
Sergio Vargas-Prada
Cherian Varghese
Arun Varghese
Jishy Varghese
Attilio Vargiu
Elisabeth Vargo
Asokan Mulayath Variyath
Erika Varkonyi-Gasic
Manuel Varlet
Jadranka Varljen
Rajat Varma
Amit Varma
Sameer Varma
Manthena Varma
Muralidhar Varma
Dileep Varma
Arul Mozhy Varman
Katarina Varnäs
Alexandre Varnek
Kurt Varner
Dickson Varner
Peeter Värnik
Atakan Varol
Christine Varon
Claudio Varotto
Serena Varotto
Antonio Varriale
David Varricchio
Lilian Varricchio
Annabelle Varrot
Arvind Varsani
Lav Varshney
Grish Varshney
Jean-Pierre Vartanian

Timothy Vartanian
Oshin Vartanian
Martina Vašáková
Senthil Vasan
Sandhya Vasan
Tuula Vasankari
Farhad Fakhrudin Vasanwala
Vasyl Vasco
José Ronnie Vasconcelos
Tiago Vasconcelos
Joanna Sousa Vasconcelos Franco
Jaromir Vasicek
Michael Vasil
Paraskevas Vasilakopoulos
Athanasios Vasilakos
Dragos Vasilescu
Vasilis Vasiliou
Athanasios Vasilopoulos
Elisavet Vasilopoulou
Alexander Vaskevich
Bruno Vaslin
Ana Carolina Vasques
Karen Vasquez
Yanira Vasquez
Paula Vasquez
Alex Vasquez
Clemens Vass
Imre Vass
Cristina Vassalle
Anne Vassalli
Massimo Vassalli
R Vassallo
Paolo Vassallo
Georges Vassaux
Eliana Vassena
R. Vassena
Paule Vasseur
Nikon Vassilakos
Andon Vassilev
Zdravko Vassilev
Tchavdar Vassilev
Alexander Vassilevski
Dimitrios Vassilopoulos
Michele Vasso
Daniel Västfjäll
Srividya Vasu
Akshya Vasudev
Lavanya Vasudevan
T.S. Vasulu
Lana Vasung
Mihaly Vaszilko
Dimitrios Vatakis
Olena Vatamaniuk
Joachim Vater
Eric Vatikiotis-Bateson
Abhay Vats
Pankaj Vats
Vatsalya Vatsalya
Matteo Vatta
Julien Vaubourgeix
Elvire Vaucher
Marc Vaudel
Gaetano Vaudo
Jefferson Vaughan
Gilberto Vaughan
Ted Vaughan
Roxanne Vaughan
Douglas Vaughan
Adam Vaughan
Allison Vaughn
Thomas Vaughn
Charlotte Vaughn
John Vaughn
Jason Vaught
Sophie Vaulont
Rebecca Vautour
David Vauzour
Alain Vavasseur
Vasileios Vavourakis
Tanya Vavouri
Jiri Vavra
Demetrios Vavvas
Reaz Vawda
Frederic Vaysse
Lara Vaz
Pedro Vaz
Marcelo Vaz
Kristyna Vazacova
Almira Vazdarjanova
Simine Vazire
Nosratola Vaziri
Santi Vazquez
Federico Vazquez
Mariano Vazquez
Fernando Vazquez
Guillermo Vazquez

Paola Vázquez-Cárdenas
Manuel Vazquez-Carrera
Rafael Vazquez-Duhalt
Alejandra Vazquez-Lobo
Rafael Vazquez-Manrique
Gerardo Vázquez-Marrufo
Roberto Vazquez-Padron
Elizabeth Veal
David Veale
Joanna Vearey
Francisco Veas
Elizabeth Veasey
Sarah Veatch
Olivia Veatch
Ashley Veatch
Christian Veauthier
Ronald Veazey
Kari Veblen
Carmine Vecchione
Elena Vecino
Cecilia Vecoli
Vinata Vedam-Mai
Vasanth Vedantham
Srinivasan Vedantham
Oscar Vedder
Maria Cristina Vedovati
Brendan Veeneman
Jan-Willem Veening
Vijay Veer
Ilya Veer
Arumugam Veera Ravi
Sudhakar Veeranki
Harini Veeraraghavan
Krishna Kumar Veeravalli
Janne Veerbeek
Everardo Vega
Tomás Vega
Aurelie Vega
Almudena Vega
Carlos Vega-Melendez
Ignacio Vega-Naredo
Maria Vega-Rodriguez
Elisabetta Vegeto
Manuela Veglia
Franco Veglio
Stefan Vegter
Rüdiger Veh
Sigrun Vehling
Maria Vehreschild
Silvio Veiga
Almudena Veiga-Lopez
Carla Veigas
Jennifer Veilleux
Jutta Veits
Eric Vejerano
Xavier Vekemans
Johan Vekemans
Alberto Vela
S. Sendhil Velan
Michael Velarde
Carlos Velasco
Ilais Velasquez
Adriana Velásquez Vacca
Thirumalaisamy Velavan
Anandh Velayutham
Paúl Velazco
Luis Velazquez
N. Velázquez-Guadarrama
Julien Velcin
Susan Veldheer
Johannes Veldhuis
James Veldhuis
Edwin Veldhuizen
Maria Veldhuizen
Coosje Veldkamp
Adam Veleba
Emir Veledar
Juan Carlos Velez
Maria Velez
María Alejandra Velez
Maria Vélez
Aaron I. Velez-Ramirez
Philippe Velge
Galina Velikova
Anvar Velji
Tibor Vellai
Elangovan Vellaichamy
Sukumar Vellakkal
Sandra Velleman
Edo Vellenga
Alfredo Vellido
Licio Velloso
Alisa Velonis
Javier Veloso
Samuel Veloz
Kiran Kumar Velpula

Marcella Velten
Eva Velthorst
Robert Veltri
Paris Veltsos
Vijayakumar Velu
Ramesh Velupillaimani
Kalyana Veluvolu
Pepijn Vemer
Lakshminarayana Vemireddy
Rao Vemuri
Julian Venables
Aida Venado
Miguel Vences
William Vencill
Tomas Venckunas
Márton Venczel
Mikkel Vendelbo
Gianluigi Vendemiale
Silvia Vendetti
Michael Vendetti
Chris Venditti
Jennifer Venditti
Francesco Vendrame
Xavier Vendrell
Mónica Venegas Calerón
Jose Luis Venero
David Venet
Jonathan Venezia
Valentina Vengeliene
Viktoria Venglovecz
Avner Vengosh
Lisa Venier
Domenica Veniero
Sreedhar Venkannagari
Manjeri Venkatachalam
Kartik Venkatachalam
Aiveliagaram Venkatakrishnan
Ramamurthy Venkataramanan
Keerthi Venkataramanan
Atheendar Venkataramani
Radha Venkatesan
Subramanian Venkatesan
Narayanan Venkatesan
Umesh Venkatesan
Arjun Venkatesh
Pradeep Venkatesh
Ananthapur Venkateshwari
Pannuru Venkatesu
Prasanna Venkatraman
E. Seshan Venkatraman
Vishwanath Venketaraman
Rudi Vennekens
Birgitte Vennervald
Stephanie Venn-Watson
Conchita Vens
Stephanus Venter
Estelle Venter
Rodney Venterea
Corey Ventetuolo
Maximo Vento
Renza Vento
Iván Ventoso
Natascia Ventura
Marco Ventura
Francesc Ventura
Tomer Ventura
Walter Ventura
Paulo Ventura
Fatima Ventura
Karen Ventura
Beth Ventura
Teresa Ventura
Alexander Ventura
Alison Ventura
Venessa Venturi
Elisabetta Venturini
Rajanbabu Venugopal
P. Dilip Venugopal
Vayalam Venugopalan
Aldo Venuti
Madelaine Venzon
Carlos Vera
Jaime Vera
Mario Vera
Lucio Vera-Cabrera
Marcos Vera-Hernández
Alexey Veraksa
Jennifer Verani
Jorge Vera-Otarola
Patricia Veras
Joren Verbeke
Tony Verboom
Scott Verbridge
Rineke Verbrugge
Heroen Verbruggen
Henri Verbrugh

Peter Verburg
Klara Verbyla
Ari Verbyla
Giuseppe Vercellotti
José Luis Vercher Conejero
Bruce Verchere
Laurent Vercueil
Pasquale Verde
Zoraida Verde
Michael Verde
Paolo Verdecchia
Gregory Verdeil
Antonio Verdejo-García
Konstantinos Verdelis
Claudia Verderio
Paolo Verderio
Thierry Verdier
Julien Verdier
Hannah Verdin
Timothy J. Verdon
Rinus Verdonschot
Elena Verdu
Jose Verdu
Ramiro Verdun
Kim Verdurmen
Marilena Vered
Beatrix Vereijken
Alexey Vereninov
Gabor Veres
Daniel Veres
Stavros Veresoglou
Laura Verga
Chiara Vergani
Diego Vergani
Patri Vergara
Giuseppe Vergaro
Jasmine Vergauwe
Pierre Verger
Guy Vergères
Adriana Vergés
Federica Verginelli
Alain Vergnenegre
Carlijn Vergouw
Mervyn Vergouwen
Stephane Verguet
Annette Vergunst
Joost Verhaagen
Marianne Verhaar
Johan Verhaeghe
Paul Verhaeghen
Lilly M Verhagen
Marcel Verheij
Rebekka Verheij
Kristen Verhey
H Verheyden
Christine Verhille
Gregory Verhoest
Marieke Verhoeven
Els Verhoeyen
Simon Verhulst
Diogo Verissimo
Diogo Veríssimo
António Veríssimo
Sergio Verjovksi-Almeida
Erwin Verkade
Gennady Verkhivker
Alexander Verkhovsky
Alexei Verkhratsky
Alan Verkman
Peter Verkoeijen
Giuseppe Verlato
Heleen Verlinden
Stefan Verlohren
Maite Verloigne
Subhash Verma
Rama Verma
Saguna Verma
Suresh Verma
Praveen Verma
Sunil Verma
Rahul Verma
Ruchi Verma
Mayank Verma
Ashutosh Verma
Akash Verma
Mihaela Verman
Harriet Vermeer
Pieter Vermeesch
Johannes Vermehren
Séverine Vermeire
Eric Vermetten
Louis Vermeulen
Cedric Vermeulen
Sonja Joy Vermeulen
Ivar Vermeulen
David Vermijlen

Sten Vermund
Dolinsky Vern
Carla Verna
Michael Verneris
Antoine Vernet
Santiago Vernia
Gianluca Vernillo
Andy Vernon
Suzanne Vernon
David Vernon
Carlijn Vernooij
Jean-Paul Vernoux
Edelyn Verona
Nicola Veronese
Giovanni Veronesi
Areti Angeliki Veroniki
Pierfrancesco Veroux
Bas Verplanken
Alexander Verpoorte
Guy Verreault
Franck Verrecchia
Frank Verreck
Julius Verrel
Tiziano Verri
Waldiceu Verri Jr
Sébastien Verrier
Nikki Verrills
Francesco Versace
Chris Verschoor
Jan Verschuuren
Alexander Vershinin
Paul Verslues
Lilly Verso
Karin Verspoor
Eric Verspoor
Nicolas Verstaevel
Brett Verstak
Veerle Versteirt
Esther Verstraete
Patrik Verstreken
Kevin Verstrepen
Timothy Verstynen
Maria Vertzoni
Bram Vervliet
Mg Vervloet
Jacques Vervoort
Renee Verway
Mihalis Verykokakis
Stephen Verzi
Roberto Verzicco
Omar Vesga
Paul Vespa
Umberto Vespasiani-Gentilucci
Cordula Vesper
Stephen Vesper
Robert Vessella
Gisle Vestergaard
Dietmar Vestweber
Szilvia Veszelka
Davide Vetrano
Monica Vetter
Ingrid Vetter
Céline Vetter
Stefan Vetter
Eric W. Vetter
Riyas Vettukattil
Ramesh Vetukuri
Paul Veugelers
Bernard Veyret
Frederic Veyrunes
Chad Vezina
Nicolas Veziris
Julien Vezoli
Dario Vezzani
Annamaria Vezzani
Antonella Vezzani
Giuseppe Vezzoli
Luigi Vezzulli
Silvia Vezzulli
Rao Vh
Laura Via
Marc Via
Flavie Vial
Laurence Vial
Silvere Vialet-Chabrand
Jean-Francois Viallard
Agathe Vialle
Vincent Vialou
Felix Viana
Duarte Viana
Maria Teresa Viana
Isabelle Viana
Milena Viana
Lucas Viana
Rosana Vianello
Monique Vianey-Liaud

Rosane Vianna-Jorge
Mariano Viapiano
Cristiano Viappiani
Diego Viasus
Victor Viau
Charles Viau-Quesnel
Francesca Viazzi
Eric Vibert
Cecile Viboud
Carmelo Vicario
Lyderson Viccini
Patricia Vicendo
Joaquin Vicente
Ana Carolina Vicente
Joana Vicente
Claudia Vicente
João Vicente
Maria Teofila Vicente-Herrero
Rebecca Vicente-Steijn
Renato Vicentini
Elisa Vicenzi
Ales Vicha
Kasey Vickers
Joan Vickers
Debi Vickers
William Vickery
Laurence Vico
Michele Vicovaro
Tamás Vicsek
Christina Victor
Benjamin Victor
Jennifer Victor
Mark Vida
Andras Vida
Carmen Vida Hinojosa
Silvia Vidal
Jorge Vidal
Dominique Vidal
Pierre-Paul Vidal
Marcos Vidal
Edison Vidal
Elena Vidal
Emmanuelle Vidal-Petiot
Manuel Vidal-Sanz
Jose Vidal-Taboada
Atanasio Vidane
Luiz Videla
John Videler
Vibeke Videm
Zoltan Vidnyanszky
Giulio Vidotto
Michele Vidotto
Rishma Vidyasagar
Dharmapuri Vidyasagar
Stephanie Vie
Wolfgang Viechtbauer
Giovanni Viegi
Alexandre Vieira
Leandro Vieira
Angelica Vieira
Gustavi Vieira
Joana Vieira
Elisabeth Meloni Vieira
Jose Vieira, Jr.
Victoria Vieira-Potter
Tania Viel
Danielle Vienneau
Elvina Viennet
Emilie Viennois
Jeff Vierstra
Bruno Viertel
Dumitru Vieru
Reinhold Vieth
Jeffrey Vietri
Rachel Vieux Vieux
Maria Viga-De Alva
Gianpiero Vigani
Ana Vigario
Davide Vigetti
Erlend Viggen
Maria Pia Viggiano
Laszlo Vigh
Marco Vighi
Benjamin Viglianti
Edward Vigmond
Nicolas Vignais
Rita Vignani
Julien Vignard
Thomas Vignaud
Ramachandran Vignesh
Rafael Vignoli
Philippe Vignon
Irene Vignon-Clementel
Marco Vignuzzi
Carlo Vigorito
Yves Vigouroux

Nathalie Viguerie
Catherine Viguié
Jorma Viikari
Leena Viiri
Shubha Vij
Dharmamony Vijai
Sandeep Vijan
Vikrant Vijay
Lakshmi Vijayakumar
V. Vijayakumar
Mathilakath Vijayan
Shibu Vijayan
Srinivasan Vijayaraghavan
Jyothi Vijayaraghavan
Dhanasekaran Vijaykrishna
Roshan Vijendravarma
Lodewijk Vijftigschild
Daniel Vijlbrief
Petter Vikman
Naval Vikram
Prashant Vikram
Kristina Viktorsson
Greisa Vila
Alejandro Vila
Jose Vila
Carles Vilà
Martin Vila-Aiub
Nuria Viladrich
Alberto Vilagrosa
Gemma Vilagut
Anna Vilanova
Enric Vilar
Eduardo Vilar Gomez
Carles Vilarino-Guell
Raquel Vilar-López
Jordi Vilaro
Juan J. Vilchez
Gustavo Vilchez
Catherine Vilchèze
Jenny Vilchis-Gil
Jose Vilela-Martin
Dan Vilenchik
Didier Vilette
Andrej Vilfan
Anna Vilgelm
Lorenzo Vilizzi
Chris Viljoen
Albertus Viljoen
Laura Villa
Erica Villa
Alessandro Villa
Paola Villa
Anna Villa
Jorge Villa Betancur
Jose Villalba
Mayte Villalba
Elena Villalba
Héctor Villalobos-Duno
Danilo Villalta
Fernando Villalta
Armando Villalta
Frederick Villamena
Sara Villani
Gianfrancesco Maria Villani
Elmer Villanueva
Roger Villanueva
Pia Villanueva
Jesús Villar
Enrique Villar
Luisa Villar
José Augusto Villar
Van Anthony Villar
Manuel Villaran
Marc-André Villard
Tracy Villareal
Dennis Villareal
Paolo Villari
Liza Villaruz
Sebastian Villasante
Laura Villa-Torres
Andrés Villaveces
Nathalie Villa-Vialaneix
Aline Villavicencio
Camila P Villavicencio
Yves Ville
Saul Villeda
Juan Camilo Villegas
Luis Eduardo Martinez Villegas
Angel Villegas-Fernandez
Daniel Villela
Corrado Villella
J. Villellas
Josep Villena
Daniel Villeneuve
Sara Villén-Pérez
Osvaldo Villet

Marie-Claire Villeval
Loic Villier
Peter Villiger
Martin Villiger
Francois Villinger
Carmen Villmann
Andreas Villunger
Daniele Vilone
Jean-Luc Vilotte
Hendrik Vilstrup
Karani Santhanakrishnan Vimaleswaran
Sornkanok Vimolmangkang
Andres Vina
Juan R. Viña
Catarina Vinagre
João Vinagre
Piergiuseppe Vinai
Jordi Vinas
Jose Luis Viñas
Arunachalam Vinayagam
A.C. Vinayaka
Frans Vinberg
Elizabeth Vincan
Christian Vincenot
Amanda Vincent
Amy Vincent
Andrea Vincent
Gregoire Vincent
Peter Vincent
John Vincent
Tonia Vincent
Grace Vincent
Francois Vincent
Stephen Vincent
Delphine Vincent
Maurice Vincent
Simone Vincenzi
Bruno Vincenzi
Marco Vinceti
Francesca Vinchi
Maria Cristina Vinci
Manlio Vinciguerra
Antonio Vinciguerra
Cecile Vincke
Sam Vine
Paolo Vineis
Jonathan Vinet
Martin Vingron
Algis Vingrys
Donald Vinh
Antony Vinh
Lucio Vinicius
Ana Viniegra
Cor Vink
Joy-Sarah Vink
Stefanie Vink
Mathieu Vinken
Christiaan Vinkers
Anna Vinkhuyzen
Lasse Vinner
K.K. Vinod
Carolina Viñoles
Marco Vinolo
Neomi Vin-Raviv
Jarmila Vinsova
Jakob Vinten-Johansen
Javier Viñuela
David Vinyard
Ronald Viola
Manuela Viola
Donata Violanti
John Violanti
Francesco Violi
Benoit Viollet
Cecile Viollet
Stephane Viollet
Aline Viott
Nicolas Viovy
Enrica Viparelli
Victoria Virador
Laszlo Virag
Jitka Virag
Gianni Virgili
Roberta Virgili
Alessandra Virgillito
Jasveer Virk
Aki Virkkula
Isabelle Virlogeux-Payant
Renu Virmani
Anders Virtanen
Marianna Virtanen
Cobus Visagie
Livia Visai
Tapio Visakorpi
Piero Visconti
Jeane Visentainer

Tatiana Vishnivetskaya
Saraswathi Vishveshwara
Rohini Vishwanathan
Mohan Vishwanathan
Karen Visick
Klaudija Viškovic
Riccardo Vismara
Shelina Visram
Bryce Vissel
Kris Vissenberg
Theo Visser
Bart Visser
Loes Visser
Susanna Visser
Nienke Visser
Frank Visseren
Arian Vistamehr
Dorte Vistisen
Laura Visu-Petra
Dimitris Visvikis
Hema Viswambharan
Srikant Viswanadha
Satish Viswanath
Vijay Viswanathan
Anand Viswanathan
Krupa Viswanathan
Siva Viswanathan
Velliyur Viswesh
Luigi Vitagliano
Sibylle Vital
Osmair Vital De Oliveira
Alessandro Vitale
Giovanni Vitale
Augusto Vitale
Ilio Vitale
Valeria Vitale
Francesco Vitale
Cristiana Vitale
Luca Vitale
Massimo Vitale
Daniel Vitales
Beatrice Vitali
Luca Agostino Vitali
Pavel Vitamvas
Antonio Vitarelli
Martha Vitaterna
Libor Vítek
Luis Vitetta
Meththika Vithanage
Carlo Viti
Mariateresa Vitiello
Chiara Vitiello
Giuseppe Vitiello
Ricardo Wagner Vitor
Silia Vitoratou
Heidi Vitrac
Olivier Vitrac
Agnes Vitry
Joar Vitterso
Eric Vittinghoff
Amy Vittor
Jean Ricardo Simões Vitule
Marco Vivarelli
Miguel Viveiros
Jonathan Viventi
Martha Vives
Cristofol Vives-Bauza
Lauraine Vivian
Jay Vivian
Roberto Viviani
Chiara Viviani Anselmi
Sheehan Vivien
Eric Vivier
Melane Vivier
Juana Maria Vivo Molina
Raul Vizcardo
Marcela P Vizcaychipi
Peter Vize
Franco Vizeacoumar
Marisa Vizuete
Gianmarco Vizzeri
Alfredo Vizzini
Giannina Vizzot
Jiri Vlach
Evangelos Vlachos
Vladimir Vladimirov
Virginie Vlaeminck-Guillem
Ross Vlahos
Srdjan Vlajkovic
Kris Vleminckx
Anh-Thu Vo
Bay Vo
Nam Vo
Don Voaklander
Anthony Vodacek
Irena Vodenska

Pavel Vodicka
Nicolas Vodovar
David Voegeli
Norbert Voelkel
Sara Voelkel
Wolfram Voelker
Mirko Voelkers
Vincent Voelz
Joseph Vogel
Heiko Vogel
Lotte Vogel
Jörg Vogel
Arndt Vogel
Erin Vogel
Adam Vogel
Matthias Vogel
Alecia Vogel
Ulla Vogel
Christina Vogelaar
Katie Vogel-Anderson
Maria Vogelauer
Antje Vogelgesang
Petra Vogelsang
Roberto Vogler
Volker Vogt
Guillaume Vogt
Katrin Vogt
Julia Vogt
Liffert Vogt
Emily Vogtmann
Marie-Claude Vohl
Kathleen Vohs
Antje Voigt
Barbara Voigt
Kristin Voigt
Stacy Voils
Catalin Voiniciuc
Christine Voisey
Aristo Vojdani
Kjetil Voje
Kinga Vojnits
Lara Vojnov
Steven Vokes
Martin Vokuka
Marco Volante
Vladislav Volarevic
M. Volbeda
Gregor Volberg
Roman Volchenkov
Filip Volckaert
John Volckens
Petr Volf
Jean-Nicolas Volff
Maxim Volgushev
Bela Volgyi
Ladislav Volicer
Oscar Volij
Stefano Volinia
Chris Volinsky
Margaritis Voliotis
Heather Volk
Timothy Volk
Hugo Alfried Volkaert
Gunnar Völkel
Uwe Völker
Rer. Nat. Volker Endris
Gesa Volkers
Michael Volkert
Dorothee Volkert
Helene Volkoff
Alex Volkov
Zeev Volkovich
Lars Voll
Niels Vollaard
David Volle
Leif Asbjørn Vøllestad
Johann Vollmann
Barbara Vollmayr
Waldemar Vollmer
Brigitte Vollmer
Catello Vollono
Margarete Vollrath
Stein Emil Vollset
Elena Volodina
Alexander Vologodskii
Elena Volokhina
Stefano Volpato
Massimo Volpe
Fernando Volpe
Stella Volpe
Elisabetta Volpe
Maria Grazia Volpe
Antonio Volpi
Giovanni Volpicelli
Rildo Volpini
Heinrich Volschenkh

Jordi Voltas
Erik Volz
David Volz
Claudia Von Bastian
Mikaela Von Bonsdorff
Johannes Von Burstin
Noreen Von Cramon-Taubadel
Peter Von Dassow
Yasmin Von Dassow
Neal Von Dawson
Elisabeth Von Dem Hagen
Karen Von Deneen
Gerhard Von Der Emde
Tobias Von Der Haar
Moritz Von Der Lippe
Titus Von Der Malsburg
Charles Von Der Meden
Werner Von Der Ohe
Pierre-Yves Von Der Weid
Arnold Von Eckardstein
Ferdinand Von Eggeling
Silke Von Esenwein
Guido Von Figura
Alexander Von Gise
Arndt Von Haeseler
Jost Von Hardenberg
Isabella Von Holstein
Mark Von Itzstein
Yskert Von Kodolitsch
Matt Von Konrat
Kathrein Von Kopylow
Rudiger Von Kummer
Marie Von Lilienfeld-Toal
Marieke Von Lindern
Johannes Von Lintig
Veronika Von Messling
Cornelius Von Morze
Georg Von Polier
Christine Von Reyn
Philipp Von Roth
Chris Von Rueden
Malcolm Von Schantz
Emily Von Scheven
Daniel Von Schiller
Johan Von Schreeb
Esther Von Stebut
Nicole Von Steinbüchel
Sophie Von Stumm
Christine Von Toerne
Frauke Von Versen-Höynck
Sibylle Von Vietinghoff
Viktor Von Wyl
Andrea Von Zuben
Constantin Von Zur Muhlen
Florian Vondran
Thomas Vondriska
Roslyn Vongsuvanh
Thapanee Vongthongleur
Kenny Voon
Maarten Voordouw
Ellen Voorhees
Roeland Voorrips
Torsten Vor
H. Martin Vordermeier
Igor Vorechovsky
Josef Vormoor
Misha Vorobyev
Denis Voronin
Andriy Voronov
Margreet Vos
Adriaan Vos
Theo Vos
Koen Vos
Chandra Voshavar
Astrid Voskamp
Konstantinos Voskarides
Michiel Voskuil
Wilna Vosloo
Till Voss
S. Randal Voss
Björn Voß
Andreas Voss
John Voss
Jameson Voss
Oliver Voss
Kenneth Voss
Ulrikke Voss
Christine Voss
Jaroslav Vostal
Carmine Votta
Romé Voulhoux
Apostol Vourdas
Valérie Vouret-Craviari
Alexandra Voutsina
Gerassimos Voutsinas
Daniel Voyer

Jovanka Voyich-Kane
Bradley Voytek
Paul Voziyan
Georgios Vrakas
Iosif Vranakis
Am Vranceanu
Jelena Vrankovic
Lukas Vrba
Hugo Vrenken
G Vrensen
Klaas Vrieling
Manouk Vrieling
Gert Vriend
Chris Vriend
Minka Vries
Robert Vrijenhoek
Karen Vrijens
Bernard Vrijens
Tanja Vrijkotte
Janna Vrijsen
Patricia Vrinten
Susanne Vrtala
Olga Vsevolozhskaya
Bao Vu
Giang Vu
John Vucetich
Domagoj Vucic
Steve Vucic
Ljubica Vucicevic
Aleksandra Vuckovic
Ivan Vuckovic
Louis Vuga
Eric Vugrin
Severine Vuilleumier
Maja Vujic
Igor Vujic
Slobodan Vukicevic
Nenad Vukmirovic
Vladana Vukojevic
Vuk Vukovic
Lucy Vulchanova
Adina Vultur
Eero Vuoksimaa
Katriina Vuolteenaho
Cuong Vuong
Ann Vuong
Linh Vuong
Tri Vuong
Thuy Vuong
Quoc Vuong
Jaana Vuopio
Jaana Vuosku
Saleha Banu Vuyyuri
Dimitri Vvedensky
Vladimir Vyadov
Nilesh Vyas
Payal Vyas
Urvi Vyas
Vivek Kumar Vyas
Ajai Vyas
Vladyslav Vyazovskiy
Slavena Vylkova
Dimitrios Vynios
Emilia Vynnycky
Denis Vysotskii
Benjamin Vyssoki
Yuliya Vystavna
Rune Waagbo
Johannes Waage
Joseph Waas
Joe Waas
Colette Wabnitz
Katarzyna Wac
Sven Wach
Wojciech Wach
María Wacher Rodarte
Malte Wachsmuth
Mitchell Wachtel
Dagmar Wachten
Rolf Wachter
Thomas Wachtler
Margarethe Wacker
Sarah Wackerbarth
Henning Wackerhage
Lawrence Wackett
Catherine Wacongne
I. Waczulikova
Jun Wada
Hisashi Wada
Takayuki Wada
Yoshinao Wada
Taira Wada
Keiko Wada
Naohisa Wada
Hiroo Wada
Takehiko Wada

Hiroshi Wada
Hideo Wada
David Waddell
Amanda Waddell
Simon Waddington
Alex Wade
Charles Wade
Andrew Wade
Timothy Wade
Kaitlin Wade
James Wade
Richard Wade-Martins
Sunil Wadhwa
Roberto Wadington
Lyn Wadley
Danielle Wadsworth
Christian Waeber
Jamie Waese
Sam Wafula
Gerry Wagenaar
Bradley Wagenaar
Dirk Wagenaar
Lars Wagenfeld
Eric-Jan Wagenmakers
Jessica Wagenseil
Adrian Wagg
Ulf Wagner
Doris Wagner
Kay-Uwe Wagner
Nicole Wagner
Martin Wagner
Bridget Wagner
Victoria Wagner
Daniel-Christoph Wagner
Robert Wagner
Carol Wagner
Uwe Wagner
Carsten Wagner
Edward Wagner
Ryan Wagner
Johanna Wagner
S. Wagner
Caroline Wagner
Sven Wagner
Norman Wagner
Katie Wagner
Gabriel Wagner
Ana Wägner
Alexandre Wagner Souza Silva
Irene Wagner-Doebler
Georges Wagnieres
Konrad Wagstyl
Satoshi Waguri
Rizwan Wahab
Hayfaa Wahabi
Grace Wahba
Magdi Waheed
Abdul Wahid
Kathryn Wahl
Martin Wahl
S. Wahl
James Wahl Iii
Niklas Wahlberg
Magnus Wahlberg
Tina Wahle
Claes Wahlestedt
Mats Wahlgren
Walter Wahli
Thomas Wahli
Karl Wahlin
Victoria Wahl-Jensen
John Wahren
Sun Wai
Jonathan Wai
Susanne Waiblinger
Sonia Waiczies
Jonas Waider
Christy Wails
Mark Wainwright
Derek Wainwright
Quinten Waisfisz
Jason Waithman
John Waitumbi
Harald Wajant
Shadi Wajih Hasan
Eric Wajnberg
Chandramohan Wakade
Kenji Wakai
Setsuko Wakao
Marvalee Wake
Richard Wakeford
Dustin Wakeman
Hironori Waki
Hiroaki Wakimoto
Motoaki Wakiyama
Ingrid Wakkee

Hiroshi Wako
Monika Waksmundzka-Hajnos
Julie Walabyeki
Daniel Walcher
Barbara Walch-Rückheim
Martin Wald
Frantisek Wald
George Waldbusser
William Walden
Hans Waldenmaier
Matthias Waldert
Joerg Waldhaus
Karen Waldie
Eliseu Waldman
Herman Waldmann
Herbert Waldmann
Levi Waldron
Sebastian Waldstein
Anna Walduck
Etienne Waleckx
Annemiek Walenkamp
Balwierz Walentyna
Nathan Wales
Alicia Walf
Harkamal Walia
Rasna Walia
Sumeet Walia
Przemko Waliszewski
Thomas Walk
Seth Walk
Henry Walke
David Walker
Mark Walker
Louise Walker
Alan Walker
Robert Walker
Brett Walker
Patrick Walker
Lori Walker
James Walker
Christopher Walker
Wade Walker
Jennifer Walker
Stephen Walker
Logan Walker
Larry Walker
William Walker
Harrison Walker
Timothy Walker
Mary Walker
Brandon Walker
Katherine Walker
Celia Walker
Pete Walker
Matthew Walker
Jane Walker
Donald Walker
Alesia Walker
John Walker
Ashley Walker
Drew Walker
Francis Walker
Ryan Walker
Johann Walker
Allan Walkey
Malcolm Walkinshaw
Steve Walkley
Carl Walkley
Gernot Walko
Judy Wall
Daniel Wall
Kristin Wall
Michael Wall
J. Gerard Wall
Carrie Wall
Benjamin Wall
Marlene Wall
Brian Wall
Peter Walla
John Wallace
Daniel Wallace
R John Wallace
David Wallace
Helen Wallace
Joseph Wallace
Richard Wallace
Mark Wallace
J. Wallace
Ian Wallace
Mikkel Wallentin
Lance Waller
Jo Waller
Donald Waller
Goran Waller
Shannon M. Wallet
Andrew Walley

Elisabeth Wallhäusser-Franke
Jacco Wallinga
Robert Wallis
Christopher Wallis
Deeann Wallis
Martin Wallner
Anders Wallqvist
Theodore Walls
Cara Wall-Scheffler
Kenneth Wallston
Katrina Walsemann
Bruce Walsh
Elizabeth Walsh
Julia Walsh
Michael Walsh
Garry Walsh
Eamonn Walsh
Ian Walsh
Jennifer Walsh
Matthew Walsh
Peter Walsh
David Walsh
Fiona Walsh
Kerryann Walsh
Ed Walsh
Darren Walsh
William Walsh
Lee Walsh
John Walsh
Tom Walsh
Stig Walsh
Ian Walshe
Judd Walson
Eric Waltari
Dagmar Waltemath
Michael Walter
W. David Walter
Scott Walter
John Walter
Klaudia Walter
Stefan Walter
Jens Walter
Steffen Walter
Jonathan Walter
Fruzsina Walter
Malcolm Walter
Van Der Merwe Walter
Carl Walters
Michael Walters
Kirsty Walters
Dylan Walters
James Walters
Stephen Walters
Theresa Walters
Giles Walters
Elizabeth Walter-Shea
Marina Walther-Antonio
Christopher Walthers
Ludo Waltman
Surrey Walton
Chad Walton
William Walton
Jonathan Walton
Ashley Walton
Ge Walton
Ann Walts
Brandon Walts
Xavier Waltz
Hasse Walum
Emily C. Walvoord
Thomas Walz
John Walz
Rosemary Walzem
Thierry Walzer
Andreas Walzer
Christian Walzer
Gerhard Walzl
Joyce Wamoyi
Erin Wamsley
Hongquan Wan
Ying Wan
Yinsheng Wan
Yanmin Wan
Xiu-Feng Wan
Emily Wan
Baikun Wan
Gwo-Hwa Wan
Jianmin Wan
Kai-Tak Wan
Yu-Jui Wan
Ji Wan
Chao Wan
Shanshan Wan
Xiang Wan
Xiao-Chun Wan
Wei Wan

Xia Wan
Haiying Wan
Zhiyu Wan
Zheng Wan
Xiaohong Wan
Jiafu Wan
Zi Yi Wan
Meher Wan
Jinrong Wan
Jinhong Wan
Ying Wooi Wan
Leo Wan
Wan Syaidatul Aqma Wan Mohd Noor
Johannes Wancata
Joshua Wand
Matthew Wand
Gilles Wandeler
Ronald Wanders
Jack Wands
David Wang
Edwin Wang
Daowen Wang
Wei Wang
Yufeng Wang
Jin Wang
Yong Wang
Ting-Fang Wang
Shaomei Wang
Shaomeng Wang
Yonghong Wang
Ping Wang
Haichao Wang
Yin Wang
Jin-Town Wang
Xifeng Wang
Ji-Ping Wang
Kevin Wang
Yanzhuang Wang
Shixia Wang
Fengjun Wang
Gene-Jack Wang
Lily Wang
Yujin Wang
Kan Wang
Qiang Wang
Conan Wang
Yanchang Wang
Hansen Wang
Dao Wen Wang
Yanming Wang
Jinhui Wang
Ching-Ho Wang
Mingyi Wang
Liang Wang
Yi-Xiang Wang
Jin-Feng Wang
Tobias Wang
Ya Wang
Xuefeng Wang
Lixin Wang
Yun Wang
Qun Wang
Kejian Wang
Gang Wang
Xiaozhong Wang
Chao Wang
Xiaowu Wang
Zhiquan Wang
Zhonghua Wang
Deshou Wang
Gary Wang
Chunxin Wang
Yibin Wang
Jian Wang
Hongbing Wang
Rong Wang
Gaofeng Wang
Lin Wang
I-Jen Wang
Feng Wang
Rennian Wang
Hurng-Yi Wang
Piwen Wang
Ying Wang
Jann-Yuan Wang
Fen Wang
Daryi Wang
Kai Wang
Chunyu Wang
Yachun Wang
Shizhen Wang
Aiming Wang
Xinglong Wang
Shi Wang
Jianhua Wang
Zhi-Liang Wang

Zhou Wang
Mei Wang
Juncun Wang
Haiyan Wang
Honggang Wang
Qinghua Wang
Chunming Wang
Jieru Wang
Dechun Wang
Yougui Wang
Zhiwei Wang
Yiming Wang
Mu Wang
Leyi Wang
Bin Wang
Zhihui Wang
Bao-Zhong Wang
Rong-Lin Wang
Jeff T. H. Wang
Congli Wang
Quanzhen Wang
Thomas Wang
Shan Wang
Jung-Der Wang
Renzhong Wang
Jiang Huai Wang
Chengshu Wang
Yin-Zheng Wang
Nian Wang
Huayan Wang
Li Wang
Deli Wang
Jun Wang
Chunyou Wang
Tao Wang
Yu-Ping Wang
Chuanxin Wang
Guoying Wang
Xiliang Wang
Chuan-Yue Wang
Jian-Ying Wang
Dong Wang
Zhong Wang
Jen-Ren Wang
Guodong Wang
Yigang Wang
Sheng Wang
Shao-Chun Wang
Liya Wang
Xinjiang Wang
Lijun Wang
Yuejin Wang
Qiong Wang
Nan Wang
Zhen Wang
Jin-Jun Wang
Haidong Wang
Tuanlao Wang
Qinglian Wang
Shusheng Wang
Yu-Chao Wang
Tong Wang
Suiping Wang
Youwei Wang
Jm Wang
Xiaonan Wang
Jianbo Wang
Shuxia Wang
Ningli Wang
Shucai Wang
Jianjun Wang
Xiping Wang
Zhiqiang Wang
Ying-Jan Wang
Henry Wang
Hui Wang
Xianhui Wang
Liangjiang Wang
Deng Wang
Ruipeng Wang
Xiumei Wang
Kun Wang
Tong-Hong Wang
Lingling Wang
Youping Wang
Qing Jun Wang
Zhixiang Wang
Tongguang Wang
Zhiyong Wang
Xuejun Wang
Mengcheng Wang
Jialiang Wang
Jianwei Wang
Minghua Wang
Li-Ya Wang
Shaolin Wang

Yaqiang Wang
Bingbo Wang
Xiue Wang
Renhou Wang
Xue-Qiang Wang
Binquan Wang
Shengjun Wang
Chunlin Wang
Yujie Wang
San Ming Wang
Guangshun Wang
Yajun Wang
Xu-Mei Wang
Maggie Haitian Wang
Hsiu-Po Wang
Rui Wang
Guirong Wang
Yongping Wang
Chengming Wang
Meilin Wang
Yunfei Wang
Jiaoyu Wang
Wei-Hua Wang
Xiang Wang
Hai-Long Wang
Han-Ching Wang
Jianxin Wang
Gao Wang
Qi Wang
Jianmin Wang
Yi-Hong Wang
Yi Wang
Ling-Zhi Wang
Szu-Heng Wang
Chi-Hwa Wang
Hui-Yun Wang
Xiaofang Wang
Shih-Wei Wang
Zhen-Bo Wang
Haizhen Wang
Yaqun Wang
Yonghua Wang
Shufang Wang
Yuanquan Wang
Jason T. L. Wang
Yongmei Wang
Huijuan Wang
Christina Wang
Hua Wang
Su-Jing Wang
Dong-Xin Wang
Faming Wang
Yan Wang
Vincent Wang
Jaw-Yuan Wang
Lei Wang
Jinghua Wang
Yan-Wu Wang
Ling Wang
Peizhong Wang
Yang Wang
Shenming Wang
Jing Wang
Jing-Houng Wang
Jianping Wang
Bo Wang
Man-Qun Wang
Cynthia Wang
Sihong Wang
Tzu-Wei Wang
Danny Wang
Richard Wang
Peigang Wang
Chenghui Wang
Yajing Wang
Yonglin Wang
Liping Wang
Le Wang
Xiaogang Wang
Yanlu Wang
Yu-Fei Wang
Hsin-Yi Wang
Dongli Wang
Panwen Wang
Sen Wang
Guoping Wang
Lianchun Wang
Wang Wang
Yongjie Wang
Dashun Wang
Jianguo Wang
Pei Wang
Xiaodan Wang
Qiuming Wang
Pa-Chun Wang
Silong Wang

Qing Wang
Pu Wang
Zaijie Wang
Daifeng Wang
Yanhui Wang
Lian-Tang Wang
Jingrong Wang
Zhanyong Wang
Xiuli Wang
Mansen Wang
Jiayi Wang
Lu Wang
Yui-Hsi Wang
Yongjun Wang
Songjie Wang
Ximing Wang
Haiying Wang
Ke Wang
Qigang Wang
Chunmei Wang
Yue Wang
Xiaofei Wang
Shunchun Wang
Huan Wang
Xuemin Wang
Xingxiang Wang
Kepeng Wang
Wen-Der Wang
Hongxin Wang
Xin Wang
Kehua Wang
Xuchu Wang
Haoran Wang
Xuerong Wang
Hui-Li Wang
Zan Wang
Chien-Kai Wang
Xinke Wang
Yucheng Wang
Tongli Wang
Lubin Wang
Jijun Wang
Jir-You Wang
Ming-Rong Wang
Peng-Hui Wang
Chong Wang
Zekun Wang
Zi-Yang Wang
Zhengjun Wang
Huali Wang
Yumei Wang
Miao Wang
Fang-Fang Wang
Liang-Jen Wang
Yu Wang
Han Wang
Haikun Wang
Wexin Wang
Xiaowei Wang
Demin Wang
Xiaoyan Wang
Ning Wang
Juan Wang
Hanzhou Wang
Guangliang Wang
Chunkao Wang
Weixin Wang
Yanjie Wang
Jingkuan Wang
Junming Wang
Ningning Wang
Mingshu Wang
Licheng Wang
Baomin Wang
Wenjian Wang
Yafeng Wang
Xu Wang
Zhaowei Wang
Yi-Zhong Wang
Yutao Wang
W. Wang
Jiebiao Wang
Zhiguo Wang
Yuan-Hung Wang
Fenghua Wang
Jiangang Wang
Wensheng Wang
Zhongkang Wang
Jiang-Hai Wang
Pengcheng Wang
Zhangying Wang
Qiaomei Wang
Yizhong Wang
Xiaoliang Wang
Jinniu Wang
Zhi Jian Wang

Qian Wang
Xiaoxi Wang
Yen-Feng Wang
Yisong Wang
Lifang Wang
Shuo Wang
Yanjun Wang
Li-Yuan Wang
Xiaodong Wang
Yongqin Wang
Jiaqi Wang
Wen-Xu Wang
Xue-Qin Wang
Yizhou Wang
Cheng Wang
Qiwei Wang
Xinjing Wang
Hong Wang
Jie Wang
Jih-Terng Wang
Hao-Ven Wang
Chun-Hou Wang
Zhongqiu Wang
Yanhong Wang
Ya-Wen Wang
Yongxiang Wang
Zhongfang Wang
Weiliang Wang
Xingzhi Wang
Xitao Wang
Wen-Ming Wang
Sonya Wang
Con Yi Wang
Yuchuan Wang
Heming Wang
Xiao Wang
Jing-Jy Wang
Yunqiang Wang
Lili Wang
Junbo Wang
Chuan Wang
Zhaohui Wang
Chun Wang
Xiaotong Wang
Zhaoxia Wang
Shenghui Wang
Wen-Hui Wang
Yan-Dong Wang
Jingyuan Wang
Shanshan Wang
Ziqing Wang
Guocan Wang
Zhijiang Wang
Lingshu Wang
Guijun Wang
Lizhong Wang
Zuoguan Wang
Zhe Wang
Zheng-Huan Wang
Xiangdong Wang
Yanping Wang
Josh Jianxin Wang
Yingying Wang
Weidong Wang
Xiaoying Wang
Xiuqin Wang
Jinkai Wang
Chung-Ju Wang
Chengbo Wang
Peng Wang
Difei Wang
Kung-Jeng Wang
Xingmin Wang
Fei Wang
Lan Wang
Siying Wang
Hongyi Wang
Hongjun Wang
Zhongxiao Wang
Xiangming Wang
Sida Wang
Baozhan Wang
Airong Wang
Shumin Wang
Lihai Wang
Junjun Wang
Xuwen Wang
Shuhang Wang
Weiwen Wang
Jinzhou Wang
Andrew Wang
Guangyu Wang
Hao Wang
Shu Wang
Zixing Wang
Xihua Wang

Donglin Wang
Guo-Qing Wang
Zhongxia Wang
Zhanwei Wang
Jaw-Fen Wang
Yi-Lei Wang
Xulong Wang
Ran Wang
Guohua Wang
Han-Cheng Wang
Guojing Wang
Jichen Wang
Chuanyuan Wang
Fang Wang
Chih-Jen Wang
Jeffrey Wang
Ruilan Wang
Shaoru Wang
Bing Wang
Yingfei Wang
Da Mao Wang
Lihon Wang
Chin-Cheng Wang
Deming Wang
Xiao-Wei Wang
Jiguang Wang
Nancy Wang
Hongwei Wang
Xiubin Wang
Wangsheng Wang
Qingfeng Wang
Fan Wang
Leyao Wang
Jinyang Wang
Dewei Wang
Yi-Ping Wang
Jian-Bo Wang
Weiwei Wang
Zhenhong Wang
Yen-Po Wang
Jennifer Wang
Qiannan Wang
Weicai Wang
Xiao Wang
Xiaohua Wang
Yina Wang
Heuy-Ching Wang
Caixia Wang
Yinyan Wang
De-Gao Wang
Changhong Wang
Quan Wang
Beixi Wang
Sherry Wang
Shu-Chi Wang
Yaping Wang
Baojing Wang
Seok Mui Wang
Baofeng Wang
Jian-Ping Wang
Su-Xia Wang
Heng Wang
Hao-Ching Wang
Tai Wang
Xuexia Wang
Honglin Wang
Tingyu Wang
Ruixuan Wang
Can Wang
Jw Wang
Xianwei Wang
Wenzhang Wang
Elizabeth Wang
Shuhui Wang
Yijie Wang
Fahui Wang
Han-I Wang
Peng-Wei Wang
Zhuoran Wang
Xiao-Dong Wang
Pichao Wang
Yulan Wang
Zeliang Wang
Jiayou Wang
Shi-Heng Wang
Moran Wang
Ligong Wang
Chen Wang
Jing-Feng Wang
Zhenjia Wang
Boyuan Wang
Limin Wang
Tian Wang
Yong-Xu Wang
Meng Wang
John Wang

Hequn Wang
Gangping Wang
Baojun Wang
Yuguang Wang
Yq Wang
Tengjiao Wang
Steven S.-S. Wang
Yuesheng Wang
Jufang Wang
Ji Wang
Yang-Kao Wang
Sc Wang
Dongxin Wang
Yi-Dong Wang
Xiaochun Wang
Haiping Wang
Junhui Wang
Jiadong Wang
Jieqiong Wang
Youxin Wang
David Zw Wang
Dongxiao Wang
Jia Wang
Pengfei Wang
Zhongyuan Wang
Guoqiang Wang
Min Wang
Edith Wang
Chun-Wei Wang
Feng-Qing Wang
Hongran Wang
Guan Wang
Guoxing Wang
Kewei Wang
Jimeng Wang
Yihan Wang
Zhongqiang Wang
Liang-Sheng Wang
Jinxiang Wang
Shuaian Wang
Ting Wang
Fanghai Wang
Xiaoli Wang
Ningtao Wang
Jean Wang
Wenyi Wang
Guangxing Wang
Feng-Sheng Wang
Luhua Wang
Zhenyu Wang
Zunyao Wang
Sha Wang
Zhen-Guang Wang
Jiejing Wang
Chienkai Wang
Jianbin Wang
Ruoxiang Wang
Yujuan Wang
Tingliang Wang
Hong-Bin Wang
Yungang Wang
Philine Wangemann
Gefu Wang-Pruski
Khursheed Wani
Jacek Waniewski
Sameh Wanis
Christine Wanke
Dierk Wanke
Erich Wanker
Kenneth Wann
George Wanna
Barry Wanner
Kevin Wanner
Samuel Wanner
Andreas Wannhoff
Sylvia Wanzala
Edina Wappler
Erik Wapstra
Jonathan Warawa
Marilyn Warburton
Bruce Warburton
Darren Warburton
Simon Warby
Peter Ward
Lawrence Ward
Joy Ward
Philip Ward
Carol Ward
Robert Ward
Leigh Ward
Tonya Ward
Alex Ward
Ashley Ward
Todd J Ward
Evan Ward
Richard J Ward

Douglas Ward
Alastair Ward
Phillip Ward
Laura Ward
Jeffrey Ward
Derek Ward
Andrew Ward
Ryan Ward
Rachel Ward
Emily Ward
Doyle Ward
Christopher Ward
Claire Wardak
Jeffrey Wardell
Grant Wardell-Johnson
Lucas Wardil
Margaret Wardle
Norma Ware
Jennifer Ware
John Ware
Evan Warfel
Anna Wargelius
Matthew Wargo
Pawel Wargocki
Justin Waring
Christina Warinner
Gulam Waris
Peter Wark
Luk Warlop
Matthew Warman
Laura Warman
Vera Warmuth
Kasturi Warnakulasuriya
Klaus Warnatz
Athanasia Warnecke
Mark Warner
Robert Warner
Timothy Warner
Digby Warner
Jacob Warner
Stephanie Warner
David Warner
Barbara Warner
Michael Warner
Ryan Warner
Andrea Warner-Czyz
Lorin Warnick
David Warnock
James Warnock
Katherine Warpeha
Jason Warram
Eric Warrant
H. Shaw Warren
Martin Warren
Sean Warren
Daniel Warren
Barbara Warren
Charles Warren
Robert Warren Ii
Anni Wärri
Sudha Warrier
David Warriner
Arthur Warrington
Nicole Warrington
Adilia Warris
Petra Warschburger
Arjumand Warsy
Roger Wartell
Kristina Warton
Clifford Warwick
Jim Warwicker
Kishore Wary
Zygmunt Warzecha
Halina Was
Gustav Waschatko
Anne Waschbisch
James Waschek
Claudia Wascher
Jens Waschke
Nicole Wäschke
Benjamin Waschki
Ahmad Waseem
Nick Waser
Markus Waser
Jason Washburn
Reynold Washington
Sierra Washington
Anthony Washington
Robert Washington-Allen
Agata Wasik
Bethany Wasik
Lucy Waskell
Marcin Krzysztof Wasko
Claudia Waskow
Thomas Wasow
Pawel Wasowicz

Stephen Wassall
Christoph Wasser
Clive Wasserfall
Edward Wasserman
Eric Wassermann
Anne Mai Wassermann
Kate Wassum
Claus Wasternack
Bohdan Wasylyk
Tetsuro Watabe
Shigeru Watanabe
Takeo Watanabe
Yuuki Watanabe
Hideto Watanabe
Michiko Watanabe
Toshio Watanabe
Norihiko Watanabe
Yoichi Watanabe
Tomohiro Watanabe
Yuichiro Watanabe
Hiroshi Watanabe
Tetsu Watanabe
Hayafumi Watanabe
Hiroyuki Watanabe
Kimio Watanabe
Atsushi Watanabe
Hirotoshi Watanabe
Tsunamasa Watanabe
Akinobu Watanabe
Yasuo Watanabe
Hirohisa Watanabe
Toshiaki Watanabe
Naoki Watanabe
Yoh-Ichi Watanabe
Chiho Watanabe
Hirofumi Watanabe
Noriya Watanabe
Kazuhito Watanabe
Yosuke Watanabe
Hajime Watanobe
Koichi Watashi
Grant Waterer
Nicholas Waterfield
Clare Waterman
Patrick Waters
Wade R. Waters
Chris Waters
Christopher Waters
Sinead Waters
Dean Waters
Andrew Waters
Jillian Waters
John Waterton
Elizabeth Watkin
Adam Watkins
Christopher Watkins
David Watkins
Siobhan Watkins
Harriet Watkins
Michael Watkinson
Les Watling
James Watling
Christopher Watling
Erwan Watrin
Andrew Watson
Cheryl Watson
Rebecca Watson
Dennis Watson
Adrian Watson
Roger Watson
Marcus Watson
Charles Watson
Emma Watson
Douglas Watson
William Watson
David Watson
Rachel Watson-Jones
Richard Watt
Penelope Watt
Eric Wattel
Kristi Watterberg
Martin Watterson
Anneleen Watteyn
Pierre Wattiau
Ma Wattiaux
Nick Wattie
Gaetan Wattieaux
Mike P. Wattjes
Joel Watts
Jennifer Watts
George Watts
Joy Watts
Heather Watts
Bradley Watts
Jeffrey Watts
Andrew Watts

Ian Watts
Sarah Watts
Milind Watve
Bernhard Watzl
Christian Waugh
Nadia Wauquier
Lucas Wauters
Jean-Luc Wautier
Nadeeka Wawegama
Mathias Wawer
Stephan Wawra
Adam Wax
Samuel Waxman
Joshua Waxman
Michael Way
Tzong-Der Way
Astri Wayadande
Mary Waye
John Waye
William Wcislo
Jessica Weafer
Dan Weary
Nathaniel Weathington
Scott Weaver
Connie Weaver
Valerie Weaver
Marcia Weaver
Mark Weaver
Ian Weaver
John B Weaver
David Weaver
Starla Weaver
Zoe Weaver Ohler
Steven Webb
Stephen Webb
Jacqueline Webb
Cameron Webb
Ian Webb
Clinton Webb
Jonathan Webb
Andrew Webb
R. Webb
Steve Webb
Ashley Webb
Ann Webb
Bruce Webber
Gail Webber
Richard Webby
Friedemann Weber
Gerhard Weber
Griffin Weber
Irene Weber
Alexander Weber
Andreas Weber
Stefanie Weber
Arthur Weber
Daniel Weber
Don Weber
Jeffrey Weber
Kirsten Weber
David Weber
Ann Weber
Lynn Weber
Ingmar Weber
K. Scott Weber
Eilika Weber-Ban
Jayne Webster
Angela Webster
Simon George Webster
Carl Webster
Ben Webster
Christopher Webster
Joshua Webster
A Wechalekar
Mihir Dilip Wechalekar
Floyd Weckerly
Byron Weckworth
Stefan Weckx
Claus Wedekind
Mathew Wedel
Mats Wedin
Sonja Wedmann
Hwee-Lin Wee
Chong-Yaw Wee
Alison Wee
Michelle Weech
Brendan Weekes
Kevin Weeks
Andrew Weeks
Emma Weeks
Justin Weeks
Jon Weeks
Stephen Weeks
Amy Weeks
Robert Weeks
Vivian Weerdesteyn

Scott Weese
Florian Wegehaupt
Christian Wegener
Alfred Wegener
Andrea Weghofer
Barbara Wegiel
Jerzy Wegiel
Pete Wegier
Joost Wegman
Lars Wegner
Mathias Wegner
Grzegorz Wegrzyn
E. Wegrzyn
Jennifer Wehmeyer
Bernhard Wehrle-Haller
Bernhard Wehrli
Fernando Wehrmeister
Fuwen Wei
Haiming Wei
Li-Na Wei
Hongping Wei
Dongqing Wei
Shu-Jun Wei
Ling Wei
Jun Wei
Wenhui Wei
Jia Wei
Gao-Xia Wei
Kaifa Wei
Kunlin Wei
Tiandi Wei
Dongtao Wei
Hairong Wei
Hui Wei
Heming Wei
Z.J. Wei
Daoyan Wei
Juncheng Wei
Binnian Wei
Wu Wei
Qing Wei
Yanzhang Wei
Jiankai Wei
Yiyong Wei
Liu Wei
Shan Wei
Jing Wei
Bin Wei
Wei Wei
Kai Wei
Dan-Dan Wei
Yu-Chung Wei
Guanghui Wei
Yumei Wei
Gh Wei
Daijun Wei
Shuo Wei
Yingying Wei
Lei Wei
Haiyang Wei
Chuanyu Wei
Guowu Wei
Jianhua Wei
Chih-Hsuan Wei
Yuanyuan Wei
Chi-Ju Wei
Xiang Wei
Lijian Wei
Lichun Wei
Wenjuan Wei
Ying Wei
Junhong Wei
Ming-Gang Wei
Yehuda Dennis Wei
Chih-Lin Wei
Alexander Wei
Jie Wei
Ke Wei
Xinghua Wei
Qi-Huo Wei
Yudan Wei
Robert Weibel
Mario Weick
Martin Weickert
Benjamin Weide
Thomas Weidemann
Christopher Weidenmaier
Claudia Weidensteiner
Gilbert Weidinger
Karel Weidinger
Manfred Weidmann
Carola Weidner
Ronald Weigel
Udo Weigel
Patrick Weigelt
Maximilian Weigend

Cora Weigert
Martin Weigt
Shannon Weigum
Dolf Weijers
Patrick Weik
Gary Weil
Robert Weil
Mark Weiland
Daniel Weile
Francois-Xavier Weill
Arved Weimann
Paul Weimer
Jill Weimer
Katja Weimer
Henri Weimerskirch
Marc Weinberg
Clarice Weinberg
Kenneth Weinberg
Ethan Weinberg
Adriana Weinberg
Armin Weinberg
Ellen Weinberg
Robert Weinberg
Peter Weinberg
Seth Weinberg
Geoffrey Weinberg
Anna Weinberg
Daniel Weinberger
Paul Weinberger
Andrea Weinberger
Catherine Weinberger
Barry Weinberger
Avi Weinbroum
Marco Weinburg
Günther Weindl
David Weindorf
Howard Weiner
David Weiner
Ina Weiner
January Weiner
Joshua Weiner
Scott Weiner
Michael Weiner
Agnes Weiner
Lucy Weinert
Sebastian Weingaertner
Helge Weingart
Toby Weingarten
Markus Weingarth
Oliver Weingärtner
Diana Weinhold
Alexander Weinhold
Claus Weinholdt
John Weinman
Orly Weinreb
Brian Weinrick
Daniel Weinstein
Aviv Weinstein
Bianca Weinstock-Guttman
June Weintraub
Andrej Weintraub
Robert Weinzierl
Bruce Weir
Tifany Weir
Matthew Weir
Dascha Weir
Gregor Weirich
Jonathan Weir-Mccall
Stephen Weis
Joachim Weis
Cornelia Weise
Christopher Weise
Sara Weisenbach
Sheri Weiser
Mark Weiser
Thomas Weiser
Nicole Weiskopf
Noah Weisleder
Ronit Weisman
Gary Weisman
Julie T. Weismoore
Eric Weiss
William Weiss
Louis Weiss
Scott Weiss
Allison Weiss
Samuel Weiss
Siegfried Weiss
Michael Weiss
Jerrold Weiss
Robert Weiss
Frank Weiss
Thomas Weiss
Ram Weiss
Günter Weiss
Curtis Weiss

Lauren Weiss
Avery Weiss
Karl-Heinz Weiss
Bjoern Weiss
Aaron Weiss
Brigitte Weiß
John Weiss
Roy Weiss
Elizabeth Weiss
Marc Weissburg
Nicole Weisschuh
Herbert Weissenböck
Hansi Weissensteiner
Lena Weissert
Tracey Weissgerber
Franz Weissing
Alison Weisskopf
Bernard Weissman
Kira Weissman
Ariel Weissman
Louis Bastien Weiswald
Alessandro Weisz
Jeremy Weisz
Giora Weisz
Nathan Weisz
Adam Weitemier
Maria Weitoft
Nathaen Weitzel
Megan Weivoda
Alon Weizer
Wu Wei-Zhong
Lior Weizman
Ronald Wek
Babeth Weksler
Robert Weladji
Warren Welbourn
Susan Welburn
Matthew Welch
Mark Welch
David Welch
Kelton Welch
Dan Welch
Boye Welde
Christopher Weldon
William Weldon
Scott Welford
Jeffrey Welge
Antje Welge-Lüssen
David Weliky
Martin Welk
Jeffrey Welker
Frido Welker
Thomas Wellems
Maren Wellenreuther
James Weller
Donald Weller
Simon Weller
Patrick Weller
Dave Weller
Sebastian Weller
Robert Welliver
Justin Wellman
Robin Wellmann
James Wells
Charles Wells
Dagan Wells
Claire Wells
Timothy Wells
Dominic Wells
Brian Wells
Harrington Wells
Jim Wells
Daniel Wells
Scott M. Wells
Robert Wells
David Wells
Wendy Wells
Michael Wells
J.R. Wells
Konstans Wells
Tony Wells
David Wellsted
Bryan Welm
Lisa Welp Smith
Michiel Wels
Ian Welsby
Paul Welsh
Timothy Welsh
Robert Welsh
Thomas, Jr. Welsh
Jane Welsh
Corrine Welt
Tobias Welte
Cornelia Welte
Ingeborg Welters
Celeste Welty

Stephen E Welty
Franziska Wemheuer
Li Wen
Yu-Mei Wen
Rong Wen
Haitao Wen
Zezhang Wen
Yongjun Wen
Shi Wu Wen
Jie Wen
Bing Wen
Fuqiang Wen
Han Wen
Chi-Kuang Wen
Yi Wen
Xiaopeng Wen
Chenglu Wen
Yefei Wen
Xiaoyun Wen
Yingxia Wen
Sicheng Wen
Yu-Wen Wen
Ting Wen
Adam Wende
Jonathan Wendel
Nicole Wenderoth
Volker Wendisch
Petra Wendler
Roman Wendner
Chris Wendt
Amanda Wendt
Bernd Wendt
Julia Wendt
Christopher Wendt
Michael Wendt
Andrew Weng
Hong-Lei Weng
Ching-Feng Weng
Ensheng Weng
Chia-Jui Weng
Meng-Shih Weng
Helen Weng
Liming Weng
Jingwei Weng
Yi-Hao Weng
Hongming Weng
Yanqiu Weng
Ti Weng
Lu-Chen Weng
Tobias Wengenmayer
Roland Wenger
Edward Wenger
Godehard Weniger
Gerd-Christian Weniger
Cao Wenjun
Gary Wenk
Malin Wennström
Michel Wensing
Annemarie Wensing
Lislaine Wensing
Emily Wentzell
Ulrich O Wenzel
Helmut Wenzel
Barbara Wenzel
Frank Wenzhöfer
Byung Mook Weon
Tamra Werbowetski-Ogilvie
Lars Werdelin
Cedric Werely
Adriano Werhli
Markus Werkle-Bergner
Jerome Werkmeister
Dirk Werling
Kai Wermker
Guilherme Werneck
Miriam Werneck
Fernanda Werneck
Renata Werneck
Jan Werner
Hauke Werner
Jeffery Werner
Erika Werner
Max Werner
Perla Werner
Jan Wernerman
Sebastian Wernicke
Kerstin Wernike
Sarah Werning
Anja M. Werno
Jonathan Werry
Denise Werry
Heike Wersching
Susan Wert
Victoria Werth
Alexander Werth
Silke Werth

Joel Wertheim
Efrat Wertheimer
Jasmin Wertz
Robert Weryk
Louise Weschler
Daniel Wescott
Lukas Weseslindtner
Cedric Wesley
Johnna Wesley
Jagila Wesley
Susan Wesmiller
Jeff Wesner
Andreas Wespi
Jürgen Wess
Tim Wess
Patrick Wessa
Niels Wessel
Jennifer Wessel
Andreas Wessel
Justus Wesseler
Jelle Wesseling
Joeri Wesseling
Robert Wessells
Michael Wessels
Andy Wessels
Oliver Wessely
Rainer Wessely
Carolyn Wessinger
Martin Wessling
Barbara Wessner
Hadley Wesson
D Wesson
Jevin West
T. Eoin West
Anne West
Andrew West
Nicholas West
Keith P. West
Daniel West
Erin West
Richard West
Sonlee West
Stephen Westaby
Jules Westbrook
John Westbrook
Chris Westbury
Erik Westein
Astrid Westendorf
Rudi Westendorp
Vincent Wester
Lisa Westerberg
Matthew Westercamp
Nelli Westercamp
Frank Westerhoff
J. Westerink
Michael Westerlund
Claudia Westermann
Gunilla Westermark
Birgit Westernströer
Theresia Westers
Klaas Westerterp
Marit Westerterp
Jake Westfall
Gerald Westheimer
Mike-Andrew Westhoff
Kieran Westley
Karin Westlund
Karen Westlund
Per-Olof Westlund
Karin Westlund High
Mark Westneat
Leslie Weston
Peter Weston
Matthew Weston
Lynn Westphal
Johanna Westra
Clara Westwell-Roper
Nicholas Westwood
Mark Wetherell
Stacey Wetmore
Cynthia Wetmore
Axel Wetter
Chad Wetterneck
Joseph Wettstein
Ronald Wetzel
Jack Wetzels
Regina Wetzer
Martin Wetzke
Reinhard Wetzker
Kimberley Wever
Rachel Wevrick
Thomas Wex
Tina Wey
Cornelia Weyand
Patrick Weydt
Jacqueline Weyer

Steven Weyers
Karsten Weylandt
Mathias Weymar
Katelyn Weymouth
Andrew Weyrich
Steve Whalan
Alex Whaley
Brigitta Whaley
Young Whang
Peter Whang
Katharine Whartenby
Robin Wharton
Amy Wharton
Mitchell Wharton
David Wheatcroft
Adam Wheatley
Michael Wheaton
James Whedon
Ward Wheeler
Derek Wheeler
Tisha Wheeler
Krista Wheeler
David Wheeler
Sarah Wheeler
Stephen Wheeler
James Wheeler
Jean Wheeler
Ana Wheelock
Nathan Whelan
Stephen Whelan
Laura Wherry
Leah Whigham
Geoff While
Ian Whishaw
Corrie Whisner
Stephen Whisson
Desley Whisson
Melissa Whitaker
Katriina Whitaker
Lucy Whitaker
David Whitall
Katherine Whitcome
Theodore White
K. Andrew White
Peter White
Laura White
Kristin White
Benjamin White
Christine White
John White
Tim White
Olivier White
Stephen White
Andrea White
Sarah White
Angela White
J. Wilson White
Patricia White
Emily White
Susan White
Nathan White
Tara White
Carl White
David White
Andrew White
James White
Helene White
Stuart White
Jen White
J. Peter White
Kate White
Daniel Kenta White
Tom White
Richard White
Helen White
Douglas White
Joyce White
Arthur White
Kevin White
Rachel White
Jeff Whiteaker
John Whited
J Whiteford
Brian White-Guay
Hal Whitehead
Ross Whitehead
Amy Whitehead
Andrew Whitehead
Sidney Whiteheart
Adrian Whitehouse
Andrew Whitehouse
Jamie Whitehouse
Christopher Whitehurst
Andrew Whitelaw
Nia Mererid Whiteley
William Whiteley

Lisa Whitenack
Luke Whitesell
Alan Whiteside
Theresa Whiteside
Omar Whiteside
Lauren Whiteside
Katrine Whiteson
Steven Whitfield
Troy Whitfield
Veronica Whitford
Steven Whitham
Charlotte Whitham
Martin Whitham
Jonathan Whitlock
Donna Whitlon
Mary Whitman
Jennifer Whitman
Andrew P. Whitmore
Robert Whitmore
Mark Whitnall
Cynthia Whitney
James Whitney
Paul Whitney
Nick Whitney
Jeffrey Whitsett
Gary Whittaker
James Whittaker
Joanne Whittaker
Alexandra Whittaker
Robyn Whittaker
William Whitten
Sarah Whittle
J. Lindsay Whitton
Chris Whitton
John Whitton
Jennifer Whitty
Stephen Whybrow
Cari Whyne
Michael Whyte
Marlene Wiart
Gudrun Wibbelt
Daric Wible
Carl Wibom
Ove Wiborg
Jan Wiborg
Nani Wibowo
Michael Wibral
Janine Wichmann
Ignacio Wichmann
Fabienne Wichmann
Søren Wichmann
Wolfgang Wick
Macdonald Wick
Bruno Wicker
Ian Wickersham
James Wickham
Carol Wicking
Kyle Wickings
Samuel Wickline
Ratnam Wickneswari
Nadi Wickramasekera
Amittha Wickrema
Eric Wickstrom
Aris Widayati
Robert Widdop
Anders Widell
Jeremy Wideman
Elisabeth Widén
Ralf Widenhorn
Micael Widerström
Alan Widgerow
Alexander Widiapradja
Thomas Widiger
Michael Widlansky
Douglas Widman
Erik Widman
Giovanni Widmer
Hans Widmer
Karen Wiebe
Ellen Wiebe
Magdalena Wiecek
Katja Wiech
Helmut Wieczorek
Kinga Wieczorek
Wolf Wiedemeyer
Steven Wiederman
Christian Wiedermann
Markus Wiedmann
Ryan Wiegand
Iris Wiegand
Susan Wiegers
Kevin Wiehe
Arno Wiehe
Lothar Wieler
Robert Wielgus
Tadeusz W. Wieloch

Ben Wielockx
Mark Wielpütz
Ben Wielstra
Philipp Wiemann
Joseph Wiemels
Erik Wiemer
Martin Wiemers
Timothy Wiemken
Swantje Wienand
Claudia Wienberg
Christian Wiencke
Jan Wiener
Martin Wiener
Klaus Wienhard
Brian Wienhold
Bruce Wienke
Gregory Wiens
W. Gil Wier
Alicja Wiercinska-Drapalo
Rik Wierenga
Anne Wierinckx
Frank Wieringa
Margaret Wierman
John Wiernikowski
R. Wiers
Rodney Wiersma
Yolanda Wiersma
Nils Wierup
Thomas Wierzba
Piotr Wierzbicki
Joanna Wierzbowska
Jennifer Wies
Richard Wiese
Holger Wiese
Eva Wiese
Andreas Wieser
Jeffrey Wieskopf
Charlotte Wiesmann
Darin Wiesner
Melanie Wiesner
Roland Wiest
Reiner Wiest
Whitney Wiest
Evie Wieters
Mareike Wieth
Maciej Wiewióra
Martin Wiewiorski
Brian Wigdahl
Vanessa Wiggermann
Christopher Wiggins
Natasha Wiggins
Janey Wiggs
Daniel Wight
John Wightman
Jeffrey Wigle
Sivaramesh Wigneshweraraj
William Wiist
Lahiru Wijedasa
Berry Wijers
Sanjay Wijesekera
Danushka Wijesundara
Peter Wijkstra
Hein Wijma
Inge Wijnberg
Ellen Wijsman
Anne Wijtzes
Jan Wikgren
Christopher Wikle
Lars Wiklund
Erik Wikstrom
John Wikswo
Dara Wilber
Willy Wilbur
Paul Wilburn
Andrew Wilby
Philip Wilby
Donna Wilcock
Jane Wilcock
David Wilcockson
George Wilcox
Mark Wilcox
Taylor Wilcox
Laurie Wilcox
Susan Wilcox
William Wilcox
Walter Wilczynski
Grzegorz Wilczynski
Martin Wild
Alex Wild
Margaret Wild
Edward Wild
Henry Wilde
Moritz Wildgruber
Karin Wildhagen
Craig Wilding
Christine Wildsoet

Ludwig Wildt
Vari Wileman
Jonna Wilen
Craig Wilen
Clayton Wiley
James Wiley
Michael Wiley
Kerrie Wiley
Julia Wilflingseder
Traci Wilgus
Dagmar Wilhelm
Steven Wilhelm
Brian Wilhelm
Barbara Wilhelm
Brendan Wilhelmi
Sherwin Wilk
Anna Wilk
Christopher Wilke
Hans-Joachim Wilke
Sarah Wilke
Stephan Wilkens
Elissa Wilker
Curtis Wilkerson
Rebecca Wilkes
Marcus Wilkes
Barbara Wilkey
Thomas Wilkie
Timothy Wilkin
Jon Wilkins
Marc Wilkins
Courtney Wilkins
Olivia Wilkins
Arnold Wilkins
Jayne Wilkins
Jenny Wilkinson
Lawrence Wilkinson
Gerald Wilkinson
Anthony Wilkinson
Brian Wilkinson
Robert Wilkinson
Katalin Wilkinson
J. Mark Wilkinson
George Wilkinson
Anna Wilkinson
Larrell Wilkinson
Patrick Wilkinson
John Wilkinson Iv
Jennifer Wilkinson-Berka
Ivy Wilkinson-Ryan
Colin Wilks
Mark Wilks
Elzbieta Wilk-Wozniak
Breelyn Wilky
Britta Will
Manuel Will
Mp Willand
Stephanie Willard
Gary Willars
Merlin Willcox
Mark Willcox
George Willcox
Holger Wille
Michelle Wille
Florian Willecke
Jose Willegaignon
Martin Willemink
Lucas Willems
Rob Willems
Rob Willemsen
Barry Willer
Henning Willers
Eske Willerslev
Karl Willert
Walter Willet
Peter Willett
Jonathan Willett
Brian Willett
James Willey
Joshua Willey
Jeffrey Willey
Kai Willführ
Yvonne Willi
Simpson William
Jeffrey William
Gwyn Williams
Brian Williams
Paul Williams
Heather Williams
Phillip Williams
Craig Williams
Redford Williams
Scott Williams
John Williams
Suzanne Williams
David Williams
Trevor Williams

Jacky Williams
Suzan Williams
Brett Williams
Julie Williams
Nigel Williams
Matthew Williams
Geoffrey Williams
Sion Williams
Christina Williams
Rohan Williams
Monique Williams
Dionna Williams
Larissa Williams
Richard Williams
Lawrence Williams
Robert Williams
Thomas Williams
Jason Williams
Lisa Williams
Joshua Williams
Amy Williams
Kristi Williams
Adam Williams
Geraint Williams
Paula G. Williams
Blythe Williams
Jacob Williams
Jon Williams
Susan Williams
Andrew Williams
Sean Williams
Perry Williams
Michelle Williams
Alun Williams
Sian Williams
Kenneth Williams
Shawn Williams
Martin Williams
Jan Williams
Nancy Williams
Timmons Williams
Allison Williams
Peter Williamson
Patrick Williamson
Anna-Lise Williamson
Ann Williamson
Esther Williamson
Amanda Willig
Craig Willis
Anne Willis
Mary S. Willis
Guillermo Willis
Stuart Willis
Hugh Willison
Kathryn Willits
William Willmore
Walter Willms
Johan Willner
Nik Willoughby
Michael Willoughby
Taryn Wills
Andrea Wills
Paul Wills
Richard Willy
Alan Wilman
Matthias Wilmanns
Christopher Wilmer
Paul Wilmes
Inge Wilms
Michael Wilschanski
Gerald Wilson
Rory Wilson
Duncan Wilson
Robbie Wilson
Keith Wilson
Jeffrey Wilson
Paul Wilson
Donald Wilson
Alan Wilson
Shaun Wilson
David Wilson
William Wilson
Patrick Wilson
Stuart Wilson
Andrew Wilson
Anne Wilson
Ian Wilson
Mark Wilson
Joyce Wilson
Gail Wilson
Heather Wilson
Michael Wilson
Brian Wilson
Daniel Wilson
Iain Wilson
Laura Wilson

Phillip Wilson
Jefferson Wilson
Fernando Wilson
Erin Wilson
S Wilson
Lisa Wilson
Alastair Wilson
Francis Wilson
Adam Wilson
Kenneth Wilson
Mark E. Wilson
Maximiliano Wilson
Justin Wilson
Brenda Wilson
Eric Wilson
Alan Wilson
Matthew Wilson
Charles Wilson
Alana Wilson
Lloyd Wilson
Aruni Wilson
Erin Wilson Rankin
Joerg Wilting
Andreas Wilting
Calistus Wilunda
Jeffrey Wilusz
William Wimley
Klaus Wimmers
Aung Ko Win
Hariyono Winarto
Cecilia Winata
Kristien Winckers
Christoph Winckler
François Windels
Danny Winder
Virginia Winder
Daniel Windhorst
Michael Winding
Brad Windle
Ceylan Windolf
L. Jack Windsor
Jeremy Windsor
Jack Windsor
Eytan Wine
Amy Winecoff
Benjamin Winegard
Amanda Winegardner
Daniel Winetsky
Mark Winey
Ian Winfield
Robert Winfield
Simon Wing
Gerhard Wingender
Quinton Winger
Paul Winger
Rebecca Wingert
Danielle Winget
Yvonne Winhofer
Katarzyna Winiarska
Michael Wininger
Ken Winkel
Alexandra Winkeler
John Winkelman
Renate Winkels
Beth Winkelstein
Melissa Winkle
Stefan Winkler
Christoph Winkler
Jürgen Winkler
Malcolm Winkler
Tilo Winkler
Alisa Winkler
Anderson Winkler
Kirsty Winkley
Mary Winn
Matthew Winn
Beate Winner
Arliss Winship
Richard Winsley
Craig Winstanlay
Craig Winstanley
Peter Winstanley
Erin Winstanley
Volker Winstel
Brent Winston
John Winston
Sebastian Winter
Stuart Winter
Bodo Winter
Lukas Winter
Julia Winter
Lilli Winter
Dorina Winter
Susanne Winter
Hanlie Winterbach
Jeffrey Winters

Sandra Winters
Ana Winters
Ole Winther
Robert Winton
Maxine Wintre
Paul Winyard
Ursula Winzer-Serhan
Didier Wion
Nadine Wiper-Bergeron
Rolf Wipfli
Anuwat Wiratsudakul
Linda Wires
Isaac Wirgin
Norman Wirsik
Aaron Wirsing
Carlheinz Wirsing Von Koenig
Dagmar Wirth
Michael Wirth
Michelle Wirthensohn
Mary Wirtz
Markus Wirz
William Wisden
Roger Wise
David Wise
Helen Wise
Paul Wise
Jennifer Wisecaver
Samantha Wisely
Frances Wiseman
Natalie Wiseman
Roger Wiseman
Brian Wisenden
Thomas Wishart
Lauren Wisk
Alan Wisler
Katherine Wisner
Stephen Wisniewski
Jacek Wisniewski
Jonathan Wisor
Bjoern Wissel
Karl Martin Wissing
Joachim Wistuba
Daniel Wiswede
Ferdinand Wit
Janneke Wit
Gemma Witcomb
Phil Withers
Adelaide Withers
Jeffrey Withey
John Withey
Christopher Witiw
Edward Witkowski
Jacek Witkowski
Lawrence Witmer
Alice Witney
Jens Witsch
Arne Witt
Michael Witt
Benjamin Witt
Joachim Wittbrodt
Wolfgang Witte
Herbert Witte
Adam Wittek
Christian Wittekind
Jurriaan Witteman
Ilana Witten
Alfred Wittinghofer
Harriet Wittink
Sergio Wittlin
Miriam Wittmann
Johannes Wittmann
Tibor Wittmann
Yohann Wittrant
Daniel Wittschieber
Kenneth Witwer
Thomas Witzel
Christoph Witzel
Carsten Witzel
Johanna Witzell
Martin Witzenrath
Florian Witzmann
Viroj Wiwanitkit
Benedykt Wladyka
Maciej Wnuk
Christian Wöber
Wendy Wobeser
Laila Woc Colburn
Andreas Wodarz
Andrew Woehler
Jens Woehnert
Birgitta Woehrl
Holger Woehrle
Johan Woelber
Gert Woerheide
Markus Woernle
Thorsten Wohland

Teddy John Wohlbold
Elizabeth Wohlfert
Nicholas Wohlgemuth
Thomas Wohlgemuth
Stephanie Wohlgemuth
Klaus Wohlrabe
Janine Wojcieszek
Edward Wojcik
Sean Wojcik
Slawomir Wójcik
Katarzyna Wojczulanis-Jakubas
Tomasz Wojdacz
Valerie Wojna
Johann Wojta
Jorgen Wojtaszewski
Johanna Wojtkowiak
Hanna Woksepp
Matthew Wolak
Cynthia Wolberger
Eric Wolbrecht
Gregor Wolbring
Anna Wolc
Randall Wolcott
Daniel Wolcott
Aaron Wolen
Dieter Wolf
Marina Wolf
Matthias Wolf
Eckhard Wolf
Bethany Wolf
Julie Wolf
Stephan Wolf
Patrick Wolf
Tiffany Wolf
Steven Wolf
Sebastian Wolf
Daniel Wolf
Steffen Wolf
Joost Wolf
Christian Wolf
Lisa Wolf
Dennis Wolf
Jennifer Wolf
Pedro Wolf
Michael Wolfe
John Wolfe
Alexander Wolfe
Alan Wolfe
Kenneth Wolfe
Kennedy Wolfe
Benjamin Wolfe
Heather Wolfe
Richard Wolfe
Brent Wolfe
Erin Wolff
Jonci Wolff
Ewan Wolff
Mary S. Wolff
George Wolff
Mark Wolff
Francois-Charles Wolff
James Wolffsohn
Michael Wolfgang
Michael Wolfinger
Ute Wölfle
Astrid Wolf-Magele
Mariana Wolfner
Karen Wolford
Julie Wolfram
Uwe Wolfrum
Christian Wolfrum
Sebastian Wolfrum
Joseph Wolfsdorf
Haim Wolfson
Christina Wolfson
Charles Wolgemuth
Michael Wolin
Nathan Wolins
Justyna Wolinska
Kerstin Wolk
Dieter Wolke
Klaus Wolkenstein
Bencie Woll
Scott Woller
Lutz Wollin
Gadi Wollstein
Gayle E. Woloschak
J. Mario Wolosin
Mieczyslaw Wolsan
Monalisa Wolski Pereira
Adrian Wolstenholme
Katja Wolthers
Steve Wolverton
Christiane Wolz
Ines Wolz
Jamie Womble

Seok Joon Won
Kyung Won
Doyeon Won
Fredric Wondisford
Joshua Wondra
Georg Thomas Wondrak
David Wong
Judy Wong
Albert Wong
Chung Wong
Carmen Wong
Oscar Wong
Cynthia A. Wong
Geoff Wong
Grace Wong
Wai Wong
Tien Wong
Samuel Yeung Shan Wong
Maria Wong
Hector Wong
Lily Wong
Jason Wong
Sek-Man Wong
Flora Wong
Luet Wong
Shun Wong
Aaron Wong
Kwong-Kwok Wong
Alex Wong
Irene O.L. Wong
Frank Wong
Richard Wong
William Wong
Ronald Wong
Stephen Wong
Tuck Seng Wong
Gerard Wong
Chongkim Wong
Chih-Shung Wong
Kwoon Wong
Vincent Wong
Evelyn Wong
Chun-Ming Wong
Kelvin Wong
Muh Wong
Ten Tsao Wong
Sally Wong
Ian Wong
Christopher Wong
Ken Wong
Wai Pong Wong
Brett Wong
Sunny Wong
Evan Wong
Darren Wong
Danny Wong
Ling-Tim Wong
Ngai Sze Wong
Madeline Wong
Won Fen Wong
Chris Wong
William Wai-Lun Wong
Jean Wong
Hin-Chung Wong
Victoria Wong
Annie Wong-Beringer
Jirasak Wong-Ekkabut
Nahathai Wongpakaran
Gabrielle Wong-Parodi
Margaret Wong-Riley
Cherry Wongtrakool
Ambroise Wonkam
Susan Wonnacott
Se Joon Woo
Robin Wood
Richard Wood
Christopher Wood
James Wood
Ian C. Wood
Tim Wood
Rachel Wood
John Wood
Andrew Wood
Jonathan Wood
Bayden Wood
Guilherme Wood
Eric Wood
Carly Wood
David Wood
Paul Wood
Nicholas Wood
Robert Wood
Susanna Wood
Joshua Wood
Stephen Wood
Troy Wood

Lara Wood
Sally Wood
Theresa (Terri) Wood
Ian Wood
Henry Wood
Michael Wood
Brian Wood
Chelsea Wood
Aaron Wood
Stephan Woodborne
Ben Woodcock
Stephen Woodcock
Abigail Woodfin
Michael Woodford
James Woodgett
James Woodhall
Jason Woodhouse
Stephen Wooding
Angela Woodiwiss
Robert Woodland
Owen Woodman
Emily Woodman
Karen Woodrow-Lafield
Cary Woodruff
Trent Woodruff
Steven Woods
Michael Woods
Jon Woods
Christopher Woods
Jeffrey Woods
Amina Woods
Andy Woods
Paul Woods
Cheryl Woods Giscombe
Sarah Woodson
Alistair Woodward
John Woodward
Holly Woodward
Jerold Woodward
Mark Woodward
Neil Woodward
Peter Wookey
Nerys Woolacott
Linda Wooldridge
Susan Woolfenden
John Woolford
Kevin Woollard
Laura Woollett
Thomas Woolley
Skip Woolley
Rossitza Wooster
R. Mark Wooten
Bharath Wootla
Denise Wootten
Sarah Wootton
Bethany Wootton
Patarawan Woratanarat
Elaine Worcester
Ruth Ann Word
Katharina Worda
Linda Wordeman
Franz Worek
Thierry Work
Biruh Workeneh
Deanna Worley
Boris Worm
Richard Wormald
Michael Wormstone
John Worobey
William Worodria
Viktoria Woronik
Adam Worrall
Greg Worrell
Caitlin Worrell
Roger Worrell
Maria Worsham
Calfee Worth
Margaret Worthington
Amy Worthington
Darrell Worthy
Harley Worthy
Samuel Wortman
Ada Wossink
Theresa Wossler
Han Wosten
Elijah Wostl
Bernd Wöstmann
Anni Woting
Emiel Wouters
Edwin Wouters
Maartje Wouters
Brian Wowk
Jennifer Woyach
Nancy Woychik
Tanja Woyke
Karen Wozniak

Daniel Wozniak
Michal Wozniak
Greta Wozniak
Grzegorz Wozniakowski
Lawrence Wrabetz
Nicole Wrage
Jens Wrammert
Selina Wray
Britta Wrede
Alexander Wree
Jonathan Wren
Brendan Wren
Yvonne Wren
Carsten Wrenger
Rosalind Wright
Geraldine Wright
Joe Wright
Phillip Wright
Timothy Wright
William Wright
Casey Wright
William Wright
Stephen Wright
Robin Wright
Gavin Wright
Nicholas Wright
Jeffrey Wright
Michael Wright
David Wright
Paul Wright
Clyde Wright
Natasha Wright
Jessica Wright
Melissa Wright
Eric Wright
Idan Wright
Nathan Wright
Julie Wright
Jennifer Wright
Alden Wright
James Wrightson
Karin Writzl
Lydia Wroblewski
Nina Wronkowitz
Stanislaw Wronski
Lisa Wruck
Michael Wrzaczek
Krzysztof Wrzesinski
Kazimierz Wrzeszczynski
Vladimir Wsol
Jiangping Wu
Louisa Wu
Reen Wu
Tangchun Wu
Chun-Fang Wu
Jiunn-Tzong Wu
Jian-Young Wu
Aiping Wu
Jianguo Wu
Allan Wu
Yaojiong Wu
Xiaosheng Wu
Zhiwei Wu
Jinyu Wu
Zunyou Wu
Yuzhang Wu
Baolin Wu
Qiao Wu
Jiong Wu
Alan Wu
Congming Wu
Zhijin Wu
Ming Wu
Peng Wu
Ruey-Meei Wu
Gen Sheng Wu
Hui Wu
Guoyao Wu
Kongming Wu
Bin Wu
Ann Wu
Hua-Lin Wu
Minghua Wu
Jun Wu
Renyi Wu
Xiao-Lun Wu
Keqiang Wu
Pingsheng Wu
Yih-Ru Wu
Chuan Wu
Ming-Shiang Wu
Ying Wu
Zhijian Wu
Kai Wu
Shu Wu
Min Wu

Te Wu
Weihua Wu
Zhaohui Wu
Jiahe Wu
Chaodong Wu
John Wu
Chen-Chi Wu
Qingfa Wu
Jixiang Wu
Henry Wu
Hung-Tsung Wu
Chuanjin Wu
Chengbiao Wu
Meiye Wu
Ping Wu
Guangyu Wu
Anhua Wu
Changwei Wu
Chenggang Wu
Wenting Wu
Wen-Bin Wu
Xiaohua Wu
Jing Wu
David Bin-Chia Wu
Ying-Li Wu
Fang-Tzy Wu
Wen-Chau Wu
Hao Wu
Keng-Liang Wu
Yen-Wen Wu
Qingyu Wu
Elwin Wu
Sheng Wu
Chunlai Wu
Shiyong Wu
Xueling Wu
Di Wu
Jianping Wu
Yiping Wu
Cen Wu
Zhengrong Wu
Wen Jin Wu
Lang Wu
Hongzhuan Wu
Zheng Wu
Xiujuan Wu
Yihua Wu
Christina Wu
Jianmin Wu
Kang Wu
I-Chien Wu
Fei Wu
Jinshan Wu
Qing Wu
Renhua Wu
Shaoling Wu
Dongyiog Wu
Chun-Ying Wu
Olivia Wu
Jiaping Wu
Qingjun Wu
Yinghao Wu
Bo Wu
Ming-Jiuan Wu
Yi Wu
Chuanfeng Wu
Jian-Li Wu
Renrong Wu
Ye Wu
Jie Wu
Ai-Ping Wu
Zhiqiang Wu
Xinchun Wu
Ximei Wu
Guangyao Wu
Dezhi Wu
Ding-Kwo Wu
Liang Wu
Tianfu Wu
Mei-Lin Wu
Jingjing Wu
Tao Wu
Xiaoqing Wu
Yilin Wu
Yanyuan Wu
Xufeng Wu
Hai Wu
Yongqi Wu
Mingxing Wu
Trong-Neng Wu
Christine Wu
Ruifang Wu
Baiyila Wu
Hongxian Wu
Yvonne Wu
Xiaohang Wu

Chaochao Wu
Haitao Wu
Hau-Tieng Wu
Hongli Wu
Wei Wu
Michael Wu
Yongwei Wu
Jiapeng Wu
Xm Wu
David Wu
Siva Wu
Wen-Chieh Wu
Jian-Ping Wu
Zeyan Wu
Zhenfang Wu
Ping-Hsun Wu
Junxiang Wu
Alexander Wu
Faqiang Wu
Went Wu
Xiaopeng Wu
Changrui Wu
Jin-Yi Wu
Nan Wu
Xiaoqun Wu
Yin Wu
Yongping Wu
Yuanyuan Wu
Geng Wu
Xinkai Wu
Wenxin Wu
Jinchang Wu
Hui-Lan Wu
Wei-Min Wu
Wei Te Wu
Simiao Wu
Guojun Wu
Can Wu
Jinhua Wu
Yu-Tse Wu
Xiao-Lei Wu
Chih-Feng Wu
Xuehai Wu
Ming-Heng Wu
Chengzhen Wu
Changqing Wu
Yonghe Wu
Chao Wu
Jayne Wu
Qian Wu
Congqing Wu
Mingfu Wu
Shengchun Wu
Jianjun Wu
Ziming Wu
Jianxin Wu
Jianhui Wu
Jian-Lin Wu
Ting Wu
Donghui Wu
Junbing Wu
Xuan Wu
Chin-Lee Wu
Yue Wu
Xiaomin Wu
Tongzhi Wu
Zhenlong Wu
Hongjie Wu
Junru Wu
Xiuyun Wu
Xiaoping Wu
Zhihao Wu
Xiaolin Wu
Wanqing Wu
Chih Wu
Shan Wu
Qin Wu
Qiaoyan Wu
Yao Wu
Ting-Fang Wu
Junjie Wu
Guangxi Wu
Joy Wu
Qihang Wu
Yi-Chun Wu
Guorong Wu
Jianwen Wu
Yeewen Candace Wu
Jianjie Wu
Junhui Wu
Kaili Wu
Shuang Wu
Juan Wu
Haiming Wu
Zhichao Wu
Qiao-Bing Wu

Andrzej Wuczynski
Darrell Wudunn
Max Wuehr
Melissa Wuellner
Karl Wuensch
Hanno Wuerbel
Jens Wuerfel
Judith Wuest
Rudolf Wuethrich
Gijs Wuite
Sabine Wulf
Heike Wulff
Mark Wulkan
Andy Wullaert
Stan Wullschleger
Claudia Wultsch
Matthew Wund
Michael Wunder
Elsio Wunder Jr
Richard Wunderink
Gerhard Wunderlich
Roshna Wunderlich
Being-Sun Wung
Li Wuping
Florian Wurm
Yannick Wurm
Susanne Wurst
Wolfgang Wurst
Rebecca Wurtz
Olivier Wurtz
Rolf Würtz
Sarah Wurz
Danielle Wurzel
Rob Wüst
Hilde Wustenberghs
Marcel Wuthrich
Wim Wuyts
Alexander Wyatt
Sean Wyatt
Michelle Wykes
David Wyles
Kristine Wylie
Blair Wylie
Stephen Wylie
Judith Wylie-Rosett
Sandy Wyllie-Echeverria
Laure Wynants
Jeanette Wyneken
Ursula Wyneken
Grace Wyngaard
James Wynn
Brandi Wynne
Jut Wynne
Lonce Wyse
Magdalena Wysocka
Charles Wysocki
William Wysocki
Marcin Wysoczynski
Markus Wyss
Matthias Wyss
Theodoros Xanthos
Michalet Xavier
Marília Brasil Xavier
Catarina Xavier
Jose Xavier-Neto
Yunhe Xe
Michalis Xenos
Panagiotis Xenoulis
Christian Xerri
Zhiyong Xi
Chuanwu Xi
Guohua Xi
Long Fu Xi
Lei Xi
Bo Xi
Jinxiang Xi
Tong Xi
Lin Xi
Ruibin Xi
Xuhua Xia
Huijing Xia
Harry Hua-Xiang Xia
Laixin Xia
Guoliang Xia
Guangmin Xia
Lijun Xia
Kai Xia
Yong Xia
Mingrui Xia
Jiahong Xia
Jian-Chuan Xia
Yiji Xia
Menghang Xia
Yun-Fei Xia
Cheng-Yi Xia
Jun Xia

Fen Xia
Yongxiang Xia
Zheng Xia
Junfeng Xia
Tian-Song Xia
Zengmin Xia
Ming Xia
Zhi-Qiang Xia
Lirong Xia
Jianguo Xia
Qijun Xia
Zhengyuan Xia
Hongfei Xia
Xiao-Jian Xia
Guoqing Xia
Chengbin Xiang
Jianhai Xiang
Ming Xiang
Shi-Hua Xiang
Yang Xiang
Ruidong Xiang
Wenhua Xiang
Shengyan Xiang
Binggang Xiang
Fang Xiang
Xiaoguo Xiang
Jianxing Xiang
Jim Xiang
Jianming Xiang
Kehui Xiang
Liming Xiang
Dehui Xiang
Wenpei Xiang
Fan Xiang
Sen Xiang
Lei Xiangdong
Wei Xiao
Xinshu Xiao
Yanghua Xiao
Wuhan Xiao
Xinhua Xiao
Guanghua Xiao
Ming Xiao
Yanni Xiao
Shu Xiao
Hui Xiao
Ningchuan Xiao
Zhi-Qiang Xiao
Haijun Xiao
Rui Xiao
Yue-Hua Xiao
Mang Xiao
Li Xiao
Yan Xiao
Meifang Xiao
Liping Xiao
Xiao Xiao
Shi Xiao
Bin Xiao
Yong Xiao
Xiaodong Xiao
Lanbo Xiao
Jiajie Xiao
Yandong Xiao
Shenyuan Xiao
Lehui Xiao
Yihong Xiao
Hongwei Xiao
Jianfeng Xiao
Shuo Xiao
Zhimin Xiao
Qiaobin Xiao
Junhua Xiao
Lihua Xiao
Mingming Xiao
Daliao Xiao
Fei Xiao
Zhijiao Xiao
Gong Xiaobao
Feng Xiaobo
Zhang Xiaojun
Ma Xiaoyin
Qi Xie
Zhi-Xun Xie
Keping Xie
Yang Xie
Zhongcong Xie
Wen Xie
Huirong Xie
Peng Xie
Jianping Xie
Ping Xie
Yuntao Xie
Guanhua Xie
Shulian Xie
Hua Xie

Grace Xie
Conghua Xie
Xueqian Xie
Xianzhi Xie
Ying Xie
Gang Xie
Dong Xie
Dan Xie
Lei Xie
Yaojie Xie
Mingxing Xie
Wen-Jie Xie
Jian-Jun Xie
Zhenping Xie
Na Xie
Fuwei Xie
Huijun Xie
Meng Xie
Huaqing Xie
Ting Xie
Jianfei Xie
Xin-Fang Xie
Yehua Xie
Yuning Xie
Yanping Xie
Yh Xie
Xianhong Xie
Tao Xie
Mingyi Xie
Chang-Gen Xie
Jia Xie
Weilong Xie
Yin Xiling
Maria Xilouri
Haiping Xin
Ruolei Xin
Yu Xin
Wei Xin
Sherman Xin
Chao Xing
Dajun Xing
Dongqi Xing
Tim Xing
Da Xing
Bengang Xing
Chengguo Xing
Chuanhua Xing
Zisheng Xing
Lei Xing
Jinliang Xing
Bo Xing
Hai Xing
Dingliang Xing
Qi Xing
Fei Xing
Jingrui Xing
Qiaorui Xing
Lianping Xing
Han Xing
Li Xingnan
He Xing-Xing
Sidong Xiong
Xu Xiong
Yulan Xiong
Lizhong Xiong
Yong Xiong
Wanfen Xiong
Liming Xiong
Ai-Sheng Xiong
Shunbin Xiong
Zhengqin Xiong
Jianyin Xiong
Xingjiang Xiong
Wen-Cheng Xiong
Xiong Xiong
Yuquan Xiong
Wei Xiong
Xiaorui Xiong
Bo Xiong
Colin Xiong
Fei Xiong
Wenyong Xiong
May Xiong
Peng Xiong
Xiwen Xiong
Zhiyong Xiong
Jinjun Xiong
Xinyu Xiong
Shigang Xiong
Li Xiong
Ye Xiong
Lize Xiong
Xinyu Xiong
Enric Xipell
Zhilong Xiu
Elena Xoplaki

Xiaolei Xu
Zuoshang Xu
Leyan Xu
Guilian Xu
Jichen Xu
Wenbo Xu
Haoxing Xu
Jin-Rong Xu
Guoheng Xu
Dongping Xu
Jian-Hong Xu
Aimin Xu
Chenwu Xu
Ting Xu
Xiaoyin Xu
Shun Xu
Haiyan Xu
Biao Xu
Yujia Xu
Jiannong Xu
Chonggang Xu
Yunyuan Xu
Xingzhi Xu
Haiming Xu
Huanbin Xu
Wu Xu
Chengfu Xu
Xiangming Xu
Qiang Xu
Songxiao Xu
Huaxi Xu
Peng Xu
Heping Xu
Bo Xu
Zhenzhu Xu
Yan Xu
Fan Xu
Duan Xu
Yuming Xu
Xiao-Ke Xu
Yidong Xu
Tian-Le Xu
Zhuwen Xu
Jian Xu
Xingshun Xu
Jianchu Xu
Guang-Yin Xu
Chun Xu
Beisi Xu
Pingyi Xu
Yihua Xu
Jiejie Xu
Xia Xu
Yong Xu
Fang Xu
Zhenghong Xu
Jialin Xu
Xiaohua Xu
Fen Xu
Ren-Ai Xu
Juan Xu
Zhi Xu
Huilei Xu
Yanming Xu
Weifeng Xu
Guoqiang Xu
Zhaohui Xu
Shiqing Xu
Yue Xu
Hongbin Xu
Wei Xu
Min Xu
Baoshan Xu
Pei Xu
Meifeng Xu
Zeng-Fu Xu
Damo Xu
Xiaolong Xu
Huanyu Xu
Weili Xu
Ming-Jiang Xu
Meiying Xu
Jiuping Xu
Baohua Xu
Ke Xu
Chang Xu
Zekuan Xu
Donghe Xu
Fuhua Xu
Rong Xu
Dan Xu
Cunlai Xu
Dingli Xu
Zhenghu Xu
Guihua Xu
Jiadi Xu

Baohui Xu
Yongjie Xu
Ying-Chun Xu
Wanpeng Xu
Xiaofeng Xu
Meng Xu
Ximeng Xu
Yuan Xu
Xinping Xu
Li Xu
Junjie Xu
Ting-Yan Xu
Shu Xu
Zheng Xu
Pengfei Xu
Yifan Xu
Fei Xu
Jia-Ping Xu
Jing Xu
Xuan Xu
Shaohua Xu
Mengsi Xu
Jianzhen Xu
Yun Xu
Xiaomeng Xu
Yin Xu
Jiru Xu
Ling Xu
Chengqi Xu
Benhua Xu
Hui Xu
Lizhen Xu
Wenqing Xu
Guang Xu
Elvis Xu
Hao Xu
Pingwen Xu
Yingjie Xu
Yiming Xu
Jun Xu
Yang Xu
Qiong Xu
Jianhua Xu
Kexiang Xu
Xiaoling Xu
Buyun Xu
Ximing Xu
Yanhui Xu
Zhiliang Xu
Lu Xu
Ye Xu
Dongrong Xu
Tengfei Xu
Feng Xu
Junqian Xu
Ri Xu
Chenfu Xu
Jie Xu
Jin Xu
Zhihui Xu
Shiyu Xu
Yiru Xu
Qi Xu
Tingting Xu
Bin Xu
Huichun Xu
Ren Xu
Vivian Xu
Weidong Xu
Pingxi Xu
Hai-Chuan Xu
Mengjia Xu
Xin-Fen Xu
Qingwen Xu
Kevin Xu
Di Xu
Liping Xu
Li-An Xu
Miao Xu
Genjiu Xu
Xiaojuan Xu
Hua Xu
Lin Xu
Kaiyuan Xu
Jingyue Xu
Chenyu Xu
Dake Xu
Yaozhan Xu
Dongying Xuan
Lixiang Xue
Ding Xue
Bin Xue
Chaoyang Xue
Yu Xue
Fuzhong Xue
Rong Xue

Dawei Xue
Yang Xue
Zhidong Xue
Junli Xue
Rui-De Xue
Da-Yong Xue
Liang Xue
Alexander Xue
Likun Xue
Allen Xue
Meilang Xue
Xiang Xue
Jianfei Xue
Hui Xue
Yingben Xue
Bingzhong Xue
Wufeng Xue
Qian-Li Xue
Dayuan Xue
Qingwu Xue
Xia Xueshan
Zijun Xu-Monette
Pengcheng Xun
Meng Xu-Welliver
Dimitris Xygalatas
Evaghelos Xynos
Beery Yaakov
Rami Yaari
Daisuke Yabe
Atsushi Yabe
Tomoyoshi Yabu
Katsumi Yabusaki
Jayne Yack
Rabi Yacoub
Talene Yacoubian
Toshihiko Yada
Prashant Yadav
Jagjit Yadav
Roopali Yadav
Sanjay Yadav
Ram Kishor Yadav
Neelu Yadav
Naresh Yadav
Nagendra Yadava
Pramod Yadava
Tejabhiram Yadavalli
Hamideh Yadegari
Liran Yadgary
Reza Yaesoubi
Hessameddin Yaghoobi
Hiroshi Yagi
Shigeo Yagi
Hideshi Yagi
Tetsuya Yagi
Pablo Yagupsky
Kazuhide Yahata
Timothy Yahr
Mahmoud Yaish
Mostafa Yakoot
Sergiy Yakovenko
Vladislav Yakovlev
Alexander Yakunin
Toshiharu Yakushi
Takayuki Yakushijin
Yavor Yalachkov
Arzu Didem Yalcin
Ozlem Yalcin
Ayfer Yalcin
Zhang Yali
W.C. Yam
Gary Hin-Fai Yam
Gary Yam
Eileen Yam
Jason Yam
Kenneth M Yamada
Tadaaki Yamada
Katsunori Yamada
Takuji Yamada
Kaori Yamada
Norikazu Yamada
Sohsuke Yamada
Tomomi Yamada
Yoshihito Yamada
Tomoya Yamada
Nicole Yamada
Miko Yamada
Sueli Yamada-Ogatta
Kazuya Yamagata
Kunihiro Yamagata
Zentaro Yamagata
Nobuhiro Yamagata
Bun Yamagata
Sho-Ichi Yamagishi
Hiroyuki Yamagishi
Kenji Yamagishi
Masakazu Yamagishi

Satoshi Yamagiwa
Nobuyuki Yamaguchi
Yoshiki Yamaguchi
Satoshi Yamaguchi
Atsuko Yamaguchi
Atsushi Yamaguchi
Ricardo Yamaguchi
Tomohiro Yamaguchi
Hiroyuki Yamaguchi
Kenichi Yamahara
Abir Yamak
Takeo Yamakawa
Yasuo Yamakoshi
Liliya Yamaleyeva
Fukashi Yamamichi
Shinya Yamamoto
Masayuki Yamamoto
Yasuhiko Yamamoto
Naohide Yamamoto
Masahiro Yamamoto
Kaneyoshi Yamamoto
Naomichi Yamamoto
Yoshiharu Yamamoto
Kei Yamamoto
S Yamamoto
Toshiyuki Yamamoto
Naoki Yamamoto
Mikihiro Yamamoto
Elichiro Yamamoto
Atsushi Yamamoto
Takeshi Yamamoto
Shuichiro Yamamoto
Tae Yamamoto
Haruko Yamamoto
Ryohei Yamamoto
Liria Yamamoto-Kimura
Ken-Ichi Yamamura
Masahiro Yamamura
Toshiro Yamanaka
Atsushi Yamanaka
Daisuke Yamane
Hisayo Yamane
Koji Yamanegi
Thespina Yamanis
Yoshihiro Yamanishi
Hiromichi Yamanishi
Keitaro Yamanouchi
Shoji Yamaoka
Masaya Yamaoka
Kunihiro Yamaoka
Sho Yamasaki
Takafumi Yamashino
Kenji Yamashiro
Tsuneo Yamashiro
Atsushi Yamashita
Masakatsu Yamashita
Takehiro Yamashita
Misuzu Yamashita
Kenichiro Yamashita
Haruyuki Yamashita
Nayuta Yamashita
Teruhito Yamashita
Hidenori Yamasue
Masahide Yamato
Koji Yamatsu
Takashi Yamauchi
Toshimasa Yamauchi
Akira Yamauchi
Kiyoshi Yamauchi
Hiroki Yamaue
Yuichi Yamaura
Hideyuki Yamawaki
Tomoyuki Yamaya
Takayoshi Yamaza
Yukiko Yamazaki
Sayuri Yamazaki
Hiroshi Yamazaki
Soh Yamazaki
Tadashi Yamazaki
Keiko Yamazaki
Jun Yamazaki
Gavin Yamey
Daniel Yamins
Sophie Yammine
Yukio Yamori
Lev Yampolsky
Hong Yan
Jun Yan
Chaogan Yan
Jie Yan
Yueming Yan
Xiaojun Yan
Jianbing Yan
Lily Yan
Qin Yan
Xiao-Xin Yan

Hongli Yan
Junqiang Yan
Guijun Yan
Jian Yan
Jing-Jou Yan
Jianhua Yan
Hao Yan
Shuangchun Yan
Qingfeng Yan
Fengming Yan
Shuicheng Yan
Tiebin Yan
Sen Yan
Robert Yan
Jin Yan
Ming Yan
Andrew Yan
Gang Yan
Bo Yan
C Yan
Lirong Yan
Pisong Yan
Yunjun Yan
Chengshi Yan
Huimin Yan
Zengguang Yan
Feng-Ming Yan
Beizhan Yan
Jiusheng Yan
Lei Yan
Ganxin Yan
Binyan Yan
Dayun Yan
Ji Yan
Yun Yan
Sun Yan
Beichun Yan
Hongjing (Holly) Yan
Qingyun Yan
Fang Yan
Fang (Alice) Yan
Xu Yan
Denise Yan
Elsa Yan
Weile Yan
Erjia Yan
Bowen Yan
Chao-Gan Yan
Qi Yan
Bin Yan
Junli Yan
Xian-Zhomg Yan
Yunrong Yan
Xiao-Yong Yan
Zhong-Qun Yan
Xinping Yan
Yue-Ming Yan
Heping Yan
K. Yanaba
Yuchio Yanagawa
Masahiro Yanagawa
Hiroshi Yanagawa
Angel Yanagihara
Dai Yanagihara
Kunio Yanagisawa
Ruth Yanai
Hideyuki Yanai
Kiran Yanamandra
Pattamawadee Yanatatsaneejit
Brian Yandell
Timothy Yandle
Oscar Yanes
Huiling Yang
Huan Yang
Xian-Jie Yang
George Yang
Xiang-Jiao Yang
Xi Yang
Samuel Yang
Zhong-Nan Yang
Yun-Liang Yang
Hanchun Yang
Xiaohan Yang
Jinn-Moon Yang
Rong-Cai Yang
Wanling Yang
Ning Yang
Wei Cai Yang
Tae-Jin Yang
Ming Yang
Zhong-Jin Yang
X. Frank Yang
Chonglin Yang
Hsin-Chou Yang
Wei-Shiung Yang
Qinglin Yang

Zhenglin Yang
Albert Yang
Wancai Yang
Jun Yang
Xiao-Ming Yang
Hannah Yang
Canchao Yang
Sheng Yang
Zhi Min Yang
Yuh-Shyong Yang
Jenny Yang
Ji Yang
Yinong Yang
Maojun Yang
Wan-Xi Yang
Runqing Yang
Jiongjiong Yang
Lin Yang
Wei Yuan Yang
Wei-Jun Yang
Baoxue Yang
Kehu Yang
Zeng-Ming Yang
Peng Yang
Bao Yang
Eddy Yang
Jian-Rong Yang
Zhe Yang
Yong Yang
Guangyou Yang
Kejian Yang
Wen-Chin Yang
Cheryl Yang
Li-Ye Yang
Jiang Yang
Yen Kuang Yang
Li Yang
Xiaobo Yang
Bin Yang
Shun-Fa Yang
Guang Yang
Xiuwei Yang
Tao Yang
Ding Yang
Fu-Sheng Yang
Peizeng Yang
Hushan Yang
Yimin Yang
Zhan-Qiu Yang
Jingchun Yang
Zhi Yang
Fanmuyi Yang
Qian Yang
Chih-Yu Yang
Zhang Yang
Jian Yang
Rong-Sen Yang
Kai Yang
Kuang-Yao Yang
Jun-Yi Yang
Junwei Yang
Zhen Yang
Xinrong Yang
Guangxiao Yang
Tsuey-Ching Yang
Hwai-I Yang
Wencai Yang
Jianyi Yang
Yan Yang
Cui Yang
Yanlian Yang
Yangfan Yang
Seungmi Yang
Hua Yang
Huijie Yang
Tsung-Lin Yang
Qintai Yang
Ping-Chang Yang
Tingzhong Yang
Hong Yang
Yang Yang
Juan Yang
Mu Yang
Can Yang
Zengjin Yang
Su-Wen Yang
Ying-Ying Yang
Wan Yang
Chengwei Yang
Yunzhi Yang
Lee-Wei Yang
Litao Yang
Song-Ran Yang
Ming-Chin Yang
Xueyun Yang
Min Yang

Dewei Yang
Shi Yang
Haifeng Yang
Jie Yang
Yeqing Yang
Wei Yang
Jianzhong Yang
Zhaogang Yang
Changqing Yang
Caiyun Yang
Hui Yang
Chaowei Yang
Zhangsheng Yang
Xiao-Jun Yang
Le Yang
Qingyong Yang
Peixin Yang
Guozheng Yang
Xiyan Yang
Meng Yang
Fan Yang
Jian-Jun Yang
Weiliang Yang
Jian-Hua Yang
Xin Yang
Shiling Yang
Liangjing Yang
Rendong Yang
Jigang Yang
Xiaoyuan Yang
Xiao Yang
Xiaoxu Yang
Xinghong Yang
Chin-Lung Yang
Yunfei Yang
Shanshan Yang
Xiao-Yang Yang
Shouhui Yang
Lifen Yang
Xiaoe Yang
Chongguang Yang
Sheng-Shun Yang
Dongshan Yang
Weili Yang
Jingxuan Yang
Chao-Yie Yang
Tewu Yang
Wentao Yang
Leixiang Yang
Lu Yang
Jianfeng Yang
Suyong Yang
Liang Yang
Wanqin Yang
Weihua Yang
Chengzhong Yang
Tai-Hua Yang
Zhi Hui Yang
Haibing Yang
Bo Yang
Sejung Yang
Guo-Yuan Yang
Fu-Hua Yang
Shang-Hsun Yang
Zhenyu Yang
Jer-Yen Yang
Chen-Chang Yang
Ping Yang
Wenjia Yang
Qiaoyan Yang
Chuanwei Yang
Hanxin Yang
Chung-Yi Yang
Yonghui Yang
Sungho Yang
Ying Yang
Shaolin Yang
Dun Sheng Yang
Xiaofang Yang
Huang-Hao Yang
Jing Yang
Xiaozhao Yousef Yang
Miyoung Yang
Yungnane Yang
Qian Yang Yang
Woong Mo Yang
Ling Yang
Claire Yang
Jian Li Yang
Yun Yang
Wensha Yang
Ding-I Yang
Chengwu Yang
Yanjiang Yang
Sheng-Xiang Yang
Yi Yang

Bing Yang Yang
Chinhua Yang
Rongchang Yang
Wen-Ju Yang
Xu Yang
Yao Yang
Haoshu Yang
Xuguang Yang
Yao-Hsu Yang
Zhaoqing Yang
Zhihui Yang
Wenlong Yang
Shunkun Yang
Xiaomei Yang
Zheng-Han Yang
Zhongzhou Yang
Shawn Yang
Xiaoli Yang
Nuo Yang
Decheng Yang
Chul-Su Yang
Jingyi Yang
Xiao-Quan Yang
Shengping Yang
Jae-Suk Yang
Qifeng Yang
Chien-Chih Yang
Xiaolong Yang
Fuqian Yang
Ya-Nan Yang
Dapeng Yang
Shihui Yang
Binxia Yang
Yanfei Yang
Shuman Yang
Feng Yang
Hai Yang
Shwu-Huey Yang
Yalian Yang
Shibing Yang
Wenli Yang
Su Yang
Yaping Yang
Jilong Yang
Yamin Yang
Roman Yangarber
Emilio Yanguez
Elizabeth Yanik
Pam Yankeelov
Anthony Yannarell
Joseph Yanni Gerges
Yuichiro Yano
Shuya Yano
Marcelo Yanovsky
Fei Yao
Wei Yao
Li-Bo Yao
Quan-Hong Yao
Qin Yao
Zhirong Yao
Yin Yao
Zhenqiang Yao
Song Yao
Jianbo Yao
Yucheng Yao
Chun-Hsu Yao
Yi-Feng Yao
Yu-Feng Yao
Zhong-Xiang Yao
Yingyin Yao
Xiaojun Yao
Shuqiao Yao
Yao Yao
Yong-Gang Yao
Zhenjiang Yao
Shanguo Yao
Yi Yao
Linong Yao
Nengliang Yao
Yuan Yao
Shuyu Yao
Bing Yao
Qiuming Yao
Jianxiu Yao
Katharine Yao
Minjie Yao
Kai Yao
Jia Yao
Junjie Yao
Kai-Ping Yao
Zhi-Qiang Yao
Yu Yao
Shuhuai Yao
Yixin Yao
Xi Yao
Mei-Cun Yao

Zhi Yao
Lin Yao
Lixia Yao
Yanhua Yao
Hui Yao
Hongwei Yao
Philip Yap
Yit-Sheung Yap
Pew-Thian Yap
Manisha Yapa
Cerer Yarar-Fisher
Kevin Yarasheski
William Yarber
Ceren Yardimci
Jane Yardley
Neil Yarlett
Marianna Yaron
Viktor Yarotskyy
Felix Yarovinsky
Kielan Yarrow
Stephen Yarwood
Yoko Yashiroda
Taha Yasseri
Aymen Yassin
Mônica Yassuda
Takanori Yasu
Takashi Yasuda
Shinsuke Yasuda
Hideto Yasuda
Yoshinari Yasuda
Satoshi Yasuda
Kazuhiro Yasufuku
Takao Yasuhara
Dag Yasui
Shin Yasui
Hiroe Yasui
Masaki Yasukawa
Yoshiki Yasukochi
Akira Yasumura
Tetsuzo Yasunari
Hidekata Yasuoka
Akihito Yasuoka
Paul Yaswen
Phillip Yates
Clayton Yates
Charles Yates
Patsy Yates
Yutaka Yatomi
Alexander Yatsenko
Keisuke Yatsu
Hiroshi Yatsuya
Stephen Yau
Jeffrey Yau
Rebecca Yau
Carole Yauk
Dileep Yavagal
Arash Yavari
Joseph Yavitt
Stan Yavno
Sukru Yavuz
Barbara Yawn
Hiromu Yawo
Yaliso Yaya
Josef Yayan
Takuya Yazawa
Daniel Constantino Yazbek
Gustavo Ybazeta
Patricia Ybot
Kristina Ydesen
Yuanqing Ye
Yuzhen Ye
Gong-Yin Ye
Ping Ye
Wen Ye
Dongqing Ye
Yihong Ye
Hong Ye
Lin Ye
Bing Ye
Xiaoqin Ye
Fengchun Ye
Kenny Ye
Xing Ye
Ling Ye
Yongquan Ye
Li Ye
Yuxiang Ye
Xingguo Ye
Weimin Ye
Duo Ye
Haihui Ye
Yinghui Ye
Jianping Ye
Wenwu Ye
Nenghui Ye
Huijing Ye

Jianqiang Ye
Yu Ye
Min Ye
Jiangfeng Ye
Wenling Ye
Cong Ye
Rongzhong Ye
Shengquan Ye
Fengchung Ye
Zhenqiang Ye
Aizhong Ye
Samuel Yearman
Todd Yeates
Karen Yeates
Sara Yeatman
Lidia Yebra
Venkat Yedavalli
Narayana Yeddula
Amy S Yee
Nelson Yee
Albert Yee
Jerry Yee
Donald Yee
Wee Yee
Brendon Yee
Douglas Yee
Behzad Yeganeh
Chih-Jung Yeh
Trai-Ming Yeh
Shuyuan Yeh
Tien-Shun Yeh
Shiou-Hwei Yeh
Shyi-Dong Yeh
Chau-Ting Yeh
Kuo-Chen Yeh
Yao-Tsung Yeh
Te-Huei Yeh
Ting-Ting Yeh
Fang-Cheng Yeh
Ming-Lun Yeh
Ching-Hui Yeh
Yi-Chun Yeh
Ting-Chun Yeh
Shauh-Der Yeh
Chuan-Ming Yeh
Lo-Yao Yeh
Kuo-Wei Yeh
Chi-Tai Yeh
Oren Yehezkel
Karen Yehle
Samantha Yeligar
Alexei Yeliseev
Derek Yellon
Peter Yellowlees
Dawit Yemane
Denys Yemshanov
Tzu-Chen Yen
Rouh-Fang Yen
Jh Yen
Hsueh-Chi Yen
Frances Yen
Nai-Shing Yen
Chueh-Chuan Yen
David H.T. Yen
Jui-Hung Yen
Midori Yenari
Craig Yencho
Christopher Yengo
Yener Yeni
Ragothaman Yennamalli
Jenna Yentes
Lynne Yenush
Lami Yeo
George Yeoh
Young Yeom
Mark Yeoman
Carl Yeoman
Yoon Keng Yeong
Manuel Yepes
Edward Yepes
Helene Yera
Justin Yerbury
Chahan Yeretzian
Stephanie Yerkovich
Martine Yerle
Daniel Yerly
Venkat Yeruva
Yaara Yeshurun
Ertan Yetkin
Ozkan Yetkin
Edwina Yeung
Sai-Ching Yeung
Henny Yeung
Kelvin Yeung
David Yeung
David Yew

Kuan Yew
Jonathan Yewdell
Benjamin Yguel
Nengjun Yi
Siyan Yi
Fan Yi
Richard Yi
Xianghua Yi
Hyunmin Yi
Bin Yi
Hongliang Yi
Jing Yi
Wei Yi
Long Yi
Muqing Yi
Sha Yi
Zhuang-Fang Yi
Joo Mi Yi
Joyce Yi-Frazier
Wonho Yih
Eda Yildirim
Ali Önder Yildirim
Ahmet Yildiz
Hakki Yilmaz
Ali Yilmaz
Burak Yilmaz
Kevin Yim
Yin Yiming
Yanbin Yin
Yongjun Yin
Huabing Yin
De-Tao Yin
Lei Yin
Yulong Yin
Dwight Yin
Lu Yin
Hengfu Yin
Changjun Yin
Min-Jean Yin
Haifang Yin
Hsien-Sheng Yin
Ping Yin
Shi-An Yin
Fei Yin
Junjun Yin
Xinhua Yin
Guohua Yin
Xue-Ren Yin
Ye Yin
Mingbo Yin
Jun Yin
Shanye Yin
Dongmin Yin
Fukang Yin
Chenghong Yin
Huiyong Yin
Cameron Yin
Yiming Yin
Huaqun Yin
Zheng Yin
Hongbin Yin
Xi-Jun Yin
Hua-Bin Yin
Erwei Yin
Louis-Marie Yindom
Hao Ying
Sun Ying
Leslie Ying
Hanjie Ying
Qu Ying
Shihui Ying
Tianlei Ying
Wen Ying
Jianming Ying
Liuhua Ying
Charilaos Yiotis
Paul Yip
Timothy Yip
Kevin Yuk-Lap Yip
Peter Yip
Cyril Yip
Tsz Leung Yip
Joel Yisraeli
Kai Hang Yiu
Niu Yiyu
Jari Ylänne
Arne Yndestad
Helger Yntema
Nigel Yoccoz
Zachary Yochum,
Torunn Yock
Charles Yocum
George Yocum
Jonathan Yoder
Kinfu Yohannes
Marielle Yohe

Mehmet Baki Yokes
Shoji Yokobori
Aki Yokohama
Kohei Yokoi
Hirohide Yokokawa
Ray Yokomi
Hiroshi Yokomichi
Hiroyuki Yokomizo
Yasuyuki Yokosaki
Takafumi Yokota
Toshifumi Yokota
Koutaro Yokote
Akira Yokoyama
Yukari Yokoyama
Kazunari Yokoyama
Yusuke Yokoyama
Ryusuke Yokoyama
Hitoshi Yokoyama
Keitaro Yokoyama
Sonja Yokum
Robert Yolken
Yoram Yom-Tov
Elad Yom-Tov
Lisa Yon
Mark Yondola
Yukio Yoneda
Makoto Yoneda
Shigenobu Yonemura
Seiichiro Yonemura
Kinuyo Yoneya
Mitsutoshi Yoneyama
Atsushi Yonezawa
Naoto Yonezawa
V. Wee Yong
Hoi-Sen Yong
Zhang Yong Gang
Liang Yongchao
Zhu Yongjun
Qin Yongmei
Puangrat Yongvanit
Kimberly Yonkers
Wan Hee Yoo
Han Sang Yoo
Wonsuk Yoo
Yun Joo Yoo
Dongwan Yoo
Hye Hyun Yoo
Sang-Dong Yoo
Changwon Yoo
Kathryn Yoo
Jung Sun Yoo
Robert Yood
Karina Yoon
Byung-Jun Yoon
Joo Chun Yoon
Geunyoung Yoon
Jeong Kyo Yoon
Sung Ok Yoon
Seung Kew Yoon
Ki Tae Yoon
Yeonyee Yoon
Hye Eun Yoon
Michael Yoon
Sung-Il Yoon
Sungwon Yoon
Hyun-Sun Yoon
A-Rum Yoon
Youngdae Yoon
Aerin Yoon
Chungsik Yoon
Yeong Sook Yoon
Kara Yopak
Daniel Yorgov
Takashi Yorifuji
Timothy York
Andrew York
Michele K. York
Ian York
Manabu Yoshida
Ryusuke Yoshida
Hiroki Yoshida
Akihiko Yoshida
Satoshi Yoshida
Akihiro Yoshida
Kumi Yoshida
Takuya Yoshida
Shigeto Yoshida
Lay-Myint Yoshida
Honami Yoshida
Hiroshi Yoshida
Toshimi Yoshida
Nobuya Yoshida
Akira Yoshida
Makoto Yoshida
Yoshio Yoshida
Kentaro Yoshida

Naohisa Yoshida
Hajime Yoshifuji
Kosuke Yoshihara
Fukuda Yoshiharu
Tohru Yoshihisa
Takahiro Yoshikawa
Kazuhiro Yoshikawa
Eisho Yoshikawa
Takeshi Yoshikawa
Michiyasu Yoshikuni
Kazuto Yoshimi
Takayuki Yoshimoto
Koji Yoshimoto
Teizo Yoshimura
Reiji Yoshimura
Yumiko Yoshimura
Koichi Yoshimura
Michihiro Yoshimura
Kiyoshi Yoshimura
Elisabeth Yoshimura
Natsue Yoshimura
Kouichi Yoshinari
Masao Yoshinari
Osamu Yoshino
Yasuhide Yoshitake
Shinpei Yoshitke
Kohei Yoshiyama
Susumu Yoshizawa
Ofer Yossepowitch
Christian Yost
Ihor Yosypiv
Karla Yotoko
Hiroshi Yotsuyanagi
Raquel Yotti
Sylvaine You
John You
Weiwei You
Minsheng You
Yaqi You
Youping You
Jinsheng You
Feng You
Chun You
Hyewon Youn
Seock-Won Youn
Mousa Younesi
Paul Young
Wise Young
Stephen Young
Pampee Young
Vincent Young
Carolyn Young
Sera Young
Jared Young
Craig Young
Marian Young
Jason Young
Terri Young
Neil Young
Heather Young
Peter Young
William Young
Kenneth Young
Matthew Young
Shuenn-Tsong Young
Jane Young
Fraser Young
Nicholas Young
Anna Young
Yuan-Nan Young
Darwin Young
Joy Young
Mary Young
Sean Young
Kelly Young
Steven Young
Kymberly Young
Andrew Young
Won-Bin Young
Brent Young
Richard Young
Jesse Young
Kyana Young
G Bryan Young
Douglas Young
Karen C Young
Alexandra Young
Jette Young
Gene Young Cho
Nick Youngblut
Jack Youngren
Curtis Youngs
Heather Youngs
Zobair Younossi
Jacob Yount
Anam Yousaf

Balal Yousaf
Aisha Yousafzai
Aa Yousef
Reza Yousefi
Ramin Yousefi
Reza Yousefi-Nooraie
John Youson
Samar Youssef
Patrick Youssef
Seema Yousuf
Jan Youtie
Lidia Yshii
Renate Ysseldyk
Hua Yu
Clare Yu
Eugene Yu
Xue-Jie Yu
Qiang Yu
Jieh-Juen Yu
Hon-Tsen Yu
Haung Yu
Jun Yu
Hengxiu Yu
Dianke Yu
Zhongtang Yu
Sung-Liang Yu
Jian Yu
Sibin Yu
Xiaochun Yu
Luis Yu
Jia-Feng Yu
Huang-Ping Yu
Fei-Hai Yu
Haining Yu
Jiujiang Yu
Ming-Lung Yu
Tianwei Yu
Lei Yu
Shixiao Yu
Hongwei Yu
Jingquan Yu
Bo Yu
Fangyou Yu
Run Yu
Shengqing Yu
Shan Ping Yu
Diqiu Yu
Qingbao Yu
Dao-Yi Yu
Guanghui Yu
Qingzhao Yu
Ziniu Yu
Lijian Yu
Deyue Yu
Jia Yu
Weiqun Yu
Jingjuan Yu
Alan Yu
Peirong Yu
Jinpu Yu
Shengyuan Yu
Jae-Ran Yu
Xue Yu
Teng Yu
Tianxin Yu
Haiyang Yu
Yan Yu
Shanlin Yu
Guichuan Yu
Min Yu
Jack Yu
Ming-Jiun Yu
Yao Yu
Zhiqiang Yu
Xiaodan Yu
Minbin Yu
Mei Yu
Ying Yu
Yi Yu
Zhiwen Yu
Hsin-Hui Yu
Dong-Jun Yu
Guanzhen Yu
Yan Hong Yu
Qiujun Yu
Xue-Zhong Yu
Danxia Yu
Yunfang Yu
Geng Yu
En-Da Yu
Hailiang Yu
A-Yong Yu
Chang Yu
Xiao-Nan Yu
Yong Yu
Guihai Yu

Xiangyang Yu
Jin-Tai Yu
Jin Yu
Ling Yu
Kai Yu
Fei Yu
Changhe Yu
John Yu
Xiezhi Yu
Zitong Yu
Hongmei Yu
Ren-Cheng Yu
Dongmei Yu
Songlin Yu
Haihan Yu
Hong Yu
Pengtao Yu
Zhibin Yu
Pengchun Yu
Bin Yu
Shudong Yu
Futong Yu
Su Jong Yu
Doris Yu
Wayne Yu
Guozhong Yu
Hui Yu
Li Yu
François Yu
Zhiguo Yu
Wenqiang Yu
Yen-Rei Yu
Ming Yu
Fudong Yu
Jiaguo Yu
Shiyuan Yu
Kristine Yu
Rwei-Ling Yu
Xiaohua Yu
Shuli Yu
Sandy Yu
James Yu
Bing Yu
Jen-Shiang Yu
Fang-Fang Yu
Long Yu
Jane Yu
Hongjie Yu
Guoqiang Yu
Ke Yu
Bei Yu
Jinhua Yu
Herbert Yu
Chenggong Yu
Sw Yu
Miao Yu
Yan Yuan
Yunfei Yuan
Shu Yuan
Jing Yuan
Lijuan Yuan
Z.Y. Yuan
Shishan Yuan
Yinyin Yuan
Weiming Yuan
Youlu Yuan
Yuan Yuan
Zengqiang Yuan
Ping Yuan
Yonggui Yuan
Lixing Yuan
Wei Yuan
Chungshin Yuan
Kai Yuan
Pu-Qing Yuan
Wu-Jie Yuan
Jianhui Yuan
Baohong Yuan
Tifei Yuan
Songtao Yuan
Jin Yuan
Jing Yuan
Shuiqiao Yuan
Zhiguo Yuan
Hong Yuan
Yifu Yuan
Sanling Yuan
Kebin Yuan
Tiezheng Yuan
Li Yuan
Jingsong Yuan
Shi-Min Yuan
Jiazheng Yuan
Xueli Yuan
Zheng Yuan
Yang Yuan

Yuana Yuana
Hu Yuanyi
Eiji Yuba
Recai Yucel
Huang Yu-Chu
Jianbo Yue
Weihua Yue
Xinping Yue
Yun Yue
Min Yue
Binglin Yue
Xiaoshan Yue
Jun Yue
Jinxing Yue
Yaqing Yue
Hong Yue
Tianli Yue
Darren Yuen
Eric Hiu-Fung Yuen
Peter Yuen
Karen Wing Yee Yuen
Ryan Yuen
Sung Yuh
Shao Yu-Hsuan Joni
Hirose Yuichi
Michi Yukawa
Junichi Yukawa
Takuo Yuki
Koichi Yuki
Gary A. Yukl
Hasan Yuksel
David Yule
Chang-Hyon Chris Yun
Byung-Wook Yun
Sung-Chul Yun
Kyeong Ho Yun
Joonkoo Yun
Katherine Yun
Eun Ju Yun
Jose Andres Yunes
José Yunes
Hong-Wa Yung
Mingo Yung
Edmond Yunis
Emran Bin Yunus
Fakir Yunus
Isa Yunusa
Theodore Yuo
S Yurgel
Elizabeth Yuriev
Andrey Yurkov
Anna Yusa
Khatijah Yusoff
Suraya Yusoff
Jose Yuste
Victor Yuste
Jason Yustein
Nabiha Yusuf
Mohammad Yusuf
Mohd Yusuf
Timur Yusufzai
Takahashi Yusuke
Li Yu-You
Frank Zaal
Wafaa Zaaraoui
Monika Zaba
Gracia Zabala
Iñigo Zabalgogeazcoa
Angel Zaballos
María Celina Zabaloy
Brian Zabel
Alex Zabeo
Piotr Zabielski
Tanja Zabka
Martin Zabka
Lydia Zablotska
Iryna Zablotska
Vitalii Zablotskii
Lorenzo Zacarias
Luciana Zaccagni
Gaetano Zaccara
Francesco Zaccardi
Mauro Zaccarelli
Serena Zacchigna
Federica Zacchini
Evelyn Zacharewicz
Philip Zachariah
Martin Zacharias
Margaret Zacharin
Venetia Zachariou
Kai Zacharowski
Hannes Zacher
Christin Zachow
Shawn Zack
David Zacks
Eldad Zacksenhaus

Miriam Zacksenhouse
Sima Zadeh
Gelareh Zadeh
Cindy Zadikoff
Karla Zadnik
Pauline Zaenker
Zafar Zafari
Mirko Zaffagnini
Nadia Zaffaroni
Stéphane Zaffran
Alessandro Zagatto
Richard Zager
Kareem Zaghloul
Miriam Zago
Ian Zagon
Brandon Zagorski
Ana-Maria Zagrean
Jean-Ralph Zahar
Gerasimos Zaharatos
Greg Zaharchuk
Eran Zahavy
Rakan Zahawi
René Zahedi
Hossein Zahedi
Asgar Zaheer
Ahmad Zaheer
Reza Zahiri
Jeffrey Zahn
Astrid Zahn
Caroline Zahn-Waxler
Ahmad Zahoor
Stephen Zahorian
Pavel Zahorik
Natalie Zahr
Peter Zahradka
Clement Zai
Ronen Zaidel-Bar
Qasim Zaidi
Syed Zaidi
Noah Zaitlen
Beryl Zaitlin
Anne Zajac
Maria Zajac-Kaye
Anna Zajacova
Urszula Zajaczkowska
Dirk Zajonc
Nadia Zakaria
Issa Zakeri
Alex Zakhartchouk
Sherif Zaki
Sanaa Zaki
Suren Zakian
Harold Zakon
Julia Zakrzewski
Samer Zaky
Ahmed Zaky
Konstantine Zakzanis
Tomasz Zal
Juan Zalapa
Polona Zalar
John Zalcberg
Nickolas Zaller
Remi Zallot
Ilana Zalmon
Michal Zalzman
Raiyan Zaman
Hasniza Zaman Huri
Roham Zamanian
Laura Zamarian
Brian Zamarron
Federico Zambelli
Tomaso Zambelli
Elias Zambidis
Joao Zambon
Paolo Zambonelli
Giuseppe Zamboni
Fausto Zamboni
Carlos Zambrana-Torrelio
Cristian Zambrano
Luiz Roberto Zamith
Christian Zammit
Regino Zamora
Pilar Zamora
Rosario Zamora
Francisco Javier Zamora-Camacho
Paola Zamparo
Fernando Zampieri
Stefania Zampieri
Gerald Zamponi
Roxana Zamudio
Antonio Zamuner
Yunlong Zan
Giulio Zanaroli
Elodie Zana-Taieb
Alberto Zanchetti
Thomas Zander

Thorsten Zander
Esther Zander
Keivan Zandi
Gisele Zandman-Goddard
Peter Zandstra
Cleslei Zanelli
Damián Zanette
Maurizio Zanetti
Michela Zanetti
Ermellina Zanetti
Maria Eugenia Zanetti
Caterina Zanetti
Maria Helena Zanettini
Laura Zanetti-Polzi
Chuanli Zang
Qun Zang
Shaoyun Zang
Jochen Zange
Ulrich Zanger
Thomas Zangle
Benjamin Zaniello
Elisa Zanier
Dott Stefano Zanigni
Martina Zaninotto
Giovanni Zanionotto
George Zanjani
Johannes Zanker
Alessandra Zannella
Markella Zanni
Emanuela Zannin
Lindsay Zanno
Gian Franco Zannoni
Davide Zannoni
Clement Zanolli
Ivan Zanoni
Erika Zanoni
Edmar Zanoteli
Giuseppe Zanotti
Ilaria Zanotti
Alfeu Zanotto-Filho
Denise Zantut-Wittmann
Gianluigi Zanusso
Pat Zanzonico
Mikolaj Zapalski
Uriel Zapata
Heidi J Zapata
Lauren Zapata
Víctor Zapata
Maria Belen Zapata Diomedi
Colby Zaph
Lucia Zappalà
Vincenzo Zara
Severino Zara
Laure-Emmanuelle Zaragosi
Oscar Zaragoza
Hassan Zaraket
Fadi Zaraket
Patrizia Zaramella
Alexander Zaranek
Apostolos Zaravinos
Peter Zarb
Marco Zarbin
Alexander Zarbock
Martha Zarco-Gonzalez
Pauline Zardo
Rafael Zardoya
Gernot Zarfel
Gregory Zaric
Jose Zariffa
John Zarifis
Nahid Zarifsanaiey
Yair Zarmi
Rasa Zarnegar
Daniela Zarnescu
Phoebe Zarnetske
Charles Zaroff
Paul Zarogoulidis
Mohammad Zarrabian
Sofia Zarraga
Raffaele Zarrilli
Amir Zarrinpar
Juan Zarruk
Kristof Zarschler
Jan Zarzycki
Inga Zasada
Romi Zäske
Alon Zaslaver
Alexander Zaslavsky
Boris Zaslavsky
Michael Zasloff
Jennifer Zaspel
Natalia Zatakaeva
Maria Chiara Zatelli
Michal Zaton
Vladimir Zatsiorsky
Mayana Zatz

Roberto Zatz
Frank Zaucke
Michael Zaugg
Egija Zaura
Daniele Zavagno
Baltazar Zavala
Barbara Zavan
Pablo Zavattieri
Eliska Zaveska
Monika Zavodna
Anna Zavodni
Adam Zawada
Laurie Zawertailo
Rosa Zayas
Ena Zayas
Andrey Zaytsev
Gianluigi Zaza
Cecilia Zazueta
Antoine Zazzo
Dimitrios Zbainos
Maciej Zborowski
Blazej Zbytek
Anselm Zdebik
Stephen Zdedric
Theodore Zderic
M. Cecelia Zea
Leslie Zebrowitz
Luigi Zecca
Astrid Zech
Ulrich Zechner
Christoph Zechner
Guenther Zeck
Melinda Zeder
Tiffany Zee
Hajo Zeeb
Benjamin Zeeb
Michael Zegans
Gianguglielmo Zehender
Grigor Zehirov
James L Zehnder
Ashley Zehnder
Susanne Zehner
E. Paul Zehr
Amer Zeidan
Asad Zeidan
Henning Zeidler
Joshua Zeidner
Steven F. Zeigler
Daniel Zeigler
Susanne Zeilinger
Mirjam Zeisel
Robert Zeiser
Axel Zeitler
Scott Zeitlin
Benjamin Zeitlin
Jennifer Zeitlin
Julia Zeitlinger
Christina Zeitz
Fjoralba Zeka
Amir Zeki
Christina Zelano
Teresa Zelante
Howard Zelaznik
Adrian Zelazny
Noam Zelcer
John Zeldis
Andrew Zele
Darla Zelenitsky
John Zelenski
David Zelený
Blanka Zelezna
Mary Zelinski
David Zella
Mark Zeller
Tanja Zeller
Jp Zellweger
Jonathan Zelner
Yuval Zelnik
Lori Zeltser
Cedric Zeltz
Nathan Zelyas
Rachel Zemans
Michal Zembala
Sergio Zeme
Rostislav Zemek
Erika Zemkova
Niklaus Zemp
Elisabeth Zemp
Asako Zempo-Miyaki
Alexander Zenchuk
Ivan Zendejas
Takeshi Zendo
Dali Zeng
Lingxia Zeng
Lingfang Zeng
Musheng Zeng

Huihui Zeng
Qing-Yin Zeng
Mu-Sheng Zeng
Changqing Zeng
Lin Zeng
Chunhua Zeng
De-Hui Zeng
Xiao Han Zeng
Gang Zeng
Su Zeng
Yu Zeng
Jin Zeng
Quan Zeng
Li Zeng
Xuhui Zeng
Linghe Zeng
Shaohua Zeng
Dexing Zeng
Zhigang Zeng
Haibo Zeng
Yanru Zeng
Bo Zeng
Xiaofeng Zeng
Wei Zeng
Zhenzhong Zeng
Fanrong Zeng
Xuemei Zeng
An Zeng
Ping Zeng
Hui Zeng
Yiwen Zeng
Hao Zeng
Yong Zeng
Hou Qing Zeng
Jianbin Zeng
Hongwu Zeng
Ling-Li Zeng
Ming Zeng
Olga Zeni
Hector Zenil
Daniel Zenklusen
Wolfgang Zenk-Möltgen
Alexandre Zénon
Zenon Zenonos
Juergen Zentek
R. Zenteno-Cuevas
Karen Zentgraf
Lorena Zentilin
Tony Zera
Jennifer Zerbato
Didier Zerbib
Paolo Zerbinati
Alex Zerbini
Luiz Zerbini
Gianpaolo Zerbini
Marcelo Zerillo
Melinda Zeron Mullins
Inga Zerr
Georgios Zervakis
Eleftherios Zervas
Katharina Zeschky
Eva-Maria Zetsche
Henrik Zetterberg
Ingo Zettler
Holger Zetzsche
Andre Zeug
Victor Zevallos
Massimo Zeviani
Jason Zevin
Maximilian Zeyda
Emil Zeynalov
Shan Zha
Jun-Wen Zhai
Chuangyan Zhai
Lingling Zhai
R. Zhai
Ping Zhai
Weiwei Zhai
Wei Zhai
Shuai Zhan
Yougen Zhan
Xiaowei Zhan
Ji-Cheng Zhan
Ming Zhan
Xiuxiu Zhan
Xi Zhan
Junpeng Zhan
Xuanzhi Zhan
Weiguo Zhang
Shicui Zhang
Byoung-Tak Zhang
Ying Zhang
Shaowu Zhang
Heping Zhang
Yun Zhang
Yiyue Zhang

Chen-Yu Zhang
Chun-Ting Zhang
Junfeng Zhang
Zhibin Zhang
Xubing Zhang
Yingze Zhang
Wenli Zhang
Huiping Zhang
Wenping Zhang
Yan Zhang
Bo Zhang
Dapeng Zhang
Yong Zhang
Aimin Zhang
Yuan-Ming Zhang
Kezhong Zhang
Yong-Yuan Zhang
Zhiqiang Zhang
Lei Zhang
Guang-Xian Zhang
Zhong-Yin Zhang
Ziding Zhang
Wan-Ke Zhang
Jin-Song Zhang
Kai Zhang
Hong-Yu Zhang
Jingqiang Zhang
Xiquan Zhang
Jianyi Zhang
Minghua Zhang
Liangfang Zhang
Xiaobo Zhang
Zhifang Zhang
Wen-Yi Zhang
Mu Zhang
Wei Zhang
Libiao Zhang
Xinli Zhang
Jie Zhang
Tao Zhang
Henggui Zhang
Zi-Ke Zhang
Deqiang Zhang
Jianying Zhang
Jiacai Zhang
Yongjie Zhang
Zheng Gang Zhang
Haili Zhang
Yu Zhang
Chuan-Xi Zhang
Rugang Zhang
Yuanwei Zhang
Rongqing Zhang
Yiying Zhang
Jifeng Zhang
Suping Zhang
Xuehong Zhang
Alice Zhang
Xiao-Kun Zhang
Yanyun Zhang
Guoping Zhang
Cheng-Cai Zhang
Yi-Cheng Zhang
Runzhi Zhang
Keqiang Zhang
Xiaojun Zhang
Junhua Zhang
Hong-Xia Zhang
Aihua Zhang
Hua Zhang
Jue Zhang
Chen Zhang
Guoquan Zhang
Junbin Zhang
Ru Zhang
Jian Zhang
Qiwei Zhang
Zhigang Zhang
Ming Zhang
Ben Zhang
Hong-Tao Zhang
Zhuoli Zhang
Xiangrong Zhang
John Zhang
Lin Zhang
Weili Zhang
Yang Zhang
Licai Zhang
Han-Ting Zhang
Lan Zhang
Han Zhang
Xiaotun Zhang
Chi Zhang
Zg Zhang
Li Zhang
Yangsong Zhang

Jing Zhang
Tiejun Zhang
Yi-Zheng Zhang
Ximei Zhang
Ming-Zhi Zhang
Ping Zhang
Mingyong Zhang
Yuqing Zhang
Lu Zhang
Yan-Zhou Zhang
Ru-Xin Zhang
Shougong Zhang
Sheng Zhang
Kam Zhang
Mingsha Zhang
Daoqiang Zhang
Tingting Zhang
Zuxin Zhang
Hailin Zhang
Qingpeng Zhang
Yi Zhang
Zhi-Gang Zhang
Dai Zhang
Lianyang Zhang
Xiaoke Zhang
Jiangyang Zhang
Ming-Rong Zhang
Mingcai Zhang
Haijun Zhang
Haifeng Zhang
Yanqiao Zhang
Guo-Jun Zhang
Yong-Mei Zhang
Chao Zhang
Guo-Qiang Zhang
Ming-Li Zhang
Zhengsheng Zhang
Mingce Zhang
Kunyan Zhang
Zichao Zhang
Yong-Liang Zhang
Yin-Gang Zhang
Wujie Zhang
Hongsheng Zhang
Jianguo Zhang
Jingyu Zhang
Hongbing Zhang
Xiurong Zhang
Jinping Zhang
Qianru Zhang
Wensheng Zhang
Huidan Zhang
Zhan Zhang
Chun Zhang
Bin Zhang
Mei Zhang
Guofan Zhang
Xizhe Zhang
Zhanyuan Zhang
Yiqiang Zhang
Jun Zhang
Yaqing Zhang
Zhao Zhang
Heye Zhang
Zhijian Zhang
Yuanyuan Zhang
Xu-Xiang Zhang
Weimin Zhang
Yunhui Zhang
Xiaoling Zhang
Shaoting Zhang
Ai-Hong Zhang
Boyu Zhang
Jinzhong Zhang
Qu Zhang
Kangling Zhang
Xiao Zhang
Yangjian Zhang
De-Liang Zhang
Haibin Zhang
Chunhua Zhang
Naixia Zhang
Zong-De Zhang
Aijun Zhang
Geoffrey Zhang
Youjun Zhang
Lingjiao Zhang
Yuxiang Zhang
Yunchun Zhang
Yanfeng Zhang
Chunxiang Zhang
Yansheng Zhang
Feng Zhang
Yue Zhang
Xinping Zhang
Guangjun Zhang

Chunyu Zhang
Zhuo Zhang
Xianzhou Zhang
Xiangqi Zhang
Liyi Zhang
Hongbo Zhang
Yongliang Zhang
Dingxiao Zhang
Lijuan Zhang
Sarah X. Zhang
Long Jiang Zhang
Liangsheng Zhang
Lubo Zhang
Xianlong Zhang
Guangming Zhang
Yin Hua Zhang
Xiaohua Zhang
Bing Zhang
Xiuying Zhang
Rui Zhang
Rongxin Zhang
Shi-Bao Zhang
Hongliang Zhang
Jiangwen Zhang
Hao Zhang
Kebin Zhang
Quanqi Zhang
Suzhi Zhang
Qingxue Zhang
Deng-Hai Zhang
Peng Zhang
Ling Zhang
Yonggang Zhang
Xiying Zhang
Ji Zhang
Jinsong Zhang
Zhaojie Zhang
Yong-Jun Zhang
Shutian Zhang
Jiantao Zhang
Weijian Zhang
Minggang Zhang
Hong Zhang
Wenbo Zhang
Jiaming Zhang
Ze Zhang
Chunyi Zhang
Dawei Zhang
Xiaofei Zhang
Jianmin Zhang
Xunzhong Zhang
Guoqiang Zhang
Gengxin Zhang
Yongsong Zhang
Zhirong Zhang
Deng-Feng Zhang
Chenggang Zhang
Wenjun Zhang
Gary Zhang
Jianhua Zhang
Xuelin Zhang
Ai Zhang
Hongyan Zhang
Shuang Zhang
Xiaoxiao Zhang
Qing Zhang
J. Zhang
Zhanjun Zhang
Zhenhai Zhang
Xifeng Zhang
Xu-Dong Zhang
Qian Zhang
Haicheng Zhang
Changqing Zhang
Juan Zhang
Hang Zhang
Li-Feng Zhang
Song Zhang
Chunfang Zhang
Weiwen Zhang
Libin Zhang
Mingliang Zhang
Huiyong Zhang
Xingdong Zhang
Ao Zhang
Xiaoguang Zhang
Si Zhang
Baoxue Zhang
Yejia Zhang
Qingwen Zhang
Guoqing Zhang
Xuchen Zhang
Rong Zhang
Tianhao Zhang
Ji-Hui Zhang
Shuning Zhang

Wei-Nan Zhang
Hairong Zhang
Tianhua Zhang
Chuanhai Zhang
Xiulan Zhang
Guohua Zhang
He Zhang
Wenzheng Zhang
Hengwei Zhang
Teng Zhang
Donglin Zhang
Xiaoge Zhang
Ji-Chun Zhang
Zhen Zhang
Kang Zhang
Wenzeng Zhang
Yahui Zhang
Wj Zhang
Xiaowei Zhang
Yili Zhang
Guang-Hui Zhang
Xinqing Zhang
Xiaoqing Zhang
Qiuheng Zhang
Yanxia Zhang
Zhi Zhang
Qinghua Zhang
Meng Zhang
Wengeng Zhang
Tongwu Zhang
Jingmei Zhang
Mingfeng Zhang
Lujia Zhang
Qiang Zhang
Qingyuan Zhang
Xinzhong Zhang
Shuwei Zhang
Xiufeng Zhang
Jingwei Zhang
Kefeng Zhang
Chengfu Zhang
Xuebo Zhang
Yafang Zhang
Zhaoqi Zhang
Zhiyong Zhang
Fei Zhang
Xinyu Zhang
Xiangning Zhang
Saijin Zhang
Zhaojun Zhang
Wen Zhang
Shaoping Zhang
Yiran Zhang
Yanqi Zhang
Chuanlun Zhang
Xilin Zhang
Xiaoyun Zhang
Shao-Qing Zhang
Fuchun Zhang
Shengping Zhang
Yong-Jie Zhang
Qin Zhang
Jiang-Hua Zhang
Kevin Zhang
Yong-Jiang Zhang
Zhengyu Zhang
Jiabao Zhang
Gui Zhang
Rumei Zhang
Siewi Zhang
Maojun Zhang
Lieyu Zhang
Sen Zhang
Gong Zhang
Cheng Zhang
Di Zhang
Xiaohui Zhang
Ziqiang Zhang
Xueqin Zhang
Yuhang Zhang
Wenlong Zhang
Ji-Fang Zhang
Qingzhu Zhang
Xiaotong Zhang
Chunyan Zhang
Zugui Zhang
Degan Zhang
Min-Ling Zhang
Fan Zhang
Huamin Zhang
Xinyou Zhang
Wenguang Zhang
Zhifeng Zhang
Caiguo Zhang
Yuwei Zhang
Xin Zhang

Renjian Zhang
Yawei Zhang
Yuanyuan Zhang
Guangxiang Zhang
Huizhen Zhang
Lisha Zhang
Huaguang Zhang
Mengxi Zhang
Yao Zhang
Xianfeng Zhang
Zhihua Zhang
Wanpeng Zhang
Ya-Nan Zhang
Chang-Yi Zhang
Yue-Miao Zhang
Dan Zhang
Enlou Zhang
Zong-Ming Zhang
Yuji Zhang
Wenbing Zhang
Fuzhong Zhang
Xuan Zhang
Dadong Zhang
Xiaoju Zhang
Xi Zhang
You Zhang
Wentao Zhang
Yongchun Zhang
Mengliang Zhang
Yimin Zhang
Yanqiong Zhang
Youzhong Zhang
Qian-Ming Zhang
Junjie Zhang
Ziming Zhang
Dongqing Zhang
Peipei Zhang
Emma Jingfei Zhang
Shaofei Zhang
Yuan Zhang
Weijun Zhang
Wenhai Zhang
Quan Zhang
Guanmin Zhang
Yumiao Zhang
Pengfei Zhang
Hongkun Zhang
Xu Zhang
Zhe Zhang
Mingzhen Zhang
Chengxin Zhang
Jin Zhang
Zijun Zhang
Tong Zhang
Jinqiang Zhang
Long-Wa Zhang
Zhaoyan Zhang
Xiang-Dong Zhang
Yujin Zhang
Xuezhen Zhang
Mingming Zhang
Lihong Zhang
Yanhao Zhang
Jiqiang Zhang
Ai-Bing Zhang
Haochun Zhang
Xiaodong Zhang
Shuai Zhang
Xueyong Zhang
Zhaoxiang Zhang
Zhengchen Zhang
Pan Zhang
Yanfei Zhang
Yingjun Zhang
Yong-Hong Zhang
Junyun Zhang
Zhi-Fen Zhang
Shaying Zhao
Mingrui Zhao
Hong-Bo Zhao
Hua Zhao
Zhangwu Zhao
Ai-Chun Zhao
Jing Zhao
Shuhong Zhao
Hui Zhao
Yong Zhao
Haiqing Zhao
Chao Zhao
Kai-Hong Zhao
Yunfeng Zhao
Yaofeng Zhao
Xiaopeng Zhao
Dawen Zhao
Min Zhao
Jincun Zhao

Ke Zhao
Youfu Zhao
Haibo Zhao
Junlong Zhao
Hong Zhao
Ping Zhao
Peng Zhao
Han Zhao
Botao Zhao
Jianyi Zhao
Zhi-Jun Zhao
Qinjian Zhao
Aiping Zhao
Gang Zhao
Bin Zhao
Yuanyin Zhao
Ying Zhao
Xinyang Zhao
Mei Zhao
Changjiu Zhao
Jianguo Zhao
Yutong Zhao
Zhuohui Zhao
Weihong Zhao
Xingquan Zhao
Dong Zhao
Feng Zhao
Hailin Zhao
Wei Zhao
Jinlei Zhao
Yi Zhao
Ying-Yong Zhao
Qunzi Zhao
Ting Zhao
Ningning Zhao
Fangqing Zhao
Isabella Zhao
Yongbing Zhao
Shanrong Zhao
Jiaying Zhao
Jun Zhao
Lingxia Zhao
Fei Zhao
Jinghong Zhao
Yagang Zhao
Zibo Zhao
Xiao-Dong Zhao
Dongyan Zhao
Yidan Zhao
Jie Zhao
Mintao Zhao
Chen Zhao
Jonathan Zhao
Lin Zhao
Xinyu Zhao
Bingzi Zhao
Zhong Zhao
Sicheng Zhao
Shanshan Zhao
Fuping Zhao
Mingwei Zhao
Ni Zhao
Huabin Zhao
Xiurong Zhao
Dawei Zhao
Xingming Zhao
Yunpeng Zhao
Wei-Gang Zhao
Bo Zhao
Dayong Zhao
Rongxin Zhao
Huixian Zhao
Quanzhi Zhao
Yan Zhao
Guangyi Zhao
Xingqiu Zhao
Shancen Zhao
Jichao Zhao
Dongxin Zhao
Zi-Hua Zhao
Pengfei Zhao
Ziming Zhao
Qian Zhao
Jichang Zhao
Pengjun Zhao
Qibin Zhao
Guang Zhao
Ping Zhao
Ming Zhao
Qi Zhao
Yanan Zhao
Qing (Grace) Zhao
Haixing Zhao
Kong-Nan Zhao
Yanping Zhao
Ruifeng Zhao

Ruogang Zhao
Yuanhui Zhao
Qiang Zhao
Quanyu Zhao
Liqin Zhao
Lujun Zhao
Zhidan Zhao
Lixia Zhao
Qijun Zhao
Shujuan Zhao
Tingchang Zhao
Kaiguang Zhao
Zhanghang Zhao
Kehao Zhao
Jiangchao Zhao
Qingwei Zhao
Qiong Zhao
Ke-Seng Zhao
Shilin Zhao
Jiawei Zhao
Xiangyu Zhao
Yanyan Zhao
C Zhao
Ting C Zhao
Yuansheng Zhao
Meixia Zhao
Xin Zhao
Linguo Zhao
Jinhao Zhao
Kexin Zhao
Alex Zhavoronkov
Vladimir Zhdanov
Zonglei Zhen
Yong-Tang Zheng
Yong-Hui Zheng
Ming-Hua Zheng
Jing Zheng
Song Guo Zheng
Zhi-Ming Zheng
Chengchao Zheng
Tongzhang Zheng
Hui Zheng
Jian Zheng
Xiaoying Zheng
Zhaoqing Zheng
Yun-Wen Zheng
Ming Zheng
Jie Zheng
Lemin Zheng
Shusen Zheng
Biao Zheng
Hairong Zheng
Xiaoqi Zheng
Hai-Lei Zheng
Shun-An Zheng
Weishi Zheng
Xiufen Zheng
Yuanrun Zheng
Du-Ping Zheng
Yalin Zheng
Bin Zheng
Wenguang Zheng
Liancun Zheng
Wenjing Zheng
Yong Zheng
Li Zheng
Jijian Zheng
Yan Zheng
Ling-Ling Zheng
Zheng Zheng
Chao Zheng
Feng Zheng
Yanling Zheng
Linfeng Zheng
Enhao Zheng
Mingwen Zheng
Xi Zheng
Jianjian Zheng
Zhe Zheng
Meiqin Zheng
Bo Zheng
Qian Zheng
Xiuwen Zheng
Guoyan Zheng
Yunhao Zheng
Ya Zheng
Yuesheng Zheng
Yingfeng Zheng
Wang Zheng
Jiajia Zheng
Y. G. Zheng
Yefeng Zheng
Yujun Zheng
Z. Zheng
Chun-Hou Zheng
Zhao Zheng

Dandan Zheng
Xunhai Zheng
Jufeng Zheng
Siyuan Zheng
Xinde Zheng
Zhen Zheng
Yaowu Zheng
Alexandra Zhernakova
Degui Zhi
Bin Zhi
Oleg Zhirnov
Boris Zhivotovsky
Alexander Zholos
Guangming Zhong
Nanshan Zhong
Xiao-Ping Zhong
Ping Zhong
Zhi Zhong
Weijian Zhong
Wenwan Zhong
Min Zhong
Shaobin Zhong
Jiang Zhong
Zhendong Zhong
Nanbert Zhong
Yuan Zhong
Suyu Zhong
Guohua Zhong
Mei Zhong
Wei Zhong
Shan Zhong
Jian-Hong Zhong
Cuncong Zhong
Haojie Zhong
Chao Zhong
Weicai Zhong
Yin Zhong
Silin Zhong
Yadong Zhong
Ling Zhong
Mingjun Zhong
Rui Zhong
Xiaojian Zhong
Jidan Zhong
Chenbo Zhong
Hua Zhong
Xiaomin Zhong
Bineng Zhong
Tianshou Zhou
Xiaofeng Zhou
Yan Zhou
Rui Zhou
Xiaodong Zhou
Hongbo Zhou
Jiawei Zhou
Hong Zhou
Xiaolin Zhou
Tianhua Zhou
Hai-Jun Zhou
Jiyong Zhou
Huan-Xiang Zhou
Yu Zhou
Yibiao Zhou
Jun Zhou
Heling Zhou
Huaijun Zhou
Yihua Zhou
Xinyue Zhou
Tao Zhou
Huiping Zhou
Chunyan Zhou
Shang-Ming Zhou
Min Zhou
Yuan Zhou
Xiang Zhou
Yan-Heng Zhou
Qi Zhou
Guang-Biao Zhou
Luping Zhou
Jiang Zhou
Daohong Zhou
Pei Zhou
Shiliang Zhou
Guangming Zhou
Xuanwei Zhou
Jianfang Zhou
Chunbao Zhou
Jian Zhou
Zhekun Zhou
Juan Zhou
Zhiahua Zhou
Wei-Xing Zhou
Yi-Hua Zhou
Junlan Zhou
Yang Zhou
Wenbin Zhou

Jiehua Zhou
Xin Zhou
Bin Zhou
Hong-Zhang Zhou
Zhiguang Zhou
Ping Zhou
Xinlin Zhou
Ming Zhou
Lei Zhou
Qian Zhou
Chengchao Zhou
Wei Zhou
Shui-Hong Zhou
Shuanhu Zhou
Meng-Liang Zhou
Qingyu Zhou
Yongming Zhou
Qingjun Zhou
Liang Zhou
Shi Zhou
Qinghua Zhou
Dawang Zhou
Xuezhong Zhou
Jiahua Zhou
Liang Fu Zhou
Yuefang Zhou
Deshan Zhou
Jinlin Zhou
Keming Zhou
Xiaoqi Zhou
Xinwen Zhou
Yong-Hong Zhou
Yin Zhou
Leming Zhou
Bo Zhou
Yue Zhou
Binghua Zhou
Lingyan Zhou
Shutang Zhou
Quan Zhou
Chunxiao Zhou
Jiliang Zhou
Lufang Zhou
Zhongliang Zhou
Zhiguo Zhou
Dong Zhou
Yun Zhou
Xiangmei Zhou
Jianying Zhou
Jie Zhou
Jue Zhou
Xiaoqin Zhou
Gaofeng Zhou
Zhongjun Zhou
Xinglian Zhou
Zhiyan Zhou
Canquan Zhou
Huiqing Zhou
Qixin Zhou
Zhou Zhou
Jianli Zhou
Shuntai Zhou
Zhongkai Zhou
Dongming Zhou
Guomei Zhou
Tianyin Zhou
Shuigen Zhou
Cindy Zhou
Peng Zhou
Jianfeng Zhou
Qingyuan Zhou
Xiao Zhou
Feng Zhou
X Zhou
Qing Zhou
Haisheng Zhou
Chao Zhou
Mi Zhou
Penghui Zhou
Lan Zhou
Jian-Hong Zhou
Guangsheng Zhou
Fei Zhou
Q.H. Zhou
Zhuxian Zhou
Yumei Zhou
Xuyu Zhou
Jiansong Zhou
En-Min Zhou
Dejian Zhou
Lian Zhou
Tian Zhou
Chengjie Zhou
Wenchao Zhou
Xiaoxue Zhou
Bolei Zhou

Zhenwen Zhou
Ruimin Zhou
H. Zhou
Jing Zhou
Hongyuan Zhou
Xikun Zhou
Nan Zhou
Yijun Zhou
Weibing Zhou
Dan Zhou
Zhouzh Zhou
Qingtao Zhou
Xuedong Zhou
Li Zhou
Sichang Zhou
Weiqi Zhou
Renke Zhou
Yifa Zhou
Man Zhou
Xiaoying Zhou
Qifa Zhou
Huakun Zhou
Zhigang Zhou
Haiming Zhou
Liqing Zhou
Xi Zhou
Shuijin Zhu
Lifeng Zhu
Huaiqiu Zhu
Lihuang Zhu
Jun Zhu
Qiyun Zhu
Shusheng Zhu
Yun Zhu
Huanzhang Zhu
Hui Zhu
Feng-Cai Zhu
Wei Zhu
Haizhen Zhu
Zeng-Rong Zhu
Xiang-Yang Zhu
Yushan Zhu
Xiaoping Zhu
Beili Zhu
Yu Cheng Zhu
Wuqiang Zhu
Meijun Zhu
Hu Zhu
Dajiang Zhu
Jianhua Zhu
Chao-Dong Zhu
Xianjun Zhu
Qian-Hao Zhu
Tao Zhu
Liqi Zhu
Danyan Zhu
Ying Zhu
Jingde Zhu
Linhai Zhu
Wei-Li Zhu
Daochen Zhu
Changfu Zhu
Hua Zhu
Runzhi Zhu
Xun Zhu
Longfu Zhu
Hailong Zhu
Lin Zhu
Mengxiao Zhu
Bin Zhu
Binggen Zhu
Yi-Fan Zhu
Yu-Xiao Zhu
Shiguo Zhu
Li Zhu
Hao Zhu
Zhaohui Zhu
Weiyun Zhu
Hongfei Zhu
Mengmeng Zhu
Meidong Zhu
Yueming Zhu
Jiangao Zhu
Xiaodong Zhu
Shouping Zhu
Chuanlong Zhu
Yezi Zhu
Yeyi Zhu
Quinn Zhu
Chengcheng Zhu
Liang Zhu
Xiangru Zhu
Jingjing Zhu
Xia Zhu
Lusha Zhu
Li-Li Zhu

Linhong Zhu
Qianhao Zhu
Bo Zhu
Yongzhang Zhu
Xiaodan Zhu
Qiuyu Zhu
Ming Zhu
Fa-Ming Zhu
Xiao Zhu
Di Zhu
De-Sheng Zhu
Nengwu Zhu
Fang Zhu
Bao-Li Zhu
Yihong Zhu
Haifeng Zhu
Yuanyuan Zhu
Wentao Zhu
Xinguang Zhu
Anning Zhu
Yanhe Zhu
Sen Zhu
Renbin Zhu
Wan Zhu
Ji Zhu
Jianzhong Zhu
Zhongjie Zhu
Jianming Zhu
Alan Zhu
Zhengge Zhu
Guo-Nian Zhu
Guangyu Zhu
Kai Zhu
Yalin Zhu
Dan Zhu
Xiongzhao Zhu
Dongxing Zhu
Mengqiang Zhu
Senhua Zhu
Lijun Zhu
Lili Zhu
Zhikai Zhu
Chengzhou Zhu
Yuwen Zhu
Fuli Zhu
Bi Zhu
Wenzhen Zhu
Yajuan Zhu
Jiangjiang Zhu
Guohui Zhu
Yongsheng Zhu
Weiren Zhu
Gefu Zhu
Frank Zhu
Kun Zhu
Yi Zhu
Xiaofeng Zhu
Qinling Zhu
Wenhan Zhu
Zude Zhu
Yuan Zhuang
Jing Zhuang
Chuxiong Zhuang
Xun Zhuang
Shougang Zhuang
Xiaofeng Zhuang
Ling Zhuang
Shunyao Zhuang
Wei Zhuang
Yijie Zhuang
Li Zhuang
Fuzhen Zhuang
Xiao Yu Zhuang
Ronghua Zhuge
Anna Zhukova
Vita Zhukova
Marianna Zhukovskaya
Mikhail Zhukovsky
Igor Zhulin
Yehong Zhuo
Yan Zhuo
Ying Zhuo
Vladimir Zhurov
Asim Zia
Shabnam Ziaee
Mounia Ziat
J. Ziauka
Muhammad Zia-Ul-Haq
Kazem Zibara
John Zibert
A. Zider
Noel Ziebarth
Wilma Ziebuhr
Toni Ziegler
Lawrence Ziegler
Regina Ziegler

Gabriel Ziegler
Dan Ziegler
Angelika Ziegler
Anna Zielak-Steciwko
Christoph Zielinski
Piotr Zielinski
Emily Zielinski-Gutierrez
Nadine Ziemert
Tjalf Ziemssen
Stéphan Zientara
Janine Ziermann
Kristina Zierold
James Zieske
Jeffrey Zigman
Michael Zigmond
Richard Zigmond
Adrienne Zihlman
Maeike Zijlmans
Andries Zijlstra
Carla Zijlstra
Sheng Zike
John Ziker
Efstathios Zikos
Alena Zikova
Jenny Zilberberg
Dan Zilberstein
Marietta Zille
Loredana Zilli
Jerri Zilli
Anton Zilman
Aleksey Zima
Christina Zimanyi
Sergey Zimatkin
Danila Zimenkov
Gert Zimmer
Marc Zimmer
Elke Zimmer
Geraldine Zimmer
Michael Zimmer
Karl Zimmerer
Laurent Zimmerli
Ludovic Zimmerlin
Lyle Zimmerman
Andy Zimmerman
Jeffrey Zimmerman
Naupaka Zimmerman
Fridolin Zimmerman
Molly Zimmerman
Jonas Zimmermann
Eckart Zimmermann
Hanna Zimmermann
Johannes Zimmermann
Uwe Zimmermann
Philippe Zimmern
Teresa Zimmers
Daniel Zinder
Bianca Zingales
Basilia Zingarelli
Lucie Zinger
Daniele Zink
Albert Zink
Mathias Zink
Brian Zink
Pascal Zinn
Steven Zinn
Elisabeth Zinser
Rutendo Zinyama-Gutsire
Pierluigi Zinzani
Wendy Zinzow-Kramer
Argyrios Ziogas
Piotr Ziolkowski
Elana Zion-Golumbic
Peter Zipfel
Andrew Zipkin
Mackenzie Zippay
Alexander Zipprich
Andreas Zirlik
Lorna Zischka
Dimitrios Zisoulis
Gernot Zissel
Ricardas Zitikis
Slavko Žitnik
Domenico Zito
Federica Zito Marino
Emanuel Zitt
Armin Zittermann
Laurence Zitvogel
Laurance Zitvogel
Elad Ziv
Naomi Ziv
Gil Ziv
Robert Zivadinov
Snezana Zivancevic-Simonovic
Elena Ziviani
Kara Zivin
Yin Ziying

Claire Zizza
Marta Zlatic
András Zlinszky
Sharon Zlochiver
Berislav Zlokovic
James Zlosnik
Andrew Zloza
Jaroslaw Zmijewski
Denis Zmirou-Navier
Sadri Znaidi
Brent Znosko
Gosia Zobel
Davide Zoccolan
Giovanna Zoccoli
Pierluigi Zoccolotti
Douglas Zochodne
D Zochodne
Sanjay Zodpey
Thomas Zoeller
Margot Zoeller
Vladislava Zohdi
Luca Zoia
Georg Zoidl
Christos Zois
Niklas Zojer
Phillip Zoladz
Massoud Zolgharni
Susan Zolla-Pazner
Heinz Zoller
Bengt Zöller
Christoph Zollikofer
Sue Anne Zollinger
Ines Zollner-Schwetz
Massimo Zollo
Bethany Zolman
Sergei Zolotukhin
Rebecca Zoltoski
Aldert Zomer
Amir Zomorodian
Zhiyong Zong
Geng Zong
Samantha Zongaro
Justin Zook
Kathryn Zoon
Harshal Zope
Regine Zopf
Giacomo Zoppini
Daniel Zoran
Todd Zorick
Michaela Zorn-Kruppa
Maria Zoroddu
Silvia Zorrilla
Graeme Zosky
Ami Zota
Teresa Zotta
Gerhard Zotz
Fei Zou
Kun Zou
Jun Zou
Haidong Zou
Liping Zou
Hejian Zou
Quanming Zou
Shu-Ming Zou
Yunzeng Zou
Xiaoming Zou
Chunqin Zou
Yonghong Zou
Yong Zou
Yiyu Zou
Wei Zou
Lin Zou
Quan Zou
Lifong Zou
Xiaowei Zou
Ling Zou
Gang Zou
Long-Hai Zou
Huasong Zou
Wen-Quan Zou
Chunbin Zou
Amina Zoubeidi
Anastasios Zouboulis
Driss Zoukhri
Anastasios Zouzias
Iva Zovkic
Thierry Zozio
Rita Zrenner
Miklós Zrínyi
Dusan Zrnic
Akos Zsembery
Zsuzsanna Zsengellér
Agustin Zsögön
Eniko Zsoldos
Youli Zu
Yao Zu

Karol Zub
Haseeb Zubair
Peter Zuber
Arkaitz Zubiaga
Barbara Zubik-Kowal
Igor Zubrzycki
Matteo Zucchetta
Marco Zucconi
David Zucker
Nancy Zucker
Andreas Zucker
Brian Zuckerbraun
Valentina Zuco
Iker Zudaire
George Zug
Alexandra Zugno
Micah Zuhl
Mozow Zuidema
Laurian Zuidmeer-Jongejan
Stephanie Zuilkowski
Malgorzata Zujko
Jennifer Zuk
Anna Zuk
Tamizi Zulkifly
Maria A Zuluaga
Eli Zunder
Matthias Zunhammer
Alfredo Zuniga
Pablo Zunino
Kaijing Zuo
Guanghong Zuo
Xi-Nian Zuo
Xian-Bo Zuo
Yuanmei Zuo
Jian-Ping Zuo
Zhiyi Zuo
Nianming Zuo
Zhentao Zuo
Yongchun Zuo
Lei Zuo
Lijun Zuo
Yi Zuo
Rantao Zuo
Yingtao Zuo
Anton Zupan
Valerio Zupo
Nicole Zur Nieden
Ryan Zurakowski
Petra Zürbig
Christian Zurbrugg
Amer Zureikat
Ludek Zurek
Todd Zurlinden
Karen Zurlo
Eyal Zussman
Roland Züst
Ellen Zuther
Nedelska Zuzana
Peter Zvara
Elena Zvereva
Laura Zwaan
Jaco Zwanenburg
Sara Zwart
Jacob Zwart
Ellen Zwarthoff
Markus Zweckstetter
Jay Zweier
Christiane Zweier
Christa Zweig
Monika Zwerger
Alice Zwerling
Werner Zwerschke
Kevin Zwetsloot
Andreas Zwick
Laurence Zwiebel
Anna Zwierzchowska
Lech Zwierzchowski
Norberto Zwirner
Joseph Zwischenberger
Rafal Zwolak
Adrian Zwolicki
Wojciech Zygner
Matthias Zytnicki
Sharon Zytynska
